# Sialic acid blockade in dendritic cells enhances CD8^+^ T cell responses by facilitating high-avidity interactions

**DOI:** 10.1007/s00018-021-04027-x

**Published:** 2022-01-28

**Authors:** N. Balneger, L. A. M. Cornelissen, M. Wassink, S. J. Moons, T. J. Boltje, Y. E. Bar-Ephraim, K. K. Das, J. N. Søndergaard, C. Büll, G. J. Adema

**Affiliations:** 1 Radiotherapy and OncoImmunology Laboratory, Department of Radiation Oncology, Radboud Institute for Molecular Life Sciences, Radboud University Medical Center, Geert Grooteplein Zuid 32, 6525 GA Nijmegen, The Netherlands; 2 Cluster for Molecular Chemistry, Institute for Molecules and Materials, Radboud University Nijmegen, Nijmegen, The Netherlands; 3 LUMICKS, Pilotenstraat 41, 1059 CH Amsterdam, The Netherlands; 4 Center for Infectious Disease Education and Research, Osaka University, Osaka, 565-0871 Japan; 5 Hubrecht Institute, Uppsalalaan 8, 3584 CT Utrecht, The Netherlands

**Keywords:** Glycosylation, Sialic acid, Dendritic cell, Cell avidity, CD8^+^ T cell, Sialic acid blockade

## Abstract

Sialic acids are negatively charged carbohydrates that cap the glycans of glycoproteins and glycolipids. Sialic acids are involved in various biological processes including cell–cell adhesion and immune recognition. In dendritic cells (DCs), the major antigen-presenting cells of the immune system, sialic acids emerge as important regulators of maturation and interaction with other lymphocytes including T cells. Many aspects of how sialic acids regulate DC functions are not well understood and tools and model systems to address these are limited. Here, we have established cultures of murine bone marrow-derived DCs (BMDCs) that lack sialic acid expression using a sialic acid-blocking mimetic Ac_5_3F_ax_Neu5Ac. Ac_5_3F_ax_Neu5Ac treatment potentiated BMDC activation via toll-like receptor (TLR) stimulation without affecting differentiation and viability. Sialic acid blockade further increased the capacity of BMDCs to induce antigen-specific CD8^+^ T cell proliferation. Transcriptome-wide gene expression analysis revealed that sialic acid mimetic treatment of BMDCs induces differential expression of genes involved in T cell activation, cell-adhesion, and cell–cell interactions. Subsequent cell clustering assays and single cell avidity measurements demonstrated that BMDCs with reduced sialylation form higher avidity interactions with CD8^+^ T cells. This increased avidity was detectable in the absence of antigens, but was especially pronounced in antigen-dependent interactions. Together, our data show that sialic acid blockade in BMDCs ameliorates maturation and enhances both cognate T cell receptor–MHC-dependent and independent T cell interactions that allow for more robust CD8^+^ T cell responses.

**Supplementary Information:**

The online version contains supplementary material available at 10.1007/s00018-021-04027-x.

## Introduction

Glycans are important regulators of the immune system that regulate immune cell development and function, modulate immune receptor interactions, and serve as ligands for glycan-binding proteins (lectins) expressed by immune cells [1, 2]. Several of the regulatory functions of glycans in the immune system are mediated by sialic acids, a family of negatively charged sugar molecules that terminate the glycan chains of glycolipids and glycoproteins at the cell surface and in secretion [3, 4]. The transfer of sialic acids to glycans, sialylation, occurs in the Golgi system and is catalyzed by 20 sialyltransferase isoenzymes that couple sialic acids to glycans via distinct linkages (α2–3, α2–6, or α2–8) [5]. Sialic acid-carrying glycans, sialoglycans, are involved in numerous molecular interactions at the cell surface and have several direct and indirect functions in the immune system [3]. For example, sialoglycans on the lymphocyte glycoprotein PSGL-1 form the ligands for selectins on endothelial cells and this interaction recruits lymphocytes to sites of inflammation [6]. Sialoglycans serve as specific ligands for the sialic acid-binding immunoglobulin-like lectins (Siglecs), a family of immune regulatory receptors [7, 8]. Siglecs are expressed by most cells of the immune system and other cell types and recognize distinct sialoglycans either on the same cell surface (*cis-*interactions) or on the surface of adjacent cells (*trans-*interactions) [9, 10]. Sialic acid–Siglec interactions induce either activatory or inhibitory signaling, depending on the Siglec family member, and are important modulators of the immune response [11, 12]. Other general functions of sialic acids in the immune systems include masking of underlying glycan structures and blocking of biophysical interactions by virtue of their negative charge [3]. Although sialic acids play an essential role in the immune system, many of their immune cell type-specific functions remain unexplored.

In DCs, sialic acids have been suggested to regulate several of the specific functions of these major antigen-presenting cells of the immune system [13]. Human and mouse DCs treated with sialidase to remove surface sialic acids and DCs derived from sialyltransferase knock-out mice (*St3Gal1*^−/−^ and *St6Gal1*^−/−^) showed enhanced maturation and increased phagocytosis of bacteria by human monocyte-derived DCs (moDCs), respectively [14–16]. Polysialic acids, linear polymers of α2–8-linked sialic acids, on the CCR7 chemokine receptor regulate chemokine-mediated migration of DCs, as they are required for binding of CCR7 to its chemokine ligand CCL21 [17, 18]. Moreover, several studies have reported that sialidase treatment enhances maturation of DCs and facilitates major histocompatibility complex I and II (MHC I/II) expression or accessibility resulting in improved stimulation of T cells [14, 15, 19]. In line with these studies, we have previously shown that sialylation can be efficiently blocked in human moDC cultures using the fluorinated sialic acid mimetic Ac_5_3F_ax_Neu5Ac that potently inhibits sialyltransferase activity [20, 21]. Ac_5_3F_ax_Neu5Ac-treated moDCs showed stronger responses to TLR stimulation compared to control moDCs and had improved ability to activate CD4^+^ T cells. Moreover, we have recently shown that intratumoral injections with Ac_5_3F_ax_Neu5Ac suppressed tumor growth in mouse tumor models [22]. This effect was largely mediated by the desialylation of tumor cells and improved eradication by activated CD8^+^ T cells. Moreover, we observed increased maturation of DCs in the tumor and tumor-draining lymph nodes of mice injected with the sialic acid-blocking mimetic [22]. These studies strongly suggest that sialic acids regulate specific steps in DC biology and that further understanding and control of sialylation, especially in DC–T cell interactions, could advance cellular immunotherapy.

Here, we report on the development of murine bone-marrow-derived DC (BMDC) cultures with blocked sialylation using Ac_5_3F_ax_Neu5Ac enabling broad dissection and discovery of the biological functions of sialic acids in DCs. Treatment with this metabolic sialyltransferase inhibitor effectively and durably blocked sialylation in BMDCs without affecting viability or differentiation. We confirm and extend previous findings that sialic acids limit DC maturation and demonstrate that blocked sialylation in BMDCs facilitates antigen-specific activation of CD8 + T cells. Using RNA sequencing (seq) analysis we revealed that Ac_5_3F_ax_Neu5Ac-treatment affects pathways involved in direct DC–T cell interactions and we demonstrate that sialic acid blockade strongly enhances the binding avidity of antigen-dependent and independent interactions between BMDCs and CD8^+^ T cells. In summary, Ac_5_3F_ax_Neu5Ac treatment generates fully differentiated BMDC cultures without sialylation that enable dissection of the role of sialic acids in DC biology and in particular their role in DC–T cell interactions.

## Materials and methods

### Mice

Female C57BL/6 J wildtype (WT) mice (7–14 weeks at the beginning of the experiments) were purchased from Charles River (Sulzfeld, Germany). OT-I mice (8–14 weeks at the beginning of the experiments) producing CD8^+^ T cells that express a transgenic T cell receptor specific for the chicken ovalbumin (OVA) epitope SIINFEKL (OVA257-264) presented on MHC class I H–2 Kb and the congenic marker CD90.1 were bred and held in house. All mice were kept under specific pathogen-free conditions at the Central Animal Laboratory (Nijmegen, The Netherlands). Drinking water and standard laboratory food pellets were provided ad libitum*.*

### Generation of BMDCs and OT-I T cell isolation

Bone marrow (BM) was harvested from the femurs and tibia of female C57BL/6 J mice. The femurs and tibia were soaked in 70% ethanol for 2 minutes (min) and washed in 1 × PBS. BM from the femurs and tibia was flushed out with RPMI 1640 medium (Gibco, Thermo Fisher Scientific) supplemented with 10% fetal calf serum (FCS; GE healthcare, Little Chalfont, UK), 1% UltraGlutamine (Lonza, Bazel, Switzerland), 0.1% β-mercapto-ethanol (Gibco) and 100 U/m penicillin G sodium and 100 μg/mL streptomycin (Pen/Strep) (Gibco). Single cell suspensions were obtained by passing the isolated BM through 100 µm cell strainers (Falcon, Corning Life Sciences, Tewksbury, MA, USA) followed by erythrocyte lysis for 2 min with cold ACK lysis buffer (150 mM NH_4_Cl, 10 mM KHCO_3_ and 0.1 mM EDTA). 4*10^6^ cells were cultured for 7 days in 13 ml medium containing 20 ng/ml granulocyte–macrophage colony stimulating factor (GM-CSF; Peprotech, London, UK) in 10 cm petri dishes (VWR, Radnor, PA, USA) at 37 °C and 5% CO_2_ under humidified conditions. On day 3, the medium was replenished by adding 4 ml medium containing 37.2 ng/ml GM-CSF and on day 6 by adding 1 ml medium containing 158 ng/ml GM-CSF. BMDCs were harvested on day 7 for experiments. Single cells suspensions were prepared from spleens of OT-I mice through mechanical dissociation using 100 µm cell strainers (Falcon). Splenocytes were resuspended in 1 × PBS containing 1 mM EDTA and 2% FBS. CD8^+^ T cells were negatively isolated using the EasySep™ Mouse CD8^+^ T cell Isolation Kit (Stemcell Technologies) following the manufacturer’s protocol.

### Reagents and antibodies

The sialic acid mimetic Ac_5_3F_ax_Neu5Ac was synthesized as described previously [21, 23]. Carbo‐free blocking solution and biotinylated lectins MALII, SNA‐I, and PNA were purchased from Vector Laboratories, Inc. (Burlingame, CA, USA). Streptavidin‐PE was purchased from BD Pharmingen (Franklin Lakes, NJ, USA), eFluor 780 and 450 viability dyes from eBioscience, Inc. (San Diego, CA, USA), CellTrace™ Violet (PSBE) and CFSE Cell Proliferation Kits from Thermo Fisher Scientific. CpG ODN 1668 (‘5-TCCATGACGTTCCTGATGCT-3’) was purchased from Sigma Genosys (Haverhill, UK), lipopolysaccharide (LPS) (*Escherichia coli* O111:B4) from Sigma-Aldrich, and murine recombinant interleukin (IL)-2 from Immunotools. For flow cytometry experiments, the following antibodies were used: BioLegend: anti-I-A/I-E-BV510 (M5/114.15.2), anti-H-2 Kb/H-2D^b^-PE (28–8-6), anti-CD86-APC/Cy7 (GL-1), Anti-CD90.1-APC/Cy7 (OX-7). Antibodychain: anti-CD80-A488 (16-10A1), anti-CD40-PE (23/3), anti-CD11c-APC (N418), anti-CD11b-PerCP (M1/70). eBioscience: anti-CD274-PE/Cy7 (MIH5). BD: anti-CD8a-V450 (53–6.7).

### Sialic acid blockade, sialidase treatment and TLR stimulation

On day 0, day 3, and day 6 of the BMDC culture, 250 µM Ac_5_3F_ax_Neu5Ac or DMSO was added to the medium, except for the dose titration experiment, where a concentration between 0 and 500 µM of Ac_5_3F_ax_Neu5Ac was used. Sialidase treatment was performed with differentiated BMDCs on day 7 by adding 250 mU/ml sialidase from *Clostridium perfringens* (Sigma-Aldrich) for 1 hour (h) at 37 °C. After incubation, the cells were washed thoroughly and used for experiments. For the TLR stimulation, 1*10^5^ day 7 BMDCs were plated into 96-well round bottom plates (Corning) and stimulated with CpG (200 and 400 ng/ml) or LPS (1 and 10 ng/ml) for 18 h at 37 °C. After the stimulation, the cells were harvested and stained for flow cytometry analysis.

### RNA sequencing and bioinformatics analysis

BMDCs differentiated for 7 days in the absence or presence of Ac_5_3F_ax_Neu5Ac (250 µM) were subjected to sorting for CD11c^+^ cells (MACS, Miltenyi) according to the manufacturer’s instructions. RNA from CD11c^+^ cells was isolated using TRIzol (Thermo Fisher Scientific) following the manufacturer’s instructions. RNA seq was performed by BGI Genomics (Hong Kong). The number of aligned reads in the bam files provided by BGI were counted using feature count in the subread package (v. 1.5.3) and the reference genome Gencode GRCm38 (v.M15). All subsequent analysis was conducted in R (v. 3.6.1). After normalization (TMM: trimmed mean of *M* values), a differential gene expression analysis was performed using edgeR (v. 3.18.1). Significant differently expressed genes (DEGs) were distinguished by a false discovery rate (FDR) under 0.05. Gene ontology analysis was performed with clusterProfiler (v. 3.12.0) and org.Mm.eg.db (v. 3.8.2). In addition, the following dependent package versions were installed: DOSE (v. 3.10.2), AnnotationDbi (v. 1.46.1), IRanges (v. 2.18.3), S4Vectors (v. 0.22.1), BiocGenerics (v. 0.30.0), and Biobase (v. 2.44.0). Results were visualized using ggplot2 (v. 3.2.1).

### Flow cytometry analysis

For lectin staining, the cells were first washed with 1 × carbo-free blocking solution. Next, the cells were incubated for 45 min at 4 °C with biotinylated MALII (5 µg/ml), SNA-I (1 µg/ml), or PNA (5 µg/ml) in 1 × carbo-free blocking solution supplemented with 1 mM MgCl_2_ and 1 mM CaCl_2_ (both from Merck). After incubation, the cells were washed with PBA (1 × PBS, 1% bovine serum albumin, 0.02% sodium azide), and incubated with Streptavidin-PE for 20 min at 4 °C. For antibody staining, the cells are harvested, washed in 1 × PBS and stained with eFluor 780 or 450 viability dye according to the manufacturer’s instructions. Fc receptors were blocked with anti-CD16/CD32 (2.4G2, BD) in PBA for 10 min at 4 °C. Afterwards, the cells were stained with fluorescent antibodies in PBA at 4 °C for 20 min, and washed three times with PBA. Samples were acquired with a CytoFLEX LX Flow Cytometer (Beckman Coulter), BD FACSVerse™ flow cytometer (BD Bioscience), Gallios (Beckman Coulter) or FACS Cyan (Beckman Coulter), respectively. Data analysis was performed using FlowJo software (Tree Star, Ashland, OR, USA).

### OT-I proliferation assay

BMDCs generated in the presence or absence of Ac_5_3F_ax_Neu5Ac (250 µM) were washed in 1 × PBS and pulsed with OVA protein (Endograde, Hyglos GmbH, Germany) for 3 h or OVA H-2 Kb peptide (257–264, AS-60193, Tebu-bio) for 1 h at 37 °C. After the incubation, the BMDCs were washed three times, and 50.000 cells were seeded per well into a round bottom plate in culture medium. The CD8^+^ OT-I T cells were labeled with 3 µM CFSE according to the manufacturer’s instructions. 50.000 CFSE-labeled CD8^+^ OT-I T cells were added to the BMDCs (1:1 ratio) in culture medium. The co-cultures were incubated for 3 days at 37 °C in the presence of 0 or 10 ng/ml IL-2. After 3 days, the cells were analyzed by flow cytometry. Interferon gamma (IFNγ) levels in the supernatants were analyzed using an IFNγ ELISA (Thermo Fisher Scientific) following the manufacturer’s protocol.

### OVA uptake and degradation assay

Control and Ac_5_3F_ax_Neu5Ac (250 µM) treated BMDCs were washed with 1 × PBS and seeded into round bottom plates at a density of 2*10^5^ cells/well in culture medium. The BMDCs were pulsed with 100 ng/ml Alexa Fluor 647-OVA to follow uptake or 1 µg/ml DQ-OVA to follow degradation (both from Thermo Fisher Scientific), respectively, washed and cultured at 37 °C. At different timepoints, the cells were washed three times and the fluorescence was determined by flow cytometry.

### BMDC:OT-I T cell clustering assay

Control and Ac_5_3F_ax_Neu5Ac (250 µM) treated BMDCs were labeled with 12 µM CellTrace Violet dye (Thermo Fisher Scientific) according to the manufacturer’s instructions. Afterwards, the BMDCs were pulsed with 0 or 1 ng/ml OVA peptide for 1 h at 37 °C. The isolated CD8^+^ OT-I T cells were labeled with 1 µM CFSE according to the manufacturer’s instructions. In total 50.000 BMDCs and 50.000 OT-I T cells were co-cultured in Falcon round-bottom polystyrene test tubes for 45 min at 37 °C. The cells were fixed with 2% PFA for 10 min at RT and subsequently analyzed using a CytoFlex LX flow cytometer.

### Cell avidity analysis

Cell–cell interaction strength between BMDCs and CD8^+^ T cells was analyzed using a z-Movi® Cell Avidity Analyzer (LUMICKS, Amsterdam, The Netherlands). BMDCs were allowed to adhere to a microfluidic z-Movi chip (LUMICKS) for 30 min after which CD8^+^ T cells were added and co-incubated with the BMDCs for indicated time-periods, before assessing cell avidity using acoustic force. Briefly, the chips were coated with poly-L-lysine (Sigma-Aldrich) for 10 min and air-dried for 60 min at 37 °C. Control and Ac_5_3F_ax_Neu5Ac treated BMDCs were pulsed with 1 ng/ml OVA H-2 Kb peptide, 1 ng/ml irrelevant murine HPV peptide (RAHYNIVTF, kindly gifted by Thorbald van Hall, Leiden University Medical Center, The Netherlands) or medium for 1 h at 37 °C. After washing thoroughly, 3–5*10^6^ BMDCs were flushed into the poly-L-lysine coated chip and incubated for 30 min at 37 °C. The chips were placed onto the z-Movi Cell Avidity Analyzer, where experiments were performed at 37 °C. To ensure attachment of the BMDC monolayer to the surface of the chip, an initial constant force of 1000 pN (as calibrated on 10 μm polystyrene beads) was applied. CD8^+^ OT-I T cells were fluorescently labeled with CellTrace Far Red dye (Thermo Fisher Scientific) according to manufacturer’s protocol and 1–3*10^5^ cells were flushed into the z-Movi Chip. CD8^+^ T cells were allowed to interact with the BMDC monolayer for indicated time-periods. Subsequently, a linear force ramp was applied from 0 to 1500 pN (as calibrated for 10 μm polystyrene beads) for 3 min 45 s (6.7 pN/s). Avidity runs were analyzed using Oceon Software (LUMICKS).

### Statistical analysis

Statistical significance was calculated by performing a *t* test or a one-way analysis of variance (ANOVA) followed by Bonferroni’s correction using Prism 8 software (GraphPad, Inc., La Jolla, CA). *P* values < 0.05 were considered significant. Significance is shown as: ns. > 0.05, *P < 0.05, **P < 0.01, ***P < 0.001.

## Results

### Ac_5_3F_ax_Neu5Ac inhibits sialic acid expression in BMDCs without affecting differentiation and viability

**Fig. 1:**
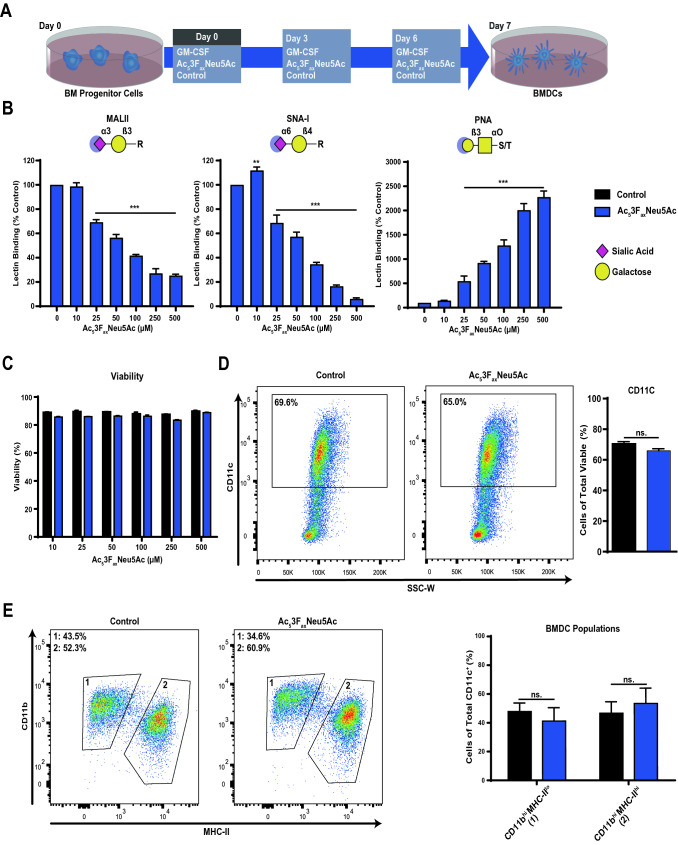
Ac_5_3F_ax_Neu5Ac blocks sialic acid expression in BMDCs without affecting differentiation. **A** Schematic representation of the generation of BMDCs. BM cells from murine femurs and tibia were seeded into petri dishes containing culture medium and supplemented with GM-CSF and Ac_5_3F_ax_Neu5Ac (0–500 µM) or DMSO. On days 3 and 6, the medium was replenished with fresh medium containing GM-CSF and Ac_5_3F_ax_Neu5Ac (0–500 µM) or control DMSO. On day 7, the BMDCs were harvested. **B** Dose-dependent inhibition of sialic acid expression with Ac_5_3F_ax_Neu5Ac. Expression of α2-3- and α2-6-linked sialic acids or uncapped galactose/Tn antigen on BMDCs was detected using the lectins MALII, SNA-I and PNA, respectively, from left to right. The lectin binding was normalized to control untreated cells. Representative bar diagrams show mean values ± SD of three technical replicates. **C** Percentage of viable cells in BMDC cultures treated with different concentrations of Ac_5_3F_ax_Neu5Ac compared to control-treated cells. Representative bar diagrams show mean values ± SD of three technical replicates. **D** Expression of the DC differentiation marker CD11c on control and treated (250 µM Ac_5_3F_ax_Neu5Ac) BMDCs. Representative dot plots show CD11c^+^ cells of control and treated (250 µM Ac_5_3F_ax_Neu5Ac) BMDCs. Mean percentages ± SD CD11c^+^ cells of total viable cells are presented as bar diagram (*n* = 2). (E) Ratio of two GM-CSF BMDC culture populations within viable and CD11c^+^ BMDCs. Population 1 (left gate) is defined as CD11b^hi^MHC-II^lo^ and population 2 (right gate) as CD11b^int^MHC-II^lo^. The gating strategy for the two populations is shown for control BMDC cultures and cultures treated with 250 µM Ac_5_3F_ax_Neu5Ac. Quantification of both BMDC populations is shown as a bar diagram (*n *= 2) and the experiment was performed three times

Previously, we have shown that sialic acid blockade with a fluorinated sialic acid mimetic Ac_5_3F_ax_Neu5Ac lowers the threshold of human moDCs for TLR-mediated activation [20]. Here, we investigated the potency of this sialic acid mimetic to block sialylation of murine BMDCs. At the start of the differentiation (day 0), concentrations ranging from 0 to 500 µM Ac_5_3F_ax_Neu5Ac were added to the GM-CSF BMDC cultures. On day 3 and 6 of the culture Ac_5_3F_ax_Neu5Ac was replenished (Fig. 1A). After 7 days of culture, the expression of sialic acids and uncapped β-galactose, was measured via flow cytometry using the lectins MALII (α2-3-linked sialic acid), SNA-I (α2-6-linked sialic acid) and PNA (β-galactose, T antigen), respectively (Fig. 1B). Sialylation of differentiated BMDCs was reduced in a dose-dependent manner without affecting viability (Fig. 1C) or the percentage of CD11c^+^ cells (Fig. 1D). GM-CSF BMDC cultures consist of two distinct DC subsets that are readily distinguished by their MHC-II and CD11b expression [24]. Sialic acid blockade had no effect on the ratio of these CD11b^hi^MHC-II^lo^ and CD11b^int^MHC-II^hi^ BMDC subsets (Fig. 1E). Notably, metabolic sialic acid blockade with Ac_5_3F_ax_Neu5Ac reduced surface sialic acid levels to a similar extent as enzymatic removal with bacterial sialidase (Figure S1A, B). More importantly, the treatment with Ac_5_3F_ax_Neu5Ac reduced sialylation for at least 3 days following removal from the culture medium, whereas desialylation with sialidase was only short-lived (< 24 h) (Figure S1C). These data demonstrate that sialic acid blockade with Ac_5_3F_ax_Neu5Ac is effective and long-lasting in BMDCs without affecting cell viability or differentiation.

### Sialic acid blockade enhances TLR-induced BMDC maturation

**Fig. 2:**
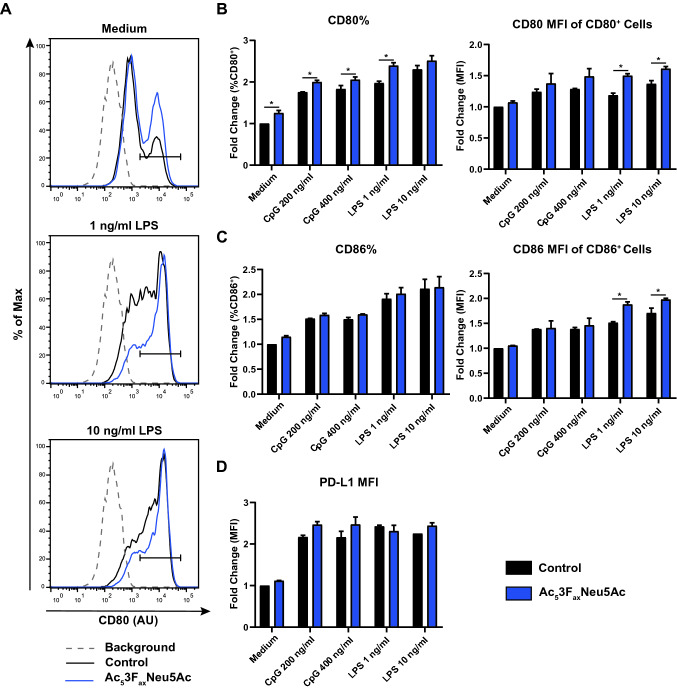
Sialic acid blockade potentiates TLR-mediated maturation of BMDCs. **A–F** Control BMDCs or BMDCs cultured with 250 µM Ac_5_3F_ax_Neu5Ac were stimulated with various concentrations of TLR ligands for 18 h. **A** Representative histograms show expression of CD80 at the cell surface of CD11c^+^ BMDCs stimulated with 1 or 10 ng/ml LPS. **B** Bar diagrams show percentage of cells that are CD80^+^ (gated on viable, CD11c^+^) (left) and fluorescence intensity (MFI) of CD80 expression (gated on viable, CD11c^+^, CD80^+^ cells) (right). **C** Bar diagrams show percentage of cells that are CD86^+^ (gated on viable, CD11c^+^) (left) and fluorescence intensity (MFI) of CD86 expression (gated on viable, CD11c^+^, CD86^+^ cells) (right). **D** Fold change PD-L1 expression (gated on viable, CD11c^+^ cells). Data are presented as mean values ± SD of two biological replicates and each experiment was performed three times

To investigate whether sialic acid blockade with Ac_5_3F_ax_Neu5Ac affects DC maturation, BMDCs were stimulated with the TLR agonists CpG (TLR9 agonist) and LPS (TLR4 agonist), or left untreated (DMSO) as control. BMDC cultures with blocked sialylation contained increased numbers of CD80^+^ cells after CpG and LPS stimulation compared with control sialylated BMDCs **(**Fig. 2A, B). Of note, a slightly higher number of CD80^+^ cells, but not CD86^+^ cells, was also present in Ac_5_3F_ax_Neu5Ac cultures without TLR stimulation. We have previously shown that the sialic acid mimetic is produced endotoxin-free, suggesting that desialylation alone induces BMDC maturation to a minor degree [20]. Expression of the DC maturation marker CD86 was moderately increased after LPS treatment in Ac_5_3F_ax_Neu5Ac-treated BMDCs compared to control treated BMDCs (Fig. 2C). Expression of the immune checkpoint inhibitor ligand PD-L1 was upregulated upon LPS and CpG stimulation, but no significant difference was found between control and Ac_5_3F_ax_Neu5Ac treatment (Fig. 2D). In addition, no significant difference in cytokine production (IL-6, IL-12p70, TNFα, IL-10, and IL-1β) was observed between control and Ac_5_3F_ax_Neu5Ac-treated BMDCs upon TLR stimulation (data not shown). Altogether, Ac_5_3F_ax_Neu5Ac treatment during BMDC differentiation resulted in a moderately increased maturation by TLR ligands.

### Ac_5_3F_ax_Neu5Ac treatment enhances the capacity of BMDCs to activate CD8^+^ OT-I T cells

**Fig. 3:**
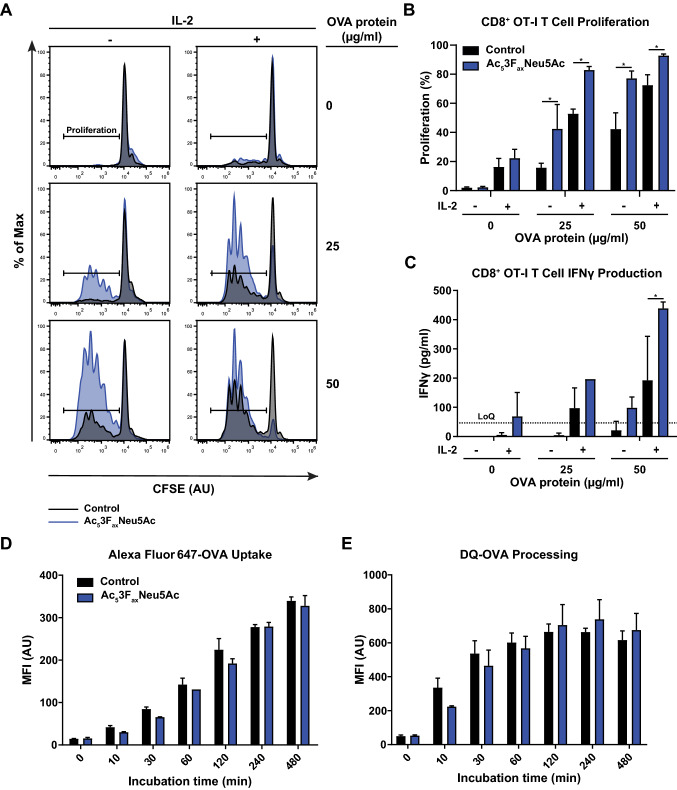
Ac_5_3F_ax_Neu5Ac-treated BMDCs have increased capacity to induce CD8^+^ OT-I T cell proliferation. **A**–**C** CFSE-labeled CD8^+^ OT-I T cells were co-cultured (1:1 ratio) with control or Ac_5_3F_ax_Neu5Ac-treated BMDCs pulsed with different concentrations of OVA protein, and in the presence or absence of IL-2. OT-I proliferation was determined by flow cytometry analysis using dilution of the CFSE signal. **A** Representative histograms depict CD8^+^ OT-I T cell proliferation after co-culture with control BMDCs (black) and Ac_5_3F_ax_Neu5Ac-treated BMDCs (blue) for the different conditions. Bar diagrams show percentage of proliferated (**B**) CD8^+^ OT-I T cells and IFNγ levels in the co-culture supernatant (**C**) as average values ± SD of two biological replicates. The dotted line represents the IFNγ detection limit (LoQ). **D**, **E** Uptake of Alexa Fluor 647-OVA (**D**) and processing of DQ-OVA (**E**) by the BMDCs over time was quantified by flow cytometry. Data are presented as mean fluorescence intensity values ± SD of two biological replicates and all experiments were performed three times

DCs are critical for CD8^+^ T cell priming and activation [25]. The long-lived reduction of sialic acids after Ac_5_3F_ax_Neu5Ac treatment in BMDCs allowed to investigate the role of sialic acids in this process in more detail. To this end, control and Ac_5_3F_ax_Neu5Ac treated BMDCs were pulsed with different OVA protein concentrations and co-cultured for 72 h with purified CFSE-labeled CD8^+^ OT-I T cells. Numbers of proliferating CD8^+^ OT-I T cells were quantified by flow cytometry. We observed higher OVA-dependent proliferation rates in co-cultures with Ac_5_3F_ax_Neu5Ac treated BMDCs compared with control BMDCs (Fig. 3A, B). Addition of IL-2 enhanced overall OVA-specific CD8^+^ OT-I T cell proliferation, but the higher proliferation rate in co-cultures with Ac_5_3F_ax_Neu5Ac-treated BMDCs remained (Fig. 3A, B). In line with the increased CD8^+^ OT-I T cell proliferation, IFNy levels were higher in the co-culture supernatants of BMDCs with sialic acid blockade compared to control (Fig. 3C). In agreement with these observations, sialic acid blockade also enhanced CD8^+^ OT-I T cell proliferation when BMDCs were externally loaded with the OVA H-2 Kb-binding peptide SIINFEKL (Figure S2). These findings strongly suggest that blocking sialylation improves the capacity of BMDCs to activate CD8^+^ T cells.

To assess whether this effect was caused by changes in OVA antigen presentation by BMDCs upon Ac_5_3F_ax_Neu5Ac treatment, we measured OVA uptake and processing using Alexa Fluor 647-OVA (uptake) and DQ-OVA (processing), respectively. No significant difference in uptake and processing of OVA protein was detected between control and Ac_5_3F_ax_Neu5Ac treated BMDCs (Fig. 3D, E). These findings combined with the enhanced OT-I proliferation upon external OVA peptide loading indicate that the increase in T cell activation is not simply explained by alterations in OVA antigen uptake and processing. Notably, we observed that BMDCs differentiated in the presence of Ac_5_3F_ax_Neu5Ac expressed higher MHC-I levels at the cell surface compared to control BMDCs (Figure S3). Enhanced MHC-I expression was, however, also detected after short treatment with sialidase, which indicates that antibody binding to MHC-I molecules is hindered by sialic acids. Desialylation thus may improve accessibility of MHC-I and other cell surface molecules rather than solely upregulating their expression.

### Sialic acid blockade affects genes involved in cell–cell interactions

**Fig. 4:**
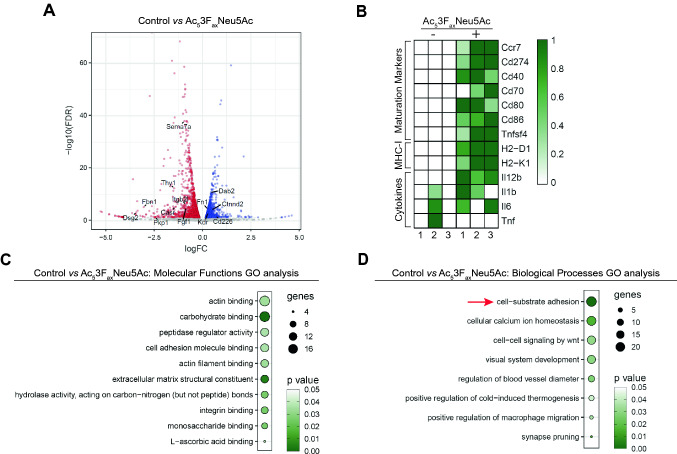
Differential gene expression in Ac_5_3F_ax_Neu5Ac treated BMDCs reveals effects on cell–cell adhesion. **A**–**D** RNA seq analysis was performed on CD11c^+^ isolated BMDCs generated in the presence or absence of Ac_5_3F_ax_Neu5Ac. **A** Comparative analysis of control and Ac_5_3F_ax_Neu5Ac treated BMDCs. The volcano plot depicts the log fold change and FDR in a minus log 10 transformation. Significantly DEG (FDR < 0.05) are shown in red. Genes from the molecular functions GO term analysis ‘cell adhesion molecule binding’ are highlighted. **B** Expression profile of genes involved in T cell co-stimulation are displayed in a row scaled heatmap. GO term enrichment analysis showing top significantly altered Molecular Functions (**C**) and Biological Processes (simplified) (**D**). **D** GO term of interest is indicated with a red arrow. RNA seq was performed with three biological replicates

Loss of sialic acids can affect interactions and functions of glycoproteins and glycolipids including that of glycosylated surface receptors. As a result, signaling events and gene expression may change. To reveal signaling pathways and cellular functions altered upon sialic acid blockade that might underly the mechanisms of the increased T cell activating capacity of BMDCs following sialic acid blockade, RNA seq and subsequent Gene Ontology (GO) analysis was performed. Comparative analysis between control and Ac_5_3F_ax_Neu5Ac treated BMDCs revealed > 2500 differentially expressed genes (FDR < 0.5) (Fig. 4A). The full list of differentially expressed genes (DEGs) is provided in appendix 1 in supplementary material. Several genes involved in T cell co-stimulation such as the DC maturation markers *CD40*, *CD80*, *CD86*, as well as MHC-I genes (*H2-D1/K1*) were higher expressed in Ac_5_3F_ax_Neu5Ac treated BMDCs compared to control (Fig. 4B). The genes encoding the cytokines IL-1 beta, IL-6, and tumor necrosis factor (TNF) were not significantly up-/down-regulated in either treatment which is in line with our data on cytokine production. GO enrichment analysis was performed on the top 250 up/down-regulated set of DEGs (Fig. 4C, D). The Molecular Functions GO pathway analysis indicated that Ac_5_3F_ax_Neu5Ac treatment affects genes involved in cellular interactions and adhesion (GO groups: actin binding, carbohydrate binding, cell adhesion molecule binding, actin filament binding, integrin binding, monosaccharide binding and L-ascorbic acid binding) (Fig. 4C). This is in line with the simplified Biological Processes GO analysis that yielded altered adhesion as first term (GO group: cell-substrate adhesion) (Fig. 4D). The lists of GO results can be found in appendix 2 in supplementary material. Overall, the RNA seq analysis supports our finding that sialic acid blockade increases the ability of BMDCs to co-stimulate T cells, but also predicts that BMDC adhesion and cell interaction events may be involved in potentiating T cells.

### Sialic acid blockade facilitates BMDCs-CD8^+^ OT I T cell clustering

**Fig. 5:**
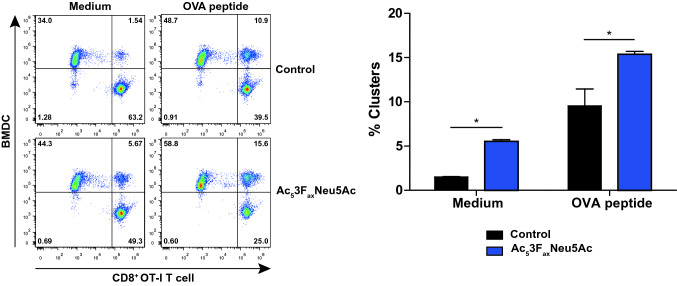
Reduced sialylation favors clustering between BMDCs and CD8^+^ OT-I T cells Ac_5_3F_ax_Neu5Ac and control-treated BMDCs were pulsed with OVA peptide or medium. For cell identification during flow cytometry acquisition, BMDCs were labeled with CellTrace Violet dye, and CD8^+^ OT-I T cells were labeled with CellTrance Far Red dye. After 45 min of incubation at 37 °C, cluster formation was assessed by flow cytometry. Representative dot plots show BMDCs–CD8^+^ OT-I T cell clusters (left) and quantification is shown as bar diagram (right). Percentages are shown as mean values ± SD of two biological replicates and experiments were performed three times

Prompted by the GO analysis, we further explored the effects of sialic acid blockade on BMDC-CD8^+^ T cell interactions. Ac_5_3F_ax_Neu5Ac and control treated BMDCs were pulsed with OVA peptide (SIINFEKL) or medium and labeled with CellTrace violet dye. The BMDCs were than co-incubated for 45 min at 37 °C with CFSE-labeled CD8^+^ OT-I T cells at a 1:1 ratio and cluster formation was analyzed by flow cytometry. In the control samples with sialylated BMDCs and no OVA peptide, about 1.5% of the cells formed clusters (Fig. 5). Ac_5_3F_ax_Neu5Ac treatment significantly increased cluster formation to > 5%, suggesting that antigen-independent DC–T cell interactions are hampered by sialic acid present on BMDCs. As expected, clustering was enhanced following loading of DCs with the nominal OVA epitope. OVA peptide loaded Ac_5_3F_ax_Neu5Ac-treated BMDCs, however, clustered more effectively with OT-I T cells as compared to control BMDCs (Fig. 5). These findings strongly indicate that sialic acid blockade facilitates antigen-independent and antigen-dependent interactions between BMDCs and CD8^+^ OT-I T cells.

### Ac_5_3F_ax_Neu5Ac treated BMDCs have a higher binding avidity to CD8^+^ OT I T cells

**Fig. 6:**
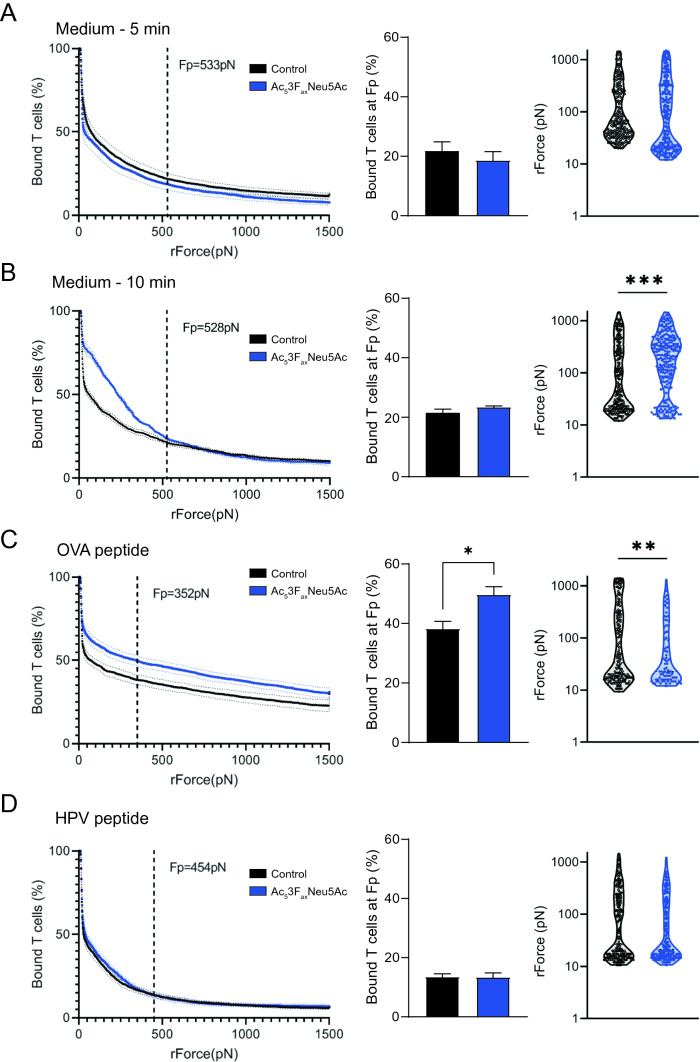
Sialic acid blockade facilitates high-avidity DC–T cell interactions. **A**–**D** Control and Ac_5_3F_ax_Neu5Ac treated BMDCs and CD8^+^ OT-I T cells were co-incubated on z-Movi chips for 5 min (**A**, **C**, **D**) or 10 min (**B**), after which a linear force ramp was applied from 0 to 1500 pN. **A**–**D** Graphs show: left: average avidity curves ± SEM of CD8^+^ OT-I T cells co-incubated with control or Ac_5_3F_ax_Neu5Ac treated BMDCs. The Plateau force (Fp) is indicated with a dotted line. Middle: percentage of bound cells for indicated conditions at Fp. Right: violin plots show detachment force of single cells in representative runs of indicated conditions. Indicated are number of cells analyzed per run, symbols indicate single cells. **A**–**B** Absence of antigens. **C** BMDCs were pulsed with OVA peptide for 1 h prior to adherence to the chip. **D** BMDCs were pulsed with an irrelevant HPV peptide for 1 h prior to adherence to the chip

Next, we measured the binding avidity between BMDCs and CD8^+^ OT-I T cells at the single cell level using the z-Movi Cell Avidity Analyzer (Figure S4) [26–28]. BMDCs were adhered to microfluidic z-Movi chips and subsequently incubated with CD8^+^ OT-I T cells. The CD8^+^ OT-I T cells were allowed to interact with the adherent BMDCs for a selected time period, after which a linear force ramp from 0 to 1500 pN was applied. We first compared the avidity curves of Ac_5_3F_ax_Neu5Ac and control treated BMDCs that interacted with CD8^+^ OT-I T cells for 5 or 10 min in the absence of antigens. After 5 min, BMDC–T cell binding was disrupted at a similar force for control and Ac_5_3F_ax_Neu5Ac treated BMDCs (Fig. 6A). The interactions with T cells formed in 10 min were significantly stronger for BMDCs without sialic acids compared to control BMDCs (Fig. 6B). Most of the control treated BMDCs lost their interaction to the CD8^+^ OT-I T cells below a force of 100 pN, while higher forces were required to break the interactions with Ac_5_3F_ax_Neu5Ac treated BMDCs. To address the antigen-specific interactions, BMDCs were pulsed with OVA peptide or irrelevant HPV control peptide and allowed to form interactions with CD8^+^ OT-I T cells for 5 min. The binding avidity was significantly higher for OVA-pulsed Ac_5_3F_ax_Neu5Ac BMDCs compared with control BMDCs (Fig. 6C). This effect was antigen-specific, because no significant changes were observed with HPV peptide pulsed BMDCs after 5 min (Fig. 6D). These findings demonstrate that Ac_5_3F_ax_Neu5Ac treated BMDCs form higher avidity interactions with CD8^+^ OT-I T cells that are formed rapidly in the presence of antigen in the MHC-I context, and slower in the absence of antigens.

## Discussion

In this study, we have applied a fluorinated sialic acid mimetic, Ac_5_3F_ax_Neu5Ac, to generate BMDC cultures with effective and long-lived blockade of sialic acid expression that enabled studies into the broader role of sialic acids in DC–CD8^+^ T cell interactions. Addition of Ac_5_3F_ax_Neu5Ac during the 7-day differentiation phase of BMDCs largely prevented incorporation of sialic acids into cell surface glycans. Importantly, sialic acid blockade had no significant effects on the viability and differentiation process of BMDCs and was long-lived after removal of the mimetic from the culture. The maturation with TLR ligands was slightly improved in BMDCs generated in the presence of Ac_5_3F_ax_Neu5Ac and sialic acid blockade markedly facilitated activation and proliferation of OVA-specific OT-I CD8^+^ T cells. The treatment with sialic acid mimetic resulted in altered expression of genes involved in biological and molecular pathways related to T cell activation and cellular interactions. Subsequent functional analysis demonstrated that the absence of sialic acids from glycans increases antigen-specific and non-specific conjugate formation between BMDCs and OT-I CD8^+^ T cells. The sialic acid blockade approach reported here is a powerful tool to unravel the role of sialic acids in DC biology and revealed that sialic acid sugars can be modulated to enhance T cell activation and proliferation.

Research into the role of sialic acids in DC biology has mainly been performed with bacterial sialidases that hydrolyze the bond between sialic acids and glycans. These studies generally showed enhanced maturation of murine and human DCs following sialidase treatment and suggested that sialic acids negatively influence T cell activation [14–16, 29–33]. Our findings support these studies and provide a metabolic route for efficient removal of surface sialic acids. Treatment with bacterial sialidases is fast; however, it has several disadvantages that can be overcome with sialic acid mimetics. First, bacterial sialidases are mainly produced in bacterial expression systems and preparations contain endotoxins making them unsuitable for immunological assays [14, 30]. However, human sialidase produced by human cells can overcome this limitation. Second, sialidases may stay bound to the cells after hydrolysis and sterically hinder glycan recognition or could influence other interactions [3, 34–36]. Finally, the effect of sialidase removal of surface sialic acids is short-lived, and in BMDCs and other cells sialylation recovers within 24 h which limits long-term culture experiments [37, 38]. Metabolic inhibition with chemically pure sialic acid mimetics such as Ac_5_3F_ax_Neu5Ac overcomes these limitations and allows long-lived inhibition of sialylation murine BMDCs and as previously demonstrated also in human moDCs [20]. Ac_5_3F_ax_Neu5Ac, however, should be used with caution as systemic administration in large concentrations (300 mg/kg) is relatively toxic [39]. Intratumoral Ac_5_3F_ax_Neu5Ac injections with a lower dose was found to be effective in mice without causing renal toxicity [22]. Our group has also encapsulated Ac_5_3F_ax_Neu5Ac into nanoparticles coated with tumor targeting antibodies to specifically and safely target the sialic acid mimetic to circulating cancer cells preventing metastases [23]. Sialidases are found to be less toxic, due to the short-lived desialylation [40]. To further limit toxicity and increase its efficacy, also sialidases have been targeted to tumors by coupling it to HER2 targeting antibodies [41–43]. Overall, the sialic acid mimetic Ac_5_3F_ax_Neu5Ac allows for global discovery of the wide functions of sialic acids in DC biology and other (immune)cells. More refined knock-out of individual sialyltransferase isoenzymes and/or sialoglycoproteins, can subsequently be performed to attribute specific functions to particular sialic acid linkages, glycoconjugates, and glycoproteins.

We have previously reported that Ac_5_3F_ax_Neu5Ac treatment enhances maturation of human DCs with TLR ligands, and accordingly other groups have reported similar findings for human and mouse DCs after bacterial sialidase treatment [15, 20, 29–32]. Here we extend these data to murine BMDCs and show that sialic acid removal also increased their sensitivity to TLR stimulation. Collectively, these findings suggest that sialic acids are generally negative regulators of DC maturation in humans and mice, particularly by limiting (TLR-mediated) activation. How sialic acids regulate DC maturation remains to be determined. Possibly, the negative charge of sialic acids influences interactions between TLRs and their ligands, and furthermore, there is evidence that TLRs carry sialic acids themselves that directly regulate their signaling [32, 44–46]. Sialoglycans form the ligands for immunomodulatory Siglec receptors and the majority of these receptors can mediate immune inhibitory signals [7–9, 12]. DCs express several Siglec family members and we have previously suggested that sialoglycans on the surface interact with inhibitory Siglecs via *cis*-interactions which could regulate DC activation and functions [20]. Loss of *cis*-interactions through removal of Siglec ligands by Ac_5_3F_ax_Neu5Ac could aid in the enhanced DC activation that we observe after TLR stimulation. Our in vitro generated BMDCs; however, lack expression of Siglec-E and only a minority of the cells express Siglec-G and -F (data not shown), which makes loss of *cis-*interactions less likely to explain the effects of Ac_5_3F_ax_Neu5Ac treatment on murine BMDC activation and subsequent T cell activation.

BMDCs cultured with Ac_5_3F_ax_Neu5Ac induced stronger CD8^+^ T cell proliferation compared to sialylated BMDCs, similar to results obtained by others using sialidases [15, 16, 33]. Potentially, this is a result of enhanced DC activation but our data indicate that this also occurs in the absence of exogenously added maturation stimuli or TLR ligands. Our findings now provide evidence that intensified formation of high-avidity interactions between BMDCs and CD8^+^ T cells contributes to the enhanced T cell activation. Importantly, we observed no differences in antigen uptake and processing in BMDCs generated with or without sialic acids. Others have found that sialidase treatment of moDCs or BMDCs derived from sialyltransferase knockout mice (*St3gal1*^−/−^ and *St6gal1*^−/−^) rather show decreased OVA uptake [14, 47]. This indicates that the observed enhanced DC–T cell interactions are not simply the result of altered antigen processing. There is some evidence that sialic acids may regulate stability of MHC-I complexes on DCs [19]. Accordingly, we have observed increased exposure of MHC-I molecules on DCs after sialidase and mimetic treatment. Increased MHC-I exposure alone, however, is unlikely to fully account for the increased DC–T cell interactions and high-avidity binding upon desialylation. The finding that DC–T cell clustering is enhanced even in the absence of the cognate peptides also supports this notion. More likely, we propose that sialic acids negatively influence multiple interactions between DCs and CD8^+^ T cells and that their global removal by sialic blockade allows for enhanced CD8^+^ T activation. This is supported by our RNA seq analysis showing that BMDCs generated without sialic acids have differential gene expression enriched for T cell activation and cellular interactions. Moreover, our functional data show enhanced cluster formation of BMDCs without sialic acids with OT-I CD8^+^ T cells in the presence and even absence of cognate antigen.

The significantly increased DC–T cell interactions could be the result of altering the negative charge at the cell surface, removal of steric hindrance formed by sialic acids, and/or generation of (specific) interaction sites for example between uncapped galactose residues that could interact with galectins on the T cells [3]. In line with our findings, it has recently been reported that BMDCs with reduced sialylation (sialidase, Ac_5_3F_ax_Neu5Ac) induce robust CD8^+^ and CD4^+^ T cell proliferation [33]. It was proposed that this effect was due to competitive binding of *cis* and *trans* sialoglycans and CD80 to the co-stimulatory T cell receptor CD28. Potentially, the increased avidity that we observed between Ac_5_3F_ax_Neu5Ac-treated BMDCs and OT-I CD8^+^ T cells could, at least partly, be explained by the removal of competing sialoglycan ligands for CD28 and enhanced interactions with CD80. Siglecs on T cells recognizing sialoglycans are other candidate receptors that could potentially be involved in the enhanced interactions. While naive murine T cells do not express Siglecs, Siglec-E can be upregulated under pathological conditions as it has been found on CD8^+^ tumor infiltrating lymphocytes [48]. In our model system, no expression of Siglecs has been found on the naïve and activated CD8^+^ OT-I T cells [22]. This makes abolished *trans* binding of T cell Siglecs to sialic acids on BMDCs in our model system an unlikely mechanism behind the increased T cell proliferation.

Interestingly, we have previously observed that intratumoral injections with Ac_5_3F_ax_Neu5Ac into subcutaneous melanoma increased DC maturation and CD8^+^ T cell responses [22]. This result may now be explained by the identified enhanced avidity of DCs–T cell interactions and suggests that sialic acid blockade could be applied to obtain robust CD8^+^ T cell responses in vivo. Future experiments will have to clarify the effects on CD8^+^ T cells in terms of immunity and autoimmunity. Concluding, our data show that the sialic acid mimetic Ac_5_3F_ax_Neu5Ac is a useful tool to potentiate the capacity of BMDCs to induce CD8^+^ T cell proliferation. Moreover, this is the first report showing that sialic acid blockade increases BMDC binding avidity to CD8^+^ T cells in an antigen-dependent and antigen-independent manner. The enhanced capability of desialylated DCs to cluster and activate antigen specific CD8^+^ T cells may be exploited in immunotherapy of cancer and infectious disease.

## Supplementary Information

Below is the link to the electronic supplementary material.

**Fig. S1 Ac**_**5**_**3F**_**ax**_**Neu5Ac blocks sialylation for several days** (A, B) Cells were treated with 250 µM Ac_5_3F_ax_Neu5Ac for 7 days or 1 h with 250 mM sialidase. Expression of α2-3- and α2-6-linked sialic acids on BMDCs was detected using the lectins MALII, SNA-I and PNA from left to right. Data is shown as representative histograms (A) and quantifications are provided as bar diagrams with mean percentage lectin binding ±SD normalized to control-treated cells of two biological replicates. (C) Recovery of sialic acid expression over 3 days after treatment with sialidase or Ac_5_3F_ax_Neu5Ac, respectively, was measured using MAL-II, SNA-I and PNA. Graphs show percentage lectin binding ±SD normalized to control treated BMDCs (dotted line) for three consecutive days after treatment (n=3). (PDF 587 KB)

**Fig. S2** **Sialic acid blockade enhances CD8**^**+**^** OT-I T cell proliferation induced by OVA peptide pulsed BMDCs** BMDCs were generated in the presence or absence of Ac_5_3F_ax_Neu5Ac and pulsed with increasing concentrations of OVA peptide prior to co-culture with CFSE-labeled CD8^+^ OT-I T cells. Bar diagrams present the percentage of proliferated CD8^+^ OT-I T cells (left) and IFNγ levels in the co-culture supernatants (right). Data are presented as average values ±SD of two biological replicates. (PDF 389 KB)

**Fig. S3:** **Ac**_**5**_**3F**_**ax**_**Neu5Ac and sialidase treatment upregulate MHC-I** Cell surface expression of MHC-I on control, Ac_5_3F_ax_Neu5Ac or sialidase treated BMDCs was measured by flow cytometry using fluorescent anti-MHC-I antibodies and is shown as representative histogram (left) and bar diagram (right) with mean fluorescence intensity values ±SD of two biological replicates representative of three independent experiments. (PDF 171 KB)

**Fig. S4 Schematic representation of avidity curves **The first part of the curve represents non-antigen specific binding. Next, the preliminary binding stage starts. When less than 2% of the T cells lose their binding over a difference of 100 pN, the plateau force (Fp) point is reached. After this point T cells are stably bound to the BMDCs, the percentage of cells that lose their binding is more phased out. At the end of the curve there might be stuck T cells, whose bounds did not break. (PDF 181 KB)

Supplementary file5 (PDF 2333 KB)

Supplementary file6 (PDF 77 KB)

## Data Availability

The RNA seq generated during the current study are available in the ArrayExpress repository, under accession number: E-MTAB-XXXX (reviewer link: http://www.ebi.ac.uk/arrayexpress/experiments/E-MTAB-XXX, username: Reviewer_E-MTAB-XXXX, password: santa). All scripts used for bioinformatics analysis are available on Github: XXX. Other data generated and analyzed during this study are included in this published article and its supplementary information files.
