# Supplementary figures and images for: Sialic acid blockade in dendritic cells enhances CD8+ T cell responses by facilitating high-avidity interactions
Source: Cell Mol Life Sci. 2022 Jan 28;79(2):98. doi: 10.1007/s00018-021-04027-x (PMC8799591; doi:10.1007/s00018-021-04027-x)

**A**

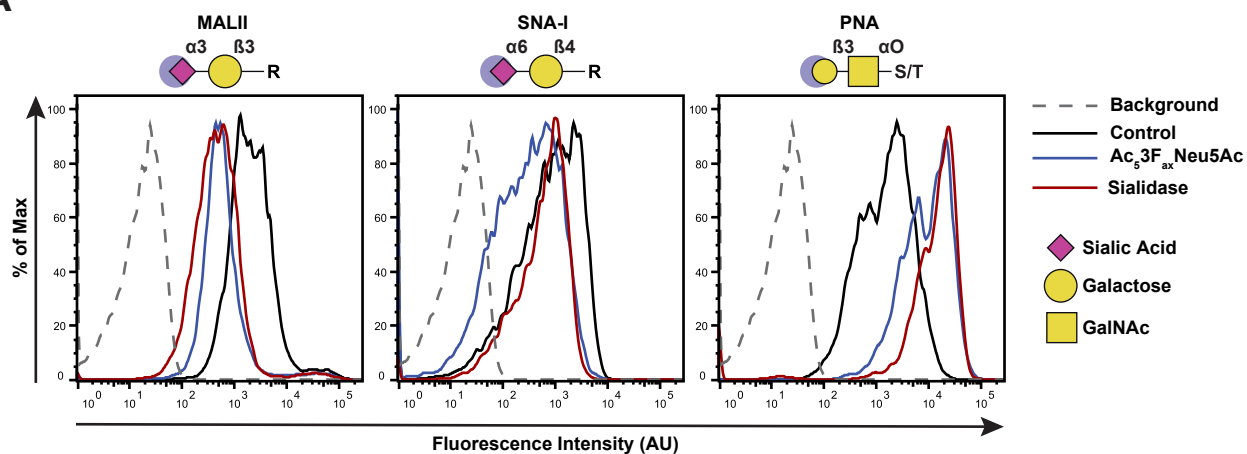

**B**

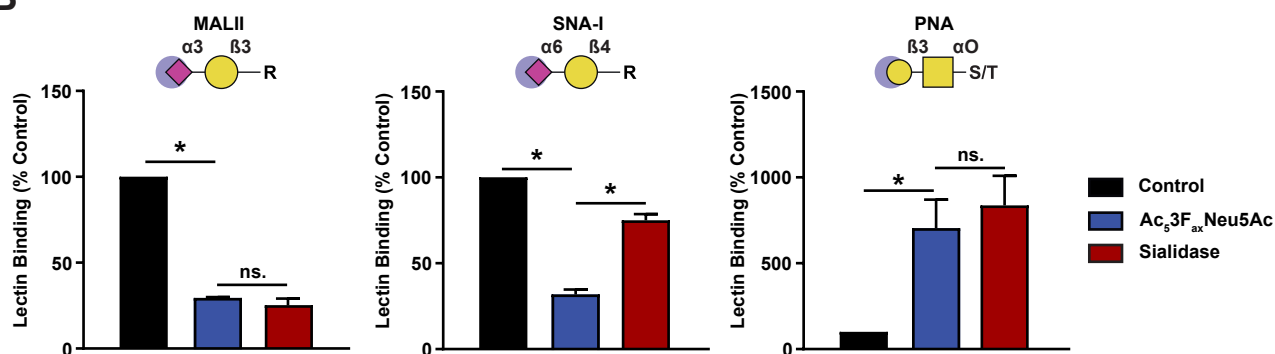

**C**

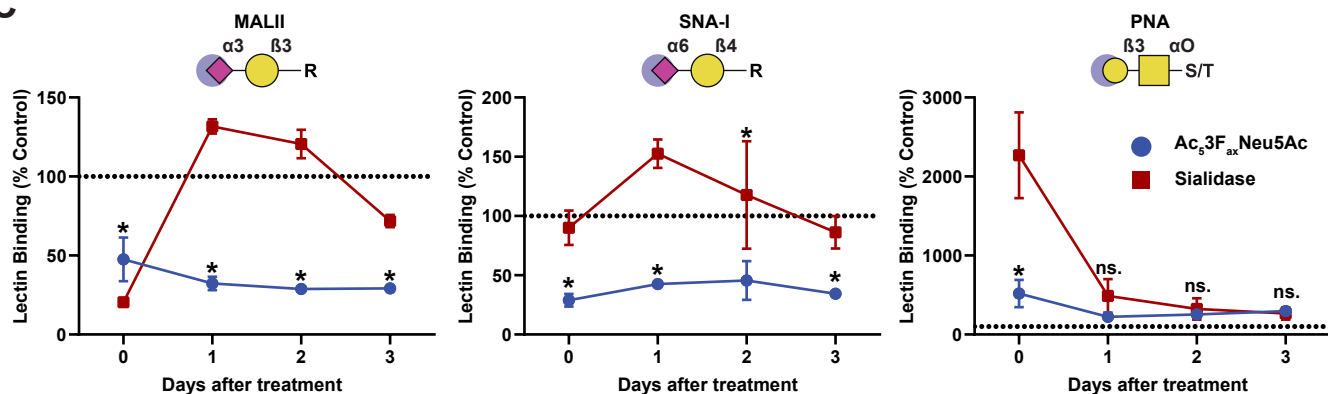

Supplement: Supplementary file 1 — Fig. S1 Ac53FaxNeu5Ac blocks sialylation for several days (A, B) Cells were treated with 250 µM Ac53FaxNeu5Ac for 7 days or 1 h with 250 mM sialidase. Expression of α2-3- and α2-6-linked sialic acids on BMDCs was detected using the lectins MALII, SNA-I and PNA from left to right. Data is shown as representative histograms (A) and quantifications are provided as bar diagrams with mean percentage lectin binding ±SD normalized to control-treated cells of two biological replicates. (C) Recovery of sialic acid expression over 3 days after treatment with sialidase or Ac53FaxNeu5Ac, respectively, was measured using MAL-II, SNA-I and PNA. Graphs show percentage lectin binding ±SD normalized to control treated BMDCs (dotted line) for three consecutive days after treatment (n=3). (PDF 587 KB) [file 18_2021_4027_MOESM1_ESM.pdf]

**CD8<sup>+</sup> OT-I T Cell Proliferation**

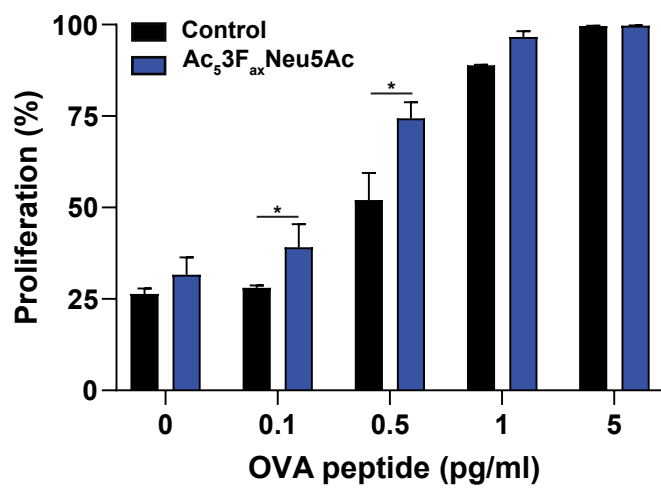

**CD8<sup>+</sup> OT-I T Cell IFN $\gamma$  Production**

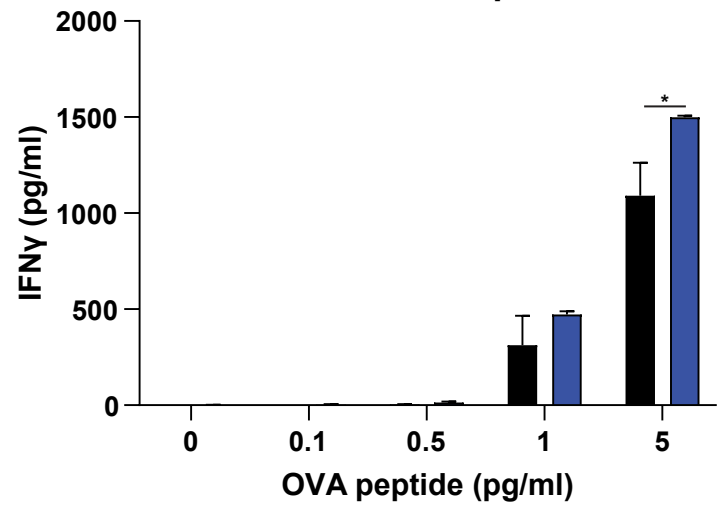

Supplement: Supplementary file 2 — Fig. S2 Sialic acid blockade enhances CD8+ OT-I T cell proliferation induced by OVA peptide pulsed BMDCs BMDCs were generated in the presence or absence of Ac53FaxNeu5Ac and pulsed with increasing concentrations of OVA peptide prior to co-culture with CFSE-labeled CD8+ OT-I T cells. Bar diagrams present the percentage of proliferated CD8+ OT-I T cells (left) and IFNγ levels in the co-culture supernatants (right). Data are presented as average values ±SD of two biological replicates. (PDF 389 KB) [file 18_2021_4027_MOESM2_ESM.pdf]

**A**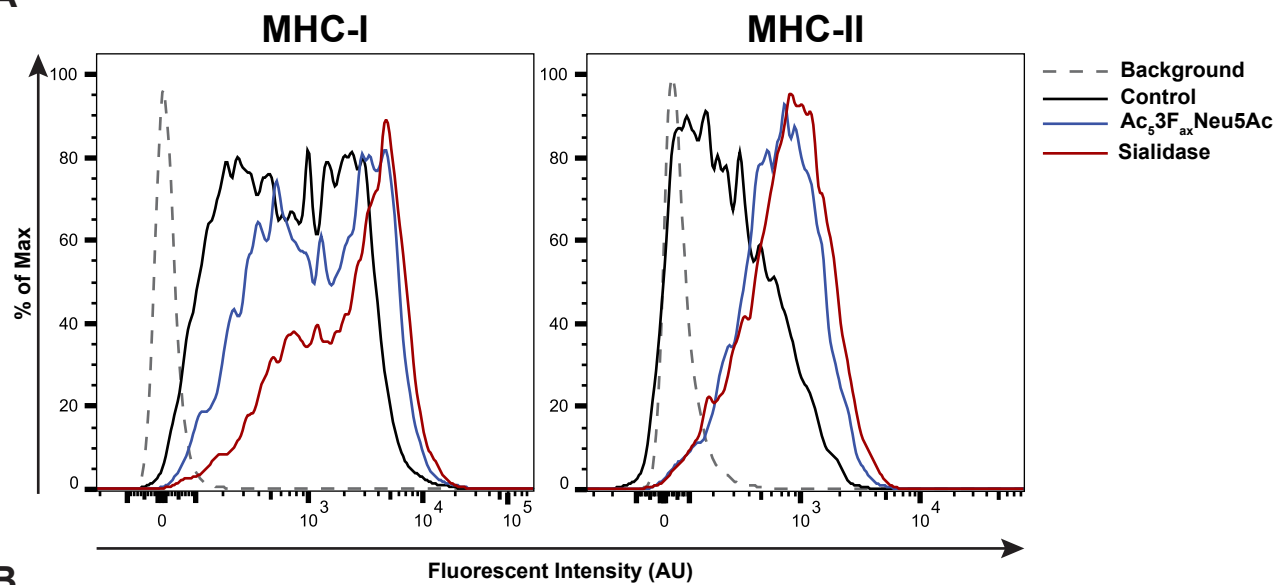**B**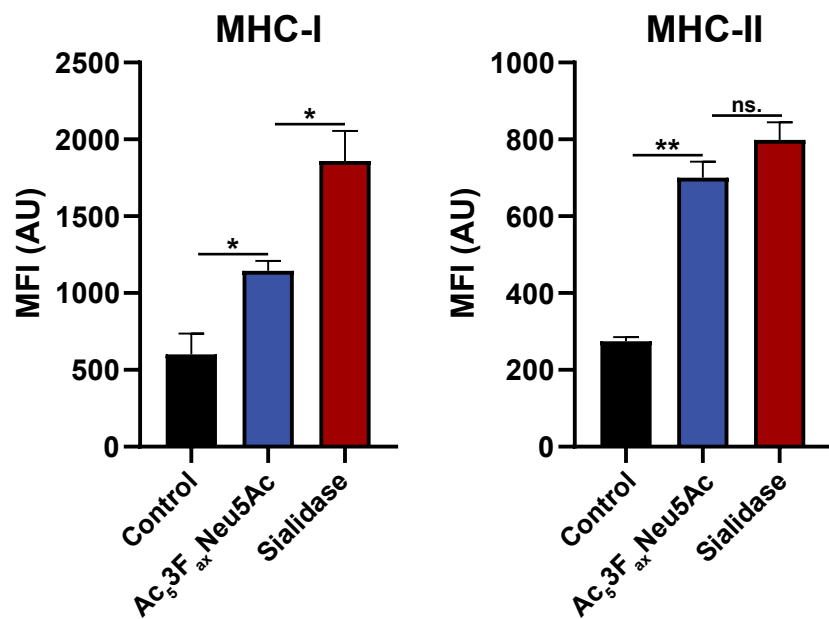

Supplement: Supplementary file 3 — Fig. S3: Ac53FaxNeu5Ac and sialidase treatment upregulate MHC-I Cell surface expression of MHC-I on control, Ac53FaxNeu5Ac or sialidase treated BMDCs was measured by flow cytometry using fluorescent anti-MHC-I antibodies and is shown as representative histogram (left) and bar diagram (right) with mean fluorescence intensity values ±SD of two biological replicates representative of three independent experiments. (PDF 171 KB) [file 18_2021_4027_MOESM3_ESM.pdf]

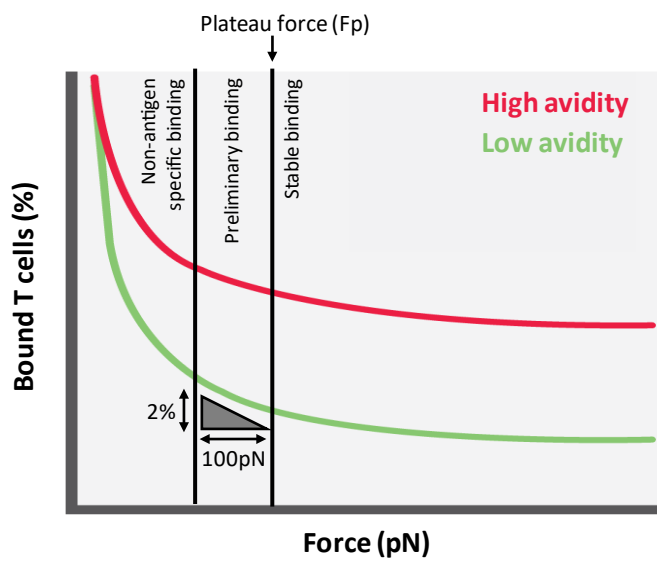

Supplement: Supplementary file 4 — Fig. S4 Schematic representation of avidity curves The first part of the curve represents non-antigen specific binding. Next, the preliminary binding stage starts. When less than 2% of the T cells lose their binding over a difference of 100 pN, the plateau force (Fp) point is reached. After this point T cells are stably bound to the BMDCs, the percentage of cells that lose their binding is more phased out. At the end of the curve there might be stuck T cells, whose bounds did not break. (PDF 181 KB) [file 18_2021_4027_MOESM4_ESM.pdf]
