# Supplementary material for: Sialic acid blockade in dendritic cells enhances CD8+ T cell responses by facilitating high-avidity interactions
Source: Cell Mol Life Sci. 2022 Jan 28;79(2):98. doi: 10.1007/s00018-021-04027-x (PMC8799591; doi:10.1007/s00018-021-04027-x)
Supplement: Supplementary file 5 — Supplementary file5 (PDF 2333 KB) [file 18_2021_4027_MOESM5_ESM.pdf]

ensembl_gene_id	logFC	logCPM	LR	PValue	FDR	external_gene_name	Fold_Change	significant(FDR<0.05)
ENSMUSG00000000001	-1,75E-01	8,181319464	9,15E+00	2,49E-03	3,32E-02	Gnai3	0,89	Yes
ENSMUSG00000000028	8,68E-02	4,11340038	9,95E-01	3,19E-01	6,19E-01	Cdc45	1,06	No
ENSMUSG00000000031	1,81E+00	-3,10765737	1,28E+00	2,58E-01	5,50E-01	H19	3,49	No
ENSMUSG00000000049	2,75E-02	-1,378340174	2,47E-03	9,60E-01	1,00E+00	Apoh	1,02	No
ENSMUSG00000000056	1,13E-01	5,651934962	2,80E+00	9,45E-02	3,85E-01	Narf	1,08	No
ENSMUSG00000000058	3,73E-01	6,953093161	3,73E+01	1,03E-09	8,36E-08	Cav2	1,30	Yes
ENSMUSG00000000078	1,16E-02	9,551376133	4,22E-02	8,37E-01	9,81E-01	Klf6	1,01	No
ENSMUSG00000000085	-1,30E-01	4,241792863	2,39E+00	1,22E-01	4,36E-01	Scmh1	0,91	No
ENSMUSG00000000088	-6,34E-02	6,690890836	1,05E+00	3,06E-01	6,03E-01	Cox5a	0,96	No
ENSMUSG00000000093	-8,99E-01	-1,448582065	2,73E+00	9,83E-02	3,91E-01	Tbx2	0,54	No
ENSMUSG00000000120	1,27E+00	-2,548168699	1,00E+00	3,17E-01	6,17E-01	Ngfr	2,42	No
ENSMUSG00000000125	-1,87E+00	-3,107637761	1,39E+00	2,38E-01	5,50E-01	Wnt3	0,27	No
ENSMUSG00000000126	1,91E+00	-1,289628039	1,13E+01	7,78E-04	1,31E-02	Wnt9a	3,76	Yes
ENSMUSG00000000127	1,04E-01	0,633479095	1,83E-01	6,69E-01	8,87E-01	Fer	1,07	No
ENSMUSG00000000131	-8,13E-02	6,67713327	1,72E+00	1,89E-01	5,50E-01	Xpo6	0,95	No
ENSMUSG00000000142	-1,37E+00	-2,655113917	1,18E+00	2,77E-01	5,73E-01	Axin2	0,39	No
ENSMUSG00000000148	9,23E-02	4,777312769	1,47E+00	2,25E-01	5,50E-01	Brat1	1,07	No
ENSMUSG00000000149	6,09E-02	6,356978578	9,19E-01	3,38E-01	6,38E-01	Gna12	1,04	No
ENSMUSG00000000154	-4,24E-05	2,477193902	1,09E-07	1,00E+00	1,00E+00	Slc22a18	1,00	No
ENSMUSG00000000157	-8,61E-01	1,935629181	3,30E+01	9,30E-09	6,50E-07	Itgb2l	0,55	Yes
ENSMUSG00000000159	6,02E-02	-2,126668009	6,05E-03	9,38E-01	1,00E+00	Igsf5	1,04	No
ENSMUSG00000000167	5,56E-03	-0,863779102	1,92E-04	9,89E-01	1,00E+00	Pih1d2	1,00	No
ENSMUSG00000000168	1,39E-01	5,810289666	4,34E+00	3,72E-02	2,29E-01	Dlat	1,10	No
ENSMUSG00000000171	3,47E-03	7,549630186	3,44E-03	9,53E-01	1,00E+00	Sdhd	1,00	No
ENSMUSG00000000182	-2,90E-01	0,010769698	9,00E-01	3,43E-01	6,43E-01	Fgf23	0,82	No
ENSMUSG00000000183	-1,89E+00	-3,1076441	1,42E+00	2,33E-01	5,50E-01	Fgf6	0,27	No
ENSMUSG00000000184	-7,83E-01	8,234256201	1,81E+02	2,32E-41	3,02E-38	Ccnd2	0,58	Yes
ENSMUSG00000000194	-8,88E-02	6,13123891	1,90E+00	1,68E-01	5,23E-01	Gpr107	0,94	No
ENSMUSG00000000202	3,53E-01	0,273945855	1,74E+00	1,87E-01	5,50E-01	Btbd17	1,28	No
ENSMUSG00000000204	-2,41E-01	1,738989451	2,23E+00	1,35E-01	4,63E-01	Slfn4	0,85	No
ENSMUSG00000000244	1,19E-01	5,135025123	2,67E+00	1,02E-01	3,95E-01	Tspan32	1,09	No

ENSMUSG00000000247	-4,03E-01	-0,641482997	1,22E+00	2,69E-01	5,65E-01	Lhx2	0,76	No
ENSMUSG00000000248	2,61E-01	0,202784655	1,01E+00	3,14E-01	6,15E-01	Clec2g	1,20	No
ENSMUSG00000000253	2,47E-01	4,092985592	7,91E+00	4,93E-03	5,54E-02	Gmpr	1,19	No
ENSMUSG00000000263	1,16E+00	-2,832034382	6,51E-01	4,20E-01	7,15E-01	Glra1	2,23	No
ENSMUSG00000000275	-1,04E-01	7,678322582	3,11E+00	7,77E-02	3,56E-01	Trim25	0,93	No
ENSMUSG00000000276	-9,48E-02	5,012243647	1,62E+00	2,04E-01	5,50E-01	Dgke	0,94	No
ENSMUSG00000000278	-5,53E-02	7,399701047	8,63E-01	3,53E-01	6,54E-01	Scpep1	0,96	No
ENSMUSG00000000282	1,23E-02	6,008539884	3,53E-02	8,51E-01	9,90E-01	Mnt	1,01	No
ENSMUSG00000000290	1,42E-01	10,67574807	6,43E+00	1,12E-02	1,02E-01	Itgb2	1,10	No
ENSMUSG00000000295	3,97E-02	2,564597266	9,29E-02	7,61E-01	9,37E-01	Hddc2	1,03	No
ENSMUSG00000000296	-6,05E-01	-2,83199595	2,89E-01	5,91E-01	8,42E-01	Tpd52l1	0,66	No
ENSMUSG00000000301	-4,73E-02	0,489976317	3,59E-02	8,50E-01	9,89E-01	Pemt	0,97	No
ENSMUSG00000000303	2,51E-01	8,222098482	1,87E+01	1,54E-05	4,90E-04	Cdh1	1,19	Yes
ENSMUSG00000000305	-4,08E-02	-2,89610675	1,04E-03	9,74E-01	1,00E+00	Cdh4	0,97	No
ENSMUSG00000000308	-9,41E-01	-1,378244425	3,58E+00	5,84E-02	2,98E-01	Ckmt1	0,52	No
ENSMUSG00000000317	-7,91E-02	-2,448704688	8,71E-03	9,26E-01	1,00E+00	Bcl6b	0,95	No
ENSMUSG00000000318	4,42E-01	7,002355747	5,20E+01	5,63E-13	7,09E-11	Clec10a	1,36	Yes
ENSMUSG00000000320	-1,02E+00	-1,711687383	2,68E+00	1,02E-01	3,94E-01	Alox12	0,49	No
ENSMUSG00000000325	4,48E-02	2,715520169	1,35E-01	7,14E-01	9,12E-01	Arvcf	1,03	No
ENSMUSG00000000326	1,32E-01	7,062837901	4,72E+00	2,98E-02	1,98E-01	Comt	1,10	No
ENSMUSG00000000339	-1,66E-02	4,419808104	4,10E-02	8,40E-01	9,83E-01	Rtca	0,99	No
ENSMUSG00000000340	9,29E-02	4,62138579	1,38E+00	2,40E-01	5,50E-01	Dbt	1,07	No
ENSMUSG00000000346	-3,61E-02	8,443005831	3,93E-01	5,31E-01	8,05E-01	Dazap2	0,98	No
ENSMUSG00000000359	8,34E-01	0,330783698	9,75E+00	1,80E-03	2,57E-02	Rem1	1,78	Yes
ENSMUSG00000000365	-3,35E-01	-0,006722016	1,30E+00	2,54E-01	5,50E-01	Rnf17	0,79	No
ENSMUSG00000000374	-7,95E-03	6,75785231	1,66E-02	8,97E-01	1,00E+00	Trappc10	0,99	No
ENSMUSG00000000378	-2,52E-02	5,483872858	1,33E-01	7,16E-01	9,13E-01	Ccm2	0,98	No
ENSMUSG00000000384	1,23E-01	5,985838263	3,48E+00	6,20E-02	3,10E-01	Tbrg4	1,09	No
ENSMUSG00000000385	1,91E+00	-2,268183397	4,44E+00	3,52E-02	2,22E-01	Tmprss2	3,76	No
ENSMUSG00000000386	-4,51E-01	8,13368984	6,04E+01	7,57E-15	1,14E-12	Mx1	0,73	Yes
ENSMUSG00000000392	-2,27E-01	-1,226212718	2,30E-01	6,32E-01	8,64E-01	Fap	0,85	No
ENSMUSG00000000394	-1,94E+00	-3,107619243	1,50E+00	2,21E-01	5,50E-01	Gcg	0,26	No

ENSMUSG00000000399	1,20E-01	5,261674853	2,83E+00	9,25E-02	3,83E-01	Ndufa9		1,09	No
ENSMUSG00000000409	-3,19E-01	-0,460697765	8,26E-01	3,63E-01	6,64E-01	Lck		0,80	No
ENSMUSG00000000411	1,57E+00	-2,226288841	2,68E+00	1,01E-01	3,94E-01	Tssk3		2,97	No
ENSMUSG00000000416	-7,67E-01	-2,600663846	6,61E-01	4,16E-01	7,11E-01	Cttnbp2		0,59	No
ENSMUSG00000000420	5,39E-02	7,547889163	8,33E-01	3,61E-01	6,63E-01	Galnt1		1,04	No
ENSMUSG00000000439	7,45E-02	5,651503431	1,20E+00	2,72E-01	5,69E-01	Mkrn2		1,05	No
ENSMUSG00000000440	2,64E-01	3,980533495	8,47E+00	3,62E-03	4,40E-02	Pparg		1,20	Yes
ENSMUSG00000000441	6,66E-02	6,508212728	1,13E+00	2,88E-01	5,84E-01	Raf1		1,05	No
ENSMUSG00000000486	1,69E-01	1,483580572	8,92E-01	3,45E-01	6,46E-01		37135	1,12	No
ENSMUSG00000000489	1,86E-01	4,891067918	6,27E+00	1,23E-02	1,09E-01	Pdgfb		1,14	No
ENSMUSG00000000530	3,50E-01	5,84397482	2,73E+01	1,78E-07	9,38E-06	Acvrl1		1,27	Yes
ENSMUSG00000000531	-5,02E-01	4,119014022	3,34E+01	7,63E-09	5,41E-07	Grasp		0,71	Yes
ENSMUSG00000000532	1,20E-01	5,829464282	3,24E+00	7,19E-02	3,40E-01	Acvr1b		1,09	No
ENSMUSG00000000538	1,12E-01	5,099323116	2,36E+00	1,24E-01	4,41E-01	Tom1l2		1,08	No
ENSMUSG00000000544	-5,61E-01	-2,832027494	2,46E-01	6,20E-01	8,56E-01	Gpa33		0,68	No
ENSMUSG00000000552	1,89E-01	6,915769716	9,54E+00	2,01E-03	2,82E-02	Zfp385a		1,14	Yes
ENSMUSG00000000555	1,46E-01	6,748682203	5,61E+00	1,79E-02	1,40E-01	Itga5		1,11	No
ENSMUSG00000000561	2,36E-02	4,582230626	8,94E-02	7,65E-01	9,39E-01	Wdr77		1,02	No
ENSMUSG00000000562	2,70E-01	4,348132907	1,05E+01	1,22E-03	1,90E-02	Adora3		1,21	Yes
ENSMUSG00000000563	-8,32E-02	6,92935404	1,87E+00	1,72E-01	5,28E-01	Atp5f1		0,94	No
ENSMUSG00000000567	2,60E+00	-2,963275374	2,63E+00	1,05E-01	3,97E-01	Sox9		6,08	No
ENSMUSG00000000568	3,40E-02	6,531334506	2,95E-01	5,87E-01	8,40E-01	Hnrnpd		1,02	No
ENSMUSG00000000579	-1,60E-04	3,361828204	2,38E-06	9,99E-01	1,00E+00	Dynlt1c		1,00	No
ENSMUSG00000000581	7,73E-02	5,032271989	1,10E+00	2,95E-01	5,91E-01	C1d		1,06	No
ENSMUSG00000000594	1,99E-01	9,857856971	1,25E+01	4,06E-04	7,71E-03	Gm2a		1,15	Yes
ENSMUSG00000000600	-1,96E-01	5,990314186	9,06E+00	2,61E-03	3,45E-02	Krit1		0,87	Yes
ENSMUSG00000000605	1,38E-01	6,536205683	4,88E+00	2,72E-02	1,86E-01	Clcn4		1,10	No
ENSMUSG00000000617	-9,59E-01	-2,770584991	8,07E-01	3,69E-01	6,70E-01	Grm6		0,51	No
ENSMUSG00000000627	6,94E-01	1,493061941	1,29E+01	3,35E-04	6,63E-03	Sema4f		1,62	Yes
ENSMUSG00000000628	1,14E-01	6,578787797	3,33E+00	6,80E-02	3,28E-01	Hk2		1,08	No
ENSMUSG00000000631	-3,47E-02	6,881098495	3,22E-01	5,70E-01	8,31E-01	Myo18a		0,98	No
ENSMUSG00000000632	-1,94E+00	-3,107619243	1,50E+00	2,21E-01	5,50E-01	Sez6		0,26	No

ENSMUSG00000000673	-1,45E-01	4,029543771	2,79E+00	9,51E-02	3,86E-01	Haao	0,90	No
ENSMUSG00000000682	6,93E-02	9,112989329	1,49E+00	2,22E-01	5,50E-01	Cd52	1,05	No
ENSMUSG00000000686	2,09E-01	2,989883605	3,46E+00	6,30E-02	3,13E-01	Abhd15	1,16	No
ENSMUSG00000000690	2,63E+00	-2,96325892	2,67E+00	1,02E-01	3,94E-01	Hoxb6	6,19	No
ENSMUSG00000000693	-5,26E-01	4,006789733	3,61E+01	1,89E-09	1,48E-07	Loxl3	0,69	Yes
ENSMUSG00000000706	-4,93E-01	-2,401345968	3,88E-01	5,33E-01	8,06E-01	Btn1a1	0,71	No
ENSMUSG00000000708	1,13E-01	5,160222655	2,43E+00	1,19E-01	4,30E-01	Kat2b	1,08	No
ENSMUSG00000000711	3,65E-03	7,197002891	3,70E-03	9,52E-01	1,00E+00	Rab5b	1,00	No
ENSMUSG00000000724	-3,59E+00	-2,600616999	5,79E+00	1,62E-02	1,32E-01	Cryba1	0,08	No
ENSMUSG00000000730	-3,77E-02	0,422270197	2,32E-02	8,79E-01	1,00E+00	Dnmt3l	0,97	No
ENSMUSG00000000731	-2,28E+00	-1,980466897	8,26E+00	4,06E-03	4,78E-02	Aire	0,21	Yes
ENSMUSG00000000732	9,20E-02	6,308704884	2,11E+00	1,47E-01	4,85E-01	Icosl	1,07	No
ENSMUSG00000000738	-2,62E-02	5,567632955	1,47E-01	7,01E-01	9,05E-01	Spg7	0,98	No
ENSMUSG00000000739	9,01E-03	-3,107646452	-5,16E-10	1,00E+00	1,00E+00	Sult5a1	1,01	No
ENSMUSG00000000740	-1,59E-01	5,638895885	5,49E+00	1,91E-02	1,47E-01	Rpl13	0,90	No
ENSMUSG00000000743	1,34E-02	7,121169159	4,92E-02	8,24E-01	9,73E-01	Chmp1a	1,01	No
ENSMUSG00000000751	3,56E-02	6,511189026	3,24E-01	5,69E-01	8,30E-01	Rpa1	1,03	No
ENSMUSG00000000753	7,22E-01	1,786241379	1,70E+01	3,64E-05	1,04E-03	Serpinf1	1,65	Yes
ENSMUSG00000000759	-2,11E-02	5,485533861	9,21E-02	7,62E-01	9,38E-01	Tubgcp3	0,99	No
ENSMUSG00000000766	-6,30E-01	-1,711617634	1,12E+00	2,91E-01	5,86E-01	Oprm1	0,65	No
ENSMUSG00000000776	2,67E-03	4,110616583	9,38E-04	9,76E-01	1,00E+00	Polr3d	1,00	No
ENSMUSG00000000782	-1,44E+00	3,337298062	1,90E+02	2,56E-43	4,03E-40	Tcf7	0,37	Yes
ENSMUSG00000000791	-6,58E-01	0,01081518	4,42E+00	3,55E-02	2,23E-01	Il12rb1	0,63	No
ENSMUSG00000000792	-2,88E-02	-2,963268425	3,79E-04	9,84E-01	1,00E+00	Slc5a5	0,98	No
ENSMUSG00000000794	2,57E-01	4,254929214	8,98E+00	2,73E-03	3,56E-02	Kcnn3	1,20	Yes
ENSMUSG00000000804	1,99E-02	6,226429903	9,63E-02	7,56E-01	9,35E-01	Usp32	1,01	No
ENSMUSG00000000805	3,77E-01	5,184875004	2,58E+01	3,79E-07	1,85E-05	Car4	1,30	Yes
ENSMUSG00000000811	6,39E-02	2,488355035	2,33E-01	6,29E-01	8,62E-01	Txnrd3	1,05	No
ENSMUSG00000000817	6,96E-01	-2,832055333	2,64E-01	6,07E-01	8,50E-01	Fasl	1,62	No
ENSMUSG00000000823	-8,50E-02	5,6298955	1,57E+00	2,10E-01	5,50E-01	Zfp512b	0,94	No
ENSMUSG00000000826	-6,52E-02	8,15081918	1,27E+00	2,60E-01	5,54E-01	Dnajc5	0,96	No
ENSMUSG00000000827	-1,27E-01	5,894692139	3,74E+00	5,33E-02	2,82E-01	Tpd52l2	0,92	No

ENSMUSG00000000861	5,92E-01	1,615766512	1,23E+01	4,49E-04	8,39E-03	Bcl11a	1,51	Yes
ENSMUSG00000000869	1,70E-01	-1,015875471	1,62E-01	6,87E-01	8,98E-01	Il4	1,13	No
ENSMUSG00000000876	1,12E-01	4,355013851	1,83E+00	1,76E-01	5,35E-01	Pxmp4	1,08	No
ENSMUSG00000000884	1,70E-01	1,491261718	9,26E-01	3,36E-01	6,37E-01	Gnb1l	1,13	No
ENSMUSG00000000889	-4,36E-01	-2,65508115	1,94E-01	6,60E-01	8,82E-01	Dbh	0,74	No
ENSMUSG00000000901	1,65E-01	2,440612056	1,55E+00	2,14E-01	5,50E-01	Mmp11	1,12	No
ENSMUSG00000000902	6,00E-02	5,774072596	8,06E-01	3,69E-01	6,71E-01	Smarcb1	1,04	No
ENSMUSG00000000903	4,64E-01	-2,655147743	1,68E-01	6,82E-01	8,94E-01	Vpreb3	1,38	No
ENSMUSG00000000915	-1,58E-01	4,43589906	3,80E+00	5,14E-02	2,76E-01	Hip1r	0,90	No
ENSMUSG00000000916	1,85E-02	3,700736515	3,79E-02	8,46E-01	9,87E-01	Nsun5	1,01	No
ENSMUSG00000000934	2,62E-01	2,723929225	4,63E+00	3,13E-02	2,05E-01	Top1mt	1,20	No
ENSMUSG00000000938	-1,86E+00	-3,033612668	1,39E+00	2,38E-01	5,50E-01	Hoxa10	0,28	No
ENSMUSG00000000942	-1,59E-01	-1,697338208	5,42E-02	8,16E-01	9,69E-01	Hoxa4	0,90	No
ENSMUSG00000000957	2,40E-01	7,198477646	1,59E+01	6,81E-05	1,76E-03	Mmp14	1,18	Yes
ENSMUSG00000000958	-7,81E-02	5,223607023	1,21E+00	2,71E-01	5,68E-01	Slc7a7	0,95	No
ENSMUSG00000000959	-3,36E-02	5,559860551	2,41E-01	6,24E-01	8,58E-01	Oxa1l	0,98	No
ENSMUSG00000000976	2,07E-02	5,560569372	9,10E-02	7,63E-01	9,38E-01	Heatr6	1,01	No
ENSMUSG00000000982	-4,73E-02	7,519878311	6,24E-01	4,29E-01	7,23E-01	Ccl3	0,97	No
ENSMUSG00000000983	2,29E-01	1,927719628	2,15E+00	1,42E-01	4,76E-01	Wfdc18	1,17	No
ENSMUSG00000001014	-6,28E-01	-1,951864368	8,06E-01	3,69E-01	6,71E-01	Icam4	0,65	No
ENSMUSG00000001016	-2,98E-02	5,169571286	1,71E-01	6,79E-01	8,93E-01	Ilf2	0,98	No
ENSMUSG00000001017	-5,16E-02	6,523028749	6,80E-01	4,10E-01	7,06E-01	Chtop	0,96	No
ENSMUSG00000001018	-2,98E-02	5,769436172	1,98E-01	6,56E-01	8,80E-01	Snapin	0,98	No
ENSMUSG00000001020	-9,65E-02	8,228362618	2,79E+00	9,51E-02	3,86E-01	S100a4	0,94	No
ENSMUSG00000001021	-1,42E+00	-2,49755461	1,67E+00	1,96E-01	5,50E-01	S100a3	0,37	No
ENSMUSG00000001023	-1,15E+00	-1,03349037	7,07E+00	7,84E-03	7,79E-02	S100a5	0,45	No
ENSMUSG00000001025	2,00E-02	8,260764831	1,19E-01	7,30E-01	9,21E-01	S100a6	1,01	No
ENSMUSG00000001027	-1,93E+00	-2,963274349	1,47E+00	2,25E-01	5,50E-01	Scn4a	0,26	No
ENSMUSG00000001029	8,88E-02	2,341405269	4,17E-01	5,19E-01	7,98E-01	Icam2	1,06	No
ENSMUSG00000001034	-6,18E-04	5,499246401	8,03E-05	9,93E-01	1,00E+00	Mapk7	1,00	No
ENSMUSG00000001036	-3,80E-01	-1,929247818	3,35E-01	5,63E-01	8,26E-01	Epn2	0,77	No
ENSMUSG00000001039	2,00E-01	-2,268092392	6,01E-02	8,06E-01	9,64E-01	B9d1	1,15	No

ENSMUSG00000001052	-8,65E-02	6,566763861	1,92E+00	1,66E-01	5,19E-01	Sec24b	0,94	No
ENSMUSG00000001053	3,97E-01	4,919741132	2,84E+01	1,01E-07	5,64E-06	N4bp3	1,32	Yes
ENSMUSG00000001054	2,25E-01	7,072811784	1,38E+01	2,04E-04	4,43E-03	Rmnd5b	1,17	Yes
ENSMUSG00000001056	6,21E-02	5,760203064	8,54E-01	3,55E-01	6,57E-01	Nhp2	1,04	No
ENSMUSG00000001062	2,91E-02	6,135937622	2,04E-01	6,52E-01	8,77E-01	Vps9d1	1,02	No
ENSMUSG00000001065	5,81E-02	5,413575414	6,96E-01	4,04E-01	7,01E-01	Zfp276	1,04	No
ENSMUSG00000001082	1,51E-01	5,66601768	4,89E+00	2,70E-02	1,84E-01	Mfsd10	1,11	No
ENSMUSG00000001089	1,49E-01	6,75952532	5,84E+00	1,57E-02	1,30E-01	Luzp1	1,11	No
ENSMUSG00000001095	-1,21E-01	0,168166012	1,89E-01	6,63E-01	8,84E-01	Slc13a2	0,92	No
ENSMUSG00000001098	1,03E-01	6,391935335	2,66E+00	1,03E-01	3,95E-01	Kctd10	1,07	No
ENSMUSG00000001100	5,34E-02	4,831276012	4,91E-01	4,83E-01	7,71E-01	Poldip2	1,04	No
ENSMUSG00000001103	-2,65E+00	-3,033633091	2,78E+00	9,55E-02	3,86E-01	Sebox	0,16	No
ENSMUSG00000001105	-2,70E-02	4,382446698	1,08E-01	7,42E-01	9,27E-01	Ift20	0,98	No
ENSMUSG00000001119	-1,81E+00	-0,816121349	1,63E+01	5,54E-05	1,48E-03	Col6a1	0,29	Yes
ENSMUSG00000001120	6,32E-01	-0,548226242	2,84E+00	9,20E-02	3,82E-01	Pcbp3	1,55	No
ENSMUSG00000001123	1,11E-02	6,579605607	3,18E-02	8,59E-01	9,94E-01	Lgals9	1,01	No
ENSMUSG00000001138	2,41E-01	5,18752163	1,11E+01	8,46E-04	1,41E-02	Cnnm3	1,18	Yes
ENSMUSG00000001143	1,37E-01	5,424880506	3,84E+00	5,01E-02	2,71E-01	Lman2l	1,10	No
ENSMUSG00000001150	7,55E-02	6,159161943	1,37E+00	2,42E-01	5,50E-01	Mcm3ap	1,05	No
ENSMUSG00000001151	5,75E-02	6,081713649	7,81E-01	3,77E-01	6,78E-01	Pcnt	1,04	No
ENSMUSG00000001156	-5,45E-01	6,042128461	7,01E+01	5,50E-17	9,99E-15	Mxd1	0,69	Yes
ENSMUSG00000001157	5,42E-02	4,833211711	5,11E-01	4,75E-01	7,63E-01	Gmcl1	1,04	No
ENSMUSG00000001158	-1,42E-01	3,871685512	2,40E+00	1,21E-01	4,35E-01	Snrnp27	0,91	No
ENSMUSG00000001166	3,55E-01	2,216607417	6,38E+00	1,16E-02	1,04E-01	Oas1c	1,28	No
ENSMUSG00000001168	1,11E+00	-2,627669497	1,17E+00	2,79E-01	5,75E-01	Oas1h	2,16	No
ENSMUSG00000001175	-9,81E-02	9,77438571	3,03E+00	8,15E-02	3,66E-01	Calm1	0,93	No
ENSMUSG00000001211	1,67E-01	4,99787202	5,05E+00	2,46E-02	1,73E-01	Agpat3	1,12	No
ENSMUSG00000001227	3,42E-01	4,19599088	1,58E+01	6,89E-05	1,77E-03	Sema6b	1,27	Yes
ENSMUSG00000001228	2,22E-01	6,056801026	1,16E+01	6,65E-04	1,16E-02	Uhrf1	1,17	Yes
ENSMUSG00000001229	1,00E-01	6,543778799	2,56E+00	1,10E-01	4,06E-01	Dpp9	1,07	No
ENSMUSG00000001240	-1,58E-03	4,596728191	3,96E-04	9,84E-01	1,00E+00	Ramp2	1,00	No
ENSMUSG00000001247	1,09E-01	4,418996283	1,75E+00	1,86E-01	5,50E-01	Lsr	1,08	No

ENSMUSG00000001248	-7,50E-02	6,187172147	1,37E+00	2,42E-01	5,50E-01	Gramd1a	0,95	No
ENSMUSG00000001249	4,15E-01	0,97063462	3,79E+00	5,15E-02	2,76E-01	Hpn	1,33	No
ENSMUSG00000001270	1,02E-01	6,004075554	2,41E+00	1,20E-01	4,32E-01	Ckb	1,07	No
ENSMUSG00000001280	-5,05E-02	7,173714168	7,06E-01	4,01E-01	6,99E-01	Sp1	0,97	No
ENSMUSG00000001281	-5,95E-02	6,566431962	9,04E-01	3,42E-01	6,42E-01	Itgb7	0,96	No
ENSMUSG00000001285	-5,28E-02	4,17494807	3,83E-01	5,36E-01	8,08E-01	Myg1	0,96	No
ENSMUSG00000001288	8,83E-02	4,810195401	1,35E+00	2,46E-01	5,50E-01	Rarg	1,06	No
ENSMUSG00000001289	-1,86E-01	4,881233924	6,11E+00	1,34E-02	1,16E-01	Pfdn5	0,88	No
ENSMUSG00000001300	-4,32E-01	3,515017364	1,96E+01	9,69E-06	3,27E-04	Efnb2	0,74	Yes
ENSMUSG00000001305	9,40E-02	3,570386082	8,92E-01	3,45E-01	6,46E-01	Rrp15	1,07	No
ENSMUSG00000001313	2,06E-01	5,317959519	8,32E+00	3,92E-03	4,66E-02	Rnd2	1,15	Yes
ENSMUSG00000001323	2,49E-02	4,346248637	9,08E-02	7,63E-01	9,39E-01	Srr	1,02	No
ENSMUSG00000001333	2,68E-01	0,313492464	9,66E-01	3,26E-01	6,26E-01	Sync	1,20	No
ENSMUSG00000001334	2,93E-01	4,381575345	1,27E+01	3,63E-04	7,06E-03	Fndc5	1,23	Yes
ENSMUSG00000001348	-2,33E-01	6,069186477	1,28E+01	3,39E-04	6,68E-03	Acp5	0,85	Yes
ENSMUSG00000001349	-1,33E+00	-2,497607802	1,11E+00	2,92E-01	5,88E-01	Cnn1	0,40	No
ENSMUSG00000001366	1,35E-01	4,602860561	2,91E+00	8,82E-02	3,79E-01	Fbxo9	1,10	No
ENSMUSG00000001376	1,91E-01	5,527324403	7,73E+00	5,43E-03	5,94E-02	Vps50	1,14	No
ENSMUSG00000001380	7,07E-02	5,683277283	1,09E+00	2,97E-01	5,93E-01	Hars	1,05	No
ENSMUSG00000001383	-1,95E-01	5,732128027	8,44E+00	3,66E-03	4,44E-02	Zmat2	0,87	Yes
ENSMUSG00000001403	1,52E-01	5,211993839	4,45E+00	3,49E-02	2,21E-01	Ube2c	1,11	No
ENSMUSG00000001415	9,76E-02	6,600354663	2,45E+00	1,17E-01	4,24E-01	Smg5	1,07	No
ENSMUSG00000001416	-5,68E-02	6,785531177	8,54E-01	3,56E-01	6,57E-01	Cct3	0,96	No
ENSMUSG00000001418	5,22E-02	7,502508559	7,75E-01	3,79E-01	6,79E-01	Glmp	1,04	No
ENSMUSG00000001419	-2,42E-02	6,437253635	1,46E-01	7,02E-01	9,05E-01	Mef2d	0,98	No
ENSMUSG00000001420	-6,16E-02	1,21629521	1,03E-01	7,48E-01	9,30E-01	Tmem79	0,96	No
ENSMUSG00000001424	-1,93E-01	8,18421999	1,11E+01	8,56E-04	1,42E-02	Snd1	0,87	Yes
ENSMUSG00000001435	6,88E-01	-1,448745814	1,71E+00	1,91E-01	5,50E-01	Col18a1	1,61	No
ENSMUSG00000001436	3,31E-01	3,449972749	1,07E+01	1,09E-03	1,73E-02	Slc19a1	1,26	Yes
ENSMUSG00000001440	4,45E-02	7,770169218	5,78E-01	4,47E-01	7,38E-01	Kpnb1	1,03	No
ENSMUSG00000001441	-3,70E-01	6,727955811	3,60E+01	1,97E-09	1,53E-07	Npepps	0,77	Yes
ENSMUSG00000001444	5,60E-01	-2,497697211	3,49E-01	5,55E-01	8,20E-01	Tbx21	1,47	No

ENSMUSG00000001445	-8,58E-03	5,24132993	1,44E-02	9,04E-01	1,00E+00	Mrpl10	0,99	No
ENSMUSG00000001467	2,43E-01	7,002946449	1,60E+01	6,38E-05	1,67E-03	Cyp51	1,18	Yes
ENSMUSG00000001472	1,74E-02	7,945170816	8,88E-02	7,66E-01	9,40E-01	Tcf25	1,01	No
ENSMUSG00000001473	-1,59E-01	7,058216735	6,88E+00	8,72E-03	8,45E-02	Tubb6	0,90	No
ENSMUSG00000001482	1,31E-01	5,803041883	3,81E+00	5,10E-02	2,75E-01	Def8	1,09	No
ENSMUSG00000001493	4,41E-01	-2,832010046	1,52E-01	6,97E-01	9,03E-01	Meox1	1,36	No
ENSMUSG00000001494	1,85E+00	-3,107657832	1,35E+00	2,46E-01	5,50E-01	Sost	3,61	No
ENSMUSG00000001504	-1,87E+00	-3,107637761	1,39E+00	2,38E-01	5,50E-01	Irx2	0,27	No
ENSMUSG00000001506	-8,87E-01	-1,051570434	3,63E+00	5,66E-02	2,93E-01	Col1a1	0,54	No
ENSMUSG00000001507	6,22E-02	1,116137167	9,74E-02	7,55E-01	9,34E-01	Itga3	1,04	No
ENSMUSG00000001508	-1,40E+00	-2,711716435	1,23E+00	2,68E-01	5,65E-01	Sgca	0,38	No
ENSMUSG00000001510	-1,04E-02	-2,963271219	-1,18E-10	1,00E+00	1,00E+00	Dlx3	0,99	No
ENSMUSG00000001517	2,95E-01	5,519540707	1,82E+01	1,96E-05	6,09E-04	Foxm1	1,23	Yes
ENSMUSG00000001518	1,61E-01	2,775218612	1,82E+00	1,77E-01	5,37E-01	Itfg2	1,12	No
ENSMUSG00000001520	1,54E-01	-0,704656548	1,62E-01	6,88E-01	8,98E-01	Nrip2	1,11	No
ENSMUSG00000001521	2,28E-01	1,232814664	1,44E+00	2,30E-01	5,50E-01	Tulp3	1,17	No
ENSMUSG00000001524	5,26E-02	3,0426905	2,22E-01	6,38E-01	8,68E-01	Gtf2h4	1,04	No
ENSMUSG00000001525	9,69E-02	9,92266551	2,96E+00	8,51E-02	3,75E-01	Tubb5	1,07	No
ENSMUSG00000001542	-1,49E-02	5,557918721	4,66E-02	8,29E-01	9,76E-01	Ell2	0,99	No
ENSMUSG00000001552	8,47E-03	7,035526512	1,96E-02	8,89E-01	1,00E+00	Jup	1,01	No
ENSMUSG00000001555	-1,97E+00	-2,088609552	6,40E+00	1,14E-02	1,03E-01	Fkbp10	0,26	No
ENSMUSG00000001558	-6,14E-01	-2,355662258	5,13E-01	4,74E-01	7,62E-01	Klhl10	0,65	No
ENSMUSG00000001569	1,35E-01	5,215287023	3,53E+00	6,03E-02	3,04E-01	Nom1	1,10	No
ENSMUSG00000001576	2,11E-02	6,549203871	1,14E-01	7,35E-01	9,24E-01	Ergic1	1,01	No
ENSMUSG00000001583	5,15E-02	-0,855711034	1,45E-02	9,04E-01	1,00E+00	Tnk1	1,04	No
ENSMUSG00000001588	1,73E-01	6,663673585	7,73E+00	5,43E-03	5,94E-02	Acap1	1,13	No
ENSMUSG00000001604	1,77E-02	3,934652544	3,90E-02	8,43E-01	9,85E-01	Tcea3	1,01	No
ENSMUSG00000001627	-1,09E-02	6,389244236	2,96E-02	8,63E-01	9,97E-01	Ifrd1	0,99	No
ENSMUSG00000001630	-1,87E-01	4,30587998	5,04E+00	2,47E-02	1,74E-01	Stk38l	0,88	No
ENSMUSG00000001632	1,15E-01	6,719974304	3,42E+00	6,46E-02	3,18E-01	Brpf1	1,08	No
ENSMUSG00000001642	1,18E-01	4,199353266	1,88E+00	1,70E-01	5,26E-01	Akr1b3	1,09	No
ENSMUSG00000001663	-1,51E-01	-0,561126783	1,90E-01	6,63E-01	8,83E-01	Gstt1	0,90	No

ENSMUSG00000001665	-2,66E-01	3,224715267	6,47E+00	1,10E-02	9,98E-02	Gstt3	0,83	No
ENSMUSG00000001666	-2,25E-03	4,014580306	6,49E-04	9,80E-01	1,00E+00	Ddt	1,00	No
ENSMUSG00000001674	-1,34E-01	4,168585091	2,48E+00	1,16E-01	4,21E-01	Ddx18	0,91	No
ENSMUSG00000001687	1,87E-01	7,683213026	1,00E+01	1,54E-03	2,28E-02	Ubl3	1,14	Yes
ENSMUSG00000001700	-3,86E-01	1,292227715	4,43E+00	3,54E-02	2,23E-01	Gramd3	0,77	No
ENSMUSG00000001707	1,04E-01	3,920544204	1,32E+00	2,51E-01	5,50E-01	Eef1e1	1,08	No
ENSMUSG00000001729	3,80E-02	8,216344771	4,32E-01	5,11E-01	7,94E-01	Akt1	1,03	No
ENSMUSG00000001739	7,71E-02	-1,378325692	2,77E-02	8,68E-01	1,00E+00	Cldn15	1,05	No
ENSMUSG00000001741	3,69E-01	3,650912801	1,52E+01	9,53E-05	2,33E-03	Il16	1,29	Yes
ENSMUSG00000001750	8,91E-02	9,138870138	2,46E+00	1,17E-01	4,23E-01	Tcirg1	1,06	No
ENSMUSG00000001751	1,40E-01	6,430836546	4,92E+00	2,65E-02	1,82E-01	Naglu	1,10	No
ENSMUSG00000001755	4,54E-02	4,595321786	3,32E-01	5,65E-01	8,27E-01	Coasy	1,03	No
ENSMUSG00000001761	8,06E-02	0,345080382	8,62E-02	7,69E-01	9,42E-01	Smo	1,06	No
ENSMUSG00000001763	-3,07E-01	4,757061746	1,65E+01	4,96E-05	1,35E-03	Tspan33	0,81	Yes
ENSMUSG00000001767	5,33E-02	4,877102051	5,01E-01	4,79E-01	7,66E-01	Crnkl1	1,04	No
ENSMUSG00000001768	1,28E-01	6,460876501	4,11E+00	4,26E-02	2,45E-01	Rin2	1,09	No
ENSMUSG00000001774	-5,94E-02	5,491340562	7,46E-01	3,88E-01	6,88E-01	Chordc1	0,96	No
ENSMUSG00000001783	-7,45E-02	6,458367443	1,41E+00	2,35E-01	5,50E-01	Rtcb	0,95	No
ENSMUSG00000001785	-2,78E-03	4,534375112	1,21E-03	9,72E-01	1,00E+00	Pwp1	1,00	No
ENSMUSG00000001786	-1,86E-01	4,698978548	5,77E+00	1,63E-02	1,33E-01	Fbxo7	0,88	No
ENSMUSG00000001794	8,23E-02	8,090451165	2,01E+00	1,56E-01	5,01E-01	Capns1	1,06	No
ENSMUSG00000001802	-1,06E-01	-3,033634182	5,86E-03	9,39E-01	1,00E+00	Lrp3	0,93	No
ENSMUSG00000001815	1,87E+00	-3,107639691	1,38E+00	2,40E-01	5,50E-01	Evx2	3,67	No
ENSMUSG00000001827	9,35E-01	-1,912633335	2,25E+00	1,34E-01	4,60E-01	Folr1	1,91	No
ENSMUSG00000001829	1,09E-01	3,891675131	1,43E+00	2,32E-01	5,50E-01	Clpb	1,08	No
ENSMUSG00000001833	9,85E-02	7,569170319	2,78E+00	9,56E-02	3,86E-01	39326	1,07	No
ENSMUSG00000001844	-7,10E-02	4,661858073	8,18E-01	3,66E-01	6,67E-01	Zdhhc4	0,95	No
ENSMUSG00000001847	1,19E-01	8,73779508	4,37E+00	3,65E-02	2,27E-01	Rac1	1,09	No
ENSMUSG00000001855	8,90E-02	6,39756332	1,99E+00	1,59E-01	5,06E-01	Nup214	1,06	No
ENSMUSG00000001864	-1,02E+00	-0,668880271	7,73E+00	5,43E-03	5,94E-02	Aif1l	0,49	No
ENSMUSG00000001865	-2,62E-01	-1,548151217	2,24E-01	6,36E-01	8,67E-01	Cpa3	0,83	No
ENSMUSG00000001870	-1,02E+00	-1,896135461	2,33E+00	1,27E-01	4,47E-01	Ltbp1	0,49	No

ENSMUSG00000001891	4,07E-02	6,394028342	4,13E-01	5,20E-01	7,99E-01	Ugp2	1,03	No
ENSMUSG00000001901	9,01E-03	-3,107646452	-5,16E-10	1,00E+00	1,00E+00	Kcnh6	1,01	No
ENSMUSG00000001909	9,55E-02	5,428574387	1,87E+00	1,71E-01	5,28E-01	Trmt1	1,07	No
ENSMUSG00000001910	2,27E-02	7,207552049	1,43E-01	7,06E-01	9,07E-01	Nacc1	1,02	No
ENSMUSG00000001911	2,02E-01	5,246368453	7,88E+00	5,00E-03	5,60E-02	Nfix	1,15	No
ENSMUSG00000001918	1,44E-01	7,293415849	5,82E+00	1,58E-02	1,31E-01	Slc1a5	1,11	No
ENSMUSG00000001930	2,89E-01	7,749988431	2,41E+01	9,18E-07	4,12E-05	Vwf	1,22	Yes
ENSMUSG00000001942	7,51E-02	5,478419518	1,17E+00	2,79E-01	5,76E-01	Siae	1,05	No
ENSMUSG00000001943	-5,69E-01	-2,600654611	2,54E-01	6,14E-01	8,53E-01	Vsig2	0,67	No
ENSMUSG00000001946	-1,29E+00	-0,412984588	1,46E+01	1,35E-04	3,10E-03	Esam	0,41	Yes
ENSMUSG00000001948	-1,53E-01	-0,497415348	2,22E-01	6,37E-01	8,68E-01	Spa17	0,90	No
ENSMUSG00000001983	8,07E-03	3,083103931	5,51E-03	9,41E-01	1,00E+00	Taco1	1,01	No
ENSMUSG00000001988	-5,54E-01	-0,574220852	2,19E+00	1,39E-01	4,70E-01	Npas1	0,68	No
ENSMUSG00000001995	5,15E-02	3,817843176	2,99E-01	5,84E-01	8,38E-01	Sipa1l2	1,04	No
ENSMUSG00000001998	2,54E-02	4,571612522	1,03E-01	7,49E-01	9,30E-01	Ap4e1	1,02	No
ENSMUSG00000001999	8,49E-02	5,725223291	1,58E+00	2,09E-01	5,50E-01	Blvra	1,06	No
ENSMUSG00000002017	-2,33E-02	4,837889201	9,43E-02	7,59E-01	9,36E-01	Fam98a	0,98	No
ENSMUSG00000002020	-6,41E-01	1,060797366	1,02E+01	1,37E-03	2,08E-02	Ltbp2	0,64	Yes
ENSMUSG00000002028	-1,41E-01	7,079384416	5,46E+00	1,94E-02	1,48E-01	Kmt2a	0,91	No
ENSMUSG00000002031	-1,86E-01	3,988384016	4,37E+00	3,67E-02	2,28E-01	Ift46	0,88	No
ENSMUSG00000002032	6,19E-01	0,095425711	4,57E+00	3,25E-02	2,10E-01	Tmem25	1,54	No
ENSMUSG00000002033	-2,62E+00	-0,912243029	3,75E+01	9,03E-10	7,43E-08	Cd3g	0,16	Yes
ENSMUSG00000002043	-3,06E-02	4,362325884	1,39E-01	7,10E-01	9,09E-01	Trappc6a	0,98	No
ENSMUSG00000002052	-5,53E-02	7,533797652	8,74E-01	3,50E-01	6,51E-01	Supt6	0,96	No
ENSMUSG00000002055	2,64E-01	5,471924776	1,44E+01	1,51E-04	3,41E-03	Spag5	1,20	Yes
ENSMUSG00000002058	7,29E-02	6,013491319	1,24E+00	2,66E-01	5,62E-01	Unc119	1,05	No
ENSMUSG00000002059	2,03E-01	1,993371923	1,71E+00	1,91E-01	5,50E-01	Rab34	1,15	No
ENSMUSG00000002064	-6,02E-02	5,726765759	8,01E-01	3,71E-01	6,72E-01	Sdf2	0,96	No
ENSMUSG00000002068	8,53E-02	5,305051752	1,45E+00	2,28E-01	5,50E-01	Ccne1	1,06	No
ENSMUSG00000002076	6,88E-01	-2,655154313	2,60E-01	6,10E-01	8,51E-01	Hsf2bp	1,61	No
ENSMUSG00000002083	1,69E-01	4,479845497	4,49E+00	3,41E-02	2,17E-01	Bbc3	1,12	No
ENSMUSG00000002100	1,56E-01	3,60500065	2,56E+00	1,10E-01	4,06E-01	Mybpc3	1,11	No

ENSMUSG00000002102	6,68E-02	6,503136582	1,13E+00	2,87E-01	5,83E-01	Psmc3	1,05	No
ENSMUSG00000002103	6,87E-03	6,290044508	1,16E-02	9,14E-01	1,00E+00	Acp2	1,00	No
ENSMUSG00000002104	8,43E-01	0,801083004	1,30E+01	3,06E-04	6,14E-03	Rapsn	1,79	Yes
ENSMUSG00000002105	7,22E-02	4,927157929	9,28E-01	3,35E-01	6,36E-01	Slc39a13	1,05	No
ENSMUSG00000002107	1,02E-01	7,283808227	2,92E+00	8,75E-02	3,78E-01	Celf2	1,07	No
ENSMUSG00000002108	2,77E-01	2,492112783	4,53E+00	3,32E-02	2,14E-01	Nr1h3	1,21	No
ENSMUSG00000002109	-1,42E-02	3,961136041	2,53E-02	8,74E-01	1,00E+00	Ddb2	0,99	No
ENSMUSG00000002111	5,65E-02	8,503196599	9,67E-01	3,25E-01	6,26E-01	Spi1	1,04	No
ENSMUSG00000002129	7,72E-02	6,163720768	1,43E+00	2,31E-01	5,50E-01	Sf3a1	1,05	No
ENSMUSG00000002147	1,06E-02	7,976978047	3,30E-02	8,56E-01	9,93E-01	Stat6	1,01	No
ENSMUSG00000002190	1,87E+00	-2,89607975	1,36E+00	2,44E-01	5,50E-01	Clgn	3,65	No
ENSMUSG00000002204	-1,46E-01	8,743103878	6,53E+00	1,06E-02	9,77E-02	Napsa	0,90	No
ENSMUSG00000002205	8,27E-02	5,049633438	1,27E+00	2,61E-01	5,54E-01	Vrk3	1,06	No
ENSMUSG00000002210	2,12E-01	4,808285887	7,68E+00	5,58E-03	6,06E-02	Smg9	1,16	No
ENSMUSG00000002221	3,00E-02	5,593194648	1,92E-01	6,61E-01	8,82E-01	Paxip1	1,02	No
ENSMUSG00000002222	-8,49E-02	7,433372256	2,05E+00	1,52E-01	4,94E-01	Rmnd5a	0,94	No
ENSMUSG00000002227	-5,15E-01	4,389563893	4,10E+01	1,51E-10	1,39E-08	Mov10	0,70	Yes
ENSMUSG00000002228	-3,75E-02	3,577920832	1,50E-01	6,99E-01	9,04E-01	Ppm1j	0,97	No
ENSMUSG00000002233	-3,05E-01	5,746514227	2,08E+01	5,06E-06	1,83E-04	Rhoc	0,81	Yes
ENSMUSG00000002249	-4,83E-02	-2,770561466	1,58E-03	9,68E-01	1,00E+00	Tead3	0,97	No
ENSMUSG00000002250	5,04E-02	5,925243855	5,85E-01	4,44E-01	7,36E-01	Ppard	1,04	No
ENSMUSG00000002257	7,62E-02	6,203567749	1,40E+00	2,36E-01	5,50E-01	Def6	1,05	No
ENSMUSG00000002265	5,74E-01	-2,600720996	3,65E-01	5,46E-01	8,14E-01	Peg3	1,49	No
ENSMUSG00000002274	1,52E-01	2,489385226	1,32E+00	2,51E-01	5,50E-01	Metrn	1,11	No
ENSMUSG00000002279	1,59E-01	4,450090414	3,80E+00	5,14E-02	2,76E-01	Lmf1	1,12	No
ENSMUSG00000002280	1,72E-01	4,89278075	5,26E+00	2,18E-02	1,59E-01	Narfl	1,13	No
ENSMUSG00000002289	4,72E-01	1,127723166	5,47E+00	1,94E-02	1,48E-01	Angptl4	1,39	No
ENSMUSG00000002297	1,27E-01	5,371466656	3,22E+00	7,29E-02	3,43E-01	Dbf4	1,09	No
ENSMUSG00000002307	3,26E-02	5,489621231	2,23E-01	6,37E-01	8,68E-01	Daxx	1,02	No
ENSMUSG00000002308	4,39E-02	3,136952072	1,62E-01	6,87E-01	8,98E-01	Cd320	1,03	No
ENSMUSG00000002319	1,03E-01	5,453460092	2,19E+00	1,39E-01	4,70E-01	Ipo4	1,07	No
ENSMUSG00000002320	3,44E-02	6,167851996	2,84E-01	5,94E-01	8,43E-01	Tm9sf1	1,02	No

ENSMUSG00000002324	-3,43E-01	-0,816321919	7,54E-01	3,85E-01	6,86E-01	Rec8	0,79	No
ENSMUSG00000002325	-1,51E-01	5,770872999	5,14E+00	2,34E-02	1,68E-01	Irf9	0,90	No
ENSMUSG00000002326	1,44E-01	5,148284065	3,98E+00	4,59E-02	2,56E-01	Gmpr2	1,10	No
ENSMUSG00000002329	-1,17E-01	4,707471363	2,34E+00	1,26E-01	4,45E-01	Mdp1	0,92	No
ENSMUSG00000002332	-1,46E-02	5,650768328	4,59E-02	8,30E-01	9,77E-01	Dhrs1	0,99	No
ENSMUSG00000002342	6,98E-02	4,55103112	7,68E-01	3,81E-01	6,82E-01	Tmem161a	1,05	No
ENSMUSG00000002343	2,82E-01	3,298990223	7,57E+00	5,94E-03	6,37E-02	Armc6	1,22	No
ENSMUSG00000002345	1,01E-02	3,950989305	1,29E-02	9,10E-01	1,00E+00	Borcs8	1,01	No
ENSMUSG00000002346	-2,24E-01	2,387697146	2,92E+00	8,78E-02	3,78E-01	Slc25a42	0,86	No
ENSMUSG00000002365	1,13E-01	5,757999421	2,82E+00	9,33E-02	3,83E-01	Snx9	1,08	No
ENSMUSG00000002372	5,85E-02	6,110271662	8,17E-01	3,66E-01	6,67E-01	Ranbp3	1,04	No
ENSMUSG00000002379	-1,61E-01	4,580463915	4,15E+00	4,16E-02	2,43E-01	Ndufa11	0,89	No
ENSMUSG00000002384	-2,96E-01	0,726908338	1,62E+00	2,03E-01	5,50E-01	Bmp8b	0,81	No
ENSMUSG00000002393	5,10E-02	4,978302211	4,72E-01	4,92E-01	7,78E-01	Nr2f6	1,04	No
ENSMUSG00000002395	-2,26E-01	5,401742827	1,05E+01	1,21E-03	1,90E-02	Use1	0,85	Yes
ENSMUSG00000002396	5,51E-02	4,697848124	5,05E-01	4,77E-01	7,65E-01	Ocel1	1,04	No
ENSMUSG00000002409	3,58E-02	3,918301642	1,56E-01	6,92E-01	9,01E-01	Dyrk1b	1,03	No
ENSMUSG00000002413	7,14E-02	5,839128786	1,15E+00	2,84E-01	5,80E-01	Braf	1,05	No
ENSMUSG00000002416	5,98E-02	3,259643115	3,25E-01	5,69E-01	8,30E-01	Ndufb2	1,04	No
ENSMUSG00000002428	1,71E-02	5,039307847	5,42E-02	8,16E-01	9,69E-01	Hltf	1,01	No
ENSMUSG00000002455	-1,07E-01	5,964090965	2,69E+00	1,01E-01	3,93E-01	Prpf6	0,93	No
ENSMUSG00000002458	-4,66E-02	6,332696161	5,38E-01	4,63E-01	7,52E-01	Rgs19	0,97	No
ENSMUSG00000002459	8,29E-01	-2,497589439	5,98E-01	4,39E-01	7,32E-01	Rgs20	1,78	No
ENSMUSG00000002475	1,16E-01	1,010426799	2,97E-01	5,86E-01	8,39E-01	Abhd3	1,08	No
ENSMUSG00000002477	3,24E-02	4,043975917	1,37E-01	7,11E-01	9,10E-01	Snrpd1	1,02	No
ENSMUSG00000002486	1,72E-01	3,299617846	2,69E+00	1,01E-01	3,92E-01	Tchp	1,13	No
ENSMUSG00000002489	2,19E-01	7,488934869	1,36E+01	2,32E-04	4,91E-03	Tiam1	1,16	Yes
ENSMUSG00000002496	3,21E-02	6,144490189	2,47E-01	6,19E-01	8,56E-01	Tsc2	1,02	No
ENSMUSG00000002500	-3,92E-01	1,299534582	4,35E+00	3,69E-02	2,28E-01	Rpl3l	0,76	No
ENSMUSG00000002504	-6,95E-01	3,329775256	4,53E+01	1,72E-11	1,78E-09	Slc9a3r2	0,62	Yes
ENSMUSG00000002524	5,94E-02	6,433723494	8,88E-01	3,46E-01	6,47E-01	Puf60	1,04	No
ENSMUSG00000002546	-3,56E-02	6,380912817	3,17E-01	5,73E-01	8,33E-01	Golga2	0,98	No

ENSMUSG00000002550	9,64E-02	4,200238686	1,30E+00	2,54E-01	5,50E-01	Uck1	1,07	No
ENSMUSG00000002565	-1,22E+00	3,841538277	1,73E+02	1,55E-39	1,78E-36	Scin	0,43	Yes
ENSMUSG00000002578	-7,31E-01	5,37290837	1,10E+02	9,15E-26	3,38E-23	Ikzf4	0,60	Yes
ENSMUSG00000002580	8,20E-03	4,206954514	9,15E-03	9,24E-01	1,00E+00	Mien1	1,01	No
ENSMUSG00000002588	1,82E+00	-3,033667329	1,31E+00	2,52E-01	5,50E-01	Pon1	3,54	No
ENSMUSG00000002602	2,33E-01	10,24825526	1,72E+01	3,31E-05	9,53E-04	Axl	1,18	Yes
ENSMUSG00000002603	1,69E-01	8,50266652	8,61E+00	3,34E-03	4,14E-02	Tgfb1	1,12	Yes
ENSMUSG00000002608	-1,45E-02	6,04645664	4,93E-02	8,24E-01	9,73E-01	Ccdc97	0,99	No
ENSMUSG00000002617	1,62E-01	2,91784544	2,04E+00	1,54E-01	4,97E-01	Zfp40	1,12	No
ENSMUSG00000002625	-3,61E-02	4,839562843	2,26E-01	6,35E-01	8,66E-01	Akap8l	0,98	No
ENSMUSG00000002635	2,11E-01	4,115432648	5,82E+00	1,59E-02	1,31E-01	Pdcd2l	1,16	No
ENSMUSG00000002658	-1,02E-01	6,166348569	2,52E+00	1,12E-01	4,13E-01	Gtf2f1	0,93	No
ENSMUSG00000002660	-7,32E-02	4,072610554	6,99E-01	4,03E-01	7,01E-01	Clpp	0,95	No
ENSMUSG00000002661	-1,17E-01	2,984434502	1,14E+00	2,86E-01	5,82E-01	Alkbh7	0,92	No
ENSMUSG00000002664	2,74E-01	-1,726285076	2,36E-01	6,27E-01	8,61E-01	Pspn	1,21	No
ENSMUSG00000002668	1,44E-01	5,282764952	4,12E+00	4,24E-02	2,45E-01	Dennd1c	1,10	No
ENSMUSG00000002679	2,01E-01	3,813927145	4,54E+00	3,31E-02	2,13E-01	Med6	1,15	No
ENSMUSG00000002688	-3,83E-02	-3,107651936	-7,91E-11	1,00E+00	1,00E+00	Prkd1	0,97	No
ENSMUSG00000002699	6,07E-02	6,470141407	9,30E-01	3,35E-01	6,36E-01	Lcp2	1,04	No
ENSMUSG00000002718	8,88E-02	6,314706781	1,95E+00	1,63E-01	5,13E-01	Cse1l	1,06	No
ENSMUSG00000002728	-4,60E-03	5,689956122	4,58E-03	9,46E-01	1,00E+00	Naa20	1,00	No
ENSMUSG00000002731	-1,59E-01	4,396618748	3,72E+00	5,36E-02	2,83E-01	Prkra	0,90	No
ENSMUSG00000002732	3,29E-01	1,156077234	2,70E+00	1,01E-01	3,92E-01	Fkbp7	1,26	No
ENSMUSG00000002733	1,59E-01	4,933114642	4,48E+00	3,42E-02	2,18E-01	Plekha3	1,12	No
ENSMUSG00000002741	2,27E-02	6,840727568	1,37E-01	7,11E-01	9,10E-01	Ykt6	1,02	No
ENSMUSG00000002748	8,46E-02	7,446973634	2,04E+00	1,53E-01	4,96E-01	Baz1b	1,06	No
ENSMUSG00000002763	6,95E-02	5,366032566	9,84E-01	3,21E-01	6,22E-01	Pex6	1,05	No
ENSMUSG00000002767	2,58E-02	4,69098258	1,08E-01	7,42E-01	9,27E-01	Mrpl2	1,02	No
ENSMUSG00000002768	-1,82E-01	5,178426521	6,42E+00	1,13E-02	1,02E-01	Mea1	0,88	No
ENSMUSG00000002769	-2,49E-02	0,554301439	1,07E-02	9,18E-01	1,00E+00	Gnmt	0,98	No
ENSMUSG00000002771	1,78E-01	0,099659166	4,23E-01	5,15E-01	7,97E-01	Grin2d	1,13	No
ENSMUSG00000002778	7,73E-03	6,460770789	1,51E-02	9,02E-01	1,00E+00	Kdelr1	1,01	No

ENSMUSG00000002781	8,59E-02	3,628660942	8,00E-01	3,71E-01	6,72E-01	Tmem143	1,06	No
ENSMUSG00000002791	1,82E+00	-3,033661385	1,31E+00	2,52E-01	5,50E-01	Pdxk-ps	3,54	No
ENSMUSG00000002797	-1,50E-01	3,050383936	1,87E+00	1,72E-01	5,29E-01	Ggct	0,90	No
ENSMUSG00000002799	2,77E-01	3,73483305	8,04E+00	4,58E-03	5,22E-02	Jag2	1,21	No
ENSMUSG00000002803	4,33E-02	3,811327621	2,21E-01	6,39E-01	8,69E-01	Btbd6	1,03	No
ENSMUSG00000002804	4,55E-02	3,326685919	1,88E-01	6,65E-01	8,85E-01	Nudt14	1,03	No
ENSMUSG00000002808	-7,75E-02	-0,683146721	4,47E-02	8,33E-01	9,78E-01	Epdr1	0,95	No
ENSMUSG00000002812	1,03E-02	7,687002558	3,05E-02	8,61E-01	9,96E-01	Flii	1,01	No
ENSMUSG00000002814	1,25E-01	4,421259065	2,33E+00	1,27E-01	4,46E-01	Top3a	1,09	No
ENSMUSG00000002820	5,25E-02	4,960955069	4,98E-01	4,80E-01	7,68E-01	Atg4d	1,04	No
ENSMUSG00000002825	2,29E-01	3,227704953	4,58E+00	3,24E-02	2,10E-01	Qtrt1	1,17	No
ENSMUSG00000002831	5,95E-01	0,111700855	4,20E+00	4,05E-02	2,38E-01	Plin4	1,51	No
ENSMUSG00000002833	-8,40E-02	5,564507937	1,50E+00	2,20E-01	5,50E-01	Hdgfl2	0,94	No
ENSMUSG00000002835	1,73E-01	5,363018445	6,09E+00	1,36E-02	1,17E-01	Chaf1a	1,13	No
ENSMUSG00000002844	-6,84E-02	6,605690048	1,21E+00	2,72E-01	5,68E-01	Adprh	0,95	No
ENSMUSG00000002845	-3,84E-01	5,277990198	2,95E+01	5,45E-08	3,22E-06	Tmem39a	0,77	Yes
ENSMUSG00000002846	2,98E-02	3,592566255	9,58E-02	7,57E-01	9,35E-01	Timmdc1	1,02	No
ENSMUSG00000002847	-8,67E-01	3,112581015	6,35E+01	1,59E-15	2,55E-13	Pla1a	0,55	Yes
ENSMUSG00000002870	1,28E-01	6,167239382	3,91E+00	4,80E-02	2,63E-01	Mcm2	1,09	No
ENSMUSG00000002871	-5,26E-02	5,544940129	5,89E-01	4,43E-01	7,34E-01	Tpra1	0,96	No
ENSMUSG00000002881	-7,96E-02	6,020873516	1,49E+00	2,23E-01	5,50E-01	Nab1	0,95	No
ENSMUSG00000002885	-2,66E-01	6,435026095	1,77E+01	2,65E-05	7,90E-04	Adgre5	0,83	Yes
ENSMUSG00000002897	3,79E-01	6,469131894	3,58E+01	2,16E-09	1,67E-07	Il17ra	1,30	Yes
ENSMUSG00000002900	-6,94E-01	-2,033628724	8,20E-01	3,65E-01	6,66E-01	Lamb1	0,62	No
ENSMUSG00000002908	4,05E-01	-2,574236739	1,95E-01	6,59E-01	8,81E-01	Kcnn1	1,32	No
ENSMUSG00000002910	3,37E-01	3,458618788	1,15E+01	7,12E-04	1,22E-02	Arrdc2	1,26	Yes
ENSMUSG00000002944	2,18E-01	8,864850929	1,46E+01	1,36E-04	3,12E-03	Cd36	1,16	Yes
ENSMUSG00000002948	2,72E-02	5,664597006	1,62E-01	6,88E-01	8,98E-01	Map2k7	1,02	No
ENSMUSG00000002949	3,62E-02	5,188608278	2,53E-01	6,15E-01	8,54E-01	Timm44	1,03	No
ENSMUSG00000002957	1,83E-01	7,045899231	9,14E+00	2,50E-03	3,33E-02	Ap2a2	1,14	Yes
ENSMUSG00000002963	1,64E-03	5,0225665	4,92E-04	9,82E-01	1,00E+00	Pnkp	1,00	No
ENSMUSG00000002968	-3,51E-02	5,896244592	2,82E-01	5,95E-01	8,44E-01	Med25	0,98	No

ENSMUSG00000002980	5,81E-02	2,930334223	2,50E-01	6,17E-01	8,55E-01	Bcam	1,04	No
ENSMUSG00000002981	-1,69E-02	6,94591729	7,71E-02	7,81E-01	9,49E-01	Clptm1	0,99	No
ENSMUSG00000002983	-7,01E-01	6,801135308	1,30E+02	3,45E-30	1,95E-27	Relb	0,62	Yes
ENSMUSG00000002984	1,15E-01	5,267359169	2,59E+00	1,07E-01	4,01E-01	Tomm40	1,08	No
ENSMUSG00000002985	-3,81E-01	4,083136942	1,93E+01	1,14E-05	3,77E-04	Apoe	0,77	Yes
ENSMUSG00000002992	-5,90E-01	0,750263578	7,02E+00	8,07E-03	7,95E-02	Apoc2	0,66	No
ENSMUSG00000002996	2,30E-03	6,176721784	1,28E-03	9,71E-01	1,00E+00	Hbp1	1,00	No
ENSMUSG00000002997	-1,29E-02	4,931571887	3,02E-02	8,62E-01	9,97E-01	Prkar2b	0,99	No
ENSMUSG00000003031	-1,90E-02	5,634955427	7,92E-02	7,78E-01	9,47E-01	Cdkn1b	0,99	No
ENSMUSG00000003032	2,98E-01	7,097355972	2,41E+01	9,15E-07	4,12E-05	Klf4	1,23	Yes
ENSMUSG00000003033	7,52E-02	6,385223196	1,41E+00	2,34E-01	5,50E-01	Ap1m1	1,05	No
ENSMUSG00000003037	-5,60E-02	6,796874653	8,35E-01	3,61E-01	6,62E-01	Rab8a	0,96	No
ENSMUSG00000003038	9,09E-02	6,884443303	2,20E+00	1,38E-01	4,68E-01	Hmgn2	1,07	No
ENSMUSG00000003039	-2,39E-01	6,749422997	1,52E+01	9,85E-05	2,39E-03	Fam32a	0,85	Yes
ENSMUSG00000003051	-8,24E-01	-2,711692991	3,73E-01	5,41E-01	8,11E-01	Elf3	0,57	No
ENSMUSG00000003053	1,81E+00	-3,10765737	1,28E+00	2,58E-01	5,50E-01	Cyp2c29	3,49	No
ENSMUSG00000003062	3,25E-02	5,160381283	2,04E-01	6,51E-01	8,77E-01	Stard3nl	1,02	No
ENSMUSG00000003068	7,08E-02	6,813002377	1,33E+00	2,49E-01	5,50E-01	Stk11	1,05	No
ENSMUSG00000003070	-9,89E-01	1,817606662	4,14E+01	1,26E-10	1,17E-08	Efna2	0,50	Yes
ENSMUSG00000003072	2,47E-02	7,317609703	1,71E-01	6,79E-01	8,93E-01	Atp5d	1,02	No
ENSMUSG00000003099	4,26E-02	5,716298354	3,99E-01	5,28E-01	8,04E-01	Ppp5c	1,03	No
ENSMUSG00000003119	-1,13E-01	6,026880085	3,02E+00	8,23E-02	3,69E-01	Cdk12	0,92	No
ENSMUSG00000003123	1,23E-02	6,188014366	3,71E-02	8,47E-01	9,87E-01	Lipe	1,01	No
ENSMUSG00000003131	3,50E-02	7,002093211	3,32E-01	5,64E-01	8,27E-01	Pafah1b2	1,02	No
ENSMUSG00000003134	-4,70E-01	7,078849933	6,06E+01	6,87E-15	1,06E-12	Tbc1d8	0,72	Yes
ENSMUSG00000003135	1,57E-02	5,091415573	4,59E-02	8,30E-01	9,77E-01	Cnot11	1,01	No
ENSMUSG00000003153	3,56E-01	3,242662566	1,12E+01	8,07E-04	1,36E-02	Slc2a3	1,28	Yes
ENSMUSG00000003154	-2,31E-02	6,091865928	1,27E-01	7,22E-01	9,16E-01	Foxj2	0,98	No
ENSMUSG00000003161	-2,52E-01	7,052883148	1,74E+01	3,07E-05	8,93E-04	Sri	0,84	Yes
ENSMUSG00000003166	9,47E-02	5,747403591	1,99E+00	1,58E-01	5,04E-01	Dgcr2	1,07	No
ENSMUSG00000003184	-1,48E-02	5,574766533	4,70E-02	8,28E-01	9,76E-01	Irf3	0,99	No
ENSMUSG00000003190	8,99E-02	3,187499842	7,12E-01	3,99E-01	6,98E-01	Bcl2l12	1,06	No

ENSMUSG00000003198	4,79E-02	2,567425411	1,42E-01	7,06E-01	9,08E-01	Zfp959		1,03	No
ENSMUSG00000003199	-7,78E-02	5,484094291	1,27E+00	2,60E-01	5,54E-01	Mpnd		0,95	No
ENSMUSG00000003200	9,62E-02	5,835473099	2,09E+00	1,49E-01	4,89E-01	Sh3gl1		1,07	No
ENSMUSG00000003206	2,02E-02	4,956779288	7,32E-02	7,87E-01	9,53E-01	Ebi3		1,01	No
ENSMUSG00000003208	7,02E-02	2,653161656	3,24E-01	5,69E-01	8,30E-01	Ccdc94		1,05	No
ENSMUSG00000003226	6,75E-02	8,064330069	1,35E+00	2,46E-01	5,50E-01	Ranbp2		1,05	No
ENSMUSG00000003227	1,81E+00	-3,10765737	1,28E+00	2,58E-01	5,50E-01	Edar		3,49	No
ENSMUSG00000003228	1,04E-01	4,848691506	1,85E+00	1,74E-01	5,32E-01	Grk5		1,07	No
ENSMUSG00000003233	2,78E-02	4,705919085	1,30E-01	7,18E-01	9,15E-01	Dvl3		1,02	No
ENSMUSG00000003234	1,99E-02	4,792072044	6,82E-02	7,94E-01	9,56E-01	Abcf3		1,01	No
ENSMUSG00000003235	-2,63E-03	5,52646154	1,46E-03	9,70E-01	1,00E+00	Eif2b5		1,00	No
ENSMUSG00000003269	-1,28E-01	5,085301129	3,05E+00	8,08E-02	3,64E-01	Cyth2		0,92	No
ENSMUSG00000003271	1,99E-01	-0,84769837	2,32E-01	6,30E-01	8,62E-01	Sult2b1		1,15	No
ENSMUSG00000003273	-6,16E-01	-2,311133661	5,17E-01	4,72E-01	7,60E-01	Car11		0,65	No
ENSMUSG00000003279	-2,46E-01	-1,997965238	1,19E-01	7,30E-01	9,21E-01	Dlgap1		0,84	No
ENSMUSG00000003282	-7,76E-02	2,492222944	3,74E-01	5,41E-01	8,11E-01	Plag1		0,95	No
ENSMUSG00000003283	-1,62E-01	6,313551992	6,56E+00	1,05E-02	9,65E-02	Hck		0,89	No
ENSMUSG00000003299	1,58E-01	5,269633673	4,90E+00	2,68E-02	1,84E-01	Mrpl4		1,12	No
ENSMUSG00000003308	6,08E-02	5,840420996	8,38E-01	3,60E-01	6,61E-01	Keap1		1,04	No
ENSMUSG00000003309	-8,90E-02	-2,146144347	1,28E-02	9,10E-01	1,00E+00	Ap1m2		0,94	No
ENSMUSG00000003316	3,49E-02	7,555350942	3,48E-01	5,55E-01	8,20E-01	Glg1		1,02	No
ENSMUSG00000003341	-1,71E+00	-2,497580728	3,23E+00	7,24E-02	3,41E-01	Atp8b3		0,31	No
ENSMUSG00000003344	5,79E-02	5,184060197	6,48E-01	4,21E-01	7,16E-01	Btbd2		1,04	No
ENSMUSG00000003345	5,36E-02	6,76841323	7,55E-01	3,85E-01	6,86E-01	Csnk1g2		1,04	No
ENSMUSG00000003346	2,48E-01	6,229521873	1,49E+01	1,16E-04	2,75E-03	Abhd17a		1,19	Yes
ENSMUSG00000003348	-2,51E-01	7,368968618	1,79E+01	2,39E-05	7,24E-04	Mob3a		0,84	Yes
ENSMUSG00000003352	-8,99E-01	8,396642398	2,41E+02	3,01E-54	1,00E-50	Cacnb3		0,54	Yes
ENSMUSG00000003354	7,63E-02	-0,614223057	4,13E-02	8,39E-01	9,83E-01	Ccdc65		1,05	No
ENSMUSG00000003355	1,22E-01	2,514066109	8,77E-01	3,49E-01	6,50E-01	Fkbp11		1,09	No
ENSMUSG00000003360	1,04E-01	6,471002204	2,72E+00	9,91E-02	3,92E-01	Ddx23		1,07	No
ENSMUSG00000003363	1,00E-01	8,659543751	3,05E+00	8,09E-02	3,65E-01	Pld3		1,07	No
ENSMUSG00000003378	-6,11E-01	1,100150272	9,89E+00	1,66E-03	2,42E-02	Grik5		0,65	Yes

ENSMUSG00000003379	-8,36E-01	-1,587318129	2,33E+00	1,27E-01	4,47E-01	Cd79a	0,56	No
ENSMUSG00000003380	2,04E-02	5,783636616	9,28E-02	7,61E-01	9,37E-01	Rabac1	1,01	No
ENSMUSG00000003382	-4,54E-01	9,159086145	6,37E+01	1,45E-15	2,35E-13	Etv3	0,73	Yes
ENSMUSG00000003402	-1,73E-02	6,242718153	7,25E-02	7,88E-01	9,53E-01	Prkcsh	0,99	No
ENSMUSG00000003410	-3,83E-02	-3,107651936	-7,91E-11	1,00E+00	1,00E+00	Elavl3	0,97	No
ENSMUSG00000003411	-1,72E+00	-2,770622427	2,06E+00	1,51E-01	4,93E-01	Rab3b	0,30	No
ENSMUSG00000003418	-7,98E-01	1,004090508	1,60E+01	6,26E-05	1,64E-03	St8sia6	0,57	Yes
ENSMUSG00000003420	3,70E-01	6,549370213	3,47E+01	3,83E-09	2,83E-07	Fcgrt	1,29	Yes
ENSMUSG00000003421	1,85E-01	5,076378985	6,33E+00	1,19E-02	1,06E-01	Nosip	1,14	No
ENSMUSG00000003423	2,09E-02	4,363034083	6,38E-02	8,01E-01	9,61E-01	Pih1d1	1,01	No
ENSMUSG00000003429	-2,63E-01	6,549707942	1,78E+01	2,50E-05	7,53E-04	Rps11	0,83	Yes
ENSMUSG00000003435	-7,98E-02	6,910379489	1,71E+00	1,91E-01	5,50E-01	Supt5	0,95	No
ENSMUSG00000003436	-3,19E+00	-2,896141896	4,27E+00	3,87E-02	2,33E-01	Dll3	0,11	No
ENSMUSG00000003437	-4,18E-02	5,716364957	3,87E-01	5,34E-01	8,07E-01	Paf1	0,97	No
ENSMUSG00000003438	1,03E-01	5,007443805	1,94E+00	1,64E-01	5,15E-01	Timm50	1,07	No
ENSMUSG00000003444	-1,44E-01	2,93829469	1,55E+00	2,13E-01	5,50E-01	Med29	0,91	No
ENSMUSG00000003452	-4,62E-01	1,379402468	6,38E+00	1,16E-02	1,04E-01	Bicd1	0,73	No
ENSMUSG00000003458	1,05E-01	7,031016671	2,99E+00	8,40E-02	3,71E-01	Ncstn	1,08	No
ENSMUSG00000003464	1,86E-02	6,536068619	8,86E-02	7,66E-01	9,40E-01	Pex19	1,01	No
ENSMUSG00000003469	-5,88E-02	-3,033616507	1,62E-03	9,68E-01	1,00E+00	Phyhip	0,96	No
ENSMUSG00000003476	-6,31E-02	-2,963274299	1,81E-03	9,66E-01	1,00E+00	Crhr2	0,96	No
ENSMUSG00000003484	1,88E-01	4,713732313	5,83E+00	1,58E-02	1,30E-01	Cyp4f18	1,14	No
ENSMUSG00000003500	2,41E-01	3,754938347	6,46E+00	1,10E-02	1,00E-01	Impdh1	1,18	No
ENSMUSG00000003518	1,28E-01	6,816216148	4,34E+00	3,72E-02	2,29E-01	Dusp3	1,09	No
ENSMUSG00000003526	-8,57E-02	2,65235784	4,85E-01	4,86E-01	7,73E-01	Prodh	0,94	No
ENSMUSG00000003527	-7,29E-02	4,238180226	7,46E-01	3,88E-01	6,88E-01	Dgcr14	0,95	No
ENSMUSG00000003528	9,25E-02	5,904553513	1,95E+00	1,62E-01	5,12E-01	Slc25a1	1,07	No
ENSMUSG00000003531	1,67E-02	3,334091548	2,60E-02	8,72E-01	1,00E+00	Dgcr6	1,01	No
ENSMUSG00000003534	-4,41E-02	2,471903524	1,22E-01	7,27E-01	9,19E-01	Ddr1	0,97	No
ENSMUSG00000003541	2,75E-01	6,787373028	1,99E+01	8,07E-06	2,77E-04	Ier3	1,21	Yes
ENSMUSG00000003545	9,62E-01	6,455282222	2,21E+02	6,03E-50	1,29E-46	Fosb	1,95	Yes
ENSMUSG00000003546	1,43E-01	5,588691661	4,36E+00	3,67E-02	2,28E-01	Klc4	1,10	No

ENSMUSG00000003549	-6,90E-02	3,288547915	4,40E-01	5,07E-01	7,91E-01	Ercc1	0,95	No
ENSMUSG00000003555	8,13E-01	-2,711753932	5,75E-01	4,48E-01	7,39E-01	Cyp17a1	1,76	No
ENSMUSG00000003559	-1,70E-01	2,624888393	1,89E+00	1,69E-01	5,23E-01	As3mt	0,89	No
ENSMUSG00000003573	1,09E-01	5,295923956	2,34E+00	1,26E-01	4,45E-01	Homer3	1,08	No
ENSMUSG00000003575	3,06E-02	4,307129251	1,34E-01	7,15E-01	9,12E-01	Crtc1	1,02	No
ENSMUSG00000003581	1,62E-01	4,388146511	3,87E+00	4,90E-02	2,67E-01	Rnf215	1,12	No
ENSMUSG00000003585	-1,04E-01	0,678103326	2,01E-01	6,54E-01	8,79E-01	Sec14l2	0,93	No
ENSMUSG00000003604	-3,76E-02	3,82375497	1,68E-01	6,82E-01	8,94E-01	Aven	0,97	No
ENSMUSG00000003617	-7,74E-02	1,477117051	2,01E-01	6,54E-01	8,79E-01	Cp	0,95	No
ENSMUSG00000003623	1,19E-01	4,797410561	2,44E+00	1,18E-01	4,28E-01	Crot	1,09	No
ENSMUSG00000003644	2,01E-01	7,057111518	1,10E+01	9,20E-04	1,51E-02	Rps6ka1	1,15	Yes
ENSMUSG00000003660	4,05E-02	7,662890464	4,75E-01	4,91E-01	7,77E-01	Snrnp200	1,03	No
ENSMUSG00000003662	-3,96E-02	4,982601142	2,85E-01	5,93E-01	8,43E-01	Ciao1	0,97	No
ENSMUSG00000003665	-5,66E-01	-2,448600914	2,51E-01	6,16E-01	8,55E-01	Has1	0,68	No
ENSMUSG00000003680	1,03E-01	5,459750807	2,20E+00	1,38E-01	4,68E-01	Taf6l	1,07	No
ENSMUSG00000003721	5,15E-02	4,414392093	3,98E-01	5,28E-01	8,04E-01	Insig2	1,04	No
ENSMUSG00000003731	1,20E-01	6,690897881	3,78E+00	5,18E-02	2,77E-01	Kpna6	1,09	No
ENSMUSG00000003746	-1,96E-01	6,37868608	9,62E+00	1,93E-03	2,72E-02	Man1a	0,87	Yes
ENSMUSG00000003752	-1,13E-01	2,887795544	9,69E-01	3,25E-01	6,26E-01	Itpkc	0,92	No
ENSMUSG00000003762	2,49E-01	3,714131398	6,89E+00	8,64E-03	8,38E-02	Coq8b	1,19	No
ENSMUSG00000003778	7,57E-02	6,024702859	1,34E+00	2,46E-01	5,50E-01	Brd8	1,05	No
ENSMUSG00000003779	2,02E-01	5,92860839	9,40E+00	2,17E-03	2,99E-02	Kif20a	1,15	Yes
ENSMUSG00000003808	-1,07E-02	5,591914835	2,42E-02	8,76E-01	1,00E+00	Farsa	0,99	No
ENSMUSG00000003809	7,51E-02	4,052266409	7,42E-01	3,89E-01	6,89E-01	Gcdh	1,05	No
ENSMUSG00000003810	1,64E-01	6,229061554	6,46E+00	1,10E-02	1,00E-01	Mast2	1,12	No
ENSMUSG00000003812	1,12E-01	6,906230956	3,37E+00	6,65E-02	3,24E-01	Dnase2a	1,08	No
ENSMUSG00000003813	-1,02E-01	5,761575127	2,30E+00	1,29E-01	4,52E-01	Rad23a	0,93	No
ENSMUSG00000003814	8,99E-02	9,859888202	2,55E+00	1,11E-01	4,08E-01	Calr	1,06	No
ENSMUSG00000003824	2,64E-02	3,421664446	6,78E-02	7,95E-01	9,56E-01	Syce2	1,02	No
ENSMUSG00000003847	-4,27E-01	7,969801043	5,36E+01	2,45E-13	3,24E-11	Nfat5	0,74	Yes
ENSMUSG00000003848	-2,80E-02	4,597681297	1,25E-01	7,24E-01	9,18E-01	Nob1	0,98	No
ENSMUSG00000003849	-3,07E-01	4,380705387	1,39E+01	1,92E-04	4,21E-03	Nqo1	0,81	Yes

ENSMUSG00000003863	7,40E-01	0,014984552	6,16E+00	1,30E-02	1,13E-01	Ppfia3		1,67	No
ENSMUSG00000003865	2,41E-01	5,69885663	1,27E+01	3,63E-04	7,07E-03	Gys1		1,18	Yes
ENSMUSG00000003868	2,04E-01	4,734342437	6,85E+00	8,88E-03	8,56E-02	Ruvbl2		1,15	No
ENSMUSG00000003872	6,18E-01	-0,863893494	2,08E+00	1,49E-01	4,90E-01	Lin7b		1,53	No
ENSMUSG00000003873	-3,80E-02	6,703790183	3,76E-01	5,39E-01	8,10E-01	Bax		0,97	No
ENSMUSG00000003882	-6,29E-02	7,420112895	1,12E+00	2,90E-01	5,86E-01	Il7r		0,96	No
ENSMUSG00000003923	1,23E-02	4,526388048	2,33E-02	8,79E-01	1,00E+00	Tfam		1,01	No
ENSMUSG00000003929	2,79E-01	2,853558077	5,83E+00	1,57E-02	1,30E-01	Zfp81		1,21	No
ENSMUSG00000003934	5,50E-02	-1,929316806	7,34E-03	9,32E-01	1,00E+00	Efnb3		1,04	No
ENSMUSG00000003948	-2,65E-01	4,913205543	1,26E+01	3,85E-04	7,41E-03	Mmd		0,83	Yes
ENSMUSG00000003949	-5,65E-01	-2,600623117	2,50E-01	6,17E-01	8,55E-01	Hlf		0,68	No
ENSMUSG00000003955	1,08E-01	4,720434289	1,91E+00	1,67E-01	5,20E-01	Fam162a		1,08	No
ENSMUSG00000003970	-4,59E-02	9,233901201	6,56E-01	4,18E-01	7,13E-01	Rpl8		0,97	No
ENSMUSG00000003974	1,81E+00	-3,10765737	1,28E+00	2,58E-01	5,50E-01	Grm3		3,49	No
ENSMUSG00000003992	-2,07E-01	1,59447747	1,50E+00	2,21E-01	5,50E-01	Ssbp2		0,87	No
ENSMUSG00000004018	-1,71E-02	3,011041073	2,38E-02	8,77E-01	1,00E+00	Fancl		0,99	No
ENSMUSG00000004032	-7,10E-03	-0,407368661	4,97E-04	9,82E-01	1,00E+00	Gstm5		1,00	No
ENSMUSG00000004035	6,18E-01	-2,268078597	4,25E-01	5,14E-01	7,96E-01	Gstm7		1,53	No
ENSMUSG00000004038	1,83E+00	-2,963282593	1,33E+00	2,50E-01	5,50E-01	Gstm3		3,56	No
ENSMUSG00000004040	-2,10E-01	7,639668655	1,27E+01	3,72E-04	7,19E-03	Stat3		0,86	Yes
ENSMUSG00000004043	-1,80E-01	6,790007436	8,68E+00	3,22E-03	4,04E-02	Stat5a		0,88	Yes
ENSMUSG00000004044	-1,77E-01	3,104616988	2,56E+00	1,09E-01	4,06E-01	Cavin1		0,88	No
ENSMUSG00000004054	9,48E-02	6,787916292	2,38E+00	1,23E-01	4,38E-01	Map3k11		1,07	No
ENSMUSG00000004056	-6,67E-02	6,008267695	1,04E+00	3,09E-01	6,07E-01	Akt2		0,95	No
ENSMUSG00000004069	8,28E-02	4,982905736	1,25E+00	2,63E-01	5,58E-01	Dnaja3		1,06	No
ENSMUSG00000004070	-8,71E-02	5,941793202	1,76E+00	1,85E-01	5,49E-01	Hmox2		0,94	No
ENSMUSG00000004071	1,07E-02	5,640376224	2,51E-02	8,74E-01	1,00E+00	Cdip1		1,01	No
ENSMUSG00000004085	1,17E-01	4,992919056	2,49E+00	1,15E-01	4,18E-01	Map3k20		1,08	No
ENSMUSG00000004096	-1,14E-01	5,787903564	2,88E+00	8,96E-02	3,80E-01	Cwc15		0,92	No
ENSMUSG00000004098	-2,04E+00	-1,86348382	9,12E+00	2,53E-03	3,36E-02	Col5a3		0,24	Yes
ENSMUSG00000004099	1,94E-01	7,306329558	1,05E+01	1,18E-03	1,86E-02	Dnmt1		1,14	Yes
ENSMUSG00000004100	1,17E-01	4,689875799	2,22E+00	1,36E-01	4,65E-01	Ppan		1,08	No

ENSMUSG00000004105	2,63E-01	5,922565907	1,55E+01	8,14E-05	2,04E-03	Angptl2	1,20	Yes
ENSMUSG00000004110	-4,98E-01	-1,424770807	6,01E-01	4,38E-01	7,31E-01	Cacna1e	0,71	No
ENSMUSG00000004113	3,93E-01	3,221714363	1,34E+01	2,55E-04	5,32E-03	Cacna1b	1,31	Yes
ENSMUSG00000004127	5,89E-02	2,12294877	1,73E-01	6,78E-01	8,92E-01	Trmt10a	1,04	No
ENSMUSG00000004151	7,54E-01	1,4800265	1,49E+01	1,12E-04	2,67E-03	Etv1	1,69	Yes
ENSMUSG00000004187	-1,73E-01	1,320791626	8,36E-01	3,61E-01	6,62E-01	Kifc2	0,89	No
ENSMUSG00000004207	2,00E-01	13,64429858	1,29E+01	3,35E-04	6,63E-03	Psap	1,15	Yes
ENSMUSG00000004233	2,38E-03	3,088335154	4,72E-04	9,83E-01	1,00E+00	Wars2	1,00	No
ENSMUSG00000004263	-8,00E-02	5,134629005	1,22E+00	2,70E-01	5,66E-01	Atn1	0,95	No
ENSMUSG00000004264	-5,13E-02	6,778991716	6,94E-01	4,05E-01	7,02E-01	Phb2	0,97	No
ENSMUSG00000004266	-2,13E-02	7,521493068	1,29E-01	7,19E-01	9,15E-01	Ptpn6	0,99	No
ENSMUSG00000004267	-5,82E-01	2,664278076	2,27E+01	1,88E-06	7,70E-05	Eno2	0,67	Yes
ENSMUSG00000004268	4,64E-02	5,52004161	4,49E-01	5,03E-01	7,88E-01	Emg1	1,03	No
ENSMUSG00000004270	2,75E-01	6,036918504	1,76E+01	2,76E-05	8,17E-04	Lpcat3	1,21	Yes
ENSMUSG00000004285	-2,70E-02	6,690051967	1,90E-01	6,63E-01	8,83E-01	Atp6v1f	0,98	No
ENSMUSG00000004296	-1,04E+00	3,070838541	8,73E+01	9,16E-21	2,18E-18	Il12b	0,49	Yes
ENSMUSG00000004319	1,31E-01	6,143157224	4,12E+00	4,23E-02	2,45E-01	Clcn3	1,10	No
ENSMUSG00000004328	1,24E+00	-2,311211973	2,09E+00	1,49E-01	4,89E-01	Hif3a	2,36	No
ENSMUSG00000004344	-1,87E+00	-3,107637761	1,39E+00	2,38E-01	5,50E-01	Gpx5	0,27	No
ENSMUSG00000004347	-1,70E-01	-1,333295691	1,18E-01	7,31E-01	9,22E-01	Pde1c	0,89	No
ENSMUSG00000004356	2,25E-01	5,784242585	1,13E+01	7,73E-04	1,31E-02	Utp20	1,17	Yes
ENSMUSG00000004359	-5,82E-01	0,52750837	5,87E+00	1,54E-02	1,28E-01	Spic	0,67	No
ENSMUSG00000004364	-1,25E-01	6,680488553	4,05E+00	4,40E-02	2,50E-01	Cul3	0,92	No
ENSMUSG00000004371	1,16E+00	-1,816474764	3,23E+00	7,23E-02	3,41E-01	Il11	2,23	No
ENSMUSG00000004383	-2,13E-01	5,666806798	9,95E+00	1,61E-03	2,35E-02	Large1	0,86	Yes
ENSMUSG00000004393	3,02E-02	4,862641882	1,60E-01	6,90E-01	8,99E-01	Ddx56	1,02	No
ENSMUSG00000004394	-2,04E-02	5,860545216	9,41E-02	7,59E-01	9,36E-01	Tmed4	0,99	No
ENSMUSG00000004446	3,84E-01	5,103925564	2,73E+01	1,73E-07	9,17E-06	Bid	1,31	Yes
ENSMUSG00000004451	6,71E-02	6,833761518	1,19E+00	2,75E-01	5,71E-01	Ralb	1,05	No
ENSMUSG00000004455	1,53E-02	6,059456993	5,50E-02	8,15E-01	9,68E-01	Ppp1cc	1,01	No
ENSMUSG00000004460	6,70E-02	6,927311617	1,20E+00	2,73E-01	5,69E-01	Dnajb11	1,05	No
ENSMUSG00000004462	1,17E-01	4,237575551	1,92E+00	1,66E-01	5,19E-01	Tbccd1	1,08	No

ENSMUSG00000004473	-5,01E-02	0,899320114	5,40E-02	8,16E-01	9,69E-01	Clec11a	0,97	No
ENSMUSG00000004500	3,95E-01	2,680003525	1,08E+01	9,89E-04	1,60E-02	Zfp324	1,31	Yes
ENSMUSG00000004508	5,96E-02	6,614184174	9,18E-01	3,38E-01	6,38E-01	Gab2	1,04	No
ENSMUSG00000004530	2,76E-01	7,946843694	2,24E+01	2,26E-06	9,02E-05	Coro1c	1,21	Yes
ENSMUSG00000004535	-1,17E-01	7,545101826	3,90E+00	4,83E-02	2,64E-01	Tax1bp1	0,92	No
ENSMUSG00000004552	3,93E-01	2,292024464	7,91E+00	4,91E-03	5,52E-02	Ctse	1,31	No
ENSMUSG00000004558	-3,41E-01	3,268483109	1,07E+01	1,09E-03	1,73E-02	Ndrg2	0,79	Yes
ENSMUSG00000004561	9,31E-02	4,548534984	1,37E+00	2,41E-01	5,50E-01	Mettl17	1,07	No
ENSMUSG00000004562	-1,72E-01	7,411260846	8,39E+00	3,77E-03	4,53E-02	Arhgef40	0,89	Yes
ENSMUSG00000004565	-8,75E-02	5,497498039	1,62E+00	2,03E-01	5,50E-01	Pnpla6	0,94	No
ENSMUSG00000004567	5,77E-02	6,109496228	7,94E-01	3,73E-01	6,74E-01	Mcoln1	1,04	No
ENSMUSG00000004568	1,08E-01	4,050853791	1,49E+00	2,22E-01	5,50E-01	Arhgef18	1,08	No
ENSMUSG00000004591	-1,10E-01	5,74824178	2,71E+00	9,99E-02	3,92E-01	Pkn2	0,93	No
ENSMUSG00000004609	3,89E-01	8,396696808	4,53E+01	1,67E-11	1,74E-09	Cd33	1,31	Yes
ENSMUSG00000004610	1,47E-01	5,833687388	4,85E+00	2,77E-02	1,87E-01	Etfb	1,11	No
ENSMUSG00000004612	-5,50E-01	-2,548167825	2,37E-01	6,26E-01	8,60E-01	Nkg7	0,68	No
ENSMUSG00000004626	1,40E-02	6,934109688	5,28E-02	8,18E-01	9,70E-01	Stxbp2	1,01	No
ENSMUSG00000004630	8,87E-02	0,392569298	1,26E-01	7,23E-01	9,17E-01	Pcp2	1,06	No
ENSMUSG00000004631	2,46E-01	-2,548244856	7,65E-02	7,82E-01	9,50E-01	Sgce	1,19	No
ENSMUSG00000004633	6,39E-02	4,657269545	6,65E-01	4,15E-01	7,10E-01	Chn2	1,05	No
ENSMUSG00000004637	-3,82E-02	2,950809204	1,13E-01	7,37E-01	9,25E-01	Wwox	0,97	No
ENSMUSG00000004642	1,33E-01	5,140058244	3,40E+00	6,52E-02	3,20E-01	Slbp	1,10	No
ENSMUSG00000004655	7,32E-01	-2,770630356	2,92E-01	5,89E-01	8,41E-01	Aqp1	1,66	No
ENSMUSG00000004661	1,22E-02	4,455143152	2,28E-02	8,80E-01	1,00E+00	Arid3b	1,01	No
ENSMUSG00000004665	-4,59E-01	7,604700485	6,06E+01	7,14E-15	1,08E-12	Cnn2	0,73	Yes
ENSMUSG00000004667	-1,39E-01	5,864305602	4,38E+00	3,63E-02	2,26E-01	Polr2e	0,91	No
ENSMUSG00000004668	-5,85E-02	-0,497794647	2,94E-02	8,64E-01	9,97E-01	Abca13	0,96	No
ENSMUSG00000004677	-5,43E-02	8,855565789	9,08E-01	3,41E-01	6,41E-01	Myo9b	0,96	No
ENSMUSG00000004698	-3,95E-03	2,684555083	1,04E-03	9,74E-01	1,00E+00	Hdac9	1,00	No
ENSMUSG00000004707	4,45E-02	5,605673853	4,26E-01	5,14E-01	7,96E-01	Ly9	1,03	No
ENSMUSG00000004709	1,56E-01	5,86827344	5,59E+00	1,81E-02	1,41E-01	Cd244	1,11	No
ENSMUSG00000004730	-7,70E-02	7,102502746	1,62E+00	2,04E-01	5,50E-01	Adgre1	0,95	No

ENSMUSG00000004748	4,56E-01	0,731617125	3,98E+00	4,60E-02	2,56E-01	Mtfp1	1,37	No
ENSMUSG00000004768	1,37E-01	3,125106909	1,59E+00	2,07E-01	5,50E-01	Rab23	1,10	No
ENSMUSG00000004771	5,86E-03	6,600449287	8,88E-03	9,25E-01	1,00E+00	Rab11a	1,00	No
ENSMUSG00000004788	-1,39E-01	5,644446263	4,18E+00	4,10E-02	2,40E-01	Eif2b2	0,91	No
ENSMUSG00000004789	-3,36E-02	7,476536336	3,22E-01	5,71E-01	8,31E-01	Dlst	0,98	No
ENSMUSG00000004791	-3,32E-01	4,853405932	1,88E+01	1,42E-05	4,56E-04	Pgf	0,79	Yes
ENSMUSG00000004798	3,71E-02	5,899782165	3,17E-01	5,74E-01	8,33E-01	Ulk2	1,03	No
ENSMUSG00000004814	4,26E-01	-1,655066518	6,48E-01	4,21E-01	7,16E-01	Ccl24	1,34	No
ENSMUSG00000004815	6,31E-02	4,692416281	6,66E-01	4,14E-01	7,10E-01	Dgkq	1,04	No
ENSMUSG00000004837	4,25E-01	3,798640771	2,12E+01	4,18E-06	1,55E-04	Grap	1,34	Yes
ENSMUSG00000004842	-1,92E+00	-2,963214917	1,46E+00	2,27E-01	5,50E-01	Pou1f1	0,26	No
ENSMUSG00000004843	8,49E-05	6,306999074	1,78E-06	9,99E-01	1,00E+00	Chmp2b	1,00	No
ENSMUSG00000004846	1,26E-01	6,475653694	3,97E+00	4,63E-02	2,57E-01	Plod3	1,09	No
ENSMUSG00000004849	3,36E-02	6,631852792	2,92E-01	5,89E-01	8,41E-01	Ap1s1	1,02	No
ENSMUSG00000004864	-4,15E-01	4,00277918	2,23E+01	2,37E-06	9,41E-05	Mapk13	0,75	Yes
ENSMUSG00000004865	-4,32E-02	5,858023238	4,27E-01	5,14E-01	7,96E-01	Srpk1	0,97	No
ENSMUSG00000004880	6,41E-03	5,378101479	8,33E-03	9,27E-01	1,00E+00	Lbr	1,00	No
ENSMUSG00000004885	1,35E+00	-2,548284495	1,86E+00	1,73E-01	5,30E-01	Crabp2	2,55	No
ENSMUSG00000004891	6,89E-01	3,024569209	3,68E+01	1,30E-09	1,04E-07	Nes	1,61	Yes
ENSMUSG00000004894	-1,87E+00	-3,107637761	1,39E+00	2,38E-01	5,50E-01	Hapln2	0,27	No
ENSMUSG00000004895	7,70E-03	5,104447423	1,13E-02	9,15E-01	1,00E+00	Prcc	1,01	No
ENSMUSG00000004896	-1,63E-02	4,726436025	4,49E-02	8,32E-01	9,78E-01	Rrnad1	0,99	No
ENSMUSG00000004897	-2,25E-03	7,653194206	1,46E-03	9,70E-01	1,00E+00	Hdgf	1,00	No
ENSMUSG00000004902	4,40E-02	1,11029187	4,62E-02	8,30E-01	9,77E-01	Slc25a18	1,03	No
ENSMUSG00000004929	1,84E-01	4,470376895	5,12E+00	2,36E-02	1,69E-01	Thop1	1,14	No
ENSMUSG00000004931	1,58E-01	4,800436821	4,24E+00	3,95E-02	2,36E-01	Apba3	1,12	No
ENSMUSG00000004933	9,06E-02	4,309427944	1,19E+00	2,76E-01	5,72E-01	Matk	1,06	No
ENSMUSG00000004934	7,02E-02	4,821434535	8,47E-01	3,57E-01	6,59E-01	Pias4	1,05	No
ENSMUSG00000004936	7,92E-03	6,861751727	1,68E-02	8,97E-01	1,00E+00	Map2k1	1,01	No
ENSMUSG00000004937	6,11E-02	6,092616257	8,86E-01	3,47E-01	6,47E-01	Sgta	1,04	No
ENSMUSG00000004945	-7,40E-02	4,983655401	9,94E-01	3,19E-01	6,19E-01	Tmem242	0,95	No
ENSMUSG00000004947	6,71E-02	4,29160109	6,44E-01	4,22E-01	7,18E-01	Dtx2	1,05	No

ENSMUSG00000004951	-8,82E-01	-1,963171952	1,40E+00	2,37E-01	5,50E-01	Hspb1	0,54	No
ENSMUSG00000004952	5,01E-02	5,83244449	5,68E-01	4,51E-01	7,41E-01	Rasa4	1,04	No
ENSMUSG00000004961	1,19E-01	-2,146142839	2,79E-02	8,67E-01	9,99E-01	Syt5	1,09	No
ENSMUSG00000004980	-1,56E-02	9,057178532	7,56E-02	7,83E-01	9,50E-01	Hnrnpa2b1	0,99	No
ENSMUSG00000004988	-2,50E-01	-1,226298777	2,49E-01	6,18E-01	8,55E-01	Fxyd4	0,84	No
ENSMUSG00000004994	1,89E-01	3,393564754	3,47E+00	6,24E-02	3,11E-01	Ccdc130	1,14	No
ENSMUSG00000004996	2,30E-01	4,545365532	8,36E+00	3,83E-03	4,58E-02	Mri1	1,17	Yes
ENSMUSG00000005034	1,47E-01	6,754442128	5,64E+00	1,75E-02	1,38E-01	Prkacb	1,11	No
ENSMUSG00000005043	4,63E-01	3,877216933	2,54E+01	4,67E-07	2,24E-05	Sgsh	1,38	Yes
ENSMUSG00000005045	5,24E-01	3,316359829	2,50E+01	5,61E-07	2,65E-05	Chd5	1,44	Yes
ENSMUSG00000005054	2,55E-02	7,994104329	1,92E-01	6,61E-01	8,82E-01	Cstb	1,02	No
ENSMUSG00000005057	2,20E-01	3,771048218	5,56E+00	1,83E-02	1,43E-01	Sh2b2	1,16	No
ENSMUSG00000005069	-2,73E-02	5,075780024	1,40E-01	7,08E-01	9,09E-01	Pex5	0,98	No
ENSMUSG00000005078	1,59E-01	4,274135533	3,56E+00	5,91E-02	3,01E-01	Jkamp	1,12	No
ENSMUSG00000005087	6,42E-02	9,116195627	1,28E+00	2,59E-01	5,51E-01	Cd44	1,05	No
ENSMUSG00000005089	-1,04E+00	-1,024477021	5,06E+00	2,45E-02	1,72E-01	Slc1a2	0,49	No
ENSMUSG00000005102	9,21E-03	5,644295344	1,83E-02	8,92E-01	1,00E+00	Eif2ak4	1,01	No
ENSMUSG00000005103	-1,66E-01	9,13716327	8,51E+00	3,54E-03	4,32E-02	Wdr1	0,89	Yes
ENSMUSG00000005107	2,21E-01	2,40277082	2,74E+00	9,76E-02	3,90E-01	Slc2a9	1,17	No
ENSMUSG00000005124	-5,29E-01	-2,770601222	2,18E-01	6,41E-01	8,70E-01	Wisp1	0,69	No
ENSMUSG00000005125	-1,71E-01	6,197494772	7,10E+00	7,69E-03	7,68E-02	Ndrg1	0,89	No
ENSMUSG00000005131	-1,45E+00	-1,832037058	3,75E+00	5,28E-02	2,80E-01	4930550C14Rik	0,37	No
ENSMUSG00000005142	1,44E-01	9,047585765	6,44E+00	1,11E-02	1,01E-01	Man2b1	1,11	No
ENSMUSG00000005148	6,66E-01	-2,268144305	6,42E-01	4,23E-01	7,18E-01	Klf5	1,59	No
ENSMUSG00000005150	-4,28E-02	3,953666819	2,27E-01	6,34E-01	8,66E-01	Wdr83	0,97	No
ENSMUSG00000005161	-8,65E-02	6,767248524	1,98E+00	1,59E-01	5,07E-01	Prdx2	0,94	No
ENSMUSG00000005198	1,09E-01	8,250278328	3,53E+00	6,01E-02	3,03E-01	Polr2a	1,08	No
ENSMUSG00000005204	1,38E-01	6,232577608	4,66E+00	3,08E-02	2,03E-01	Senp3	1,10	No
ENSMUSG00000005220	3,07E-01	-2,033638744	1,73E-01	6,77E-01	8,92E-01	Corin	1,24	No
ENSMUSG00000005225	-1,66E-01	3,712341337	3,09E+00	7,87E-02	3,59E-01	Plekha8	0,89	No
ENSMUSG00000005233	8,49E-02	4,671263566	1,17E+00	2,79E-01	5,75E-01	Spc25	1,06	No
ENSMUSG00000005237	-1,02E+00	3,223433955	9,40E+01	3,22E-22	9,19E-20	Dnah2	0,49	Yes

ENSMUSG00000005251	-1,50E+00	-0,0154297	2,60E+01	3,42E-07	1,69E-05	Ripk4	0,35	Yes
ENSMUSG00000005262	-2,22E-02	5,149376538	9,31E-02	7,60E-01	9,37E-01	Ufd1	0,98	No
ENSMUSG00000005267	2,09E-01	3,17794084	3,85E+00	4,98E-02	2,70E-01	Zfp287	1,16	No
ENSMUSG00000005268	-2,69E+00	-2,929262057	2,87E+00	9,03E-02	3,81E-01	Prlr	0,16	No
ENSMUSG00000005299	1,98E-02	5,957876111	9,10E-02	7,63E-01	9,38E-01	Letm1	1,01	No
ENSMUSG00000005312	2,52E-02	7,060160623	1,73E-01	6,77E-01	8,92E-01	Ubqln1	1,02	No
ENSMUSG00000005338	-5,43E-01	-0,205589127	3,24E+00	7,17E-02	3,39E-01	Cadm3	0,69	No
ENSMUSG00000005339	-2,65E+00	-3,033633091	2,78E+00	9,55E-02	3,86E-01	Fcer1a	0,16	No
ENSMUSG00000005354	9,27E-02	7,048526662	2,34E+00	1,26E-01	4,45E-01	Txn2	1,07	No
ENSMUSG00000005360	-2,33E+00	-2,35556104	7,29E+00	6,95E-03	7,13E-02	Slc1a3	0,20	No
ENSMUSG00000005362	-7,19E-02	4,903460626	9,21E-01	3,37E-01	6,38E-01	Crbn	0,95	No
ENSMUSG00000005364	1,14E+00	-2,600685118	1,24E+00	2,65E-01	5,60E-01	Il5ra	2,21	No
ENSMUSG00000005370	1,40E-01	5,035797829	3,65E+00	5,62E-02	2,91E-01	Msh6	1,10	No
ENSMUSG00000005371	-1,65E-01	5,673459188	5,97E+00	1,46E-02	1,23E-01	Fbxo11	0,89	No
ENSMUSG00000005373	-3,57E+00	-2,711650889	5,74E+00	1,66E-02	1,34E-01	Mlxipl	0,08	No
ENSMUSG00000005374	1,61E-01	4,592183696	4,07E+00	4,36E-02	2,49E-01	Tbl2	1,12	No
ENSMUSG00000005378	-1,17E-02	4,574339624	2,15E-02	8,84E-01	1,00E+00	Wbscr22	0,99	No
ENSMUSG00000005397	-1,14E+00	0,678328311	2,53E+01	4,86E-07	2,32E-05	Nid1	0,46	Yes
ENSMUSG00000005410	1,21E-01	6,64550219	3,79E+00	5,16E-02	2,76E-01	Mcm5	1,09	No
ENSMUSG00000005413	1,13E-01	7,581279181	3,68E+00	5,50E-02	2,87E-01	Hmox1	1,08	No
ENSMUSG00000005417	-1,03E-01	6,78571301	2,81E+00	9,39E-02	3,84E-01	Mprip	0,93	No
ENSMUSG00000005442	8,27E-02	8,379184831	2,06E+00	1,51E-01	4,93E-01	Cic	1,06	No
ENSMUSG00000005447	-2,02E-01	4,097635415	5,45E+00	1,96E-02	1,49E-01	Pafah1b3	0,87	No
ENSMUSG00000005465	-3,62E-01	2,74289028	9,12E+00	2,53E-03	3,36E-02	Il27ra	0,78	Yes
ENSMUSG00000005469	1,21E-01	6,141136868	3,55E+00	5,95E-02	3,02E-01	Prkaca	1,09	No
ENSMUSG00000005470	2,37E-01	5,775018698	1,24E+01	4,31E-04	8,10E-03	Asf1b	1,18	Yes
ENSMUSG00000005474	8,81E-02	-1,205858293	3,44E-02	8,53E-01	9,91E-01	Myl10	1,06	No
ENSMUSG00000005481	-3,23E-02	6,181337752	2,51E-01	6,16E-01	8,55E-01	Ddx39	0,98	No
ENSMUSG00000005483	7,91E-02	5,689710419	1,37E+00	2,42E-01	5,50E-01	Dnajb1	1,06	No
ENSMUSG00000005493	3,45E-01	-2,548108026	1,21E-01	7,28E-01	9,19E-01	Msh4	1,27	No
ENSMUSG00000005501	-5,41E-02	4,260636939	4,26E-01	5,14E-01	7,96E-01	Usp40	0,96	No
ENSMUSG00000005505	-1,80E-02	4,189054276	4,48E-02	8,32E-01	9,78E-01	Kbtbd4	0,99	No

ENSMUSG00000005506	5,94E-02	6,977088976	9,61E-01	3,27E-01	6,28E-01	Celf1	1,04	No
ENSMUSG00000005510	-4,39E-02	3,529830106	2,00E-01	6,55E-01	8,79E-01	Ndufs3	0,97	No
ENSMUSG00000005514	1,48E-01	5,788327222	4,89E+00	2,70E-02	1,85E-01	Por	1,11	No
ENSMUSG00000005533	-1,45E-01	4,100385376	2,77E+00	9,61E-02	3,87E-01	Igf1r	0,90	No
ENSMUSG00000005534	7,56E-02	6,495929718	1,45E+00	2,29E-01	5,50E-01	Insr	1,05	No
ENSMUSG00000005540	-3,94E-01	-2,401342266	1,96E-01	6,58E-01	8,81E-01	Fcer2a	0,76	No
ENSMUSG00000005547	1,87E+00	-3,107639691	1,38E+00	2,40E-01	5,50E-01	Cyp2a5	3,67	No
ENSMUSG00000005553	-7,32E-02	-2,35544627	6,13E-03	9,38E-01	1,00E+00	Atp4a	0,95	No
ENSMUSG00000005566	2,22E-02	7,049933813	1,34E-01	7,14E-01	9,12E-01	Trim28	1,02	No
ENSMUSG00000005575	3,34E-02	6,148074622	2,68E-01	6,05E-01	8,49E-01	Ube2m	1,02	No
ENSMUSG00000005580	2,26E-01	3,81341291	5,90E+00	1,51E-02	1,27E-01	Adcy9	1,17	No
ENSMUSG00000005583	2,65E-01	3,689695973	7,63E+00	5,75E-03	6,20E-02	Mef2c	1,20	No
ENSMUSG00000005609	1,45E-01	4,776846583	3,64E+00	5,66E-02	2,92E-01	Ctr9	1,11	No
ENSMUSG00000005610	3,26E-02	9,679504508	3,34E-01	5,63E-01	8,26E-01	Eif4g2	1,02	No
ENSMUSG00000005611	2,98E-01	-0,366938118	7,97E-01	3,72E-01	6,73E-01	Mrvi1	1,23	No
ENSMUSG00000005615	-1,90E-01	7,516167266	1,03E+01	1,31E-03	2,01E-02	Pcyt1a	0,88	Yes
ENSMUSG00000005621	8,60E-02	6,161736082	1,78E+00	1,82E-01	5,43E-01	Zfp592	1,06	No
ENSMUSG00000005625	-1,08E-01	6,121869596	2,80E+00	9,40E-02	3,84E-01	Psmd4	0,93	No
ENSMUSG00000005628	4,69E-01	-1,0611013	1,07E+00	3,00E-01	5,96E-01	Tmod4	1,38	No
ENSMUSG00000005640	-2,66E+00	-2,770571569	2,82E+00	9,29E-02	3,83E-01	Insrr	0,16	No
ENSMUSG00000005656	-2,22E-02	5,95893589	1,14E-01	7,35E-01	9,24E-01	Snx6	0,98	No
ENSMUSG00000005667	-1,74E-01	6,08484121	7,21E+00	7,26E-03	7,38E-02	Mthfd2	0,89	No
ENSMUSG00000005672	1,87E-01	5,646066363	7,52E+00	6,10E-03	6,49E-02	Kit	1,14	No
ENSMUSG00000005674	1,06E-01	3,991722918	1,42E+00	2,34E-01	5,50E-01	Tomm40l	1,08	No
ENSMUSG00000005677	2,94E-02	3,341649126	8,03E-02	7,77E-01	9,46E-01	Nr1i3	1,02	No
ENSMUSG00000005681	-7,69E-01	-0,711716198	3,83E+00	5,04E-02	2,72E-01	Apoa2	0,59	No
ENSMUSG00000005682	-4,72E-02	4,646888909	3,66E-01	5,45E-01	8,13E-01	Pan2	0,97	No
ENSMUSG00000005683	3,57E-02	8,160050814	3,80E-01	5,38E-01	8,09E-01	Cs	1,03	No
ENSMUSG00000005686	2,16E-01	3,985015459	5,89E+00	1,52E-02	1,27E-01	Ampd3	1,16	No
ENSMUSG00000005687	3,77E-02	5,304193288	2,85E-01	5,93E-01	8,43E-01	Bcas2	1,03	No
ENSMUSG00000005698	7,58E-03	6,503600507	1,47E-02	9,04E-01	1,00E+00	Ctcf	1,01	No
ENSMUSG00000005699	3,00E-01	2,61858811	6,00E+00	1,43E-02	1,22E-01	Pard6a	1,23	No

ENSMUSG00000005705	-1,04E-01	-1,015963107	5,93E-02	8,08E-01	9,64E-01	Agrp	0,93	No
ENSMUSG00000005716	7,18E-01	-2,96326129	2,80E-01	5,97E-01	8,45E-01	Pvalb	1,65	No
ENSMUSG00000005718	4,58E-01	1,236543491	6,19E+00	1,28E-02	1,12E-01	Tfap4	1,37	No
ENSMUSG00000005732	1,18E-01	5,915067953	3,20E+00	7,36E-02	3,45E-01	Ranbp1	1,09	No
ENSMUSG00000005763	-2,37E-01	2,629682984	3,65E+00	5,60E-02	2,90E-01	Cd247	0,85	No
ENSMUSG00000005774	-1,42E-01	4,459534227	3,16E+00	7,53E-02	3,49E-01	Rfx5	0,91	No
ENSMUSG00000005779	5,29E-03	6,800739474	7,41E-03	9,31E-01	1,00E+00	Psmb4	1,00	No
ENSMUSG00000005800	-4,08E-01	8,781138863	5,03E+01	1,32E-12	1,60E-10	Mmp8	0,75	Yes
ENSMUSG00000005802	-4,38E-02	7,055019281	5,21E-01	4,70E-01	7,59E-01	Slc30a4	0,97	No
ENSMUSG00000005803	7,53E-02	5,504165065	1,18E+00	2,77E-01	5,74E-01	Sqor	1,05	No
ENSMUSG00000005804	-1,36E-01	5,96255335	4,30E+00	3,81E-02	2,32E-01	Bloc1s6	0,91	No
ENSMUSG00000005813	-3,71E-02	5,823330352	3,11E-01	5,77E-01	8,35E-01	Metap1	0,97	No
ENSMUSG00000005823	5,47E-02	5,747004122	6,64E-01	4,15E-01	7,10E-01	Gpr108	1,04	No
ENSMUSG00000005824	2,43E-01	2,562367671	3,55E+00	5,95E-02	3,02E-01	Tnfsf14	1,18	No
ENSMUSG00000005836	1,07E+00	-1,548234921	3,49E+00	6,19E-02	3,10E-01	Gata6	2,10	No
ENSMUSG00000005846	-2,71E-02	6,099245141	1,75E-01	6,76E-01	8,91E-01	Rsl1d1	0,98	No
ENSMUSG00000005871	1,79E-01	6,450309322	8,11E+00	4,39E-03	5,07E-02	Apc	1,13	No
ENSMUSG00000005873	6,27E-02	7,706135132	1,14E+00	2,86E-01	5,82E-01	Reep5	1,04	No
ENSMUSG00000005881	-3,94E-02	5,659361574	3,34E-01	5,63E-01	8,26E-01	Ergic3	0,97	No
ENSMUSG00000005882	-9,43E-03	4,099455576	1,17E-02	9,14E-01	1,00E+00	Uqcc1	0,99	No
ENSMUSG00000005883	-1,21E+00	0,062316792	1,80E+01	2,16E-05	6,66E-04	Spo11	0,43	Yes
ENSMUSG00000005886	-2,15E-01	6,744511189	1,21E+01	4,95E-04	9,10E-03	Ncoa2	0,86	Yes
ENSMUSG00000005893	-4,48E-02	5,721370133	4,48E-01	5,03E-01	7,88E-01	Nr2c2	0,97	No
ENSMUSG00000005897	4,62E-02	3,798129799	2,54E-01	6,14E-01	8,53E-01	Nr2c1	1,03	No
ENSMUSG00000005899	1,04E-01	5,330413348	2,19E+00	1,39E-01	4,71E-01	Smpd4	1,08	No
ENSMUSG00000005907	1,25E-01	4,156833316	2,07E+00	1,50E-01	4,91E-01	Pex1	1,09	No
ENSMUSG00000005917	2,68E-01	-1,378211334	3,01E-01	5,83E-01	8,38E-01	Otx1	1,20	No
ENSMUSG00000005936	1,01E-01	6,551078651	2,60E+00	1,07E-01	4,00E-01	Kctd20	1,07	No
ENSMUSG00000005947	3,67E-01	5,99612037	3,06E+01	3,14E-08	1,95E-06	Itgae	1,29	Yes
ENSMUSG00000005949	1,07E-01	5,539506362	2,44E+00	1,19E-01	4,28E-01	Ctns	1,08	No
ENSMUSG00000005950	-2,60E-01	6,272799359	1,67E+01	4,31E-05	1,20E-03	P2rx5	0,83	Yes
ENSMUSG00000005951	-2,00E-01	2,943483128	3,14E+00	7,64E-02	3,52E-01	Shpk	0,87	No

ENSMUSG00000005952	-3,57E+00	-2,831996158	5,74E+00	1,65E-02	1,34E-01	Trpv1	0,08	No
ENSMUSG00000005958	1,60E+00	-2,655183664	1,76E+00	1,84E-01	5,48E-01	Ephb3	3,04	No
ENSMUSG00000005968	2,43E-01	1,543487671	1,95E+00	1,62E-01	5,12E-01	Tuft1	1,18	No
ENSMUSG00000005973	6,64E-02	1,368377427	1,24E-01	7,25E-01	9,18E-01	Rcn1	1,05	No
ENSMUSG00000005980	5,21E-02	1,656735379	9,78E-02	7,54E-01	9,34E-01	Dnase1	1,04	No
ENSMUSG00000005981	-3,56E-02	5,992084763	2,96E-01	5,86E-01	8,39E-01	Trap1	0,98	No
ENSMUSG00000005982	8,96E-02	6,478016255	2,03E+00	1,54E-01	4,97E-01	Naa60	1,06	No
ENSMUSG00000005983	1,55E-01	3,970083096	3,02E+00	8,22E-02	3,68E-01	1700037C18Rik	1,11	No
ENSMUSG00000005986	-6,38E-02	2,460788491	2,38E-01	6,26E-01	8,60E-01	Ankrd13d	0,96	No
ENSMUSG00000006005	7,15E-02	7,960506646	1,51E+00	2,20E-01	5,50E-01	Tpr	1,05	No
ENSMUSG00000006007	-8,93E-02	-2,96327978	3,63E-03	9,52E-01	1,00E+00	Pdc	0,94	No
ENSMUSG00000006010	5,24E-02	5,081329889	5,11E-01	4,75E-01	7,63E-01	BC003331	1,04	No
ENSMUSG00000006014	-2,47E-01	1,074950322	1,60E+00	2,07E-01	5,50E-01	Prg4	0,84	No
ENSMUSG00000006019	1,85E-01	4,751481649	5,78E+00	1,62E-02	1,32E-01	Dhx34	1,14	No
ENSMUSG00000006021	-9,38E-02	2,432100598	5,01E-01	4,79E-01	7,66E-01	Kptn	0,94	No
ENSMUSG00000006024	-3,97E-02	6,893794638	4,23E-01	5,16E-01	7,97E-01	Napa	0,97	No
ENSMUSG00000006050	-1,07E-02	5,38423596	2,34E-02	8,78E-01	1,00E+00	Sra1	0,99	No
ENSMUSG00000006057	4,05E-02	4,554964959	2,60E-01	6,10E-01	8,51E-01	Atp5g1	1,03	No
ENSMUSG00000006058	-1,94E-01	4,837040731	6,57E+00	1,04E-02	9,58E-02	Snf8	0,87	No
ENSMUSG00000006095	-1,35E-01	5,552402212	3,84E+00	4,99E-02	2,70E-01	Tbcb	0,91	No
ENSMUSG00000006127	-4,21E-02	6,275650408	4,36E-01	5,09E-01	7,92E-01	Inpp5k	0,97	No
ENSMUSG00000006134	1,26E-01	5,671590613	3,52E+00	6,06E-02	3,05E-01	Crkl	1,09	No
ENSMUSG00000006143	-1,94E+00	-3,107619243	1,50E+00	2,21E-01	5,50E-01	Upk3bl	0,26	No
ENSMUSG00000006154	-1,44E-01	3,180804282	1,87E+00	1,72E-01	5,28E-01	Eps8l1	0,91	No
ENSMUSG00000006169	-9,59E-03	7,748085167	2,67E-02	8,70E-01	1,00E+00	Clint1	0,99	No
ENSMUSG00000006179	9,50E-02	1,474429589	2,87E-01	5,92E-01	8,42E-01	Prss16	1,07	No
ENSMUSG00000006191	-1,92E-01	3,490142329	3,78E+00	5,20E-02	2,78E-01	Cdkal1	0,88	No
ENSMUSG00000006205	-1,38E-01	-0,389859527	1,91E-01	6,62E-01	8,83E-01	Htra1	0,91	No
ENSMUSG00000006215	-1,79E-01	5,006696254	5,78E+00	1,62E-02	1,32E-01	Zbtb17	0,88	No
ENSMUSG00000006216	-8,86E-01	-2,289399721	1,05E+00	3,06E-01	6,04E-01	Clcnkb	0,54	No
ENSMUSG00000006219	-5,65E-02	5,856819954	7,21E-01	3,96E-01	6,96E-01	Fblim1	0,96	No
ENSMUSG00000006221	-1,08E+00	-1,185649449	5,67E+00	1,72E-02	1,37E-01	Hspb7	0,47	No

ENSMUSG00000006235	-1,20E+00	-2,070127633	1,88E+00	1,71E-01	5,26E-01	Epor	0,43	No
ENSMUSG00000006241	-3,26E-02	-0,216043561	1,09E-02	9,17E-01	1,00E+00	Ccdc159	0,98	No
ENSMUSG00000006262	6,02E-02	6,349428364	8,98E-01	3,43E-01	6,44E-01	Mob1b	1,04	No
ENSMUSG00000006269	-1,77E+00	-2,8319149	2,19E+00	1,39E-01	4,71E-01	Atp6v1b1	0,29	No
ENSMUSG00000006273	1,12E-02	9,122585289	3,92E-02	8,43E-01	9,85E-01	Atp6v1b2	1,01	No
ENSMUSG00000006276	-4,08E-02	6,289999856	4,10E-01	5,22E-01	8,00E-01	Eps15l1	0,97	No
ENSMUSG00000006281	1,73E-01	8,249236114	8,92E+00	2,82E-03	3,66E-02	Tep1	1,13	Yes
ENSMUSG00000006288	-1,27E-01	4,278325559	2,33E+00	1,27E-01	4,47E-01	Ttc5	0,92	No
ENSMUSG00000006289	1,40E-01	3,756646002	2,25E+00	1,33E-01	4,60E-01	Osgep	1,10	No
ENSMUSG00000006299	-8,36E-02	6,690798724	1,83E+00	1,76E-01	5,35E-01	Aamp	0,94	No
ENSMUSG00000006301	9,77E-02	6,588585634	2,47E+00	1,16E-01	4,21E-01	Tmbim1	1,07	No
ENSMUSG00000006304	1,58E-03	8,794177263	7,67E-04	9,78E-01	1,00E+00	Arpc2	1,00	No
ENSMUSG00000006307	-1,02E-01	6,655303821	2,73E+00	9,82E-02	3,91E-01	Kmt2b	0,93	No
ENSMUSG00000006310	7,84E-02	-0,755540487	4,22E-02	8,37E-01	9,81E-01	Zbtb32	1,06	No
ENSMUSG00000006311	1,84E+00	-3,033649193	1,33E+00	2,49E-01	5,50E-01	Etv2	3,58	No
ENSMUSG00000006313	-5,59E-01	-0,48501344	2,16E+00	1,42E-01	4,76E-01	Upk1a	0,68	No
ENSMUSG00000006315	1,18E-01	4,320176417	1,99E+00	1,58E-01	5,05E-01	Tmem147	1,09	No
ENSMUSG00000006333	-2,38E-01	8,346913097	1,71E+01	3,57E-05	1,02E-03	Rps9	0,85	Yes
ENSMUSG00000006335	-5,92E-03	3,918112579	4,25E-03	9,48E-01	1,00E+00	Tfpt	1,00	No
ENSMUSG00000006342	2,53E-01	-0,711738635	4,01E-01	5,27E-01	8,03E-01	Susd2	1,19	No
ENSMUSG00000006344	2,05E-01	1,889223942	1,61E+00	2,04E-01	5,50E-01	Ggt5	1,15	No
ENSMUSG00000006345	-7,22E-01	-2,226289424	8,87E-01	3,46E-01	6,47E-01	Ggt1	0,61	No
ENSMUSG00000006356	1,36E-02	1,222021706	5,13E-03	9,43E-01	1,00E+00	Crip2	1,01	No
ENSMUSG00000006360	-1,67E-01	6,758854512	7,35E+00	6,70E-03	6,96E-02	Crip1	0,89	No
ENSMUSG00000006362	6,59E-02	7,35387336	1,23E+00	2,67E-01	5,64E-01	Cbfa2t3	1,05	No
ENSMUSG00000006369	-3,23E+00	-2,832003132	4,38E+00	3,64E-02	2,27E-01	Fbln1	0,11	No
ENSMUSG00000006378	1,24E-01	3,598645153	1,61E+00	2,05E-01	5,50E-01	Gcat	1,09	No
ENSMUSG00000006386	7,04E-01	-2,401368935	7,19E-01	3,96E-01	6,97E-01	Tek	1,63	No
ENSMUSG00000006389	-4,13E-02	-3,033659495	7,08E-04	9,79E-01	1,00E+00	Mpl	0,97	No
ENSMUSG00000006390	1,21E-01	6,973511862	3,93E+00	4,74E-02	2,61E-01	Elovl1	1,09	No
ENSMUSG00000006392	-2,03E-02	5,326106688	8,32E-02	7,73E-01	9,44E-01	Med8	0,99	No
ENSMUSG00000006395	-1,72E-01	3,773673578	3,47E+00	6,24E-02	3,11E-01	Hyi	0,89	No

ENSMUSG00000006398	1,44E-01	4,610899953	3,30E+00	6,91E-02	3,31E-01	Cdc20	1,10	No
ENSMUSG00000006403	-9,73E-01	-1,289343699	3,76E+00	5,26E-02	2,79E-01	Adamts4	0,51	No
ENSMUSG00000006411	-1,68E-02	2,810460426	1,97E-02	8,88E-01	1,00E+00	Nectin4	0,99	No
ENSMUSG00000006412	4,16E-02	4,505809001	2,68E-01	6,05E-01	8,49E-01	Pfdn2	1,03	No
ENSMUSG00000006418	-2,17E-01	6,929130631	1,27E+01	3,60E-04	7,03E-03	Rnf114	0,86	Yes
ENSMUSG00000006435	-5,38E-01	2,784772841	2,14E+01	3,66E-06	1,39E-04	Neurl1a	0,69	Yes
ENSMUSG00000006442	2,09E-01	5,012395862	7,92E+00	4,89E-03	5,51E-02	Srm	1,16	No
ENSMUSG00000006445	1,87E-01	2,634459967	2,20E+00	1,38E-01	4,68E-01	Epha2	1,14	No
ENSMUSG00000006456	5,74E-02	5,539783439	7,00E-01	4,03E-01	7,01E-01	Rbm14	1,04	No
ENSMUSG00000006457	-5,74E-01	-2,711657388	2,58E-01	6,12E-01	8,52E-01	Actn3	0,67	No
ENSMUSG00000006462	-2,96E-01	-0,57416712	5,10E-01	4,75E-01	7,63E-01	A530013C23Rik	0,81	No
ENSMUSG00000006463	3,44E-03	3,222483242	1,07E-03	9,74E-01	1,00E+00	Zdhhc24	1,00	No
ENSMUSG00000006464	4,32E-02	0,053673818	2,10E-02	8,85E-01	1,00E+00	Bbs1	1,03	No
ENSMUSG00000006469	-2,63E-02	-3,033653157	2,33E-04	9,88E-01	1,00E+00	Slc34a3	0,98	No
ENSMUSG00000006471	-5,71E-02	5,528319851	6,91E-01	4,06E-01	7,02E-01	Ndor1	0,96	No
ENSMUSG00000006476	4,32E-02	5,132958032	3,54E-01	5,52E-01	8,18E-01	Nsmf	1,03	No
ENSMUSG00000006494	2,94E-01	5,682300391	1,87E+01	1,53E-05	4,88E-04	Pdk1	1,23	Yes
ENSMUSG00000006498	1,08E-01	7,576581678	3,36E+00	6,68E-02	3,25E-01	Ptbp1	1,08	No
ENSMUSG00000006517	2,32E-01	6,330841715	1,33E+01	2,71E-04	5,59E-03	Mvd	1,17	Yes
ENSMUSG00000006519	7,55E-02	8,143411283	1,69E+00	1,93E-01	5,50E-01	Cyba	1,05	No
ENSMUSG00000006526	-1,44E-01	4,13137515	2,82E+00	9,34E-02	3,83E-01	Tmem110	0,90	No
ENSMUSG00000006527	-1,17E-01	5,256246544	2,67E+00	1,02E-01	3,95E-01	Sfmbt1	0,92	No
ENSMUSG00000006529	7,67E-01	-2,963261668	3,24E-01	5,69E-01	8,30E-01	Itih1	1,70	No
ENSMUSG00000006538	-4,28E-01	-1,896054193	5,31E-01	4,66E-01	7,54E-01	Ihh	0,74	No
ENSMUSG00000006542	1,46E+00	-1,998016669	4,81E+00	2,83E-02	1,91E-01	Prkag3	2,75	No
ENSMUSG00000006567	8,34E-01	-1,548318033	2,17E+00	1,41E-01	4,75E-01	Atp7b	1,78	No
ENSMUSG00000006575	1,33E-01	1,119788473	4,23E-01	5,16E-01	7,97E-01	Rundc3a	1,10	No
ENSMUSG00000006576	-2,46E-01	2,905910018	4,86E+00	2,75E-02	1,87E-01	Slc4a3	0,84	No
ENSMUSG00000006585	3,48E-01	5,100482327	2,27E+01	1,91E-06	7,77E-05	Cdt1	1,27	Yes
ENSMUSG00000006586	-1,90E+00	-2,963209436	1,44E+00	2,31E-01	5,50E-01	Runx1t1	0,27	No
ENSMUSG00000006587	2,74E-01	0,477272039	1,21E+00	2,72E-01	5,68E-01	Snai3	1,21	No
ENSMUSG00000006589	8,60E-02	6,177978978	1,78E+00	1,82E-01	5,45E-01	Aprt	1,06	No

ENSMUSG00000006599	5,65E-02	6,191641446	7,71E-01	3,80E-01	6,81E-01	Gtf2h1	1,04	No
ENSMUSG00000006611	1,12E-01	4,813594889	2,14E+00	1,44E-01	4,79E-01	Hfe	1,08	No
ENSMUSG00000006638	-4,17E-03	3,057557279	1,41E-03	9,70E-01	1,00E+00	Abhd1	1,00	No
ENSMUSG00000006641	4,31E-01	3,137880436	1,56E+01	7,71E-05	1,95E-03	Slc5a6	1,35	Yes
ENSMUSG00000006642	1,87E+00	-3,107639691	1,38E+00	2,40E-01	5,50E-01	Tcf23	3,67	No
ENSMUSG00000006649	-1,48E-01	5,797016904	4,90E+00	2,69E-02	1,84E-01	Nphs1	0,90	No
ENSMUSG00000006651	3,21E-02	0,731840983	1,88E-02	8,91E-01	1,00E+00	Aplp1	1,02	No
ENSMUSG00000006673	5,07E-02	6,808927536	6,80E-01	4,10E-01	7,06E-01	Qrich1	1,04	No
ENSMUSG00000006675	-6,29E-01	0,136459207	4,74E+00	2,95E-02	1,96E-01	P4htm	0,65	No
ENSMUSG00000006676	5,28E-02	7,55683307	7,97E-01	3,72E-01	6,73E-01	Usp19	1,04	No
ENSMUSG00000006699	-2,74E-02	9,197680108	2,33E-01	6,29E-01	8,62E-01	Cdc42	0,98	No
ENSMUSG00000006705	7,51E-02	5,097038366	1,07E+00	3,01E-01	5,97E-01	Pknox1	1,05	No
ENSMUSG00000006711	-1,87E+00	-3,107637761	1,39E+00	2,38E-01	5,50E-01	D130043K22Rik	0,27	No
ENSMUSG00000006715	1,65E-01	4,606156149	4,40E+00	3,59E-02	2,25E-01	Gmnn	1,12	No
ENSMUSG00000006717	6,41E-02	4,248771991	5,72E-01	4,50E-01	7,40E-01	Acot13	1,05	No
ENSMUSG00000006720	-8,40E-02	-1,896143632	1,47E-02	9,03E-01	1,00E+00	Zfp184	0,94	No
ENSMUSG00000006724	7,02E-01	-1,683099665	1,33E+00	2,49E-01	5,50E-01	Cyp27b1	1,63	No
ENSMUSG00000006728	1,22E-01	7,031689065	4,05E+00	4,41E-02	2,50E-01	Cdk4	1,09	No
ENSMUSG00000006731	-1,62E-01	7,361437161	7,40E+00	6,53E-03	6,82E-02	B4galnt1	0,89	No
ENSMUSG00000006732	3,12E-01	3,165377409	8,24E+00	4,09E-03	4,81E-02	Mettl1	1,24	Yes
ENSMUSG00000006736	-4,45E-02	5,969239691	4,60E-01	4,98E-01	7,83E-01	Tspan31	0,97	No
ENSMUSG00000006740	4,00E-02	7,378575087	4,50E-01	5,02E-01	7,87E-01	Kif5b	1,03	No
ENSMUSG00000006763	3,84E-03	3,247085935	1,30E-03	9,71E-01	1,00E+00	Saal1	1,00	No
ENSMUSG00000006782	9,30E-03	5,667323459	1,88E-02	8,91E-01	1,00E+00	Cnp	1,01	No
ENSMUSG00000006784	-5,96E-01	0,934816116	7,97E+00	4,76E-03	5,39E-02	Ttc25	0,66	No
ENSMUSG00000006800	2,95E-01	7,860620756	2,54E+01	4,75E-07	2,27E-05	Sulf2	1,23	Yes
ENSMUSG00000006818	-8,54E-02	6,733944438	1,91E+00	1,67E-01	5,20E-01	Sod2	0,94	No
ENSMUSG00000006850	-1,74E-02	3,049765649	2,50E-02	8,74E-01	1,00E+00	Tmco6	0,99	No
ENSMUSG00000006906	8,42E-02	4,468885365	1,07E+00	3,00E-01	5,97E-01	Stambp	1,06	No
ENSMUSG00000006920	-5,70E-02	5,95151037	7,60E-01	3,83E-01	6,85E-01	Ezh1	0,96	No
ENSMUSG00000006930	-5,01E-01	0,858705135	5,35E+00	2,07E-02	1,55E-01	Hap1	0,71	No
ENSMUSG00000006931	1,47E-01	-1,683182228	7,02E-02	7,91E-01	9,55E-01	P3h4	1,11	No

ENSMUSG00000006932	1,09E-01	8,291952975	3,55E+00	5,96E-02	3,02E-01	Ctnnb1	1,08	No
ENSMUSG00000006941	7,43E-02	4,932964526	9,82E-01	3,22E-01	6,22E-01	Eif1b	1,05	No
ENSMUSG00000006948	4,83E-01	-2,070069903	3,16E-01	5,74E-01	8,33E-01	Klk4	1,40	No
ENSMUSG00000006998	1,85E-02	6,965331708	9,27E-02	7,61E-01	9,37E-01	Psmd2	1,01	No
ENSMUSG00000007021	-9,31E-01	-1,051641759	4,87E+00	2,73E-02	1,86E-01	Syngr3	0,52	No
ENSMUSG00000007029	-3,70E-02	6,260425194	3,35E-01	5,63E-01	8,26E-01	Vars	0,97	No
ENSMUSG00000007030	1,32E+00	-2,088800304	2,43E+00	1,19E-01	4,29E-01	Vwa7	2,50	No
ENSMUSG00000007033	6,86E-02	-0,215920699	4,41E-02	8,34E-01	9,79E-01	Hspa1l	1,05	No
ENSMUSG00000007034	-3,65E-01	-2,49761737	1,68E-01	6,82E-01	8,94E-01	Slc44a4	0,78	No
ENSMUSG00000007035	4,35E-01	0,2214859	2,68E+00	1,02E-01	3,94E-01	Msh5	1,35	No
ENSMUSG00000007036	2,62E-02	4,736648566	1,15E-01	7,35E-01	9,24E-01	Abhd16a	1,02	No
ENSMUSG00000007038	1,19E-02	6,907422704	3,81E-02	8,45E-01	9,86E-01	Neu1	1,01	No
ENSMUSG00000007039	-2,69E-02	0,750600547	1,46E-02	9,04E-01	1,00E+00	Ddah2	0,98	No
ENSMUSG00000007041	2,58E-02	8,374904609	2,00E-01	6,54E-01	8,79E-01	Clic1	1,02	No
ENSMUSG00000007050	6,26E-02	4,165567844	5,27E-01	4,68E-01	7,56E-01	Lsm2	1,04	No
ENSMUSG00000007080	1,89E-01	5,189663284	6,88E+00	8,74E-03	8,46E-02	Pole	1,14	No
ENSMUSG00000007097	1,05E-02	-3,070147673	-1,08E-11	1,00E+00	1,00E+00	Atp1a2	1,01	No
ENSMUSG00000007122	-2,44E-02	-1,146088775	2,83E-03	9,58E-01	1,00E+00	Casq1	0,98	No
ENSMUSG00000007207	-4,62E-01	0,095846954	2,59E+00	1,08E-01	4,02E-01	Stx1a	0,73	No
ENSMUSG00000007209	1,81E+00	-3,10765737	1,28E+00	2,58E-01	5,50E-01	Ceacam9	3,49	No
ENSMUSG00000007216	-2,64E-03	2,274106866	3,82E-04	9,84E-01	1,00E+00	Zfp775	1,00	No
ENSMUSG00000007279	-1,36E+00	-2,896122487	1,16E+00	2,81E-01	5,78E-01	Scube2	0,39	No
ENSMUSG00000007338	2,34E-01	4,491331165	8,52E+00	3,52E-03	4,31E-02	Mrpl49	1,18	Yes
ENSMUSG00000007379	5,71E-01	2,723965254	2,18E+01	3,06E-06	1,18E-04	Dennd2c	1,49	Yes
ENSMUSG00000007411	8,83E-02	5,801907769	1,74E+00	1,87E-01	5,50E-01	Mark3	1,06	No
ENSMUSG00000007415	4,29E-02	5,894972805	4,21E-01	5,16E-01	7,97E-01	Gatad1	1,03	No
ENSMUSG00000007440	4,82E-03	-3,17446542	1,60E-11	1,00E+00	1,00E+00	Pcdha11	1,00	No
ENSMUSG00000007458	-7,68E-03	8,453351771	1,78E-02	8,94E-01	1,00E+00	M6pr	0,99	No
ENSMUSG00000007476	1,60E-01	3,456288875	2,57E+00	1,09E-01	4,05E-01	Lrrc8a	1,12	No
ENSMUSG00000007480	-3,83E-02	-3,107651936	-7,91E-11	1,00E+00	1,00E+00	Mc5r	0,97	No
ENSMUSG00000007564	-2,34E-03	7,425999334	1,55E-03	9,69E-01	1,00E+00	Ppp2r1a	1,00	No
ENSMUSG00000007570	5,11E-02	4,021660495	3,31E-01	5,65E-01	8,28E-01	Fance	1,04	No

ENSMUSG00000007589	3,76E-02	4,371082953	2,06E-01	6,50E-01	8,76E-01	Tinf2		1,03	No
ENSMUSG00000007591	7,17E-02	-0,424907965	4,75E-02	8,27E-01	9,76E-01	Tssk4		1,05	No
ENSMUSG00000007594	3,22E-01	-2,033535928	1,91E-01	6,62E-01	8,83E-01	Hapln4		1,25	No
ENSMUSG00000007603	5,73E-02	5,009654264	5,94E-01	4,41E-01	7,33E-01	Dus3l		1,04	No
ENSMUSG00000007610	7,93E-02	4,203372839	8,81E-01	3,48E-01	6,49E-01	Gtpbp3		1,06	No
ENSMUSG00000007613	3,14E-01	8,246595806	2,94E+01	5,80E-08	3,40E-06	Tgfbr1		1,24	Yes
ENSMUSG00000007617	1,72E-01	6,33009917	7,28E+00	6,97E-03	7,16E-02	Homer1		1,13	No
ENSMUSG00000007646	-3,90E-02	1,136106767	3,78E-02	8,46E-01	9,87E-01	Rad51c		0,97	No
ENSMUSG00000007655	9,18E-01	6,741351356	2,14E+02	1,96E-48	3,91E-45	Cav1		1,89	Yes
ENSMUSG00000007656	-1,40E-01	5,981973514	4,55E+00	3,29E-02	2,12E-01	Arpp19		0,91	No
ENSMUSG00000007659	1,76E-01	6,819385955	8,18E+00	4,24E-03	4,94E-02	Bcl2l1		1,13	Yes
ENSMUSG00000007670	8,15E-02	6,814759377	1,77E+00	1,84E-01	5,47E-01	Khsrp		1,06	No
ENSMUSG00000007682	-5,77E-01	2,008840449	1,50E+01	1,05E-04	2,53E-03	Dio2		0,67	Yes
ENSMUSG00000007721	-4,44E-02	5,457897251	4,06E-01	5,24E-01	8,01E-01	Ccdc124		0,97	No
ENSMUSG00000007739	-1,01E-01	7,290226181	2,88E+00	8,97E-02	3,80E-01	Cct4		0,93	No
ENSMUSG00000007777	5,91E-02	3,498644877	3,58E-01	5,50E-01	8,17E-01	0610009B22Rik		1,04	No
ENSMUSG00000007783	-3,55E-01	2,213761563	6,57E+00	1,04E-02	9,58E-02	Cpt1c		0,78	No
ENSMUSG00000007812	1,07E-01	5,214227826	2,26E+00	1,33E-01	4,58E-01	Zfp655		1,08	No
ENSMUSG00000007815	-4,32E-03	8,099842168	5,54E-03	9,41E-01	1,00E+00	Rhoa		1,00	No
ENSMUSG00000007817	1,32E-01	8,553634179	5,28E+00	2,16E-02	1,58E-01	Zmiz1		1,10	No
ENSMUSG00000007827	1,81E-01	3,003920984	2,57E+00	1,09E-01	4,04E-01	Ankrd26		1,13	No
ENSMUSG00000007833	8,62E-02	5,683824293	1,63E+00	2,02E-01	5,50E-01	Aldh16a1		1,06	No
ENSMUSG00000007836	8,67E-02	7,651076046	2,16E+00	1,41E-01	4,75E-01	Hnrnpa0		1,06	No
ENSMUSG00000007837	-9,75E-02	4,317691977	1,37E+00	2,41E-01	5,50E-01	Prrg2		0,93	No
ENSMUSG00000007850	-1,49E-02	8,133023254	6,58E-02	7,98E-01	9,58E-01	Hnrnph1		0,99	No
ENSMUSG00000007867	-2,56E-01	1,66453294	2,29E+00	1,30E-01	4,52E-01	Ift43		0,84	No
ENSMUSG00000007872	-9,41E-01	1,576112094	2,63E+01	2,91E-07	1,46E-05	Id3		0,52	Yes
ENSMUSG00000007877	-8,78E-02	-2,963255397	3,51E-03	9,53E-01	1,00E+00	Tcap		0,94	No
ENSMUSG00000007880	9,07E-02	7,54787532	2,36E+00	1,25E-01	4,42E-01	Arid1a		1,06	No
ENSMUSG00000007888	-5,84E-01	-2,770605859	2,68E-01	6,05E-01	8,49E-01	Crlf1		0,67	No
ENSMUSG00000007891	3,54E-02	11,7463612	4,02E-01	5,26E-01	8,03E-01	Ctsd		1,02	No
ENSMUSG00000007892	-1,05E-01	8,558232219	3,34E+00	6,75E-02	3,27E-01	Rplp1		0,93	No

ENSMUSG00000007944	-8,96E-02	-0,937704512	4,92E-02	8,24E-01	9,74E-01	Ttc9b	0,94	No
ENSMUSG00000007946	-3,45E-01	-1,333133814	4,57E-01	4,99E-01	7,84E-01	Phox2a	0,79	No
ENSMUSG00000007950	2,98E-03	3,335784337	8,11E-04	9,77E-01	1,00E+00	Abhd8	1,00	No
ENSMUSG00000007987	1,16E-01	4,097725208	1,78E+00	1,82E-01	5,44E-01	Ift22	1,08	No
ENSMUSG00000007989	-4,52E+00	-2,600647612	1,15E+01	6,95E-04	1,20E-02	Fzd3	0,04	Yes
ENSMUSG00000008028	1,34E+00	-2,165863166	2,86E+00	9,09E-02	3,82E-01	1700008O03Rik	2,53	No
ENSMUSG00000008036	7,93E-02	6,950171655	1,69E+00	1,93E-01	5,50E-01	Ap2s1	1,06	No
ENSMUSG00000008090	-2,09E-02	0,358705675	6,22E-03	9,37E-01	1,00E+00	Fgfrl1	0,99	No
ENSMUSG00000008129	2,19E-02	-0,816520394	2,92E-03	9,57E-01	1,00E+00	4930432K21Rik	1,02	No
ENSMUSG00000008136	6,30E-01	-1,896134119	8,07E-01	3,69E-01	6,70E-01	Fhl2	1,55	No
ENSMUSG00000008140	9,59E-02	6,16108731	2,19E+00	1,39E-01	4,70E-01	Emc10	1,07	No
ENSMUSG00000008153	-2,72E-01	-0,509863221	6,45E-01	4,22E-01	7,17E-01	Clstn3	0,83	No
ENSMUSG00000008167	-2,66E-02	3,439582821	7,03E-02	7,91E-01	9,55E-01	Fbxw9	0,98	No
ENSMUSG00000008193	-8,35E-01	4,574631227	1,11E+02	5,05E-26	1,89E-23	Spib	0,56	Yes
ENSMUSG00000008200	2,10E-02	6,597684867	1,14E-01	7,36E-01	9,24E-01	Fnbp4	1,01	No
ENSMUSG00000008206	-2,74E-02	-0,600587719	5,50E-03	9,41E-01	1,00E+00	Cers4	0,98	No
ENSMUSG00000008226	3,55E-01	3,684998472	1,43E+01	1,55E-04	3,50E-03	Scrn3	1,28	Yes
ENSMUSG00000008301	3,35E-02	4,778822497	1,91E-01	6,62E-01	8,83E-01	Phax	1,02	No
ENSMUSG00000008305	1,57E-01	3,684228259	2,68E+00	1,01E-01	3,94E-01	Tle1	1,12	No
ENSMUSG00000008307	-4,19E-01	3,086812546	1,39E+01	1,96E-04	4,27E-03	1700109H08Rik	0,75	Yes
ENSMUSG00000008318	-1,50E-01	6,308551534	5,59E+00	1,81E-02	1,41E-01	Relt	0,90	No
ENSMUSG00000008333	-9,36E-02	4,804121204	1,50E+00	2,21E-01	5,50E-01	Snrpb2	0,94	No
ENSMUSG00000008348	-1,45E-01	11,13958142	6,73E+00	9,49E-03	8,98E-02	Ubc	0,90	No
ENSMUSG00000008373	4,31E-02	6,146488734	4,46E-01	5,04E-01	7,89E-01	Prpf31	1,03	No
ENSMUSG00000008384	-1,63E-02	4,52070506	4,17E-02	8,38E-01	9,82E-01	Sertad1	0,99	No
ENSMUSG00000008393	1,32E-01	6,309543299	4,25E+00	3,92E-02	2,35E-01	Carhsp1	1,10	No
ENSMUSG00000008398	-3,17E-02	5,793302226	2,26E-01	6,34E-01	8,66E-01	Elk3	0,98	No
ENSMUSG00000008429	-1,92E-02	6,03228496	8,62E-02	7,69E-01	9,42E-01	Herpud2	0,99	No
ENSMUSG00000008435	-1,17E-02	4,337233866	2,03E-02	8,87E-01	1,00E+00	Rdh13	0,99	No
ENSMUSG00000008438	1,60E-01	-2,107648518	3,84E-02	8,45E-01	9,86E-01	Adam21	1,12	No
ENSMUSG00000008450	-4,06E-02	3,487055109	1,68E-01	6,82E-01	8,94E-01	Nutf2	0,97	No
ENSMUSG00000008475	-3,70E-02	9,216324331	4,25E-01	5,14E-01	7,96E-01	Arpc5	0,97	No

ENSMUSG00000008482	-2,88E-02	-2,896100165	4,24E-04	9,84E-01	1,00E+00	Rnf151	0,98	No
ENSMUSG00000008496	-6,85E-02	0,873195461	9,95E-02	7,52E-01	9,32E-01	Pou2f2	0,95	No
ENSMUSG00000008540	2,03E-01	5,744281442	9,03E+00	2,66E-03	3,49E-02	Mgst1	1,15	Yes
ENSMUSG00000008575	1,12E+00	-2,497601997	1,20E+00	2,74E-01	5,71E-01	Nfib	2,18	No
ENSMUSG00000008590	2,36E+00	-2,600762357	4,75E+00	2,92E-02	1,95E-01	Htr3b	5,14	No
ENSMUSG00000008601	-1,29E-01	-0,963181358	9,55E-02	7,57E-01	9,35E-01	Rab25	0,91	No
ENSMUSG00000008604	7,73E-02	5,740449627	1,32E+00	2,50E-01	5,50E-01	Ubqln4	1,06	No
ENSMUSG00000008668	-2,28E-01	6,639108003	1,36E+01	2,28E-04	4,85E-03	Rps18	0,85	Yes
ENSMUSG00000008683	-2,59E-01	5,342025189	1,35E+01	2,34E-04	4,96E-03	Rps15a	0,84	Yes
ENSMUSG00000008690	5,26E-02	5,624658517	5,96E-01	4,40E-01	7,32E-01	Ncaph2	1,04	No
ENSMUSG00000008730	1,03E-01	8,290836041	3,19E+00	7,42E-02	3,46E-01	Hipk1	1,07	No
ENSMUSG00000008734	-1,21E-01	0,906332072	3,13E-01	5,76E-01	8,34E-01	Gprc5b	0,92	No
ENSMUSG00000008763	1,64E-01	6,270320548	6,54E+00	1,06E-02	9,72E-02	Man1a2	1,12	No
ENSMUSG00000008822	3,14E-01	1,85390692	3,94E+00	4,71E-02	2,60E-01	Acyp1	1,24	No
ENSMUSG00000008843	-8,98E-01	-2,770592541	7,00E-01	4,03E-01	7,01E-01	Cldn13	0,54	No
ENSMUSG00000008845	5,08E-01	2,781591705	1,70E+01	3,69E-05	1,05E-03	Cd163	1,42	Yes
ENSMUSG00000008855	1,80E-01	6,367922678	8,08E+00	4,49E-03	5,14E-02	Hdac5	1,13	No
ENSMUSG00000008859	-1,50E-01	5,570246609	4,83E+00	2,80E-02	1,89E-01	Rala	0,90	No
ENSMUSG00000008892	-1,20E-02	4,983654842	2,63E-02	8,71E-01	1,00E+00	Vdac3	0,99	No
ENSMUSG00000008958	-1,18E-01	4,622643606	2,25E+00	1,34E-01	4,60E-01	Vps72	0,92	No
ENSMUSG00000008976	4,16E-02	6,116988129	4,14E-01	5,20E-01	7,99E-01	Gabpa	1,03	No
ENSMUSG00000009013	1,01E-01	6,097325337	2,44E+00	1,18E-01	4,27E-01	Dynll1	1,07	No
ENSMUSG00000009030	-8,04E-03	5,518185823	1,35E-02	9,07E-01	1,00E+00	Pdcl	0,99	No
ENSMUSG00000009035	-1,99E-02	6,663406446	1,03E-01	7,49E-01	9,30E-01	Tmem184b	0,99	No
ENSMUSG00000009073	2,01E-02	6,444917328	1,01E-01	7,50E-01	9,31E-01	Nf2	1,01	No
ENSMUSG00000009075	3,16E-02	1,130057507	2,54E-02	8,73E-01	1,00E+00	Cabp7	1,02	No
ENSMUSG00000009076	-1,72E-01	2,903703246	2,27E+00	1,32E-01	4,57E-01	Zmat5	0,89	No
ENSMUSG00000009079	-2,20E-02	7,006788398	1,31E-01	7,17E-01	9,13E-01	Ewsr1	0,98	No
ENSMUSG00000009090	1,54E-01	7,603464273	6,77E+00	9,28E-03	8,83E-02	Ap1b1	1,11	No
ENSMUSG00000009092	4,53E-01	-0,257504437	1,54E+00	2,14E-01	5,50E-01	Derl3	1,37	No
ENSMUSG00000009093	4,34E-01	-2,711732088	1,47E-01	7,02E-01	9,05E-01	Gstt4	1,35	No
ENSMUSG00000009097	9,01E-03	-3,107646452	-5,16E-10	1,00E+00	1,00E+00	Tbx1	1,01	No

ENSMUSG00000009108	-7,40E-01	-1,079401812	2,97E+00	8,50E-02	3,74E-01	Gnat2	0,60	No
ENSMUSG00000009112	-6,32E-02	5,717870132	8,82E-01	3,48E-01	6,49E-01	Bcl2l13	0,96	No
ENSMUSG00000009114	-5,29E-01	-1,755629771	8,38E-01	3,60E-01	6,61E-01	2610028H24Rik	0,69	No
ENSMUSG00000009115	4,09E-01	-2,963255991	8,45E-02	7,71E-01	9,43E-01	Spatc1l	1,33	No
ENSMUSG00000009145	-2,98E-02	2,214843649	4,67E-02	8,29E-01	9,76E-01	Dqx1	0,98	No
ENSMUSG00000009185	-8,14E-02	-2,832040318	4,55E-03	9,46E-01	1,00E+00	Ccl8	0,95	No
ENSMUSG00000009207	2,42E-01	4,614695747	9,36E+00	2,21E-03	3,03E-02	Lnpk	1,18	Yes
ENSMUSG00000009210	1,87E+00	-3,107639691	1,38E+00	2,40E-01	5,50E-01	Prr29	3,67	No
ENSMUSG00000009214	2,63E+00	-2,963269893	2,67E+00	1,02E-01	3,94E-01	Mymk	6,19	No
ENSMUSG00000009246	-1,13E-01	0,372486477	1,70E-01	6,80E-01	8,93E-01	Trpm5	0,92	No
ENSMUSG00000009248	7,13E-01	-2,655138649	2,78E-01	5,98E-01	8,45E-01	Ascl2	1,64	No
ENSMUSG00000009281	-1,71E-01	1,986689669	1,35E+00	2,45E-01	5,50E-01	Rarres2	0,89	No
ENSMUSG00000009291	1,25E-01	8,141728465	4,66E+00	3,08E-02	2,02E-01	Pttg1ip	1,09	No
ENSMUSG00000009292	-3,94E-01	2,283530334	8,16E+00	4,29E-03	4,99E-02	Trpm2	0,76	Yes
ENSMUSG00000009293	-8,00E-02	6,181177201	1,56E+00	2,12E-01	5,50E-01	Ube2g2	0,95	No
ENSMUSG00000009350	1,56E-01	-1,785711804	5,82E-02	8,09E-01	9,65E-01	Mpo	1,11	No
ENSMUSG00000009356	6,94E-01	-2,963285211	2,59E-01	6,11E-01	8,52E-01	Lpo	1,62	No
ENSMUSG00000009376	-1,42E-01	6,176224033	4,89E+00	2,70E-02	1,84E-01	Met	0,91	No
ENSMUSG00000009378	1,85E+00	-3,107657832	1,35E+00	2,46E-01	5,50E-01	Slc16a12	3,61	No
ENSMUSG00000009394	-2,93E-01	-1,548321858	3,01E-01	5,83E-01	8,38E-01	Syn2	0,82	No
ENSMUSG00000009418	1,35E-01	6,92414466	4,92E+00	2,66E-02	1,83E-01	Nav1	1,10	No
ENSMUSG00000009470	8,49E-02	7,45507036	2,05E+00	1,52E-01	4,94E-01	Tnpo1	1,06	No
ENSMUSG00000009487	5,91E-01	-2,448685441	3,90E-01	5,32E-01	8,06E-01	Otog	1,51	No
ENSMUSG00000009535	2,46E-02	5,257309726	1,18E-01	7,31E-01	9,22E-01	Rnmt	1,02	No
ENSMUSG00000009545	1,51E-01	0,1245466	2,79E-01	5,98E-01	8,45E-01	Kcnq1	1,11	No
ENSMUSG00000009549	1,53E-02	6,043776687	5,54E-02	8,14E-01	9,68E-01	Srp14	1,01	No
ENSMUSG00000009551	1,55E-01	-1,535410776	9,41E-02	7,59E-01	9,36E-01	6330409D20Rik	1,11	No
ENSMUSG00000009555	6,65E-02	6,718325648	1,15E+00	2,83E-01	5,79E-01	Cdk9	1,05	No
ENSMUSG00000009563	1,19E-01	5,520641533	2,96E+00	8,52E-02	3,75E-01	Tor2a	1,09	No
ENSMUSG00000009566	1,10E-01	3,006519692	9,48E-01	3,30E-01	6,31E-01	Fpgs	1,08	No
ENSMUSG00000009569	1,57E-01	6,436211717	6,22E+00	1,26E-02	1,11E-01	Mkl2	1,12	No
ENSMUSG00000009575	2,56E-01	7,049568732	1,79E+01	2,28E-05	6,96E-04	Cbx5	1,19	Yes

ENSMUSG00000009585	-3,32E-01	7,118269535	3,06E+01	3,24E-08	2,00E-06	Apobec3	0,79	Yes
ENSMUSG00000009588	-1,87E+00	-3,107637761	1,39E+00	2,38E-01	5,50E-01	St6galnac1	0,27	No
ENSMUSG00000009614	6,70E-01	-0,295020578	3,68E+00	5,52E-02	2,88E-01	Sardh	1,59	No
ENSMUSG00000009621	3,08E-02	6,334884827	2,35E-01	6,28E-01	8,61E-01	Vav2	1,02	No
ENSMUSG00000009628	-7,33E-02	1,146669906	1,50E-01	6,99E-01	9,04E-01	Tex15	0,95	No
ENSMUSG00000009630	-1,86E-01	5,838538457	7,76E+00	5,33E-03	5,86E-02	Ppp2cb	0,88	No
ENSMUSG00000009633	3,48E-01	-1,683246991	4,21E-01	5,16E-01	7,97E-01	G0s2	1,27	No
ENSMUSG00000009640	2,23E-02	3,980962421	6,23E-02	8,03E-01	9,62E-01	Dmap1	1,02	No
ENSMUSG00000009646	-1,87E+00	-3,107637761	1,39E+00	2,38E-01	5,50E-01	Pla2g12b	0,27	No
ENSMUSG00000009647	1,61E-01	3,290035396	2,36E+00	1,25E-01	4,42E-01	Mcu	1,12	No
ENSMUSG00000009654	-1,47E-01	1,829107142	9,02E-01	3,42E-01	6,42E-01	Oit3	0,90	No
ENSMUSG00000009681	-1,19E-01	2,087246101	6,77E-01	4,10E-01	7,07E-01	Bcr	0,92	No
ENSMUSG00000009687	1,79E-01	7,637242189	9,21E+00	2,40E-03	3,24E-02	Fxyd5	1,13	Yes
ENSMUSG00000009733	-1,32E-02	2,538440371	1,07E-02	9,18E-01	1,00E+00	Tfcp2	0,99	No
ENSMUSG00000009739	2,51E-01	1,927194332	2,59E+00	1,08E-01	4,02E-01	Pou6f1	1,19	No
ENSMUSG00000009741	-9,96E-03	6,800243027	2,63E-02	8,71E-01	1,00E+00	Ubp1	0,99	No
ENSMUSG00000009772	-4,23E-01	5,708128128	4,00E+01	2,50E-10	2,22E-08	Nuak2	0,75	Yes
ENSMUSG00000009828	-2,15E-02	3,681846012	5,07E-02	8,22E-01	9,72E-01	Ick	0,99	No
ENSMUSG00000009863	3,88E-02	6,092774399	3,57E-01	5,50E-01	8,17E-01	Sdhb	1,03	No
ENSMUSG00000009876	8,02E-01	-1,627619382	1,79E+00	1,81E-01	5,42E-01	Cox4i2	1,74	No
ENSMUSG00000009894	-2,36E-01	4,14884855	7,54E+00	6,02E-03	6,43E-02	Snap47	0,85	No
ENSMUSG00000009900	3,14E-02	-3,107617333	-7,69E-10	1,00E+00	1,00E+00	Wnt3a	1,02	No
ENSMUSG00000009905	2,09E-01	4,170184299	5,93E+00	1,49E-02	1,25E-01	Kdsr	1,16	No
ENSMUSG00000009907	-4,44E-02	6,912821258	5,31E-01	4,66E-01	7,55E-01	Vps4b	0,97	No
ENSMUSG00000009927	-2,21E-02	6,056515152	1,15E-01	7,35E-01	9,24E-01	Rps25	0,98	No
ENSMUSG00000010021	2,08E-01	0,358384145	6,22E-01	4,30E-01	7,24E-01	Kif19a	1,16	No
ENSMUSG00000010025	2,06E-02	6,519527407	1,09E-01	7,42E-01	9,27E-01	Aldh3a2	1,01	No
ENSMUSG00000010044	1,12E+00	-1,863707699	1,89E+00	1,70E-01	5,25E-01	Zmynd10	2,18	No
ENSMUSG00000010045	2,10E-01	5,258643881	8,64E+00	3,29E-03	4,11E-02	Tmem115	1,16	Yes
ENSMUSG00000010047	8,79E-02	3,140039239	6,74E-01	4,12E-01	7,07E-01	Hyal2	1,06	No
ENSMUSG00000010048	4,35E-02	3,787249948	2,22E-01	6,37E-01	8,68E-01	Ifrd2	1,03	No
ENSMUSG00000010051	7,17E-03	1,037476388	1,25E-03	9,72E-01	1,00E+00	Hyal1	1,00	No

ENSMUSG00000010054	6,46E-03	4,188936143	5,72E-03	9,40E-01	1,00E+00	Tusc2	1,00	No
ENSMUSG00000010057	-1,33E-01	3,142268293	1,49E+00	2,22E-01	5,50E-01	Nprl2	0,91	No
ENSMUSG00000010066	-8,17E-01	2,302357246	3,70E+01	1,21E-09	9,74E-08	Cacna2d2	0,57	Yes
ENSMUSG00000010067	8,65E-02	5,547548491	1,58E+00	2,09E-01	5,50E-01	Rassf1	1,06	No
ENSMUSG00000010080	3,14E-02	-3,107617333	-7,69E-10	1,00E+00	1,00E+00	Epn3	1,02	No
ENSMUSG00000010095	-3,61E-02	8,101563965	3,87E-01	5,34E-01	8,07E-01	Slc3a2	0,98	No
ENSMUSG00000010097	-1,47E-01	7,152820542	6,00E+00	1,43E-02	1,22E-01	Nxf1	0,90	No
ENSMUSG00000010110	-1,58E-01	5,785908303	5,60E+00	1,79E-02	1,40E-01	Stx5a	0,90	No
ENSMUSG00000010122	-1,46E+00	-2,600629085	2,20E+00	1,38E-01	4,68E-01	Slc47a1	0,36	No
ENSMUSG00000010136	1,85E+00	-3,107657832	1,35E+00	2,46E-01	5,50E-01	Pifo	3,61	No
ENSMUSG00000010142	2,52E-01	6,07798455	1,50E+01	1,07E-04	2,58E-03	Tnfrsf13b	1,19	Yes
ENSMUSG00000010154	1,22E+00	-2,711680537	9,22E-01	3,37E-01	6,37E-01	Spire2	2,33	No
ENSMUSG00000010175	3,50E-01	-0,770638961	7,90E-01	3,74E-01	6,75E-01	Prox1	1,27	No
ENSMUSG00000010205	9,29E-02	5,702471779	1,90E+00	1,68E-01	5,23E-01	Raver1	1,07	No
ENSMUSG00000010277	4,67E-02	8,577717955	6,63E-01	4,16E-01	7,11E-01	2610507B11Rik	1,03	No
ENSMUSG00000010290	9,59E-02	4,084087184	1,21E+00	2,72E-01	5,68E-01	AI597479	1,07	No
ENSMUSG00000010307	1,17E-01	5,491865795	2,84E+00	9,21E-02	3,82E-01	Tmem86a	1,08	No
ENSMUSG00000010342	-1,11E+00	1,15889281	3,39E+01	5,72E-09	4,16E-07	Tex14	0,46	Yes
ENSMUSG00000010358	-2,53E-01	5,292618316	1,29E+01	3,21E-04	6,40E-03	Ifi35	0,84	Yes
ENSMUSG00000010362	-3,15E-01	1,856749849	4,13E+00	4,20E-02	2,44E-01	Rdm1	0,80	No
ENSMUSG00000010376	-5,26E-02	6,162072383	6,67E-01	4,14E-01	7,09E-01	Nedd8	0,96	No
ENSMUSG00000010392	8,26E-02	5,751338289	1,52E+00	2,18E-01	5,50E-01	Gosr1	1,06	No
ENSMUSG00000010406	-1,67E-02	4,016904278	3,54E-02	8,51E-01	9,90E-01	Mrpl52	0,99	No
ENSMUSG00000010453	-3,13E-01	6,932873269	2,65E+01	2,63E-07	1,33E-05	Kansl3	0,80	Yes
ENSMUSG00000010461	4,14E-02	0,496136229	3,39E-02	8,54E-01	9,92E-01	Eya4	1,03	No
ENSMUSG00000010476	-1,33E-02	-1,522626588	6,63E-04	9,79E-01	1,00E+00	Ebf3	0,99	No
ENSMUSG00000010492	-2,68E-02	2,210987842	3,75E-02	8,46E-01	9,87E-01	Uckl1os	0,98	No
ENSMUSG00000010517	-2,01E-01	5,033373172	7,54E+00	6,04E-03	6,45E-02	Faf1	0,87	No
ENSMUSG00000010529	-9,37E-01	2,804348606	6,38E+01	1,38E-15	2,26E-13	Gm266	0,52	Yes
ENSMUSG00000010538	4,38E-01	-1,136587442	9,80E-01	3,22E-01	6,22E-01	Tsacc	1,35	No
ENSMUSG00000010554	-3,19E-02	4,293678642	1,48E-01	7,01E-01	9,05E-01	Mettl16	0,98	No
ENSMUSG00000010592	-5,78E-01	-1,627564382	1,11E+00	2,91E-01	5,87E-01	Dazl	0,67	No

ENSMUSG00000010601	-1,24E+00	-2,655120477	1,47E+00	2,25E-01	5,50E-01	Apol7a	0,42	No
ENSMUSG00000010607	-2,23E-01	4,017907582	6,42E+00	1,13E-02	1,02E-01	Pigyl	0,86	No
ENSMUSG00000010608	-8,15E-02	7,148519701	1,83E+00	1,76E-01	5,35E-01	Rbm25	0,95	No
ENSMUSG00000010609	-1,60E-01	5,570686475	5,49E+00	1,91E-02	1,47E-01	Psen2	0,90	No
ENSMUSG00000010651	1,26E-01	3,97167154	1,97E+00	1,61E-01	5,09E-01	Acaa1b	1,09	No
ENSMUSG00000010660	2,44E-01	3,247712627	5,14E+00	2,33E-02	1,67E-01	Plcd1	1,18	No
ENSMUSG00000010663	2,92E-01	6,671419404	2,21E+01	2,53E-06	9,89E-05	Fads1	1,22	Yes
ENSMUSG00000010721	1,88E-01	1,608409519	1,23E+00	2,67E-01	5,63E-01	Lmbr1	1,14	No
ENSMUSG00000010751	2,69E-01	3,132900973	6,10E+00	1,35E-02	1,17E-01	Tnfrsf22	1,21	No
ENSMUSG00000010755	5,95E-02	4,652152604	5,77E-01	4,47E-01	7,38E-01	Cars	1,04	No
ENSMUSG00000010760	3,51E+00	-2,770631773	5,34E+00	2,08E-02	1,55E-01	Phlda2	11,37	No
ENSMUSG00000010797	-1,94E+00	-3,107619243	1,50E+00	2,21E-01	5,50E-01	Wnt2	0,26	No
ENSMUSG00000010825	4,15E-01	-2,963274112	8,72E-02	7,68E-01	9,41E-01	Grid2ip	1,33	No
ENSMUSG00000010830	2,93E-01	0,839358979	1,73E+00	1,88E-01	5,50E-01	Kdelr3	1,23	No
ENSMUSG00000010841	-1,44E+00	-1,574012045	5,27E+00	2,17E-02	1,59E-01	1700006E09Rik	0,37	No
ENSMUSG00000010911	1,03E-02	3,672052999	1,17E-02	9,14E-01	1,00E+00	Apip	1,01	No
ENSMUSG00000010914	1,02E-01	4,33727466	1,51E+00	2,19E-01	5,50E-01	Pdhx	1,07	No
ENSMUSG00000010936	7,77E-02	6,87062998	1,61E+00	2,04E-01	5,50E-01	Vac14	1,06	No
ENSMUSG00000011008	2,95E-01	5,226390401	1,70E+01	3,81E-05	1,08E-03	Mcoln2	1,23	Yes
ENSMUSG00000011034	3,14E-02	-3,107617333	-7,69E-10	1,00E+00	1,00E+00	Slc5a1	1,02	No
ENSMUSG00000011052	-6,09E-02	-2,831981081	2,08E-03	9,64E-01	1,00E+00	1700049J03Rik	0,96	No
ENSMUSG00000011096	4,59E-02	4,964140497	3,81E-01	5,37E-01	8,09E-01	Akt1s1	1,03	No
ENSMUSG00000011114	-9,25E-02	6,112019209	2,05E+00	1,52E-01	4,94E-01	Tbrg1	0,94	No
ENSMUSG00000011118	-1,38E+00	-2,831931116	1,19E+00	2,75E-01	5,71E-01	Panx3	0,38	No
ENSMUSG00000011148	1,43E-01	4,474508613	3,10E+00	7,81E-02	3,57E-01	Adssl1	1,10	No
ENSMUSG00000011154	3,06E-01	-0,980664725	5,18E-01	4,72E-01	7,60E-01	Cfap161	1,24	No
ENSMUSG00000011158	2,37E-02	5,44895848	1,16E-01	7,34E-01	9,23E-01	Brf1	1,02	No
ENSMUSG00000011171	5,99E-01	0,057908609	4,15E+00	4,15E-02	2,42E-01	Vipr2	1,51	No
ENSMUSG00000011179	-1,11E-01	6,16891942	2,94E+00	8,66E-02	3,78E-01	Odc1	0,93	No
ENSMUSG00000011254	1,18E-01	2,850251415	1,02E+00	3,13E-01	6,14E-01	Thg1l	1,09	No
ENSMUSG00000011256	-2,88E-01	5,886509633	1,92E+01	1,17E-05	3,88E-04	Adam19	0,82	Yes
ENSMUSG00000011257	-6,53E-02	5,172102563	8,14E-01	3,67E-01	6,68E-01	Pabpc4	0,96	No

ENSMUSG00000011263	-7,10E-02	-2,448674888	6,98E-03	9,33E-01	1,00E+00	Exoc3l2	0,95	No
ENSMUSG00000011267	-3,52E-01	3,002074102	1,03E+01	1,30E-03	2,00E-02	Zfp296	0,78	Yes
ENSMUSG00000011305	-8,80E-01	-2,832047555	4,24E-01	5,15E-01	7,96E-01	Plin5	0,54	No
ENSMUSG00000011306	-7,54E-02	4,870074292	1,01E+00	3,16E-01	6,16E-01	Sugp1	0,95	No
ENSMUSG00000011350	-9,34E-01	-2,600674645	7,65E-01	3,82E-01	6,83E-01	Gm5893	0,52	No
ENSMUSG00000011382	-5,60E-02	3,619924792	3,44E-01	5,58E-01	8,22E-01	Dhdh	0,96	No
ENSMUSG00000011427	1,86E-01	2,541371786	2,30E+00	1,30E-01	4,52E-01	Zfp790	1,14	No
ENSMUSG00000011463	1,87E+00	-3,107639691	1,38E+00	2,40E-01	5,50E-01	Cpb1	3,67	No
ENSMUSG00000011486	2,74E-02	-3,033621357	9,16E-12	1,00E+00	1,00E+00	Slc25a41	1,02	No
ENSMUSG00000011589	1,54E-01	-2,107624009	4,13E-02	8,39E-01	9,83E-01	Fsd1	1,11	No
ENSMUSG00000011658	5,76E-02	4,472299077	5,09E-01	4,75E-01	7,63E-01	Fuz	1,04	No
ENSMUSG00000011751	-6,85E-01	-0,460813043	3,71E+00	5,41E-02	2,84E-01	Sptbn4	0,62	No
ENSMUSG00000011752	2,03E-01	8,080970247	1,21E+01	4,91E-04	9,04E-03	Pgam1	1,15	Yes
ENSMUSG00000011831	1,92E-01	5,658814529	7,93E+00	4,87E-03	5,49E-02	Evi5	1,14	No
ENSMUSG00000011832	7,47E-02	3,895759508	6,69E-01	4,14E-01	7,09E-01	Evi5l	1,05	No
ENSMUSG00000011837	-2,65E-02	4,002163035	8,91E-02	7,65E-01	9,40E-01	Snapc2	0,98	No
ENSMUSG00000011877	2,76E-02	6,429051605	1,92E-01	6,61E-01	8,82E-01	Git1	1,02	No
ENSMUSG00000011884	5,01E-02	8,095282776	7,46E-01	3,88E-01	6,88E-01	Gltp	1,04	No
ENSMUSG00000011958	2,96E-02	7,076489041	2,40E-01	6,24E-01	8,59E-01	Bnip2	1,02	No
ENSMUSG00000011960	-6,26E-03	5,699332373	8,68E-03	9,26E-01	1,00E+00	Ccnt1	1,00	No
ENSMUSG00000012017	-7,41E-02	1,825403211	2,27E-01	6,34E-01	8,66E-01	Scarf2	0,95	No
ENSMUSG00000012042	-1,87E+00	-3,107637761	1,39E+00	2,38E-01	5,50E-01	4930579F01Rik	0,27	No
ENSMUSG00000012076	1,77E-01	3,529076912	3,26E+00	7,12E-02	3,38E-01	Brms1l	1,13	No
ENSMUSG00000012114	1,07E-01	7,025486337	3,12E+00	7,74E-02	3,55E-01	Med15	1,08	No
ENSMUSG00000012117	1,73E-01	5,970227071	6,93E+00	8,50E-03	8,28E-02	Dhdds	1,13	No
ENSMUSG00000012123	-1,33E-01	0,299245573	2,59E-01	6,11E-01	8,52E-01	Crybg2	0,91	No
ENSMUSG00000012126	4,81E-02	6,643988707	6,03E-01	4,37E-01	7,30E-01	Ubxn11	1,03	No
ENSMUSG00000012187	9,01E-03	-3,107646452	-5,16E-10	1,00E+00	1,00E+00	Mogat1	1,01	No
ENSMUSG00000012211	-1,97E+00	-2,800969006	2,04E+00	1,53E-01	4,96E-01	Tex22	0,26	No
ENSMUSG00000012296	-3,15E-02	4,526021847	1,55E-01	6,94E-01	9,02E-01	Tjap1	0,98	No
ENSMUSG00000012350	4,67E-01	1,741715403	9,03E+00	2,66E-03	3,49E-02	Ehf	1,38	Yes
ENSMUSG00000012405	-2,65E-01	6,23989988	1,72E+01	3,44E-05	9,86E-04	Rpl15	0,83	Yes

ENSMUSG00000012422	-4,40E-02	6,372721947	4,82E-01	4,87E-01	7,74E-01	Tmem167	0,97	No
ENSMUSG00000012429	9,15E-02	3,011374801	6,69E-01	4,13E-01	7,09E-01	Mplkip	1,07	No
ENSMUSG00000012443	2,79E-01	6,000297665	1,81E+01	2,11E-05	6,51E-04	Kif11	1,21	Yes
ENSMUSG00000012483	-1,68E-01	2,819963281	1,98E+00	1,60E-01	5,07E-01	Rpa3	0,89	No
ENSMUSG00000012519	4,49E-02	4,394268975	3,01E-01	5,84E-01	8,38E-01	Mlkl	1,03	No
ENSMUSG00000012535	1,29E-01	6,28844084	4,12E+00	4,23E-02	2,45E-01	Tnpo3	1,09	No
ENSMUSG00000012609	6,58E-02	3,500726918	4,37E-01	5,09E-01	7,92E-01	Ttll5	1,05	No
ENSMUSG00000012640	2,17E-01	5,072067374	8,80E+00	3,01E-03	3,84E-02	Zfp715	1,16	Yes
ENSMUSG00000012705	1,03E+00	-2,401477134	1,36E+00	2,44E-01	5,50E-01	Retn	2,04	No
ENSMUSG00000012777	2,18E-01	3,30748169	4,21E+00	4,01E-02	2,38E-01	Acpt	1,16	No
ENSMUSG00000012819	3,83E-01	3,57811301	1,54E+01	8,66E-05	2,15E-03	Cdh23	1,30	Yes
ENSMUSG00000012848	-1,21E-01	8,487668081	4,45E+00	3,48E-02	2,21E-01	Rps5	0,92	No
ENSMUSG00000012889	4,21E-01	-2,711707266	1,38E-01	7,10E-01	9,09E-01	Podnl1	1,34	No
ENSMUSG00000013033	-8,64E-02	1,908259686	3,04E-01	5,81E-01	8,37E-01	Adgrl1	0,94	No
ENSMUSG00000013076	3,17E-01	1,229095183	2,69E+00	1,01E-01	3,92E-01	Amotl1	1,25	No
ENSMUSG00000013083	-2,65E+00	-3,033633091	2,78E+00	9,55E-02	3,86E-01	2200002J24Rik	0,16	No
ENSMUSG00000013089	2,74E-01	5,009879273	1,37E+01	2,16E-04	4,63E-03	Etv5	1,21	Yes
ENSMUSG00000013091	6,69E-01	-1,355548541	1,60E+00	2,06E-01	5,50E-01	Tmem190	1,59	No
ENSMUSG00000013150	-1,77E-01	0,814507883	6,68E-01	4,14E-01	7,09E-01	Gfod2	0,88	No
ENSMUSG00000013155	3,02E-01	2,03977716	3,99E+00	4,57E-02	2,55E-01	Enkd1	1,23	No
ENSMUSG00000013158	9,17E-02	-1,006784418	4,56E-02	8,31E-01	9,78E-01	4933405L10Rik	1,07	No
ENSMUSG00000013160	3,24E-02	7,993116783	3,11E-01	5,77E-01	8,35E-01	Atp6v0d1	1,02	No
ENSMUSG00000013236	-1,82E-01	6,863395168	8,81E+00	2,99E-03	3,82E-02	Ptprs	0,88	Yes
ENSMUSG00000013275	-1,52E-01	5,467061687	4,90E+00	2,68E-02	1,83E-01	Slc41a1	0,90	No
ENSMUSG00000013338	7,19E-01	-2,655190923	2,82E-01	5,95E-01	8,44E-01	Fer1l4	1,65	No
ENSMUSG00000013353	-8,62E-01	-2,574116638	8,67E-01	3,52E-01	6,53E-01	4931406B18Rik	0,55	No
ENSMUSG00000013367	1,43E-01	0,545062344	3,78E-01	5,39E-01	8,09E-01	Iglon5	1,10	No
ENSMUSG00000013415	-3,71E-01	0,375789516	2,25E+00	1,34E-01	4,60E-01	Igf2bp1	0,77	No
ENSMUSG00000013418	-3,67E-01	0,73197791	2,57E+00	1,09E-01	4,04E-01	B4galnt2	0,78	No
ENSMUSG00000013419	-5,28E-01	0,647620634	5,07E+00	2,44E-02	1,72E-01	Zfp651	0,69	No
ENSMUSG00000013465	-6,52E-02	6,126917995	1,02E+00	3,13E-01	6,12E-01	Nelfb	0,96	No
ENSMUSG00000013483	8,37E-02	2,290383098	3,75E-01	5,40E-01	8,10E-01	Card14	1,06	No

ENSMUSG00000013495	9,66E-02	4,47357423	1,45E+00	2,28E-01	5,50E-01	Tmem175	1,07	No
ENSMUSG00000013523	-3,70E-01	2,19504905	6,79E+00	9,16E-03	8,74E-02	Bcas1	0,77	No
ENSMUSG00000013539	8,49E-02	4,71994945	1,21E+00	2,72E-01	5,68E-01	Tango2	1,06	No
ENSMUSG00000013562	3,91E-01	-1,801049151	3,69E-01	5,44E-01	8,12E-01	4833413E03Rik	1,31	No
ENSMUSG00000013584	-6,42E-01	7,133761692	1,14E+02	1,62E-26	6,39E-24	Aldh1a2	0,64	Yes
ENSMUSG00000013593	-2,09E-02	6,740121375	1,15E-01	7,35E-01	9,24E-01	Ndufs2	0,99	No
ENSMUSG00000013622	1,14E-01	4,860357841	2,25E+00	1,33E-01	4,59E-01	Atraid	1,08	No
ENSMUSG00000013629	2,75E-02	6,996291098	2,06E-01	6,50E-01	8,76E-01	Cad	1,02	No
ENSMUSG00000013646	1,58E-01	4,610376016	4,07E+00	4,37E-02	2,49E-01	Sh3bp5l	1,12	No
ENSMUSG00000013662	5,66E-02	5,999351509	7,47E-01	3,87E-01	6,87E-01	Atad1	1,04	No
ENSMUSG00000013663	8,04E-03	7,237615835	1,80E-02	8,93E-01	1,00E+00	Pten	1,01	No
ENSMUSG00000013698	1,38E-01	8,277477982	5,70E+00	1,69E-02	1,36E-01	Pea15a	1,10	No
ENSMUSG00000013701	5,91E-02	4,486660648	5,25E-01	4,69E-01	7,57E-01	Timm23	1,04	No
ENSMUSG00000013707	1,52E-01	5,622679884	4,96E+00	2,60E-02	1,80E-01	Tnfaip8l2	1,11	No
ENSMUSG00000013736	1,23E-01	4,9724152	2,76E+00	9,69E-02	3,88E-01	Trnt1	1,09	No
ENSMUSG00000013766	2,61E+00	-2,929330338	2,64E+00	1,04E-01	3,97E-01	Ly6g6e	6,10	No
ENSMUSG00000013787	-1,19E-01	7,669044196	4,10E+00	4,28E-02	2,46E-01	Ehmt2	0,92	No
ENSMUSG00000013822	-5,31E-02	5,366581219	5,70E-01	4,50E-01	7,40E-01	Elof1	0,96	No
ENSMUSG00000013833	-1,38E-01	5,268930155	3,74E+00	5,31E-02	2,81E-01	Med16	0,91	No
ENSMUSG00000013846	6,85E-02	7,728697594	1,36E+00	2,43E-01	5,50E-01	St3gal1	1,05	No
ENSMUSG00000013858	-2,35E-02	6,367537938	1,38E-01	7,10E-01	9,10E-01	Tmem259	0,98	No
ENSMUSG00000013878	-7,92E-02	3,97405964	7,99E-01	3,71E-01	6,72E-01	Rnf170	0,95	No
ENSMUSG00000013921	-1,70E+00	-2,497510488	3,20E+00	7,38E-02	3,45E-01	Clip3	0,31	No
ENSMUSG00000013928	4,93E-02	-0,03345436	2,71E-02	8,69E-01	1,00E+00	Thap8	1,03	No
ENSMUSG00000013936	-3,09E-02	-2,832023973	6,09E-04	9,80E-01	1,00E+00	Myl2	0,98	No
ENSMUSG00000013973	-1,07E-01	4,346603459	1,70E+00	1,92E-01	5,50E-01	Dedd	0,93	No
ENSMUSG00000013974	-1,34E-02	4,537945907	2,79E-02	8,67E-01	9,99E-01	Mcemp1	0,99	No
ENSMUSG00000013997	-1,13E-01	5,290313459	2,54E+00	1,11E-01	4,10E-01	Nit1	0,92	No
ENSMUSG00000014030	-2,03E+00	-1,587306643	8,86E+00	2,92E-03	3,76E-02	Pax5	0,25	Yes
ENSMUSG00000014039	6,37E-03	4,105863997	5,52E-03	9,41E-01	1,00E+00	Prdm15	1,00	No
ENSMUSG00000014074	2,00E-01	4,804328407	6,96E+00	8,36E-03	8,19E-02	Rnf168	1,15	No
ENSMUSG00000014075	-3,59E-01	2,736541353	8,80E+00	3,01E-03	3,84E-02	Tctex1d2	0,78	Yes

ENSMUSG00000014077	-5,64E-02	7,287903015	8,91E-01	3,45E-01	6,46E-01	Chp1	0,96	No
ENSMUSG00000014158	4,41E-01	4,443995988	2,80E+01	1,22E-07	6,68E-06	Trpv4	1,36	Yes
ENSMUSG00000014164	1,29E-01	3,632205338	1,79E+00	1,81E-01	5,43E-01	Klhl3	1,09	No
ENSMUSG00000014177	4,52E-02	3,729597287	2,31E-01	6,31E-01	8,63E-01	Tvp23b	1,03	No
ENSMUSG00000014195	-1,45E-01	6,162015347	5,07E+00	2,43E-02	1,71E-01	Dnajc7	0,90	No
ENSMUSG00000014198	-1,66E-01	0,26992866	3,66E-01	5,45E-01	8,13E-01	Zfp385c	0,89	No
ENSMUSG00000014226	5,11E-02	5,557781351	5,53E-01	4,57E-01	7,46E-01	Cacybp	1,04	No
ENSMUSG00000014232	-3,37E-02	2,583660301	6,97E-02	7,92E-01	9,55E-01	Cluap1	0,98	No
ENSMUSG00000014243	2,90E-02	2,709253129	5,52E-02	8,14E-01	9,68E-01	Zswim7	1,02	No
ENSMUSG00000014245	3,49E-02	2,704142232	8,16E-02	7,75E-01	9,45E-01	Pigl	1,02	No
ENSMUSG00000014294	-7,25E-02	5,531736572	1,11E+00	2,93E-01	5,88E-01	Ndufa2	0,95	No
ENSMUSG00000014301	-1,64E-01	0,511912271	4,56E-01	4,99E-01	7,85E-01	Pam16	0,89	No
ENSMUSG00000014303	4,25E-01	0,206389079	2,38E+00	1,23E-01	4,38E-01	Glis2	1,34	No
ENSMUSG00000014313	-3,90E-02	5,197094592	2,94E-01	5,88E-01	8,40E-01	Cox6c	0,97	No
ENSMUSG00000014329	-5,62E-01	-2,574192075	3,38E-01	5,61E-01	8,24E-01	Bicc1	0,68	No
ENSMUSG00000014349	-7,58E-02	7,122142017	1,59E+00	2,08E-01	5,50E-01	Ube2z	0,95	No
ENSMUSG00000014351	1,36E-02	-2,963265738	-4,74E-10	1,00E+00	1,00E+00	Gip	1,01	No
ENSMUSG00000014353	1,44E-01	5,92713247	4,77E+00	2,90E-02	1,94E-01	Tmem87b	1,11	No
ENSMUSG00000014355	4,73E-02	7,025675406	6,08E-01	4,35E-01	7,28E-01	Anapc1	1,03	No
ENSMUSG00000014361	1,98E-01	6,663562865	1,01E+01	1,48E-03	2,21E-02	Mertk	1,15	Yes
ENSMUSG00000014402	4,07E-02	5,499096798	3,47E-01	5,56E-01	8,21E-01	Tsg101	1,03	No
ENSMUSG00000014418	1,54E-01	6,254641819	5,85E+00	1,56E-02	1,29E-01	Hps5	1,11	No
ENSMUSG00000014426	2,51E-01	4,838247559	1,08E+01	1,01E-03	1,63E-02	Map3k4	1,19	Yes
ENSMUSG00000014444	1,34E-01	8,07343462	5,34E+00	2,08E-02	1,55E-01	Piezo1	1,10	No
ENSMUSG00000014453	2,61E-01	-1,015752117	2,84E-01	5,94E-01	8,43E-01	Blk	1,20	No
ENSMUSG00000014470	1,64E-01	6,267672099	6,57E+00	1,03E-02	9,58E-02	Rnf166	1,12	No
ENSMUSG00000014496	2,67E-01	4,482250818	1,07E+01	1,09E-03	1,74E-02	Ankrd28	1,20	Yes
ENSMUSG00000014498	2,69E-02	7,057487185	1,99E-01	6,56E-01	8,80E-01	Ankrd52	1,02	No
ENSMUSG00000014503	-9,77E-02	3,789307132	1,12E+00	2,90E-01	5,86E-01	Pkd2l2	0,93	No
ENSMUSG00000014504	-1,44E-02	4,958862858	3,67E-02	8,48E-01	9,88E-01	Srp19	0,99	No
ENSMUSG00000014529	-1,67E-02	-2,963250293	1,29E-04	9,91E-01	1,00E+00	Tmbim7	0,99	No
ENSMUSG00000014543	-5,88E-02	2,01476345	1,53E-01	6,96E-01	9,03E-01	Klra17	0,96	No

ENSMUSG00000014547	2,94E-01	8,341196707	2,59E+01	3,62E-07	1,78E-05	Wdfy2		1,23	Yes
ENSMUSG00000014550	4,37E-02	5,364115748	3,84E-01	5,35E-01	8,08E-01	Rbsn		1,03	No
ENSMUSG00000014551	-1,08E-01	5,152595885	2,23E+00	1,35E-01	4,63E-01	Mrps25		0,93	No
ENSMUSG00000014554	-5,55E-02	4,432677242	4,77E-01	4,90E-01	7,76E-01	Dguok		0,96	No
ENSMUSG00000014592	5,78E-02	2,602485823	2,17E-01	6,42E-01	8,71E-01	Camta1		1,04	No
ENSMUSG00000014599	-3,12E-01	6,450710023	2,42E+01	8,58E-07	3,89E-05	Csf1		0,81	Yes
ENSMUSG00000014601	2,07E-01	5,649578791	9,23E+00	2,38E-03	3,22E-02	Strip1		1,15	Yes
ENSMUSG00000014602	5,65E-01	3,756955361	3,56E+01	2,43E-09	1,86E-07	Kif1a		1,48	Yes
ENSMUSG00000014606	-9,12E-03	6,681038074	2,17E-02	8,83E-01	1,00E+00	Slc25a11		0,99	No
ENSMUSG00000014609	5,08E-02	-1,10765267	1,36E-02	9,07E-01	1,00E+00	Chrne		1,04	No
ENSMUSG00000014633	-4,14E-01	4,124367224	2,32E+01	1,43E-06	6,08E-05	Cmc2		0,75	Yes
ENSMUSG00000014668	-3,47E-02	6,319084294	2,99E-01	5,85E-01	8,38E-01	Chfr		0,98	No
ENSMUSG00000014686	9,77E-02	0,934370469	2,11E-01	6,46E-01	8,74E-01	Ceacam16		1,07	No
ENSMUSG00000014704	-8,63E-02	-2,548227689	6,73E-03	9,35E-01	1,00E+00	Hoxa2		0,94	No
ENSMUSG00000014747	2,07E-01	-0,185724501	4,38E-01	5,08E-01	7,92E-01	Ankrd53		1,15	No
ENSMUSG00000014748	1,44E-01	6,940180344	5,61E+00	1,79E-02	1,40E-01	Tex261		1,11	No
ENSMUSG00000014763	-1,13E-01	5,004486245	2,36E+00	1,25E-01	4,42E-01	Fam120b		0,92	No
ENSMUSG00000014767	-1,52E-02	4,832476694	4,04E-02	8,41E-01	9,84E-01	Tbp		0,99	No
ENSMUSG00000014769	-1,92E-01	6,429109045	9,23E+00	2,39E-03	3,23E-02	Psmb1		0,88	Yes
ENSMUSG00000014771	-3,29E-02	2,974534794	8,38E-02	7,72E-01	9,43E-01	Pdcd2		0,98	No
ENSMUSG00000014773	-1,40E+00	-0,704421173	1,25E+01	4,03E-04	7,67E-03	Dll1		0,38	Yes
ENSMUSG00000014776	-2,54E-01	0,42614927	9,77E-01	3,23E-01	6,23E-01	Nol3		0,84	No
ENSMUSG00000014778	1,24E-02	5,587146499	3,32E-02	8,55E-01	9,92E-01	Fhod1		1,01	No
ENSMUSG00000014782	-3,75E-01	0,429318	2,11E+00	1,47E-01	4,85E-01	Plekhg4		0,77	No
ENSMUSG00000014786	9,82E-02	3,667732482	1,06E+00	3,03E-01	6,00E-01	Slc9a5		1,07	No
ENSMUSG00000014791	-1,32E-01	1,538061962	5,73E-01	4,49E-01	7,39E-01	Elmo3		0,91	No
ENSMUSG00000014813	-1,92E+00	-2,963268013	1,46E+00	2,27E-01	5,50E-01	Stc1		0,26	No
ENSMUSG00000014837	-8,75E-02	1,878207089	3,29E-01	5,66E-01	8,29E-01	4931428F04Rik		0,94	No
ENSMUSG00000014846	4,68E-01	4,2630299	2,97E+01	5,13E-08	3,04E-06	Tppp3		1,38	Yes
ENSMUSG00000014850	1,46E-01	3,647496337	2,24E+00	1,34E-01	4,61E-01	Msh3		1,11	No
ENSMUSG00000014852	-7,02E-01	-0,785452221	2,70E+00	1,00E-01	3,92E-01	Adamts13		0,61	No
ENSMUSG00000014856	3,47E-02	4,374746449	1,80E-01	6,71E-01	8,89E-01	Tmem208		1,02	No

ENSMUSG00000014859	2,84E-02	6,001257664	1,89E-01	6,64E-01	8,84E-01	E2f4		1,02	No
ENSMUSG00000014867	5,45E-02	8,430329789	8,96E-01	3,44E-01	6,44E-01	Surf4		1,04	No
ENSMUSG00000014873	1,78E-02	4,380771708	4,71E-02	8,28E-01	9,76E-01	Surf2		1,01	No
ENSMUSG00000014905	1,52E-01	5,295189347	4,59E+00	3,21E-02	2,08E-01	Dnajb9		1,11	No
ENSMUSG00000014907	2,24E-01	3,206777467	4,36E+00	3,69E-02	2,28E-01	Naf1		1,17	No
ENSMUSG00000014932	-7,88E-01	1,814849538	2,55E+01	4,37E-07	2,11E-05	Yes1		0,58	Yes
ENSMUSG00000014956	5,11E-02	7,108689803	7,18E-01	3,97E-01	6,97E-01	Ppp1cb		1,04	No
ENSMUSG00000014959	-2,38E-02	6,836553532	1,51E-01	6,97E-01	9,03E-01	Gorasp2		0,98	No
ENSMUSG00000014980	2,92E-01	2,47261733	4,91E+00	2,67E-02	1,83E-01	Tsen15		1,22	No
ENSMUSG00000015001	7,35E-01	-2,896163881	2,94E-01	5,87E-01	8,40E-01	Oc90		1,66	No
ENSMUSG00000015002	6,80E-02	6,81475992	1,23E+00	2,68E-01	5,65E-01	Efr3a		1,05	No
ENSMUSG00000015013	6,16E-02	4,432465642	5,51E-01	4,58E-01	7,47E-01	Trappc2l		1,04	No
ENSMUSG00000015016	2,93E-02	3,970669977	1,07E-01	7,43E-01	9,28E-01	Acsf3		1,02	No
ENSMUSG00000015023	-4,08E-02	5,501107853	3,46E-01	5,56E-01	8,21E-01	Ddx19a		0,97	No
ENSMUSG00000015027	4,56E-02	5,035755107	3,83E-01	5,36E-01	8,08E-01	Galns		1,03	No
ENSMUSG00000015053	-3,21E-01	2,097741062	5,01E+00	2,52E-02	1,76E-01	Gata2		0,80	No
ENSMUSG00000015083	-5,75E-02	2,027016104	1,39E-01	7,10E-01	9,09E-01	C8g		0,96	No
ENSMUSG00000015085	-9,39E-01	-1,929212473	1,62E+00	2,03E-01	5,50E-01	Entpd2		0,52	No
ENSMUSG00000015087	5,82E-02	6,187302332	8,16E-01	3,66E-01	6,67E-01	Rabl6		1,04	No
ENSMUSG00000015090	9,01E-03	-3,107646452	-5,16E-10	1,00E+00	1,00E+00	Ptgds		1,01	No
ENSMUSG00000015092	-1,10E-01	5,831778312	2,74E+00	9,77E-02	3,90E-01	Edf1		0,93	No
ENSMUSG00000015093	-6,00E-01	-0,561093951	2,61E+00	1,06E-01	3,99E-01	Clic3		0,66	No
ENSMUSG00000015094	-6,34E-02	1,198939168	1,08E-01	7,42E-01	9,28E-01	Npdc1		0,96	No
ENSMUSG00000015095	1,65E-02	5,762003574	6,08E-02	8,05E-01	9,63E-01	Fbxw5		1,01	No
ENSMUSG00000015112	9,43E-02	5,000092788	1,64E+00	2,01E-01	5,50E-01	Slc25a13		1,07	No
ENSMUSG00000015120	-1,55E-01	6,708730491	6,29E+00	1,21E-02	1,08E-01	Ube2i		0,90	No
ENSMUSG00000015126	-2,01E-01	3,312578517	3,70E+00	5,45E-02	2,86E-01	Tsr3		0,87	No
ENSMUSG00000015127	-2,24E-02	3,744104012	5,86E-02	8,09E-01	9,65E-01	Unkl		0,98	No
ENSMUSG00000015133	-2,94E-01	7,772209489	2,52E+01	5,20E-07	2,47E-05	Lrrk1		0,82	Yes
ENSMUSG00000015134	-1,12E+00	-1,831997996	2,86E+00	9,06E-02	3,82E-01	Aldh1a3		0,46	No
ENSMUSG00000015143	-1,63E-01	8,250732185	7,95E+00	4,81E-03	5,43E-02	Actn1		0,89	No
ENSMUSG00000015149	-1,00E-03	6,147114796	2,41E-04	9,88E-01	1,00E+00	Sirt2		1,00	No

ENSMUSG00000015165	-4,41E-03	7,680126739	5,62E-03	9,40E-01	1,00E+00	Hnrnpl	1,00	No
ENSMUSG00000015176	1,33E-01	5,949202795	4,05E+00	4,42E-02	2,50E-01	Nolc1	1,10	No
ENSMUSG00000015189	1,34E-01	4,621938294	2,92E+00	8,74E-02	3,78E-01	Casd1	1,10	No
ENSMUSG00000015202	1,78E-01	4,767906126	5,32E+00	2,11E-02	1,57E-01	Cnksr3	1,13	No
ENSMUSG00000015222	-3,84E-01	-1,896180708	3,42E-01	5,58E-01	8,22E-01	Map2	0,77	No
ENSMUSG00000015224	2,22E-01	-2,401386689	6,22E-02	8,03E-01	9,62E-01	Cyp2j9	1,17	No
ENSMUSG00000015243	1,80E-01	7,777295265	9,46E+00	2,10E-03	2,90E-02	Abca1	1,13	Yes
ENSMUSG00000015247	3,63E-02	5,459293187	2,72E-01	6,02E-01	8,48E-01	Nipsnap3b	1,03	No
ENSMUSG00000015305	2,23E-01	5,427203297	9,94E+00	1,61E-03	2,36E-02	Sash1	1,17	Yes
ENSMUSG00000015312	-5,31E-01	4,991631392	5,28E+01	3,65E-13	4,72E-11	Gadd45b	0,69	Yes
ENSMUSG00000015314	3,59E-03	2,852042547	9,35E-04	9,76E-01	1,00E+00	Slamf6	1,00	No
ENSMUSG00000015316	-1,05E+00	2,199328046	5,78E+01	2,85E-14	4,07E-12	Slamf1	0,48	Yes
ENSMUSG00000015335	9,33E-02	4,542096444	1,36E+00	2,43E-01	5,50E-01	Zdhhc12	1,07	No
ENSMUSG00000015337	9,39E-02	3,189686166	7,65E-01	3,82E-01	6,83E-01	Endog	1,07	No
ENSMUSG00000015341	-2,40E-02	6,746880479	1,52E-01	6,97E-01	9,03E-01	Golga7	0,98	No
ENSMUSG00000015354	-3,83E-02	-3,107651936	-7,91E-11	1,00E+00	1,00E+00	Pcolce2	0,97	No
ENSMUSG00000015355	-5,17E-03	6,906694374	7,20E-03	9,32E-01	1,00E+00	Cd48	1,00	No
ENSMUSG00000015357	1,01E-01	4,581794984	1,62E+00	2,03E-01	5,50E-01	Clpx	1,07	No
ENSMUSG00000015363	-4,80E-02	6,181661879	5,58E-01	4,55E-01	7,45E-01	Trabd	0,97	No
ENSMUSG00000015365	1,11E+00	-2,655174566	1,17E+00	2,79E-01	5,75E-01	Mov10l1	2,16	No
ENSMUSG00000015377	-2,66E-01	2,106262323	3,38E+00	6,61E-02	3,23E-01	Dennd6b	0,83	No
ENSMUSG00000015396	-7,48E-01	7,461340037	1,58E+02	2,75E-36	2,50E-33	Cd83	0,60	Yes
ENSMUSG00000015443	-1,94E+00	-3,107619243	1,50E+00	2,21E-01	5,50E-01	Gzmn	0,26	No
ENSMUSG00000015451	-2,49E-01	1,349112669	1,81E+00	1,78E-01	5,39E-01	C4a	0,84	No
ENSMUSG00000015452	-1,41E-01	1,049621663	4,78E-01	4,89E-01	7,76E-01	Ager	0,91	No
ENSMUSG00000015461	1,18E-01	5,537415567	2,96E+00	8,54E-02	3,76E-01	Atf6b	1,09	No
ENSMUSG00000015467	9,27E-02	0,836852383	1,71E-01	6,79E-01	8,93E-01	Egfl8	1,07	No
ENSMUSG00000015468	6,84E-03	-1,378271254	1,70E-04	9,90E-01	1,00E+00	Notch4	1,00	No
ENSMUSG00000015474	1,38E-01	4,484584837	2,88E+00	8,94E-02	3,79E-01	Ppt2	1,10	No
ENSMUSG00000015476	7,89E-01	-1,448820184	1,94E+00	1,64E-01	5,15E-01	Prrt1	1,73	No
ENSMUSG00000015478	-8,05E-02	4,072698429	8,41E-01	3,59E-01	6,61E-01	Rnf5	0,95	No
ENSMUSG00000015484	-3,83E-02	-3,107651936	-7,91E-11	1,00E+00	1,00E+00	Fam163a	0,97	No

ENSMUSG00000015488	-1,22E-01	4,472078247	2,33E+00	1,27E-01	4,46E-01	Cacfd1	0,92	No
ENSMUSG00000015501	-2,65E-01	4,02904833	9,22E+00	2,39E-03	3,23E-02	Hivep2	0,83	Yes
ENSMUSG00000015522	1,11E-01	6,04217031	2,88E+00	8,97E-02	3,80E-01	Arnt	1,08	No
ENSMUSG00000015533	1,73E-01	-0,69721133	1,75E-01	6,76E-01	8,91E-01	Itga2	1,13	No
ENSMUSG00000015536	6,64E-02	4,81450136	7,60E-01	3,83E-01	6,85E-01	Mocs2	1,05	No
ENSMUSG00000015542	5,07E-02	3,406631695	2,50E-01	6,17E-01	8,55E-01	Nat9	1,04	No
ENSMUSG00000015568	3,51E-02	11,34658762	3,95E-01	5,30E-01	8,05E-01	Lpl	1,02	No
ENSMUSG00000015575	-2,86E-02	7,81574644	2,38E-01	6,26E-01	8,60E-01	Atp6v0e	0,98	No
ENSMUSG00000015579	1,85E+00	-3,107657832	1,35E+00	2,46E-01	5,50E-01	Nkx2-5	3,61	No
ENSMUSG00000015597	-4,11E-02	5,634901345	3,68E-01	5,44E-01	8,13E-01	Zfp318	0,97	No
ENSMUSG00000015599	-9,47E-01	-2,655129153	7,86E-01	3,75E-01	6,76E-01	Ttbk1	0,52	No
ENSMUSG00000015605	-1,80E-02	6,176591309	7,74E-02	7,81E-01	9,49E-01	Srf	0,99	No
ENSMUSG00000015619	6,91E-01	-1,574219033	1,43E+00	2,33E-01	5,50E-01	Gata3	1,61	No
ENSMUSG00000015647	-1,25E+00	1,181555975	3,91E+01	4,12E-10	3,59E-08	Lama5	0,42	Yes
ENSMUSG00000015652	7,31E-01	-2,963267542	2,91E-01	5,90E-01	8,41E-01	Steap1	1,66	No
ENSMUSG00000015653	-2,65E+00	-2,896109668	2,80E+00	9,45E-02	3,85E-01	Steap2	0,16	No
ENSMUSG00000015656	-1,13E-01	9,320187062	3,99E+00	4,59E-02	2,55E-01	Hspa8	0,92	No
ENSMUSG00000015659	1,56E-01	3,031072285	2,00E+00	1,57E-01	5,02E-01	Serac1	1,11	No
ENSMUSG00000015671	-1,10E-01	5,183095365	2,33E+00	1,27E-01	4,46E-01	Psma2	0,93	No
ENSMUSG00000015672	-1,52E-01	4,274192119	3,27E+00	7,06E-02	3,36E-01	Mrpl32	0,90	No
ENSMUSG00000015697	1,96E-01	6,277773374	9,46E+00	2,10E-03	2,91E-02	Setdb1	1,15	Yes
ENSMUSG00000015702	6,28E-02	2,140438111	1,90E-01	6,63E-01	8,83E-01	Anxa9	1,04	No
ENSMUSG00000015709	2,68E-02	3,908845614	8,66E-02	7,69E-01	9,41E-01	Arnt2	1,02	No
ENSMUSG00000015711	-8,80E-02	4,82634913	1,32E+00	2,51E-01	5,50E-01	Prune1	0,94	No
ENSMUSG00000015714	7,12E-02	7,331080241	1,42E+00	2,34E-01	5,50E-01	Cers2	1,05	No
ENSMUSG00000015733	8,96E-02	8,643907035	2,45E+00	1,18E-01	4,26E-01	Capza2	1,06	No
ENSMUSG00000015745	2,20E-02	7,664365171	1,39E-01	7,09E-01	9,09E-01	Plekho1	1,02	No
ENSMUSG00000015747	1,47E-01	4,328353404	3,12E+00	7,74E-02	3,55E-01	Vps45	1,11	No
ENSMUSG00000015748	8,58E-02	4,832512049	1,29E+00	2,57E-01	5,50E-01	Prpf3	1,06	No
ENSMUSG00000015749	9,47E-02	6,943884753	2,41E+00	1,20E-01	4,32E-01	Anp32e	1,07	No
ENSMUSG00000015750	1,79E-02	6,383039864	7,99E-02	7,77E-01	9,47E-01	Aph1a	1,01	No
ENSMUSG00000015755	-5,11E-02	7,2615515	7,29E-01	3,93E-01	6,93E-01	Tab2	0,97	No

ENSMUSG00000015757	1,17E-02	5,099669036	2,58E-02	8,72E-01	1,00E+00	Ppil4	1,01	No
ENSMUSG00000015759	6,36E-03	6,154975928	9,70E-03	9,22E-01	1,00E+00	Cnih1	1,00	No
ENSMUSG00000015766	3,24E-01	7,228090504	2,90E+01	7,29E-08	4,18E-06	Eps8	1,25	Yes
ENSMUSG00000015776	3,45E-02	6,005560554	2,80E-01	5,96E-01	8,45E-01	Med22	1,02	No
ENSMUSG00000015790	-1,46E-01	4,211087766	2,96E+00	8,52E-02	3,75E-01	Surf1	0,90	No
ENSMUSG00000015804	-8,35E-02	5,621027542	1,50E+00	2,20E-01	5,50E-01	Med28	0,94	No
ENSMUSG00000015806	5,15E-02	5,931688804	6,10E-01	4,35E-01	7,28E-01	Qdpr	1,04	No
ENSMUSG00000015812	-2,70E-01	0,309945691	1,12E+00	2,91E-01	5,86E-01	Gnrh1	0,83	No
ENSMUSG00000015829	-7,93E-02	-1,522538944	2,20E-02	8,82E-01	1,00E+00	Tnr	0,95	No
ENSMUSG00000015837	-2,78E-01	8,555279389	2,34E+01	1,32E-06	5,72E-05	Sqstm1	0,83	Yes
ENSMUSG00000015839	1,36E-01	8,353752885	5,55E+00	1,85E-02	1,44E-01	Nfe2l2	1,10	No
ENSMUSG00000015846	3,54E-01	7,110248849	3,39E+01	5,88E-09	4,27E-07	Rxra	1,28	Yes
ENSMUSG00000015850	-3,06E-01	4,457706076	1,43E+01	1,57E-04	3,53E-03	Adamtsl4	0,81	Yes
ENSMUSG00000015852	7,39E-01	7,350698899	1,50E+02	1,91E-34	1,47E-31	Fcrls	1,67	Yes
ENSMUSG00000015854	-2,56E-01	2,203122011	3,14E+00	7,64E-02	3,52E-01	Cd5l	0,84	No
ENSMUSG00000015869	-4,58E-02	5,141583759	4,02E-01	5,26E-01	8,03E-01	Prpsap1	0,97	No
ENSMUSG00000015879	1,83E-01	-1,060891142	1,66E-01	6,83E-01	8,95E-01	Fam184b	1,14	No
ENSMUSG00000015880	2,27E-01	4,931725623	9,28E+00	2,32E-03	3,15E-02	Ncapg	1,17	Yes
ENSMUSG00000015882	9,60E-02	4,677613352	1,51E+00	2,20E-01	5,50E-01	Lcorl	1,07	No
ENSMUSG00000015889	1,09E-01	7,370592227	3,35E+00	6,74E-02	3,27E-01	Lta4h	1,08	No
ENSMUSG00000015890	3,99E-01	-2,107482367	2,54E-01	6,14E-01	8,53E-01	Amdhd1	1,32	No
ENSMUSG00000015932	-1,23E-01	8,345456194	4,54E+00	3,31E-02	2,13E-01	Dstn	0,92	No
ENSMUSG00000015937	-4,41E-02	8,138004569	5,77E-01	4,47E-01	7,38E-01	H2afy	0,97	No
ENSMUSG00000015942	-2,43E-02	5,95852784	1,37E-01	7,12E-01	9,11E-01	Gtf2ird2	0,98	No
ENSMUSG00000015943	7,54E-02	2,628385238	3,59E-01	5,49E-01	8,16E-01	Bola1	1,05	No
ENSMUSG00000015944	-4,38E-01	6,575336404	4,98E+01	1,74E-12	2,06E-10	Gatsl2	0,74	Yes
ENSMUSG00000015947	4,80E-01	6,920732552	6,10E+01	5,81E-15	9,07E-13	Fcgr1	1,39	Yes
ENSMUSG00000015950	-6,65E-02	8,669971763	1,35E+00	2,45E-01	5,50E-01	Ncf1	0,95	No
ENSMUSG00000015957	-5,97E-01	3,166091979	3,08E+01	2,89E-08	1,81E-06	Wnt11	0,66	Yes
ENSMUSG00000015961	4,39E-02	5,628069806	4,15E-01	5,20E-01	7,99E-01	Adss	1,03	No
ENSMUSG00000015962	9,01E-03	-3,107646452	-5,16E-10	1,00E+00	1,00E+00	1700016C15Rik	1,01	No
ENSMUSG00000015966	-1,06E-01	-1,561033296	3,49E-02	8,52E-01	9,90E-01	Il17rb	0,93	No

ENSMUSG00000015968	-5,79E-01	3,224593594	3,08E+01	2,88E-08	1,80E-06	Cacna1d	0,67	Yes
ENSMUSG00000015970	-1,93E-01	0,079065771	4,77E-01	4,90E-01	7,76E-01	Chdh	0,87	No
ENSMUSG00000015971	1,70E-02	4,683667389	4,81E-02	8,26E-01	9,75E-01	Actr8	1,01	No
ENSMUSG00000015980	-6,06E-02	2,546369244	2,21E-01	6,38E-01	8,68E-01	Lrrc27	0,96	No
ENSMUSG00000015981	2,39E-02	4,17304781	7,86E-02	7,79E-01	9,48E-01	Stk32c	1,02	No
ENSMUSG00000015994	6,43E-02	5,568330136	8,80E-01	3,48E-01	6,49E-01	Fnta	1,05	No
ENSMUSG00000016018	9,37E-03	5,546813283	1,85E-02	8,92E-01	1,00E+00	Skiv2l2	1,01	No
ENSMUSG00000016024	8,44E-02	-0,998032625	3,33E-02	8,55E-01	9,92E-01	Lbp	1,06	No
ENSMUSG00000016028	5,48E-01	2,25430511	1,41E+01	1,71E-04	3,81E-03	Celsr1	1,46	Yes
ENSMUSG00000016087	8,52E-02	5,222579038	1,39E+00	2,38E-01	5,50E-01	Fli1	1,06	No
ENSMUSG00000016128	3,84E-01	-1,126685921	6,81E-01	4,09E-01	7,06E-01	Stard13	1,31	No
ENSMUSG00000016179	-2,65E+00	-3,033633091	2,78E+00	9,55E-02	3,86E-01	Camk1g	0,16	No
ENSMUSG00000016181	1,79E-01	3,976980528	4,14E+00	4,19E-02	2,43E-01	Diexf	1,13	No
ENSMUSG00000016194	-1,98E-01	1,52088824	1,22E+00	2,70E-01	5,66E-01	Hsd11b1	0,87	No
ENSMUSG00000016200	-3,49E-02	-2,9632767	7,31E-11	1,00E+00	1,00E+00	Syt14	0,98	No
ENSMUSG00000016206	-3,68E-02	5,494629501	2,83E-01	5,95E-01	8,44E-01	H2-M3	0,97	No
ENSMUSG00000016252	-1,20E-01	6,791354223	3,78E+00	5,19E-02	2,78E-01	Atp5e	0,92	No
ENSMUSG00000016253	-1,51E-01	5,173296217	4,38E+00	3,65E-02	2,27E-01	Nelfcd	0,90	No
ENSMUSG00000016255	5,16E-01	-0,413112368	2,47E+00	1,16E-01	4,21E-01	Tubb1	1,43	No
ENSMUSG00000016256	-6,11E-02	10,34377873	1,19E+00	2,76E-01	5,72E-01	Ctsz	0,96	No
ENSMUSG00000016257	-8,51E-02	5,885907752	1,65E+00	1,99E-01	5,50E-01	Prelid3b	0,94	No
ENSMUSG00000016262	-2,74E+00	-3,033596063	2,99E+00	8,38E-02	3,71E-01	Sertad4	0,15	No
ENSMUSG00000016283	-1,69E-01	7,526933286	8,20E+00	4,18E-03	4,89E-02	H2-M2	0,89	Yes
ENSMUSG00000016344	-5,75E-01	5,106731293	6,33E+01	1,76E-15	2,79E-13	Ppdpf	0,67	Yes
ENSMUSG00000016346	-1,14E-02	-3,033635021	1,50E-05	9,97E-01	1,00E+00	Kcnq2	0,99	No
ENSMUSG00000016349	3,67E-01	3,087459396	1,11E+01	8,71E-04	1,44E-02	Eef1a2	1,29	Yes
ENSMUSG00000016356	3,97E-01	1,511384894	5,03E+00	2,49E-02	1,74E-01	Col20a1	1,32	No
ENSMUSG00000016458	-1,94E+00	-3,107619243	1,50E+00	2,21E-01	5,50E-01	Wt1	0,26	No
ENSMUSG00000016477	1,38E-01	4,432576618	2,85E+00	9,16E-02	3,82E-01	E2f3	1,10	No
ENSMUSG00000016481	-2,15E-03	5,200837906	8,93E-04	9,76E-01	1,00E+00	Cr1l	1,00	No
ENSMUSG00000016487	1,47E-01	6,08563104	5,12E+00	2,37E-02	1,69E-01	Ppfibp1	1,11	No
ENSMUSG00000016493	-4,20E-02	0,122848063	2,02E-02	8,87E-01	1,00E+00	Cd46	0,97	No

ENSMUSG00000016494	1,00E-01	4,07379515	1,33E+00	2,49E-01	5,50E-01	Cd34	1,07	No
ENSMUSG00000016495	-3,73E-02	6,628316466	3,62E-01	5,47E-01	8,15E-01	Plgrkt	0,97	No
ENSMUSG00000016496	-3,49E-01	8,972486452	3,76E+01	8,67E-10	7,16E-08	Cd274	0,78	Yes
ENSMUSG00000016498	-7,59E-02	7,095166275	1,58E+00	2,08E-01	5,50E-01	Pdcd1lg2	0,95	No
ENSMUSG00000016503	1,23E-01	4,345550294	2,22E+00	1,36E-01	4,65E-01	Gtf3a	1,09	No
ENSMUSG00000016510	2,50E-02	4,112839891	8,41E-02	7,72E-01	9,43E-01	Mtif3	1,02	No
ENSMUSG00000016520	-2,80E-01	4,306432786	1,15E+01	7,14E-04	1,23E-02	Lnx2	0,82	Yes
ENSMUSG00000016524	-7,38E-02	-3,033634644	2,66E-03	9,59E-01	1,00E+00	Il19	0,95	No
ENSMUSG00000016526	-4,06E-02	2,919371417	1,24E-01	7,25E-01	9,18E-01	Dyrk3	0,97	No
ENSMUSG00000016528	-6,44E-02	7,735338376	1,20E+00	2,73E-01	5,69E-01	Mapkapk2	0,96	No
ENSMUSG00000016529	-6,07E-02	-2,770591764	2,49E-03	9,60E-01	1,00E+00	Il10	0,96	No
ENSMUSG00000016541	2,45E-05	6,219124128	1,46E-07	1,00E+00	1,00E+00	Atxn10	1,00	No
ENSMUSG00000016552	2,54E-01	5,620788869	1,39E+01	1,97E-04	4,29E-03	Foxred2	1,19	Yes
ENSMUSG00000016554	-1,66E-02	6,419217157	6,89E-02	7,93E-01	9,56E-01	Eif3d	0,99	No
ENSMUSG00000016559	-1,01E-01	9,03394394	3,18E+00	7,46E-02	3,47E-01	H3f3b	0,93	No
ENSMUSG00000016619	3,36E-02	6,03637502	2,66E-01	6,06E-01	8,50E-01	Nup50	1,02	No
ENSMUSG00000016624	1,71E-01	-1,165790208	1,26E-01	7,23E-01	9,17E-01	Phf21b	1,13	No
ENSMUSG00000016637	9,99E-02	2,9439547	7,41E-01	3,89E-01	6,90E-01	Ift27	1,07	No
ENSMUSG00000016664	5,39E-02	7,593682977	8,33E-01	3,61E-01	6,63E-01	Pacsin2	1,04	No
ENSMUSG00000016756	-3,13E-01	0,213977379	1,46E+00	2,27E-01	5,50E-01	Cmah	0,80	No
ENSMUSG00000016757	1,07E-01	4,899024568	2,03E+00	1,54E-01	4,97E-01	Ttll12	1,08	No
ENSMUSG00000016758	-1,40E-01	-1,107697542	9,80E-02	7,54E-01	9,34E-01	Bik	0,91	No
ENSMUSG00000016763	-2,02E+00	1,244732052	1,03E+02	4,02E-24	1,32E-21	Scube1	0,25	Yes
ENSMUSG00000016831	8,09E-03	6,739452737	1,72E-02	8,96E-01	1,00E+00	Tox4	1,01	No
ENSMUSG00000016833	-1,64E-01	1,720202641	1,00E+00	3,17E-01	6,18E-01	Mrps18c	0,89	No
ENSMUSG00000016918	-8,38E-01	-1,54822644	2,54E+00	1,11E-01	4,08E-01	Sulf1	0,56	No
ENSMUSG00000016921	6,17E-02	7,742277353	1,10E+00	2,93E-01	5,89E-01	Srsf6	1,04	No
ENSMUSG00000016933	-1,79E-02	4,757604317	5,45E-02	8,15E-01	9,69E-01	Plcg1	0,99	No
ENSMUSG00000016940	2,47E-02	4,181986837	8,50E-02	7,71E-01	9,42E-01	Kctd2	1,02	No
ENSMUSG00000016942	6,95E-01	-2,311205898	7,01E-01	4,02E-01	7,01E-01	Tmprss6	1,62	No
ENSMUSG00000016946	7,05E-02	4,856298333	8,73E-01	3,50E-01	6,51E-01	Kctd5	1,05	No
ENSMUSG00000016982	-2,70E+00	-2,896043557	2,88E+00	8,95E-02	3,79E-01	Pom121l2	0,15	No

ENSMUSG00000016984	9,53E-02	3,7771204	1,05E+00	3,07E-01	6,04E-01	Etaa1	1,07	No
ENSMUSG00000016995	-1,87E+00	-3,107637761	1,39E+00	2,38E-01	5,50E-01	Matn4	0,27	No
ENSMUSG00000017002	9,96E-02	2,673674526	6,31E-01	4,27E-01	7,21E-01	Slpi	1,07	No
ENSMUSG00000017009	1,56E-01	4,129652484	3,23E+00	7,24E-02	3,41E-01	Sdc4	1,11	No
ENSMUSG00000017119	5,20E-02	6,294669434	6,71E-01	4,13E-01	7,08E-01	Nbr1	1,04	No
ENSMUSG00000017132	5,27E-03	7,266202472	7,77E-03	9,30E-01	1,00E+00	Cyth1	1,00	No
ENSMUSG00000017144	1,25E-01	5,342275019	3,17E+00	7,51E-02	3,49E-01	Rnd3	1,09	No
ENSMUSG00000017146	1,34E-01	5,214494194	3,48E+00	6,22E-02	3,10E-01	Brca1	1,10	No
ENSMUSG00000017165	-1,87E+00	-3,107637761	1,39E+00	2,38E-01	5,50E-01	Gast	0,27	No
ENSMUSG00000017167	4,03E-02	1,664913073	6,01E-02	8,06E-01	9,64E-01	Cntnap1	1,03	No
ENSMUSG00000017176	1,73E-01	3,070139454	2,37E+00	1,24E-01	4,40E-01	Nt5c3b	1,13	No
ENSMUSG00000017188	-3,98E-02	5,077027911	2,95E-01	5,87E-01	8,40E-01	Coa3	0,97	No
ENSMUSG00000017195	2,74E-02	-3,033621357	9,16E-12	1,00E+00	1,00E+00	Zpbp2	1,02	No
ENSMUSG00000017204	1,59E-01	-2,107596949	4,37E-02	8,34E-01	9,79E-01	Gsdma	1,12	No
ENSMUSG00000017210	1,11E-01	5,3802825	2,50E+00	1,14E-01	4,17E-01	Med24	1,08	No
ENSMUSG00000017211	1,85E+00	-3,107657832	1,35E+00	2,46E-01	5,50E-01	Gsdma2	3,61	No
ENSMUSG00000017221	6,66E-02	6,608312754	1,14E+00	2,86E-01	5,82E-01	Psmd3	1,05	No
ENSMUSG00000017264	1,30E-01	5,318376094	3,35E+00	6,71E-02	3,26E-01	Exosc10	1,09	No
ENSMUSG00000017286	-1,60E-01	5,2555518	5,06E+00	2,45E-02	1,72E-01	Glod4	0,90	No
ENSMUSG00000017288	3,93E-02	5,58442358	3,27E-01	5,67E-01	8,29E-01	Vps53	1,03	No
ENSMUSG00000017291	-2,72E-02	7,125796644	2,04E-01	6,51E-01	8,77E-01	Taok1	0,98	No
ENSMUSG00000017299	-9,69E-02	3,046642409	7,58E-01	3,84E-01	6,85E-01	Dnttip1	0,94	No
ENSMUSG00000017300	6,62E-01	-2,378255279	5,62E-01	4,53E-01	7,43E-01	Tnnc2	1,58	No
ENSMUSG00000017307	-5,42E-02	4,388086854	4,41E-01	5,07E-01	7,91E-01	Acot8	0,96	No
ENSMUSG00000017309	3,80E-01	3,143510831	1,19E+01	5,49E-04	9,91E-03	Cd300lg	1,30	Yes
ENSMUSG00000017314	-4,92E-01	1,299489017	7,14E+00	7,54E-03	7,58E-02	Mpp2	0,71	No
ENSMUSG00000017344	-1,94E+00	-3,107619243	1,50E+00	2,21E-01	5,50E-01	Vtn	0,26	No
ENSMUSG00000017376	1,53E-02	4,699941511	3,93E-02	8,43E-01	9,85E-01	Nlk	1,01	No
ENSMUSG00000017386	-2,94E-01	4,519453301	1,29E+01	3,27E-04	6,50E-03	Traf4	0,82	Yes
ENSMUSG00000017390	3,57E-01	6,549620732	3,23E+01	1,32E-08	8,94E-07	Aldoc	1,28	Yes
ENSMUSG00000017400	3,91E-01	6,901974985	4,06E+01	1,88E-10	1,71E-08	Stac2	1,31	Yes
ENSMUSG00000017404	-1,72E-01	5,613396714	6,39E+00	1,15E-02	1,03E-01	Rpl19	0,89	No

ENSMUSG00000017405	-1,39E-01	3,26586192	1,80E+00	1,79E-01	5,40E-01	Nek8		0,91	No
ENSMUSG00000017412	-1,52E-01	1,576257417	8,24E-01	3,64E-01	6,65E-01	Cacnb4		0,90	No
ENSMUSG00000017417	5,02E-01	4,102044412	3,20E+01	1,57E-08	1,04E-06	Plxdc1		1,42	Yes
ENSMUSG00000017418	-5,39E-02	4,678749122	4,72E-01	4,92E-01	7,78E-01	Arl5b		0,96	No
ENSMUSG00000017421	-6,47E-02	7,273223893	1,17E+00	2,80E-01	5,76E-01	Zfp207		0,96	No
ENSMUSG00000017428	2,66E-03	5,088202631	1,33E-03	9,71E-01	1,00E+00	Psmd11		1,00	No
ENSMUSG00000017446	-6,38E-01	-2,401393438	6,23E-01	4,30E-01	7,23E-01	C1qtnf1		0,64	No
ENSMUSG00000017453	-7,38E-02	-3,033634644	2,66E-03	9,59E-01	1,00E+00	Pipox		0,95	No
ENSMUSG00000017466	2,50E-01	6,174837098	1,49E+01	1,13E-04	2,69E-03	Timp2		1,19	Yes
ENSMUSG00000017478	5,64E-02	5,883393249	7,24E-01	3,95E-01	6,95E-01	Zc3h18		1,04	No
ENSMUSG00000017485	6,32E-02	6,946600772	1,08E+00	2,99E-01	5,95E-01	Top2b		1,04	No
ENSMUSG00000017491	1,80E-02	-0,401402941	3,02E-03	9,56E-01	1,00E+00	Rarb		1,01	No
ENSMUSG00000017493	-6,83E-01	3,868399472	5,67E+01	5,09E-14	7,15E-12	Igfbp4		0,62	Yes
ENSMUSG00000017499	4,17E-01	3,926948768	2,11E+01	4,30E-06	1,59E-04	Cdc6		1,34	Yes
ENSMUSG00000017548	-8,99E-02	6,528458164	2,07E+00	1,51E-01	4,92E-01	Suz12		0,94	No
ENSMUSG00000017550	1,81E-01	4,504769529	5,06E+00	2,45E-02	1,72E-01	Atad5		1,13	No
ENSMUSG00000017561	1,36E-01	5,561565823	4,01E+00	4,53E-02	2,54E-01	Crlf3		1,10	No
ENSMUSG00000017607	-5,78E-03	5,270669532	6,63E-03	9,35E-01	1,00E+00	Tns4		1,00	No
ENSMUSG00000017615	1,58E-01	5,745817668	5,49E+00	1,91E-02	1,47E-01	Tnfaip1		1,12	No
ENSMUSG00000017631	1,58E-01	8,335949447	7,46E+00	6,32E-03	6,67E-02	Abr		1,12	No
ENSMUSG00000017639	1,55E-01	3,658090774	2,61E+00	1,06E-01	3,99E-01	Rab11fip4		1,11	No
ENSMUSG00000017652	-6,08E-01	3,7433681	4,34E+01	4,37E-11	4,32E-09	Cd40		0,66	Yes
ENSMUSG00000017664	2,31E-01	6,446506584	1,34E+01	2,46E-04	5,17E-03	Slc35c2		1,17	Yes
ENSMUSG00000017667	-4,04E-01	-0,627498871	1,16E+00	2,82E-01	5,79E-01	Zfp334		0,76	No
ENSMUSG00000017670	2,39E-01	6,908219396	1,53E+01	9,08E-05	2,24E-03	Elmo2		1,18	Yes
ENSMUSG00000017677	1,32E-01	6,828763875	4,63E+00	3,15E-02	2,05E-01	Wsb1		1,10	No
ENSMUSG00000017679	5,93E-02	5,445931213	7,32E-01	3,92E-01	6,93E-01	Ttpal		1,04	No
ENSMUSG00000017686	1,57E-02	5,356053423	4,99E-02	8,23E-01	9,73E-01	Rhot1		1,01	No
ENSMUSG00000017692	-5,47E-01	-1,574169923	8,97E-01	3,44E-01	6,44E-01	Rhbdl3		0,68	No
ENSMUSG00000017697	2,53E-01	1,116349067	1,57E+00	2,10E-01	5,50E-01	Ada		1,19	No
ENSMUSG00000017707	1,48E-02	8,821280888	6,74E-02	7,95E-01	9,57E-01	Serinc3		1,01	No
ENSMUSG00000017713	1,21E-01	2,369653447	8,37E-01	3,60E-01	6,61E-01	Tha1		1,09	No

ENSMUSG00000017715	1,11E-01	6,830771095	3,26E+00	7,12E-02	3,38E-01	Pgs1		1,08	No
ENSMUSG00000017716	1,71E-01	5,635444215	6,35E+00	1,17E-02	1,05E-01	Birc5		1,13	No
ENSMUSG00000017718	-4,87E-01	3,81750648	2,65E+01	2,60E-07	1,32E-05	Afmid		0,71	Yes
ENSMUSG00000017720	-2,27E-02	-1,697399938	1,60E-03	9,68E-01	1,00E+00	Trp53tg5		0,98	No
ENSMUSG00000017721	4,55E-02	6,074103788	4,85E-01	4,86E-01	7,73E-01	Pigt		1,03	No
ENSMUSG00000017724	1,66E-01	0,036325249	2,98E-01	5,85E-01	8,39E-01	Etv4		1,12	No
ENSMUSG00000017734	-1,08E-01	2,722259507	7,91E-01	3,74E-01	6,75E-01	Dbndd2		0,93	No
ENSMUSG00000017737	-5,23E-01	1,976056653	1,18E+01	5,83E-04	1,04E-02	Mmp9		0,70	Yes
ENSMUSG00000017740	-9,06E-01	-1,448702548	3,05E+00	8,08E-02	3,64E-01	Slc12a5		0,53	No
ENSMUSG00000017747	5,64E-02	3,830923175	3,69E-01	5,44E-01	8,12E-01	Ghdc		1,04	No
ENSMUSG00000017754	3,82E-01	5,496510236	2,97E+01	5,12E-08	3,04E-06	Pltp		1,30	Yes
ENSMUSG00000017756	1,98E-01	5,62832693	8,45E+00	3,64E-03	4,42E-02	Slc12a7		1,15	Yes
ENSMUSG00000017760	1,70E-01	8,930888961	8,89E+00	2,87E-03	3,70E-02	Ctsa		1,13	Yes
ENSMUSG00000017764	1,62E-01	3,61292046	2,76E+00	9,65E-02	3,88E-01	Zswim1		1,12	No
ENSMUSG00000017765	-4,40E-02	6,11690323	4,60E-01	4,98E-01	7,83E-01	Slc12a4		0,97	No
ENSMUSG00000017767	3,27E-01	0,666608148	1,88E+00	1,70E-01	5,26E-01	Spata25		1,25	No
ENSMUSG00000017774	-6,66E-02	7,727003036	1,29E+00	2,57E-01	5,50E-01	Myo1c		0,95	No
ENSMUSG00000017776	1,90E-01	6,532878629	9,28E+00	2,32E-03	3,15E-02	Crk		1,14	Yes
ENSMUSG00000017778	-1,57E-01	4,56031724	3,80E+00	5,12E-02	2,75E-01	Cox7c		0,90	No
ENSMUSG00000017781	3,71E-02	8,467000372	4,17E-01	5,19E-01	7,98E-01	Pitpna		1,03	No
ENSMUSG00000017801	4,41E-02	4,757790373	3,30E-01	5,66E-01	8,28E-01	Mlx		1,03	No
ENSMUSG00000017802	9,40E-02	6,645952772	2,30E+00	1,29E-01	4,52E-01	Retreg3		1,07	No
ENSMUSG00000017817	-1,89E+00	-3,1076441	1,42E+00	2,33E-01	5,50E-01	Jph2		0,27	No
ENSMUSG00000017830	-1,20E-01	4,428692688	2,21E+00	1,37E-01	4,67E-01	Dhx58		0,92	No
ENSMUSG00000017831	8,86E-03	5,959316418	1,81E-02	8,93E-01	1,00E+00	Rab5a		1,01	No
ENSMUSG00000017832	4,73E-02	-0,322035108	1,89E-02	8,91E-01	1,00E+00	Hspb9		1,03	No
ENSMUSG00000017837	-7,27E-03	6,041813078	1,25E-02	9,11E-01	1,00E+00	Nkiras2		0,99	No
ENSMUSG00000017843	3,23E-02	7,648763588	3,00E-01	5,84E-01	8,38E-01	Ppp2r5c		1,02	No
ENSMUSG00000017858	8,46E-03	4,743997924	1,21E-02	9,12E-01	1,00E+00	Ift52		1,01	No
ENSMUSG00000017861	1,14E-01	4,18024736	1,77E+00	1,83E-01	5,46E-01	Mybl2		1,08	No
ENSMUSG00000017868	-1,86E+00	-3,033612668	1,39E+00	2,38E-01	5,50E-01	Sgk2		0,28	No
ENSMUSG00000017897	1,86E+00	-2,96321844	1,35E+00	2,46E-01	5,50E-01	Eya2		3,62	No

ENSMUSG00000017929	2,68E-02	7,096334002	1,97E-01	6,57E-01	8,80E-01	B4galt5	1,02	No
ENSMUSG00000017943	5,64E-01	-2,226312201	5,39E-01	4,63E-01	7,52E-01	Gdap1l1	1,48	No
ENSMUSG00000017950	-2,65E+00	-2,963248364	2,80E+00	9,42E-02	3,84E-01	Hnf4a	0,16	No
ENSMUSG00000017969	6,02E-01	2,652656708	2,23E+01	2,30E-06	9,16E-05	Ptgis	1,52	Yes
ENSMUSG00000017978	7,96E-01	-2,770643118	5,49E-01	4,59E-01	7,48E-01	Cadps2	1,74	No
ENSMUSG00000017999	3,43E-02	5,308644358	2,34E-01	6,29E-01	8,62E-01	Ddx27	1,02	No
ENSMUSG00000018001	-5,46E-02	5,876884236	6,82E-01	4,09E-01	7,05E-01	Cyth3	0,96	No
ENSMUSG00000018008	1,83E-01	8,430651698	1,01E+01	1,50E-03	2,24E-02	Cyth4	1,13	Yes
ENSMUSG00000018012	2,36E-01	0,527481419	8,98E-01	3,43E-01	6,44E-01	Rac3	1,18	No
ENSMUSG00000018040	7,63E-02	5,487447203	1,21E+00	2,71E-01	5,68E-01	Rrp7a	1,05	No
ENSMUSG00000018042	2,52E-02	7,082631501	1,74E-01	6,76E-01	8,91E-01	Cyb5r3	1,02	No
ENSMUSG00000018068	1,76E-01	4,680133176	5,15E+00	2,33E-02	1,67E-01	Ints2	1,13	No
ENSMUSG00000018076	4,67E-02	7,750672809	6,31E-01	4,27E-01	7,21E-01	Med13l	1,03	No
ENSMUSG00000018102	-2,36E-01	5,738153478	1,23E+01	4,47E-04	8,38E-03	Hist1h2bc	0,85	Yes
ENSMUSG00000018126	4,67E-01	-2,832028547	1,71E-01	6,79E-01	8,93E-01	Baiap2l2	1,38	No
ENSMUSG00000018143	5,09E-02	5,716014205	5,60E-01	4,54E-01	7,44E-01	Mafk	1,04	No
ENSMUSG00000018160	-2,15E-03	6,025887639	1,09E-03	9,74E-01	1,00E+00	Med1	1,00	No
ENSMUSG00000018166	7,90E-02	2,232441899	3,15E-01	5,75E-01	8,34E-01	Erbb3	1,06	No
ENSMUSG00000018167	5,27E-02	5,183803907	5,34E-01	4,65E-01	7,54E-01	Stard3	1,04	No
ENSMUSG00000018168	-2,16E+00	-2,107565107	6,04E+00	1,40E-02	1,20E-01	Ikzf3	0,22	No
ENSMUSG00000018169	2,23E-03	3,56010901	5,27E-04	9,82E-01	1,00E+00	Mfng	1,00	No
ENSMUSG00000018171	7,77E-02	6,505502415	1,54E+00	2,15E-01	5,50E-01	Vmp1	1,06	No
ENSMUSG00000018189	5,25E-03	4,554186823	4,34E-03	9,47E-01	1,00E+00	Uchl5	1,00	No
ENSMUSG00000018196	3,44E-03	4,311120547	1,70E-03	9,67E-01	1,00E+00	Glrx2	1,00	No
ENSMUSG00000018199	7,14E-02	3,379762951	4,91E-01	4,83E-01	7,71E-01	Trove2	1,05	No
ENSMUSG00000018209	-1,75E-01	8,248414316	9,15E+00	2,49E-03	3,32E-02	Stk4	0,89	Yes
ENSMUSG00000018217	3,24E-02	5,993748367	2,41E-01	6,23E-01	8,58E-01	Pmp22	1,02	No
ENSMUSG00000018238	-5,96E-01	-1,205580473	1,68E+00	1,95E-01	5,50E-01	Gdf9	0,66	No
ENSMUSG00000018239	-3,97E-03	2,859967369	1,15E-03	9,73E-01	1,00E+00	Zcchc10	1,00	No
ENSMUSG00000018263	-1,94E+00	-3,107619243	1,50E+00	2,21E-01	5,50E-01	Tbx5	0,26	No
ENSMUSG00000018286	-3,91E-02	6,516322492	3,89E-01	5,33E-01	8,06E-01	Psmb6	0,97	No
ENSMUSG00000018287	-3,31E-02	4,409867548	1,64E-01	6,85E-01	8,97E-01	Spag7	0,98	No

ENSMUSG00000018293	-3,44E-02	10,04001885	3,74E-01	5,41E-01	8,11E-01	Pfn1	0,98	No
ENSMUSG00000018322	-9,26E-02	5,838807171	1,94E+00	1,64E-01	5,15E-01	Tomm34	0,94	No
ENSMUSG00000018326	6,59E-02	8,157736166	1,30E+00	2,55E-01	5,50E-01	Ywhab	1,05	No
ENSMUSG00000018334	-2,05E-01	5,570892137	8,83E+00	2,96E-03	3,80E-02	Ksr1	0,87	Yes
ENSMUSG00000018339	6,69E-02	5,927023049	1,02E+00	3,13E-01	6,13E-01	Gpx3	1,05	No
ENSMUSG00000018340	6,12E-02	6,673872337	9,72E-01	3,24E-01	6,25E-01	Anxa6	1,04	No
ENSMUSG00000018341	-3,09E-01	5,991496051	2,25E+01	2,08E-06	8,41E-05	Il12rb2	0,81	Yes
ENSMUSG00000018347	-5,51E-02	3,89823813	3,65E-01	5,46E-01	8,14E-01	Zkscan6	0,96	No
ENSMUSG00000018362	2,28E-01	4,882361364	9,04E+00	2,65E-03	3,47E-02	Kpna2	1,17	Yes
ENSMUSG00000018363	4,87E-02	5,012136387	4,35E-01	5,10E-01	7,93E-01	Smurf2	1,03	No
ENSMUSG00000018372	-5,81E-02	3,039156517	2,87E-01	5,92E-01	8,42E-01	Cep95	0,96	No
ENSMUSG00000018377	-8,18E-02	5,231079393	1,31E+00	2,52E-01	5,50E-01	Vezf1	0,94	No
ENSMUSG00000018378	-6,34E-01	2,470248924	2,37E+01	1,15E-06	5,04E-05	Cuedc1	0,64	Yes
ENSMUSG00000018379	5,59E-02	7,722662678	9,07E-01	3,41E-01	6,42E-01	Srsf1	1,04	No
ENSMUSG00000018381	-1,25E-01	5,406610377	3,21E+00	7,30E-02	3,43E-01	Abi3	0,92	No
ENSMUSG00000018387	4,67E-01	-1,333219928	8,51E-01	3,56E-01	6,58E-01	Shroom1	1,38	No
ENSMUSG00000018395	9,98E-02	3,70795074	1,08E+00	2,98E-01	5,94E-01	Kif3a	1,07	No
ENSMUSG00000018398	1,44E-01	6,22782911	5,02E+00	2,51E-02	1,75E-01	39692	1,10	No
ENSMUSG00000018401	-3,02E-01	8,412806087	2,76E+01	1,47E-07	7,88E-06	Mtmr4	0,81	Yes
ENSMUSG00000018405	4,50E-02	3,307982453	1,88E-01	6,64E-01	8,84E-01	Mrm1	1,03	No
ENSMUSG00000018411	-5,62E-02	-2,831969586	2,13E-03	9,63E-01	1,00E+00	Mapt	0,96	No
ENSMUSG00000018412	-7,92E-02	6,563383727	1,61E+00	2,04E-01	5,50E-01	Kansl1	0,95	No
ENSMUSG00000018415	1,61E-02	4,768844338	4,48E-02	8,32E-01	9,78E-01	Gid4	1,01	No
ENSMUSG00000018417	-1,73E-01	4,100661565	3,95E+00	4,67E-02	2,58E-01	Myo1b	0,89	No
ENSMUSG00000018425	2,51E-02	4,42253939	9,44E-02	7,59E-01	9,36E-01	Dhx40	1,02	No
ENSMUSG00000018427	-1,35E-01	4,651527322	3,03E+00	8,16E-02	3,67E-01	Ypel2	0,91	No
ENSMUSG00000018428	1,60E-01	4,124068185	3,44E+00	6,37E-02	3,15E-01	Akap1	1,12	No
ENSMUSG00000018433	1,25E-01	4,510821909	2,41E+00	1,21E-01	4,33E-01	Nol11	1,09	No
ENSMUSG00000018442	-5,17E-02	5,938870204	6,14E-01	4,33E-01	7,27E-01	Derl2	0,96	No
ENSMUSG00000018446	-3,66E-02	6,034674674	3,15E-01	5,75E-01	8,34E-01	C1qbp	0,97	No
ENSMUSG00000018449	-2,28E-01	4,407139619	7,98E+00	4,74E-03	5,37E-02	Rpain	0,85	No
ENSMUSG00000018451	-1,22E+00	-2,268042388	1,43E+00	2,32E-01	5,50E-01	6330403K07Rik	0,43	No

ENSMUSG00000018459	6,41E-01	-0,697456301	2,92E+00	8,74E-02	3,78E-01	Slc13a3	1,56	No
ENSMUSG00000018470	2,11E-02	-1,289528693	1,62E-03	9,68E-01	1,00E+00	Kcnab3	1,01	No
ENSMUSG00000018474	-2,71E-01	5,79902538	1,64E+01	5,07E-05	1,37E-03	Chd3	0,83	Yes
ENSMUSG00000018476	-2,33E-01	5,895699398	1,21E+01	5,07E-04	9,27E-03	Kdm6b	0,85	Yes
ENSMUSG00000018481	8,31E-02	5,837548689	1,55E+00	2,14E-01	5,50E-01	Appbp2	1,06	No
ENSMUSG00000018486	-1,31E+00	-2,896141453	1,07E+00	3,01E-01	5,98E-01	Wnt9b	0,40	No
ENSMUSG00000018500	-2,20E-02	3,889740986	5,77E-02	8,10E-01	9,66E-01	Adora2b	0,98	No
ENSMUSG00000018501	-1,42E-02	7,69376433	5,84E-02	8,09E-01	9,65E-01	Ncor1	0,99	No
ENSMUSG00000018507	1,81E-01	8,157872804	9,72E+00	1,82E-03	2,59E-02	Trpv2	1,13	Yes
ENSMUSG00000018509	3,34E-01	2,430133576	6,39E+00	1,15E-02	1,03E-01	Cenpv	1,26	No
ENSMUSG00000018537	-7,16E-02	2,790302766	3,79E-01	5,38E-01	8,09E-01	Pcgf2	0,95	No
ENSMUSG00000018541	-4,31E-02	4,756713808	3,12E-01	5,76E-01	8,35E-01	Cwc25	0,97	No
ENSMUSG00000018543	-7,54E-02	-2,355540117	6,49E-03	9,36E-01	1,00E+00	1700001P01Rik	0,95	No
ENSMUSG00000018547	1,81E-01	6,464981635	8,34E+00	3,87E-03	4,62E-02	Pip4k2b	1,13	Yes
ENSMUSG00000018548	1,66E-01	5,110980999	5,15E+00	2,32E-02	1,67E-01	Trim37	1,12	No
ENSMUSG00000018554	-4,57E-01	-2,497558768	2,13E-01	6,44E-01	8,72E-01	Ybx2	0,73	No
ENSMUSG00000018559	2,88E-02	6,704849474	2,16E-01	6,42E-01	8,71E-01	Ctdnep1	1,02	No
ENSMUSG00000018565	1,40E-02	5,406544319	4,04E-02	8,41E-01	9,84E-01	Elp5	1,01	No
ENSMUSG00000018566	-1,20E+00	-2,355564997	1,87E+00	1,71E-01	5,27E-01	Slc2a4	0,43	No
ENSMUSG00000018567	-4,38E-02	8,624459861	5,84E-01	4,45E-01	7,36E-01	Gabarap	0,97	No
ENSMUSG00000018569	-1,30E+00	-1,997894275	2,90E+00	8,87E-02	3,79E-01	Cldn7	0,41	No
ENSMUSG00000018570	8,47E-03	-0,711789403	5,48E-04	9,81E-01	1,00E+00	2810408A11Rik	1,01	No
ENSMUSG00000018572	1,15E-01	5,640543812	2,90E+00	8,87E-02	3,79E-01	Phf23	1,08	No
ENSMUSG00000018574	-4,56E-02	4,655151239	3,37E-01	5,61E-01	8,24E-01	Acadvl	0,97	No
ENSMUSG00000018581	3,55E-01	1,443441174	3,71E+00	5,41E-02	2,84E-01	Dnah11	1,28	No
ENSMUSG00000018583	-4,11E-03	7,063101666	4,62E-03	9,46E-01	1,00E+00	G3bp1	1,00	No
ENSMUSG00000018585	-1,67E-01	6,73085826	7,32E+00	6,84E-03	7,05E-02	Atox1	0,89	No
ENSMUSG00000018593	-1,16E+00	-0,586963301	9,12E+00	2,53E-03	3,36E-02	Sparc	0,45	Yes
ENSMUSG00000018599	-7,44E-02	3,981178218	7,08E-01	4,00E-01	6,99E-01	Mief2	0,95	No
ENSMUSG00000018604	-2,67E+00	-2,472887592	4,48E+00	3,43E-02	2,18E-01	Tbx3	0,16	No
ENSMUSG00000018620	4,65E-01	-2,711737645	1,69E-01	6,81E-01	8,94E-01	Mmp20	1,38	No
ENSMUSG00000018623	-1,89E+00	-2,522622711	4,17E+00	4,11E-02	2,40E-01	Mmp7	0,27	No

ENSMUSG00000018648	-1,10E+00	0,010981337	1,47E+01	1,27E-04	2,96E-03	Dusp14	0,47	Yes
ENSMUSG00000018651	1,97E-01	2,492602546	2,34E+00	1,26E-01	4,45E-01	Tada2a	1,15	No
ENSMUSG00000018654	-3,94E-02	7,023228554	4,23E-01	5,15E-01	7,97E-01	Ikzf1	0,97	No
ENSMUSG00000018659	2,82E-02	4,640470586	1,29E-01	7,20E-01	9,15E-01	Pnpo	1,02	No
ENSMUSG00000018661	3,87E-02	5,758414617	3,33E-01	5,64E-01	8,26E-01	Cog1	1,03	No
ENSMUSG00000018666	3,45E-02	5,207570198	2,32E-01	6,30E-01	8,62E-01	Cbx1	1,02	No
ENSMUSG00000018669	-2,29E-02	5,00626346	9,68E-02	7,56E-01	9,34E-01	Cdk5rap3	0,98	No
ENSMUSG00000018672	3,14E-02	1,341396383	2,72E-02	8,69E-01	1,00E+00	Copz2	1,02	No
ENSMUSG00000018677	9,78E-02	6,555801029	2,44E+00	1,18E-01	4,27E-01	Slc25a39	1,07	No
ENSMUSG00000018678	-8,88E-03	4,916434261	1,38E-02	9,06E-01	1,00E+00	Sp2	0,99	No
ENSMUSG00000018697	1,72E-01	3,951206522	3,63E+00	5,66E-02	2,93E-01	Aatf	1,13	No
ENSMUSG00000018698	-1,70E+00	-2,770623743	2,01E+00	1,57E-01	5,02E-01	Lhx1	0,31	No
ENSMUSG00000018707	6,80E-02	9,389282123	1,45E+00	2,29E-01	5,50E-01	Dync1h1	1,05	No
ENSMUSG00000018727	-2,29E-01	-1,015816059	3,23E-01	5,70E-01	8,31E-01	Cpsf4l	0,85	No
ENSMUSG00000018733	4,90E-02	3,027401132	1,92E-01	6,61E-01	8,82E-01	Pex12	1,03	No
ENSMUSG00000018736	-2,87E-02	6,547817613	2,10E-01	6,47E-01	8,74E-01	Ndel1	0,98	No
ENSMUSG00000018740	-1,73E-01	2,785917748	2,09E+00	1,48E-01	4,87E-01	Slc25a35	0,89	No
ENSMUSG00000018750	-2,09E-01	4,429039148	6,67E+00	9,78E-03	9,18E-02	Zbtb4	0,87	No
ENSMUSG00000018752	1,97E-01	5,538916252	8,13E+00	4,35E-03	5,03E-02	Tnfsfm13	1,15	No
ENSMUSG00000018761	-6,48E-02	6,174207983	1,01E+00	3,14E-01	6,14E-01	Mpdu1	0,96	No
ENSMUSG00000018765	2,17E-02	5,803220034	1,06E-01	7,45E-01	9,29E-01	Fxr2	1,02	No
ENSMUSG00000018770	-2,24E-02	7,731633033	1,45E-01	7,03E-01	9,06E-01	Atp5g3	0,98	No
ENSMUSG00000018774	1,69E-01	8,501607637	8,64E+00	3,29E-03	4,11E-02	Cd68	1,12	Yes
ENSMUSG00000018776	2,89E-01	-2,497531182	8,50E-02	7,71E-01	9,42E-01	Slc35g3	1,22	No
ENSMUSG00000018796	-7,35E-02	5,67582888	1,18E+00	2,78E-01	5,74E-01	Acsl1	0,95	No
ENSMUSG00000018800	-4,00E-01	1,644470472	5,98E+00	1,45E-02	1,23E-01	Abca5	0,76	No
ENSMUSG00000018809	-1,09E-01	3,324747823	1,09E+00	2,96E-01	5,92E-01	Smyd4	0,93	No
ENSMUSG00000018819	-1,60E-01	9,207902944	7,99E+00	4,71E-03	5,34E-02	Lsp1	0,89	No
ENSMUSG00000018820	1,09E-01	5,412704886	2,44E+00	1,18E-01	4,27E-01	Zfyve27	1,08	No
ENSMUSG00000018821	-4,28E-01	3,922208546	2,30E+01	1,58E-06	6,66E-05	Avpi1	0,74	Yes
ENSMUSG00000018822	-4,12E+00	-2,548189487	8,62E+00	3,32E-03	4,14E-02	Sfrp5	0,06	Yes
ENSMUSG00000018830	-4,04E-01	-0,980405252	8,56E-01	3,55E-01	6,56E-01	Myh11	0,76	No

ENSMUSG00000018841	-9,80E-02	4,383462072	1,45E+00	2,29E-01	5,50E-01	Rad51d	0,93	No
ENSMUSG00000018844	-5,44E-01	-1,896004924	6,81E-01	4,09E-01	7,06E-01	Fndc8	0,69	No
ENSMUSG00000018845	-1,11E+00	-2,472911154	9,33E-01	3,34E-01	6,35E-01	Unc45b	0,46	No
ENSMUSG00000018846	1,91E-01	6,54600427	9,39E+00	2,19E-03	3,00E-02	Pank3	1,14	Yes
ENSMUSG00000018848	4,95E-02	5,818224827	5,51E-01	4,58E-01	7,47E-01	Rars	1,03	No
ENSMUSG00000018849	1,55E-01	-1,963198979	4,16E-02	8,38E-01	9,82E-01	Wwc1	1,11	No
ENSMUSG00000018858	5,95E-02	3,570181859	3,69E-01	5,43E-01	8,12E-01	Mrpl58	1,04	No
ENSMUSG00000018861	-1,34E-01	2,996614883	1,45E+00	2,29E-01	5,50E-01	Fdxr	0,91	No
ENSMUSG00000018865	-5,24E-02	-2,963220811	1,25E-03	9,72E-01	1,00E+00	Sult4a1	0,96	No
ENSMUSG00000018868	3,14E-02	-3,107617333	-7,69E-10	1,00E+00	1,00E+00	Pnpla5	1,02	No
ENSMUSG00000018882	-2,59E-01	4,965876231	1,22E+01	4,85E-04	8,94E-03	Mrpl45	0,84	Yes
ENSMUSG00000018899	-3,47E-01	4,952194752	2,22E+01	2,43E-06	9,66E-05	Irf1	0,79	Yes
ENSMUSG00000018900	6,54E-02	4,294132137	6,16E-01	4,33E-01	7,26E-01	Slc22a5	1,05	No
ENSMUSG00000018906	8,67E-01	0,228799402	9,79E+00	1,75E-03	2,52E-02	P4ha2	1,82	Yes
ENSMUSG00000018907	4,85E-01	-2,146158244	3,19E-01	5,72E-01	8,32E-01	Alox12e	1,40	No
ENSMUSG00000018909	-4,44E-02	8,748639064	6,04E-01	4,37E-01	7,30E-01	Arrb1	0,97	No
ENSMUSG00000018919	-3,97E-01	0,451993614	2,69E+00	1,01E-01	3,93E-01	Tm4sf5	0,76	No
ENSMUSG00000018920	-1,32E-01	7,859675133	5,13E+00	2,35E-02	1,68E-01	Cxcl16	0,91	No
ENSMUSG00000018921	2,50E-01	5,330510782	1,25E+01	4,06E-04	7,71E-03	Pelp1	1,19	Yes
ENSMUSG00000018923	-3,72E-02	4,562578575	2,23E-01	6,36E-01	8,67E-01	Med11	0,97	No
ENSMUSG00000018924	7,38E-01	4,489271878	7,83E+01	8,99E-19	1,86E-16	Alox15	1,67	Yes
ENSMUSG00000018925	-8,17E-01	2,617927696	4,45E+01	2,54E-11	2,58E-09	Heatr9	0,57	Yes
ENSMUSG00000018927	4,90E-02	12,49308878	7,75E-01	3,79E-01	6,79E-01	Ccl6	1,03	No
ENSMUSG00000018930	-3,19E-02	6,467882932	2,50E-01	6,17E-01	8,55E-01	Ccl4	0,98	No
ENSMUSG00000018931	2,41E-01	5,268570085	1,15E+01	6,80E-04	1,18E-02	Natd1	1,18	Yes
ENSMUSG00000018932	1,06E-01	6,746530181	2,92E+00	8,73E-02	3,78E-01	Map2k3	1,08	No
ENSMUSG00000018965	2,62E-02	8,644896601	2,10E-01	6,47E-01	8,74E-01	Ywhah	1,02	No
ENSMUSG00000018974	6,13E-02	5,732399936	8,27E-01	3,63E-01	6,64E-01	Sart3	1,04	No
ENSMUSG00000018983	5,49E-01	5,820213359	6,69E+01	2,87E-16	4,91E-14	E2f2	1,46	Yes
ENSMUSG00000018986	2,88E-01	3,723955921	9,36E+00	2,22E-03	3,04E-02	Slfn3	1,22	Yes
ENSMUSG00000018995	1,73E-02	2,601919059	1,81E-02	8,93E-01	1,00E+00	Nars2	1,01	No
ENSMUSG00000018999	-5,36E-02	6,01987623	6,82E-01	4,09E-01	7,05E-01	Slc35b4	0,96	No

ENSMUSG00000019027	-6,29E-01	-1,655154634	1,23E+00	2,68E-01	5,64E-01	Dnah1	0,65	No
ENSMUSG00000019039	-1,22E-01	5,093054515	2,83E+00	9,28E-02	3,83E-01	Dalrd3	0,92	No
ENSMUSG00000019054	-7,49E-02	5,643384832	1,22E+00	2,70E-01	5,67E-01	Fis1	0,95	No
ENSMUSG00000019055	2,45E-01	5,993677373	1,37E+01	2,11E-04	4,55E-03	Plod1	1,18	Yes
ENSMUSG00000019066	1,60E-01	6,805194842	6,80E+00	9,13E-03	8,72E-02	Rab3d	1,12	No
ENSMUSG00000019080	1,34E-01	2,723431303	1,23E+00	2,68E-01	5,65E-01	Mfsd3	1,10	No
ENSMUSG00000019082	2,91E-01	4,953681439	1,54E+01	8,84E-05	2,19E-03	Slc25a22	1,22	Yes
ENSMUSG00000019102	3,04E-01	0,251501641	1,36E+00	2,44E-01	5,50E-01	Aldh3a1	1,23	No
ENSMUSG00000019122	-5,16E-02	10,19076642	8,43E-01	3,59E-01	6,60E-01	Ccl9	0,96	No
ENSMUSG00000019132	2,79E-03	8,724553184	2,38E-03	9,61E-01	1,00E+00	BC005537	1,00	No
ENSMUSG00000019139	7,44E-02	6,656188299	1,43E+00	2,31E-01	5,50E-01	Isyna1	1,05	No
ENSMUSG00000019143	1,67E-02	4,719094789	4,72E-02	8,28E-01	9,76E-01	Hars2	1,01	No
ENSMUSG00000019158	8,42E-02	4,937867456	1,26E+00	2,62E-01	5,56E-01	Tmem160	1,06	No
ENSMUSG00000019173	-1,43E-03	8,14539885	6,05E-04	9,80E-01	1,00E+00	Rab5c	1,00	No
ENSMUSG00000019178	-8,21E-01	-2,963266074	3,67E-01	5,45E-01	8,13E-01	Styxl1	0,57	No
ENSMUSG00000019179	2,79E-02	7,233843597	2,17E-01	6,42E-01	8,71E-01	Mdh2	1,02	No
ENSMUSG00000019188	1,82E-01	6,819155689	8,79E+00	3,03E-03	3,85E-02	H13	1,13	Yes
ENSMUSG00000019189	4,58E-02	6,383114285	5,24E-01	4,69E-01	7,57E-01	Rnf145	1,03	No
ENSMUSG00000019194	1,43E-01	4,156929072	2,77E+00	9,61E-02	3,87E-01	Scn1b	1,10	No
ENSMUSG00000019210	8,51E-02	6,964575532	1,95E+00	1,62E-01	5,12E-01	Atp6v1e1	1,06	No
ENSMUSG00000019214	3,40E-01	3,668283216	1,26E+01	3,87E-04	7,45E-03	Chtf18	1,27	Yes
ENSMUSG00000019230	2,45E-01	-1,863738102	1,38E-01	7,10E-01	9,09E-01	Lhx9	1,18	No
ENSMUSG00000019232	-2,71E+00	-3,033620915	2,92E+00	8,75E-02	3,78E-01	Etnppl	0,15	No
ENSMUSG00000019235	8,26E-02	1,101736516	1,73E-01	6,78E-01	8,92E-01	Rps6kl1	1,06	No
ENSMUSG00000019254	7,24E-02	6,820863048	1,39E+00	2,38E-01	5,50E-01	Ppp1r12c	1,05	No
ENSMUSG00000019256	-7,57E-02	7,390753	1,57E+00	2,10E-01	5,50E-01	Ahr	0,95	No
ENSMUSG00000019261	2,42E-01	6,066107612	1,38E+01	2,02E-04	4,39E-03	Map1s	1,18	Yes
ENSMUSG00000019278	7,07E-01	-2,963242697	2,70E-01	6,03E-01	8,48E-01	Dpep1	1,63	No
ENSMUSG00000019295	1,71E-01	4,924702304	5,25E+00	2,19E-02	1,60E-01	Tmem129	1,13	No
ENSMUSG00000019297	1,23E-01	5,791896806	3,39E+00	6,57E-02	3,22E-01	Nop9	1,09	No
ENSMUSG00000019301	-2,23E-01	-1,28961097	2,32E-01	6,30E-01	8,62E-01	Hsd17b1	0,86	No
ENSMUSG00000019302	1,95E-01	8,433217044	1,14E+01	7,18E-04	1,23E-02	Atp6v0a1	1,14	Yes

ENSMUSG00000019303	1,89E-02	2,557149713	2,25E-02	8,81E-01	1,00E+00	Psmc3ip	1,01	No
ENSMUSG00000019312	-1,02E+00	-1,77050039	2,67E+00	1,02E-01	3,94E-01	Grb7	0,49	No
ENSMUSG00000019320	-3,09E-01	0,694364057	1,93E+00	1,65E-01	5,18E-01	Noxo1	0,81	No
ENSMUSG00000019326	-1,84E+00	-2,401297759	3,95E+00	4,68E-02	2,58E-01	Aoc3	0,28	No
ENSMUSG00000019338	-2,12E-02	4,579206373	7,22E-02	7,88E-01	9,53E-01	Zfp687	0,99	No
ENSMUSG00000019362	3,95E-02	5,987236278	3,62E-01	5,48E-01	8,15E-01	D8Ertd738e	1,03	No
ENSMUSG00000019368	-4,75E-02	-2,10760978	4,18E-03	9,48E-01	1,00E+00	Sec14l4	0,97	No
ENSMUSG00000019370	-1,11E-02	7,995629254	3,62E-02	8,49E-01	9,89E-01	Calm3	0,99	No
ENSMUSG00000019373	-1,78E-02	4,764887449	5,34E-02	8,17E-01	9,70E-01	Cops3	0,99	No
ENSMUSG00000019428	-3,01E-02	7,680593303	2,62E-01	6,09E-01	8,51E-01	Fkbp8	0,98	No
ENSMUSG00000019429	-3,88E-03	-3,03362684	4,02E-12	1,00E+00	1,00E+00	Ffar3	1,00	No
ENSMUSG00000019432	-2,61E-02	7,750437592	1,98E-01	6,56E-01	8,80E-01	Ddx39b	0,98	No
ENSMUSG00000019433	7,42E-02	6,507384999	1,40E+00	2,37E-01	5,50E-01	Gipc1	1,05	No
ENSMUSG00000019437	-1,01E-01	3,61241304	1,11E+00	2,92E-01	5,87E-01	Tlcd1	0,93	No
ENSMUSG00000019461	7,32E-02	5,083995661	9,85E-01	3,21E-01	6,21E-01	Plscr3	1,05	No
ENSMUSG00000019464	3,65E-02	6,837308647	3,55E-01	5,51E-01	8,17E-01	Ptger1	1,03	No
ENSMUSG00000019467	-2,71E-01	1,536788976	2,64E+00	1,04E-01	3,97E-01	Arhgef25	0,83	No
ENSMUSG00000019470	-1,22E-01	5,880276741	3,41E+00	6,47E-02	3,19E-01	Xab2	0,92	No
ENSMUSG00000019471	-1,11E-01	7,654201982	3,56E+00	5,92E-02	3,01E-01	Cdc37	0,93	No
ENSMUSG00000019478	1,41E-01	0,551274461	3,10E-01	5,78E-01	8,35E-01	Rab4a	1,10	No
ENSMUSG00000019487	-9,21E-02	5,311416033	1,72E+00	1,90E-01	5,50E-01	Trip10	0,94	No
ENSMUSG00000019489	-1,00E+00	-0,01572308	1,31E+01	2,99E-04	6,05E-03	Cd70	0,50	Yes
ENSMUSG00000019494	2,84E-02	5,274970164	1,58E-01	6,91E-01	9,00E-01	Cops6	1,02	No
ENSMUSG00000019505	1,38E-02	10,00695319	6,00E-02	8,06E-01	9,64E-01	Ubb	1,01	No
ENSMUSG00000019518	2,63E-02	4,926843452	1,25E-01	7,23E-01	9,17E-01	Ap4m1	1,02	No
ENSMUSG00000019528	-4,54E-01	7,992027354	6,08E+01	6,22E-15	9,63E-13	Gyg	0,73	Yes
ENSMUSG00000019539	3,27E-01	1,957231875	4,40E+00	3,59E-02	2,25E-01	Rcn3	1,25	No
ENSMUSG00000019564	-3,06E-02	5,245476317	1,86E-01	6,67E-01	8,86E-01	Arid3a	0,98	No
ENSMUSG00000019577	-3,82E-01	-1,355578555	6,42E-01	4,23E-01	7,18E-01	Pdk4	0,77	No
ENSMUSG00000019578	4,25E-02	5,73447136	4,00E-01	5,27E-01	8,04E-01	Ubxn6	1,03	No
ENSMUSG00000019579	2,97E-02	5,487415307	1,83E-01	6,69E-01	8,87E-01	Mydgf	1,02	No
ENSMUSG00000019590	2,14E-01	4,288691513	6,48E+00	1,09E-02	9,95E-02	Cyb561	1,16	No

ENSMUSG00000019647	3,18E-02	-0,333238422	9,67E-03	9,22E-01	1,00E+00	Sema6a	1,02	No
ENSMUSG00000019659	-6,33E-02	5,595202745	8,62E-01	3,53E-01	6,54E-01	Ccdc12	0,96	No
ENSMUSG00000019689	1,03E-01	2,984850922	8,35E-01	3,61E-01	6,62E-01	Fmc1	1,07	No
ENSMUSG00000019699	-3,90E-02	5,83105757	3,46E-01	5,56E-01	8,21E-01	Akt3	0,97	No
ENSMUSG00000019710	-1,83E-01	5,613544155	7,22E+00	7,19E-03	7,33E-02	Mrpl24	0,88	No
ENSMUSG00000019715	9,11E-03	5,012175615	1,51E-02	9,02E-01	1,00E+00	Gle1	1,01	No
ENSMUSG00000019718	4,54E-01	-0,912587034	9,70E-01	3,25E-01	6,25E-01	L3hypdh	1,37	No
ENSMUSG00000019726	-1,12E-01	6,557425716	3,18E+00	7,46E-02	3,47E-01	Lyst	0,93	No
ENSMUSG00000019731	1,11E-01	7,041162014	3,34E+00	6,77E-02	3,28E-01	Slc35e1	1,08	No
ENSMUSG00000019732	2,84E-01	-1,070096199	3,84E-01	5,35E-01	8,08E-01	Calr3	1,22	No
ENSMUSG00000019734	-9,15E-02	1,643696084	3,05E-01	5,81E-01	8,37E-01	Tmc4	0,94	No
ENSMUSG00000019737	-3,20E-01	0,337803695	1,63E+00	2,02E-01	5,50E-01	Syne4	0,80	No
ENSMUSG00000019738	-2,25E-01	2,120484569	2,32E+00	1,28E-01	4,49E-01	Polr2i	0,86	No
ENSMUSG00000019761	-4,63E-01	-0,1169109	2,26E+00	1,33E-01	4,59E-01	Krt10	0,73	No
ENSMUSG00000019763	1,31E-01	3,631609062	1,88E+00	1,71E-01	5,27E-01	Rmnd1	1,10	No
ENSMUSG00000019767	2,07E-01	-1,205801151	1,65E-01	6,84E-01	8,96E-01	Ccdc170	1,15	No
ENSMUSG00000019768	1,67E-01	4,978170852	5,02E+00	2,50E-02	1,75E-01	Esr1	1,12	No
ENSMUSG00000019772	-1,94E+00	-3,107619243	1,50E+00	2,21E-01	5,50E-01	Vip	0,26	No
ENSMUSG00000019773	1,95E-01	4,335610074	5,47E+00	1,93E-02	1,48E-01	Fbxo5	1,15	No
ENSMUSG00000019774	4,93E-03	3,000407928	1,94E-03	9,65E-01	1,00E+00	Mtrf1l	1,00	No
ENSMUSG00000019777	1,43E-01	5,012405706	3,77E+00	5,21E-02	2,78E-01	Hdac2	1,10	No
ENSMUSG00000019779	-4,60E-01	-2,311169208	3,91E-01	5,32E-01	8,06E-01	Frk	0,73	No
ENSMUSG00000019782	3,82E-02	3,716395182	1,63E-01	6,87E-01	8,97E-01	Rwdd1	1,03	No
ENSMUSG00000019787	1,87E+00	-3,107639691	1,38E+00	2,40E-01	5,50E-01	Trdn	3,67	No
ENSMUSG00000019789	7,56E-01	-2,963268003	3,13E-01	5,76E-01	8,34E-01	Hey2	1,69	No
ENSMUSG00000019790	1,74E-01	5,647244828	6,48E+00	1,09E-02	9,97E-02	Stxbp5	1,13	No
ENSMUSG00000019791	-3,55E-01	3,646626594	1,38E+01	2,06E-04	4,46E-03	Hint3	0,78	Yes
ENSMUSG00000019792	3,40E-02	2,299259558	6,06E-02	8,06E-01	9,63E-01	Trmt11	1,02	No
ENSMUSG00000019794	-1,33E-02	4,285841697	2,56E-02	8,73E-01	1,00E+00	Katna1	0,99	No
ENSMUSG00000019795	5,16E-03	5,849363398	6,03E-03	9,38E-01	1,00E+00	Pcmt1	1,00	No
ENSMUSG00000019796	-8,44E-02	3,089295357	5,87E-01	4,44E-01	7,35E-01	Lrp11	0,94	No
ENSMUSG00000019797	1,82E-01	3,281828012	2,99E+00	8,36E-02	3,71E-01	1700021F05Rik	1,13	No

ENSMUSG00000019802	-2,08E-02	6,485474857	1,09E-01	7,41E-01	9,27E-01	Sec63	0,99	No
ENSMUSG00000019804	1,08E-01	7,894315997	3,41E+00	6,47E-02	3,19E-01	Snx3	1,08	No
ENSMUSG00000019806	-3,59E-01	2,37099857	7,19E+00	7,33E-03	7,42E-02	Aig1	0,78	No
ENSMUSG00000019808	-3,89E-02	2,378385932	8,30E-02	7,73E-01	9,44E-01	Adat2	0,97	No
ENSMUSG00000019809	-8,66E-03	3,517407	7,66E-03	9,30E-01	1,00E+00	Pex3	0,99	No
ENSMUSG00000019810	1,17E-01	7,584401937	3,95E+00	4,68E-02	2,59E-01	Fuca2	1,08	No
ENSMUSG00000019813	-7,03E-02	3,250395305	4,56E-01	5,00E-01	7,85E-01	Cep57l1	0,95	No
ENSMUSG00000019814	1,72E-01	5,003861647	5,35E+00	2,07E-02	1,55E-01	Ltv1	1,13	No
ENSMUSG00000019815	-1,89E+00	-3,1076441	1,42E+00	2,33E-01	5,50E-01	Zc2hc1b	0,27	No
ENSMUSG00000019817	-4,24E-02	1,539435134	6,07E-02	8,05E-01	9,63E-01	Plagl1	0,97	No
ENSMUSG00000019818	-1,55E-01	9,158327335	7,45E+00	6,35E-03	6,69E-02	Cd164	0,90	No
ENSMUSG00000019820	-1,29E-01	5,287901675	3,32E+00	6,86E-02	3,29E-01	Utrn	0,91	No
ENSMUSG00000019822	2,34E-02	5,146867302	1,04E-01	7,48E-01	9,30E-01	Smpd2	1,02	No
ENSMUSG00000019823	6,22E-02	7,54725989	1,10E+00	2,93E-01	5,89E-01	Mical1	1,04	No
ENSMUSG00000019826	6,33E-02	3,938297983	5,00E-01	4,79E-01	7,67E-01	Zbtb24	1,04	No
ENSMUSG00000019831	-5,59E-01	-2,600719053	2,45E-01	6,20E-01	8,57E-01	Wasf1	0,68	No
ENSMUSG00000019832	1,54E-01	6,289186739	5,81E+00	1,60E-02	1,31E-01	Rab32	1,11	No
ENSMUSG00000019836	2,49E-01	2,140061181	2,84E+00	9,21E-02	3,82E-01	Amd-ps4	1,19	No
ENSMUSG00000019837	-8,32E-03	4,352298885	1,02E-02	9,19E-01	1,00E+00	Gtf3c6	0,99	No
ENSMUSG00000019838	-4,50E-02	4,146664612	2,71E-01	6,02E-01	8,48E-01	Slc16a10	0,97	No
ENSMUSG00000019841	5,15E-02	5,727177369	5,85E-01	4,45E-01	7,36E-01	Rev3l	1,04	No
ENSMUSG00000019842	-1,87E-01	2,505706775	2,19E+00	1,39E-01	4,71E-01	Traf3ip2	0,88	No
ENSMUSG00000019843	8,66E-03	6,447713935	1,89E-02	8,91E-01	1,00E+00	Fyn	1,01	No
ENSMUSG00000019845	2,35E-01	2,970695504	4,20E+00	4,05E-02	2,38E-01	Tube1	1,18	No
ENSMUSG00000019846	-8,38E-01	-2,268023365	1,25E+00	2,63E-01	5,57E-01	Lama4	0,56	No
ENSMUSG00000019848	-2,37E-01	-1,863649169	1,24E-01	7,25E-01	9,18E-01	Popdc3	0,85	No
ENSMUSG00000019849	4,64E-02	6,557375871	5,48E-01	4,59E-01	7,48E-01	Prep	1,03	No
ENSMUSG00000019850	-4,44E-01	6,870116267	5,25E+01	4,30E-13	5,48E-11	Tnfaip3	0,73	Yes
ENSMUSG00000019851	1,22E-01	1,715632509	5,63E-01	4,53E-01	7,43E-01	Perp	1,09	No
ENSMUSG00000019852	2,46E-01	4,077331082	7,85E+00	5,09E-03	5,66E-02	Arfgef3	1,19	No
ENSMUSG00000019853	9,26E-02	1,575275582	2,98E-01	5,85E-01	8,38E-01	Hebp2	1,07	No
ENSMUSG00000019854	1,12E-01	5,693267513	2,73E+00	9,85E-02	3,91E-01	Reps1	1,08	No

ENSMUSG00000019856	7,07E-01	-2,655190462	2,74E-01	6,01E-01	8,47E-01	Fam184a	1,63	No
ENSMUSG00000019857	1,42E-02	3,627801828	2,16E-02	8,83E-01	1,00E+00	Asf1a	1,01	No
ENSMUSG00000019861	8,82E-02	5,222008715	1,54E+00	2,15E-01	5,50E-01	Gopc	1,06	No
ENSMUSG00000019863	1,11E-01	3,944555438	1,51E+00	2,19E-01	5,50E-01	Qrsl1	1,08	No
ENSMUSG00000019864	1,20E-01	4,436760395	2,21E+00	1,37E-01	4,68E-01	Rtn4ip1	1,09	No
ENSMUSG00000019865	3,14E-02	-3,107617333	-7,69E-10	1,00E+00	1,00E+00	Nmbr	1,02	No
ENSMUSG00000019866	-1,28E-01	7,232534828	4,61E+00	3,17E-02	2,06E-01	Crybg1	0,91	No
ENSMUSG00000019868	-5,78E-02	4,76788477	5,64E-01	4,53E-01	7,42E-01	Vta1	0,96	No
ENSMUSG00000019872	-1,59E-01	6,515938624	6,47E+00	1,10E-02	9,98E-02	Smpdl3a	0,90	No
ENSMUSG00000019873	-6,60E-02	7,206233657	1,21E+00	2,72E-01	5,68E-01	Reep3	0,96	No
ENSMUSG00000019874	-8,55E-01	0,266805036	9,63E+00	1,91E-03	2,70E-02	Fabp7	0,55	Yes
ENSMUSG00000019876	-6,51E-01	7,048377616	1,16E+02	5,89E-27	2,45E-24	Pkib	0,64	Yes
ENSMUSG00000019877	3,28E-02	8,070152226	3,19E-01	5,72E-01	8,32E-01	Serinc1	1,02	No
ENSMUSG00000019878	-4,79E-01	5,894287393	5,28E+01	3,61E-13	4,69E-11	Hsf2	0,72	Yes
ENSMUSG00000019883	-7,67E-02	4,905364586	1,06E+00	3,04E-01	6,01E-01	Echdc1	0,95	No
ENSMUSG00000019889	-7,88E-02	-2,89616692	4,36E-03	9,47E-01	1,00E+00	Ptprk	0,95	No
ENSMUSG00000019890	-2,70E+00	-2,896126596	2,88E+00	8,95E-02	3,79E-01	Nts	0,15	No
ENSMUSG00000019891	-6,78E-02	-2,185664644	7,44E-03	9,31E-01	1,00E+00	Dcbld1	0,95	No
ENSMUSG00000019894	-1,29E+00	-0,389658206	1,53E+01	8,98E-05	2,22E-03	Slc6a15	0,41	Yes
ENSMUSG00000019897	-5,47E-02	5,407793096	6,10E-01	4,35E-01	7,28E-01	Ccdc59	0,96	No
ENSMUSG00000019899	1,19E+00	-0,676303549	9,24E+00	2,36E-03	3,20E-02	Lama2	2,28	Yes
ENSMUSG00000019907	-2,13E-01	7,096071555	1,24E+01	4,25E-04	8,01E-03	Ppp1r12a	0,86	Yes
ENSMUSG00000019909	-1,94E+00	-3,107619243	1,50E+00	2,21E-01	5,50E-01	Fam162b	0,26	No
ENSMUSG00000019916	3,07E-01	6,240072617	2,28E+01	1,80E-06	7,42E-05	P4ha1	1,24	Yes
ENSMUSG00000019917	1,42E-01	3,477245945	2,08E+00	1,49E-01	4,90E-01	40422	1,10	No
ENSMUSG00000019920	1,05E-01	7,552116293	3,18E+00	7,46E-02	3,47E-01	Lims1	1,08	No
ENSMUSG00000019923	-1,11E-02	5,662193785	2,68E-02	8,70E-01	1,00E+00	Zwint	0,99	No
ENSMUSG00000019927	1,38E-01	4,345371189	2,78E+00	9,52E-02	3,86E-01	Ube2d1	1,10	No
ENSMUSG00000019929	-2,62E+00	-1,069994506	2,99E+01	4,61E-08	2,78E-06	Dcn	0,16	Yes
ENSMUSG00000019933	-8,98E-01	-2,033542381	1,72E+00	1,90E-01	5,50E-01	Mrln	0,54	No
ENSMUSG00000019935	2,35E-01	1,334000775	1,66E+00	1,98E-01	5,50E-01	Slc17a8	1,18	No
ENSMUSG00000019942	1,79E-01	6,014778463	7,41E+00	6,47E-03	6,78E-02	Cdk1	1,13	No

ENSMUSG00000019943	2,25E-01	7,988910756	1,48E+01	1,21E-04	2,85E-03	Atp2b1	1,17	Yes
ENSMUSG00000019944	4,33E-01	2,740934696	1,32E+01	2,73E-04	5,63E-03	Rhobtb1	1,35	Yes
ENSMUSG00000019945	6,44E-01	-2,6276819	3,43E-01	5,58E-01	8,22E-01	1700040L02Rik	1,56	No
ENSMUSG00000019947	2,59E-02	5,556197162	1,41E-01	7,08E-01	9,08E-01	Arid5b	1,02	No
ENSMUSG00000019948	2,13E-01	2,470464095	2,70E+00	1,00E-01	3,92E-01	Actr6	1,16	No
ENSMUSG00000019951	4,44E-02	5,792708498	4,37E-01	5,09E-01	7,92E-01	Uhrf1bp1l	1,03	No
ENSMUSG00000019952	3,12E-02	4,024584795	1,24E-01	7,25E-01	9,18E-01	Poc1b	1,02	No
ENSMUSG00000019960	2,50E-01	5,711849067	1,35E+01	2,43E-04	5,11E-03	Dusp6	1,19	Yes
ENSMUSG00000019961	1,70E-01	7,351760124	8,08E+00	4,47E-03	5,13E-02	Tmpo	1,12	No
ENSMUSG00000019966	-5,94E-01	1,435767157	1,13E+01	7,66E-04	1,30E-02	Kitl	0,66	Yes
ENSMUSG00000019969	1,30E-01	6,825075617	4,51E+00	3,38E-02	2,16E-01	Psen1	1,09	No
ENSMUSG00000019970	7,56E-02	7,605267789	1,63E+00	2,01E-01	5,50E-01	Sgk1	1,05	No
ENSMUSG00000019971	1,51E-01	3,689503561	2,48E+00	1,15E-01	4,20E-01	Cep290	1,11	No
ENSMUSG00000019975	1,50E-01	2,755206854	1,56E+00	2,12E-01	5,50E-01	Ikbip	1,11	No
ENSMUSG00000019977	-2,34E-02	5,18635892	1,06E-01	7,44E-01	9,29E-01	Hbs1l	0,98	No
ENSMUSG00000019978	2,79E-02	7,051795189	2,12E-01	6,45E-01	8,73E-01	Epb41l2	1,02	No
ENSMUSG00000019979	5,64E-02	6,588414955	8,20E-01	3,65E-01	6,66E-01	Apaf1	1,04	No
ENSMUSG00000019982	1,48E-01	1,316484642	6,30E-01	4,28E-01	7,21E-01	Myb	1,11	No
ENSMUSG00000019984	4,06E-02	5,088907382	3,07E-01	5,80E-01	8,36E-01	Med23	1,03	No
ENSMUSG00000019986	6,16E-02	2,605242354	2,30E-01	6,31E-01	8,63E-01	Ahi1	1,04	No
ENSMUSG00000019987	7,77E-01	1,807101393	2,27E+01	1,89E-06	7,74E-05	Arg1	1,71	Yes
ENSMUSG00000019988	1,64E-01	4,506759881	4,20E+00	4,04E-02	2,38E-01	Nedd1	1,12	No
ENSMUSG00000019989	-4,83E-02	-2,770561466	1,58E-03	9,68E-01	1,00E+00	Enpp3	0,97	No
ENSMUSG00000019990	-1,40E-01	3,333424511	1,89E+00	1,70E-01	5,25E-01	Pde7b	0,91	No
ENSMUSG00000019992	1,12E-01	4,219008817	1,70E+00	1,92E-01	5,50E-01	Mtfr2	1,08	No
ENSMUSG00000019996	-1,32E-01	1,336259452	5,04E-01	4,78E-01	7,65E-01	Map7	0,91	No
ENSMUSG00000019997	-1,12E+00	-1,236377801	5,42E+00	1,99E-02	1,51E-01	Ctgf	0,46	No
ENSMUSG00000019998	-5,06E-02	7,289944084	7,16E-01	3,98E-01	6,98E-01	Stx7	0,97	No
ENSMUSG00000020003	-3,24E-02	3,480942372	1,05E-01	7,46E-01	9,30E-01	Pex7	0,98	No
ENSMUSG00000020007	-1,89E+00	-3,033648118	1,42E+00	2,33E-01	5,50E-01	Il20ra	0,27	No
ENSMUSG00000020009	3,64E-02	7,073772723	3,61E-01	5,48E-01	8,15E-01	Ifngr1	1,03	No
ENSMUSG00000020010	1,83E-01	-0,015769736	3,81E-01	5,37E-01	8,09E-01	Vnn3	1,14	No

ENSMUSG00000020014	1,31E-01	-1,401199253	5,62E-02	8,13E-01	9,67E-01	Cfap54	1,10	No
ENSMUSG00000020015	-8,46E-02	5,225058626	1,40E+00	2,37E-01	5,50E-01	Cdk17	0,94	No
ENSMUSG00000020017	2,09E-01	2,363999287	2,41E+00	1,21E-01	4,33E-01	Hal	1,16	No
ENSMUSG00000020018	-2,50E-02	2,380810254	3,47E-02	8,52E-01	9,91E-01	Snrpf	0,98	No
ENSMUSG00000020019	-6,43E-02	1,402279769	1,29E-01	7,20E-01	9,15E-01	Ntn4	0,96	No
ENSMUSG00000020020	-7,36E-02	-1,740943076	8,75E-03	9,25E-01	1,00E+00	Usp44	0,95	No
ENSMUSG00000020021	2,15E-01	5,843145935	1,04E+01	1,24E-03	1,93E-02	Fgd6	1,16	Yes
ENSMUSG00000020022	-1,08E-01	3,402827568	1,12E+00	2,89E-01	5,85E-01	Ndufa12	0,93	No
ENSMUSG00000020023	-3,06E-01	5,674115717	2,06E+01	5,52E-06	1,98E-04	Tmcc3	0,81	Yes
ENSMUSG00000020024	-4,48E-02	5,207890169	3,85E-01	5,35E-01	8,07E-01	Cep83	0,97	No
ENSMUSG00000020027	-2,84E-01	8,266713393	2,41E+01	9,06E-07	4,09E-05	Socs2	0,82	Yes
ENSMUSG00000020029	1,06E-03	6,345185999	2,79E-04	9,87E-01	1,00E+00	Nudt4	1,00	No
ENSMUSG00000020032	7,51E-02	4,252003839	8,10E-01	3,68E-01	6,69E-01	Nuak1	1,05	No
ENSMUSG00000020033	-1,98E-02	-3,033655952	2,83E-11	1,00E+00	1,00E+00	4930463O16Rik	0,99	No
ENSMUSG00000020034	4,00E-02	3,703506079	1,76E-01	6,75E-01	8,91E-01	Tcp11l2	1,03	No
ENSMUSG00000020037	7,45E-01	-2,832009043	3,02E-01	5,83E-01	8,38E-01	Rfx4	1,68	No
ENSMUSG00000020038	1,91E-01	3,816327866	4,23E+00	3,98E-02	2,37E-01	Cry1	1,14	No
ENSMUSG00000020042	5,72E-01	-0,425017227	2,74E+00	9,77E-02	3,90E-01	Btbd11	1,49	No
ENSMUSG00000020044	-1,49E+00	1,693678425	8,46E+01	3,62E-20	8,35E-18	Timp3	0,36	Yes
ENSMUSG00000020048	8,40E-02	9,240032326	2,19E+00	1,38E-01	4,70E-01	Hsp90b1	1,06	No
ENSMUSG00000020051	-7,38E-02	-3,033634644	2,66E-03	9,59E-01	1,00E+00	Pah	0,95	No
ENSMUSG00000020053	1,15E-01	5,736680528	2,89E+00	8,89E-02	3,79E-01	Igf1	1,08	No
ENSMUSG00000020056	1,16E-02	4,090509604	1,79E-02	8,94E-01	1,00E+00	Washc3	1,01	No
ENSMUSG00000020057	3,82E-02	5,879319489	3,30E-01	5,66E-01	8,28E-01	Dram1	1,03	No
ENSMUSG00000020059	2,12E-01	-0,211029151	4,40E-01	5,07E-01	7,91E-01	Sycp3	1,16	No
ENSMUSG00000020061	-7,69E-02	-2,963220349	2,69E-03	9,59E-01	1,00E+00	Mybpc1	0,95	No
ENSMUSG00000020062	-3,73E-01	-2,49761691	1,76E-01	6,75E-01	8,91E-01	Slc5a8	0,77	No
ENSMUSG00000020063	2,68E-02	4,877522978	1,28E-01	7,21E-01	9,16E-01	Sirt1	1,02	No
ENSMUSG00000020064	1,25E-02	5,797619839	3,52E-02	8,51E-01	9,90E-01	Herc4	1,01	No
ENSMUSG00000020069	-3,12E-01	4,668090522	1,62E+01	5,76E-05	1,53E-03	Hnrnph3	0,81	Yes
ENSMUSG00000020070	-7,16E-03	4,473984897	7,96E-03	9,29E-01	1,00E+00	Rufy2	1,00	No
ENSMUSG00000020072	-1,60E-01	-0,740946398	1,68E-01	6,82E-01	8,94E-01	Pbld2	0,90	No

ENSMUSG00000020074	-1,25E-02	6,843623519	4,19E-02	8,38E-01	9,82E-01	Ccar1	0,99	No
ENSMUSG00000020075	5,46E-02	7,211964411	8,26E-01	3,63E-01	6,64E-01	Ddx21	1,04	No
ENSMUSG00000020076	-1,23E-01	5,566693872	3,23E+00	7,24E-02	3,41E-01	Ddx50	0,92	No
ENSMUSG00000020077	-3,73E-02	7,916302747	4,06E-01	5,24E-01	8,01E-01	Srgn	0,97	No
ENSMUSG00000020078	1,30E-01	7,086114533	4,60E+00	3,19E-02	2,07E-01	Vps26a	1,09	No
ENSMUSG00000020079	-9,99E-02	4,305909894	1,43E+00	2,31E-01	5,50E-01	Supv3l1	0,93	No
ENSMUSG00000020080	-3,06E-02	-2,600629127	7,06E-04	9,79E-01	1,00E+00	Hkdc1	0,98	No
ENSMUSG00000020081	-1,87E+00	-3,107637761	1,39E+00	2,38E-01	5,50E-01	Tacr2	0,27	No
ENSMUSG00000020083	3,98E-01	-2,107590952	2,54E-01	6,15E-01	8,53E-01	2010107G23Rik	1,32	No
ENSMUSG00000020085	-1,27E-01	4,650728973	2,61E+00	1,06E-01	3,99E-01	Aifm2	0,92	No
ENSMUSG00000020086	-4,34E-02	-0,024656269	2,02E-02	8,87E-01	1,00E+00	H2afy2	0,97	No
ENSMUSG00000020087	2,05E-01	4,847512033	7,25E+00	7,09E-03	7,25E-02	Tysnd1	1,15	No
ENSMUSG00000020088	-3,48E-02	6,83033029	3,22E-01	5,70E-01	8,31E-01	Sar1a	0,98	No
ENSMUSG00000020089	-1,16E-01	4,047897734	1,72E+00	1,90E-01	5,50E-01	Ppa1	0,92	No
ENSMUSG00000020090	-2,33E-01	0,079213716	7,08E-01	4,00E-01	6,99E-01	Npffr1	0,85	No
ENSMUSG00000020091	9,00E-02	7,969105853	2,39E+00	1,22E-01	4,37E-01	Eif4ebp2	1,06	No
ENSMUSG00000020092	3,77E-01	7,080337175	3,85E+01	5,56E-10	4,69E-08	Pald1	1,30	Yes
ENSMUSG00000020096	-8,50E-01	-2,896168286	3,96E-01	5,29E-01	8,05E-01	Tbata	0,55	No
ENSMUSG00000020097	6,72E-02	8,54258961	1,37E+00	2,42E-01	5,50E-01	Sgpl1	1,05	No
ENSMUSG00000020098	-9,93E-01	-0,300139397	8,19E+00	4,21E-03	4,91E-02	Pcbd1	0,50	Yes
ENSMUSG00000020099	-2,13E+00	0,558130657	7,03E+01	5,03E-17	9,25E-15	Unc5b	0,23	Yes
ENSMUSG00000020100	3,23E-01	7,81207585	3,04E+01	3,56E-08	2,18E-06	Slc29a3	1,25	Yes
ENSMUSG00000020101	-5,39E-02	7,638534168	8,37E-01	3,60E-01	6,62E-01	Vsir	0,96	No
ENSMUSG00000020102	3,53E-01	3,460100135	1,18E+01	5,79E-04	1,04E-02	Slc16a7	1,28	Yes
ENSMUSG00000020105	2,31E-01	0,337942099	7,56E-01	3,85E-01	6,86E-01	Lrig3	1,17	No
ENSMUSG00000020107	-1,20E-02	4,981575724	2,61E-02	8,72E-01	1,00E+00	Anapc16	0,99	No
ENSMUSG00000020108	-3,93E-01	2,600214499	1,03E+01	1,32E-03	2,02E-02	Ddit4	0,76	Yes
ENSMUSG00000020109	3,88E-02	5,508984761	3,15E-01	5,75E-01	8,34E-01	Dnajb12	1,03	No
ENSMUSG00000020111	3,80E-03	5,461620241	2,99E-03	9,56E-01	1,00E+00	Micu1	1,00	No
ENSMUSG00000020114	-9,33E-02	6,716518784	2,29E+00	1,30E-01	4,53E-01	Cand1	0,94	No
ENSMUSG00000020115	-3,63E-02	6,294088138	3,24E-01	5,69E-01	8,30E-01	Tbk1	0,98	No
ENSMUSG00000020116	7,35E-02	5,091018355	1,01E+00	3,15E-01	6,16E-01	Pno1	1,05	No

ENSMUSG00000020120	-6,53E-03	9,419629986	1,33E-02	9,08E-01	1,00E+00	Plek	1,00	No
ENSMUSG00000020121	-7,16E-01	-0,175354611	5,04E+00	2,48E-02	1,74E-01	Srgap1	0,61	No
ENSMUSG00000020122	2,59E-01	2,913225695	4,91E+00	2,67E-02	1,83E-01	Egfr	1,20	No
ENSMUSG00000020123	1,87E+00	-3,107639691	1,38E+00	2,40E-01	5,50E-01	Avpr1a	3,67	No
ENSMUSG00000020124	4,59E-02	6,475008766	5,32E-01	4,66E-01	7,54E-01	Usp15	1,03	No
ENSMUSG00000020125	-6,04E-01	-2,548240663	4,97E-01	4,81E-01	7,69E-01	Elane	0,66	No
ENSMUSG00000020128	3,38E-02	5,809540224	2,57E-01	6,12E-01	8,52E-01	Vps54	1,02	No
ENSMUSG00000020130	-2,87E-01	6,426884068	2,09E+01	4,82E-06	1,75E-04	Tbc1d15	0,82	Yes
ENSMUSG00000020131	-2,39E-01	-0,69726892	4,38E-01	5,08E-01	7,92E-01	Pcsk4	0,85	No
ENSMUSG00000020132	-1,44E-01	6,617997018	5,39E+00	2,02E-02	1,52E-01	Rab21	0,91	No
ENSMUSG00000020133	1,43E-02	5,767066659	4,56E-02	8,31E-01	9,78E-01	2310011J03Rik	1,01	No
ENSMUSG00000020134	-1,76E-01	5,222463688	6,11E+00	1,34E-02	1,16E-01	Peli1	0,89	No
ENSMUSG00000020135	-7,22E-01	0,362604689	7,61E+00	5,79E-03	6,24E-02	Apc2	0,61	No
ENSMUSG00000020137	2,87E-01	3,009529841	6,83E+00	8,96E-03	8,60E-02	Thap2	1,22	No
ENSMUSG00000020140	-1,04E-02	-2,963271219	-1,18E-10	1,00E+00	1,00E+00	Lgr5	0,99	No
ENSMUSG00000020142	-3,74E-01	4,102817	1,93E+01	1,09E-05	3,64E-04	Slc1a4	0,77	Yes
ENSMUSG00000020143	1,77E-02	8,247863095	9,42E-02	7,59E-01	9,36E-01	Dock2	1,01	No
ENSMUSG00000020149	8,84E-02	7,879426709	2,29E+00	1,31E-01	4,54E-01	Rab1a	1,06	No
ENSMUSG00000020150	3,41E-01	3,01411194	8,71E+00	3,16E-03	3,99E-02	Gamt	1,27	Yes
ENSMUSG00000020151	1,22E-02	-1,236733964	5,71E-04	9,81E-01	1,00E+00	Ptprr	1,01	No
ENSMUSG00000020152	-1,49E-02	9,290112735	6,89E-02	7,93E-01	9,56E-01	Actr2	0,99	No
ENSMUSG00000020153	1,73E-01	4,916306855	5,28E+00	2,15E-02	1,58E-01	Ndufs7	1,13	No
ENSMUSG00000020154	1,84E+00	-3,033632273	1,33E+00	2,49E-01	5,50E-01	Ptprb	3,58	No
ENSMUSG00000020155	-8,52E-01	-2,896155156	3,98E-01	5,28E-01	8,04E-01	Kcnmb1	0,55	No
ENSMUSG00000020156	2,05E-01	5,141222132	8,05E+00	4,55E-03	5,20E-02	Mum1	1,15	No
ENSMUSG00000020160	2,28E-03	2,241709996	2,85E-04	9,87E-01	1,00E+00	Meis1	1,00	No
ENSMUSG00000020163	6,87E-02	3,543506677	4,85E-01	4,86E-01	7,73E-01	Uqcr11	1,05	No
ENSMUSG00000020164	-1,91E+00	-3,107631671	1,46E+00	2,27E-01	5,50E-01	Kcnmb4os1	0,27	No
ENSMUSG00000020166	-6,40E-02	5,486439148	8,57E-01	3,55E-01	6,56E-01	Cnot2	0,96	No
ENSMUSG00000020167	1,06E-02	5,742964929	2,51E-02	8,74E-01	1,00E+00	Tcf3	1,01	No
ENSMUSG00000020169	-2,71E+00	-2,963235336	2,92E+00	8,75E-02	3,78E-01	Best3	0,15	No
ENSMUSG00000020170	-4,25E-02	5,311276163	3,58E-01	5,50E-01	8,17E-01	Frs2	0,97	No

ENSMUSG00000020171	1,06E-01	5,118849365	2,11E+00	1,47E-01	4,85E-01	Yeats4	1,08	No
ENSMUSG00000020173	7,18E-01	-2,96326129	2,80E-01	5,97E-01	8,45E-01	Cobl	1,65	No
ENSMUSG00000020175	4,22E-01	0,783838392	3,38E+00	6,60E-02	3,23E-01	Rab36	1,34	No
ENSMUSG00000020176	-1,66E+00	1,063148634	6,61E+01	4,26E-16	7,21E-14	Grb10	0,32	Yes
ENSMUSG00000020178	-7,14E-01	4,336717284	7,62E+01	2,55E-18	5,09E-16	Adora2a	0,61	Yes
ENSMUSG00000020180	-8,20E-02	5,421032941	1,37E+00	2,41E-01	5,50E-01	Snrpd3	0,94	No
ENSMUSG00000020181	9,01E-03	-3,107646452	-5,16E-10	1,00E+00	1,00E+00	Nav3	1,01	No
ENSMUSG00000020182	1,80E+00	-2,770661771	1,30E+00	2,54E-01	5,50E-01	Ddc	3,49	No
ENSMUSG00000020183	-1,71E-01	-0,033482524	3,71E-01	5,43E-01	8,12E-01	Cpm	0,89	No
ENSMUSG00000020184	-1,24E-01	7,551336337	4,37E+00	3,67E-02	2,28E-01	Mdm2	0,92	No
ENSMUSG00000020185	4,63E-01	3,959377438	2,62E+01	3,12E-07	1,55E-05	E2f7	1,38	Yes
ENSMUSG00000020186	1,24E-02	2,198837344	7,72E-03	9,30E-01	1,00E+00	Csrp2	1,01	No
ENSMUSG00000020189	1,42E-02	8,292006151	6,05E-02	8,06E-01	9,63E-01	Osbpl8	1,01	No
ENSMUSG00000020190	-5,65E-02	7,922949014	9,39E-01	3,33E-01	6,33E-01	Mknk2	0,96	No
ENSMUSG00000020191	-7,67E-01	-1,413028159	2,08E+00	1,49E-01	4,90E-01	4930415F15Rik	0,59	No
ENSMUSG00000020193	2,89E-01	-0,141293058	8,71E-01	3,51E-01	6,52E-01	Zpbp	1,22	No
ENSMUSG00000020196	1,09E-01	6,959891707	3,23E+00	7,23E-02	3,41E-01	Cabin1	1,08	No
ENSMUSG00000020198	9,47E-02	7,651991642	2,59E+00	1,08E-01	4,02E-01	Ap3d1	1,07	No
ENSMUSG00000020205	1,17E-01	4,06085531	1,72E+00	1,90E-01	5,50E-01	Phlda1	1,08	No
ENSMUSG00000020211	8,14E-02	5,515364255	1,38E+00	2,39E-01	5,50E-01	Sf3a2	1,06	No
ENSMUSG00000020212	-1,22E-01	2,917714116	1,14E+00	2,87E-01	5,83E-01	Mdm1	0,92	No
ENSMUSG00000020216	6,76E-01	-2,185738294	6,62E-01	4,16E-01	7,11E-01	Jsrp1	1,60	No
ENSMUSG00000020218	-1,94E+00	-3,107619243	1,50E+00	2,21E-01	5,50E-01	Wif1	0,26	No
ENSMUSG00000020219	1,80E-02	5,608585244	6,93E-02	7,92E-01	9,55E-01	Timm13	1,01	No
ENSMUSG00000020220	1,45E-01	7,149446411	5,81E+00	1,59E-02	1,31E-01	Vps13d	1,11	No
ENSMUSG00000020224	-6,89E-02	4,673734746	7,86E-01	3,75E-01	6,76E-01	Llph	0,95	No
ENSMUSG00000020225	-9,91E-02	5,455714534	2,03E+00	1,54E-01	4,98E-01	Tmbim4	0,93	No
ENSMUSG00000020226	-3,83E-02	-3,107651936	-7,91E-11	1,00E+00	1,00E+00	Slc5a4b	0,97	No
ENSMUSG00000020227	9,68E-03	4,848052035	1,62E-02	8,99E-01	1,00E+00	Irak3	1,01	No
ENSMUSG00000020228	1,55E-01	5,038751102	4,43E+00	3,53E-02	2,22E-01	Helb	1,11	No
ENSMUSG00000020229	-3,83E-02	-3,107651936	-7,91E-11	1,00E+00	1,00E+00	Slc5a4a	0,97	No
ENSMUSG00000020230	-7,83E-02	4,25618224	8,75E-01	3,50E-01	6,51E-01	Prmt2	0,95	No

ENSMUSG00000020231	1,41E-01	5,656204651	4,29E+00	3,83E-02	2,32E-01	Dip2a	1,10	No
ENSMUSG00000020232	-3,82E-02	6,079989212	3,48E-01	5,55E-01	8,20E-01	Hmg20b	0,97	No
ENSMUSG00000020234	2,87E-01	5,937029023	1,90E+01	1,30E-05	4,23E-04	4930404N11Rik	1,22	Yes
ENSMUSG00000020235	3,85E-02	5,315123091	2,97E-01	5,86E-01	8,39E-01	Fzr1	1,03	No
ENSMUSG00000020238	1,54E-01	6,895353821	6,31E+00	1,20E-02	1,07E-01	Ncln	1,11	No
ENSMUSG00000020241	-1,68E+00	-1,355218452	8,87E+00	2,90E-03	3,73E-02	Col6a2	0,31	Yes
ENSMUSG00000020246	-1,44E-03	3,388070206	1,95E-04	9,89E-01	1,00E+00	Hcfc2	1,00	No
ENSMUSG00000020248	3,70E-02	4,355187235	2,01E-01	6,54E-01	8,79E-01	Nfyb	1,03	No
ENSMUSG00000020250	-2,26E-02	8,154236629	1,51E-01	6,97E-01	9,03E-01	Txnrd1	0,98	No
ENSMUSG00000020251	-4,11E-01	-1,683131862	4,42E-01	5,06E-01	7,91E-01	Glt8d2	0,75	No
ENSMUSG00000020253	6,55E-02	6,496959408	1,09E+00	2,96E-01	5,92E-01	Ppm1m	1,05	No
ENSMUSG00000020255	-1,00E-02	5,891394671	2,27E-02	8,80E-01	1,00E+00	D10Wsu102e	0,99	No
ENSMUSG00000020256	-5,69E-01	-1,740826674	8,01E-01	3,71E-01	6,72E-01	Aldh1l2	0,67	No
ENSMUSG00000020257	1,91E-02	6,645732944	9,53E-02	7,57E-01	9,35E-01	Wdr82	1,01	No
ENSMUSG00000020258	-5,62E-02	0,635797405	6,05E-02	8,06E-01	9,63E-01	Glyctk	0,96	No
ENSMUSG00000020260	1,51E-01	5,360233206	4,56E+00	3,28E-02	2,12E-01	Pofut2	1,11	No
ENSMUSG00000020261	3,34E-01	7,018632622	3,01E+01	4,09E-08	2,48E-06	Slc36a1	1,26	Yes
ENSMUSG00000020262	4,08E-01	2,618452558	1,01E+01	1,51E-03	2,24E-02	Adarb1	1,33	Yes
ENSMUSG00000020263	1,56E-01	5,954733254	5,63E+00	1,76E-02	1,39E-01	Appl2	1,11	No
ENSMUSG00000020264	8,67E-02	3,335915553	6,82E-01	4,09E-01	7,05E-01	Slc36a2	1,06	No
ENSMUSG00000020265	-3,92E-02	6,300893813	3,80E-01	5,38E-01	8,09E-01	Sumo3	0,97	No
ENSMUSG00000020267	4,17E-02	6,628433858	4,49E-01	5,03E-01	7,88E-01	Hint1	1,03	No
ENSMUSG00000020268	3,14E-01	0,128212509	1,19E+00	2,75E-01	5,71E-01	Lyrm7	1,24	No
ENSMUSG00000020271	-8,92E-02	6,701133099	2,08E+00	1,50E-01	4,90E-01	Fbxw11	0,94	No
ENSMUSG00000020272	3,26E-01	6,842862976	2,81E+01	1,17E-07	6,42E-06	Stk10	1,25	Yes
ENSMUSG00000020273	-2,26E-01	4,543191374	8,54E+00	3,48E-03	4,27E-02	Papolg	0,85	Yes
ENSMUSG00000020275	-6,08E-01	8,61594014	1,12E+02	3,88E-26	1,47E-23	Rel	0,66	Yes
ENSMUSG00000020277	1,01E-01	6,1641922	2,47E+00	1,16E-01	4,21E-01	Pfkl	1,07	No
ENSMUSG00000020279	-5,31E-01	0,010741848	3,28E+00	7,01E-02	3,34E-01	Il9r	0,69	No
ENSMUSG00000020280	-2,45E-01	4,574328254	9,73E+00	1,81E-03	2,58E-02	Pus10	0,84	Yes
ENSMUSG00000020282	-3,59E-01	0,483723855	2,05E+00	1,52E-01	4,95E-01	Rhbdf1	0,78	No
ENSMUSG00000020283	-1,40E-01	4,643932443	3,22E+00	7,28E-02	3,42E-01	Pex13	0,91	No

ENSMUSG00000020284	1,39E-01	3,422371951	1,90E+00	1,68E-01	5,23E-01	1810043G02Rik	1,10	No
ENSMUSG00000020286	-3,55E+00	-2,711700534	5,65E+00	1,75E-02	1,38E-01	1700093K21Rik	0,09	No
ENSMUSG00000020287	1,03E-01	3,211818557	9,21E-01	3,37E-01	6,38E-01	Mpg	1,07	No
ENSMUSG00000020288	-4,33E-02	4,853275354	3,27E-01	5,68E-01	8,29E-01	Ahsa2	0,97	No
ENSMUSG00000020289	1,13E-01	4,537687858	2,00E+00	1,58E-01	5,04E-01	Nprl3	1,08	No
ENSMUSG00000020290	7,59E-02	7,381751671	1,63E+00	2,02E-01	5,50E-01	Xpo1	1,05	No
ENSMUSG00000020295	-8,69E-01	-2,963204587	4,16E-01	5,19E-01	7,98E-01	Hbq1a	0,55	No
ENSMUSG00000020297	-5,99E-02	-2,963282181	-7,02E-11	1,00E+00	1,00E+00	Nsg2	0,96	No
ENSMUSG00000020299	6,95E-01	-2,355648396	7,01E-01	4,03E-01	7,01E-01	4930524B15Rik	1,62	No
ENSMUSG00000020300	-1,82E-01	7,071279388	9,06E+00	2,61E-03	3,45E-02	Cpeb4	0,88	Yes
ENSMUSG00000020303	7,15E-01	-2,107696675	7,40E-01	3,90E-01	6,90E-01	Stc2	1,64	No
ENSMUSG00000020305	-2,00E-01	3,450168861	4,00E+00	4,54E-02	2,54E-01	Asb3	0,87	No
ENSMUSG00000020307	1,09E-01	5,508867284	2,48E+00	1,15E-01	4,19E-01	Cdc34	1,08	No
ENSMUSG00000020308	9,64E-02	4,603110217	1,49E+00	2,22E-01	5,50E-01	Tpgs1	1,07	No
ENSMUSG00000020309	1,51E-01	2,154240066	1,13E+00	2,87E-01	5,83E-01	Chac2	1,11	No
ENSMUSG00000020310	-2,68E+00	-2,963261035	2,84E+00	9,17E-02	3,82E-01	Madcam1	0,16	No
ENSMUSG00000020311	-7,75E-02	4,085840905	7,92E-01	3,73E-01	6,74E-01	Erlec1	0,95	No
ENSMUSG00000020312	5,42E-01	-1,088760876	1,51E+00	2,20E-01	5,50E-01	Shc2	1,46	No
ENSMUSG00000020315	1,98E-03	7,797306245	1,14E-03	9,73E-01	1,00E+00	Sptbn1	1,00	No
ENSMUSG00000020317	-8,93E-02	-2,96327978	3,63E-03	9,52E-01	1,00E+00	Theg	0,94	No
ENSMUSG00000020319	1,94E-01	0,913706673	8,23E-01	3,64E-01	6,65E-01	Wdpcp	1,14	No
ENSMUSG00000020321	-9,93E-03	6,803236242	2,62E-02	8,71E-01	1,00E+00	Mdh1	0,99	No
ENSMUSG00000020323	8,44E-01	-0,395739542	5,42E+00	1,99E-02	1,51E-01	Prss57	1,79	No
ENSMUSG00000020325	1,23E-01	-0,669098309	1,06E-01	7,45E-01	9,29E-01	Fstl3	1,09	No
ENSMUSG00000020326	6,35E-02	8,454303403	1,22E+00	2,69E-01	5,65E-01	Ccng1	1,05	No
ENSMUSG00000020327	1,08E-01	-0,620773269	7,96E-02	7,78E-01	9,47E-01	Fgf22	1,08	No
ENSMUSG00000020328	6,97E-02	5,765309871	1,08E+00	2,99E-01	5,95E-01	Nudcd2	1,05	No
ENSMUSG00000020329	1,72E-01	4,3944962	4,42E+00	3,56E-02	2,24E-01	Polrmt	1,13	No
ENSMUSG00000020330	1,41E-01	5,719576093	4,38E+00	3,63E-02	2,26E-01	Hmmr	1,10	No
ENSMUSG00000020331	-1,69E-01	0,062259805	3,50E-01	5,54E-01	8,20E-01	Hcn2	0,89	No
ENSMUSG00000020333	-5,88E-02	-3,033616507	1,62E-03	9,68E-01	1,00E+00	Acsl6	0,96	No
ENSMUSG00000020334	-2,97E-01	3,488202728	8,85E+00	2,94E-03	3,77E-02	Slc22a4	0,81	Yes

ENSMUSG00000020335	1,85E+00	-3,107657832	1,35E+00	2,46E-01	5,50E-01	Zfp354b	3,61	No
ENSMUSG00000020340	-2,67E-01	6,315567268	1,77E+01	2,62E-05	7,81E-04	Cyfip2	0,83	Yes
ENSMUSG00000020346	1,16E-01	6,883511405	3,59E+00	5,81E-02	2,97E-01	Mgat1	1,08	No
ENSMUSG00000020349	-3,58E-02	7,627307106	3,68E-01	5,44E-01	8,13E-01	Ppp2ca	0,98	No
ENSMUSG00000020354	-1,89E+00	-3,1076441	1,42E+00	2,33E-01	5,50E-01	Sgcd	0,27	No
ENSMUSG00000020357	1,99E+00	-0,740978224	2,18E+01	3,10E-06	1,20E-04	Flt4	3,96	Yes
ENSMUSG00000020358	9,45E-02	8,163500643	2,66E+00	1,03E-01	3,95E-01	Hnrnpab	1,07	No
ENSMUSG00000020359	8,54E-02	6,715247388	1,91E+00	1,67E-01	5,21E-01	Phykpl	1,06	No
ENSMUSG00000020361	1,02E-01	7,671504865	2,98E+00	8,43E-02	3,72E-01	Hspa4	1,07	No
ENSMUSG00000020362	5,86E-02	6,995672192	9,33E-01	3,34E-01	6,35E-01	Cnot6	1,04	No
ENSMUSG00000020363	-6,35E-02	-0,185585351	4,02E-02	8,41E-01	9,84E-01	Gfpt2	0,96	No
ENSMUSG00000020364	1,82E+00	-3,033667329	1,31E+00	2,52E-01	5,50E-01	Zfp354a	3,54	No
ENSMUSG00000020366	3,84E-02	6,105160436	3,50E-01	5,54E-01	8,20E-01	Mapk9	1,03	No
ENSMUSG00000020368	1,43E-01	8,86103257	6,26E+00	1,24E-02	1,09E-01	Canx	1,10	No
ENSMUSG00000020372	-1,01E-01	9,469172542	3,17E+00	7,51E-02	3,49E-01	Rack1	0,93	No
ENSMUSG00000020374	-5,60E-01	-2,655162252	2,46E-01	6,20E-01	8,57E-01	Rasgef1c	0,68	No
ENSMUSG00000020375	2,08E-01	5,298436174	8,50E+00	3,54E-03	4,32E-02	Rufy1	1,15	Yes
ENSMUSG00000020376	3,55E-02	6,7840021	3,31E-01	5,65E-01	8,27E-01	Rnf130	1,02	No
ENSMUSG00000020377	-5,21E-01	3,192033345	2,40E+01	9,45E-07	4,23E-05	Ltc4s	0,70	Yes
ENSMUSG00000020380	1,77E-01	5,298870657	6,28E+00	1,22E-02	1,08E-01	Rad50	1,13	No
ENSMUSG00000020381	-5,85E-02	0,791496937	7,12E-02	7,90E-01	9,54E-01	Mrnip	0,96	No
ENSMUSG00000020385	-2,60E-02	5,737086437	1,49E-01	6,99E-01	9,04E-01	Clk4	0,98	No
ENSMUSG00000020386	7,76E-02	5,098074705	1,12E+00	2,91E-01	5,86E-01	Sar1b	1,06	No
ENSMUSG00000020387	-6,60E-02	4,297733563	6,31E-01	4,27E-01	7,21E-01	Jade2	0,96	No
ENSMUSG00000020388	-6,91E-01	2,172533684	2,33E+01	1,37E-06	5,88E-05	Pdlim4	0,62	Yes
ENSMUSG00000020389	-8,88E-02	0,705370028	1,60E-01	6,89E-01	8,99E-01	Cdkl3	0,94	No
ENSMUSG00000020390	-1,47E-01	5,912668586	5,00E+00	2,54E-02	1,77E-01	Ube2b	0,90	No
ENSMUSG00000020392	-2,97E-02	5,214625316	1,72E-01	6,79E-01	8,93E-01	Cdkn2aipnl	0,98	No
ENSMUSG00000020393	-6,51E-02	5,40456611	8,76E-01	3,49E-01	6,50E-01	Kremen1	0,96	No
ENSMUSG00000020395	-1,36E+00	-2,711658292	1,16E+00	2,81E-01	5,77E-01	Itk	0,39	No
ENSMUSG00000020396	-1,71E-01	1,183530183	8,07E-01	3,69E-01	6,70E-01	Nefh	0,89	No
ENSMUSG00000020397	-2,46E-02	3,542712343	6,31E-02	8,02E-01	9,61E-01	Med7	0,98	No

ENSMUSG00000020399	4,94E-01	4,253334451	3,25E+01	1,17E-08	8,04E-07	Havcr2	1,41	Yes
ENSMUSG00000020400	-1,97E-01	6,508752132	9,89E+00	1,67E-03	2,42E-02	Tnip1	0,87	Yes
ENSMUSG00000020401	-2,55E-02	-2,711712527	5,34E-04	9,82E-01	1,00E+00	Fam71b	0,98	No
ENSMUSG00000020402	5,59E-02	7,493905241	8,89E-01	3,46E-01	6,47E-01	Vdac1	1,04	No
ENSMUSG00000020407	-6,77E-02	3,531242421	4,66E-01	4,95E-01	7,80E-01	Upp1	0,95	No
ENSMUSG00000020409	-5,18E-02	5,699271618	5,91E-01	4,42E-01	7,34E-01	Slu7	0,96	No
ENSMUSG00000020411	-1,36E+00	-2,548126199	1,17E+00	2,80E-01	5,77E-01	Nipal4	0,39	No
ENSMUSG00000020412	1,15E-01	6,298025858	3,29E+00	6,99E-02	3,34E-01	Ascc2	1,08	No
ENSMUSG00000020413	4,99E-02	4,10669354	3,26E-01	5,68E-01	8,29E-01	Hus1	1,04	No
ENSMUSG00000020415	-7,42E-02	3,611568233	5,98E-01	4,39E-01	7,32E-01	Pttg1	0,95	No
ENSMUSG00000020419	4,41E-01	-2,711701398	1,52E-01	6,97E-01	9,03E-01	Hormad2	1,36	No
ENSMUSG00000020420	1,27E-02	2,267846257	8,95E-03	9,25E-01	1,00E+00	Zfp607a	1,01	No
ENSMUSG00000020422	1,92E-01	7,148084834	1,02E+01	1,42E-03	2,14E-02	Tns3	1,14	Yes
ENSMUSG00000020423	3,89E-01	6,441511976	3,84E+01	5,72E-10	4,81E-08	Btg2	1,31	Yes
ENSMUSG00000020424	-3,69E-01	2,229884566	7,19E+00	7,33E-03	7,42E-02	Gatsl3	0,77	No
ENSMUSG00000020427	-7,98E-01	-2,770529399	3,51E-01	5,54E-01	8,19E-01	Igfbp3	0,58	No
ENSMUSG00000020430	2,96E-02	5,453679416	1,82E-01	6,70E-01	8,88E-01	Pes1	1,02	No
ENSMUSG00000020432	1,04E-02	6,552924498	2,75E-02	8,68E-01	1,00E+00	Tcn2	1,01	No
ENSMUSG00000020434	2,20E-01	-0,389976269	3,98E-01	5,28E-01	8,04E-01	4921536K21Rik	1,16	No
ENSMUSG00000020435	-3,29E-01	-0,668978647	7,17E-01	3,97E-01	6,97E-01	Osbp2	0,80	No
ENSMUSG00000020437	-4,06E-01	9,431284326	5,15E+01	7,01E-13	8,68E-11	Myo1g	0,75	Yes
ENSMUSG00000020439	3,47E-01	1,899044519	4,95E+00	2,62E-02	1,81E-01	Smtn	1,27	No
ENSMUSG00000020440	5,52E-04	6,861659163	8,16E-05	9,93E-01	1,00E+00	Arf5	1,00	No
ENSMUSG00000020441	1,03E-01	2,978025982	8,13E-01	3,67E-01	6,68E-01	2310033P09Rik	1,07	No
ENSMUSG00000020444	7,07E-02	4,536674677	7,81E-01	3,77E-01	6,78E-01	Guk1	1,05	No
ENSMUSG00000020447	-1,35E+00	-2,655140323	1,14E+00	2,86E-01	5,82E-01	Npc1l1	0,39	No
ENSMUSG00000020448	7,72E-02	4,717828541	1,01E+00	3,15E-01	6,15E-01	Rnf185	1,05	No
ENSMUSG00000020451	-4,72E-02	5,275641427	4,45E-01	5,05E-01	7,89E-01	Limk2	0,97	No
ENSMUSG00000020453	1,08E-01	4,264842467	1,66E+00	1,98E-01	5,50E-01	Patz1	1,08	No
ENSMUSG00000020454	3,05E-02	5,816388799	2,10E-01	6,47E-01	8,74E-01	Eif4enif1	1,02	No
ENSMUSG00000020455	1,21E-01	5,458412988	3,03E+00	8,18E-02	3,67E-01	Trim11	1,09	No
ENSMUSG00000020456	9,56E-02	7,787083983	2,66E+00	1,03E-01	3,95E-01	Ogdh	1,07	No

ENSMUSG00000020457	9,13E-03	5,698731411	1,83E-02	8,92E-01	1,00E+00	Drg1	1,01	No
ENSMUSG00000020458	-3,89E-02	8,05504733	4,48E-01	5,04E-01	7,88E-01	Rtn4	0,97	No
ENSMUSG00000020459	1,02E-01	4,809754514	1,78E+00	1,82E-01	5,44E-01	Mtif2	1,07	No
ENSMUSG00000020460	-1,70E-01	4,920416968	5,20E+00	2,26E-02	1,63E-01	Rps27a	0,89	No
ENSMUSG00000020461	1,51E-01	-1,48524039	7,48E-02	7,85E-01	9,51E-01	Clhc1	1,11	No
ENSMUSG00000020462	-2,00E-01	3,699166943	4,36E+00	3,69E-02	2,28E-01	Cfap36	0,87	No
ENSMUSG00000020463	1,01E-01	6,551556739	2,61E+00	1,06E-01	3,99E-01	Ppp4r3b	1,07	No
ENSMUSG00000020464	-5,32E-02	3,998999523	3,54E-01	5,52E-01	8,18E-01	Pnpt1	0,96	No
ENSMUSG00000020469	1,84E+00	-3,033649193	1,33E+00	2,49E-01	5,50E-01	Myl7	3,58	No
ENSMUSG00000020471	1,17E-01	4,281560894	1,92E+00	1,66E-01	5,19E-01	Pold2	1,08	No
ENSMUSG00000020472	-2,52E-02	5,812743987	1,42E-01	7,06E-01	9,08E-01	Zkscan17	0,98	No
ENSMUSG00000020473	-1,70E+00	-0,770330257	1,64E+01	5,18E-05	1,40E-03	Aebp1	0,31	Yes
ENSMUSG00000020474	8,93E-02	3,944566715	1,01E+00	3,15E-01	6,15E-01	Polm	1,06	No
ENSMUSG00000020475	-1,26E-01	3,004799217	1,23E+00	2,67E-01	5,63E-01	Pgam2	0,92	No
ENSMUSG00000020476	-1,26E-01	7,346797031	4,47E+00	3,46E-02	2,19E-01	Dbnl	0,92	No
ENSMUSG00000020477	7,34E-02	4,624472446	8,70E-01	3,51E-01	6,52E-01	Mrps24	1,05	No
ENSMUSG00000020481	-6,87E-01	-2,070084575	1,08E+00	3,00E-01	5,96E-01	Ankrd36	0,62	No
ENSMUSG00000020482	9,15E-03	4,919545296	1,51E-02	9,02E-01	1,00E+00	Ccdc117	1,01	No
ENSMUSG00000020483	-2,49E-01	6,742259468	1,65E+01	4,96E-05	1,35E-03	Dynll2	0,84	Yes
ENSMUSG00000020484	-7,40E-02	8,849502109	1,68E+00	1,95E-01	5,50E-01	Xbp1	0,95	No
ENSMUSG00000020485	-3,08E-01	4,813662659	1,66E+01	4,71E-05	1,30E-03	Supt4a	0,81	Yes
ENSMUSG00000020486	-1,20E+00	2,997996426	1,13E+02	2,39E-26	9,30E-24	SEPT4	0,44	Yes
ENSMUSG00000020491	7,23E-02	2,602074403	3,32E-01	5,65E-01	8,27E-01	2810021J22Rik	1,05	No
ENSMUSG00000020492	2,45E-01	3,439038701	5,97E+00	1,45E-02	1,23E-01	Ska2	1,19	No
ENSMUSG00000020493	2,33E-01	5,120693554	1,03E+01	1,35E-03	2,06E-02	Prr11	1,18	Yes
ENSMUSG00000020495	-1,24E-02	4,964767995	2,82E-02	8,67E-01	9,99E-01	Smg8	0,99	No
ENSMUSG00000020496	5,76E-02	6,846462687	8,82E-01	3,48E-01	6,49E-01	Rnf187	1,04	No
ENSMUSG00000020513	1,79E-01	2,638832026	2,05E+00	1,52E-01	4,94E-01	Tubd1	1,13	No
ENSMUSG00000020514	5,41E-02	2,326936478	1,60E-01	6,89E-01	8,99E-01	Mrpl22	1,04	No
ENSMUSG00000020515	4,59E-02	5,371659814	4,31E-01	5,11E-01	7,94E-01	Cnot8	1,03	No
ENSMUSG00000020516	-3,95E-03	5,595517109	3,34E-03	9,54E-01	1,00E+00	Rps6kb1	1,00	No
ENSMUSG00000020519	1,05E-01	4,073466728	1,45E+00	2,28E-01	5,50E-01	Sap30l	1,08	No

ENSMUSG00000020520	3,04E-01	5,964631634	2,14E+01	3,82E-06	1,44E-04	Galnt10	1,23	Yes
ENSMUSG00000020521	1,24E-01	5,786091739	3,45E+00	6,32E-02	3,14E-01	Rnft1	1,09	No
ENSMUSG00000020522	1,88E-01	6,115202805	8,46E+00	3,62E-03	4,40E-02	Mfap3	1,14	Yes
ENSMUSG00000020523	6,61E-03	5,623315086	9,44E-03	9,23E-01	1,00E+00	Fam114a2	1,00	No
ENSMUSG00000020525	1,51E-01	4,185220209	3,20E+00	7,36E-02	3,45E-01	Ppm1d	1,11	No
ENSMUSG00000020526	1,38E-01	2,97989903	1,46E+00	2,27E-01	5,50E-01	Znhit3	1,10	No
ENSMUSG00000020527	3,61E-01	3,548887523	1,32E+01	2,86E-04	5,82E-03	Myo19	1,28	Yes
ENSMUSG00000020528	-1,91E-02	3,392222092	3,57E-02	8,50E-01	9,89E-01	Prpsap2	0,99	No
ENSMUSG00000020530	-8,83E-02	6,598325275	2,01E+00	1,57E-01	5,01E-01	Ggnbp2	0,94	No
ENSMUSG00000020532	1,55E-01	5,380529407	4,86E+00	2,75E-02	1,87E-01	Acaca	1,11	No
ENSMUSG00000020534	3,90E-02	3,597721466	1,58E-01	6,91E-01	9,00E-01	Shmt1	1,03	No
ENSMUSG00000020536	-1,17E-01	5,154910117	2,61E+00	1,06E-01	3,99E-01	Llgl1	0,92	No
ENSMUSG00000020537	2,36E-02	4,562932495	8,81E-02	7,67E-01	9,40E-01	Drg2	1,02	No
ENSMUSG00000020538	2,84E-01	7,20340748	2,22E+01	2,48E-06	9,76E-05	Srebf1	1,22	Yes
ENSMUSG00000020541	8,83E-02	2,264602541	4,09E-01	5,23E-01	8,01E-01	Tom1l1	1,06	No
ENSMUSG00000020544	-1,20E-01	2,861776199	1,06E+00	3,04E-01	6,01E-01	Cox11	0,92	No
ENSMUSG00000020545	-8,86E-02	-2,963249454	3,57E-03	9,52E-01	1,00E+00	Lrrc72	0,94	No
ENSMUSG00000020546	-1,41E-01	1,904124549	7,74E-01	3,79E-01	6,80E-01	Stxbp4	0,91	No
ENSMUSG00000020547	-8,22E-03	4,988855016	1,22E-02	9,12E-01	1,00E+00	Bzw2	0,99	No
ENSMUSG00000020549	2,24E-02	4,57358505	7,93E-02	7,78E-01	9,47E-01	Elac2	1,02	No
ENSMUSG00000020553	3,83E-02	4,747544749	2,46E-01	6,20E-01	8,57E-01	Pctp	1,03	No
ENSMUSG00000020561	5,97E-02	4,661518133	5,80E-01	4,46E-01	7,37E-01	Twistnb	1,04	No
ENSMUSG00000020564	-2,65E-01	6,177627853	1,70E+01	3,75E-05	1,06E-03	Atxn7l1	0,83	Yes
ENSMUSG00000020566	6,80E-01	-1,770634609	1,17E+00	2,79E-01	5,76E-01	Atp6v1c2	1,60	No
ENSMUSG00000020570	1,25E-01	6,246311126	3,77E+00	5,22E-02	2,78E-01	Sypl	1,09	No
ENSMUSG00000020571	1,02E-01	7,854304265	3,03E+00	8,17E-02	3,67E-01	Pdia6	1,07	No
ENSMUSG00000020572	1,04E-01	7,681326786	3,13E+00	7,70E-02	3,54E-01	Nampt	1,08	No
ENSMUSG00000020573	-1,43E-02	8,071634807	6,07E-02	8,05E-01	9,63E-01	Pik3cg	0,99	No
ENSMUSG00000020576	8,60E-02	4,617407146	1,19E+00	2,75E-01	5,72E-01	Nbas	1,06	No
ENSMUSG00000020577	-6,32E-01	4,061952764	5,47E+01	1,43E-13	1,95E-11	Tspan13	0,65	Yes
ENSMUSG00000020580	-3,05E-02	6,990463119	2,52E-01	6,16E-01	8,54E-01	Rock2	0,98	No
ENSMUSG00000020583	1,22E+00	-2,896168849	9,19E-01	3,38E-01	6,38E-01	Matn3	2,33	No

ENSMUSG00000020585	-2,41E-02	7,478845208	1,65E-01	6,85E-01	8,97E-01	Laptm4a	0,98	No
ENSMUSG00000020589	-4,23E-01	6,348530568	4,49E+01	2,09E-11	2,14E-09	Fam49a	0,75	Yes
ENSMUSG00000020590	1,29E-01	5,64725416	3,63E+00	5,69E-02	2,93E-01	Snx13	1,09	No
ENSMUSG00000020592	-2,91E-01	6,417110961	2,11E+01	4,26E-06	1,58E-04	Sdc1	0,82	Yes
ENSMUSG00000020593	3,36E-01	5,965520507	2,62E+01	3,09E-07	1,54E-05	Lpin1	1,26	Yes
ENSMUSG00000020594	5,56E-02	7,770792914	9,02E-01	3,42E-01	6,43E-01	Pum2	1,04	No
ENSMUSG00000020598	-8,33E-01	-0,963228689	3,77E+00	5,22E-02	2,78E-01	Nrcam	0,56	No
ENSMUSG00000020599	1,42E+00	-0,378389802	1,46E+01	1,36E-04	3,13E-03	Rgs9	2,67	Yes
ENSMUSG00000020600	6,46E-01	-0,655014231	2,59E+00	1,07E-01	4,01E-01	Slc7a15	1,56	No
ENSMUSG00000020601	9,19E-01	-0,00697814	9,53E+00	2,02E-03	2,83E-02	Trib2	1,89	Yes
ENSMUSG00000020604	-2,64E-01	1,881374337	2,87E+00	9,04E-02	3,81E-01	Arsg	0,83	No
ENSMUSG00000020605	1,69E-01	5,010221477	5,30E+00	2,13E-02	1,57E-01	Hs1bp3	1,12	No
ENSMUSG00000020608	3,21E-02	6,64362592	2,68E-01	6,05E-01	8,49E-01	Smc6	1,02	No
ENSMUSG00000020609	-4,13E-02	-3,033659495	7,08E-04	9,79E-01	1,00E+00	Apob	0,97	No
ENSMUSG00000020610	4,27E-02	4,882677372	3,19E-01	5,72E-01	8,32E-01	Amz2	1,03	No
ENSMUSG00000020611	-7,68E-02	7,877750363	1,73E+00	1,89E-01	5,50E-01	Gna13	0,95	No
ENSMUSG00000020612	-4,13E-02	8,074205598	5,06E-01	4,77E-01	7,65E-01	Prkar1a	0,97	No
ENSMUSG00000020614	4,80E-01	-2,033612225	4,46E-01	5,04E-01	7,89E-01	Fam20a	1,39	No
ENSMUSG00000020617	1,17E+00	-3,14612376	6,40E-01	4,24E-01	7,18E-01	1700012B07Rik	2,26	No
ENSMUSG00000020620	-2,20E-01	0,894911973	1,10E+00	2,94E-01	5,89E-01	Abca8b	0,86	No
ENSMUSG00000020621	1,24E-01	3,200450731	1,36E+00	2,43E-01	5,50E-01	Rdh14	1,09	No
ENSMUSG00000020622	-1,22E+00	-2,655152082	1,43E+00	2,32E-01	5,50E-01	Nt5c1b	0,43	No
ENSMUSG00000020623	5,02E-01	1,532949466	7,64E+00	5,69E-03	6,15E-02	Map2k6	1,42	No
ENSMUSG00000020627	-5,66E-01	-2,655104584	2,51E-01	6,17E-01	8,55E-01	Klhl29	0,68	No
ENSMUSG00000020628	-8,72E-02	5,867607058	1,75E+00	1,86E-01	5,50E-01	Trappc12	0,94	No
ENSMUSG00000020629	3,53E-02	5,087828878	2,36E-01	6,27E-01	8,61E-01	Adi1	1,02	No
ENSMUSG00000020630	-1,72E-02	3,837122502	3,53E-02	8,51E-01	9,90E-01	Rnaseh1	0,99	No
ENSMUSG00000020634	-2,01E-01	5,395030334	8,29E+00	3,98E-03	4,71E-02	Ubxn2a	0,87	Yes
ENSMUSG00000020635	1,68E-02	4,189072564	3,94E-02	8,43E-01	9,85E-01	Fkbp1b	1,01	No
ENSMUSG00000020638	-2,27E-01	2,583163437	3,35E+00	6,71E-02	3,26E-01	Cmpk2	0,85	No
ENSMUSG00000020639	-8,00E-01	-1,816355046	1,23E+00	2,68E-01	5,65E-01	Pfn4	0,57	No
ENSMUSG00000020640	5,60E-02	7,735502506	9,10E-01	3,40E-01	6,41E-01	Itsn2	1,04	No

ENSMUSG00000020641	-6,48E-01	3,509954054	4,37E+01	3,91E-11	3,89E-09	Rsad2	0,64	Yes
ENSMUSG00000020642	-8,23E-02	-2,107697469	1,09E-02	9,17E-01	1,00E+00	Rnf144a	0,94	No
ENSMUSG00000020644	-1,66E-01	8,891259507	8,51E+00	3,53E-03	4,32E-02	Id2	0,89	Yes
ENSMUSG00000020646	3,84E+00	-2,770625943	6,81E+00	9,06E-03	8,68E-02	Mboat2	14,30	No
ENSMUSG00000020647	-7,53E-03	5,7872324	1,25E-02	9,11E-01	1,00E+00	Ncoa1	0,99	No
ENSMUSG00000020648	2,48E-01	1,658354432	2,24E+00	1,35E-01	4,62E-01	Dus4l	1,19	No
ENSMUSG00000020649	1,40E-01	6,530524961	4,95E+00	2,60E-02	1,80E-01	Rrm2	1,10	No
ENSMUSG00000020650	7,19E-02	5,649715906	1,12E+00	2,89E-01	5,85E-01	Bcap29	1,05	No
ENSMUSG00000020651	-3,80E-01	-1,497405064	4,86E-01	4,86E-01	7,73E-01	Slc26a4	0,77	No
ENSMUSG00000020652	1,10E-01	4,126438366	1,62E+00	2,03E-01	5,50E-01	Cenpo	1,08	No
ENSMUSG00000020653	2,15E-01	3,199040968	3,84E+00	5,01E-02	2,71E-01	Klf11	1,16	No
ENSMUSG00000020654	3,43E-01	2,648919883	7,42E+00	6,45E-03	6,76E-02	Adcy3	1,27	No
ENSMUSG00000020656	2,48E-02	1,610619788	2,10E-02	8,85E-01	1,00E+00	Grhl1	1,02	No
ENSMUSG00000020657	6,30E-02	2,913292337	3,01E-01	5,83E-01	8,38E-01	Dnajc27	1,04	No
ENSMUSG00000020658	1,51E-01	2,436471086	1,37E+00	2,41E-01	5,50E-01	Efr3b	1,11	No
ENSMUSG00000020659	-6,18E-03	3,904155633	4,68E-03	9,45E-01	1,00E+00	Cbll1	1,00	No
ENSMUSG00000020660	4,16E-01	-1,195632939	6,87E-01	4,07E-01	7,04E-01	Pomc	1,33	No
ENSMUSG00000020661	-7,37E-02	7,706908051	1,58E+00	2,09E-01	5,50E-01	Dnmt3a	0,95	No
ENSMUSG00000020664	2,17E-02	6,222821542	1,14E-01	7,35E-01	9,24E-01	Dld	1,02	No
ENSMUSG00000020668	-2,25E-01	3,71729958	5,69E+00	1,71E-02	1,36E-01	Kif3c	0,86	No
ENSMUSG00000020669	-3,09E-01	-0,155910131	1,05E+00	3,04E-01	6,01E-01	Sh3yl1	0,81	No
ENSMUSG00000020671	-1,16E-01	7,299582876	3,79E+00	5,16E-02	2,76E-01	Rab10	0,92	No
ENSMUSG00000020674	-5,67E-02	-2,831986495	2,17E-03	9,63E-01	1,00E+00	Pxdn	0,96	No
ENSMUSG00000020676	9,01E-03	-3,107646452	-5,16E-10	1,00E+00	1,00E+00	Ccl11	1,01	No
ENSMUSG00000020677	-5,86E-02	5,281634325	6,79E-01	4,10E-01	7,06E-01	Ddx52	0,96	No
ENSMUSG00000020679	-8,38E-01	-1,896174176	1,90E+00	1,68E-01	5,22E-01	Hnf1b	0,56	No
ENSMUSG00000020680	-1,05E-01	5,593913691	2,36E+00	1,25E-01	4,42E-01	Taf15	0,93	No
ENSMUSG00000020681	7,02E-01	3,46920208	4,61E+01	1,10E-11	1,15E-09	Ace	1,63	Yes
ENSMUSG00000020682	6,92E-01	-0,407279257	3,82E+00	5,08E-02	2,73E-01	Mmp28	1,62	No
ENSMUSG00000020684	-6,07E-01	-1,126741235	1,86E+00	1,72E-01	5,29E-01	Rasl10b	0,66	No
ENSMUSG00000020686	-3,83E-02	-3,107651936	-7,91E-11	1,00E+00	1,00E+00	Gas2l2	0,97	No
ENSMUSG00000020687	8,62E-02	5,973322322	1,73E+00	1,88E-01	5,50E-01	Cdc27	1,06	No

ENSMUSG00000020689	-6,00E-01	2,34077886	2,04E+01	6,24E-06	2,19E-04	Itgb3	0,66	Yes
ENSMUSG00000020691	-3,19E-02	3,741485334	1,17E-01	7,33E-01	9,23E-01	Mettl2	0,98	No
ENSMUSG00000020692	7,48E-02	3,534999965	5,60E-01	4,54E-01	7,44E-01	Nle1	1,05	No
ENSMUSG00000020694	-4,86E-02	4,842612695	4,11E-01	5,21E-01	8,00E-01	Tlk2	0,97	No
ENSMUSG00000020695	-4,99E-01	-0,509911679	1,95E+00	1,62E-01	5,12E-01	Mrc2	0,71	No
ENSMUSG00000020696	3,48E-02	4,504139769	1,85E-01	6,67E-01	8,86E-01	Rffl	1,02	No
ENSMUSG00000020697	7,25E-02	4,818536542	9,11E-01	3,40E-01	6,41E-01	Lig3	1,05	No
ENSMUSG00000020698	7,67E-01	-1,726176939	1,61E+00	2,04E-01	5,50E-01	Cct6b	1,70	No
ENSMUSG00000020700	-3,75E-02	6,504993597	3,59E-01	5,49E-01	8,16E-01	Map3k3	0,97	No
ENSMUSG00000020701	-1,87E+00	-3,107637761	1,39E+00	2,38E-01	5,50E-01	Tmem132e	0,27	No
ENSMUSG00000020702	-9,55E-01	-2,711690055	7,99E-01	3,71E-01	6,72E-01	Ccl1	0,52	No
ENSMUSG00000020703	1,85E+00	-3,107657832	1,35E+00	2,46E-01	5,50E-01	5530401A14Rik	3,61	No
ENSMUSG00000020705	1,99E-03	6,32780603	9,80E-04	9,75E-01	1,00E+00	Ddx42	1,00	No
ENSMUSG00000020706	3,18E-02	5,55349379	2,13E-01	6,44E-01	8,72E-01	Ftsj3	1,02	No
ENSMUSG00000020707	-9,26E-02	3,248842709	8,07E-01	3,69E-01	6,70E-01	Rnf135	0,94	No
ENSMUSG00000020708	-4,98E-02	5,721221151	5,45E-01	4,60E-01	7,49E-01	Psmc5	0,97	No
ENSMUSG00000020709	-1,61E-02	3,404477011	2,41E-02	8,77E-01	1,00E+00	Adap2	0,99	No
ENSMUSG00000020712	-1,33E+00	-1,863693165	3,04E+00	8,14E-02	3,66E-01	Tcam1	0,40	No
ENSMUSG00000020713	-1,19E+00	-2,268035937	2,29E+00	1,30E-01	4,54E-01	Gh	0,44	No
ENSMUSG00000020715	-1,92E-02	5,661880006	8,12E-02	7,76E-01	9,45E-01	Ern1	0,99	No
ENSMUSG00000020716	3,64E-02	5,819008389	2,96E-01	5,86E-01	8,40E-01	Nf1	1,03	No
ENSMUSG00000020717	3,05E-01	2,256339507	4,58E+00	3,23E-02	2,09E-01	Pecam1	1,24	No
ENSMUSG00000020718	1,82E-01	2,571362855	2,06E+00	1,51E-01	4,93E-01	Polg2	1,13	No
ENSMUSG00000020719	-1,16E-01	9,542348655	4,22E+00	4,00E-02	2,38E-01	Ddx5	0,92	No
ENSMUSG00000020720	5,19E-03	5,630940233	5,81E-03	9,39E-01	1,00E+00	Psmd12	1,00	No
ENSMUSG00000020721	-7,15E-02	5,744282423	1,14E+00	2,86E-01	5,82E-01	Helz	0,95	No
ENSMUSG00000020723	-5,25E-02	-3,033661434	4,08E-11	1,00E+00	1,00E+00	Cacng4	0,96	No
ENSMUSG00000020728	-4,07E-01	-1,600617323	5,19E-01	4,71E-01	7,60E-01	Cep112	0,75	No
ENSMUSG00000020732	2,69E-01	1,399279933	1,99E+00	1,58E-01	5,05E-01	Rab37	1,21	No
ENSMUSG00000020733	2,73E-02	7,117881492	2,05E-01	6,51E-01	8,76E-01	Slc9a3r1	1,02	No
ENSMUSG00000020734	-1,09E+00	0,362327624	1,93E+01	1,12E-05	3,72E-04	Grin2c	0,47	Yes
ENSMUSG00000020736	-1,26E-01	5,632965714	3,42E+00	6,43E-02	3,17E-01	Nt5c	0,92	No

ENSMUSG00000020737	1,03E-02	7,433293293	2,98E-02	8,63E-01	9,97E-01	Jpt1	1,01	No
ENSMUSG00000020738	-1,45E-01	4,878899307	3,71E+00	5,41E-02	2,84E-01	Sumo2	0,90	No
ENSMUSG00000020739	-3,71E-02	5,357493428	2,79E-01	5,97E-01	8,45E-01	Nup85	0,97	No
ENSMUSG00000020740	-1,25E-01	5,773122152	3,52E+00	6,07E-02	3,05E-01	Gga3	0,92	No
ENSMUSG00000020741	1,01E-01	6,914297816	2,75E+00	9,70E-02	3,88E-01	Cluh	1,07	No
ENSMUSG00000020743	-2,11E-01	5,588609297	9,58E+00	1,97E-03	2,76E-02	Mif4gd	0,86	Yes
ENSMUSG00000020744	-5,00E-02	4,783181443	4,26E-01	5,14E-01	7,96E-01	Slc25a19	0,97	No
ENSMUSG00000020745	1,03E-02	7,292992466	2,95E-02	8,64E-01	9,97E-01	Pafah1b1	1,01	No
ENSMUSG00000020747	9,67E-02	5,457272859	1,94E+00	1,64E-01	5,15E-01	Tmem94	1,07	No
ENSMUSG00000020752	1,73E-01	4,903670523	5,42E+00	1,99E-02	1,51E-01	Recql5	1,13	No
ENSMUSG00000020755	8,10E-02	4,77982482	1,12E+00	2,90E-01	5,85E-01	Sap30bp	1,06	No
ENSMUSG00000020758	-7,57E-01	-0,997887306	3,04E+00	8,15E-02	3,66E-01	Itgb4	0,59	No
ENSMUSG00000020766	2,40E-01	4,019822873	7,27E+00	7,00E-03	7,18E-02	Galk1	1,18	No
ENSMUSG00000020770	6,71E-02	4,993686736	8,26E-01	3,63E-01	6,65E-01	Unk	1,05	No
ENSMUSG00000020773	3,80E-01	4,016540906	1,79E+01	2,39E-05	7,23E-04	Trim47	1,30	Yes
ENSMUSG00000020774	1,84E+00	-2,770627672	1,33E+00	2,49E-01	5,50E-01	Aspa	3,58	No
ENSMUSG00000020775	1,24E-01	3,961098272	1,94E+00	1,64E-01	5,15E-01	Mrpl38	1,09	No
ENSMUSG00000020776	3,47E-02	4,334148012	1,78E-01	6,73E-01	8,90E-01	Fbf1	1,02	No
ENSMUSG00000020777	9,15E-02	6,864156868	2,23E+00	1,35E-01	4,63E-01	Acox1	1,07	No
ENSMUSG00000020778	2,82E-01	4,662823055	1,28E+01	3,37E-04	6,66E-03	Ten1	1,22	Yes
ENSMUSG00000020780	-2,70E-02	5,515710916	1,52E-01	6,97E-01	9,03E-01	Srp68	0,98	No
ENSMUSG00000020781	-5,81E-02	3,624013132	3,76E-01	5,40E-01	8,10E-01	Tsen54	0,96	No
ENSMUSG00000020782	1,08E-01	3,430529566	1,14E+00	2,86E-01	5,82E-01	Llgl2	1,08	No
ENSMUSG00000020783	7,44E-03	5,642390103	1,20E-02	9,13E-01	1,00E+00	Ncbp3	1,01	No
ENSMUSG00000020785	-1,02E+00	-0,785521987	6,71E+00	9,59E-03	9,04E-02	Camkk1	0,49	No
ENSMUSG00000020787	-2,18E-01	0,432272158	7,11E-01	3,99E-01	6,98E-01	P2rx1	0,86	No
ENSMUSG00000020788	6,15E-02	7,135694149	1,03E+00	3,10E-01	6,08E-01	Atp2a3	1,04	No
ENSMUSG00000020790	8,98E-02	7,542844046	2,30E+00	1,29E-01	4,51E-01	Ankfy1	1,06	No
ENSMUSG00000020792	3,16E-02	4,945120207	1,80E-01	6,71E-01	8,89E-01	Exoc7	1,02	No
ENSMUSG00000020793	6,22E-01	-0,998209429	1,71E+00	1,91E-01	5,50E-01	Galr2	1,54	No
ENSMUSG00000020794	1,09E-02	5,451360206	2,46E-02	8,75E-01	1,00E+00	Ube2g1	1,01	No
ENSMUSG00000020798	-3,59E-01	1,99410835	5,91E+00	1,51E-02	1,26E-01	Spns3	0,78	No

ENSMUSG00000020801	1,49E-01	2,205365748	1,09E+00	2,97E-01	5,92E-01	Med31	1,11	No
ENSMUSG00000020802	4,47E-02	4,689830076	3,28E-01	5,67E-01	8,29E-01	Ube2o	1,03	No
ENSMUSG00000020803	-1,82E-01	6,466138185	8,40E+00	3,76E-03	4,52E-02	Txndc17	0,88	Yes
ENSMUSG00000020804	-1,87E+00	-2,185550852	4,11E+00	4,27E-02	2,46E-01	Aanat	0,27	No
ENSMUSG00000020806	9,17E-02	6,31017699	2,07E+00	1,50E-01	4,91E-01	Rhbdf2	1,07	No
ENSMUSG00000020807	7,35E-02	3,745627709	6,27E-01	4,28E-01	7,22E-01	4933427D14Rik	1,05	No
ENSMUSG00000020808	1,13E-01	4,090467437	1,69E+00	1,94E-01	5,50E-01	Pimreg	1,08	No
ENSMUSG00000020810	5,11E-01	-1,006779088	1,11E+00	2,93E-01	5,88E-01	Cygb	1,43	No
ENSMUSG00000020812	-2,95E-01	3,050203714	7,00E+00	8,16E-03	8,02E-02	1810032O08Rik	0,82	No
ENSMUSG00000020814	-3,55E-01	-1,126575625	6,15E-01	4,33E-01	7,26E-01	Mxra7	0,78	No
ENSMUSG00000020817	1,71E-02	6,466118074	7,38E-02	7,86E-01	9,52E-01	Rabep1	1,01	No
ENSMUSG00000020818	2,19E-01	6,326096662	1,18E+01	5,82E-04	1,04E-02	Mfsd11	1,16	Yes
ENSMUSG00000020821	-7,34E-03	7,682233923	1,56E-02	9,01E-01	1,00E+00	Kif1c	0,99	No
ENSMUSG00000020823	1,12E-01	6,281571336	3,10E+00	7,84E-02	3,58E-01	Sec14l1	1,08	No
ENSMUSG00000020826	5,91E-02	0,939980905	8,03E-02	7,77E-01	9,46E-01	Nos2	1,04	No
ENSMUSG00000020827	-1,92E-01	7,769313638	1,08E+01	1,02E-03	1,64E-02	Mink1	0,88	Yes
ENSMUSG00000020828	-1,42E-02	5,802777209	4,58E-02	8,30E-01	9,77E-01	Pld2	0,99	No
ENSMUSG00000020829	2,16E-01	1,240156169	1,22E+00	2,70E-01	5,66E-01	Slc46a1	1,16	No
ENSMUSG00000020830	-4,86E-02	-2,896158361	1,09E-03	9,74E-01	1,00E+00	Vmo1	0,97	No
ENSMUSG00000020831	-6,28E-02	3,322932681	3,61E-01	5,48E-01	8,15E-01	0610010K14Rik	0,96	No
ENSMUSG00000020832	9,79E-03	4,060526936	1,22E-02	9,12E-01	1,00E+00	Eral1	1,01	No
ENSMUSG00000020834	1,63E-01	2,625467581	1,69E+00	1,94E-01	5,50E-01	Dhrs13	1,12	No
ENSMUSG00000020836	-1,17E-01	2,119929303	6,33E-01	4,26E-01	7,21E-01	Coro6	0,92	No
ENSMUSG00000020838	7,61E-01	5,11987344	1,04E+02	1,88E-24	6,42E-22	Slc6a4	1,69	Yes
ENSMUSG00000020839	-1,89E+00	-3,1076441	1,42E+00	2,33E-01	5,50E-01	Tmigd1	0,27	No
ENSMUSG00000020840	8,96E-03	5,302680596	1,59E-02	9,00E-01	1,00E+00	Blmh	1,01	No
ENSMUSG00000020841	1,50E-01	7,863421261	6,57E+00	1,04E-02	9,58E-02	Cpd	1,11	No
ENSMUSG00000020843	8,62E-02	4,579415128	1,18E+00	2,77E-01	5,74E-01	Timm22	1,06	No
ENSMUSG00000020844	1,31E-01	3,627351077	1,84E+00	1,75E-01	5,34E-01	Nxn	1,09	No
ENSMUSG00000020846	-2,48E-02	2,897812957	4,49E-02	8,32E-01	9,78E-01	Rflnb	0,98	No
ENSMUSG00000020847	-9,78E-01	-1,683060196	3,00E+00	8,33E-02	3,71E-01	Rph3al	0,51	No
ENSMUSG00000020848	3,14E-02	-3,107617333	-7,69E-10	1,00E+00	1,00E+00	Doc2b	1,02	No

ENSMUSG00000020849	2,36E-02	8,488081826	1,68E-01	6,82E-01	8,94E-01	Ywhae	1,02	No
ENSMUSG00000020850	5,68E-02	8,632379032	9,83E-01	3,22E-01	6,22E-01	Prpf8	1,04	No
ENSMUSG00000020857	-1,62E-02	5,108876228	4,94E-02	8,24E-01	9,73E-01	Nme2	0,99	No
ENSMUSG00000020859	-9,70E-02	8,11314734	2,80E+00	9,43E-02	3,84E-01	Spag9	0,94	No
ENSMUSG00000020863	-3,32E-02	5,956070968	2,57E-01	6,12E-01	8,52E-01	Luc7l3	0,98	No
ENSMUSG00000020864	-5,36E-02	6,11207619	6,83E-01	4,09E-01	7,05E-01	Ankrd40	0,96	No
ENSMUSG00000020865	-1,91E-02	3,771838812	4,08E-02	8,40E-01	9,83E-01	Abcc3	0,99	No
ENSMUSG00000020866	-8,41E-01	-2,89611984	3,88E-01	5,33E-01	8,06E-01	Cacna1g	0,56	No
ENSMUSG00000020868	1,15E-01	5,251993863	2,57E+00	1,09E-01	4,05E-01	Xylt2	1,08	No
ENSMUSG00000020869	5,72E-02	7,75123985	9,48E-01	3,30E-01	6,31E-01	Lrrc59	1,04	No
ENSMUSG00000020870	-1,16E-01	4,728826711	2,25E+00	1,34E-01	4,60E-01	Cdc34b	0,92	No
ENSMUSG00000020871	-8,42E-01	-2,896108869	3,88E-01	5,33E-01	8,06E-01	Dlx4	0,56	No
ENSMUSG00000020872	-1,97E+00	-1,89607874	8,28E+00	4,01E-03	4,74E-02	Tac4	0,25	Yes
ENSMUSG00000020873	1,16E-01	5,890883795	3,04E+00	8,13E-02	3,66E-01	Slc35b1	1,08	No
ENSMUSG00000020876	3,18E-02	5,436932635	2,10E-01	6,46E-01	8,74E-01	Snx11	1,02	No
ENSMUSG00000020877	8,36E-05	1,65300073	2,60E-07	1,00E+00	1,00E+00	Scrn2	1,00	No
ENSMUSG00000020878	-6,92E-01	-1,863656664	1,09E+00	2,96E-01	5,91E-01	Lrrc46	0,62	No
ENSMUSG00000020882	-1,76E-01	3,693717037	3,53E+00	6,02E-02	3,04E-01	Cacnb1	0,89	No
ENSMUSG00000020883	-1,06E-01	6,73619568	2,96E+00	8,51E-02	3,75E-01	Fbxl20	0,93	No
ENSMUSG00000020884	-1,42E+00	-2,896085476	1,27E+00	2,60E-01	5,53E-01	Asgr1	0,37	No
ENSMUSG00000020886	-4,43E-02	2,081357184	8,84E-02	7,66E-01	9,40E-01	Dlg4	0,97	No
ENSMUSG00000020887	6,05E-01	-2,863749462	1,46E-01	7,03E-01	9,06E-01	A230052G05Rik	1,52	No
ENSMUSG00000020888	9,89E-02	4,764472048	1,66E+00	1,97E-01	5,50E-01	Dvl2	1,07	No
ENSMUSG00000020889	1,04E-01	2,048781559	4,72E-01	4,92E-01	7,78E-01	Nr1d1	1,07	No
ENSMUSG00000020890	-4,51E-01	-2,033646651	3,75E-01	5,40E-01	8,10E-01	Gucy2e	0,73	No
ENSMUSG00000020891	-1,27E+00	-2,355604143	1,55E+00	2,14E-01	5,50E-01	Alox8	0,41	No
ENSMUSG00000020892	3,07E-01	-0,522810237	7,72E-01	3,79E-01	6,80E-01	Aloxe3	1,24	No
ENSMUSG00000020893	-3,66E-02	6,221609127	3,20E-01	5,71E-01	8,32E-01	Per1	0,97	No
ENSMUSG00000020894	-1,23E-01	5,677929502	3,33E+00	6,81E-02	3,28E-01	Vamp2	0,92	No
ENSMUSG00000020895	-1,08E-01	1,74776437	4,50E-01	5,02E-01	7,87E-01	Tmem107	0,93	No
ENSMUSG00000020897	2,43E-01	5,410910914	1,20E+01	5,29E-04	9,59E-03	Aurkb	1,18	Yes
ENSMUSG00000020898	-1,28E-02	5,272859043	3,23E-02	8,57E-01	9,94E-01	Ctc1	0,99	No

ENSMUSG00000020899	2,24E-01	4,972913569	9,12E+00	2,52E-03	3,36E-02	Pfas	1,17	Yes
ENSMUSG00000020900	-2,03E-01	3,662188942	4,47E+00	3,45E-02	2,19E-01	Myh10	0,87	No
ENSMUSG00000020901	-2,82E-01	8,111241881	2,37E+01	1,13E-06	4,93E-05	Pik3r5	0,82	Yes
ENSMUSG00000020902	-1,56E+00	-1,963136046	4,52E+00	3,34E-02	2,14E-01	Ntn1	0,34	No
ENSMUSG00000020903	-8,68E-02	4,401063515	1,13E+00	2,88E-01	5,83E-01	Stx8	0,94	No
ENSMUSG00000020904	-5,32E-01	-2,83201529	2,19E-01	6,40E-01	8,70E-01	Cfap52	0,69	No
ENSMUSG00000020905	1,83E-01	-2,683145648	2,84E-02	8,66E-01	9,99E-01	Usp43	1,14	No
ENSMUSG00000020907	2,07E+00	-2,226374873	5,44E+00	1,97E-02	1,50E-01	Rcvrn	4,19	No
ENSMUSG00000020908	-2,66E-01	-2,145931622	1,23E-01	7,26E-01	9,18E-01	Myh3	0,83	No
ENSMUSG00000020910	-1,17E-01	3,544299627	1,44E+00	2,31E-01	5,50E-01	Adprm	0,92	No
ENSMUSG00000020911	1,85E+00	-3,107657832	1,35E+00	2,46E-01	5,50E-01	Krt19	3,61	No
ENSMUSG00000020914	2,09E-01	8,003458337	1,29E+01	3,37E-04	6,66E-03	Top2a	1,16	Yes
ENSMUSG00000020917	1,29E-01	8,48267692	5,02E+00	2,50E-02	1,75E-01	Acly	1,09	No
ENSMUSG00000020918	1,52E-01	4,859294691	4,02E+00	4,49E-02	2,53E-01	Kat2a	1,11	No
ENSMUSG00000020919	-7,69E-02	6,602038395	1,54E+00	2,15E-01	5,50E-01	Stat5b	0,95	No
ENSMUSG00000020921	-5,90E-02	3,797960517	4,03E-01	5,26E-01	8,03E-01	Tmem101	0,96	No
ENSMUSG00000020922	1,08E-01	5,083819127	2,17E+00	1,41E-01	4,74E-01	Lsm12	1,08	No
ENSMUSG00000020923	8,70E-02	6,774282523	2,00E+00	1,57E-01	5,03E-01	Ubtf	1,06	No
ENSMUSG00000020925	8,52E-02	4,65295852	1,18E+00	2,77E-01	5,74E-01	Ccdc43	1,06	No
ENSMUSG00000020926	-8,55E-01	5,415498341	1,51E+02	1,31E-34	1,06E-31	Adam11	0,55	Yes
ENSMUSG00000020928	-1,86E+00	-3,033612668	1,39E+00	2,38E-01	5,50E-01	Higd1b	0,28	No
ENSMUSG00000020929	9,09E-02	6,114526987	1,97E+00	1,61E-01	5,09E-01	Eftud2	1,07	No
ENSMUSG00000020930	1,87E+00	-3,107639691	1,38E+00	2,40E-01	5,50E-01	Ccdc103	3,67	No
ENSMUSG00000020932	-7,37E-02	-1,37832256	2,03E-02	8,87E-01	1,00E+00	Gfap	0,95	No
ENSMUSG00000020935	1,68E-01	4,002546075	3,62E+00	5,72E-02	2,94E-01	Dcakd	1,12	No
ENSMUSG00000020936	-2,93E-02	6,90028344	2,30E-01	6,31E-01	8,64E-01	Nmt1	0,98	No
ENSMUSG00000020937	2,22E-01	-0,831916736	2,93E-01	5,88E-01	8,40E-01	Plcd3	1,17	No
ENSMUSG00000020940	-9,31E-01	-1,654960623	2,14E+00	1,44E-01	4,79E-01	1700023F06Rik	0,52	No
ENSMUSG00000020941	-4,14E-01	7,135754843	4,75E+01	5,59E-12	6,25E-10	Map3k14	0,75	Yes
ENSMUSG00000020946	-6,50E-02	7,02701389	1,15E+00	2,83E-01	5,79E-01	Gosr2	0,96	No
ENSMUSG00000020948	-3,01E-03	4,754552439	1,58E-03	9,68E-01	1,00E+00	Klhl28	1,00	No
ENSMUSG00000020949	6,11E-03	4,209310854	5,18E-03	9,43E-01	1,00E+00	Fkbp3	1,00	No

ENSMUSG00000020952	2,40E-02	3,449346017	5,66E-02	8,12E-01	9,67E-01	Scfd1	1,02	No
ENSMUSG00000020953	-1,78E+00	-0,78534219	2,24E+01	2,26E-06	9,02E-05	Coch	0,29	Yes
ENSMUSG00000020954	5,62E-02	5,225147548	6,14E-01	4,33E-01	7,27E-01	Strn3	1,04	No
ENSMUSG00000020955	-1,12E-01	3,794241656	1,47E+00	2,26E-01	5,50E-01	Ap4s1	0,93	No
ENSMUSG00000020956	9,67E-02	3,201773239	8,23E-01	3,64E-01	6,65E-01	Dtd2	1,07	No
ENSMUSG00000020961	-1,54E-01	5,097368839	4,44E+00	3,51E-02	2,22E-01	Ston2	0,90	No
ENSMUSG00000020962	1,27E-01	5,4174378	3,35E+00	6,73E-02	3,26E-01	Gtf2a1	1,09	No
ENSMUSG00000020963	-5,87E-01	-2,832045155	2,71E-01	6,03E-01	8,48E-01	Tshr	0,67	No
ENSMUSG00000020964	2,14E-02	7,99878818	1,36E-01	7,13E-01	9,11E-01	Sel1l	1,01	No
ENSMUSG00000020973	1,95E-01	2,798799986	2,74E+00	9,78E-02	3,90E-01	Dnaaf2	1,15	No
ENSMUSG00000020974	1,17E-01	1,966133509	5,93E-01	4,41E-01	7,33E-01	Pole2	1,08	No
ENSMUSG00000020978	2,56E-02	4,098318874	8,45E-02	7,71E-01	9,43E-01	Klhdc2	1,02	No
ENSMUSG00000020982	-3,04E-02	5,773434946	2,06E-01	6,50E-01	8,76E-01	Nemf	0,98	No
ENSMUSG00000020986	1,16E-01	4,539738782	2,13E+00	1,45E-01	4,81E-01	Sec23a	1,08	No
ENSMUSG00000020988	6,83E-02	2,1524019	2,30E-01	6,32E-01	8,64E-01	L2hgdh	1,05	No
ENSMUSG00000020990	1,15E+00	-3,070184988	6,40E-01	4,24E-01	7,18E-01	Cdkl1	2,22	No
ENSMUSG00000020992	7,54E-02	-0,614101319	4,46E-02	8,33E-01	9,78E-01	4930512B01Rik	1,05	No
ENSMUSG00000020993	-9,85E-02	4,765568558	1,65E+00	2,00E-01	5,50E-01	Trappc6b	0,93	No
ENSMUSG00000020994	-6,05E-02	6,345864955	9,07E-01	3,41E-01	6,42E-01	Pnn	0,96	No
ENSMUSG00000021000	1,10E-01	6,474884732	3,07E+00	7,98E-02	3,61E-01	Ctage5	1,08	No
ENSMUSG00000021003	-2,96E-02	5,052933376	1,62E-01	6,87E-01	8,98E-01	Galc	0,98	No
ENSMUSG00000021007	3,53E-01	0,794079694	2,37E+00	1,23E-01	4,39E-01	Spata7	1,28	No
ENSMUSG00000021009	3,05E-01	3,073208548	7,60E+00	5,83E-03	6,27E-02	Ptpn21	1,24	No
ENSMUSG00000021010	1,81E+00	-3,10765737	1,28E+00	2,58E-01	5,50E-01	Npas3	3,49	No
ENSMUSG00000021012	-1,08E-02	5,616485063	2,52E-02	8,74E-01	1,00E+00	Zc3h14	0,99	No
ENSMUSG00000021013	3,03E-01	1,41555871	2,90E+00	8,83E-02	3,79E-01	Ttc8	1,23	No
ENSMUSG00000021018	6,50E-02	2,815542154	3,06E-01	5,80E-01	8,37E-01	Polr2h	1,05	No
ENSMUSG00000021022	-5,80E-02	4,877410626	5,94E-01	4,41E-01	7,33E-01	Ppp2r3c	0,96	No
ENSMUSG00000021023	1,13E-01	3,44266407	1,24E+00	2,65E-01	5,61E-01	1110008L16Rik	1,08	No
ENSMUSG00000021024	-1,62E-01	6,609194753	6,79E+00	9,17E-03	8,76E-02	Psma6	0,89	No
ENSMUSG00000021025	-4,39E-01	7,408991063	5,43E+01	1,69E-13	2,27E-11	Nfkbia	0,74	Yes
ENSMUSG00000021027	-6,64E-02	6,450639488	1,11E+00	2,91E-01	5,87E-01	Ralgapa1	0,96	No

ENSMUSG00000021028	-8,40E-02	3,845517045	8,27E-01	3,63E-01	6,64E-01	Mbip	0,94	No
ENSMUSG00000021032	8,66E-01	-2,226246734	1,15E+00	2,83E-01	5,79E-01	Ngb	1,82	No
ENSMUSG00000021033	8,89E-02	3,447855872	7,83E-01	3,76E-01	6,77E-01	Gstz1	1,06	No
ENSMUSG00000021036	4,44E-02	6,781509927	5,21E-01	4,70E-01	7,59E-01	Sptlc2	1,03	No
ENSMUSG00000021037	8,52E-02	5,850948543	1,64E+00	2,00E-01	5,50E-01	Ahsa1	1,06	No
ENSMUSG00000021038	1,37E-01	5,135502278	3,55E+00	5,96E-02	3,02E-01	Vipas39	1,10	No
ENSMUSG00000021039	-5,82E-04	5,470840594	7,06E-05	9,93E-01	1,00E+00	Snw1	1,00	No
ENSMUSG00000021040	-6,52E-02	4,39179493	6,42E-01	4,23E-01	7,18E-01	Slirp	0,96	No
ENSMUSG00000021044	-8,24E-03	3,666527849	7,44E-03	9,31E-01	1,00E+00	Adck1	0,99	No
ENSMUSG00000021047	7,07E-01	-2,963242697	2,70E-01	6,03E-01	8,48E-01	Nova1	1,63	No
ENSMUSG00000021048	2,28E-01	5,017451849	9,50E+00	2,05E-03	2,86E-02	Mthfd1	1,17	Yes
ENSMUSG00000021051	-4,19E-02	6,0922167	4,19E-01	5,17E-01	7,98E-01	Ppp2r5e	0,97	No
ENSMUSG00000021054	2,51E-01	6,443825333	1,59E+01	6,85E-05	1,76E-03	Sgpp1	1,19	Yes
ENSMUSG00000021055	3,15E+00	-2,896141349	4,06E+00	4,39E-02	2,50E-01	Esr2	8,91	No
ENSMUSG00000021057	-6,18E-01	2,41483041	2,29E+01	1,74E-06	7,18E-05	Akap5	0,65	Yes
ENSMUSG00000021061	-7,70E-02	2,044218073	2,63E-01	6,08E-01	8,51E-01	Sptb	0,95	No
ENSMUSG00000021062	3,21E-01	2,419032089	5,62E+00	1,77E-02	1,39E-01	Rab15	1,25	No
ENSMUSG00000021065	-1,31E-01	4,467321696	2,65E+00	1,03E-01	3,95E-01	Fut8	0,91	No
ENSMUSG00000021066	8,24E-02	5,316084315	1,35E+00	2,45E-01	5,50E-01	Atl1	1,06	No
ENSMUSG00000021067	1,19E-01	7,159901022	3,87E+00	4,91E-02	2,67E-01	Sav1	1,09	No
ENSMUSG00000021068	2,96E-02	7,476681141	2,49E-01	6,18E-01	8,55E-01	Nin	1,02	No
ENSMUSG00000021069	1,26E-01	7,521765383	4,50E+00	3,39E-02	2,17E-01	Pygl	1,09	No
ENSMUSG00000021070	-8,16E-01	-0,641359214	4,59E+00	3,21E-02	2,08E-01	Bdkrb2	0,57	No
ENSMUSG00000021071	-7,95E-02	-2,333214728	7,22E-03	9,32E-01	1,00E+00	Trim9	0,95	No
ENSMUSG00000021072	6,00E-02	6,323547698	8,88E-01	3,46E-01	6,47E-01	Tmx1	1,04	No
ENSMUSG00000021076	-4,36E-02	4,815369266	3,23E-01	5,70E-01	8,31E-01	Actr10	0,97	No
ENSMUSG00000021078	-1,71E-01	-2,126718075	5,25E-02	8,19E-01	9,71E-01	Tomm20l	0,89	No
ENSMUSG00000021079	1,49E-01	3,327447031	2,06E+00	1,52E-01	4,94E-01	Timm9	1,11	No
ENSMUSG00000021086	1,30E-01	0,111762881	2,11E-01	6,46E-01	8,74E-01	Ccdc175	1,09	No
ENSMUSG00000021087	3,11E-02	4,309821386	1,39E-01	7,09E-01	9,09E-01	Rtn1	1,02	No
ENSMUSG00000021090	-2,69E+00	-2,963260181	2,87E+00	9,01E-02	3,80E-01	Lrrc9	0,16	No
ENSMUSG00000021091	7,56E-01	-2,963268003	3,13E-01	5,76E-01	8,34E-01	Serpina3n	1,69	No

ENSMUSG00000021094	-5,22E-02	4,742733612	4,51E-01	5,02E-01	7,87E-01	Dhrs7		0,96	No
ENSMUSG00000021095	1,47E+00	-1,033774163	1,10E+01	9,24E-04	1,51E-02	Gsc		2,77	Yes
ENSMUSG00000021096	-1,16E-02	6,404727192	3,38E-02	8,54E-01	9,92E-01	Ppm1a		0,99	No
ENSMUSG00000021097	-9,69E-02	3,177665834	8,12E-01	3,67E-01	6,69E-01	Clmn		0,94	No
ENSMUSG00000021098	1,27E+00	-2,863717917	1,00E+00	3,16E-01	6,17E-01	4930447C04Rik		2,41	No
ENSMUSG00000021099	2,06E-02	-3,146103739	-9,26E-10	1,00E+00	1,00E+00	Six6		1,01	No
ENSMUSG00000021101	3,23E-01	-2,711770618	1,06E-01	7,44E-01	9,29E-01	4930408O17Rik		1,25	No
ENSMUSG00000021102	1,02E-01	5,308399787	2,05E+00	1,52E-01	4,94E-01	Glrx5		1,07	No
ENSMUSG00000021103	-7,60E-02	3,954274145	7,12E-01	3,99E-01	6,98E-01	Mnat1		0,95	No
ENSMUSG00000021108	2,35E-01	4,220014677	7,47E+00	6,29E-03	6,65E-02	Prkch		1,18	No
ENSMUSG00000021109	-7,59E-02	7,728231908	1,66E+00	1,97E-01	5,50E-01	Hif1a		0,95	No
ENSMUSG00000021111	1,40E-01	7,174734843	5,43E+00	1,98E-02	1,50E-01	Papola		1,10	No
ENSMUSG00000021112	-2,43E-03	4,613121782	9,72E-04	9,75E-01	1,00E+00	Mpp5		1,00	No
ENSMUSG00000021113	-1,66E-02	4,019990188	3,54E-02	8,51E-01	9,90E-01	Snapc1		0,99	No
ENSMUSG00000021114	-1,52E-02	6,028785505	5,39E-02	8,16E-01	9,69E-01	Atp6v1d		0,99	No
ENSMUSG00000021115	-2,61E-03	6,834949906	1,81E-03	9,66E-01	1,00E+00	Vrk1		1,00	No
ENSMUSG00000021116	-4,94E-02	5,827221104	5,51E-01	4,58E-01	7,47E-01	Eif2s1		0,97	No
ENSMUSG00000021118	-1,21E+00	2,53551166	9,39E+01	3,26E-22	9,23E-20	Plek2		0,43	Yes
ENSMUSG00000021120	-6,57E-03	2,655025643	2,91E-03	9,57E-01	1,00E+00	Pigh		1,00	No
ENSMUSG00000021123	-1,01E+00	-0,116906546	1,14E+01	7,52E-04	1,28E-02	Rdh12		0,50	Yes
ENSMUSG00000021124	8,36E-02	5,901835256	1,60E+00	2,06E-01	5,50E-01	Vti1b		1,06	No
ENSMUSG00000021125	1,71E-02	1,620223339	9,97E-03	9,20E-01	1,00E+00	Arg2		1,01	No
ENSMUSG00000021127	-2,14E-01	7,73909873	1,33E+01	2,66E-04	5,50E-03	Zfp36l1		0,86	Yes
ENSMUSG00000021130	-1,92E+00	-3,0336536	1,46E+00	2,27E-01	5,50E-01	Galnt16		0,26	No
ENSMUSG00000021131	-5,81E-02	5,204289331	6,57E-01	4,18E-01	7,12E-01	Erh		0,96	No
ENSMUSG00000021133	2,42E-02	7,372162394	1,65E-01	6,85E-01	8,96E-01	Susd6		1,02	No
ENSMUSG00000021134	3,45E-02	7,243407976	3,32E-01	5,65E-01	8,27E-01	Srsf5		1,02	No
ENSMUSG00000021135	1,08E+00	-1,574218343	3,18E+00	7,47E-02	3,47E-01	Slc10a1		2,12	No
ENSMUSG00000021136	7,20E-01	0,704975643	8,16E+00	4,28E-03	4,97E-02	Smoc1		1,65	Yes
ENSMUSG00000021139	6,24E-02	2,879397884	2,86E-01	5,93E-01	8,42E-01	Gm20498		1,04	No
ENSMUSG00000021140	-8,32E-02	5,774855912	1,54E+00	2,14E-01	5,50E-01	Pcnx		0,94	No
ENSMUSG00000021143	1,47E-01	7,446669383	6,10E+00	1,35E-02	1,17E-01	Pacs2		1,11	No

ENSMUSG00000021144	-1,08E-02	5,278140929	2,31E-02	8,79E-01	1,00E+00	Mta1	0,99	No
ENSMUSG00000021147	-5,52E-02	5,76505412	6,85E-01	4,08E-01	7,04E-01	Wdr37	0,96	No
ENSMUSG00000021148	-7,54E-01	-1,831961184	1,49E+00	2,22E-01	5,50E-01	Gm9745	0,59	No
ENSMUSG00000021149	-2,73E-01	6,164535553	1,80E+01	2,24E-05	6,87E-04	Gtpbp4	0,83	Yes
ENSMUSG00000021156	-8,09E-02	6,053326645	1,55E+00	2,14E-01	5,50E-01	Zmynd11	0,95	No
ENSMUSG00000021171	8,83E-02	7,222656007	2,16E+00	1,41E-01	4,75E-01	Esyt2	1,06	No
ENSMUSG00000021175	1,72E-01	3,955182797	3,63E+00	5,67E-02	2,93E-01	Cdca7l	1,13	No
ENSMUSG00000021176	-1,38E-01	1,661211909	6,70E-01	4,13E-01	7,09E-01	Efcab11	0,91	No
ENSMUSG00000021177	4,82E-02	3,2088097	2,07E-01	6,49E-01	8,75E-01	Tdp1	1,03	No
ENSMUSG00000021178	-6,31E-02	5,548926573	8,38E-01	3,60E-01	6,61E-01	Psmc1	0,96	No
ENSMUSG00000021179	-1,32E-01	4,877550956	3,08E+00	7,93E-02	3,60E-01	Nrde2	0,91	No
ENSMUSG00000021180	-1,41E-01	4,541794577	3,12E+00	7,76E-02	3,56E-01	Rps6ka5	0,91	No
ENSMUSG00000021182	-2,40E-01	5,715397541	1,27E+01	3,66E-04	7,11E-03	Ccdc88c	0,85	Yes
ENSMUSG00000021185	-1,72E-02	5,872388146	6,74E-02	7,95E-01	9,57E-01	9030617O03Rik	0,99	No
ENSMUSG00000021186	-2,72E-01	-1,600650127	2,60E-01	6,10E-01	8,51E-01	Fbln5	0,83	No
ENSMUSG00000021187	-4,64E-01	-2,711695019	2,21E-01	6,39E-01	8,69E-01	Tc2n	0,73	No
ENSMUSG00000021188	6,84E-02	6,333865078	1,16E+00	2,81E-01	5,78E-01	Trip11	1,05	No
ENSMUSG00000021189	1,10E-01	6,050648382	2,84E+00	9,20E-02	3,82E-01	Atxn3	1,08	No
ENSMUSG00000021190	1,07E-01	8,243438784	3,44E+00	6,36E-02	3,15E-01	Lgmn	1,08	No
ENSMUSG00000021192	4,24E-03	5,720721025	3,96E-03	9,50E-01	1,00E+00	Golga5	1,00	No
ENSMUSG00000021193	7,87E-02	5,344172015	1,25E+00	2,64E-01	5,58E-01	Pitrm1	1,06	No
ENSMUSG00000021194	4,01E-01	-2,831991084	1,24E-01	7,25E-01	9,18E-01	Chga	1,32	No
ENSMUSG00000021196	-6,92E-02	9,342100217	1,49E+00	2,23E-01	5,50E-01	Pfkp	0,95	No
ENSMUSG00000021198	-1,89E+00	-3,033648118	1,42E+00	2,33E-01	5,50E-01	Unc79	0,27	No
ENSMUSG00000021200	-1,01E+00	5,214174736	1,97E+02	9,81E-45	1,63E-41	Asb2	0,50	Yes
ENSMUSG00000021203	6,17E-02	1,16399364	9,99E-02	7,52E-01	9,32E-01	Otub2	1,04	No
ENSMUSG00000021207	-1,36E+00	-2,770613073	1,16E+00	2,81E-01	5,78E-01	Akr1c21	0,39	No
ENSMUSG00000021208	-1,87E+00	-3,107637761	1,39E+00	2,38E-01	5,50E-01	Ifi27l2b	0,27	No
ENSMUSG00000021209	-2,66E-01	-2,268107485	1,23E-01	7,26E-01	9,19E-01	Ppp4r4	0,83	No
ENSMUSG00000021211	9,22E-02	1,091409727	2,18E-01	6,41E-01	8,70E-01	Akr1c12	1,07	No
ENSMUSG00000021213	-2,92E-02	3,354578934	8,00E-02	7,77E-01	9,47E-01	Akr1c13	0,98	No
ENSMUSG00000021214	-4,54E-01	-2,185585694	3,80E-01	5,38E-01	8,09E-01	Akr1c18	0,73	No

ENSMUSG00000021215	-5,13E-01	6,776288212	6,98E+01	6,70E-17	1,20E-14	Net1	0,70	Yes
ENSMUSG00000021217	-1,74E-01	-0,816397956	1,84E-01	6,68E-01	8,87E-01	Tshz3	0,89	No
ENSMUSG00000021218	-1,79E-02	9,13323093	9,98E-02	7,52E-01	9,32E-01	Gdi2	0,99	No
ENSMUSG00000021219	1,04E+00	-2,800939709	6,29E-01	4,28E-01	7,21E-01	Rgs6	2,05	No
ENSMUSG00000021221	-4,49E-01	1,376026009	6,18E+00	1,30E-02	1,13E-01	Dpf3	0,73	No
ENSMUSG00000021222	-1,18E-01	3,538611732	1,45E+00	2,28E-01	5,50E-01	Dcaf4	0,92	No
ENSMUSG00000021223	4,61E-01	-1,600627629	5,85E-01	4,44E-01	7,36E-01	Papln	1,38	No
ENSMUSG00000021224	9,21E-02	4,961645036	1,53E+00	2,17E-01	5,50E-01	Numb	1,07	No
ENSMUSG00000021226	-3,19E-01	3,8282163	1,22E+01	4,90E-04	9,03E-03	Acot2	0,80	Yes
ENSMUSG00000021228	-2,14E+00	-2,42481685	4,70E+00	3,02E-02	1,99E-01	Acot3	0,23	No
ENSMUSG00000021234	9,51E-02	-0,020196367	9,75E-02	7,55E-01	9,34E-01	Fam161b	1,07	No
ENSMUSG00000021235	-4,88E-02	3,343863204	2,17E-01	6,41E-01	8,71E-01	Coq6	0,97	No
ENSMUSG00000021236	8,31E-02	4,616429022	1,10E+00	2,94E-01	5,89E-01	Entpd5	1,06	No
ENSMUSG00000021238	1,60E-01	3,828173035	2,99E+00	8,35E-02	3,71E-01	Aldh6a1	1,12	No
ENSMUSG00000021239	1,87E+00	-3,107639691	1,38E+00	2,40E-01	5,50E-01	Vsx2	3,67	No
ENSMUSG00000021240	2,29E-01	3,680694079	5,79E+00	1,61E-02	1,32E-01	Abcd4	1,17	No
ENSMUSG00000021241	-1,76E-02	4,33803597	4,49E-02	8,32E-01	9,78E-01	Isca2	0,99	No
ENSMUSG00000021242	4,61E-02	9,213943802	6,61E-01	4,16E-01	7,11E-01	Npc2	1,03	No
ENSMUSG00000021243	-2,42E-01	2,26775851	2,94E+00	8,65E-02	3,78E-01	Fcf1	0,85	No
ENSMUSG00000021244	-3,52E-02	6,222386676	3,02E-01	5,83E-01	8,38E-01	Ylpm1	0,98	No
ENSMUSG00000021245	1,01E-01	3,988060317	1,32E+00	2,50E-01	5,50E-01	Mlh3	1,07	No
ENSMUSG00000021248	1,09E-01	7,599721567	3,39E+00	6,56E-02	3,21E-01	Tmed10	1,08	No
ENSMUSG00000021250	7,80E-01	6,531780023	1,49E+02	3,00E-34	2,19E-31	Fos	1,72	Yes
ENSMUSG00000021252	1,00E-01	4,985977683	1,83E+00	1,76E-01	5,34E-01	Erg28	1,07	No
ENSMUSG00000021253	-8,58E-01	1,362754516	2,15E+01	3,54E-06	1,35E-04	Tgfb3	0,55	Yes
ENSMUSG00000021254	-6,94E-03	5,560012743	1,03E-02	9,19E-01	1,00E+00	Gpatch2l	1,00	No
ENSMUSG00000021255	1,81E+00	-3,10765737	1,28E+00	2,58E-01	5,50E-01	Esrrb	3,49	No
ENSMUSG00000021256	2,68E-01	3,239456871	6,38E+00	1,15E-02	1,04E-01	Vash1	1,20	No
ENSMUSG00000021257	2,36E-01	2,65995287	3,65E+00	5,61E-02	2,90E-01	Angel1	1,18	No
ENSMUSG00000021258	-2,14E-02	5,467292856	9,55E-02	7,57E-01	9,35E-01	Ccnk	0,99	No
ENSMUSG00000021259	3,16E+00	-2,963281728	4,07E+00	4,36E-02	2,49E-01	Cyp46a1	8,92	No
ENSMUSG00000021260	-5,17E-02	-2,497606716	3,05E-03	9,56E-01	1,00E+00	Hhipl1	0,96	No

ENSMUSG00000021262	3,02E-01	6,236727713	2,22E+01	2,51E-06	9,85E-05	Evl	1,23	Yes
ENSMUSG00000021263	2,49E-01	3,602292417	6,66E+00	9,87E-03	9,24E-02	Degs2	1,19	No
ENSMUSG00000021264	-2,14E-02	6,656477406	1,20E-01	7,29E-01	9,20E-01	Yy1	0,99	No
ENSMUSG00000021265	-7,55E-01	2,935820931	4,67E+01	8,31E-12	8,86E-10	Slc25a29	0,59	Yes
ENSMUSG00000021266	-1,53E-01	6,097340223	5,61E+00	1,79E-02	1,40E-01	Wars	0,90	No
ENSMUSG00000021268	-3,88E-03	-3,03362684	4,02E-12	1,00E+00	1,00E+00	Meg3	1,00	No
ENSMUSG00000021270	3,35E-02	6,727729014	2,96E-01	5,86E-01	8,40E-01	Hsp90aa1	1,02	No
ENSMUSG00000021271	1,82E-01	3,811533825	3,99E+00	4,56E-02	2,55E-01	Zfp839	1,13	No
ENSMUSG00000021273	1,73E-01	5,475626249	6,22E+00	1,26E-02	1,11E-01	Fdft1	1,13	No
ENSMUSG00000021275	1,21E-01	4,533877998	2,29E+00	1,30E-01	4,53E-01	Tecpr2	1,09	No
ENSMUSG00000021276	6,83E-02	4,588103414	7,40E-01	3,90E-01	6,90E-01	Cinp	1,05	No
ENSMUSG00000021277	-2,15E-01	6,044236937	1,09E+01	9,59E-04	1,56E-02	Traf3	0,86	Yes
ENSMUSG00000021278	3,23E-01	-1,35565587	4,72E-01	4,92E-01	7,78E-01	Amn	1,25	No
ENSMUSG00000021279	-5,53E-02	6,781934985	8,08E-01	3,69E-01	6,70E-01	Cdc42bpb	0,96	No
ENSMUSG00000021280	-6,88E-01	3,479521277	4,98E+01	1,69E-12	2,00E-10	Exoc3l4	0,62	Yes
ENSMUSG00000021281	-1,72E-01	8,539272141	8,98E+00	2,72E-03	3,55E-02	Tnfaip2	0,89	Yes
ENSMUSG00000021282	2,90E-02	8,463053294	2,54E-01	6,14E-01	8,53E-01	Eif5	1,02	No
ENSMUSG00000021285	2,33E-01	5,110567236	1,04E+01	1,28E-03	1,98E-02	Ppp1r13b	1,18	Yes
ENSMUSG00000021286	2,26E-01	3,330652148	4,66E+00	3,10E-02	2,03E-01	Zfyve21	1,17	No
ENSMUSG00000021287	3,99E-02	4,053242493	2,07E-01	6,49E-01	8,75E-01	Xrcc3	1,03	No
ENSMUSG00000021288	-1,32E-02	6,375109249	4,38E-02	8,34E-01	9,79E-01	Klc1	0,99	No
ENSMUSG00000021290	-1,22E-01	5,481170363	3,09E+00	7,86E-02	3,58E-01	2010107E04Rik	0,92	No
ENSMUSG00000021294	-2,66E+00	-2,896122337	2,83E+00	9,26E-02	3,83E-01	Kif26a	0,16	No
ENSMUSG00000021298	-5,84E-01	7,192664432	9,45E+01	2,45E-22	7,12E-20	Gpr132	0,67	Yes
ENSMUSG00000021301	1,85E+00	-3,107657832	1,35E+00	2,46E-01	5,50E-01	Hecw1	3,61	No
ENSMUSG00000021302	6,40E-02	4,095594266	5,51E-01	4,58E-01	7,47E-01	Ggps1	1,05	No
ENSMUSG00000021303	-4,33E-01	-1,627547441	6,96E-01	4,04E-01	7,01E-01	Gng4	0,74	No
ENSMUSG00000021306	1,31E-01	6,388396572	4,27E+00	3,88E-02	2,33E-01	Gpr137b	1,09	No
ENSMUSG00000021311	1,94E-01	5,464456597	7,76E+00	5,34E-03	5,86E-02	Mtr	1,14	No
ENSMUSG00000021313	-7,20E-01	-2,497566786	5,81E-01	4,46E-01	7,37E-01	Ryr2	0,61	No
ENSMUSG00000021314	-1,87E+00	-3,03364178	1,41E+00	2,36E-01	5,50E-01	Amph	0,27	No
ENSMUSG00000021318	-4,09E-01	-0,311087367	1,69E+00	1,93E-01	5,50E-01	Gli3	0,75	No

ENSMUSG00000021319	-3,45E+00	-2,1855089	1,41E+01	1,74E-04	3,86E-03	Sfrp4	0,09	Yes
ENSMUSG00000021322	2,41E-01	4,819462663	9,83E+00	1,72E-03	2,47E-02	Aoah	1,18	Yes
ENSMUSG00000021326	-1,46E-02	5,318423774	4,31E-02	8,36E-01	9,80E-01	Trim27	0,99	No
ENSMUSG00000021327	-9,26E-02	5,914737529	2,00E+00	1,57E-01	5,03E-01	Zkscan3	0,94	No
ENSMUSG00000021336	-5,88E-02	-3,033652696	1,62E-03	9,68E-01	1,00E+00	Slc17a4	0,96	No
ENSMUSG00000021338	4,04E-02	2,807716357	1,17E-01	7,32E-01	9,22E-01	Carmil1	1,03	No
ENSMUSG00000021339	9,36E-02	5,952225883	2,03E+00	1,54E-01	4,98E-01	Mrs2	1,07	No
ENSMUSG00000021340	5,81E-02	4,07114626	4,44E-01	5,05E-01	7,90E-01	Gpld1	1,04	No
ENSMUSG00000021356	-2,08E-01	8,723642362	1,32E+01	2,84E-04	5,79E-03	Irf4	0,87	Yes
ENSMUSG00000021357	-1,21E-02	6,215871066	3,55E-02	8,51E-01	9,90E-01	Exoc2	0,99	No
ENSMUSG00000021359	2,97E-01	-1,963247285	1,72E-01	6,78E-01	8,92E-01	Tfap2a	1,23	No
ENSMUSG00000021360	5,66E-02	6,115540538	7,56E-01	3,85E-01	6,86E-01	Gcnt2	1,04	No
ENSMUSG00000021361	5,51E-02	6,427667436	7,61E-01	3,83E-01	6,84E-01	Tmem14c	1,04	No
ENSMUSG00000021362	-1,41E-01	-0,548309313	1,23E-01	7,26E-01	9,19E-01	Gcm2	0,91	No
ENSMUSG00000021363	1,96E-01	2,921142674	2,75E+00	9,70E-02	3,88E-01	Mak	1,15	No
ENSMUSG00000021364	-5,56E-01	-2,832021161	2,41E-01	6,23E-01	8,58E-01	Elovl2	0,68	No
ENSMUSG00000021365	1,67E-01	6,091399281	6,54E+00	1,05E-02	9,71E-02	Nedd9	1,12	No
ENSMUSG00000021366	-3,11E-01	7,490351563	2,76E+01	1,49E-07	7,96E-06	Hivep1	0,81	Yes
ENSMUSG00000021367	-6,62E-01	1,188491007	1,01E+01	1,45E-03	2,17E-02	Edn1	0,63	Yes
ENSMUSG00000021368	-2,61E-02	2,926380299	5,22E-02	8,19E-01	9,71E-01	Tbc1d7	0,98	No
ENSMUSG00000021371	1,18E-02	6,339737613	3,47E-02	8,52E-01	9,91E-01	Mcur1	1,01	No
ENSMUSG00000021373	1,05E+00	-1,401443877	3,72E+00	5,37E-02	2,83E-01	Cap2	2,07	No
ENSMUSG00000021374	6,93E-02	7,716833625	1,39E+00	2,38E-01	5,50E-01	Nup153	1,05	No
ENSMUSG00000021375	-5,06E-03	6,128859312	6,08E-03	9,38E-01	1,00E+00	Kif13a	1,00	No
ENSMUSG00000021376	1,71E-01	1,980710024	1,21E+00	2,71E-01	5,68E-01	Tpmt	1,13	No
ENSMUSG00000021377	8,34E-02	7,337664981	1,95E+00	1,62E-01	5,12E-01	Dek	1,06	No
ENSMUSG00000021379	-1,94E+00	-3,107619243	1,50E+00	2,21E-01	5,50E-01	Id4	0,26	No
ENSMUSG00000021384	-6,34E-03	4,911214074	7,18E-03	9,32E-01	1,00E+00	Susd3	1,00	No
ENSMUSG00000021385	2,33E-01	3,299226278	4,94E+00	2,63E-02	1,81E-01	Ippk	1,18	No
ENSMUSG00000021388	8,35E-01	-2,107641426	8,39E-01	3,60E-01	6,61E-01	Aspn	1,78	No
ENSMUSG00000021390	-1,33E+00	-2,600661346	1,11E+00	2,92E-01	5,87E-01	Ogn	0,40	No
ENSMUSG00000021391	1,90E-01	1,193444136	9,28E-01	3,35E-01	6,36E-01	Cenpp	1,14	No

ENSMUSG00000021392	2,06E-01	4,407154172	6,37E+00	1,16E-02	1,04E-01	Nol8	1,15	No
ENSMUSG00000021395	-3,76E-03	5,746339624	3,15E-03	9,55E-01	1,00E+00	Spin1	1,00	No
ENSMUSG00000021396	-2,71E+00	-2,963182239	2,92E+00	8,75E-02	3,78E-01	Nxnl2	0,15	No
ENSMUSG00000021400	-3,16E-02	5,181900424	1,92E-01	6,61E-01	8,82E-01	Wrnip1	0,98	No
ENSMUSG00000021403	-3,75E-01	3,595008599	1,53E+01	9,17E-05	2,26E-03	Serpinb9b	0,77	Yes
ENSMUSG00000021408	-9,65E-03	6,885004606	2,49E-02	8,75E-01	1,00E+00	Ripk1	0,99	No
ENSMUSG00000021411	7,09E-02	4,530466845	7,61E-01	3,83E-01	6,84E-01	Pxdc1	1,05	No
ENSMUSG00000021413	1,49E-02	6,990213898	6,02E-02	8,06E-01	9,64E-01	Prpf4b	1,01	No
ENSMUSG00000021414	-4,09E+00	-2,311069297	8,48E+00	3,60E-03	4,38E-02	Fam217a	0,06	Yes
ENSMUSG00000021415	-4,86E-01	-2,711728345	2,13E-01	6,44E-01	8,72E-01	4933417A18Rik	0,71	No
ENSMUSG00000021416	-2,48E-01	0,385887765	9,62E-01	3,27E-01	6,28E-01	Eci3	0,84	No
ENSMUSG00000021417	2,49E-04	5,56742538	1,32E-05	9,97E-01	1,00E+00	Eci2	1,00	No
ENSMUSG00000021418	-4,69E-02	1,425967264	6,85E-02	7,93E-01	9,56E-01	Rpp40	0,97	No
ENSMUSG00000021420	1,43E-01	4,010979297	2,59E+00	1,08E-01	4,02E-01	Fars2	1,10	No
ENSMUSG00000021423	7,50E-02	6,329504221	1,38E+00	2,40E-01	5,50E-01	Ly86	1,05	No
ENSMUSG00000021427	9,02E-02	7,927042247	2,39E+00	1,22E-01	4,37E-01	Ssr1	1,06	No
ENSMUSG00000021428	-1,38E-01	4,819563656	3,23E+00	7,24E-02	3,41E-01	Riok1	0,91	No
ENSMUSG00000021431	-7,60E-02	5,162392742	1,11E+00	2,92E-01	5,88E-01	Snrnp48	0,95	No
ENSMUSG00000021432	1,79E-02	5,838888917	7,18E-02	7,89E-01	9,54E-01	Slc35b3	1,01	No
ENSMUSG00000021448	-8,94E-01	-2,83199835	4,38E-01	5,08E-01	7,92E-01	Shc3	0,54	No
ENSMUSG00000021451	1,22E-01	7,69552918	4,33E+00	3,74E-02	2,30E-01	Sema4d	1,09	No
ENSMUSG00000021453	3,56E-02	4,852998838	2,16E-01	6,42E-01	8,71E-01	Gadd45g	1,03	No
ENSMUSG00000021457	-1,10E-01	8,794571834	3,68E+00	5,51E-02	2,88E-01	Syk	0,93	No
ENSMUSG00000021458	1,91E-01	5,775030499	8,02E+00	4,62E-03	5,26E-02	2010111I01Rik	1,14	No
ENSMUSG00000021460	-3,46E-01	5,056970207	2,26E+01	2,02E-06	8,18E-05	Auh	0,79	Yes
ENSMUSG00000021461	2,25E-02	3,52395394	5,22E-02	8,19E-01	9,71E-01	Fancc	1,02	No
ENSMUSG00000021464	9,01E-03	-3,107646452	-5,16E-10	1,00E+00	1,00E+00	Ror2	1,01	No
ENSMUSG00000021466	1,59E-01	4,525229318	3,93E+00	4,75E-02	2,61E-01	Ptch1	1,12	No
ENSMUSG00000021468	7,39E-02	5,452470774	1,12E+00	2,89E-01	5,85E-01	Sptlc1	1,05	No
ENSMUSG00000021470	1,34E-01	4,774239633	3,03E+00	8,16E-02	3,67E-01	Ercc6l2	1,10	No
ENSMUSG00000021474	5,24E-02	5,722397318	5,98E-01	4,39E-01	7,32E-01	Sfxn1	1,04	No
ENSMUSG00000021476	8,54E-02	4,110264978	9,64E-01	3,26E-01	6,27E-01	Habp4	1,06	No

ENSMUSG00000021477	-1,37E-01	8,469536807	5,65E+00	1,75E-02	1,38E-01	Ctsl	0,91	No
ENSMUSG00000021478	2,20E-01	-1,070137339	2,37E-01	6,26E-01	8,60E-01	Drd1	1,16	No
ENSMUSG00000021481	4,88E-02	3,915593185	3,06E-01	5,80E-01	8,37E-01	Zfp346	1,03	No
ENSMUSG00000021482	1,32E-01	4,850812489	3,04E+00	8,11E-02	3,66E-01	Aaed1	1,10	No
ENSMUSG00000021483	2,29E-01	3,258436369	4,76E+00	2,91E-02	1,94E-01	Cdk20	1,17	No
ENSMUSG00000021484	1,12E-01	7,771810256	3,65E+00	5,60E-02	2,90E-01	Lman2	1,08	No
ENSMUSG00000021485	6,49E-02	4,86803243	7,38E-01	3,90E-01	6,91E-01	Mxd3	1,05	No
ENSMUSG00000021486	6,56E-02	7,485306956	1,22E+00	2,69E-01	5,65E-01	Prelid1	1,05	No
ENSMUSG00000021488	-7,61E-02	7,609410064	1,66E+00	1,97E-01	5,50E-01	Nsd1	0,95	No
ENSMUSG00000021490	2,63E+00	-2,497532259	2,68E+00	1,02E-01	3,94E-01	Slc34a1	6,20	No
ENSMUSG00000021492	-1,87E+00	-3,03364178	1,41E+00	2,36E-01	5,50E-01	F12	0,27	No
ENSMUSG00000021493	1,88E-01	4,819979901	6,02E+00	1,41E-02	1,21E-01	Pdlim7	1,14	No
ENSMUSG00000021494	2,61E-02	5,128174662	1,29E-01	7,20E-01	9,15E-01	Ddx41	1,02	No
ENSMUSG00000021495	-6,61E-02	6,037185562	1,04E+00	3,08E-01	6,06E-01	Fam193b	0,96	No
ENSMUSG00000021496	-1,41E-01	3,378806713	1,92E+00	1,66E-01	5,18E-01	Pcbd2	0,91	No
ENSMUSG00000021497	-1,19E-02	5,572988137	2,99E-02	8,63E-01	9,97E-01	Txndc15	0,99	No
ENSMUSG00000021499	-3,57E-02	-2,711633193	8,91E-04	9,76E-01	1,00E+00	Catsper3	0,98	No
ENSMUSG00000021500	4,20E-02	6,887833646	4,74E-01	4,91E-01	7,77E-01	Ddx46	1,03	No
ENSMUSG00000021501	-5,57E-02	4,541449118	4,95E-01	4,82E-01	7,69E-01	Caml	0,96	No
ENSMUSG00000021504	2,26E-01	4,8762915	8,91E+00	2,84E-03	3,67E-02	B4galt7	1,17	Yes
ENSMUSG00000021506	-7,47E-02	-2,033711201	1,03E-02	9,19E-01	1,00E+00	Pitx1	0,95	No
ENSMUSG00000021508	-1,73E-01	0,08752771	3,99E-01	5,27E-01	8,04E-01	Cxcl14	0,89	No
ENSMUSG00000021509	1,82E+00	-3,033667329	1,31E+00	2,52E-01	5,50E-01	Slc25a48	3,54	No
ENSMUSG00000021510	1,71E-01	4,185006115	4,05E+00	4,41E-02	2,50E-01	Zfp729a	1,13	No
ENSMUSG00000021514	1,65E-02	4,491586204	4,26E-02	8,36E-01	9,81E-01	Zfp369	1,01	No
ENSMUSG00000021518	5,79E-02	6,113651031	7,98E-01	3,72E-01	6,73E-01	Ptdss1	1,04	No
ENSMUSG00000021519	3,50E-02	4,573371577	1,93E-01	6,61E-01	8,82E-01	Mterf3	1,02	No
ENSMUSG00000021520	3,25E-02	4,284544423	1,50E-01	6,99E-01	9,04E-01	Uqcrb	1,02	No
ENSMUSG00000021532	1,58E-01	3,678593519	2,79E+00	9,50E-02	3,86E-01	Fastkd3	1,12	No
ENSMUSG00000021534	9,01E-03	-3,107646452	-5,16E-10	1,00E+00	1,00E+00	1700001L19Rik	1,01	No
ENSMUSG00000021536	-3,18E+00	-2,896135562	4,24E+00	3,94E-02	2,35E-01	Adcy2	0,11	No
ENSMUSG00000021537	1,92E-02	5,117278172	7,00E-02	7,91E-01	9,55E-01	Cetn3	1,01	No

ENSMUSG00000021539	1,87E+00	-3,107639691	1,38E+00	2,40E-01	5,50E-01	Lect2	3,67	No
ENSMUSG00000021540	4,66E-02	4,538103466	3,46E-01	5,56E-01	8,21E-01	Smad5	1,03	No
ENSMUSG00000021546	5,26E-03	7,948190503	8,14E-03	9,28E-01	1,00E+00	Hnrnpk	1,00	No
ENSMUSG00000021548	2,09E-01	3,696303121	4,78E+00	2,88E-02	1,93E-01	Ccnh	1,16	No
ENSMUSG00000021549	1,36E-01	5,638858843	4,00E+00	4,54E-02	2,54E-01	Rasa1	1,10	No
ENSMUSG00000021550	5,79E-02	5,523132656	7,00E-01	4,03E-01	7,01E-01	2210016F16Rik	1,04	No
ENSMUSG00000021552	7,66E-02	2,513421479	3,45E-01	5,57E-01	8,21E-01	Gkap1	1,05	No
ENSMUSG00000021553	-1,66E-01	-0,020032211	2,96E-01	5,87E-01	8,40E-01	Slc28a3	0,89	No
ENSMUSG00000021555	5,17E-02	4,915415864	4,79E-01	4,89E-01	7,76E-01	Naa35	1,04	No
ENSMUSG00000021556	1,47E-01	5,620831864	4,66E+00	3,09E-02	2,03E-01	Golm1	1,11	No
ENSMUSG00000021557	-1,39E-02	5,270221161	3,78E-02	8,46E-01	9,87E-01	Agtpbp1	0,99	No
ENSMUSG00000021559	8,23E-02	7,301860644	1,89E+00	1,69E-01	5,24E-01	Dapk1	1,06	No
ENSMUSG00000021565	-2,95E-01	-0,785532637	5,45E-01	4,60E-01	7,49E-01	Slc6a19	0,82	No
ENSMUSG00000021567	-2,67E+00	-2,831936341	2,83E+00	9,22E-02	3,82E-01	Nkd2	0,16	No
ENSMUSG00000021569	8,60E-02	3,629175466	7,97E-01	3,72E-01	6,73E-01	Trip13	1,06	No
ENSMUSG00000021572	1,30E-01	3,16546193	1,43E+00	2,31E-01	5,50E-01	Cep72	1,09	No
ENSMUSG00000021573	-7,54E-02	-2,497623035	6,53E-03	9,36E-01	1,00E+00	Tppp	0,95	No
ENSMUSG00000021575	-3,32E-01	6,451778616	2,80E+01	1,24E-07	6,77E-06	Ahrr	0,79	Yes
ENSMUSG00000021576	5,51E-02	6,394052495	7,61E-01	3,83E-01	6,85E-01	Pdcd6	1,04	No
ENSMUSG00000021577	-1,27E-02	7,895137533	4,76E-02	8,27E-01	9,76E-01	Sdha	0,99	No
ENSMUSG00000021578	5,90E-02	4,950041675	6,35E-01	4,25E-01	7,20E-01	Ccdc127	1,04	No
ENSMUSG00000021579	-1,85E-02	1,163917772	9,20E-03	9,24E-01	1,00E+00	Lrrc14b	0,99	No
ENSMUSG00000021583	8,49E-03	6,627226516	1,87E-02	8,91E-01	1,00E+00	Erap1	1,01	No
ENSMUSG00000021585	7,54E-02	6,250656463	1,39E+00	2,39E-01	5,50E-01	Cast	1,05	No
ENSMUSG00000021587	-3,83E-02	-3,107651936	-7,91E-11	1,00E+00	1,00E+00	Pcsk1	0,97	No
ENSMUSG00000021589	-1,03E+00	-1,711732385	2,34E+00	1,26E-01	4,44E-01	Rhobtb3	0,49	No
ENSMUSG00000021590	-1,23E+00	-2,600642831	1,46E+00	2,27E-01	5,50E-01	Spata9	0,43	No
ENSMUSG00000021591	1,27E-01	6,567053298	4,09E+00	4,32E-02	2,47E-01	Glrx	1,09	No
ENSMUSG00000021592	2,29E-01	4,217867763	7,32E+00	6,82E-03	7,04E-02	Arsk	1,17	No
ENSMUSG00000021594	-1,47E-01	1,582390917	6,98E-01	4,03E-01	7,01E-01	Srd5a1	0,90	No
ENSMUSG00000021595	1,14E-01	6,270463023	3,20E+00	7,38E-02	3,45E-01	Nsun2	1,08	No
ENSMUSG00000021596	1,59E-01	4,296293318	3,55E+00	5,94E-02	3,02E-01	Mctp1	1,12	No

ENSMUSG00000021597	2,43E-01	3,476325051	5,85E+00	1,56E-02	1,29E-01	Slf1	1,18	No
ENSMUSG00000021598	-1,50E-01	4,207907214	3,11E+00	7,76E-02	3,56E-01	Med10	0,90	No
ENSMUSG00000021606	-1,04E-01	3,220805892	9,39E-01	3,32E-01	6,33E-01	Ndufs6	0,93	No
ENSMUSG00000021607	1,55E-01	4,957856593	4,29E+00	3,82E-02	2,32E-01	Mrpl36	1,11	No
ENSMUSG00000021608	-2,30E-01	6,246397028	1,31E+01	3,02E-04	6,09E-03	Lpcat1	0,85	Yes
ENSMUSG00000021609	3,23E-01	-1,770663598	2,78E-01	5,98E-01	8,45E-01	Slc6a3	1,25	No
ENSMUSG00000021610	7,17E-02	6,786470645	1,36E+00	2,44E-01	5,50E-01	Clptm1l	1,05	No
ENSMUSG00000021611	-1,48E+00	-1,574172298	7,36E+00	6,66E-03	6,93E-02	Tert	0,36	No
ENSMUSG00000021614	-1,84E-01	6,428833125	8,52E+00	3,51E-03	4,30E-02	Vcan	0,88	Yes
ENSMUSG00000021615	-8,43E-02	2,945680323	5,33E-01	4,65E-01	7,54E-01	Xrcc4	0,94	No
ENSMUSG00000021619	-1,40E-01	2,379869769	1,07E+00	3,02E-01	5,98E-01	Atg10	0,91	No
ENSMUSG00000021620	5,75E-01	-2,998032116	1,32E-01	7,16E-01	9,13E-01	Acot12	1,49	No
ENSMUSG00000021621	-1,70E-01	4,835727814	5,02E+00	2,51E-02	1,75E-01	Zcchc9	0,89	No
ENSMUSG00000021624	6,82E-02	6,645008531	1,20E+00	2,74E-01	5,71E-01	Cd180	1,05	No
ENSMUSG00000021629	1,04E-01	5,935831208	2,46E+00	1,16E-01	4,23E-01	Slc30a5	1,07	No
ENSMUSG00000021631	-6,39E-02	3,89593363	4,86E-01	4,86E-01	7,73E-01	Mrps36-ps1	0,96	No
ENSMUSG00000021635	1,66E-01	4,422235422	4,19E+00	4,07E-02	2,39E-01	Rad17	1,12	No
ENSMUSG00000021636	-1,73E-01	2,661867035	2,00E+00	1,57E-01	5,03E-01	Marveld2	0,89	No
ENSMUSG00000021638	-6,53E-01	1,615946472	1,61E+01	6,12E-05	1,61E-03	Ocln	0,64	Yes
ENSMUSG00000021639	6,00E-02	3,775075857	4,02E-01	5,26E-01	8,03E-01	Gtf2h2	1,04	No
ENSMUSG00000021640	1,17E-01	1,792392975	5,47E-01	4,59E-01	7,49E-01	Naip1	1,08	No
ENSMUSG00000021643	1,51E-02	1,410829747	6,94E-03	9,34E-01	1,00E+00	Serf1	1,01	No
ENSMUSG00000021645	1,45E-01	3,999767616	2,62E+00	1,06E-01	3,98E-01	Smn1	1,11	No
ENSMUSG00000021646	-1,34E-01	3,008525421	1,39E+00	2,39E-01	5,50E-01	Mccc2	0,91	No
ENSMUSG00000021650	1,01E-01	4,894420895	1,80E+00	1,79E-01	5,40E-01	Ptcd2	1,07	No
ENSMUSG00000021660	-4,17E-02	5,664149885	3,83E-01	5,36E-01	8,08E-01	Btf3	0,97	No
ENSMUSG00000021661	-1,26E-01	3,861852451	1,87E+00	1,72E-01	5,28E-01	Ankra2	0,92	No
ENSMUSG00000021662	6,97E-01	-2,070312088	8,66E-01	3,52E-01	6,53E-01	Arhgef28	1,62	No
ENSMUSG00000021665	1,06E-01	6,497288891	2,84E+00	9,20E-02	3,82E-01	Hexb	1,08	No
ENSMUSG00000021666	1,02E-01	5,210680812	2,02E+00	1,55E-01	4,98E-01	Gfm2	1,07	No
ENSMUSG00000021668	2,22E-01	4,540862308	7,85E+00	5,10E-03	5,67E-02	Polk	1,17	No
ENSMUSG00000021669	5,81E-02	6,640987119	8,74E-01	3,50E-01	6,51E-01	Col4a3bp	1,04	No

ENSMUSG00000021670	1,86E-01	7,610010761	9,88E+00	1,67E-03	2,42E-02	Hmgcr	1,14	Yes
ENSMUSG00000021671	1,75E-01	4,130183482	4,07E+00	4,37E-02	2,49E-01	Poc5	1,13	No
ENSMUSG00000021675	2,96E-01	6,426516872	2,17E+01	3,16E-06	1,22E-04	F2rl2	1,23	Yes
ENSMUSG00000021676	-1,36E-01	4,235595511	2,53E+00	1,12E-01	4,11E-01	Iqgap2	0,91	No
ENSMUSG00000021678	-1,13E+00	-1,654956846	3,82E+00	5,06E-02	2,73E-01	F2rl1	0,46	No
ENSMUSG00000021680	-1,69E+00	-2,146090028	4,32E+00	3,76E-02	2,30E-01	Crhbp	0,31	No
ENSMUSG00000021681	4,34E-02	5,850919363	4,28E-01	5,13E-01	7,95E-01	Aggf1	1,03	No
ENSMUSG00000021684	2,84E-01	3,07155816	6,45E+00	1,11E-02	1,01E-01	Pde8b	1,22	No
ENSMUSG00000021686	-2,11E-01	7,454133433	1,26E+01	3,82E-04	7,37E-03	Ap3b1	0,86	Yes
ENSMUSG00000021687	-6,68E-03	6,686633734	1,16E-02	9,14E-01	1,00E+00	Scamp1	1,00	No
ENSMUSG00000021690	1,31E-01	4,018679996	2,19E+00	1,39E-01	4,70E-01	Jmy	1,09	No
ENSMUSG00000021692	5,46E-02	1,557071603	9,70E-02	7,55E-01	9,34E-01	Dimt1	1,04	No
ENSMUSG00000021693	-7,46E-02	4,939297215	9,98E-01	3,18E-01	6,19E-01	Kif2a	0,95	No
ENSMUSG00000021694	2,78E-01	2,411708024	4,22E+00	3,99E-02	2,37E-01	Ercc8	1,21	No
ENSMUSG00000021696	1,48E-01	4,118539803	2,89E+00	8,91E-02	3,79E-01	Elovl7	1,11	No
ENSMUSG00000021697	1,94E-01	3,262470823	3,28E+00	7,01E-02	3,34E-01	Depdc1b	1,14	No
ENSMUSG00000021699	-3,47E-01	3,993494596	1,54E+01	8,93E-05	2,21E-03	Pde4d	0,79	Yes
ENSMUSG00000021700	-1,89E+00	-3,1076441	1,42E+00	2,33E-01	5,50E-01	Rab3c	0,27	No
ENSMUSG00000021701	-4,14E-02	7,209968085	4,74E-01	4,91E-01	7,77E-01	Plk2	0,97	No
ENSMUSG00000021702	-8,82E-01	-1,401261442	3,34E+00	6,78E-02	3,28E-01	Thbs4	0,54	No
ENSMUSG00000021703	-4,45E-01	5,202780214	3,88E+01	4,69E-10	4,04E-08	Serinc5	0,73	Yes
ENSMUSG00000021704	2,44E-02	4,259948281	8,38E-02	7,72E-01	9,43E-01	Mtx3	1,02	No
ENSMUSG00000021706	1,16E-01	4,73052773	2,27E+00	1,32E-01	4,56E-01	Zfyve16	1,08	No
ENSMUSG00000021707	8,38E-02	4,094747734	9,27E-01	3,36E-01	6,37E-01	Dhfr	1,06	No
ENSMUSG00000021708	5,34E-01	2,493930483	1,62E+01	5,59E-05	1,49E-03	Rasgrf2	1,45	Yes
ENSMUSG00000021709	-5,84E-02	7,228027687	9,46E-01	3,31E-01	6,31E-01	Erbin	0,96	No
ENSMUSG00000021710	1,69E-01	5,993000407	6,67E+00	9,83E-03	9,22E-02	Nln	1,12	No
ENSMUSG00000021711	6,93E-02	5,046177996	8,93E-01	3,45E-01	6,46E-01	Trappc13	1,05	No
ENSMUSG00000021712	-1,07E-01	3,974304319	1,46E+00	2,26E-01	5,50E-01	Trim23	0,93	No
ENSMUSG00000021713	2,01E-01	3,699550059	4,51E+00	3,37E-02	2,16E-01	Ppwd1	1,15	No
ENSMUSG00000021714	1,05E-01	2,362059329	6,11E-01	4,34E-01	7,27E-01	Cenpk	1,08	No
ENSMUSG00000021715	8,65E-03	3,118915867	6,20E-03	9,37E-01	1,00E+00	Cwc27	1,01	No

ENSMUSG00000021716	8,83E-02	4,032420782	1,01E+00	3,14E-01	6,14E-01	Srek1ip1	1,06	No
ENSMUSG00000021718	7,02E-01	-2,18562437	7,15E-01	3,98E-01	6,98E-01	4933425L06Rik	1,63	No
ENSMUSG00000021719	-9,37E-02	2,832804968	6,67E-01	4,14E-01	7,09E-01	Rgs7bp	0,94	No
ENSMUSG00000021720	6,27E-02	4,467860321	6,08E-01	4,36E-01	7,28E-01	Rnf180	1,04	No
ENSMUSG00000021725	-8,46E-02	3,56621065	7,57E-01	3,84E-01	6,86E-01	Parp8	0,94	No
ENSMUSG00000021728	-1,02E-01	8,270951709	3,10E+00	7,85E-02	3,58E-01	Emb	0,93	No
ENSMUSG00000021730	2,45E-01	-1,655161291	1,39E-01	7,09E-01	9,09E-01	Hcn1	1,19	No
ENSMUSG00000021731	-4,36E-03	4,610722671	3,04E-03	9,56E-01	1,00E+00	Mrps30	1,00	No
ENSMUSG00000021733	1,67E-03	6,642041638	7,18E-04	9,79E-01	1,00E+00	Slc4a7	1,00	No
ENSMUSG00000021737	-6,16E-02	6,135979262	9,10E-01	3,40E-01	6,41E-01	Psmd6	0,96	No
ENSMUSG00000021738	-8,90E-02	5,516532798	1,68E+00	1,95E-01	5,50E-01	Atxn7	0,94	No
ENSMUSG00000021741	-1,77E-01	1,44391218	9,37E-01	3,33E-01	6,33E-01	Gm5457	0,88	No
ENSMUSG00000021743	-1,39E+00	0,593352511	3,74E+01	9,81E-10	8,05E-08	Fezf2	0,38	Yes
ENSMUSG00000021745	-5,72E-01	-2,831971956	2,57E-01	6,12E-01	8,52E-01	Ptprg	0,67	No
ENSMUSG00000021747	-7,19E-01	-1,289362063	2,24E+00	1,34E-01	4,61E-01	4930452B06Rik	0,61	No
ENSMUSG00000021748	1,05E-01	5,635048407	2,40E+00	1,21E-01	4,34E-01	Pdhb	1,08	No
ENSMUSG00000021749	-1,51E+00	-2,355512635	2,37E+00	1,24E-01	4,40E-01	Oit1	0,35	No
ENSMUSG00000021750	9,01E-03	-3,107646452	-5,16E-10	1,00E+00	1,00E+00	Fam107a	1,01	No
ENSMUSG00000021751	-1,33E+00	-2,896104821	1,11E+00	2,92E-01	5,88E-01	Acox2	0,40	No
ENSMUSG00000021752	-2,52E-02	3,95280927	7,71E-02	7,81E-01	9,49E-01	Kctd6	0,98	No
ENSMUSG00000021754	-1,79E-01	6,512320299	8,24E+00	4,11E-03	4,82E-02	Map3k1	0,88	Yes
ENSMUSG00000021756	-8,74E-02	7,809466845	2,23E+00	1,35E-01	4,63E-01	Il6st	0,94	No
ENSMUSG00000021758	6,97E-01	-1,01592621	2,19E+00	1,39E-01	4,70E-01	Ddx4	1,62	No
ENSMUSG00000021759	1,74E-01	2,88241798	2,25E+00	1,34E-01	4,60E-01	Plpp1	1,13	No
ENSMUSG00000021760	-5,99E-02	-2,49756396	3,28E-03	9,54E-01	1,00E+00	Gpx8	0,96	No
ENSMUSG00000021763	2,15E+00	-2,497602254	3,72E+00	5,39E-02	2,84E-01	BC067074	4,45	No
ENSMUSG00000021764	-7,06E-02	4,728503071	8,38E-01	3,60E-01	6,61E-01	Ndufs4	0,95	No
ENSMUSG00000021765	2,77E-02	1,730576565	2,94E-02	8,64E-01	9,97E-01	Fst	1,02	No
ENSMUSG00000021767	-6,39E-02	4,506942908	6,18E-01	4,32E-01	7,25E-01	Kat6b	0,96	No
ENSMUSG00000021768	-1,13E-01	1,111346673	3,40E-01	5,60E-01	8,23E-01	Dusp13	0,92	No
ENSMUSG00000021770	1,82E-01	6,331279535	8,15E+00	4,30E-03	4,99E-02	Samd8	1,13	Yes
ENSMUSG00000021771	-3,02E-02	7,415375886	2,57E-01	6,12E-01	8,52E-01	Vdac2	0,98	No

ENSMUSG00000021772	-1,90E-02	4,467261099	5,54E-02	8,14E-01	9,68E-01	Nkiras1	0,99	No
ENSMUSG00000021773	1,48E-01	3,786882926	2,53E+00	1,12E-01	4,11E-01	Comtd1	1,11	No
ENSMUSG00000021774	3,51E-03	4,952782324	2,21E-03	9,62E-01	1,00E+00	Ube2e1	1,00	No
ENSMUSG00000021775	3,36E-03	4,37797148	1,64E-03	9,68E-01	1,00E+00	Nr1d2	1,00	No
ENSMUSG00000021779	3,97E-01	0,638436443	3,07E+00	7,96E-02	3,61E-01	Thrb	1,32	No
ENSMUSG00000021782	4,31E-01	-0,093725899	2,05E+00	1,52E-01	4,94E-01	Dlg5	1,35	No
ENSMUSG00000021785	-9,39E-02	4,897237345	1,57E+00	2,10E-01	5,50E-01	Ngly1	0,94	No
ENSMUSG00000021786	1,59E-01	3,787678414	2,99E+00	8,37E-02	3,71E-01	Oxsm	1,12	No
ENSMUSG00000021792	2,85E-01	3,292950833	7,23E+00	7,18E-03	7,32E-02	Fam213a	1,22	No
ENSMUSG00000021794	6,07E-02	8,176600795	1,10E+00	2,94E-01	5,89E-01	Glud1	1,04	No
ENSMUSG00000021796	-1,50E-01	1,768031116	9,09E-01	3,40E-01	6,41E-01	Bmpr1a	0,90	No
ENSMUSG00000021798	9,17E-01	-1,832050441	2,14E+00	1,44E-01	4,79E-01	Ldb3	1,89	No
ENSMUSG00000021803	-4,38E-01	-0,060911286	2,45E+00	1,18E-01	4,26E-01	Cdhr1	0,74	No
ENSMUSG00000021804	1,81E+00	-3,10765737	1,28E+00	2,58E-01	5,50E-01	Rgr	3,49	No
ENSMUSG00000021806	-2,23E-02	2,013270776	2,30E-02	8,79E-01	1,00E+00	Nid2	0,98	No
ENSMUSG00000021807	-3,09E-01	4,986023913	1,75E+01	2,93E-05	8,58E-04	2700060E02Rik	0,81	Yes
ENSMUSG00000021809	1,78E-02	3,444230868	3,18E-02	8,58E-01	9,94E-01	Nudt13	1,01	No
ENSMUSG00000021810	6,33E-02	5,455064424	8,27E-01	3,63E-01	6,64E-01	Ecd	1,04	No
ENSMUSG00000021811	8,98E-02	5,313081137	1,60E+00	2,06E-01	5,50E-01	Dnajc9	1,06	No
ENSMUSG00000021814	-6,59E-02	7,120883115	1,19E+00	2,75E-01	5,71E-01	Anxa7	0,96	No
ENSMUSG00000021815	4,21E-01	0,375374419	2,99E+00	8,39E-02	3,71E-01	Mss51	1,34	No
ENSMUSG00000021816	7,39E-02	5,79094346	1,23E+00	2,67E-01	5,63E-01	Ppp3cb	1,05	No
ENSMUSG00000021819	-3,54E-02	7,441421196	3,55E-01	5,51E-01	8,17E-01	Zswim8	0,98	No
ENSMUSG00000021820	2,25E-02	6,247929179	1,25E-01	7,24E-01	9,17E-01	Camk2g	1,02	No
ENSMUSG00000021822	2,84E-01	7,830975083	2,34E+01	1,35E-06	5,80E-05	Plau	1,22	Yes
ENSMUSG00000021823	1,29E-01	6,595939726	4,25E+00	3,92E-02	2,35E-01	Vcl	1,09	No
ENSMUSG00000021824	1,51E-01	6,585464762	5,87E+00	1,54E-02	1,28E-01	Ap3m1	1,11	No
ENSMUSG00000021830	2,42E-01	5,135657679	1,10E+01	9,29E-04	1,52E-02	Txndc16	1,18	Yes
ENSMUSG00000021831	1,62E-01	6,355786217	6,48E+00	1,09E-02	9,95E-02	Ero1l	1,12	No
ENSMUSG00000021832	4,60E-02	5,251194242	4,14E-01	5,20E-01	7,99E-01	Psmc6	1,03	No
ENSMUSG00000021835	-1,89E+00	-3,1076441	1,42E+00	2,33E-01	5,50E-01	Bmp4	0,27	No
ENSMUSG00000021838	-2,19E+00	0,393138045	5,91E+01	1,51E-14	2,21E-12	Samd4	0,22	Yes

ENSMUSG00000021840	8,86E-02	6,961721176	2,12E+00	1,45E-01	4,82E-01	Mapk1ip1l	1,06	No
ENSMUSG00000021843	-3,19E-01	5,277881451	2,03E+01	6,52E-06	2,28E-04	Ktn1	0,80	Yes
ENSMUSG00000021846	1,64E-01	6,349934336	6,59E+00	1,03E-02	9,52E-02	Peli2	1,12	No
ENSMUSG00000021850	-1,89E+00	-3,1076441	1,42E+00	2,33E-01	5,50E-01	1700011H14Rik	0,27	No
ENSMUSG00000021852	-2,74E+00	-3,033596063	2,99E+00	8,38E-02	3,71E-01	Slc35f4	0,15	No
ENSMUSG00000021866	-2,32E-02	5,588088433	1,15E-01	7,35E-01	9,24E-01	Anxa11	0,98	No
ENSMUSG00000021867	1,36E-01	2,520161899	1,12E+00	2,89E-01	5,85E-01	Tmem254b	1,10	No
ENSMUSG00000021868	-2,91E-03	4,338058302	1,24E-03	9,72E-01	1,00E+00	Ppif	1,00	No
ENSMUSG00000021870	-6,99E-02	6,687536295	1,27E+00	2,59E-01	5,52E-01	Slmap	0,95	No
ENSMUSG00000021871	6,56E-02	7,257541477	1,20E+00	2,74E-01	5,70E-01	Pnp	1,05	No
ENSMUSG00000021872	1,84E+00	-3,033649193	1,33E+00	2,49E-01	5,50E-01	Rnase10	3,58	No
ENSMUSG00000021874	6,62E-02	-1,740902571	1,07E-02	9,18E-01	1,00E+00	4933413J09Rik	1,05	No
ENSMUSG00000021876	3,42E-01	4,602404568	1,80E+01	2,23E-05	6,84E-04	Rnase4	1,27	Yes
ENSMUSG00000021877	-1,38E-01	7,149817224	5,24E+00	2,21E-02	1,61E-01	Arf4	0,91	No
ENSMUSG00000021879	-8,82E-01	-2,963247103	4,30E-01	5,12E-01	7,95E-01	Dnah12	0,54	No
ENSMUSG00000021880	1,72E-01	5,587648366	6,25E+00	1,24E-02	1,10E-01	Rnase6	1,13	No
ENSMUSG00000021884	1,11E-01	2,052963408	5,39E-01	4,63E-01	7,52E-01	Hacl1	1,08	No
ENSMUSG00000021886	-5,17E-01	4,303039928	3,86E+01	5,28E-10	4,48E-08	Gpr65	0,70	Yes
ENSMUSG00000021890	6,08E-02	6,602510289	9,50E-01	3,30E-01	6,30E-01	Eaf1	1,04	No
ENSMUSG00000021891	1,17E-01	4,296144398	1,98E+00	1,60E-01	5,07E-01	Mettl6	1,08	No
ENSMUSG00000021892	6,50E-02	4,623887306	6,78E-01	4,10E-01	7,06E-01	Sh3bp5	1,05	No
ENSMUSG00000021893	-3,70E-02	6,183626994	3,32E-01	5,65E-01	8,27E-01	Capn7	0,97	No
ENSMUSG00000021895	-1,17E-01	5,33411163	2,77E+00	9,59E-02	3,87E-01	Arhgef3	0,92	No
ENSMUSG00000021898	-2,77E-01	0,062193991	9,52E-01	3,29E-01	6,30E-01	Asb14	0,83	No
ENSMUSG00000021900	2,09E-01	3,940747942	5,30E+00	2,13E-02	1,57E-01	Btd	1,16	No
ENSMUSG00000021901	-5,40E-02	6,082988505	6,94E-01	4,05E-01	7,02E-01	Bap1	0,96	No
ENSMUSG00000021902	1,42E-01	2,635677321	1,28E+00	2,58E-01	5,51E-01	Phf7	1,10	No
ENSMUSG00000021903	-6,87E-01	-2,070090537	1,08E+00	3,00E-01	5,96E-01	Galnt15	0,62	No
ENSMUSG00000021904	-7,38E-02	-3,033634644	2,66E-03	9,59E-01	1,00E+00	Sema3g	0,95	No
ENSMUSG00000021905	3,59E-02	4,375959462	1,89E-01	6,64E-01	8,84E-01	Dph3	1,03	No
ENSMUSG00000021906	6,01E-02	3,370001941	3,43E-01	5,58E-01	8,22E-01	Oxnad1	1,04	No
ENSMUSG00000021907	-1,94E+00	-3,107619243	1,50E+00	2,21E-01	5,50E-01	Msmb	0,26	No

ENSMUSG00000021908	-1,93E-01	6,70427897	9,84E+00	1,71E-03	2,46E-02	Gm6768	0,87	Yes
ENSMUSG00000021910	-6,81E-02	8,377331249	1,40E+00	2,37E-01	5,50E-01	Nisch	0,95	No
ENSMUSG00000021911	1,17E-01	5,203494581	2,64E+00	1,04E-01	3,97E-01	Parg	1,08	No
ENSMUSG00000021913	6,73E-01	0,062262696	4,95E+00	2,61E-02	1,80E-01	Ogdhl	1,59	No
ENSMUSG00000021916	2,12E-02	3,971999071	5,67E-02	8,12E-01	9,67E-01	Glt8d1	1,01	No
ENSMUSG00000021917	-4,69E-02	5,836859027	4,95E-01	4,82E-01	7,69E-01	Spcs1	0,97	No
ENSMUSG00000021918	1,42E-01	3,275938067	1,83E+00	1,76E-01	5,35E-01	Nek4	1,10	No
ENSMUSG00000021922	1,87E+00	-2,83194184	1,36E+00	2,43E-01	5,50E-01	Itih4	3,67	No
ENSMUSG00000021927	-3,83E-03	1,302596849	3,95E-04	9,84E-01	1,00E+00	Hnrnpa1l2-ps2	1,00	No
ENSMUSG00000021928	4,09E-02	3,537615881	1,72E-01	6,78E-01	8,92E-01	Ebpl	1,03	No
ENSMUSG00000021929	-4,23E-02	6,012358124	4,20E-01	5,17E-01	7,98E-01	Kpna3	0,97	No
ENSMUSG00000021930	8,68E-02	3,903026622	9,00E-01	3,43E-01	6,43E-01	Spryd7	1,06	No
ENSMUSG00000021932	7,93E-02	3,977098182	7,81E-01	3,77E-01	6,78E-01	Rnaseh2b	1,06	No
ENSMUSG00000021933	1,25E+00	-2,711729423	9,72E-01	3,24E-01	6,25E-01	Gucy1b2	2,38	No
ENSMUSG00000021936	3,85E-02	3,970501916	1,85E-01	6,67E-01	8,86E-01	Mapk8	1,03	No
ENSMUSG00000021938	5,55E-02	4,888219897	5,39E-01	4,63E-01	7,51E-01	Pspc1	1,04	No
ENSMUSG00000021939	1,54E-01	11,73936746	7,61E+00	5,81E-03	6,26E-02	Ctsb	1,11	No
ENSMUSG00000021940	9,01E-03	-3,107646452	-5,16E-10	1,00E+00	1,00E+00	Ptpn20	1,01	No
ENSMUSG00000021944	-1,87E+00	-3,107637761	1,39E+00	2,38E-01	5,50E-01	Gata4	0,27	No
ENSMUSG00000021945	9,89E-03	5,78185339	2,21E-02	8,82E-01	1,00E+00	Zmym2	1,01	No
ENSMUSG00000021947	1,24E-01	4,715339466	2,53E+00	1,12E-01	4,10E-01	Cryl1	1,09	No
ENSMUSG00000021948	6,40E-02	9,910191707	1,29E+00	2,56E-01	5,50E-01	Prkcd	1,05	No
ENSMUSG00000021950	2,68E-01	0,144275053	9,89E-01	3,20E-01	6,21E-01	Anxa8	1,20	No
ENSMUSG00000021951	1,38E-01	4,287434288	2,68E+00	1,02E-01	3,94E-01	Eef1akmt1	1,10	No
ENSMUSG00000021952	4,65E-02	5,453973256	4,48E-01	5,03E-01	7,88E-01	Xpo4	1,03	No
ENSMUSG00000021953	-7,85E-01	-1,175455494	3,22E+00	7,29E-02	3,43E-01	Tdh	0,58	No
ENSMUSG00000021957	1,17E-01	9,08119094	4,21E+00	4,01E-02	2,38E-01	Tkt	1,08	No
ENSMUSG00000021958	9,05E-02	3,204395805	7,19E-01	3,97E-01	6,97E-01	Pinx1	1,06	No
ENSMUSG00000021959	1,96E-01	4,75278164	6,37E+00	1,16E-02	1,04E-01	Lats2	1,15	No
ENSMUSG00000021962	4,90E-02	5,40657591	4,95E-01	4,81E-01	7,69E-01	Dcp1a	1,03	No
ENSMUSG00000021963	-3,30E-02	4,645259347	1,77E-01	6,74E-01	8,90E-01	Sap18	0,98	No
ENSMUSG00000021965	2,04E-01	3,669435019	4,57E+00	3,26E-02	2,11E-01	Ska3	1,15	No

ENSMUSG00000021966	3,11E+00	-2,963280805	3,94E+00	4,72E-02	2,60E-01	Prss52	8,63	No
ENSMUSG00000021967	-1,01E-02	4,533975511	1,56E-02	9,01E-01	1,00E+00	Mrpl57	0,99	No
ENSMUSG00000021969	4,83E-02	6,523101016	5,92E-01	4,42E-01	7,34E-01	Zdhhc20	1,03	No
ENSMUSG00000021972	5,17E-02	6,072637784	6,35E-01	4,26E-01	7,20E-01	Hmbox1	1,04	No
ENSMUSG00000021973	-1,99E-02	5,643671144	8,61E-02	7,69E-01	9,42E-01	Micu2	0,99	No
ENSMUSG00000021974	-1,87E+00	-3,107637761	1,39E+00	2,38E-01	5,50E-01	Fgf9	0,27	No
ENSMUSG00000021975	5,66E-02	4,728755537	5,35E-01	4,64E-01	7,53E-01	Ints9	1,04	No
ENSMUSG00000021978	2,46E-01	6,155961686	1,45E+01	1,42E-04	3,25E-03	Extl3	1,19	Yes
ENSMUSG00000021981	-3,29E-02	5,596557637	2,33E-01	6,29E-01	8,62E-01	Cab39l	0,98	No
ENSMUSG00000021982	-3,43E-02	4,661471258	1,92E-01	6,61E-01	8,82E-01	Cdadc1	0,98	No
ENSMUSG00000021983	1,10E+00	-2,051805806	2,41E+00	1,21E-01	4,33E-01	Atp8a2	2,14	No
ENSMUSG00000021986	-1,34E+00	-2,770595692	1,12E+00	2,90E-01	5,85E-01	Amer2	0,40	No
ENSMUSG00000021987	-6,25E-02	7,154191598	1,08E+00	2,98E-01	5,94E-01	Mtmr6	0,96	No
ENSMUSG00000021990	3,84E-01	4,165823815	1,99E+01	7,96E-06	2,74E-04	Spata13	1,30	Yes
ENSMUSG00000021993	-7,59E-02	3,07968151	4,67E-01	4,94E-01	7,80E-01	Mipep	0,95	No
ENSMUSG00000021994	-1,87E+00	-3,033619006	1,41E+00	2,36E-01	5,50E-01	Wnt5a	0,27	No
ENSMUSG00000021996	4,30E-02	6,359913808	4,56E-01	5,00E-01	7,85E-01	Esd	1,03	No
ENSMUSG00000021997	-1,24E-01	-0,770767362	9,44E-02	7,59E-01	9,36E-01	Lrrc63	0,92	No
ENSMUSG00000021998	1,13E-01	10,8413618	4,09E+00	4,31E-02	2,47E-01	Lcp1	1,08	No
ENSMUSG00000021999	-1,89E+00	-3,1076441	1,42E+00	2,33E-01	5,50E-01	Cpb2	0,27	No
ENSMUSG00000022000	4,37E-02	5,777383064	4,27E-01	5,14E-01	7,96E-01	Zc3h13	1,03	No
ENSMUSG00000022002	-3,83E-02	-3,107651936	-7,91E-11	1,00E+00	1,00E+00	Erich6b	0,97	No
ENSMUSG00000022003	1,16E-01	3,945671811	1,69E+00	1,94E-01	5,50E-01	Slc25a30	1,08	No
ENSMUSG00000022008	-1,19E-01	5,980646795	3,28E+00	7,03E-02	3,35E-01	Gpalpp1	0,92	No
ENSMUSG00000022009	-1,29E-01	4,593223415	2,65E+00	1,04E-01	3,96E-01	Nufip1	0,91	No
ENSMUSG00000022010	-6,63E-03	4,925199176	7,93E-03	9,29E-01	1,00E+00	Tsc22d1	1,00	No
ENSMUSG00000022012	4,42E-01	-2,655163718	1,53E-01	6,96E-01	9,03E-01	Enox1	1,36	No
ENSMUSG00000022013	8,42E-02	4,728170123	1,16E+00	2,81E-01	5,78E-01	Dnajc15	1,06	No
ENSMUSG00000022014	-5,41E-01	4,629449991	4,74E+01	5,80E-12	6,41E-10	Epsti1	0,69	Yes
ENSMUSG00000022015	-1,36E+00	-2,896086315	1,16E+00	2,81E-01	5,78E-01	Tnfsf11	0,39	No
ENSMUSG00000022016	8,39E-02	5,865105442	1,60E+00	2,05E-01	5,50E-01	Akap11	1,06	No
ENSMUSG00000022018	2,68E-02	2,896023024	5,21E-02	8,19E-01	9,71E-01	Rgcc	1,02	No

ENSMUSG00000022019	1,12E-01	4,045346913	1,60E+00	2,06E-01	5,50E-01	Tdrd3	1,08	No
ENSMUSG00000022020	1,18E-01	4,002699446	1,78E+00	1,83E-01	5,45E-01	Naa16	1,09	No
ENSMUSG00000022021	1,21E-01	4,568972512	2,31E+00	1,29E-01	4,50E-01	Diaph3	1,09	No
ENSMUSG00000022022	-1,60E-01	2,073044794	1,14E+00	2,85E-01	5,82E-01	Mtrf1	0,90	No
ENSMUSG00000022023	-8,49E-02	4,880390198	1,28E+00	2,58E-01	5,51E-01	Wbp4	0,94	No
ENSMUSG00000022024	-3,39E-01	5,052081771	2,13E+01	3,97E-06	1,49E-04	Sugt1	0,79	Yes
ENSMUSG00000022026	-9,85E-01	-2,548226909	8,50E-01	3,57E-01	6,58E-01	Olfm4	0,51	No
ENSMUSG00000022031	-7,16E-02	4,154885126	6,91E-01	4,06E-01	7,02E-01	Elp3	0,95	No
ENSMUSG00000022032	7,78E-01	-2,963243536	3,33E-01	5,64E-01	8,26E-01	Scara5	1,71	No
ENSMUSG00000022033	1,50E-01	3,382029676	2,12E+00	1,46E-01	4,83E-01	Pbk	1,11	No
ENSMUSG00000022034	4,23E-01	4,379848256	2,62E+01	3,06E-07	1,53E-05	Esco2	1,34	Yes
ENSMUSG00000022035	-4,26E-02	4,883634565	3,22E-01	5,71E-01	8,31E-01	Ccdc25	0,97	No
ENSMUSG00000022037	-1,07E-01	5,010646409	2,14E+00	1,43E-01	4,78E-01	Clu	0,93	No
ENSMUSG00000022039	-2,49E-01	-1,561092416	1,84E-01	6,68E-01	8,86E-01	Adam2	0,84	No
ENSMUSG00000022040	-8,28E-01	-1,24700863	3,10E+00	7,81E-02	3,57E-01	Ephx2	0,56	No
ENSMUSG00000022041	-1,51E-01	1,028735163	5,04E-01	4,78E-01	7,65E-01	Chrna2	0,90	No
ENSMUSG00000022043	-1,56E-01	7,289247438	6,85E+00	8,85E-03	8,54E-02	Trim35	0,90	No
ENSMUSG00000022044	8,52E-01	-2,548198116	8,73E-01	3,50E-01	6,51E-01	Stmn4	1,81	No
ENSMUSG00000022048	-3,90E-02	4,987551103	2,76E-01	6,00E-01	8,46E-01	Dpysl2	0,97	No
ENSMUSG00000022051	6,18E-02	6,747251934	1,00E+00	3,16E-01	6,17E-01	Bnip3l	1,04	No
ENSMUSG00000022052	1,30E-01	4,973838082	3,07E+00	8,00E-02	3,62E-01	Ppp2r2a	1,09	No
ENSMUSG00000022054	-5,27E+00	-2,311154008	1,98E+01	8,61E-06	2,94E-04	Nefm	0,03	Yes
ENSMUSG00000022057	2,74E-02	-3,033621357	9,16E-12	1,00E+00	1,00E+00	Adamdec1	1,02	No
ENSMUSG00000022061	-3,83E-02	-3,107651936	-7,91E-11	1,00E+00	1,00E+00	Nkx3-1	0,97	No
ENSMUSG00000022064	3,53E-04	3,153127641	1,05E-05	9,97E-01	1,00E+00	Pibf1	1,00	No
ENSMUSG00000022066	4,77E-02	4,019179485	2,88E-01	5,92E-01	8,42E-01	Entpd4b	1,03	No
ENSMUSG00000022070	1,75E-01	4,326196693	4,45E+00	3,49E-02	2,21E-01	Bora	1,13	No
ENSMUSG00000022074	3,43E-02	3,565607418	1,27E-01	7,22E-01	9,16E-01	Tnfrsf10b	1,02	No
ENSMUSG00000022075	3,30E-01	3,976247417	1,41E+01	1,69E-04	3,77E-03	Rhobtb2	1,26	Yes
ENSMUSG00000022085	-2,73E-02	-2,963256629	3,40E-04	9,85E-01	1,00E+00	Pebp4	0,98	No
ENSMUSG00000022089	1,05E-01	5,882038243	2,52E+00	1,13E-01	4,13E-01	Bin3	1,08	No
ENSMUSG00000022090	1,96E-01	4,462096274	5,81E+00	1,59E-02	1,31E-01	Pdlim2	1,15	No

ENSMUSG00000022091	1,38E-01	3,020718014	1,43E+00	2,32E-01	5,50E-01	Sorbs3	1,10	No
ENSMUSG00000022092	-2,43E-01	3,246644752	5,37E+00	2,05E-02	1,54E-01	Ppp3cc	0,85	No
ENSMUSG00000022094	5,17E-02	5,62164284	5,69E-01	4,51E-01	7,41E-01	Slc39a14	1,04	No
ENSMUSG00000022095	9,26E-02	5,638258793	1,86E+00	1,72E-01	5,29E-01	Fam160b2	1,07	No
ENSMUSG00000022096	1,53E-01	7,413648758	6,62E+00	1,01E-02	9,38E-02	Hr	1,11	No
ENSMUSG00000022097	1,87E+00	-3,107639691	1,38E+00	2,40E-01	5,50E-01	Sftpc	3,67	No
ENSMUSG00000022098	-8,99E-01	2,519734573	4,97E+01	1,83E-12	2,15E-10	Bmp1	0,54	Yes
ENSMUSG00000022099	-2,53E-01	0,12021982	8,30E-01	3,62E-01	6,64E-01	Dmtn	0,84	No
ENSMUSG00000022100	4,02E-02	6,081037141	3,83E-01	5,36E-01	8,08E-01	Xpo7	1,03	No
ENSMUSG00000022102	2,07E-01	8,240201448	1,28E+01	3,55E-04	6,94E-03	Dok2	1,15	Yes
ENSMUSG00000022103	5,67E-01	5,513892092	6,64E+01	3,59E-16	6,12E-14	Gfra2	1,48	Yes
ENSMUSG00000022105	-2,97E-02	6,286810499	2,18E-01	6,41E-01	8,70E-01	Rb1	0,98	No
ENSMUSG00000022106	-2,30E-01	6,834808431	1,41E+01	1,77E-04	3,93E-03	Rcbtb2	0,85	Yes
ENSMUSG00000022108	-1,01E-01	9,467465659	3,17E+00	7,48E-02	3,48E-01	Itm2b	0,93	No
ENSMUSG00000022109	8,21E-02	3,7224197	7,60E-01	3,83E-01	6,85E-01	Med4	1,06	No
ENSMUSG00000022110	-5,51E-02	5,37149275	6,13E-01	4,34E-01	7,27E-01	Sucla2	0,96	No
ENSMUSG00000022111	9,51E-02	1,957125238	3,98E-01	5,28E-01	8,04E-01	Uchl3	1,07	No
ENSMUSG00000022114	-1,56E-01	0,490258219	3,84E-01	5,35E-01	8,08E-01	Spry2	0,90	No
ENSMUSG00000022119	-9,02E-03	5,75738544	1,81E-02	8,93E-01	1,00E+00	Rbm26	0,99	No
ENSMUSG00000022120	-2,09E-01	3,534078244	4,86E+00	2,75E-02	1,87E-01	Rnf219	0,87	No
ENSMUSG00000022122	-4,23E-01	-0,785606923	1,04E+00	3,08E-01	6,06E-01	Ednrb	0,75	No
ENSMUSG00000022123	4,61E-01	2,641460819	1,36E+01	2,28E-04	4,84E-03	Scel	1,38	Yes
ENSMUSG00000022124	2,67E-02	6,349041869	1,77E-01	6,74E-01	8,90E-01	Fbxl3	1,02	No
ENSMUSG00000022125	4,26E-02	6,957895994	4,89E-01	4,84E-01	7,72E-01	Cln5	1,03	No
ENSMUSG00000022126	-5,37E-01	5,517198002	6,00E+01	9,35E-15	1,39E-12	Acod1	0,69	Yes
ENSMUSG00000022130	1,39E-02	3,240110169	1,73E-02	8,95E-01	1,00E+00	Tgds	1,01	No
ENSMUSG00000022131	3,34E-01	3,891974041	1,33E+01	2,66E-04	5,49E-03	Gpr180	1,26	Yes
ENSMUSG00000022132	-6,77E-01	1,136263924	1,17E+01	6,19E-04	1,10E-02	Cldn10	0,63	Yes
ENSMUSG00000022136	8,67E-02	7,176391674	2,08E+00	1,50E-01	4,91E-01	Dnajc3	1,06	No
ENSMUSG00000022139	1,80E-01	6,793601618	8,51E+00	3,53E-03	4,32E-02	Mbnl2	1,13	Yes
ENSMUSG00000022141	-6,21E-02	7,067745222	1,05E+00	3,05E-01	6,02E-01	Nipbl	0,96	No
ENSMUSG00000022142	1,30E-01	5,411955492	3,47E+00	6,25E-02	3,11E-01	Nup155	1,09	No

ENSMUSG00000022144	-1,04E-02	-2,963271219	-1,18E-10	1,00E+00	1,00E+00	Gdnf		0,99	No
ENSMUSG00000022146	-4,35E-01	-2,033548944	3,47E-01	5,56E-01	8,21E-01	Osmr		0,74	No
ENSMUSG00000022148	-1,32E-02	5,54522335	3,63E-02	8,49E-01	9,88E-01	Fyb		0,99	No
ENSMUSG00000022149	-1,94E+00	-3,107619243	1,50E+00	2,21E-01	5,50E-01	C9		0,26	No
ENSMUSG00000022150	4,17E-01	9,131216393	5,34E+01	2,77E-13	3,65E-11	Dab2		1,34	Yes
ENSMUSG00000022151	7,84E-03	4,71049445	1,04E-02	9,19E-01	1,00E+00	Ttc33		1,01	No
ENSMUSG00000022157	-7,25E-01	-2,333151573	5,89E-01	4,43E-01	7,34E-01	Mcpt8		0,61	No
ENSMUSG00000022159	1,53E-02	2,450256296	1,36E-02	9,07E-01	1,00E+00	Rab2b		1,01	No
ENSMUSG00000022160	1,70E-01	4,584759079	4,62E+00	3,16E-02	2,06E-01	Mettl3		1,13	No
ENSMUSG00000022174	8,34E-02	6,204565773	1,68E+00	1,95E-01	5,50E-01	Dad1		1,06	No
ENSMUSG00000022175	-3,15E-03	7,836603484	2,90E-03	9,57E-01	1,00E+00	Lrp10		1,00	No
ENSMUSG00000022176	1,61E-01	-2,355620036	3,87E-02	8,44E-01	9,86E-01	Rem2		1,12	No
ENSMUSG00000022177	1,25E-01	4,429655732	2,36E+00	1,24E-01	4,41E-01	Haus4		1,09	No
ENSMUSG00000022178	1,74E-01	1,028181282	7,40E-01	3,90E-01	6,90E-01	Ajuba		1,13	No
ENSMUSG00000022179	2,82E-01	2,055513429	3,67E+00	5,54E-02	2,88E-01	4931414P19Rik		1,22	No
ENSMUSG00000022180	1,42E-01	6,341350542	5,00E+00	2,54E-02	1,77E-01	Slc7a8		1,10	No
ENSMUSG00000022181	3,14E-02	-3,107617333	-7,69E-10	1,00E+00	1,00E+00	C6		1,02	No
ENSMUSG00000022184	1,19E-02	3,609404236	1,56E-02	9,01E-01	1,00E+00	Fbxo4		1,01	No
ENSMUSG00000022185	-2,56E-02	7,506786253	1,87E-01	6,66E-01	8,85E-01	Acin1		0,98	No
ENSMUSG00000022186	1,59E-01	7,865449798	7,42E+00	6,44E-03	6,76E-02	Oxct1		1,12	No
ENSMUSG00000022191	1,07E-01	4,198069827	1,57E+00	2,11E-01	5,50E-01	Drosha		1,08	No
ENSMUSG00000022193	-8,43E-02	5,839746457	1,60E+00	2,06E-01	5,50E-01	Psmb5		0,94	No
ENSMUSG00000022194	-1,11E-01	5,369668191	2,52E+00	1,13E-01	4,13E-01	Pabpn1		0,93	No
ENSMUSG00000022195	-9,60E-03	4,736710118	1,56E-02	9,01E-01	1,00E+00	6030458C11Rik		0,99	No
ENSMUSG00000022197	4,38E-01	0,167909936	2,30E+00	1,29E-01	4,51E-01	Pdzd2		1,36	No
ENSMUSG00000022199	-5,88E-01	0,379380635	5,10E+00	2,39E-02	1,70E-01	Slc22a17		0,67	No
ENSMUSG00000022200	-4,13E-02	6,839257178	4,54E-01	5,01E-01	7,86E-01	Golph3		0,97	No
ENSMUSG00000022201	-3,53E-02	6,722887729	3,28E-01	5,67E-01	8,29E-01	Zfr		0,98	No
ENSMUSG00000022203	-1,89E+00	-3,1076441	1,42E+00	2,33E-01	5,50E-01	Efs		0,27	No
ENSMUSG00000022204	-5,51E-02	4,501905625	4,67E-01	4,95E-01	7,80E-01	Ngdn		0,96	No
ENSMUSG00000022205	-2,00E-01	6,617305167	1,03E+01	1,31E-03	2,01E-02	Sub1		0,87	Yes
ENSMUSG00000022206	7,31E-01	-2,963267542	2,91E-01	5,90E-01	8,41E-01	Npr3		1,66	No

ENSMUSG00000022208	-5,69E-02	-2,711704538	2,92E-03	9,57E-01	1,00E+00	Jph4	0,96	No
ENSMUSG00000022210	-2,58E-02	3,561611107	7,11E-02	7,90E-01	9,54E-01	Dhrs4	0,98	No
ENSMUSG00000022211	-9,44E-01	0,266739925	1,18E+01	5,81E-04	1,04E-02	Carmil3	0,52	Yes
ENSMUSG00000022212	-2,95E-01	0,489890553	1,37E+00	2,41E-01	5,50E-01	Cpne6	0,81	No
ENSMUSG00000022214	-1,46E-01	6,236880353	5,21E+00	2,24E-02	1,63E-01	Dcaf11	0,90	No
ENSMUSG00000022215	-3,19E+00	-2,770600616	4,28E+00	3,86E-02	2,33E-01	Fitm1	0,11	No
ENSMUSG00000022216	-2,60E-01	5,628825974	1,47E+01	1,26E-04	2,95E-03	Psme1	0,83	Yes
ENSMUSG00000022217	-1,05E-02	2,775049158	7,80E-03	9,30E-01	1,00E+00	Emc9	0,99	No
ENSMUSG00000022218	3,35E-01	2,693453868	7,25E+00	7,10E-03	7,26E-02	Tgm1	1,26	No
ENSMUSG00000022219	-1,21E-02	1,620436009	5,06E-03	9,43E-01	1,00E+00	Cideb	0,99	No
ENSMUSG00000022220	7,16E-01	0,910692812	1,01E+01	1,49E-03	2,22E-02	Adcy4	1,64	Yes
ENSMUSG00000022221	6,12E-02	5,932264235	8,55E-01	3,55E-01	6,56E-01	Ripk3	1,04	No
ENSMUSG00000022223	-1,02E-01	4,635021487	1,71E+00	1,91E-01	5,50E-01	Sdr39u1	0,93	No
ENSMUSG00000022225	-3,83E-02	-3,107651936	-7,91E-11	1,00E+00	1,00E+00	Cma1	0,97	No
ENSMUSG00000022226	-1,94E+00	-3,107619243	1,50E+00	2,21E-01	5,50E-01	Mcpt2	0,26	No
ENSMUSG00000022227	-6,05E-02	-1,831980854	8,52E-03	9,26E-01	1,00E+00	Mcpt1	0,96	No
ENSMUSG00000022228	2,25E-01	5,365599102	1,05E+01	1,22E-03	1,91E-02	Zscan26	1,17	Yes
ENSMUSG00000022229	9,01E-03	-3,107646452	-5,16E-10	1,00E+00	1,00E+00	Atp12a	1,01	No
ENSMUSG00000022231	-1,87E+00	-3,033619006	1,41E+00	2,36E-01	5,50E-01	Sema5a	0,27	No
ENSMUSG00000022234	-3,93E-03	7,549001015	4,42E-03	9,47E-01	1,00E+00	Cct5	1,00	No
ENSMUSG00000022235	1,65E-01	-1,268178903	1,10E-01	7,40E-01	9,26E-01	Cmbl	1,12	No
ENSMUSG00000022236	2,15E-01	1,489784287	1,47E+00	2,26E-01	5,50E-01	Ropn1l	1,16	No
ENSMUSG00000022237	-8,94E-01	5,067293571	1,49E+02	2,74E-34	2,05E-31	Ankrd33b	0,54	Yes
ENSMUSG00000022240	5,08E-01	3,178346106	2,12E+01	4,13E-06	1,54E-04	Ctnnd2	1,42	Yes
ENSMUSG00000022241	8,48E-02	5,528555207	1,51E+00	2,19E-01	5,50E-01	Tars	1,06	No
ENSMUSG00000022244	8,25E-02	2,32050073	3,62E-01	5,47E-01	8,15E-01	Amacr	1,06	No
ENSMUSG00000022245	1,26E+00	-2,770589435	9,81E-01	3,22E-01	6,22E-01	Skor1	2,39	No
ENSMUSG00000022246	-1,44E-01	6,332810321	5,20E+00	2,26E-02	1,64E-01	Rai14	0,90	No
ENSMUSG00000022247	1,42E-01	4,482600249	3,08E+00	7,91E-02	3,60E-01	Brix1	1,10	No
ENSMUSG00000022248	2,68E-02	2,672072267	4,87E-02	8,25E-01	9,74E-01	Rad1	1,02	No
ENSMUSG00000022249	2,61E+00	-3,033672762	2,63E+00	1,05E-01	3,97E-01	Ttc23l	6,10	No
ENSMUSG00000022253	6,97E-02	3,446105735	4,76E-01	4,90E-01	7,76E-01	Nadk2	1,05	No

ENSMUSG00000022255	8,63E-02	7,397461225	2,10E+00	1,47E-01	4,86E-01	Mtdh		1,06	No
ENSMUSG00000022257	-1,92E-01	3,222268311	3,25E+00	7,15E-02	3,39E-01	Laptm4b		0,88	No
ENSMUSG00000022261	-4,61E-01	0,438915315	3,59E+00	5,82E-02	2,98E-01	Sdc2		0,73	No
ENSMUSG00000022262	1,85E+00	-3,107657832	1,35E+00	2,46E-01	5,50E-01	Dnah5		3,61	No
ENSMUSG00000022263	1,79E-01	7,53940142	9,18E+00	2,45E-03	3,29E-02	Trio		1,13	Yes
ENSMUSG00000022265	-1,10E-02	6,42452409	3,02E-02	8,62E-01	9,96E-01	Ank		0,99	No
ENSMUSG00000022270	-7,99E-02	5,913069478	1,46E+00	2,27E-01	5,50E-01	Retreg1		0,95	No
ENSMUSG00000022272	-1,37E-01	4,807122385	3,27E+00	7,06E-02	3,36E-01	Myo10		0,91	No
ENSMUSG00000022280	7,50E-02	5,188819886	1,09E+00	2,97E-01	5,93E-01	Rnf19a		1,05	No
ENSMUSG00000022283	-1,23E-01	8,817055091	4,62E+00	3,16E-02	2,06E-01	Pabpc1		0,92	No
ENSMUSG00000022285	7,42E-02	8,490837918	1,67E+00	1,96E-01	5,50E-01	Ywhaz		1,05	No
ENSMUSG00000022286	-3,78E-01	0,438973076	2,44E+00	1,18E-01	4,27E-01	Grhl2		0,77	No
ENSMUSG00000022292	2,50E-01	3,864255025	7,43E+00	6,41E-03	6,74E-02	Rrm2b		1,19	No
ENSMUSG00000022295	1,02E-01	6,52124699	2,66E+00	1,03E-01	3,95E-01	Atp6v1c1		1,07	No
ENSMUSG00000022296	-4,74E-02	-2,896161048	1,45E-03	9,70E-01	1,00E+00	Baalc		0,97	No
ENSMUSG00000022297	2,32E-02	-0,311164403	5,41E-03	9,41E-01	1,00E+00	Fzd6		1,02	No
ENSMUSG00000022299	1,64E-01	3,851514678	3,15E+00	7,61E-02	3,52E-01	Slc25a32		1,12	No
ENSMUSG00000022300	-1,99E-02	4,85769795	6,90E-02	7,93E-01	9,56E-01	Dcaf13		0,99	No
ENSMUSG00000022303	3,78E-01	8,053666369	4,21E+01	8,75E-11	8,37E-09	Dcstamp		1,30	Yes
ENSMUSG00000022304	4,90E-01	2,058814193	1,01E+01	1,49E-03	2,22E-02	Dpys		1,40	Yes
ENSMUSG00000022305	1,49E-01	8,402867433	6,66E+00	9,85E-03	9,22E-02	Lrp12		1,11	No
ENSMUSG00000022306	1,01E+00	-1,146242688	5,11E+00	2,38E-02	1,69E-01	Zfpm2		2,01	No
ENSMUSG00000022307	1,79E-01	5,800363744	7,14E+00	7,54E-03	7,58E-02	Oxr1		1,13	No
ENSMUSG00000022309	-3,53E+00	-2,896127731	5,59E+00	1,81E-02	1,41E-01	Angpt1		0,09	No
ENSMUSG00000022312	-1,57E-01	6,624084012	6,40E+00	1,14E-02	1,03E-01	Eif3h		0,90	No
ENSMUSG00000022313	5,36E-02	3,755197926	3,27E-01	5,68E-01	8,29E-01	Utp23		1,04	No
ENSMUSG00000022314	5,53E-02	7,472361562	8,68E-01	3,51E-01	6,53E-01	Rad21		1,04	No
ENSMUSG00000022321	-6,88E-02	-2,896163841	2,08E-03	9,64E-01	1,00E+00	Cdh10		0,95	No
ENSMUSG00000022322	2,69E-01	4,446618099	1,08E+01	9,89E-04	1,60E-02	Shcbp1		1,20	Yes
ENSMUSG00000022323	-4,70E-01	2,398220392	1,25E+01	4,09E-04	7,76E-03	Rida		0,72	Yes
ENSMUSG00000022324	4,45E-01	0,44815869	3,18E+00	7,46E-02	3,47E-01	Matn2		1,36	No
ENSMUSG00000022325	1,21E-01	3,960663029	1,84E+00	1,75E-01	5,33E-01	Pop1		1,09	No

ENSMUSG00000022329	-4,82E-03	4,42893325	3,51E-03	9,53E-01	1,00E+00	Stk3	1,00	No
ENSMUSG00000022330	-3,83E-02	-3,107651936	-7,91E-11	1,00E+00	1,00E+00	Osr2	0,97	No
ENSMUSG00000022332	4,85E-02	0,045434322	3,15E-02	8,59E-01	9,95E-01	Khdrbs3	1,03	No
ENSMUSG00000022335	6,66E-02	3,312150751	4,10E-01	5,22E-01	8,00E-01	Zfat	1,05	No
ENSMUSG00000022336	-1,60E-01	5,04615325	4,77E+00	2,89E-02	1,93E-01	Eif3e	0,89	No
ENSMUSG00000022337	3,98E-02	4,436300483	2,42E-01	6,23E-01	8,58E-01	Emc2	1,03	No
ENSMUSG00000022338	-2,52E-02	3,136467383	5,34E-02	8,17E-01	9,70E-01	Eny2	0,98	No
ENSMUSG00000022339	6,49E-02	3,474997999	4,19E-01	5,17E-01	7,98E-01	Ebag9	1,05	No
ENSMUSG00000022340	9,01E-03	-3,107646452	-5,16E-10	1,00E+00	1,00E+00	Sybu	1,01	No
ENSMUSG00000022342	-1,89E+00	-3,1076441	1,42E+00	2,33E-01	5,50E-01	Kcnv1	0,27	No
ENSMUSG00000022346	2,35E-01	5,354159367	1,08E+01	9,95E-04	1,61E-02	Myc	1,18	Yes
ENSMUSG00000022350	1,29E-01	6,831037438	4,43E+00	3,52E-02	2,22E-01	Washc5	1,09	No
ENSMUSG00000022351	2,78E-01	6,741175561	2,02E+01	6,87E-06	2,39E-04	Sqle	1,21	Yes
ENSMUSG00000022353	2,15E-01	7,663777266	1,33E+01	2,69E-04	5,55E-03	Mtss1	1,16	Yes
ENSMUSG00000022354	-5,20E-02	5,999436918	6,30E-01	4,27E-01	7,21E-01	Ndufb9	0,96	No
ENSMUSG00000022357	-5,52E-02	-2,497608827	3,83E-03	9,51E-01	1,00E+00	Klhl38	0,96	No
ENSMUSG00000022358	-2,67E-01	4,110879775	9,67E+00	1,87E-03	2,66E-02	Fbxo32	0,83	Yes
ENSMUSG00000022359	-9,53E-02	3,141240542	7,71E-01	3,80E-01	6,81E-01	Wdyhv1	0,94	No
ENSMUSG00000022360	2,61E-01	6,626082175	1,75E+01	2,86E-05	8,42E-04	Atad2	1,20	Yes
ENSMUSG00000022361	1,04E-01	6,2194527	2,63E+00	1,05E-01	3,97E-01	Zhx1	1,07	No
ENSMUSG00000022362	1,43E-02	3,881043918	2,48E-02	8,75E-01	1,00E+00	Gm29394	1,01	No
ENSMUSG00000022364	8,44E-02	4,690346433	1,18E+00	2,78E-01	5,74E-01	Tbc1d31	1,06	No
ENSMUSG00000022365	1,19E-01	6,948214815	3,83E+00	5,02E-02	2,72E-01	Derl1	1,09	No
ENSMUSG00000022367	-3,18E+00	-2,831933991	4,25E+00	3,92E-02	2,35E-01	Has2	0,11	No
ENSMUSG00000022369	3,08E-02	3,677295214	1,05E-01	7,45E-01	9,29E-01	Mtbp	1,02	No
ENSMUSG00000022370	-2,19E-01	4,407898887	7,24E+00	7,12E-03	7,28E-02	Mrpl13	0,86	No
ENSMUSG00000022371	-1,55E+00	-1,548057585	8,22E+00	4,14E-03	4,85E-02	Col14a1	0,34	Yes
ENSMUSG00000022372	4,07E-01	7,60363827	4,71E+01	6,67E-12	7,29E-10	Sla	1,33	Yes
ENSMUSG00000022376	-1,89E+00	-3,1076441	1,42E+00	2,33E-01	5,50E-01	Adcy8	0,27	No
ENSMUSG00000022377	-1,38E-01	8,051794539	5,64E+00	1,76E-02	1,39E-01	Asap1	0,91	No
ENSMUSG00000022378	1,02E-02	6,916443296	2,77E-02	8,68E-01	1,00E+00	Fam49b	1,01	No
ENSMUSG00000022385	2,29E-01	5,038691955	9,70E+00	1,84E-03	2,62E-02	Gtse1	1,17	Yes

ENSMUSG00000022386	-1,39E-01	2,516491914	1,18E+00	2,77E-01	5,74E-01	Trmu	0,91	No
ENSMUSG00000022387	7,27E-02	6,486411831	1,34E+00	2,47E-01	5,50E-01	Brd1	1,05	No
ENSMUSG00000022388	-7,88E-02	-2,89616692	4,36E-03	9,47E-01	1,00E+00	Ttll8	0,95	No
ENSMUSG00000022389	-3,53E-02	5,571586054	2,68E-01	6,05E-01	8,49E-01	Tef	0,98	No
ENSMUSG00000022390	-2,45E-01	5,93408118	1,39E+01	1,93E-04	4,23E-03	Zc3h7b	0,84	Yes
ENSMUSG00000022391	5,94E-02	6,828517907	9,37E-01	3,33E-01	6,33E-01	Rangap1	1,04	No
ENSMUSG00000022394	1,95E-01	4,749703448	6,41E+00	1,13E-02	1,02E-01	L3mbtl2	1,14	No
ENSMUSG00000022400	-8,39E-03	3,968964427	8,84E-03	9,25E-01	1,00E+00	Rbx1	0,99	No
ENSMUSG00000022401	1,87E-01	3,956609372	4,37E+00	3,65E-02	2,27E-01	Xpnpep3	1,14	No
ENSMUSG00000022403	-4,29E-02	6,953424645	4,97E-01	4,81E-01	7,69E-01	St13	0,97	No
ENSMUSG00000022404	-3,28E-02	5,009705833	1,98E-01	6,56E-01	8,80E-01	Slc25a17	0,98	No
ENSMUSG00000022407	6,09E-02	4,938799469	6,62E-01	4,16E-01	7,11E-01	Adsl	1,04	No
ENSMUSG00000022408	2,17E-01	0,251855884	6,16E-01	4,32E-01	7,26E-01	Fam83f	1,16	No
ENSMUSG00000022412	-4,30E-02	5,340798433	3,74E-01	5,41E-01	8,11E-01	Mief1	0,97	No
ENSMUSG00000022414	3,21E-01	4,482558401	1,60E+01	6,43E-05	1,68E-03	Tab1	1,25	Yes
ENSMUSG00000022415	1,66E-01	4,14521705	3,58E+00	5,85E-02	2,99E-01	Syngr1	1,12	No
ENSMUSG00000022416	7,43E-01	-2,896145754	3,01E-01	5,83E-01	8,38E-01	Cacna1i	1,67	No
ENSMUSG00000022419	3,03E-01	5,688000797	1,98E+01	8,63E-06	2,94E-04	Deptor	1,23	Yes
ENSMUSG00000022420	1,10E-01	3,984733301	1,52E+00	2,18E-01	5,50E-01	Dnal4	1,08	No
ENSMUSG00000022421	3,74E-01	2,00153437	6,22E+00	1,26E-02	1,11E-01	Nptxr	1,30	No
ENSMUSG00000022422	6,20E-02	2,128036919	1,96E-01	6,58E-01	8,81E-01	Dscc1	1,04	No
ENSMUSG00000022425	-8,33E-01	-0,770548994	4,63E+00	3,15E-02	2,06E-01	Enpp2	0,56	No
ENSMUSG00000022426	-8,90E-02	5,942250464	1,85E+00	1,74E-01	5,32E-01	Josd1	0,94	No
ENSMUSG00000022427	1,96E-02	6,146359331	9,20E-02	7,62E-01	9,38E-01	Tomm22	1,01	No
ENSMUSG00000022428	2,18E-01	3,208433918	4,00E+00	4,55E-02	2,54E-01	Cby1	1,16	No
ENSMUSG00000022429	-1,16E+00	-2,448691686	1,74E+00	1,87E-01	5,50E-01	Dmc1	0,45	No
ENSMUSG00000022431	1,85E+00	-3,107657832	1,35E+00	2,46E-01	5,50E-01	Ribc2	3,61	No
ENSMUSG00000022432	-1,14E+00	-2,070028454	2,57E+00	1,09E-01	4,05E-01	Smc1b	0,45	No
ENSMUSG00000022433	-4,30E-02	5,365324113	3,71E-01	5,43E-01	8,12E-01	Csnk1e	0,97	No
ENSMUSG00000022434	-1,45E-01	4,673174876	3,51E+00	6,09E-02	3,06E-01	Fam118a	0,90	No
ENSMUSG00000022436	-2,48E-01	6,969024222	1,68E+01	4,24E-05	1,18E-03	Sh3bp1	0,84	Yes
ENSMUSG00000022437	1,43E-02	5,561127562	4,34E-02	8,35E-01	9,80E-01	Samm50	1,01	No

ENSMUSG00000022438	3,12E-01	5,313564165	1,91E+01	1,22E-05	3,99E-04	Parvb		1,24	Yes
ENSMUSG00000022439	9,64E-02	7,207866453	2,57E+00	1,09E-01	4,04E-01	Parvg		1,07	No
ENSMUSG00000022440	-1,65E-01	4,89950445	4,84E+00	2,78E-02	1,88E-01	C1qtnf6		0,89	No
ENSMUSG00000022441	-3,52E-01	-0,015809018	1,47E+00	2,25E-01	5,50E-01	Efcab6		0,78	No
ENSMUSG00000022442	1,82E-02	2,820825669	2,28E-02	8,80E-01	1,00E+00	Ttll1		1,01	No
ENSMUSG00000022443	-1,52E-01	10,72925814	7,33E+00	6,79E-03	7,03E-02	Myh9		0,90	No
ENSMUSG00000022445	-3,23E-01	-2,401352556	1,56E-01	6,92E-01	9,01E-01	Cyp2d26		0,80	No
ENSMUSG00000022449	-1,87E+00	-3,107637761	1,39E+00	2,38E-01	5,50E-01	Adamts20		0,27	No
ENSMUSG00000022450	2,71E-02	5,686389252	1,61E-01	6,88E-01	8,98E-01	Ndufa6		1,02	No
ENSMUSG00000022451	-3,88E-02	6,333817159	3,71E-01	5,43E-01	8,12E-01	Twf1		0,97	No
ENSMUSG00000022452	3,99E-02	6,74114433	4,17E-01	5,19E-01	7,98E-01	Smdt1		1,03	No
ENSMUSG00000022453	2,16E-01	7,79389645	1,35E+01	2,40E-04	5,06E-03	Naga		1,16	Yes
ENSMUSG00000022455	-8,56E-01	-2,832005432	4,01E-01	5,26E-01	8,03E-01	Wbp2nl		0,55	No
ENSMUSG00000022456	6,49E-01	6,015130372	9,41E+01	3,01E-22	8,67E-20	37865		1,57	Yes
ENSMUSG00000022462	1,73E-01	6,902746444	8,11E+00	4,40E-03	5,07E-02	Slc38a2		1,13	No
ENSMUSG00000022463	1,44E-01	8,355395456	6,20E+00	1,28E-02	1,12E-01	Srebf2		1,10	No
ENSMUSG00000022464	2,64E+00	-2,963240788	2,69E+00	1,01E-01	3,92E-01	Slc38a4		6,24	No
ENSMUSG00000022466	1,60E-02	3,008768117	2,01E-02	8,87E-01	1,00E+00	Rpap3		1,01	No
ENSMUSG00000022469	1,63E-01	-2,522620202	2,57E-02	8,73E-01	1,00E+00	Rapgef3		1,12	No
ENSMUSG00000022471	1,17E-01	4,157131133	1,83E+00	1,76E-01	5,35E-01	Xrcc6		1,08	No
ENSMUSG00000022472	-2,21E-02	4,664772088	8,04E-02	7,77E-01	9,46E-01	Desi1		0,98	No
ENSMUSG00000022474	-2,70E-01	3,802923828	8,62E+00	3,33E-03	4,14E-02	Pmm1		0,83	Yes
ENSMUSG00000022475	4,71E-02	3,306488418	2,05E-01	6,51E-01	8,76E-01	Hdac7		1,03	No
ENSMUSG00000022476	6,01E-03	3,827215129	4,26E-03	9,48E-01	1,00E+00	Polr3h		1,00	No
ENSMUSG00000022477	-3,60E-02	7,134783508	3,57E-01	5,50E-01	8,17E-01	Aco2		0,98	No
ENSMUSG00000022479	-3,60E-01	6,937256627	3,52E+01	3,01E-09	2,29E-07	Vdr		0,78	Yes
ENSMUSG00000022483	4,32E-01	-2,655193999	1,45E-01	7,03E-01	9,06E-01	Col2a1		1,35	No
ENSMUSG00000022484	-1,89E+00	-3,1076441	1,42E+00	2,33E-01	5,50E-01	Hoxc10		0,27	No
ENSMUSG00000022487	-6,83E-02	-2,896110758	2,05E-03	9,64E-01	1,00E+00	Gtsf1		0,95	No
ENSMUSG00000022488	-2,19E-01	9,535813722	1,51E+01	1,04E-04	2,50E-03	Nckap1l		0,86	Yes
ENSMUSG00000022489	-1,82E-01	6,393516275	8,34E+00	3,88E-03	4,62E-02	Pde1b		0,88	Yes
ENSMUSG00000022490	-1,19E-01	2,195526838	6,76E-01	4,11E-01	7,07E-01	Ppp1r1a		0,92	No

ENSMUSG00000022491	-7,76E-02	-2,963273445	2,73E-03	9,58E-01	1,00E+00	Glycam1	0,95	No
ENSMUSG00000022494	-2,94E-01	-2,268118813	1,50E-01	6,98E-01	9,04E-01	Shisa9	0,82	No
ENSMUSG00000022496	-1,98E-02	-3,033655952	2,83E-11	1,00E+00	1,00E+00	Tnfrsf17	0,99	No
ENSMUSG00000022498	7,45E-02	5,816161867	1,25E+00	2,64E-01	5,59E-01	Txndc11	1,05	No
ENSMUSG00000022500	8,67E-02	9,370336841	2,34E+00	1,26E-01	4,44E-01	Litaf	1,06	No
ENSMUSG00000022501	-1,10E+00	-2,424839297	1,92E+00	1,66E-01	5,18E-01	Prm1	0,47	No
ENSMUSG00000022503	-4,45E-02	5,168509209	3,80E-01	5,38E-01	8,09E-01	Nubp1	0,97	No
ENSMUSG00000022504	2,02E-01	8,306824415	1,22E+01	4,68E-04	8,69E-03	Ciita	1,15	Yes
ENSMUSG00000022505	9,96E-02	5,266714463	1,95E+00	1,62E-01	5,12E-01	Emp2	1,07	No
ENSMUSG00000022507	-2,99E-02	5,800324424	2,01E-01	6,54E-01	8,79E-01	1810013L24Rik	0,98	No
ENSMUSG00000022508	1,54E-01	6,015548	5,55E+00	1,85E-02	1,44E-01	Bcl6	1,11	No
ENSMUSG00000022510	-1,89E+00	-3,1076441	1,42E+00	2,33E-01	5,50E-01	Trp63	0,27	No
ENSMUSG00000022512	2,30E-01	6,694984488	1,38E+01	2,06E-04	4,45E-03	Cldn1	1,17	Yes
ENSMUSG00000022514	2,05E-01	5,042947999	7,57E+00	5,94E-03	6,37E-02	Il1rap	1,15	No
ENSMUSG00000022515	2,33E-01	5,200524397	1,07E+01	1,09E-03	1,74E-02	Anks3	1,18	Yes
ENSMUSG00000022516	-1,99E-01	4,818601019	6,87E+00	8,79E-03	8,49E-02	Nudt16l1	0,87	No
ENSMUSG00000022517	1,00E-01	6,492597739	2,54E+00	1,11E-01	4,10E-01	Mgrn1	1,07	No
ENSMUSG00000022518	3,74E-01	0,206592838	1,87E+00	1,72E-01	5,28E-01	4930562C15Rik	1,30	No
ENSMUSG00000022519	1,18E-01	1,405892715	4,34E-01	5,10E-01	7,93E-01	Srl	1,08	No
ENSMUSG00000022521	-1,70E-01	6,279144491	7,06E+00	7,86E-03	7,80E-02	Crebbp	0,89	No
ENSMUSG00000022523	1,85E+00	-3,107657832	1,35E+00	2,46E-01	5,50E-01	Fgf12	3,61	No
ENSMUSG00000022526	1,92E-01	3,272447146	3,51E+00	6,11E-02	3,07E-01	Zfp251	1,14	No
ENSMUSG00000022528	-3,60E-01	-2,185503636	1,63E-01	6,87E-01	8,97E-01	Hes1	0,78	No
ENSMUSG00000022529	-1,07E-01	5,878010544	2,64E+00	1,04E-01	3,97E-01	Zfp263	0,93	No
ENSMUSG00000022533	7,56E-02	7,991800621	1,69E+00	1,94E-01	5,50E-01	Atp13a3	1,05	No
ENSMUSG00000022534	-3,46E-01	1,711404013	4,49E+00	3,41E-02	2,17E-01	Mefv	0,79	No
ENSMUSG00000022536	-6,63E-02	7,456791306	1,25E+00	2,63E-01	5,58E-01	Glyr1	0,96	No
ENSMUSG00000022537	-1,73E+00	-2,548213078	2,08E+00	1,49E-01	4,90E-01	Tmem44	0,30	No
ENSMUSG00000022538	6,79E-02	5,145808722	8,74E-01	3,50E-01	6,51E-01	Lsg1	1,05	No
ENSMUSG00000022540	-5,39E-01	7,073226583	7,97E+01	4,30E-19	9,27E-17	Rogdi	0,69	Yes
ENSMUSG00000022543	7,74E-02	-0,669029766	4,12E-02	8,39E-01	9,83E-01	4930451G09Rik	1,06	No
ENSMUSG00000022544	2,87E-01	4,144306861	1,11E+01	8,74E-04	1,45E-02	Eef2kmt	1,22	Yes

ENSMUSG00000022545	4,60E-02	4,800430611	3,62E-01	5,47E-01	8,15E-01	Ercc4	1,03	No
ENSMUSG00000022546	1,24E-01	1,901542298	6,79E-01	4,10E-01	7,06E-01	Gpt	1,09	No
ENSMUSG00000022548	-8,37E-01	-2,832007507	3,84E-01	5,35E-01	8,08E-01	Apod	0,56	No
ENSMUSG00000022550	1,01E-02	3,315337903	9,52E-03	9,22E-01	1,00E+00	Adck5	1,01	No
ENSMUSG00000022551	2,44E-02	6,720660278	1,56E-01	6,93E-01	9,01E-01	Cyc1	1,02	No
ENSMUSG00000022552	-8,03E-02	5,818528915	1,45E+00	2,28E-01	5,50E-01	Sharpin	0,95	No
ENSMUSG00000022553	-5,43E-02	5,958242832	6,83E-01	4,09E-01	7,05E-01	Maf1	0,96	No
ENSMUSG00000022554	1,54E-01	2,649414589	1,57E+00	2,10E-01	5,50E-01	Hgh1	1,11	No
ENSMUSG00000022555	-1,60E-01	3,892540622	3,15E+00	7,59E-02	3,51E-01	Dgat1	0,90	No
ENSMUSG00000022556	1,56E-01	4,684101145	4,01E+00	4,53E-02	2,54E-01	Hsf1	1,11	No
ENSMUSG00000022557	7,56E-04	5,454502856	1,19E-04	9,91E-01	1,00E+00	Bop1	1,00	No
ENSMUSG00000022558	1,55E-01	7,293747375	6,71E+00	9,60E-03	9,04E-02	Mroh1	1,11	No
ENSMUSG00000022559	2,59E-02	4,766808206	1,14E-01	7,36E-01	9,24E-01	Fbxl6	1,02	No
ENSMUSG00000022560	5,36E-02	4,258015285	4,02E-01	5,26E-01	8,03E-01	Slc52a2	1,04	No
ENSMUSG00000022561	6,72E-02	5,534445042	9,50E-01	3,30E-01	6,30E-01	Gpaa1	1,05	No
ENSMUSG00000022562	-3,40E-01	2,130915124	5,49E+00	1,91E-02	1,47E-01	Oplah	0,79	No
ENSMUSG00000022564	-2,08E-02	7,230925674	1,20E-01	7,29E-01	9,20E-01	Grina	0,99	No
ENSMUSG00000022565	2,98E-01	8,873308599	2,72E+01	1,85E-07	9,69E-06	Plec	1,23	Yes
ENSMUSG00000022568	7,35E-02	5,729159617	1,18E+00	2,76E-01	5,73E-01	Scrib	1,05	No
ENSMUSG00000022570	-1,73E-01	4,799022511	5,13E+00	2,35E-02	1,68E-01	Tsta3	0,89	No
ENSMUSG00000022571	5,64E-02	3,989914955	4,00E-01	5,27E-01	8,04E-01	Pycrl	1,04	No
ENSMUSG00000022574	-7,58E-01	1,545705693	2,09E+01	4,75E-06	1,72E-04	Naprt	0,59	Yes
ENSMUSG00000022575	-2,55E-01	6,863986235	1,73E+01	3,16E-05	9,16E-04	Gsdmd	0,84	Yes
ENSMUSG00000022580	-8,79E-01	-0,27869004	7,07E+00	7,85E-03	7,79E-02	Rhpn1	0,54	No
ENSMUSG00000022582	8,56E-01	-2,770602728	6,41E-01	4,23E-01	7,18E-01	Ly6g	1,81	No
ENSMUSG00000022584	2,35E-01	4,584460483	8,61E+00	3,34E-03	4,15E-02	Ly6c2	1,18	Yes
ENSMUSG00000022586	-8,79E-01	1,89984329	3,01E+01	4,20E-08	2,55E-06	Ly6i	0,54	Yes
ENSMUSG00000022587	3,21E-02	9,041925488	3,18E-01	5,73E-01	8,32E-01	Ly6e	1,02	No
ENSMUSG00000022591	-5,88E-02	-3,033634602	1,62E-03	9,68E-01	1,00E+00	Gm9747	0,96	No
ENSMUSG00000022594	1,35E-01	0,661338301	3,14E-01	5,75E-01	8,34E-01	Lynx1	1,10	No
ENSMUSG00000022596	-8,09E-02	-3,107638307	2,93E-03	9,57E-01	1,00E+00	Slurp1	0,95	No
ENSMUSG00000022598	-1,36E+00	-2,185623112	2,55E+00	1,10E-01	4,07E-01	Psca	0,39	No

ENSMUSG00000022601	9,21E-02	4,632193038	1,38E+00	2,39E-01	5,50E-01	Zbtb11	1,07	No
ENSMUSG00000022602	-4,86E-01	4,454285181	3,77E+01	8,38E-10	6,94E-08	Arc	0,71	Yes
ENSMUSG00000022604	2,14E-01	1,950530153	2,10E+00	1,48E-01	4,87E-01	Cep97	1,16	No
ENSMUSG00000022607	-1,82E-01	4,205889512	4,65E+00	3,11E-02	2,04E-01	Ptk2	0,88	No
ENSMUSG00000022610	6,85E-01	-2,896150766	2,55E-01	6,14E-01	8,53E-01	Mapk12	1,61	No
ENSMUSG00000022614	8,38E-02	5,766981651	1,55E+00	2,13E-01	5,50E-01	Lmf2	1,06	No
ENSMUSG00000022615	2,11E-01	3,25358337	3,92E+00	4,77E-02	2,62E-01	Tymp	1,16	No
ENSMUSG00000022617	4,68E-02	5,322597379	4,35E-01	5,10E-01	7,93E-01	Chkb	1,03	No
ENSMUSG00000022619	-7,69E-02	-2,963220349	2,69E-03	9,59E-01	1,00E+00	Mapk8ip2	0,95	No
ENSMUSG00000022620	6,32E-02	5,665806318	8,69E-01	3,51E-01	6,53E-01	Arsa	1,04	No
ENSMUSG00000022621	1,40E-01	1,920316311	8,20E-01	3,65E-01	6,66E-01	Rabl2	1,10	No
ENSMUSG00000022623	7,66E-01	-2,963249872	3,22E-01	5,70E-01	8,31E-01	Shank3	1,70	No
ENSMUSG00000022629	9,60E-02	-0,711772159	5,69E-02	8,12E-01	9,67E-01	Kif21a	1,07	No
ENSMUSG00000022634	-1,00E-01	4,391466232	1,47E+00	2,25E-01	5,50E-01	Yaf2	0,93	No
ENSMUSG00000022635	-1,49E-01	4,858967897	3,94E+00	4,73E-02	2,60E-01	Zcrb1	0,90	No
ENSMUSG00000022636	2,82E-01	7,699005901	2,29E+01	1,72E-06	7,17E-05	Alcam	1,22	Yes
ENSMUSG00000022637	-3,60E-01	5,781506485	2,93E+01	6,25E-08	3,65E-06	Cblb	0,78	Yes
ENSMUSG00000022639	2,67E-01	5,171997506	1,38E+01	2,04E-04	4,43E-03	Dubr	1,20	Yes
ENSMUSG00000022641	2,32E-02	5,250029624	1,06E-01	7,45E-01	9,29E-01	Bbx	1,02	No
ENSMUSG00000022650	-1,94E+00	-3,107619243	1,50E+00	2,21E-01	5,50E-01	Retnlb	0,26	No
ENSMUSG00000022651	-3,18E+00	-2,770591119	4,25E+00	3,92E-02	2,35E-01	Retnlg	0,11	No
ENSMUSG00000022652	-2,68E+00	-3,033645766	2,84E+00	9,17E-02	3,82E-01	Morc1	0,16	No
ENSMUSG00000022656	-1,18E+00	-2,185582663	2,76E+00	9,69E-02	3,88E-01	Nectin3	0,44	No
ENSMUSG00000022657	-1,45E+00	-2,226308158	3,74E+00	5,30E-02	2,81E-01	Cd96	0,37	No
ENSMUSG00000022658	7,67E-01	-2,963261668	3,24E-01	5,69E-01	8,30E-01	Tagln3	1,70	No
ENSMUSG00000022659	-1,47E-02	-1,847721765	6,70E-04	9,79E-01	1,00E+00	Gcsam	0,99	No
ENSMUSG00000022661	-5,36E-01	4,145766908	4,04E+01	2,06E-10	1,85E-08	Cd200	0,69	Yes
ENSMUSG00000022663	1,63E-01	5,945422465	6,13E+00	1,33E-02	1,16E-01	Atg3	1,12	No
ENSMUSG00000022664	3,58E-01	5,000180844	2,33E+01	1,40E-06	5,97E-05	Slc35a5	1,28	Yes
ENSMUSG00000022665	6,02E-01	6,618904884	9,13E+01	1,27E-21	3,33E-19	Ccdc80	1,52	Yes
ENSMUSG00000022667	3,13E-01	7,795751802	2,85E+01	9,29E-08	5,25E-06	Cd200r1	1,24	Yes
ENSMUSG00000022668	1,32E-01	4,364798108	2,63E+00	1,05E-01	3,97E-01	Gtpbp8	1,10	No

ENSMUSG00000022671	-1,07E-01	3,220535757	1,01E+00	3,14E-01	6,14E-01	Mzt2	0,93	No
ENSMUSG00000022672	-1,08E-02	5,019444387	2,14E-02	8,84E-01	1,00E+00	Prkdc	0,99	No
ENSMUSG00000022673	1,98E-01	6,448559378	9,86E+00	1,69E-03	2,45E-02	Mcm4	1,15	Yes
ENSMUSG00000022674	1,38E-01	3,530877439	1,98E+00	1,59E-01	5,06E-01	Ube2v2	1,10	No
ENSMUSG00000022676	4,37E-01	-2,655117831	1,49E-01	6,99E-01	9,04E-01	Snai2	1,35	No
ENSMUSG00000022677	2,85E-02	4,316443222	1,17E-01	7,33E-01	9,23E-01	Fopnl	1,02	No
ENSMUSG00000022678	5,86E-02	5,95169536	8,03E-01	3,70E-01	6,72E-01	Nde1	1,04	No
ENSMUSG00000022679	4,20E-01	0,489885253	2,80E+00	9,42E-02	3,84E-01	Mpv17l	1,34	No
ENSMUSG00000022680	6,48E-02	5,635319483	9,08E-01	3,41E-01	6,41E-01	Pdxdc1	1,05	No
ENSMUSG00000022681	-7,01E-03	4,931283493	8,79E-03	9,25E-01	1,00E+00	Ntan1	1,00	No
ENSMUSG00000022682	2,57E-02	5,530523381	1,40E-01	7,09E-01	9,09E-01	Rrn3	1,02	No
ENSMUSG00000022683	1,23E+00	-2,896126345	9,34E-01	3,34E-01	6,34E-01	Pla2g10	2,34	No
ENSMUSG00000022684	6,33E-02	5,592191574	8,63E-01	3,53E-01	6,54E-01	Bfar	1,04	No
ENSMUSG00000022685	-4,92E-02	3,589838742	2,61E-01	6,09E-01	8,51E-01	Parn	0,97	No
ENSMUSG00000022686	-7,72E-01	2,990849102	4,95E+01	1,98E-12	2,31E-10	B3gnt5	0,59	Yes
ENSMUSG00000022687	6,05E-01	-1,126881387	1,50E+00	2,21E-01	5,50E-01	Boc	1,52	No
ENSMUSG00000022696	-8,24E-01	-2,711703956	3,73E-01	5,41E-01	8,11E-01	Sidt1	0,57	No
ENSMUSG00000022698	1,70E-01	7,222928304	7,98E+00	4,72E-03	5,35E-02	Naa50	1,12	No
ENSMUSG00000022701	1,72E-01	2,422045581	1,72E+00	1,90E-01	5,50E-01	Ccdc191	1,13	No
ENSMUSG00000022702	6,78E-02	4,995930924	8,38E-01	3,60E-01	6,61E-01	Hira	1,05	No
ENSMUSG00000022704	3,72E-01	3,240446261	1,22E+01	4,82E-04	8,91E-03	Qtrt2	1,29	Yes
ENSMUSG00000022706	-4,16E-02	4,779774188	2,93E-01	5,88E-01	8,40E-01	Mrpl40	0,97	No
ENSMUSG00000022707	-1,96E-02	3,84270991	4,52E-02	8,32E-01	9,78E-01	Gbe1	0,99	No
ENSMUSG00000022708	-3,78E-02	4,796430515	2,46E-01	6,20E-01	8,57E-01	Zbtb20	0,97	No
ENSMUSG00000022710	1,78E-02	6,933920421	8,50E-02	7,71E-01	9,42E-01	Usp7	1,01	No
ENSMUSG00000022711	1,10E-01	5,764305583	2,69E+00	1,01E-01	3,93E-01	Pmm2	1,08	No
ENSMUSG00000022715	4,73E-01	-2,226335835	3,04E-01	5,82E-01	8,38E-01	Tmem114	1,39	No
ENSMUSG00000022718	2,08E-01	5,105000976	8,18E+00	4,24E-03	4,94E-02	Dgcr8	1,15	Yes
ENSMUSG00000022721	4,87E-02	5,779262246	5,30E-01	4,67E-01	7,55E-01	Trmt2a	1,03	No
ENSMUSG00000022722	-4,28E-02	2,807401366	1,34E-01	7,15E-01	9,12E-01	Arl6	0,97	No
ENSMUSG00000022723	-9,88E-03	5,639662787	2,10E-02	8,85E-01	1,00E+00	Crybg3	0,99	No
ENSMUSG00000022724	6,30E-02	4,668288263	6,58E-01	4,17E-01	7,12E-01	Riox2	1,04	No

ENSMUSG00000022738	1,60E+00	-2,711721439	1,76E+00	1,85E-01	5,49E-01	Gsc2	3,03	No
ENSMUSG00000022742	-2,27E-01	5,857978225	1,17E+01	6,10E-04	1,08E-02	Cpox	0,85	Yes
ENSMUSG00000022744	-2,67E-01	8,038545089	2,10E+01	4,51E-06	1,65E-04	Cldnd1	0,83	Yes
ENSMUSG00000022747	2,40E-02	1,925252897	2,49E-02	8,75E-01	1,00E+00	St3gal6	1,02	No
ENSMUSG00000022748	1,78E-01	0,574987291	5,39E-01	4,63E-01	7,51E-01	Cmss1	1,13	No
ENSMUSG00000022749	-6,51E-02	5,612342119	9,10E-01	3,40E-01	6,41E-01	Tbc1d23	0,96	No
ENSMUSG00000022750	-9,03E-02	4,131365613	1,10E+00	2,93E-01	5,89E-01	Klhl22	0,94	No
ENSMUSG00000022751	1,89E-01	2,81996138	2,59E+00	1,08E-01	4,02E-01	Nit2	1,14	No
ENSMUSG00000022752	3,84E-02	6,523781527	3,78E-01	5,39E-01	8,09E-01	Tomm70a	1,03	No
ENSMUSG00000022753	3,14E-02	-3,107617333	-7,69E-10	1,00E+00	1,00E+00	Tmem30c	1,02	No
ENSMUSG00000022754	-1,94E+00	-3,107619243	1,50E+00	2,21E-01	5,50E-01	Tmem45a	0,26	No
ENSMUSG00000022755	3,82E+00	-2,655144808	6,73E+00	9,48E-03	8,98E-02	Adgrg7	14,10	No
ENSMUSG00000022756	1,06E-01	2,068693246	4,93E-01	4,82E-01	7,70E-01	Slc7a4	1,08	No
ENSMUSG00000022757	1,25E-02	6,321998095	3,87E-02	8,44E-01	9,86E-01	Tfg	1,01	No
ENSMUSG00000022758	1,42E-01	-0,412999667	1,62E-01	6,88E-01	8,98E-01	P2rx6	1,10	No
ENSMUSG00000022759	-9,44E-01	-1,929196503	2,20E+00	1,38E-01	4,69E-01	Lrrc74b	0,52	No
ENSMUSG00000022760	5,91E-02	4,24133905	4,89E-01	4,84E-01	7,72E-01	Thap7	1,04	No
ENSMUSG00000022761	-3,39E-03	5,846756337	2,62E-03	9,59E-01	1,00E+00	Lztr1	1,00	No
ENSMUSG00000022762	1,22E+00	-1,185651198	5,80E+00	1,60E-02	1,32E-01	Ncam2	2,32	No
ENSMUSG00000022763	-9,62E-01	-0,097779683	1,07E+01	1,06E-03	1,70E-02	Aifm3	0,51	Yes
ENSMUSG00000022765	2,31E-02	6,668355824	1,39E-01	7,09E-01	9,09E-01	Snap29	1,02	No
ENSMUSG00000022766	-6,84E-01	-1,9292547	1,07E+00	3,01E-01	5,98E-01	Serpind1	0,62	No
ENSMUSG00000022768	8,11E-02	0,866025628	1,39E-01	7,09E-01	9,09E-01	Ccdc116	1,06	No
ENSMUSG00000022769	1,20E-01	6,973214069	3,89E+00	4,85E-02	2,65E-01	Sdf2l1	1,09	No
ENSMUSG00000022770	-2,14E-02	6,082344173	1,08E-01	7,42E-01	9,27E-01	Dlg1	0,99	No
ENSMUSG00000022771	7,31E-02	4,928968562	9,63E-01	3,26E-01	6,27E-01	Ppil2	1,05	No
ENSMUSG00000022772	4,53E-02	5,172658448	3,98E-01	5,28E-01	8,04E-01	Senp5	1,03	No
ENSMUSG00000022773	-2,42E-01	2,649781188	3,93E+00	4,73E-02	2,60E-01	Ypel1	0,85	No
ENSMUSG00000022774	1,17E-01	5,571462296	2,91E+00	8,80E-02	3,79E-01	Ncbp2	1,08	No
ENSMUSG00000022779	-1,24E-01	5,625559875	3,35E+00	6,73E-02	3,27E-01	Top3b	0,92	No
ENSMUSG00000022781	3,77E-02	7,746738878	4,13E-01	5,21E-01	7,99E-01	Pak2	1,03	No
ENSMUSG00000022783	-1,87E+00	-3,107637761	1,39E+00	2,38E-01	5,50E-01	Spag6l	0,27	No

ENSMUSG00000022787	-8,37E-02	3,405375709	6,92E-01	4,05E-01	7,02E-01	Wdr53	0,94	No
ENSMUSG00000022788	1,06E-01	5,218478676	2,17E+00	1,41E-01	4,74E-01	Fgd4	1,08	No
ENSMUSG00000022789	-5,53E-02	6,766179062	8,10E-01	3,68E-01	6,69E-01	Dnm1l	0,96	No
ENSMUSG00000022790	7,75E-01	-2,711631285	3,25E-01	5,69E-01	8,30E-01	Igsf11	1,71	No
ENSMUSG00000022791	-2,26E-02	2,485358119	2,89E-02	8,65E-01	9,98E-01	Tnk2	0,98	No
ENSMUSG00000022792	3,02E-01	3,573036977	9,51E+00	2,04E-03	2,84E-02	Yars2	1,23	Yes
ENSMUSG00000022793	2,92E-01	3,100454976	7,09E+00	7,77E-03	7,72E-02	B4galt4	1,22	No
ENSMUSG00000022797	3,85E-01	6,684378071	3,87E+01	4,99E-10	4,29E-08	Tfrc	1,31	Yes
ENSMUSG00000022799	2,57E-02	8,088613267	1,97E-01	6,57E-01	8,80E-01	Arhgap31	1,02	No
ENSMUSG00000022800	-8,77E-02	5,826003246	1,73E+00	1,88E-01	5,50E-01	Fyttd1	0,94	No
ENSMUSG00000022801	-1,46E-01	5,804605169	4,79E+00	2,86E-02	1,92E-01	Lrch3	0,90	No
ENSMUSG00000022802	4,96E-01	3,413520578	2,36E+01	1,20E-06	5,23E-05	Lmln	1,41	Yes
ENSMUSG00000022803	-4,49E-01	-0,528841327	1,53E+00	2,16E-01	5,50E-01	Popdc2	0,73	No
ENSMUSG00000022805	-7,98E-02	-2,497578469	5,75E-03	9,40E-01	1,00E+00	Maats1	0,95	No
ENSMUSG00000022807	1,35E-01	5,542339842	3,85E+00	4,96E-02	2,69E-01	Osbpl11	1,10	No
ENSMUSG00000022808	2,14E-02	5,844261102	1,03E-01	7,48E-01	9,30E-01	Snx4	1,01	No
ENSMUSG00000022809	2,40E-02	-2,896127038	-3,28E-11	1,00E+00	1,00E+00	Nr1i2	1,02	No
ENSMUSG00000022811	2,01E-02	5,976489578	9,40E-02	7,59E-01	9,36E-01	Zfp148	1,01	No
ENSMUSG00000022812	-1,75E-02	6,909335498	8,13E-02	7,75E-01	9,45E-01	Gsk3b	0,99	No
ENSMUSG00000022814	7,50E-02	5,250324886	1,10E+00	2,93E-01	5,89E-01	Umps	1,05	No
ENSMUSG00000022816	1,87E-01	5,10096795	6,32E+00	1,19E-02	1,06E-01	Fstl1	1,14	No
ENSMUSG00000022817	2,48E-01	6,626242263	1,58E+01	7,13E-05	1,82E-03	Itgb5	1,19	Yes
ENSMUSG00000022818	6,97E-01	2,957061103	3,59E+01	2,06E-09	1,60E-07	Cyp2ab1	1,62	Yes
ENSMUSG00000022820	-1,50E-01	2,409516082	1,34E+00	2,48E-01	5,50E-01	Ndufb4	0,90	No
ENSMUSG00000022821	-1,46E+00	-2,548201536	2,21E+00	1,37E-01	4,66E-01	Hgd	0,36	No
ENSMUSG00000022822	1,92E-02	7,26562002	1,03E-01	7,49E-01	9,30E-01	Abcc5	1,01	No
ENSMUSG00000022824	-1,01E+00	-2,185581928	1,57E+00	2,10E-01	5,50E-01	Muc13	0,50	No
ENSMUSG00000022827	-1,17E-01	3,91390686	1,62E+00	2,04E-01	5,50E-01	Rabl3	0,92	No
ENSMUSG00000022828	1,14E-01	4,227279186	1,84E+00	1,75E-01	5,33E-01	Gtf2e1	1,08	No
ENSMUSG00000022829	1,81E+00	-3,10765737	1,28E+00	2,58E-01	5,50E-01	Stxbp5l	3,49	No
ENSMUSG00000022831	-1,75E-02	7,885350789	9,00E-02	7,64E-01	9,39E-01	Hcls1	0,99	No
ENSMUSG00000022832	9,01E-03	-3,107646452	-5,16E-10	1,00E+00	1,00E+00	Ropn1	1,01	No

ENSMUSG00000022833	2,79E-01	2,730010935	5,33E+00	2,09E-02	1,56E-01	Ccdc14	1,21	No
ENSMUSG00000022836	-8,78E-01	5,118495769	1,47E+02	7,81E-34	5,57E-31	Mylk	0,54	Yes
ENSMUSG00000022837	-4,45E-01	2,022824108	8,35E+00	3,85E-03	4,60E-02	Iqcb1	0,73	Yes
ENSMUSG00000022838	-2,76E-01	-0,997982938	4,18E-01	5,18E-01	7,98E-01	Eaf2	0,83	No
ENSMUSG00000022840	3,61E-01	-1,289367948	6,34E-01	4,26E-01	7,20E-01	Adcy5	1,28	No
ENSMUSG00000022841	7,98E-02	7,195273527	1,76E+00	1,84E-01	5,48E-01	Ap2m1	1,06	No
ENSMUSG00000022842	2,08E-01	4,358139119	6,24E+00	1,25E-02	1,10E-01	Ece2	1,15	No
ENSMUSG00000022843	-1,55E-03	0,732041387	4,55E-05	9,95E-01	1,00E+00	Clcn2	1,00	No
ENSMUSG00000022844	2,72E-01	1,601015614	2,61E+00	1,06E-01	3,99E-01	Pdia5	1,21	No
ENSMUSG00000022848	8,74E-03	5,690523478	1,68E-02	8,97E-01	1,00E+00	Dirc2	1,01	No
ENSMUSG00000022849	-4,94E-02	4,057137748	3,26E-01	5,68E-01	8,30E-01	Hspbap1	0,97	No
ENSMUSG00000022853	-5,36E-02	3,525987834	3,00E-01	5,84E-01	8,38E-01	Ehhadh	0,96	No
ENSMUSG00000022855	1,39E-01	6,238263521	4,69E+00	3,03E-02	2,00E-01	Senp2	1,10	No
ENSMUSG00000022856	1,47E-01	3,34040658	1,97E+00	1,61E-01	5,09E-01	Tmem41a	1,11	No
ENSMUSG00000022858	5,42E-02	6,487522001	7,48E-01	3,87E-01	6,87E-01	Tra2b	1,04	No
ENSMUSG00000022861	3,34E-01	1,202894971	2,87E+00	9,04E-02	3,81E-01	Dgkg	1,26	No
ENSMUSG00000022863	-6,76E-02	2,178896152	2,13E-01	6,45E-01	8,73E-01	Btg3	0,95	No
ENSMUSG00000022864	-1,83E-01	3,932536763	3,91E+00	4,79E-02	2,63E-01	D16Ertd472e	0,88	No
ENSMUSG00000022865	-1,95E-01	-1,344148276	1,54E-01	6,95E-01	9,02E-01	Cxadr	0,87	No
ENSMUSG00000022867	5,48E-02	6,756922633	7,91E-01	3,74E-01	6,75E-01	Usp25	1,04	No
ENSMUSG00000022871	-3,81E-01	-2,31118857	1,83E-01	6,69E-01	8,87E-01	Fetub	0,77	No
ENSMUSG00000022875	-1,03E-01	-2,831985655	7,40E-03	9,31E-01	1,00E+00	Kng1	0,93	No
ENSMUSG00000022876	-6,91E-01	6,784555783	1,27E+02	2,23E-29	1,13E-26	Samsn1	0,62	Yes
ENSMUSG00000022878	-7,22E-02	-2,655052582	3,49E-03	9,53E-01	1,00E+00	Adipoq	0,95	No
ENSMUSG00000022881	-9,03E-02	2,962878093	6,28E-01	4,28E-01	7,22E-01	Rfc4	0,94	No
ENSMUSG00000022883	-7,10E-02	-2,832035322	3,45E-03	9,53E-01	1,00E+00	Robo1	0,95	No
ENSMUSG00000022884	-3,17E-01	7,757753304	2,91E+01	6,89E-08	3,98E-06	Eif4a2	0,80	Yes
ENSMUSG00000022885	4,52E-01	6,077825144	4,77E+01	4,88E-12	5,52E-10	St6gal1	1,37	Yes
ENSMUSG00000022887	6,97E-01	-1,963296861	8,66E-01	3,52E-01	6,53E-01	Masp1	1,62	No
ENSMUSG00000022889	-2,05E-01	4,313913873	6,07E+00	1,38E-02	1,18E-01	Mrpl39	0,87	No
ENSMUSG00000022890	-9,03E-02	6,548532758	2,08E+00	1,49E-01	4,89E-01	Atp5j	0,94	No
ENSMUSG00000022892	8,14E-04	9,302978044	2,06E-04	9,89E-01	1,00E+00	App	1,00	No

ENSMUSG00000022893	-4,98E-03	-0,051841254	2,69E-04	9,87E-01	1,00E+00	Adamts1	1,00	No
ENSMUSG00000022895	-1,85E-01	6,699197751	8,78E+00	3,05E-03	3,87E-02	Ets2	0,88	Yes
ENSMUSG00000022897	-1,66E-02	6,833449129	7,31E-02	7,87E-01	9,53E-01	Dyrk1a	0,99	No
ENSMUSG00000022898	9,34E-02	6,628449093	2,27E+00	1,32E-01	4,56E-01	Dscr3	1,07	No
ENSMUSG00000022899	-4,63E-01	2,954130096	1,72E+01	3,30E-05	9,53E-04	Slc15a2	0,73	Yes
ENSMUSG00000022900	-8,38E-02	1,334675384	1,99E-01	6,56E-01	8,79E-01	Ildr1	0,94	No
ENSMUSG00000022901	-6,04E-01	7,055694375	9,93E+01	2,15E-23	6,78E-21	Cd86	0,66	Yes
ENSMUSG00000022902	1,60E+00	0,872412319	4,61E+01	1,13E-11	1,18E-09	Stfa2	3,04	Yes
ENSMUSG00000022905	4,78E-02	6,360627766	5,66E-01	4,52E-01	7,41E-01	Kpna1	1,03	No
ENSMUSG00000022906	-1,45E-01	5,998968394	4,94E+00	2,62E-02	1,81E-01	Parp9	0,90	No
ENSMUSG00000022911	-3,67E-02	3,466662592	1,33E-01	7,15E-01	9,13E-01	Arl13b	0,97	No
ENSMUSG00000022912	2,53E-01	6,265249507	1,54E+01	8,48E-05	2,12E-03	Pros1	1,19	Yes
ENSMUSG00000022913	-3,09E-03	3,397614345	9,05E-04	9,76E-01	1,00E+00	Psmg1	1,00	No
ENSMUSG00000022914	8,49E-02	5,883672952	1,64E+00	2,00E-01	5,50E-01	Brwd1	1,06	No
ENSMUSG00000022915	1,82E+00	-3,033661385	1,31E+00	2,52E-01	5,50E-01	1700093J21Rik	3,54	No
ENSMUSG00000022938	2,61E-02	-0,31124622	6,71E-03	9,35E-01	1,00E+00	Fam3b	1,02	No
ENSMUSG00000022940	-2,33E-01	2,303807653	3,01E+00	8,25E-02	3,69E-01	Pigp	0,85	No
ENSMUSG00000022941	-5,64E-01	-1,26813811	1,14E+00	2,86E-01	5,82E-01	Ripply3	0,68	No
ENSMUSG00000022945	8,27E-02	4,679344717	1,10E+00	2,94E-01	5,90E-01	Chaf1b	1,06	No
ENSMUSG00000022946	6,34E-02	6,196922602	9,75E-01	3,23E-01	6,24E-01	Dopey2	1,04	No
ENSMUSG00000022947	4,45E-02	3,848827413	2,30E-01	6,31E-01	8,64E-01	Cbr3	1,03	No
ENSMUSG00000022948	1,03E-01	2,091775882	5,19E-01	4,71E-01	7,60E-01	Setd4	1,07	No
ENSMUSG00000022949	1,12E-01	-0,333287346	1,13E-01	7,37E-01	9,25E-01	Clic6	1,08	No
ENSMUSG00000022951	7,50E-02	4,174892006	7,73E-01	3,79E-01	6,80E-01	Rcan1	1,05	No
ENSMUSG00000022952	1,85E-01	7,376542829	9,60E+00	1,95E-03	2,75E-02	Runx1	1,14	Yes
ENSMUSG00000022956	1,31E-03	4,206285047	2,38E-04	9,88E-01	1,00E+00	Atp5o	1,00	No
ENSMUSG00000022957	2,21E-01	5,433425445	1,00E+01	1,56E-03	2,30E-02	Itsn1	1,17	Yes
ENSMUSG00000022960	-1,01E-01	4,14263974	1,40E+00	2,37E-01	5,50E-01	Donson	0,93	No
ENSMUSG00000022961	-2,08E-02	9,337240416	1,35E-01	7,13E-01	9,12E-01	Son	0,99	No
ENSMUSG00000022962	1,19E-01	6,046900642	3,36E+00	6,70E-02	3,26E-01	Gart	1,09	No
ENSMUSG00000022964	6,09E-02	6,882052338	9,93E-01	3,19E-01	6,19E-01	Tmem50b	1,04	No
ENSMUSG00000022965	1,32E-02	6,748359315	4,62E-02	8,30E-01	9,77E-01	Ifngr2	1,01	No

ENSMUSG00000022967	-4,81E-02	7,163413782	6,39E-01	4,24E-01	7,19E-01	Ifnar1	0,97	No
ENSMUSG00000022969	3,93E-02	6,231212067	3,77E-01	5,39E-01	8,10E-01	Il10rb	1,03	No
ENSMUSG00000022971	6,07E-02	5,610007367	7,88E-01	3,75E-01	6,76E-01	Ifnar2	1,04	No
ENSMUSG00000022972	1,30E-01	2,616788383	1,06E+00	3,02E-01	5,99E-01	1110004E09Rik	1,09	No
ENSMUSG00000022973	6,03E-02	7,285279752	1,02E+00	3,13E-01	6,13E-01	Synj1	1,04	No
ENSMUSG00000022974	-1,12E-01	6,755684179	3,32E+00	6,86E-02	3,29E-01	Paxbp1	0,93	No
ENSMUSG00000022978	1,21E-01	4,398537458	2,14E+00	1,44E-01	4,79E-01	Mis18a	1,09	No
ENSMUSG00000022982	-4,78E-02	4,652128035	3,77E-01	5,39E-01	8,09E-01	Sod1	0,97	No
ENSMUSG00000022983	4,50E-02	5,549931539	4,24E-01	5,15E-01	7,96E-01	Scaf4	1,03	No
ENSMUSG00000022986	2,67E+00	-3,033636951	2,76E+00	9,68E-02	3,88E-01	Krt75	6,36	No
ENSMUSG00000022987	7,35E-01	-2,896146969	2,94E-01	5,87E-01	8,40E-01	Zfp641	1,66	No
ENSMUSG00000022991	1,81E+00	-3,10765737	1,28E+00	2,58E-01	5,50E-01	Lalba	3,49	No
ENSMUSG00000022992	-5,86E-02	4,265621068	4,83E-01	4,87E-01	7,74E-01	Kansl2	0,96	No
ENSMUSG00000022993	-5,37E-01	-0,929122123	1,90E+00	1,68E-01	5,23E-01	4930415O20Rik	0,69	No
ENSMUSG00000022994	-8,16E-01	7,218713626	1,84E+02	6,64E-42	9,48E-39	Adcy6	0,57	Yes
ENSMUSG00000022995	-9,09E-02	6,63281096	2,14E+00	1,43E-01	4,79E-01	Enah	0,94	No
ENSMUSG00000022996	-4,39E-01	-2,448707129	1,97E-01	6,57E-01	8,80E-01	Wnt10b	0,74	No
ENSMUSG00000022997	7,34E-01	-2,896122591	2,93E-01	5,88E-01	8,40E-01	Wnt1	1,66	No
ENSMUSG00000022999	-1,73E-01	4,593696949	4,76E+00	2,92E-02	1,94E-01	Lmbr1l	0,89	No
ENSMUSG00000023000	1,60E+00	-2,770621501	1,75E+00	1,86E-01	5,50E-01	Dhh	3,02	No
ENSMUSG00000023004	1,19E-01	9,062889278	4,40E+00	3,59E-02	2,25E-01	Tuba1b	1,09	No
ENSMUSG00000023007	-9,37E-02	2,283120424	4,64E-01	4,96E-01	7,81E-01	Prpf40b	0,94	No
ENSMUSG00000023008	8,88E-02	4,936252936	1,37E+00	2,42E-01	5,50E-01	Fmnl3	1,06	No
ENSMUSG00000023009	4,84E-02	3,904800936	2,83E-01	5,95E-01	8,44E-01	Nckap5l	1,03	No
ENSMUSG00000023010	3,17E-02	8,776561168	3,08E-01	5,79E-01	8,36E-01	Tmbim6	1,02	No
ENSMUSG00000023013	1,87E+00	-3,107639691	1,38E+00	2,40E-01	5,50E-01	Aqp2	3,67	No
ENSMUSG00000023015	2,10E-01	5,956630001	1,02E+01	1,40E-03	2,12E-02	Racgap1	1,16	Yes
ENSMUSG00000023017	-4,21E-01	0,604866737	3,13E+00	7,68E-02	3,53E-01	Asic1	0,75	No
ENSMUSG00000023018	-9,26E-02	4,793958578	1,47E+00	2,25E-01	5,50E-01	Smarcd1	0,94	No
ENSMUSG00000023019	-6,40E-01	-0,060611252	4,77E+00	2,90E-02	1,94E-01	Gpd1	0,64	No
ENSMUSG00000023020	-7,71E-03	4,90892197	1,06E-02	9,18E-01	1,00E+00	Cox14	0,99	No
ENSMUSG00000023021	-3,87E-02	6,287458449	3,71E-01	5,42E-01	8,12E-01	Cers5	0,97	No

ENSMUSG00000023022	-2,99E-01	6,884609701	2,41E+01	8,98E-07	4,06E-05	Lima1	0,81	Yes
ENSMUSG00000023025	3,07E-02	6,144039218	2,27E-01	6,33E-01	8,65E-01	Larp4	1,02	No
ENSMUSG00000023026	1,19E-02	6,56836147	3,64E-02	8,49E-01	9,88E-01	Dip2b	1,01	No
ENSMUSG00000023027	2,02E-02	5,294863625	8,10E-02	7,76E-01	9,46E-01	Atf1	1,01	No
ENSMUSG00000023030	-2,72E-02	5,284672037	1,46E-01	7,03E-01	9,06E-01	Slc11a2	0,98	No
ENSMUSG00000023031	4,24E-01	-0,770504491	1,13E+00	2,87E-01	5,83E-01	Cela1	1,34	No
ENSMUSG00000023032	-6,70E-01	5,893216886	1,04E+02	1,67E-24	5,77E-22	Slc4a8	0,63	Yes
ENSMUSG00000023033	-1,92E+00	-2,963214917	1,46E+00	2,27E-01	5,50E-01	Scn8a	0,26	No
ENSMUSG00000023034	-1,46E-01	3,698664543	2,03E+00	1,55E-01	4,98E-01	Nr4a1	0,90	No
ENSMUSG00000023036	-4,45E-01	-1,317757456	7,75E-01	3,79E-01	6,79E-01	Pcdhgc4	0,73	No
ENSMUSG00000023039	-1,74E+00	-2,770603931	2,12E+00	1,46E-01	4,83E-01	Krt7	0,30	No
ENSMUSG00000023043	-1,94E+00	-3,107619243	1,50E+00	2,21E-01	5,50E-01	Krt18	0,26	No
ENSMUSG00000023044	-2,49E-01	2,335993996	3,41E+00	6,47E-02	3,19E-01	Csad	0,84	No
ENSMUSG00000023045	-2,84E-01	0,502835099	1,37E+00	2,41E-01	5,50E-01	Soat2	0,82	No
ENSMUSG00000023046	-4,90E-02	-2,711701209	1,12E-03	9,73E-01	1,00E+00	Igfbp6	0,97	No
ENSMUSG00000023047	-2,71E+00	-2,896125742	2,90E+00	8,85E-02	3,79E-01	Amhr2	0,15	No
ENSMUSG00000023048	-1,39E-01	7,881225736	5,63E+00	1,76E-02	1,39E-01	Prr13	0,91	No
ENSMUSG00000023050	-9,30E-02	5,394787444	1,76E+00	1,84E-01	5,48E-01	Map3k12	0,94	No
ENSMUSG00000023051	-1,48E-02	4,09018842	2,90E-02	8,65E-01	9,98E-01	Tarbp2	0,99	No
ENSMUSG00000023052	-4,32E-01	0,253245328	2,43E+00	1,19E-01	4,30E-01	Npff	0,74	No
ENSMUSG00000023055	-1,87E-01	4,921468189	6,26E+00	1,24E-02	1,09E-01	Calcoco1	0,88	No
ENSMUSG00000023064	-2,73E+00	-2,896107236	2,95E+00	8,57E-02	3,76E-01	Sncg	0,15	No
ENSMUSG00000023066	3,09E-01	4,403674975	1,41E+01	1,74E-04	3,87E-03	Rttn	1,24	Yes
ENSMUSG00000023067	-1,81E-01	9,383410659	1,02E+01	1,42E-03	2,14E-02	Cdkn1a	0,88	Yes
ENSMUSG00000023068	1,76E-04	7,073428166	8,36E-06	9,98E-01	1,00E+00	Nus1	1,00	No
ENSMUSG00000023072	2,50E-01	3,445214459	6,12E+00	1,34E-02	1,16E-01	Cep89	1,19	No
ENSMUSG00000023075	-2,28E-02	6,649067912	1,34E-01	7,14E-01	9,12E-01	Akirin1	0,98	No
ENSMUSG00000023079	-1,73E-01	3,996213037	3,74E+00	5,30E-02	2,81E-01	Gtf2ird1	0,89	No
ENSMUSG00000023083	1,87E+00	-3,03364371	1,36E+00	2,43E-01	5,50E-01	H2-M10.2	3,66	No
ENSMUSG00000023084	1,75E-01	2,899233827	2,26E+00	1,33E-01	4,59E-01	Lrrc71	1,13	No
ENSMUSG00000023087	8,13E-02	4,762439388	1,13E+00	2,88E-01	5,84E-01	Noct	1,06	No
ENSMUSG00000023088	3,14E-02	7,640058447	2,84E-01	5,94E-01	8,43E-01	Abcc1	1,02	No

ENSMUSG00000023089	1,96E-01	3,568443009	4,00E+00	4,55E-02	2,54E-01	Ndufa5	1,15	No
ENSMUSG00000023094	-1,41E-01	2,484106715	1,16E+00	2,81E-01	5,78E-01	Msrb2	0,91	No
ENSMUSG00000023104	1,18E-01	6,314151601	3,46E+00	6,27E-02	3,12E-01	Rfc2	1,09	No
ENSMUSG00000023106	5,13E-02	5,513770465	5,52E-01	4,58E-01	7,47E-01	Denr	1,04	No
ENSMUSG00000023110	7,70E-02	4,581995931	9,46E-01	3,31E-01	6,31E-01	Prmt5	1,05	No
ENSMUSG00000023118	4,60E-02	7,112982442	5,82E-01	4,46E-01	7,37E-01	Sympk	1,03	No
ENSMUSG00000023140	1,87E+00	-3,107639691	1,38E+00	2,40E-01	5,50E-01	Reg2	3,67	No
ENSMUSG00000023143	1,96E-01	6,403921805	9,52E+00	2,03E-03	2,83E-02	Nagpa	1,15	Yes
ENSMUSG00000023147	8,44E-02	2,87118756	5,26E-01	4,68E-01	7,57E-01	Wrb	1,06	No
ENSMUSG00000023150	1,23E-02	8,330094666	4,53E-02	8,31E-01	9,78E-01	Ivns1abp	1,01	No
ENSMUSG00000023151	-7,90E-02	-2,831990502	2,64E-03	9,59E-01	1,00E+00	Lrrc69	0,95	No
ENSMUSG00000023153	-9,23E-01	-1,355380086	3,45E+00	6,32E-02	3,14E-01	Tmem52	0,53	No
ENSMUSG00000023156	1,70E-01	3,606841879	3,11E+00	7,78E-02	3,56E-01	Rpp14	1,13	No
ENSMUSG00000023159	9,01E-03	-3,107646452	-5,16E-10	1,00E+00	1,00E+00	Psg29	1,01	No
ENSMUSG00000023169	1,33E-01	7,441545066	5,02E+00	2,51E-02	1,75E-01	Slc38a1	1,10	No
ENSMUSG00000023170	-4,67E-02	4,695952194	3,59E-01	5,49E-01	8,16E-01	Gps2	0,97	No
ENSMUSG00000023175	1,32E-01	7,633538501	5,05E+00	2,47E-02	1,73E-01	Bsg	1,10	No
ENSMUSG00000023176	2,57E+00	-3,0336723	2,56E+00	1,10E-01	4,06E-01	Cpn2	5,96	No
ENSMUSG00000023186	-1,88E-01	7,445136604	1,00E+01	1,53E-03	2,26E-02	Vwa5a	0,88	Yes
ENSMUSG00000023191	4,89E-02	1,448559072	7,46E-02	7,85E-01	9,51E-01	P3h3	1,03	No
ENSMUSG00000023192	1,87E+00	-3,107639691	1,38E+00	2,40E-01	5,50E-01	Grm2	3,67	No
ENSMUSG00000023206	-5,13E-01	4,078846143	3,58E+01	2,15E-09	1,67E-07	Il15ra	0,70	Yes
ENSMUSG00000023216	-1,44E+00	-2,033542874	3,68E+00	5,51E-02	2,88E-01	Epb42	0,37	No
ENSMUSG00000023224	-1,97E+00	-0,682829293	2,25E+01	2,11E-06	8,50E-05	Serping1	0,25	Yes
ENSMUSG00000023232	3,66E-01	-1,683156871	3,23E-01	5,70E-01	8,31E-01	Serinc2	1,29	No
ENSMUSG00000023235	-1,11E-01	2,591673419	7,87E-01	3,75E-01	6,76E-01	Ccl25	0,93	No
ENSMUSG00000023236	2,76E-01	-2,548241042	7,75E-02	7,81E-01	9,49E-01	Scg5	1,21	No
ENSMUSG00000023243	-7,15E-02	-2,311130876	7,07E-03	9,33E-01	1,00E+00	Kcnk5	0,95	No
ENSMUSG00000023247	1,55E+00	-2,10763576	3,62E+00	5,69E-02	2,93E-01	Guca2a	2,94	No
ENSMUSG00000023249	-4,69E-02	5,66186857	4,80E-01	4,88E-01	7,75E-01	Parp3	0,97	No
ENSMUSG00000023259	1,76E-01	3,444776353	3,11E+00	7,80E-02	3,57E-01	Slc26a6	1,13	No
ENSMUSG00000023262	-1,09E-01	2,514395594	6,74E-01	4,12E-01	7,07E-01	Acy1	0,93	No

ENSMUSG00000023266	5,51E-01	0,344548316	4,40E+00	3,59E-02	2,25E-01	Frs3	1,46	No
ENSMUSG00000023267	1,26E+00	-2,655126047	9,87E-01	3,20E-01	6,21E-01	Gabrr2	2,40	No
ENSMUSG00000023272	4,59E-02	5,297163157	4,15E-01	5,20E-01	7,99E-01	Creld2	1,03	No
ENSMUSG00000023274	-1,66E+00	-0,136036558	2,30E+01	1,60E-06	6,70E-05	Cd4	0,32	Yes
ENSMUSG00000023277	1,45E-01	6,810822143	5,59E+00	1,80E-02	1,41E-01	Twf2	1,11	No
ENSMUSG00000023284	6,03E-02	2,426717088	2,10E-01	6,46E-01	8,74E-01	Zfp605	1,04	No
ENSMUSG00000023286	-7,33E-02	5,489999003	1,11E+00	2,91E-01	5,87E-01	Ube2j2	0,95	No
ENSMUSG00000023307	-1,29E-01	5,728476085	3,68E+00	5,52E-02	2,88E-01	March5	0,91	No
ENSMUSG00000023328	-5,93E-01	-2,185650891	4,79E-01	4,89E-01	7,76E-01	Ache	0,66	No
ENSMUSG00000023330	2,72E-01	1,545127357	2,40E+00	1,22E-01	4,35E-01	Dtwd1	1,21	No
ENSMUSG00000023333	4,55E-01	-2,44872376	2,80E-01	5,97E-01	8,45E-01	Gcm1	1,37	No
ENSMUSG00000023341	-3,60E-01	3,326778207	1,26E+01	3,95E-04	7,57E-03	Mx2	0,78	Yes
ENSMUSG00000023345	2,00E-01	3,488145012	3,95E+00	4,70E-02	2,59E-01	Poc1a	1,15	No
ENSMUSG00000023348	1,63E-01	1,955984717	1,15E+00	2,84E-01	5,81E-01	Trip6	1,12	No
ENSMUSG00000023349	9,57E-02	10,57342336	2,91E+00	8,78E-02	3,78E-01	Clec4n	1,07	No
ENSMUSG00000023353	1,31E-01	6,241459167	4,21E+00	4,02E-02	2,38E-01	Agap3	1,10	No
ENSMUSG00000023367	-7,66E-01	6,048317123	1,40E+02	2,72E-32	1,81E-29	Tmem176a	0,59	Yes
ENSMUSG00000023393	1,43E-01	2,960792762	1,56E+00	2,11E-01	5,50E-01	Slc17a9	1,10	No
ENSMUSG00000023411	-2,82E-01	-0,800813311	5,18E-01	4,72E-01	7,60E-01	Nfatc4	0,82	No
ENSMUSG00000023439	8,28E-02	-0,322039642	6,19E-02	8,04E-01	9,62E-01	Gnb3	1,06	No
ENSMUSG00000023452	5,10E-02	6,414822177	6,51E-01	4,20E-01	7,15E-01	Pisd	1,04	No
ENSMUSG00000023456	2,28E-01	7,961131712	1,53E+01	9,33E-05	2,28E-03	Tpi1	1,17	Yes
ENSMUSG00000023460	-1,92E-01	5,714930914	8,12E+00	4,38E-03	5,06E-02	Rab12	0,88	No
ENSMUSG00000023467	-2,35E-01	0,730499592	1,10E+00	2,94E-01	5,89E-01	Tulp2	0,85	No
ENSMUSG00000023473	2,12E-01	3,678396223	4,89E+00	2,71E-02	1,85E-01	Celsr3	1,16	No
ENSMUSG00000023484	-2,30E-01	-1,185753742	2,47E-01	6,19E-01	8,56E-01	Prph	0,85	No
ENSMUSG00000023495	-3,90E-01	2,974470113	1,18E+01	5,89E-04	1,05E-02	Pcbp4	0,76	Yes
ENSMUSG00000023505	1,64E-01	5,867098506	6,07E+00	1,37E-02	1,18E-01	Cdca3	1,12	No
ENSMUSG00000023571	-3,34E-02	3,395411226	1,08E-01	7,42E-01	9,27E-01	C1qtnf12	0,98	No
ENSMUSG00000023572	-1,06E-01	4,93833425	2,04E+00	1,53E-01	4,96E-01	Ccndbp1	0,93	No
ENSMUSG00000023707	5,11E-02	4,113500942	3,45E-01	5,57E-01	8,21E-01	Ogfod2	1,04	No
ENSMUSG00000023723	-6,08E-03	5,384619239	7,51E-03	9,31E-01	1,00E+00	Mrps23	1,00	No

ENSMUSG00000023737	-9,16E-01	-2,711720553	5,96E-01	4,40E-01	7,32E-01	1700082M22Rik	0,53	No
ENSMUSG00000023755	-1,11E-01	2,299112532	6,35E-01	4,25E-01	7,20E-01	Rhebl1	0,93	No
ENSMUSG00000023764	-2,46E-01	4,513775531	9,65E+00	1,90E-03	2,69E-02	Sfi1	0,84	Yes
ENSMUSG00000023781	6,32E-01	-2,126825472	6,95E-01	4,04E-01	7,02E-01	Hes7	1,55	No
ENSMUSG00000023791	-7,06E-03	4,830161875	8,60E-03	9,26E-01	1,00E+00	Pigx	1,00	No
ENSMUSG00000023795	-4,12E-01	1,542641551	5,59E+00	1,81E-02	1,41E-01	Pisd-ps2	0,75	No
ENSMUSG00000023800	1,32E-01	3,620164504	1,88E+00	1,71E-01	5,26E-01	Tiam2	1,10	No
ENSMUSG00000023805	1,29E-01	6,119174894	3,92E+00	4,76E-02	2,61E-01	Synj2	1,09	No
ENSMUSG00000023806	-2,43E-03	4,110177156	7,86E-04	9,78E-01	1,00E+00	Rsph3b	1,00	No
ENSMUSG00000023809	-8,01E-02	6,061818786	1,52E+00	2,18E-01	5,50E-01	Rps6ka2	0,95	No
ENSMUSG00000023826	-2,44E-01	-1,247147882	2,36E-01	6,27E-01	8,61E-01	Park2	0,84	No
ENSMUSG00000023827	1,78E-01	4,703003344	5,21E+00	2,25E-02	1,63E-01	Agpat4	1,13	No
ENSMUSG00000023829	2,62E+00	-3,033654625	2,66E+00	1,03E-01	3,95E-01	Slc22a1	6,16	No
ENSMUSG00000023830	3,08E-01	7,94252008	2,78E+01	1,37E-07	7,36E-06	Igf2r	1,24	Yes
ENSMUSG00000023832	5,74E-03	5,451202535	6,91E-03	9,34E-01	1,00E+00	Acat2	1,00	No
ENSMUSG00000023845	4,89E-02	7,204306075	6,62E-01	4,16E-01	7,11E-01	Lnpep	1,03	No
ENSMUSG00000023852	-3,20E-02	6,692421895	2,67E-01	6,05E-01	8,49E-01	Chd1	0,98	No
ENSMUSG00000023861	2,31E-02	5,079472165	9,92E-02	7,53E-01	9,33E-01	Mpc1	1,02	No
ENSMUSG00000023868	-5,82E-02	-1,740726957	9,47E-03	9,22E-01	1,00E+00	Pde10a	0,96	No
ENSMUSG00000023873	4,42E-01	0,843755069	3,67E+00	5,54E-02	2,88E-01	1700010I14Rik	1,36	No
ENSMUSG00000023882	5,02E-01	1,483487845	7,95E+00	4,81E-03	5,43E-02	Zfp54	1,42	No
ENSMUSG00000023883	-1,21E-02	5,382801421	2,97E-02	8,63E-01	9,97E-01	Phf10	0,99	No
ENSMUSG00000023885	-1,86E+00	-2,963227946	1,40E+00	2,36E-01	5,50E-01	Thbs2	0,28	No
ENSMUSG00000023886	-2,70E+00	-3,033614577	2,89E+00	8,93E-02	3,79E-01	Smoc2	0,15	No
ENSMUSG00000023892	-2,66E-01	3,728322499	7,86E+00	5,04E-03	5,63E-02	Zfp51	0,83	No
ENSMUSG00000023902	-2,63E-02	-3,033641358	2,33E-04	9,88E-01	1,00E+00	Zscan10	0,98	No
ENSMUSG00000023903	-7,39E-01	6,312075358	1,35E+02	2,75E-31	1,72E-28	Mmp25	0,60	Yes
ENSMUSG00000023904	-6,33E-02	5,53309118	8,51E-01	3,56E-01	6,57E-01	Hcfc1r1	0,96	No
ENSMUSG00000023905	3,99E-01	2,910428817	1,12E+01	8,21E-04	1,37E-02	Tnfrsf12a	1,32	Yes
ENSMUSG00000023908	1,76E-01	4,962121905	5,51E+00	1,89E-02	1,46E-01	Pkmyt1	1,13	No
ENSMUSG00000023909	2,58E-01	4,866001424	1,15E+01	7,10E-04	1,22E-02	Paqr4	1,20	Yes
ENSMUSG00000023911	-4,17E-01	1,105749305	4,04E+00	4,45E-02	2,51E-01	Flywch2	0,75	No

ENSMUSG00000023912	-3,98E-01	-0,246964495	1,60E+00	2,06E-01	5,50E-01	Slc25a27	0,76	No
ENSMUSG00000023913	-5,31E-02	8,274074934	8,44E-01	3,58E-01	6,60E-01	Pla2g7	0,96	No
ENSMUSG00000023914	3,04E-01	-0,816585065	6,11E-01	4,35E-01	7,27E-01	Mep1a	1,23	No
ENSMUSG00000023915	4,14E-01	6,048627976	4,01E+01	2,47E-10	2,20E-08	Tnfrsf21	1,33	Yes
ENSMUSG00000023918	1,92E+00	-2,655179137	2,75E+00	9,72E-02	3,89E-01	Adgrf4	3,78	No
ENSMUSG00000023919	2,69E-01	3,730238284	8,11E+00	4,40E-03	5,07E-02	Cenpq	1,21	No
ENSMUSG00000023921	1,09E-01	4,982580617	2,17E+00	1,41E-01	4,74E-01	Mut	1,08	No
ENSMUSG00000023923	1,33E-01	6,052131969	4,15E+00	4,16E-02	2,42E-01	Tbc1d5	1,10	No
ENSMUSG00000023926	-5,99E-01	-2,832013989	2,83E-01	5,95E-01	8,44E-01	Rhag	0,66	No
ENSMUSG00000023927	-8,50E-01	0,527458898	1,20E+01	5,27E-04	9,57E-03	Satb1	0,55	Yes
ENSMUSG00000023930	2,64E+00	-2,963257704	2,69E+00	1,01E-01	3,92E-01	Crisp2	6,23	No
ENSMUSG00000023931	-8,69E-01	-2,963204587	4,16E-01	5,19E-01	7,98E-01	Efhb	0,55	No
ENSMUSG00000023932	9,77E-02	4,928317002	1,72E+00	1,90E-01	5,50E-01	Cdc5l	1,07	No
ENSMUSG00000023935	-1,53E+00	-1,711587635	7,05E+00	7,92E-03	7,84E-02	Spats1	0,35	No
ENSMUSG00000023938	1,09E-04	3,395937024	1,13E-06	9,99E-01	1,00E+00	Aars2	1,00	No
ENSMUSG00000023939	-1,48E-01	4,26238544	3,13E+00	7,67E-02	3,53E-01	Mrpl14	0,90	No
ENSMUSG00000023940	1,45E-01	3,862648303	2,52E+00	1,12E-01	4,13E-01	Sgo1	1,11	No
ENSMUSG00000023942	1,80E-01	6,36887268	8,01E+00	4,66E-03	5,29E-02	Slc29a1	1,13	No
ENSMUSG00000023944	-6,82E-02	10,38508135	1,48E+00	2,24E-01	5,50E-01	Hsp90ab1	0,95	No
ENSMUSG00000023947	-1,32E-01	6,32446625	4,35E+00	3,69E-02	2,28E-01	Nfkbie	0,91	No
ENSMUSG00000023949	-8,89E-01	-0,48518482	7,28E+00	6,97E-03	7,16E-02	Tcte1	0,54	No
ENSMUSG00000023951	5,83E-02	5,150089853	6,33E-01	4,26E-01	7,20E-01	Vegfa	1,04	No
ENSMUSG00000023952	-2,40E-01	6,771244594	1,53E+01	9,22E-05	2,26E-03	Gtpbp2	0,85	Yes
ENSMUSG00000023953	2,90E-01	4,058772536	1,11E+01	8,53E-04	1,42E-02	Polh	1,22	Yes
ENSMUSG00000023959	-4,53E+00	-2,355378183	1,16E+01	6,71E-04	1,16E-02	Clic5	0,04	Yes
ENSMUSG00000023960	-3,43E-01	-1,051756879	4,52E-01	5,01E-01	7,86E-01	Enpp5	0,79	No
ENSMUSG00000023961	1,61E-01	0,358360356	4,23E-01	5,15E-01	7,97E-01	Enpp4	1,12	No
ENSMUSG00000023963	3,05E-01	-1,378381311	3,45E-01	5,57E-01	8,21E-01	Cyp39a1	1,24	No
ENSMUSG00000023965	-1,74E-02	5,12967993	5,69E-02	8,11E-01	9,67E-01	Fbxl17	0,99	No
ENSMUSG00000023966	-4,16E-01	-0,460694544	1,49E+00	2,22E-01	5,50E-01	Rsph9	0,75	No
ENSMUSG00000023967	2,40E-02	4,574762229	9,11E-02	7,63E-01	9,38E-01	Mrps18a	1,02	No
ENSMUSG00000023968	2,55E+00	-2,185647803	5,84E+00	1,57E-02	1,30E-01	Crip3	5,84	No

ENSMUSG00000023971	-7,42E-02	4,439852196	8,44E-01	3,58E-01	6,60E-01	Rrp36	0,95	No
ENSMUSG00000023972	-5,68E-01	-1,070048049	1,60E+00	2,05E-01	5,50E-01	Ptk7	0,67	No
ENSMUSG00000023973	4,49E-03	6,900645212	5,42E-03	9,41E-01	1,00E+00	Cnpy3	1,00	No
ENSMUSG00000023977	-2,10E-02	6,71888449	1,16E-01	7,34E-01	9,23E-01	Ubr2	0,99	No
ENSMUSG00000023978	-3,88E-03	-3,03362684	4,02E-12	1,00E+00	1,00E+00	Prph2	1,00	No
ENSMUSG00000023979	-7,07E-02	-0,344386693	4,85E-02	8,26E-01	9,74E-01	Guca1b	0,95	No
ENSMUSG00000023980	-1,07E-01	4,443425407	1,78E+00	1,83E-01	5,45E-01	Taf8	0,93	No
ENSMUSG00000023982	2,48E-01	3,136830583	5,17E+00	2,30E-02	1,65E-01	Guca1a	1,19	No
ENSMUSG00000023984	1,04E-01	3,336309252	1,03E+00	3,10E-01	6,08E-01	Gm20517	1,07	No
ENSMUSG00000023987	2,64E+00	-3,033673223	2,69E+00	1,01E-01	3,92E-01	Pgc	6,23	No
ENSMUSG00000023988	9,56E-02	5,563193306	1,94E+00	1,64E-01	5,15E-01	Bysl	1,07	No
ENSMUSG00000023990	1,51E-01	5,781610397	5,04E+00	2,47E-02	1,74E-01	Tfeb	1,11	No
ENSMUSG00000023991	-4,54E-01	5,860281416	4,70E+01	6,96E-12	7,53E-10	Foxp4	0,73	Yes
ENSMUSG00000023992	1,87E-01	6,980415706	9,38E+00	2,20E-03	3,01E-02	Trem2	1,14	Yes
ENSMUSG00000023993	2,96E-01	0,655221466	1,63E+00	2,01E-01	5,50E-01	Treml1	1,23	No
ENSMUSG00000023994	-2,55E-02	7,233815716	1,82E-01	6,69E-01	8,88E-01	Nfya	0,98	No
ENSMUSG00000024002	5,79E-02	7,20398153	9,29E-01	3,35E-01	6,36E-01	Brd4	1,04	No
ENSMUSG00000024006	1,27E-01	7,009308755	4,41E+00	3,56E-02	2,24E-01	Stk38	1,09	No
ENSMUSG00000024007	1,21E-01	5,03952757	2,67E+00	1,02E-01	3,94E-01	Ppil1	1,09	No
ENSMUSG00000024008	-7,75E-02	0,136337386	7,46E-02	7,85E-01	9,51E-01	Cpne5	0,95	No
ENSMUSG00000024011	1,09E-01	0,655517507	2,07E-01	6,49E-01	8,75E-01	Pi16	1,08	No
ENSMUSG00000024012	7,92E-02	6,736347515	1,65E+00	1,99E-01	5,50E-01	Mtch1	1,06	No
ENSMUSG00000024013	2,13E-01	5,721109218	9,89E+00	1,67E-03	2,42E-02	Fgd2	1,16	Yes
ENSMUSG00000024014	-3,48E-02	9,082742713	3,75E-01	5,40E-01	8,10E-01	Pim1	0,98	No
ENSMUSG00000024018	1,75E-02	3,681286491	3,34E-02	8,55E-01	9,92E-01	Ccdc167	1,01	No
ENSMUSG00000024019	-9,23E-02	5,534886565	1,80E+00	1,80E-01	5,42E-01	Cmtr1	0,94	No
ENSMUSG00000024026	1,26E-01	4,344930276	2,31E+00	1,29E-01	4,50E-01	Glo1	1,09	No
ENSMUSG00000024029	1,87E+00	-3,107639691	1,38E+00	2,40E-01	5,50E-01	Tff3	3,67	No
ENSMUSG00000024030	2,95E-01	8,864467259	2,68E+01	2,24E-07	1,17E-05	Abcg1	1,23	Yes
ENSMUSG00000024032	3,08E-01	-2,929288075	5,90E-02	8,08E-01	9,65E-01	Tff1	1,24	No
ENSMUSG00000024033	2,08E-01	-0,929406666	2,67E-01	6,05E-01	8,49E-01	Rsph1	1,16	No
ENSMUSG00000024034	1,21E+00	-2,896186976	9,04E-01	3,42E-01	6,42E-01	Tmprss3	2,31	No

ENSMUSG00000024036	2,77E-05	4,015982974	9,88E-08	1,00E+00	1,00E+00	Slc37a1	1,00	No
ENSMUSG00000024037	8,58E-02	4,79436497	1,25E+00	2,63E-01	5,57E-01	Wdr4	1,06	No
ENSMUSG00000024038	-3,33E-02	5,887737767	2,54E-01	6,14E-01	8,53E-01	Ndufv3	0,98	No
ENSMUSG00000024039	-1,75E+00	-2,655154493	2,12E+00	1,46E-01	4,83E-01	Cbs	0,30	No
ENSMUSG00000024041	-8,55E-01	-2,963271948	4,02E-01	5,26E-01	8,03E-01	Cryaa	0,55	No
ENSMUSG00000024042	3,64E-01	4,120371123	1,78E+01	2,43E-05	7,33E-04	Sik1	1,29	Yes
ENSMUSG00000024043	-9,25E-01	2,452719634	4,94E+01	2,05E-12	2,38E-10	Arhgap28	0,53	Yes
ENSMUSG00000024044	1,63E-01	4,312614376	3,82E+00	5,06E-02	2,73E-01	Epb41l3	1,12	No
ENSMUSG00000024045	-3,83E-02	6,42479775	3,70E-01	5,43E-01	8,12E-01	Akap8	0,97	No
ENSMUSG00000024048	-1,25E-01	8,427867315	4,75E+00	2,93E-02	1,95E-01	Myl12a	0,92	No
ENSMUSG00000024049	-4,30E-01	-0,641423105	1,41E+00	2,35E-01	5,50E-01	Myom1	0,74	No
ENSMUSG00000024050	6,21E-02	5,515961738	8,09E-01	3,68E-01	6,70E-01	Wiz	1,04	No
ENSMUSG00000024052	6,03E-03	5,425133684	7,47E-03	9,31E-01	1,00E+00	Lpin2	1,00	No
ENSMUSG00000024053	2,86E-01	8,591366186	2,47E+01	6,83E-07	3,17E-05	Emilin2	1,22	Yes
ENSMUSG00000024054	-2,05E-03	6,62340886	1,08E-03	9,74E-01	1,00E+00	Smchd1	1,00	No
ENSMUSG00000024055	-5,78E-02	4,466527822	5,15E-01	4,73E-01	7,61E-01	Cyp4f13	0,96	No
ENSMUSG00000024056	1,56E-01	4,093699024	3,21E+00	7,30E-02	3,43E-01	Ndc80	1,11	No
ENSMUSG00000024059	-3,95E-02	-2,963274761	7,08E-04	9,79E-01	1,00E+00	Clip4	0,97	No
ENSMUSG00000024063	3,09E-01	5,747868793	2,11E+01	4,36E-06	1,61E-04	Lbh	1,24	Yes
ENSMUSG00000024064	-5,06E-01	-0,311108783	2,35E+00	1,25E-01	4,42E-01	Galnt14	0,70	No
ENSMUSG00000024065	-1,03E+00	-2,146074968	1,98E+00	1,59E-01	5,06E-01	Ehd3	0,49	No
ENSMUSG00000024066	-2,45E-03	5,894173006	1,36E-03	9,71E-01	1,00E+00	Xdh	1,00	No
ENSMUSG00000024067	-4,19E-02	3,901504704	2,12E-01	6,45E-01	8,73E-01	Dpy30	0,97	No
ENSMUSG00000024068	1,19E-01	5,069949589	2,68E+00	1,01E-01	3,94E-01	Spast	1,09	No
ENSMUSG00000024069	7,18E-02	3,124572272	4,29E-01	5,12E-01	7,95E-01	Slc30a6	1,05	No
ENSMUSG00000024070	1,20E-01	7,451439769	4,09E+00	4,31E-02	2,47E-01	Prkd3	1,09	No
ENSMUSG00000024072	7,67E-03	5,246404663	1,16E-02	9,14E-01	1,00E+00	Yipf4	1,01	No
ENSMUSG00000024073	7,12E-02	7,991936289	1,50E+00	2,21E-01	5,50E-01	Birc6	1,05	No
ENSMUSG00000024074	2,72E-01	2,298886204	3,82E+00	5,06E-02	2,73E-01	Crim1	1,21	No
ENSMUSG00000024077	4,53E-02	6,55518678	5,26E-01	4,68E-01	7,57E-01	Strn	1,03	No
ENSMUSG00000024078	1,58E-01	3,314440589	2,32E+00	1,27E-01	4,48E-01	Ttc27	1,12	No
ENSMUSG00000024079	5,20E-02	5,851525507	6,18E-01	4,32E-01	7,25E-01	Eif2ak2	1,04	No

ENSMUSG00000024081	4,90E-03	5,497870504	4,99E-03	9,44E-01	1,00E+00	Cebpz	1,00	No
ENSMUSG00000024082	4,27E-02	3,542419775	1,88E-01	6,65E-01	8,85E-01	Ndufaf7	1,03	No
ENSMUSG00000024083	6,90E-02	6,091995446	1,13E+00	2,87E-01	5,83E-01	Pja2	1,05	No
ENSMUSG00000024084	1,12E-01	6,403621569	3,16E+00	7,56E-02	3,50E-01	Qpct	1,08	No
ENSMUSG00000024085	6,89E-03	6,957830861	1,28E-02	9,10E-01	1,00E+00	Man2a1	1,00	No
ENSMUSG00000024087	-2,61E+00	3,929590021	6,74E+02	#######	#######	Cyp1b1	0,16	Yes
ENSMUSG00000024091	3,10E-02	6,55695565	2,45E-01	6,20E-01	8,57E-01	Vapa	1,02	No
ENSMUSG00000024095	-2,80E-01	8,124201171	2,32E+01	1,43E-06	6,08E-05	Hnrnpll	0,82	Yes
ENSMUSG00000024096	-2,02E-02	6,990504431	1,11E-01	7,39E-01	9,26E-01	Ralbp1	0,99	No
ENSMUSG00000024097	-1,47E-01	6,648205762	5,59E+00	1,81E-02	1,41E-01	Srsf7	0,90	No
ENSMUSG00000024098	-8,78E-02	6,416075935	1,94E+00	1,64E-01	5,15E-01	Twsg1	0,94	No
ENSMUSG00000024099	-8,39E-02	5,48402016	1,46E+00	2,26E-01	5,50E-01	Ndufv2	0,94	No
ENSMUSG00000024101	-1,94E-01	5,455439007	7,87E+00	5,02E-03	5,61E-02	Washc1	0,87	No
ENSMUSG00000024104	-2,32E-01	7,72761497	1,57E+01	7,44E-05	1,89E-03	Washc2	0,85	Yes
ENSMUSG00000024105	1,87E+00	-3,107639691	1,38E+00	2,40E-01	5,50E-01	Themis3	3,67	No
ENSMUSG00000024107	-2,68E+00	-3,033645766	2,84E+00	9,17E-02	3,82E-01	Lhcgr	0,16	No
ENSMUSG00000024109	-8,91E-01	-1,901667637	1,81E+00	1,78E-01	5,39E-01	Nrxn1	0,54	No
ENSMUSG00000024112	-1,87E+00	-3,107637761	1,39E+00	2,38E-01	5,50E-01	Cacna1h	0,27	No
ENSMUSG00000024116	-2,65E+00	-3,033633091	2,78E+00	9,55E-02	3,86E-01	Prss21	0,16	No
ENSMUSG00000024118	1,34E-01	2,888426842	1,32E+00	2,50E-01	5,50E-01	1600002H07Rik	1,10	No
ENSMUSG00000024120	1,01E-01	5,565508043	2,16E+00	1,42E-01	4,76E-01	Lrpprc	1,07	No
ENSMUSG00000024121	-1,83E-02	8,277659409	1,00E-01	7,52E-01	9,32E-01	Atp6v0c	0,99	No
ENSMUSG00000024122	8,61E-03	6,821429121	1,97E-02	8,88E-01	1,00E+00	Pdpk1	1,01	No
ENSMUSG00000024124	-5,05E-01	-0,26789786	2,40E+00	1,21E-01	4,34E-01	Prss30	0,70	No
ENSMUSG00000024127	2,53E-02	4,18750641	8,70E-02	7,68E-01	9,41E-01	Prepl	1,02	No
ENSMUSG00000024130	-3,02E-02	7,129181947	2,51E-01	6,16E-01	8,54E-01	Abca3	0,98	No
ENSMUSG00000024131	-2,28E-01	0,313171752	7,42E-01	3,89E-01	6,89E-01	Slc3a1	0,85	No
ENSMUSG00000024132	-3,42E-01	2,324599838	6,37E+00	1,16E-02	1,04E-01	Eci1	0,79	No
ENSMUSG00000024135	1,77E-01	3,965813985	3,94E+00	4,72E-02	2,60E-01	Srbd1	1,13	No
ENSMUSG00000024136	2,95E-01	-0,117306177	8,02E-01	3,70E-01	6,72E-01	Dnase1l2	1,23	No
ENSMUSG00000024137	2,70E-02	4,472976894	1,13E-01	7,37E-01	9,25E-01	E4f1	1,02	No
ENSMUSG00000024140	1,37E-02	2,620808779	1,15E-02	9,14E-01	1,00E+00	Epas1	1,01	No

ENSMUSG00000024142	1,66E-01	4,171963361	3,80E+00	5,13E-02	2,75E-01	Mlst8	1,12	No
ENSMUSG00000024143	2,11E-01	6,589375708	1,15E+01	7,02E-04	1,21E-02	Rhoq	1,16	Yes
ENSMUSG00000024145	7,04E-02	3,750074192	5,62E-01	4,54E-01	7,43E-01	Pigf	1,05	No
ENSMUSG00000024146	8,69E-02	5,086748229	1,42E+00	2,34E-01	5,50E-01	Cript	1,06	No
ENSMUSG00000024150	6,59E-02	6,186611141	1,05E+00	3,05E-01	6,02E-01	Mcfd2	1,05	No
ENSMUSG00000024151	2,40E-02	4,664991908	9,57E-02	7,57E-01	9,35E-01	Msh2	1,02	No
ENSMUSG00000024155	-1,06E-01	-3,033634182	5,86E-03	9,39E-01	1,00E+00	Meiob	0,93	No
ENSMUSG00000024158	4,97E-02	4,020784691	3,09E-01	5,78E-01	8,36E-01	Hagh	1,04	No
ENSMUSG00000024160	1,03E-01	4,753574503	1,78E+00	1,82E-01	5,45E-01	Spsb3	1,07	No
ENSMUSG00000024163	-7,36E-02	7,181128217	1,51E+00	2,20E-01	5,50E-01	Mapk8ip3	0,95	No
ENSMUSG00000024164	-5,73E-02	8,19987797	9,77E-01	3,23E-01	6,23E-01	C3	0,96	No
ENSMUSG00000024165	1,45E-01	6,183702506	5,05E+00	2,46E-02	1,73E-01	Hn1l	1,11	No
ENSMUSG00000024168	-2,44E-01	-0,726236549	4,43E-01	5,06E-01	7,90E-01	Tmem204	0,84	No
ENSMUSG00000024169	-6,02E-02	5,511689	7,64E-01	3,82E-01	6,83E-01	Ift140	0,96	No
ENSMUSG00000024170	1,43E-01	3,609622274	2,20E+00	1,38E-01	4,70E-01	Telo2	1,10	No
ENSMUSG00000024174	-7,56E-02	4,456075495	8,91E-01	3,45E-01	6,46E-01	Pot1b	0,95	No
ENSMUSG00000024175	7,31E-01	-2,963279337	2,91E-01	5,90E-01	8,41E-01	Tekt4	1,66	No
ENSMUSG00000024177	-1,50E-01	0,5694001	3,89E-01	5,33E-01	8,06E-01	Nme4	0,90	No
ENSMUSG00000024180	-7,12E-01	5,779381763	1,14E+02	1,03E-26	4,13E-24	Tmem8	0,61	Yes
ENSMUSG00000024181	9,24E-02	5,45044119	1,76E+00	1,85E-01	5,49E-01	Mrpl28	1,07	No
ENSMUSG00000024182	-9,33E-02	5,873489995	1,98E+00	1,59E-01	5,07E-01	Axin1	0,94	No
ENSMUSG00000024184	-1,24E+00	-2,998030423	7,19E-01	3,97E-01	6,97E-01	Pdia2	0,42	No
ENSMUSG00000024186	1,86E-01	2,760000622	2,43E+00	1,19E-01	4,29E-01	Rgs11	1,14	No
ENSMUSG00000024187	3,28E-02	5,937110412	2,47E-01	6,19E-01	8,56E-01	Fam234a	1,02	No
ENSMUSG00000024188	-5,88E-02	6,065317527	8,20E-01	3,65E-01	6,66E-01	Luc7l	0,96	No
ENSMUSG00000024190	2,91E-01	7,526738237	2,37E+01	1,12E-06	4,91E-05	Dusp1	1,22	Yes
ENSMUSG00000024191	7,87E-02	4,463021153	9,26E-01	3,36E-01	6,37E-01	Bnip1	1,06	No
ENSMUSG00000024193	-1,19E-01	3,727744402	1,63E+00	2,02E-01	5,50E-01	Phf1	0,92	No
ENSMUSG00000024194	9,08E-04	5,26071198	1,60E-04	9,90E-01	1,00E+00	Cuta	1,00	No
ENSMUSG00000024197	9,85E-02	7,179847189	2,69E+00	1,01E-01	3,93E-01	Plin3	1,07	No
ENSMUSG00000024201	-2,28E-03	5,374045597	1,05E-03	9,74E-01	1,00E+00	Kdm4b	1,00	No
ENSMUSG00000024205	-1,99E-01	5,990297121	9,24E+00	2,37E-03	3,21E-02	Rpl36-ps2	0,87	Yes

ENSMUSG00000024206	5,12E-01	1,278696044	7,04E+00	7,98E-03	7,88E-02	Rfx2	1,43	No
ENSMUSG00000024207	1,85E+00	-3,107657832	1,35E+00	2,46E-01	5,50E-01	Acsbg2	3,61	No
ENSMUSG00000024208	-3,93E-02	3,003894115	1,19E-01	7,30E-01	9,21E-01	Uqcc2	0,97	No
ENSMUSG00000024209	-5,74E-02	1,977974862	1,46E-01	7,03E-01	9,06E-01	1700061G19Rik	0,96	No
ENSMUSG00000024210	-9,89E-01	0,019599898	1,17E+01	6,25E-04	1,10E-02	Ip6k3	0,50	Yes
ENSMUSG00000024211	-1,90E+00	-3,033647263	1,44E+00	2,30E-01	5,50E-01	Grm8	0,27	No
ENSMUSG00000024212	2,97E-02	5,284247281	1,75E-01	6,76E-01	8,91E-01	Mllt1	1,02	No
ENSMUSG00000024213	3,79E-03	6,257392269	3,52E-03	9,53E-01	1,00E+00	Nudt3	1,00	No
ENSMUSG00000024215	-8,07E-01	-2,033542072	9,50E-01	3,30E-01	6,30E-01	Spdef	0,57	No
ENSMUSG00000024217	-4,66E-02	3,481477843	2,20E-01	6,39E-01	8,69E-01	Snrpc	0,97	No
ENSMUSG00000024218	-1,50E-01	4,564021105	3,58E+00	5,86E-02	2,99E-01	Taf11	0,90	No
ENSMUSG00000024219	-8,83E-03	5,441373274	1,60E-02	8,99E-01	1,00E+00	Anks1	0,99	No
ENSMUSG00000024220	5,31E-02	4,238874086	3,88E-01	5,33E-01	8,06E-01	Zfp523	1,04	No
ENSMUSG00000024222	2,14E-01	6,020627577	1,07E+01	1,06E-03	1,70E-02	Fkbp5	1,16	Yes
ENSMUSG00000024223	-2,71E+00	-2,896162395	2,92E+00	8,76E-02	3,78E-01	Armc12	0,15	No
ENSMUSG00000024227	-2,66E+00	-3,033639428	2,81E+00	9,35E-02	3,83E-01	Pdzph1	0,16	No
ENSMUSG00000024228	7,70E-02	1,503759624	1,89E-01	6,63E-01	8,84E-01	Nudt12	1,05	No
ENSMUSG00000024231	-8,17E-02	5,236088717	1,30E+00	2,54E-01	5,50E-01	Cul2	0,94	No
ENSMUSG00000024232	-2,05E-01	1,595930945	1,50E+00	2,21E-01	5,50E-01	Bambi	0,87	No
ENSMUSG00000024234	1,38E-02	5,096372393	3,58E-02	8,50E-01	9,89E-01	Mtpap	1,01	No
ENSMUSG00000024235	-6,93E-02	4,649406801	7,95E-01	3,73E-01	6,74E-01	Map3k8	0,95	No
ENSMUSG00000024236	2,57E-01	7,412138216	1,86E+01	1,61E-05	5,12E-04	Svil	1,20	Yes
ENSMUSG00000024238	-3,32E-01	4,507574476	1,76E+01	2,74E-05	8,11E-04	Zeb1	0,79	Yes
ENSMUSG00000024240	3,33E-02	5,201541666	2,17E-01	6,41E-01	8,71E-01	Epc1	1,02	No
ENSMUSG00000024241	8,85E-02	4,982881142	1,42E+00	2,33E-01	5,50E-01	Sos1	1,06	No
ENSMUSG00000024242	-4,34E-02	4,165126371	2,50E-01	6,17E-01	8,55E-01	Map4k3	0,97	No
ENSMUSG00000024245	-1,25E+00	-2,070096188	3,21E+00	7,34E-02	3,44E-01	Tmem178	0,42	No
ENSMUSG00000024246	3,26E-01	1,938800524	4,49E+00	3,42E-02	2,18E-01	Thumpd2	1,25	No
ENSMUSG00000024247	2,09E-01	5,431280619	9,05E+00	2,63E-03	3,46E-02	Pkdcc	1,16	Yes
ENSMUSG00000024248	-1,67E-01	7,699681384	8,06E+00	4,53E-03	5,18E-02	Cox7a2l	0,89	No
ENSMUSG00000024251	4,29E-02	4,661801615	3,03E-01	5,82E-01	8,38E-01	Thada	1,03	No
ENSMUSG00000024253	1,13E+00	-1,268193694	5,37E+00	2,05E-02	1,54E-01	Dync2li1	2,19	No

ENSMUSG00000024254	-1,89E+00	-3,1076441	1,42E+00	2,33E-01	5,50E-01	Abcg8	0,27	No
ENSMUSG00000024258	9,54E-02	4,584243118	1,44E+00	2,30E-01	5,50E-01	Polr2d	1,07	No
ENSMUSG00000024259	1,14E-01	5,783043252	2,89E+00	8,93E-02	3,79E-01	Slc25a46	1,08	No
ENSMUSG00000024260	9,36E-02	5,517935045	1,85E+00	1,73E-01	5,30E-01	Sap130	1,07	No
ENSMUSG00000024268	-2,89E-01	-0,117152432	9,49E-01	3,30E-01	6,30E-01	Celf4	0,82	No
ENSMUSG00000024269	2,41E-01	3,026445467	4,63E+00	3,14E-02	2,05E-01	Tpgs2	1,18	No
ENSMUSG00000024270	9,70E-02	5,732057189	2,08E+00	1,49E-01	4,90E-01	Slc39a6	1,07	No
ENSMUSG00000024271	-1,04E-02	5,564542511	2,30E-02	8,79E-01	1,00E+00	Elp2	0,99	No
ENSMUSG00000024273	-1,16E-01	2,894840098	1,01E+00	3,15E-01	6,16E-01	2700062C07Rik	0,92	No
ENSMUSG00000024274	9,96E-02	-0,627543907	7,83E-02	7,80E-01	9,48E-01	Zscan30	1,07	No
ENSMUSG00000024276	-1,11E-01	4,592378138	2,01E+00	1,56E-01	5,00E-01	Zfp397	0,93	No
ENSMUSG00000024277	-1,23E-01	6,951405767	4,11E+00	4,27E-02	2,46E-01	Mapre2	0,92	No
ENSMUSG00000024283	-6,45E-02	6,237637813	1,01E+00	3,14E-01	6,14E-01	Wac	0,96	No
ENSMUSG00000024286	7,73E-02	7,314885552	1,67E+00	1,96E-01	5,50E-01	Ccny	1,06	No
ENSMUSG00000024287	7,34E-02	4,793432226	9,24E-01	3,36E-01	6,37E-01	Thoc1	1,05	No
ENSMUSG00000024290	6,84E-02	6,543337003	1,19E+00	2,75E-01	5,71E-01	Rock1	1,05	No
ENSMUSG00000024292	1,87E+00	-3,107639691	1,38E+00	2,40E-01	5,50E-01	Cyp4f14	3,67	No
ENSMUSG00000024293	-2,92E-02	5,320524834	1,70E-01	6,80E-01	8,93E-01	Esco1	0,98	No
ENSMUSG00000024294	8,91E-02	7,339516237	2,23E+00	1,35E-01	4,63E-01	Mib1	1,06	No
ENSMUSG00000024295	-2,86E-01	-0,284075395	7,84E-01	3,76E-01	6,77E-01	Cyp4f41-ps	0,82	No
ENSMUSG00000024298	-1,70E-02	6,023137754	6,79E-02	7,94E-01	9,56E-01	Zfp871	0,99	No
ENSMUSG00000024299	1,98E-01	5,834333437	8,78E+00	3,05E-03	3,87E-02	Adamts10	1,15	Yes
ENSMUSG00000024300	1,02E-01	9,02878883	3,24E+00	7,21E-02	3,40E-01	Myo1f	1,07	No
ENSMUSG00000024301	2,74E-01	3,853249751	8,95E+00	2,78E-03	3,61E-02	Kifc5b	1,21	Yes
ENSMUSG00000024302	-1,94E+00	-3,107619243	1,50E+00	2,21E-01	5,50E-01	Dtna	0,26	No
ENSMUSG00000024304	-5,95E-01	3,321745596	3,48E+01	3,65E-09	2,72E-07	Cdh2	0,66	Yes
ENSMUSG00000024308	-6,38E-02	8,396789129	1,23E+00	2,67E-01	5,64E-01	Tapbp	0,96	No
ENSMUSG00000024309	-1,72E-01	4,351433629	4,26E+00	3,90E-02	2,34E-01	Pfdn6	0,89	No
ENSMUSG00000024312	6,80E-02	4,269971023	6,52E-01	4,20E-01	7,15E-01	Wdr46	1,05	No
ENSMUSG00000024317	9,97E-02	3,945294812	1,22E+00	2,69E-01	5,66E-01	Rnf138	1,07	No
ENSMUSG00000024319	3,38E-02	5,991587075	2,66E-01	6,06E-01	8,50E-01	Vps52	1,02	No
ENSMUSG00000024325	-5,05E-02	4,304329935	3,62E-01	5,47E-01	8,15E-01	Ring1	0,97	No

ENSMUSG00000024327	1,10E-01	6,124863643	2,90E+00	8,88E-02	3,79E-01	Slc39a7	1,08	No
ENSMUSG00000024330	1,32E-01	1,302403955	5,04E-01	4,78E-01	7,65E-01	Col11a2	1,10	No
ENSMUSG00000024331	-3,57E+00	-2,831967066	5,72E+00	1,68E-02	1,35E-01	Dsc2	0,08	No
ENSMUSG00000024334	-3,72E-01	4,651193038	2,29E+01	1,72E-06	7,15E-05	H2-Oa	0,77	Yes
ENSMUSG00000024335	2,23E-02	8,379570549	1,49E-01	6,99E-01	9,04E-01	Brd2	1,02	No
ENSMUSG00000024338	-2,71E-01	7,575474441	2,11E+01	4,28E-06	1,58E-04	Psmb8	0,83	Yes
ENSMUSG00000024339	-5,65E-02	7,115416429	8,77E-01	3,49E-01	6,50E-01	Tap2	0,96	No
ENSMUSG00000024340	6,90E-01	-1,070165721	2,15E+00	1,43E-01	4,77E-01	Btnl2	1,61	No
ENSMUSG00000024346	-2,71E-02	4,787615376	1,25E-01	7,24E-01	9,18E-01	Pfdn1	0,98	No
ENSMUSG00000024347	2,36E-01	-2,070201642	7,00E-02	7,91E-01	9,55E-01	Psd2	1,18	No
ENSMUSG00000024349	7,89E-02	7,539491471	1,78E+00	1,82E-01	5,44E-01	Tmem173	1,06	No
ENSMUSG00000024350	8,24E-02	3,826954792	7,98E-01	3,72E-01	6,73E-01	Dnajc18	1,06	No
ENSMUSG00000024352	3,36E-01	0,710622085	2,07E+00	1,50E-01	4,91E-01	Spata24	1,26	No
ENSMUSG00000024353	-2,18E-02	-2,711703514	3,51E-04	9,85E-01	1,00E+00	Mzb1	0,99	No
ENSMUSG00000024354	-3,54E-01	-0,366916067	1,18E+00	2,77E-01	5,73E-01	Slc23a1	0,78	No
ENSMUSG00000024357	-4,08E-03	3,891247458	1,99E-03	9,64E-01	1,00E+00	Sil1	1,00	No
ENSMUSG00000024359	3,44E-03	7,486084584	3,36E-03	9,54E-01	1,00E+00	Hspa9	1,00	No
ENSMUSG00000024360	-7,07E-02	7,612436256	1,43E+00	2,31E-01	5,50E-01	Etf1	0,95	No
ENSMUSG00000024365	1,17E+00	-3,14612376	6,40E-01	4,24E-01	7,18E-01	Cyp21a1	2,26	No
ENSMUSG00000024369	-1,65E-01	4,991782386	4,95E+00	2,61E-02	1,81E-01	Nelfe	0,89	No
ENSMUSG00000024370	2,26E-01	5,206666041	1,00E+01	1,57E-03	2,31E-02	Cdc23	1,17	Yes
ENSMUSG00000024371	-4,56E-01	0,752998599	4,14E+00	4,19E-02	2,43E-01	C2	0,73	No
ENSMUSG00000024376	-8,51E-01	-2,770645726	3,97E-01	5,28E-01	8,04E-01	Epb41l4a	0,55	No
ENSMUSG00000024378	2,90E-01	6,445624173	2,12E+01	4,04E-06	1,51E-04	Stard4	1,22	Yes
ENSMUSG00000024379	-6,39E-01	-2,401351322	5,57E-01	4,56E-01	7,45E-01	Tslp	0,64	No
ENSMUSG00000024381	1,04E-01	6,302082846	2,65E+00	1,03E-01	3,95E-01	Bin1	1,07	No
ENSMUSG00000024382	3,20E-02	5,598974698	2,21E-01	6,38E-01	8,69E-01	Ercc3	1,02	No
ENSMUSG00000024383	5,27E-02	6,269375105	6,81E-01	4,09E-01	7,06E-01	Map3k2	1,04	No
ENSMUSG00000024384	4,89E-02	5,718319634	5,24E-01	4,69E-01	7,57E-01	Iws1	1,03	No
ENSMUSG00000024387	-8,90E-03	6,284369762	1,95E-02	8,89E-01	1,00E+00	Csnk2b	0,99	No
ENSMUSG00000024388	1,85E+00	-3,107657832	1,35E+00	2,46E-01	5,50E-01	Myo7b	3,61	No
ENSMUSG00000024391	-5,86E-01	-1,711687261	9,50E-01	3,30E-01	6,30E-01	Apom	0,67	No

ENSMUSG00000024392	1,27E-02	7,310510574	4,50E-02	8,32E-01	9,78E-01	Bag6	1,01	No
ENSMUSG00000024393	-7,84E-03	8,558505282	1,87E-02	8,91E-01	1,00E+00	Prrc2a	0,99	No
ENSMUSG00000024395	1,51E-01	-0,808830266	1,45E-01	7,04E-01	9,06E-01	Lims2	1,11	No
ENSMUSG00000024397	1,41E-01	4,602605233	3,19E+00	7,42E-02	3,47E-01	Aif1	1,10	No
ENSMUSG00000024399	-5,38E-01	3,445621041	2,92E+01	6,36E-08	3,70E-06	Ltb	0,69	Yes
ENSMUSG00000024400	7,83E-02	5,549654532	1,29E+00	2,55E-01	5,50E-01	Wdr33	1,06	No
ENSMUSG00000024401	2,30E-01	5,41529266	1,03E+01	1,31E-03	2,01E-02	Tnf	1,17	Yes
ENSMUSG00000024402	-2,55E-01	4,427792686	9,76E+00	1,79E-03	2,56E-02	Lta	0,84	Yes
ENSMUSG00000024403	-3,37E-01	2,410843125	6,62E+00	1,01E-02	9,40E-02	Atp6v1g2	0,79	No
ENSMUSG00000024404	-1,86E-01	8,3057534	1,04E+01	1,27E-03	1,96E-02	Riok3	0,88	Yes
ENSMUSG00000024409	7,31E-01	-2,963267542	2,91E-01	5,90E-01	8,41E-01	Psors1c2	1,66	No
ENSMUSG00000024410	1,63E-01	5,853641828	6,00E+00	1,43E-02	1,22E-01	3110002H16Rik	1,12	No
ENSMUSG00000024413	2,01E-01	7,985637971	1,19E+01	5,69E-04	1,02E-02	Npc1	1,15	Yes
ENSMUSG00000024414	7,31E-03	4,287635027	7,57E-03	9,31E-01	1,00E+00	Mrpl27	1,01	No
ENSMUSG00000024420	1,97E-01	-1,473006293	1,08E-01	7,42E-01	9,27E-01	Zfp521	1,15	No
ENSMUSG00000024421	-6,07E-01	-1,424882413	1,44E+00	2,30E-01	5,50E-01	Lama3	0,66	No
ENSMUSG00000024422	7,36E-02	5,775971696	1,21E+00	2,72E-01	5,68E-01	Dhx16	1,05	No
ENSMUSG00000024423	-4,21E-02	6,120836965	4,23E-01	5,16E-01	7,97E-01	Impact	0,97	No
ENSMUSG00000024424	-2,75E-01	5,23886081	1,49E+01	1,15E-04	2,72E-03	Ttc39c	0,83	Yes
ENSMUSG00000024425	-3,84E-02	6,195259343	3,57E-01	5,50E-01	8,17E-01	Ndfip1	0,97	No
ENSMUSG00000024426	-1,73E-01	2,469925414	1,89E+00	1,69E-01	5,24E-01	Atat1	0,89	No
ENSMUSG00000024427	-1,12E+00	-0,998014654	6,23E+00	1,26E-02	1,11E-01	Spry4	0,46	No
ENSMUSG00000024429	-6,42E-02	5,899751885	9,36E-01	3,33E-01	6,34E-01	Gnl1	0,96	No
ENSMUSG00000024430	-4,77E-01	0,839736145	4,19E+00	4,06E-02	2,39E-01	Cabyr	0,72	No
ENSMUSG00000024431	6,11E-02	6,461498779	9,51E-01	3,30E-01	6,30E-01	Nr3c1	1,04	No
ENSMUSG00000024436	-2,71E-02	3,620467568	7,89E-02	7,79E-01	9,47E-01	Mrps18b	0,98	No
ENSMUSG00000024437	1,65E-01	4,919810632	4,83E+00	2,80E-02	1,89E-01	Gm8615	1,12	No
ENSMUSG00000024442	2,11E-01	4,017365794	5,73E+00	1,67E-02	1,35E-01	0610009O20Rik	1,16	No
ENSMUSG00000024446	-2,48E-02	3,056132401	4,87E-02	8,25E-01	9,74E-01	Rpp21	0,98	No
ENSMUSG00000024451	1,73E-02	3,035523287	2,42E-02	8,76E-01	1,00E+00	Arap3	1,01	No
ENSMUSG00000024454	-7,68E-02	5,142801073	1,13E+00	2,87E-01	5,83E-01	Hdac3	0,95	No
ENSMUSG00000024456	-3,16E-03	9,093214364	3,09E-03	9,56E-01	1,00E+00	Diaph1	1,00	No

ENSMUSG00000024457	-8,87E-02	6,075004629	1,87E+00	1,71E-01	5,27E-01	Trim26	0,94	No
ENSMUSG00000024459	6,19E-01	-2,311143536	4,27E-01	5,14E-01	7,96E-01	H2-M5	1,54	No
ENSMUSG00000024462	9,19E-02	3,820464434	9,78E-01	3,23E-01	6,23E-01	Gabbr1	1,07	No
ENSMUSG00000024472	1,21E-01	6,066542449	3,50E+00	6,14E-02	3,08E-01	Dcp2	1,09	No
ENSMUSG00000024474	-3,68E-02	6,149415921	3,24E-01	5,69E-01	8,30E-01	Ik	0,97	No
ENSMUSG00000024477	6,38E-02	5,010154923	7,53E-01	3,86E-01	6,87E-01	Pggt1b	1,05	No
ENSMUSG00000024479	1,86E+00	-3,033661846	1,35E+00	2,46E-01	5,50E-01	Mal2	3,62	No
ENSMUSG00000024480	6,04E-02	5,655778059	7,93E-01	3,73E-01	6,74E-01	Ap3s1	1,04	No
ENSMUSG00000024481	-1,87E+00	-3,107637761	1,39E+00	2,38E-01	5,50E-01	Lvrn	0,27	No
ENSMUSG00000024483	8,09E-02	6,874614961	1,75E+00	1,85E-01	5,50E-01	Ankhd1	1,06	No
ENSMUSG00000024485	8,12E-01	-2,711754475	5,73E-01	4,49E-01	7,39E-01	Slc4a9	1,76	No
ENSMUSG00000024486	-1,38E-01	3,652609115	1,97E+00	1,61E-01	5,09E-01	Hbegf	0,91	No
ENSMUSG00000024487	1,56E-01	5,315528572	4,81E+00	2,82E-02	1,90E-01	Yipf5	1,11	No
ENSMUSG00000024491	9,71E-02	5,587627253	2,03E+00	1,54E-01	4,98E-01	Rbm27	1,07	No
ENSMUSG00000024493	1,53E-01	5,634019181	5,09E+00	2,40E-02	1,70E-01	Lars	1,11	No
ENSMUSG00000024497	-1,14E-02	-3,033635021	1,50E-05	9,97E-01	1,00E+00	Pou4f3	0,99	No
ENSMUSG00000024498	1,10E-01	6,274458448	2,97E+00	8,46E-02	3,73E-01	Tcerg1	1,08	No
ENSMUSG00000024500	9,01E-03	-3,107646452	-5,16E-10	1,00E+00	1,00E+00	Ppp2r2b	1,01	No
ENSMUSG00000024501	-9,77E-01	-2,311177799	1,16E+00	2,82E-01	5,79E-01	Dpysl3	0,51	No
ENSMUSG00000024505	1,12E-01	1,889974934	5,22E-01	4,70E-01	7,58E-01	Dtwd2	1,08	No
ENSMUSG00000024507	-1,04E-01	6,400765774	2,71E+00	1,00E-01	3,92E-01	Hsd17b4	0,93	No
ENSMUSG00000024511	-9,03E-02	-1,998136327	1,51E-02	9,02E-01	1,00E+00	Rab27b	0,94	No
ENSMUSG00000024513	-2,34E-03	6,579222158	1,41E-03	9,70E-01	1,00E+00	Mbd2	1,00	No
ENSMUSG00000024515	-4,15E-02	6,211018498	4,19E-01	5,17E-01	7,98E-01	Smad4	0,97	No
ENSMUSG00000024516	-1,12E-01	6,46137661	3,16E+00	7,57E-02	3,50E-01	Sec11c	0,93	No
ENSMUSG00000024518	1,81E+00	-3,10765737	1,28E+00	2,58E-01	5,50E-01	Rax	3,49	No
ENSMUSG00000024519	-1,87E+00	-3,107637761	1,39E+00	2,38E-01	5,50E-01	Cplx4	0,27	No
ENSMUSG00000024521	-5,38E-01	7,417927287	8,17E+01	1,62E-19	3,55E-17	Pmaip1	0,69	Yes
ENSMUSG00000024524	8,62E-03	3,419664429	7,20E-03	9,32E-01	1,00E+00	Gnal	1,01	No
ENSMUSG00000024525	1,91E-01	4,85346685	6,26E+00	1,24E-02	1,09E-01	Impa2	1,14	No
ENSMUSG00000024526	-2,70E+00	-2,96323619	2,89E+00	8,89E-02	3,79E-01	Cidea	0,15	No
ENSMUSG00000024527	2,03E-02	5,911860275	9,48E-02	7,58E-01	9,36E-01	Afg3l2	1,01	No

ENSMUSG00000024528	-1,03E-01	3,275186019	9,46E-01	3,31E-01	6,31E-01	Srfbp1	0,93	No
ENSMUSG00000024529	-2,75E-01	0,392889463	1,20E+00	2,73E-01	5,69E-01	Lox	0,83	No
ENSMUSG00000024530	-2,45E-01	-0,448519942	4,61E-01	4,97E-01	7,82E-01	Prelid3a	0,84	No
ENSMUSG00000024533	-2,55E-01	5,845898777	1,49E+01	1,15E-04	2,73E-03	Spire1	0,84	Yes
ENSMUSG00000024534	1,82E+00	-3,033661385	1,31E+00	2,52E-01	5,50E-01	Sncaip	3,54	No
ENSMUSG00000024535	2,88E-01	4,657720681	1,33E+01	2,69E-04	5,55E-03	Snx24	1,22	Yes
ENSMUSG00000024537	-7,51E-02	3,381830809	5,27E-01	4,68E-01	7,56E-01	Psmg2	0,95	No
ENSMUSG00000024538	3,14E-01	4,741299223	1,62E+01	5,76E-05	1,53E-03	Ppic	1,24	Yes
ENSMUSG00000024539	5,14E-02	5,290375284	5,23E-01	4,70E-01	7,58E-01	Ptpn2	1,04	No
ENSMUSG00000024542	9,31E-02	6,218675459	2,11E+00	1,47E-01	4,85E-01	Cep192	1,07	No
ENSMUSG00000024544	4,21E-01	2,951135483	1,32E+01	2,87E-04	5,84E-03	Ldlrad4	1,34	Yes
ENSMUSG00000024548	3,49E-01	-2,033641209	2,24E-01	6,36E-01	8,67E-01	Setbp1	1,27	No
ENSMUSG00000024552	1,28E-01	-2,146149414	3,25E-02	8,57E-01	9,94E-01	Slc14a2	1,09	No
ENSMUSG00000024556	-5,44E-03	5,620635673	6,37E-03	9,36E-01	1,00E+00	Me2	1,00	No
ENSMUSG00000024558	-1,29E+00	-2,268111265	2,83E+00	9,27E-02	3,83E-01	Mapk4	0,41	No
ENSMUSG00000024560	-2,91E-02	5,775977288	1,89E-01	6,64E-01	8,84E-01	Cxxc1	0,98	No
ENSMUSG00000024561	-8,97E-02	6,317329361	1,99E+00	1,58E-01	5,05E-01	Mbd1	0,94	No
ENSMUSG00000024563	2,20E-02	5,777077435	1,08E-01	7,43E-01	9,28E-01	Smad2	1,02	No
ENSMUSG00000024565	-2,67E+00	-2,831989413	2,83E+00	9,23E-02	3,83E-01	Sall3	0,16	No
ENSMUSG00000024566	-2,32E-02	6,39331366	1,34E-01	7,14E-01	9,12E-01	Atp9b	0,98	No
ENSMUSG00000024570	-3,40E-02	4,726593278	1,92E-01	6,61E-01	8,82E-01	Rbfa	0,98	No
ENSMUSG00000024571	4,69E-04	6,779125534	5,82E-05	9,94E-01	1,00E+00	Gm16286	1,00	No
ENSMUSG00000024575	-4,08E-01	-0,535102262	1,21E+00	2,71E-01	5,67E-01	Pde6a	0,75	No
ENSMUSG00000024576	-8,80E-03	7,901713082	2,28E-02	8,80E-01	1,00E+00	Csnk1a1	0,99	No
ENSMUSG00000024579	4,00E-01	2,956818821	1,21E+01	5,12E-04	9,35E-03	Pcyox1l	1,32	Yes
ENSMUSG00000024580	2,24E-01	3,814933414	6,02E+00	1,41E-02	1,21E-01	Grpel2	1,17	No
ENSMUSG00000024581	9,27E-03	5,802741423	1,93E-02	8,89E-01	1,00E+00	Napg	1,01	No
ENSMUSG00000024583	-8,56E-03	5,770847899	1,63E-02	8,98E-01	1,00E+00	Txnl1	0,99	No
ENSMUSG00000024587	5,62E-03	6,956183408	8,51E-03	9,26E-01	1,00E+00	Nars	1,00	No
ENSMUSG00000024588	7,32E-02	5,478822788	1,11E+00	2,92E-01	5,87E-01	Fech	1,05	No
ENSMUSG00000024589	-7,64E-04	4,601004736	9,37E-05	9,92E-01	1,00E+00	Nedd4l	1,00	No
ENSMUSG00000024590	2,27E-01	6,391757079	1,29E+01	3,34E-04	6,61E-03	Lmnb1	1,17	Yes

ENSMUSG00000024592	3,02E-01	3,159735046	8,02E+00	4,63E-03	5,27E-02	C330018D20Rik	1,23	No
ENSMUSG00000024593	-5,58E-01	-2,711748377	2,44E-01	6,21E-01	8,57E-01	Megf10	0,68	No
ENSMUSG00000024594	1,30E-01	5,392438644	3,42E+00	6,46E-02	3,18E-01	Prrc1	1,09	No
ENSMUSG00000024597	6,97E-02	2,266267365	2,51E-01	6,16E-01	8,54E-01	Slc12a2	1,05	No
ENSMUSG00000024598	-1,87E+00	-3,107637761	1,39E+00	2,38E-01	5,50E-01	Fbn2	0,27	No
ENSMUSG00000024600	3,19E-02	0,858976171	2,24E-02	8,81E-01	1,00E+00	Slc27a6	1,02	No
ENSMUSG00000024601	2,05E-02	5,756812794	9,29E-02	7,60E-01	9,37E-01	Isoc1	1,01	No
ENSMUSG00000024603	-1,20E-01	6,292669728	3,56E+00	5,93E-02	3,01E-01	Dctn4	0,92	No
ENSMUSG00000024604	2,97E-02	5,8755281	2,01E-01	6,54E-01	8,79E-01	Rbm22	1,02	No
ENSMUSG00000024608	-3,84E-01	6,385864279	3,67E+01	1,34E-09	1,07E-07	Rps14	0,77	Yes
ENSMUSG00000024610	-4,99E-02	12,88651039	8,03E-01	3,70E-01	6,72E-01	Cd74	0,97	No
ENSMUSG00000024613	3,10E-01	6,178629528	2,31E+01	1,54E-06	6,49E-05	Tcof1	1,24	Yes
ENSMUSG00000024614	3,96E-02	6,358425483	3,92E-01	5,31E-01	8,05E-01	Tmx3	1,03	No
ENSMUSG00000024617	3,73E-01	1,952313714	5,97E+00	1,46E-02	1,23E-01	Camk2a	1,30	No
ENSMUSG00000024620	-5,80E-01	-0,300001583	3,16E+00	7,56E-02	3,50E-01	Pdgfrb	0,67	No
ENSMUSG00000024621	1,46E-01	9,884864063	6,71E+00	9,56E-03	9,02E-02	Csf1r	1,11	No
ENSMUSG00000024622	1,36E-01	5,057983992	3,47E+00	6,24E-02	3,11E-01	Hmgxb3	1,10	No
ENSMUSG00000024639	7,22E-02	6,945911374	1,40E+00	2,36E-01	5,50E-01	Gnaq	1,05	No
ENSMUSG00000024640	1,92E-01	5,641279377	7,88E+00	5,01E-03	5,61E-02	Psat1	1,14	No
ENSMUSG00000024642	2,01E-01	4,826832264	6,90E+00	8,62E-03	8,37E-02	Tle4	1,15	No
ENSMUSG00000024644	2,60E-01	8,6536746	2,05E+01	5,88E-06	2,08E-04	Cndp2	1,20	Yes
ENSMUSG00000024645	1,59E-01	2,975181403	1,99E+00	1,58E-01	5,05E-01	Timm21	1,12	No
ENSMUSG00000024646	4,03E-02	7,084420497	4,43E-01	5,05E-01	7,90E-01	Cyb5a	1,03	No
ENSMUSG00000024653	-2,25E+00	-2,548194896	4,13E+00	4,21E-02	2,44E-01	Scgb1a1	0,21	No
ENSMUSG00000024654	-4,88E-01	2,365175109	1,35E+01	2,36E-04	4,98E-03	Asrgl1	0,71	Yes
ENSMUSG00000024658	-4,94E-02	-2,015655484	5,09E-03	9,43E-01	1,00E+00	Gm9750	0,97	No
ENSMUSG00000024659	1,29E-01	8,882919426	5,12E+00	2,36E-02	1,68E-01	Anxa1	1,09	No
ENSMUSG00000024660	2,10E-01	6,502085445	1,12E+01	8,36E-04	1,39E-02	Incenp	1,16	Yes
ENSMUSG00000024661	-6,59E-03	12,58082695	1,40E-02	9,06E-01	1,00E+00	Fth1	1,00	No
ENSMUSG00000024663	5,05E-01	7,008756952	6,83E+01	1,38E-16	2,43E-14	Rab3il1	1,42	Yes
ENSMUSG00000024664	2,83E-01	4,131091305	1,06E+01	1,11E-03	1,75E-02	Fads3	1,22	Yes
ENSMUSG00000024665	3,15E-01	4,983556845	1,78E+01	2,49E-05	7,50E-04	Fads2	1,24	Yes

ENSMUSG00000024666	1,60E-01	2,891370843	1,97E+00	1,61E-01	5,09E-01	Tmem138	1,12	No
ENSMUSG00000024667	-6,36E-02	3,030435211	3,20E-01	5,71E-01	8,32E-01	Tmem216	0,96	No
ENSMUSG00000024668	-1,75E-01	6,510811389	7,82E+00	5,18E-03	5,74E-02	Sdhaf2	0,89	No
ENSMUSG00000024669	-8,06E-01	-0,497394818	5,06E+00	2,45E-02	1,72E-01	Cd5	0,57	No
ENSMUSG00000024670	-2,06E-01	4,549132567	6,48E+00	1,09E-02	9,95E-02	Cd6	0,87	No
ENSMUSG00000024672	4,29E-02	2,869853718	1,33E-01	7,16E-01	9,13E-01	Ms4a7	1,03	No
ENSMUSG00000024673	-1,28E+00	-1,600588096	5,13E+00	2,36E-02	1,68E-01	Ms4a1	0,41	No
ENSMUSG00000024675	1,66E-02	0,873671853	5,97E-03	9,38E-01	1,00E+00	Ms4a4c	1,01	No
ENSMUSG00000024677	-2,50E-01	0,477567894	1,03E+00	3,10E-01	6,09E-01	Ms4a6b	0,84	No
ENSMUSG00000024678	1,87E+00	-3,107639691	1,38E+00	2,40E-01	5,50E-01	Ms4a4d	3,67	No
ENSMUSG00000024679	2,46E-01	6,200813962	1,46E+01	1,36E-04	3,12E-03	Ms4a6d	1,19	Yes
ENSMUSG00000024680	-1,35E+00	-2,355564206	2,50E+00	1,14E-01	4,17E-01	Ms4a2	0,39	No
ENSMUSG00000024681	-1,62E-01	-1,333262308	1,07E-01	7,43E-01	9,28E-01	Ms4a3	0,89	No
ENSMUSG00000024682	1,38E-01	-2,37833947	1,84E-02	8,92E-01	1,00E+00	Gif	1,10	No
ENSMUSG00000024683	5,11E-02	4,01463846	3,32E-01	5,65E-01	8,27E-01	Mrpl16	1,04	No
ENSMUSG00000024687	1,39E-01	6,521470969	4,91E+00	2,67E-02	1,83E-01	Osbp	1,10	No
ENSMUSG00000024691	2,71E-01	6,177640793	1,78E+01	2,50E-05	7,52E-04	Fam111a	1,21	Yes
ENSMUSG00000024695	1,70E-02	7,477493201	8,19E-02	7,75E-01	9,45E-01	Zfp91	1,01	No
ENSMUSG00000024696	2,30E-01	5,436161556	1,08E+01	1,03E-03	1,65E-02	Lpxn	1,17	Yes
ENSMUSG00000024697	3,12E+00	-2,8961712	3,97E+00	4,64E-02	2,57E-01	Gna14	8,68	No
ENSMUSG00000024712	-4,15E-02	6,455806182	4,37E-01	5,08E-01	7,92E-01	Rfk	0,97	No
ENSMUSG00000024725	-4,53E-02	7,200467279	5,68E-01	4,51E-01	7,41E-01	Ostf1	0,97	No
ENSMUSG00000024726	3,40E-01	2,790938667	8,13E+00	4,36E-03	5,04E-02	Carnmt1	1,27	No
ENSMUSG00000024727	2,74E-01	-0,367043884	6,80E-01	4,09E-01	7,06E-01	Trpm6	1,21	No
ENSMUSG00000024729	3,24E-01	-2,448592511	1,07E-01	7,44E-01	9,28E-01	1700017D01Rik	1,25	No
ENSMUSG00000024730	-1,32E-01	2,076501652	8,04E-01	3,70E-01	6,71E-01	Ms4a8a	0,91	No
ENSMUSG00000024732	4,33E-02	5,770359848	4,12E-01	5,21E-01	8,00E-01	Ccdc86	1,03	No
ENSMUSG00000024734	-1,86E+00	-2,963205176	1,40E+00	2,36E-01	5,50E-01	Zp1	0,28	No
ENSMUSG00000024735	8,37E-02	5,866112557	1,59E+00	2,07E-01	5,50E-01	Prpf19	1,06	No
ENSMUSG00000024736	3,01E-02	6,062925422	2,14E-01	6,44E-01	8,72E-01	Tmem132a	1,02	No
ENSMUSG00000024737	-9,55E-03	8,334887817	2,74E-02	8,69E-01	1,00E+00	Slc15a3	0,99	No
ENSMUSG00000024738	1,87E+00	-3,107639691	1,38E+00	2,40E-01	5,50E-01	Pga5	3,67	No

ENSMUSG00000024740	-4,20E-02	8,41290496	5,33E-01	4,65E-01	7,54E-01	Ddb1	0,97	No
ENSMUSG00000024742	1,84E-01	5,649963429	7,32E+00	6,82E-03	7,05E-02	Fen1	1,14	No
ENSMUSG00000024743	-4,72E-01	2,9025914	1,75E+01	2,87E-05	8,43E-04	Syt7	0,72	Yes
ENSMUSG00000024747	-9,81E-01	-2,355615569	1,16E+00	2,81E-01	5,77E-01	Aldh1a7	0,51	No
ENSMUSG00000024749	-1,15E+00	-1,998014421	3,05E+00	8,07E-02	3,64E-01	Tmc1	0,45	No
ENSMUSG00000024750	4,77E-02	7,825526441	6,64E-01	4,15E-01	7,10E-01	Zfand5	1,03	No
ENSMUSG00000024754	2,04E-01	4,836483895	7,10E+00	7,73E-03	7,70E-02	Tmem2	1,15	No
ENSMUSG00000024758	1,16E-02	7,832996833	3,94E-02	8,43E-01	9,85E-01	Rtn3	1,01	No
ENSMUSG00000024759	-4,62E-02	7,626817895	6,17E-01	4,32E-01	7,26E-01	Atl3	0,97	No
ENSMUSG00000024761	-3,52E-01	5,079098273	2,32E+01	1,44E-06	6,10E-05	Gm16437	0,78	Yes
ENSMUSG00000024764	1,06E-01	6,330960208	2,77E+00	9,61E-02	3,87E-01	Naa40	1,08	No
ENSMUSG00000024766	8,36E-02	4,211513345	9,69E-01	3,25E-01	6,26E-01	Lipo3	1,06	No
ENSMUSG00000024767	-4,84E-02	6,030064742	5,50E-01	4,58E-01	7,47E-01	Otub1	0,97	No
ENSMUSG00000024768	-7,76E-02	-2,963273445	2,73E-03	9,58E-01	1,00E+00	Lipf	0,95	No
ENSMUSG00000024769	-3,35E-01	4,980685375	2,04E+01	6,35E-06	2,23E-04	Cdc42bpg	0,79	Yes
ENSMUSG00000024770	1,75E-01	3,472422317	3,08E+00	7,93E-02	3,60E-01	Lipn	1,13	No
ENSMUSG00000024772	-6,03E-01	8,121681003	1,08E+02	3,28E-25	1,17E-22	Ehd1	0,66	Yes
ENSMUSG00000024773	-8,73E-02	6,425836627	1,92E+00	1,65E-01	5,18E-01	Atg2a	0,94	No
ENSMUSG00000024774	-4,66E-02	-2,711655481	1,49E-03	9,69E-01	1,00E+00	Ankrd22	0,97	No
ENSMUSG00000024776	2,84E-01	4,143918013	1,09E+01	9,71E-04	1,58E-02	Stambpl1	1,22	Yes
ENSMUSG00000024777	-1,22E-01	5,019146567	2,72E+00	9,89E-02	3,91E-01	Ppp2r5b	0,92	No
ENSMUSG00000024778	-4,12E-01	4,938796374	3,10E+01	2,63E-08	1,67E-06	Fas	0,75	Yes
ENSMUSG00000024780	-5,52E-02	5,405078565	6,30E-01	4,27E-01	7,21E-01	Cdc37l1	0,96	No
ENSMUSG00000024781	1,06E-03	9,095921808	3,47E-04	9,85E-01	1,00E+00	Lipa	1,00	No
ENSMUSG00000024782	-1,14E-02	6,699771021	3,43E-02	8,53E-01	9,91E-01	Ak3	0,99	No
ENSMUSG00000024784	-2,02E+00	-2,655103724	3,10E+00	7,83E-02	3,57E-01	Gpha2	0,25	No
ENSMUSG00000024785	-7,85E-02	4,417793821	9,39E-01	3,33E-01	6,33E-01	Rcl1	0,95	No
ENSMUSG00000024786	3,41E-01	-2,226232298	1,18E-01	7,31E-01	9,22E-01	Majin	1,27	No
ENSMUSG00000024787	3,23E-02	4,778008953	1,76E-01	6,75E-01	8,91E-01	Snx15	1,02	No
ENSMUSG00000024789	-3,96E-01	9,613403074	4,92E+01	2,35E-12	2,71E-10	Jak2	0,76	Yes
ENSMUSG00000024790	-9,76E-02	4,173486639	1,31E+00	2,52E-01	5,50E-01	Sac3d1	0,93	No
ENSMUSG00000024791	1,72E-01	4,734607054	4,91E+00	2,66E-02	1,83E-01	Cdca5	1,13	No

ENSMUSG00000024792	-4,16E-02	4,44138949	2,64E-01	6,07E-01	8,50E-01	Zfpl1	0,97	No
ENSMUSG00000024793	1,54E+00	-1,574289374	6,46E+00	1,10E-02	1,00E-01	Tnfrsf25	2,91	No
ENSMUSG00000024795	3,09E-01	5,233259517	1,87E+01	1,52E-05	4,85E-04	Kif20b	1,24	Yes
ENSMUSG00000024797	-7,96E-02	6,053079427	1,50E+00	2,20E-01	5,50E-01	Vps51	0,95	No
ENSMUSG00000024798	-5,62E-01	3,931206809	3,89E+01	4,50E-10	3,90E-08	Htr7	0,68	Yes
ENSMUSG00000024799	-1,25E-01	4,405854428	2,39E+00	1,22E-01	4,36E-01	Tm7sf2	0,92	No
ENSMUSG00000024800	1,24E-03	2,567605872	9,47E-05	9,92E-01	1,00E+00	Rpp30	1,00	No
ENSMUSG00000024803	1,87E+00	-3,033632735	1,36E+00	2,43E-01	5,50E-01	Ankrd1	3,66	No
ENSMUSG00000024805	-4,44E-01	5,357759013	4,02E+01	2,34E-10	2,09E-08	Pcgf5	0,74	Yes
ENSMUSG00000024806	3,54E+00	-2,831991944	5,43E+00	1,98E-02	1,50E-01	Mlana	11,60	No
ENSMUSG00000024807	8,53E-02	6,048963994	1,71E+00	1,92E-01	5,50E-01	Syvn1	1,06	No
ENSMUSG00000024810	-2,68E-02	-0,669156372	5,77E-03	9,39E-01	1,00E+00	Il33	0,98	No
ENSMUSG00000024811	4,08E-02	7,323207839	4,67E-01	4,95E-01	7,80E-01	Tnks2	1,03	No
ENSMUSG00000024812	-5,44E-02	5,168648307	5,71E-01	4,50E-01	7,40E-01	Tjp2	0,96	No
ENSMUSG00000024816	1,71E-03	6,754174114	7,73E-04	9,78E-01	1,00E+00	Frmd8	1,00	No
ENSMUSG00000024817	-2,66E-02	6,146769096	1,71E-01	6,79E-01	8,93E-01	Uhrf2	0,98	No
ENSMUSG00000024818	-3,28E-02	6,187264408	2,61E-01	6,10E-01	8,51E-01	Slc25a45	0,98	No
ENSMUSG00000024824	2,01E-01	5,841180225	9,15E+00	2,49E-03	3,32E-02	Rad9a	1,15	Yes
ENSMUSG00000024826	-5,21E-02	6,200982842	6,57E-01	4,17E-01	7,12E-01	Dpf2	0,96	No
ENSMUSG00000024827	1,37E+00	-2,14600657	1,93E+00	1,65E-01	5,17E-01	Gldc	2,59	No
ENSMUSG00000024829	7,49E-02	3,007961302	4,31E-01	5,11E-01	7,94E-01	Mrpl21	1,05	No
ENSMUSG00000024830	-7,58E-02	3,867026985	6,91E-01	4,06E-01	7,02E-01	Rps6kb2	0,95	No
ENSMUSG00000024831	4,10E-02	3,787436782	1,92E-01	6,61E-01	8,82E-01	Ighmbp2	1,03	No
ENSMUSG00000024833	6,20E-02	4,987366691	7,05E-01	4,01E-01	6,99E-01	Pola2	1,04	No
ENSMUSG00000024835	-6,98E-02	8,415320586	1,47E+00	2,25E-01	5,50E-01	Coro1b	0,95	No
ENSMUSG00000024841	-9,71E-02	5,513119699	1,97E+00	1,60E-01	5,09E-01	Eif1ad	0,93	No
ENSMUSG00000024842	-5,49E-01	-0,963210748	1,32E+00	2,50E-01	5,50E-01	Cabp4	0,68	No
ENSMUSG00000024843	-3,47E-01	6,313692375	2,96E+01	5,19E-08	3,07E-06	Chka	0,79	Yes
ENSMUSG00000024844	-4,38E-02	5,802863863	4,31E-01	5,12E-01	7,94E-01	Banf1	0,97	No
ENSMUSG00000024845	-1,76E-01	5,77960342	6,93E+00	8,48E-03	8,28E-02	Tmem134	0,89	No
ENSMUSG00000024846	3,30E-01	-0,561108728	7,55E-01	3,85E-01	6,86E-01	Cst6	1,26	No
ENSMUSG00000024847	-8,62E-02	6,079997751	1,75E+00	1,85E-01	5,50E-01	Aip	0,94	No

ENSMUSG00000024851	3,66E-02	7,100315189	3,69E-01	5,44E-01	8,12E-01	Pitpnm1	1,03	No
ENSMUSG00000024853	6,61E-03	7,553042495	1,25E-02	9,11E-01	1,00E+00	Sf3b2	1,00	No
ENSMUSG00000024854	3,18E-02	5,491301307	2,12E-01	6,45E-01	8,73E-01	Pold4	1,02	No
ENSMUSG00000024855	-2,97E-02	5,534669303	1,87E-01	6,65E-01	8,85E-01	Pacs1	0,98	No
ENSMUSG00000024856	1,13E-01	6,084764513	3,02E+00	8,25E-02	3,69E-01	Cdk2ap2	1,08	No
ENSMUSG00000024858	1,65E-02	7,588265282	7,85E-02	7,79E-01	9,48E-01	Grk2	1,01	No
ENSMUSG00000024862	-1,08E-01	4,079465586	1,52E+00	2,18E-01	5,50E-01	Klc2	0,93	No
ENSMUSG00000024866	3,79E-01	1,243705629	3,65E+00	5,60E-02	2,90E-01	Acy3	1,30	No
ENSMUSG00000024867	4,89E-01	-1,7706881	6,02E-01	4,38E-01	7,30E-01	Pip5k1b	1,40	No
ENSMUSG00000024868	9,01E-03	-3,107646452	-5,16E-10	1,00E+00	1,00E+00	Dkk1	1,01	No
ENSMUSG00000024869	3,56E-02	2,882000985	9,17E-02	7,62E-01	9,38E-01	Nudt8	1,02	No
ENSMUSG00000024870	7,38E-02	7,84327018	1,59E+00	2,07E-01	5,50E-01	Rab1b	1,05	No
ENSMUSG00000024871	1,75E-01	1,036804036	7,30E-01	3,93E-01	6,93E-01	Doc2g	1,13	No
ENSMUSG00000024873	-4,27E-01	-0,195678775	1,65E+00	1,99E-01	5,50E-01	Cnih2	0,74	No
ENSMUSG00000024875	1,40E-01	4,435032185	2,94E+00	8,65E-02	3,78E-01	Yif1a	1,10	No
ENSMUSG00000024878	2,61E-01	2,425612362	3,83E+00	5,02E-02	2,72E-01	Cbwd1	1,20	No
ENSMUSG00000024883	3,58E-02	0,9931722	2,99E-02	8,63E-01	9,97E-01	Rin1	1,03	No
ENSMUSG00000024885	-1,46E-02	6,526739211	5,42E-02	8,16E-01	9,69E-01	Aldh3b1	0,99	No
ENSMUSG00000024887	-1,28E-01	3,469763338	1,62E+00	2,04E-01	5,50E-01	Asah2	0,92	No
ENSMUSG00000024889	-4,42E-02	4,397772169	2,92E-01	5,89E-01	8,41E-01	Rce1	0,97	No
ENSMUSG00000024891	-9,47E-03	1,508835338	3,10E-03	9,56E-01	1,00E+00	Slc29a2	0,99	No
ENSMUSG00000024892	-2,18E-01	3,78724379	5,64E+00	1,76E-02	1,39E-01	Pcx	0,86	No
ENSMUSG00000024896	-3,52E-02	5,351227206	2,47E-01	6,19E-01	8,56E-01	Minpp1	0,98	No
ENSMUSG00000024897	1,47E-01	5,622602195	4,68E+00	3,05E-02	2,01E-01	Apba1	1,11	No
ENSMUSG00000024899	-1,40E-01	6,47901567	5,00E+00	2,54E-02	1,77E-01	Papss2	0,91	No
ENSMUSG00000024900	2,27E-01	7,205954476	1,43E+01	1,60E-04	3,59E-03	Cpt1a	1,17	Yes
ENSMUSG00000024901	3,56E-01	2,314407935	6,45E+00	1,11E-02	1,01E-01	Peli3	1,28	No
ENSMUSG00000024902	-8,05E-02	4,418761519	9,59E-01	3,27E-01	6,28E-01	Mrpl11	0,95	No
ENSMUSG00000024905	1,88E-01	0,015063556	4,11E-01	5,21E-01	8,00E-01	Tesmin	1,14	No
ENSMUSG00000024906	2,67E-01	3,313947369	6,68E+00	9,73E-03	9,15E-02	Mus81	1,20	No
ENSMUSG00000024907	-4,65E-01	-1,600679714	7,50E-01	3,86E-01	6,87E-01	Gal	0,72	No
ENSMUSG00000024908	5,16E-02	6,797101872	7,04E-01	4,01E-01	6,99E-01	Ppp6r3	1,04	No

ENSMUSG00000024909	-3,99E-02	2,399535822	8,94E-02	7,65E-01	9,39E-01	Efemp2	0,97	No
ENSMUSG00000024910	-1,15E+00	-2,107629914	2,16E+00	1,42E-01	4,76E-01	Ctsw	0,45	No
ENSMUSG00000024911	-6,17E-02	4,993957811	6,93E-01	4,05E-01	7,02E-01	Fibp	0,96	No
ENSMUSG00000024912	1,37E-02	0,521172016	3,03E-03	9,56E-01	1,00E+00	Fosl1	1,01	No
ENSMUSG00000024913	2,07E-03	5,094996521	7,87E-04	9,78E-01	1,00E+00	Lrp5	1,00	No
ENSMUSG00000024914	-1,16E-01	4,888587405	2,36E+00	1,24E-01	4,41E-01	Drap1	0,92	No
ENSMUSG00000024921	4,10E-02	6,014725615	3,93E-01	5,31E-01	8,05E-01	Smarca2	1,03	No
ENSMUSG00000024922	-2,79E-03	-1,726162952	2,21E-05	9,96E-01	1,00E+00	Ovol1	1,00	No
ENSMUSG00000024924	8,78E-01	-1,01576363	3,68E+00	5,52E-02	2,88E-01	Vldlr	1,84	No
ENSMUSG00000024925	-3,87E-03	4,66498452	2,44E-03	9,61E-01	1,00E+00	Rnaseh2c	1,00	No
ENSMUSG00000024926	4,47E-02	4,601532324	3,22E-01	5,70E-01	8,31E-01	Kat5	1,03	No
ENSMUSG00000024927	-1,58E-01	6,625629543	6,47E+00	1,10E-02	9,98E-02	Rela	0,90	No
ENSMUSG00000024935	-5,42E-01	-2,929296767	1,49E-01	7,00E-01	9,04E-01	Slc1a1	0,69	No
ENSMUSG00000024936	-3,27E-01	1,794460901	4,28E+00	3,85E-02	2,33E-01	Kcnk7	0,80	No
ENSMUSG00000024937	3,90E-02	7,839502865	4,46E-01	5,04E-01	7,89E-01	Ehbp1l1	1,03	No
ENSMUSG00000024939	1,25E-01	5,333406599	3,14E+00	7,62E-02	3,52E-01	Fam89b	1,09	No
ENSMUSG00000024940	-2,68E-01	4,716887879	1,18E+01	5,90E-04	1,05E-02	Ltbp3	0,83	Yes
ENSMUSG00000024941	5,58E-02	6,077848024	7,34E-01	3,91E-01	6,91E-01	Scyl1	1,04	No
ENSMUSG00000024942	-2,70E-02	6,894946396	1,95E-01	6,59E-01	8,81E-01	Capn1	0,98	No
ENSMUSG00000024943	6,32E-02	6,019999946	9,34E-01	3,34E-01	6,34E-01	Smc5	1,04	No
ENSMUSG00000024944	1,65E-01	4,047021747	3,52E+00	6,06E-02	3,05E-01	Arl2	1,12	No
ENSMUSG00000024947	4,52E-02	5,581600587	4,40E-01	5,07E-01	7,91E-01	Men1	1,03	No
ENSMUSG00000024948	-5,21E-02	6,090664004	6,48E-01	4,21E-01	7,16E-01	Map4k2	0,96	No
ENSMUSG00000024949	-2,57E-02	7,957200228	1,95E-01	6,59E-01	8,81E-01	Sf1	0,98	No
ENSMUSG00000024952	-8,47E-02	6,628653829	1,86E+00	1,72E-01	5,29E-01	Rps6ka4	0,94	No
ENSMUSG00000024953	-1,39E-01	7,855318603	5,63E+00	1,77E-02	1,39E-01	Prdx5	0,91	No
ENSMUSG00000024955	2,81E-02	5,569539852	1,69E-01	6,81E-01	8,94E-01	Esrra	1,02	No
ENSMUSG00000024957	6,85E-01	-2,311104076	6,81E-01	4,09E-01	7,06E-01	Kcnk4	1,61	No
ENSMUSG00000024958	1,10E-01	4,973915227	2,18E+00	1,40E-01	4,72E-01	Gpr137	1,08	No
ENSMUSG00000024959	-1,34E-01	4,919518338	3,22E+00	7,28E-02	3,42E-01	Bad	0,91	No
ENSMUSG00000024960	1,25E-01	5,480055792	3,27E+00	7,06E-02	3,36E-01	Plcb3	1,09	No
ENSMUSG00000024962	1,70E-01	3,779287072	3,31E+00	6,90E-02	3,31E-01	Vegfb	1,12	No

ENSMUSG00000024963	-6,69E-02	3,942159545	5,50E-01	4,58E-01	7,47E-01	Dnajc4	0,95	No
ENSMUSG00000024965	8,14E-02	8,209559842	1,98E+00	1,60E-01	5,07E-01	Fermt3	1,06	No
ENSMUSG00000024966	-7,87E-02	6,393471645	1,55E+00	2,13E-01	5,50E-01	Stip1	0,95	No
ENSMUSG00000024968	9,07E-01	-0,980884405	4,26E+00	3,90E-02	2,34E-01	Rcor2	1,88	No
ENSMUSG00000024969	1,55E-01	6,407918736	6,00E+00	1,43E-02	1,22E-01	Mark2	1,11	No
ENSMUSG00000024970	-1,26E-01	3,941013115	1,98E+00	1,59E-01	5,06E-01	AI846148	0,92	No
ENSMUSG00000024972	-1,88E+00	-2,963233427	1,42E+00	2,33E-01	5,50E-01	Lgals12	0,27	No
ENSMUSG00000024974	7,51E-02	5,891938295	1,29E+00	2,56E-01	5,50E-01	Smc3	1,05	No
ENSMUSG00000024975	-5,02E-01	5,40803876	5,28E+01	3,69E-13	4,74E-11	Pdcd4	0,71	Yes
ENSMUSG00000024976	8,56E-02	5,944793645	1,70E+00	1,92E-01	5,50E-01	Shoc2	1,06	No
ENSMUSG00000024978	1,69E-01	3,150309528	2,46E+00	1,17E-01	4,23E-01	Gpam	1,12	No
ENSMUSG00000024981	4,28E-02	7,062441306	5,01E-01	4,79E-01	7,66E-01	Acsl5	1,03	No
ENSMUSG00000024982	-3,46E-02	5,667605979	2,62E-01	6,09E-01	8,51E-01	Zdhhc6	0,98	No
ENSMUSG00000024983	-1,21E-01	5,449866459	3,05E+00	8,07E-02	3,64E-01	Vti1a	0,92	No
ENSMUSG00000024985	1,40E-01	5,234084292	3,78E+00	5,18E-02	2,77E-01	Tcf7l2	1,10	No
ENSMUSG00000024986	-1,20E-01	5,341508727	2,84E+00	9,22E-02	3,82E-01	Hhex	0,92	No
ENSMUSG00000024987	-4,13E-02	-3,033659495	7,08E-04	9,79E-01	1,00E+00	Cyp26a1	0,97	No
ENSMUSG00000024989	1,49E-01	5,117837192	4,20E+00	4,05E-02	2,38E-01	Cep55	1,11	No
ENSMUSG00000024990	-3,17E-02	3,807385626	1,16E-01	7,34E-01	9,23E-01	Rbp4	0,98	No
ENSMUSG00000024991	-3,94E-02	8,924165052	4,79E-01	4,89E-01	7,76E-01	Eif3a	0,97	No
ENSMUSG00000024992	6,94E-01	-2,89617475	2,61E-01	6,09E-01	8,51E-01	Pde6c	1,62	No
ENSMUSG00000024993	5,16E-02	5,278219255	5,23E-01	4,70E-01	7,58E-01	Fam45a	1,04	No
ENSMUSG00000024997	-1,03E-01	6,103456461	2,55E+00	1,10E-01	4,07E-01	Prdx3	0,93	No
ENSMUSG00000024998	1,54E+00	-1,929396785	4,50E+00	3,39E-02	2,16E-01	Plce1	2,90	No
ENSMUSG00000024999	9,32E-02	3,889673322	1,08E+00	2,99E-01	5,95E-01	Noc3l	1,07	No
ENSMUSG00000025001	2,34E-01	4,344555286	7,99E+00	4,70E-03	5,34E-02	Hells	1,18	No
ENSMUSG00000025002	-1,89E+00	-3,1076441	1,42E+00	2,33E-01	5,50E-01	Cyp2c55	0,27	No
ENSMUSG00000025006	-8,17E-01	-0,946116071	4,01E+00	4,52E-02	2,54E-01	Sorbs1	0,57	No
ENSMUSG00000025007	-6,59E-02	5,721401196	9,69E-01	3,25E-01	6,26E-01	Aldh18a1	0,96	No
ENSMUSG00000025008	2,65E-01	3,398698943	6,68E+00	9,75E-03	9,16E-02	Tctn3	1,20	No
ENSMUSG00000025010	5,71E-02	3,400615313	3,14E-01	5,75E-01	8,34E-01	Ccnj	1,04	No
ENSMUSG00000025013	3,14E-02	-3,107617333	-7,69E-10	1,00E+00	1,00E+00	Tll2	1,02	No

ENSMUSG00000025014	-7,42E-02	-2,401435329	6,29E-03	9,37E-01	1,00E+00	Dntt	0,95	No
ENSMUSG00000025016	8,44E-02	7,997425828	2,10E+00	1,47E-01	4,86E-01	Tm9sf3	1,06	No
ENSMUSG00000025017	1,61E-03	7,016493648	6,99E-04	9,79E-01	1,00E+00	Pik3ap1	1,00	No
ENSMUSG00000025019	3,64E-02	4,45469335	2,03E-01	6,52E-01	8,77E-01	Lcor	1,03	No
ENSMUSG00000025020	6,92E-01	-1,311195329	1,73E+00	1,88E-01	5,50E-01	Slit1	1,62	No
ENSMUSG00000025024	-5,60E-02	5,708270694	6,88E-01	4,07E-01	7,03E-01	Smndc1	0,96	No
ENSMUSG00000025025	-5,87E-02	5,417669474	7,10E-01	3,99E-01	6,98E-01	Mxi1	0,96	No
ENSMUSG00000025026	-2,69E-02	6,652240976	1,88E-01	6,65E-01	8,85E-01	Add3	0,98	No
ENSMUSG00000025027	6,25E-02	5,673519112	8,52E-01	3,56E-01	6,57E-01	Xpnpep1	1,04	No
ENSMUSG00000025034	9,17E-02	6,32951129	2,07E+00	1,50E-01	4,91E-01	Trim8	1,07	No
ENSMUSG00000025035	-2,30E-01	3,891893774	6,30E+00	1,20E-02	1,07E-01	Arl3	0,85	No
ENSMUSG00000025036	2,16E-01	4,157801873	6,22E+00	1,26E-02	1,11E-01	Sfxn2	1,16	No
ENSMUSG00000025041	-6,94E-02	5,073749144	8,88E-01	3,46E-01	6,47E-01	Nt5c2	0,95	No
ENSMUSG00000025044	1,26E-01	8,35501455	4,78E+00	2,89E-02	1,93E-01	Msr1	1,09	No
ENSMUSG00000025047	1,47E-01	5,580559651	4,65E+00	3,11E-02	2,04E-01	Pdcd11	1,11	No
ENSMUSG00000025049	1,15E-01	3,200609587	1,18E+00	2,78E-01	5,74E-01	Taf5	1,08	No
ENSMUSG00000025050	6,45E-02	2,352287345	2,34E-01	6,29E-01	8,62E-01	Pcgf6	1,05	No
ENSMUSG00000025060	4,91E-02	8,095541931	7,16E-01	3,97E-01	6,98E-01	Slk	1,03	No
ENSMUSG00000025064	-1,02E+00	-0,600573175	7,77E+00	5,30E-03	5,83E-02	Col17a1	0,49	No
ENSMUSG00000025066	-5,62E-02	6,044534184	7,44E-01	3,88E-01	6,88E-01	Sfr1	0,96	No
ENSMUSG00000025068	-1,93E-01	7,242573587	1,04E+01	1,24E-03	1,93E-02	Gsto1	0,87	Yes
ENSMUSG00000025069	1,70E-01	-1,070162612	1,25E-01	7,24E-01	9,18E-01	Gsto2	1,13	No
ENSMUSG00000025075	1,17E+00	-3,14612376	6,40E-01	4,24E-01	7,18E-01	Habp2	2,26	No
ENSMUSG00000025076	1,19E-01	6,123149641	3,39E+00	6,56E-02	3,21E-01	Casp7	1,09	No
ENSMUSG00000025077	9,26E-02	4,314687526	1,26E+00	2,62E-01	5,56E-01	Dclre1a	1,07	No
ENSMUSG00000025078	-1,13E-01	5,34023622	2,57E+00	1,09E-01	4,05E-01	Nhlrc2	0,92	No
ENSMUSG00000025081	9,01E-03	-3,107646452	-5,16E-10	1,00E+00	1,00E+00	Tdrd1	1,01	No
ENSMUSG00000025083	-2,85E-01	1,396204395	2,79E+00	9,48E-02	3,85E-01	Afap1l2	0,82	No
ENSMUSG00000025085	-6,48E-01	3,674734599	4,73E+01	5,99E-12	6,60E-10	Ablim1	0,64	Yes
ENSMUSG00000025086	1,88E-01	3,100573546	2,91E+00	8,78E-02	3,78E-01	Trub1	1,14	No
ENSMUSG00000025089	3,89E-01	0,266096304	2,04E+00	1,53E-01	4,95E-01	Gfra1	1,31	No
ENSMUSG00000025091	9,01E-03	-3,107646452	-5,16E-10	1,00E+00	1,00E+00	Pnliprp2	1,01	No

ENSMUSG00000025092	9,90E-02	-1,311189973	3,89E-02	8,44E-01	9,85E-01	Hspa12a		1,07	No
ENSMUSG00000025094	1,56E-02	1,364874753	7,53E-03	9,31E-01	1,00E+00	Slc18a2		1,01	No
ENSMUSG00000025102	-2,75E-01	3,199899871	6,82E+00	9,00E-03	8,64E-02	3110040N11Rik		0,83	No
ENSMUSG00000025103	4,95E-02	5,670674526	5,32E-01	4,66E-01	7,54E-01	Btbd1		1,03	No
ENSMUSG00000025104	1,70E-01	6,05351478	6,79E+00	9,15E-03	8,74E-02	Hdgfl3		1,13	No
ENSMUSG00000025105	1,87E+00	-3,107639691	1,38E+00	2,40E-01	5,50E-01	Bnc1		3,67	No
ENSMUSG00000025127	7,18E-01	-2,770576837	2,82E-01	5,96E-01	8,44E-01	Gcgr		1,65	No
ENSMUSG00000025128	3,14E-02	-3,107617333	-7,69E-10	1,00E+00	1,00E+00	Bhlhe22		1,02	No
ENSMUSG00000025129	-2,73E-02	-2,963256629	3,40E-04	9,85E-01	1,00E+00	Ppp1r27		0,98	No
ENSMUSG00000025130	1,36E-01	9,628413773	5,79E+00	1,61E-02	1,32E-01	P4hb		1,10	No
ENSMUSG00000025132	4,10E-02	9,830167576	5,29E-01	4,67E-01	7,55E-01	Arhgdia		1,03	No
ENSMUSG00000025133	1,09E-01	5,208523689	2,31E+00	1,28E-01	4,50E-01	Ints4		1,08	No
ENSMUSG00000025134	8,73E-02	5,887380799	1,74E+00	1,88E-01	5,50E-01	Alyref		1,06	No
ENSMUSG00000025135	9,43E-02	6,086600743	2,11E+00	1,46E-01	4,84E-01	Anapc11		1,07	No
ENSMUSG00000025137	1,12E-01	5,715188122	2,76E+00	9,66E-02	3,88E-01	Pcyt2		1,08	No
ENSMUSG00000025138	1,04E-02	6,163414624	2,62E-02	8,71E-01	1,00E+00	Sirt7		1,01	No
ENSMUSG00000025139	8,30E-02	7,069235244	1,88E+00	1,70E-01	5,26E-01	Tollip		1,06	No
ENSMUSG00000025140	7,46E-01	-0,778159139	3,33E+00	6,80E-02	3,28E-01	Pycr1		1,68	No
ENSMUSG00000025142	-2,54E-02	4,880080995	1,13E-01	7,37E-01	9,25E-01	Aspscr1		0,98	No
ENSMUSG00000025144	9,22E-02	4,630712468	1,37E+00	2,42E-01	5,50E-01	Cenpx		1,07	No
ENSMUSG00000025145	1,03E-01	4,754452628	1,82E+00	1,77E-01	5,37E-01	Lrrc45		1,07	No
ENSMUSG00000025147	3,50E-02	4,769207314	2,09E-01	6,47E-01	8,75E-01	Mob2		1,02	No
ENSMUSG00000025150	8,11E-01	-1,600725113	1,83E+00	1,76E-01	5,35E-01	Cbr2		1,75	No
ENSMUSG00000025153	3,14E-01	8,474233318	2,98E+01	4,85E-08	2,90E-06	Fasn		1,24	Yes
ENSMUSG00000025154	2,84E-01	5,503559512	1,69E+01	4,02E-05	1,13E-03	Arhgap19		1,22	Yes
ENSMUSG00000025155	5,27E-02	5,011494123	5,05E-01	4,77E-01	7,65E-01	Dus1l		1,04	No
ENSMUSG00000025156	-2,04E-02	4,816752351	7,13E-02	7,89E-01	9,54E-01	Gps1		0,99	No
ENSMUSG00000025157	1,57E-02	4,516027234	3,91E-02	8,43E-01	9,85E-01	Zdhhc16		1,01	No
ENSMUSG00000025158	-7,28E-03	4,387521272	7,76E-03	9,30E-01	1,00E+00	Rfng		0,99	No
ENSMUSG00000025159	2,78E-03	6,178281163	1,87E-03	9,66E-01	1,00E+00	Mms19		1,00	No
ENSMUSG00000025161	1,95E-01	7,36729964	1,06E+01	1,12E-03	1,77E-02	Slc16a3		1,14	Yes
ENSMUSG00000025162	-7,41E-02	7,568218715	1,58E+00	2,09E-01	5,50E-01	Csnk1d		0,95	No

ENSMUSG00000025163	-8,89E-02	0,696823393	1,50E-01	6,99E-01	9,04E-01	Cd7	0,94	No
ENSMUSG00000025165	1,01E+00	1,312535087	2,79E+01	1,31E-07	7,10E-06	Sectm1a	2,02	Yes
ENSMUSG00000025169	-1,12E-01	1,856069643	5,18E-01	4,72E-01	7,60E-01	Ogfod3	0,93	No
ENSMUSG00000025170	9,80E-01	-1,497578289	3,15E+00	7,57E-02	3,50E-01	Rab40b	1,97	No
ENSMUSG00000025171	9,51E-02	5,616288933	1,92E+00	1,66E-01	5,18E-01	Ubtd1	1,07	No
ENSMUSG00000025172	7,34E-01	-2,896175673	2,93E-01	5,88E-01	8,40E-01	Ankrd2	1,66	No
ENSMUSG00000025173	-2,59E-03	5,828299332	1,51E-03	9,69E-01	1,00E+00	Wdr45b	1,00	No
ENSMUSG00000025175	1,10E+00	-2,401451033	1,15E+00	2,83E-01	5,79E-01	Fn3k	2,15	No
ENSMUSG00000025176	-3,49E-01	0,351929825	1,79E+00	1,81E-01	5,43E-01	Hoga1	0,79	No
ENSMUSG00000025178	7,28E-02	7,278700561	1,48E+00	2,24E-01	5,50E-01	Pi4k2a	1,05	No
ENSMUSG00000025184	-1,69E-03	4,200487011	3,97E-04	9,84E-01	1,00E+00	R3hcc1l	1,00	No
ENSMUSG00000025185	-6,18E-01	-2,185707564	5,21E-01	4,71E-01	7,59E-01	Loxl4	0,65	No
ENSMUSG00000025188	8,23E-02	5,026506872	1,25E+00	2,64E-01	5,59E-01	Hps1	1,06	No
ENSMUSG00000025189	-1,89E+00	-3,1076441	1,42E+00	2,33E-01	5,50E-01	Cnnm1	0,27	No
ENSMUSG00000025190	1,13E-01	6,61948382	3,29E+00	6,97E-02	3,33E-01	Got1	1,08	No
ENSMUSG00000025192	2,44E-01	4,299256683	8,58E+00	3,39E-03	4,20E-02	Entpd7	1,18	Yes
ENSMUSG00000025193	1,09E-01	2,508134055	7,08E-01	4,00E-01	6,99E-01	Cutc	1,08	No
ENSMUSG00000025194	-1,31E+00	-1,522465906	6,11E+00	1,35E-02	1,16E-01	Abcc2	0,40	No
ENSMUSG00000025195	2,10E-01	4,30362656	6,28E+00	1,22E-02	1,08E-01	Dnmbp	1,16	No
ENSMUSG00000025196	5,07E-01	-2,401341631	3,50E-01	5,54E-01	8,20E-01	Cpn1	1,42	No
ENSMUSG00000025197	-1,86E+00	-3,033612668	1,39E+00	2,38E-01	5,50E-01	Cyp2c44	0,28	No
ENSMUSG00000025198	-1,40E-01	5,525183145	4,20E+00	4,04E-02	2,38E-01	Erlin1	0,91	No
ENSMUSG00000025199	-1,42E-02	6,115710355	4,83E-02	8,26E-01	9,75E-01	Chuk	0,99	No
ENSMUSG00000025200	4,36E-02	3,500192493	1,93E-01	6,61E-01	8,82E-01	Cwf19l1	1,03	No
ENSMUSG00000025201	-1,72E-01	4,923377011	5,24E+00	2,21E-02	1,61E-01	Bloc1s2-ps	0,89	No
ENSMUSG00000025202	8,77E-02	1,91906259	3,30E-01	5,66E-01	8,28E-01	Scd3	1,06	No
ENSMUSG00000025203	4,76E-01	10,4820825	7,18E+01	2,40E-17	4,65E-15	Scd2	1,39	Yes
ENSMUSG00000025204	7,40E-02	5,772207154	1,21E+00	2,71E-01	5,67E-01	Ndufb8	1,05	No
ENSMUSG00000025207	7,66E-01	-1,711759838	1,44E+00	2,31E-01	5,50E-01	Sema4g	1,70	No
ENSMUSG00000025208	-4,96E-02	5,670205283	5,34E-01	4,65E-01	7,54E-01	Mrpl43	0,97	No
ENSMUSG00000025209	-2,48E-02	3,757199868	7,00E-02	7,91E-01	9,55E-01	Twnk	0,98	No
ENSMUSG00000025212	2,76E-01	6,105458342	1,81E+01	2,09E-05	6,48E-04	Sfxn3	1,21	Yes

ENSMUSG00000025213	-4,10E-02	2,216191934	7,89E-02	7,79E-01	9,47E-01	Kazald1	0,97	No
ENSMUSG00000025217	-1,05E-01	4,355376354	1,62E+00	2,03E-01	5,50E-01	Btrc	0,93	No
ENSMUSG00000025218	-3,22E-02	3,771452705	1,22E-01	7,27E-01	9,19E-01	Poll	0,98	No
ENSMUSG00000025220	-1,53E-01	7,343447461	6,62E+00	1,01E-02	9,38E-02	Mgea5	0,90	No
ENSMUSG00000025221	6,07E-01	-2,548194757	4,11E-01	5,21E-01	8,00E-01	Kcnip2	1,52	No
ENSMUSG00000025223	-9,23E-02	6,4123612	2,14E+00	1,44E-01	4,79E-01	Ldb1	0,94	No
ENSMUSG00000025224	9,23E-03	6,443848174	2,14E-02	8,84E-01	1,00E+00	Gbf1	1,01	No
ENSMUSG00000025225	-4,88E-01	7,308658691	6,70E+01	2,68E-16	4,61E-14	Nfkb2	0,71	Yes
ENSMUSG00000025226	-3,27E-02	2,561941507	6,52E-02	7,98E-01	9,59E-01	Fbxl15	0,98	No
ENSMUSG00000025227	1,05E-01	5,150432482	2,08E+00	1,49E-01	4,90E-01	Mfsd13a	1,08	No
ENSMUSG00000025228	3,18E-02	7,633306979	2,92E-01	5,89E-01	8,41E-01	Actr1a	1,02	No
ENSMUSG00000025231	9,21E-04	4,54762606	1,33E-04	9,91E-01	1,00E+00	Sufu	1,00	No
ENSMUSG00000025232	1,85E-01	8,242642455	1,02E+01	1,40E-03	2,12E-02	Hexa	1,14	Yes
ENSMUSG00000025234	1,46E-02	7,299424749	5,99E-02	8,07E-01	9,64E-01	Arih1	1,01	No
ENSMUSG00000025235	-4,33E-02	2,294656768	9,73E-02	7,55E-01	9,34E-01	Bbs4	0,97	No
ENSMUSG00000025236	7,51E-02	5,837790062	1,28E+00	2,58E-01	5,51E-01	Adpgk	1,05	No
ENSMUSG00000025237	-1,76E-01	3,959409222	3,91E+00	4,81E-02	2,64E-01	Parp6	0,89	No
ENSMUSG00000025239	-7,44E-03	7,568780582	1,59E-02	9,00E-01	1,00E+00	Limd1	0,99	No
ENSMUSG00000025240	3,05E-02	5,15123457	1,77E-01	6,74E-01	8,90E-01	Sacm1l	1,02	No
ENSMUSG00000025241	-2,36E-01	6,012929116	1,32E+01	2,81E-04	5,75E-03	Fyco1	0,85	Yes
ENSMUSG00000025243	5,53E-02	4,255014262	4,33E-01	5,11E-01	7,94E-01	Slc6a20b	1,04	No
ENSMUSG00000025245	-1,87E-01	3,688903651	3,85E+00	4,99E-02	2,70E-01	Lztfl1	0,88	No
ENSMUSG00000025255	1,25E+00	-2,600671436	9,71E-01	3,24E-01	6,25E-01	Zfhx4	2,38	No
ENSMUSG00000025277	-1,66E-01	-0,485155752	2,11E-01	6,46E-01	8,74E-01	Abhd6	0,89	No
ENSMUSG00000025278	-2,25E-01	5,938740441	1,18E+01	5,91E-04	1,05E-02	Flnb	0,86	Yes
ENSMUSG00000025279	-6,78E-01	-0,573930471	3,30E+00	6,95E-02	3,32E-01	Dnase1l3	0,63	No
ENSMUSG00000025280	1,15E-01	4,802024151	2,26E+00	1,33E-01	4,59E-01	Polr3a	1,08	No
ENSMUSG00000025290	-8,88E-02	6,275013137	1,94E+00	1,63E-01	5,14E-01	Rps24	0,94	No
ENSMUSG00000025314	7,06E-02	7,725624819	1,44E+00	2,30E-01	5,50E-01	Ptprj	1,05	No
ENSMUSG00000025316	9,93E-02	4,116168658	1,34E+00	2,47E-01	5,50E-01	Banp	1,07	No
ENSMUSG00000025317	2,67E-01	-2,770639914	4,41E-02	8,34E-01	9,79E-01	Car5a	1,20	No
ENSMUSG00000025318	3,03E-01	-0,236636932	8,23E-01	3,64E-01	6,65E-01	Jph3	1,23	No

ENSMUSG00000025321	-3,16E-01	-1,682983136	2,79E-01	5,97E-01	8,45E-01	Itgb8	0,80	No
ENSMUSG00000025323	2,00E-02	3,244663834	3,58E-02	8,50E-01	9,89E-01	Sp4	1,01	No
ENSMUSG00000025324	2,26E-01	5,081174734	9,46E+00	2,09E-03	2,90E-02	Atp10a	1,17	Yes
ENSMUSG00000025326	1,94E-01	6,024378948	8,82E+00	2,98E-03	3,82E-02	Ube3a	1,14	Yes
ENSMUSG00000025328	1,33E-01	-1,127033698	9,29E-02	7,61E-01	9,37E-01	Padi3	1,10	No
ENSMUSG00000025329	-2,76E-02	-2,929311596	3,75E-04	9,85E-01	1,00E+00	Padi1	0,98	No
ENSMUSG00000025330	-8,77E-03	1,488881195	2,47E-03	9,60E-01	1,00E+00	Padi4	0,99	No
ENSMUSG00000025337	-1,70E-01	5,368830535	5,90E+00	1,51E-02	1,27E-01	Sbds	0,89	No
ENSMUSG00000025340	-1,91E-01	5,354410395	7,32E+00	6,80E-03	7,03E-02	Rabgef1	0,88	No
ENSMUSG00000025348	-6,21E-02	-2,033624901	8,07E-03	9,28E-01	1,00E+00	Itga7	0,96	No
ENSMUSG00000025350	-1,65E-01	1,94360857	1,17E+00	2,79E-01	5,76E-01	Rdh5	0,89	No
ENSMUSG00000025351	-5,74E-03	6,103523679	7,84E-03	9,29E-01	1,00E+00	Cd63	1,00	No
ENSMUSG00000025352	-6,24E-01	-0,355733976	3,38E+00	6,60E-02	3,23E-01	Gdf11	0,65	No
ENSMUSG00000025353	2,28E-02	4,505403065	7,97E-02	7,78E-01	9,47E-01	Ormdl2	1,02	No
ENSMUSG00000025354	2,00E-02	6,674673449	1,05E-01	7,46E-01	9,29E-01	Dnajc14	1,01	No
ENSMUSG00000025355	3,97E-03	7,427935638	4,43E-03	9,47E-01	1,00E+00	Mmp19	1,00	No
ENSMUSG00000025357	-5,72E-01	5,599038547	7,11E+01	3,45E-17	6,51E-15	Dgka	0,67	Yes
ENSMUSG00000025358	-8,82E-02	6,284731085	1,92E+00	1,66E-01	5,18E-01	Cdk2	0,94	No
ENSMUSG00000025359	-3,78E-01	2,446242804	8,65E+00	3,28E-03	4,10E-02	Pmel	0,77	Yes
ENSMUSG00000025362	-2,52E-01	6,164820856	1,53E+01	9,39E-05	2,30E-03	Rps26	0,84	Yes
ENSMUSG00000025364	6,86E-02	6,065442378	1,11E+00	2,93E-01	5,88E-01	Pa2g4	1,05	No
ENSMUSG00000025366	1,91E-01	9,131342126	1,13E+01	7,57E-04	1,29E-02	Esyt1	1,14	Yes
ENSMUSG00000025369	2,58E-03	7,190372222	1,84E-03	9,66E-01	1,00E+00	Smarcc2	1,00	No
ENSMUSG00000025370	-1,87E+00	-3,107637761	1,39E+00	2,38E-01	5,50E-01	Cdh9	0,27	No
ENSMUSG00000025371	1,37E-01	4,678050021	3,10E+00	7,83E-02	3,57E-01	Chmp6	1,10	No
ENSMUSG00000025372	7,95E-03	6,296587452	1,57E-02	9,00E-01	1,00E+00	Baiap2	1,01	No
ENSMUSG00000025373	1,37E-01	5,652330465	4,05E+00	4,41E-02	2,50E-01	Rnf41	1,10	No
ENSMUSG00000025374	-1,16E-01	4,86644291	2,33E+00	1,27E-01	4,47E-01	Nabp2	0,92	No
ENSMUSG00000025375	4,04E-01	4,69429566	2,66E+01	2,47E-07	1,27E-05	Aatk	1,32	Yes
ENSMUSG00000025377	1,45E-01	4,977052849	3,79E+00	5,14E-02	2,76E-01	Tepsin	1,11	No
ENSMUSG00000025380	-3,36E-01	-0,338401346	8,90E-01	3,45E-01	6,46E-01	Fscn2	0,79	No
ENSMUSG00000025381	-7,05E-02	4,966744313	8,94E-01	3,44E-01	6,45E-01	Cnpy2	0,95	No

ENSMUSG00000025383	-1,25E+00	-1,801016055	3,70E+00	5,43E-02	2,85E-01	Il23a	0,42	No
ENSMUSG00000025384	-3,16E-02	4,511629625	1,56E-01	6,92E-01	9,01E-01	Faap100	0,98	No
ENSMUSG00000025386	-1,31E+00	-2,83198662	1,06E+00	3,02E-01	5,99E-01	Pde6g	0,40	No
ENSMUSG00000025389	1,70E-01	-2,627601804	2,44E-02	8,76E-01	1,00E+00	Mip	1,12	No
ENSMUSG00000025393	6,85E-03	9,85476963	1,48E-02	9,03E-01	1,00E+00	Atp5b	1,00	No
ENSMUSG00000025395	2,23E-01	3,948165352	6,03E+00	1,41E-02	1,20E-01	Prim1	1,17	No
ENSMUSG00000025401	1,71E-01	-1,711737954	8,89E-02	7,66E-01	9,40E-01	Myo1a	1,13	No
ENSMUSG00000025402	3,00E-01	4,822052695	1,58E+01	7,07E-05	1,82E-03	Nab2	1,23	Yes
ENSMUSG00000025403	1,19E-01	5,487576828	2,94E+00	8,66E-02	3,78E-01	Shmt2	1,09	No
ENSMUSG00000025404	-2,81E-02	6,195416295	1,91E-01	6,62E-01	8,83E-01	R3hdm2	0,98	No
ENSMUSG00000025407	3,01E-01	0,532896254	1,53E+00	2,17E-01	5,50E-01	Gli1	1,23	No
ENSMUSG00000025408	-1,13E-01	4,382424948	1,91E+00	1,67E-01	5,21E-01	Ddit3	0,92	No
ENSMUSG00000025409	7,07E-02	6,197571449	1,22E+00	2,69E-01	5,65E-01	Mbd6	1,05	No
ENSMUSG00000025410	-8,92E-02	6,951065781	2,15E+00	1,43E-01	4,77E-01	Dctn2	0,94	No
ENSMUSG00000025413	-1,22E-01	4,67601466	2,49E+00	1,15E-01	4,18E-01	Ttc4	0,92	No
ENSMUSG00000025417	-8,90E-02	6,586855571	2,05E+00	1,53E-01	4,95E-01	Pip4k2c	0,94	No
ENSMUSG00000025420	-5,24E-01	-2,18554363	4,41E-01	5,07E-01	7,91E-01	Katnal2	0,70	No
ENSMUSG00000025421	-3,65E-04	4,801053694	2,29E-05	9,96E-01	1,00E+00	Hdhd2	1,00	No
ENSMUSG00000025422	2,03E-01	4,640063176	6,77E+00	9,30E-03	8,84E-02	Agap2	1,15	No
ENSMUSG00000025423	6,51E-02	4,762647266	7,13E-01	3,98E-01	6,98E-01	Pias2	1,05	No
ENSMUSG00000025425	1,81E+00	-3,10765737	1,28E+00	2,58E-01	5,50E-01	St8sia5	3,49	No
ENSMUSG00000025427	1,81E+00	-3,10765737	1,28E+00	2,58E-01	5,50E-01	Rnf165	3,49	No
ENSMUSG00000025428	1,24E-02	9,527348965	4,81E-02	8,26E-01	9,75E-01	Atp5a1	1,01	No
ENSMUSG00000025429	-8,01E-02	4,208587525	8,86E-01	3,47E-01	6,47E-01	Pstpip2	0,95	No
ENSMUSG00000025431	3,41E-01	1,590445773	3,67E+00	5,55E-02	2,89E-01	Crisp1	1,27	No
ENSMUSG00000025432	-2,54E-01	-0,367100333	6,11E-01	4,34E-01	7,27E-01	Avil	0,84	No
ENSMUSG00000025433	-8,34E-02	-1,770504141	1,78E-02	8,94E-01	1,00E+00	Crisp3	0,94	No
ENSMUSG00000025436	1,27E-01	2,157617313	7,79E-01	3,77E-01	6,78E-01	Atp23	1,09	No
ENSMUSG00000025437	-9,32E-03	5,628766129	1,86E-02	8,91E-01	1,00E+00	Usp33	0,99	No
ENSMUSG00000025439	3,00E-02	5,316236872	1,79E-01	6,72E-01	8,89E-01	Clns1a	1,02	No
ENSMUSG00000025450	-1,60E-01	2,245893201	1,35E+00	2,46E-01	5,50E-01	Gm9752	0,89	No
ENSMUSG00000025451	1,03E-01	5,000250889	1,94E+00	1,64E-01	5,15E-01	Paip1	1,07	No

ENSMUSG00000025453	-2,69E-03	4,509009188	1,12E-03	9,73E-01	1,00E+00	Nnt	1,00	No
ENSMUSG00000025464	4,55E-01	3,041604913	1,67E+01	4,30E-05	1,20E-03	Paox	1,37	Yes
ENSMUSG00000025465	7,30E-02	6,005548488	1,23E+00	2,67E-01	5,63E-01	Echs1	1,05	No
ENSMUSG00000025466	1,97E-01	2,094675288	1,80E+00	1,80E-01	5,41E-01	Fuom	1,15	No
ENSMUSG00000025467	-1,25E+00	-3,070160443	7,24E-01	3,95E-01	6,95E-01	Prap1	0,42	No
ENSMUSG00000025468	-6,06E-01	-2,226321941	4,99E-01	4,80E-01	7,67E-01	Caly	0,66	No
ENSMUSG00000025470	-1,27E-01	3,779780984	1,86E+00	1,73E-01	5,30E-01	Zfp511	0,92	No
ENSMUSG00000025473	-7,65E-02	9,481510113	1,83E+00	1,76E-01	5,35E-01	Adam8	0,95	No
ENSMUSG00000025474	-2,10E-02	5,165034424	8,49E-02	7,71E-01	9,42E-01	Tubgcp2	0,99	No
ENSMUSG00000025475	1,87E+00	-3,107639691	1,38E+00	2,40E-01	5,50E-01	Adgra1	3,67	No
ENSMUSG00000025477	7,34E-02	4,270144445	7,51E-01	3,86E-01	6,87E-01	Inpp5a	1,05	No
ENSMUSG00000025478	-1,37E+00	-1,997878098	3,96E+00	4,66E-02	2,58E-01	Dpysl4	0,39	No
ENSMUSG00000025479	-1,89E+00	-3,033648118	1,42E+00	2,33E-01	5,50E-01	Cyp2e1	0,27	No
ENSMUSG00000025480	-3,83E-02	-3,107651936	-7,91E-11	1,00E+00	1,00E+00	Syce1	0,97	No
ENSMUSG00000025481	-2,71E+00	-2,963235336	2,92E+00	8,75E-02	3,78E-01	Urah	0,15	No
ENSMUSG00000025482	-6,43E-02	-2,600633366	2,78E-03	9,58E-01	1,00E+00	Odf3	0,96	No
ENSMUSG00000025484	-3,08E-02	5,187024786	1,82E-01	6,69E-01	8,88E-01	Bet1l	0,98	No
ENSMUSG00000025485	-1,76E-02	6,882125426	8,32E-02	7,73E-01	9,44E-01	Ric8a	0,99	No
ENSMUSG00000025486	-3,45E-02	3,853183174	1,43E-01	7,06E-01	9,07E-01	Sirt3	0,98	No
ENSMUSG00000025487	4,50E-02	6,000911352	4,69E-01	4,93E-01	7,79E-01	Psmd13	1,03	No
ENSMUSG00000025488	-1,87E+00	-3,03364178	1,41E+00	2,36E-01	5,50E-01	Cox8b	0,27	No
ENSMUSG00000025489	-7,76E-01	-2,205726774	7,76E-01	3,78E-01	6,79E-01	Ifitm5	0,58	No
ENSMUSG00000025491	-8,41E-01	7,724128077	2,00E+02	2,45E-45	4,33E-42	Ifitm1	0,56	Yes
ENSMUSG00000025492	-9,42E-01	6,772102458	2,28E+02	2,06E-51	5,14E-48	Ifitm3	0,52	Yes
ENSMUSG00000025494	-2,32E-01	3,555815494	5,80E+00	1,60E-02	1,31E-01	Sigirr	0,85	No
ENSMUSG00000025495	-9,72E-02	4,737936981	1,62E+00	2,04E-01	5,50E-01	Ptdss2	0,93	No
ENSMUSG00000025496	-1,87E+00	-3,03364178	1,41E+00	2,36E-01	5,50E-01	Drd4	0,27	No
ENSMUSG00000025497	-1,94E+00	-3,107619243	1,50E+00	2,21E-01	5,50E-01	Cdhr5	0,26	No
ENSMUSG00000025498	-3,63E-01	2,987089585	1,06E+01	1,15E-03	1,82E-02	Irf7	0,78	Yes
ENSMUSG00000025499	2,43E-02	5,522041372	1,25E-01	7,24E-01	9,17E-01	Hras	1,02	No
ENSMUSG00000025500	-4,72E-01	0,524375864	3,71E+00	5,42E-02	2,85E-01	Lmntd2	0,72	No
ENSMUSG00000025503	-1,76E-02	7,853459137	9,04E-02	7,64E-01	9,39E-01	Taldo1	0,99	No

ENSMUSG00000025504	-1,11E+00	-0,107406263	1,24E+01	4,31E-04	8,10E-03	Eps8l2	0,46	Yes
ENSMUSG00000025505	-1,18E-01	3,155007805	1,22E+00	2,70E-01	5,67E-01	Tmem80	0,92	No
ENSMUSG00000025507	1,31E-01	4,348530104	2,54E+00	1,11E-01	4,09E-01	Pidd1	1,09	No
ENSMUSG00000025508	3,79E-02	4,091031088	1,88E-01	6,65E-01	8,84E-01	Rplp2	1,03	No
ENSMUSG00000025509	4,01E-02	6,371314842	4,00E-01	5,27E-01	8,04E-01	Pnpla2	1,03	No
ENSMUSG00000025510	3,32E-01	5,268940467	2,17E+01	3,20E-06	1,23E-04	Cd151	1,26	Yes
ENSMUSG00000025511	2,19E-01	4,343609424	6,79E+00	9,19E-03	8,77E-02	Tspan4	1,16	No
ENSMUSG00000025512	1,25E-01	5,365533059	3,14E+00	7,64E-02	3,52E-01	Chid1	1,09	No
ENSMUSG00000025515	5,69E-01	-1,929350851	5,49E-01	4,59E-01	7,48E-01	Muc2	1,48	No
ENSMUSG00000025519	-1,88E+00	-2,770579395	1,42E+00	2,33E-01	5,50E-01	Tktl2	0,27	No
ENSMUSG00000025521	1,58E-01	4,868998872	4,30E+00	3,82E-02	2,32E-01	Tmem192	1,12	No
ENSMUSG00000025532	-5,56E-03	4,491749869	4,85E-03	9,45E-01	1,00E+00	Crcp	1,00	No
ENSMUSG00000025533	-6,89E-02	4,938639914	8,61E-01	3,54E-01	6,55E-01	Asl	0,95	No
ENSMUSG00000025534	1,65E-01	8,603227651	8,32E+00	3,92E-03	4,66E-02	Gusb	1,12	Yes
ENSMUSG00000025537	6,84E-02	-1,43678971	1,89E-02	8,91E-01	1,00E+00	Phkg1	1,05	No
ENSMUSG00000025538	3,04E-01	3,569194076	9,64E+00	1,90E-03	2,69E-02	Sumf2	1,23	Yes
ENSMUSG00000025544	5,55E-02	6,777160931	8,13E-01	3,67E-01	6,68E-01	Tm9sf2	1,04	No
ENSMUSG00000025545	1,83E-01	2,833176409	2,43E+00	1,19E-01	4,28E-01	Clybl	1,14	No
ENSMUSG00000025555	-7,43E-02	-0,535437205	4,74E-02	8,28E-01	9,76E-01	Farp1	0,95	No
ENSMUSG00000025557	-1,89E+00	-3,1076441	1,42E+00	2,33E-01	5,50E-01	Slc15a1	0,27	No
ENSMUSG00000025558	-8,15E-01	3,967545658	8,48E+01	3,26E-20	7,57E-18	Dock9	0,57	Yes
ENSMUSG00000025571	1,38E-01	5,533872133	3,97E+00	4,63E-02	2,57E-01	Tnrc6c	1,10	No
ENSMUSG00000025572	-3,13E-01	5,434240295	2,05E+01	6,00E-06	2,13E-04	Tmc6	0,80	Yes
ENSMUSG00000025573	-3,77E-01	0,493427647	2,52E+00	1,13E-01	4,13E-01	6030468B19Rik	0,77	No
ENSMUSG00000025574	-3,21E-01	5,984881967	2,41E+01	9,09E-07	4,10E-05	Tk1	0,80	Yes
ENSMUSG00000025575	3,30E-02	6,364165459	2,70E-01	6,03E-01	8,48E-01	Cant1	1,02	No
ENSMUSG00000025576	-1,89E+00	-3,1076441	1,42E+00	2,33E-01	5,50E-01	Rbfox3	0,27	No
ENSMUSG00000025577	-8,08E-02	2,441912424	4,11E-01	5,21E-01	8,00E-01	Cbx2	0,95	No
ENSMUSG00000025578	7,28E-01	1,010171559	1,31E+01	2,93E-04	5,95E-03	Cbx8	1,66	Yes
ENSMUSG00000025579	5,71E-02	5,937233047	7,52E-01	3,86E-01	6,87E-01	Gaa	1,04	No
ENSMUSG00000025580	3,17E-02	4,346480269	1,47E-01	7,02E-01	9,05E-01	Eif4a3	1,02	No
ENSMUSG00000025582	-5,40E-02	-2,832035784	1,96E-03	9,65E-01	1,00E+00	Nptx1	0,96	No

ENSMUSG00000025583	-2,10E-01	5,905451847	1,02E+01	1,38E-03	2,09E-02	Rptor	0,86	Yes
ENSMUSG00000025584	1,69E-01	5,402871968	5,73E+00	1,67E-02	1,35E-01	Pde8a	1,12	No
ENSMUSG00000025586	4,44E-01	3,969680646	2,43E+01	8,35E-07	3,81E-05	Cpeb1	1,36	Yes
ENSMUSG00000025588	2,62E+00	-2,963288024	2,65E+00	1,03E-01	3,95E-01	Nat1	6,14	No
ENSMUSG00000025591	-1,49E-01	6,401175742	5,51E+00	1,89E-02	1,46E-01	Tma16	0,90	No
ENSMUSG00000025602	1,79E-01	2,630205217	2,04E+00	1,53E-01	4,96E-01	Zfp202	1,13	No
ENSMUSG00000025607	-1,50E-02	5,292629233	4,46E-02	8,33E-01	9,78E-01	Copg2	0,99	No
ENSMUSG00000025608	-1,32E+00	-2,655080948	1,09E+00	2,97E-01	5,93E-01	Podxl	0,40	No
ENSMUSG00000025609	-1,65E-01	5,989663012	6,28E+00	1,22E-02	1,08E-01	Mkln1	0,89	No
ENSMUSG00000025610	9,01E-03	-3,107646452	-5,16E-10	1,00E+00	1,00E+00	Map3k7cl	1,01	No
ENSMUSG00000025612	2,11E-01	7,187533025	1,22E+01	4,67E-04	8,68E-03	Bach1	1,16	Yes
ENSMUSG00000025613	3,45E-02	7,155447938	3,28E-01	5,67E-01	8,29E-01	Cct8	1,02	No
ENSMUSG00000025616	-1,15E-01	5,548661646	2,78E+00	9,56E-02	3,86E-01	Usp16	0,92	No
ENSMUSG00000025645	-2,97E-02	2,505975909	5,59E-02	8,13E-01	9,67E-01	Ccdc51	0,98	No
ENSMUSG00000025646	7,88E-02	2,979864521	4,85E-01	4,86E-01	7,73E-01	Atrip	1,06	No
ENSMUSG00000025647	1,10E-01	7,966617018	3,56E+00	5,92E-02	3,01E-01	Shisa5	1,08	No
ENSMUSG00000025648	2,07E-01	5,081487018	8,06E+00	4,53E-03	5,18E-02	Pfkfb4	1,15	No
ENSMUSG00000025650	2,21E-01	0,233073196	6,30E-01	4,27E-01	7,21E-01	Col7a1	1,17	No
ENSMUSG00000025651	1,11E-01	6,930778143	3,33E+00	6,81E-02	3,28E-01	Uqcrc1	1,08	No
ENSMUSG00000025652	-1,73E+00	-2,448645098	3,32E+00	6,84E-02	3,29E-01	Tmem89	0,30	No
ENSMUSG00000025701	-2,36E-01	6,699376136	1,46E+01	1,34E-04	3,09E-03	Alox5	0,85	Yes
ENSMUSG00000025702	2,46E-01	6,833404695	1,61E+01	6,14E-05	1,62E-03	March8	1,19	Yes
ENSMUSG00000025716	1,81E+00	-3,10765737	1,28E+00	2,58E-01	5,50E-01	Myo3a	3,49	No
ENSMUSG00000025722	5,69E-02	3,567660109	3,42E-01	5,59E-01	8,22E-01	Wdr73	1,04	No
ENSMUSG00000025723	-1,03E+00	2,538654612	6,54E+01	6,03E-16	1,02E-13	Nmb	0,49	Yes
ENSMUSG00000025724	7,34E-03	6,347248065	1,33E-02	9,08E-01	1,00E+00	Sec11a	1,01	No
ENSMUSG00000025726	9,01E-03	-3,107646452	-5,16E-10	1,00E+00	1,00E+00	Slc28a1	1,01	No
ENSMUSG00000025727	-3,19E+00	-2,896088812	4,27E+00	3,87E-02	2,33E-01	A930017K11Rik	0,11	No
ENSMUSG00000025728	4,64E-02	6,106345637	5,13E-01	4,74E-01	7,62E-01	Pigq	1,03	No
ENSMUSG00000025730	-1,39E-02	4,480602152	2,96E-02	8,63E-01	9,97E-01	Rab40c	0,99	No
ENSMUSG00000025731	-7,04E-02	2,309726839	2,60E-01	6,10E-01	8,52E-01	Mettl26	0,95	No
ENSMUSG00000025732	1,11E-01	2,127684631	5,88E-01	4,43E-01	7,35E-01	Mcrip2	1,08	No

ENSMUSG00000025733	-1,54E-02	4,495060088	3,69E-02	8,48E-01	9,88E-01	Rhot2	0,99	No
ENSMUSG00000025735	9,11E-02	1,197008114	2,18E-01	6,40E-01	8,70E-01	Rhbdl1	1,07	No
ENSMUSG00000025736	6,47E-02	4,748817091	7,08E-01	4,00E-01	6,99E-01	Jmjd8	1,05	No
ENSMUSG00000025737	-1,26E-01	4,719763307	2,66E+00	1,03E-01	3,95E-01	Wdr24	0,92	No
ENSMUSG00000025738	-6,33E-01	-1,929298422	1,01E+00	3,15E-01	6,15E-01	Fbxl16	0,64	No
ENSMUSG00000025739	2,04E-01	-0,690315612	2,83E-01	5,95E-01	8,43E-01	Gng13	1,15	No
ENSMUSG00000025743	1,82E-01	9,342537036	1,03E+01	1,30E-03	2,00E-02	Sdc3	1,13	Yes
ENSMUSG00000025745	5,92E-02	6,789424341	9,27E-01	3,36E-01	6,37E-01	Hadha	1,04	No
ENSMUSG00000025746	-7,41E-01	1,016575134	1,23E+01	4,63E-04	8,61E-03	Il6	0,60	Yes
ENSMUSG00000025747	1,85E-01	4,521129591	5,33E+00	2,09E-02	1,56E-01	Tyms	1,14	No
ENSMUSG00000025757	4,62E-02	5,860429822	4,82E-01	4,87E-01	7,74E-01	Hspa4l	1,03	No
ENSMUSG00000025758	1,81E-01	4,620757415	5,29E+00	2,14E-02	1,58E-01	Plk4	1,13	No
ENSMUSG00000025759	1,11E-01	3,910417583	1,53E+00	2,17E-01	5,50E-01	Mfsd8	1,08	No
ENSMUSG00000025762	-1,86E-02	3,928680595	4,29E-02	8,36E-01	9,80E-01	Larp1b	0,99	No
ENSMUSG00000025764	8,75E-02	4,850895849	1,35E+00	2,45E-01	5,50E-01	Jade1	1,06	No
ENSMUSG00000025766	-1,91E-01	2,284404989	2,03E+00	1,54E-01	4,97E-01	D3Ertd751e	0,88	No
ENSMUSG00000025777	-4,19E-01	-2,711738795	1,78E-01	6,73E-01	8,90E-01	Gdap1	0,75	No
ENSMUSG00000025779	-1,09E-01	2,434098363	7,05E-01	4,01E-01	6,99E-01	Ly96	0,93	No
ENSMUSG00000025780	-9,97E-02	-2,355592991	1,13E-02	9,15E-01	1,00E+00	Itih5	0,93	No
ENSMUSG00000025781	2,60E-02	6,57136592	1,73E-01	6,78E-01	8,92E-01	Atp5c1	1,02	No
ENSMUSG00000025782	1,53E-01	4,006976204	2,92E+00	8,75E-02	3,78E-01	Taf3	1,11	No
ENSMUSG00000025783	1,17E+00	-3,14612376	6,40E-01	4,24E-01	7,18E-01	4930412O13Rik	2,26	No
ENSMUSG00000025784	-5,55E-02	-2,401368306	4,25E-03	9,48E-01	1,00E+00	Clec3b	0,96	No
ENSMUSG00000025785	-1,35E-01	3,584108514	2,01E+00	1,56E-01	5,00E-01	Exosc7	0,91	No
ENSMUSG00000025786	-1,23E-01	7,033039454	4,15E+00	4,17E-02	2,43E-01	Zdhhc3	0,92	No
ENSMUSG00000025787	-8,95E-02	2,587946692	5,06E-01	4,77E-01	7,65E-01	Tgm4	0,94	No
ENSMUSG00000025789	-1,31E+00	-2,831997589	1,07E+00	3,02E-01	5,98E-01	St8sia2	0,40	No
ENSMUSG00000025790	6,13E-03	5,566504208	8,01E-03	9,29E-01	1,00E+00	Slco3a1	1,00	No
ENSMUSG00000025791	1,53E-01	5,506751279	4,92E+00	2,65E-02	1,82E-01	Pgm2	1,11	No
ENSMUSG00000025792	-2,04E-01	5,347368919	8,42E+00	3,70E-03	4,47E-02	Slc25a10	0,87	Yes
ENSMUSG00000025793	3,34E-03	5,954795044	2,58E-03	9,60E-01	1,00E+00	Hgs	1,00	No
ENSMUSG00000025794	-1,21E-01	7,164201169	4,05E+00	4,43E-02	2,51E-01	Rpl14	0,92	No

ENSMUSG00000025795	-1,63E-01	6,633938049	6,95E+00	8,40E-03	8,22E-02	Rassf3	0,89	No
ENSMUSG00000025804	6,81E-02	8,537476819	1,40E+00	2,37E-01	5,50E-01	Ccr1	1,05	No
ENSMUSG00000025808	-6,79E-01	-0,395380527	4,03E+00	4,46E-02	2,51E-01	Ccdc7a	0,62	No
ENSMUSG00000025809	-1,89E-01	9,32205919	1,11E+01	8,59E-04	1,42E-02	Itgb1	0,88	Yes
ENSMUSG00000025810	1,91E-01	6,638353702	9,38E+00	2,19E-03	3,01E-02	Nrp1	1,14	Yes
ENSMUSG00000025812	1,32E-01	-2,146174575	3,04E-02	8,62E-01	9,96E-01	Pard3	1,10	No
ENSMUSG00000025813	7,24E-01	-2,770606383	2,86E-01	5,93E-01	8,42E-01	Homer2	1,65	No
ENSMUSG00000025815	6,02E-02	-1,033692114	1,86E-02	8,91E-01	1,00E+00	Dhtkd1	1,04	No
ENSMUSG00000025816	-1,61E-01	4,029072688	3,29E+00	6,95E-02	3,32E-01	Sec61a2	0,89	No
ENSMUSG00000025817	-2,01E-03	5,133488589	7,81E-04	9,78E-01	1,00E+00	Nudt5	1,00	No
ENSMUSG00000025821	3,14E-01	5,117380978	1,89E+01	1,40E-05	4,51E-04	Zfp282	1,24	Yes
ENSMUSG00000025823	1,89E-01	8,065723362	1,05E+01	1,19E-03	1,88E-02	Pdia4	1,14	Yes
ENSMUSG00000025825	-3,78E-01	5,378161517	2,93E+01	6,35E-08	3,69E-06	Iscu	0,77	Yes
ENSMUSG00000025854	-2,08E-02	7,850000237	1,27E-01	7,21E-01	9,16E-01	Fam20c	0,99	No
ENSMUSG00000025855	-1,02E-01	1,099680354	2,45E-01	6,21E-01	8,57E-01	Prkar1b	0,93	No
ENSMUSG00000025856	-1,22E-01	4,270441969	2,13E+00	1,44E-01	4,80E-01	Pdgfa	0,92	No
ENSMUSG00000025857	9,31E-02	4,042172767	1,10E+00	2,93E-01	5,89E-01	Dnaaf5	1,07	No
ENSMUSG00000025858	1,29E-01	5,164520031	3,17E+00	7,51E-02	3,49E-01	Get4	1,09	No
ENSMUSG00000025867	-2,66E+00	-3,033639428	2,81E+00	9,35E-02	3,83E-01	Cplx2	0,16	No
ENSMUSG00000025868	-5,90E-02	6,14613661	8,34E-01	3,61E-01	6,63E-01	Higd2a	0,96	No
ENSMUSG00000025869	-1,07E-01	5,490841094	2,42E+00	1,20E-01	4,31E-01	Nop16	0,93	No
ENSMUSG00000025870	1,52E-01	4,486741564	3,60E+00	5,78E-02	2,97E-01	Arl10	1,11	No
ENSMUSG00000025871	6,41E-02	6,601317314	1,06E+00	3,03E-01	6,00E-01	4833439L19Rik	1,05	No
ENSMUSG00000025872	4,65E-02	5,026147185	3,95E-01	5,30E-01	8,05E-01	Thoc3	1,03	No
ENSMUSG00000025873	2,73E-02	6,028629454	1,75E-01	6,76E-01	8,91E-01	Faf2	1,02	No
ENSMUSG00000025875	1,30E-01	3,995091369	2,12E+00	1,46E-01	4,83E-01	Tspan17	1,09	No
ENSMUSG00000025876	2,60E-02	-0,1216012	6,52E-03	9,36E-01	1,00E+00	Unc5a	1,02	No
ENSMUSG00000025877	1,46E-01	7,171590142	5,90E+00	1,51E-02	1,27E-01	Hk3	1,11	No
ENSMUSG00000025878	-1,05E-01	6,121568139	2,64E+00	1,04E-01	3,97E-01	Uimc1	0,93	No
ENSMUSG00000025880	1,69E-01	3,312388243	2,58E+00	1,08E-01	4,02E-01	Smad7	1,12	No
ENSMUSG00000025885	-9,34E-01	-0,195464089	9,50E+00	2,06E-03	2,86E-02	Myo5b	0,52	Yes
ENSMUSG00000025887	-8,60E-01	-1,472785131	2,93E+00	8,72E-02	3,78E-01	Casp12	0,55	No

ENSMUSG00000025888	-1,99E-02	5,628961091	8,55E-02	7,70E-01	9,42E-01	Casp1	0,99	No
ENSMUSG00000025892	1,87E+00	-3,107639691	1,38E+00	2,40E-01	5,50E-01	Gria4	3,67	No
ENSMUSG00000025893	-1,93E-01	2,327118412	2,01E+00	1,56E-01	5,00E-01	Kbtbd3	0,88	No
ENSMUSG00000025894	-3,40E-02	3,866585711	1,40E-01	7,09E-01	9,09E-01	Aasdhppt	0,98	No
ENSMUSG00000025898	-6,43E-02	4,100878164	5,56E-01	4,56E-01	7,45E-01	Cwf19l2	0,96	No
ENSMUSG00000025899	2,62E-02	3,64909982	7,36E-02	7,86E-01	9,52E-01	Alkbh8	1,02	No
ENSMUSG00000025902	-1,89E+00	-3,1076441	1,42E+00	2,33E-01	5,50E-01	Sox17	0,27	No
ENSMUSG00000025903	6,33E-02	5,938751958	9,27E-01	3,36E-01	6,37E-01	Lypla1	1,04	No
ENSMUSG00000025907	-1,56E-02	5,578810584	5,25E-02	8,19E-01	9,71E-01	Rb1cc1	0,99	No
ENSMUSG00000025909	3,11E+00	-2,963280805	3,94E+00	4,72E-02	2,60E-01	Sntg1	8,63	No
ENSMUSG00000025911	1,23E-01	0,7529299	3,04E-01	5,81E-01	8,37E-01	Adhfe1	1,09	No
ENSMUSG00000025912	4,60E-01	2,435087642	1,26E+01	3,81E-04	7,35E-03	Mybl1	1,38	Yes
ENSMUSG00000025915	1,95E-01	5,809584812	8,54E+00	3,47E-03	4,27E-02	Sgk3	1,14	Yes
ENSMUSG00000025916	6,24E-01	-2,74087404	3,21E-01	5,71E-01	8,32E-01	Ppp1r42	1,54	No
ENSMUSG00000025917	-4,09E-02	5,822369819	3,76E-01	5,40E-01	8,10E-01	Cops5	0,97	No
ENSMUSG00000025920	-3,46E-01	2,762435031	8,66E+00	3,25E-03	4,07E-02	Stau2	0,79	Yes
ENSMUSG00000025921	3,21E-02	2,444901128	5,87E-02	8,09E-01	9,65E-01	Rdh10	1,02	No
ENSMUSG00000025922	-5,10E-01	4,652930637	4,27E+01	6,52E-11	6,35E-09	Gm5045	0,70	Yes
ENSMUSG00000025925	-1,03E-01	3,254964761	9,52E-01	3,29E-01	6,30E-01	Terf1	0,93	No
ENSMUSG00000025927	-1,87E+00	-3,03364178	1,41E+00	2,36E-01	5,50E-01	Tfap2b	0,27	No
ENSMUSG00000025930	-9,89E-01	-2,44871126	1,51E+00	2,20E-01	5,50E-01	Msc	0,50	No
ENSMUSG00000025931	-1,50E-01	2,673939016	1,57E+00	2,10E-01	5,50E-01	Paqr8	0,90	No
ENSMUSG00000025932	-2,80E-01	0,929629372	1,76E+00	1,85E-01	5,49E-01	Eya1	0,82	No
ENSMUSG00000025933	-2,89E-01	0,794242266	1,72E+00	1,90E-01	5,50E-01	Tmem14a	0,82	No
ENSMUSG00000025934	-8,12E-02	-0,042796778	7,15E-02	7,89E-01	9,54E-01	Gsta3	0,95	No
ENSMUSG00000025935	1,72E-01	6,808042848	7,80E+00	5,21E-03	5,76E-02	Tram1	1,13	No
ENSMUSG00000025936	-3,04E-01	0,87814419	2,32E+00	1,27E-01	4,47E-01	Gm4956	0,81	No
ENSMUSG00000025937	1,18E-01	4,294317709	2,01E+00	1,56E-01	5,01E-01	Lactb2	1,09	No
ENSMUSG00000025938	-8,90E-01	4,603354293	1,27E+02	2,05E-29	1,06E-26	Slco5a1	0,54	Yes
ENSMUSG00000025939	-6,78E-02	5,004982927	8,46E-01	3,58E-01	6,59E-01	Ube2w	0,95	No
ENSMUSG00000025940	-2,16E-01	5,310929786	9,49E+00	2,07E-03	2,88E-02	Tmem70	0,86	Yes
ENSMUSG00000025946	4,81E-01	-2,832017219	1,83E-01	6,69E-01	8,87E-01	Pth2r	1,40	No

ENSMUSG00000025949	3,20E-02	6,85959201	2,74E-01	6,00E-01	8,47E-01	Pikfyve	1,02	No
ENSMUSG00000025950	-5,23E-02	7,685747509	7,91E-01	3,74E-01	6,75E-01	Idh1	0,96	No
ENSMUSG00000025955	-2,25E-01	0,43253918	7,77E-01	3,78E-01	6,79E-01	Akr1cl	0,86	No
ENSMUSG00000025956	-3,33E-02	4,214090162	1,57E-01	6,92E-01	9,00E-01	Mettl21a	0,98	No
ENSMUSG00000025958	4,18E-02	6,239497397	4,28E-01	5,13E-01	7,95E-01	Creb1	1,03	No
ENSMUSG00000025959	-1,48E-01	6,182905349	5,12E+00	2,36E-02	1,69E-01	Klf7	0,90	No
ENSMUSG00000025961	-6,07E-02	-2,770596883	2,49E-03	9,60E-01	1,00E+00	4933402D24Rik	0,96	No
ENSMUSG00000025962	1,09E-01	3,95497563	1,48E+00	2,23E-01	5,50E-01	Fastkd2	1,08	No
ENSMUSG00000025963	1,85E+00	-3,107657832	1,35E+00	2,46E-01	5,50E-01	Mdh1b	3,61	No
ENSMUSG00000025964	-3,46E-01	7,298919475	3,36E+01	6,85E-09	4,91E-07	Adam23	0,79	Yes
ENSMUSG00000025967	-1,77E-01	7,952049073	9,25E+00	2,36E-03	3,20E-02	Eef1b2	0,88	Yes
ENSMUSG00000025968	1,34E-01	6,066671184	4,23E+00	3,97E-02	2,36E-01	Ndufs1	1,10	No
ENSMUSG00000025969	1,57E-01	9,086534828	7,61E+00	5,82E-03	6,26E-02	Nrp2	1,11	No
ENSMUSG00000025971	-5,50E-02	3,807180294	3,48E-01	5,55E-01	8,20E-01	Maip1	0,96	No
ENSMUSG00000025977	1,55E+00	-2,185621666	3,62E+00	5,72E-02	2,94E-01	Boll	2,94	No
ENSMUSG00000025978	-1,38E-02	-0,268121865	2,01E-03	9,64E-01	1,00E+00	Rftn2	0,99	No
ENSMUSG00000025979	8,35E-02	4,823735678	1,20E+00	2,73E-01	5,70E-01	Mob4	1,06	No
ENSMUSG00000025980	1,12E-01	6,272581013	3,06E+00	8,03E-02	3,63E-01	Hspd1	1,08	No
ENSMUSG00000025981	-8,65E-02	4,51805592	1,16E+00	2,82E-01	5,78E-01	Coq10b	0,94	No
ENSMUSG00000025982	-1,25E-01	8,680055491	4,75E+00	2,92E-02	1,95E-01	Sf3b1	0,92	No
ENSMUSG00000025983	-6,12E-02	-2,033623443	6,95E-03	9,34E-01	1,00E+00	Ccdc150	0,96	No
ENSMUSG00000025986	3,27E-01	5,199738161	2,05E+01	6,01E-06	2,13E-04	Slc39a10	1,25	Yes
ENSMUSG00000025993	2,76E-01	3,973390378	9,56E+00	1,99E-03	2,79E-02	Slc40a1	1,21	Yes
ENSMUSG00000025995	1,17E-01	5,145501574	2,58E+00	1,08E-01	4,04E-01	Wdr75	1,08	No
ENSMUSG00000025997	1,63E-01	3,873350183	3,29E+00	6,98E-02	3,33E-01	Ikzf2	1,12	No
ENSMUSG00000026000	-3,51E-01	3,866698859	1,50E+01	1,05E-04	2,53E-03	Lancl1	0,78	Yes
ENSMUSG00000026003	-4,40E-02	6,221129422	4,72E-01	4,92E-01	7,78E-01	Acadl	0,97	No
ENSMUSG00000026004	1,04E-01	2,980936369	8,43E-01	3,59E-01	6,60E-01	Kansl1l	1,07	No
ENSMUSG00000026005	4,56E-02	5,661332574	4,53E-01	5,01E-01	7,86E-01	Rpe	1,03	No
ENSMUSG00000026009	-1,09E+00	2,8732384	8,91E+01	3,75E-21	9,37E-19	Icos	0,47	Yes
ENSMUSG00000026011	1,26E+00	-2,896169772	9,87E-01	3,21E-01	6,21E-01	Ctla4	2,39	No
ENSMUSG00000026012	-3,74E-01	4,081223052	1,75E+01	2,92E-05	8,56E-04	Cd28	0,77	Yes

ENSMUSG00000026014	3,56E-01	6,060188069	2,97E+01	5,05E-08	3,01E-06	Raph1	1,28	Yes
ENSMUSG00000026017	1,32E-01	3,032061701	1,37E+00	2,41E-01	5,50E-01	Carf	1,10	No
ENSMUSG00000026018	4,38E-02	2,987614723	1,49E-01	7,00E-01	9,04E-01	Ica1l	1,03	No
ENSMUSG00000026019	8,15E-02	3,311134944	6,28E-01	4,28E-01	7,22E-01	Wdr12	1,06	No
ENSMUSG00000026020	-4,22E-02	5,346949363	3,57E-01	5,50E-01	8,17E-01	Nop58	0,97	No
ENSMUSG00000026021	-1,77E-01	5,733767553	6,93E+00	8,50E-03	8,28E-02	Sumo1	0,88	No
ENSMUSG00000026023	-1,89E-01	-1,683085568	9,62E-02	7,56E-01	9,35E-01	Cdk15	0,88	No
ENSMUSG00000026024	1,66E-01	5,611111198	5,97E+00	1,45E-02	1,23E-01	Als2	1,12	No
ENSMUSG00000026027	9,07E-02	4,001273097	1,04E+00	3,07E-01	6,04E-01	Stradb	1,06	No
ENSMUSG00000026028	3,81E-03	6,202414261	3,52E-03	9,53E-01	1,00E+00	Trak2	1,00	No
ENSMUSG00000026029	3,61E-02	6,717797944	3,41E-01	5,59E-01	8,22E-01	Casp8	1,03	No
ENSMUSG00000026031	-4,97E-02	6,907435849	6,69E-01	4,14E-01	7,09E-01	Cflar	0,97	No
ENSMUSG00000026032	3,51E-02	4,218825427	1,71E-01	6,80E-01	8,93E-01	Ndufb3	1,02	No
ENSMUSG00000026034	-1,45E-01	6,22161222	5,13E+00	2,35E-02	1,68E-01	Clk1	0,90	No
ENSMUSG00000026035	1,17E-01	3,284196232	1,26E+00	2,61E-01	5,55E-01	Ppil3	1,08	No
ENSMUSG00000026036	4,93E-02	4,335400529	3,52E-01	5,53E-01	8,19E-01	Nif3l1	1,03	No
ENSMUSG00000026037	1,51E-01	4,522064277	3,56E+00	5,91E-02	3,01E-01	Orc2	1,11	No
ENSMUSG00000026039	4,13E-01	3,381321102	1,64E+01	5,01E-05	1,36E-03	Sgo2a	1,33	Yes
ENSMUSG00000026042	-3,36E+00	-1,655023646	2,15E+01	3,54E-06	1,35E-04	Col5a2	0,10	Yes
ENSMUSG00000026043	-1,42E+00	-1,800908471	5,11E+00	2,38E-02	1,69E-01	Col3a1	0,37	No
ENSMUSG00000026047	1,16E-01	3,93257439	1,68E+00	1,95E-01	5,50E-01	Kdelc1	1,08	No
ENSMUSG00000026048	-1,25E-02	4,681696002	2,59E-02	8,72E-01	1,00E+00	Ercc5	0,99	No
ENSMUSG00000026049	6,32E-02	3,146558696	3,37E-01	5,61E-01	8,24E-01	Tex30	1,04	No
ENSMUSG00000026051	-4,79E-01	-1,510070135	7,05E-01	4,01E-01	6,99E-01	1500015O10Rik	0,72	No
ENSMUSG00000026062	-4,72E-01	0,911367873	4,47E+00	3,45E-02	2,19E-01	Slc9a2	0,72	No
ENSMUSG00000026064	1,63E-01	5,852941815	5,99E+00	1,44E-02	1,22E-01	Ptp4a1	1,12	No
ENSMUSG00000026065	1,09E-02	2,409494828	6,62E-03	9,35E-01	1,00E+00	Slc9a4	1,01	No
ENSMUSG00000026068	3,78E-01	4,904928171	2,55E+01	4,52E-07	2,18E-05	Il18rap	1,30	Yes
ENSMUSG00000026069	4,37E-01	5,235223651	3,69E+01	1,24E-09	9,95E-08	Il1rl1	1,35	Yes
ENSMUSG00000026070	8,15E-02	4,117076908	9,25E-01	3,36E-01	6,37E-01	Il18r1	1,06	No
ENSMUSG00000026072	4,59E-01	3,150243995	1,79E+01	2,27E-05	6,95E-04	Il1r1	1,37	Yes
ENSMUSG00000026073	-5,68E-01	6,641005555	8,06E+01	2,72E-19	5,91E-17	Il1r2	0,67	Yes

ENSMUSG00000026074	-4,34E-01	7,67246348	5,43E+01	1,70E-13	2,27E-11	Map4k4	0,74	Yes
ENSMUSG00000026077	-9,27E-01	0,496246473	1,36E+01	2,21E-04	4,72E-03	Npas2	0,53	Yes
ENSMUSG00000026078	-1,64E-03	5,287838102	5,36E-04	9,82E-01	1,00E+00	Pdcl3	1,00	No
ENSMUSG00000026080	5,12E-03	1,633603441	8,59E-04	9,77E-01	1,00E+00	Chst10	1,00	No
ENSMUSG00000026082	1,05E-01	4,584686218	1,74E+00	1,87E-01	5,50E-01	Rev1	1,08	No
ENSMUSG00000026083	3,82E-02	6,980201037	3,93E-01	5,31E-01	8,05E-01	Eif5b	1,03	No
ENSMUSG00000026087	-1,20E-01	3,84085513	1,73E+00	1,88E-01	5,50E-01	Mrpl30	0,92	No
ENSMUSG00000026088	3,17E-01	3,162355065	8,81E+00	3,00E-03	3,83E-02	Mitd1	1,25	Yes
ENSMUSG00000026090	-2,69E-01	0,191129937	1,06E+00	3,02E-01	5,99E-01	2010300C02Rik	0,83	No
ENSMUSG00000026094	-6,74E-03	7,574973416	1,30E-02	9,09E-01	1,00E+00	Stk17b	1,00	No
ENSMUSG00000026095	-6,28E-02	5,797937095	8,85E-01	3,47E-01	6,48E-01	Asnsd1	0,96	No
ENSMUSG00000026096	-2,57E-02	2,524950169	4,03E-02	8,41E-01	9,84E-01	Osgepl1	0,98	No
ENSMUSG00000026097	1,18E-01	4,603125793	2,24E+00	1,34E-01	4,61E-01	Ormdl1	1,09	No
ENSMUSG00000026098	9,28E-02	2,274124734	4,75E-01	4,91E-01	7,77E-01	Pms1	1,07	No
ENSMUSG00000026102	2,58E-02	3,016748102	5,28E-02	8,18E-01	9,70E-01	Inpp1	1,02	No
ENSMUSG00000026103	-1,93E-02	7,522408	1,06E-01	7,44E-01	9,29E-01	Gls	0,99	No
ENSMUSG00000026104	-8,38E-02	6,285393726	1,75E+00	1,86E-01	5,50E-01	Stat1	0,94	No
ENSMUSG00000026107	-2,94E-01	8,789589607	2,65E+01	2,67E-07	1,35E-05	Nabp1	0,82	Yes
ENSMUSG00000026109	-1,09E-02	-2,896111597	8,93E-05	9,92E-01	1,00E+00	Tmeff2	0,99	No
ENSMUSG00000026110	-1,11E-01	6,85568852	3,28E+00	7,03E-02	3,35E-01	Mgat4a	0,93	No
ENSMUSG00000026111	-9,09E-02	5,136192942	1,57E+00	2,10E-01	5,50E-01	Unc50	0,94	No
ENSMUSG00000026112	-6,96E-02	7,85817966	1,42E+00	2,33E-01	5,50E-01	Coa5	0,95	No
ENSMUSG00000026113	2,15E-01	5,852923123	1,05E+01	1,22E-03	1,91E-02	Inpp4a	1,16	Yes
ENSMUSG00000026114	-1,83E+00	-2,14603272	5,28E+00	2,15E-02	1,58E-01	Cnga3	0,28	No
ENSMUSG00000026116	-2,89E-01	7,615023063	2,40E+01	9,58E-07	4,27E-05	Tmem131	0,82	Yes
ENSMUSG00000026117	4,34E-01	-2,711749443	1,47E-01	7,01E-01	9,05E-01	Zap70	1,35	No
ENSMUSG00000026121	-5,46E-01	2,719806863	2,03E+01	6,74E-06	2,35E-04	Sema4c	0,69	Yes
ENSMUSG00000026123	-5,32E-02	8,021926948	8,38E-01	3,60E-01	6,61E-01	Plekhb2	0,96	No
ENSMUSG00000026126	-5,99E-02	3,996308918	4,49E-01	5,03E-01	7,88E-01	Ptpn18	0,96	No
ENSMUSG00000026127	3,73E-02	6,021155994	3,26E-01	5,68E-01	8,30E-01	Imp4	1,03	No
ENSMUSG00000026131	2,83E-01	7,374100658	2,24E+01	2,20E-06	8,82E-05	Dst	1,22	Yes
ENSMUSG00000026134	2,36E-02	4,087207445	7,22E-02	7,88E-01	9,53E-01	Prim2	1,02	No

ENSMUSG00000026135	1,52E-01	4,938261216	4,18E+00	4,09E-02	2,40E-01	Zfp142	1,11	No
ENSMUSG00000026141	3,14E-02	-3,107617333	-7,69E-10	1,00E+00	1,00E+00	Col19a1	1,02	No
ENSMUSG00000026142	9,23E-02	5,370827101	1,72E+00	1,89E-01	5,50E-01	Rhbdd1	1,07	No
ENSMUSG00000026147	-2,71E+00	-2,896102977	2,90E+00	8,85E-02	3,79E-01	Col9a1	0,15	No
ENSMUSG00000026149	7,39E-01	-2,770612239	2,97E-01	5,86E-01	8,39E-01	Tm4sf20	1,67	No
ENSMUSG00000026150	-9,31E-02	6,317617862	2,14E+00	1,44E-01	4,79E-01	Mff	0,94	No
ENSMUSG00000026153	2,04E-01	2,051294612	1,83E+00	1,76E-01	5,36E-01	Fam135a	1,15	No
ENSMUSG00000026154	-4,04E-02	2,712839933	1,08E-01	7,42E-01	9,27E-01	Sdhaf4	0,97	No
ENSMUSG00000026155	5,09E-02	5,119085467	4,91E-01	4,83E-01	7,71E-01	Smap1	1,04	No
ENSMUSG00000026156	2,32E-02	4,382581292	7,94E-02	7,78E-01	9,47E-01	B3gat2	1,02	No
ENSMUSG00000026158	-4,55E-01	6,501384343	5,30E+01	3,34E-13	4,37E-11	Ogfrl1	0,73	Yes
ENSMUSG00000026159	-4,60E-02	6,710171408	5,54E-01	4,57E-01	7,46E-01	Agfg1	0,97	No
ENSMUSG00000026162	1,87E-02	2,011075967	1,55E-02	9,01E-01	1,00E+00	Nhej1	1,01	No
ENSMUSG00000026166	-2,70E+00	-3,033614577	2,89E+00	8,93E-02	3,79E-01	Ccl20	0,15	No
ENSMUSG00000026167	-9,70E-01	-2,655145946	8,25E-01	3,64E-01	6,65E-01	Wnt10a	0,51	No
ENSMUSG00000026170	1,84E-01	4,696625415	5,54E+00	1,85E-02	1,44E-01	Cyp27a1	1,14	No
ENSMUSG00000026171	2,05E-02	3,8750035	5,07E-02	8,22E-01	9,72E-01	Rnf25	1,01	No
ENSMUSG00000026172	1,06E-02	3,116504779	9,38E-03	9,23E-01	1,00E+00	Bcs1l	1,01	No
ENSMUSG00000026173	3,11E-01	1,413844391	2,99E+00	8,37E-02	3,71E-01	Plcd4	1,24	No
ENSMUSG00000026174	1,44E-01	6,254113957	5,07E+00	2,43E-02	1,71E-01	Cnot9	1,11	No
ENSMUSG00000026175	3,57E-01	-1,711698178	3,23E-01	5,70E-01	8,31E-01	Vil1	1,28	No
ENSMUSG00000026176	-1,70E-02	6,218679293	7,01E-02	7,91E-01	9,55E-01	Ctdsp1	0,99	No
ENSMUSG00000026177	7,48E-02	5,51332427	1,16E+00	2,80E-01	5,77E-01	Slc11a1	1,05	No
ENSMUSG00000026179	-5,08E-01	5,494459331	5,54E+01	9,99E-14	1,37E-11	Pnkd	0,70	Yes
ENSMUSG00000026180	-2,10E-01	2,385408185	2,51E+00	1,13E-01	4,15E-01	Cxcr2	0,86	No
ENSMUSG00000026181	-1,08E-01	5,270979021	2,31E+00	1,28E-01	4,50E-01	Ppm1f	0,93	No
ENSMUSG00000026185	-1,89E+00	-2,401225495	4,16E+00	4,13E-02	2,41E-01	Igfbp5	0,27	No
ENSMUSG00000026187	4,49E-02	-0,126619774	2,22E-02	8,81E-01	1,00E+00	Xrcc5	1,03	No
ENSMUSG00000026188	-2,11E-01	-1,963268022	1,09E-01	7,42E-01	9,27E-01	Tmem169	0,86	No
ENSMUSG00000026189	-1,52E-01	0,742282046	4,82E-01	4,88E-01	7,75E-01	Pecr	0,90	No
ENSMUSG00000026192	1,24E-01	6,535965291	3,91E+00	4,79E-02	2,63E-01	Atic	1,09	No
ENSMUSG00000026193	2,76E-01	9,803172564	2,40E+01	9,78E-07	4,35E-05	Fn1	1,21	Yes

ENSMUSG00000026196	4,56E-01	3,698618481	2,25E+01	2,07E-06	8,38E-05	Bard1	1,37	Yes
ENSMUSG00000026197	-1,81E-02	3,391156786	3,18E-02	8,58E-01	9,94E-01	Zfand2b	0,99	No
ENSMUSG00000026198	3,42E-01	1,822656009	4,53E+00	3,32E-02	2,14E-01	Abcb6	1,27	No
ENSMUSG00000026199	6,19E-02	4,641809542	6,27E-01	4,29E-01	7,22E-01	Ankzf1	1,04	No
ENSMUSG00000026200	1,33E-01	3,853967543	2,11E+00	1,47E-01	4,85E-01	Glb1l	1,10	No
ENSMUSG00000026201	9,67E-02	5,099444178	1,75E+00	1,86E-01	5,50E-01	Stk16	1,07	No
ENSMUSG00000026202	2,05E-01	6,103887182	9,91E+00	1,64E-03	2,39E-02	Tuba4a	1,15	Yes
ENSMUSG00000026203	-2,69E-01	3,230338764	6,62E+00	1,01E-02	9,39E-02	Dnajb2	0,83	No
ENSMUSG00000026204	2,45E-01	-0,567753184	4,58E-01	4,99E-01	7,84E-01	Ptprn	1,19	No
ENSMUSG00000026205	1,78E+00	-1,683309727	7,98E+00	4,73E-03	5,36E-02	Slc23a3	3,44	No
ENSMUSG00000026207	3,10E-01	5,922152086	2,19E+01	2,94E-06	1,14E-04	Speg	1,24	Yes
ENSMUSG00000026208	1,87E+00	-3,107639691	1,38E+00	2,40E-01	5,50E-01	Des	3,67	No
ENSMUSG00000026209	1,66E-03	4,808356647	4,74E-04	9,83E-01	1,00E+00	Dnpep	1,00	No
ENSMUSG00000026211	-3,00E-01	-1,397700658	3,11E-01	5,77E-01	8,35E-01	Obsl1	0,81	No
ENSMUSG00000026213	1,43E-01	6,373384824	5,05E+00	2,46E-02	1,73E-01	Stk11ip	1,10	No
ENSMUSG00000026219	-1,11E-01	8,637325087	3,76E+00	5,25E-02	2,79E-01	Trip12	0,93	No
ENSMUSG00000026220	3,23E-01	2,867875718	7,51E+00	6,15E-03	6,54E-02	Slc16a14	1,25	No
ENSMUSG00000026222	-8,09E-02	6,19770179	1,60E+00	2,06E-01	5,50E-01	Sp100	0,95	No
ENSMUSG00000026223	-2,56E-01	7,781562164	1,91E+01	1,25E-05	4,09E-04	Itm2c	0,84	Yes
ENSMUSG00000026224	-3,94E-01	0,295612316	2,26E+00	1,33E-01	4,58E-01	4933407L21Rik	0,76	No
ENSMUSG00000026226	-2,81E-02	-2,963245656	3,59E-04	9,85E-01	1,00E+00	Spata3	0,98	No
ENSMUSG00000026227	-6,01E-01	-2,770562995	2,85E-01	5,94E-01	8,43E-01	2810459M11Rik	0,66	No
ENSMUSG00000026228	-5,39E-01	-0,047107121	3,01E+00	8,29E-02	3,70E-01	Htr2b	0,69	No
ENSMUSG00000026229	-7,02E-05	6,755765328	1,30E-06	9,99E-01	1,00E+00	Psmd1	1,00	No
ENSMUSG00000026234	2,42E-02	8,212150805	1,75E-01	6,75E-01	8,91E-01	Ncl	1,02	No
ENSMUSG00000026235	4,77E-02	1,281309522	6,02E-02	8,06E-01	9,64E-01	Epha4	1,03	No
ENSMUSG00000026237	-2,69E+00	-2,963260181	2,87E+00	9,01E-02	3,80E-01	Nmur1	0,16	No
ENSMUSG00000026238	1,78E-02	8,558637812	9,63E-02	7,56E-01	9,35E-01	Ptma	1,01	No
ENSMUSG00000026239	-1,41E-01	4,314631614	2,89E+00	8,92E-02	3,79E-01	Pde6d	0,91	No
ENSMUSG00000026240	-6,91E-03	4,204871865	6,61E-03	9,35E-01	1,00E+00	Cops7b	1,00	No
ENSMUSG00000026241	1,96E-01	-2,107632884	5,71E-02	8,11E-01	9,66E-01	Nppc	1,15	No
ENSMUSG00000026245	8,11E-02	5,042062546	1,21E+00	2,72E-01	5,68E-01	Farsb	1,06	No

ENSMUSG00000026247	1,81E+00	-3,10765737	1,28E+00	2,58E-01	5,50E-01	Ecel1	3,49	No
ENSMUSG00000026248	-8,88E-02	4,456706121	1,20E+00	2,74E-01	5,70E-01	Mrpl44	0,94	No
ENSMUSG00000026249	-3,55E-01	0,633069011	2,64E+00	1,04E-01	3,96E-01	Serpine2	0,78	No
ENSMUSG00000026253	1,81E+00	-3,10765737	1,28E+00	2,58E-01	5,50E-01	Chrng	3,49	No
ENSMUSG00000026254	-6,66E-02	6,160395389	1,07E+00	3,01E-01	5,97E-01	Eif4e2	0,95	No
ENSMUSG00000026255	-7,38E-02	-3,033659033	2,66E-03	9,59E-01	1,00E+00	Efhd1	0,95	No
ENSMUSG00000026259	-1,94E+00	-3,107619243	1,50E+00	2,21E-01	5,50E-01	Ngef	0,26	No
ENSMUSG00000026260	-4,13E-02	5,924416259	3,91E-01	5,32E-01	8,06E-01	Ndufa10	0,97	No
ENSMUSG00000026269	1,59E-01	6,859744673	6,72E+00	9,52E-03	8,99E-02	Rnpepl1	1,12	No
ENSMUSG00000026270	2,31E-01	3,835172131	6,39E+00	1,15E-02	1,03E-01	Capn10	1,17	No
ENSMUSG00000026271	5,19E-01	5,124784482	5,10E+01	9,09E-13	1,11E-10	Gpr35	1,43	Yes
ENSMUSG00000026273	-2,47E-01	3,810676389	7,29E+00	6,94E-03	7,13E-02	Mterf4	0,84	No
ENSMUSG00000026274	2,02E-01	3,632887131	4,44E+00	3,52E-02	2,22E-01	Pask	1,15	No
ENSMUSG00000026275	1,97E-01	5,319158971	7,72E+00	5,46E-03	5,96E-02	Ppp1r7	1,15	No
ENSMUSG00000026276	-2,60E-03	7,142085625	1,87E-03	9,66E-01	1,00E+00	37500	1,00	No
ENSMUSG00000026277	-4,50E-02	6,039349549	4,77E-01	4,90E-01	7,76E-01	Stk25	0,97	No
ENSMUSG00000026278	1,68E-01	-0,257375487	2,78E-01	5,98E-01	8,45E-01	Bok	1,12	No
ENSMUSG00000026279	-1,54E-01	4,357407567	3,57E+00	5,89E-02	3,00E-01	Thap4	0,90	No
ENSMUSG00000026280	-2,11E-02	5,159685348	8,56E-02	7,70E-01	9,42E-01	Atg4b	0,99	No
ENSMUSG00000026281	6,60E-02	4,889138511	7,74E-01	3,79E-01	6,80E-01	Dtymk	1,05	No
ENSMUSG00000026283	1,73E-01	4,571814627	4,72E+00	2,98E-02	1,98E-01	Ing5	1,13	No
ENSMUSG00000026285	-4,26E-02	0,036592227	2,59E-02	8,72E-01	1,00E+00	Pdcd1	0,97	No
ENSMUSG00000026288	1,68E-01	7,894868965	8,22E+00	4,15E-03	4,86E-02	Inpp5d	1,12	Yes
ENSMUSG00000026289	-3,32E-02	6,049895649	2,58E-01	6,11E-01	8,52E-01	Atg16l1	0,98	No
ENSMUSG00000026295	1,87E+00	-3,107639691	1,38E+00	2,40E-01	5,50E-01	Spp2	3,67	No
ENSMUSG00000026301	-3,19E+00	-2,770626535	4,29E+00	3,84E-02	2,33E-01	Iqca	0,11	No
ENSMUSG00000026303	-1,29E-01	-0,088986607	1,63E-01	6,86E-01	8,97E-01	Mlph	0,91	No
ENSMUSG00000026304	1,02E-01	-1,929390042	2,05E-02	8,86E-01	1,00E+00	Rab17	1,07	No
ENSMUSG00000026305	4,81E-02	7,75250945	6,71E-01	4,13E-01	7,08E-01	Lrrfip1	1,03	No
ENSMUSG00000026307	7,10E-02	5,371577407	1,02E+00	3,13E-01	6,12E-01	Scly	1,05	No
ENSMUSG00000026308	1,59E+00	-2,711739009	1,74E+00	1,88E-01	5,50E-01	Klhl30	3,02	No
ENSMUSG00000026309	-1,01E-01	5,327153571	2,07E+00	1,50E-01	4,92E-01	Ilkap	0,93	No

ENSMUSG00000026311	1,09E-01	4,319476813	1,72E+00	1,89E-01	5,50E-01	Asb1	1,08	No
ENSMUSG00000026313	2,04E-01	5,455889904	8,47E+00	3,62E-03	4,40E-02	Hdac4	1,15	Yes
ENSMUSG00000026315	-1,87E-01	4,764506513	5,79E+00	1,61E-02	1,32E-01	Serpinb8	0,88	No
ENSMUSG00000026317	1,82E-01	6,136186949	7,90E+00	4,93E-03	5,54E-02	Cln8	1,13	No
ENSMUSG00000026319	5,27E-02	6,011776408	6,47E-01	4,21E-01	7,16E-01	2310035C23Rik	1,04	No
ENSMUSG00000026321	-4,18E-01	6,072822629	4,16E+01	1,10E-10	1,04E-08	Tnfrsf11a	0,75	Yes
ENSMUSG00000026322	-1,87E+00	-3,107637761	1,39E+00	2,38E-01	5,50E-01	Htr4	0,27	No
ENSMUSG00000026333	-6,53E-02	2,854849688	3,13E-01	5,76E-01	8,34E-01	Gin1	0,96	No
ENSMUSG00000026335	-4,31E-02	3,369168226	1,80E-01	6,71E-01	8,89E-01	Pam	0,97	No
ENSMUSG00000026339	6,47E-02	5,797888311	9,41E-01	3,32E-01	6,32E-01	Ccdc93	1,05	No
ENSMUSG00000026341	5,61E-03	8,753070659	9,64E-03	9,22E-01	1,00E+00	Actr3	1,00	No
ENSMUSG00000026342	2,04E-01	4,546342443	6,47E+00	1,10E-02	9,98E-02	Slc35f5	1,15	No
ENSMUSG00000026343	-2,77E+00	-2,548117539	7,25E+00	7,09E-03	7,25E-02	Gpr39	0,15	No
ENSMUSG00000026344	-2,95E+00	-2,963237934	3,55E+00	5,97E-02	3,02E-01	Lypd1	0,13	No
ENSMUSG00000026347	4,49E-01	-0,029071183	2,16E+00	1,42E-01	4,76E-01	Tmem163	1,36	No
ENSMUSG00000026348	9,95E-01	-3,033653241	4,20E-01	5,17E-01	7,98E-01	Acmsd	1,99	No
ENSMUSG00000026349	7,58E-02	6,159166302	1,40E+00	2,38E-01	5,50E-01	Ccnt2	1,05	No
ENSMUSG00000026353	5,50E-02	6,586636534	7,76E-01	3,78E-01	6,79E-01	Ubxn4	1,04	No
ENSMUSG00000026354	-7,28E-01	-1,574151831	1,69E+00	1,93E-01	5,50E-01	Lct	0,60	No
ENSMUSG00000026355	7,89E-02	6,796600162	1,65E+00	1,99E-01	5,50E-01	Mcm6	1,06	No
ENSMUSG00000026356	1,59E-01	5,105428075	4,76E+00	2,91E-02	1,94E-01	Dars	1,12	No
ENSMUSG00000026357	2,81E-01	3,577823672	8,25E+00	4,08E-03	4,81E-02	Rgs18	1,21	Yes
ENSMUSG00000026358	3,36E-01	8,281845636	3,32E+01	8,53E-09	6,00E-07	Rgs1	1,26	Yes
ENSMUSG00000026360	4,22E-01	6,842901645	4,71E+01	6,82E-12	7,41E-10	Rgs2	1,34	Yes
ENSMUSG00000026361	-8,70E-03	5,428894158	1,55E-02	9,01E-01	1,00E+00	Cdc73	0,99	No
ENSMUSG00000026365	-7,17E-01	0,587044671	8,67E+00	3,24E-03	4,06E-02	Cfh	0,61	Yes
ENSMUSG00000026374	-1,78E-02	6,55133218	8,16E-02	7,75E-01	9,45E-01	Tsn	0,99	No
ENSMUSG00000026377	1,06E-01	4,962295879	2,03E+00	1,54E-01	4,98E-01	Nifk	1,08	No
ENSMUSG00000026380	-5,37E-01	-2,31114923	4,28E-01	5,13E-01	7,95E-01	Tfcp2l1	0,69	No
ENSMUSG00000026383	-8,05E-02	-0,641401623	4,23E-02	8,37E-01	9,81E-01	Epb41l5	0,95	No
ENSMUSG00000026384	1,25E-01	6,144447884	3,74E+00	5,33E-02	2,82E-01	Ptpn4	1,09	No
ENSMUSG00000026385	1,30E-01	6,257501702	4,09E+00	4,33E-02	2,47E-01	Dbi	1,09	No

ENSMUSG00000026387	1,04E-01	-1,929313121	2,37E-02	8,78E-01	1,00E+00	Sctr	1,07	No
ENSMUSG00000026388	2,11E-04	-0,419192901	3,05E-07	1,00E+00	1,00E+00	3110009E18Rik	1,00	No
ENSMUSG00000026389	3,79E-01	5,299465297	2,81E+01	1,15E-07	6,34E-06	Steap3	1,30	Yes
ENSMUSG00000026390	-4,84E-01	2,078124052	9,75E+00	1,79E-03	2,56E-02	Marco	0,72	Yes
ENSMUSG00000026393	1,49E-01	7,290164777	6,15E+00	1,32E-02	1,14E-01	Nek7	1,11	No
ENSMUSG00000026395	7,98E-02	9,190727837	1,98E+00	1,60E-01	5,07E-01	Ptprc	1,06	No
ENSMUSG00000026398	-1,89E+00	-3,1076441	1,42E+00	2,33E-01	5,50E-01	Nr5a2	0,27	No
ENSMUSG00000026399	-6,31E-01	-1,073257116	1,97E+00	1,60E-01	5,09E-01	Cd55	0,65	No
ENSMUSG00000026401	1,85E+00	-3,010054956	1,34E+00	2,47E-01	5,50E-01	Cd55b	3,60	No
ENSMUSG00000026404	-4,33E-02	2,613428198	1,19E-01	7,30E-01	9,21E-01	Ddx59	0,97	No
ENSMUSG00000026407	-1,06E+00	-2,247062922	1,80E+00	1,80E-01	5,41E-01	Cacna1s	0,48	No
ENSMUSG00000026409	2,08E-01	3,588380662	4,61E+00	3,19E-02	2,07E-01	Pfkfb2	1,16	No
ENSMUSG00000026411	8,34E-02	2,346227479	3,69E-01	5,44E-01	8,12E-01	Tmem9	1,06	No
ENSMUSG00000026413	-2,45E+00	-2,35548519	8,41E+00	3,72E-03	4,48E-02	Pkp1	0,18	Yes
ENSMUSG00000026414	-5,73E-01	-0,980569661	1,82E+00	1,78E-01	5,38E-01	Tnnt2	0,67	No
ENSMUSG00000026415	7,15E-01	-2,896163419	2,78E-01	5,98E-01	8,45E-01	Fcamr	1,64	No
ENSMUSG00000026418	4,88E-01	-2,401434966	3,24E-01	5,69E-01	8,30E-01	Tnni1	1,40	No
ENSMUSG00000026421	-7,34E-02	7,539025028	1,54E+00	2,14E-01	5,50E-01	Csrp1	0,95	No
ENSMUSG00000026425	5,58E-02	6,899230332	8,36E-01	3,60E-01	6,62E-01	Srgap2	1,04	No
ENSMUSG00000026426	6,19E-02	6,535566347	9,77E-01	3,23E-01	6,23E-01	Arl8a	1,04	No
ENSMUSG00000026427	4,02E-02	5,301648143	3,20E-01	5,72E-01	8,32E-01	Eif2d	1,03	No
ENSMUSG00000026429	2,22E-01	3,06585064	4,02E+00	4,50E-02	2,53E-01	Ube2t	1,17	No
ENSMUSG00000026430	1,54E-01	7,06658995	6,48E+00	1,09E-02	9,96E-02	Rassf5	1,11	No
ENSMUSG00000026432	2,67E+00	-3,033636951	2,76E+00	9,68E-02	3,88E-01	Avpr1b	6,36	No
ENSMUSG00000026433	-1,29E-02	4,69049164	2,77E-02	8,68E-01	1,00E+00	Rab29	0,99	No
ENSMUSG00000026434	7,37E-02	6,944174371	1,46E+00	2,27E-01	5,50E-01	Nucks1	1,05	No
ENSMUSG00000026435	-4,39E-01	0,65581546	3,60E+00	5,78E-02	2,97E-01	Slc45a3	0,74	No
ENSMUSG00000026436	9,60E-02	5,469560877	1,90E+00	1,68E-01	5,23E-01	Elk4	1,07	No
ENSMUSG00000026437	2,12E-01	5,939632348	1,03E+01	1,31E-03	2,01E-02	Cdk18	1,16	Yes
ENSMUSG00000026439	1,59E-01	5,189564835	4,96E+00	2,59E-02	1,80E-01	Rbbp5	1,12	No
ENSMUSG00000026442	-1,94E+00	-3,107619243	1,50E+00	2,21E-01	5,50E-01	Nfasc	0,26	No
ENSMUSG00000026443	-8,99E-02	-2,448698162	7,23E-03	9,32E-01	1,00E+00	Lrrn2	0,94	No

ENSMUSG00000026447	-9,96E-04	0,111917553	1,19E-05	9,97E-01	1,00E+00	Pik3c2b	1,00	No
ENSMUSG00000026450	-3,59E+00	-2,770598504	5,79E+00	1,61E-02	1,32E-01	Chit1	0,08	No
ENSMUSG00000026452	9,91E-01	-1,832026685	2,24E+00	1,34E-01	4,61E-01	Syt2	1,99	No
ENSMUSG00000026455	-2,53E-01	1,861579106	2,66E+00	1,03E-01	3,95E-01	Klhl12	0,84	No
ENSMUSG00000026456	2,06E-01	5,412653473	8,71E+00	3,16E-03	3,98E-02	Cyb5r1	1,15	Yes
ENSMUSG00000026457	-2,96E-02	7,424707747	2,48E-01	6,19E-01	8,56E-01	Adipor1	0,98	No
ENSMUSG00000026458	3,29E-01	5,220291616	2,09E+01	4,74E-06	1,72E-04	Ppfia4	1,26	Yes
ENSMUSG00000026463	-5,04E-02	3,146848755	2,20E-01	6,39E-01	8,69E-01	Atp2b4	0,97	No
ENSMUSG00000026466	4,93E-02	7,098847031	6,67E-01	4,14E-01	7,09E-01	Tor1aip1	1,03	No
ENSMUSG00000026469	1,87E-01	7,094971201	9,52E+00	2,03E-03	2,83E-02	Xpr1	1,14	Yes
ENSMUSG00000026470	3,60E-02	6,751086661	3,41E-01	5,59E-01	8,22E-01	Stx6	1,03	No
ENSMUSG00000026471	2,96E-01	5,079309828	1,61E+01	5,94E-05	1,57E-03	Mr1	1,23	Yes
ENSMUSG00000026473	-1,99E-01	6,372528254	9,92E+00	1,64E-03	2,39E-02	Glul	0,87	Yes
ENSMUSG00000026475	-4,69E-01	0,233689088	2,95E+00	8,61E-02	3,78E-01	Rgs16	0,72	No
ENSMUSG00000026478	2,47E-01	5,487561203	1,26E+01	3,77E-04	7,28E-03	Lamc1	1,19	Yes
ENSMUSG00000026479	-4,42E-02	-2,22626851	3,15E-03	9,55E-01	1,00E+00	Lamc2	0,97	No
ENSMUSG00000026480	1,11E-01	7,690398954	3,55E+00	5,94E-02	3,02E-01	Ncf2	1,08	No
ENSMUSG00000026482	-1,14E-01	6,099752915	3,04E+00	8,14E-02	3,66E-01	Rgl1	0,92	No
ENSMUSG00000026483	-1,66E-01	7,832519705	8,10E+00	4,42E-03	5,09E-02	Fam129a	0,89	No
ENSMUSG00000026484	-7,05E-02	4,695170908	8,25E-01	3,64E-01	6,65E-01	Rnf2	0,95	No
ENSMUSG00000026489	-2,61E-01	2,8553448	5,18E+00	2,29E-02	1,65E-01	Coq8a	0,83	No
ENSMUSG00000026490	-7,70E-02	3,996483186	7,54E-01	3,85E-01	6,86E-01	Cdc42bpa	0,95	No
ENSMUSG00000026491	6,10E-02	6,512365389	9,50E-01	3,30E-01	6,30E-01	Ahctf1	1,04	No
ENSMUSG00000026492	9,17E-02	3,871561645	1,01E+00	3,15E-01	6,16E-01	Tfb2m	1,07	No
ENSMUSG00000026494	-1,06E+00	-0,541792132	7,83E+00	5,13E-03	5,69E-02	Kif26b	0,48	No
ENSMUSG00000026495	-2,54E-01	0,309816863	9,91E-01	3,19E-01	6,20E-01	Efcab2	0,84	No
ENSMUSG00000026496	1,31E-01	6,429514337	4,33E+00	3,75E-02	2,30E-01	Parp1	1,10	No
ENSMUSG00000026497	-2,71E+00	-2,89615606	2,90E+00	8,84E-02	3,79E-01	Mixl1	0,15	No
ENSMUSG00000026499	4,87E-02	6,35783831	5,90E-01	4,42E-01	7,34E-01	Acbd3	1,03	No
ENSMUSG00000026500	-1,34E-01	2,997034316	1,42E+00	2,34E-01	5,50E-01	Cox20	0,91	No
ENSMUSG00000026502	1,02E-01	5,899600557	2,37E+00	1,24E-01	4,40E-01	Desi2	1,07	No
ENSMUSG00000026504	-2,64E-02	4,712658222	1,17E-01	7,33E-01	9,23E-01	Sdccag8	0,98	No

ENSMUSG00000026509	1,42E-01	7,329847036	5,63E+00	1,77E-02	1,39E-01	Capn2	1,10	No
ENSMUSG00000026510	-1,26E-01	5,505856482	3,34E+00	6,78E-02	3,28E-01	Trp53bp2	0,92	No
ENSMUSG00000026511	-2,89E-03	6,110156728	1,98E-03	9,65E-01	1,00E+00	Srp9	1,00	No
ENSMUSG00000026516	4,49E-02	5,491653727	4,25E-01	5,15E-01	7,96E-01	Nvl	1,03	No
ENSMUSG00000026519	1,96E-01	5,248460841	7,53E+00	6,07E-03	6,47E-02	Tmem63a	1,15	No
ENSMUSG00000026520	1,51E-01	5,080384821	4,23E+00	3,96E-02	2,36E-01	Pycr2	1,11	No
ENSMUSG00000026525	4,44E-02	4,061828627	2,55E-01	6,14E-01	8,53E-01	Opn3	1,03	No
ENSMUSG00000026526	1,60E-02	6,416710891	6,45E-02	8,00E-01	9,60E-01	Fh1	1,01	No
ENSMUSG00000026532	-2,48E+00	-2,497588707	5,32E+00	2,11E-02	1,56E-01	Spta1	0,18	No
ENSMUSG00000026536	-6,94E-02	3,733702256	5,54E-01	4,57E-01	7,46E-01	Ifi211	0,95	No
ENSMUSG00000026544	1,59E-01	3,306636933	2,27E+00	1,31E-01	4,56E-01	Dusp23	1,12	No
ENSMUSG00000026546	-1,04E-01	1,91019362	4,78E-01	4,90E-01	7,76E-01	Cfap45	0,93	No
ENSMUSG00000026547	-5,92E-02	9,69869464	1,10E+00	2,93E-01	5,89E-01	Tagln2	0,96	No
ENSMUSG00000026548	3,17E-01	5,671398361	2,17E+01	3,20E-06	1,23E-04	Slamf9	1,25	Yes
ENSMUSG00000026553	-6,19E-04	8,324682177	1,15E-04	9,91E-01	1,00E+00	Copa	1,00	No
ENSMUSG00000026554	-4,14E-02	6,687812183	4,48E-01	5,03E-01	7,88E-01	Dcaf8	0,97	No
ENSMUSG00000026556	-2,41E-01	-0,436700547	4,59E-01	4,98E-01	7,83E-01	Vangl2	0,85	No
ENSMUSG00000026558	4,24E-02	8,057792795	5,28E-01	4,67E-01	7,56E-01	Uck2	1,03	No
ENSMUSG00000026563	-2,72E-02	4,619364247	1,22E-01	7,27E-01	9,19E-01	Tada1	0,98	No
ENSMUSG00000026564	3,17E-01	-2,44866192	1,02E-01	7,49E-01	9,31E-01	Dusp27	1,25	No
ENSMUSG00000026565	-7,54E-02	5,09425276	1,07E+00	3,01E-01	5,97E-01	Pou2f1	0,95	No
ENSMUSG00000026566	1,55E-01	3,288095901	2,20E+00	1,38E-01	4,68E-01	Mpzl1	1,11	No
ENSMUSG00000026567	-9,70E-01	-2,548199559	8,23E-01	3,64E-01	6,65E-01	Adcy10	0,51	No
ENSMUSG00000026568	-1,87E-02	4,789675414	5,93E-02	8,08E-01	9,64E-01	Mpc2	0,99	No
ENSMUSG00000026571	-1,15E-01	4,678110863	2,17E+00	1,40E-01	4,73E-01	Dcaf6	0,92	No
ENSMUSG00000026574	1,87E+00	-3,107639691	1,38E+00	2,40E-01	5,50E-01	Dpt	3,67	No
ENSMUSG00000026575	1,81E-02	2,560363192	1,97E-02	8,88E-01	1,00E+00	Nme7	1,01	No
ENSMUSG00000026576	-3,85E-01	1,066793165	3,84E+00	5,00E-02	2,71E-01	Atp1b1	0,77	No
ENSMUSG00000026577	1,26E-01	4,350826836	2,32E+00	1,28E-01	4,49E-01	Blzf1	1,09	No
ENSMUSG00000026578	-3,19E-02	2,931989157	7,81E-02	7,80E-01	9,48E-01	Ccdc181	0,98	No
ENSMUSG00000026579	-3,83E-01	3,050442533	1,16E+01	6,52E-04	1,14E-02	F5	0,77	Yes
ENSMUSG00000026580	1,94E-01	-0,333423246	3,46E-01	5,56E-01	8,21E-01	Selp	1,14	No

ENSMUSG00000026581	-2,34E-01	2,800751453	3,89E+00	4,85E-02	2,65E-01	Sell	0,85	No
ENSMUSG00000026582	-1,37E+00	-2,89609265	1,18E+00	2,78E-01	5,74E-01	Sele	0,39	No
ENSMUSG00000026584	7,64E-02	5,383849404	1,20E+00	2,74E-01	5,70E-01	Scyl3	1,05	No
ENSMUSG00000026585	5,82E-02	4,159658083	4,60E-01	4,98E-01	7,83E-01	Kifap3	1,04	No
ENSMUSG00000026586	-1,20E+00	-2,355469149	1,86E+00	1,73E-01	5,30E-01	Prrx1	0,44	No
ENSMUSG00000026587	2,64E+00	-3,033673223	2,69E+00	1,01E-01	3,92E-01	Astn1	6,23	No
ENSMUSG00000026589	-1,19E-01	0,281084824	2,08E-01	6,48E-01	8,75E-01	Sec16b	0,92	No
ENSMUSG00000026592	-2,02E+00	-2,548143853	3,11E+00	7,80E-02	3,57E-01	Tex35	0,25	No
ENSMUSG00000026594	-4,97E-02	0,621857025	4,44E-02	8,33E-01	9,78E-01	Ralgps2	0,97	No
ENSMUSG00000026596	8,47E-02	5,940969016	1,65E+00	1,99E-01	5,50E-01	Abl2	1,06	No
ENSMUSG00000026600	1,85E-01	8,592951484	1,04E+01	1,26E-03	1,95E-02	Soat1	1,14	Yes
ENSMUSG00000026601	-1,00E-01	-0,648371138	6,82E-02	7,94E-01	9,56E-01	Axdnd1	0,93	No
ENSMUSG00000026602	2,66E+00	-2,896131654	2,73E+00	9,86E-02	3,91E-01	Nphs2	6,32	No
ENSMUSG00000026603	4,39E-02	4,567847328	3,06E-01	5,80E-01	8,37E-01	Smyd2	1,03	No
ENSMUSG00000026604	-6,97E-01	0,295723044	6,90E+00	8,62E-03	8,37E-02	Ptpn14	0,62	No
ENSMUSG00000026605	3,47E-01	5,757827123	2,66E+01	2,46E-07	1,27E-05	Cenpf	1,27	Yes
ENSMUSG00000026608	2,48E-01	4,105615966	8,12E+00	4,37E-03	5,05E-02	Kctd3	1,19	No
ENSMUSG00000026609	-1,14E-02	-3,033635021	1,50E-05	9,97E-01	1,00E+00	Ush2a	0,99	No
ENSMUSG00000026610	-3,83E-02	-3,107651936	-7,91E-11	1,00E+00	1,00E+00	Esrrg	0,97	No
ENSMUSG00000026611	-9,02E-02	-2,83204665	5,62E-03	9,40E-01	1,00E+00	Spata17	0,94	No
ENSMUSG00000026614	-1,89E+00	-3,1076441	1,42E+00	2,33E-01	5,50E-01	Slc30a10	0,27	No
ENSMUSG00000026615	4,48E-02	6,912666372	5,40E-01	4,63E-01	7,51E-01	Eprs	1,03	No
ENSMUSG00000026616	1,55E+00	-2,355611994	3,61E+00	5,75E-02	2,95E-01	Cr2	2,93	No
ENSMUSG00000026617	9,96E-02	4,311152883	1,43E+00	2,31E-01	5,50E-01	Bpnt1	1,07	No
ENSMUSG00000026618	1,14E-01	6,136368553	3,14E+00	7,65E-02	3,53E-01	Iars2	1,08	No
ENSMUSG00000026620	-2,71E+00	-2,896132076	2,92E+00	8,76E-02	3,78E-01	Mark1	0,15	No
ENSMUSG00000026621	1,68E+00	-2,185662076	4,41E+00	3,58E-02	2,24E-01	Marc1	3,20	No
ENSMUSG00000026622	2,51E-01	4,796782232	1,08E+01	1,04E-03	1,66E-02	Nek2	1,19	Yes
ENSMUSG00000026623	1,15E-01	6,812112737	3,53E+00	6,02E-02	3,04E-01	Lpgat1	1,08	No
ENSMUSG00000026626	4,84E-02	6,505522797	5,96E-01	4,40E-01	7,32E-01	Ppp2r5a	1,03	No
ENSMUSG00000026627	2,40E-01	5,136174897	1,09E+01	9,76E-04	1,59E-02	Tmem206	1,18	Yes
ENSMUSG00000026628	2,33E-03	7,888721677	1,58E-03	9,68E-01	1,00E+00	Atf3	1,00	No

ENSMUSG00000026630	-4,92E-01	5,203263094	4,76E+01	5,32E-12	6,00E-10	Batf3	0,71	Yes
ENSMUSG00000026632	-7,25E-02	2,970117896	4,15E-01	5,20E-01	7,99E-01	Tatdn3	0,95	No
ENSMUSG00000026634	7,31E-03	5,742443737	1,18E-02	9,13E-01	1,00E+00	Angel2	1,01	No
ENSMUSG00000026637	2,00E-01	5,344731883	8,13E+00	4,37E-03	5,05E-02	Traf5	1,15	No
ENSMUSG00000026638	-5,29E-01	-0,210885122	3,14E+00	7,64E-02	3,52E-01	Irf6	0,69	No
ENSMUSG00000026639	8,48E-02	2,287262354	3,73E-01	5,41E-01	8,11E-01	Lamb3	1,06	No
ENSMUSG00000026640	2,69E-01	1,750146	2,75E+00	9,74E-02	3,89E-01	Plxna2	1,20	No
ENSMUSG00000026641	1,20E-02	5,447286678	3,04E-02	8,62E-01	9,96E-01	Usf1	1,01	No
ENSMUSG00000026643	9,96E-02	5,67597564	2,15E+00	1,42E-01	4,77E-01	Nmt2	1,07	No
ENSMUSG00000026644	1,81E+00	-3,10765737	1,28E+00	2,58E-01	5,50E-01	Acbd7	3,49	No
ENSMUSG00000026646	-2,01E-01	3,000128737	3,30E+00	6,94E-02	3,32E-01	Suv39h2	0,87	No
ENSMUSG00000026648	-3,80E-01	5,51179516	3,09E+01	2,69E-08	1,70E-06	Dclre1c	0,77	Yes
ENSMUSG00000026649	5,77E-01	0,655827803	6,18E+00	1,29E-02	1,13E-01	Cfap126	1,49	No
ENSMUSG00000026650	-7,64E-01	-2,497597187	6,54E-01	4,19E-01	7,14E-01	Meig1	0,59	No
ENSMUSG00000026655	-1,36E-01	6,126959153	4,45E+00	3,49E-02	2,21E-01	Fam107b	0,91	No
ENSMUSG00000026656	2,99E-01	9,273501738	2,77E+01	1,38E-07	7,41E-06	Fcgr2b	1,23	Yes
ENSMUSG00000026657	5,26E-02	6,151459248	6,69E-01	4,13E-01	7,09E-01	Frmd4a	1,04	No
ENSMUSG00000026659	2,76E-02	2,870089106	5,67E-02	8,12E-01	9,67E-01	Dusp12	1,02	No
ENSMUSG00000026662	-9,01E-02	5,638768894	1,78E+00	1,83E-01	5,45E-01	Sephs1	0,94	No
ENSMUSG00000026663	1,43E-01	6,340821625	5,07E+00	2,43E-02	1,72E-01	Atf6	1,10	No
ENSMUSG00000026664	-3,48E-01	4,382666631	1,84E+01	1,78E-05	5,57E-04	Phyh	0,79	Yes
ENSMUSG00000026667	-5,96E-02	8,324721872	1,07E+00	3,01E-01	5,97E-01	Uhmk1	0,96	No
ENSMUSG00000026668	1,11E+00	-2,600732664	1,17E+00	2,79E-01	5,76E-01	Ucma	2,16	No
ENSMUSG00000026669	3,29E-01	4,849438693	1,88E+01	1,48E-05	4,74E-04	Mcm10	1,26	Yes
ENSMUSG00000026670	-2,93E-01	5,866010135	1,94E+01	1,05E-05	3,52E-04	Uap1	0,82	Yes
ENSMUSG00000026672	-9,02E-02	3,469771135	8,19E-01	3,65E-01	6,66E-01	Optn	0,94	No
ENSMUSG00000026674	-9,81E-01	-2,145955068	1,49E+00	2,23E-01	5,50E-01	Ddr2	0,51	No
ENSMUSG00000026675	2,72E-01	4,663563035	1,21E+01	5,18E-04	9,45E-03	Hsd17b7	1,21	Yes
ENSMUSG00000026678	-3,18E+00	-2,896135562	4,24E+00	3,94E-02	2,35E-01	Rgs5	0,11	No
ENSMUSG00000026679	2,98E-02	-1,355507437	3,43E-03	9,53E-01	1,00E+00	Enkur	1,02	No
ENSMUSG00000026683	2,25E-01	4,487412285	7,81E+00	5,19E-03	5,75E-02	Nuf2	1,17	No
ENSMUSG00000026687	-4,98E-02	7,580602318	7,11E-01	3,99E-01	6,98E-01	Aldh9a1	0,97	No

ENSMUSG00000026688	3,06E-01	3,19540343	7,87E+00	5,04E-03	5,62E-02	Mgst3	1,24	No
ENSMUSG00000026691	-3,83E-02	-3,107651936	-7,91E-11	1,00E+00	1,00E+00	Fmo3	0,97	No
ENSMUSG00000026692	9,01E-03	-3,107646452	-5,16E-10	1,00E+00	1,00E+00	Fmo4	1,01	No
ENSMUSG00000026694	3,04E-01	4,415727039	1,37E+01	2,09E-04	4,51E-03	Mettl13	1,23	Yes
ENSMUSG00000026696	7,27E-02	5,743864591	1,17E+00	2,80E-01	5,77E-01	Vamp4	1,05	No
ENSMUSG00000026697	1,81E+00	-3,10765737	1,28E+00	2,58E-01	5,50E-01	Myoc	3,49	No
ENSMUSG00000026698	4,86E-02	4,307487265	3,43E-01	5,58E-01	8,22E-01	Pigc	1,03	No
ENSMUSG00000026700	-5,00E-01	4,946374916	4,62E+01	1,06E-11	1,12E-09	Tnfsf4	0,71	Yes
ENSMUSG00000026701	7,60E-02	7,406878543	1,63E+00	2,02E-01	5,50E-01	Prdx6	1,05	No
ENSMUSG00000026705	1,67E-01	5,098862041	5,30E+00	2,13E-02	1,57E-01	Klhl20	1,12	No
ENSMUSG00000026707	1,43E-01	2,747297686	1,42E+00	2,33E-01	5,50E-01	Nsun6	1,10	No
ENSMUSG00000026708	1,39E-01	4,265625459	2,74E+00	9,79E-02	3,90E-01	Cenpl	1,10	No
ENSMUSG00000026709	1,42E-01	3,847952658	2,41E+00	1,21E-01	4,34E-01	Dars2	1,10	No
ENSMUSG00000026712	3,89E-01	9,296114942	4,67E+01	8,08E-12	8,65E-10	Mrc1	1,31	Yes
ENSMUSG00000026715	-1,27E-01	1,414152333	4,99E-01	4,80E-01	7,67E-01	Serpinc1	0,92	No
ENSMUSG00000026718	-3,41E-02	4,60377058	1,86E-01	6,66E-01	8,85E-01	Stam	0,98	No
ENSMUSG00000026721	-7,01E-01	6,864962036	1,31E+02	2,17E-30	1,28E-27	Rabgap1l	0,62	Yes
ENSMUSG00000026723	8,29E-02	2,819143795	4,97E-01	4,81E-01	7,69E-01	Trdmt1	1,06	No
ENSMUSG00000026725	-2,65E+00	-2,770536152	2,80E+00	9,41E-02	3,84E-01	Tnn	0,16	No
ENSMUSG00000026726	-5,51E-01	-1,060731905	1,42E+00	2,33E-01	5,50E-01	Cubn	0,68	No
ENSMUSG00000026727	3,28E-02	6,850227361	2,86E-01	5,93E-01	8,42E-01	Rsu1	1,02	No
ENSMUSG00000026728	-3,66E-02	11,75953715	4,30E-01	5,12E-01	7,95E-01	Vim	0,97	No
ENSMUSG00000026729	-2,64E-01	2,129456618	3,49E+00	6,18E-02	3,09E-01	4930562F07Rik	0,83	No
ENSMUSG00000026730	3,45E-01	2,870549906	8,43E+00	3,69E-03	4,46E-02	Pter	1,27	Yes
ENSMUSG00000026734	1,85E+00	-3,107657832	1,35E+00	2,46E-01	5,50E-01	4921504E06Rik	3,61	No
ENSMUSG00000026735	1,60E+00	-2,378335931	1,75E+00	1,86E-01	5,50E-01	Ptf1a	3,03	No
ENSMUSG00000026736	-1,62E-01	0,070698299	3,22E-01	5,71E-01	8,31E-01	4930426L09Rik	0,89	No
ENSMUSG00000026737	-1,50E-01	7,658776114	6,47E+00	1,10E-02	9,98E-02	Pip4k2a	0,90	No
ENSMUSG00000026739	-7,90E-02	5,17232719	1,21E+00	2,72E-01	5,68E-01	Bmi1	0,95	No
ENSMUSG00000026740	-9,56E-02	5,982650957	2,14E+00	1,44E-01	4,79E-01	Dnajc1	0,94	No
ENSMUSG00000026743	-1,27E-01	5,541318056	3,39E+00	6,55E-02	3,21E-01	Mllt10	0,92	No
ENSMUSG00000026748	-5,84E-03	8,338013023	1,02E-02	9,20E-01	1,00E+00	Plxdc2	1,00	No

ENSMUSG00000026749	-1,98E-02	8,097487394	1,16E-01	7,33E-01	9,23E-01	Nek6	0,99	No
ENSMUSG00000026750	-4,73E-02	5,369041467	4,51E-01	5,02E-01	7,87E-01	Psmb7	0,97	No
ENSMUSG00000026751	-2,75E+00	-2,963216826	2,99E+00	8,38E-02	3,71E-01	Nr5a1	0,15	No
ENSMUSG00000026753	5,15E-02	5,932940979	6,11E-01	4,34E-01	7,27E-01	Ppp6c	1,04	No
ENSMUSG00000026754	-8,03E-02	4,042006007	8,61E-01	3,53E-01	6,55E-01	Golga1	0,95	No
ENSMUSG00000026755	7,71E-03	5,869322433	1,35E-02	9,07E-01	1,00E+00	Arpc5l	1,01	No
ENSMUSG00000026761	7,28E-02	4,60173899	8,52E-01	3,56E-01	6,57E-01	Orc4	1,05	No
ENSMUSG00000026764	4,11E-01	3,119974713	1,44E+01	1,45E-04	3,30E-03	Kif5c	1,33	Yes
ENSMUSG00000026765	-4,28E-01	-2,35553828	1,87E-01	6,65E-01	8,85E-01	Lypd6b	0,74	No
ENSMUSG00000026766	8,17E-02	5,360977797	1,34E+00	2,47E-01	5,50E-01	Mmadhc	1,06	No
ENSMUSG00000026767	7,40E-02	4,889750138	9,55E-01	3,29E-01	6,29E-01	Mindy3	1,05	No
ENSMUSG00000026768	8,72E-01	-2,015666061	1,17E+00	2,79E-01	5,76E-01	Itga8	1,83	No
ENSMUSG00000026770	-5,26E-01	6,601623398	7,17E+01	2,47E-17	4,75E-15	Il2ra	0,69	Yes
ENSMUSG00000026771	-5,13E-02	5,555448917	5,60E-01	4,54E-01	7,44E-01	Spopl	0,97	No
ENSMUSG00000026773	-2,75E-01	6,170180656	1,86E+01	1,65E-05	5,22E-04	Pfkfb3	0,83	Yes
ENSMUSG00000026774	-9,57E-02	-2,770631803	6,20E-03	9,37E-01	1,00E+00	4931423N10Rik	0,94	No
ENSMUSG00000026775	-2,56E-02	6,616398887	1,70E-01	6,80E-01	8,93E-01	Yme1l1	0,98	No
ENSMUSG00000026778	3,16E-01	2,468143841	5,75E+00	1,65E-02	1,34E-01	Prkcq	1,24	No
ENSMUSG00000026779	3,42E-01	4,301882152	1,67E+01	4,36E-05	1,21E-03	Mastl	1,27	Yes
ENSMUSG00000026781	1,95E-01	5,748922372	8,36E+00	3,84E-03	4,59E-02	Acbd5	1,14	Yes
ENSMUSG00000026782	1,12E-02	5,031211069	2,29E-02	8,80E-01	1,00E+00	Abi2	1,01	No
ENSMUSG00000026784	2,94E-01	2,93849279	6,50E+00	1,08E-02	9,89E-02	Pdss1	1,23	No
ENSMUSG00000026785	2,73E-01	3,349990531	6,99E+00	8,21E-03	8,06E-02	Pkn3	1,21	No
ENSMUSG00000026786	6,88E-02	6,515618025	1,20E+00	2,73E-01	5,69E-01	Apbb1ip	1,05	No
ENSMUSG00000026788	-1,56E-01	4,680295272	3,94E+00	4,73E-02	2,60E-01	Zbtb43	0,90	No
ENSMUSG00000026790	1,72E-01	6,374849638	7,39E+00	6,56E-03	6,85E-02	Odf2	1,13	No
ENSMUSG00000026791	2,05E-02	4,088473189	5,54E-02	8,14E-01	9,68E-01	Slc2a8	1,01	No
ENSMUSG00000026792	-6,70E-02	4,235004599	6,32E-01	4,27E-01	7,21E-01	Lrsam1	0,95	No
ENSMUSG00000026796	1,93E-01	8,816966924	1,14E+01	7,18E-04	1,23E-02	Fam129b	1,14	Yes
ENSMUSG00000026797	-1,71E-01	4,014967471	3,74E+00	5,31E-02	2,81E-01	Stxbp1	0,89	No
ENSMUSG00000026798	8,45E-02	2,587732373	4,51E-01	5,02E-01	7,87E-01	Coq4	1,06	No
ENSMUSG00000026799	-9,05E-02	3,606017322	8,61E-01	3,53E-01	6,55E-01	Med27	0,94	No

ENSMUSG00000026803	8,29E-02	4,252365399	9,91E-01	3,20E-01	6,20E-01	Ttf1	1,06	No
ENSMUSG00000026805	2,67E-01	-1,740797509	1,64E-01	6,85E-01	8,97E-01	Barhl1	1,20	No
ENSMUSG00000026806	3,34E-01	3,689325734	1,23E+01	4,54E-04	8,48E-03	Ddx31	1,26	Yes
ENSMUSG00000026807	3,76E-01	3,683177093	1,49E+01	1,13E-04	2,69E-03	Ak8	1,30	Yes
ENSMUSG00000026809	3,97E-01	2,172921971	7,45E+00	6,36E-03	6,70E-02	Spaca9	1,32	No
ENSMUSG00000026810	3,77E-02	5,051925814	2,64E-01	6,07E-01	8,50E-01	Dpm2	1,03	No
ENSMUSG00000026811	-1,40E-01	5,985441384	4,60E+00	3,20E-02	2,08E-01	St6galnac6	0,91	No
ENSMUSG00000026812	-2,00E-01	6,21294642	9,79E+00	1,75E-03	2,52E-02	Tsc1	0,87	Yes
ENSMUSG00000026814	8,04E-02	5,421060099	1,32E+00	2,50E-01	5,50E-01	Eng	1,06	No
ENSMUSG00000026815	-1,83E-01	2,52556343	2,01E+00	1,56E-01	5,01E-01	Gfi1b	0,88	No
ENSMUSG00000026816	1,07E-01	4,657990254	1,85E+00	1,73E-01	5,30E-01	Gtf3c5	1,08	No
ENSMUSG00000026817	1,48E-01	3,466999004	2,15E+00	1,43E-01	4,77E-01	Ak1	1,11	No
ENSMUSG00000026818	-1,90E+00	-2,832003574	1,44E+00	2,30E-01	5,50E-01	Cel	0,27	No
ENSMUSG00000026819	9,58E-02	4,935471428	1,63E+00	2,01E-01	5,50E-01	Slc25a25	1,07	No
ENSMUSG00000026820	9,70E-02	4,354831669	1,37E+00	2,43E-01	5,50E-01	Ptges2	1,07	No
ENSMUSG00000026821	7,19E-03	7,43193697	1,45E-02	9,04E-01	1,00E+00	Ralgds	1,00	No
ENSMUSG00000026822	-4,09E-01	0,144516729	2,25E+00	1,34E-01	4,60E-01	Lcn2	0,75	No
ENSMUSG00000026825	-1,01E+00	-0,945891564	5,60E+00	1,80E-02	1,41E-01	Dnm1	0,50	No
ENSMUSG00000026826	-1,74E-02	3,791890324	3,32E-02	8,55E-01	9,92E-01	Nr4a2	0,99	No
ENSMUSG00000026827	3,14E-02	6,034925418	2,32E-01	6,30E-01	8,62E-01	Gpd2	1,02	No
ENSMUSG00000026829	3,38E-01	4,217392982	1,55E+01	8,10E-05	2,03E-03	Gbgt1	1,26	Yes
ENSMUSG00000026831	-7,81E-02	0,112144439	7,56E-02	7,83E-01	9,50E-01	1700007K13Rik	0,95	No
ENSMUSG00000026832	-6,74E-01	9,098191538	1,40E+02	3,09E-32	2,02E-29	Cytip	0,63	Yes
ENSMUSG00000026833	3,22E-01	8,457258053	3,12E+01	2,37E-08	1,52E-06	Olfm1	1,25	Yes
ENSMUSG00000026834	-2,50E+00	-1,509915217	1,59E+01	6,80E-05	1,76E-03	Acvr1c	0,18	Yes
ENSMUSG00000026835	-1,22E+00	-1,963243315	3,56E+00	5,94E-02	3,01E-01	Fcnb	0,43	No
ENSMUSG00000026836	2,70E-02	4,087942381	9,61E-02	7,57E-01	9,35E-01	Acvr1	1,02	No
ENSMUSG00000026837	-1,79E-01	1,978153822	1,42E+00	2,33E-01	5,50E-01	Col5a1	0,88	No
ENSMUSG00000026839	3,18E-01	0,036637239	1,32E+00	2,51E-01	5,50E-01	Upp2	1,25	No
ENSMUSG00000026840	1,87E+00	-3,107639691	1,38E+00	2,40E-01	5,50E-01	Lamc3	3,67	No
ENSMUSG00000026841	-6,22E-02	-2,711722651	3,52E-03	9,53E-01	1,00E+00	Fibcd1	0,96	No
ENSMUSG00000026842	4,56E-02	6,727040491	5,43E-01	4,61E-01	7,50E-01	Abl1	1,03	No

ENSMUSG00000026843	4,01E-02	5,013593183	2,96E-01	5,86E-01	8,39E-01	Fubp3	1,03	No
ENSMUSG00000026848	8,94E-02	6,405895817	2,00E+00	1,57E-01	5,03E-01	Tor1b	1,06	No
ENSMUSG00000026849	9,93E-02	5,590557411	2,09E+00	1,48E-01	4,88E-01	Tor1a	1,07	No
ENSMUSG00000026851	3,49E-03	4,668501906	1,99E-03	9,64E-01	1,00E+00	BC005624	1,00	No
ENSMUSG00000026853	2,41E-01	7,107028871	1,59E+01	6,82E-05	1,76E-03	Crat	1,18	Yes
ENSMUSG00000026854	1,01E-01	4,238847313	1,43E+00	2,32E-01	5,50E-01	Usp20	1,07	No
ENSMUSG00000026856	2,12E-01	5,758939547	9,97E+00	1,59E-03	2,33E-02	Dolpp1	1,16	Yes
ENSMUSG00000026857	-3,96E-02	4,744633809	2,64E-01	6,07E-01	8,50E-01	Ntmt1	0,97	No
ENSMUSG00000026858	3,36E-01	4,11671521	1,48E+01	1,20E-04	2,83E-03	Miga2	1,26	Yes
ENSMUSG00000026860	6,77E-02	5,625513923	9,88E-01	3,20E-01	6,21E-01	Sh3glb2	1,05	No
ENSMUSG00000026864	1,05E-01	9,63009923	3,44E+00	6,35E-02	3,15E-01	Hspa5	1,08	No
ENSMUSG00000026866	-2,90E-01	5,40327871	1,76E+01	2,73E-05	8,08E-04	Kynu	0,82	Yes
ENSMUSG00000026867	2,99E-02	7,026591091	2,44E-01	6,21E-01	8,57E-01	Gapvd1	1,02	No
ENSMUSG00000026869	6,34E-02	5,355886323	8,13E-01	3,67E-01	6,68E-01	Psmd5	1,04	No
ENSMUSG00000026870	-6,88E-02	-2,896141077	2,08E-03	9,64E-01	1,00E+00	Cutal	0,95	No
ENSMUSG00000026872	3,13E-01	8,117130798	2,89E+01	7,57E-08	4,32E-06	Zeb2	1,24	Yes
ENSMUSG00000026873	2,36E-01	3,751698864	6,44E+00	1,11E-02	1,01E-01	Phf19	1,18	No
ENSMUSG00000026874	-8,55E-01	-1,522665633	2,89E+00	8,89E-02	3,79E-01	Hc	0,55	No
ENSMUSG00000026875	-6,63E-01	6,888834569	1,18E+02	1,52E-27	6,92E-25	Traf1	0,63	Yes
ENSMUSG00000026878	-4,42E-02	8,267311629	5,87E-01	4,44E-01	7,35E-01	Rab14	0,97	No
ENSMUSG00000026879	8,78E-02	8,546084459	2,34E+00	1,26E-01	4,45E-01	Gsn	1,06	No
ENSMUSG00000026880	8,17E-02	6,87242582	1,78E+00	1,82E-01	5,43E-01	Stom	1,06	No
ENSMUSG00000026883	-2,31E-01	0,804408196	1,08E+00	2,99E-01	5,95E-01	Dab2ip	0,85	No
ENSMUSG00000026885	-3,84E-01	0,962241544	3,36E+00	6,67E-02	3,25E-01	Ttll11	0,77	No
ENSMUSG00000026887	5,26E-03	3,205778998	2,47E-03	9,60E-01	1,00E+00	Mrrf	1,00	No
ENSMUSG00000026888	-5,46E-01	-1,472857174	1,07E+00	3,02E-01	5,98E-01	Grb14	0,68	No
ENSMUSG00000026889	-9,10E-02	5,02073883	1,50E+00	2,20E-01	5,50E-01	Rbm18	0,94	No
ENSMUSG00000026890	9,01E-03	-3,107646452	-5,16E-10	1,00E+00	1,00E+00	Lhx6	1,01	No
ENSMUSG00000026893	-3,89E-01	3,593378525	1,66E+01	4,52E-05	1,25E-03	Gca	0,76	Yes
ENSMUSG00000026894	-5,57E-01	-2,711723534	2,43E-01	6,22E-01	8,58E-01	Morn5	0,68	No
ENSMUSG00000026895	1,57E-02	5,656181126	5,32E-02	8,18E-01	9,70E-01	Ndufa8	1,01	No
ENSMUSG00000026896	7,42E-04	5,098268132	1,05E-04	9,92E-01	1,00E+00	Ifih1	1,00	No

ENSMUSG00000026904	-7,48E-01	-2,448680383	6,28E-01	4,28E-01	7,22E-01	Slc4a10	0,60	No
ENSMUSG00000026914	-5,72E-02	5,790007271	7,32E-01	3,92E-01	6,93E-01	Psmd14	0,96	No
ENSMUSG00000026915	-4,87E-01	4,103626958	3,18E+01	1,71E-08	1,12E-06	Strbp	0,71	Yes
ENSMUSG00000026917	5,27E-02	5,45573183	5,74E-01	4,49E-01	7,39E-01	Wdr5	1,04	No
ENSMUSG00000026918	1,61E-01	5,11159878	4,96E+00	2,60E-02	1,80E-01	Brd3	1,12	No
ENSMUSG00000026919	1,87E+00	-3,107639691	1,38E+00	2,40E-01	5,50E-01	Lcn4	3,67	No
ENSMUSG00000026921	-1,97E-01	-0,401308161	3,11E-01	5,77E-01	8,35E-01	Egfl7	0,87	No
ENSMUSG00000026922	9,24E-02	4,424182785	1,28E+00	2,57E-01	5,50E-01	Agpat2	1,07	No
ENSMUSG00000026923	1,62E-01	7,812968713	7,59E+00	5,86E-03	6,30E-02	Notch1	1,12	No
ENSMUSG00000026924	8,05E-02	7,357549246	1,82E+00	1,77E-01	5,36E-01	Sec16a	1,06	No
ENSMUSG00000026925	7,78E-02	5,36183559	1,23E+00	2,68E-01	5,65E-01	Inpp5e	1,06	No
ENSMUSG00000026926	-8,14E-02	6,272025443	1,62E+00	2,03E-01	5,50E-01	Pmpca	0,95	No
ENSMUSG00000026927	-3,43E-02	5,936261111	2,70E-01	6,03E-01	8,48E-01	Sdccag3	0,98	No
ENSMUSG00000026928	3,12E-02	6,818473158	2,58E-01	6,11E-01	8,52E-01	Card9	1,02	No
ENSMUSG00000026930	-1,49E-01	2,94546763	1,72E+00	1,90E-01	5,50E-01	Gpsm1	0,90	No
ENSMUSG00000026932	6,61E-02	4,998332078	7,93E-01	3,73E-01	6,74E-01	Nacc2	1,05	No
ENSMUSG00000026933	1,82E-01	4,491480283	5,18E+00	2,29E-02	1,65E-01	Camsap1	1,13	No
ENSMUSG00000026937	-2,63E-02	-3,033641358	2,33E-04	9,88E-01	1,00E+00	Lcn5	0,98	No
ENSMUSG00000026938	-2,87E-01	1,064272823	1,98E+00	1,60E-01	5,07E-01	Fcna	0,82	No
ENSMUSG00000026939	1,65E-01	3,526924293	2,69E+00	1,01E-01	3,92E-01	Tmem141	1,12	No
ENSMUSG00000026940	3,48E-01	-2,311213564	2,08E-01	6,49E-01	8,75E-01	Ccdc183	1,27	No
ENSMUSG00000026941	1,30E-01	2,431889566	9,80E-01	3,22E-01	6,23E-01	Mamdc4	1,09	No
ENSMUSG00000026942	-1,41E-01	4,708647233	3,41E+00	6,49E-02	3,19E-01	Traf2	0,91	No
ENSMUSG00000026943	-4,37E-01	-2,600662862	1,95E-01	6,59E-01	8,81E-01	Lcn12	0,74	No
ENSMUSG00000026944	8,14E-02	6,365610387	1,64E+00	2,00E-01	5,50E-01	Abca2	1,06	No
ENSMUSG00000026946	-7,48E-02	3,711085534	6,36E-01	4,25E-01	7,19E-01	Nmi	0,95	No
ENSMUSG00000026950	2,76E-01	1,791747902	3,00E+00	8,33E-02	3,71E-01	Neb	1,21	No
ENSMUSG00000026955	2,82E-01	3,042265902	6,48E+00	1,09E-02	9,95E-02	Sapcd2	1,22	No
ENSMUSG00000026956	2,59E-01	6,894247546	1,79E+01	2,38E-05	7,22E-04	Uap1l1	1,20	Yes
ENSMUSG00000026958	1,09E-01	6,999786006	3,22E+00	7,28E-02	3,42E-01	Dpp7	1,08	No
ENSMUSG00000026959	-2,76E-01	-1,322024619	2,83E-01	5,94E-01	8,43E-01	Grin1	0,83	No
ENSMUSG00000026960	1,61E-01	4,28024513	3,65E+00	5,60E-02	2,90E-01	Arl6ip6	1,12	No

ENSMUSG00000026961	-2,70E+00	-3,033614577	2,89E+00	8,93E-02	3,79E-01	Lrrc26	0,15	No
ENSMUSG00000026965	-5,59E-02	6,961418765	8,47E-01	3,57E-01	6,59E-01	Anapc2	0,96	No
ENSMUSG00000026966	-1,18E-02	5,603345325	2,95E-02	8,64E-01	9,97E-01	Ssna1	0,99	No
ENSMUSG00000026969	3,79E-02	2,881309745	1,05E-01	7,46E-01	9,29E-01	Fam166a	1,03	No
ENSMUSG00000026970	5,38E-02	7,797593099	8,43E-01	3,59E-01	6,60E-01	Rbms1	1,04	No
ENSMUSG00000026971	-1,87E+00	-3,107637761	1,39E+00	2,38E-01	5,50E-01	Itgb6	0,27	No
ENSMUSG00000026972	-1,28E-01	6,39592213	4,12E+00	4,23E-02	2,45E-01	Arrdc1	0,92	No
ENSMUSG00000026974	-6,24E-02	4,252479146	5,50E-01	4,58E-01	7,47E-01	Zmynd19	0,96	No
ENSMUSG00000026975	-1,43E-01	3,233979976	1,83E+00	1,77E-01	5,36E-01	Dph7	0,91	No
ENSMUSG00000026976	-1,89E-01	-0,762854221	1,93E-01	6,61E-01	8,82E-01	Pax8	0,88	No
ENSMUSG00000026977	-1,32E-01	6,637062811	4,53E+00	3,32E-02	2,14E-01	March7	0,91	No
ENSMUSG00000026979	1,33E-01	7,680828143	5,13E+00	2,35E-02	1,68E-01	Psd4	1,10	No
ENSMUSG00000026980	-5,58E-01	6,268193191	7,68E+01	1,91E-18	3,87E-16	Ly75	0,68	Yes
ENSMUSG00000026981	-1,50E-01	7,564985026	6,44E+00	1,11E-02	1,01E-01	Il1rn	0,90	No
ENSMUSG00000026984	-2,71E+00	-3,033620915	2,92E+00	8,75E-02	3,78E-01	Il1f6	0,15	No
ENSMUSG00000026985	1,81E+00	-3,10765737	1,28E+00	2,58E-01	5,50E-01	Il1f8	3,49	No
ENSMUSG00000026986	1,89E-01	2,276782534	1,80E+00	1,80E-01	5,42E-01	Hnmt	1,14	No
ENSMUSG00000026987	3,45E-03	6,772442651	3,13E-03	9,55E-01	1,00E+00	Baz2b	1,00	No
ENSMUSG00000026988	-1,29E-01	3,412697898	1,62E+00	2,02E-01	5,50E-01	Wdsub1	0,91	No
ENSMUSG00000026989	1,81E+00	-3,10765737	1,28E+00	2,58E-01	5,50E-01	Dapl1	3,49	No
ENSMUSG00000026991	1,35E-01	5,845680287	4,11E+00	4,27E-02	2,46E-01	Pkp4	1,10	No
ENSMUSG00000026994	3,38E-01	5,076020151	2,08E+01	5,19E-06	1,87E-04	Galnt3	1,26	Yes
ENSMUSG00000026999	1,82E-01	2,504267735	2,03E+00	1,55E-01	4,98E-01	Nup35	1,13	No
ENSMUSG00000027001	1,02E-01	3,220005697	9,44E-01	3,31E-01	6,32E-01	Dusp19	1,07	No
ENSMUSG00000027002	-9,30E-02	3,760163787	9,92E-01	3,19E-01	6,20E-01	Nckap1	0,94	No
ENSMUSG00000027004	-8,58E-01	-1,015586885	4,24E+00	3,95E-02	2,36E-01	Frzb	0,55	No
ENSMUSG00000027006	-1,99E-01	6,943708271	1,08E+01	1,02E-03	1,65E-02	Dnajc10	0,87	Yes
ENSMUSG00000027007	-1,79E-01	7,109456041	8,81E+00	2,99E-03	3,83E-02	Ssfa2	0,88	Yes
ENSMUSG00000027009	-4,00E-01	6,718665663	4,21E+01	8,61E-11	8,27E-09	Itga4	0,76	Yes
ENSMUSG00000027010	-3,53E-02	5,973736277	2,89E-01	5,91E-01	8,41E-01	Slc25a12	0,98	No
ENSMUSG00000027011	-7,05E-02	4,953090093	9,01E-01	3,43E-01	6,43E-01	Ube2e3	0,95	No
ENSMUSG00000027012	2,84E-02	6,727389407	2,13E-01	6,44E-01	8,73E-01	Dync1i2	1,02	No

ENSMUSG00000027014	4,57E-02	3,83331036	2,49E-01	6,18E-01	8,55E-01	Cwc22	1,03	No
ENSMUSG00000027015	-1,30E+00	-2,16571753	2,91E+00	8,82E-02	3,79E-01	Cybrd1	0,40	No
ENSMUSG00000027018	-8,78E-02	5,424010838	1,59E+00	2,07E-01	5,50E-01	Hat1	0,94	No
ENSMUSG00000027030	-1,49E-01	5,013706171	4,11E+00	4,27E-02	2,46E-01	Stk39	0,90	No
ENSMUSG00000027035	-1,35E-01	7,252967384	5,04E+00	2,48E-02	1,74E-01	Cers6	0,91	No
ENSMUSG00000027048	-5,72E-01	-2,548220847	2,56E-01	6,13E-01	8,53E-01	Abcb11	0,67	No
ENSMUSG00000027067	4,50E-02	7,258159697	5,64E-01	4,53E-01	7,42E-01	Ssrp1	1,03	No
ENSMUSG00000027068	4,88E-01	6,341475769	5,87E+01	1,87E-14	2,70E-12	Dhrs9	1,40	Yes
ENSMUSG00000027070	1,12E+00	-2,711765755	1,20E+00	2,74E-01	5,70E-01	Lrp2	2,18	No
ENSMUSG00000027071	-1,11E-02	0,448509577	2,15E-03	9,63E-01	1,00E+00	P2rx3	0,99	No
ENSMUSG00000027072	-2,55E+00	-2,226305156	9,41E+00	2,16E-03	2,98E-02	Prg3	0,17	Yes
ENSMUSG00000027073	-1,99E+00	1,392410197	1,16E+02	5,78E-27	2,44E-24	Prg2	0,25	Yes
ENSMUSG00000027074	3,89E-02	5,227320991	2,95E-01	5,87E-01	8,40E-01	Slc43a3	1,03	No
ENSMUSG00000027075	4,53E-01	-2,655122927	1,60E-01	6,89E-01	8,99E-01	Slc43a1	1,37	No
ENSMUSG00000027076	1,64E-01	3,326665857	2,42E+00	1,20E-01	4,30E-01	Timm10	1,12	No
ENSMUSG00000027078	-3,30E-01	5,158244038	2,10E+01	4,51E-06	1,65E-04	Ube2l6	0,80	Yes
ENSMUSG00000027079	6,36E-02	3,842827876	4,80E-01	4,88E-01	7,75E-01	Clp1	1,05	No
ENSMUSG00000027080	4,51E-02	4,227221836	2,86E-01	5,93E-01	8,42E-01	Med19	1,03	No
ENSMUSG00000027082	1,19E-01	3,530739676	1,43E+00	2,32E-01	5,50E-01	Tfpi	1,09	No
ENSMUSG00000027086	1,31E-01	3,311990166	1,57E+00	2,10E-01	5,50E-01	Fastkd1	1,09	No
ENSMUSG00000027087	1,38E-01	5,747241438	4,21E+00	4,02E-02	2,38E-01	Itgav	1,10	No
ENSMUSG00000027088	1,48E-01	4,319928929	3,20E+00	7,36E-02	3,45E-01	Phospho2	1,11	No
ENSMUSG00000027091	-4,71E-02	5,389786839	4,50E-01	5,03E-01	7,87E-01	Zc3h15	0,97	No
ENSMUSG00000027099	2,64E-02	4,424271891	1,03E-01	7,49E-01	9,30E-01	Mtx2	1,02	No
ENSMUSG00000027104	1,11E-01	5,745876733	2,71E+00	9,97E-02	3,92E-01	Atf2	1,08	No
ENSMUSG00000027107	-3,49E-02	-2,9632767	7,31E-11	1,00E+00	1,00E+00	Chrna1	0,98	No
ENSMUSG00000027108	-5,38E-02	5,213492904	5,62E-01	4,53E-01	7,43E-01	Ola1	0,96	No
ENSMUSG00000027109	-1,42E-04	6,574422248	5,16E-06	9,98E-01	1,00E+00	Sp3	1,00	No
ENSMUSG00000027111	-2,40E-01	5,072729516	1,09E+01	9,66E-04	1,57E-02	Itga6	0,85	Yes
ENSMUSG00000027115	2,23E-01	3,097081122	4,05E+00	4,42E-02	2,50E-01	Kif18a	1,17	No
ENSMUSG00000027122	-1,07E-02	4,37126684	1,70E-02	8,96E-01	1,00E+00	Arl14ep	0,99	No
ENSMUSG00000027130	3,05E-02	6,537359012	2,38E-01	6,25E-01	8,60E-01	Slc12a6	1,02	No

ENSMUSG00000027131	-4,43E-03	5,038764857	3,60E-03	9,52E-01	1,00E+00	Emc4	1,00	No
ENSMUSG00000027132	9,35E-02	4,398538012	1,32E+00	2,51E-01	5,50E-01	Katnbl1	1,07	No
ENSMUSG00000027133	-4,04E-02	4,668546313	2,67E-01	6,05E-01	8,49E-01	Nop10	0,97	No
ENSMUSG00000027134	-1,12E-01	5,261275132	2,48E+00	1,15E-01	4,20E-01	Lpcat4	0,93	No
ENSMUSG00000027160	1,93E-01	3,557098047	3,88E+00	4,89E-02	2,67E-01	Ccdc34	1,14	No
ENSMUSG00000027162	4,07E-02	6,651740824	4,29E-01	5,13E-01	7,95E-01	Lin7c	1,03	No
ENSMUSG00000027163	1,67E-01	4,320564769	4,01E+00	4,51E-02	2,53E-01	Commd9	1,12	No
ENSMUSG00000027164	-3,10E-01	7,151770668	2,64E+01	2,71E-07	1,37E-05	Traf6	0,81	Yes
ENSMUSG00000027165	-1,25E-01	2,733527448	1,07E+00	3,01E-01	5,97E-01	B230118H07Rik	0,92	No
ENSMUSG00000027166	1,62E-01	2,792871293	1,78E+00	1,82E-01	5,45E-01	Dnajc24	1,12	No
ENSMUSG00000027167	7,09E-02	2,568300198	3,04E-01	5,81E-01	8,37E-01	Elp4	1,05	No
ENSMUSG00000027168	8,66E-01	-0,78588827	4,55E+00	3,29E-02	2,12E-01	Pax6	1,82	No
ENSMUSG00000027170	-2,15E-01	3,767744788	5,29E+00	2,14E-02	1,58E-01	Eif3m	0,86	No
ENSMUSG00000027171	-4,97E-01	3,184313367	2,26E+01	1,99E-06	8,09E-05	Prrg4	0,71	Yes
ENSMUSG00000027173	-2,46E-01	3,927186514	7,72E+00	5,45E-03	5,95E-02	Depdc7	0,84	No
ENSMUSG00000027175	7,08E-03	5,912821947	1,16E-02	9,14E-01	1,00E+00	Tcp11l1	1,00	No
ENSMUSG00000027176	6,72E-02	5,038472405	8,49E-01	3,57E-01	6,58E-01	Cstf3	1,05	No
ENSMUSG00000027177	9,19E-03	7,400720532	2,39E-02	8,77E-01	1,00E+00	Hipk3	1,01	No
ENSMUSG00000027180	1,40E-01	5,768286804	4,36E+00	3,68E-02	2,28E-01	Fbxo3	1,10	No
ENSMUSG00000027184	4,18E-02	8,463040067	5,30E-01	4,67E-01	7,55E-01	Caprin1	1,03	No
ENSMUSG00000027185	1,03E-01	4,923000724	1,90E+00	1,68E-01	5,23E-01	Nat10	1,07	No
ENSMUSG00000027186	1,85E+00	-3,107657832	1,35E+00	2,46E-01	5,50E-01	Elf5	3,61	No
ENSMUSG00000027187	-6,25E-02	7,526081091	1,12E+00	2,91E-01	5,86E-01	Cat	0,96	No
ENSMUSG00000027188	-6,90E-02	-1,424765017	1,67E-02	8,97E-01	1,00E+00	Pamr1	0,95	No
ENSMUSG00000027189	-5,60E-04	6,682486474	8,20E-05	9,93E-01	1,00E+00	Trim44	1,00	No
ENSMUSG00000027193	4,90E-02	7,319347739	6,73E-01	4,12E-01	7,08E-01	Api5	1,03	No
ENSMUSG00000027194	-1,26E-01	5,213920968	3,05E+00	8,08E-02	3,64E-01	Ttc17	0,92	No
ENSMUSG00000027195	1,74E-01	6,582470686	7,77E+00	5,31E-03	5,84E-02	Hsd17b12	1,13	No
ENSMUSG00000027196	-7,77E-02	0,156069045	7,95E-02	7,78E-01	9,47E-01	Alkbh3os1	0,95	No
ENSMUSG00000027198	-4,01E-02	5,836875367	3,61E-01	5,48E-01	8,15E-01	Ext2	0,97	No
ENSMUSG00000027199	3,91E-01	5,732612667	3,35E+01	7,08E-09	5,04E-07	Gatm	1,31	Yes
ENSMUSG00000027200	-3,83E-02	7,847770662	4,28E-01	5,13E-01	7,95E-01	Sema6d	0,97	No

ENSMUSG00000027201	9,47E-03	3,87725915	1,10E-02	9,16E-01	1,00E+00	Myef2	1,01	No
ENSMUSG00000027202	3,14E-02	-3,107617333	-7,69E-10	1,00E+00	1,00E+00	Slc12a1	1,02	No
ENSMUSG00000027203	1,40E-02	4,443297575	2,96E-02	8,63E-01	9,97E-01	Dut	1,01	No
ENSMUSG00000027204	-3,04E+00	-1,246744336	2,83E+01	1,06E-07	5,90E-06	Fbn1	0,12	Yes
ENSMUSG00000027206	-1,04E-01	6,124420825	2,59E+00	1,07E-01	4,01E-01	Cops2	0,93	No
ENSMUSG00000027207	5,28E-02	5,852890289	6,32E-01	4,27E-01	7,21E-01	Galk2	1,04	No
ENSMUSG00000027208	-2,86E+00	-2,226151693	7,91E+00	4,90E-03	5,52E-02	Fgf7	0,14	No
ENSMUSG00000027209	2,97E-01	0,607390615	1,57E+00	2,10E-01	5,50E-01	Fam227b	1,23	No
ENSMUSG00000027210	3,14E-02	-3,107617333	-7,69E-10	1,00E+00	1,00E+00	Meis2	1,02	No
ENSMUSG00000027215	5,45E-02	6,159983587	7,13E-01	3,98E-01	6,98E-01	Cd82	1,04	No
ENSMUSG00000027217	-4,45E-01	1,38631987	5,74E+00	1,66E-02	1,34E-01	Tspan18	0,73	No
ENSMUSG00000027219	3,10E-02	1,678139404	3,35E-02	8,55E-01	9,92E-01	Slc28a2	1,02	No
ENSMUSG00000027220	-2,24E+00	-2,35552396	4,08E+00	4,34E-02	2,48E-01	Syt13	0,21	No
ENSMUSG00000027221	-1,85E-01	-0,963078844	2,07E-01	6,49E-01	8,75E-01	Chst1	0,88	No
ENSMUSG00000027222	-1,10E-01	3,544077802	1,22E+00	2,70E-01	5,66E-01	Pex16	0,93	No
ENSMUSG00000027223	-4,96E-01	-1,146021642	1,10E+00	2,95E-01	5,91E-01	Mapk8ip1	0,71	No
ENSMUSG00000027224	-1,94E+00	-3,107619243	1,50E+00	2,21E-01	5,50E-01	Duoxa1	0,26	No
ENSMUSG00000027227	1,06E-02	4,901126994	1,99E-02	8,88E-01	1,00E+00	Sord	1,01	No
ENSMUSG00000027230	6,65E-02	2,744478231	3,01E-01	5,83E-01	8,38E-01	Creb3l1	1,05	No
ENSMUSG00000027233	-2,12E-01	-1,655098213	1,21E-01	7,28E-01	9,20E-01	Patl2	0,86	No
ENSMUSG00000027236	1,07E-01	5,392308729	2,34E+00	1,26E-01	4,44E-01	Eif3j1	1,08	No
ENSMUSG00000027238	-2,95E-01	1,240381741	2,65E+00	1,03E-01	3,95E-01	Frmd5	0,82	No
ENSMUSG00000027239	3,97E-01	0,8726752	3,12E+00	7,74E-02	3,55E-01	Mdk	1,32	No
ENSMUSG00000027242	1,97E-01	4,507886294	6,12E+00	1,34E-02	1,16E-01	Wdr76	1,15	No
ENSMUSG00000027243	-1,54E-01	1,924938678	1,06E+00	3,02E-01	5,99E-01	Harbi1	0,90	No
ENSMUSG00000027244	-3,10E-02	5,483991908	2,01E-01	6,54E-01	8,79E-01	Atg13	0,98	No
ENSMUSG00000027245	-5,92E-02	4,399117203	5,27E-01	4,68E-01	7,56E-01	Hypk	0,96	No
ENSMUSG00000027246	1,74E-01	-1,726139732	8,81E-02	7,67E-01	9,40E-01	Ell3	1,13	No
ENSMUSG00000027247	-7,41E-02	6,690476586	1,44E+00	2,31E-01	5,50E-01	Arhgap1	0,95	No
ENSMUSG00000027248	1,01E-01	9,010849956	3,14E+00	7,65E-02	3,52E-01	Pdia3	1,07	No
ENSMUSG00000027249	-2,26E+00	-2,497628959	4,14E+00	4,18E-02	2,43E-01	F2	0,21	No
ENSMUSG00000027253	-1,53E-01	3,88782587	2,84E+00	9,19E-02	3,82E-01	Lrp4	0,90	No

ENSMUSG00000027254	2,13E-01	-0,024683118	5,03E-01	4,78E-01	7,66E-01	Map1a	1,16	No
ENSMUSG00000027255	1,17E-02	5,484865092	2,85E-02	8,66E-01	9,99E-01	Arfgap2	1,01	No
ENSMUSG00000027257	6,07E-01	-1,627495067	9,60E-01	3,27E-01	6,28E-01	Pacsin3	1,52	No
ENSMUSG00000027259	2,82E-01	2,510519358	4,81E+00	2,83E-02	1,90E-01	Adal	1,22	No
ENSMUSG00000027261	-6,87E-02	-2,600672294	4,30E-03	9,48E-01	1,00E+00	Hao1	0,95	No
ENSMUSG00000027263	-1,42E-01	5,599342424	4,37E+00	3,67E-02	2,28E-01	Tubgcp4	0,91	No
ENSMUSG00000027272	3,43E-02	5,08785886	2,22E-01	6,38E-01	8,68E-01	Ubr1	1,02	No
ENSMUSG00000027273	1,87E+00	-3,107639691	1,38E+00	2,40E-01	5,50E-01	Snap25	3,67	No
ENSMUSG00000027274	1,45E-01	4,259034878	3,01E+00	8,29E-02	3,70E-01	Mkks	1,11	No
ENSMUSG00000027276	3,34E-02	2,890299172	8,76E-02	7,67E-01	9,40E-01	Jag1	1,02	No
ENSMUSG00000027281	1,68E-01	3,669965252	3,11E+00	7,76E-02	3,56E-01	Slx4ip	1,12	No
ENSMUSG00000027282	-1,32E-02	5,629931244	3,72E-02	8,47E-01	9,87E-01	Mtch2	0,99	No
ENSMUSG00000027284	1,89E-01	4,959582702	6,54E+00	1,06E-02	9,72E-02	Cdan1	1,14	No
ENSMUSG00000027285	-3,66E-02	4,526465218	2,06E-01	6,50E-01	8,76E-01	Haus2	0,97	No
ENSMUSG00000027286	1,65E-01	3,901178886	3,27E+00	7,07E-02	3,36E-01	Lrrc57	1,12	No
ENSMUSG00000027287	-9,08E-03	7,106149179	2,27E-02	8,80E-01	1,00E+00	Snap23	0,99	No
ENSMUSG00000027288	-3,75E-02	8,527986436	4,26E-01	5,14E-01	7,96E-01	Zfp106	0,97	No
ENSMUSG00000027291	-1,02E-01	6,459127376	2,67E+00	1,03E-01	3,95E-01	Vps39	0,93	No
ENSMUSG00000027293	1,73E-01	7,896033306	8,71E+00	3,17E-03	4,00E-02	Ehd4	1,13	Yes
ENSMUSG00000027296	4,00E-01	0,966236693	3,51E+00	6,09E-02	3,06E-01	Itpka	1,32	No
ENSMUSG00000027297	-9,56E-01	-1,963179741	2,25E+00	1,34E-01	4,60E-01	Ltk	0,52	No
ENSMUSG00000027298	4,09E-01	4,601479147	2,66E+01	2,56E-07	1,30E-05	Tyro3	1,33	Yes
ENSMUSG00000027300	-8,74E-03	3,572544848	8,29E-03	9,27E-01	1,00E+00	Ubox5	0,99	No
ENSMUSG00000027301	-3,88E-03	-3,03362684	4,02E-12	1,00E+00	1,00E+00	Oxt	1,00	No
ENSMUSG00000027303	1,77E-01	6,260120603	7,66E+00	5,66E-03	6,12E-02	Ptpra	1,13	No
ENSMUSG00000027304	-6,58E-03	5,978883913	1,01E-02	9,20E-01	1,00E+00	Rtf1	1,00	No
ENSMUSG00000027305	-2,32E-02	3,442157735	5,35E-02	8,17E-01	9,70E-01	Ndufaf1	0,98	No
ENSMUSG00000027306	1,89E-01	5,721907035	7,85E+00	5,07E-03	5,65E-02	Nusap1	1,14	No
ENSMUSG00000027309	8,33E-02	5,470708293	1,43E+00	2,31E-01	5,50E-01	4930402H24Rik	1,06	No
ENSMUSG00000027312	1,52E-01	6,05979848	5,44E+00	1,96E-02	1,49E-01	Atrn	1,11	No
ENSMUSG00000027313	1,79E-01	0,804474162	7,06E-01	4,01E-01	6,99E-01	Chac1	1,13	No
ENSMUSG00000027314	-7,93E-01	-0,682982517	3,18E+00	7,45E-02	3,47E-01	Dll4	0,58	No

ENSMUSG00000027315	8,57E-02	8,758587484	2,25E+00	1,34E-01	4,61E-01	Spint1	1,06	No
ENSMUSG00000027316	-4,97E-01	-1,366835406	9,01E-01	3,42E-01	6,43E-01	Gfra4	0,71	No
ENSMUSG00000027317	-8,83E-02	-1,711729117	2,18E-02	8,83E-01	1,00E+00	Ppp1r14d	0,94	No
ENSMUSG00000027318	7,98E-01	0,399072552	8,62E+00	3,33E-03	4,14E-02	Adam33	1,74	Yes
ENSMUSG00000027322	1,69E-01	3,978415396	3,49E+00	6,17E-02	3,09E-01	Siglec1	1,12	No
ENSMUSG00000027323	1,31E-01	4,873747456	2,98E+00	8,41E-02	3,72E-01	Rad51	1,09	No
ENSMUSG00000027324	1,14E-02	2,355346146	6,95E-03	9,34E-01	1,00E+00	Rpusd2	1,01	No
ENSMUSG00000027326	1,48E-01	4,946822014	3,97E+00	4,63E-02	2,57E-01	Knl1	1,11	No
ENSMUSG00000027327	7,34E-02	4,455544836	8,02E-01	3,70E-01	6,72E-01	1700037H04Rik	1,05	No
ENSMUSG00000027329	2,73E-01	2,274323633	3,86E+00	4,96E-02	2,69E-01	Spef1	1,21	No
ENSMUSG00000027330	2,14E-01	5,363836011	9,20E+00	2,42E-03	3,26E-02	Cdc25b	1,16	Yes
ENSMUSG00000027331	2,42E-01	4,720884279	9,89E+00	1,66E-03	2,41E-02	Knstrn	1,18	Yes
ENSMUSG00000027332	-7,65E-02	6,524557436	1,49E+00	2,22E-01	5,50E-01	Ivd	0,95	No
ENSMUSG00000027333	-2,99E-01	6,185911007	2,13E+01	3,89E-06	1,46E-04	Smox	0,81	Yes
ENSMUSG00000027335	-1,10E+00	-2,863660892	7,07E-01	4,01E-01	6,99E-01	Adra1d	0,47	No
ENSMUSG00000027338	7,92E-01	-2,627655899	4,42E-01	5,06E-01	7,91E-01	Prnd	1,73	No
ENSMUSG00000027339	-3,69E-01	6,918031177	3,70E+01	1,19E-09	9,63E-08	Rassf2	0,77	Yes
ENSMUSG00000027340	1,98E-01	7,62019502	1,12E+01	8,13E-04	1,36E-02	Slc23a2	1,15	Yes
ENSMUSG00000027341	5,81E-02	4,769655959	5,67E-01	4,51E-01	7,41E-01	Tmem230	1,04	No
ENSMUSG00000027342	5,67E-02	6,897254084	8,59E-01	3,54E-01	6,55E-01	Pcna	1,04	No
ENSMUSG00000027344	-3,59E+00	-2,800973446	5,80E+00	1,60E-02	1,32E-01	Fsip1	0,08	No
ENSMUSG00000027346	-1,47E-01	6,623564885	5,63E+00	1,76E-02	1,39E-01	Gpcpd1	0,90	No
ENSMUSG00000027347	2,48E-01	6,550066263	1,57E+01	7,33E-05	1,87E-03	Rasgrp1	1,19	Yes
ENSMUSG00000027349	7,41E-02	5,070497328	1,03E+00	3,11E-01	6,10E-01	Fam98b	1,05	No
ENSMUSG00000027350	3,16E+00	-2,770597723	4,06E+00	4,40E-02	2,50E-01	Chgb	8,91	No
ENSMUSG00000027351	-1,92E-01	7,035943078	1,00E+01	1,57E-03	2,31E-02	Spred1	0,88	Yes
ENSMUSG00000027353	1,65E-01	2,91307903	2,03E+00	1,54E-01	4,98E-01	Mcm8	1,12	No
ENSMUSG00000027355	-2,66E+00	-3,033639428	2,81E+00	9,35E-02	3,83E-01	Tmco5	0,16	No
ENSMUSG00000027356	-1,86E+00	-3,033612668	1,39E+00	2,38E-01	5,50E-01	Fermt1	0,28	No
ENSMUSG00000027357	1,63E-01	3,918983656	3,25E+00	7,13E-02	3,38E-01	Crls1	1,12	No
ENSMUSG00000027358	-1,76E+00	-0,668894353	1,93E+01	1,13E-05	3,76E-04	Bmp2	0,29	Yes
ENSMUSG00000027359	-1,48E+00	-2,289477248	2,27E+00	1,32E-01	4,56E-01	Slc27a2	0,36	No

ENSMUSG00000027360	-5,31E-01	0,070731001	3,34E+00	6,76E-02	3,28E-01	Hdc	0,69	No
ENSMUSG00000027361	-1,49E-01	4,56558915	3,52E+00	6,05E-02	3,05E-01	Gabpb1	0,90	No
ENSMUSG00000027363	2,37E-02	7,239666449	1,57E-01	6,92E-01	9,00E-01	Usp8	1,02	No
ENSMUSG00000027364	9,99E-02	3,783450753	1,14E+00	2,85E-01	5,82E-01	Usp50	1,07	No
ENSMUSG00000027365	6,99E-03	7,097137564	1,34E-02	9,08E-01	1,00E+00	Trpm7	1,00	No
ENSMUSG00000027366	5,68E-02	7,68904505	9,33E-01	3,34E-01	6,35E-01	Sppl2a	1,04	No
ENSMUSG00000027367	-2,60E-01	6,883970593	1,82E+01	1,98E-05	6,15E-04	Stard7	0,83	Yes
ENSMUSG00000027368	-2,33E-01	0,621967028	9,94E-01	3,19E-01	6,19E-01	Dusp2	0,85	No
ENSMUSG00000027371	5,95E-02	3,821231848	4,16E-01	5,19E-01	7,98E-01	Fahd2a	1,04	No
ENSMUSG00000027374	1,57E-01	3,505673298	2,50E+00	1,14E-01	4,16E-01	Mrps5	1,12	No
ENSMUSG00000027376	-1,41E-01	-0,50994938	1,75E-01	6,76E-01	8,91E-01	Prom2	0,91	No
ENSMUSG00000027377	-8,92E-01	0,715998142	1,63E+01	5,31E-05	1,43E-03	Mall	0,54	Yes
ENSMUSG00000027378	1,30E-01	1,990790649	7,36E-01	3,91E-01	6,91E-01	Nphp1	1,09	No
ENSMUSG00000027379	2,36E-01	5,290195166	1,10E+01	8,96E-04	1,48E-02	Bub1	1,18	Yes
ENSMUSG00000027380	1,38E-01	-1,205818281	9,05E-02	7,64E-01	9,39E-01	Acoxl	1,10	No
ENSMUSG00000027381	1,67E-01	6,497591718	7,11E+00	7,66E-03	7,66E-02	Bcl2l11	1,12	No
ENSMUSG00000027384	-2,38E-02	2,327561897	3,12E-02	8,60E-01	9,95E-01	Ndufaf5	0,98	No
ENSMUSG00000027387	2,47E-01	1,652186108	2,27E+00	1,32E-01	4,56E-01	Zc3h8	1,19	No
ENSMUSG00000027394	1,62E-01	4,402981134	3,94E+00	4,70E-02	2,60E-01	Ttl	1,12	No
ENSMUSG00000027395	2,15E-01	4,349587151	6,70E+00	9,65E-03	9,08E-02	Polr1b	1,16	No
ENSMUSG00000027397	-1,93E-01	7,905162795	1,08E+01	9,89E-04	1,60E-02	Slc20a1	0,87	Yes
ENSMUSG00000027398	-6,90E-01	4,943053069	7,95E+01	4,84E-19	1,04E-16	Il1b	0,62	Yes
ENSMUSG00000027399	-6,55E-01	6,401777657	1,04E+02	2,30E-24	7,67E-22	Il1a	0,64	Yes
ENSMUSG00000027400	-1,27E+00	-2,65510207	1,54E+00	2,15E-01	5,50E-01	Pdyn	0,42	No
ENSMUSG00000027401	1,87E+00	-3,107639691	1,38E+00	2,40E-01	5,50E-01	Tgm3	3,67	No
ENSMUSG00000027404	3,94E-02	7,025570686	4,22E-01	5,16E-01	7,97E-01	Snrpb	1,03	No
ENSMUSG00000027405	1,63E-02	5,842317951	5,99E-02	8,07E-01	9,64E-01	Nop56	1,01	No
ENSMUSG00000027406	-6,72E-02	6,436188105	1,14E+00	2,86E-01	5,82E-01	Idh3b	0,95	No
ENSMUSG00000027408	-5,61E-01	-0,344073936	2,43E+00	1,19E-01	4,30E-01	Cpxm1	0,68	No
ENSMUSG00000027409	7,19E-01	-2,033576791	8,37E-01	3,60E-01	6,62E-01	1700020A23Rik	1,65	No
ENSMUSG00000027411	-9,53E-02	5,543533114	1,91E+00	1,67E-01	5,20E-01	Vps16	0,94	No
ENSMUSG00000027412	-8,50E-01	-1,070122133	4,16E+00	4,13E-02	2,41E-01	Lpin3	0,55	No

ENSMUSG00000027416	1,82E+00	-3,033667329	1,31E+00	2,52E-01	5,50E-01	Otor	3,54	No
ENSMUSG00000027419	-1,13E+00	-2,929298189	7,47E-01	3,88E-01	6,88E-01	Pcsk2	0,46	No
ENSMUSG00000027420	3,05E-01	1,536508754	2,86E+00	9,11E-02	3,82E-01	Bfsp1	1,24	No
ENSMUSG00000027422	1,70E-02	8,11365715	8,56E-02	7,70E-01	9,42E-01	Rrbp1	1,01	No
ENSMUSG00000027423	1,18E-01	8,458870361	4,22E+00	3,99E-02	2,37E-01	Snx5	1,09	No
ENSMUSG00000027424	3,23E-01	3,315340132	9,93E+00	1,63E-03	2,38E-02	Mgme1	1,25	Yes
ENSMUSG00000027425	1,93E-01	5,515065206	7,84E+00	5,11E-03	5,67E-02	Kat14	1,14	No
ENSMUSG00000027427	-3,31E-02	4,881712713	1,89E-01	6,64E-01	8,84E-01	Polr3f	0,98	No
ENSMUSG00000027428	-2,44E-01	2,768284314	4,18E+00	4,09E-02	2,40E-01	Rbbp9	0,84	No
ENSMUSG00000027429	7,51E-02	5,875858905	1,28E+00	2,59E-01	5,51E-01	Sec23b	1,05	No
ENSMUSG00000027430	3,04E-01	1,395642369	2,72E+00	9,93E-02	3,92E-01	Dtd1	1,23	No
ENSMUSG00000027433	-6,59E-02	6,412208076	1,09E+00	2,97E-01	5,92E-01	Xrn2	0,96	No
ENSMUSG00000027435	6,92E-02	8,469414508	1,44E+00	2,29E-01	5,50E-01	Cd93	1,05	No
ENSMUSG00000027438	8,70E-03	1,811828177	3,08E-03	9,56E-01	1,00E+00	Napb	1,01	No
ENSMUSG00000027439	6,25E-02	4,959440324	7,11E-01	3,99E-01	6,98E-01	Gzf1	1,04	No
ENSMUSG00000027446	-8,66E-01	-2,770614961	4,10E-01	5,22E-01	8,00E-01	9230104L09Rik	0,55	No
ENSMUSG00000027447	-3,56E-01	11,38858294	4,07E+01	1,79E-10	1,63E-08	Cst3	0,78	Yes
ENSMUSG00000027452	3,63E-01	4,802256292	2,24E+01	2,22E-06	8,89E-05	Acss1	1,29	Yes
ENSMUSG00000027454	1,94E-01	2,535826061	2,28E+00	1,31E-01	4,56E-01	Gins1	1,14	No
ENSMUSG00000027455	-1,85E-01	4,955859525	6,24E+00	1,25E-02	1,10E-01	Nsfl1c	0,88	No
ENSMUSG00000027456	-1,15E-01	1,026333569	3,09E-01	5,78E-01	8,35E-01	Sdcbp2	0,92	No
ENSMUSG00000027457	1,40E-01	1,956038599	8,31E-01	3,62E-01	6,63E-01	Snph	1,10	No
ENSMUSG00000027459	3,69E-02	3,82376673	1,65E-01	6,85E-01	8,97E-01	Fam110a	1,03	No
ENSMUSG00000027460	-1,16E+00	-1,711682783	2,63E+00	1,05E-01	3,97E-01	Angpt4	0,45	No
ENSMUSG00000027463	7,56E-02	5,331296576	1,15E+00	2,84E-01	5,81E-01	Slc52a3	1,05	No
ENSMUSG00000027465	-3,94E-02	7,311085926	4,35E-01	5,10E-01	7,93E-01	Tbc1d20	0,97	No
ENSMUSG00000027466	1,86E-02	6,886351778	9,25E-02	7,61E-01	9,37E-01	Rbck1	1,01	No
ENSMUSG00000027469	1,94E-01	6,087311014	9,00E+00	2,70E-03	3,53E-02	Tpx2	1,14	Yes
ENSMUSG00000027470	-6,84E-02	-2,711771061	4,27E-03	9,48E-01	1,00E+00	Mylk2	0,95	No
ENSMUSG00000027472	2,22E-02	4,603566172	8,16E-02	7,75E-01	9,45E-01	Pdrg1	1,02	No
ENSMUSG00000027474	1,52E-01	-1,740955407	6,98E-02	7,92E-01	9,55E-01	Ccm2l	1,11	No
ENSMUSG00000027475	-7,42E-02	7,504206157	1,57E+00	2,10E-01	5,50E-01	Kif3b	0,95	No

ENSMUSG00000027478	3,92E-01	1,723294768	5,50E+00	1,90E-02	1,46E-01	Dnmt3b	1,31	No
ENSMUSG00000027479	-6,15E-02	7,663190126	1,09E+00	2,96E-01	5,92E-01	Mapre1	0,96	No
ENSMUSG00000027487	4,98E-02	2,532641267	1,60E-01	6,90E-01	8,99E-01	Cdk5rap1	1,04	No
ENSMUSG00000027488	4,65E-02	1,369285458	6,33E-02	8,01E-01	9,61E-01	Snta1	1,03	No
ENSMUSG00000027489	2,73E-01	-0,14617134	7,01E-01	4,02E-01	7,01E-01	Necab3	1,21	No
ENSMUSG00000027490	1,99E-01	5,462331221	8,20E+00	4,19E-03	4,90E-02	E2f1	1,15	Yes
ENSMUSG00000027495	-2,05E-01	4,370280718	6,23E+00	1,26E-02	1,11E-01	Fam210b	0,87	No
ENSMUSG00000027496	1,36E-01	5,116270477	3,47E+00	6,23E-02	3,11E-01	Aurka	1,10	No
ENSMUSG00000027498	9,18E-02	5,079263256	1,58E+00	2,08E-01	5,50E-01	Cstf1	1,07	No
ENSMUSG00000027499	-2,66E+00	-2,831990267	2,82E+00	9,31E-02	3,83E-01	Pkia	0,16	No
ENSMUSG00000027500	1,85E+00	-3,107657832	1,35E+00	2,46E-01	5,50E-01	Stmn2	3,61	No
ENSMUSG00000027502	-3,30E-02	6,790452229	2,88E-01	5,91E-01	8,42E-01	Rtfdc1	0,98	No
ENSMUSG00000027505	-2,69E-01	1,47807563	2,10E+00	1,47E-01	4,85E-01	Fam209	0,83	No
ENSMUSG00000027506	6,59E-02	6,516551332	1,10E+00	2,94E-01	5,89E-01	Tpd52	1,05	No
ENSMUSG00000027508	3,32E-01	6,231582342	2,65E+01	2,65E-07	1,34E-05	Pag1	1,26	Yes
ENSMUSG00000027509	-3,53E-02	4,444481125	1,90E-01	6,63E-01	8,83E-01	Rae1	0,98	No
ENSMUSG00000027510	-2,46E-01	3,680099112	6,59E+00	1,03E-02	9,52E-02	Rbm38	0,84	No
ENSMUSG00000027514	-3,01E-02	4,802070914	1,56E-01	6,93E-01	9,01E-01	Zbp1	0,98	No
ENSMUSG00000027519	1,61E-02	6,061827058	6,19E-02	8,04E-01	9,62E-01	Rab22a	1,01	No
ENSMUSG00000027520	-5,88E-02	-3,033616507	1,62E-03	9,68E-01	1,00E+00	Zdbf2	0,96	No
ENSMUSG00000027522	9,92E-03	6,494722889	2,52E-02	8,74E-01	1,00E+00	Stx16	1,01	No
ENSMUSG00000027523	-6,14E-02	8,035653923	1,12E+00	2,91E-01	5,86E-01	Gnas	0,96	No
ENSMUSG00000027524	-2,70E+00	-2,963266517	2,89E+00	8,90E-02	3,79E-01	Edn3	0,15	No
ENSMUSG00000027525	-4,61E-02	-2,832015797	1,00E-03	9,75E-01	1,00E+00	Phactr3	0,97	No
ENSMUSG00000027528	-6,80E-01	-2,998019532	1,86E-01	6,66E-01	8,86E-01	Fabp9	0,62	No
ENSMUSG00000027530	-5,28E-02	-2,711731759	1,90E-03	9,65E-01	1,00E+00	Fabp12	0,96	No
ENSMUSG00000027531	3,48E-02	5,719811043	2,67E-01	6,05E-01	8,49E-01	Impa1	1,02	No
ENSMUSG00000027533	-2,11E-01	8,036630699	1,32E+01	2,78E-04	5,70E-03	Fabp5	0,86	Yes
ENSMUSG00000027534	-1,38E-01	2,645132053	1,27E+00	2,59E-01	5,52E-01	Snx16	0,91	No
ENSMUSG00000027536	-4,51E-01	1,367309193	6,37E+00	1,16E-02	1,04E-01	Chmp4c	0,73	No
ENSMUSG00000027540	-1,26E-02	9,056377253	4,89E-02	8,25E-01	9,74E-01	Ptpn1	0,99	No
ENSMUSG00000027544	1,83E-01	6,582212867	8,61E+00	3,34E-03	4,15E-02	Nfatc2	1,14	Yes

ENSMUSG00000027546	8,06E-02	0,906314606	1,36E-01	7,12E-01	9,11E-01	Atp9a	1,06	No
ENSMUSG00000027547	3,14E-02	-3,107617333	-7,69E-10	1,00E+00	1,00E+00	Sall4	1,02	No
ENSMUSG00000027550	6,19E-02	5,16656317	7,43E-01	3,89E-01	6,89E-01	Lrrcc1	1,04	No
ENSMUSG00000027551	7,95E-02	4,198844457	8,65E-01	3,52E-01	6,53E-01	Zfp64	1,06	No
ENSMUSG00000027552	-1,12E-01	3,923647143	1,55E+00	2,13E-01	5,50E-01	E2f5	0,93	No
ENSMUSG00000027555	-8,59E-02	4,898691282	1,32E+00	2,51E-01	5,50E-01	Car13	0,94	No
ENSMUSG00000027559	7,07E-01	-2,963242697	2,70E-01	6,03E-01	8,48E-01	Car3	1,63	No
ENSMUSG00000027562	-1,18E+00	6,163103869	3,28E+02	2,88E-73	4,32E-69	Car2	0,44	Yes
ENSMUSG00000027566	-1,18E-01	6,729144603	3,68E+00	5,51E-02	2,88E-01	Psma7	0,92	No
ENSMUSG00000027568	-1,89E+00	-3,1076441	1,42E+00	2,33E-01	5,50E-01	Ntsr1	0,27	No
ENSMUSG00000027569	5,02E-02	4,463897917	3,83E-01	5,36E-01	8,08E-01	Mrgbp	1,04	No
ENSMUSG00000027570	-2,00E-01	-0,778002108	2,63E-01	6,08E-01	8,51E-01	Col9a3	0,87	No
ENSMUSG00000027573	9,58E-02	5,342876936	1,85E+00	1,74E-01	5,32E-01	Gid8	1,07	No
ENSMUSG00000027574	-1,89E+00	-3,1076441	1,42E+00	2,33E-01	5,50E-01	Nkain4	0,27	No
ENSMUSG00000027575	1,25E-01	5,541344512	3,31E+00	6,87E-02	3,30E-01	Arfgap1	1,09	No
ENSMUSG00000027577	-2,70E+00	-2,896072657	2,90E+00	8,86E-02	3,79E-01	Chrna4	0,15	No
ENSMUSG00000027579	-1,08E+00	2,425476874	6,97E+01	6,76E-17	1,21E-14	Srms	0,47	Yes
ENSMUSG00000027580	-5,50E-01	8,128323802	8,96E+01	2,86E-21	7,32E-19	Helz2	0,68	Yes
ENSMUSG00000027581	-1,74E+00	-2,71166179	2,10E+00	1,47E-01	4,85E-01	Stmn3	0,30	No
ENSMUSG00000027582	-2,11E-01	5,568801895	9,63E+00	1,91E-03	2,70E-02	Zgpat	0,86	Yes
ENSMUSG00000027583	-1,10E-01	5,622073424	2,67E+00	1,02E-01	3,94E-01	Zbtb46	0,93	No
ENSMUSG00000027584	1,81E-01	-2,226292246	4,88E-02	8,25E-01	9,74E-01	Oprl1	1,13	No
ENSMUSG00000027589	-5,18E-02	4,570571984	4,31E-01	5,12E-01	7,94E-01	Pcmtd2	0,96	No
ENSMUSG00000027593	-5,37E-02	7,18532229	7,98E-01	3,72E-01	6,73E-01	Raly	0,96	No
ENSMUSG00000027596	-1,94E-02	2,987739898	2,94E-02	8,64E-01	9,97E-01	a	0,99	No
ENSMUSG00000027597	9,66E-02	4,529427181	1,46E+00	2,28E-01	5,50E-01	Ahcy	1,07	No
ENSMUSG00000027598	-1,81E-01	7,025267356	8,91E+00	2,84E-03	3,68E-02	Itch	0,88	Yes
ENSMUSG00000027599	8,53E-02	5,239497173	1,42E+00	2,33E-01	5,50E-01	Armc1	1,06	No
ENSMUSG00000027601	1,37E-01	4,118813283	2,53E+00	1,12E-01	4,11E-01	Mtfr1	1,10	No
ENSMUSG00000027602	-1,06E-01	3,931948129	1,36E+00	2,43E-01	5,50E-01	Map1lc3a	0,93	No
ENSMUSG00000027603	2,11E-02	-0,002490999	5,15E-03	9,43E-01	1,00E+00	Ggt7	1,01	No
ENSMUSG00000027605	6,92E-02	3,776564005	5,45E-01	4,61E-01	7,49E-01	Acss2	1,05	No

ENSMUSG00000027610	1,28E-01	4,633952636	2,60E+00	1,07E-01	4,00E-01	Gss	1,09	No
ENSMUSG00000027611	1,26E-01	5,056812791	2,94E+00	8,64E-02	3,78E-01	Procr	1,09	No
ENSMUSG00000027612	-3,86E-02	2,930396802	1,13E-01	7,37E-01	9,25E-01	Mmp24	0,97	No
ENSMUSG00000027613	-1,11E-01	6,448881273	3,09E+00	7,88E-02	3,59E-01	Eif6	0,93	No
ENSMUSG00000027615	1,67E-01	6,322438266	6,90E+00	8,62E-03	8,37E-02	Hps3	1,12	No
ENSMUSG00000027618	-2,61E-02	4,898534028	1,20E-01	7,29E-01	9,20E-01	Nfs1	0,98	No
ENSMUSG00000027620	-1,69E-01	8,058166036	8,47E+00	3,62E-03	4,40E-02	Rbm39	0,89	Yes
ENSMUSG00000027624	-8,68E-03	4,104287888	9,64E-03	9,22E-01	1,00E+00	Epb41l1	0,99	No
ENSMUSG00000027628	1,61E-01	4,749164593	4,37E+00	3,66E-02	2,27E-01	Aar2	1,12	No
ENSMUSG00000027630	8,75E-02	6,960180188	2,06E+00	1,51E-01	4,93E-01	Tbl1xr1	1,06	No
ENSMUSG00000027634	4,16E-02	5,043724051	3,20E-01	5,71E-01	8,32E-01	Ndrg3	1,03	No
ENSMUSG00000027635	1,17E-02	3,786760675	1,60E-02	8,99E-01	1,00E+00	Dsn1	1,01	No
ENSMUSG00000027636	2,06E-02	5,084033157	7,84E-02	7,79E-01	9,48E-01	Sla2	1,01	No
ENSMUSG00000027637	3,22E-02	6,150556543	2,48E-01	6,19E-01	8,56E-01	1110008F13Rik	1,02	No
ENSMUSG00000027639	-2,51E-01	8,793331425	1,94E+01	1,08E-05	3,60E-04	Samhd1	0,84	Yes
ENSMUSG00000027641	1,19E-01	5,61234485	3,06E+00	8,03E-02	3,63E-01	Rbl1	1,09	No
ENSMUSG00000027642	1,08E-01	7,936553025	3,44E+00	6,38E-02	3,16E-01	Rpn2	1,08	No
ENSMUSG00000027646	-2,77E-01	4,832795469	1,34E+01	2,56E-04	5,34E-03	Src	0,83	Yes
ENSMUSG00000027649	-1,24E-02	4,485512916	2,35E-02	8,78E-01	1,00E+00	Ctnnbl1	0,99	No
ENSMUSG00000027650	4,23E-02	4,941878977	3,24E-01	5,69E-01	8,30E-01	Tti1	1,03	No
ENSMUSG00000027651	5,45E-02	5,014605859	5,47E-01	4,60E-01	7,49E-01	Rprd1b	1,04	No
ENSMUSG00000027652	-7,08E-02	6,873001848	1,34E+00	2,47E-01	5,50E-01	Ralgapb	0,95	No
ENSMUSG00000027654	3,71E-01	3,361755987	1,31E+01	3,00E-04	6,06E-03	Fam83d	1,29	Yes
ENSMUSG00000027655	5,80E-02	4,286862809	4,79E-01	4,89E-01	7,76E-01	Dhx35	1,04	No
ENSMUSG00000027656	-1,90E+00	-2,311094963	4,23E+00	3,97E-02	2,36E-01	Wisp2	0,27	No
ENSMUSG00000027660	-2,17E-01	7,244499091	1,31E+01	2,94E-04	5,97E-03	Skil	0,86	Yes
ENSMUSG00000027661	3,14E-02	-3,107617333	-7,69E-10	1,00E+00	1,00E+00	Slc2a10	1,02	No
ENSMUSG00000027663	2,42E-01	6,196172328	1,41E+01	1,78E-04	3,94E-03	Zmat3	1,18	Yes
ENSMUSG00000027665	6,08E-02	6,019126367	8,70E-01	3,51E-01	6,52E-01	Pik3ca	1,04	No
ENSMUSG00000027667	9,12E-03	4,754163379	1,40E-02	9,06E-01	1,00E+00	Zfp639	1,01	No
ENSMUSG00000027668	3,58E-03	5,456142721	2,65E-03	9,59E-01	1,00E+00	Mfn1	1,00	No
ENSMUSG00000027669	-5,31E-01	7,031299057	7,73E+01	1,49E-18	3,03E-16	Gnb4	0,69	Yes

ENSMUSG00000027670	7,64E-01	3,992389014	7,12E+01	3,28E-17	6,22E-15	Ocstamp	1,70	Yes
ENSMUSG00000027671	-8,73E-03	5,009185218	1,38E-02	9,07E-01	1,00E+00	Actl6a	0,99	No
ENSMUSG00000027673	5,07E-03	5,047412995	4,73E-03	9,45E-01	1,00E+00	Ndufb5	1,00	No
ENSMUSG00000027674	1,87E+00	-3,107639691	1,38E+00	2,40E-01	5,50E-01	Pex5l	3,67	No
ENSMUSG00000027676	-2,58E-01	1,651845318	2,32E+00	1,28E-01	4,49E-01	Ccdc39	0,84	No
ENSMUSG00000027677	-6,20E-02	6,072643894	9,14E-01	3,39E-01	6,40E-01	Ttc14	0,96	No
ENSMUSG00000027678	-1,55E-01	7,514707163	6,89E+00	8,67E-03	8,40E-02	Ncoa3	0,90	No
ENSMUSG00000027679	9,93E-02	2,488303927	5,81E-01	4,46E-01	7,37E-01	Dnajc19	1,07	No
ENSMUSG00000027680	-1,42E-01	5,997479882	4,70E+00	3,02E-02	2,00E-01	Fxr1	0,91	No
ENSMUSG00000027684	2,60E+00	-2,963258458	2,63E+00	1,05E-01	3,97E-01	Mecom	6,08	No
ENSMUSG00000027690	-5,55E-01	-2,770636996	2,41E-01	6,23E-01	8,58E-01	Slc2a2	0,68	No
ENSMUSG00000027692	-8,90E-01	1,592646331	2,98E+01	4,86E-08	2,90E-06	Tnik	0,54	Yes
ENSMUSG00000027694	-8,45E-02	5,229782324	1,38E+00	2,40E-01	5,50E-01	Gm8325	0,94	No
ENSMUSG00000027695	9,33E-02	4,542438186	1,36E+00	2,43E-01	5,50E-01	Pld1	1,07	No
ENSMUSG00000027698	1,10E-01	7,42780233	3,41E+00	6,48E-02	3,19E-01	Nceh1	1,08	No
ENSMUSG00000027699	1,32E-01	4,421011922	2,64E+00	1,04E-01	3,97E-01	Ect2	1,10	No
ENSMUSG00000027702	4,86E-01	-2,20582768	3,20E-01	5,71E-01	8,32E-01	Lrrc34	1,40	No
ENSMUSG00000027703	5,97E-03	-3,1461183	-3,40E-10	1,00E+00	1,00E+00	Lrriq4	1,00	No
ENSMUSG00000027706	6,15E-02	6,180244745	9,10E-01	3,40E-01	6,41E-01	Sec62	1,04	No
ENSMUSG00000027708	-9,50E-02	4,97715379	1,65E+00	1,99E-01	5,50E-01	Dcun1d1	0,94	No
ENSMUSG00000027709	1,73E-01	3,925033496	3,70E+00	5,45E-02	2,86E-01	Mccc1	1,13	No
ENSMUSG00000027710	-1,22E-02	4,539546941	2,33E-02	8,79E-01	1,00E+00	Acad9	0,99	No
ENSMUSG00000027712	3,95E-02	7,724869342	4,53E-01	5,01E-01	7,86E-01	Anxa5	1,03	No
ENSMUSG00000027713	1,54E-01	-1,344433674	8,99E-02	7,64E-01	9,39E-01	1810062G17Rik	1,11	No
ENSMUSG00000027714	7,10E-02	4,97599114	9,09E-01	3,40E-01	6,41E-01	Exosc9	1,05	No
ENSMUSG00000027715	2,32E-01	6,423407996	1,35E+01	2,44E-04	5,14E-03	Ccna2	1,17	Yes
ENSMUSG00000027720	-4,13E-01	0,144209802	2,02E+00	1,55E-01	4,99E-01	Il2	0,75	No
ENSMUSG00000027722	2,60E-02	4,393205677	1,01E-01	7,50E-01	9,32E-01	Spata5	1,02	No
ENSMUSG00000027737	6,97E-02	8,177157645	1,44E+00	2,30E-01	5,50E-01	Slc7a11	1,05	No
ENSMUSG00000027739	2,36E-01	3,899049129	6,72E+00	9,52E-03	8,99E-02	Rab33b	1,18	No
ENSMUSG00000027742	5,12E-02	4,561267877	4,18E-01	5,18E-01	7,98E-01	Cog6	1,04	No
ENSMUSG00000027744	-8,32E-01	-2,896126913	3,79E-01	5,38E-01	8,09E-01	Stoml3	0,56	No

ENSMUSG00000027746	3,64E-02	6,129224538	3,15E-01	5,75E-01	8,34E-01	Ufm1	1,03	No
ENSMUSG00000027748	3,82E-01	-1,051942017	8,38E-01	3,60E-01	6,61E-01	Trpc4	1,30	No
ENSMUSG00000027750	6,88E-01	-0,755698986	3,56E+00	5,91E-02	3,01E-01	Postn	1,61	No
ENSMUSG00000027751	-4,63E-02	6,089018252	5,11E-01	4,75E-01	7,63E-01	Supt20	0,97	No
ENSMUSG00000027752	3,16E-02	4,357838462	1,48E-01	7,01E-01	9,05E-01	Exosc8	1,02	No
ENSMUSG00000027763	2,48E-01	9,823875445	1,93E+01	1,11E-05	3,69E-04	Mbnl1	1,19	Yes
ENSMUSG00000027765	2,56E-01	4,542371536	1,02E+01	1,38E-03	2,09E-02	P2ry1	1,19	Yes
ENSMUSG00000027770	-2,87E-02	5,477233484	1,73E-01	6,78E-01	8,92E-01	Dhx36	0,98	No
ENSMUSG00000027774	1,78E-02	5,496218566	6,64E-02	7,97E-01	9,58E-01	Gfm1	1,01	No
ENSMUSG00000027775	9,54E-02	7,311685828	2,54E+00	1,11E-01	4,08E-01	Mfsd1	1,07	No
ENSMUSG00000027776	-7,57E-01	-2,497560684	6,43E-01	4,23E-01	7,18E-01	Il12a	0,59	No
ENSMUSG00000027777	1,27E-01	-1,436619819	5,99E-02	8,07E-01	9,64E-01	Schip1	1,09	No
ENSMUSG00000027778	9,71E-03	3,940215648	1,18E-02	9,13E-01	1,00E+00	Ift80	1,01	No
ENSMUSG00000027782	-1,67E-01	6,972575875	7,48E+00	6,24E-03	6,61E-02	Kpna4	0,89	No
ENSMUSG00000027784	1,21E-02	0,742587538	2,94E-03	9,57E-01	1,00E+00	Ppm1l	1,01	No
ENSMUSG00000027787	1,98E-02	4,548242545	6,08E-02	8,05E-01	9,63E-01	Nmd3	1,01	No
ENSMUSG00000027794	-3,83E-02	-3,107651936	-7,91E-11	1,00E+00	1,00E+00	Sohlh2	0,97	No
ENSMUSG00000027796	1,23E+00	-2,831989638	9,43E-01	3,31E-01	6,32E-01	Smad9	2,35	No
ENSMUSG00000027797	-1,18E+00	-2,333196713	1,79E+00	1,81E-01	5,43E-01	Dclk1	0,44	No
ENSMUSG00000027799	-1,38E+00	-2,770611757	1,20E+00	2,74E-01	5,70E-01	Nbea	0,38	No
ENSMUSG00000027800	-4,33E-01	-2,31111303	1,91E-01	6,62E-01	8,83E-01	Tm4sf1	0,74	No
ENSMUSG00000027803	7,17E-01	-1,998118265	9,17E-01	3,38E-01	6,39E-01	Wwtr1	1,64	No
ENSMUSG00000027804	-1,50E-01	4,778955395	3,83E+00	5,04E-02	2,72E-01	Ppid	0,90	No
ENSMUSG00000027805	-5,72E-01	1,383121305	9,98E+00	1,58E-03	2,32E-02	Pfn2	0,67	Yes
ENSMUSG00000027806	1,33E-01	5,674448674	3,86E+00	4,94E-02	2,68E-01	Tsc22d2	1,10	No
ENSMUSG00000027808	8,56E-02	9,180061038	2,27E+00	1,32E-01	4,56E-01	Serp1	1,06	No
ENSMUSG00000027809	-1,08E-01	5,552365579	2,50E+00	1,14E-01	4,17E-01	Etfdh	0,93	No
ENSMUSG00000027810	3,81E-02	5,445547218	2,99E-01	5,84E-01	8,38E-01	Eif2a	1,03	No
ENSMUSG00000027811	1,17E-01	3,61773273	1,46E+00	2,26E-01	5,50E-01	4930579G24Rik	1,08	No
ENSMUSG00000027820	3,14E-02	-3,107617333	-7,69E-10	1,00E+00	1,00E+00	Mme	1,02	No
ENSMUSG00000027822	-1,66E-01	5,772656948	6,27E+00	1,23E-02	1,09E-01	Slc33a1	0,89	No
ENSMUSG00000027823	-4,75E-02	5,496663382	4,72E-01	4,92E-01	7,78E-01	Gmps	0,97	No

ENSMUSG00000027824	-5,87E-02	-2,896151655	1,54E-03	9,69E-01	1,00E+00	Vmn2r1	0,96	No
ENSMUSG00000027827	2,01E-01	-0,160723665	3,82E-01	5,36E-01	8,08E-01	Kcnab1	1,15	No
ENSMUSG00000027828	8,61E-02	8,60851601	2,26E+00	1,33E-01	4,59E-01	Ssr3	1,06	No
ENSMUSG00000027829	-4,05E-02	7,636548004	4,72E-01	4,92E-01	7,78E-01	Ccnl1	0,97	No
ENSMUSG00000027831	6,12E-01	-2,4729761	5,28E-01	4,67E-01	7,56E-01	Veph1	1,53	No
ENSMUSG00000027832	-4,47E-01	6,394853753	4,86E+01	3,12E-12	3,56E-10	Ptx3	0,73	Yes
ENSMUSG00000027833	8,24E-01	-2,574242326	5,91E-01	4,42E-01	7,34E-01	Shox2	1,77	No
ENSMUSG00000027834	-3,28E-01	-0,655225647	7,62E-01	3,83E-01	6,84E-01	Serpini1	0,80	No
ENSMUSG00000027835	6,69E-03	4,575760519	7,04E-03	9,33E-01	1,00E+00	Pdcd10	1,00	No
ENSMUSG00000027840	2,29E-01	-0,088992726	5,79E-01	4,47E-01	7,37E-01	Wnt2b	1,17	No
ENSMUSG00000027843	1,84E-01	4,849891678	5,79E+00	1,61E-02	1,32E-01	Ptpn22	1,14	No
ENSMUSG00000027845	1,66E-01	4,827579373	4,77E+00	2,89E-02	1,93E-01	Dclre1b	1,12	No
ENSMUSG00000027848	-3,17E-01	0,37219032	1,68E+00	1,95E-01	5,50E-01	Olfml3	0,80	No
ENSMUSG00000027849	-3,85E-02	3,023396011	1,24E-01	7,25E-01	9,18E-01	Syt6	0,97	No
ENSMUSG00000027852	6,85E-02	7,37740752	1,32E+00	2,50E-01	5,50E-01	Nras	1,05	No
ENSMUSG00000027854	1,18E-01	5,495053473	2,92E+00	8,75E-02	3,78E-01	Sike1	1,09	No
ENSMUSG00000027857	8,17E-02	-0,770512854	4,58E-02	8,31E-01	9,77E-01	Tshb	1,06	No
ENSMUSG00000027858	-1,60E-01	4,133207124	3,49E+00	6,17E-02	3,09E-01	Tspan2	0,89	No
ENSMUSG00000027860	3,39E-01	-1,522605679	4,00E-01	5,27E-01	8,04E-01	Vangl1	1,27	No
ENSMUSG00000027863	8,38E-02	0,699480122	1,31E-01	7,18E-01	9,14E-01	Cd2	1,06	No
ENSMUSG00000027864	1,37E-01	4,429207865	2,80E+00	9,43E-02	3,84E-01	Ptgfrn	1,10	No
ENSMUSG00000027865	2,05E-01	5,120870611	7,92E+00	4,89E-03	5,51E-02	Gdap2	1,15	No
ENSMUSG00000027867	3,76E-01	-0,002528603	1,59E+00	2,07E-01	5,50E-01	Spag17	1,30	No
ENSMUSG00000027868	-9,71E-01	-2,35552255	1,14E+00	2,85E-01	5,82E-01	Tbx15	0,51	No
ENSMUSG00000027875	-1,87E+00	-3,107637761	1,39E+00	2,38E-01	5,50E-01	Hmgcs2	0,27	No
ENSMUSG00000027876	-1,87E+00	-3,107637761	1,39E+00	2,38E-01	5,50E-01	Reg4	0,27	No
ENSMUSG00000027878	-1,38E-01	8,555904244	5,82E+00	1,59E-02	1,31E-01	Notch2	0,91	No
ENSMUSG00000027879	1,76E-02	5,81206191	6,93E-02	7,92E-01	9,55E-01	Sec22b	1,01	No
ENSMUSG00000027880	-1,86E+00	-2,963227946	1,40E+00	2,36E-01	5,50E-01	Slc25a54	0,28	No
ENSMUSG00000027881	-1,24E-01	7,610644241	4,42E+00	3,55E-02	2,23E-01	Prpf38b	0,92	No
ENSMUSG00000027882	-2,29E-01	5,712791223	1,16E+01	6,45E-04	1,13E-02	Stxbp3	0,85	Yes
ENSMUSG00000027883	2,97E-01	4,486127121	1,34E+01	2,58E-04	5,36E-03	Gpsm2	1,23	Yes

ENSMUSG00000027884	1,02E-01	5,151048784	1,95E+00	1,62E-01	5,12E-01	Clcc1	1,07	No
ENSMUSG00000027889	-1,43E-01	6,932556397	5,52E+00	1,88E-02	1,45E-01	Ampd2	0,91	No
ENSMUSG00000027890	-1,30E-01	3,619932433	1,80E+00	1,80E-01	5,41E-01	Gstm4	0,91	No
ENSMUSG00000027893	2,94E-02	7,264027407	2,42E-01	6,23E-01	8,58E-01	Ahcyl1	1,02	No
ENSMUSG00000027894	-2,66E+00	-3,033639428	2,81E+00	9,35E-02	3,83E-01	Slc6a17	0,16	No
ENSMUSG00000027896	3,07E-01	-0,117430511	9,91E-01	3,20E-01	6,20E-01	Slc16a4	1,24	No
ENSMUSG00000027900	2,69E-02	6,0750147	1,72E-01	6,79E-01	8,93E-01	Dram2	1,02	No
ENSMUSG00000027901	1,52E-02	3,53402721	2,41E-02	8,77E-01	1,00E+00	Dennd2d	1,01	No
ENSMUSG00000027902	-1,98E-02	-3,033655952	2,83E-11	1,00E+00	1,00E+00	Chil6	0,99	No
ENSMUSG00000027905	-1,29E-02	4,552441464	2,62E-02	8,71E-01	1,00E+00	Ddx20	0,99	No
ENSMUSG00000027907	-4,75E-02	7,844012589	6,60E-01	4,16E-01	7,12E-01	S100a11	0,97	No
ENSMUSG00000027908	2,64E+00	-3,033673223	2,69E+00	1,01E-01	3,92E-01	Tchhl1	6,23	No
ENSMUSG00000027913	9,01E-03	-3,107646452	-5,16E-10	1,00E+00	1,00E+00	Crct1	1,01	No
ENSMUSG00000027931	-6,71E-01	5,570499701	9,72E+01	6,21E-23	1,90E-20	Npr1	0,63	Yes
ENSMUSG00000027932	1,27E-01	5,664908684	3,54E+00	6,00E-02	3,03E-01	Slc27a3	1,09	No
ENSMUSG00000027933	-1,96E-01	6,188379547	9,40E+00	2,17E-03	2,98E-02	Ints3	0,87	Yes
ENSMUSG00000027935	-3,33E-01	3,028597885	9,13E+00	2,52E-03	3,35E-02	Rab13	0,79	Yes
ENSMUSG00000027936	-2,94E-01	6,184814858	2,09E+01	4,72E-06	1,72E-04	Crtc2	0,82	Yes
ENSMUSG00000027937	-1,03E-01	5,5862469	2,25E+00	1,34E-01	4,60E-01	Jtb	0,93	No
ENSMUSG00000027938	-1,15E-01	0,288182316	1,92E-01	6,62E-01	8,83E-01	Creb3l4	0,92	No
ENSMUSG00000027939	-3,91E-01	1,058228097	3,73E+00	5,33E-02	2,82E-01	Nup210l	0,76	No
ENSMUSG00000027940	-2,53E-02	8,965286147	1,97E-01	6,57E-01	8,80E-01	Tpm3	0,98	No
ENSMUSG00000027942	4,41E-02	4,975802937	3,54E-01	5,52E-01	8,18E-01	4933434E20Rik	1,03	No
ENSMUSG00000027944	-1,64E-01	3,850935772	3,26E+00	7,09E-02	3,37E-01	Hax1	0,89	No
ENSMUSG00000027947	3,99E-01	5,722970587	3,48E+01	3,68E-09	2,73E-07	Il6ra	1,32	Yes
ENSMUSG00000027950	-1,83E-01	4,370932081	5,07E+00	2,44E-02	1,72E-01	Chrnb2	0,88	No
ENSMUSG00000027951	-1,28E-01	6,864211476	4,43E+00	3,54E-02	2,23E-01	Adar	0,91	No
ENSMUSG00000027952	-6,96E-04	4,845114889	8,50E-05	9,93E-01	1,00E+00	Pmvk	1,00	No
ENSMUSG00000027953	-9,65E-02	4,545731518	1,45E+00	2,29E-01	5,50E-01	Slc50a1	0,94	No
ENSMUSG00000027954	1,59E+00	-2,548225812	1,74E+00	1,87E-01	5,50E-01	Efna1	3,02	No
ENSMUSG00000027955	4,11E-01	5,910886846	3,80E+01	6,91E-10	5,80E-08	Fam198b	1,33	Yes
ENSMUSG00000027956	2,58E-01	3,50152439	6,66E+00	9,85E-03	9,22E-02	Tmem144	1,20	No

ENSMUSG00000027957	1,04E-01	6,001324078	2,50E+00	1,14E-01	4,16E-01	Slc35a3	1,07	No
ENSMUSG00000027959	1,67E-01	3,565086507	2,91E+00	8,78E-02	3,78E-01	Sass6	1,12	No
ENSMUSG00000027961	-1,16E-01	2,095045995	6,39E-01	4,24E-01	7,18E-01	Lrrc39	0,92	No
ENSMUSG00000027962	-2,72E-01	2,117721777	3,80E+00	5,12E-02	2,75E-01	Vcam1	0,83	No
ENSMUSG00000027963	2,85E-01	3,920445774	9,96E+00	1,60E-03	2,34E-02	Extl2	1,22	Yes
ENSMUSG00000027967	7,06E-01	-2,896175605	2,71E-01	6,03E-01	8,48E-01	Neurog2	1,63	No
ENSMUSG00000027968	1,03E-01	4,278803678	1,46E+00	2,28E-01	5,50E-01	Larp7	1,07	No
ENSMUSG00000027978	-3,09E-02	-2,832023973	6,09E-04	9,80E-01	1,00E+00	Prss12	0,98	No
ENSMUSG00000027981	-7,53E-02	3,386299507	5,31E-01	4,66E-01	7,55E-01	Rnpc3	0,95	No
ENSMUSG00000027983	1,69E-01	-1,548156603	8,97E-02	7,65E-01	9,39E-01	Cyp2u1	1,12	No
ENSMUSG00000027984	7,34E-02	5,05697055	9,98E-01	3,18E-01	6,19E-01	Hadh	1,05	No
ENSMUSG00000027985	-5,23E-01	-0,887856182	1,56E+00	2,11E-01	5,50E-01	Lef1	0,70	No
ENSMUSG00000027993	4,24E-02	3,101941067	1,49E-01	7,00E-01	9,04E-01	Trim2	1,03	No
ENSMUSG00000027994	1,87E-01	6,251343947	8,54E+00	3,48E-03	4,27E-02	Mcub	1,14	Yes
ENSMUSG00000027995	-9,46E-02	8,320286145	2,67E+00	1,02E-01	3,94E-01	Tlr2	0,94	No
ENSMUSG00000027996	-4,49E-01	-2,051763865	3,71E-01	5,43E-01	8,12E-01	Sfrp2	0,73	No
ENSMUSG00000027997	9,05E-02	4,95440584	1,46E+00	2,26E-01	5,50E-01	Casp6	1,06	No
ENSMUSG00000027998	-7,81E-03	4,788208527	1,04E-02	9,19E-01	1,00E+00	Plrg1	0,99	No
ENSMUSG00000027999	-7,02E-02	3,350585342	4,55E-01	5,00E-01	7,85E-01	Pla2g12a	0,95	No
ENSMUSG00000028005	-2,69E+00	-2,896150581	2,87E+00	9,05E-02	3,81E-01	Gucy1b3	0,16	No
ENSMUSG00000028007	-3,02E-02	4,199743184	1,24E-01	7,24E-01	9,18E-01	Snx7	0,98	No
ENSMUSG00000028008	-1,89E+00	-3,1076441	1,42E+00	2,33E-01	5,50E-01	Asic5	0,27	No
ENSMUSG00000028010	-3,08E-02	3,637992247	1,02E-01	7,49E-01	9,31E-01	Gar1	0,98	No
ENSMUSG00000028011	7,60E-01	-1,801045949	1,22E+00	2,69E-01	5,65E-01	Tdo2	1,69	No
ENSMUSG00000028012	-2,24E-01	-0,641280267	3,44E-01	5,58E-01	8,22E-01	Rrh	0,86	No
ENSMUSG00000028013	-5,57E-02	3,592383087	3,31E-01	5,65E-01	8,28E-01	Ppa2	0,96	No
ENSMUSG00000028015	1,11E-01	5,015752718	2,25E+00	1,33E-01	4,60E-01	Ctso	1,08	No
ENSMUSG00000028016	4,42E-02	5,144527172	3,72E-01	5,42E-01	8,11E-01	Ints12	1,03	No
ENSMUSG00000028017	-4,33E-01	1,799288502	7,50E+00	6,18E-03	6,56E-02	Egf	0,74	No
ENSMUSG00000028018	5,29E-02	3,983680727	3,54E-01	5,52E-01	8,18E-01	Gstcd	1,04	No
ENSMUSG00000028019	5,18E-01	3,247250467	2,31E+01	1,54E-06	6,49E-05	Pdgfc	1,43	Yes
ENSMUSG00000028020	3,14E-02	-3,107617333	-7,69E-10	1,00E+00	1,00E+00	Glrb	1,02	No

ENSMUSG00000028023	-1,87E+00	-3,033619006	1,41E+00	2,36E-01	5,50E-01	Pitx2	0,27	No
ENSMUSG00000028024	9,01E-03	-3,107646452	-5,16E-10	1,00E+00	1,00E+00	Enpep	1,01	No
ENSMUSG00000028028	1,26E-01	5,09517082	3,01E+00	8,29E-02	3,70E-01	Alpk1	1,09	No
ENSMUSG00000028029	-1,23E-01	5,080047081	2,84E+00	9,21E-02	3,82E-01	Aimp1	0,92	No
ENSMUSG00000028030	1,62E-01	4,616592584	4,26E+00	3,91E-02	2,35E-01	Tbck	1,12	No
ENSMUSG00000028031	5,79E-01	-2,226295553	5,68E-01	4,51E-01	7,41E-01	Dkk2	1,49	No
ENSMUSG00000028032	1,49E-01	5,364741944	4,48E+00	3,43E-02	2,18E-01	Papss1	1,11	No
ENSMUSG00000028033	-3,58E+00	-2,770569053	5,77E+00	1,63E-02	1,33E-01	Kcnq5	0,08	No
ENSMUSG00000028034	-1,10E-01	6,295493688	2,98E+00	8,41E-02	3,72E-01	Fubp1	0,93	No
ENSMUSG00000028035	8,69E-02	4,146572395	1,01E+00	3,15E-01	6,16E-01	Dnajb4	1,06	No
ENSMUSG00000028036	-8,41E-01	-2,896156011	3,88E-01	5,33E-01	8,06E-01	Ptgfr	0,56	No
ENSMUSG00000028037	-7,50E-01	0,844748347	1,30E+01	3,12E-04	6,26E-03	Ifi44	0,59	Yes
ENSMUSG00000028039	2,99E-01	-2,929300307	5,55E-02	8,14E-01	9,68E-01	Efna3	1,23	No
ENSMUSG00000028040	-6,16E-02	-2,107661822	7,05E-03	9,33E-01	1,00E+00	Efna4	0,96	No
ENSMUSG00000028041	2,36E-01	7,483720553	1,57E+01	7,39E-05	1,88E-03	Adam15	1,18	Yes
ENSMUSG00000028042	1,41E-01	6,659771623	5,22E+00	2,23E-02	1,62E-01	Zbtb7b	1,10	No
ENSMUSG00000028044	1,54E-01	4,787768928	4,01E+00	4,53E-02	2,54E-01	Cks1b	1,11	No
ENSMUSG00000028047	-5,65E-01	-0,580763298	2,42E+00	1,20E-01	4,31E-01	Thbs3	0,68	No
ENSMUSG00000028048	8,52E-02	7,144057202	2,00E+00	1,57E-01	5,03E-01	Gba	1,06	No
ENSMUSG00000028049	8,54E-02	5,66403156	1,58E+00	2,09E-01	5,50E-01	Scamp3	1,06	No
ENSMUSG00000028051	-1,82E-01	3,176614697	2,96E+00	8,54E-02	3,76E-01	Hcn3	0,88	No
ENSMUSG00000028053	-5,29E-02	6,406982382	7,03E-01	4,02E-01	7,00E-01	Ash1l	0,96	No
ENSMUSG00000028057	-5,29E-02	5,335236765	5,62E-01	4,53E-01	7,43E-01	Rit1	0,96	No
ENSMUSG00000028059	-7,86E-02	7,216919062	1,73E+00	1,89E-01	5,50E-01	Arhgef2	0,95	No
ENSMUSG00000028060	-9,09E-02	5,888448217	1,89E+00	1,69E-01	5,24E-01	2810403A07Rik	0,94	No
ENSMUSG00000028062	-1,18E-02	5,03875387	2,55E-02	8,73E-01	1,00E+00	Lamtor2	0,99	No
ENSMUSG00000028063	2,57E-01	6,679333201	1,70E+01	3,75E-05	1,06E-03	Lmna	1,20	Yes
ENSMUSG00000028064	-3,22E-02	9,054095686	3,21E-01	5,71E-01	8,31E-01	Sema4a	0,98	No
ENSMUSG00000028066	4,34E-02	4,405901999	2,74E-01	6,00E-01	8,47E-01	Pmf1	1,03	No
ENSMUSG00000028068	2,84E-01	5,623138552	1,73E+01	3,16E-05	9,17E-04	Iqgap3	1,22	Yes
ENSMUSG00000028069	6,40E-02	3,569338204	4,34E-01	5,10E-01	7,93E-01	Gpatch4	1,05	No
ENSMUSG00000028070	-4,54E-01	5,780550078	4,67E+01	8,37E-12	8,89E-10	Naxe	0,73	Yes

ENSMUSG00000028071	-2,71E+00	-2,896102977	2,90E+00	8,85E-02	3,79E-01	Sh2d2a	0,15	No
ENSMUSG00000028073	-1,27E+00	-2,448638756	1,54E+00	2,14E-01	5,50E-01	Pear1	0,41	No
ENSMUSG00000028076	-4,44E-02	6,39634515	4,93E-01	4,83E-01	7,70E-01	Cd1d1	0,97	No
ENSMUSG00000028078	-1,13E-01	1,259545579	3,63E-01	5,47E-01	8,14E-01	Dclk2	0,92	No
ENSMUSG00000028080	5,31E-02	5,176812699	5,39E-01	4,63E-01	7,51E-01	Lrba	1,04	No
ENSMUSG00000028081	-4,72E-02	6,808136968	5,92E-01	4,42E-01	7,34E-01	Rps3a1	0,97	No
ENSMUSG00000028082	2,13E-03	2,31522618	2,50E-04	9,87E-01	1,00E+00	Sh3d19	1,00	No
ENSMUSG00000028085	6,78E-02	3,395560169	4,45E-01	5,05E-01	7,89E-01	Gatb	1,05	No
ENSMUSG00000028086	1,26E-01	4,98696155	2,84E+00	9,18E-02	3,82E-01	Fbxw7	1,09	No
ENSMUSG00000028088	-7,80E-02	2,88183476	4,54E-01	5,00E-01	7,85E-01	Fmo5	0,95	No
ENSMUSG00000028089	-4,10E-02	3,590667504	1,79E-01	6,72E-01	8,89E-01	Chd1l	0,97	No
ENSMUSG00000028093	2,26E-01	3,365647097	4,87E+00	2,74E-02	1,86E-01	Acp6	1,17	No
ENSMUSG00000028096	1,68E-01	4,673302273	4,61E+00	3,18E-02	2,07E-01	Gpr89	1,12	No
ENSMUSG00000028098	-3,64E-01	6,765663203	3,51E+01	3,11E-09	2,35E-07	Rnf115	0,78	Yes
ENSMUSG00000028099	-2,98E-01	6,35131651	2,23E+01	2,34E-06	9,32E-05	Polr3c	0,81	Yes
ENSMUSG00000028100	-7,86E-01	5,383823033	1,28E+02	1,38E-29	7,68E-27	Nudt17	0,58	Yes
ENSMUSG00000028101	-2,10E-01	4,86377724	7,96E+00	4,78E-03	5,41E-02	Pias3	0,86	No
ENSMUSG00000028102	7,32E-02	3,941635899	6,73E-01	4,12E-01	7,08E-01	Pex11b	1,05	No
ENSMUSG00000028104	-1,99E-02	3,300340668	3,60E-02	8,50E-01	9,89E-01	Polr3gl	0,99	No
ENSMUSG00000028106	-9,48E-02	6,077381517	2,14E+00	1,43E-01	4,79E-01	Rprd2	0,94	No
ENSMUSG00000028107	-2,57E-02	4,922746373	1,19E-01	7,31E-01	9,21E-01	Tars2	0,98	No
ENSMUSG00000028108	8,44E-02	4,999800429	1,27E+00	2,60E-01	5,53E-01	Ecm1	1,06	No
ENSMUSG00000028109	-5,14E-02	-2,600715381	2,98E-03	9,56E-01	1,00E+00	Hormad1	0,96	No
ENSMUSG00000028111	3,99E-01	5,603016693	3,36E+01	6,62E-09	4,77E-07	Ctsk	1,32	Yes
ENSMUSG00000028114	2,17E-01	4,352464956	6,81E+00	9,06E-03	8,68E-02	Mettl14	1,16	No
ENSMUSG00000028115	-4,89E-01	-1,401292542	7,06E-01	4,01E-01	6,99E-01	Bnipl	0,71	No
ENSMUSG00000028121	3,73E-01	6,062085011	3,23E+01	1,31E-08	8,91E-07	Bcar3	1,30	Yes
ENSMUSG00000028124	1,21E-01	7,176341614	4,03E+00	4,47E-02	2,52E-01	Gclm	1,09	No
ENSMUSG00000028125	1,84E+00	-3,033632273	1,33E+00	2,49E-01	5,50E-01	Abca4	3,58	No
ENSMUSG00000028126	1,13E-02	5,363468837	2,60E-02	8,72E-01	1,00E+00	Pip5k1a	1,01	No
ENSMUSG00000028127	1,61E-02	4,827295835	4,54E-02	8,31E-01	9,78E-01	Abcd3	1,01	No
ENSMUSG00000028128	4,97E-02	1,128717337	6,88E-02	7,93E-01	9,56E-01	F3	1,04	No

ENSMUSG00000028132	3,14E-02	-3,107617333	-7,69E-10	1,00E+00	1,00E+00	Tmem56	1,02	No
ENSMUSG00000028133	-2,61E-03	-0,785736984	3,68E-05	9,95E-01	1,00E+00	Rwdd3	1,00	No
ENSMUSG00000028134	-1,22E-01	5,438721151	3,02E+00	8,22E-02	3,68E-01	Ptbp2	0,92	No
ENSMUSG00000028136	9,75E-02	6,47572094	2,40E+00	1,21E-01	4,35E-01	Snx27	1,07	No
ENSMUSG00000028137	2,56E-01	0,616018862	1,12E+00	2,90E-01	5,86E-01	Celf3	1,19	No
ENSMUSG00000028138	-3,08E-02	6,284638295	2,32E-01	6,30E-01	8,62E-01	Adh5	0,98	No
ENSMUSG00000028139	-1,12E-02	-0,96326913	6,57E-04	9,80E-01	1,00E+00	Riiad1	0,99	No
ENSMUSG00000028140	-9,12E-02	5,087887026	1,56E+00	2,12E-01	5,50E-01	Mrpl9	0,94	No
ENSMUSG00000028141	-3,16E-01	1,551457902	3,30E+00	6,93E-02	3,32E-01	Oaz3	0,80	No
ENSMUSG00000028145	2,84E-01	2,249184935	4,27E+00	3,89E-02	2,34E-01	Them4	1,22	No
ENSMUSG00000028148	9,01E-03	-3,107646452	-5,16E-10	1,00E+00	1,00E+00	Them5	1,01	No
ENSMUSG00000028149	6,67E-02	6,97087091	1,20E+00	2,73E-01	5,69E-01	Rap1gds1	1,05	No
ENSMUSG00000028150	-1,90E+00	-3,033647263	1,44E+00	2,30E-01	5,50E-01	Rorc	0,27	No
ENSMUSG00000028152	1,49E-01	6,250784443	5,41E+00	2,00E-02	1,51E-01	Tspan5	1,11	No
ENSMUSG00000028156	1,04E-01	5,522551789	2,26E+00	1,33E-01	4,59E-01	Eif4e	1,07	No
ENSMUSG00000028158	-4,86E-01	0,369074231	3,58E+00	5,86E-02	2,99E-01	Mttp	0,71	No
ENSMUSG00000028159	-2,76E-01	5,408678847	1,59E+01	6,79E-05	1,76E-03	Dapp1	0,83	Yes
ENSMUSG00000028161	-1,85E-01	6,659257148	8,90E+00	2,85E-03	3,68E-02	Ppp3ca	0,88	Yes
ENSMUSG00000028163	-2,66E-01	8,053675326	2,10E+01	4,67E-06	1,70E-04	Nfkb1	0,83	Yes
ENSMUSG00000028164	1,38E-01	6,761507961	5,00E+00	2,54E-02	1,77E-01	Manba	1,10	No
ENSMUSG00000028165	-7,08E-03	6,55107923	1,28E-02	9,10E-01	1,00E+00	Cisd2	1,00	No
ENSMUSG00000028167	-9,81E-01	-2,448591755	1,17E+00	2,80E-01	5,77E-01	Bdh2	0,51	No
ENSMUSG00000028173	2,88E-02	6,838554199	2,20E-01	6,39E-01	8,69E-01	Wls	1,02	No
ENSMUSG00000028175	1,99E-01	3,002618406	3,01E+00	8,27E-02	3,70E-01	Depdc1a	1,15	No
ENSMUSG00000028176	-1,90E+00	-3,033647263	1,44E+00	2,30E-01	5,50E-01	Lrrc7	0,27	No
ENSMUSG00000028177	-1,87E+00	-3,107637761	1,39E+00	2,38E-01	5,50E-01	1810013D15Rik	0,27	No
ENSMUSG00000028179	-7,02E-01	-2,146004908	8,39E-01	3,60E-01	6,61E-01	Cth	0,61	No
ENSMUSG00000028180	-2,86E-03	5,550504175	1,75E-03	9,67E-01	1,00E+00	Zranb2	1,00	No
ENSMUSG00000028182	4,19E-02	-0,832118767	1,11E-02	9,16E-01	1,00E+00	Lrriq3	1,03	No
ENSMUSG00000028184	1,01E-01	4,994930876	1,80E+00	1,80E-01	5,42E-01	Adgrl2	1,07	No
ENSMUSG00000028185	3,14E-02	-3,107617333	-7,69E-10	1,00E+00	1,00E+00	Dnase2b	1,02	No
ENSMUSG00000028186	1,85E+00	-3,107657832	1,35E+00	2,46E-01	5,50E-01	Uox	3,61	No

ENSMUSG00000028187	-6,16E-03	4,275172279	5,41E-03	9,41E-01	1,00E+00	Rpf1		1,00	No
ENSMUSG00000028188	2,62E-01	2,025900096	3,13E+00	7,69E-02	3,54E-01	Spata1		1,20	No
ENSMUSG00000028189	2,15E-01	4,119649189	6,23E+00	1,25E-02	1,10E-01	Ctbs		1,16	No
ENSMUSG00000028191	-4,02E-02	6,214349164	3,93E-01	5,31E-01	8,05E-01	Bcl10		0,97	No
ENSMUSG00000028194	-8,69E-01	-2,963204587	4,16E-01	5,19E-01	7,98E-01	Ddah1		0,55	No
ENSMUSG00000028195	-6,00E-01	-1,424739823	1,20E+00	2,73E-01	5,69E-01	Cyr61		0,66	No
ENSMUSG00000028197	-6,36E-02	1,639046614	1,49E-01	6,99E-01	9,04E-01	Col24a1		0,96	No
ENSMUSG00000028199	2,17E-01	2,451983278	2,64E+00	1,04E-01	3,97E-01	Cryz		1,16	No
ENSMUSG00000028207	3,47E-02	5,737957542	2,66E-01	6,06E-01	8,49E-01	Asph		1,02	No
ENSMUSG00000028211	7,34E-02	6,9529055	1,46E+00	2,27E-01	5,50E-01	Trp53inp1		1,05	No
ENSMUSG00000028212	3,45E-01	4,178432888	1,62E+01	5,84E-05	1,55E-03	Ccne2		1,27	Yes
ENSMUSG00000028214	-5,28E-01	3,251311089	2,41E+01	9,16E-07	4,12E-05	Gem		0,69	Yes
ENSMUSG00000028217	2,56E-01	3,135152189	5,38E+00	2,03E-02	1,53E-01	Cdh17		1,19	No
ENSMUSG00000028218	-4,22E-02	2,553240019	1,09E-01	7,42E-01	9,27E-01	Fam92a		0,97	No
ENSMUSG00000028221	1,14E-01	4,733147318	2,17E+00	1,41E-01	4,74E-01	Tmem55a		1,08	No
ENSMUSG00000028222	-2,74E+00	-3,033596063	2,99E+00	8,38E-02	3,71E-01	Calb1		0,15	No
ENSMUSG00000028223	-7,26E-02	4,269832993	7,57E-01	3,84E-01	6,86E-01	Decr1		0,95	No
ENSMUSG00000028224	-1,72E-01	4,109819943	3,95E+00	4,70E-02	2,59E-01	Nbn		0,89	No
ENSMUSG00000028226	-2,65E+00	-2,963225595	2,80E+00	9,41E-02	3,84E-01	Mmp16		0,16	No
ENSMUSG00000028228	1,22E-01	6,522567769	3,78E+00	5,19E-02	2,77E-01	Cpne3		1,09	No
ENSMUSG00000028229	6,78E-02	2,237538382	2,34E-01	6,29E-01	8,62E-01	Rmdn1		1,05	No
ENSMUSG00000028232	2,03E-01	5,097556325	7,64E+00	5,71E-03	6,17E-02	Tmem68		1,15	No
ENSMUSG00000028233	1,30E-01	5,774662808	3,80E+00	5,13E-02	2,75E-01	Tgs1		1,09	No
ENSMUSG00000028234	-1,82E-01	7,058691654	9,09E+00	2,57E-03	3,41E-02	Rps20		0,88	Yes
ENSMUSG00000028238	9,82E-02	8,697198184	2,95E+00	8,61E-02	3,78E-01	Atp6v0d2		1,07	No
ENSMUSG00000028240	-7,38E-02	-3,033659033	2,66E-03	9,59E-01	1,00E+00	Cyp7a1		0,95	No
ENSMUSG00000028243	1,88E-01	3,576180742	3,70E+00	5,45E-02	2,86E-01	Ubxn2b		1,14	No
ENSMUSG00000028245	2,82E-02	5,423969602	1,64E-01	6,86E-01	8,97E-01	Nsmaf		1,02	No
ENSMUSG00000028246	-1,94E+00	-3,107619243	1,50E+00	2,21E-01	5,50E-01	Faxc		0,26	No
ENSMUSG00000028247	2,81E-01	3,474377258	7,79E+00	5,25E-03	5,79E-02	Coq3		1,21	No
ENSMUSG00000028248	-1,75E-01	6,757658999	8,10E+00	4,42E-03	5,09E-02	Pnisr		0,89	No
ENSMUSG00000028249	1,07E-01	9,051205587	3,56E+00	5,92E-02	3,01E-01	Sdcbp		1,08	No

ENSMUSG00000028251	9,44E-02	4,397758551	1,32E+00	2,50E-01	5,50E-01	Tstd3	1,07	No
ENSMUSG00000028252	9,71E-02	4,732258249	1,59E+00	2,07E-01	5,50E-01	Ccnc	1,07	No
ENSMUSG00000028256	1,82E-01	3,424994222	3,22E+00	7,27E-02	3,42E-01	Odf2l	1,13	No
ENSMUSG00000028261	5,95E-03	4,099232373	4,64E-03	9,46E-01	1,00E+00	Ndufaf4	1,00	No
ENSMUSG00000028262	-1,89E+00	-3,1076441	1,42E+00	2,33E-01	5,50E-01	Clca3a2	0,27	No
ENSMUSG00000028264	-7,98E-01	-2,963241606	3,44E-01	5,57E-01	8,21E-01	Spaca1	0,58	No
ENSMUSG00000028266	2,47E-01	5,637715703	1,31E+01	2,96E-04	5,99E-03	Lmo4	1,19	Yes
ENSMUSG00000028268	-1,48E-01	5,838201072	4,97E+00	2,57E-02	1,78E-01	Gbp3	0,90	No
ENSMUSG00000028270	-3,32E-01	7,24107615	3,09E+01	2,78E-08	1,74E-06	Gbp2	0,79	Yes
ENSMUSG00000028271	-1,40E-01	5,666328793	4,29E+00	3,84E-02	2,33E-01	Gtf2b	0,91	No
ENSMUSG00000028273	-4,71E-02	7,366150069	6,27E-01	4,28E-01	7,22E-01	Pdlim5	0,97	No
ENSMUSG00000028274	1,26E-01	4,358189878	2,32E+00	1,28E-01	4,48E-01	Rngtt	1,09	No
ENSMUSG00000028277	3,67E-03	6,770051924	3,54E-03	9,53E-01	1,00E+00	Ube2j1	1,00	No
ENSMUSG00000028278	-4,25E-01	3,771136359	2,12E+01	4,21E-06	1,56E-04	Rragd	0,74	Yes
ENSMUSG00000028280	-9,24E-01	-2,448697831	7,49E-01	3,87E-01	6,87E-01	Gabrr1	0,53	No
ENSMUSG00000028282	1,49E-01	5,114856651	4,20E+00	4,04E-02	2,38E-01	Casp8ap2	1,11	No
ENSMUSG00000028284	2,25E-02	6,076704779	1,19E-01	7,30E-01	9,21E-01	Map3k7	1,02	No
ENSMUSG00000028289	-8,68E-02	-2,226296834	1,40E-02	9,06E-01	1,00E+00	Epha7	0,94	No
ENSMUSG00000028291	2,03E-01	5,248179988	8,11E+00	4,40E-03	5,07E-02	Akirin2	1,15	No
ENSMUSG00000028292	6,37E-02	2,881172163	3,06E-01	5,80E-01	8,37E-01	Rars2	1,05	No
ENSMUSG00000028293	9,82E-02	4,14369659	1,29E+00	2,55E-01	5,50E-01	Slc35a1	1,07	No
ENSMUSG00000028294	-8,69E-01	-2,963204587	4,16E-01	5,19E-01	7,98E-01	Cfap206	0,55	No
ENSMUSG00000028295	-1,85E-01	2,516674877	2,10E+00	1,48E-01	4,87E-01	Smim8	0,88	No
ENSMUSG00000028300	-9,77E-03	5,276784342	1,91E-02	8,90E-01	1,00E+00	3110043O21Rik	0,99	No
ENSMUSG00000028307	-7,94E-01	-2,896102912	3,44E-01	5,57E-01	8,21E-01	Aldob	0,58	No
ENSMUSG00000028309	-2,97E-02	6,274588708	2,17E-01	6,41E-01	8,71E-01	Rnf20	0,98	No
ENSMUSG00000028310	-2,66E+00	-3,033639428	2,81E+00	9,35E-02	3,83E-01	Ppp3r2	0,16	No
ENSMUSG00000028312	1,99E-01	6,590971754	1,01E+01	1,46E-03	2,19E-02	Smc2	1,15	Yes
ENSMUSG00000028318	1,56E-01	2,63114465	1,53E+00	2,16E-01	5,50E-01	Polr1e	1,11	No
ENSMUSG00000028322	1,42E-02	4,018160407	2,55E-02	8,73E-01	1,00E+00	Exosc3	1,01	No
ENSMUSG00000028327	1,72E-01	4,405264937	4,51E+00	3,37E-02	2,16E-01	Stra6l	1,13	No
ENSMUSG00000028328	3,89E-02	2,805974284	1,10E-01	7,40E-01	9,26E-01	Tmod1	1,03	No

ENSMUSG00000028329	-2,08E-01	3,128235287	3,61E+00	5,74E-02	2,95E-01	Xpa	0,87	No
ENSMUSG00000028330	3,86E-02	5,579710343	3,17E-01	5,73E-01	8,33E-01	Ncbp1	1,03	No
ENSMUSG00000028331	1,83E-01	2,164167814	1,67E+00	1,96E-01	5,50E-01	Trmo	1,14	No
ENSMUSG00000028332	1,99E+00	-0,801464899	2,02E+01	6,96E-06	2,42E-04	Hemgn	3,98	Yes
ENSMUSG00000028333	5,81E-02	6,985221607	9,12E-01	3,40E-01	6,40E-01	Anp32b	1,04	No
ENSMUSG00000028334	-8,28E-02	6,955462462	1,85E+00	1,74E-01	5,31E-01	Nans	0,94	No
ENSMUSG00000028337	-2,42E-02	8,915000856	1,81E-01	6,71E-01	8,88E-01	Coro2a	0,98	No
ENSMUSG00000028339	4,01E-01	2,627095159	1,03E+01	1,35E-03	2,06E-02	Col15a1	1,32	Yes
ENSMUSG00000028341	-4,87E-01	8,207707103	7,08E+01	3,88E-17	7,23E-15	Nr4a3	0,71	Yes
ENSMUSG00000028343	8,18E-02	6,030749263	1,57E+00	2,11E-01	5,50E-01	Erp44	1,06	No
ENSMUSG00000028344	-8,27E-02	3,779336293	7,82E-01	3,76E-01	6,77E-01	Invs	0,94	No
ENSMUSG00000028345	5,16E-02	5,027275148	4,93E-01	4,83E-01	7,70E-01	Tex10	1,04	No
ENSMUSG00000028347	-1,10E-01	2,200000434	5,82E-01	4,46E-01	7,37E-01	Tmeff1	0,93	No
ENSMUSG00000028348	-3,75E-01	-0,587033766	1,09E+00	2,97E-01	5,92E-01	Cavin4	0,77	No
ENSMUSG00000028356	1,24E+00	-2,770611728	9,59E-01	3,27E-01	6,28E-01	Ambp	2,37	No
ENSMUSG00000028357	1,85E+00	-3,107657832	1,35E+00	2,46E-01	5,50E-01	Kif12	3,61	No
ENSMUSG00000028358	-8,95E-01	1,306579346	2,32E+01	1,44E-06	6,11E-05	Zfp618	0,54	Yes
ENSMUSG00000028359	-9,77E-02	1,672597187	3,63E-01	5,47E-01	8,14E-01	Orm3	0,93	No
ENSMUSG00000028360	2,97E-01	-1,800894786	2,56E-01	6,13E-01	8,53E-01	Slc44a5	1,23	No
ENSMUSG00000028362	3,64E-01	6,022232665	3,09E+01	2,77E-08	1,74E-06	Tnfsf8	1,29	Yes
ENSMUSG00000028364	4,55E-01	1,575358009	7,20E+00	7,27E-03	7,38E-02	Tnc	1,37	No
ENSMUSG00000028367	-8,53E-02	6,878873734	1,95E+00	1,63E-01	5,14E-01	Txn1	0,94	No
ENSMUSG00000028369	3,91E-01	0,191305393	1,93E+00	1,65E-01	5,17E-01	Svep1	1,31	No
ENSMUSG00000028370	-1,94E+00	-3,107619243	1,50E+00	2,21E-01	5,50E-01	Pappa	0,26	No
ENSMUSG00000028378	6,62E-02	5,399312216	8,93E-01	3,45E-01	6,46E-01	Ptgr1	1,05	No
ENSMUSG00000028381	1,87E-01	6,057968027	8,29E+00	3,99E-03	4,73E-02	Ugcg	1,14	Yes
ENSMUSG00000028382	1,08E-01	8,656768418	3,53E+00	6,02E-02	3,04E-01	Ptbp3	1,08	No
ENSMUSG00000028383	-5,46E-02	5,02697812	5,56E-01	4,56E-01	7,45E-01	Hsdl2	0,96	No
ENSMUSG00000028385	2,14E-01	7,298332985	1,28E+01	3,45E-04	6,79E-03	Snx30	1,16	Yes
ENSMUSG00000028386	-8,14E-01	-2,65506688	3,66E-01	5,45E-01	8,13E-01	Slc46a2	0,57	No
ENSMUSG00000028389	-7,37E-01	-2,448753987	6,08E-01	4,35E-01	7,28E-01	Zfp37	0,60	No
ENSMUSG00000028391	2,91E-01	-2,54827754	8,63E-02	7,69E-01	9,42E-01	Wdr31	1,22	No

ENSMUSG00000028392	-9,67E-01	-1,448653517	3,24E+00	7,18E-02	3,40E-01	Bspry	0,51	No
ENSMUSG00000028393	3,13E-01	2,358457831	5,29E+00	2,15E-02	1,58E-01	Alad	1,24	No
ENSMUSG00000028394	-3,40E-02	4,964218781	2,06E-01	6,50E-01	8,76E-01	Pole3	0,98	No
ENSMUSG00000028397	2,45E-02	4,816921495	1,02E-01	7,49E-01	9,31E-01	Kdm4c	1,02	No
ENSMUSG00000028398	1,09E-01	4,730850122	1,95E+00	1,62E-01	5,12E-01	Tmem261	1,08	No
ENSMUSG00000028399	-1,71E+00	-2,655090037	2,03E+00	1,54E-01	4,98E-01	Ptprd	0,31	No
ENSMUSG00000028402	-2,67E+00	-2,896062063	2,83E+00	9,24E-02	3,83E-01	Mpdz	0,16	No
ENSMUSG00000028403	3,10E-02	6,394916047	2,40E-01	6,24E-01	8,59E-01	Zdhhc21	1,02	No
ENSMUSG00000028405	7,29E-02	6,79870176	1,41E+00	2,35E-01	5,50E-01	Aco1	1,05	No
ENSMUSG00000028407	-1,41E-01	2,633891403	1,27E+00	2,59E-01	5,52E-01	Toporsos	0,91	No
ENSMUSG00000028409	-7,20E-02	5,924547435	1,19E+00	2,75E-01	5,72E-01	Smu1	0,95	No
ENSMUSG00000028410	-7,07E-02	5,729073036	1,10E+00	2,93E-01	5,89E-01	Dnaja1	0,95	No
ENSMUSG00000028411	1,76E-02	3,775417713	3,58E-02	8,50E-01	9,89E-01	Aptx	1,01	No
ENSMUSG00000028412	-1,83E-01	5,920955761	7,79E+00	5,26E-03	5,80E-02	Slc44a1	0,88	No
ENSMUSG00000028413	4,41E-02	7,617023077	5,58E-01	4,55E-01	7,45E-01	B4galt1	1,03	No
ENSMUSG00000028414	-1,66E-01	3,953303813	3,60E+00	5,79E-02	2,97E-01	Fktn	0,89	No
ENSMUSG00000028415	1,20E+00	-2,711750105	8,89E-01	3,46E-01	6,47E-01	Spink4	2,29	No
ENSMUSG00000028416	8,39E-02	6,836617567	1,87E+00	1,71E-01	5,28E-01	Bag1	1,06	No
ENSMUSG00000028417	-2,84E-01	-1,165701446	3,68E-01	5,44E-01	8,13E-01	Tal2	0,82	No
ENSMUSG00000028419	-4,69E-02	5,862455463	5,00E-01	4,79E-01	7,67E-01	Chmp5	0,97	No
ENSMUSG00000028420	-7,08E-02	4,789220321	8,53E-01	3,56E-01	6,57E-01	Tmem38b	0,95	No
ENSMUSG00000028423	8,42E-02	6,426416221	1,79E+00	1,81E-01	5,42E-01	Nfx1	1,06	No
ENSMUSG00000028426	8,37E-02	7,365108295	1,97E+00	1,61E-01	5,09E-01	Rad23b	1,06	No
ENSMUSG00000028427	-8,16E-02	-2,226302148	1,08E-02	9,17E-01	1,00E+00	Aqp7	0,95	No
ENSMUSG00000028430	5,40E-02	6,154876422	6,96E-01	4,04E-01	7,01E-01	Nol6	1,04	No
ENSMUSG00000028431	6,66E-02	5,062627574	8,27E-01	3,63E-01	6,64E-01	Ikbkap	1,05	No
ENSMUSG00000028433	5,14E-02	6,12295191	6,30E-01	4,27E-01	7,21E-01	Ubap2	1,04	No
ENSMUSG00000028434	8,31E-01	-1,401484279	2,38E+00	1,23E-01	4,38E-01	Epb41l4b	1,78	No
ENSMUSG00000028435	2,43E-01	3,916312331	7,14E+00	7,54E-03	7,58E-02	Aqp3	1,18	No
ENSMUSG00000028436	1,46E-01	6,289106476	5,29E+00	2,15E-02	1,58E-01	Dcaf12	1,11	No
ENSMUSG00000028437	9,29E-03	5,755623263	1,92E-02	8,90E-01	1,00E+00	Ubap1	1,01	No
ENSMUSG00000028438	2,50E-01	3,798379795	7,26E+00	7,05E-03	7,22E-02	Kif24	1,19	No

ENSMUSG00000028439	1,07E-01	5,449002845	2,38E+00	1,23E-01	4,38E-01	Fam219a	1,08	No
ENSMUSG00000028441	6,17E-01	-2,548244722	4,24E-01	5,15E-01	7,96E-01	1110017D15Rik	1,53	No
ENSMUSG00000028443	-7,89E-02	3,882684178	7,58E-01	3,84E-01	6,85E-01	Nudt2	0,95	No
ENSMUSG00000028444	2,63E+00	-2,96325892	2,67E+00	1,02E-01	3,94E-01	Cntfr	6,19	No
ENSMUSG00000028445	2,12E-01	-1,070208595	2,20E-01	6,39E-01	8,69E-01	Enho	1,16	No
ENSMUSG00000028447	-2,34E-02	4,937034954	9,77E-02	7,55E-01	9,34E-01	Dctn3	0,98	No
ENSMUSG00000028451	-1,14E-02	5,00517951	2,37E-02	8,78E-01	1,00E+00	1700022I11Rik	0,99	No
ENSMUSG00000028452	-5,05E-02	6,917292495	6,87E-01	4,07E-01	7,04E-01	Vcp	0,97	No
ENSMUSG00000028453	7,18E-02	4,599767435	8,28E-01	3,63E-01	6,64E-01	Fancg	1,05	No
ENSMUSG00000028454	5,61E-02	4,813665208	5,38E-01	4,63E-01	7,52E-01	Pigo	1,04	No
ENSMUSG00000028455	9,63E-02	4,142944709	1,25E+00	2,63E-01	5,57E-01	Stoml2	1,07	No
ENSMUSG00000028456	3,01E-01	0,738701671	1,67E+00	1,96E-01	5,50E-01	Unc13b	1,23	No
ENSMUSG00000028457	1,35E+00	-1,355711307	6,03E+00	1,41E-02	1,20E-01	Atp8b5	2,54	No
ENSMUSG00000028458	-5,06E-03	5,201139725	4,95E-03	9,44E-01	1,00E+00	Tesk1	1,00	No
ENSMUSG00000028459	1,15E-01	5,072534427	2,46E+00	1,17E-01	4,23E-01	Cd72	1,08	No
ENSMUSG00000028460	1,85E+00	-3,107657832	1,35E+00	2,46E-01	5,50E-01	Sit1	3,61	No
ENSMUSG00000028461	5,62E-02	3,172044652	2,71E-01	6,03E-01	8,48E-01	Ccdc107	1,04	No
ENSMUSG00000028463	1,65E-01	2,955931295	2,08E+00	1,50E-01	4,91E-01	Car9	1,12	No
ENSMUSG00000028464	4,59E-01	-0,96337758	1,23E+00	2,67E-01	5,64E-01	Tpm2	1,37	No
ENSMUSG00000028465	7,00E-02	9,619418938	1,54E+00	2,15E-01	5,50E-01	Tln1	1,05	No
ENSMUSG00000028466	2,57E-02	5,131507069	1,26E-01	7,23E-01	9,17E-01	Creb3	1,02	No
ENSMUSG00000028467	2,64E-02	4,3578167	1,04E-01	7,48E-01	9,30E-01	Gba2	1,02	No
ENSMUSG00000028468	-5,73E-02	5,887165707	7,56E-01	3,84E-01	6,86E-01	Rgp1	0,96	No
ENSMUSG00000028469	-9,73E-01	-1,216022341	4,19E+00	4,07E-02	2,39E-01	Npr2	0,51	No
ENSMUSG00000028470	-2,15E-01	3,188989085	4,03E+00	4,48E-02	2,52E-01	Hint2	0,86	No
ENSMUSG00000028476	-4,51E-01	1,124619783	5,60E+00	1,79E-02	1,40E-01	Reck	0,73	No
ENSMUSG00000028478	-1,03E-02	8,483515868	3,23E-02	8,57E-01	9,94E-01	Clta	0,99	No
ENSMUSG00000028479	1,33E-01	4,539792582	2,79E+00	9,46E-02	3,85E-01	Gne	1,10	No
ENSMUSG00000028480	-6,26E-01	8,203237824	1,17E+02	3,31E-27	1,44E-24	Glipr2	0,65	Yes
ENSMUSG00000028483	-2,21E-01	4,452389476	7,52E+00	6,10E-03	6,49E-02	Snapc3	0,86	No
ENSMUSG00000028484	-7,68E-02	5,154067585	1,14E+00	2,86E-01	5,82E-01	Psip1	0,95	No
ENSMUSG00000028487	-1,87E+00	-2,963234282	1,41E+00	2,35E-01	5,50E-01	Bnc2	0,27	No

ENSMUSG00000028494	1,44E-01	7,841433097	6,02E+00	1,41E-02	1,20E-01	Plin2	1,10	No
ENSMUSG00000028495	-1,87E-01	6,977765935	9,44E+00	2,13E-03	2,94E-02	Rps6	0,88	Yes
ENSMUSG00000028496	1,33E-01	3,769246604	1,97E+00	1,61E-01	5,09E-01	Mllt3	1,10	No
ENSMUSG00000028497	1,55E-01	6,027140198	5,63E+00	1,77E-02	1,39E-01	Hacd4	1,11	No
ENSMUSG00000028514	1,03E-01	6,572028953	2,72E+00	9,88E-02	3,91E-01	Usp24	1,07	No
ENSMUSG00000028517	-1,21E-01	4,172411225	2,00E+00	1,58E-01	5,04E-01	Plpp3	0,92	No
ENSMUSG00000028518	2,56E-01	0,028054498	7,94E-01	3,73E-01	6,74E-01	Prkaa2	1,19	No
ENSMUSG00000028519	-1,92E+00	-3,0336536	1,46E+00	2,27E-01	5,50E-01	Dab1	0,26	No
ENSMUSG00000028520	-3,83E-02	-3,107651936	-7,91E-11	1,00E+00	1,00E+00	4921539E11Rik	0,97	No
ENSMUSG00000028521	1,26E-01	3,897969692	1,93E+00	1,64E-01	5,16E-01	Slc35d1	1,09	No
ENSMUSG00000028522	-6,04E-02	6,11674446	8,72E-01	3,50E-01	6,52E-01	Mier1	0,96	No
ENSMUSG00000028524	8,54E-01	-0,529129161	5,20E+00	2,26E-02	1,64E-01	Sgip1	1,81	No
ENSMUSG00000028525	-8,83E-01	6,846172553	2,04E+02	3,50E-46	6,56E-43	Pde4b	0,54	Yes
ENSMUSG00000028527	4,21E-01	3,920454886	2,13E+01	3,94E-06	1,48E-04	Ak4	1,34	Yes
ENSMUSG00000028528	8,32E-01	-2,268148767	8,33E-01	3,61E-01	6,63E-01	Dnajc6	1,78	No
ENSMUSG00000028530	-5,39E-02	8,068386841	8,62E-01	3,53E-01	6,54E-01	Jak1	0,96	No
ENSMUSG00000028532	3,48E+00	-2,896200697	5,25E+00	2,19E-02	1,60E-01	Cachd1	11,17	No
ENSMUSG00000028536	3,14E-02	-3,107617333	-7,69E-10	1,00E+00	1,00E+00	2610528J11Rik	1,02	No
ENSMUSG00000028538	-3,49E-01	5,40430471	2,50E+01	5,72E-07	2,70E-05	St3gal3	0,78	Yes
ENSMUSG00000028539	5,00E-01	-0,641508401	1,63E+00	2,02E-01	5,50E-01	Artn	1,41	No
ENSMUSG00000028540	1,87E-01	3,185540019	3,08E+00	7,93E-02	3,60E-01	Dph2	1,14	No
ENSMUSG00000028541	1,49E-01	-1,535456199	7,21E-02	7,88E-01	9,53E-01	B4galt2	1,11	No
ENSMUSG00000028542	2,67E-01	-0,621024354	5,44E-01	4,61E-01	7,49E-01	Slc6a9	1,20	No
ENSMUSG00000028544	1,87E+00	-3,107639691	1,38E+00	2,40E-01	5,50E-01	Slc5a9	3,67	No
ENSMUSG00000028545	-2,68E+00	-2,963253845	2,85E+00	9,15E-02	3,82E-01	Bend5	0,16	No
ENSMUSG00000028546	-4,44E-01	-2,185624361	3,63E-01	5,47E-01	8,14E-01	Elavl4	0,73	No
ENSMUSG00000028549	1,96E-01	2,186974516	1,86E+00	1,72E-01	5,29E-01	Itgb3bp	1,15	No
ENSMUSG00000028550	6,37E-02	4,058335587	5,32E-01	4,66E-01	7,54E-01	Atg4c	1,05	No
ENSMUSG00000028551	1,68E-01	5,051762519	5,24E+00	2,21E-02	1,61E-01	Cdkn2c	1,12	No
ENSMUSG00000028552	-4,93E-02	6,81098522	6,47E-01	4,21E-01	7,16E-01	Eps15	0,97	No
ENSMUSG00000028553	-2,14E-01	0,229219762	5,73E-01	4,49E-01	7,39E-01	Angptl3	0,86	No
ENSMUSG00000028555	-4,96E-01	3,25290632	2,26E+01	1,95E-06	7,95E-05	Ttc39a	0,71	Yes

ENSMUSG00000028556	3,70E-02	6,2575105	3,35E-01	5,63E-01	8,26E-01	Dock7	1,03	No
ENSMUSG00000028557	-8,68E-02	5,944515979	1,75E+00	1,86E-01	5,50E-01	Rnf11	0,94	No
ENSMUSG00000028558	-6,05E-01	-2,401366734	4,99E-01	4,80E-01	7,68E-01	Calr4	0,66	No
ENSMUSG00000028559	-9,25E-02	6,378518982	2,13E+00	1,45E-01	4,81E-01	Osbpl9	0,94	No
ENSMUSG00000028560	1,17E-01	6,145175586	3,28E+00	7,02E-02	3,35E-01	Usp1	1,08	No
ENSMUSG00000028563	1,97E-01	2,006276922	1,65E+00	1,98E-01	5,50E-01	Tm2d1	1,15	No
ENSMUSG00000028565	2,26E-01	0,454797422	8,24E-01	3,64E-01	6,65E-01	Nfia	1,17	No
ENSMUSG00000028567	-1,57E-03	4,957847999	4,42E-04	9,83E-01	1,00E+00	Txndc12	1,00	No
ENSMUSG00000028568	-5,79E-02	4,039344635	4,21E-01	5,16E-01	7,97E-01	Btf3l4	0,96	No
ENSMUSG00000028572	-4,56E-01	4,341861704	3,05E+01	3,31E-08	2,04E-06	Hook1	0,73	Yes
ENSMUSG00000028573	-1,07E-01	3,319601081	1,07E+00	3,00E-01	5,96E-01	Fggy	0,93	No
ENSMUSG00000028575	-7,54E-01	-0,424768762	4,83E+00	2,80E-02	1,89E-01	Eqtn	0,59	No
ENSMUSG00000028576	2,61E-01	1,418409864	2,14E+00	1,44E-01	4,80E-01	Ift74	1,20	No
ENSMUSG00000028577	-7,36E-02	6,227963844	1,32E+00	2,51E-01	5,50E-01	Plaa	0,95	No
ENSMUSG00000028578	1,11E-01	3,166909383	1,08E+00	2,99E-01	5,95E-01	Caap1	1,08	No
ENSMUSG00000028580	6,32E-02	6,353763183	9,93E-01	3,19E-01	6,20E-01	Pum1	1,04	No
ENSMUSG00000028581	4,71E-02	10,67894183	7,07E-01	4,00E-01	6,99E-01	Laptm5	1,03	No
ENSMUSG00000028582	5,72E-02	5,758601005	7,26E-01	3,94E-01	6,94E-01	Cc2d1b	1,04	No
ENSMUSG00000028583	4,62E-01	6,059444206	4,96E+01	1,90E-12	2,22E-10	Pdpn	1,38	Yes
ENSMUSG00000028587	6,73E-02	2,860731437	3,28E-01	5,67E-01	8,29E-01	Orc1	1,05	No
ENSMUSG00000028597	-6,25E-01	-1,088720183	1,98E+00	1,59E-01	5,07E-01	Gpx7	0,65	No
ENSMUSG00000028599	1,66E-01	8,907360816	8,51E+00	3,54E-03	4,32E-02	Tnfrsf1b	1,12	Yes
ENSMUSG00000028600	1,13E+00	-2,548192458	1,22E+00	2,69E-01	5,65E-01	Podn	2,19	No
ENSMUSG00000028601	-7,81E-02	-2,600766637	5,55E-03	9,41E-01	1,00E+00	Echdc2	0,95	No
ENSMUSG00000028602	5,49E-01	2,299852224	1,57E+01	7,47E-05	1,90E-03	Tnfrsf8	1,46	Yes
ENSMUSG00000028603	2,33E-02	5,983435961	1,27E-01	7,22E-01	9,16E-01	Scp2	1,02	No
ENSMUSG00000028607	8,84E-02	4,404982841	1,17E+00	2,79E-01	5,75E-01	Cpt2	1,06	No
ENSMUSG00000028608	7,06E-02	3,804730443	5,82E-01	4,45E-01	7,36E-01	0610037L13Rik	1,05	No
ENSMUSG00000028609	-2,46E-02	4,322912077	8,77E-02	7,67E-01	9,40E-01	Magoh	0,98	No
ENSMUSG00000028612	4,24E-02	-3,033592245	-1,17E-09	1,00E+00	1,00E+00	Lrp8os1	1,03	No
ENSMUSG00000028613	3,30E-01	4,644530103	1,71E+01	3,61E-05	1,03E-03	Lrp8	1,26	Yes
ENSMUSG00000028614	1,51E-01	5,329227366	4,56E+00	3,28E-02	2,12E-01	Ndc1	1,11	No

ENSMUSG00000028617	2,38E-02	4,414336781	8,46E-02	7,71E-01	9,43E-01	Lrrc42	1,02	No
ENSMUSG00000028618	-1,57E-02	6,592035634	6,32E-02	8,02E-01	9,61E-01	Tmem59	0,99	No
ENSMUSG00000028619	1,72E-01	4,985886556	5,42E+00	1,99E-02	1,51E-01	Tceanc2	1,13	No
ENSMUSG00000028621	1,73E-01	2,407485276	1,68E+00	1,95E-01	5,50E-01	Cyb5rl	1,13	No
ENSMUSG00000028622	7,17E-02	5,362768256	1,03E+00	3,09E-01	6,07E-01	Mrpl37	1,05	No
ENSMUSG00000028626	-1,20E+00	-1,65512432	4,37E+00	3,65E-02	2,27E-01	Col9a2	0,44	No
ENSMUSG00000028629	2,55E-01	3,834957571	8,04E+00	4,56E-03	5,21E-02	Exo5	1,19	No
ENSMUSG00000028630	-1,40E-02	5,416844527	3,99E-02	8,42E-01	9,85E-01	Dyrk2	0,99	No
ENSMUSG00000028631	-1,89E+00	-3,1076441	1,42E+00	2,33E-01	5,50E-01	Kcnq4	0,27	No
ENSMUSG00000028633	1,94E-01	3,189859915	3,13E+00	7,69E-02	3,54E-01	Ctps	1,14	No
ENSMUSG00000028634	8,36E-02	5,2683709	1,36E+00	2,44E-01	5,50E-01	Hivep3	1,06	No
ENSMUSG00000028636	1,34E-02	2,394398558	1,04E-02	9,19E-01	1,00E+00	Ppcs	1,01	No
ENSMUSG00000028637	-8,70E-01	-2,896137506	4,16E-01	5,19E-01	7,98E-01	Ccdc30	0,55	No
ENSMUSG00000028639	4,75E-02	8,593150175	6,86E-01	4,08E-01	7,04E-01	Ybx1	1,03	No
ENSMUSG00000028640	-2,68E+00	-3,033645766	2,84E+00	9,17E-02	3,82E-01	Tfap2c	0,16	No
ENSMUSG00000028641	2,30E-01	3,898842933	6,35E+00	1,17E-02	1,05E-01	P3h1	1,17	No
ENSMUSG00000028642	2,04E-01	-2,355607365	5,75E-02	8,10E-01	9,66E-01	Tmem269	1,15	No
ENSMUSG00000028643	5,72E-03	3,945349399	4,01E-03	9,50E-01	1,00E+00	Svbp	1,00	No
ENSMUSG00000028644	-4,40E-01	-2,497595259	1,98E-01	6,56E-01	8,80E-01	Ermap	0,74	No
ENSMUSG00000028645	1,65E-01	7,530417908	7,72E+00	5,47E-03	5,97E-02	Slc2a1	1,12	No
ENSMUSG00000028646	7,75E-02	7,712758809	1,74E+00	1,88E-01	5,50E-01	Rragc	1,06	No
ENSMUSG00000028647	1,10E-01	2,970877544	9,35E-01	3,34E-01	6,34E-01	Mycbp	1,08	No
ENSMUSG00000028648	3,57E-02	3,403845155	1,23E-01	7,26E-01	9,18E-01	Ndufs5	1,03	No
ENSMUSG00000028649	1,53E-01	8,774295302	7,21E+00	7,24E-03	7,37E-02	Macf1	1,11	No
ENSMUSG00000028651	3,84E-02	3,789937985	1,72E-01	6,78E-01	8,92E-01	Ppie	1,03	No
ENSMUSG00000028653	2,31E-01	3,605284159	5,81E+00	1,59E-02	1,31E-01	Trit1	1,17	No
ENSMUSG00000028654	-4,70E-01	5,533666759	4,75E+01	5,44E-12	6,11E-10	Mycl	0,72	Yes
ENSMUSG00000028655	3,28E-01	3,39481134	1,04E+01	1,26E-03	1,96E-02	Mfsd2a	1,26	Yes
ENSMUSG00000028656	5,00E-03	9,224146541	7,79E-03	9,30E-01	1,00E+00	Cap1	1,00	No
ENSMUSG00000028657	2,76E-01	7,02479002	2,07E+01	5,45E-06	1,95E-04	Ppt1	1,21	Yes
ENSMUSG00000028661	2,63E+00	-2,831994022	2,67E+00	1,02E-01	3,95E-01	Epha8	6,18	No
ENSMUSG00000028664	-1,21E+00	-2,401373792	1,91E+00	1,67E-01	5,20E-01	Ephb2	0,43	No

ENSMUSG00000028668	4,19E-02	7,176278513	4,87E-01	4,85E-01	7,73E-01	Eloa	1,03	No
ENSMUSG00000028669	-6,60E-02	4,63965502	7,15E-01	3,98E-01	6,98E-01	Pithd1	0,96	No
ENSMUSG00000028670	8,78E-02	6,350664131	1,91E+00	1,66E-01	5,19E-01	Lypla2	1,06	No
ENSMUSG00000028671	1,95E-01	4,252448118	5,31E+00	2,12E-02	1,57E-01	Gale	1,14	No
ENSMUSG00000028672	1,17E-01	5,322827963	2,74E+00	9,79E-02	3,91E-01	Hmgcl	1,08	No
ENSMUSG00000028673	-1,15E-01	7,562945138	3,79E+00	5,16E-02	2,77E-01	Fuca1	0,92	No
ENSMUSG00000028675	-1,96E-02	6,408156855	9,72E-02	7,55E-01	9,34E-01	Pnrc2	0,99	No
ENSMUSG00000028676	-9,61E-02	6,533838375	2,37E+00	1,24E-01	4,40E-01	Srsf10	0,94	No
ENSMUSG00000028677	1,53E-02	5,581330742	5,01E-02	8,23E-01	9,73E-01	Rnf220	1,01	No
ENSMUSG00000028678	3,72E-01	4,014930473	1,81E+01	2,15E-05	6,64E-04	Kif2c	1,29	Yes
ENSMUSG00000028680	1,79E-01	5,960019001	7,11E+00	7,65E-03	7,65E-02	Plk3	1,13	No
ENSMUSG00000028681	-2,69E+00	-2,448627672	2,87E+00	9,03E-02	3,81E-01	Ptch2	0,16	No
ENSMUSG00000028683	5,21E-02	3,282537979	2,43E-01	6,22E-01	8,58E-01	Eif2b3	1,04	No
ENSMUSG00000028684	5,44E-02	4,107776859	3,92E-01	5,31E-01	8,05E-01	Urod	1,04	No
ENSMUSG00000028687	1,07E-01	1,566109973	3,72E-01	5,42E-01	8,12E-01	Mutyh	1,08	No
ENSMUSG00000028688	-2,82E-03	3,880268465	9,62E-04	9,75E-01	1,00E+00	Toe1	1,00	No
ENSMUSG00000028689	-1,16E-01	3,675735944	1,50E+00	2,20E-01	5,50E-01	Ccdc163	0,92	No
ENSMUSG00000028690	6,60E-02	3,251830527	3,96E-01	5,29E-01	8,05E-01	Mmachc	1,05	No
ENSMUSG00000028691	-6,54E-03	8,361693207	1,29E-02	9,10E-01	1,00E+00	Prdx1	1,00	No
ENSMUSG00000028692	6,13E-02	9,88104623	1,19E+00	2,76E-01	5,73E-01	Akr1a1	1,04	No
ENSMUSG00000028693	1,82E-01	5,247236128	6,46E+00	1,10E-02	1,00E-01	Nasp	1,13	No
ENSMUSG00000028696	8,59E-02	2,848816888	5,34E-01	4,65E-01	7,54E-01	Ipp	1,06	No
ENSMUSG00000028698	-3,83E-01	2,580329894	9,48E+00	2,08E-03	2,89E-02	Pik3r3	0,77	Yes
ENSMUSG00000028699	2,57E+00	-3,0336723	2,56E+00	1,10E-01	4,06E-01	Tspan1	5,96	No
ENSMUSG00000028700	7,40E-02	4,072831891	7,11E-01	3,99E-01	6,98E-01	Pomgnt1	1,05	No
ENSMUSG00000028701	4,54E-02	0,554332813	3,73E-02	8,47E-01	9,87E-01	Lurap1	1,03	No
ENSMUSG00000028702	7,37E-03	5,269179451	1,08E-02	9,17E-01	1,00E+00	Rad54l	1,01	No
ENSMUSG00000028703	2,73E-02	5,674329986	1,64E-01	6,86E-01	8,97E-01	Lrrc41	1,02	No
ENSMUSG00000028706	-3,05E-01	4,194501461	1,32E+01	2,82E-04	5,77E-03	Nsun4	0,81	Yes
ENSMUSG00000028707	-1,87E+00	-3,107637761	1,39E+00	2,38E-01	5,50E-01	Dmbx1	0,27	No
ENSMUSG00000028708	1,20E-01	6,297809814	3,57E+00	5,88E-02	3,00E-01	Mknk1	1,09	No
ENSMUSG00000028709	1,39E-01	3,981011983	2,45E+00	1,18E-01	4,25E-01	Mob3c	1,10	No

ENSMUSG00000028710	5,76E-02	3,281396656	3,02E-01	5,82E-01	8,38E-01	Atpaf1	1,04	No
ENSMUSG00000028712	-1,87E+00	-3,107637761	1,39E+00	2,38E-01	5,50E-01	Cyp4a31	0,27	No
ENSMUSG00000028715	2,64E+00	-3,033673223	2,69E+00	1,01E-01	3,92E-01	Cyp4a14	6,23	No
ENSMUSG00000028716	-2,02E-01	3,98674627	5,30E+00	2,14E-02	1,58E-01	Pdzk1ip1	0,87	No
ENSMUSG00000028717	3,05E-02	5,094409406	1,77E-01	6,74E-01	8,90E-01	Tal1	1,02	No
ENSMUSG00000028718	2,09E-01	4,885274765	7,52E+00	6,10E-03	6,49E-02	Stil	1,16	No
ENSMUSG00000028719	2,25E-02	6,686498188	1,32E-01	7,16E-01	9,13E-01	Cmpk1	1,02	No
ENSMUSG00000028729	-1,07E-01	4,97081188	2,05E+00	1,52E-01	4,94E-01	Ebna1bp2	0,93	No
ENSMUSG00000028730	7,32E-01	-2,770588265	2,92E-01	5,89E-01	8,41E-01	Cfap57	1,66	No
ENSMUSG00000028737	3,78E-02	4,516905498	2,24E-01	6,36E-01	8,67E-01	Aldh4a1	1,03	No
ENSMUSG00000028738	1,86E+00	-3,033646451	1,35E+00	2,46E-01	5,50E-01	Tas1r2	3,62	No
ENSMUSG00000028741	-1,39E-01	4,001151073	2,40E+00	1,22E-01	4,35E-01	Mrto4	0,91	No
ENSMUSG00000028743	8,78E-02	4,791276781	1,34E+00	2,47E-01	5,50E-01	Akr7a5	1,06	No
ENSMUSG00000028744	1,28E-01	4,272375349	2,36E+00	1,24E-01	4,41E-01	Pqlc2	1,09	No
ENSMUSG00000028745	-2,28E-02	8,521594262	1,58E-01	6,91E-01	9,00E-01	Capzb	0,98	No
ENSMUSG00000028751	-2,30E-01	-1,831974361	1,17E-01	7,32E-01	9,22E-01	Pla2g2e	0,85	No
ENSMUSG00000028753	1,87E+00	-3,107639691	1,38E+00	2,40E-01	5,50E-01	Vwa5b1	3,67	No
ENSMUSG00000028755	-7,94E-01	-2,401357693	9,16E-01	3,39E-01	6,39E-01	Cda	0,58	No
ENSMUSG00000028756	-5,49E-02	5,494441894	6,34E-01	4,26E-01	7,20E-01	Pink1	0,96	No
ENSMUSG00000028757	9,07E-02	7,210696612	2,28E+00	1,31E-01	4,55E-01	Ddost	1,06	No
ENSMUSG00000028758	-3,47E-01	-1,355517475	3,85E-01	5,35E-01	8,07E-01	Kif17	0,79	No
ENSMUSG00000028759	-2,18E-02	7,372094894	1,34E-01	7,15E-01	9,12E-01	Hp1bp3	0,99	No
ENSMUSG00000028760	7,45E-03	6,987030859	1,50E-02	9,02E-01	1,00E+00	Eif4g3	1,01	No
ENSMUSG00000028763	-2,28E-01	0,477507126	8,82E-01	3,48E-01	6,49E-01	Hspg2	0,85	No
ENSMUSG00000028766	-3,88E+00	-2,548257857	7,22E+00	7,20E-03	7,34E-02	Alpl	0,07	No
ENSMUSG00000028771	6,43E-02	7,264567859	1,15E+00	2,85E-01	5,81E-01	Ptpn12	1,05	No
ENSMUSG00000028772	7,64E-02	4,621492454	9,43E-01	3,32E-01	6,32E-01	Zcchc17	1,05	No
ENSMUSG00000028773	-1,36E-01	-0,763086211	1,12E-01	7,38E-01	9,26E-01	Fabp3	0,91	No
ENSMUSG00000028776	-3,59E+00	-2,770545444	5,79E+00	1,61E-02	1,32E-01	Tinagl1	0,08	No
ENSMUSG00000028777	-1,65E-01	-1,35546962	8,55E-02	7,70E-01	9,42E-01	Gnat3	0,89	No
ENSMUSG00000028778	-1,96E+00	-2,896145386	1,50E+00	2,21E-01	5,50E-01	Hcrtr1	0,26	No
ENSMUSG00000028779	2,68E-02	5,973766158	1,67E-01	6,83E-01	8,95E-01	Pef1	1,02	No

ENSMUSG00000028780	-9,80E-01	-2,401330208	1,16E+00	2,81E-01	5,77E-01	Sema3c	0,51	No
ENSMUSG00000028782	-1,48E+00	-2,268049272	2,26E+00	1,33E-01	4,59E-01	Adgrb2	0,36	No
ENSMUSG00000028784	-4,80E-02	-2,89615251	1,06E-03	9,74E-01	1,00E+00	Spocd1	0,97	No
ENSMUSG00000028785	-1,01E+00	-2,355499655	1,22E+00	2,69E-01	5,65E-01	Hpca	0,50	No
ENSMUSG00000028788	-3,59E-02	8,307778158	3,87E-01	5,34E-01	8,07E-01	Ptp4a2	0,98	No
ENSMUSG00000028789	-1,28E+00	1,188235728	4,24E+01	7,50E-11	7,23E-09	Azin2	0,41	Yes
ENSMUSG00000028790	6,36E-02	7,295319998	1,13E+00	2,87E-01	5,83E-01	Khdrbs1	1,05	No
ENSMUSG00000028792	-2,08E-01	6,65446151	1,13E+01	7,91E-04	1,33E-02	Ak2	0,87	Yes
ENSMUSG00000028793	-5,86E-01	7,414406947	9,67E+01	8,01E-23	2,42E-20	Rnf19b	0,67	Yes
ENSMUSG00000028794	-3,18E-01	0,920677438	2,17E+00	1,41E-01	4,75E-01	A3galt2	0,80	No
ENSMUSG00000028795	-4,37E-02	3,427594735	1,94E-01	6,60E-01	8,82E-01	Ccdc28b	0,97	No
ENSMUSG00000028796	-1,68E-01	6,967476745	7,59E+00	5,87E-03	6,31E-02	Phc2	0,89	No
ENSMUSG00000028797	-6,85E-02	6,80802859	1,24E+00	2,65E-01	5,60E-01	Tmem234	0,95	No
ENSMUSG00000028798	-1,20E-01	5,794402729	3,23E+00	7,25E-02	3,41E-01	Eif3i	0,92	No
ENSMUSG00000028799	-2,42E-01	4,98511091	1,07E+01	1,06E-03	1,69E-02	Zfp362	0,85	Yes
ENSMUSG00000028800	-1,22E-01	4,92504535	2,66E+00	1,03E-01	3,95E-01	Hdac1	0,92	No
ENSMUSG00000028801	-1,57E-01	-2,126700633	4,43E-02	8,33E-01	9,79E-01	Stpg1	0,90	No
ENSMUSG00000028803	7,03E-02	5,713652592	1,09E+00	2,95E-01	5,91E-01	Nipal3	1,05	No
ENSMUSG00000028804	-1,77E+00	-2,600685171	2,18E+00	1,40E-01	4,73E-01	Csmd2	0,29	No
ENSMUSG00000028807	3,06E-01	2,54017614	5,78E+00	1,62E-02	1,32E-01	Zbtb8a	1,24	No
ENSMUSG00000028809	-3,66E-02	7,255637922	3,73E-01	5,41E-01	8,11E-01	Srrm1	0,97	No
ENSMUSG00000028811	2,57E-02	5,64176713	1,42E-01	7,06E-01	9,08E-01	Yars	1,02	No
ENSMUSG00000028820	5,49E-02	7,977741352	8,90E-01	3,45E-01	6,46E-01	Sfpq	1,04	No
ENSMUSG00000028821	-2,86E-02	5,716112826	1,80E-01	6,71E-01	8,89E-01	Syf2	0,98	No
ENSMUSG00000028822	9,36E-02	7,082458673	2,39E+00	1,22E-01	4,36E-01	Tmem50a	1,07	No
ENSMUSG00000028825	1,06E-01	-1,998059314	2,49E-02	8,75E-01	1,00E+00	Rhd	1,08	No
ENSMUSG00000028826	-1,47E-02	6,372771506	5,42E-02	8,16E-01	9,69E-01	Tmem57	0,99	No
ENSMUSG00000028830	7,63E-03	5,590648917	1,24E-02	9,11E-01	1,00E+00	AU040320	1,01	No
ENSMUSG00000028832	1,92E-01	5,767073075	8,14E+00	4,32E-03	5,01E-02	Stmn1	1,14	No
ENSMUSG00000028833	9,04E-02	5,740048623	1,80E+00	1,80E-01	5,41E-01	Ncdn	1,06	No
ENSMUSG00000028834	-3,16E+00	-2,963246013	4,20E+00	4,04E-02	2,38E-01	Trim63	0,11	No
ENSMUSG00000028836	-8,72E-01	-2,185589888	1,37E+00	2,43E-01	5,50E-01	Slc30a2	0,55	No

ENSMUSG00000028837	-1,31E-02	6,183072572	4,12E-02	8,39E-01	9,83E-01	Psmb2		0,99	No
ENSMUSG00000028838	-9,43E-01	2,17100838	4,43E+01	2,81E-11	2,85E-09	Extl1		0,52	Yes
ENSMUSG00000028840	-6,44E-02	2,734808866	2,72E-01	6,02E-01	8,48E-01	Zfp593		0,96	No
ENSMUSG00000028841	1,21E-02	-3,033650469	7,34E-11	1,00E+00	1,00E+00	Cnksr1		1,01	No
ENSMUSG00000028842	-1,14E-02	5,635295537	2,80E-02	8,67E-01	9,99E-01	Ago3		0,99	No
ENSMUSG00000028843	1,28E-02	9,209617496	5,06E-02	8,22E-01	9,72E-01	Sh3bgrl3		1,01	No
ENSMUSG00000028845	6,11E-01	-0,655125628	2,51E+00	1,13E-01	4,14E-01	Tekt2		1,53	No
ENSMUSG00000028847	-8,46E-02	5,96246271	1,66E+00	1,98E-01	5,50E-01	Trappc3		0,94	No
ENSMUSG00000028848	1,09E-02	4,062379276	1,54E-02	9,01E-01	1,00E+00	Gpn2		1,01	No
ENSMUSG00000028849	1,68E-01	7,417500715	7,95E+00	4,81E-03	5,43E-02	Map7d1		1,12	No
ENSMUSG00000028850	5,48E-02	3,93368639	3,66E-01	5,45E-01	8,13E-01	Gpatch3		1,04	No
ENSMUSG00000028851	9,78E-02	5,57978659	2,02E+00	1,55E-01	4,99E-01	Nudc		1,07	No
ENSMUSG00000028854	1,49E-01	6,528829313	5,64E+00	1,75E-02	1,38E-01	Slc9a1		1,11	No
ENSMUSG00000028857	3,19E-02	5,280580572	2,00E-01	6,55E-01	8,79E-01	Tmem222		1,02	No
ENSMUSG00000028859	-2,85E-01	2,955930225	6,13E+00	1,33E-02	1,15E-01	Csf3r		0,82	No
ENSMUSG00000028860	-1,85E-01	-0,879891891	2,00E-01	6,55E-01	8,79E-01	Sytl1		0,88	No
ENSMUSG00000028861	3,56E-02	4,681691732	2,06E-01	6,50E-01	8,76E-01	Mrps15		1,02	No
ENSMUSG00000028862	4,36E-01	3,373485427	1,76E+01	2,69E-05	8,00E-04	Map3k6		1,35	Yes
ENSMUSG00000028863	2,52E-02	4,771724145	1,08E-01	7,43E-01	9,28E-01	Meaf6		1,02	No
ENSMUSG00000028864	2,78E-01	3,506351957	7,55E+00	6,01E-03	6,43E-02	Hgf		1,21	No
ENSMUSG00000028865	1,17E-01	-2,600717295	1,32E-02	9,09E-01	1,00E+00	Cd164l2		1,08	No
ENSMUSG00000028868	-1,44E-02	7,972852065	6,07E-02	8,05E-01	9,63E-01	Wasf2		0,99	No
ENSMUSG00000028869	-1,10E-01	5,055793441	2,26E+00	1,33E-01	4,59E-01	Gnl2		0,93	No
ENSMUSG00000028871	-7,22E-02	-2,655052582	3,49E-03	9,53E-01	1,00E+00	Rspo1		0,95	No
ENSMUSG00000028873	1,70E-01	5,409509122	5,92E+00	1,49E-02	1,25E-01	Cdca8		1,13	No
ENSMUSG00000028874	8,21E-02	7,049079484	1,84E+00	1,75E-01	5,33E-01	Fgr		1,06	No
ENSMUSG00000028876	-7,62E-01	-1,998032447	1,18E+00	2,77E-01	5,73E-01	Epha10		0,59	No
ENSMUSG00000028878	7,70E-02	5,827246645	1,33E+00	2,49E-01	5,50E-01	Fam76a		1,05	No
ENSMUSG00000028879	-3,77E-02	6,482522129	3,60E-01	5,49E-01	8,16E-01	Stx12		0,97	No
ENSMUSG00000028882	5,72E-02	4,981145714	5,94E-01	4,41E-01	7,33E-01	Ppp1r8		1,04	No
ENSMUSG00000028883	-7,63E-02	-2,185699107	9,43E-03	9,23E-01	1,00E+00	Sema3a		0,95	No
ENSMUSG00000028884	1,22E-01	4,857481916	2,62E+00	1,06E-01	3,98E-01	Rpa2		1,09	No

ENSMUSG00000028885	-2,35E-01	4,783116144	9,43E+00	2,13E-03	2,94E-02	Smpdl3b	0,85	Yes
ENSMUSG00000028886	-2,66E-02	6,361620002	1,77E-01	6,74E-01	8,90E-01	Eya3	0,98	No
ENSMUSG00000028889	1,43E-02	5,015105467	3,69E-02	8,48E-01	9,88E-01	Yrdc	1,01	No
ENSMUSG00000028890	-1,13E-01	5,344760564	2,61E+00	1,06E-01	3,99E-01	Mtf1	0,92	No
ENSMUSG00000028893	-1,98E-01	6,765575386	1,04E+01	1,29E-03	1,99E-02	Sesn2	0,87	Yes
ENSMUSG00000028894	-5,07E-02	6,498655987	6,56E-01	4,18E-01	7,13E-01	Inpp5b	0,97	No
ENSMUSG00000028896	1,95E-01	5,170106331	7,32E+00	6,80E-03	7,03E-02	Rcc1	1,15	No
ENSMUSG00000028898	7,43E-03	4,21093375	7,59E-03	9,31E-01	1,00E+00	Trnau1ap	1,01	No
ENSMUSG00000028899	-1,58E-04	4,368815205	3,73E-06	9,98E-01	1,00E+00	Taf12	1,00	No
ENSMUSG00000028901	1,70E-01	4,693048003	4,71E+00	2,99E-02	1,98E-01	Gmeb1	1,12	No
ENSMUSG00000028902	-4,31E-02	4,497173673	2,88E-01	5,92E-01	8,42E-01	Sf3a3	0,97	No
ENSMUSG00000028906	-8,59E-02	6,737938998	1,94E+00	1,64E-01	5,15E-01	Epb41	0,94	No
ENSMUSG00000028907	-5,31E-02	4,162409642	3,79E-01	5,38E-01	8,09E-01	Utp11	0,96	No
ENSMUSG00000028909	5,05E-01	-1,57415713	7,90E-01	3,74E-01	6,75E-01	Ptpru	1,42	No
ENSMUSG00000028910	8,40E-02	3,397338914	6,69E-01	4,13E-01	7,09E-01	Mecr	1,06	No
ENSMUSG00000028911	5,56E-02	5,613135587	6,64E-01	4,15E-01	7,10E-01	Srsf4	1,04	No
ENSMUSG00000028914	1,32E-01	6,041090323	4,14E+00	4,18E-02	2,43E-01	Casp9	1,10	No
ENSMUSG00000028917	-1,91E-01	7,155628857	1,01E+01	1,52E-03	2,25E-02	Plekhm2	0,88	Yes
ENSMUSG00000028919	3,14E-02	-3,107617333	-7,69E-10	1,00E+00	1,00E+00	Arhgef19	1,02	No
ENSMUSG00000028920	7,48E-02	5,508448624	1,19E+00	2,76E-01	5,73E-01	Fbxo42	1,05	No
ENSMUSG00000028923	-4,84E-02	7,531353308	6,72E-01	4,12E-01	7,08E-01	Necap2	0,97	No
ENSMUSG00000028926	2,14E-01	4,243195666	6,39E+00	1,15E-02	1,03E-01	Cdk14	1,16	No
ENSMUSG00000028927	1,06E-01	2,815345295	7,61E-01	3,83E-01	6,85E-01	Padi2	1,08	No
ENSMUSG00000028931	2,09E-01	6,998193838	1,18E+01	5,95E-04	1,06E-02	Kcnab2	1,16	Yes
ENSMUSG00000028932	-2,53E-02	6,069413434	1,52E-01	6,97E-01	9,03E-01	Psmc2	0,98	No
ENSMUSG00000028933	1,75E-01	2,624406874	1,95E+00	1,62E-01	5,12E-01	Xrcc2	1,13	No
ENSMUSG00000028936	-1,96E-01	6,268658948	9,47E+00	2,08E-03	2,90E-02	Rpl22	0,87	Yes
ENSMUSG00000028937	-1,55E-01	6,468065718	6,06E+00	1,39E-02	1,19E-01	Acot7	0,90	No
ENSMUSG00000028938	-1,87E+00	-3,107637761	1,39E+00	2,38E-01	5,50E-01	Galntl5	0,27	No
ENSMUSG00000028940	5,98E-01	0,457745391	5,43E+00	1,98E-02	1,50E-01	Hes2	1,51	No
ENSMUSG00000028943	1,17E-01	2,979697412	1,08E+00	2,98E-01	5,94E-01	Espn	1,08	No
ENSMUSG00000028944	2,16E-02	4,792544638	8,03E-02	7,77E-01	9,46E-01	Prkag2	1,02	No

ENSMUSG00000028945	4,96E-02	5,750813705	5,44E-01	4,61E-01	7,49E-01	Rheb	1,04	No
ENSMUSG00000028946	-8,79E-01	-2,89610048	4,25E-01	5,15E-01	7,96E-01	Hes3	0,54	No
ENSMUSG00000028948	2,71E-01	4,886395378	1,29E+01	3,31E-04	6,57E-03	Nol9	1,21	Yes
ENSMUSG00000028949	8,30E-02	4,388162205	1,02E+00	3,14E-01	6,14E-01	Smarcd3	1,06	No
ENSMUSG00000028950	3,50E-01	-1,47302537	3,95E-01	5,30E-01	8,05E-01	Tas1r1	1,27	No
ENSMUSG00000028952	2,55E-01	2,847630302	4,76E+00	2,91E-02	1,94E-01	Zbtb48	1,19	No
ENSMUSG00000028953	6,04E-02	6,039788526	8,60E-01	3,54E-01	6,55E-01	Abcf2	1,04	No
ENSMUSG00000028954	-2,43E-01	7,047794518	1,61E+01	5,91E-05	1,56E-03	Nub1	0,85	Yes
ENSMUSG00000028955	1,28E-01	6,691346423	4,27E+00	3,88E-02	2,33E-01	Vamp3	1,09	No
ENSMUSG00000028957	2,60E-01	2,358382612	3,78E+00	5,18E-02	2,77E-01	Per3	1,20	No
ENSMUSG00000028958	-9,98E-02	3,89331972	1,20E+00	2,73E-01	5,70E-01	Tmub1	0,93	No
ENSMUSG00000028959	1,62E-01	6,07030336	6,18E+00	1,29E-02	1,13E-01	Fastk	1,12	No
ENSMUSG00000028960	7,43E-02	6,343089571	1,37E+00	2,41E-01	5,50E-01	Ube4b	1,05	No
ENSMUSG00000028961	1,32E-01	8,809606424	5,33E+00	2,09E-02	1,56E-01	Pgd	1,10	No
ENSMUSG00000028962	9,23E-02	6,151538146	2,05E+00	1,52E-01	4,94E-01	Slc4a2	1,07	No
ENSMUSG00000028964	-1,34E-01	4,774769392	3,04E+00	8,11E-02	3,66E-01	Park7	0,91	No
ENSMUSG00000028965	-8,35E-01	6,743554652	1,83E+02	8,64E-42	1,18E-38	Tnfrsf9	0,56	Yes
ENSMUSG00000028967	-2,31E-02	5,00928235	9,41E-02	7,59E-01	9,36E-01	Errfi1	0,98	No
ENSMUSG00000028969	2,58E-02	3,882183527	8,06E-02	7,77E-01	9,46E-01	Cdk5	1,02	No
ENSMUSG00000028970	2,45E-01	3,654102566	6,44E+00	1,12E-02	1,01E-01	Abcb1b	1,18	No
ENSMUSG00000028971	8,12E-01	-2,497564179	5,74E-01	4,49E-01	7,39E-01	Cort	1,76	No
ENSMUSG00000028972	-1,37E+00	-2,355429764	2,57E+00	1,09E-01	4,04E-01	Car6	0,39	No
ENSMUSG00000028973	4,99E-02	4,919277194	4,46E-01	5,04E-01	7,89E-01	Abcb8	1,04	No
ENSMUSG00000028974	9,56E-02	5,114255932	1,72E+00	1,90E-01	5,50E-01	Dffa	1,07	No
ENSMUSG00000028975	-1,25E-01	4,157796773	2,11E+00	1,47E-01	4,85E-01	Pex14	0,92	No
ENSMUSG00000028976	-1,54E+00	-2,107591405	3,42E+00	6,43E-02	3,17E-01	Slc2a5	0,34	No
ENSMUSG00000028977	-1,37E-01	4,389239674	2,85E+00	9,15E-02	3,82E-01	Casz1	0,91	No
ENSMUSG00000028978	5,55E-02	-1,697391224	8,25E-03	9,28E-01	1,00E+00	Nos3	1,04	No
ENSMUSG00000028979	-3,60E-01	3,258736703	1,13E+01	7,85E-04	1,32E-02	Masp2	0,78	Yes
ENSMUSG00000028980	2,48E-01	5,144096875	1,17E+01	6,12E-04	1,08E-02	H6pd	1,19	Yes
ENSMUSG00000028982	-2,06E-02	2,53170588	2,43E-02	8,76E-01	1,00E+00	Slc25a33	0,99	No
ENSMUSG00000028986	2,36E-02	4,022678834	7,01E-02	7,91E-01	9,55E-01	Klhl7	1,02	No

ENSMUSG00000028988	1,22E-01	4,115940959	1,99E+00	1,58E-01	5,05E-01	Ctnnbip1	1,09	No
ENSMUSG00000028989	-4,61E-01	-1,896111237	3,91E-01	5,32E-01	8,05E-01	Angptl7	0,73	No
ENSMUSG00000028990	4,03E-03	4,353311381	2,40E-03	9,61E-01	1,00E+00	Lzic	1,00	No
ENSMUSG00000028991	-2,17E-01	6,477300575	1,20E+01	5,33E-04	9,65E-03	Mtor	0,86	Yes
ENSMUSG00000028992	-1,28E-01	3,40740632	1,63E+00	2,02E-01	5,50E-01	Nmnat1	0,92	No
ENSMUSG00000028995	1,32E-01	5,895343117	4,02E+00	4,50E-02	2,53E-01	Fam126a	1,10	No
ENSMUSG00000028996	1,89E+00	-3,033614598	1,38E+00	2,40E-01	5,50E-01	Rbp7	3,70	No
ENSMUSG00000028998	-1,65E-01	5,320184293	5,43E+00	1,98E-02	1,50E-01	Tomm7	0,89	No
ENSMUSG00000028999	1,79E-01	3,688817855	3,61E+00	5,73E-02	2,95E-01	Rint1	1,13	No
ENSMUSG00000029001	-2,44E-01	2,176042237	2,84E+00	9,21E-02	3,82E-01	Fbxo44	0,84	No
ENSMUSG00000029003	1,62E-01	2,891425018	1,92E+00	1,66E-01	5,19E-01	Mad2l2	1,12	No
ENSMUSG00000029004	9,40E-02	6,831456206	2,35E+00	1,25E-01	4,43E-01	Kmt2e	1,07	No
ENSMUSG00000029005	-3,55E+00	-2,8319544	5,67E+00	1,73E-02	1,37E-01	Draxin	0,09	No
ENSMUSG00000029007	2,96E-02	6,946445839	2,36E-01	6,27E-01	8,61E-01	Agtrap	1,02	No
ENSMUSG00000029009	1,46E-01	5,893862275	4,93E+00	2,64E-02	1,82E-01	Mthfr	1,11	No
ENSMUSG00000029012	2,77E-02	3,45743161	7,69E-02	7,82E-01	9,49E-01	Orc5	1,02	No
ENSMUSG00000029014	4,36E-02	4,968012229	3,42E-01	5,59E-01	8,22E-01	Dnajc2	1,03	No
ENSMUSG00000029016	1,80E-01	4,801851576	5,50E+00	1,90E-02	1,46E-01	Clcn6	1,13	No
ENSMUSG00000029017	-1,06E-01	5,034497946	2,04E+00	1,53E-01	4,96E-01	Pmpcb	0,93	No
ENSMUSG00000029019	-3,73E-01	0,266610117	1,84E+00	1,75E-01	5,33E-01	Nppb	0,77	No
ENSMUSG00000029020	9,66E-02	6,356801719	2,32E+00	1,28E-01	4,49E-01	Mfn2	1,07	No
ENSMUSG00000029022	-2,31E-01	5,210862831	1,04E+01	1,23E-03	1,92E-02	Miip	0,85	Yes
ENSMUSG00000029026	8,10E-01	-2,770570729	5,69E-01	4,51E-01	7,41E-01	Trp73	1,75	No
ENSMUSG00000029027	-4,89E-02	3,290588788	2,22E-01	6,38E-01	8,68E-01	Dffb	0,97	No
ENSMUSG00000029028	6,43E-02	5,808158396	9,26E-01	3,36E-01	6,37E-01	Lrrc47	1,05	No
ENSMUSG00000029029	-1,03E-01	3,45758006	1,06E+00	3,03E-01	5,99E-01	Wrap73	0,93	No
ENSMUSG00000029030	-1,53E-01	6,730330917	6,14E+00	1,32E-02	1,15E-01	Tprgl	0,90	No
ENSMUSG00000029032	-5,72E-01	-2,831971956	2,57E-01	6,12E-01	8,52E-01	Arhgef16	0,67	No
ENSMUSG00000029033	1,42E-01	5,075098554	3,75E+00	5,29E-02	2,81E-01	Acap3	1,10	No
ENSMUSG00000029034	-1,13E-01	5,187085629	2,48E+00	1,15E-01	4,19E-01	Ints11	0,92	No
ENSMUSG00000029036	1,59E-01	4,471544742	3,78E+00	5,18E-02	2,77E-01	Atad3a	1,12	No
ENSMUSG00000029038	-7,03E-02	6,136240334	1,19E+00	2,76E-01	5,73E-01	Ssu72	0,95	No

ENSMUSG00000029047	6,24E-02	2,327295368	2,09E-01	6,48E-01	8,75E-01	Pex10	1,04	No
ENSMUSG00000029048	9,17E-02	6,278171203	2,06E+00	1,51E-01	4,93E-01	Rer1	1,07	No
ENSMUSG00000029049	1,68E-01	0,062160154	3,52E-01	5,53E-01	8,19E-01	Morn1	1,12	No
ENSMUSG00000029050	2,89E-01	7,657909551	2,39E+01	1,03E-06	4,56E-05	Ski	1,22	Yes
ENSMUSG00000029053	-2,88E-01	-1,333222792	3,98E-01	5,28E-01	8,04E-01	Prkcz	0,82	No
ENSMUSG00000029054	-5,64E-01	-1,998042796	6,96E-01	4,04E-01	7,01E-01	Gabrd	0,68	No
ENSMUSG00000029055	-8,91E-02	-2,896169321	3,42E-03	9,53E-01	1,00E+00	Plch2	0,94	No
ENSMUSG00000029056	1,70E-01	5,05859182	5,36E+00	2,06E-02	1,54E-01	Pank4	1,13	No
ENSMUSG00000029059	1,70E-01	1,727760204	1,07E+00	3,01E-01	5,97E-01	Fam213b	1,12	No
ENSMUSG00000029060	1,16E-01	5,479276594	2,80E+00	9,42E-02	3,84E-01	Mib2	1,08	No
ENSMUSG00000029061	-2,17E-01	3,906891663	6,07E+00	1,37E-02	1,18E-01	Mmp23	0,86	No
ENSMUSG00000029062	-2,49E-02	6,294757397	1,53E-01	6,96E-01	9,02E-01	Cdk11b	0,98	No
ENSMUSG00000029063	4,41E-02	7,330218933	5,45E-01	4,60E-01	7,49E-01	Nadk	1,03	No
ENSMUSG00000029064	-5,66E-02	8,355696849	9,64E-01	3,26E-01	6,27E-01	Gnb1	0,96	No
ENSMUSG00000029066	-6,62E-02	5,074845698	8,18E-01	3,66E-01	6,67E-01	Mrpl20	0,96	No
ENSMUSG00000029068	-9,03E-02	7,196710472	2,27E+00	1,32E-01	4,57E-01	Ccnl2	0,94	No
ENSMUSG00000029070	-4,05E-01	-0,442634379	1,43E+00	2,32E-01	5,50E-01	Mxra8	0,76	No
ENSMUSG00000029071	-1,18E-01	5,322515212	2,78E+00	9,55E-02	3,86E-01	Dvl1	0,92	No
ENSMUSG00000029072	8,58E-02	0,791502419	1,43E-01	7,05E-01	9,07E-01	Tas1r3	1,06	No
ENSMUSG00000029073	1,52E-01	4,505294743	3,59E+00	5,81E-02	2,97E-01	Cptp	1,11	No
ENSMUSG00000029074	-2,21E+00	-2,448535489	6,30E+00	1,21E-02	1,07E-01	Ttll10	0,22	No
ENSMUSG00000029075	-7,62E-01	2,966283344	4,74E+01	5,72E-12	6,37E-10	Tnfrsf4	0,59	Yes
ENSMUSG00000029076	-1,61E-01	7,832275274	7,55E+00	6,00E-03	6,42E-02	Sdf4	0,89	No
ENSMUSG00000029082	-1,60E-01	6,610604007	6,59E+00	1,03E-02	9,53E-02	Bst1	0,89	No
ENSMUSG00000029084	-3,85E-01	4,397395248	2,20E+01	2,75E-06	1,07E-04	Cd38	0,77	Yes
ENSMUSG00000029086	-2,15E-01	0,233023641	6,19E-01	4,31E-01	7,25E-01	Prom1	0,86	No
ENSMUSG00000029088	-1,43E-01	0,621440669	3,79E-01	5,38E-01	8,09E-01	Kcnip4	0,91	No
ENSMUSG00000029089	-2,65E-02	3,059729105	5,74E-02	8,11E-01	9,66E-01	5730480H06Rik	0,98	No
ENSMUSG00000029090	9,04E-03	1,486746311	2,69E-03	9,59E-01	1,00E+00	Adgra3	1,01	No
ENSMUSG00000029092	-2,23E-02	-2,832006671	3,01E-04	9,86E-01	1,00E+00	D5Ertd615e	0,98	No
ENSMUSG00000029094	-4,59E-02	3,882404284	2,51E-01	6,17E-01	8,55E-01	Afap1	0,97	No
ENSMUSG00000029095	6,75E-01	0,545030857	7,58E+00	5,89E-03	6,33E-02	Ablim2	1,60	No

ENSMUSG00000029096	8,35E-01	2,244337023	3,28E+01	1,03E-08	7,12E-07	Htra3	1,78	Yes
ENSMUSG00000029097	9,63E-02	3,705147552	1,05E+00	3,04E-01	6,01E-01	Trmt44	1,07	No
ENSMUSG00000029098	1,11E-01	8,054040623	3,66E+00	5,56E-02	2,89E-01	Acox3	1,08	No
ENSMUSG00000029101	-2,21E-01	5,019889514	9,18E+00	2,45E-03	3,28E-02	Rgs12	0,86	Yes
ENSMUSG00000029102	4,76E-02	5,562633125	4,84E-01	4,87E-01	7,74E-01	Hgfac	1,03	No
ENSMUSG00000029103	1,83E-01	8,824646101	1,02E+01	1,38E-03	2,09E-02	Lrpap1	1,13	Yes
ENSMUSG00000029104	1,43E-01	7,389253301	5,73E+00	1,66E-02	1,35E-01	Htt	1,10	No
ENSMUSG00000029106	-1,21E-01	7,877189479	4,28E+00	3,85E-02	2,33E-01	Add1	0,92	No
ENSMUSG00000029108	4,54E-01	3,498003371	2,04E+01	6,13E-06	2,17E-04	Pcdh7	1,37	Yes
ENSMUSG00000029110	6,36E-02	7,179350079	1,12E+00	2,91E-01	5,86E-01	Rnf4	1,05	No
ENSMUSG00000029111	8,15E-02	4,237229908	9,30E-01	3,35E-01	6,36E-01	Nelfa	1,06	No
ENSMUSG00000029119	1,18E-01	7,190170429	3,85E+00	4,97E-02	2,69E-01	Man2b2	1,09	No
ENSMUSG00000029120	1,12E+00	-2,655168239	1,19E+00	2,76E-01	5,73E-01	Ppp2r2c	2,17	No
ENSMUSG00000029121	-3,83E-02	-3,107651936	-7,91E-11	1,00E+00	1,00E+00	Crmp1	0,97	No
ENSMUSG00000029125	6,04E-02	3,803797229	4,18E-01	5,18E-01	7,98E-01	Stx18	1,04	No
ENSMUSG00000029126	-5,55E-02	0,276174878	4,29E-02	8,36E-01	9,80E-01	Nsg1	0,96	No
ENSMUSG00000029127	1,37E-01	3,035252828	1,52E+00	2,18E-01	5,50E-01	Zbtb49	1,10	No
ENSMUSG00000029128	5,55E-02	4,168196251	4,16E-01	5,19E-01	7,98E-01	Rab28	1,04	No
ENSMUSG00000029130	-2,50E-01	-1,740851608	1,23E-01	7,25E-01	9,18E-01	Rnf32	0,84	No
ENSMUSG00000029131	-1,60E-01	5,988073676	5,94E+00	1,48E-02	1,25E-01	Dnajb6	0,90	No
ENSMUSG00000029134	3,48E-01	-1,278711558	4,90E-01	4,84E-01	7,71E-01	Plb1	1,27	No
ENSMUSG00000029135	-1,02E-01	7,690265162	2,96E+00	8,55E-02	3,76E-01	Fosl2	0,93	No
ENSMUSG00000029136	-5,44E-01	1,602081874	1,07E+01	1,08E-03	1,72E-02	Rbks	0,69	Yes
ENSMUSG00000029138	1,35E+00	-1,912714423	3,94E+00	4,72E-02	2,60E-01	4930548H24Rik	2,54	No
ENSMUSG00000029141	3,18E-02	4,418056621	1,53E-01	6,95E-01	9,02E-01	Slc4a1ap	1,02	No
ENSMUSG00000029145	-1,04E-01	4,247246369	1,55E+00	2,13E-01	5,50E-01	Eif2b4	0,93	No
ENSMUSG00000029146	7,18E-02	5,989160303	1,20E+00	2,74E-01	5,70E-01	Snx17	1,05	No
ENSMUSG00000029147	5,58E-02	6,628330611	8,04E-01	3,70E-01	6,71E-01	Ppm1g	1,04	No
ENSMUSG00000029148	-6,61E-02	6,294289234	1,08E+00	2,98E-01	5,94E-01	Nrbp1	0,96	No
ENSMUSG00000029149	-9,00E-02	1,661109584	3,03E-01	5,82E-01	8,38E-01	Krtcap3	0,94	No
ENSMUSG00000029151	7,14E-01	-2,896146507	2,77E-01	5,98E-01	8,45E-01	Slc30a3	1,64	No
ENSMUSG00000029152	-2,20E-02	6,591917748	1,25E-01	7,23E-01	9,17E-01	Ociad1	0,98	No

ENSMUSG00000029153	-4,19E-01	-1,107606169	8,76E-01	3,49E-01	6,50E-01	Ociad2	0,75	No
ENSMUSG00000029154	-3,18E+00	-2,963258684	4,26E+00	3,90E-02	2,34E-01	Cwh43	0,11	No
ENSMUSG00000029156	4,83E-02	3,614661754	2,44E-01	6,21E-01	8,57E-01	Sgcb	1,03	No
ENSMUSG00000029158	4,19E-01	-2,600743634	1,37E-01	7,11E-01	9,10E-01	Yipf7	1,34	No
ENSMUSG00000029161	-5,29E-01	1,06288769	6,95E+00	8,39E-03	8,22E-02	Cgref1	0,69	No
ENSMUSG00000029162	-6,68E-02	3,281473758	4,04E-01	5,25E-01	8,02E-01	Khk	0,95	No
ENSMUSG00000029163	1,35E-01	6,40975102	4,51E+00	3,37E-02	2,16E-01	Emilin1	1,10	No
ENSMUSG00000029165	-3,54E-02	3,956011393	1,57E-01	6,92E-01	9,00E-01	Agbl5	0,98	No
ENSMUSG00000029166	5,84E-02	4,10152207	4,48E-01	5,03E-01	7,88E-01	Mapre3	1,04	No
ENSMUSG00000029167	-7,79E-01	-1,683192917	1,79E+00	1,81E-01	5,42E-01	Ppargc1a	0,58	No
ENSMUSG00000029168	-7,85E-01	4,454162521	9,73E+01	5,96E-23	1,84E-20	Dpysl5	0,58	Yes
ENSMUSG00000029169	-8,66E-02	7,778325695	2,18E+00	1,39E-01	4,71E-01	Dhx15	0,94	No
ENSMUSG00000029171	-4,93E-02	4,988954162	4,44E-01	5,05E-01	7,90E-01	Pgm1	0,97	No
ENSMUSG00000029173	-4,01E-01	5,676682138	3,56E+01	2,42E-09	1,85E-07	Sepsecs	0,76	Yes
ENSMUSG00000029174	-1,21E-01	6,255335991	3,62E+00	5,71E-02	2,94E-01	Tbc1d1	0,92	No
ENSMUSG00000029175	2,51E-01	7,328255265	1,75E+01	2,86E-05	8,42E-04	Slc35f6	1,19	Yes
ENSMUSG00000029176	-9,94E-03	4,735265957	1,68E-02	8,97E-01	1,00E+00	Anapc4	0,99	No
ENSMUSG00000029177	1,23E-01	6,728349402	3,96E+00	4,65E-02	2,57E-01	Cenpa	1,09	No
ENSMUSG00000029178	2,32E-01	5,306810889	1,09E+01	9,54E-04	1,56E-02	Klf3	1,17	Yes
ENSMUSG00000029179	1,02E-01	3,637447997	1,14E+00	2,85E-01	5,81E-01	Zcchc4	1,07	No
ENSMUSG00000029184	-3,76E-01	0,372320228	2,29E+00	1,30E-01	4,53E-01	Olfr109	0,77	No
ENSMUSG00000029185	5,29E-02	2,152137674	1,32E-01	7,17E-01	9,13E-01	Fam114a1	1,04	No
ENSMUSG00000029186	-2,24E-01	5,004574084	9,19E+00	2,43E-03	3,26E-02	Pi4k2b	0,86	Yes
ENSMUSG00000029188	-4,13E-02	-3,033659495	7,08E-04	9,79E-01	1,00E+00	Slc34a2	0,97	No
ENSMUSG00000029189	1,88E-01	-1,267937585	1,60E-01	6,89E-01	8,99E-01	Sel1l3	1,14	No
ENSMUSG00000029190	7,88E-02	5,764562063	1,38E+00	2,40E-01	5,50E-01	D5Ertd579e	1,06	No
ENSMUSG00000029191	1,22E-01	6,052711382	3,48E+00	6,20E-02	3,10E-01	Rfc1	1,09	No
ENSMUSG00000029192	-1,17E-01	6,569811988	3,51E+00	6,09E-02	3,06E-01	Tbc1d14	0,92	No
ENSMUSG00000029193	3,14E-02	-3,107617333	-7,69E-10	1,00E+00	1,00E+00	Cckar	1,02	No
ENSMUSG00000029195	-2,02E+00	-2,655085615	3,08E+00	7,92E-02	3,60E-01	Klb	0,25	No
ENSMUSG00000029196	8,82E-02	4,192474391	1,08E+00	2,99E-01	5,96E-01	Tada2b	1,06	No
ENSMUSG00000029198	2,45E-02	5,953160312	1,38E-01	7,10E-01	9,09E-01	Grpel1	1,02	No

ENSMUSG00000029199	-8,75E-02	4,085667081	1,01E+00	3,15E-01	6,16E-01	Lias	0,94	No
ENSMUSG00000029201	7,36E-02	6,06250074	1,28E+00	2,59E-01	5,51E-01	Ugdh	1,05	No
ENSMUSG00000029202	1,61E-02	7,474867918	7,36E-02	7,86E-01	9,52E-01	Pds5a	1,01	No
ENSMUSG00000029203	-6,92E-02	5,561467589	1,01E+00	3,14E-01	6,14E-01	Ube2k	0,95	No
ENSMUSG00000029204	1,70E-01	5,281732426	5,52E+00	1,88E-02	1,45E-01	Rhoh	1,12	No
ENSMUSG00000029205	-3,40E-01	-2,31122142	1,73E-01	6,78E-01	8,92E-01	Chrna9	0,79	No
ENSMUSG00000029206	-5,49E-01	-2,655091964	2,36E-01	6,27E-01	8,60E-01	Nsun7	0,68	No
ENSMUSG00000029207	2,38E-01	5,262872496	1,12E+01	8,28E-04	1,38E-02	Apbb2	1,18	Yes
ENSMUSG00000029208	3,05E-02	4,566956768	1,51E-01	6,98E-01	9,04E-01	Guf1	1,02	No
ENSMUSG00000029209	1,04E-01	3,883016406	1,32E+00	2,51E-01	5,50E-01	Gnpda2	1,07	No
ENSMUSG00000029211	1,21E+00	-2,896162599	9,04E-01	3,42E-01	6,42E-01	Gabra4	2,31	No
ENSMUSG00000029212	-2,79E-01	-0,215860516	7,76E-01	3,78E-01	6,79E-01	Gabrb1	0,82	No
ENSMUSG00000029213	4,96E-02	6,117424905	5,88E-01	4,43E-01	7,35E-01	Commd8	1,03	No
ENSMUSG00000029217	8,99E-02	6,051681981	1,91E+00	1,67E-01	5,20E-01	Tec	1,06	No
ENSMUSG00000029221	8,68E-02	5,465857557	1,57E+00	2,10E-01	5,50E-01	Slc30a9	1,06	No
ENSMUSG00000029223	3,79E-01	3,669927423	1,57E+01	7,33E-05	1,87E-03	Uchl1	1,30	Yes
ENSMUSG00000029227	-3,44E-02	5,650387992	2,54E-01	6,14E-01	8,53E-01	Fip1l1	0,98	No
ENSMUSG00000029228	2,99E-01	0,943261284	1,87E+00	1,72E-01	5,28E-01	Lnx1	1,23	No
ENSMUSG00000029229	2,50E-02	5,158207702	1,19E-01	7,30E-01	9,21E-01	Chic2	1,02	No
ENSMUSG00000029231	-4,07E-01	-1,497566785	6,06E-01	4,36E-01	7,29E-01	Pdgfra	0,75	No
ENSMUSG00000029233	1,97E-01	5,656578353	8,37E+00	3,81E-03	4,56E-02	Srd5a3	1,15	Yes
ENSMUSG00000029234	3,95E-02	6,570685352	4,02E-01	5,26E-01	8,03E-01	Tmem165	1,03	No
ENSMUSG00000029236	-6,12E-01	-2,831952993	2,96E-01	5,87E-01	8,40E-01	Nmu	0,65	No
ENSMUSG00000029238	1,15E-01	5,841979497	2,95E+00	8,58E-02	3,77E-01	Clock	1,08	No
ENSMUSG00000029245	1,82E+00	-3,033661385	1,31E+00	2,52E-01	5,50E-01	Epha5	3,54	No
ENSMUSG00000029246	8,73E-02	4,373650312	1,12E+00	2,91E-01	5,86E-01	Ppat	1,06	No
ENSMUSG00000029247	-4,99E-02	6,471299099	6,32E-01	4,27E-01	7,21E-01	Paics	0,97	No
ENSMUSG00000029248	3,43E-01	-1,800955608	3,13E-01	5,76E-01	8,34E-01	Thegl	1,27	No
ENSMUSG00000029249	5,49E-03	5,742013235	6,84E-03	9,34E-01	1,00E+00	Rest	1,00	No
ENSMUSG00000029250	4,33E-02	6,001566914	4,38E-01	5,08E-01	7,92E-01	Polr2b	1,03	No
ENSMUSG00000029253	2,55E-02	4,193403285	9,01E-02	7,64E-01	9,39E-01	Cenpc1	1,02	No
ENSMUSG00000029254	-3,38E-01	4,842715038	1,96E+01	9,66E-06	3,27E-04	Stap1	0,79	Yes

ENSMUSG00000029255	1,85E+00	-3,107657832	1,35E+00	2,46E-01	5,50E-01	Gnrhr	3,61	No
ENSMUSG00000029263	7,75E-02	3,51214197	6,14E-01	4,33E-01	7,27E-01	Pigg	1,06	No
ENSMUSG00000029265	5,34E-02	6,770224776	7,55E-01	3,85E-01	6,86E-01	Dr1	1,04	No
ENSMUSG00000029267	1,39E-01	5,048017595	3,60E+00	5,79E-02	2,97E-01	Mtf2	1,10	No
ENSMUSG00000029270	9,89E-02	4,986186346	1,77E+00	1,83E-01	5,45E-01	Fam69a	1,07	No
ENSMUSG00000029275	3,54E-01	1,245730225	3,43E+00	6,41E-02	3,17E-01	Gfi1	1,28	No
ENSMUSG00000029276	-1,50E-01	2,075840535	1,08E+00	2,99E-01	5,95E-01	Glmn	0,90	No
ENSMUSG00000029279	1,17E-01	0,710316197	2,76E-01	6,00E-01	8,46E-01	Brdt	1,08	No
ENSMUSG00000029283	4,00E-01	4,591800935	2,58E+01	3,70E-07	1,81E-05	Cdc7	1,32	Yes
ENSMUSG00000029286	-8,82E-01	-2,963247103	4,30E-01	5,12E-01	7,95E-01	Enam	0,54	No
ENSMUSG00000029287	2,02E-01	2,976462884	2,99E+00	8,37E-02	3,71E-01	Tgfbr3	1,15	No
ENSMUSG00000029290	1,80E-02	5,174872592	6,24E-02	8,03E-01	9,62E-01	Zfp326	1,01	No
ENSMUSG00000029291	-1,12E-01	4,961357857	2,25E+00	1,34E-01	4,60E-01	Rufy3	0,93	No
ENSMUSG00000029298	-3,30E-02	4,947220316	1,99E-01	6,56E-01	8,80E-01	Gbp9	0,98	No
ENSMUSG00000029299	6,53E-01	1,61102142	1,35E+01	2,36E-04	4,98E-03	Abcg3	1,57	Yes
ENSMUSG00000029304	3,41E-01	10,06874027	3,68E+01	1,34E-09	1,07E-07	Spp1	1,27	Yes
ENSMUSG00000029306	-1,94E+00	-3,107619243	1,50E+00	2,21E-01	5,50E-01	Ibsp	0,26	No
ENSMUSG00000029307	-2,66E+00	-3,033639428	2,81E+00	9,35E-02	3,83E-01	Dmp1	0,16	No
ENSMUSG00000029309	-1,94E+00	-3,107619243	1,50E+00	2,21E-01	5,50E-01	Sparcl1	0,26	No
ENSMUSG00000029310	-1,23E-01	5,570187187	3,24E+00	7,17E-02	3,40E-01	Nudt9	0,92	No
ENSMUSG00000029311	4,47E-02	6,496478883	5,08E-01	4,76E-01	7,64E-01	Hsd17b11	1,03	No
ENSMUSG00000029312	-2,37E-01	3,121920266	5,02E+00	2,51E-02	1,75E-01	Klhl8	0,85	No
ENSMUSG00000029313	2,62E-02	8,038683673	2,02E-01	6,53E-01	8,78E-01	Aff1	1,02	No
ENSMUSG00000029314	-5,49E-01	-0,946160388	1,62E+00	2,04E-01	5,50E-01	Gpat3	0,68	No
ENSMUSG00000029319	3,33E-02	3,467911763	1,11E-01	7,39E-01	9,26E-01	Coq2	1,02	No
ENSMUSG00000029321	-1,94E+00	-3,107619243	1,50E+00	2,21E-01	5,50E-01	Slc10a6	0,26	No
ENSMUSG00000029322	-6,06E-01	-1,401314909	1,65E+00	1,99E-01	5,50E-01	Plac8	0,66	No
ENSMUSG00000029326	1,15E-01	5,334531925	2,63E+00	1,05E-01	3,97E-01	Enoph1	1,08	No
ENSMUSG00000029328	5,31E-02	6,896056013	7,54E-01	3,85E-01	6,86E-01	Hnrnpdl	1,04	No
ENSMUSG00000029330	1,02E-01	4,279836518	1,43E+00	2,31E-01	5,50E-01	Cds1	1,07	No
ENSMUSG00000029334	3,51E+00	-2,71177175	5,33E+00	2,09E-02	1,56E-01	Prkg2	11,35	No
ENSMUSG00000029338	1,74E-01	5,978606228	7,07E+00	7,85E-03	7,79E-02	Antxr2	1,13	No

ENSMUSG00000029343	-3,09E-01	-2,401424848	1,43E-01	7,05E-01	9,07E-01	Crybb1	0,81	No
ENSMUSG00000029344	1,35E-01	5,071022092	3,35E+00	6,72E-02	3,26E-01	Tpst2	1,10	No
ENSMUSG00000029345	4,60E-02	5,822018569	4,79E-01	4,89E-01	7,76E-01	Tfip11	1,03	No
ENSMUSG00000029346	-1,36E-01	3,920377942	2,27E+00	1,32E-01	4,56E-01	Srrd	0,91	No
ENSMUSG00000029348	-8,42E-01	-2,896131633	3,89E-01	5,33E-01	8,06E-01	Asphd2	0,56	No
ENSMUSG00000029352	1,62E+00	-2,770658576	1,81E+00	1,79E-01	5,39E-01	Crybb3	3,08	No
ENSMUSG00000029359	-7,01E-01	1,24439205	1,51E+01	1,02E-04	2,45E-03	Tesc	0,62	Yes
ENSMUSG00000029360	1,31E-01	3,541926618	1,81E+00	1,78E-01	5,39E-01	Gm9754	1,10	No
ENSMUSG00000029361	-3,72E-02	-2,497646819	1,83E-03	9,66E-01	1,00E+00	Nos1	0,97	No
ENSMUSG00000029363	1,06E-01	5,225904303	2,17E+00	1,41E-01	4,74E-01	Rfc5	1,08	No
ENSMUSG00000029364	2,38E-02	6,694969364	1,48E-01	7,00E-01	9,04E-01	Wsb2	1,02	No
ENSMUSG00000029366	1,31E-01	7,079910826	4,65E+00	3,10E-02	2,03E-01	Dck	1,09	No
ENSMUSG00000029368	-3,83E-02	-3,107651936	-7,91E-11	1,00E+00	1,00E+00	Alb	0,97	No
ENSMUSG00000029370	-8,92E-01	-1,600759188	2,43E+00	1,19E-01	4,30E-01	Rassf6	0,54	No
ENSMUSG00000029371	-1,51E+00	-1,711552884	5,91E+00	1,50E-02	1,26E-01	Cxcl5	0,35	No
ENSMUSG00000029372	-9,41E-01	1,455496593	2,58E+01	3,86E-07	1,88E-05	Ppbp	0,52	Yes
ENSMUSG00000029373	-1,48E-01	4,116726887	2,90E+00	8,85E-02	3,79E-01	Pf4	0,90	No
ENSMUSG00000029375	9,01E-03	-3,107646452	-5,16E-10	1,00E+00	1,00E+00	Cxcl15	1,01	No
ENSMUSG00000029376	8,68E-02	2,232982633	3,91E-01	5,32E-01	8,06E-01	Mthfd2l	1,06	No
ENSMUSG00000029377	-9,50E-01	-2,497435104	7,90E-01	3,74E-01	6,75E-01	Ereg	0,52	No
ENSMUSG00000029378	2,09E-01	-1,088689795	2,14E-01	6,44E-01	8,72E-01	Areg	1,16	No
ENSMUSG00000029379	-1,12E+00	4,182603062	1,55E+02	1,36E-35	1,16E-32	Cxcl3	0,46	Yes
ENSMUSG00000029380	-6,64E-01	1,877240199	1,73E+01	3,15E-05	9,14E-04	Cxcl1	0,63	Yes
ENSMUSG00000029381	-7,99E-01	1,664563082	2,37E+01	1,13E-06	4,95E-05	Shroom3	0,57	Yes
ENSMUSG00000029384	-9,73E-01	-2,497528941	8,29E-01	3,63E-01	6,64E-01	2010109A12Rik	0,51	No
ENSMUSG00000029385	5,51E-02	5,388925808	6,28E-01	4,28E-01	7,22E-01	Ccng2	1,04	No
ENSMUSG00000029386	9,17E-01	-0,344578425	7,34E+00	6,73E-03	6,98E-02	Tctn2	1,89	No
ENSMUSG00000029387	-3,27E-03	3,174312588	9,29E-04	9,76E-01	1,00E+00	Gtf2h3	1,00	No
ENSMUSG00000029388	2,54E-02	4,402093155	9,65E-02	7,56E-01	9,35E-01	Eif2b1	1,02	No
ENSMUSG00000029389	-6,85E-02	4,012120552	6,09E-01	4,35E-01	7,28E-01	Ddx55	0,95	No
ENSMUSG00000029390	4,66E-02	7,448856292	6,14E-01	4,33E-01	7,27E-01	Tmed2	1,03	No
ENSMUSG00000029392	6,82E-02	2,010892562	2,05E-01	6,51E-01	8,76E-01	Rilpl1	1,05	No

ENSMUSG00000029394	-1,76E-02	5,787143928	6,95E-02	7,92E-01	9,55E-01	Cdk2ap1	0,99	No
ENSMUSG00000029397	-1,67E-01	5,450729475	5,81E+00	1,59E-02	1,31E-01	Rchy1	0,89	No
ENSMUSG00000029401	-2,22E-01	6,298676995	1,21E+01	5,01E-04	9,19E-03	Rilpl2	0,86	Yes
ENSMUSG00000029402	-9,48E-02	3,680128666	9,82E-01	3,22E-01	6,22E-01	Snrnp35	0,94	No
ENSMUSG00000029403	8,73E-02	4,711831595	1,25E+00	2,63E-01	5,58E-01	Cdkl2	1,06	No
ENSMUSG00000029404	-6,12E-02	4,843859519	6,51E-01	4,20E-01	7,15E-01	Arl6ip4	0,96	No
ENSMUSG00000029405	8,29E-02	7,188637975	1,90E+00	1,68E-01	5,22E-01	G3bp2	1,06	No
ENSMUSG00000029406	2,18E-01	1,58960721	1,51E+00	2,19E-01	5,50E-01	Pitpnm2	1,16	No
ENSMUSG00000029407	8,17E-02	6,79877757	1,77E+00	1,83E-01	5,46E-01	Uso1	1,06	No
ENSMUSG00000029408	5,21E-01	1,171856953	6,82E+00	9,04E-03	8,66E-02	Abcb9	1,43	No
ENSMUSG00000029409	8,13E-01	-2,711681644	5,75E-01	4,48E-01	7,39E-01	U90926	1,76	No
ENSMUSG00000029410	8,18E-01	4,016383161	8,45E+01	3,91E-20	8,94E-18	Ppef2	1,76	Yes
ENSMUSG00000029413	3,64E-01	9,609674033	4,12E+01	1,36E-10	1,26E-08	Naaa	1,29	Yes
ENSMUSG00000029414	1,60E-01	4,929128684	4,52E+00	3,36E-02	2,15E-01	Kntc1	1,12	No
ENSMUSG00000029415	3,20E-01	6,019806239	2,39E+01	1,03E-06	4,56E-05	Sdad1	1,25	Yes
ENSMUSG00000029416	-3,45E-02	5,748859956	2,64E-01	6,07E-01	8,50E-01	Slc15a4	0,98	No
ENSMUSG00000029417	-8,43E-01	-1,997964856	1,93E+00	1,65E-01	5,17E-01	Cxcl9	0,56	No
ENSMUSG00000029419	-1,57E+00	-2,627595555	1,63E+00	2,01E-01	5,50E-01	Gm996	0,34	No
ENSMUSG00000029422	-3,83E-02	6,008780741	3,42E-01	5,59E-01	8,22E-01	Rsrc2	0,97	No
ENSMUSG00000029426	2,79E-02	7,295966542	2,18E-01	6,41E-01	8,70E-01	Scarb2	1,02	No
ENSMUSG00000029427	6,02E-02	5,491348572	7,59E-01	3,84E-01	6,85E-01	Zcchc8	1,04	No
ENSMUSG00000029428	1,98E-01	6,265657104	9,60E+00	1,94E-03	2,74E-02	Stx2	1,15	Yes
ENSMUSG00000029430	1,26E-03	6,717481235	4,15E-04	9,84E-01	1,00E+00	Ran	1,00	No
ENSMUSG00000029431	-3,82E-01	0,490089022	2,53E+00	1,11E-01	4,10E-01	B3gnt4	0,77	No
ENSMUSG00000029432	-1,72E-02	5,350374031	5,98E-02	8,07E-01	9,64E-01	Nipsnap2	0,99	No
ENSMUSG00000029433	-2,10E-01	5,551010397	9,46E+00	2,10E-03	2,91E-02	Diablo	0,86	Yes
ENSMUSG00000029434	1,85E-01	6,266350944	8,42E+00	3,71E-03	4,48E-02	Vps33a	1,14	Yes
ENSMUSG00000029436	1,80E-01	-2,033553032	4,83E-02	8,26E-01	9,75E-01	Mmp17	1,13	No
ENSMUSG00000029438	-1,20E-01	2,613465627	9,23E-01	3,37E-01	6,37E-01	Bcl7a	0,92	No
ENSMUSG00000029439	6,14E-02	5,924835748	8,68E-01	3,51E-01	6,53E-01	Sfswap	1,04	No
ENSMUSG00000029440	6,01E-02	4,574301739	5,63E-01	4,53E-01	7,43E-01	Psmd9	1,04	No
ENSMUSG00000029442	-3,48E-01	-1,136418795	6,82E-01	4,09E-01	7,05E-01	Wdr66	0,79	No

ENSMUSG00000029446	-4,23E-02	3,661320691	1,93E-01	6,60E-01	8,82E-01	Psph	0,97	No
ENSMUSG00000029447	1,88E-02	7,057208932	9,71E-02	7,55E-01	9,34E-01	Cct6a	1,01	No
ENSMUSG00000029449	-2,51E-01	4,833668029	1,10E+01	9,02E-04	1,49E-02	Rhof	0,84	Yes
ENSMUSG00000029452	-2,61E-02	4,346603675	9,88E-02	7,53E-01	9,33E-01	Tmem116	0,98	No
ENSMUSG00000029454	-8,77E-02	4,11235953	1,03E+00	3,10E-01	6,08E-01	Mapkapk5	0,94	No
ENSMUSG00000029455	1,10E-02	7,761286262	3,51E-02	8,51E-01	9,90E-01	Aldh2	1,01	No
ENSMUSG00000029456	-2,85E-02	2,320911856	4,52E-02	8,32E-01	9,78E-01	Acad10	0,98	No
ENSMUSG00000029458	3,56E-02	6,35804515	3,15E-01	5,75E-01	8,34E-01	Brap	1,02	No
ENSMUSG00000029461	-3,73E-03	6,262636371	3,42E-03	9,53E-01	1,00E+00	Fam168a	1,00	No
ENSMUSG00000029462	-8,54E-02	6,427067687	1,83E+00	1,76E-01	5,35E-01	Vps29	0,94	No
ENSMUSG00000029463	-1,05E-01	1,686199866	3,82E-01	5,37E-01	8,08E-01	Fam216a	0,93	No
ENSMUSG00000029464	-6,91E-02	3,831568614	5,62E-01	4,53E-01	7,43E-01	Gpn3	0,95	No
ENSMUSG00000029465	-1,08E-01	7,259928609	3,26E+00	7,12E-02	3,38E-01	Arpc3	0,93	No
ENSMUSG00000029466	9,24E-02	4,867072576	1,50E+00	2,20E-01	5,50E-01	Anapc7	1,07	No
ENSMUSG00000029467	1,04E-01	7,967797115	3,16E+00	7,57E-02	3,50E-01	Atp2a2	1,07	No
ENSMUSG00000029468	4,27E-02	5,809247618	4,10E-01	5,22E-01	8,00E-01	P2rx7	1,03	No
ENSMUSG00000029469	2,02E-01	-1,22633501	2,08E-01	6,48E-01	8,75E-01	Ift81	1,15	No
ENSMUSG00000029470	-6,03E-02	7,329647443	1,02E+00	3,12E-01	6,12E-01	P2rx4	0,96	No
ENSMUSG00000029471	8,77E-02	6,370867387	1,91E+00	1,67E-01	5,20E-01	Camkk2	1,06	No
ENSMUSG00000029472	1,02E-01	6,873322849	2,77E+00	9,60E-02	3,87E-01	Anapc5	1,07	No
ENSMUSG00000029474	3,50E-04	5,173321858	2,37E-05	9,96E-01	1,00E+00	Rnf34	1,00	No
ENSMUSG00000029475	-5,77E-02	5,815895327	7,55E-01	3,85E-01	6,86E-01	Kdm2b	0,96	No
ENSMUSG00000029477	-3,46E-01	1,607666702	3,90E+00	4,83E-02	2,64E-01	Morn3	0,79	No
ENSMUSG00000029478	1,21E-01	7,418714332	4,16E+00	4,15E-02	2,42E-01	Ncor2	1,09	No
ENSMUSG00000029480	1,63E-02	4,624175319	4,29E-02	8,36E-01	9,80E-01	Dhx37	1,01	No
ENSMUSG00000029482	2,01E-01	5,683709786	8,84E+00	2,95E-03	3,79E-02	Aacs	1,15	Yes
ENSMUSG00000029484	-3,40E-01	7,3499036	3,25E+01	1,20E-08	8,24E-07	Anxa3	0,79	Yes
ENSMUSG00000029486	-1,31E-01	3,14477309	1,44E+00	2,30E-01	5,50E-01	Mrpl1	0,91	No
ENSMUSG00000029490	4,85E-02	4,723560159	3,96E-01	5,29E-01	8,05E-01	Mfsd7a	1,03	No
ENSMUSG00000029491	6,59E-01	-1,44863151	1,28E+00	2,58E-01	5,51E-01	Pde6b	1,58	No
ENSMUSG00000029499	-5,74E-01	1,824835753	1,39E+01	1,88E-04	4,13E-03	Pxmp2	0,67	Yes
ENSMUSG00000029500	5,35E-02	4,480720204	4,47E-01	5,04E-01	7,89E-01	Pgam5	1,04	No

ENSMUSG00000029501	8,93E-02	6,309125093	1,97E+00	1,60E-01	5,08E-01	Ankle2	1,06	No
ENSMUSG00000029502	1,08E-01	5,984818158	2,74E+00	9,79E-02	3,91E-01	Golga3	1,08	No
ENSMUSG00000029503	-8,90E-02	-2,107639497	1,28E-02	9,10E-01	1,00E+00	P2rx2	0,94	No
ENSMUSG00000029504	1,72E-02	4,522008278	4,57E-02	8,31E-01	9,77E-01	Ddx51	1,01	No
ENSMUSG00000029505	8,83E-02	7,256520482	2,17E+00	1,41E-01	4,74E-01	Ep400	1,06	No
ENSMUSG00000029507	-2,67E-02	4,686148255	1,16E-01	7,34E-01	9,23E-01	Pus1	0,98	No
ENSMUSG00000029510	3,92E-01	-0,2522571	1,51E+00	2,19E-01	5,50E-01	Gpc2	1,31	No
ENSMUSG00000029512	6,20E-03	5,234143828	7,52E-03	9,31E-01	1,00E+00	Ulk1	1,00	No
ENSMUSG00000029513	4,56E-02	5,00164326	3,79E-01	5,38E-01	8,09E-01	Prkab1	1,03	No
ENSMUSG00000029516	2,25E-01	4,82222718	8,68E+00	3,21E-03	4,04E-02	Cit	1,17	Yes
ENSMUSG00000029517	1,21E+00	-2,832024207	9,06E-01	3,41E-01	6,42E-01	Ankrd7	2,31	No
ENSMUSG00000029518	4,85E-02	7,029744225	6,42E-01	4,23E-01	7,18E-01	Rab35	1,03	No
ENSMUSG00000029521	2,04E-01	4,333991778	6,03E+00	1,41E-02	1,20E-01	Chek2	1,15	No
ENSMUSG00000029522	9,01E-03	-3,107646452	-5,16E-10	1,00E+00	1,00E+00	Pla2g1b	1,01	No
ENSMUSG00000029524	-1,26E-01	2,571363262	1,03E+00	3,10E-01	6,09E-01	Sirt4	0,92	No
ENSMUSG00000029528	7,48E-03	7,687759275	1,62E-02	8,99E-01	1,00E+00	Pxn	1,01	No
ENSMUSG00000029530	1,27E-01	-0,012784731	1,86E-01	6,67E-01	8,86E-01	Ccr9	1,09	No
ENSMUSG00000029534	2,83E-01	5,485826176	1,64E+01	5,14E-05	1,39E-03	St7	1,22	Yes
ENSMUSG00000029535	4,35E-02	5,297402389	3,78E-01	5,39E-01	8,09E-01	Triap1	1,03	No
ENSMUSG00000029536	-1,13E-02	4,782623137	2,18E-02	8,83E-01	1,00E+00	Gatc	0,99	No
ENSMUSG00000029538	1,88E-02	5,924008313	8,15E-02	7,75E-01	9,45E-01	Srsf9	1,01	No
ENSMUSG00000029541	-1,27E-01	-2,896123576	1,17E-02	9,14E-01	1,00E+00	Cyp2w1	0,92	No
ENSMUSG00000029544	-3,66E-02	-2,896156051	8,10E-04	9,77E-01	1,00E+00	Cabp1	0,97	No
ENSMUSG00000029545	-9,12E-03	5,344739193	1,68E-02	8,97E-01	1,00E+00	Acads	0,99	No
ENSMUSG00000029547	1,07E-01	6,221790426	2,76E+00	9,65E-02	3,88E-01	Ints1	1,08	No
ENSMUSG00000029550	5,96E-02	5,390231476	7,29E-01	3,93E-01	6,93E-01	Sppl3	1,04	No
ENSMUSG00000029551	-1,97E-01	3,279631706	3,55E+00	5,96E-02	3,02E-01	Psmg3	0,87	No
ENSMUSG00000029552	-3,71E-01	8,090275184	4,09E+01	1,62E-10	1,48E-08	Tes	0,77	Yes
ENSMUSG00000029553	2,71E-01	6,49259695	1,85E+01	1,66E-05	5,25E-04	Tfec	1,21	Yes
ENSMUSG00000029554	-8,46E-03	4,796064908	1,21E-02	9,12E-01	1,00E+00	Mad1l1	0,99	No
ENSMUSG00000029556	8,11E-01	-2,600686135	4,63E-01	4,96E-01	7,81E-01	Hnf1a	1,75	No
ENSMUSG00000029557	-3,73E-02	2,689981074	9,48E-02	7,58E-01	9,36E-01	Mrm2	0,97	No

ENSMUSG00000029559	-7,11E-02	4,102639325	6,64E-01	4,15E-01	7,10E-01	2210016L21Rik	0,95	No
ENSMUSG00000029560	1,31E-01	7,034300832	4,66E+00	3,10E-02	2,03E-01	Snx8	1,09	No
ENSMUSG00000029561	-2,13E-01	4,179769682	6,47E+00	1,10E-02	9,98E-02	Oasl2	0,86	No
ENSMUSG00000029569	9,35E-02	6,88343851	2,35E+00	1,26E-01	4,44E-01	Tmem168	1,07	No
ENSMUSG00000029570	-2,96E-02	6,585811756	2,28E-01	6,33E-01	8,65E-01	Lfng	0,98	No
ENSMUSG00000029571	-1,41E-02	6,148358153	4,76E-02	8,27E-01	9,76E-01	Tmem106b	0,99	No
ENSMUSG00000029575	6,40E-02	3,296262193	3,79E-01	5,38E-01	8,09E-01	Mmab	1,05	No
ENSMUSG00000029576	5,60E-02	2,448610982	1,80E-01	6,72E-01	8,89E-01	Radil	1,04	No
ENSMUSG00000029577	8,19E-02	6,688418907	1,76E+00	1,85E-01	5,49E-01	Ube3b	1,06	No
ENSMUSG00000029578	2,01E-02	5,690726321	8,96E-02	7,65E-01	9,39E-01	Wipi2	1,01	No
ENSMUSG00000029580	-9,19E-02	12,30676656	2,72E+00	9,91E-02	3,92E-01	Actb	0,94	No
ENSMUSG00000029581	-9,11E-01	8,708448587	2,50E+02	2,59E-56	1,11E-52	Fscn1	0,53	Yes
ENSMUSG00000029587	-2,31E-02	3,553583612	5,69E-02	8,11E-01	9,67E-01	Zfp12	0,98	No
ENSMUSG00000029591	3,46E-01	3,529890412	1,20E+01	5,44E-04	9,84E-03	Ung	1,27	Yes
ENSMUSG00000029592	7,44E-02	4,904670706	9,87E-01	3,20E-01	6,21E-01	Usp30	1,05	No
ENSMUSG00000029594	2,42E-01	3,985971414	7,34E+00	6,75E-03	7,00E-02	Rbm19	1,18	No
ENSMUSG00000029595	1,26E+00	-2,770612189	9,82E-01	3,22E-01	6,22E-01	Lhx5	2,39	No
ENSMUSG00000029596	8,95E-01	-1,655141883	1,77E+00	1,84E-01	5,48E-01	Sdsl	1,86	No
ENSMUSG00000029597	7,68E-01	-1,257710393	2,28E+00	1,31E-01	4,55E-01	Sds	1,70	No
ENSMUSG00000029598	1,70E-01	6,430474976	7,17E+00	7,42E-03	7,49E-02	Plbd2	1,12	No
ENSMUSG00000029599	-1,00E-03	6,417994668	2,53E-04	9,87E-01	1,00E+00	Ddx54	1,00	No
ENSMUSG00000029600	-1,67E-02	2,271470047	1,35E-02	9,07E-01	1,00E+00	Rita1	0,99	No
ENSMUSG00000029601	-6,10E-01	-0,937529913	1,93E+00	1,65E-01	5,18E-01	Iqcd	0,66	No
ENSMUSG00000029602	-6,05E-01	2,08393318	1,84E+01	1,78E-05	5,57E-04	Rasal1	0,66	Yes
ENSMUSG00000029603	-7,37E-01	-0,069959706	6,45E+00	1,11E-02	1,01E-01	Dtx1	0,60	No
ENSMUSG00000029605	8,20E-02	1,281080627	1,97E-01	6,57E-01	8,80E-01	Oas1b	1,06	No
ENSMUSG00000029607	-2,45E-01	2,333986868	3,13E+00	7,69E-02	3,54E-01	Ankrd61	0,84	No
ENSMUSG00000029608	2,89E-02	-1,401357317	2,63E-03	9,59E-01	1,00E+00	Rph3a	1,02	No
ENSMUSG00000029610	2,16E-01	4,008874098	5,92E+00	1,49E-02	1,25E-01	Aimp2	1,16	No
ENSMUSG00000029613	1,07E-01	6,224023547	2,76E+00	9,68E-02	3,88E-01	Eif2ak1	1,08	No
ENSMUSG00000029614	-2,26E-01	6,709297813	1,35E+01	2,43E-04	5,11E-03	Rpl6	0,85	Yes
ENSMUSG00000029616	-8,05E-03	7,979385351	1,91E-02	8,90E-01	1,00E+00	Erp29	0,99	No

ENSMUSG00000029617	-7,98E-02	5,468114426	1,32E+00	2,50E-01	5,50E-01	Ccz1	0,95	No
ENSMUSG00000029621	-2,23E-02	6,07870667	1,18E-01	7,32E-01	9,22E-01	Arpc1a	0,98	No
ENSMUSG00000029622	7,87E-02	8,784393948	1,90E+00	1,68E-01	5,23E-01	Arpc1b	1,06	No
ENSMUSG00000029623	-3,31E-02	5,681937478	2,39E-01	6,25E-01	8,59E-01	Pdap1	0,98	No
ENSMUSG00000029624	-9,68E-02	5,163151459	1,83E+00	1,76E-01	5,35E-01	Ptcd1	0,94	No
ENSMUSG00000029625	-3,00E-02	4,782971085	1,56E-01	6,93E-01	9,01E-01	Cpsf4	0,98	No
ENSMUSG00000029627	-8,49E-02	3,140106988	6,25E-01	4,29E-01	7,23E-01	Zkscan14	0,94	No
ENSMUSG00000029629	6,16E-02	4,799568095	6,50E-01	4,20E-01	7,15E-01	Phf14	1,04	No
ENSMUSG00000029632	-8,61E-02	4,877388731	1,30E+00	2,54E-01	5,50E-01	Ndufa4	0,94	No
ENSMUSG00000029633	7,03E-02	5,816166796	1,11E+00	2,93E-01	5,88E-01	Gm5578	1,05	No
ENSMUSG00000029634	5,52E-02	6,036611221	7,14E-01	3,98E-01	6,98E-01	Rnf6	1,04	No
ENSMUSG00000029635	7,69E-02	5,643728719	1,28E+00	2,57E-01	5,50E-01	Cdk8	1,05	No
ENSMUSG00000029638	-9,71E-02	3,818090897	1,15E+00	2,84E-01	5,80E-01	Glcci1	0,93	No
ENSMUSG00000029640	-9,67E-02	6,606760425	2,42E+00	1,20E-01	4,30E-01	Usp12	0,94	No
ENSMUSG00000029641	1,29E-01	-2,070130265	3,70E-02	8,47E-01	9,88E-01	Rasl11a	1,09	No
ENSMUSG00000029642	-5,88E-02	5,977141082	8,04E-01	3,70E-01	6,71E-01	Polr1d	0,96	No
ENSMUSG00000029647	-3,99E-02	6,331125474	3,97E-01	5,29E-01	8,04E-01	Pan3	0,97	No
ENSMUSG00000029648	4,60E-01	4,720860982	3,45E+01	4,21E-09	3,10E-07	Flt1	1,38	Yes
ENSMUSG00000029649	-9,30E-02	6,10644109	2,06E+00	1,51E-01	4,93E-01	Pomp	0,94	No
ENSMUSG00000029650	-2,90E-02	4,798115959	1,46E-01	7,02E-01	9,05E-01	Slc46a3	0,98	No
ENSMUSG00000029651	4,09E-02	0,970980766	3,76E-02	8,46E-01	9,87E-01	Mtus2	1,03	No
ENSMUSG00000029655	-3,62E-03	5,423506516	2,69E-03	9,59E-01	1,00E+00	N4bp2l2	1,00	No
ENSMUSG00000029656	-3,83E-02	-3,107651936	-7,91E-11	1,00E+00	1,00E+00	C8b	0,97	No
ENSMUSG00000029657	-1,12E-01	5,694747905	2,79E+00	9,49E-02	3,86E-01	Hsph1	0,93	No
ENSMUSG00000029658	-6,52E-02	-2,355526694	5,88E-03	9,39E-01	1,00E+00	Wdr95	0,96	No
ENSMUSG00000029659	-1,21E+00	-3,107627547	6,96E-01	4,04E-01	7,01E-01	Medag	0,43	No
ENSMUSG00000029660	4,45E-01	-2,600697781	1,54E-01	6,94E-01	9,02E-01	Tex26	1,36	No
ENSMUSG00000029661	-1,96E+00	-0,164849413	3,04E+01	3,56E-08	2,18E-06	Col1a2	0,26	Yes
ENSMUSG00000029664	-5,71E-01	-1,711801441	7,13E-01	3,99E-01	6,98E-01	Tfpi2	0,67	No
ENSMUSG00000029669	-7,69E-02	-2,963220349	2,69E-03	9,59E-01	1,00E+00	Tspan12	0,95	No
ENSMUSG00000029670	9,25E-02	3,92067186	1,04E+00	3,08E-01	6,06E-01	Ing3	1,07	No
ENSMUSG00000029671	1,13E-01	-0,641325321	9,50E-02	7,58E-01	9,35E-01	Wnt16	1,08	No

ENSMUSG00000029672	1,68E-01	5,981197511	6,52E+00	1,06E-02	9,78E-02	Fam3c	1,12	No
ENSMUSG00000029673	-8,26E-01	-0,832084513	4,32E+00	3,77E-02	2,31E-01	Auts2	0,56	No
ENSMUSG00000029674	9,01E-02	5,82444536	1,83E+00	1,76E-01	5,35E-01	Limk1	1,06	No
ENSMUSG00000029676	1,55E-01	3,867776533	2,99E+00	8,39E-02	3,71E-01	Pot1a	1,11	No
ENSMUSG00000029681	6,56E-03	4,520197322	6,69E-03	9,35E-01	1,00E+00	Bcl7b	1,00	No
ENSMUSG00000029683	1,81E+00	-3,10765737	1,28E+00	2,58E-01	5,50E-01	Lmod2	3,49	No
ENSMUSG00000029684	-1,46E-01	5,082859055	3,96E+00	4,65E-02	2,57E-01	Wasl	0,90	No
ENSMUSG00000029685	9,01E-03	-3,107646452	-5,16E-10	1,00E+00	1,00E+00	Asb15	1,01	No
ENSMUSG00000029686	2,51E-02	6,540424633	1,62E-01	6,88E-01	8,98E-01	Cul1	1,02	No
ENSMUSG00000029687	9,80E-02	5,242117596	1,92E+00	1,66E-01	5,19E-01	Ezh2	1,07	No
ENSMUSG00000029699	5,76E-01	1,147835417	8,31E+00	3,94E-03	4,69E-02	Ssc4d	1,49	Yes
ENSMUSG00000029700	-9,89E-01	-1,311188054	4,04E+00	4,43E-02	2,51E-01	Slc13a1	0,50	No
ENSMUSG00000029701	6,38E-02	5,31542707	8,07E-01	3,69E-01	6,70E-01	Rbm28	1,05	No
ENSMUSG00000029703	5,29E-02	5,295201151	5,59E-01	4,55E-01	7,44E-01	Lrwd1	1,04	No
ENSMUSG00000029705	1,65E-01	6,519682745	6,91E+00	8,58E-03	8,35E-02	Cux1	1,12	No
ENSMUSG00000029707	-1,88E-01	-2,333243765	5,50E-02	8,15E-01	9,68E-01	Fscn3	0,88	No
ENSMUSG00000029708	6,79E-02	4,432093798	7,11E-01	3,99E-01	6,98E-01	Gcc1	1,05	No
ENSMUSG00000029710	-1,36E+00	-2,832027061	1,15E+00	2,83E-01	5,79E-01	Ephb4	0,39	No
ENSMUSG00000029711	3,75E-02	2,031569547	6,20E-02	8,03E-01	9,62E-01	Epo	1,03	No
ENSMUSG00000029712	3,10E-01	-2,448675378	9,77E-02	7,55E-01	9,34E-01	Actl6b	1,24	No
ENSMUSG00000029713	8,90E-02	8,340567944	2,38E+00	1,23E-01	4,38E-01	Gnb2	1,06	No
ENSMUSG00000029714	5,56E-02	5,677227206	6,80E-01	4,09E-01	7,06E-01	Gigyf1	1,04	No
ENSMUSG00000029715	-3,00E-02	3,935851605	1,08E-01	7,43E-01	9,28E-01	Pop7	0,98	No
ENSMUSG00000029716	7,61E-01	-1,655145017	1,75E+00	1,85E-01	5,49E-01	Tfr2	1,69	No
ENSMUSG00000029718	-2,54E-01	1,14229476	1,75E+00	1,86E-01	5,50E-01	Pcolce	0,84	No
ENSMUSG00000029720	-1,01E-01	6,639100815	2,68E+00	1,02E-01	3,94E-01	Gm20605	0,93	No
ENSMUSG00000029722	1,57E-01	5,516456616	5,17E+00	2,30E-02	1,66E-01	Agfg2	1,11	No
ENSMUSG00000029723	1,03E-01	6,376547901	2,62E+00	1,06E-01	3,98E-01	Tsc22d4	1,07	No
ENSMUSG00000029725	-1,48E-01	3,625113984	2,34E+00	1,26E-01	4,44E-01	Ppp1r35	0,90	No
ENSMUSG00000029726	6,48E-02	5,011474026	7,85E-01	3,76E-01	6,77E-01	Mepce	1,05	No
ENSMUSG00000029727	-7,57E-01	-1,627694419	1,83E+00	1,76E-01	5,35E-01	Cyp3a13	0,59	No
ENSMUSG00000029729	1,04E-01	4,288020577	1,56E+00	2,12E-01	5,50E-01	Zkscan1	1,08	No

ENSMUSG00000029730	2,07E-01	5,126984503	8,10E+00	4,42E-03	5,09E-02	Mcm7	1,15	No
ENSMUSG00000029735	1,52E-02	3,621958218	2,53E-02	8,74E-01	1,00E+00	Tpk1	1,01	No
ENSMUSG00000029736	2,63E+00	-2,963275836	2,67E+00	1,02E-01	3,94E-01	Nobox	6,19	No
ENSMUSG00000029752	1,01E-01	3,519696564	1,03E+00	3,09E-01	6,07E-01	Asns	1,07	No
ENSMUSG00000029754	-1,94E+00	-3,107619243	1,50E+00	2,21E-01	5,50E-01	Dlx6	0,26	No
ENSMUSG00000029757	-1,89E+00	-3,1076441	1,42E+00	2,33E-01	5,50E-01	Dync1i1	0,27	No
ENSMUSG00000029759	5,46E-02	5,056758807	5,47E-01	4,59E-01	7,48E-01	Pon3	1,04	No
ENSMUSG00000029761	3,63E-01	3,215045644	1,12E+01	8,38E-04	1,40E-02	Cald1	1,29	Yes
ENSMUSG00000029762	-2,27E-02	1,214460326	1,36E-02	9,07E-01	1,00E+00	Akr1b8	0,98	No
ENSMUSG00000029763	-6,88E-02	6,670128905	1,23E+00	2,67E-01	5,63E-01	Exoc4	0,95	No
ENSMUSG00000029765	-7,44E-01	0,851320911	1,15E+01	6,92E-04	1,20E-02	Plxna4	0,60	Yes
ENSMUSG00000029767	8,33E-02	7,201064042	1,92E+00	1,66E-01	5,18E-01	Calu	1,06	No
ENSMUSG00000029769	-7,46E-01	-1,477090741	1,99E+00	1,58E-01	5,05E-01	Ccdc136	0,60	No
ENSMUSG00000029771	-4,25E-02	8,888998261	5,55E-01	4,56E-01	7,46E-01	Irf5	0,97	No
ENSMUSG00000029772	-7,16E-02	7,249929731	1,44E+00	2,31E-01	5,50E-01	Ahcyl2	0,95	No
ENSMUSG00000029775	7,80E-02	6,048856767	1,43E+00	2,32E-01	5,50E-01	Klhdc10	1,06	No
ENSMUSG00000029776	-1,08E-01	5,96189348	2,71E+00	9,96E-02	3,92E-01	Hibadh	0,93	No
ENSMUSG00000029777	8,41E-02	6,754606752	1,87E+00	1,72E-01	5,29E-01	Gars	1,06	No
ENSMUSG00000029778	-1,38E+00	-2,896135155	1,20E+00	2,74E-01	5,71E-01	Adcyap1r1	0,38	No
ENSMUSG00000029780	-2,06E-02	4,1563599	5,79E-02	8,10E-01	9,66E-01	Nt5c3	0,99	No
ENSMUSG00000029781	-7,42E-02	2,915869613	4,04E-01	5,25E-01	8,02E-01	Fkbp9	0,95	No
ENSMUSG00000029782	1,76E-01	4,482430481	4,79E+00	2,87E-02	1,92E-01	Tmem209	1,13	No
ENSMUSG00000029784	1,85E+00	-3,107657832	1,35E+00	2,46E-01	5,50E-01	Ssmem1	3,61	No
ENSMUSG00000029787	3,01E-02	5,942545332	2,09E-01	6,47E-01	8,75E-01	Avl9	1,02	No
ENSMUSG00000029788	1,81E+00	-3,10765737	1,28E+00	2,58E-01	5,50E-01	Cpa5	3,49	No
ENSMUSG00000029790	1,66E-01	2,144055562	1,33E+00	2,49E-01	5,50E-01	Cep41	1,12	No
ENSMUSG00000029797	-3,75E-01	3,863127635	1,70E+01	3,69E-05	1,05E-03	Sspo	0,77	Yes
ENSMUSG00000029798	-4,19E-01	4,941354008	3,24E+01	1,27E-08	8,65E-07	Herc6	0,75	Yes
ENSMUSG00000029802	1,44E-01	2,926343055	1,57E+00	2,11E-01	5,50E-01	Abcg2	1,11	No
ENSMUSG00000029804	-9,78E-02	4,265244492	1,37E+00	2,41E-01	5,50E-01	Herc3	0,93	No
ENSMUSG00000029810	-6,57E-01	6,303087579	1,07E+02	3,96E-25	1,39E-22	Tmem176b	0,63	Yes
ENSMUSG00000029811	-1,24E+00	2,38010976	8,74E+01	8,69E-21	2,08E-18	Aoc1	0,42	Yes

ENSMUSG00000029814	2,65E-02	2,742662085	4,60E-02	8,30E-01	9,77E-01	Igf2bp3	1,02	No
ENSMUSG00000029815	-1,56E-02	3,821363419	2,90E-02	8,65E-01	9,98E-01	Malsu1	0,99	No
ENSMUSG00000029816	-3,16E-01	11,85355917	3,21E+01	1,48E-08	9,81E-07	Gpnmb	0,80	Yes
ENSMUSG00000029817	-8,14E-02	6,49940845	1,69E+00	1,93E-01	5,50E-01	Tra2a	0,95	No
ENSMUSG00000029819	1,17E-01	1,771327436	5,40E-01	4,62E-01	7,51E-01	Npy	1,08	No
ENSMUSG00000029821	1,15E-01	4,207363807	1,80E+00	1,79E-01	5,40E-01	Dfna5	1,08	No
ENSMUSG00000029822	-5,87E-03	5,034303559	6,25E-03	9,37E-01	1,00E+00	Osbpl3	1,00	No
ENSMUSG00000029823	-2,27E-02	7,852882755	1,51E-01	6,98E-01	9,04E-01	Luc7l2	0,98	No
ENSMUSG00000029826	-1,32E-02	7,465395251	5,00E-02	8,23E-01	9,73E-01	Zc3hav1	0,99	No
ENSMUSG00000029828	1,05E+00	-2,355660455	1,42E+00	2,33E-01	5,50E-01	4921507P07Rik	2,07	No
ENSMUSG00000029830	-1,14E-02	-3,033635021	1,50E-05	9,97E-01	1,00E+00	Svopl	0,99	No
ENSMUSG00000029832	-2,53E-02	0,897016524	1,42E-02	9,05E-01	1,00E+00	Nfe2l3	0,98	No
ENSMUSG00000029833	-2,62E-01	4,926458891	1,25E+01	4,11E-04	7,78E-03	Trim24	0,83	Yes
ENSMUSG00000029836	1,28E-02	4,822936481	2,83E-02	8,66E-01	9,99E-01	Cbx3	1,01	No
ENSMUSG00000029840	1,56E-01	9,219353552	7,55E+00	6,01E-03	6,43E-02	Mtpn	1,11	No
ENSMUSG00000029843	-3,20E+00	-2,896154564	4,32E+00	3,77E-02	2,31E-01	Slc13a4	0,11	No
ENSMUSG00000029844	8,65E-01	-0,533559519	5,66E+00	1,73E-02	1,37E-01	Hoxa1	1,82	No
ENSMUSG00000029847	1,82E+00	-1,247131959	1,15E+01	7,04E-04	1,21E-02	Slc23a4	3,54	Yes
ENSMUSG00000029848	-7,32E-02	-2,033584538	1,25E-02	9,11E-01	1,00E+00	Stra8	0,95	No
ENSMUSG00000029851	-8,02E-01	2,301792773	3,77E+01	8,38E-10	6,94E-08	Tcaf2	0,57	Yes
ENSMUSG00000029859	4,17E-01	2,995441483	1,33E+01	2,72E-04	5,60E-03	Epha1	1,34	Yes
ENSMUSG00000029860	2,52E-01	10,1580286	2,00E+01	7,56E-06	2,61E-04	Zyx	1,19	Yes
ENSMUSG00000029861	3,34E-01	-0,175683269	1,16E+00	2,82E-01	5,79E-01	Fam131b	1,26	No
ENSMUSG00000029862	5,02E-01	-1,015742876	1,14E+00	2,86E-01	5,82E-01	Clcn1	1,42	No
ENSMUSG00000029863	1,96E-01	4,936679249	6,93E+00	8,49E-03	8,28E-02	Casp2	1,15	No
ENSMUSG00000029864	6,99E-02	0,899454995	1,10E-01	7,40E-01	9,27E-01	Gstk1	1,05	No
ENSMUSG00000029869	2,64E-01	-1,963174724	1,45E-01	7,04E-01	9,06E-01	Ephb6	1,20	No
ENSMUSG00000029875	-2,33E+00	-1,145933983	2,44E+01	7,87E-07	3,60E-05	Ccdc184	0,20	Yes
ENSMUSG00000029878	4,74E-01	-2,832010425	1,77E-01	6,74E-01	8,90E-01	Dbpht2	1,39	No
ENSMUSG00000029883	-1,89E+00	-3,1076441	1,42E+00	2,33E-01	5,50E-01	1700074P13Rik	0,27	No
ENSMUSG00000029885	1,25E+00	-2,832026261	9,70E-01	3,25E-01	6,25E-01	Moxd2	2,38	No
ENSMUSG00000029909	-5,49E-01	-2,655145816	2,37E-01	6,27E-01	8,60E-01	Prss37	0,68	No

ENSMUSG00000029910	1,90E-01	5,260151115	7,08E+00	7,79E-03	7,74E-02	Mad2l1	1,14	No
ENSMUSG00000029911	-1,26E-01	3,400278856	1,51E+00	2,19E-01	5,50E-01	Ssbp1	0,92	No
ENSMUSG00000029913	-8,16E-01	-1,088752632	3,02E+00	8,24E-02	3,69E-01	Prdm5	0,57	No
ENSMUSG00000029915	1,27E-01	6,035670187	3,71E+00	5,40E-02	2,84E-01	Clec5a	1,09	No
ENSMUSG00000029916	5,00E-02	2,843894039	1,82E-01	6,70E-01	8,88E-01	Agk	1,04	No
ENSMUSG00000029918	5,42E-03	3,51238464	3,05E-03	9,56E-01	1,00E+00	Mrps33	1,00	No
ENSMUSG00000029919	1,45E-01	4,550837232	3,32E+00	6,86E-02	3,29E-01	Hpgds	1,11	No
ENSMUSG00000029920	1,74E-01	4,968080576	5,48E+00	1,93E-02	1,47E-01	Smarcad1	1,13	No
ENSMUSG00000029922	1,20E-02	6,965639657	3,86E-02	8,44E-01	9,86E-01	Mkrn1	1,01	No
ENSMUSG00000029923	1,88E-01	3,94479003	4,30E+00	3,81E-02	2,32E-01	Rab19	1,14	No
ENSMUSG00000029924	3,65E-01	4,936465388	2,37E+01	1,10E-06	4,84E-05	Slc37a3	1,29	Yes
ENSMUSG00000029925	1,86E-01	7,962335674	1,01E+01	1,46E-03	2,19E-02	Tbxas1	1,14	Yes
ENSMUSG00000029992	-3,06E-01	6,193238628	2,29E+01	1,73E-06	7,17E-05	Gfpt1	0,81	Yes
ENSMUSG00000029993	1,12E-01	3,613784112	1,34E+00	2,47E-01	5,50E-01	Nfu1	1,08	No
ENSMUSG00000029994	3,68E-02	8,895433856	4,18E-01	5,18E-01	7,98E-01	Anxa4	1,03	No
ENSMUSG00000029998	5,44E-02	6,589168166	7,60E-01	3,83E-01	6,85E-01	Pcyox1	1,04	No
ENSMUSG00000029999	1,25E+00	-1,344350825	4,48E+00	3,43E-02	2,18E-01	Tgfa	2,38	No
ENSMUSG00000030000	-3,65E-01	-0,954732226	7,05E-01	4,01E-01	6,99E-01	Add2	0,78	No
ENSMUSG00000030002	-1,32E-02	7,242037661	4,88E-02	8,25E-01	9,74E-01	Dusp11	0,99	No
ENSMUSG00000030007	-2,32E-02	7,022508572	1,46E-01	7,02E-01	9,05E-01	Cct7	0,98	No
ENSMUSG00000030008	-3,37E-02	3,171043113	9,65E-02	7,56E-01	9,35E-01	Pradc1	0,98	No
ENSMUSG00000030016	9,22E-02	6,226187085	2,09E+00	1,48E-01	4,88E-01	Zfp638	1,07	No
ENSMUSG00000030019	1,79E-01	5,411333021	6,50E+00	1,08E-02	9,89E-02	Fbxl14	1,13	No
ENSMUSG00000030020	4,50E-01	2,207389133	9,70E+00	1,84E-03	2,62E-02	Prickle2	1,37	Yes
ENSMUSG00000030022	-1,75E+00	-0,954484861	1,75E+01	2,93E-05	8,58E-04	Adamts9	0,30	Yes
ENSMUSG00000030029	-1,05E+00	1,168323636	2,78E+01	1,33E-07	7,17E-06	Lrig1	0,48	Yes
ENSMUSG00000030030	5,95E-01	0,103692279	4,08E+00	4,34E-02	2,48E-01	1700003E16Rik	1,51	No
ENSMUSG00000030031	2,12E-01	1,001843237	1,10E+00	2,93E-01	5,89E-01	Kbtbd8	1,16	No
ENSMUSG00000030032	4,10E-01	1,567622172	5,56E+00	1,84E-02	1,43E-01	Wdr54	1,33	No
ENSMUSG00000030034	8,69E-03	3,927145581	9,16E-03	9,24E-01	1,00E+00	Ino80b	1,01	No
ENSMUSG00000030035	-9,42E-02	5,109503932	1,68E+00	1,95E-01	5,50E-01	Wbp1	0,94	No
ENSMUSG00000030036	5,95E-02	6,09361016	8,41E-01	3,59E-01	6,61E-01	Mogs	1,04	No

ENSMUSG00000030037	-5,51E-02	2,765528451	2,09E-01	6,47E-01	8,75E-01	Mrpl53	0,96	No
ENSMUSG00000030041	-2,81E-01	2,12876457	3,92E+00	4,77E-02	2,62E-01	M1ap	0,82	No
ENSMUSG00000030042	9,80E-02	4,525723453	1,51E+00	2,20E-01	5,50E-01	Pole4	1,07	No
ENSMUSG00000030045	5,62E-02	4,689984042	5,25E-01	4,69E-01	7,57E-01	Mrpl19	1,04	No
ENSMUSG00000030046	4,70E-01	-2,226236577	2,99E-01	5,84E-01	8,38E-01	Bmp10	1,38	No
ENSMUSG00000030047	2,20E-01	6,905815013	1,29E+01	3,21E-04	6,39E-03	Arhgap25	1,16	Yes
ENSMUSG00000030051	9,77E-02	2,921289814	7,23E-01	3,95E-01	6,96E-01	Aplf	1,07	No
ENSMUSG00000030054	-8,88E-02	-1,831994252	2,21E-02	8,82E-01	1,00E+00	Gp9	0,94	No
ENSMUSG00000030055	5,15E-02	6,870049645	7,10E-01	3,99E-01	6,98E-01	Rab43	1,04	No
ENSMUSG00000030056	-1,59E-01	4,794357999	4,30E+00	3,82E-02	2,32E-01	Isy1	0,90	No
ENSMUSG00000030057	-1,01E-01	7,992400373	3,03E+00	8,19E-02	3,68E-01	Cnbp	0,93	No
ENSMUSG00000030058	-9,93E-03	6,875534556	2,64E-02	8,71E-01	1,00E+00	Copg1	0,99	No
ENSMUSG00000030059	1,11E-01	5,691069541	2,67E+00	1,03E-01	3,95E-01	Tmf1	1,08	No
ENSMUSG00000030060	-1,24E-01	3,678507048	1,70E+00	1,92E-01	5,50E-01	Hmces	0,92	No
ENSMUSG00000030061	-1,95E-02	4,971636151	6,86E-02	7,93E-01	9,56E-01	Uba3	0,99	No
ENSMUSG00000030062	8,52E-02	8,489409685	2,20E+00	1,38E-01	4,70E-01	Rpn1	1,06	No
ENSMUSG00000030064	3,37E-01	4,781809264	1,88E+01	1,45E-05	4,66E-04	Frmd4b	1,26	Yes
ENSMUSG00000030067	-1,39E-01	5,466336192	4,00E+00	4,54E-02	2,54E-01	Foxp1	0,91	No
ENSMUSG00000030068	2,08E-01	1,759601216	1,64E+00	2,00E-01	5,50E-01	Gm20696	1,16	No
ENSMUSG00000030069	1,37E+00	-2,54821431	1,92E+00	1,66E-01	5,18E-01	Prok2	2,59	No
ENSMUSG00000030074	-3,15E-02	-2,770572643	5,40E-04	9,81E-01	1,00E+00	Gxylt2	0,98	No
ENSMUSG00000030077	-2,54E+00	-1,600536198	1,67E+01	4,45E-05	1,23E-03	Chl1	0,17	Yes
ENSMUSG00000030079	-2,17E-01	2,894321446	3,48E+00	6,22E-02	3,10E-01	Ruvbl1	0,86	No
ENSMUSG00000030082	5,30E-02	7,824689189	8,21E-01	3,65E-01	6,66E-01	Sec61a1	1,04	No
ENSMUSG00000030083	8,90E-02	5,261663693	1,56E+00	2,11E-01	5,50E-01	Abtb1	1,06	No
ENSMUSG00000030084	2,09E-01	9,370211438	1,36E+01	2,29E-04	4,85E-03	Plxna1	1,16	Yes
ENSMUSG00000030086	7,42E-02	2,762563318	3,86E-01	5,34E-01	8,07E-01	Chchd6	1,05	No
ENSMUSG00000030087	-1,34E+00	-2,711670455	1,12E+00	2,89E-01	5,85E-01	Klf15	0,40	No
ENSMUSG00000030088	1,47E-01	4,262209366	3,02E+00	8,24E-02	3,69E-01	Aldh1l1	1,11	No
ENSMUSG00000030089	2,38E-01	4,187621642	7,64E+00	5,71E-03	6,17E-02	Slc41a3	1,18	No
ENSMUSG00000030091	-2,26E-01	6,61163838	1,33E+01	2,63E-04	5,45E-03	Nup210	0,85	Yes
ENSMUSG00000030094	8,99E-02	3,943983379	1,02E+00	3,12E-01	6,12E-01	Xpc	1,06	No

ENSMUSG00000030095	1,91E-01	6,52920746	9,25E+00	2,35E-03	3,19E-02	Tmem43	1,14	Yes
ENSMUSG00000030096	-5,42E-02	9,689293339	9,24E-01	3,36E-01	6,37E-01	Slc6a6	0,96	No
ENSMUSG00000030098	4,91E-01	-0,19602889	2,49E+00	1,15E-01	4,19E-01	Grip2	1,41	No
ENSMUSG00000030101	1,17E-02	6,356374351	3,42E-02	8,53E-01	9,91E-01	Sumf1	1,01	No
ENSMUSG00000030102	5,80E-02	5,76992283	7,44E-01	3,88E-01	6,89E-01	Itpr1	1,04	No
ENSMUSG00000030103	-2,78E-01	10,03060816	2,44E+01	7,84E-07	3,59E-05	Bhlhe40	0,82	Yes
ENSMUSG00000030104	-1,72E-02	8,633920912	9,08E-02	7,63E-01	9,39E-01	Edem1	0,99	No
ENSMUSG00000030105	1,03E-01	8,168831642	3,18E+00	7,48E-02	3,48E-01	Arl8b	1,07	No
ENSMUSG00000030107	-4,77E-01	4,171504188	3,22E+01	1,42E-08	9,45E-07	Usp18	0,72	Yes
ENSMUSG00000030108	-3,09E-01	2,568404218	5,91E+00	1,50E-02	1,26E-01	Slc6a13	0,81	No
ENSMUSG00000030109	-8,42E-02	5,206417785	1,39E+00	2,38E-01	5,50E-01	Slc6a12	0,94	No
ENSMUSG00000030110	-1,60E-01	-1,145985651	1,11E-01	7,39E-01	9,26E-01	Ret	0,89	No
ENSMUSG00000030117	8,12E-01	-2,311239038	7,92E-01	3,73E-01	6,74E-01	Gdf3	1,76	No
ENSMUSG00000030120	4,97E-02	6,941673943	6,64E-01	4,15E-01	7,10E-01	Mlf2	1,04	No
ENSMUSG00000030122	9,08E-02	7,909516028	2,42E+00	1,20E-01	4,31E-01	Ptms	1,06	No
ENSMUSG00000030123	-3,55E-02	9,746311122	3,97E-01	5,29E-01	8,04E-01	Plxnd1	0,98	No
ENSMUSG00000030124	2,80E-01	1,490026882	2,47E+00	1,16E-01	4,21E-01	Lag3	1,21	No
ENSMUSG00000030125	6,95E-01	-2,963260828	2,60E-01	6,10E-01	8,51E-01	Lrrc23	1,62	No
ENSMUSG00000030126	-5,70E-02	8,330285507	9,78E-01	3,23E-01	6,23E-01	Tmcc1	0,96	No
ENSMUSG00000030127	4,82E-02	5,083270065	4,39E-01	5,08E-01	7,92E-01	Cops7a	1,03	No
ENSMUSG00000030134	-1,05E-01	2,503617849	6,70E-01	4,13E-01	7,09E-01	Rasgef1a	0,93	No
ENSMUSG00000030137	-7,40E-01	1,712186837	2,10E+01	4,62E-06	1,68E-04	Tuba8	0,60	Yes
ENSMUSG00000030138	1,50E-01	6,507338003	5,69E+00	1,70E-02	1,36E-01	Bms1	1,11	No
ENSMUSG00000030142	-2,44E-01	5,397943771	1,20E+01	5,22E-04	9,51E-03	Clec4e	0,84	Yes
ENSMUSG00000030144	-6,62E-02	5,919771248	9,89E-01	3,20E-01	6,21E-01	Clec4d	0,96	No
ENSMUSG00000030145	-6,27E-03	0,632696932	7,69E-04	9,78E-01	1,00E+00	Zfp248	1,00	No
ENSMUSG00000030147	2,64E-01	3,724511951	7,65E+00	5,69E-03	6,15E-02	Clec4b1	1,20	No
ENSMUSG00000030148	3,41E-01	7,08789032	3,14E+01	2,07E-08	1,34E-06	Clec4a2	1,27	Yes
ENSMUSG00000030149	-4,15E-01	4,159023449	2,34E+01	1,34E-06	5,76E-05	Klrk1	0,75	Yes
ENSMUSG00000030154	3,88E-01	2,989537627	1,17E+01	6,25E-04	1,10E-02	Klrb1f	1,31	Yes
ENSMUSG00000030155	9,01E-03	-3,107646452	-5,16E-10	1,00E+00	1,00E+00	Clec2e	1,01	No
ENSMUSG00000030156	-2,88E-01	4,966832642	1,56E+01	7,85E-05	1,98E-03	Cd69	0,82	Yes

ENSMUSG00000030157	-2,40E-01	4,535027957	9,30E+00	2,30E-03	3,13E-02	Clec2d	0,85	Yes
ENSMUSG00000030158	7,16E-01	-2,401418105	7,45E-01	3,88E-01	6,88E-01	Clec12b	1,64	No
ENSMUSG00000030159	-1,22E-01	3,572394368	1,59E+00	2,07E-01	5,50E-01	Clec1b	0,92	No
ENSMUSG00000030160	-6,26E-01	-0,912464211	2,21E+00	1,37E-01	4,68E-01	Tmem52b	0,65	No
ENSMUSG00000030161	5,65E-03	6,792937397	8,45E-03	9,27E-01	1,00E+00	Gabarapl1	1,00	No
ENSMUSG00000030162	4,50E-02	5,310542358	4,05E-01	5,25E-01	8,02E-01	Olr1	1,03	No
ENSMUSG00000030165	-1,96E-01	0,309736524	5,58E-01	4,55E-01	7,45E-01	Klrd1	0,87	No
ENSMUSG00000030166	-3,81E-02	2,497651272	9,11E-02	7,63E-01	9,38E-01	Rad52	0,97	No
ENSMUSG00000030167	-1,87E+00	-3,107637761	1,39E+00	2,38E-01	5,50E-01	Klrc1	0,27	No
ENSMUSG00000030168	6,02E-04	8,097746815	1,08E-04	9,92E-01	1,00E+00	Adipor2	1,00	No
ENSMUSG00000030170	6,95E-01	-2,963260828	2,60E-01	6,10E-01	8,51E-01	Wnt5b	1,62	No
ENSMUSG00000030172	8,80E-02	4,536662604	1,23E+00	2,68E-01	5,65E-01	Erc1	1,06	No
ENSMUSG00000030177	8,67E-02	3,296377495	6,88E-01	4,07E-01	7,03E-01	Ccdc77	1,06	No
ENSMUSG00000030178	-5,88E-02	-3,033652696	1,62E-03	9,68E-01	1,00E+00	Klra13-ps	0,96	No
ENSMUSG00000030180	-2,00E-02	6,612419494	1,03E-01	7,48E-01	9,30E-01	Kdm5a	0,99	No
ENSMUSG00000030187	2,83E-01	3,217598917	6,75E+00	9,38E-03	8,90E-02	Klra2	1,22	No
ENSMUSG00000030188	2,43E-02	2,129507795	2,76E-02	8,68E-01	1,00E+00	Magohb	1,02	No
ENSMUSG00000030189	1,24E-01	6,942087223	4,12E+00	4,24E-02	2,45E-01	Ybx3	1,09	No
ENSMUSG00000030199	-1,33E-01	7,424358422	5,03E+00	2,50E-02	1,75E-01	Etv6	0,91	No
ENSMUSG00000030200	-4,64E-01	1,750091386	9,09E+00	2,58E-03	3,41E-02	Bcl2l14	0,72	Yes
ENSMUSG00000030201	1,00E-01	7,508077943	2,84E+00	9,17E-02	3,82E-01	Lrp6	1,07	No
ENSMUSG00000030203	-2,15E-01	5,790924896	1,04E+01	1,27E-03	1,96E-02	Dusp16	0,86	Yes
ENSMUSG00000030204	-2,15E-02	5,722151208	1,02E-01	7,50E-01	9,31E-01	Ddx47	0,99	No
ENSMUSG00000030205	-1,04E+00	-1,800950427	2,39E+00	1,22E-01	4,37E-01	Gprc5d	0,49	No
ENSMUSG00000030206	6,06E-01	-0,75576117	2,41E+00	1,20E-01	4,32E-01	Gsg1	1,52	No
ENSMUSG00000030207	2,69E-01	4,765830579	1,21E+01	5,04E-04	9,23E-03	Fam234b	1,21	Yes
ENSMUSG00000030208	-7,63E-02	6,032805194	1,34E+00	2,48E-01	5,50E-01	Emp1	0,95	No
ENSMUSG00000030209	9,01E-03	-3,107646452	-5,16E-10	1,00E+00	1,00E+00	Grin2b	1,01	No
ENSMUSG00000030213	1,78E-02	6,10014512	7,64E-02	7,82E-01	9,50E-01	Atf7ip	1,01	No
ENSMUSG00000030214	1,87E-01	7,964989132	1,02E+01	1,38E-03	2,08E-02	Plbd1	1,14	Yes
ENSMUSG00000030216	3,39E-02	6,873535467	3,06E-01	5,80E-01	8,37E-01	Wbp11	1,02	No
ENSMUSG00000030217	-7,98E-01	-2,311050566	9,26E-01	3,36E-01	6,37E-01	Art4	0,58	No

ENSMUSG00000030218	-4,86E-01	-1,86367251	5,70E-01	4,50E-01	7,40E-01	Mgp	0,71	No
ENSMUSG00000030219	4,13E-01	-0,816537389	1,08E+00	2,99E-01	5,95E-01	Erp27	1,33	No
ENSMUSG00000030220	4,91E-02	8,064904195	7,15E-01	3,98E-01	6,98E-01	Arhgdib	1,03	No
ENSMUSG00000030222	-5,67E-01	0,477190834	5,70E+00	1,69E-02	1,36E-01	Rerg	0,67	No
ENSMUSG00000030223	1,90E-01	5,295072031	7,12E+00	7,63E-03	7,64E-02	Ptpro	1,14	No
ENSMUSG00000030224	6,93E-03	6,682785639	1,25E-02	9,11E-01	1,00E+00	Strap	1,00	No
ENSMUSG00000030225	1,08E-01	4,748833062	1,99E+00	1,58E-01	5,05E-01	Dera	1,08	No
ENSMUSG00000030226	1,87E+00	-3,107639691	1,38E+00	2,40E-01	5,50E-01	Lmo3	3,67	No
ENSMUSG00000030231	1,50E-01	4,490287232	3,49E+00	6,17E-02	3,09E-01	Plekha5	1,11	No
ENSMUSG00000030232	-2,34E-01	7,087847595	1,50E+01	1,05E-04	2,53E-03	Aebp2	0,85	Yes
ENSMUSG00000030243	1,75E-01	4,989326315	5,57E+00	1,83E-02	1,42E-01	Recql	1,13	No
ENSMUSG00000030244	2,57E+00	-3,0336723	2,56E+00	1,10E-01	4,06E-01	Gys2	5,96	No
ENSMUSG00000030245	1,93E-01	5,87007451	8,44E+00	3,68E-03	4,45E-02	Golt1b	1,14	Yes
ENSMUSG00000030246	1,48E-01	3,238772111	1,90E+00	1,68E-01	5,22E-01	Ldhb	1,11	No
ENSMUSG00000030249	-1,94E+00	-3,107619243	1,50E+00	2,21E-01	5,50E-01	Abcc9	0,26	No
ENSMUSG00000030254	5,37E-02	4,075515601	3,78E-01	5,39E-01	8,09E-01	Rad18	1,04	No
ENSMUSG00000030255	1,04E+00	-1,247149395	4,34E+00	3,73E-02	2,30E-01	Sspn	2,05	No
ENSMUSG00000030256	-4,99E-02	5,705327823	5,46E-01	4,60E-01	7,49E-01	Bhlhe41	0,97	No
ENSMUSG00000030257	2,26E-01	4,282206855	7,21E+00	7,27E-03	7,38E-02	Srgap3	1,17	No
ENSMUSG00000030259	-1,11E-01	6,11939735	2,96E+00	8,53E-02	3,75E-01	Rassf8	0,93	No
ENSMUSG00000030263	1,62E-01	6,735898576	6,84E+00	8,92E-03	8,58E-02	Lrmp	1,12	No
ENSMUSG00000030264	3,14E-02	4,947932019	1,79E-01	6,73E-01	8,90E-01	Thumpd3	1,02	No
ENSMUSG00000030265	-1,75E-03	5,57878843	6,52E-04	9,80E-01	1,00E+00	Kras	1,00	No
ENSMUSG00000030268	-1,44E-01	-0,963368271	1,09E-01	7,42E-01	9,27E-01	Bcat1	0,91	No
ENSMUSG00000030269	-1,86E-01	6,102586667	8,31E+00	3,95E-03	4,69E-02	Mtmr14	0,88	Yes
ENSMUSG00000030270	-8,39E-02	-1,522627979	2,47E-02	8,75E-01	1,00E+00	Cpne9	0,94	No
ENSMUSG00000030271	2,32E-01	3,269072198	4,84E+00	2,79E-02	1,89E-01	Ogg1	1,17	No
ENSMUSG00000030272	2,48E-01	5,46118025	1,26E+01	3,82E-04	7,37E-03	Camk1	1,19	Yes
ENSMUSG00000030275	-1,43E-01	6,77634318	5,46E+00	1,94E-02	1,48E-01	Etnk1	0,91	No
ENSMUSG00000030276	1,76E-02	6,30249231	7,64E-02	7,82E-01	9,50E-01	Ttll3	1,01	No
ENSMUSG00000030278	4,31E-01	-2,770577482	1,44E-01	7,04E-01	9,06E-01	Cidec	1,35	No
ENSMUSG00000030279	1,08E-01	5,379969373	2,36E+00	1,25E-01	4,42E-01	C2cd5	1,08	No

ENSMUSG00000030281	1,40E-01	-0,548166437	1,60E-01	6,89E-01	8,99E-01	Il17rc	1,10	No
ENSMUSG00000030282	2,04E-01	5,668101812	8,97E+00	2,75E-03	3,58E-02	Cmas	1,15	Yes
ENSMUSG00000030283	4,13E-01	2,506013958	1,00E+01	1,57E-03	2,31E-02	St8sia1	1,33	Yes
ENSMUSG00000030284	1,22E-01	4,684450072	2,41E+00	1,20E-01	4,32E-01	Creld1	1,09	No
ENSMUSG00000030286	8,87E-02	6,224433605	1,91E+00	1,67E-01	5,20E-01	Emc3	1,06	No
ENSMUSG00000030287	2,71E-02	5,762048572	1,63E-01	6,86E-01	8,97E-01	Itpr2	1,02	No
ENSMUSG00000030291	-9,44E-02	4,754405141	1,51E+00	2,20E-01	5,50E-01	Med21	0,94	No
ENSMUSG00000030298	-4,51E-02	6,081343013	4,82E-01	4,88E-01	7,75E-01	Sec13	0,97	No
ENSMUSG00000030301	-3,86E-02	3,661785226	1,63E-01	6,87E-01	8,97E-01	Ccdc91	0,97	No
ENSMUSG00000030302	-7,49E-01	-0,980449502	3,31E+00	6,88E-02	3,30E-01	Atp2b2	0,60	No
ENSMUSG00000030303	-1,22E-01	3,982454548	1,86E+00	1,73E-01	5,29E-01	Far2	0,92	No
ENSMUSG00000030304	-6,98E-02	5,455541535	1,00E+00	3,16E-01	6,17E-01	Ergic2	0,95	No
ENSMUSG00000030306	-5,11E-01	-2,107603209	4,19E-01	5,18E-01	7,98E-01	Tmtc1	0,70	No
ENSMUSG00000030307	-3,72E-01	-2,355616718	1,74E-01	6,76E-01	8,91E-01	Slc6a11	0,77	No
ENSMUSG00000030309	-2,55E-01	3,323394529	6,30E+00	1,21E-02	1,07E-01	Caprin2	0,84	No
ENSMUSG00000030310	-1,01E+00	-2,107519672	1,91E+00	1,67E-01	5,20E-01	Slc6a1	0,50	No
ENSMUSG00000030313	-5,79E-01	0,779340857	6,06E+00	1,38E-02	1,19E-01	Dennd5b	0,67	No
ENSMUSG00000030314	7,92E-02	6,717373337	1,64E+00	2,00E-01	5,50E-01	Atg7	1,06	No
ENSMUSG00000030315	1,55E-01	5,078895854	4,56E+00	3,27E-02	2,11E-01	Vgll4	1,11	No
ENSMUSG00000030316	1,08E-01	2,456561266	6,99E-01	4,03E-01	7,01E-01	Tamm41	1,08	No
ENSMUSG00000030317	-3,88E-03	-3,03362684	4,02E-12	1,00E+00	1,00E+00	Timp4	1,00	No
ENSMUSG00000030319	-2,66E-01	4,324854757	1,03E+01	1,36E-03	2,07E-02	Cand2	0,83	Yes
ENSMUSG00000030321	-1,11E-01	-0,289517274	1,14E-01	7,36E-01	9,24E-01	Efcab12	0,93	No
ENSMUSG00000030322	3,00E-01	3,810085045	1,07E+01	1,06E-03	1,69E-02	Mbd4	1,23	Yes
ENSMUSG00000030323	2,35E-01	3,856014775	6,42E+00	1,13E-02	1,02E-01	Ift122	1,18	No
ENSMUSG00000030324	1,36E-02	-1,785658201	4,16E-04	9,84E-01	1,00E+00	Rho	1,01	No
ENSMUSG00000030325	4,76E-01	-0,648238095	1,55E+00	2,13E-01	5,50E-01	Klrb1c	1,39	No
ENSMUSG00000030327	-2,35E-02	5,801691171	1,22E-01	7,27E-01	9,19E-01	Necap1	0,98	No
ENSMUSG00000030329	6,06E-01	3,379688462	3,40E+01	5,64E-09	4,11E-07	Pianp	1,52	Yes
ENSMUSG00000030330	-4,12E-02	4,530525149	2,71E-01	6,02E-01	8,48E-01	Ing4	0,97	No
ENSMUSG00000030335	1,20E-02	5,198840175	2,76E-02	8,68E-01	1,00E+00	Mrpl51	1,01	No
ENSMUSG00000030336	1,09E-01	0,062040686	1,47E-01	7,01E-01	9,05E-01	Cd27	1,08	No

ENSMUSG00000030337	1,31E-02	3,974615584	2,15E-02	8,83E-01	1,00E+00	Vamp1	1,01	No
ENSMUSG00000030339	1,61E-01	6,091040347	6,11E+00	1,34E-02	1,16E-01	Ltbr	1,12	No
ENSMUSG00000030340	4,96E-01	0,444977328	3,28E+00	7,01E-02	3,34E-01	Scnn1a	1,41	No
ENSMUSG00000030341	1,47E-01	6,995476775	5,84E+00	1,57E-02	1,30E-01	Tnfrsf1a	1,11	No
ENSMUSG00000030342	6,92E-02	8,061706999	1,42E+00	2,34E-01	5,50E-01	Cd9	1,05	No
ENSMUSG00000030344	-3,81E-01	-1,711755841	4,90E-01	4,84E-01	7,71E-01	Akap3	0,77	No
ENSMUSG00000030345	1,11E+00	-2,655150212	1,18E+00	2,78E-01	5,75E-01	Dyrk4	2,16	No
ENSMUSG00000030346	1,20E-01	4,511340173	2,26E+00	1,33E-01	4,59E-01	Rad51ap1	1,09	No
ENSMUSG00000030347	1,72E-01	5,406476112	6,09E+00	1,36E-02	1,17E-01	D6Wsu163e	1,13	No
ENSMUSG00000030351	-3,88E-03	-3,03362684	4,02E-12	1,00E+00	1,00E+00	Tspan11	1,00	No
ENSMUSG00000030352	1,29E+00	-0,785882883	9,81E+00	1,73E-03	2,49E-02	Tspan9	2,45	Yes
ENSMUSG00000030353	3,14E-02	-3,107617333	-7,69E-10	1,00E+00	1,00E+00	Tead4	1,02	No
ENSMUSG00000030357	1,83E-01	5,600890396	7,10E+00	7,69E-03	7,68E-02	Fkbp4	1,13	No
ENSMUSG00000030359	1,08E+00	-0,904376896	6,63E+00	1,01E-02	9,38E-02	Pzp	2,12	No
ENSMUSG00000030361	-1,23E-01	1,233158932	4,05E-01	5,24E-01	8,01E-01	Klrb1a	0,92	No
ENSMUSG00000030364	-6,04E-01	-1,711625451	9,62E-01	3,27E-01	6,28E-01	Clec2h	0,66	No
ENSMUSG00000030365	-2,58E-01	5,505861293	1,41E+01	1,73E-04	3,84E-03	Clec2i	0,84	Yes
ENSMUSG00000030374	5,31E-02	6,353880656	7,03E-01	4,02E-01	7,00E-01	Strn4	1,04	No
ENSMUSG00000030376	-4,99E-01	-1,033401217	1,41E+00	2,35E-01	5,50E-01	Slc8a2	0,71	No
ENSMUSG00000030380	2,98E-01	0,103877075	1,13E+00	2,87E-01	5,83E-01	Mzf1	1,23	No
ENSMUSG00000030382	6,25E-01	-2,44868796	4,36E-01	5,09E-01	7,92E-01	Slc27a5	1,54	No
ENSMUSG00000030386	-8,89E-02	2,277623391	4,34E-01	5,10E-01	7,93E-01	Zfp606	0,94	No
ENSMUSG00000030393	5,19E-01	-0,669209459	1,99E+00	1,58E-01	5,05E-01	Zik1	1,43	No
ENSMUSG00000030397	-1,28E-01	4,926421614	2,92E+00	8,74E-02	3,78E-01	Mark4	0,92	No
ENSMUSG00000030399	3,59E-01	-1,322169294	4,85E-01	4,86E-01	7,73E-01	Ckm	1,28	No
ENSMUSG00000030400	6,87E-02	4,305730259	6,77E-01	4,11E-01	7,07E-01	Ercc2	1,05	No
ENSMUSG00000030401	-2,20E-01	-1,333172149	2,04E-01	6,52E-01	8,77E-01	Rtn2	0,86	No
ENSMUSG00000030402	4,84E-01	-1,770476005	5,89E-01	4,43E-01	7,34E-01	Ppm1n	1,40	No
ENSMUSG00000030403	2,25E-02	8,196967205	1,51E-01	6,98E-01	9,04E-01	Vasp	1,02	No
ENSMUSG00000030406	-4,94E-02	-2,268185578	4,48E-03	9,47E-01	1,00E+00	Gipr	0,97	No
ENSMUSG00000030407	2,14E-02	5,063787008	8,55E-02	7,70E-01	9,42E-01	Qpctl	1,01	No
ENSMUSG00000030409	5,12E-01	3,626102988	2,68E+01	2,26E-07	1,17E-05	Dmpk	1,43	Yes

ENSMUSG00000030410	7,18E-02	4,070173089	6,61E-01	4,16E-01	7,11E-01	Dmwd	1,05	No
ENSMUSG00000030411	-1,37E+00	-2,770535185	1,18E+00	2,77E-01	5,74E-01	Nova2	0,39	No
ENSMUSG00000030413	-9,48E-01	2,276516858	4,71E+01	6,73E-12	7,33E-10	Pglyrp1	0,52	Yes
ENSMUSG00000030417	-1,30E-01	2,684942368	1,10E+00	2,95E-01	5,91E-01	Pdcd5	0,91	No
ENSMUSG00000030421	-1,62E-01	5,582445948	5,64E+00	1,76E-02	1,39E-01	Uri1	0,89	No
ENSMUSG00000030423	-7,51E-02	5,531291093	1,19E+00	2,75E-01	5,72E-01	Pop4	0,95	No
ENSMUSG00000030424	1,81E-01	1,053766061	7,87E-01	3,75E-01	6,76E-01	Zfp939	1,13	No
ENSMUSG00000030427	3,69E-01	4,648783798	2,19E+01	2,85E-06	1,11E-04	Lilra6	1,29	Yes
ENSMUSG00000030428	-1,77E-01	-2,424911764	4,86E-02	8,26E-01	9,74E-01	Ttyh1	0,88	No
ENSMUSG00000030431	2,52E-01	2,790981413	4,55E+00	3,28E-02	2,12E-01	Tmem238	1,19	No
ENSMUSG00000030432	-1,45E-01	3,856840342	2,54E+00	1,11E-01	4,09E-01	Rpl28	0,90	No
ENSMUSG00000030433	7,72E-01	0,603900645	1,03E+01	1,36E-03	2,07E-02	Sbk2	1,71	Yes
ENSMUSG00000030435	-3,50E-02	6,692618729	3,21E-01	5,71E-01	8,32E-01	U2af2	0,98	No
ENSMUSG00000030443	6,86E-02	0,609867279	8,99E-02	7,64E-01	9,39E-01	Zfp583	1,05	No
ENSMUSG00000030446	6,11E-01	0,693792083	7,59E+00	5,87E-03	6,31E-02	Zfp273	1,53	No
ENSMUSG00000030447	-1,29E-01	7,793793048	4,84E+00	2,79E-02	1,89E-01	Cyfip1	0,91	No
ENSMUSG00000030451	7,63E-02	7,523464667	1,66E+00	1,98E-01	5,50E-01	Herc2	1,05	No
ENSMUSG00000030452	5,65E-02	5,884960336	7,27E-01	3,94E-01	6,94E-01	Nipa2	1,04	No
ENSMUSG00000030465	1,81E-01	3,91929991	4,01E+00	4,52E-02	2,54E-01	Psd3	1,13	No
ENSMUSG00000030468	-2,41E-01	4,775077663	1,01E+01	1,51E-03	2,24E-02	Siglecg	0,85	Yes
ENSMUSG00000030469	1,11E-01	2,526573126	7,73E-01	3,79E-01	6,80E-01	Zfp719	1,08	No
ENSMUSG00000030470	2,96E-01	-2,655172721	8,88E-02	7,66E-01	9,40E-01	Csrp3	1,23	No
ENSMUSG00000030471	6,32E-02	4,68374371	6,62E-01	4,16E-01	7,11E-01	Zdhhc13	1,04	No
ENSMUSG00000030472	6,04E-01	-2,401408796	4,07E-01	5,24E-01	8,01E-01	Ceacam18	1,52	No
ENSMUSG00000030474	4,71E-02	2,068357099	1,00E-01	7,52E-01	9,32E-01	Siglece	1,03	No
ENSMUSG00000030484	1,81E+00	-3,10765737	1,28E+00	2,58E-01	5,50E-01	Lypd5	3,49	No
ENSMUSG00000030486	3,34E-01	-0,350076938	1,05E+00	3,05E-01	6,02E-01	Zfp108	1,26	No
ENSMUSG00000030491	3,52E+00	-2,655199196	5,37E+00	2,05E-02	1,54E-01	Tdrd12	11,45	No
ENSMUSG00000030493	8,68E-02	3,462408996	7,43E-01	3,89E-01	6,89E-01	Faap24	1,06	No
ENSMUSG00000030494	1,21E+00	-1,800808134	1,70E+00	1,93E-01	5,50E-01	Rhpn2	2,32	No
ENSMUSG00000030495	-1,92E+00	-3,0336536	1,46E+00	2,27E-01	5,50E-01	Slc7a10	0,26	No
ENSMUSG00000030498	-1,85E-01	1,702186723	1,28E+00	2,57E-01	5,50E-01	Gas2	0,88	No

ENSMUSG00000030499	-1,89E+00	-3,1076441	1,42E+00	2,33E-01	5,50E-01	Kctd15	0,27	No
ENSMUSG00000030500	9,01E-03	-3,107646452	-5,16E-10	1,00E+00	1,00E+00	Slc17a6	1,01	No
ENSMUSG00000030505	2,10E-01	4,107445512	5,83E+00	1,57E-02	1,30E-01	Prmt3	1,16	No
ENSMUSG00000030509	1,40E-01	3,840839407	2,36E+00	1,25E-01	4,42E-01	Asb7	1,10	No
ENSMUSG00000030510	1,57E+00	-2,311173878	2,70E+00	1,00E-01	3,92E-01	Cers3	2,98	No
ENSMUSG00000030512	-1,49E-02	4,305492683	3,14E-02	8,59E-01	9,95E-01	Snrpa1	0,99	No
ENSMUSG00000030513	1,87E+00	-3,107639691	1,38E+00	2,40E-01	5,50E-01	Pcsk6	3,67	No
ENSMUSG00000030515	1,38E-01	3,532818777	1,96E+00	1,61E-01	5,10E-01	Tarsl2	1,10	No
ENSMUSG00000030516	3,09E-02	3,026776632	7,60E-02	7,83E-01	9,50E-01	Tjp1	1,02	No
ENSMUSG00000030518	1,81E+00	-3,10765737	1,28E+00	2,58E-01	5,50E-01	Fam189a1	3,49	No
ENSMUSG00000030521	1,16E-02	4,75574494	2,28E-02	8,80E-01	1,00E+00	Mphosph10	1,01	No
ENSMUSG00000030522	9,93E-02	4,375564052	1,39E+00	2,38E-01	5,50E-01	Mtmr10	1,07	No
ENSMUSG00000030523	-8,52E-01	-2,896118986	3,98E-01	5,28E-01	8,04E-01	Trpm1	0,55	No
ENSMUSG00000030527	-6,22E-02	6,146401964	9,23E-01	3,37E-01	6,37E-01	Crtc3	0,96	No
ENSMUSG00000030528	2,12E-01	4,795466907	7,58E+00	5,91E-03	6,34E-02	Blm	1,16	No
ENSMUSG00000030530	5,39E-01	8,870036092	8,80E+01	6,55E-21	1,60E-18	Furin	1,45	Yes
ENSMUSG00000030532	2,13E-01	3,759921379	5,13E+00	2,35E-02	1,68E-01	Hddc3	1,16	No
ENSMUSG00000030533	3,88E-02	5,879068662	3,42E-01	5,59E-01	8,22E-01	Unc45a	1,03	No
ENSMUSG00000030534	5,92E-02	3,798917078	4,09E-01	5,22E-01	8,01E-01	Vps33b	1,04	No
ENSMUSG00000030536	4,70E-02	10,41963064	7,01E-01	4,02E-01	7,01E-01	Iqgap1	1,03	No
ENSMUSG00000030538	7,67E-02	5,361176909	1,19E+00	2,76E-01	5,73E-01	Cib1	1,05	No
ENSMUSG00000030539	2,46E-01	5,724859566	1,32E+01	2,78E-04	5,70E-03	Sema4b	1,19	Yes
ENSMUSG00000030541	1,46E-01	5,796351291	4,74E+00	2,95E-02	1,96E-01	Idh2	1,11	No
ENSMUSG00000030543	-2,86E-01	-1,863695392	1,42E-01	7,06E-01	9,08E-01	Mesp2	0,82	No
ENSMUSG00000030545	-4,35E-02	2,320269944	9,82E-02	7,54E-01	9,33E-01	Pex11a	0,97	No
ENSMUSG00000030546	-5,22E-01	-2,832008958	2,11E-01	6,46E-01	8,74E-01	Plin1	0,70	No
ENSMUSG00000030549	-5,36E-02	-2,770579584	1,94E-03	9,65E-01	1,00E+00	Rhcg	0,96	No
ENSMUSG00000030551	-1,94E+00	-3,107619243	1,50E+00	2,21E-01	5,50E-01	Nr2f2	0,26	No
ENSMUSG00000030553	-2,97E-01	-0,83194635	6,47E-01	4,21E-01	7,16E-01	Pgpep1l	0,81	No
ENSMUSG00000030554	-1,38E-01	-1,641272956	6,09E-02	8,05E-01	9,63E-01	Synm	0,91	No
ENSMUSG00000030555	-3,57E-01	0,483745302	2,04E+00	1,53E-01	4,96E-01	Ttc23	0,78	No
ENSMUSG00000030556	-1,22E-01	3,20949187	1,32E+00	2,50E-01	5,50E-01	Lrrc28	0,92	No

ENSMUSG00000030557	6,10E-02	7,43110062	1,05E+00	3,06E-01	6,03E-01	Mef2a	1,04	No
ENSMUSG00000030559	-3,56E-02	1,73206599	4,90E-02	8,25E-01	9,74E-01	Rab38	0,98	No
ENSMUSG00000030560	1,50E-01	9,909038082	7,12E+00	7,60E-03	7,62E-02	Ctsc	1,11	No
ENSMUSG00000030577	2,97E-01	4,240482132	1,22E+01	4,87E-04	8,96E-03	Cd22	1,23	Yes
ENSMUSG00000030579	-1,94E-02	8,510861976	1,14E-01	7,36E-01	9,24E-01	Tyrobp	0,99	No
ENSMUSG00000030583	5,97E-02	7,133668365	9,79E-01	3,23E-01	6,23E-01	Sipa1l3	1,04	No
ENSMUSG00000030584	3,16E-01	-0,980737306	5,32E-01	4,66E-01	7,54E-01	Dpf1	1,24	No
ENSMUSG00000030587	-1,06E-01	2,734214201	7,84E-01	3,76E-01	6,77E-01	2200002D01Rik	0,93	No
ENSMUSG00000030588	-7,89E-02	4,978454761	1,13E+00	2,87E-01	5,83E-01	Yif1b	0,95	No
ENSMUSG00000030589	3,64E-02	6,54765382	3,40E-01	5,60E-01	8,23E-01	Rasgrp4	1,03	No
ENSMUSG00000030590	-1,83E-01	4,942246908	6,09E+00	1,36E-02	1,17E-01	Fam98c	0,88	No
ENSMUSG00000030591	4,45E-02	7,761300814	5,76E-01	4,48E-01	7,38E-01	Psmd8	1,03	No
ENSMUSG00000030592	-2,32E-01	3,43568792	5,43E+00	1,98E-02	1,50E-01	Ryr1	0,85	No
ENSMUSG00000030595	-3,30E-01	5,59418592	2,33E+01	1,36E-06	5,86E-05	Nfkbib	0,80	Yes
ENSMUSG00000030598	-2,61E-02	-0,574111215	5,32E-03	9,42E-01	1,00E+00	Fbxo17	0,98	No
ENSMUSG00000030600	-1,65E-01	1,310092047	7,34E-01	3,92E-01	6,92E-01	Lrfn1	0,89	No
ENSMUSG00000030602	2,04E-01	4,987632098	7,68E+00	5,57E-03	6,05E-02	Pak4	1,15	No
ENSMUSG00000030603	6,68E-03	6,216589682	1,08E-02	9,17E-01	1,00E+00	Psmc4	1,00	No
ENSMUSG00000030604	-8,58E-02	3,337142111	7,21E-01	3,96E-01	6,96E-01	Zfp626	0,94	No
ENSMUSG00000030605	-1,88E-01	10,05012513	1,12E+01	8,22E-04	1,38E-02	Mfge8	0,88	Yes
ENSMUSG00000030606	-4,34E-01	2,022507944	8,20E+00	4,19E-03	4,89E-02	Hapln3	0,74	Yes
ENSMUSG00000030607	-2,32E-01	-1,126431633	2,26E-01	6,35E-01	8,66E-01	Acan	0,85	No
ENSMUSG00000030609	1,69E-01	5,198588592	5,54E+00	1,86E-02	1,44E-01	Aen	1,12	No
ENSMUSG00000030610	2,43E-01	3,206602941	5,31E+00	2,12E-02	1,57E-01	Det1	1,18	No
ENSMUSG00000030611	1,61E-01	3,305860779	2,37E+00	1,24E-01	4,40E-01	Mrps11	1,12	No
ENSMUSG00000030612	1,29E-01	4,061910238	2,15E+00	1,43E-01	4,77E-01	Mrpl46	1,09	No
ENSMUSG00000030613	4,00E-02	3,003995363	1,25E-01	7,24E-01	9,17E-01	Ccdc90b	1,03	No
ENSMUSG00000030614	2,00E-01	3,620546343	4,50E+00	3,40E-02	2,17E-01	Tmem126b	1,15	No
ENSMUSG00000030615	-5,10E-02	4,199963484	3,58E-01	5,50E-01	8,17E-01	Tmem126a	0,97	No
ENSMUSG00000030616	-1,94E+00	-3,107619243	1,50E+00	2,21E-01	5,50E-01	Sytl2	0,26	No
ENSMUSG00000030619	7,09E-02	5,289559897	9,97E-01	3,18E-01	6,19E-01	Eed	1,05	No
ENSMUSG00000030623	-2,49E-02	-3,146121042	-4,44E-11	1,00E+00	1,00E+00	Prss23os	0,98	No

ENSMUSG00000030629	-5,57E-01	7,443122766	8,77E+01	7,56E-21	1,83E-18	Zfand6	0,68	Yes
ENSMUSG00000030630	-4,61E-01	1,438962004	7,01E+00	8,09E-03	7,97E-02	Fah	0,73	No
ENSMUSG00000030636	-4,65E-02	-2,770633497	1,47E-03	9,69E-01	1,00E+00	1700010L04Rik	0,97	No
ENSMUSG00000030638	-5,50E-02	-2,832003153	1,36E-03	9,71E-01	1,00E+00	Sh3gl3	0,96	No
ENSMUSG00000030641	1,39E-01	4,437004321	2,94E+00	8,62E-02	3,78E-01	Ddias	1,10	No
ENSMUSG00000030643	-8,25E-01	2,189753013	3,61E+01	1,89E-09	1,48E-07	Rab30	0,56	Yes
ENSMUSG00000030647	1,85E-02	5,586028935	7,33E-02	7,87E-01	9,52E-01	Ndufc2	1,01	No
ENSMUSG00000030649	-4,67E-02	3,703193663	2,41E-01	6,23E-01	8,58E-01	Anapc15	0,97	No
ENSMUSG00000030650	-1,74E+00	-2,548142149	3,34E+00	6,77E-02	3,28E-01	Tmc5	0,30	No
ENSMUSG00000030651	-2,78E-01	-1,800890729	2,52E-01	6,16E-01	8,54E-01	Art2b	0,82	No
ENSMUSG00000030652	7,87E-02	3,445041698	5,99E-01	4,39E-01	7,32E-01	Coq7	1,06	No
ENSMUSG00000030653	7,14E-02	4,24702525	7,15E-01	3,98E-01	6,98E-01	Gm45837	1,05	No
ENSMUSG00000030654	2,24E-03	7,709061127	1,45E-03	9,70E-01	1,00E+00	Arl6ip1	1,00	No
ENSMUSG00000030655	-2,30E-02	8,144756177	1,58E-01	6,91E-01	9,00E-01	Smg1	0,98	No
ENSMUSG00000030657	3,09E-01	4,743727257	1,56E+01	8,02E-05	2,02E-03	Xylt1	1,24	Yes
ENSMUSG00000030659	3,48E-01	4,523760838	1,83E+01	1,87E-05	5,82E-04	Nucb2	1,27	Yes
ENSMUSG00000030660	9,97E-02	6,091635325	2,34E+00	1,26E-01	4,44E-01	Pik3c2a	1,07	No
ENSMUSG00000030662	9,86E-02	6,586167113	2,49E+00	1,14E-01	4,18E-01	Ipo5	1,07	No
ENSMUSG00000030663	-4,98E-02	5,848752478	5,63E-01	4,53E-01	7,43E-01	1110004F10Rik	0,97	No
ENSMUSG00000030666	-7,59E-01	1,313357165	1,61E+01	5,93E-05	1,57E-03	Calcb	0,59	Yes
ENSMUSG00000030670	3,03E-02	0,351906386	1,28E-02	9,10E-01	1,00E+00	Cyp2r1	1,02	No
ENSMUSG00000030671	-6,35E-02	3,286003006	3,69E-01	5,43E-01	8,12E-01	Pde3b	0,96	No
ENSMUSG00000030672	-2,00E-01	-1,424849337	1,16E-01	7,33E-01	9,23E-01	Mylpf	0,87	No
ENSMUSG00000030674	-2,25E-01	-1,33323883	2,13E-01	6,45E-01	8,73E-01	Qprt	0,86	No
ENSMUSG00000030677	1,89E-01	4,998170832	6,48E+00	1,09E-02	9,95E-02	Kif22	1,14	No
ENSMUSG00000030678	9,44E-03	7,330858156	2,51E-02	8,74E-01	1,00E+00	Maz	1,01	No
ENSMUSG00000030680	-3,50E-02	3,824370125	1,44E-01	7,04E-01	9,06E-01	Pagr1a	0,98	No
ENSMUSG00000030681	-2,05E-01	7,782784807	1,22E+01	4,68E-04	8,69E-03	Mvp	0,87	Yes
ENSMUSG00000030682	1,30E-01	6,623039146	4,35E+00	3,70E-02	2,29E-01	Cdipt	1,09	No
ENSMUSG00000030683	-3,80E-01	-0,252309101	1,39E+00	2,38E-01	5,50E-01	Sez6l2	0,77	No
ENSMUSG00000030685	-4,61E-02	3,579708885	2,26E-01	6,34E-01	8,66E-01	Kctd13	0,97	No
ENSMUSG00000030688	-6,52E-01	2,995470625	3,51E+01	3,05E-09	2,32E-07	Stard10	0,64	Yes

ENSMUSG00000030689	1,64E-02	5,111845335	5,04E-02	8,22E-01	9,72E-01	Ino80e	1,01	No
ENSMUSG00000030691	-1,57E-01	5,400560664	5,08E+00	2,42E-02	1,71E-01	Fchsd2	0,90	No
ENSMUSG00000030695	6,99E-02	9,479673718	1,53E+00	2,16E-01	5,50E-01	Aldoa	1,05	No
ENSMUSG00000030697	-4,40E-02	6,930394758	5,21E-01	4,71E-01	7,59E-01	Ppp4c	0,97	No
ENSMUSG00000030699	5,20E-01	-0,912881615	1,54E+00	2,14E-01	5,50E-01	Tbx6	1,43	No
ENSMUSG00000030701	-1,20E-01	-0,711696427	8,52E-02	7,70E-01	9,42E-01	Plekhb1	0,92	No
ENSMUSG00000030703	-1,07E-01	1,087474521	2,83E-01	5,95E-01	8,43E-01	Gdpd3	0,93	No
ENSMUSG00000030704	7,51E-02	6,481236951	1,43E+00	2,32E-01	5,50E-01	Rab6a	1,05	No
ENSMUSG00000030706	1,30E-01	4,035625505	2,13E+00	1,44E-01	4,80E-01	Mrpl48	1,09	No
ENSMUSG00000030707	-9,44E-02	8,979458953	2,75E+00	9,72E-02	3,89E-01	Coro1a	0,94	No
ENSMUSG00000030708	7,16E-02	3,405030824	4,94E-01	4,82E-01	7,69E-01	Dnajb13	1,05	No
ENSMUSG00000030711	2,66E-01	1,607090444	2,33E+00	1,27E-01	4,47E-01	Sult1a1	1,20	No
ENSMUSG00000030714	8,63E-02	2,568768915	4,56E-01	5,00E-01	7,85E-01	Sgf29	1,06	No
ENSMUSG00000030717	7,24E-02	2,2211695	2,78E-01	5,98E-01	8,45E-01	Nupr1	1,05	No
ENSMUSG00000030718	5,28E-02	5,086518423	5,20E-01	4,71E-01	7,59E-01	Ppme1	1,04	No
ENSMUSG00000030720	2,26E-01	5,78493434	1,13E+01	7,86E-04	1,33E-02	Cln3	1,17	Yes
ENSMUSG00000030722	2,02E-01	3,96642252	5,19E+00	2,27E-02	1,64E-01	Nfatc2ip	1,15	No
ENSMUSG00000030724	-1,43E+00	-1,740822382	5,19E+00	2,27E-02	1,64E-01	Cd19	0,37	No
ENSMUSG00000030725	3,80E-01	2,118033961	6,94E+00	8,45E-03	8,25E-02	Lipt2	1,30	No
ENSMUSG00000030726	9,83E-02	5,206743824	1,88E+00	1,70E-01	5,26E-01	Pold3	1,07	No
ENSMUSG00000030727	-4,00E-02	4,76290754	2,73E-01	6,01E-01	8,47E-01	Rabep2	0,97	No
ENSMUSG00000030729	4,49E-01	5,58872954	4,24E+01	7,37E-11	7,13E-09	Pgm2l1	1,36	Yes
ENSMUSG00000030730	4,67E-01	-0,401510695	1,70E+00	1,92E-01	5,50E-01	Atp2a1	1,38	No
ENSMUSG00000030731	-8,57E-01	-2,963247565	4,04E-01	5,25E-01	8,02E-01	Syt3	0,55	No
ENSMUSG00000030732	-1,89E+00	-3,1076441	1,42E+00	2,33E-01	5,50E-01	Chrdl2	0,27	No
ENSMUSG00000030733	2,23E-02	5,576244732	1,07E-01	7,44E-01	9,28E-01	Sh2b1	1,02	No
ENSMUSG00000030735	3,40E-02	6,110306954	2,77E-01	5,99E-01	8,46E-01	Gm9755	1,02	No
ENSMUSG00000030737	-1,38E-01	1,074811706	4,85E-01	4,86E-01	7,73E-01	Slco2b1	0,91	No
ENSMUSG00000030738	-1,40E-02	7,612035144	5,61E-02	8,13E-01	9,67E-01	Eif3c	0,99	No
ENSMUSG00000030739	-6,08E-01	-2,401394958	5,04E-01	4,78E-01	7,65E-01	Myh14	0,66	No
ENSMUSG00000030741	1,61E-01	6,206613589	6,28E+00	1,22E-02	1,08E-01	Spns1	1,12	No
ENSMUSG00000030742	-1,44E-01	0,416137934	3,41E-01	5,59E-01	8,22E-01	Lat	0,90	No

ENSMUSG00000030744	-1,61E-01	8,719834758	7,91E+00	4,91E-03	5,52E-02	Rps3		0,89	No
ENSMUSG00000030745	-2,38E-01	8,007710883	1,67E+01	4,28E-05	1,19E-03	Il21r		0,85	Yes
ENSMUSG00000030747	6,32E-02	1,160659059	1,02E-01	7,49E-01	9,31E-01	Dgat2		1,04	No
ENSMUSG00000030748	-1,25E-01	8,501257699	4,75E+00	2,94E-02	1,95E-01	Il4ra		0,92	No
ENSMUSG00000030750	-2,35E-01	4,085120171	7,43E+00	6,42E-03	6,74E-02	Nsmce1		0,85	No
ENSMUSG00000030751	5,47E-03	6,268603682	7,33E-03	9,32E-01	1,00E+00	Psma1		1,00	No
ENSMUSG00000030752	-3,54E-02	1,841341748	5,13E-02	8,21E-01	9,72E-01	Kdm8		0,98	No
ENSMUSG00000030753	-3,05E-01	6,968322308	2,52E+01	5,28E-07	2,50E-05	Thap12		0,81	Yes
ENSMUSG00000030754	1,23E-02	7,109773673	4,16E-02	8,38E-01	9,82E-01	Copb1		1,01	No
ENSMUSG00000030759	7,66E-02	7,776481659	1,71E+00	1,91E-01	5,50E-01	Far1		1,05	No
ENSMUSG00000030760	2,04E-01	5,587635379	8,85E+00	2,94E-03	3,77E-02	Acer3		1,15	Yes
ENSMUSG00000030761	3,29E-01	6,710768277	2,82E+01	1,09E-07	6,03E-06	Myo7a		1,26	Yes
ENSMUSG00000030762	1,85E+00	-3,107657832	1,35E+00	2,46E-01	5,50E-01	Aqp8		3,61	No
ENSMUSG00000030763	1,98E-01	3,978028114	4,91E+00	2,67E-02	1,83E-01	Lcmt1		1,15	No
ENSMUSG00000030766	2,53E-01	6,39854769	1,60E+01	6,39E-05	1,67E-03	Arhgap17		1,19	Yes
ENSMUSG00000030768	-7,98E-03	3,278107149	5,69E-03	9,40E-01	1,00E+00	Disp1		0,99	No
ENSMUSG00000030769	1,06E-01	0,295495819	1,63E-01	6,87E-01	8,97E-01	Slc5a11		1,08	No
ENSMUSG00000030770	-9,92E-01	-2,311154579	1,52E+00	2,17E-01	5,50E-01	Parva		0,50	No
ENSMUSG00000030771	4,17E-01	1,006405949	4,14E+00	4,18E-02	2,43E-01	Micalcl		1,33	No
ENSMUSG00000030772	-3,55E+00	-2,8319919	5,65E+00	1,74E-02	1,38E-01	Dkk3		0,09	No
ENSMUSG00000030774	7,48E-02	6,047332278	1,32E+00	2,51E-01	5,50E-01	Pak1		1,05	No
ENSMUSG00000030779	1,25E-01	6,921605442	4,16E+00	4,13E-02	2,41E-01	Rbbp6		1,09	No
ENSMUSG00000030780	2,69E-01	3,975182208	9,05E+00	2,63E-03	3,46E-02	BC017158		1,20	Yes
ENSMUSG00000030781	2,74E-01	1,843811318	2,95E+00	8,59E-02	3,77E-01	Slc5a2		1,21	No
ENSMUSG00000030782	-3,87E-01	-1,401271243	5,40E-01	4,63E-01	7,51E-01	Tgfb1i1		0,76	No
ENSMUSG00000030785	-7,40E-01	-1,24703584	2,57E+00	1,09E-01	4,05E-01	Cox6a2		0,60	No
ENSMUSG00000030786	2,85E-02	9,560770073	2,54E-01	6,14E-01	8,53E-01	Itgam		1,02	No
ENSMUSG00000030787	2,37E+00	-2,497636428	4,80E+00	2,84E-02	1,91E-01	Lyve1		5,18	No
ENSMUSG00000030788	1,63E-01	5,685486553	5,79E+00	1,61E-02	1,32E-01	Rnf141		1,12	No
ENSMUSG00000030789	2,17E-01	10,5535783	1,50E+01	1,09E-04	2,62E-03	Itgax		1,16	Yes
ENSMUSG00000030790	-1,05E+00	-1,24692184	4,61E+00	3,18E-02	2,07E-01	Adm		0,48	No
ENSMUSG00000030792	7,16E-02	0,175866137	6,18E-02	8,04E-01	9,62E-01	Dkkl1		1,05	No

ENSMUSG00000030793	1,89E-02	7,044278039	9,75E-02	7,55E-01	9,34E-01	Pycard	1,01	No
ENSMUSG00000030795	9,33E-03	7,510735538	2,49E-02	8,75E-01	1,00E+00	Fus	1,01	No
ENSMUSG00000030796	-2,75E-01	-0,205714366	7,68E-01	3,81E-01	6,82E-01	Tead2	0,83	No
ENSMUSG00000030798	9,35E-02	5,840924796	1,98E+00	1,59E-01	5,07E-01	Cd37	1,07	No
ENSMUSG00000030800	-8,01E-01	-1,770652573	1,71E+00	1,91E-01	5,50E-01	Prss8	0,57	No
ENSMUSG00000030801	-1,21E-01	3,317938946	1,36E+00	2,43E-01	5,50E-01	Kat8	0,92	No
ENSMUSG00000030802	1,51E-01	5,311808655	4,53E+00	3,34E-02	2,14E-01	Bckdk	1,11	No
ENSMUSG00000030804	1,57E-01	3,082025503	1,99E+00	1,58E-01	5,05E-01	Gm21974	1,11	No
ENSMUSG00000030805	-3,93E-02	5,868444384	3,51E-01	5,53E-01	8,19E-01	Stx4a	0,97	No
ENSMUSG00000030806	-7,42E-01	-0,170503408	5,79E+00	1,61E-02	1,32E-01	Stx1b	0,60	No
ENSMUSG00000030811	-8,70E-02	4,601824443	1,24E+00	2,66E-01	5,62E-01	Fbxl19	0,94	No
ENSMUSG00000030814	-1,95E-01	3,292204733	3,53E+00	6,04E-02	3,04E-01	Bcl7c	0,87	No
ENSMUSG00000030815	-2,32E-02	5,323781135	1,08E-01	7,42E-01	9,27E-01	Phkg2	0,98	No
ENSMUSG00000030816	2,38E-03	6,165622319	1,37E-03	9,71E-01	1,00E+00	Rnf40	1,00	No
ENSMUSG00000030822	-9,99E-02	6,312189842	2,48E+00	1,16E-01	4,21E-01	Prr14	0,93	No
ENSMUSG00000030823	2,03E-01	1,545425305	1,39E+00	2,39E-01	5,50E-01	9130019O22Rik	1,15	No
ENSMUSG00000030824	6,88E-02	7,423621002	1,34E+00	2,48E-01	5,50E-01	Nucb1	1,05	No
ENSMUSG00000030825	-8,72E-01	-1,246864935	3,26E+00	7,10E-02	3,37E-01	Hsd17b14	0,55	No
ENSMUSG00000030826	-5,32E-03	6,009296165	6,61E-03	9,35E-01	1,00E+00	Bcat2	1,00	No
ENSMUSG00000030830	2,49E-01	7,780148724	1,80E+01	2,25E-05	6,91E-04	Itgal	1,19	Yes
ENSMUSG00000030834	-8,19E-01	-2,963254278	3,66E-01	5,45E-01	8,13E-01	Abcc6	0,57	No
ENSMUSG00000030835	3,35E-03	5,95093826	2,58E-03	9,60E-01	1,00E+00	Nomo1	1,00	No
ENSMUSG00000030838	-5,81E-02	-2,548179111	3,08E-03	9,56E-01	1,00E+00	Ush1c	0,96	No
ENSMUSG00000030839	-7,46E-03	2,821585401	3,91E-03	9,50E-01	1,00E+00	Sergef	0,99	No
ENSMUSG00000030842	-2,44E-02	6,498964249	1,52E-01	6,97E-01	9,03E-01	Lamtor1	0,98	No
ENSMUSG00000030844	-1,28E-01	6,246166762	4,04E+00	4,45E-02	2,51E-01	Rgs10	0,91	No
ENSMUSG00000030846	1,05E-01	5,650107135	2,39E+00	1,22E-01	4,36E-01	Tial1	1,08	No
ENSMUSG00000030847	2,13E-01	5,466321227	9,43E+00	2,13E-03	2,94E-02	Bag3	1,16	Yes
ENSMUSG00000030849	1,46E-01	0,551443359	3,53E-01	5,52E-01	8,19E-01	Fgfr2	1,11	No
ENSMUSG00000030850	8,08E-02	5,539299328	1,38E+00	2,40E-01	5,50E-01	Ate1	1,06	No
ENSMUSG00000030851	7,92E-01	-0,816424126	3,71E+00	5,42E-02	2,85E-01	Ldhc	1,73	No
ENSMUSG00000030852	-1,03E-01	3,330818513	9,98E-01	3,18E-01	6,18E-01	Tacc2	0,93	No

ENSMUSG00000030854	1,20E+00	-1,497707806	3,54E+00	5,99E-02	3,03E-01	Ptpn5	2,30	No
ENSMUSG00000030858	-1,72E+00	-2,497540378	3,26E+00	7,10E-02	3,37E-01	1700007K09Rik	0,30	No
ENSMUSG00000030859	-3,66E-01	-2,311150316	1,68E-01	6,82E-01	8,94E-01	Fam24a	0,78	No
ENSMUSG00000030861	1,07E-01	5,94251763	2,66E+00	1,03E-01	3,95E-01	Acadsb	1,08	No
ENSMUSG00000030865	7,38E-01	-1,963326052	1,15E+00	2,83E-01	5,80E-01	Chp2	1,67	No
ENSMUSG00000030866	-2,98E+00	-2,99797937	3,63E+00	5,66E-02	2,92E-01	Ern2	0,13	No
ENSMUSG00000030867	1,40E-01	4,841763709	3,39E+00	6,56E-02	3,21E-01	Plk1	1,10	No
ENSMUSG00000030868	7,13E-02	6,04865063	1,20E+00	2,73E-01	5,70E-01	Dctn5	1,05	No
ENSMUSG00000030869	-8,19E-02	3,644644924	7,29E-01	3,93E-01	6,93E-01	Ndufab1	0,94	No
ENSMUSG00000030870	-4,70E-02	5,659948517	4,85E-01	4,86E-01	7,73E-01	Ubfd1	0,97	No
ENSMUSG00000030871	-4,73E-02	3,09917861	1,89E-01	6,64E-01	8,84E-01	Ears2	0,97	No
ENSMUSG00000030872	-1,07E-01	5,838395934	2,63E+00	1,05E-01	3,97E-01	Gga2	0,93	No
ENSMUSG00000030873	1,27E+00	-2,832019569	1,01E+00	3,15E-01	6,16E-01	Scnn1b	2,42	No
ENSMUSG00000030876	1,38E-01	5,322161893	3,76E+00	5,25E-02	2,79E-01	Mettl9	1,10	No
ENSMUSG00000030877	-9,56E-02	-2,548221048	1,06E-02	9,18E-01	1,00E+00	Mfsd13b	0,94	No
ENSMUSG00000030878	2,63E-01	3,105990833	5,72E+00	1,68E-02	1,35E-01	Cdr2	1,20	No
ENSMUSG00000030879	3,34E-02	5,181958141	2,15E-01	6,43E-01	8,72E-01	Mrpl17	1,02	No
ENSMUSG00000030880	5,59E-02	4,594090697	4,95E-01	4,82E-01	7,69E-01	Polr3e	1,04	No
ENSMUSG00000030881	-6,12E-03	5,260070684	7,37E-03	9,32E-01	1,00E+00	Arfip2	1,00	No
ENSMUSG00000030882	3,49E-01	1,50951088	3,95E+00	4,70E-02	2,60E-01	Dnhd1	1,27	No
ENSMUSG00000030884	-6,39E-02	6,238845527	9,94E-01	3,19E-01	6,19E-01	Uqcrc2	0,96	No
ENSMUSG00000030887	-1,63E-01	3,047888837	2,09E+00	1,48E-01	4,88E-01	Pdzd9	0,89	No
ENSMUSG00000030888	2,60E-02	4,914514206	1,22E-01	7,27E-01	9,19E-01	Rrp8	1,02	No
ENSMUSG00000030890	-2,41E-02	5,553495837	1,24E-01	7,25E-01	9,18E-01	Ilk	0,98	No
ENSMUSG00000030894	6,44E-02	8,010712126	1,22E+00	2,69E-01	5,65E-01	Tpp1	1,05	No
ENSMUSG00000030895	-1,82E-01	-0,726120554	2,25E-01	6,35E-01	8,67E-01	Hpx	0,88	No
ENSMUSG00000030911	1,87E+00	-3,107639691	1,38E+00	2,40E-01	5,50E-01	Zp2	3,67	No
ENSMUSG00000030917	9,88E-02	4,430457976	1,47E+00	2,25E-01	5,50E-01	Tmem159	1,07	No
ENSMUSG00000030921	-8,29E-02	6,861035072	1,85E+00	1,74E-01	5,32E-01	Trim30a	0,94	No
ENSMUSG00000030922	6,06E-02	1,857542196	1,47E-01	7,01E-01	9,05E-01	Lyrm1	1,04	No
ENSMUSG00000030924	-2,44E-02	2,901715868	4,55E-02	8,31E-01	9,78E-01	Rexo5	0,98	No
ENSMUSG00000030929	2,90E-01	3,096412395	7,05E+00	7,92E-03	7,84E-02	Eri2	1,22	No

ENSMUSG00000030930	-6,99E-01	2,144133192	2,48E+01	6,25E-07	2,91E-05	Chst15	0,62	Yes
ENSMUSG00000030934	-8,60E-03	6,73447038	1,95E-02	8,89E-01	1,00E+00	Oat	0,99	No
ENSMUSG00000030935	3,44E-01	1,277122895	3,27E+00	7,05E-02	3,36E-01	Acsm3	1,27	No
ENSMUSG00000030942	1,96E-02	5,235614973	7,56E-02	7,83E-01	9,50E-01	Thumpd1	1,01	No
ENSMUSG00000030946	-1,97E-01	3,166088143	3,31E+00	6,91E-02	3,31E-01	Lhpp	0,87	No
ENSMUSG00000030956	-1,61E-01	6,579378254	6,66E+00	9,85E-03	9,22E-02	Fam53b	0,89	No
ENSMUSG00000030960	-1,16E-01	2,604944429	8,47E-01	3,57E-01	6,59E-01	Eef1akmt2	0,92	No
ENSMUSG00000030963	-1,87E+00	-3,107637761	1,39E+00	2,38E-01	5,50E-01	Umod	0,27	No
ENSMUSG00000030965	8,07E-02	5,52232166	1,38E+00	2,40E-01	5,50E-01	Abraxas2	1,06	No
ENSMUSG00000030966	-6,94E-02	4,170279924	6,70E-01	4,13E-01	7,09E-01	Trim21	0,95	No
ENSMUSG00000030967	-1,09E-01	5,615172796	2,57E+00	1,09E-01	4,05E-01	Zranb1	0,93	No
ENSMUSG00000030968	3,14E-02	-3,107617333	-7,69E-10	1,00E+00	1,00E+00	Pdilt	1,02	No
ENSMUSG00000030970	1,38E-01	5,815398188	4,24E+00	3,95E-02	2,36E-01	Ctbp2	1,10	No
ENSMUSG00000030978	1,74E-01	7,140418178	8,33E+00	3,91E-03	4,65E-02	Rrm1	1,13	Yes
ENSMUSG00000030979	6,32E-03	2,919524656	2,99E-03	9,56E-01	1,00E+00	Uros	1,00	No
ENSMUSG00000030980	1,48E-01	5,558204707	4,69E+00	3,04E-02	2,00E-01	Knop1	1,11	No
ENSMUSG00000030981	-3,75E-01	-2,185560412	1,77E-01	6,74E-01	8,90E-01	Mmp21	0,77	No
ENSMUSG00000030982	4,28E-02	6,243417517	4,45E-01	5,05E-01	7,89E-01	9030624J02Rik	1,03	No
ENSMUSG00000030983	-2,49E-02	4,583886809	9,88E-02	7,53E-01	9,33E-01	Bccip	0,98	No
ENSMUSG00000030986	8,49E-02	4,249737969	1,01E+00	3,15E-01	6,16E-01	Dhx32	1,06	No
ENSMUSG00000030987	9,06E-02	5,919132997	1,86E+00	1,72E-01	5,29E-01	Stim1	1,06	No
ENSMUSG00000030990	6,87E-02	6,687367111	1,24E+00	2,66E-01	5,61E-01	Pgap2	1,05	No
ENSMUSG00000030996	2,06E-02	-3,146103739	-9,26E-10	1,00E+00	1,00E+00	Art1	1,01	No
ENSMUSG00000031004	2,60E-01	8,711039407	2,07E+01	5,47E-06	1,96E-04	Mki67	1,20	Yes
ENSMUSG00000031015	-1,85E-01	6,88211428	9,20E+00	2,42E-03	3,26E-02	Swap70	0,88	Yes
ENSMUSG00000031016	1,41E-01	4,032522852	2,56E+00	1,10E-01	4,06E-01	Wee1	1,10	No
ENSMUSG00000031021	-6,92E-02	6,107482425	1,14E+00	2,85E-01	5,82E-01	Tmem9b	0,95	No
ENSMUSG00000031022	-5,71E-01	-2,2894782	4,83E-01	4,87E-01	7,74E-01	BC051019	0,67	No
ENSMUSG00000031023	-7,24E-02	3,01018717	4,18E-01	5,18E-01	7,98E-01	Akip1	0,95	No
ENSMUSG00000031024	-2,30E-01	1,395902012	1,84E+00	1,75E-01	5,33E-01	St5	0,85	No
ENSMUSG00000031026	6,46E-01	-0,980613987	1,98E+00	1,59E-01	5,06E-01	Trim66	1,56	No
ENSMUSG00000031028	-7,07E-02	-2,683166064	3,97E-03	9,50E-01	1,00E+00	Tub	0,95	No

ENSMUSG00000031029	-1,10E-01	7,569600902	3,45E+00	6,34E-02	3,14E-01	Eif3f		0,93	No
ENSMUSG00000031068	-8,88E-02	4,633334253	1,29E+00	2,56E-01	5,50E-01	Glrx3		0,94	No
ENSMUSG00000031072	2,43E-01	5,304495296	1,17E+01	6,38E-04	1,12E-02	Oraov1		1,18	Yes
ENSMUSG00000031074	-1,94E+00	-3,107619243	1,50E+00	2,21E-01	5,50E-01	Fgf3		0,26	No
ENSMUSG00000031075	-5,80E-02	-2,896170637	1,51E-03	9,69E-01	1,00E+00	Ano1		0,96	No
ENSMUSG00000031077	3,28E-02	4,126195493	1,46E-01	7,02E-01	9,05E-01	Fadd		1,02	No
ENSMUSG00000031078	4,58E-02	4,82012302	3,61E-01	5,48E-01	8,15E-01	Cttn		1,03	No
ENSMUSG00000031090	2,36E-01	2,877177476	4,12E+00	4,23E-02	2,45E-01	Nadsyn1		1,18	No
ENSMUSG00000031097	3,41E-01	2,302791288	6,28E+00	1,22E-02	1,08E-01	Tnni2		1,27	No
ENSMUSG00000031098	-5,05E-03	-0,946057952	1,40E-04	9,91E-01	1,00E+00	Syt8		1,00	No
ENSMUSG00000031129	2,00E-01	3,474698253	3,89E+00	4,87E-02	2,66E-01	Slc9a9		1,15	No
ENSMUSG00000031286	-5,95E-01	-1,366869465	9,36E-01	3,33E-01	6,34E-01	Glt28d2		0,66	No
ENSMUSG00000031294	-3,45E-01	-2,548215208	1,49E-01	7,00E-01	9,04E-01	D630029K05Rik		0,79	No
ENSMUSG00000031441	-3,17E-01	7,640295325	2,90E+01	7,39E-08	4,23E-06	Atp11a		0,80	Yes
ENSMUSG00000031442	2,71E-01	5,630992321	1,59E+01	6,69E-05	1,74E-03	Mcf2l		1,21	Yes
ENSMUSG00000031443	3,29E-01	7,973395901	3,17E+01	1,82E-08	1,19E-06	F7		1,26	Yes
ENSMUSG00000031444	1,65E-01	8,372347436	8,15E+00	4,30E-03	4,99E-02	F10		1,12	Yes
ENSMUSG00000031445	7,21E-02	3,212595327	4,45E-01	5,05E-01	7,89E-01	Proz		1,05	No
ENSMUSG00000031446	7,36E-02	6,198654898	1,31E+00	2,52E-01	5,50E-01	Cul4a		1,05	No
ENSMUSG00000031447	-1,01E-02	10,02603815	3,20E-02	8,58E-01	9,94E-01	Lamp1		0,99	No
ENSMUSG00000031448	-1,23E-02	-0,755505954	1,01E-03	9,75E-01	1,00E+00	Adprhl1		0,99	No
ENSMUSG00000031449	-9,30E-01	-2,711745545	7,56E-01	3,85E-01	6,86E-01	Atp4b		0,52	No
ENSMUSG00000031450	5,84E-01	-2,31118767	3,81E-01	5,37E-01	8,09E-01	Grk1		1,50	No
ENSMUSG00000031451	3,40E-01	3,107949253	8,95E+00	2,78E-03	3,61E-02	Gas6		1,27	Yes
ENSMUSG00000031452	6,57E-01	-2,033662303	6,25E-01	4,29E-01	7,23E-01	1700029H14Rik		1,58	No
ENSMUSG00000031453	9,33E-02	6,382719336	2,16E+00	1,42E-01	4,75E-01	Rasa3		1,07	No
ENSMUSG00000031458	5,77E-02	3,165651605	2,90E-01	5,91E-01	8,41E-01	Coprs		1,04	No
ENSMUSG00000031461	-1,95E+00	-3,033628749	1,50E+00	2,21E-01	5,50E-01	Myom2		0,26	No
ENSMUSG00000031465	-4,07E-01	-0,522414042	1,17E+00	2,80E-01	5,76E-01	Angpt2		0,75	No
ENSMUSG00000031467	1,24E-01	4,851254983	2,68E+00	1,02E-01	3,94E-01	Agpat5		1,09	No
ENSMUSG00000031478	-3,27E-01	-0,088524251	1,13E+00	2,88E-01	5,83E-01	Nek3		0,80	No
ENSMUSG00000031479	1,48E-01	4,936883506	3,87E+00	4,92E-02	2,68E-01	Vps36		1,11	No

ENSMUSG00000031480	2,30E-01	-1,40138455	1,90E-01	6,63E-01	8,83E-01	Thsd1	1,17	No
ENSMUSG00000031481	4,51E-01	-2,832041212	1,59E-01	6,90E-01	8,99E-01	Tpte	1,37	No
ENSMUSG00000031482	1,16E-01	3,514836954	1,34E+00	2,47E-01	5,50E-01	Slc25a15	1,08	No
ENSMUSG00000031483	7,90E-02	6,398881161	1,57E+00	2,10E-01	5,50E-01	Erlin2	1,06	No
ENSMUSG00000031485	6,67E-02	6,601693038	1,15E+00	2,84E-01	5,80E-01	Prosc	1,05	No
ENSMUSG00000031486	4,60E-01	2,593664851	1,30E+01	3,16E-04	6,32E-03	Adgra2	1,38	Yes
ENSMUSG00000031487	5,39E-02	2,968260645	2,25E-01	6,35E-01	8,67E-01	Brf2	1,04	No
ENSMUSG00000031488	-8,85E-02	6,642925987	2,03E+00	1,55E-01	4,98E-01	Rab11fip1	0,94	No
ENSMUSG00000031489	1,60E+00	-2,832030097	1,75E+00	1,86E-01	5,50E-01	Adrb3	3,03	No
ENSMUSG00000031490	2,82E-01	5,622632322	1,69E+01	3,90E-05	1,10E-03	Eif4ebp1	1,22	Yes
ENSMUSG00000031493	-9,73E-02	1,863770076	3,85E-01	5,35E-01	8,07E-01	Ggn	0,93	No
ENSMUSG00000031494	6,02E-01	6,024314197	8,28E+01	9,20E-20	2,03E-17	Cd209a	1,52	Yes
ENSMUSG00000031495	4,24E-01	0,915934712	3,80E+00	5,13E-02	2,76E-01	Cd209d	1,34	No
ENSMUSG00000031497	5,58E-01	1,175220818	8,04E+00	4,57E-03	5,22E-02	Tnfsf13b	1,47	No
ENSMUSG00000031502	-1,01E+00	-0,895877267	5,62E+00	1,78E-02	1,40E-01	Col4a1	0,49	No
ENSMUSG00000031503	-9,27E-01	0,688966046	1,54E+01	8,87E-05	2,20E-03	Col4a2	0,53	Yes
ENSMUSG00000031504	-5,49E-01	3,660548491	3,21E+01	1,48E-08	9,81E-07	Rab20	0,68	Yes
ENSMUSG00000031505	-2,28E-01	4,694651999	8,61E+00	3,35E-03	4,15E-02	Naxd	0,85	Yes
ENSMUSG00000031506	3,22E-01	7,170631114	2,85E+01	9,53E-08	5,37E-06	Ptpn7	1,25	Yes
ENSMUSG00000031508	6,52E-02	5,345551626	8,59E-01	3,54E-01	6,55E-01	Ankrd10	1,05	No
ENSMUSG00000031509	1,87E+00	-3,107639691	1,38E+00	2,40E-01	5,50E-01	1700016D06Rik	3,67	No
ENSMUSG00000031511	2,19E-01	6,711030659	1,26E+01	3,93E-04	7,53E-03	Arhgef7	1,16	Yes
ENSMUSG00000031512	-1,70E+00	-2,472818181	3,20E+00	7,34E-02	3,44E-01	Tex29	0,31	No
ENSMUSG00000031513	8,08E-02	6,207429213	1,58E+00	2,09E-01	5,50E-01	Leprotl1	1,06	No
ENSMUSG00000031516	-1,11E-01	4,837632007	2,14E+00	1,44E-01	4,79E-01	Dctn6	0,93	No
ENSMUSG00000031519	4,89E-02	2,775584777	1,63E-01	6,86E-01	8,97E-01	Asb5	1,03	No
ENSMUSG00000031520	-8,51E-01	0,07083989	9,55E+00	2,00E-03	2,81E-02	Vegfc	0,55	Yes
ENSMUSG00000031521	1,50E-02	4,288072637	3,18E-02	8,59E-01	9,94E-01	Aga	1,01	No
ENSMUSG00000031523	-5,82E-01	-1,24694083	1,60E+00	2,05E-01	5,50E-01	Dlc1	0,67	No
ENSMUSG00000031527	2,37E-02	6,095267012	1,34E-01	7,15E-01	9,12E-01	Eri1	1,02	No
ENSMUSG00000031529	8,33E-02	7,177617035	1,91E+00	1,67E-01	5,21E-01	Tnks	1,06	No
ENSMUSG00000031530	-3,81E-01	2,974102793	1,06E+01	1,12E-03	1,78E-02	Dusp4	0,77	Yes

ENSMUSG00000031532	7,78E-02	6,788295521	1,60E+00	2,06E-01	5,50E-01	Saraf	1,06	No
ENSMUSG00000031533	7,99E-02	3,382749083	6,13E-01	4,34E-01	7,27E-01	Mrps31	1,06	No
ENSMUSG00000031534	3,03E-02	3,034793049	7,16E-02	7,89E-01	9,54E-01	Smim19	1,02	No
ENSMUSG00000031535	-1,88E+00	-2,963233427	1,42E+00	2,33E-01	5,50E-01	Dkk4	0,27	No
ENSMUSG00000031536	-3,12E-02	4,142478511	1,33E-01	7,15E-01	9,13E-01	Polb	0,98	No
ENSMUSG00000031537	-6,32E-02	6,926962862	1,08E+00	2,99E-01	5,95E-01	Ikbkb	0,96	No
ENSMUSG00000031538	-4,41E-01	-1,711518206	5,17E-01	4,72E-01	7,60E-01	Plat	0,74	No
ENSMUSG00000031539	-5,31E-02	4,055553884	3,81E-01	5,37E-01	8,09E-01	Ap3m2	0,96	No
ENSMUSG00000031540	5,72E-02	6,630599569	8,52E-01	3,56E-01	6,57E-01	Kat6a	1,04	No
ENSMUSG00000031543	7,31E-01	0,269779348	6,76E+00	9,30E-03	8,84E-02	Ank1	1,66	No
ENSMUSG00000031545	8,50E-02	6,146071201	1,73E+00	1,88E-01	5,50E-01	Gpat4	1,06	No
ENSMUSG00000031546	-1,11E-02	4,382302637	1,82E-02	8,93E-01	1,00E+00	Gins4	0,99	No
ENSMUSG00000031548	1,81E+00	-3,10765737	1,28E+00	2,58E-01	5,50E-01	Sfrp1	3,49	No
ENSMUSG00000031549	-7,65E-01	-0,042475607	6,68E+00	9,73E-03	9,15E-02	Ido2	0,59	No
ENSMUSG00000031551	-4,53E-01	0,002166312	2,37E+00	1,24E-01	4,40E-01	Ido1	0,73	No
ENSMUSG00000031553	-1,34E+00	-2,655111252	1,13E+00	2,88E-01	5,83E-01	Adam3	0,39	No
ENSMUSG00000031554	-3,83E-02	-3,107651936	-7,91E-11	1,00E+00	1,00E+00	Adam5	0,97	No
ENSMUSG00000031555	5,77E-02	7,016895194	9,03E-01	3,42E-01	6,42E-01	Adam9	1,04	No
ENSMUSG00000031556	-4,41E-02	6,610397403	5,01E-01	4,79E-01	7,66E-01	Tm2d2	0,97	No
ENSMUSG00000031557	-3,36E-02	6,966450742	3,08E-01	5,79E-01	8,36E-01	Plekha2	0,98	No
ENSMUSG00000031558	-3,21E+00	-2,770578099	4,34E+00	3,72E-02	2,29E-01	Slit2	0,11	No
ENSMUSG00000031561	-2,66E+00	-3,033639428	2,81E+00	9,35E-02	3,83E-01	Tenm3	0,16	No
ENSMUSG00000031562	6,89E-01	0,649543286	8,35E+00	3,85E-03	4,60E-02	Dctd	1,61	Yes
ENSMUSG00000031563	2,60E-01	4,692144306	1,09E+01	9,52E-04	1,55E-02	Wwc2	1,20	Yes
ENSMUSG00000031565	6,29E-02	5,546962228	8,36E-01	3,60E-01	6,62E-01	Fgfr1	1,04	No
ENSMUSG00000031568	3,68E-02	4,39099634	2,01E-01	6,54E-01	8,79E-01	Rwdd4a	1,03	No
ENSMUSG00000031570	1,16E-01	4,533906761	2,07E+00	1,50E-01	4,91E-01	Plpp5	1,08	No
ENSMUSG00000031574	9,38E-03	1,273479316	2,41E-03	9,61E-01	1,00E+00	Star	1,01	No
ENSMUSG00000031575	5,03E-02	5,019443309	4,67E-01	4,94E-01	7,80E-01	Ash2l	1,04	No
ENSMUSG00000031576	-4,13E-02	-3,033659495	7,08E-04	9,79E-01	1,00E+00	Kcnu1	0,97	No
ENSMUSG00000031577	1,22E-01	4,518819401	2,35E+00	1,26E-01	4,44E-01	Tti2	1,09	No
ENSMUSG00000031578	1,40E-01	4,492684624	3,00E+00	8,32E-02	3,71E-01	Mak16	1,10	No

ENSMUSG00000031583	1,93E-02	5,484548128	7,85E-02	7,79E-01	9,48E-01	Wrn	1,01	No
ENSMUSG00000031584	-1,48E-01	6,153516268	5,26E+00	2,18E-02	1,59E-01	Gsr	0,90	No
ENSMUSG00000031585	5,01E-02	4,900564868	4,46E-01	5,04E-01	7,89E-01	Gtf2e2	1,04	No
ENSMUSG00000031586	-3,87E-01	3,447161653	1,52E+01	9,87E-05	2,40E-03	Rbpms	0,76	Yes
ENSMUSG00000031590	-1,26E-01	4,112672696	2,11E+00	1,46E-01	4,84E-01	Frg1	0,92	No
ENSMUSG00000031591	6,34E-02	7,632763191	1,16E+00	2,82E-01	5,78E-01	Asah1	1,04	No
ENSMUSG00000031592	4,54E-02	5,701519894	4,49E-01	5,03E-01	7,88E-01	Pcm1	1,03	No
ENSMUSG00000031594	3,14E-02	-3,107617333	-7,69E-10	1,00E+00	1,00E+00	Fgl1	1,02	No
ENSMUSG00000031595	-7,82E-01	-1,863687561	1,25E+00	2,64E-01	5,59E-01	Pdgfrl	0,58	No
ENSMUSG00000031596	5,45E-01	8,346755691	8,84E+01	5,35E-21	1,31E-18	Slc7a2	1,46	Yes
ENSMUSG00000031600	1,69E-01	5,433184329	5,86E+00	1,55E-02	1,29E-01	Vps37a	1,12	No
ENSMUSG00000031601	-4,24E-02	5,745612681	3,96E-01	5,29E-01	8,05E-01	Cnot7	0,97	No
ENSMUSG00000031603	7,77E-01	-2,226288296	9,02E-01	3,42E-01	6,43E-01	Fgf20	1,71	No
ENSMUSG00000031604	2,43E-01	6,50154796	1,50E+01	1,06E-04	2,55E-03	Msmo1	1,18	Yes
ENSMUSG00000031605	4,91E-02	5,164546233	4,64E-01	4,96E-01	7,81E-01	Klhl2	1,03	No
ENSMUSG00000031608	-3,92E-02	6,936706963	4,15E-01	5,19E-01	7,99E-01	Galnt7	0,97	No
ENSMUSG00000031609	1,09E-01	4,227424384	1,66E+00	1,97E-01	5,50E-01	Sap30	1,08	No
ENSMUSG00000031613	8,11E-02	-0,322247297	5,94E-02	8,07E-01	9,64E-01	Hpgd	1,06	No
ENSMUSG00000031616	-4,08E-01	-2,600670305	1,70E-01	6,80E-01	8,93E-01	Ednra	0,75	No
ENSMUSG00000031617	6,05E-02	5,139586176	6,91E-01	4,06E-01	7,02E-01	Tmem184c	1,04	No
ENSMUSG00000031618	4,15E-01	-0,832115776	1,09E+00	2,97E-01	5,92E-01	Nr3c2	1,33	No
ENSMUSG00000031622	-2,80E-02	6,274573068	1,94E-01	6,60E-01	8,82E-01	Sin3b	0,98	No
ENSMUSG00000031626	1,87E+00	-3,03364371	1,36E+00	2,43E-01	5,50E-01	Sorbs2	3,66	No
ENSMUSG00000031627	1,85E-01	6,163926929	8,24E+00	4,11E-03	4,82E-02	Irf2	1,14	Yes
ENSMUSG00000031628	-4,47E-01	6,498548305	5,10E+01	9,28E-13	1,13E-10	Casp3	0,73	Yes
ENSMUSG00000031629	-5,95E-02	2,848292869	2,63E-01	6,08E-01	8,51E-01	Cenpu	0,96	No
ENSMUSG00000031631	1,72E-01	4,109416217	4,02E+00	4,51E-02	2,53E-01	Cfap97	1,13	No
ENSMUSG00000031633	-1,34E-01	7,46387135	5,12E+00	2,37E-02	1,69E-01	Slc25a4	0,91	No
ENSMUSG00000031634	3,91E-02	5,258718582	2,99E-01	5,84E-01	8,38E-01	Ufsp2	1,03	No
ENSMUSG00000031636	1,11E+00	-2,60067749	1,17E+00	2,80E-01	5,77E-01	Pdlim3	2,16	No
ENSMUSG00000031637	1,82E-01	0,899540707	6,58E-01	4,17E-01	7,12E-01	Lrp2bp	1,13	No
ENSMUSG00000031639	5,25E-01	3,077536559	2,25E+01	2,11E-06	8,50E-05	Tlr3	1,44	Yes

ENSMUSG00000031640	7,72E-02	2,769944604	4,18E-01	5,18E-01	7,98E-01	Gm45753		1,05	No
ENSMUSG00000031641	-2,09E-02	3,458131877	4,45E-02	8,33E-01	9,78E-01	Cbr4		0,99	No
ENSMUSG00000031642	-2,58E-02	2,824312188	4,68E-02	8,29E-01	9,76E-01	Sh3rf1		0,98	No
ENSMUSG00000031644	-1,34E-01	3,897450865	2,22E+00	1,37E-01	4,66E-01	Nek1		0,91	No
ENSMUSG00000031645	1,81E+00	-3,10765737	1,28E+00	2,58E-01	5,50E-01	F11		3,49	No
ENSMUSG00000031647	1,90E-01	3,17230242	3,08E+00	7,94E-02	3,61E-01	Mfap3l		1,14	No
ENSMUSG00000031652	-1,40E-02	6,73870017	5,15E-02	8,20E-01	9,72E-01	N4bp1		0,99	No
ENSMUSG00000031654	8,62E-01	1,42461953	2,26E+01	2,00E-06	8,11E-05	Cbln1		1,82	Yes
ENSMUSG00000031657	1,79E-01	4,901018326	5,62E+00	1,78E-02	1,39E-01	Heatr3		1,13	No
ENSMUSG00000031659	2,85E-01	8,835059142	2,49E+01	5,96E-07	2,78E-05	Adcy7		1,22	Yes
ENSMUSG00000031660	-1,14E-02	5,976781909	3,05E-02	8,61E-01	9,96E-01	Brd7		0,99	No
ENSMUSG00000031661	-3,20E+00	-2,54817416	4,31E+00	3,79E-02	2,31E-01	Nkd1		0,11	No
ENSMUSG00000031662	-1,54E-01	7,005695387	6,52E+00	1,07E-02	9,80E-02	Snx20		0,90	No
ENSMUSG00000031666	-3,04E-02	6,396936896	2,33E-01	6,29E-01	8,62E-01	Rbl2		0,98	No
ENSMUSG00000031667	-3,82E-02	4,061729138	1,90E-01	6,63E-01	8,83E-01	Aktip		0,97	No
ENSMUSG00000031668	5,16E-02	5,773773533	5,98E-01	4,39E-01	7,32E-01	Eif2ak3		1,04	No
ENSMUSG00000031669	-3,31E-01	3,45081041	1,11E+01	8,76E-04	1,45E-02	Gins3		0,79	Yes
ENSMUSG00000031671	1,61E-02	3,146042423	2,24E-02	8,81E-01	1,00E+00	Setd6		1,01	No
ENSMUSG00000031672	-9,20E-02	6,157991087	2,04E+00	1,53E-01	4,96E-01	Got2		0,94	No
ENSMUSG00000031673	-8,19E-01	-0,236331114	6,73E+00	9,49E-03	8,98E-02	Cdh11		0,57	No
ENSMUSG00000031681	1,57E-01	4,266861598	3,54E+00	6,01E-02	3,03E-01	Smad1		1,12	No
ENSMUSG00000031682	-3,19E+00	-2,655081865	4,30E+00	3,82E-02	2,32E-01	1700011L22Rik		0,11	No
ENSMUSG00000031683	5,76E-02	4,036621932	4,29E-01	5,13E-01	7,95E-01	Lsm6		1,04	No
ENSMUSG00000031684	1,36E-01	4,739002869	3,09E+00	7,90E-02	3,60E-01	Slc10a7		1,10	No
ENSMUSG00000031691	1,14E-01	6,156811321	3,14E+00	7,66E-02	3,53E-01	Tnpo2		1,08	No
ENSMUSG00000031696	1,22E-01	7,991270434	4,39E+00	3,62E-02	2,26E-01	Vps35		1,09	No
ENSMUSG00000031697	6,38E-02	4,662999763	6,57E-01	4,18E-01	7,13E-01	Orc6		1,05	No
ENSMUSG00000031698	-3,78E-01	-2,497596744	1,80E-01	6,71E-01	8,89E-01	Mylk3		0,77	No
ENSMUSG00000031700	1,51E-01	3,829070038	2,74E+00	9,82E-02	3,91E-01	Gpt2		1,11	No
ENSMUSG00000031701	-7,58E-02	7,03653534	1,56E+00	2,11E-01	5,50E-01	Dnaja2		0,95	No
ENSMUSG00000031703	3,74E-02	5,605361842	3,00E-01	5,84E-01	8,38E-01	Itfg1		1,03	No
ENSMUSG00000031706	4,21E-02	4,872534396	3,12E-01	5,76E-01	8,35E-01	Rfx1		1,03	No

ENSMUSG00000031708	7,05E-02	5,297727853	9,81E-01	3,22E-01	6,22E-01	Tecr		1,05	No
ENSMUSG00000031709	2,31E-01	7,102562386	1,46E+01	1,34E-04	3,09E-03	Tbc1d9		1,17	Yes
ENSMUSG00000031710	-6,81E-01	-0,980385365	2,39E+00	1,22E-01	4,36E-01	Ucp1		0,62	No
ENSMUSG00000031711	1,21E-01	4,58931279	2,36E+00	1,25E-01	4,42E-01	Zfp330		1,09	No
ENSMUSG00000031712	-3,22E-01	3,424599451	1,04E+01	1,29E-03	1,99E-02	Il15		0,80	Yes
ENSMUSG00000031714	1,81E-02	4,057866022	4,12E-02	8,39E-01	9,83E-01	Gab1		1,01	No
ENSMUSG00000031715	1,44E-01	5,374945262	4,21E+00	4,02E-02	2,38E-01	Smarca5		1,11	No
ENSMUSG00000031722	-4,88E-01	3,634190011	2,59E+01	3,55E-07	1,74E-05	Hp		0,71	Yes
ENSMUSG00000031723	1,04E-01	2,750172416	7,65E-01	3,82E-01	6,83E-01	Txnl4b		1,07	No
ENSMUSG00000031727	1,87E+00	-3,107639691	1,38E+00	2,40E-01	5,50E-01	Pmfbp1		3,67	No
ENSMUSG00000031728	-5,90E-02	2,663738135	2,30E-01	6,31E-01	8,64E-01	Zfp821		0,96	No
ENSMUSG00000031729	-5,97E-02	6,614759148	9,22E-01	3,37E-01	6,38E-01	Ist1		0,96	No
ENSMUSG00000031730	1,54E-01	3,242181259	2,11E+00	1,46E-01	4,85E-01	Dhodh		1,11	No
ENSMUSG00000031731	1,14E-01	6,781093094	3,42E+00	6,45E-02	3,18E-01	Ap1g1		1,08	No
ENSMUSG00000031732	-1,48E-01	4,219389223	3,06E+00	8,02E-02	3,63E-01	Phlpp2		0,90	No
ENSMUSG00000031734	-3,18E+00	-2,77059745	4,27E+00	3,88E-02	2,34E-01	Irx3		0,11	No
ENSMUSG00000031736	7,78E-01	0,489627375	9,74E+00	1,81E-03	2,58E-02	Crnde		1,72	Yes
ENSMUSG00000031737	5,14E-01	-1,522645762	9,67E-01	3,26E-01	6,26E-01	Irx5		1,43	No
ENSMUSG00000031740	-1,85E-01	3,887858013	4,13E+00	4,23E-02	2,45E-01	Mmp2		0,88	No
ENSMUSG00000031748	-4,52E-02	-2,033651585	3,81E-03	9,51E-01	1,00E+00	Gnao1		0,97	No
ENSMUSG00000031749	-2,65E-01	5,589381723	1,51E+01	1,00E-04	2,42E-03	St3gal2		0,83	Yes
ENSMUSG00000031750	-3,61E+00	-2,65506321	5,86E+00	1,54E-02	1,29E-01	Il34		0,08	No
ENSMUSG00000031751	2,57E-02	7,540015881	1,89E-01	6,64E-01	8,84E-01	Amfr		1,02	No
ENSMUSG00000031753	1,80E-01	5,223207138	6,37E+00	1,16E-02	1,04E-01	Cog4		1,13	No
ENSMUSG00000031754	5,49E-02	4,559309239	4,73E-01	4,92E-01	7,78E-01	Nudt21		1,04	No
ENSMUSG00000031755	-5,44E-02	2,015710855	1,33E-01	7,15E-01	9,13E-01	Bbs2		0,96	No
ENSMUSG00000031756	-6,72E-02	3,662700331	4,92E-01	4,83E-01	7,70E-01	Cenpn		0,95	No
ENSMUSG00000031758	-3,35E-01	2,791967326	7,85E+00	5,09E-03	5,66E-02	Cdyl2		0,79	No
ENSMUSG00000031760	-3,45E-01	-0,485077137	9,37E-01	3,33E-01	6,34E-01	Mt3		0,79	No
ENSMUSG00000031762	-1,97E-03	5,440836093	8,05E-04	9,77E-01	1,00E+00	Mt2		1,00	No
ENSMUSG00000031765	1,19E-01	7,663158056	4,07E+00	4,37E-02	2,49E-01	Mt1		1,09	No
ENSMUSG00000031766	-7,86E-02	-1,22620341	3,03E-02	8,62E-01	9,96E-01	Slc12a3		0,95	No

ENSMUSG00000031767	3,48E-01	3,355925119	1,13E+01	7,72E-04	1,31E-02	Nudt7	1,27	Yes
ENSMUSG00000031770	-1,31E-02	7,228919587	4,75E-02	8,27E-01	9,76E-01	Herpud1	0,99	No
ENSMUSG00000031774	-7,05E-03	5,286919781	9,97E-03	9,20E-01	1,00E+00	Fam192a	1,00	No
ENSMUSG00000031775	-1,33E+00	-2,015796997	3,71E+00	5,40E-02	2,84E-01	Pllp	0,40	No
ENSMUSG00000031776	-7,28E-02	6,206884529	1,28E+00	2,58E-01	5,50E-01	Arl2bp	0,95	No
ENSMUSG00000031778	-7,33E-01	5,761713303	1,21E+02	4,35E-28	2,14E-25	Cx3cl1	0,60	Yes
ENSMUSG00000031779	-9,48E-01	10,90712646	2,82E+02	3,33E-63	2,00E-59	Ccl22	0,52	Yes
ENSMUSG00000031780	2,86E-02	10,35099992	2,59E-01	6,10E-01	8,52E-01	Ccl17	1,02	No
ENSMUSG00000031781	-7,92E-02	5,196638348	1,21E+00	2,70E-01	5,67E-01	Ciapin1	0,95	No
ENSMUSG00000031782	-1,92E-03	5,155838305	7,10E-04	9,79E-01	1,00E+00	Coq9	1,00	No
ENSMUSG00000031783	1,67E-01	3,729104523	3,07E+00	7,97E-02	3,61E-01	Polr2c	1,12	No
ENSMUSG00000031785	5,07E-01	1,032082988	5,80E+00	1,60E-02	1,32E-01	Adgrg1	1,42	No
ENSMUSG00000031786	-1,24E+00	-2,600637655	1,47E+00	2,26E-01	5,50E-01	Drc7	0,42	No
ENSMUSG00000031787	8,08E-02	4,222232047	9,03E-01	3,42E-01	6,42E-01	Katnb1	1,06	No
ENSMUSG00000031788	2,20E-01	2,298784932	2,47E+00	1,16E-01	4,21E-01	Kifc3	1,17	No
ENSMUSG00000031789	-8,01E-01	-1,896127859	1,52E+00	2,18E-01	5,50E-01	Cngb1	0,57	No
ENSMUSG00000031790	-9,83E-01	-2,107640593	1,82E+00	1,78E-01	5,38E-01	Mmp15	0,51	No
ENSMUSG00000031791	2,82E-02	1,120281549	1,95E-02	8,89E-01	1,00E+00	Tmem38a	1,02	No
ENSMUSG00000031792	3,55E-02	5,433184298	2,57E-01	6,12E-01	8,52E-01	Usb1	1,02	No
ENSMUSG00000031796	4,34E-02	5,024894076	3,44E-01	5,58E-01	8,21E-01	Cfap20	1,03	No
ENSMUSG00000031799	-1,20E-02	8,554196538	4,37E-02	8,34E-01	9,79E-01	Tpm4	0,99	No
ENSMUSG00000031802	-7,89E-02	-2,226368205	1,16E-02	9,14E-01	1,00E+00	Phxr4	0,95	No
ENSMUSG00000031803	2,59E-01	4,014972566	8,57E+00	3,41E-03	4,22E-02	B3gnt3	1,20	Yes
ENSMUSG00000031805	-2,72E-01	4,97047666	1,35E+01	2,34E-04	4,96E-03	Jak3	0,83	Yes
ENSMUSG00000031807	-4,43E-02	6,013959955	4,62E-01	4,97E-01	7,82E-01	Pgls	0,97	No
ENSMUSG00000031808	3,23E-01	6,10903131	2,46E+01	6,90E-07	3,19E-05	Slc27a1	1,25	Yes
ENSMUSG00000031809	-4,80E-02	-2,896135597	1,06E-03	9,74E-01	1,00E+00	1700018B08Rik	0,97	No
ENSMUSG00000031812	1,76E-02	7,30618941	8,65E-02	7,69E-01	9,41E-01	Map1lc3b	1,01	No
ENSMUSG00000031813	-1,75E-02	4,866468955	5,33E-02	8,17E-01	9,70E-01	Mvb12a	0,99	No
ENSMUSG00000031816	-5,99E-02	3,720012885	4,15E-01	5,20E-01	7,99E-01	Mthfsd	0,96	No
ENSMUSG00000031818	-7,11E-02	7,961729521	1,49E+00	2,22E-01	5,50E-01	Cox4i1	0,95	No
ENSMUSG00000031819	-1,58E-01	5,422221345	5,15E+00	2,32E-02	1,67E-01	Emc8	0,90	No

ENSMUSG00000031820	-7,97E-02	5,841695762	1,43E+00	2,32E-01	5,50E-01	Babam1	0,95	No
ENSMUSG00000031821	7,84E-02	5,194295007	1,19E+00	2,75E-01	5,72E-01	Gins2	1,06	No
ENSMUSG00000031822	9,39E-02	5,995206297	2,07E+00	1,50E-01	4,91E-01	Gse1	1,07	No
ENSMUSG00000031823	7,15E-02	5,985708012	1,19E+00	2,76E-01	5,72E-01	Zdhhc7	1,05	No
ENSMUSG00000031824	1,98E-01	5,566553184	8,37E+00	3,81E-03	4,56E-02	6430548M08Rik	1,15	Yes
ENSMUSG00000031825	-1,06E+00	2,995695573	9,32E+01	4,83E-22	1,35E-19	Crispld2	0,48	Yes
ENSMUSG00000031826	2,11E-01	4,842668911	7,77E+00	5,32E-03	5,85E-02	Usp10	1,16	No
ENSMUSG00000031827	8,49E-02	9,938603195	2,28E+00	1,31E-01	4,56E-01	Cotl1	1,06	No
ENSMUSG00000031828	1,44E-01	4,106849163	2,74E+00	9,76E-02	3,90E-01	Klhl36	1,11	No
ENSMUSG00000031831	1,43E-01	0,92756405	4,62E-01	4,97E-01	7,82E-01	Dnaaf1	1,10	No
ENSMUSG00000031832	2,70E-01	3,824211176	8,65E+00	3,27E-03	4,09E-02	Taf1c	1,21	Yes
ENSMUSG00000031833	1,78E-01	6,509598349	8,09E+00	4,44E-03	5,10E-02	Mast3	1,13	No
ENSMUSG00000031834	1,88E-02	5,672443693	7,71E-02	7,81E-01	9,49E-01	Pik3r2	1,01	No
ENSMUSG00000031835	3,34E-02	6,529899657	2,87E-01	5,92E-01	8,42E-01	Mbtps1	1,02	No
ENSMUSG00000031837	-2,68E+00	-2,896144247	2,85E+00	9,15E-02	3,82E-01	Necab2	0,16	No
ENSMUSG00000031838	-1,83E-02	8,187736831	9,99E-02	7,52E-01	9,32E-01	Ifi30	0,99	No
ENSMUSG00000031839	2,32E-02	6,273167246	1,32E-01	7,16E-01	9,13E-01	Hsbp1	1,02	No
ENSMUSG00000031840	4,99E-01	1,191015107	6,60E+00	1,02E-02	9,49E-02	Rab3a	1,41	No
ENSMUSG00000031842	-3,74E-01	-2,401456946	1,76E-01	6,75E-01	8,91E-01	Pde4c	0,77	No
ENSMUSG00000031843	2,32E-01	3,577451455	5,58E+00	1,81E-02	1,42E-01	Mphosph6	1,17	No
ENSMUSG00000031845	7,05E-01	-2,963278876	2,69E-01	6,04E-01	8,49E-01	Bco1	1,63	No
ENSMUSG00000031847	-1,71E-01	0,425813137	4,88E-01	4,85E-01	7,72E-01	1700030J22Rik	0,89	No
ENSMUSG00000031848	-1,11E-01	5,369591123	2,47E+00	1,16E-01	4,21E-01	Lsm4	0,93	No
ENSMUSG00000031849	-2,45E-02	0,244551169	8,14E-03	9,28E-01	1,00E+00	Comp	0,98	No
ENSMUSG00000031851	8,31E-02	3,94243219	8,49E-01	3,57E-01	6,58E-01	Ntpcr	1,06	No
ENSMUSG00000031853	2,67E+00	-3,033636951	2,76E+00	9,68E-02	3,88E-01	Map3k21	6,36	No
ENSMUSG00000031858	-1,31E-02	6,462165734	4,35E-02	8,35E-01	9,80E-01	Mau2	0,99	No
ENSMUSG00000031860	2,12E-01	-2,448618302	5,62E-02	8,13E-01	9,67E-01	Pbx4	1,16	No
ENSMUSG00000031861	2,19E-01	2,323944517	2,61E+00	1,07E-01	3,99E-01	Lpar2	1,16	No
ENSMUSG00000031862	1,38E-01	6,444677498	4,80E+00	2,85E-02	1,91E-01	Atp13a1	1,10	No
ENSMUSG00000031864	1,44E-01	4,765922154	3,56E+00	5,92E-02	3,01E-01	Ints10	1,11	No
ENSMUSG00000031865	2,73E-02	7,279874055	2,08E-01	6,48E-01	8,75E-01	Dctn1	1,02	No

ENSMUSG00000031870	8,65E-01	-2,448637117	9,01E-01	3,43E-01	6,43E-01	Pgr	1,82	No
ENSMUSG00000031871	4,13E-01	1,428553774	5,11E+00	2,38E-02	1,69E-01	Cdh5	1,33	No
ENSMUSG00000031872	-1,21E-01	-1,185691451	6,41E-02	8,00E-01	9,60E-01	Bean1	0,92	No
ENSMUSG00000031875	1,88E-01	6,924140653	9,53E+00	2,02E-03	2,83E-02	Cmtm3	1,14	Yes
ENSMUSG00000031876	1,55E-01	-2,370500987	2,93E-02	8,64E-01	9,97E-01	Gm45711	1,11	No
ENSMUSG00000031877	-7,47E-01	-2,497598382	6,26E-01	4,29E-01	7,23E-01	Ces2g	0,60	No
ENSMUSG00000031878	-2,08E-01	3,998316691	5,50E+00	1,90E-02	1,46E-01	Nae1	0,87	No
ENSMUSG00000031879	-2,31E-01	3,649984108	5,81E+00	1,60E-02	1,31E-01	Fam96b	0,85	No
ENSMUSG00000031880	-4,47E-01	0,458196844	3,19E+00	7,43E-02	3,47E-01	Rrad	0,73	No
ENSMUSG00000031881	-2,74E+00	-3,033596063	2,99E+00	8,38E-02	3,71E-01	Cdh16	0,15	No
ENSMUSG00000031883	2,14E-01	-0,389782407	3,85E-01	5,35E-01	8,08E-01	Car7	1,16	No
ENSMUSG00000031884	-5,04E-02	-2,740892023	1,73E-03	9,67E-01	1,00E+00	Ces2d-ps	0,97	No
ENSMUSG00000031885	1,80E-01	7,441213931	9,17E+00	2,47E-03	3,30E-02	Cbfb	1,13	Yes
ENSMUSG00000031887	-5,53E-02	4,760677264	5,21E-01	4,71E-01	7,59E-01	Tradd	0,96	No
ENSMUSG00000031889	1,20E-01	4,2274053	2,05E+00	1,52E-01	4,94E-01	D230025D16Rik	1,09	No
ENSMUSG00000031891	3,38E-01	3,238910317	9,91E+00	1,65E-03	2,39E-02	Hsd11b2	1,26	Yes
ENSMUSG00000031893	1,26E+00	-1,801134937	3,68E+00	5,51E-02	2,88E-01	Tsnaxip1	2,40	No
ENSMUSG00000031896	5,17E-01	0,078984004	3,37E+00	6,64E-02	3,24E-01	Ctrl	1,43	No
ENSMUSG00000031897	-1,59E-01	5,764777809	5,61E+00	1,79E-02	1,40E-01	Psmb10	0,90	No
ENSMUSG00000031898	-2,39E-01	4,261193419	7,89E+00	4,96E-03	5,57E-02	Dpep3	0,85	No
ENSMUSG00000031901	-4,19E-02	3,280733638	1,57E-01	6,92E-01	9,00E-01	Dus2	0,97	No
ENSMUSG00000031902	9,15E-02	6,844052092	2,23E+00	1,35E-01	4,63E-01	Nfatc3	1,07	No
ENSMUSG00000031903	1,33E-01	7,206165267	4,95E+00	2,61E-02	1,80E-01	Pla2g15	1,10	No
ENSMUSG00000031904	-1,34E-01	5,159696197	3,47E+00	6,26E-02	3,12E-01	Slc7a6	0,91	No
ENSMUSG00000031906	3,04E-02	-0,195806954	8,53E-03	9,26E-01	1,00E+00	Smpd3	1,02	No
ENSMUSG00000031907	3,13E-01	4,104299609	1,30E+01	3,16E-04	6,32E-03	Zfp90	1,24	Yes
ENSMUSG00000031910	3,47E-02	3,499848172	1,22E-01	7,27E-01	9,19E-01	Has3	1,02	No
ENSMUSG00000031913	-1,12E-02	5,434570834	2,58E-02	8,72E-01	1,00E+00	Vps4a	0,99	No
ENSMUSG00000031916	1,03E-01	5,583004284	2,26E+00	1,33E-01	4,59E-01	Cog8	1,07	No
ENSMUSG00000031917	-6,47E-02	4,743664767	6,98E-01	4,04E-01	7,01E-01	Nip7	0,96	No
ENSMUSG00000031918	4,69E-02	5,388355967	4,43E-01	5,06E-01	7,90E-01	Mtmr2	1,03	No
ENSMUSG00000031919	1,35E+00	-1,831981257	3,94E+00	4,71E-02	2,60E-01	Tmed6	2,54	No

ENSMUSG00000031921	3,15E-02	4,562307841	1,62E-01	6,88E-01	8,98E-01	Terf2	1,02	No
ENSMUSG00000031922	1,15E-02	4,918375268	2,36E-02	8,78E-01	1,00E+00	Cep57	1,01	No
ENSMUSG00000031924	1,72E-01	7,727766864	8,60E+00	3,36E-03	4,17E-02	Cyb5b	1,13	Yes
ENSMUSG00000031925	3,81E-01	4,11939073	1,91E+01	1,24E-05	4,07E-04	Maml2	1,30	Yes
ENSMUSG00000031927	-1,89E+00	-3,1076441	1,42E+00	2,33E-01	5,50E-01	1700012B09Rik	0,27	No
ENSMUSG00000031928	9,35E-02	4,760902998	1,48E+00	2,24E-01	5,50E-01	Mre11a	1,07	No
ENSMUSG00000031930	3,36E-02	6,216573245	2,75E-01	6,00E-01	8,47E-01	Wwp2	1,02	No
ENSMUSG00000031931	7,43E-02	4,343837693	8,11E-01	3,68E-01	6,69E-01	Ankrd49	1,05	No
ENSMUSG00000031932	-1,18E+00	-2,655080443	1,33E+00	2,48E-01	5,50E-01	Gpr83	0,44	No
ENSMUSG00000031933	1,81E+00	-3,10765737	1,28E+00	2,58E-01	5,50E-01	Izumo1r	3,49	No
ENSMUSG00000031934	-2,26E-01	4,298612939	7,30E+00	6,89E-03	7,09E-02	Panx1	0,86	No
ENSMUSG00000031935	6,64E-02	5,469740999	9,22E-01	3,37E-01	6,38E-01	Med17	1,05	No
ENSMUSG00000031936	-3,19E+00	-2,896117911	4,30E+00	3,81E-02	2,32E-01	Hephl1	0,11	No
ENSMUSG00000031937	3,80E+00	-2,600708295	6,68E+00	9,76E-03	9,16E-02	Vstm5	13,97	No
ENSMUSG00000031938	-1,00E-02	5,117703586	1,91E-02	8,90E-01	1,00E+00	4931406C07Rik	0,99	No
ENSMUSG00000031939	-2,12E-01	5,33543342	8,95E+00	2,77E-03	3,60E-02	Taf1d	0,86	Yes
ENSMUSG00000031948	-6,77E-03	5,885243583	1,05E-02	9,19E-01	1,00E+00	Kars	1,00	No
ENSMUSG00000031949	-1,08E-01	1,002097506	3,05E-01	5,81E-01	8,37E-01	Adat1	0,93	No
ENSMUSG00000031950	-1,09E-01	4,120567544	1,59E+00	2,07E-01	5,50E-01	Gabarapl2	0,93	No
ENSMUSG00000031951	2,69E-01	0,464576193	1,10E+00	2,95E-01	5,90E-01	Tmem231	1,21	No
ENSMUSG00000031953	1,37E-01	0,943766064	4,49E-01	5,03E-01	7,88E-01	Tmem170	1,10	No
ENSMUSG00000031954	-1,00E-01	5,350542155	2,03E+00	1,54E-01	4,97E-01	Cfdp1	0,93	No
ENSMUSG00000031955	7,43E-01	-1,289611598	2,36E+00	1,25E-01	4,42E-01	Bcar1	1,67	No
ENSMUSG00000031957	3,14E-02	-3,107617333	-7,69E-10	1,00E+00	1,00E+00	Ctrb1	1,02	No
ENSMUSG00000031958	-8,59E-02	0,999643063	1,72E-01	6,78E-01	8,92E-01	Ldhd	0,94	No
ENSMUSG00000031959	1,09E-01	3,644452062	1,27E+00	2,59E-01	5,52E-01	Wdr59	1,08	No
ENSMUSG00000031960	4,79E-02	7,195093276	6,36E-01	4,25E-01	7,19E-01	Aars	1,03	No
ENSMUSG00000031962	7,30E-01	-2,655143745	2,91E-01	5,90E-01	8,41E-01	Cdh15	1,66	No
ENSMUSG00000031963	-1,46E-01	-1,165802316	1,02E-01	7,49E-01	9,31E-01	Bmper	0,90	No
ENSMUSG00000031965	-1,89E+00	-3,1076441	1,42E+00	2,33E-01	5,50E-01	Tbx20	0,27	No
ENSMUSG00000031967	1,18E-01	5,710047588	3,06E+00	8,04E-02	3,64E-01	Afg3l1	1,09	No
ENSMUSG00000031969	5,05E-02	4,539149933	4,02E-01	5,26E-01	8,03E-01	Acad8	1,04	No

ENSMUSG00000031970	-9,62E-02	1,708227219	3,33E-01	5,64E-01	8,27E-01	Dbndd1	0,94	No
ENSMUSG00000031971	2,67E-01	2,406678805	4,21E+00	4,03E-02	2,38E-01	Ccsap	1,20	No
ENSMUSG00000031972	-1,87E+00	-3,107637761	1,39E+00	2,38E-01	5,50E-01	Acta1	0,27	No
ENSMUSG00000031974	1,56E-01	4,829845047	4,17E+00	4,11E-02	2,41E-01	Abcb10	1,11	No
ENSMUSG00000031976	1,70E-01	4,373971638	4,28E+00	3,86E-02	2,33E-01	Urb2	1,13	No
ENSMUSG00000031979	4,95E-02	5,284489478	4,85E-01	4,86E-01	7,73E-01	Cog2	1,03	No
ENSMUSG00000031980	-2,65E+00	-2,963248364	2,80E+00	9,42E-02	3,84E-01	Agt	0,16	No
ENSMUSG00000031981	-6,25E-02	-2,831990963	1,72E-03	9,67E-01	1,00E+00	Capn9	0,96	No
ENSMUSG00000031982	3,55E-01	1,988265019	5,52E+00	1,88E-02	1,45E-01	Arv1	1,28	No
ENSMUSG00000031983	2,41E-01	4,536794263	9,01E+00	2,68E-03	3,51E-02	2310022B05Rik	1,18	Yes
ENSMUSG00000031984	1,19E-01	4,466945842	2,16E+00	1,42E-01	4,76E-01	2810004N23Rik	1,09	No
ENSMUSG00000031985	8,69E-02	5,677187685	1,64E+00	2,00E-01	5,50E-01	Gnpat	1,06	No
ENSMUSG00000031986	2,59E-05	5,081293088	1,20E-07	1,00E+00	1,00E+00	Sprtn	1,00	No
ENSMUSG00000031987	1,52E-01	6,18694667	5,57E+00	1,83E-02	1,42E-01	Egln1	1,11	No
ENSMUSG00000031988	8,07E-02	5,942708942	1,50E+00	2,20E-01	5,50E-01	Vps26b	1,06	No
ENSMUSG00000031990	5,74E-01	1,097630772	7,57E+00	5,93E-03	6,36E-02	Jam3	1,49	No
ENSMUSG00000031993	1,16E-01	5,776403392	2,98E+00	8,46E-02	3,73E-01	Snx19	1,08	No
ENSMUSG00000031994	-1,22E+00	-3,070169701	7,07E-01	4,01E-01	6,99E-01	Adamts8	0,43	No
ENSMUSG00000031995	-4,80E-01	4,83865481	4,04E+01	2,07E-10	1,85E-08	St14	0,72	Yes
ENSMUSG00000031996	-4,82E-02	9,10160949	7,21E-01	3,96E-01	6,96E-01	Aplp2	0,97	No
ENSMUSG00000031997	3,14E-02	-3,107617333	-7,69E-10	1,00E+00	1,00E+00	Trpc6	1,02	No
ENSMUSG00000032000	-3,53E-01	6,582426499	3,23E+01	1,33E-08	8,96E-07	Birc3	0,78	Yes
ENSMUSG00000032002	4,01E-02	5,430807381	3,30E-01	5,66E-01	8,28E-01	Dcun1d5	1,03	No
ENSMUSG00000032009	-3,70E-01	5,711997315	3,06E+01	3,24E-08	2,00E-06	Sesn3	0,77	Yes
ENSMUSG00000032010	1,47E-01	0,995272983	5,09E-01	4,76E-01	7,64E-01	Usp2	1,11	No
ENSMUSG00000032011	-1,47E+00	1,338305721	6,26E+01	2,59E-15	4,09E-13	Thy1	0,36	Yes
ENSMUSG00000032012	-3,39E-01	3,671411384	1,29E+01	3,37E-04	6,66E-03	Nectin1	0,79	Yes
ENSMUSG00000032013	1,10E-01	2,725245845	8,06E-01	3,69E-01	6,71E-01	Trim29	1,08	No
ENSMUSG00000032014	6,71E-01	0,804197553	8,73E+00	3,13E-03	3,95E-02	Oaf	1,59	Yes
ENSMUSG00000032017	-5,45E-02	-2,832025911	1,34E-03	9,71E-01	1,00E+00	Grik4	0,96	No
ENSMUSG00000032018	1,50E-01	5,609550858	4,86E+00	2,74E-02	1,87E-01	Sc5d	1,11	No
ENSMUSG00000032020	8,77E-03	6,733802684	2,02E-02	8,87E-01	1,00E+00	Ubash3b	1,01	No

ENSMUSG00000032021	-7,40E-01	-0,355411012	3,68E+00	5,51E-02	2,88E-01	Crtam	0,60	No
ENSMUSG00000032024	4,13E-02	4,613530306	2,76E-01	5,99E-01	8,46E-01	Clmp	1,03	No
ENSMUSG00000032026	-4,48E-02	5,881869209	4,56E-01	4,99E-01	7,85E-01	Rexo2	0,97	No
ENSMUSG00000032028	-1,68E+00	-2,401364405	3,09E+00	7,87E-02	3,59E-01	Nxpe2	0,31	No
ENSMUSG00000032030	1,27E-01	4,977105099	2,92E+00	8,73E-02	3,78E-01	Cul5	1,09	No
ENSMUSG00000032033	-1,22E+00	-2,44860177	1,93E+00	1,65E-01	5,18E-01	Barx2	0,43	No
ENSMUSG00000032034	-1,89E+00	-3,033648118	1,42E+00	2,33E-01	5,50E-01	Kcnj5	0,27	No
ENSMUSG00000032035	3,09E-01	0,666365533	1,87E+00	1,71E-01	5,27E-01	Ets1	1,24	No
ENSMUSG00000032036	-3,56E-01	-0,485250186	7,80E-01	3,77E-01	6,78E-01	Kirrel3	0,78	No
ENSMUSG00000032038	-1,15E-02	5,628958515	2,85E-02	8,66E-01	9,99E-01	St3gal4	0,99	No
ENSMUSG00000032040	-7,07E-02	4,30477275	7,18E-01	3,97E-01	6,97E-01	Dcps	0,95	No
ENSMUSG00000032041	3,29E-01	4,317344065	1,55E+01	8,29E-05	2,07E-03	Tirap	1,26	Yes
ENSMUSG00000032042	3,59E-02	6,256834421	3,15E-01	5,75E-01	8,34E-01	Srpr	1,03	No
ENSMUSG00000032044	5,84E-02	3,964747723	4,32E-01	5,11E-01	7,94E-01	Rpusd4	1,04	No
ENSMUSG00000032046	1,37E-01	6,390161784	4,71E+00	3,00E-02	1,99E-01	Abhd12	1,10	No
ENSMUSG00000032047	-2,85E-01	6,400466653	2,04E+01	6,20E-06	2,18E-04	Acat1	0,82	Yes
ENSMUSG00000032050	-6,66E-02	7,072875881	1,22E+00	2,70E-01	5,66E-01	Rdx	0,95	No
ENSMUSG00000032051	-3,59E-02	3,36596152	1,21E-01	7,28E-01	9,20E-01	Fdx1	0,98	No
ENSMUSG00000032053	-1,00E+00	-2,40135181	1,22E+00	2,69E-01	5,66E-01	Pou2af1	0,50	No
ENSMUSG00000032056	-8,61E-01	-2,896108407	4,07E-01	5,23E-01	8,01E-01	Btg4	0,55	No
ENSMUSG00000032057	-3,74E-01	-2,070172102	3,24E-01	5,69E-01	8,30E-01	4833427G06Rik	0,77	No
ENSMUSG00000032058	7,73E-02	5,599275657	1,28E+00	2,57E-01	5,50E-01	Ppp2r1b	1,06	No
ENSMUSG00000032059	-3,71E-02	4,358053984	2,03E-01	6,52E-01	8,77E-01	Alg9	0,97	No
ENSMUSG00000032060	1,73E-01	1,175764913	8,01E-01	3,71E-01	6,72E-01	Cryab	1,13	No
ENSMUSG00000032062	-5,70E-02	-1,963290753	6,04E-03	9,38E-01	1,00E+00	2310030G06Rik	0,96	No
ENSMUSG00000032064	3,13E-01	-0,070362458	1,05E+00	3,05E-01	6,02E-01	Dixdc1	1,24	No
ENSMUSG00000032065	8,89E-01	-1,711734775	2,27E+00	1,32E-01	4,57E-01	Tex12	1,85	No
ENSMUSG00000032066	2,78E-01	1,438650229	2,31E+00	1,28E-01	4,49E-01	Bco2	1,21	No
ENSMUSG00000032067	-1,11E-01	5,682713826	2,71E+00	9,99E-02	3,92E-01	Pts	0,93	No
ENSMUSG00000032068	2,25E-01	10,42192372	1,61E+01	6,11E-05	1,61E-03	Plet1	1,17	Yes
ENSMUSG00000032076	2,41E-01	1,991062795	2,52E+00	1,13E-01	4,14E-01	Cadm1	1,18	No
ENSMUSG00000032077	-6,97E-03	3,782798546	5,52E-03	9,41E-01	1,00E+00	Bud13	1,00	No

ENSMUSG00000032078	-5,29E-02	5,28986474	5,57E-01	4,55E-01	7,45E-01	Zpr1	0,96	No
ENSMUSG00000032079	-1,27E+00	-3,146104694	7,49E-01	3,87E-01	6,87E-01	Apoa5	0,42	No
ENSMUSG00000032080	-1,23E+00	-3,146117124	7,12E-01	3,99E-01	6,98E-01	Apoa4	0,42	No
ENSMUSG00000032083	-1,92E+00	-3,033623267	1,46E+00	2,27E-01	5,50E-01	Apoa1	0,26	No
ENSMUSG00000032085	1,17E-01	2,155452464	6,46E-01	4,22E-01	7,17E-01	Tagln	1,08	No
ENSMUSG00000032086	8,33E-03	4,498008362	1,08E-02	9,17E-01	1,00E+00	Bace1	1,01	No
ENSMUSG00000032087	-4,97E-01	1,66293803	9,76E+00	1,79E-03	2,56E-02	Dscaml1	0,71	Yes
ENSMUSG00000032089	-1,62E-02	7,742372332	7,59E-02	7,83E-01	9,50E-01	Il10ra	0,99	No
ENSMUSG00000032091	-7,93E-02	-1,436775059	2,58E-02	8,72E-01	1,00E+00	Tmprss4	0,95	No
ENSMUSG00000032092	-1,66E+00	1,963794455	1,17E+02	2,25E-27	1,01E-24	Mpzl2	0,32	Yes
ENSMUSG00000032093	-3,65E-01	-2,800993776	9,79E-02	7,54E-01	9,34E-01	Cd3e	0,78	No
ENSMUSG00000032094	-1,69E+00	-2,107523357	5,50E+00	1,90E-02	1,46E-01	Cd3d	0,31	No
ENSMUSG00000032096	-4,84E-02	7,799548919	6,83E-01	4,09E-01	7,05E-01	Arcn1	0,97	No
ENSMUSG00000032097	-7,56E-02	8,531892348	1,74E+00	1,88E-01	5,50E-01	Ddx6	0,95	No
ENSMUSG00000032098	3,06E-01	-2,548195936	9,54E-02	7,57E-01	9,35E-01	Treh	1,24	No
ENSMUSG00000032099	5,97E-03	-3,1461183	-3,40E-10	1,00E+00	1,00E+00	Pate4	1,00	No
ENSMUSG00000032101	-1,89E+00	-3,1076441	1,42E+00	2,33E-01	5,50E-01	Ddx25	0,27	No
ENSMUSG00000032103	-2,07E-02	2,13083179	2,14E-02	8,84E-01	1,00E+00	Pus3	0,99	No
ENSMUSG00000032105	-1,15E-01	-0,561123751	1,03E-01	7,48E-01	9,30E-01	Pdzd3	0,92	No
ENSMUSG00000032109	1,90E-01	5,458568102	7,50E+00	6,17E-03	6,56E-02	Nlrx1	1,14	No
ENSMUSG00000032110	2,25E+00	-2,998050941	1,94E+00	1,64E-01	5,15E-01	Acrv1	4,74	No
ENSMUSG00000032112	-1,11E-02	5,447032054	2,56E-02	8,73E-01	1,00E+00	Trappc4	0,99	No
ENSMUSG00000032113	-9,33E-03	2,951601893	6,61E-03	9,35E-01	1,00E+00	Chek1	0,99	No
ENSMUSG00000032114	1,23E-01	4,576526451	2,41E+00	1,21E-01	4,33E-01	Slc37a4	1,09	No
ENSMUSG00000032115	1,25E-01	7,412134388	4,39E+00	3,62E-02	2,26E-01	Hyou1	1,09	No
ENSMUSG00000032116	1,19E-01	7,421955456	3,98E+00	4,60E-02	2,56E-01	Stt3a	1,09	No
ENSMUSG00000032118	1,87E+00	-3,03364371	1,36E+00	2,43E-01	5,50E-01	Fez1	3,66	No
ENSMUSG00000032119	3,16E-02	4,976237176	1,82E-01	6,70E-01	8,88E-01	Hinfp	1,02	No
ENSMUSG00000032120	8,66E-02	6,078316044	1,76E+00	1,84E-01	5,48E-01	C2cd2l	1,06	No
ENSMUSG00000032121	2,68E-01	2,001846007	3,20E+00	7,38E-02	3,45E-01	Tmem218	1,20	No
ENSMUSG00000032122	2,42E-01	5,656761445	1,28E+01	3,54E-04	6,92E-03	Slc37a2	1,18	Yes
ENSMUSG00000032123	5,45E-02	5,73136991	6,56E-01	4,18E-01	7,13E-01	Dpagt1	1,04	No

ENSMUSG00000032125	-1,53E+00	-0,311106154	2,39E+01	1,01E-06	4,47E-05	Robo4	0,35	Yes
ENSMUSG00000032126	2,41E-01	3,638468094	6,33E+00	1,18E-02	1,06E-01	Hmbs	1,18	No
ENSMUSG00000032127	1,73E-02	6,602757277	7,75E-02	7,81E-01	9,49E-01	Vps11	1,01	No
ENSMUSG00000032128	-3,77E-01	-0,816353095	8,42E-01	3,59E-01	6,60E-01	Robo3	0,77	No
ENSMUSG00000032131	-8,79E-01	1,542771346	2,49E+01	5,95E-07	2,78E-05	Abcg4	0,54	Yes
ENSMUSG00000032135	-3,82E-01	0,33789686	2,14E+00	1,44E-01	4,79E-01	Mcam	0,77	No
ENSMUSG00000032171	-1,15E-02	5,078490943	2,45E-02	8,76E-01	1,00E+00	Pin1	0,99	No
ENSMUSG00000032172	8,67E-01	-1,896237312	1,41E+00	2,36E-01	5,50E-01	Olfm2	1,82	No
ENSMUSG00000032174	-5,49E-01	-0,503825688	2,36E+00	1,25E-01	4,42E-01	Icam5	0,68	No
ENSMUSG00000032175	-7,10E-02	7,948275947	1,49E+00	2,22E-01	5,50E-01	Tyk2	0,95	No
ENSMUSG00000032177	1,61E-01	4,265940886	3,69E+00	5,47E-02	2,86E-01	Pde4a	1,12	No
ENSMUSG00000032178	3,90E-02	5,91908171	3,50E-01	5,54E-01	8,20E-01	Ilf3	1,03	No
ENSMUSG00000032179	-1,23E+00	-3,146117124	7,12E-01	3,99E-01	6,98E-01	Bmp5	0,42	No
ENSMUSG00000032180	1,49E-01	2,667961477	1,44E+00	2,30E-01	5,50E-01	Tmed1	1,11	No
ENSMUSG00000032181	1,85E+00	-3,107657832	1,35E+00	2,46E-01	5,50E-01	Scg3	3,61	No
ENSMUSG00000032182	1,05E-02	4,135809324	1,48E-02	9,03E-01	1,00E+00	Yipf2	1,01	No
ENSMUSG00000032184	-7,42E-01	0,386080287	8,97E+00	2,75E-03	3,58E-02	Lysmd2	0,60	Yes
ENSMUSG00000032185	-8,78E-02	5,240581386	1,51E+00	2,19E-01	5,50E-01	Carm1	0,94	No
ENSMUSG00000032186	-8,87E-01	3,344029875	7,64E+01	2,34E-18	4,71E-16	Tmod2	0,54	Yes
ENSMUSG00000032187	5,92E-02	7,593117635	1,01E+00	3,16E-01	6,16E-01	Smarca4	1,04	No
ENSMUSG00000032191	1,87E+00	-3,107639691	1,38E+00	2,40E-01	5,50E-01	Bcl2l10	3,67	No
ENSMUSG00000032192	4,02E-02	1,899156414	6,62E-02	7,97E-01	9,58E-01	Gnb5	1,03	No
ENSMUSG00000032193	2,23E-01	7,863841562	1,46E+01	1,34E-04	3,09E-03	Ldlr	1,17	Yes
ENSMUSG00000032194	-1,59E-01	1,966581079	1,08E+00	2,98E-01	5,94E-01	Kank2	0,90	No
ENSMUSG00000032198	-2,70E-01	3,07029378	5,94E+00	1,48E-02	1,25E-01	Dock6	0,83	No
ENSMUSG00000032199	-2,57E-02	6,795301634	1,75E-01	6,75E-01	8,91E-01	Polr2m	0,98	No
ENSMUSG00000032202	-5,10E-01	4,678507235	4,33E+01	4,59E-11	4,53E-09	Rab27a	0,70	Yes
ENSMUSG00000032204	-1,68E-01	5,58329822	6,13E+00	1,33E-02	1,15E-01	Aqp9	0,89	No
ENSMUSG00000032207	-2,35E-01	-2,226386544	1,08E-01	7,42E-01	9,27E-01	Lipc	0,85	No
ENSMUSG00000032212	-2,69E-02	6,239683075	1,78E-01	6,73E-01	8,90E-01	Sltm	0,98	No
ENSMUSG00000032215	8,56E-02	4,711691182	1,22E+00	2,69E-01	5,66E-01	Rsl24d1	1,06	No
ENSMUSG00000032216	-3,34E-01	6,792300644	2,99E+01	4,65E-08	2,80E-06	Nedd4	0,79	Yes

ENSMUSG00000032217	8,74E-02	6,141898588	1,84E+00	1,75E-01	5,33E-01	Rnf111	1,06	No
ENSMUSG00000032218	1,31E-01	5,349976531	3,48E+00	6,21E-02	3,10E-01	Ccnb2	1,10	No
ENSMUSG00000032220	3,62E-01	7,125588535	3,58E+01	2,21E-09	1,69E-07	Myo1e	1,29	Yes
ENSMUSG00000032221	2,17E-01	2,817375612	3,29E+00	6,96E-02	3,32E-01	Mns1	1,16	No
ENSMUSG00000032224	-3,83E-02	-3,107651936	-7,91E-11	1,00E+00	1,00E+00	Fam81a	0,97	No
ENSMUSG00000032228	4,50E-04	6,071848906	4,76E-05	9,94E-01	1,00E+00	Tcf12	1,00	No
ENSMUSG00000032231	1,25E-01	8,43150978	4,70E+00	3,01E-02	1,99E-01	Anxa2	1,09	No
ENSMUSG00000032232	2,45E-01	7,542094572	1,72E+01	3,44E-05	9,86E-04	Cgnl1	1,19	Yes
ENSMUSG00000032235	5,29E-02	3,60732743	3,08E-01	5,79E-01	8,36E-01	Ice2	1,04	No
ENSMUSG00000032238	4,18E-02	4,298730595	2,50E-01	6,17E-01	8,55E-01	Rora	1,03	No
ENSMUSG00000032239	-8,11E-02	5,070023933	1,23E+00	2,68E-01	5,65E-01	Rp9	0,95	No
ENSMUSG00000032243	-7,19E-01	-2,548196401	5,79E-01	4,47E-01	7,37E-01	Itga11	0,61	No
ENSMUSG00000032244	1,87E-02	5,501080501	7,40E-02	7,86E-01	9,52E-01	Fem1b	1,01	No
ENSMUSG00000032245	2,00E-01	5,266697922	7,82E+00	5,17E-03	5,73E-02	Cln6	1,15	No
ENSMUSG00000032246	2,78E-01	-1,801010129	1,60E-01	6,89E-01	8,99E-01	Calml4	1,21	No
ENSMUSG00000032249	-2,12E-02	5,783900731	1,00E-01	7,51E-01	9,32E-01	Anp32a	0,99	No
ENSMUSG00000032251	-1,69E-03	1,309471157	8,34E-05	9,93E-01	1,00E+00	Irak1bp1	1,00	No
ENSMUSG00000032252	6,77E-02	4,725043235	7,71E-01	3,80E-01	6,81E-01	Glce	1,05	No
ENSMUSG00000032253	-9,49E-02	7,048825882	2,46E+00	1,17E-01	4,23E-01	Phip	0,94	No
ENSMUSG00000032254	1,81E-01	5,668097145	7,10E+00	7,69E-03	7,68E-02	Kif23	1,13	No
ENSMUSG00000032258	3,41E-01	1,723338375	4,47E+00	3,46E-02	2,19E-01	Lca5	1,27	No
ENSMUSG00000032261	4,66E-01	3,857135393	2,50E+01	5,86E-07	2,75E-05	Sh3bgrl2	1,38	Yes
ENSMUSG00000032262	-1,99E+00	-2,65512892	2,99E+00	8,39E-02	3,71E-01	Elovl4	0,25	No
ENSMUSG00000032263	-2,09E-02	1,79148362	1,63E-02	8,99E-01	1,00E+00	Bckdhb	0,99	No
ENSMUSG00000032264	-1,50E-01	5,450814621	4,70E+00	3,02E-02	1,99E-01	Zw10	0,90	No
ENSMUSG00000032265	-2,54E-01	6,273024697	1,58E+01	7,08E-05	1,82E-03	Fam46a	0,84	Yes
ENSMUSG00000032267	2,11E-02	4,315416184	6,41E-02	8,00E-01	9,60E-01	Usp28	1,01	No
ENSMUSG00000032268	-5,23E-02	-2,963273906	1,24E-03	9,72E-01	1,00E+00	Tmprss5	0,96	No
ENSMUSG00000032271	-1,94E-01	-1,600649005	1,09E-01	7,41E-01	9,27E-01	Nnmt	0,87	No
ENSMUSG00000032274	1,86E+00	-3,033661846	1,35E+00	2,46E-01	5,50E-01	Cyp19a1	3,62	No
ENSMUSG00000032278	6,28E-01	-2,033644301	8,00E-01	3,71E-01	6,72E-01	Paqr5	1,55	No
ENSMUSG00000032279	8,20E-02	6,071790967	1,59E+00	2,08E-01	5,50E-01	Idh3a	1,06	No

ENSMUSG00000032280	-4,64E-02	7,237671253	6,00E-01	4,39E-01	7,31E-01	Tle3	0,97	No
ENSMUSG00000032281	5,93E-01	0,81428553	6,77E+00	9,29E-03	8,84E-02	Acsbg1	1,51	No
ENSMUSG00000032285	-6,30E-01	1,71780932	1,48E+01	1,20E-04	2,83E-03	Dnaja4	0,65	Yes
ENSMUSG00000032288	4,45E-02	5,25940433	3,89E-01	5,33E-01	8,06E-01	Imp3	1,03	No
ENSMUSG00000032289	1,42E-01	-2,401387661	2,96E-02	8,63E-01	9,97E-01	Thsd4	1,10	No
ENSMUSG00000032290	-1,34E-01	7,105268211	4,98E+00	2,56E-02	1,78E-01	Ptpn9	0,91	No
ENSMUSG00000032292	1,83E+00	-2,963282593	1,33E+00	2,50E-01	5,50E-01	Nr2e3	3,56	No
ENSMUSG00000032293	1,22E-01	6,368850517	3,69E+00	5,46E-02	2,86E-01	Ireb2	1,09	No
ENSMUSG00000032294	1,69E-01	9,71684688	8,98E+00	2,73E-03	3,56E-02	Pkm	1,12	Yes
ENSMUSG00000032295	2,49E-02	5,986589389	1,45E-01	7,03E-01	9,06E-01	Man2c1	1,02	No
ENSMUSG00000032297	-9,05E-02	-1,832040213	2,30E-02	8,79E-01	1,00E+00	Celf6	0,94	No
ENSMUSG00000032298	-5,77E-02	3,346604867	3,17E-01	5,74E-01	8,33E-01	Neil1	0,96	No
ENSMUSG00000032299	-3,59E-02	5,259144644	2,52E-01	6,15E-01	8,54E-01	Commd4	0,98	No
ENSMUSG00000032300	1,67E-01	5,653283686	5,99E+00	1,44E-02	1,22E-01	1700017B05Rik	1,12	No
ENSMUSG00000032301	-4,82E-02	5,663069268	5,06E-01	4,77E-01	7,65E-01	Psma4	0,97	No
ENSMUSG00000032303	1,81E+00	-3,10765737	1,28E+00	2,58E-01	5,50E-01	Chrna3	3,49	No
ENSMUSG00000032305	1,40E-01	4,42086932	3,01E+00	8,29E-02	3,70E-01	Fam219b	1,10	No
ENSMUSG00000032306	-2,78E-01	4,931722076	1,41E+01	1,72E-04	3,83E-03	Mpi	0,82	Yes
ENSMUSG00000032307	7,13E-02	4,899630427	9,00E-01	3,43E-01	6,43E-01	Ube2q2	1,05	No
ENSMUSG00000032308	1,50E-01	4,394956157	3,36E+00	6,66E-02	3,25E-01	Ulk3	1,11	No
ENSMUSG00000032309	1,33E-01	5,467049756	3,65E+00	5,59E-02	2,90E-01	Fbxo22	1,10	No
ENSMUSG00000032311	1,88E-01	3,31329405	3,20E+00	7,37E-02	3,45E-01	Nrg4	1,14	No
ENSMUSG00000032312	1,21E-01	7,634342973	4,24E+00	3,96E-02	2,36E-01	Csk	1,09	No
ENSMUSG00000032313	-2,25E-01	-1,226188776	2,14E-01	6,44E-01	8,72E-01	Tmem266	0,86	No
ENSMUSG00000032314	-3,66E-02	4,383651512	2,01E-01	6,54E-01	8,79E-01	Etfa	0,97	No
ENSMUSG00000032315	-3,39E+00	-1,205629843	3,91E+01	3,95E-10	3,45E-08	Cyp1a1	0,10	Yes
ENSMUSG00000032316	-7,16E-02	6,294832652	1,26E+00	2,62E-01	5,55E-01	Clk3	0,95	No
ENSMUSG00000032318	-8,97E-01	-2,711689245	4,38E-01	5,08E-01	7,92E-01	Isl2	0,54	No
ENSMUSG00000032320	1,11E-01	5,046568962	2,25E+00	1,33E-01	4,60E-01	Rcn2	1,08	No
ENSMUSG00000032322	-7,10E-03	5,512884493	1,05E-02	9,18E-01	1,00E+00	Pstpip1	1,00	No
ENSMUSG00000032323	-7,90E-01	-0,316546156	5,01E+00	2,51E-02	1,75E-01	Cyp11a1	0,58	No
ENSMUSG00000032324	-1,31E-01	7,036046149	4,71E+00	3,00E-02	1,99E-01	Tspan3	0,91	No

ENSMUSG00000032327	-1,35E+00	-2,711637134	1,13E+00	2,87E-01	5,83E-01	Stra6	0,39	No
ENSMUSG00000032328	8,74E-02	7,690879774	2,21E+00	1,37E-01	4,67E-01	Tmem30a	1,06	No
ENSMUSG00000032329	2,13E-02	5,015237732	8,49E-02	7,71E-01	9,42E-01	Hmg20a	1,01	No
ENSMUSG00000032330	-1,24E-01	5,454690433	3,18E+00	7,45E-02	3,47E-01	Cox7a2	0,92	No
ENSMUSG00000032332	-1,10E+00	-0,895788652	6,40E+00	1,14E-02	1,03E-01	Col12a1	0,47	No
ENSMUSG00000032333	-3,22E-01	4,428781044	1,62E+01	5,84E-05	1,55E-03	Stoml1	0,80	Yes
ENSMUSG00000032334	-1,17E+00	-2,069990812	2,24E+00	1,34E-01	4,61E-01	Loxl1	0,44	No
ENSMUSG00000032336	1,73E-01	7,918796974	8,79E+00	3,02E-03	3,85E-02	Nptn	1,13	Yes
ENSMUSG00000032338	1,85E+00	-3,107657832	1,35E+00	2,46E-01	5,50E-01	Hcn4	3,61	No
ENSMUSG00000032340	-7,17E-02	0,737208369	9,62E-02	7,56E-01	9,35E-01	Neo1	0,95	No
ENSMUSG00000032342	1,45E-01	4,021912284	2,67E+00	1,02E-01	3,95E-01	Mto1	1,11	No
ENSMUSG00000032343	1,87E+00	-3,107639691	1,38E+00	2,40E-01	5,50E-01	Impg1	3,67	No
ENSMUSG00000032344	1,40E-01	4,547358806	3,09E+00	7,86E-02	3,58E-01	Mb21d1	1,10	No
ENSMUSG00000032348	-1,25E+00	-2,548191348	1,51E+00	2,19E-01	5,50E-01	Gsta4	0,42	No
ENSMUSG00000032349	1,19E-01	7,722802873	4,13E+00	4,22E-02	2,44E-01	Elovl5	1,09	No
ENSMUSG00000032350	-4,55E-02	5,529001758	4,35E-01	5,09E-01	7,93E-01	Gclc	0,97	No
ENSMUSG00000032352	1,79E-01	3,02467085	2,60E+00	1,07E-01	4,00E-01	Lrrc1	1,13	No
ENSMUSG00000032353	1,19E-01	5,860457244	3,20E+00	7,36E-02	3,45E-01	Tmed3	1,09	No
ENSMUSG00000032355	-3,83E-02	-3,107651936	-7,91E-11	1,00E+00	1,00E+00	Mlip	0,97	No
ENSMUSG00000032356	7,51E-02	-1,165692585	2,50E-02	8,74E-01	1,00E+00	Rasgrf1	1,05	No
ENSMUSG00000032359	-1,06E-01	8,186743345	3,35E+00	6,71E-02	3,26E-01	Ctsh	0,93	No
ENSMUSG00000032363	-5,56E-01	2,039875231	1,43E+01	1,55E-04	3,50E-03	Adamts7	0,68	Yes
ENSMUSG00000032366	-1,21E-01	5,606926764	3,17E+00	7,51E-02	3,49E-01	Tpm1	0,92	No
ENSMUSG00000032369	-3,15E-01	4,481813325	1,52E+01	9,84E-05	2,39E-03	Plscr1	0,80	Yes
ENSMUSG00000032370	-5,32E-02	6,033486753	6,68E-01	4,14E-01	7,09E-01	Lactb	0,96	No
ENSMUSG00000032372	-1,96E+00	-2,896145386	1,50E+00	2,21E-01	5,50E-01	Plscr2	0,26	No
ENSMUSG00000032373	3,39E-01	-1,146130244	5,85E-01	4,44E-01	7,36E-01	Car12	1,26	No
ENSMUSG00000032374	-8,48E-01	0,66685642	1,49E+01	1,16E-04	2,75E-03	Plod2	0,56	Yes
ENSMUSG00000032375	1,37E-01	4,920151335	3,32E+00	6,84E-02	3,29E-01	Aph1b	1,10	No
ENSMUSG00000032376	-2,09E-01	5,675909012	9,64E+00	1,90E-03	2,69E-02	Usp3	0,86	Yes
ENSMUSG00000032377	2,91E-02	2,258498691	4,33E-02	8,35E-01	9,80E-01	Plscr4	1,02	No
ENSMUSG00000032380	2,08E-02	1,904415238	1,77E-02	8,94E-01	1,00E+00	Dapk2	1,01	No

ENSMUSG00000032381	4,42E-02	6,905574346	5,23E-01	4,69E-01	7,58E-01	Fam96a	1,03	No
ENSMUSG00000032382	1,83E-01	6,91955684	8,97E+00	2,75E-03	3,58E-02	Snx1	1,13	Yes
ENSMUSG00000032383	3,20E-02	7,268444756	2,86E-01	5,93E-01	8,42E-01	Ppib	1,02	No
ENSMUSG00000032384	-7,10E-02	5,852190875	1,15E+00	2,83E-01	5,79E-01	Csnk1g1	0,95	No
ENSMUSG00000032386	-2,10E-02	5,269544438	8,76E-02	7,67E-01	9,40E-01	Trip4	0,99	No
ENSMUSG00000032387	-1,23E+00	0,054287361	1,65E+01	4,87E-05	1,33E-03	Rbpms2	0,43	Yes
ENSMUSG00000032388	-6,90E-04	6,631394659	1,23E-04	9,91E-01	1,00E+00	Spg21	1,00	No
ENSMUSG00000032392	2,10E-02	2,28954699	2,39E-02	8,77E-01	1,00E+00	Parp16	1,01	No
ENSMUSG00000032393	9,30E-02	7,106431104	2,38E+00	1,23E-01	4,39E-01	Dpp8	1,07	No
ENSMUSG00000032396	3,02E-03	4,58072572	1,46E-03	9,69E-01	1,00E+00	Dis3l	1,00	No
ENSMUSG00000032397	-4,58E-03	4,148408707	2,86E-03	9,57E-01	1,00E+00	Tipin	1,00	No
ENSMUSG00000032398	-1,33E-01	4,05884735	2,34E+00	1,26E-01	4,44E-01	Snapc5	0,91	No
ENSMUSG00000032399	-1,37E-01	8,72067186	5,72E+00	1,67E-02	1,35E-01	Rpl4	0,91	No
ENSMUSG00000032400	1,42E-01	4,031172206	2,60E+00	1,07E-01	3,99E-01	Zwilch	1,10	No
ENSMUSG00000032401	4,14E-01	0,853595579	3,52E+00	6,05E-02	3,05E-01	Lctl	1,33	No
ENSMUSG00000032402	-3,47E-01	2,297756375	6,24E+00	1,25E-02	1,10E-01	Smad3	0,79	No
ENSMUSG00000032403	1,20E-02	1,8306403	5,58E-03	9,40E-01	1,00E+00	2300009A05Rik	1,01	No
ENSMUSG00000032405	3,06E-02	5,579831441	2,00E-01	6,55E-01	8,79E-01	Pias1	1,02	No
ENSMUSG00000032407	4,52E-02	6,286393763	5,02E-01	4,78E-01	7,66E-01	U2surp	1,03	No
ENSMUSG00000032409	9,34E-03	4,762193711	1,48E-02	9,03E-01	1,00E+00	Atr	1,01	No
ENSMUSG00000032410	3,86E-02	6,040787864	3,51E-01	5,53E-01	8,19E-01	Xrn1	1,03	No
ENSMUSG00000032411	8,35E-02	5,124072919	1,32E+00	2,50E-01	5,50E-01	Tfdp2	1,06	No
ENSMUSG00000032412	3,73E-02	7,567908787	3,99E-01	5,28E-01	8,04E-01	Atp1b3	1,03	No
ENSMUSG00000032413	-2,92E-01	4,723612649	1,46E+01	1,33E-04	3,09E-03	Rasa2	0,82	Yes
ENSMUSG00000032415	-4,28E-01	0,259519171	2,61E+00	1,07E-01	3,99E-01	Ube2cbp	0,74	No
ENSMUSG00000032417	-4,46E-03	1,745393996	7,60E-04	9,78E-01	1,00E+00	Rwdd2a	1,00	No
ENSMUSG00000032418	6,83E-02	1,117597316	1,13E-01	7,37E-01	9,25E-01	Me1	1,05	No
ENSMUSG00000032419	2,31E+00	-2,929290301	2,04E+00	1,54E-01	4,97E-01	Tbx18	4,97	No
ENSMUSG00000032420	-1,20E-01	2,346322025	7,77E-01	3,78E-01	6,79E-01	Nt5e	0,92	No
ENSMUSG00000032422	1,55E-02	4,770509724	4,07E-02	8,40E-01	9,83E-01	Snx14	1,01	No
ENSMUSG00000032423	7,28E-02	6,791729939	1,40E+00	2,37E-01	5,50E-01	Syncrip	1,05	No
ENSMUSG00000032425	-1,71E-01	3,252555256	2,71E+00	9,97E-02	3,92E-01	Zfp949	0,89	No

ENSMUSG00000032431	1,35E-01	5,355539471	3,66E+00	5,58E-02	2,90E-01	Crtap	1,10	No
ENSMUSG00000032434	-1,63E-01	8,957709718	8,15E+00	4,30E-03	4,99E-02	Cmtm6	0,89	Yes
ENSMUSG00000032435	-2,00E-01	6,445798165	1,01E+01	1,49E-03	2,23E-02	Dync1li1	0,87	Yes
ENSMUSG00000032436	-1,52E-02	6,453104809	5,84E-02	8,09E-01	9,65E-01	Cmtm7	0,99	No
ENSMUSG00000032437	1,17E-01	7,476136033	3,90E+00	4,82E-02	2,64E-01	Stt3b	1,08	No
ENSMUSG00000032440	1,44E-01	7,67537817	5,99E+00	1,44E-02	1,22E-01	Tgfbr2	1,11	No
ENSMUSG00000032443	-2,50E+00	-2,355487253	5,42E+00	1,99E-02	1,51E-01	Zcwpw2	0,18	No
ENSMUSG00000032446	-1,94E+00	-3,107619243	1,50E+00	2,21E-01	5,50E-01	Eomes	0,26	No
ENSMUSG00000032449	7,74E-02	6,014043531	1,41E+00	2,34E-01	5,50E-01	Slc25a36	1,06	No
ENSMUSG00000032452	4,24E-02	-3,033592245	-1,17E-09	1,00E+00	1,00E+00	Clstn2	1,03	No
ENSMUSG00000032454	3,14E-02	-3,107617333	-7,69E-10	1,00E+00	1,00E+00	Rbp2	1,02	No
ENSMUSG00000032456	-1,21E-01	3,687531086	1,66E+00	1,98E-01	5,50E-01	Nmnat3	0,92	No
ENSMUSG00000032458	5,18E-04	6,971125759	7,24E-05	9,93E-01	1,00E+00	Copb2	1,00	No
ENSMUSG00000032459	-4,28E-02	3,202722581	1,60E-01	6,89E-01	8,99E-01	Mrps22	0,97	No
ENSMUSG00000032462	3,65E-02	6,16532958	3,21E-01	5,71E-01	8,32E-01	Pik3cb	1,03	No
ENSMUSG00000032463	-4,15E-02	4,740185105	2,88E-01	5,91E-01	8,42E-01	Faim	0,97	No
ENSMUSG00000032468	6,70E-02	4,730594021	7,56E-01	3,85E-01	6,86E-01	Armc8	1,05	No
ENSMUSG00000032469	1,08E-02	4,59642621	1,88E-02	8,91E-01	1,00E+00	Dbr1	1,01	No
ENSMUSG00000032470	3,98E-01	6,002217571	3,64E+01	1,57E-09	1,25E-07	Mras	1,32	Yes
ENSMUSG00000032475	1,23E-01	5,318418037	3,07E+00	7,98E-02	3,61E-01	Nck1	1,09	No
ENSMUSG00000032477	2,13E-01	4,613720537	7,38E+00	6,60E-03	6,88E-02	Cdc25a	1,16	No
ENSMUSG00000032478	4,67E-02	2,699378354	1,46E-01	7,03E-01	9,06E-01	Nme6	1,03	No
ENSMUSG00000032479	-6,13E-02	8,251030684	1,13E+00	2,88E-01	5,84E-01	Map4	0,96	No
ENSMUSG00000032480	5,80E-02	5,553746629	7,16E-01	3,98E-01	6,98E-01	Dhx30	1,04	No
ENSMUSG00000032481	1,02E-01	5,949523041	2,37E+00	1,24E-01	4,40E-01	Smarcc1	1,07	No
ENSMUSG00000032482	3,93E-01	-2,10765596	2,47E-01	6,19E-01	8,56E-01	Cspg5	1,31	No
ENSMUSG00000032484	-3,79E-01	-0,236726687	1,52E+00	2,18E-01	5,50E-01	Ngp	0,77	No
ENSMUSG00000032485	9,00E-02	5,891984405	1,85E+00	1,74E-01	5,32E-01	Scap	1,06	No
ENSMUSG00000032487	-3,36E-01	3,083271953	9,17E+00	2,45E-03	3,29E-02	Ptgs2	0,79	Yes
ENSMUSG00000032489	1,29E-01	1,535995999	5,27E-01	4,68E-01	7,56E-01	Kif9	1,09	No
ENSMUSG00000032491	7,78E-02	1,868310104	2,58E-01	6,11E-01	8,52E-01	Nradd	1,06	No
ENSMUSG00000032492	-1,19E+00	-2,226164644	1,82E+00	1,77E-01	5,37E-01	Pth1r	0,44	No

ENSMUSG00000032493	9,01E-03	-3,107646452	-5,16E-10	1,00E+00	1,00E+00	Prss44	1,01	No
ENSMUSG00000032495	2,67E+00	-3,033636951	2,76E+00	9,68E-02	3,88E-01	Lrrc2	6,36	No
ENSMUSG00000032496	1,89E-01	0,167674713	5,25E-01	4,69E-01	7,57E-01	Ltf	1,14	No
ENSMUSG00000032497	7,71E-02	6,554347172	1,51E+00	2,18E-01	5,50E-01	Lrrfip2	1,05	No
ENSMUSG00000032498	1,09E-01	3,993663623	1,50E+00	2,21E-01	5,50E-01	Mlh1	1,08	No
ENSMUSG00000032500	3,73E-01	0,632975521	2,56E+00	1,09E-01	4,06E-01	Dclk3	1,30	No
ENSMUSG00000032501	6,24E-02	7,084732256	1,06E+00	3,03E-01	6,00E-01	Trib1	1,04	No
ENSMUSG00000032502	2,27E+00	-2,801010027	2,93E+00	8,69E-02	3,78E-01	Stac	4,82	No
ENSMUSG00000032503	-1,87E+00	-3,107637761	1,39E+00	2,38E-01	5,50E-01	Arpp21	0,27	No
ENSMUSG00000032504	9,03E-02	8,167983332	2,43E+00	1,19E-01	4,30E-01	Pdcd6ip	1,06	No
ENSMUSG00000032507	2,36E-01	4,062256238	7,13E+00	7,56E-03	7,59E-02	Fbxl2	1,18	No
ENSMUSG00000032508	6,27E-02	7,029132788	1,07E+00	3,01E-01	5,98E-01	Myd88	1,04	No
ENSMUSG00000032511	-1,98E+00	-2,655099849	2,97E+00	8,47E-02	3,73E-01	Scn5a	0,25	No
ENSMUSG00000032512	-1,30E-01	5,589535508	3,64E+00	5,62E-02	2,91E-01	Wdr48	0,91	No
ENSMUSG00000032513	3,28E-02	4,559615661	1,70E-01	6,80E-01	8,93E-01	Gorasp1	1,02	No
ENSMUSG00000032514	-8,39E-01	1,156223234	1,91E+01	1,25E-05	4,09E-04	Ttc21a	0,56	Yes
ENSMUSG00000032515	7,01E-02	6,290784326	1,21E+00	2,72E-01	5,68E-01	Csrnp1	1,05	No
ENSMUSG00000032518	-2,22E-01	8,250522308	1,47E+01	1,25E-04	2,92E-03	Rpsa	0,86	Yes
ENSMUSG00000032519	-1,47E-01	5,492768895	4,55E+00	3,30E-02	2,13E-01	Slc25a38	0,90	No
ENSMUSG00000032523	-8,21E-01	-2,963266074	3,67E-01	5,45E-01	8,13E-01	Hhatl	0,57	No
ENSMUSG00000032525	1,23E-02	7,954181254	4,45E-02	8,33E-01	9,78E-01	Nktr	1,01	No
ENSMUSG00000032526	2,72E-03	3,513726568	7,50E-04	9,78E-01	1,00E+00	Ss18l2	1,00	No
ENSMUSG00000032527	4,16E-02	4,273366687	2,42E-01	6,23E-01	8,58E-01	Pccb	1,03	No
ENSMUSG00000032528	8,02E-01	4,17228575	8,44E+01	4,13E-20	9,30E-18	Vipr1	1,74	Yes
ENSMUSG00000032530	1,46E-01	0,365606039	2,96E-01	5,87E-01	8,40E-01	Lyzl4	1,11	No
ENSMUSG00000032531	1,20E+00	-1,832046832	3,49E+00	6,18E-02	3,09E-01	Amotl2	2,29	No
ENSMUSG00000032532	-3,60E+00	-0,388991256	7,87E+01	7,08E-19	1,48E-16	Cck	0,08	Yes
ENSMUSG00000032534	4,00E-02	4,000671667	2,04E-01	6,52E-01	8,77E-01	Cep63	1,03	No
ENSMUSG00000032536	9,04E-03	6,814206994	2,16E-02	8,83E-01	1,00E+00	Trak1	1,01	No
ENSMUSG00000032540	1,35E-01	5,840330472	4,12E+00	4,24E-02	2,45E-01	Abhd5	1,10	No
ENSMUSG00000032547	4,13E-01	0,419211881	2,44E+00	1,18E-01	4,27E-01	Ryk	1,33	No
ENSMUSG00000032548	-3,72E-01	-0,155716019	1,22E+00	2,70E-01	5,67E-01	Slco2a1	0,77	No

ENSMUSG00000032549	-3,30E-01	2,041404974	4,95E+00	2,62E-02	1,81E-01	Rab6b	0,80	No
ENSMUSG00000032551	2,23E-02	4,156897385	6,65E-02	7,97E-01	9,58E-01	1110059G10Rik	1,02	No
ENSMUSG00000032553	1,66E-01	5,061716611	5,08E+00	2,42E-02	1,71E-01	Srprb	1,12	No
ENSMUSG00000032554	-1,67E-02	6,49664011	7,03E-02	7,91E-01	9,55E-01	Trf	0,99	No
ENSMUSG00000032555	9,00E-02	7,127958765	2,22E+00	1,36E-01	4,65E-01	Topbp1	1,06	No
ENSMUSG00000032556	2,61E-02	-1,136362713	3,37E-03	9,54E-01	1,00E+00	Bfsp2	1,02	No
ENSMUSG00000032557	3,67E-02	5,366959865	2,72E-01	6,02E-01	8,48E-01	Uba5	1,03	No
ENSMUSG00000032558	2,13E-01	3,14383909	3,82E+00	5,07E-02	2,73E-01	Nphp3	1,16	No
ENSMUSG00000032560	4,80E-02	7,002085902	6,24E-01	4,30E-01	7,23E-01	Dnajc13	1,03	No
ENSMUSG00000032561	-3,29E-01	6,786633034	2,85E+01	9,61E-08	5,40E-06	Acpp	0,80	Yes
ENSMUSG00000032562	-3,18E-03	9,98772272	3,21E-03	9,55E-01	1,00E+00	Gnai2	1,00	No
ENSMUSG00000032563	-3,21E-02	4,975854971	1,86E-01	6,67E-01	8,86E-01	Mrpl3	0,98	No
ENSMUSG00000032565	-4,34E-01	3,654965746	2,05E+01	5,87E-06	2,08E-04	Nudt16	0,74	Yes
ENSMUSG00000032567	2,01E-01	3,39755669	3,82E+00	5,07E-02	2,73E-01	Aste1	1,15	No
ENSMUSG00000032570	1,10E-01	6,320727699	2,97E+00	8,47E-02	3,73E-01	Atp2c1	1,08	No
ENSMUSG00000032571	1,40E-01	5,404519086	4,00E+00	4,56E-02	2,54E-01	Pik3r4	1,10	No
ENSMUSG00000032572	-8,37E-01	-2,770504191	3,85E-01	5,35E-01	8,07E-01	Col6a4	0,56	No
ENSMUSG00000032575	9,78E-03	6,636300006	2,47E-02	8,75E-01	1,00E+00	Manf	1,01	No
ENSMUSG00000032577	3,52E-03	7,511132628	3,53E-03	9,53E-01	1,00E+00	Mapkapk3	1,00	No
ENSMUSG00000032578	7,70E-02	8,234148525	1,78E+00	1,82E-01	5,45E-01	Cish	1,05	No
ENSMUSG00000032579	2,29E-01	5,836781136	1,19E+01	5,57E-04	1,00E-02	Hemk1	1,17	Yes
ENSMUSG00000032580	5,30E-02	6,559294835	7,25E-01	3,94E-01	6,95E-01	Rbm5	1,04	No
ENSMUSG00000032582	-8,75E-02	5,376093146	1,57E+00	2,10E-01	5,50E-01	Rbm6	0,94	No
ENSMUSG00000032583	-2,60E-02	3,961816211	8,49E-02	7,71E-01	9,42E-01	Mon1a	0,98	No
ENSMUSG00000032584	-5,96E-01	-2,401337343	4,83E-01	4,87E-01	7,74E-01	Mst1r	0,66	No
ENSMUSG00000032586	1,70E-01	3,227813751	2,48E+00	1,16E-01	4,21E-01	Traip	1,13	No
ENSMUSG00000032589	3,79E-01	3,676820608	1,59E+01	6,84E-05	1,76E-03	Bsn	1,30	Yes
ENSMUSG00000032590	5,46E-02	5,470184441	6,23E-01	4,30E-01	7,23E-01	Apeh	1,04	No
ENSMUSG00000032591	1,89E-01	0,801680445	7,03E-01	4,02E-01	7,00E-01	Mst1	1,14	No
ENSMUSG00000032593	3,93E-02	4,036090166	2,01E-01	6,54E-01	8,79E-01	Amigo3	1,03	No
ENSMUSG00000032594	-8,47E-02	6,771667829	1,90E+00	1,68E-01	5,22E-01	Ip6k1	0,94	No
ENSMUSG00000032595	2,29E-02	-1,998057404	1,00E-03	9,75E-01	1,00E+00	Cdhr4	1,02	No

ENSMUSG00000032596	-2,97E-01	6,353303433	2,20E+01	2,69E-06	1,05E-04	Uba7	0,81	Yes
ENSMUSG00000032598	1,77E-02	5,614339081	6,80E-02	7,94E-01	9,56E-01	Nckipsd	1,01	No
ENSMUSG00000032599	-2,21E-01	4,576702908	7,80E+00	5,24E-03	5,78E-02	Ip6k2	0,86	No
ENSMUSG00000032601	9,23E-02	7,116277025	2,34E+00	1,26E-01	4,45E-01	Prkar2a	1,07	No
ENSMUSG00000032602	6,02E-02	4,449338545	5,42E-01	4,62E-01	7,50E-01	Slc25a20	1,04	No
ENSMUSG00000032604	-3,02E-02	6,296206733	2,25E-01	6,35E-01	8,67E-01	Qars	0,98	No
ENSMUSG00000032606	-1,74E-01	3,161833029	2,54E+00	1,11E-01	4,09E-01	Nicn1	0,89	No
ENSMUSG00000032607	7,58E-02	0,160004964	7,87E-02	7,79E-01	9,47E-01	Amt	1,05	No
ENSMUSG00000032609	-4,61E-01	2,557902543	1,36E+01	2,23E-04	4,75E-03	Klhdc8b	0,73	Yes
ENSMUSG00000032611	3,51E-01	-1,205916851	5,99E-01	4,39E-01	7,32E-01	1700102P08Rik	1,28	No
ENSMUSG00000032612	-3,43E-02	7,056905424	3,22E-01	5,70E-01	8,31E-01	Usp4	0,98	No
ENSMUSG00000032615	1,42E-01	4,034923709	2,57E+00	1,09E-01	4,05E-01	Nt5m	1,10	No
ENSMUSG00000032621	1,23E-01	5,915377012	3,45E+00	6,33E-02	3,14E-01	Srek1	1,09	No
ENSMUSG00000032623	-4,08E-02	-2,963255775	7,59E-04	9,78E-01	1,00E+00	Oas1d	0,97	No
ENSMUSG00000032624	-2,27E-02	5,882384461	1,18E-01	7,32E-01	9,22E-01	Eml4	0,98	No
ENSMUSG00000032625	-1,87E+00	-3,107637761	1,39E+00	2,38E-01	5,50E-01	Thsd7a	0,27	No
ENSMUSG00000032633	1,31E-01	6,001637069	4,02E+00	4,49E-02	2,53E-01	Flcn	1,10	No
ENSMUSG00000032637	-3,30E-02	7,818444877	3,19E-01	5,72E-01	8,32E-01	Atxn2l	0,98	No
ENSMUSG00000032640	1,73E-01	5,977585328	6,85E+00	8,86E-03	8,54E-02	Chsy1	1,13	No
ENSMUSG00000032641	3,92E-01	1,775728933	5,85E+00	1,56E-02	1,29E-01	Gpr19	1,31	No
ENSMUSG00000032643	5,75E-02	4,614134108	5,37E-01	4,64E-01	7,52E-01	Fhl3	1,04	No
ENSMUSG00000032648	-9,35E-02	0,868744305	1,65E-01	6,85E-01	8,97E-01	Pygm	0,94	No
ENSMUSG00000032649	1,86E+00	-1,497761848	8,83E+00	2,96E-03	3,80E-02	Colgalt2	3,62	Yes
ENSMUSG00000032652	-3,97E-01	5,097123153	3,06E+01	3,21E-08	1,99E-06	Crebl2	0,76	Yes
ENSMUSG00000032656	3,96E-01	4,425451039	2,29E+01	1,71E-06	7,13E-05	March3	1,32	Yes
ENSMUSG00000032657	9,67E-02	2,987043449	7,05E-01	4,01E-01	6,99E-01	Fam189b	1,07	No
ENSMUSG00000032661	-7,27E-02	4,725375912	9,03E-01	3,42E-01	6,42E-01	Oas3	0,95	No
ENSMUSG00000032666	-2,68E-02	5,780318938	1,61E-01	6,88E-01	8,98E-01	1700025G04Rik	0,98	No
ENSMUSG00000032667	-1,08E-01	6,840429936	3,11E+00	7,80E-02	3,57E-01	Pon2	0,93	No
ENSMUSG00000032673	-9,21E-02	4,333754324	1,24E+00	2,66E-01	5,62E-01	Prorsd1	0,94	No
ENSMUSG00000032679	-9,30E-02	1,051860122	2,08E-01	6,48E-01	8,75E-01	Cd59a	0,94	No
ENSMUSG00000032680	-5,88E-02	-3,033616507	1,62E-03	9,68E-01	1,00E+00	6820408C15Rik	0,96	No

ENSMUSG00000032688	-3,19E-01	9,287833273	3,14E+01	2,15E-08	1,39E-06	Malt1	0,80	Yes
ENSMUSG00000032690	-1,35E-01	2,393687095	1,10E+00	2,94E-01	5,89E-01	Oas2	0,91	No
ENSMUSG00000032691	1,24E-01	8,306714608	4,57E+00	3,26E-02	2,11E-01	Nlrp3	1,09	No
ENSMUSG00000032698	-1,69E-01	5,737122617	6,32E+00	1,19E-02	1,06E-01	Lmo2	0,89	No
ENSMUSG00000032702	3,35E-01	-1,4729841	3,89E-01	5,33E-01	8,06E-01	Kank1	1,26	No
ENSMUSG00000032705	7,07E-02	4,187859009	6,93E-01	4,05E-01	7,02E-01	Exd2	1,05	No
ENSMUSG00000032712	-2,06E-01	7,338737998	1,20E+01	5,43E-04	9,82E-03	2810474O19Rik	0,87	Yes
ENSMUSG00000032714	4,75E-01	-1,879886473	5,01E-01	4,79E-01	7,66E-01	Syde1	1,39	No
ENSMUSG00000032715	3,94E-01	2,930149849	1,19E+01	5,51E-04	9,93E-03	Trib3	1,31	Yes
ENSMUSG00000032717	1,02E+00	-1,963315913	2,04E+00	1,53E-01	4,96E-01	Mdfi	2,03	No
ENSMUSG00000032718	4,52E-03	2,99790944	1,65E-03	9,68E-01	1,00E+00	Mansc1	1,00	No
ENSMUSG00000032719	1,11E-02	-2,165753032	2,31E-04	9,88E-01	1,00E+00	Sbspon	1,01	No
ENSMUSG00000032724	-5,66E-01	4,653303923	5,33E+01	2,91E-13	3,83E-11	Abtb2	0,68	Yes
ENSMUSG00000032725	-1,91E-01	-1,42491056	1,51E-01	6,98E-01	9,04E-01	Folr2	0,88	No
ENSMUSG00000032726	7,74E-01	-0,816428304	3,15E+00	7,61E-02	3,52E-01	Bmp8a	1,71	No
ENSMUSG00000032727	-9,72E-02	3,917880693	1,20E+00	2,74E-01	5,71E-01	Mier3	0,93	No
ENSMUSG00000032733	7,43E-02	3,368066942	5,40E-01	4,63E-01	7,51E-01	Snx33	1,05	No
ENSMUSG00000032735	-2,68E+00	-3,033645766	2,84E+00	9,17E-02	3,82E-01	Ablim3	0,16	No
ENSMUSG00000032737	-1,17E-01	5,977748846	3,18E+00	7,46E-02	3,47E-01	Inppl1	0,92	No
ENSMUSG00000032739	-4,03E-01	3,844870953	2,00E+01	7,93E-06	2,73E-04	Pram1	0,76	Yes
ENSMUSG00000032740	-1,22E-01	7,566045622	4,25E+00	3,92E-02	2,35E-01	Ccdc88a	0,92	No
ENSMUSG00000032741	-1,47E-01	5,294821817	4,31E+00	3,78E-02	2,31E-01	Tpcn1	0,90	No
ENSMUSG00000032743	1,47E-01	4,59108576	3,43E+00	6,40E-02	3,16E-01	D430042O09Rik	1,11	No
ENSMUSG00000032744	-1,95E+00	-3,033628749	1,50E+00	2,21E-01	5,50E-01	Heyl	0,26	No
ENSMUSG00000032745	-2,12E-01	6,784953143	1,20E+01	5,42E-04	9,81E-03	Gpbp1	0,86	Yes
ENSMUSG00000032754	1,20E-01	5,725273023	3,19E+00	7,43E-02	3,47E-01	Slc8b1	1,09	No
ENSMUSG00000032757	1,28E-01	4,43478907	2,46E+00	1,17E-01	4,24E-01	Bet1	1,09	No
ENSMUSG00000032763	-1,01E-01	4,774003892	1,71E+00	1,91E-01	5,50E-01	Ilvbl	0,93	No
ENSMUSG00000032766	3,48E-01	2,163437789	5,84E+00	1,57E-02	1,30E-01	Gng11	1,27	No
ENSMUSG00000032769	-1,87E+00	-3,107637761	1,39E+00	2,38E-01	5,50E-01	Trpa1	0,27	No
ENSMUSG00000032773	-1,91E+00	-2,998001842	1,45E+00	2,28E-01	5,50E-01	Chrm1	0,27	No
ENSMUSG00000032776	-3,29E-01	-1,165695685	4,67E-01	4,94E-01	7,80E-01	Mctp2	0,80	No

ENSMUSG00000032777	5,03E-02	6,447046767	6,40E-01	4,24E-01	7,18E-01	Gtf3c1	1,04	No
ENSMUSG00000032782	1,18E-01	3,653641566	1,54E+00	2,14E-01	5,50E-01	Cntrob	1,09	No
ENSMUSG00000032783	3,75E-01	3,33096729	1,33E+01	2,66E-04	5,50E-03	Troap	1,30	Yes
ENSMUSG00000032786	2,54E-03	8,76816263	1,98E-03	9,65E-01	1,00E+00	Alas1	1,00	No
ENSMUSG00000032788	3,14E-01	6,185149232	2,37E+01	1,15E-06	5,03E-05	Pdxk	1,24	Yes
ENSMUSG00000032796	-6,48E-02	-2,770611747	3,82E-03	9,51E-01	1,00E+00	Lama1	0,96	No
ENSMUSG00000032802	2,71E-03	7,121785545	2,01E-03	9,64E-01	1,00E+00	Srxn1	1,00	No
ENSMUSG00000032803	6,81E-02	7,519984109	1,33E+00	2,49E-01	5,50E-01	Cdv3	1,05	No
ENSMUSG00000032812	9,24E-02	7,486376052	2,43E+00	1,19E-01	4,30E-01	Arap1	1,07	No
ENSMUSG00000032815	1,47E-01	5,070142311	4,02E+00	4,50E-02	2,53E-01	Fanca	1,11	No
ENSMUSG00000032816	-3,98E-02	-2,497586676	1,48E-03	9,69E-01	1,00E+00	Igdcc4	0,97	No
ENSMUSG00000032818	-1,34E+00	-2,896122948	1,12E+00	2,89E-01	5,85E-01	Loxhd1	0,39	No
ENSMUSG00000032826	-3,86E-01	1,014890205	3,41E+00	6,49E-02	3,19E-01	Ank2	0,77	No
ENSMUSG00000032827	-2,33E-01	1,493233892	1,73E+00	1,88E-01	5,50E-01	Ppp1r9a	0,85	No
ENSMUSG00000032834	8,28E-02	4,386331186	1,02E+00	3,14E-01	6,14E-01	Pwp2	1,06	No
ENSMUSG00000032839	-7,36E-01	-2,548221181	6,07E-01	4,36E-01	7,29E-01	Trpc1	0,60	No
ENSMUSG00000032840	1,28E-01	4,319705773	2,37E+00	1,23E-01	4,39E-01	2410131K14Rik	1,09	No
ENSMUSG00000032841	3,82E-02	5,413268075	3,07E-01	5,80E-01	8,36E-01	Prr5l	1,03	No
ENSMUSG00000032842	2,79E-02	3,688996114	8,73E-02	7,68E-01	9,41E-01	Abcc10	1,02	No
ENSMUSG00000032845	1,75E-01	3,854476912	3,63E+00	5,66E-02	2,93E-01	Alpk2	1,13	No
ENSMUSG00000032846	1,86E-01	5,111200345	6,32E+00	1,19E-02	1,06E-01	Zswim6	1,14	No
ENSMUSG00000032849	2,08E-01	5,292873543	8,37E+00	3,81E-03	4,56E-02	Abcc4	1,15	Yes
ENSMUSG00000032850	-2,67E-01	0,477137291	1,24E+00	2,65E-01	5,60E-01	Rnft2	0,83	No
ENSMUSG00000032852	1,87E+00	-3,107639691	1,38E+00	2,40E-01	5,50E-01	Rspo4	3,67	No
ENSMUSG00000032855	9,16E-02	6,834475249	2,24E+00	1,35E-01	4,62E-01	Pkd1	1,07	No
ENSMUSG00000032860	2,16E-01	3,501014425	4,72E+00	2,98E-02	1,97E-01	P2ry2	1,16	No
ENSMUSG00000032864	6,32E-01	-3,07016611	1,62E-01	6,88E-01	8,98E-01	Rag2	1,55	No
ENSMUSG00000032867	-2,60E-02	6,037433263	1,58E-01	6,91E-01	9,00E-01	Fbxw8	0,98	No
ENSMUSG00000032869	-1,87E-02	4,975542474	6,39E-02	8,00E-01	9,61E-01	Psmf1	0,99	No
ENSMUSG00000032870	-2,14E-01	7,437062472	1,30E+01	3,20E-04	6,39E-03	Smap2	0,86	Yes
ENSMUSG00000032872	5,20E-02	5,261011704	5,32E-01	4,66E-01	7,54E-01	Cyb5r4	1,04	No
ENSMUSG00000032875	-4,34E-01	1,128905977	4,53E+00	3,32E-02	2,14E-01	Arhgef17	0,74	No

ENSMUSG00000032878	-4,30E-02	3,890667281	2,19E-01	6,40E-01	8,70E-01	Ccdc85a	0,97	No
ENSMUSG00000032883	6,50E-01	4,781333821	7,03E+01	4,98E-17	9,22E-15	Acsl3	1,57	Yes
ENSMUSG00000032889	-1,17E-01	1,881573014	5,90E-01	4,43E-01	7,34E-01	Gm6685	0,92	No
ENSMUSG00000032890	-7,87E-02	1,024016903	1,52E-01	6,97E-01	9,03E-01	Rims3	0,95	No
ENSMUSG00000032892	-2,33E-01	-0,093409588	6,01E-01	4,38E-01	7,31E-01	Rangrf	0,85	No
ENSMUSG00000032897	1,78E-01	4,217022595	4,41E+00	3,58E-02	2,24E-01	Nfyc	1,13	No
ENSMUSG00000032898	-1,53E-01	4,525628174	3,69E+00	5,48E-02	2,87E-01	Fbxo21	0,90	No
ENSMUSG00000032899	1,13E+00	-2,070312207	2,61E+00	1,06E-01	3,99E-01	Styk1	2,19	No
ENSMUSG00000032902	3,37E-01	4,92235451	2,01E+01	7,45E-06	2,58E-04	Slc16a1	1,26	Yes
ENSMUSG00000032905	-1,26E-01	5,727074034	3,53E+00	6,02E-02	3,04E-01	Atg12	0,92	No
ENSMUSG00000032908	3,06E-01	-1,522664898	3,46E-01	5,56E-01	8,21E-01	Sgpp2	1,24	No
ENSMUSG00000032911	5,18E-01	-0,4727403	2,27E+00	1,32E-01	4,56E-01	Cspg4	1,43	No
ENSMUSG00000032913	1,07E-01	4,237941382	1,62E+00	2,03E-01	5,50E-01	Lrig2	1,08	No
ENSMUSG00000032915	-8,26E-01	2,915676151	4,99E+01	1,65E-12	1,96E-10	Adgre4	0,56	Yes
ENSMUSG00000032932	5,24E-02	5,250018178	5,37E-01	4,64E-01	7,52E-01	Hspa13	1,04	No
ENSMUSG00000032937	1,81E+00	-3,10765737	1,28E+00	2,58E-01	5,50E-01	Fshr	3,49	No
ENSMUSG00000032939	-9,41E-03	5,859657797	2,01E-02	8,87E-01	1,00E+00	Nup93	0,99	No
ENSMUSG00000032940	-1,78E+00	-0,547886572	2,36E+01	1,20E-06	5,22E-05	Rbm11	0,29	Yes
ENSMUSG00000032942	-3,79E-01	-0,980463794	6,34E-01	4,26E-01	7,20E-01	Ucp3	0,77	No
ENSMUSG00000032946	-1,00E-01	1,539292373	3,20E-01	5,72E-01	8,32E-01	Rasgrp2	0,93	No
ENSMUSG00000032952	3,94E-02	4,115038693	2,05E-01	6,51E-01	8,76E-01	Ap4b1	1,03	No
ENSMUSG00000032959	7,02E-02	6,409592206	1,24E+00	2,66E-01	5,62E-01	Pebp1	1,05	No
ENSMUSG00000032965	-2,66E-01	3,728166844	8,11E+00	4,40E-03	5,07E-02	Ift57	0,83	No
ENSMUSG00000032966	-4,33E-04	7,796808678	5,48E-05	9,94E-01	1,00E+00	Fkbp1a	1,00	No
ENSMUSG00000032968	-1,94E+00	-3,107619243	1,50E+00	2,21E-01	5,50E-01	Inha	0,26	No
ENSMUSG00000032977	8,67E-02	4,419757919	1,15E+00	2,84E-01	5,80E-01	Fam207a	1,06	No
ENSMUSG00000032978	9,01E-03	-3,107646452	-5,16E-10	1,00E+00	1,00E+00	Guca2b	1,01	No
ENSMUSG00000032985	7,26E-01	-2,896176066	2,87E-01	5,92E-01	8,42E-01	5730522E02Rik	1,65	No
ENSMUSG00000032988	-2,07E-01	-0,980459885	2,10E-01	6,47E-01	8,74E-01	Slc16a8	0,87	No
ENSMUSG00000032997	1,63E-01	4,712282749	4,37E+00	3,66E-02	2,27E-01	Chpf	1,12	No
ENSMUSG00000032998	-8,61E-02	5,545110272	1,56E+00	2,12E-01	5,50E-01	Foxj3	0,94	No
ENSMUSG00000032999	-9,41E-01	-2,497567965	7,76E-01	3,78E-01	6,79E-01	Nlrp4f	0,52	No

ENSMUSG00000033004	8,21E-02	8,535881361	2,05E+00	1,53E-01	4,95E-01	Mycbp2	1,06	No
ENSMUSG00000033007	-2,20E-02	-3,107641896	1,16E-04	9,91E-01	1,00E+00	Asic4	0,98	No
ENSMUSG00000033009	1,78E-01	4,750468929	5,30E+00	2,13E-02	1,57E-01	Ogfod1	1,13	No
ENSMUSG00000033014	-6,20E-02	5,81604569	8,74E-01	3,50E-01	6,51E-01	Trim33	0,96	No
ENSMUSG00000033015	-1,87E+00	-3,107637761	1,39E+00	2,38E-01	5,50E-01	4933427E13Rik	0,27	No
ENSMUSG00000033016	4,57E-02	6,485399961	5,28E-01	4,67E-01	7,56E-01	Nfatc1	1,03	No
ENSMUSG00000033020	1,10E-01	3,815271885	1,39E+00	2,38E-01	5,50E-01	Polr2f	1,08	No
ENSMUSG00000033021	9,00E-02	5,458915568	1,68E+00	1,95E-01	5,50E-01	Gmppa	1,06	No
ENSMUSG00000033022	-1,16E-01	-0,561250615	1,08E-01	7,42E-01	9,27E-01	Cdo1	0,92	No
ENSMUSG00000033024	6,88E-01	-1,86378749	1,12E+00	2,90E-01	5,86E-01	Klra9	1,61	No
ENSMUSG00000033029	-7,04E-04	4,412497822	7,49E-05	9,93E-01	1,00E+00	1700088E04Rik	1,00	No
ENSMUSG00000033031	1,28E-01	5,396046761	3,34E+00	6,77E-02	3,28E-01	C330027C09Rik	1,09	No
ENSMUSG00000033032	3,19E-01	0,045082261	1,10E+00	2,94E-01	5,89E-01	Afap1l1	1,25	No
ENSMUSG00000033033	4,49E-01	3,513657549	2,15E+01	3,58E-06	1,36E-04	Calhm2	1,36	Yes
ENSMUSG00000033036	1,06E-02	2,622446999	7,22E-03	9,32E-01	1,00E+00	Gm7879	1,01	No
ENSMUSG00000033039	4,51E-02	5,956597262	4,74E-01	4,91E-01	7,77E-01	Micall1	1,03	No
ENSMUSG00000033044	1,09E+00	-2,655198148	1,14E+00	2,86E-01	5,82E-01	Dhrs7c	2,14	No
ENSMUSG00000033047	1,51E-02	7,0185558	6,24E-02	8,03E-01	9,62E-01	Eif3l	1,01	No
ENSMUSG00000033054	5,23E-02	4,723572529	4,55E-01	5,00E-01	7,85E-01	Npat	1,04	No
ENSMUSG00000033055	6,14E-02	4,190708236	5,23E-01	4,69E-01	7,58E-01	Ankrd54	1,04	No
ENSMUSG00000033059	-4,04E-02	5,811866995	3,66E-01	5,45E-01	8,13E-01	Pygb	0,97	No
ENSMUSG00000033060	-7,18E-01	-2,497524352	5,77E-01	4,47E-01	7,38E-01	Lmo7	0,61	No
ENSMUSG00000033065	1,62E-01	2,766436787	1,76E+00	1,85E-01	5,49E-01	Pfkm	1,12	No
ENSMUSG00000033066	4,57E-02	7,235626694	5,78E-01	4,47E-01	7,38E-01	Gas7	1,03	No
ENSMUSG00000033068	1,71E-01	3,696953611	3,33E+00	6,82E-02	3,29E-01	Entpd6	1,13	No
ENSMUSG00000033075	-1,81E-02	5,549172598	6,97E-02	7,92E-01	9,55E-01	Senp1	0,99	No
ENSMUSG00000033082	3,20E-01	2,065109866	4,56E+00	3,27E-02	2,11E-01	Clec1a	1,25	No
ENSMUSG00000033083	-6,08E-01	6,57355807	9,54E+01	1,54E-22	4,62E-20	Tbc1d4	0,66	Yes
ENSMUSG00000033088	-3,07E-01	6,189094814	2,31E+01	1,57E-06	6,62E-05	Triobp	0,81	Yes
ENSMUSG00000033096	-5,79E-02	5,0659006	6,17E-01	4,32E-01	7,26E-01	Apmap	0,96	No
ENSMUSG00000033099	1,26E-02	4,605594293	2,59E-02	8,72E-01	1,00E+00	Nol12	1,01	No
ENSMUSG00000033102	-1,46E-01	4,630696756	3,52E+00	6,07E-02	3,05E-01	Cdc14b	0,90	No

ENSMUSG00000033105	3,59E-01	5,423352291	2,66E+01	2,53E-07	1,30E-05	Lss	1,28	Yes
ENSMUSG00000033106	-7,43E-02	5,02316007	1,02E+00	3,12E-01	6,12E-01	Slc7a6os	0,95	No
ENSMUSG00000033107	-8,37E-01	-0,785615423	3,81E+00	5,09E-02	2,74E-01	Rnf125	0,56	No
ENSMUSG00000033111	-9,42E-02	4,000891597	1,15E+00	2,84E-01	5,80E-01	3830406C13Rik	0,94	No
ENSMUSG00000033114	-7,98E-02	3,284373117	5,86E-01	4,44E-01	7,35E-01	Slc35d2	0,95	No
ENSMUSG00000033124	1,35E-01	5,563863896	3,86E+00	4,95E-02	2,69E-01	Atg9a	1,10	No
ENSMUSG00000033126	2,47E-01	1,986355724	2,79E+00	9,49E-02	3,86E-01	Ybey	1,19	No
ENSMUSG00000033128	5,31E-02	6,589448399	7,26E-01	3,94E-01	6,95E-01	Gga1	1,04	No
ENSMUSG00000033147	-1,90E-01	4,535907257	5,77E+00	1,63E-02	1,33E-01	Slc22a15	0,88	No
ENSMUSG00000033149	-2,43E+00	-2,146044648	8,30E+00	3,97E-03	4,71E-02	Phldb2	0,19	Yes
ENSMUSG00000033152	5,15E-01	-1,236652064	9,41E-01	3,32E-01	6,32E-01	Podxl2	1,43	No
ENSMUSG00000033156	3,18E+00	-2,963245465	4,13E+00	4,21E-02	2,44E-01	Cst10	9,06	No
ENSMUSG00000033157	3,75E-02	3,328770073	1,32E-01	7,17E-01	9,13E-01	Abhd10	1,03	No
ENSMUSG00000033159	-8,48E-03	6,781526766	1,91E-02	8,90E-01	1,00E+00	Cnppd1	0,99	No
ENSMUSG00000033161	7,64E-02	8,842697	1,79E+00	1,81E-01	5,42E-01	Atp1a1	1,05	No
ENSMUSG00000033166	1,30E-01	4,113475014	2,21E+00	1,37E-01	4,66E-01	Dis3	1,09	No
ENSMUSG00000033170	1,04E+00	0,412453332	1,42E+01	1,63E-04	3,66E-03	Card10	2,06	Yes
ENSMUSG00000033174	1,57E-01	7,995511665	7,28E+00	6,97E-03	7,15E-02	Mgll	1,12	No
ENSMUSG00000033182	3,81E-01	-0,683235071	9,01E-01	3,42E-01	6,43E-01	Kbtbd12	1,30	No
ENSMUSG00000033184	9,81E-02	7,477382488	2,74E+00	9,81E-02	3,91E-01	Tmed7	1,07	No
ENSMUSG00000033186	1,65E-02	5,456180347	5,60E-02	8,13E-01	9,67E-01	Mzt1	1,01	No
ENSMUSG00000033187	7,81E-01	-2,896104841	3,33E-01	5,64E-01	8,26E-01	BC016579	1,72	No
ENSMUSG00000033191	-1,19E+00	-0,912536611	7,70E+00	5,52E-03	6,01E-02	Tie1	0,44	No
ENSMUSG00000033192	5,43E-02	5,838509699	6,62E-01	4,16E-01	7,11E-01	Lpcat2	1,04	No
ENSMUSG00000033196	1,86E+00	-3,033661846	1,35E+00	2,46E-01	5,50E-01	Myh2	3,62	No
ENSMUSG00000033207	3,41E-01	5,479858507	2,41E+01	9,32E-07	4,17E-05	Mamdc2	1,27	Yes
ENSMUSG00000033208	1,31E+00	-0,980853698	8,24E+00	4,09E-03	4,81E-02	S100b	2,48	Yes
ENSMUSG00000033209	-1,22E-01	1,681253877	4,91E-01	4,83E-01	7,71E-01	Ttc28	0,92	No
ENSMUSG00000033210	1,46E+00	-2,800989455	1,40E+00	2,36E-01	5,50E-01	Slc9c1	2,76	No
ENSMUSG00000033213	9,09E-02	6,909946764	2,21E+00	1,38E-01	4,68E-01	AA467197	1,07	No
ENSMUSG00000033214	1,82E+00	-2,963269943	1,32E+00	2,51E-01	5,50E-01	Slitrk5	3,54	No
ENSMUSG00000033216	7,37E-02	3,936778109	6,67E-01	4,14E-01	7,09E-01	Eefsec	1,05	No

ENSMUSG00000033220	-5,29E-02	8,393698868	8,42E-01	3,59E-01	6,60E-01	Rac2	0,96	No
ENSMUSG00000033222	2,36E-01	5,246957858	1,10E+01	9,09E-04	1,50E-02	Ttf2	1,18	Yes
ENSMUSG00000033227	2,64E-01	2,823939889	4,86E+00	2,74E-02	1,87E-01	Wnt6	1,20	No
ENSMUSG00000033228	-8,56E-03	7,152054092	2,02E-02	8,87E-01	1,00E+00	Scaf11	0,99	No
ENSMUSG00000033233	2,65E-01	1,057856844	1,75E+00	1,85E-01	5,49E-01	Trim45	1,20	No
ENSMUSG00000033237	-3,18E-02	6,364041267	2,50E-01	6,17E-01	8,55E-01	Arid2	0,98	No
ENSMUSG00000033249	-1,95E-01	1,849239483	1,56E+00	2,12E-01	5,50E-01	Hsf4	0,87	No
ENSMUSG00000033253	7,40E-02	5,678594419	1,20E+00	2,73E-01	5,69E-01	Szt2	1,05	No
ENSMUSG00000033255	2,16E-02	-1,472839343	1,63E-03	9,68E-01	1,00E+00	Gm5134	1,02	No
ENSMUSG00000033256	4,82E-01	-0,711706948	1,30E+00	2,54E-01	5,50E-01	Shf	1,40	No
ENSMUSG00000033257	1,11E-01	5,549691545	2,63E+00	1,05E-01	3,97E-01	Ttll4	1,08	No
ENSMUSG00000033268	-7,38E-02	-3,033634644	2,66E-03	9,59E-01	1,00E+00	Duox1	0,95	No
ENSMUSG00000033272	3,17E-02	6,640048762	2,60E-01	6,10E-01	8,52E-01	Slc35a4	1,02	No
ENSMUSG00000033276	2,92E-01	0,001817631	9,53E-01	3,29E-01	6,30E-01	Stk36	1,22	No
ENSMUSG00000033278	-5,01E-01	-1,800954426	6,07E-01	4,36E-01	7,29E-01	Ptprm	0,71	No
ENSMUSG00000033282	2,75E-01	2,865439848	5,52E+00	1,88E-02	1,45E-01	Rpgrip1l	1,21	No
ENSMUSG00000033285	1,10E-01	5,367384538	2,44E+00	1,18E-01	4,27E-01	Wdr3	1,08	No
ENSMUSG00000033287	1,34E-01	5,628929365	3,83E+00	5,03E-02	2,72E-01	Kctd17	1,10	No
ENSMUSG00000033294	1,19E-01	4,849383899	2,44E+00	1,18E-01	4,28E-01	Noc4l	1,09	No
ENSMUSG00000033295	-6,22E-01	2,026161849	1,74E+01	3,06E-05	8,93E-04	Ptprf	0,65	Yes
ENSMUSG00000033306	-8,74E-02	7,012944671	2,08E+00	1,50E-01	4,91E-01	Lpp	0,94	No
ENSMUSG00000033307	1,81E-01	5,121122862	6,13E+00	1,33E-02	1,15E-01	Mif	1,13	No
ENSMUSG00000033313	2,16E-01	3,105668873	3,99E+00	4,57E-02	2,55E-01	Fbxl8	1,16	No
ENSMUSG00000033316	2,30E-01	5,102456405	9,96E+00	1,60E-03	2,35E-02	Galnt9	1,17	Yes
ENSMUSG00000033318	-1,48E-01	3,175142496	1,89E+00	1,69E-01	5,25E-01	Gstt2	0,90	No
ENSMUSG00000033319	5,78E-02	6,582723171	8,61E-01	3,54E-01	6,55E-01	Fem1c	1,04	No
ENSMUSG00000033323	1,86E-01	5,318865463	6,90E+00	8,60E-03	8,36E-02	Ctdp1	1,14	No
ENSMUSG00000033326	-1,75E-01	6,952800905	8,34E+00	3,87E-03	4,62E-02	Kdm4a	0,89	Yes
ENSMUSG00000033327	7,62E-01	-2,831967678	3,15E-01	5,74E-01	8,34E-01	Tnxb	1,70	No
ENSMUSG00000033335	-2,24E-03	7,956381966	1,47E-03	9,69E-01	1,00E+00	Dnm2	1,00	No
ENSMUSG00000033350	-4,11E-01	-0,91277331	1,02E+00	3,13E-01	6,12E-01	Chst2	0,75	No
ENSMUSG00000033352	1,32E-01	6,963716131	4,69E+00	3,04E-02	2,00E-01	Map2k4	1,10	No

ENSMUSG00000033355	-5,75E-01	2,386634789	1,88E+01	1,44E-05	4,62E-04	Rtp4	0,67	Yes
ENSMUSG00000033356	2,73E-01	2,597126468	4,61E+00	3,18E-02	2,07E-01	Pus7l	1,21	No
ENSMUSG00000033364	5,77E-02	4,854579977	5,78E-01	4,47E-01	7,38E-01	Usp37	1,04	No
ENSMUSG00000033365	2,77E-01	6,302637745	1,89E+01	1,34E-05	4,35E-04	Ipo13	1,21	Yes
ENSMUSG00000033368	-1,15E-01	-1,165709088	5,37E-02	8,17E-01	9,69E-01	Trim69	0,92	No
ENSMUSG00000033373	2,41E-01	3,359807184	5,56E+00	1,84E-02	1,43E-01	Fntb	1,18	No
ENSMUSG00000033377	-1,85E+00	-2,96319884	1,39E+00	2,38E-01	5,50E-01	Palmd	0,28	No
ENSMUSG00000033379	6,34E-02	7,36071135	1,13E+00	2,88E-01	5,84E-01	Atp6v0b	1,04	No
ENSMUSG00000033382	1,33E-01	6,649725654	4,58E+00	3,23E-02	2,10E-01	Trappc8	1,10	No
ENSMUSG00000033386	1,02E-01	7,060728143	2,86E+00	9,09E-02	3,82E-01	Frrs1	1,07	No
ENSMUSG00000033389	-8,41E-01	-2,424818802	1,03E+00	3,10E-01	6,08E-01	Arhgap44	0,56	No
ENSMUSG00000033392	-4,66E-02	6,371973998	5,42E-01	4,62E-01	7,50E-01	Clasp2	0,97	No
ENSMUSG00000033396	-1,79E-02	6,188951847	7,81E-02	7,80E-01	9,48E-01	Spg11	0,99	No
ENSMUSG00000033400	6,76E-02	6,164173771	1,10E+00	2,95E-01	5,91E-01	Agl	1,05	No
ENSMUSG00000033405	1,29E-01	1,507137741	5,66E-01	4,52E-01	7,41E-01	Nudt15	1,09	No
ENSMUSG00000033409	-1,42E-01	0,327510624	2,99E-01	5,84E-01	8,38E-01	Syce1l	0,91	No
ENSMUSG00000033411	1,26E-01	4,49337479	2,46E+00	1,17E-01	4,23E-01	Ctdspl2	1,09	No
ENSMUSG00000033416	-1,85E-01	5,821649254	7,80E+00	5,22E-03	5,77E-02	Gucd1	0,88	No
ENSMUSG00000033417	2,34E-02	6,788801428	1,45E-01	7,04E-01	9,06E-01	Cacul1	1,02	No
ENSMUSG00000033419	-5,23E-02	-2,963273906	1,24E-03	9,72E-01	1,00E+00	Snap91	0,96	No
ENSMUSG00000033420	-9,23E-02	1,04742126	2,07E-01	6,49E-01	8,75E-01	Antxr1	0,94	No
ENSMUSG00000033423	7,29E-02	4,480674676	8,06E-01	3,69E-01	6,71E-01	Eri3	1,05	No
ENSMUSG00000033427	-2,08E-01	-0,448735667	3,48E-01	5,55E-01	8,20E-01	Upb1	0,87	No
ENSMUSG00000033429	-1,45E-01	3,504441119	2,11E+00	1,46E-01	4,85E-01	Mcee	0,90	No
ENSMUSG00000033430	-8,32E-03	4,310036036	1,01E-02	9,20E-01	1,00E+00	Terf2ip	0,99	No
ENSMUSG00000033434	-8,65E-03	3,767275172	8,56E-03	9,26E-01	1,00E+00	Gtpbp6	0,99	No
ENSMUSG00000033439	-8,36E-02	3,920707237	8,49E-01	3,57E-01	6,58E-01	Trmt13	0,94	No
ENSMUSG00000033444	-8,44E-02	6,242982476	1,74E+00	1,87E-01	5,50E-01	Specc1l	0,94	No
ENSMUSG00000033446	-1,02E-01	5,699955839	2,28E+00	1,31E-01	4,55E-01	Lpar6	0,93	No
ENSMUSG00000033450	6,36E-02	3,942760403	5,03E-01	4,78E-01	7,66E-01	Tagap	1,05	No
ENSMUSG00000033454	7,91E-02	4,770955658	1,09E+00	2,96E-01	5,92E-01	Zbtb1	1,06	No
ENSMUSG00000033458	7,39E-02	2,815798922	3,97E-01	5,29E-01	8,04E-01	Fan1	1,05	No

ENSMUSG00000033467	1,33E-01	6,02630587	4,08E+00	4,33E-02	2,47E-01	Crlf2	1,10	No
ENSMUSG00000033470	9,58E-02	0,726510611	1,63E-01	6,87E-01	8,98E-01	Cysltr2	1,07	No
ENSMUSG00000033475	3,66E-02	4,878227431	2,33E-01	6,29E-01	8,62E-01	Tomm6	1,03	No
ENSMUSG00000033478	6,81E-02	5,410833067	9,58E-01	3,28E-01	6,29E-01	Fam160b1	1,05	No
ENSMUSG00000033486	-3,70E-01	0,691486331	2,76E+00	9,68E-02	3,88E-01	Catsper2	0,77	No
ENSMUSG00000033487	1,10E-01	7,516929152	3,43E+00	6,40E-02	3,16E-01	Fndc3a	1,08	No
ENSMUSG00000033488	-5,01E-02	4,013931871	3,16E-01	5,74E-01	8,33E-01	BC026585	0,97	No
ENSMUSG00000033498	7,56E-01	-2,963268003	3,13E-01	5,76E-01	8,34E-01	Strc	1,69	No
ENSMUSG00000033499	1,08E-01	7,446515532	3,32E+00	6,83E-02	3,29E-01	Larp4b	1,08	No
ENSMUSG00000033501	3,14E-02	-3,107617333	-7,69E-10	1,00E+00	1,00E+00	Crygs	1,02	No
ENSMUSG00000033502	-1,51E-01	4,871373419	4,04E+00	4,43E-02	2,51E-01	Cdc14a	0,90	No
ENSMUSG00000033508	-1,31E+00	-2,146048501	2,95E+00	8,61E-02	3,78E-01	Asprv1	0,40	No
ENSMUSG00000033520	-1,44E+00	-0,740596874	1,45E+01	1,40E-04	3,20E-03	Idi2	0,37	Yes
ENSMUSG00000033526	2,87E-02	4,69536758	1,37E-01	7,12E-01	9,11E-01	Ppip5k1	1,02	No
ENSMUSG00000033530	-1,18E-01	4,92866165	2,48E+00	1,15E-01	4,20E-01	Ttc7b	0,92	No
ENSMUSG00000033538	-3,04E-01	3,999832825	1,16E+01	6,50E-04	1,14E-02	Casp4	0,81	Yes
ENSMUSG00000033540	5,80E-02	5,336367389	6,75E-01	4,11E-01	7,07E-01	Idua	1,04	No
ENSMUSG00000033542	1,38E-01	-0,904284095	8,52E-02	7,70E-01	9,42E-01	Arhgef5	1,10	No
ENSMUSG00000033543	3,00E-02	4,38451318	1,34E-01	7,14E-01	9,12E-01	Gtf2a2	1,02	No
ENSMUSG00000033544	4,22E-01	-2,963241197	8,99E-02	7,64E-01	9,39E-01	Angptl1	1,34	No
ENSMUSG00000033545	-5,83E-02	5,456385707	7,10E-01	3,99E-01	6,98E-01	Znrf1	0,96	No
ENSMUSG00000033554	-9,63E-02	3,210010533	8,14E-01	3,67E-01	6,68E-01	Dph5	0,94	No
ENSMUSG00000033557	4,76E-02	6,423163513	5,69E-01	4,51E-01	7,40E-01	Fam20b	1,03	No
ENSMUSG00000033565	2,97E-01	3,718933396	9,80E+00	1,75E-03	2,51E-02	Rbfox2	1,23	Yes
ENSMUSG00000033576	-3,83E-02	-3,107651936	-7,91E-11	1,00E+00	1,00E+00	Apol6	0,97	No
ENSMUSG00000033577	3,58E-01	3,885681933	1,50E+01	1,08E-04	2,60E-03	Myo6	1,28	Yes
ENSMUSG00000033581	-3,48E-01	3,870370622	1,49E+01	1,12E-04	2,67E-03	Igf2bp2	0,79	Yes
ENSMUSG00000033585	1,84E+00	-3,033632273	1,33E+00	2,49E-01	5,50E-01	Ndn	3,58	No
ENSMUSG00000033589	7,67E-02	5,429017378	1,20E+00	2,73E-01	5,69E-01	Reep4	1,05	No
ENSMUSG00000033590	-2,12E-01	-1,998046952	1,10E-01	7,40E-01	9,26E-01	Myo5c	0,86	No
ENSMUSG00000033594	2,83E-01	3,15025223	6,99E+00	8,20E-03	8,05E-02	Spata2l	1,22	No
ENSMUSG00000033595	5,45E-01	1,25903549	7,52E+00	6,09E-03	6,49E-02	Lgi3	1,46	No

ENSMUSG00000033596	1,18E-01	6,046440043	3,29E+00	6,99E-02	3,34E-01	Rfwd3	1,09	No
ENSMUSG00000033597	2,46E-01	0,396086284	9,06E-01	3,41E-01	6,42E-01	Caskin1	1,19	No
ENSMUSG00000033610	1,64E-01	2,750565531	1,87E+00	1,71E-01	5,28E-01	Pank1	1,12	No
ENSMUSG00000033615	9,01E-03	-3,107646452	-5,16E-10	1,00E+00	1,00E+00	Cplx1	1,01	No
ENSMUSG00000033618	5,00E-01	-0,963205841	1,35E+00	2,46E-01	5,50E-01	Map3k13	1,41	No
ENSMUSG00000033623	1,60E-01	5,171650135	4,90E+00	2,68E-02	1,84E-01	Pcgf3	1,12	No
ENSMUSG00000033624	1,86E-01	5,717886505	7,70E+00	5,54E-03	6,02E-02	Pdpr	1,14	No
ENSMUSG00000033628	-1,71E-02	4,837804532	5,08E-02	8,22E-01	9,72E-01	Pik3c3	0,99	No
ENSMUSG00000033629	9,87E-02	5,354139369	1,95E+00	1,63E-01	5,13E-01	Hacd3	1,07	No
ENSMUSG00000033632	-5,36E-02	2,888819292	2,12E-01	6,45E-01	8,73E-01	AW554918	0,96	No
ENSMUSG00000033634	-1,88E+00	-2,998026507	1,42E+00	2,33E-01	5,50E-01	Nat8f2	0,27	No
ENSMUSG00000033644	3,25E-01	5,035090037	1,92E+01	1,19E-05	3,94E-04	Piwil2	1,25	Yes
ENSMUSG00000033653	2,30E-01	4,808253166	9,05E+00	2,63E-03	3,46E-02	Vps8	1,17	Yes
ENSMUSG00000033658	5,71E-02	5,789417458	7,23E-01	3,95E-01	6,96E-01	Ddx19b	1,04	No
ENSMUSG00000033669	1,10E-01	2,225000498	6,32E-01	4,27E-01	7,21E-01	Zfp7	1,08	No
ENSMUSG00000033671	-9,70E-02	7,655050975	2,72E+00	9,91E-02	3,92E-01	Cep350	0,93	No
ENSMUSG00000033676	-2,71E+00	-3,033620915	2,92E+00	8,75E-02	3,78E-01	Gabrb3	0,15	No
ENSMUSG00000033684	2,39E-02	5,575355212	1,22E-01	7,27E-01	9,19E-01	Qsox1	1,02	No
ENSMUSG00000033685	1,83E-01	10,15000228	1,06E+01	1,11E-03	1,76E-02	Ucp2	1,14	Yes
ENSMUSG00000033688	7,45E-01	-2,832031801	3,02E-01	5,83E-01	8,38E-01	1300017J02Rik	1,68	No
ENSMUSG00000033697	2,78E-01	5,174193716	1,47E+01	1,29E-04	3,01E-03	Arhgap39	1,21	Yes
ENSMUSG00000033701	-6,44E-03	4,602045681	6,67E-03	9,35E-01	1,00E+00	Acbd6	1,00	No
ENSMUSG00000033703	1,80E-02	3,408538761	3,20E-02	8,58E-01	9,94E-01	Fuk	1,01	No
ENSMUSG00000033705	2,41E-01	7,132447863	1,59E+01	6,55E-05	1,70E-03	Stard9	1,18	Yes
ENSMUSG00000033706	1,89E-01	4,598769884	5,72E+00	1,68E-02	1,35E-01	Smyd5	1,14	No
ENSMUSG00000033707	4,37E-02	2,91626824	1,45E-01	7,03E-01	9,06E-01	Lrrc24	1,03	No
ENSMUSG00000033712	7,29E-04	5,753915573	1,18E-04	9,91E-01	1,00E+00	Ccar2	1,00	No
ENSMUSG00000033713	-4,76E-02	4,932256879	3,99E-01	5,27E-01	8,04E-01	Foxn3	0,97	No
ENSMUSG00000033715	-1,90E+00	-3,033594154	1,44E+00	2,30E-01	5,50E-01	Akr1c14	0,27	No
ENSMUSG00000033717	8,64E-02	6,099326315	1,80E+00	1,80E-01	5,42E-01	Adra2a	1,06	No
ENSMUSG00000033720	1,84E-01	2,791248175	2,38E+00	1,23E-01	4,38E-01	Sfxn5	1,14	No
ENSMUSG00000033721	-1,09E-01	4,636655911	1,93E+00	1,65E-01	5,16E-01	Vav3	0,93	No

ENSMUSG00000033722	5,01E-01	-1,333195733	7,76E-01	3,78E-01	6,79E-01	BC034090	1,42	No
ENSMUSG00000033728	7,78E-02	4,358817447	9,04E-01	3,42E-01	6,42E-01	Lrrc14	1,06	No
ENSMUSG00000033730	-2,21E-01	5,863698093	1,03E+01	1,36E-03	2,07E-02	Egr3	0,86	Yes
ENSMUSG00000033731	2,63E+00	-2,9293308	2,68E+00	1,02E-01	3,94E-01	3300002A11Rik	6,20	No
ENSMUSG00000033732	6,89E-02	7,18074318	1,31E+00	2,52E-01	5,50E-01	Sf3b3	1,05	No
ENSMUSG00000033735	-1,57E-01	5,56023011	5,30E+00	2,13E-02	1,57E-01	Spr	0,90	No
ENSMUSG00000033739	2,13E-02	2,247876267	2,48E-02	8,75E-01	1,00E+00	Fkbpl	1,01	No
ENSMUSG00000033740	6,78E-02	3,000502858	3,49E-01	5,55E-01	8,20E-01	St18	1,05	No
ENSMUSG00000033751	-7,72E-02	4,714648003	9,98E-01	3,18E-01	6,18E-01	Gadd45gip1	0,95	No
ENSMUSG00000033752	1,77E-01	-1,278746789	1,28E-01	7,21E-01	9,16E-01	Mnd1	1,13	No
ENSMUSG00000033760	-2,20E-01	4,39296465	7,37E+00	6,62E-03	6,89E-02	Rbm4b	0,86	No
ENSMUSG00000033762	2,05E-01	3,341452408	3,80E+00	5,12E-02	2,75E-01	Recql4	1,15	No
ENSMUSG00000033763	-1,84E-01	1,644428135	1,28E+00	2,59E-01	5,51E-01	Mtss1l	0,88	No
ENSMUSG00000033765	2,46E-01	-1,740914261	1,40E-01	7,08E-01	9,09E-01	Calm4	1,19	No
ENSMUSG00000033767	-5,05E-01	7,22899015	7,09E+01	3,75E-17	7,02E-15	Tmem131l	0,70	Yes
ENSMUSG00000033768	7,10E-01	0,348221259	6,77E+00	9,25E-03	8,81E-02	Nrxn2	1,64	No
ENSMUSG00000033769	-5,37E-02	4,731610381	4,88E-01	4,85E-01	7,72E-01	Exoc6b	0,96	No
ENSMUSG00000033770	-1,94E+00	-3,107619243	1,50E+00	2,21E-01	5,50E-01	Clcnka	0,26	No
ENSMUSG00000033773	-1,76E-01	3,516278613	3,24E+00	7,19E-02	3,40E-01	Rpap2	0,89	No
ENSMUSG00000033781	-2,02E-02	3,486470194	4,14E-02	8,39E-01	9,82E-01	Asb13	0,99	No
ENSMUSG00000033788	-8,94E-01	-2,20576704	1,07E+00	3,02E-01	5,98E-01	Dysf	0,54	No
ENSMUSG00000033790	3,57E-01	4,698640584	2,13E+01	3,85E-06	1,45E-04	Tubgcp5	1,28	Yes
ENSMUSG00000033793	3,77E-02	6,259154398	3,47E-01	5,56E-01	8,21E-01	Atp6v1h	1,03	No
ENSMUSG00000033794	1,81E+00	-3,10765737	1,28E+00	2,58E-01	5,50E-01	Lpcat2b	3,49	No
ENSMUSG00000033799	8,43E-03	5,230185762	1,39E-02	9,06E-01	1,00E+00	Fam208b	1,01	No
ENSMUSG00000033805	-5,99E-02	-2,963282181	-7,02E-11	1,00E+00	1,00E+00	Ephx4	0,96	No
ENSMUSG00000033808	8,71E-02	4,906532526	1,35E+00	2,45E-01	5,50E-01	Tmem87a	1,06	No
ENSMUSG00000033809	2,53E-01	4,047575037	8,19E+00	4,21E-03	4,91E-02	Alg3	1,19	Yes
ENSMUSG00000033813	-1,29E-01	5,458452639	3,42E+00	6,43E-02	3,17E-01	Tcea1	0,91	No
ENSMUSG00000033819	-7,85E-02	4,796933309	1,07E+00	3,02E-01	5,98E-01	Ppp1r16a	0,95	No
ENSMUSG00000033826	5,06E-01	-2,289538269	3,90E-01	5,32E-01	8,06E-01	Dnah8	1,42	No
ENSMUSG00000033837	-1,74E-02	-2,831976759	1,74E-04	9,89E-01	1,00E+00	Foxh1	0,99	No

ENSMUSG00000033845	8,21E-02	4,946603482	1,21E+00	2,71E-01	5,67E-01	Mrpl15	1,06	No
ENSMUSG00000033847	-7,89E-02	-0,69750995	4,93E-02	8,24E-01	9,73E-01	Pla2g4c	0,95	No
ENSMUSG00000033849	1,35E-01	2,379998947	9,25E-01	3,36E-01	6,37E-01	B3galt2	1,10	No
ENSMUSG00000033852	2,78E-01	2,480723614	4,67E+00	3,07E-02	2,02E-01	Gm28042	1,21	No
ENSMUSG00000033854	1,33E+00	-2,268161136	1,81E+00	1,79E-01	5,39E-01	Kcnk10	2,51	No
ENSMUSG00000033855	-9,34E-01	0,569402515	1,54E+01	8,64E-05	2,15E-03	Ston1	0,52	Yes
ENSMUSG00000033857	2,30E-01	2,908113105	4,02E+00	4,50E-02	2,53E-01	Engase	1,17	No
ENSMUSG00000033862	3,55E-02	4,076275983	1,68E-01	6,82E-01	8,94E-01	Cdk10	1,02	No
ENSMUSG00000033863	2,83E-01	5,846809057	1,80E+01	2,26E-05	6,92E-04	Klf9	1,22	Yes
ENSMUSG00000033871	1,42E-01	4,532172367	3,07E+00	7,99E-02	3,62E-01	Ppargc1b	1,10	No
ENSMUSG00000033872	-6,85E-02	-2,896124416	3,23E-03	9,55E-01	1,00E+00	Best4-ps	0,95	No
ENSMUSG00000033880	8,66E-02	8,345377243	2,26E+00	1,33E-01	4,59E-01	Lgals3bp	1,06	No
ENSMUSG00000033883	-3,57E-03	3,481068837	1,29E-03	9,71E-01	1,00E+00	D3Ertd254e	1,00	No
ENSMUSG00000033885	-5,10E-02	4,94688023	4,65E-01	4,95E-01	7,81E-01	Pxk	0,97	No
ENSMUSG00000033898	-2,54E+00	-2,770550806	3,90E+00	4,83E-02	2,64E-01	Cfhr2	0,17	No
ENSMUSG00000033900	2,56E-01	-1,963344626	1,35E-01	7,13E-01	9,11E-01	Map9	1,19	No
ENSMUSG00000033902	3,31E-03	4,54637056	1,74E-03	9,67E-01	1,00E+00	Mapkbp1	1,00	No
ENSMUSG00000033904	7,40E-02	3,593398644	5,83E-01	4,45E-01	7,36E-01	Ccp110	1,05	No
ENSMUSG00000033909	7,27E-02	4,875258358	9,20E-01	3,38E-01	6,38E-01	Usp36	1,05	No
ENSMUSG00000033910	4,30E-01	-2,548281644	1,45E-01	7,04E-01	9,06E-01	Gucy1a3	1,35	No
ENSMUSG00000033916	-6,65E-02	6,094614051	1,05E+00	3,05E-01	6,02E-01	Chmp2a	0,95	No
ENSMUSG00000033917	6,72E-02	5,646902546	9,77E-01	3,23E-01	6,23E-01	Gde1	1,05	No
ENSMUSG00000033918	-5,49E-02	5,192698481	5,83E-01	4,45E-01	7,36E-01	Parl	0,96	No
ENSMUSG00000033931	1,03E-02	4,152027329	1,42E-02	9,05E-01	1,00E+00	Rbm34	1,01	No
ENSMUSG00000033933	-1,31E-01	6,651044264	4,49E+00	3,41E-02	2,18E-01	Vhl	0,91	No
ENSMUSG00000033938	4,10E-03	5,870242099	3,81E-03	9,51E-01	1,00E+00	Ndufb7	1,00	No
ENSMUSG00000033940	-8,64E-02	7,234250889	2,08E+00	1,49E-01	4,90E-01	Brk1	0,94	No
ENSMUSG00000033943	4,80E-02	6,899399936	6,20E-01	4,31E-01	7,25E-01	Mga	1,03	No
ENSMUSG00000033948	-1,53E+00	-2,226218715	3,39E+00	6,56E-02	3,21E-01	Zswim5	0,35	No
ENSMUSG00000033949	3,10E-01	5,408773985	1,97E+01	8,99E-06	3,05E-04	Trim36	1,24	Yes
ENSMUSG00000033952	3,46E-01	4,987251758	2,22E+01	2,50E-06	9,81E-05	Aspm	1,27	Yes
ENSMUSG00000033953	1,31E-02	7,615839755	4,95E-02	8,24E-01	9,73E-01	Ppp3r1	1,01	No

ENSMUSG00000033955	-8,19E-02	3,869301082	7,98E-01	3,72E-01	6,73E-01	Tnks1bp1	0,94	No
ENSMUSG00000033960	-1,30E-01	5,166522385	3,31E+00	6,88E-02	3,30E-01	Jcad	0,91	No
ENSMUSG00000033961	1,16E-01	2,043209855	6,40E-01	4,24E-01	7,18E-01	Zfp446	1,08	No
ENSMUSG00000033963	-2,17E-01	-1,34430162	1,80E-01	6,72E-01	8,89E-01	Fancd2os	0,86	No
ENSMUSG00000033964	-2,77E-02	4,277205854	1,08E-01	7,42E-01	9,27E-01	Zbtb41	0,98	No
ENSMUSG00000033966	-1,87E-01	-0,912663622	1,97E-01	6,57E-01	8,81E-01	Cdkl4	0,88	No
ENSMUSG00000033967	-1,36E-01	0,148341432	2,06E-01	6,50E-01	8,76E-01	Rnf225	0,91	No
ENSMUSG00000033970	-2,03E-01	3,836175383	4,89E+00	2,70E-02	1,85E-01	Rfc3	0,87	No
ENSMUSG00000033972	-9,21E-02	3,953709705	1,06E+00	3,02E-01	5,99E-01	Zfp944	0,94	No
ENSMUSG00000033983	2,88E-03	3,006077091	6,64E-04	9,79E-01	1,00E+00	Coil	1,00	No
ENSMUSG00000033985	3,55E-02	3,734561709	1,41E-01	7,07E-01	9,08E-01	Tesk2	1,02	No
ENSMUSG00000033987	2,03E-01	3,803619627	4,63E+00	3,14E-02	2,05E-01	Dnah17	1,15	No
ENSMUSG00000033991	1,43E-01	4,500906351	3,14E+00	7,66E-02	3,53E-01	Ttc37	1,10	No
ENSMUSG00000034006	2,76E-01	5,180032161	1,47E+01	1,25E-04	2,94E-03	Pqlc1	1,21	Yes
ENSMUSG00000034007	-1,66E-01	3,770813454	3,23E+00	7,24E-02	3,41E-01	Scaper	0,89	No
ENSMUSG00000034009	-1,13E-01	-2,963254935	5,86E-03	9,39E-01	1,00E+00	Rxfp1	0,92	No
ENSMUSG00000034021	5,69E-02	5,202618206	6,30E-01	4,27E-01	7,21E-01	Pds5b	1,04	No
ENSMUSG00000034022	2,19E-02	6,246832145	1,17E-01	7,32E-01	9,23E-01	Cpsf1	1,02	No
ENSMUSG00000034023	2,77E-01	3,899675251	9,11E+00	2,54E-03	3,37E-02	Fancd2	1,21	Yes
ENSMUSG00000034024	-3,96E-02	7,354147563	4,42E-01	5,06E-01	7,91E-01	Cct2	0,97	No
ENSMUSG00000034028	6,83E-01	1,163520225	1,12E+01	8,18E-04	1,37E-02	Cd226	1,61	Yes
ENSMUSG00000034032	1,10E-01	5,095280069	2,28E+00	1,31E-01	4,54E-01	Rpap1	1,08	No
ENSMUSG00000034035	-4,59E-01	1,688431228	7,56E+00	5,96E-03	6,38E-02	Ccdc17	0,73	No
ENSMUSG00000034037	-1,45E+00	-0,267822024	2,27E+01	1,87E-06	7,65E-05	Fgd5	0,37	Yes
ENSMUSG00000034039	-1,89E+00	-3,1076441	1,42E+00	2,33E-01	5,50E-01	Prss29	0,27	No
ENSMUSG00000034041	4,23E-01	2,921048051	1,30E+01	3,18E-04	6,36E-03	Lyl1	1,34	Yes
ENSMUSG00000034042	-3,90E-03	5,735089176	3,39E-03	9,54E-01	1,00E+00	Gpbp1l1	1,00	No
ENSMUSG00000034059	7,93E-01	-2,548257236	5,47E-01	4,60E-01	7,49E-01	Ypel4	1,73	No
ENSMUSG00000034063	-6,87E-03	-0,117089032	5,12E-04	9,82E-01	1,00E+00	4930590J08Rik	1,00	No
ENSMUSG00000034064	-4,30E-01	6,435573687	4,69E+01	7,50E-12	8,05E-10	Poglut1	0,74	Yes
ENSMUSG00000034066	1,85E-01	2,995656834	2,65E+00	1,03E-01	3,95E-01	Farp2	1,14	No
ENSMUSG00000034071	7,11E-02	-0,755651689	3,82E-02	8,45E-01	9,86E-01	Zfp551	1,05	No

ENSMUSG00000034075	7,80E-02	7,21439002	1,69E+00	1,94E-01	5,50E-01	Zdhhc5	1,06	No
ENSMUSG00000034083	1,34E-02	3,804675506	2,09E-02	8,85E-01	1,00E+00	Ccdc174	1,01	No
ENSMUSG00000034088	3,37E-02	8,300180512	3,42E-01	5,59E-01	8,22E-01	Hdlbp	1,02	No
ENSMUSG00000034101	1,60E-01	6,713669766	6,74E+00	9,41E-03	8,92E-02	Ctnnd1	1,12	No
ENSMUSG00000034105	1,08E-01	3,920285518	1,46E+00	2,27E-01	5,50E-01	Tldc1	1,08	No
ENSMUSG00000034107	-5,37E-01	-1,185451267	1,26E+00	2,61E-01	5,54E-01	Ano7	0,69	No
ENSMUSG00000034108	8,40E-03	3,77876255	8,27E-03	9,28E-01	1,00E+00	Ccs	1,01	No
ENSMUSG00000034109	2,03E-01	4,483654301	6,30E+00	1,20E-02	1,07E-01	Golim4	1,15	No
ENSMUSG00000034110	1,50E-01	3,256779894	2,04E+00	1,53E-01	4,96E-01	Kctd7	1,11	No
ENSMUSG00000034111	1,19E-01	5,521774622	2,99E+00	8,39E-02	3,71E-01	Tmed8	1,09	No
ENSMUSG00000034112	8,30E-01	-2,548222695	5,99E-01	4,39E-01	7,31E-01	Atp2c2	1,78	No
ENSMUSG00000034115	3,14E-02	-3,107617333	-7,69E-10	1,00E+00	1,00E+00	Scn11a	1,02	No
ENSMUSG00000034116	7,69E-02	6,546541893	1,51E+00	2,19E-01	5,50E-01	Vav1	1,05	No
ENSMUSG00000034117	-2,22E-01	1,222011538	1,31E+00	2,53E-01	5,50E-01	Ptgdr2	0,86	No
ENSMUSG00000034118	1,84E-01	4,086165755	4,43E+00	3,53E-02	2,22E-01	Tpst1	1,14	No
ENSMUSG00000034120	1,68E-01	7,918504221	8,30E+00	3,96E-03	4,71E-02	Srsf2	1,12	Yes
ENSMUSG00000034121	2,39E-02	2,894538097	4,17E-02	8,38E-01	9,82E-01	Mks1	1,02	No
ENSMUSG00000034126	1,50E-01	3,495540026	2,22E+00	1,36E-01	4,65E-01	Pomt2	1,11	No
ENSMUSG00000034127	-1,44E-01	0,732245259	3,68E-01	5,44E-01	8,13E-01	Tspan8	0,90	No
ENSMUSG00000034135	-2,32E-01	6,174364782	1,29E+01	3,20E-04	6,39E-03	Sik3	0,85	Yes
ENSMUSG00000034139	7,25E-01	-2,896157085	2,86E-01	5,93E-01	8,42E-01	Serpini2	1,65	No
ENSMUSG00000034145	9,01E-03	-3,107646452	-5,16E-10	1,00E+00	1,00E+00	Tmem63c	1,01	No
ENSMUSG00000034152	-8,51E-02	6,250377692	1,77E+00	1,84E-01	5,47E-01	Exoc3	0,94	No
ENSMUSG00000034154	9,45E-02	5,697267635	1,95E+00	1,62E-01	5,12E-01	Ino80	1,07	No
ENSMUSG00000034156	-4,93E-01	7,866173844	7,12E+01	3,24E-17	6,18E-15	Tspoap1	0,71	Yes
ENSMUSG00000034157	3,31E-02	4,880330974	1,95E-01	6,59E-01	8,81E-01	Cipc	1,02	No
ENSMUSG00000034158	-8,19E-02	8,516869445	2,04E+00	1,54E-01	4,97E-01	Lrrc58	0,94	No
ENSMUSG00000034159	-3,83E-02	-3,107651936	-7,91E-11	1,00E+00	1,00E+00	2310007B03Rik	0,97	No
ENSMUSG00000034161	-1,30E-01	-0,697296879	1,12E-01	7,37E-01	9,25E-01	Scx	0,91	No
ENSMUSG00000034163	-4,63E-01	6,59395906	5,54E+01	9,64E-14	1,33E-11	Zfc3h1	0,73	Yes
ENSMUSG00000034164	-3,53E+00	-2,896127731	5,59E+00	1,81E-02	1,41E-01	Emid1	0,09	No
ENSMUSG00000034165	4,63E-02	7,134687098	5,90E-01	4,42E-01	7,34E-01	Ccnd3	1,03	No

ENSMUSG00000034168	2,50E-01	4,894983666	1,08E+01	1,03E-03	1,65E-02	Irf2bpl		1,19	Yes
ENSMUSG00000034171	-1,57E-01	0,966612342	5,74E-01	4,49E-01	7,39E-01	Faah		0,90	No
ENSMUSG00000034173	8,63E-02	4,210317041	1,05E+00	3,06E-01	6,04E-01	Zbed5		1,06	No
ENSMUSG00000034175	6,13E-02	3,587680527	3,96E-01	5,29E-01	8,05E-01	Rhbdd3		1,04	No
ENSMUSG00000034177	-1,22E-01	2,076891384	7,22E-01	3,96E-01	6,96E-01	Rnf43		0,92	No
ENSMUSG00000034187	8,63E-02	6,735857029	1,95E+00	1,62E-01	5,12E-01	Nsf		1,06	No
ENSMUSG00000034189	6,76E-02	5,748151049	1,02E+00	3,12E-01	6,12E-01	Hsdl1		1,05	No
ENSMUSG00000034190	1,12E-01	5,357311321	2,55E+00	1,10E-01	4,07E-01	Chmp7		1,08	No
ENSMUSG00000034192	-4,66E-02	3,957380243	2,66E-01	6,06E-01	8,50E-01	Lsm3		0,97	No
ENSMUSG00000034194	1,34E-01	2,777333442	1,22E+00	2,69E-01	5,65E-01	R3hcc1		1,10	No
ENSMUSG00000034201	-9,23E-02	5,681256344	1,86E+00	1,73E-01	5,30E-01	Gas2l1		0,94	No
ENSMUSG00000034203	-2,46E-02	4,390165867	8,88E-02	7,66E-01	9,40E-01	Chchd4		0,98	No
ENSMUSG00000034205	-1,54E-01	-0,954576655	1,39E-01	7,09E-01	9,09E-01	Loxl2		0,90	No
ENSMUSG00000034206	2,04E-01	4,240346485	5,84E+00	1,57E-02	1,30E-01	Polq		1,15	No
ENSMUSG00000034209	-9,09E-01	-1,535222177	2,03E+00	1,54E-01	4,97E-01	Rasl10a		0,53	No
ENSMUSG00000034210	4,30E-02	5,436558347	3,83E-01	5,36E-01	8,08E-01	Efcab14		1,03	No
ENSMUSG00000034211	5,89E-02	5,210173913	6,73E-01	4,12E-01	7,08E-01	Mrps17		1,04	No
ENSMUSG00000034212	-6,16E-01	2,478752004	2,28E+01	1,82E-06	7,50E-05	Ankmy1		0,65	Yes
ENSMUSG00000034216	1,87E-01	6,717279853	9,21E+00	2,41E-03	3,25E-02	Vps18		1,14	Yes
ENSMUSG00000034218	2,34E-01	5,044247495	1,01E+01	1,51E-03	2,24E-02	Atm		1,18	Yes
ENSMUSG00000034219	-1,87E+00	-3,107637761	1,39E+00	2,38E-01	5,50E-01		41883	0,27	No
ENSMUSG00000034220	-6,61E-01	6,741573049	1,15E+02	9,19E-27	3,72E-24	Gpc1		0,63	Yes
ENSMUSG00000034226	-5,39E-02	3,199955204	2,37E-01	6,26E-01	8,60E-01	Rhov		0,96	No
ENSMUSG00000034227	-6,91E-01	-1,015789131	2,90E+00	8,88E-02	3,79E-01	Foxj1		0,62	No
ENSMUSG00000034235	1,25E-02	3,145349724	1,30E-02	9,09E-01	1,00E+00	Usp54		1,01	No
ENSMUSG00000034243	1,01E-02	7,389953008	2,91E-02	8,64E-01	9,98E-01	Golgb1		1,01	No
ENSMUSG00000034245	-1,01E-01	-0,655110762	7,08E-02	7,90E-01	9,54E-01	Hdac11		0,93	No
ENSMUSG00000034247	2,11E-01	6,673285217	1,16E+01	6,53E-04	1,14E-02	Plekhm1		1,16	Yes
ENSMUSG00000034248	8,27E-02	5,400105949	1,40E+00	2,37E-01	5,50E-01	Slc25a37		1,06	No
ENSMUSG00000034252	-1,02E-01	5,90672859	2,36E+00	1,24E-01	4,41E-01	Senp6		0,93	No
ENSMUSG00000034254	4,11E-02	6,226592021	4,11E-01	5,21E-01	8,00E-01	Agpat1		1,03	No
ENSMUSG00000034255	1,66E-01	5,688923979	6,06E+00	1,38E-02	1,19E-01	Arhgap27		1,12	No

ENSMUSG00000034258	3,08E-02	0,55149469	1,44E-02	9,05E-01	1,00E+00	Mfsd7c	1,02	No
ENSMUSG00000034259	-5,43E-02	4,014974149	3,74E-01	5,41E-01	8,11E-01	Exosc4	0,96	No
ENSMUSG00000034263	-1,35E-02	4,97194771	3,27E-02	8,56E-01	9,93E-01	Ints14	0,99	No
ENSMUSG00000034265	-1,30E-02	4,230535244	2,35E-02	8,78E-01	1,00E+00	Zdhhc14	0,99	No
ENSMUSG00000034266	7,83E-02	3,877800543	7,36E-01	3,91E-01	6,91E-01	Batf	1,06	No
ENSMUSG00000034269	1,46E-02	7,349083567	5,96E-02	8,07E-01	9,64E-01	Setd5	1,01	No
ENSMUSG00000034271	1,45E-01	3,36126828	2,02E+00	1,56E-01	5,00E-01	Jdp2	1,11	No
ENSMUSG00000034274	3,69E-02	4,657894187	2,23E-01	6,37E-01	8,68E-01	Thoc5	1,03	No
ENSMUSG00000034275	-1,92E-01	0,183611887	5,35E-01	4,65E-01	7,53E-01	Igsf9b	0,88	No
ENSMUSG00000034278	-2,63E-01	1,396121378	2,05E+00	1,53E-01	4,95E-01	Dnajc17	0,83	No
ENSMUSG00000034282	-1,55E-01	-1,126713801	1,16E-01	7,34E-01	9,23E-01	Evpl	0,90	No
ENSMUSG00000034285	-5,33E-01	3,202973355	2,50E+01	5,80E-07	2,72E-05	Nipsnap1	0,69	Yes
ENSMUSG00000034290	6,08E-02	7,266244891	1,03E+00	3,10E-01	6,09E-01	Nek9	1,04	No
ENSMUSG00000034292	3,34E-01	2,094812559	5,20E+00	2,26E-02	1,64E-01	Traf3ip1	1,26	No
ENSMUSG00000034295	-3,66E-01	0,317179726	2,10E+00	1,48E-01	4,87E-01	Fhod3	0,78	No
ENSMUSG00000034297	7,70E-03	6,870655564	1,59E-02	9,00E-01	1,00E+00	Med13	1,01	No
ENSMUSG00000034300	1,01E-01	5,708479729	2,27E+00	1,32E-01	4,57E-01	Fam53c	1,07	No
ENSMUSG00000034303	1,19E-01	1,50412746	4,37E-01	5,08E-01	7,92E-01	Ccdc15	1,09	No
ENSMUSG00000034308	3,16E-01	2,392168177	5,52E+00	1,88E-02	1,45E-01	Sdr42e1	1,25	No
ENSMUSG00000034312	1,32E-01	7,132128831	4,76E+00	2,90E-02	1,94E-01	Iqsec1	1,10	No
ENSMUSG00000034317	1,74E-02	4,0431715	3,99E-02	8,42E-01	9,84E-01	Trim59	1,01	No
ENSMUSG00000034320	-2,05E-01	6,678946918	1,10E+01	9,01E-04	1,49E-02	Slc26a2	0,87	Yes
ENSMUSG00000034321	9,03E-03	3,270894476	7,34E-03	9,32E-01	1,00E+00	Exosc1	1,01	No
ENSMUSG00000034324	7,05E-01	-2,963278876	2,69E-01	6,04E-01	8,49E-01	Tmem132c	1,63	No
ENSMUSG00000034327	6,26E-03	5,049332909	7,38E-03	9,32E-01	1,00E+00	Kctd9	1,00	No
ENSMUSG00000034329	2,89E-01	4,144429101	1,12E+01	8,27E-04	1,38E-02	Brip1	1,22	Yes
ENSMUSG00000034330	6,74E-02	7,346422534	1,27E+00	2,60E-01	5,52E-01	Plcg2	1,05	No
ENSMUSG00000034333	2,77E-01	4,602000471	1,25E+01	3,96E-04	7,57E-03	Zbed4	1,21	Yes
ENSMUSG00000034334	-1,06E-01	1,604558632	3,68E-01	5,44E-01	8,13E-01	Fam151b	0,93	No
ENSMUSG00000034336	8,12E-01	-2,185667326	1,20E+00	2,72E-01	5,69E-01	Ina	1,76	No
ENSMUSG00000034341	5,93E-02	6,641319955	9,15E-01	3,39E-01	6,39E-01	Wbp2	1,04	No
ENSMUSG00000034342	-8,06E-02	8,968918566	2,01E+00	1,57E-01	5,01E-01	Cbl	0,95	No

ENSMUSG00000034343	-8,25E-02	5,529086106	1,45E+00	2,28E-01	5,50E-01	Ube2f	0,94	No
ENSMUSG00000034345	-2,88E-02	4,982443466	1,51E-01	6,97E-01	9,03E-01	Gtf2h5	0,98	No
ENSMUSG00000034349	1,28E-01	7,065230352	4,46E+00	3,46E-02	2,20E-01	Smc4	1,09	No
ENSMUSG00000034353	2,36E-01	5,830752542	1,23E+01	4,44E-04	8,33E-03	Ramp1	1,18	Yes
ENSMUSG00000034354	-9,90E-03	7,64824506	2,82E-02	8,67E-01	9,99E-01	Mtmr3	0,99	No
ENSMUSG00000034361	-1,18E-01	5,966200684	3,23E+00	7,22E-02	3,41E-01	Cpne2	0,92	No
ENSMUSG00000034362	1,85E+00	-3,107657832	1,35E+00	2,46E-01	5,50E-01	Csta1	3,61	No
ENSMUSG00000034371	-2,84E-02	3,698655778	9,09E-02	7,63E-01	9,39E-01	Tkfc	0,98	No
ENSMUSG00000034377	2,40E-02	7,419064899	1,63E-01	6,87E-01	8,97E-01	Tulp4	1,02	No
ENSMUSG00000034379	4,58E-02	2,116296579	1,00E-01	7,51E-01	9,32E-01	Wdr5b	1,03	No
ENSMUSG00000034382	1,01E-01	1,365371123	3,08E-01	5,79E-01	8,36E-01	AI661453	1,07	No
ENSMUSG00000034384	9,01E-03	-3,107646452	-5,16E-10	1,00E+00	1,00E+00	Barhl2	1,01	No
ENSMUSG00000034390	-1,66E-01	6,541665465	7,03E+00	8,02E-03	7,92E-02	Cmip	0,89	No
ENSMUSG00000034391	1,87E+00	-3,107639691	1,38E+00	2,40E-01	5,50E-01	Fbxo15	3,67	No
ENSMUSG00000034394	-9,64E-01	2,232418363	4,48E+01	2,17E-11	2,22E-09	Lif	0,51	Yes
ENSMUSG00000034401	1,14E-01	4,962221035	2,33E+00	1,27E-01	4,46E-01	Spata6	1,08	No
ENSMUSG00000034402	-1,87E+00	-3,107637761	1,39E+00	2,38E-01	5,50E-01	Kcnh5	0,27	No
ENSMUSG00000034412	-2,03E-02	4,650220964	6,69E-02	7,96E-01	9,57E-01	Tbc1d10a	0,99	No
ENSMUSG00000034413	3,23E-01	3,028339223	8,60E+00	3,37E-03	4,17E-02	Neurl1b	1,25	Yes
ENSMUSG00000034416	-9,41E-02	-0,079276079	8,87E-02	7,66E-01	9,40E-01	Pkd1l2	0,94	No
ENSMUSG00000034422	7,75E-02	6,750612794	1,60E+00	2,06E-01	5,50E-01	Parp14	1,06	No
ENSMUSG00000034424	1,03E-01	4,433932837	1,56E+00	2,11E-01	5,50E-01	Gcsh	1,07	No
ENSMUSG00000034427	3,75E-02	-1,96322517	2,68E-03	9,59E-01	1,00E+00	Myo15b	1,03	No
ENSMUSG00000034429	1,08E-01	2,575134537	7,26E-01	3,94E-01	6,94E-01	Zfp707	1,08	No
ENSMUSG00000034430	2,81E-01	4,982038902	1,45E+01	1,37E-04	3,14E-03	Zxdc	1,22	Yes
ENSMUSG00000034432	-2,75E-02	5,849145302	1,71E-01	6,79E-01	8,93E-01	Cops8	0,98	No
ENSMUSG00000034435	-7,56E-01	-2,401333048	6,41E-01	4,24E-01	7,18E-01	Tmem30b	0,59	No
ENSMUSG00000034437	-1,77E-02	6,27587102	7,70E-02	7,81E-01	9,49E-01	Gm9761	0,99	No
ENSMUSG00000034438	-2,13E-01	5,367982807	9,29E+00	2,30E-03	3,13E-02	Gbp8	0,86	Yes
ENSMUSG00000034442	1,05E-01	3,376278237	1,05E+00	3,05E-01	6,02E-01	Trmt5	1,08	No
ENSMUSG00000034445	-1,97E-01	5,790267249	8,79E+00	3,03E-03	3,85E-02	Cyb561a3	0,87	Yes
ENSMUSG00000034449	-7,25E-02	4,468116477	7,96E-01	3,72E-01	6,73E-01	Dhrs11	0,95	No

ENSMUSG00000034452	-6,58E-01	-0,863667517	2,09E+00	1,48E-01	4,88E-01	Slc24a1	0,63	No
ENSMUSG00000034453	1,42E-01	4,437098208	3,05E+00	8,08E-02	3,64E-01	Polr3b	1,10	No
ENSMUSG00000034456	-7,72E-02	-1,267951836	2,65E-02	8,71E-01	1,00E+00	Uroc1	0,95	No
ENSMUSG00000034459	-3,06E-01	2,202162951	4,80E+00	2,85E-02	1,91E-01	Ifit1	0,81	No
ENSMUSG00000034460	3,09E-01	1,369102362	2,37E+00	1,24E-01	4,40E-01	Six4	1,24	No
ENSMUSG00000034462	-1,96E-02	4,9045304	6,90E-02	7,93E-01	9,56E-01	Pkd2	0,99	No
ENSMUSG00000034463	3,37E-02	0,882888616	2,71E-02	8,69E-01	1,00E+00	Scara3	1,02	No
ENSMUSG00000034471	3,04E-02	3,889660545	1,13E-01	7,37E-01	9,25E-01	Caskin2	1,02	No
ENSMUSG00000034472	-6,90E-01	-0,946053387	2,88E+00	8,96E-02	3,80E-01	Rasd2	0,62	No
ENSMUSG00000034473	-3,23E-02	3,876412792	1,28E-01	7,21E-01	9,16E-01	Sec22a	0,98	No
ENSMUSG00000034484	1,95E-01	7,435853761	1,07E+01	1,08E-03	1,72E-02	Snx2	1,14	Yes
ENSMUSG00000034485	8,75E-02	4,231546588	1,06E+00	3,03E-01	5,99E-01	Uaca	1,06	No
ENSMUSG00000034486	3,90E-01	-2,424908769	1,80E-01	6,71E-01	8,89E-01	Gbx2	1,31	No
ENSMUSG00000034487	4,05E-02	3,065925415	1,37E-01	7,11E-01	9,10E-01	Kdelc2	1,03	No
ENSMUSG00000034488	5,13E-01	-0,473235311	2,24E+00	1,35E-01	4,62E-01	Edil3	1,43	No
ENSMUSG00000034493	1,94E-01	0,627453292	6,40E-01	4,24E-01	7,18E-01	4930556J24Rik	1,14	No
ENSMUSG00000034501	2,48E-01	3,419274881	5,86E+00	1,55E-02	1,29E-01	Pcnx4	1,19	No
ENSMUSG00000034509	-1,67E-01	4,602041943	4,49E+00	3,41E-02	2,18E-01	Mad2l1bp	0,89	No
ENSMUSG00000034518	2,15E-01	4,583083899	7,43E+00	6,42E-03	6,74E-02	Hmgxb4	1,16	No
ENSMUSG00000034520	-2,93E-01	-2,185661065	1,49E-01	7,00E-01	9,04E-01	Gjc1	0,82	No
ENSMUSG00000034522	4,49E-01	3,096511115	1,63E+01	5,34E-05	1,43E-03	Zfp395	1,37	Yes
ENSMUSG00000034525	-3,76E-02	6,544108496	3,63E-01	5,47E-01	8,14E-01	Ice1	0,97	No
ENSMUSG00000034528	-8,69E-01	-2,963204587	4,16E-01	5,19E-01	7,98E-01	Hsd17b13	0,55	No
ENSMUSG00000034538	-2,37E-01	-0,088678891	6,03E-01	4,37E-01	7,30E-01	Zfp418	0,85	No
ENSMUSG00000034543	1,23E-01	5,631277123	3,29E+00	6,95E-02	3,32E-01	Morc2a	1,09	No
ENSMUSG00000034544	-6,03E-02	4,326954056	5,30E-01	4,67E-01	7,55E-01	Rsrc1	0,96	No
ENSMUSG00000034552	-1,87E+00	-3,107637761	1,39E+00	2,38E-01	5,50E-01	Zswim2	0,27	No
ENSMUSG00000034557	3,40E-03	2,287441343	5,97E-04	9,81E-01	1,00E+00	Zfyve9	1,00	No
ENSMUSG00000034560	1,68E-01	6,238128837	6,92E+00	8,54E-03	8,31E-02	Washc4	1,12	No
ENSMUSG00000034563	-1,61E-02	5,167705237	4,98E-02	8,23E-01	9,73E-01	Ccpg1	0,99	No
ENSMUSG00000034566	-4,71E-02	4,486317112	3,43E-01	5,58E-01	8,22E-01	Atp5h	0,97	No
ENSMUSG00000034570	4,54E-02	1,342582603	5,85E-02	8,09E-01	9,65E-01	Inpp5j	1,03	No

ENSMUSG00000034573	-1,35E+00	-2,896129282	1,14E+00	2,85E-01	5,81E-01	Ptpn13	0,39	No
ENSMUSG00000034574	-6,93E-02	4,409997831	7,20E-01	3,96E-01	6,97E-01	Daam1	0,95	No
ENSMUSG00000034575	1,20E-01	5,155747801	2,77E+00	9,61E-02	3,87E-01	Papd7	1,09	No
ENSMUSG00000034579	1,41E-01	-1,195796021	9,71E-02	7,55E-01	9,34E-01	Pla2g3	1,10	No
ENSMUSG00000034584	-1,26E+00	-1,378161737	5,50E+00	1,90E-02	1,46E-01	Exph5	0,42	No
ENSMUSG00000034586	-1,20E+00	-1,28954812	4,88E+00	2,72E-02	1,86E-01	Hid1	0,44	No
ENSMUSG00000034587	-2,22E-02	1,991974738	2,16E-02	8,83E-01	1,00E+00	8430429K09Rik	0,98	No
ENSMUSG00000034591	1,58E-01	5,4318732	5,12E+00	2,37E-02	1,69E-01	Slc41a2	1,12	No
ENSMUSG00000034593	9,36E-02	8,692534834	2,68E+00	1,02E-01	3,94E-01	Myo5a	1,07	No
ENSMUSG00000034595	2,96E-03	7,595643499	2,51E-03	9,60E-01	1,00E+00	Ppp1r18	1,00	No
ENSMUSG00000034601	1,16E-01	3,6255794	1,45E+00	2,29E-01	5,50E-01	2700049A03Rik	1,08	No
ENSMUSG00000034602	1,09E-01	6,603878251	3,05E+00	8,05E-02	3,64E-01	Mon2	1,08	No
ENSMUSG00000034610	-1,09E-01	4,373483529	1,74E+00	1,87E-01	5,50E-01	Zcchc11	0,93	No
ENSMUSG00000034612	1,93E-02	6,973354459	1,01E-01	7,51E-01	9,32E-01	Chst11	1,01	No
ENSMUSG00000034613	-9,74E-02	6,385646868	2,36E+00	1,25E-01	4,42E-01	Ppm1h	0,93	No
ENSMUSG00000034614	1,80E-01	1,959038004	1,48E+00	2,24E-01	5,50E-01	Pik3ip1	1,13	No
ENSMUSG00000034616	3,30E-02	6,141520427	2,62E-01	6,09E-01	8,51E-01	Ssh3	1,02	No
ENSMUSG00000034617	7,95E-02	3,864539413	7,48E-01	3,87E-01	6,87E-01	Mtrr	1,06	No
ENSMUSG00000034620	8,39E-02	4,681129111	1,16E+00	2,82E-01	5,78E-01	Tmem5	1,06	No
ENSMUSG00000034621	4,72E-02	5,809744509	5,05E-01	4,77E-01	7,65E-01	Gpatch8	1,03	No
ENSMUSG00000034634	-6,93E-02	-1,711773846	1,12E-02	9,16E-01	1,00E+00	Ly6d	0,95	No
ENSMUSG00000034636	4,05E-02	6,167992696	3,97E-01	5,29E-01	8,04E-01	Zyg11b	1,03	No
ENSMUSG00000034639	4,35E-02	1,120307964	4,70E-02	8,28E-01	9,76E-01	Setmar	1,03	No
ENSMUSG00000034640	-1,76E-01	7,019748491	8,41E+00	3,72E-03	4,48E-02	Tiparp	0,89	Yes
ENSMUSG00000034641	-9,91E-02	5,279978036	1,92E+00	1,66E-01	5,19E-01	Cd300ld	0,93	No
ENSMUSG00000034647	-9,70E-02	5,760053339	2,09E+00	1,48E-01	4,88E-01	Ankrd12	0,93	No
ENSMUSG00000034652	8,14E-02	6,476487024	1,67E+00	1,96E-01	5,50E-01	Cd300a	1,06	No
ENSMUSG00000034653	1,67E-01	4,483117119	4,27E+00	3,88E-02	2,33E-01	Ythdc2	1,12	No
ENSMUSG00000034656	-3,38E-01	-0,472907408	9,45E-01	3,31E-01	6,31E-01	Cacna1a	0,79	No
ENSMUSG00000034659	1,00E-01	6,838391828	2,67E+00	1,02E-01	3,94E-01	Tmem109	1,07	No
ENSMUSG00000034663	-3,81E-01	7,9676923	4,28E+01	6,12E-11	5,97E-09	Bmp2k	0,77	Yes
ENSMUSG00000034664	-6,11E-01	1,409592513	1,13E+01	7,64E-04	1,30E-02	Itga2b	0,65	Yes

ENSMUSG00000034667	1,78E-01	5,652940352	6,85E+00	8,85E-03	8,54E-02	Xpot	1,13	No
ENSMUSG00000034673	-1,03E-01	5,885109705	2,44E+00	1,18E-01	4,27E-01	Pbx2	0,93	No
ENSMUSG00000034674	9,20E-02	4,470216467	1,29E+00	2,56E-01	5,50E-01	Tdg	1,07	No
ENSMUSG00000034675	6,11E-01	2,892657357	2,57E+01	3,92E-07	1,91E-05	Dbn1	1,53	Yes
ENSMUSG00000034681	-5,92E-03	5,241213359	6,85E-03	9,34E-01	1,00E+00	Rnps1	1,00	No
ENSMUSG00000034683	-3,53E+00	-2,831995661	5,58E+00	1,82E-02	1,42E-01	Ppp1r1c	0,09	No
ENSMUSG00000034684	-2,47E+00	-2,355553582	5,31E+00	2,12E-02	1,57E-01	Sema3f	0,18	No
ENSMUSG00000034685	3,80E-01	0,630500858	2,46E+00	1,17E-01	4,23E-01	Fam171a2	1,30	No
ENSMUSG00000034686	-1,53E-01	0,86136891	4,88E-01	4,85E-01	7,72E-01	Prr7	0,90	No
ENSMUSG00000034687	-2,26E+00	-2,378202308	6,74E+00	9,44E-03	8,94E-02	Fras1	0,21	No
ENSMUSG00000034690	-3,83E-02	-3,107651936	-7,91E-11	1,00E+00	1,00E+00	Nlrp4c	0,97	No
ENSMUSG00000034701	-1,53E+00	-2,863717471	1,53E+00	2,16E-01	5,50E-01	Neurod1	0,35	No
ENSMUSG00000034706	-9,35E-01	-2,600669529	7,65E-01	3,82E-01	6,83E-01	Dnaic2	0,52	No
ENSMUSG00000034707	1,22E-01	8,923273622	4,57E+00	3,26E-02	2,11E-01	Gns	1,09	No
ENSMUSG00000034708	1,87E-01	10,12884552	1,10E+01	9,08E-04	1,50E-02	Grn	1,14	Yes
ENSMUSG00000034709	1,37E-01	6,374669459	4,70E+00	3,01E-02	1,99E-01	Ppp1r21	1,10	No
ENSMUSG00000034714	-8,48E-02	5,111794129	1,37E+00	2,42E-01	5,50E-01	Ttyh2	0,94	No
ENSMUSG00000034723	1,97E-01	5,053809965	7,11E+00	7,67E-03	7,66E-02	Tmx4	1,15	No
ENSMUSG00000034724	1,79E-02	6,445837063	8,11E-02	7,76E-01	9,45E-01	Cnot6l	1,01	No
ENSMUSG00000034729	-9,94E-03	3,383759329	9,46E-03	9,23E-01	1,00E+00	Mrps10	0,99	No
ENSMUSG00000034730	8,54E-01	-2,311207953	1,12E+00	2,90E-01	5,86E-01	Adgrb1	1,81	No
ENSMUSG00000034731	1,66E-01	2,462720943	1,64E+00	2,01E-01	5,50E-01	Dgkh	1,12	No
ENSMUSG00000034738	-1,59E-01	4,041451995	3,33E+00	6,81E-02	3,28E-01	Nostrin	0,90	No
ENSMUSG00000034739	-3,81E-01	-2,811458701	8,98E-02	7,64E-01	9,39E-01	Mfrp	0,77	No
ENSMUSG00000034744	7,46E-02	5,433688148	1,16E+00	2,82E-01	5,79E-01	Nagk	1,05	No
ENSMUSG00000034748	5,09E-02	3,996269924	3,29E-01	5,66E-01	8,28E-01	Sirt6	1,04	No
ENSMUSG00000034751	4,26E-01	4,282689003	2,54E+01	4,72E-07	2,26E-05	Mast4	1,34	Yes
ENSMUSG00000034757	1,13E-01	5,630600367	2,77E+00	9,61E-02	3,87E-01	Tmub2	1,08	No
ENSMUSG00000034758	-1,18E-01	2,650751186	9,02E-01	3,42E-01	6,43E-01	Tle6	0,92	No
ENSMUSG00000034761	-1,81E-01	3,621236595	3,52E+00	6,05E-02	3,05E-01	Map4k5	0,88	No
ENSMUSG00000034762	-7,54E-01	-2,401420905	6,36E-01	4,25E-01	7,19E-01	Glis1	0,59	No
ENSMUSG00000034764	1,96E-01	-2,051774412	7,74E-02	7,81E-01	9,49E-01	1700006J14Rik	1,15	No

ENSMUSG00000034765	-5,98E-01	6,560350923	9,14E+01	1,15E-21	3,07E-19	Dusp5	0,66	Yes
ENSMUSG00000034768	9,09E-01	1,461023306	2,39E+01	1,04E-06	4,56E-05	Asb16	1,88	Yes
ENSMUSG00000034771	-1,28E-01	3,771408352	1,93E+00	1,64E-01	5,16E-01	Tle2	0,91	No
ENSMUSG00000034773	4,08E-01	4,057945575	2,18E+01	2,98E-06	1,15E-04	BC030867	1,33	Yes
ENSMUSG00000034777	-3,83E-02	-3,107651936	-7,91E-11	1,00E+00	1,00E+00	Vax2	0,97	No
ENSMUSG00000034780	1,79E+00	-3,033666867	1,28E+00	2,58E-01	5,50E-01	B3galt1	3,46	No
ENSMUSG00000034781	-2,87E-02	4,289266023	1,17E-01	7,32E-01	9,22E-01	Gna11	0,98	No
ENSMUSG00000034783	2,13E+00	1,751225054	1,20E+02	6,19E-28	2,94E-25	Cd207	4,36	Yes
ENSMUSG00000034785	1,13E+00	-2,71172953	1,22E+00	2,69E-01	5,65E-01	Dio1	2,20	No
ENSMUSG00000034786	-9,70E-02	6,069315853	2,23E+00	1,35E-01	4,62E-01	Gpsm3	0,93	No
ENSMUSG00000034789	1,62E-01	4,966161556	4,70E+00	3,02E-02	1,99E-01	Rab24	1,12	No
ENSMUSG00000034792	1,63E-01	6,447336184	6,72E+00	9,53E-03	8,99E-02	Gna15	1,12	No
ENSMUSG00000034793	6,34E-02	5,475865328	8,32E-01	3,62E-01	6,63E-01	G6pc3	1,04	No
ENSMUSG00000034795	-3,10E-01	3,09513719	8,10E+00	4,42E-03	5,09E-02	Ccdc122	0,81	No
ENSMUSG00000034796	-2,70E+00	-3,033614577	2,89E+00	8,93E-02	3,79E-01	Cpne7	0,15	No
ENSMUSG00000034799	9,73E-02	6,077071089	2,25E+00	1,33E-01	4,60E-01	Unc13a	1,07	No
ENSMUSG00000034800	4,82E-01	1,309107657	6,91E+00	8,56E-03	8,33E-02	Zfp661	1,40	No
ENSMUSG00000034801	3,63E-04	5,682860245	2,89E-05	9,96E-01	1,00E+00	Sos2	1,00	No
ENSMUSG00000034807	1,04E-01	9,266870338	3,35E+00	6,70E-02	3,26E-01	Colgalt1	1,07	No
ENSMUSG00000034813	2,26E-01	-2,448635567	6,42E-02	8,00E-01	9,60E-01	Grip1	1,17	No
ENSMUSG00000034818	-9,53E-01	0,274138149	1,26E+01	3,76E-04	7,27E-03	Celf5	0,52	Yes
ENSMUSG00000034820	-1,09E-01	6,627492908	3,06E+00	8,01E-02	3,62E-01	Cpsf7	0,93	No
ENSMUSG00000034825	3,73E-01	-1,832069154	3,36E-01	5,62E-01	8,25E-01	Nrip3	1,30	No
ENSMUSG00000034826	-9,83E-02	4,566927716	1,53E+00	2,16E-01	5,50E-01	Nup54	0,93	No
ENSMUSG00000034829	1,14E-01	-0,641425658	8,83E-02	7,66E-01	9,40E-01	Nxnl1	1,08	No
ENSMUSG00000034832	2,10E-01	7,229866442	1,22E+01	4,73E-04	8,76E-03	Tet3	1,16	Yes
ENSMUSG00000034833	6,02E-01	0,644112197	6,60E+00	1,02E-02	9,49E-02	Tespa1	1,52	No
ENSMUSG00000034837	-2,82E+00	-2,49751131	7,68E+00	5,58E-03	6,06E-02	Gnat1	0,14	No
ENSMUSG00000034839	-4,36E-01	-2,497590272	1,94E-01	6,59E-01	8,82E-01	Larp6	0,74	No
ENSMUSG00000034842	1,36E+00	-2,088743841	2,96E+00	8,51E-02	3,75E-01	Art3	2,57	No
ENSMUSG00000034845	8,57E-02	-2,107713066	1,44E-02	9,05E-01	1,00E+00	Plvap	1,06	No
ENSMUSG00000034848	2,11E-01	4,370097503	6,39E+00	1,14E-02	1,03E-01	Ttc21b	1,16	No

ENSMUSG00000034850	-1,06E-01	7,137850095	3,08E+00	7,92E-02	3,60E-01	Tmem127	0,93	No
ENSMUSG00000034853	2,40E-01	4,85852958	1,02E+01	1,37E-03	2,08E-02	Acot11	1,18	Yes
ENSMUSG00000034854	2,94E-01	7,664656733	2,49E+01	5,97E-07	2,79E-05	Mfsd12	1,23	Yes
ENSMUSG00000034855	-4,88E-01	1,041908354	5,73E+00	1,67E-02	1,35E-01	Cxcl10	0,71	No
ENSMUSG00000034858	7,03E-03	4,540119123	7,89E-03	9,29E-01	1,00E+00	Fam214a	1,00	No
ENSMUSG00000034863	9,82E-02	4,715929312	1,63E+00	2,02E-01	5,50E-01	Ano8	1,07	No
ENSMUSG00000034867	-1,18E-01	5,099011208	2,62E+00	1,06E-01	3,98E-01	Ankrd27	0,92	No
ENSMUSG00000034868	-8,12E-02	7,758444789	1,92E+00	1,66E-01	5,19E-01	Myl12b	0,95	No
ENSMUSG00000034871	3,77E-01	2,92013718	1,10E+01	8,90E-04	1,47E-02	Fam151a	1,30	Yes
ENSMUSG00000034872	1,29E-01	1,083230103	4,24E-01	5,15E-01	7,96E-01	Gipc3	1,09	No
ENSMUSG00000034875	1,23E-02	4,567005933	2,35E-02	8,78E-01	1,00E+00	Nudt19	1,01	No
ENSMUSG00000034880	4,81E-02	4,43499707	3,51E-01	5,54E-01	8,19E-01	Mrpl34	1,03	No
ENSMUSG00000034881	3,14E+00	-2,711708169	4,03E+00	4,47E-02	2,52E-01	Tbxa2r	8,83	No
ENSMUSG00000034883	3,89E-01	2,153043949	7,19E+00	7,33E-03	7,42E-02	Lrr1	1,31	No
ENSMUSG00000034889	9,59E-02	5,71251401	2,01E+00	1,56E-01	5,00E-01	Cactin	1,07	No
ENSMUSG00000034892	-1,88E-01	1,248322681	1,00E+00	3,17E-01	6,18E-01	Rps29	0,88	No
ENSMUSG00000034893	-3,29E-02	5,415849183	2,24E-01	6,36E-01	8,67E-01	Cog3	0,98	No
ENSMUSG00000034898	-6,71E-01	-0,413020262	3,60E+00	5,79E-02	2,97E-01	Filip1	0,63	No
ENSMUSG00000034902	-2,98E-03	8,539131743	2,69E-03	9,59E-01	1,00E+00	Pip5k1c	1,00	No
ENSMUSG00000034903	2,38E-01	1,502080713	1,68E+00	1,95E-01	5,50E-01	Cobll1	1,18	No
ENSMUSG00000034906	1,58E-01	5,004219973	4,53E+00	3,32E-02	2,14E-01	Ncaph	1,12	No
ENSMUSG00000034908	1,74E-01	8,48421148	9,19E+00	2,43E-03	3,26E-02	Sidt2	1,13	Yes
ENSMUSG00000034910	-2,71E+00	-2,963235336	2,92E+00	8,75E-02	3,78E-01	Pygo1	0,15	No
ENSMUSG00000034911	-5,74E-01	-2,49760605	2,58E-01	6,11E-01	8,52E-01	Ushbp1	0,67	No
ENSMUSG00000034917	-1,83E-01	2,907427047	2,56E+00	1,09E-01	4,06E-01	Tjp3	0,88	No
ENSMUSG00000034918	2,64E+00	-2,963240788	2,69E+00	1,01E-01	3,92E-01	Cdhr2	6,24	No
ENSMUSG00000034926	2,39E-01	7,732459206	1,65E+01	4,78E-05	1,31E-03	Dhcr24	1,18	Yes
ENSMUSG00000034928	1,18E-01	7,304171599	3,92E+00	4,78E-02	2,62E-01	Rnf44	1,09	No
ENSMUSG00000034930	4,18E-01	1,665037977	6,28E+00	1,22E-02	1,08E-01	Rtkn	1,34	No
ENSMUSG00000034931	1,02E-01	5,441740035	2,17E+00	1,41E-01	4,74E-01	Dhx8	1,07	No
ENSMUSG00000034932	1,10E-01	4,573158031	1,94E+00	1,64E-01	5,15E-01	Mrpl54	1,08	No
ENSMUSG00000034936	-3,16E-02	-0,436829952	7,09E-03	9,33E-01	1,00E+00	Arl4d	0,98	No

ENSMUSG00000034940	1,39E-01	6,32471567	4,76E+00	2,91E-02	1,94E-01	Synrg	1,10	No
ENSMUSG00000034947	2,02E-02	6,465485666	1,04E-01	7,47E-01	9,30E-01	Tmem106a	1,01	No
ENSMUSG00000034949	-1,01E+00	1,230187415	2,79E+01	1,28E-07	6,95E-06	Zfr2	0,50	Yes
ENSMUSG00000034951	8,67E-02	4,823812682	1,29E+00	2,56E-01	5,50E-01	Cog7	1,06	No
ENSMUSG00000034957	2,55E-01	7,122784086	1,77E+01	2,52E-05	7,57E-04	Cebpa	1,19	Yes
ENSMUSG00000034958	-8,49E-01	-0,67592889	5,37E+00	2,04E-02	1,54E-01	Atcay	0,56	No
ENSMUSG00000034959	1,80E-01	3,5789382	3,41E+00	6,49E-02	3,19E-01	Rubcnl	1,13	No
ENSMUSG00000034968	1,08E-01	-0,548167746	8,88E-02	7,66E-01	9,40E-01	Lbx2	1,08	No
ENSMUSG00000034973	2,01E-04	6,11657814	9,73E-06	9,98E-01	1,00E+00	Dopey1	1,00	No
ENSMUSG00000034974	4,88E-02	5,239924338	4,69E-01	4,93E-01	7,79E-01	Dapk3	1,03	No
ENSMUSG00000034981	-3,63E-01	-0,946274268	8,83E-01	3,47E-01	6,48E-01	Parm1	0,78	No
ENSMUSG00000034987	7,03E-02	1,922759408	2,18E-01	6,41E-01	8,70E-01	Hrh2	1,05	No
ENSMUSG00000034990	2,45E-01	-2,448630166	7,58E-02	7,83E-01	9,50E-01	Otoa	1,19	No
ENSMUSG00000034993	1,75E-01	9,104683819	9,46E+00	2,10E-03	2,91E-02	Vat1	1,13	Yes
ENSMUSG00000034994	-1,07E-01	10,90190522	3,67E+00	5,52E-02	2,88E-01	Eef2	0,93	No
ENSMUSG00000034997	-3,63E-02	1,809641811	4,73E-02	8,28E-01	9,76E-01	Htr2a	0,98	No
ENSMUSG00000034998	-4,41E-02	6,15819646	4,74E-01	4,91E-01	7,77E-01	Foxn2	0,97	No
ENSMUSG00000035000	-5,29E-01	5,576970089	6,03E+01	8,01E-15	1,20E-12	Dpp4	0,69	Yes
ENSMUSG00000035004	3,94E-02	6,174466439	3,76E-01	5,40E-01	8,10E-01	Igsf6	1,03	No
ENSMUSG00000035007	1,90E-01	4,651895768	5,87E+00	1,54E-02	1,28E-01	Rundc1	1,14	No
ENSMUSG00000035011	1,33E-01	6,858147962	4,73E+00	2,96E-02	1,97E-01	Zbtb7a	1,10	No
ENSMUSG00000035020	-5,88E-02	-3,033652696	1,62E-03	9,68E-01	1,00E+00	Epgn	0,96	No
ENSMUSG00000035021	1,12E-01	6,982026992	3,40E+00	6,52E-02	3,20E-01	Baz1a	1,08	No
ENSMUSG00000035024	2,06E-01	5,3680417	8,54E+00	3,47E-03	4,27E-02	Ncapd3	1,15	Yes
ENSMUSG00000035027	2,68E-02	6,34312444	1,78E-01	6,73E-01	8,90E-01	Map2k2	1,02	No
ENSMUSG00000035031	5,44E-01	0,070421226	3,37E+00	6,65E-02	3,24E-01	C8a	1,46	No
ENSMUSG00000035032	-3,70E-01	-2,088761642	2,45E-01	6,20E-01	8,57E-01	Nek11	0,77	No
ENSMUSG00000035033	-2,02E+00	-2,355535679	3,09E+00	7,88E-02	3,59E-01	Tbr1	0,25	No
ENSMUSG00000035041	1,54E-01	-1,088733677	1,08E-01	7,42E-01	9,27E-01	Creb3l3	1,11	No
ENSMUSG00000035042	-9,13E-01	7,39528083	2,32E+02	1,89E-52	5,67E-49	Ccl5	0,53	Yes
ENSMUSG00000035047	5,86E-02	4,775355975	5,88E-01	4,43E-01	7,35E-01	Kri1	1,04	No
ENSMUSG00000035048	-1,45E-01	3,632265202	2,24E+00	1,35E-01	4,62E-01	Anapc13	0,90	No

ENSMUSG00000035049	1,31E-01	4,456473354	2,53E+00	1,12E-01	4,11E-01	Rrp12	1,10	No
ENSMUSG00000035051	-1,57E-01	4,844999104	4,35E+00	3,70E-02	2,29E-01	Dhx57	0,90	No
ENSMUSG00000035064	1,06E-01	3,765605827	1,25E+00	2,64E-01	5,58E-01	Eef2k	1,08	No
ENSMUSG00000035067	-6,11E-02	-1,929295672	7,81E-03	9,30E-01	1,00E+00	Xkr6	0,96	No
ENSMUSG00000035069	1,64E-01	3,990912134	3,39E+00	6,57E-02	3,21E-01	Oma1	1,12	No
ENSMUSG00000035078	5,18E-02	5,832049127	6,07E-01	4,36E-01	7,29E-01	Mtmr9	1,04	No
ENSMUSG00000035085	-1,88E-01	-0,503683011	2,89E-01	5,91E-01	8,41E-01	1700020L24Rik	0,88	No
ENSMUSG00000035086	-1,01E-01	6,516130432	2,63E+00	1,05E-01	3,97E-01	Becn1	0,93	No
ENSMUSG00000035093	1,32E-01	7,073086342	4,73E+00	2,96E-02	1,96E-01	Secisbp2l	1,10	No
ENSMUSG00000035095	-3,62E-01	0,809244452	2,74E+00	9,77E-02	3,90E-01	Fam167a	0,78	No
ENSMUSG00000035104	-8,26E-01	-1,800802823	1,85E+00	1,74E-01	5,32E-01	Eva1a	0,56	No
ENSMUSG00000035105	6,43E-01	4,130056542	5,28E+01	3,73E-13	4,77E-11	Egln3	1,56	Yes
ENSMUSG00000035107	-1,55E-01	4,634219008	3,97E+00	4,62E-02	2,57E-01	Dcbld2	0,90	No
ENSMUSG00000035109	4,99E-02	3,453580756	2,45E-01	6,21E-01	8,57E-01	Shc4	1,04	No
ENSMUSG00000035112	-9,59E-01	-1,321998543	3,19E+00	7,43E-02	3,47E-01	Wnk4	0,51	No
ENSMUSG00000035121	-7,85E-03	1,661441149	2,12E-03	9,63E-01	1,00E+00	Neil2	0,99	No
ENSMUSG00000035125	2,18E-01	3,314850619	4,40E+00	3,60E-02	2,25E-01	Gcfc2	1,16	No
ENSMUSG00000035126	6,32E-01	0,865909025	8,35E+00	3,85E-03	4,60E-02	Wdr78	1,55	Yes
ENSMUSG00000035129	1,32E-01	4,154391298	2,38E+00	1,23E-01	4,38E-01	Gm6781	1,10	No
ENSMUSG00000035133	1,02E-01	5,53883311	2,21E+00	1,37E-01	4,67E-01	Arhgap5	1,07	No
ENSMUSG00000035139	-7,60E-03	4,952345435	1,04E-02	9,19E-01	1,00E+00	Secisbp2	0,99	No
ENSMUSG00000035142	-4,37E-02	0,752920064	3,78E-02	8,46E-01	9,87E-01	Nubpl	0,97	No
ENSMUSG00000035148	-5,21E-01	-0,997948305	1,26E+00	2,62E-01	5,56E-01	Gpr33	0,70	No
ENSMUSG00000035151	1,05E-01	4,683727623	1,84E+00	1,75E-01	5,33E-01	Elmod2	1,08	No
ENSMUSG00000035152	-5,62E-03	7,94467833	9,30E-03	9,23E-01	1,00E+00	Ap2b1	1,00	No
ENSMUSG00000035158	1,06E-01	6,391026083	2,81E+00	9,35E-02	3,83E-01	Mitf	1,08	No
ENSMUSG00000035161	4,23E-02	5,582539208	3,84E-01	5,35E-01	8,08E-01	Ints6	1,03	No
ENSMUSG00000035164	-1,78E-01	6,884818336	8,42E+00	3,71E-03	4,47E-02	Zc3h12c	0,88	Yes
ENSMUSG00000035165	7,91E-01	2,180623471	2,86E+01	8,83E-08	5,01E-06	Kcne3	1,73	Yes
ENSMUSG00000035168	-4,26E-02	4,71197395	3,02E-01	5,83E-01	8,38E-01	Tanc1	0,97	No
ENSMUSG00000035171	3,53E-02	4,09689904	1,63E-01	6,86E-01	8,97E-01	1110059E24Rik	1,02	No
ENSMUSG00000035172	3,10E-01	1,616972447	3,40E+00	6,54E-02	3,21E-01	Plekhh3	1,24	No

ENSMUSG00000035173	8,39E-02	5,465690776	1,46E+00	2,27E-01	5,50E-01	Ccdc186	1,06	No
ENSMUSG00000035179	1,09E+00	-0,831991161	4,86E+00	2,75E-02	1,87E-01	Ppp1r32	2,13	No
ENSMUSG00000035181	1,77E-01	6,233045752	7,61E+00	5,79E-03	6,24E-02	Heatr5a	1,13	No
ENSMUSG00000035183	6,38E-03	2,102548579	1,98E-03	9,65E-01	1,00E+00	Slc24a5	1,00	No
ENSMUSG00000035184	-1,45E-01	1,028244804	4,98E-01	4,80E-01	7,68E-01	Fam124a	0,90	No
ENSMUSG00000035186	-1,94E+00	-3,107619243	1,50E+00	2,21E-01	5,50E-01	Ubd	0,26	No
ENSMUSG00000035187	1,03E-01	-1,963335706	2,09E-02	8,85E-01	1,00E+00	Nkx6-1	1,07	No
ENSMUSG00000035191	-4,13E-02	-3,033659495	7,08E-04	9,79E-01	1,00E+00	Rfpl4	0,97	No
ENSMUSG00000035198	1,71E-01	4,746715552	4,92E+00	2,66E-02	1,83E-01	Tubg1	1,13	No
ENSMUSG00000035199	7,54E-03	7,515748926	1,62E-02	8,99E-01	1,00E+00	Arl6ip5	1,01	No
ENSMUSG00000035200	-7,14E-01	-2,355547957	8,67E-01	3,52E-01	6,53E-01	Chrnb4	0,61	No
ENSMUSG00000035202	-7,44E-01	9,008858113	1,66E+02	4,57E-38	4,72E-35	Lars2	0,60	Yes
ENSMUSG00000035203	1,03E-01	7,235560338	2,92E+00	8,76E-02	3,78E-01	Epn1	1,07	No
ENSMUSG00000035206	9,06E-02	4,811444671	1,43E+00	2,32E-01	5,50E-01	Sppl2b	1,06	No
ENSMUSG00000035208	-9,15E-02	3,734734455	9,81E-01	3,22E-01	6,22E-01	Slfn8	0,94	No
ENSMUSG00000035211	2,45E-01	-0,748393973	3,99E-01	5,28E-01	8,04E-01	Xrra1	1,19	No
ENSMUSG00000035212	-1,28E-02	7,437046034	4,62E-02	8,30E-01	9,77E-01	Leprot	0,99	No
ENSMUSG00000035215	-6,79E-02	1,989041177	1,99E-01	6,55E-01	8,79E-01	Lsm7	0,95	No
ENSMUSG00000035226	-1,89E+00	-3,1076441	1,42E+00	2,33E-01	5,50E-01	Rims4	0,27	No
ENSMUSG00000035227	1,83E-01	6,550022689	8,54E+00	3,48E-03	4,27E-02	Spcs2	1,14	Yes
ENSMUSG00000035228	-1,78E-01	-0,992148381	1,77E-01	6,74E-01	8,90E-01	Ccdc106	0,88	No
ENSMUSG00000035234	2,12E-01	2,751118929	3,15E+00	7,60E-02	3,52E-01	Abraxas1	1,16	No
ENSMUSG00000035235	-1,16E-01	3,033400094	1,08E+00	2,98E-01	5,94E-01	Trim13	0,92	No
ENSMUSG00000035236	-3,50E-01	4,593004345	1,98E+01	8,60E-06	2,94E-04	Scai	0,78	Yes
ENSMUSG00000035237	-7,81E-01	-0,289208086	5,24E+00	2,21E-02	1,61E-01	Lcat	0,58	No
ENSMUSG00000035239	-2,70E-01	1,192961098	2,12E+00	1,46E-01	4,83E-01	Neu3	0,83	No
ENSMUSG00000035242	-7,32E-02	6,753204131	1,41E+00	2,35E-01	5,50E-01	Oaz1	0,95	No
ENSMUSG00000035245	3,30E-02	4,742771987	1,83E-01	6,69E-01	8,87E-01	Eogt	1,02	No
ENSMUSG00000035247	3,06E-02	7,44816296	2,66E-01	6,06E-01	8,49E-01	Hectd1	1,02	No
ENSMUSG00000035248	-1,20E-02	6,996968095	3,92E-02	8,43E-01	9,85E-01	Zcchc6	0,99	No
ENSMUSG00000035258	-1,17E+00	1,244480749	4,13E+01	1,30E-10	1,21E-08	Abi3bp	0,44	Yes
ENSMUSG00000035262	-2,44E-01	-1,165783836	2,51E-01	6,16E-01	8,55E-01	Amh	0,84	No

ENSMUSG00000035266	4,76E-02	3,008384797	1,76E-01	6,75E-01	8,91E-01	Helq	1,03	No
ENSMUSG00000035268	-2,93E-03	4,637099732	1,39E-03	9,70E-01	1,00E+00	Pkig	1,00	No
ENSMUSG00000035270	-7,40E-01	-0,614106183	3,68E+00	5,51E-02	2,88E-01	Impg2	0,60	No
ENSMUSG00000035273	1,78E-02	5,542881188	6,69E-02	7,96E-01	9,57E-01	Hpse	1,01	No
ENSMUSG00000035274	-4,88E-01	0,615886368	4,44E+00	3,51E-02	2,22E-01	Tpbg	0,71	No
ENSMUSG00000035275	-4,34E-01	-0,770604666	1,15E+00	2,84E-01	5,81E-01	Raver2	0,74	No
ENSMUSG00000035278	-6,84E-02	4,905789959	8,33E-01	3,61E-01	6,63E-01	Plekhj1	0,95	No
ENSMUSG00000035279	3,22E-01	0,087374177	1,41E+00	2,35E-01	5,50E-01	Ssc5d	1,25	No
ENSMUSG00000035283	-6,36E-04	0,477157277	6,17E-06	9,98E-01	1,00E+00	Adrb1	1,00	No
ENSMUSG00000035284	2,42E-01	7,407092758	1,65E+01	4,92E-05	1,34E-03	Vps13c	1,18	Yes
ENSMUSG00000035285	2,44E-01	-0,479034218	4,92E-01	4,83E-01	7,70E-01	Nat14	1,18	No
ENSMUSG00000035293	2,17E-01	4,304521447	6,89E+00	8,67E-03	8,40E-02	G2e3	1,16	No
ENSMUSG00000035295	-1,14E-01	4,185024448	1,79E+00	1,81E-01	5,42E-01	Wdr38	0,92	No
ENSMUSG00000035296	-5,88E-02	-3,033652696	1,62E-03	9,68E-01	1,00E+00	Sgcg	0,96	No
ENSMUSG00000035297	-1,49E-01	5,21095542	4,33E+00	3,74E-02	2,30E-01	Cops4	0,90	No
ENSMUSG00000035298	-9,37E-01	-0,497467537	7,14E+00	7,53E-03	7,58E-02	Klhl35	0,52	No
ENSMUSG00000035305	-7,75E-02	-2,600634094	5,47E-03	9,41E-01	1,00E+00	Ror1	0,95	No
ENSMUSG00000035310	1,20E-01	5,093162233	2,71E+00	9,96E-02	3,92E-01	Lin54	1,09	No
ENSMUSG00000035311	9,71E-02	6,238670402	2,29E+00	1,31E-01	4,54E-01	Gnptab	1,07	No
ENSMUSG00000035314	2,63E-01	3,962435635	8,56E+00	3,44E-03	4,24E-02	Gdpd5	1,20	Yes
ENSMUSG00000035325	2,87E-02	7,021692514	2,23E-01	6,36E-01	8,67E-01	Sec31a	1,02	No
ENSMUSG00000035329	1,13E-01	5,628401898	2,74E+00	9,81E-02	3,91E-01	Fbxo33	1,08	No
ENSMUSG00000035337	-1,49E-01	3,427995587	2,20E+00	1,38E-01	4,69E-01	Uchl4	0,90	No
ENSMUSG00000035342	8,89E-02	3,630086337	8,57E-01	3,55E-01	6,56E-01	Lzts2	1,06	No
ENSMUSG00000035351	2,22E-01	2,660331434	3,23E+00	7,21E-02	3,40E-01	Nup37	1,17	No
ENSMUSG00000035352	-3,24E-02	0,819577629	1,83E-02	8,92E-01	1,00E+00	Ccl12	0,98	No
ENSMUSG00000035354	-3,21E-01	6,730148677	2,71E+01	1,98E-07	1,04E-05	Uvrag	0,80	Yes
ENSMUSG00000035355	-4,30E-01	-0,96321321	1,03E+00	3,10E-01	6,09E-01	Kcnh4	0,74	No
ENSMUSG00000035356	-1,15E-02	4,559031938	2,16E-02	8,83E-01	1,00E+00	Nfkbiz	0,99	No
ENSMUSG00000035357	7,58E-01	-2,655092762	3,11E-01	5,77E-01	8,35E-01	Pdzrn3	1,69	No
ENSMUSG00000035364	4,36E-01	-0,419256453	1,73E+00	1,89E-01	5,50E-01	4930524J08Rik	1,35	No
ENSMUSG00000035365	2,69E-01	3,580627168	7,74E+00	5,39E-03	5,91E-02	Parpbp	1,21	No

ENSMUSG00000035367	1,73E-01	4,349423255	4,40E+00	3,59E-02	2,25E-01	Rmi1	1,13	No
ENSMUSG00000035370	1,20E-01	4,65827064	2,35E+00	1,25E-01	4,43E-01	Adat3	1,09	No
ENSMUSG00000035372	7,54E-02	3,576666821	5,78E-01	4,47E-01	7,38E-01	1810055G02Rik	1,05	No
ENSMUSG00000035373	1,17E-01	4,516025572	1,97E+00	1,60E-01	5,09E-01	Ccl7	1,08	No
ENSMUSG00000035376	-1,59E-02	3,29387209	2,33E-02	8,79E-01	1,00E+00	Hacd2	0,99	No
ENSMUSG00000035378	2,04E-01	2,861063014	2,93E+00	8,68E-02	3,78E-01	Shq1	1,15	No
ENSMUSG00000035382	8,02E-02	5,772850864	1,43E+00	2,32E-01	5,50E-01	Pcsk7	1,06	No
ENSMUSG00000035383	1,66E-02	0,369019486	4,31E-03	9,48E-01	1,00E+00	Pmch	1,01	No
ENSMUSG00000035385	-1,37E-02	7,181353383	5,03E-02	8,23E-01	9,73E-01	Ccl2	0,99	No
ENSMUSG00000035390	7,72E-01	0,330517331	8,45E+00	3,66E-03	4,43E-02	Brsk1	1,71	Yes
ENSMUSG00000035392	2,00E-01	5,4423679	8,05E+00	4,54E-03	5,19E-02	Dennd1a	1,15	No
ENSMUSG00000035394	-7,05E-01	-1,146036109	1,88E+00	1,71E-01	5,27E-01	Cfap53	0,61	No
ENSMUSG00000035397	-1,07E-01	4,633694164	1,90E+00	1,68E-01	5,23E-01	Klf16	0,93	No
ENSMUSG00000035399	1,82E-01	4,723622877	5,53E+00	1,87E-02	1,45E-01	Oser1	1,13	No
ENSMUSG00000035401	-3,20E-02	5,550709015	2,20E-01	6,39E-01	8,69E-01	Emsy	0,98	No
ENSMUSG00000035403	-6,63E-02	-1,522642035	1,39E-02	9,06E-01	1,00E+00	Crb2	0,96	No
ENSMUSG00000035413	8,66E-01	-1,165762641	3,33E+00	6,80E-02	3,28E-01	Tmem98	1,82	No
ENSMUSG00000035429	-8,69E-01	-0,627509097	5,77E+00	1,63E-02	1,33E-01	Ptprh	0,55	No
ENSMUSG00000035435	3,40E-01	0,514655295	2,04E+00	1,53E-01	4,96E-01	Abca17	1,27	No
ENSMUSG00000035437	1,90E-02	5,482457648	7,53E-02	7,84E-01	9,51E-01	Rabgap1	1,01	No
ENSMUSG00000035439	-9,20E-02	6,648923482	2,21E+00	1,37E-01	4,67E-01	Haus8	0,94	No
ENSMUSG00000035441	2,46E-01	2,916628064	4,35E+00	3,69E-02	2,28E-01	Myo1d	1,19	No
ENSMUSG00000035443	4,63E-02	4,470843326	3,29E-01	5,66E-01	8,29E-01	Thyn1	1,03	No
ENSMUSG00000035448	-2,71E-01	-2,226308329	1,27E-01	7,22E-01	9,16E-01	Ccr3	0,83	No
ENSMUSG00000035451	1,87E+00	-3,107639691	1,38E+00	2,40E-01	5,50E-01	Foxa1	3,67	No
ENSMUSG00000035455	3,76E-01	4,446741243	2,15E+01	3,56E-06	1,36E-04	Fignl1	1,30	Yes
ENSMUSG00000035456	-9,84E-01	0,08358014	1,18E+01	6,07E-04	1,08E-02	Prdm8	0,51	Yes
ENSMUSG00000035458	-4,52E-01	3,120880419	1,80E+01	2,21E-05	6,81E-04	Tnni3	0,73	Yes
ENSMUSG00000035459	-1,16E-01	-0,946280318	7,86E-02	7,79E-01	9,48E-01	Stab2	0,92	No
ENSMUSG00000035469	7,95E-02	5,171684804	1,21E+00	2,71E-01	5,67E-01	Rcbtb1	1,06	No
ENSMUSG00000035472	3,14E-02	-3,107617333	-7,69E-10	1,00E+00	1,00E+00	Slc25a21	1,02	No
ENSMUSG00000035473	-2,23E-01	5,829774698	1,14E+01	7,46E-04	1,27E-02	Galm	0,86	Yes

ENSMUSG00000035478	7,71E-02	5,950013753	1,37E+00	2,41E-01	5,50E-01	Mbd3	1,05	No
ENSMUSG00000035486	-1,88E+00	-2,896101069	1,42E+00	2,33E-01	5,50E-01	Plk5	0,27	No
ENSMUSG00000035493	1,83E-01	9,284523051	1,04E+01	1,23E-03	1,91E-02	Tgfbi	1,14	Yes
ENSMUSG00000035495	3,65E-02	5,020806806	2,48E-01	6,18E-01	8,56E-01	Tstd2	1,03	No
ENSMUSG00000035498	-1,51E+00	1,13889621	5,79E+01	2,71E-14	3,88E-12	Cdcp1	0,35	Yes
ENSMUSG00000035504	1,89E-01	-0,614078655	2,37E-01	6,26E-01	8,60E-01	Reep6	1,14	No
ENSMUSG00000035505	6,69E-02	3,110832381	3,70E-01	5,43E-01	8,12E-01	Cox18	1,05	No
ENSMUSG00000035506	-2,00E-02	-2,89610696	2,29E-04	9,88E-01	1,00E+00	Slc12a8	0,99	No
ENSMUSG00000035509	6,25E-01	-1,311324772	1,73E+00	1,88E-01	5,50E-01	Fbxl21	1,54	No
ENSMUSG00000035513	-3,53E-01	4,96234191	2,29E+01	1,73E-06	7,17E-05	Ntng2	0,78	Yes
ENSMUSG00000035517	-3,77E-02	5,545010587	3,00E-01	5,84E-01	8,38E-01	Tdrd7	0,97	No
ENSMUSG00000035521	-8,68E-02	3,201303572	6,59E-01	4,17E-01	7,12E-01	Gnptg	0,94	No
ENSMUSG00000035529	1,11E-01	5,351488471	2,46E+00	1,17E-01	4,23E-01	Prdm4	1,08	No
ENSMUSG00000035530	-1,76E-01	7,3831122	8,71E+00	3,16E-03	3,98E-02	Eif1	0,89	Yes
ENSMUSG00000035539	-4,74E-01	0,302698133	2,96E+00	8,53E-02	3,75E-01	Ccdc180	0,72	No
ENSMUSG00000035545	-6,26E-02	7,590049027	1,13E+00	2,89E-01	5,84E-01	Leng8	0,96	No
ENSMUSG00000035547	1,05E-01	3,516915687	1,09E+00	2,95E-01	5,91E-01	Capn5	1,08	No
ENSMUSG00000035551	1,21E-02	-3,033650469	7,34E-11	1,00E+00	1,00E+00	Igfbpl1	1,01	No
ENSMUSG00000035557	-1,89E+00	-3,1076441	1,42E+00	2,33E-01	5,50E-01	Krt17	0,27	No
ENSMUSG00000035559	-2,29E-02	4,710412524	8,65E-02	7,69E-01	9,41E-01	Mpv17l2	0,98	No
ENSMUSG00000035560	-5,50E-01	-1,226139804	1,21E+00	2,72E-01	5,69E-01	Wdr20rt	0,68	No
ENSMUSG00000035561	1,41E-01	5,484903476	4,14E+00	4,19E-02	2,43E-01	Aldh1b1	1,10	No
ENSMUSG00000035566	4,72E-01	-2,770639385	1,75E-01	6,76E-01	8,91E-01	Pcdh17	1,39	No
ENSMUSG00000035569	-2,67E-03	7,984621793	2,10E-03	9,63E-01	1,00E+00	Ankrd11	1,00	No
ENSMUSG00000035572	-4,32E-03	5,28282949	3,71E-03	9,51E-01	1,00E+00	Dcaf10	1,00	No
ENSMUSG00000035575	6,60E-02	5,329396665	8,78E-01	3,49E-01	6,50E-01	Utp6	1,05	No
ENSMUSG00000035576	-2,27E+00	-2,448622978	4,20E+00	4,05E-02	2,38E-01	L3mbtl1	0,21	No
ENSMUSG00000035578	1,72E-01	0,288450156	4,25E-01	5,15E-01	7,96E-01	Iqcg	1,13	No
ENSMUSG00000035585	1,11E-02	4,71592884	2,08E-02	8,85E-01	1,00E+00	Tsen34	1,01	No
ENSMUSG00000035594	-1,24E+00	-2,548191728	1,47E+00	2,26E-01	5,50E-01	Chrna5	0,42	No
ENSMUSG00000035595	-3,33E-03	2,619477163	7,11E-04	9,79E-01	1,00E+00	1600002K03Rik	1,00	No
ENSMUSG00000035596	-2,53E-02	6,039531273	1,51E-01	6,98E-01	9,04E-01	Mboat7	0,98	No

ENSMUSG00000035597	-3,48E-02	6,28626817	2,96E-01	5,86E-01	8,39E-01	Prpf39	0,98	No
ENSMUSG00000035601	1,84E-01	2,326267155	1,79E+00	1,81E-01	5,42E-01	Trmt10b	1,14	No
ENSMUSG00000035606	-3,19E-01	0,569445423	1,90E+00	1,68E-01	5,23E-01	Ky	0,80	No
ENSMUSG00000035614	-9,48E-02	5,357659967	1,84E+00	1,75E-01	5,33E-01	Togaram1	0,94	No
ENSMUSG00000035615	-4,43E-01	-2,548168849	2,01E-01	6,54E-01	8,79E-01	Frmpd1	0,74	No
ENSMUSG00000035620	7,85E-02	4,11009766	8,21E-01	3,65E-01	6,66E-01	Ric8b	1,06	No
ENSMUSG00000035621	8,56E-02	5,971206316	1,72E+00	1,89E-01	5,50E-01	Midn	1,06	No
ENSMUSG00000035623	7,06E-03	5,438696857	1,04E-02	9,19E-01	1,00E+00	Rsf1	1,00	No
ENSMUSG00000035629	-7,29E-02	5,688641158	1,18E+00	2,77E-01	5,73E-01	Rubcn	0,95	No
ENSMUSG00000035632	3,02E-02	5,060351878	1,71E-01	6,79E-01	8,93E-01	Cnot3	1,02	No
ENSMUSG00000035637	4,01E-01	3,066795772	1,26E+01	3,96E-04	7,57E-03	Grhpr	1,32	Yes
ENSMUSG00000035640	-2,69E-01	3,196694941	6,47E+00	1,10E-02	9,98E-02	Cbarp	0,83	No
ENSMUSG00000035642	-1,54E-01	2,089838864	1,06E+00	3,03E-01	6,00E-01	Aamdc	0,90	No
ENSMUSG00000035649	-3,09E-02	5,174260813	1,85E-01	6,68E-01	8,86E-01	Zcchc7	0,98	No
ENSMUSG00000035666	2,24E-01	5,338580533	1,02E+01	1,41E-03	2,13E-02	Gtf3c4	1,17	Yes
ENSMUSG00000035671	-1,94E-01	5,844691726	8,42E+00	3,71E-03	4,48E-02	Zswim4	0,87	Yes
ENSMUSG00000035673	-4,05E-02	7,283827206	4,60E-01	4,97E-01	7,83E-01	Sbno2	0,97	No
ENSMUSG00000035674	-1,36E-01	4,293792511	2,68E+00	1,02E-01	3,94E-01	Ndufa3	0,91	No
ENSMUSG00000035678	1,95E-01	2,439712445	2,27E+00	1,32E-01	4,56E-01	Tnfsf9	1,14	No
ENSMUSG00000035683	4,48E-02	4,541463744	3,18E-01	5,73E-01	8,32E-01	Melk	1,03	No
ENSMUSG00000035686	1,82E+00	-3,033661385	1,31E+00	2,52E-01	5,50E-01	Thrsp	3,54	No
ENSMUSG00000035692	-2,50E-01	2,697680647	4,36E+00	3,69E-02	2,28E-01	Isg15	0,84	No
ENSMUSG00000035696	5,47E-02	6,567806344	7,72E-01	3,80E-01	6,81E-01	Rnf38	1,04	No
ENSMUSG00000035697	-6,52E-02	8,570022773	1,30E+00	2,55E-01	5,50E-01	Arhgap45	0,96	No
ENSMUSG00000035699	1,34E-01	-2,185622287	3,11E-02	8,60E-01	9,95E-01	Slc51a	1,10	No
ENSMUSG00000035704	2,29E-01	4,208427867	7,35E+00	6,71E-03	6,97E-02	Alg8	1,17	No
ENSMUSG00000035711	-2,92E-02	6,279012395	2,08E-01	6,48E-01	8,75E-01	Dok3	0,98	No
ENSMUSG00000035713	-3,93E-01	4,081368201	2,10E+01	4,63E-06	1,69E-04	Usp35	0,76	Yes
ENSMUSG00000035722	-1,07E-01	7,391885472	3,24E+00	7,17E-02	3,40E-01	Abca7	0,93	No
ENSMUSG00000035726	3,53E-04	6,426083549	3,13E-05	9,96E-01	1,00E+00	Supt16	1,00	No
ENSMUSG00000035735	3,42E-01	3,502445174	1,17E+01	6,26E-04	1,11E-02	Dagla	1,27	Yes
ENSMUSG00000035745	-8,01E-02	1,189610133	1,71E-01	6,79E-01	8,93E-01	Grin3b	0,95	No

ENSMUSG00000035754	2,16E-01	5,185736451	8,90E+00	2,85E-03	3,68E-02	Wdr18	1,16	Yes
ENSMUSG00000035757	-1,18E-02	4,981679364	2,55E-02	8,73E-01	1,00E+00	Selenoo	0,99	No
ENSMUSG00000035759	-4,48E-02	2,18897343	1,01E-01	7,50E-01	9,31E-01	Bbs10	0,97	No
ENSMUSG00000035762	6,30E-02	4,428986478	5,88E-01	4,43E-01	7,35E-01	Tmem161b	1,04	No
ENSMUSG00000035764	5,81E-02	5,176103752	6,49E-01	4,20E-01	7,16E-01	Fbxo45	1,04	No
ENSMUSG00000035765	8,22E-02	5,038238345	1,24E+00	2,65E-01	5,60E-01	Dym	1,06	No
ENSMUSG00000035769	4,35E-01	2,905862404	1,38E+01	1,99E-04	4,33E-03	Xylb	1,35	Yes
ENSMUSG00000035770	2,41E-01	6,145419688	1,40E+01	1,86E-04	4,09E-03	Dync1li2	1,18	Yes
ENSMUSG00000035772	8,83E-02	5,226526821	1,50E+00	2,21E-01	5,50E-01	Mrps2	1,06	No
ENSMUSG00000035773	-1,51E-01	-0,50377926	1,99E-01	6,56E-01	8,80E-01	Kiss1r	0,90	No
ENSMUSG00000035778	-3,54E-01	7,184400228	3,48E+01	3,57E-09	2,68E-07	Ggta1	0,78	Yes
ENSMUSG00000035781	-1,08E-01	5,84893528	2,65E+00	1,03E-01	3,95E-01	R3hdm4	0,93	No
ENSMUSG00000035783	3,70E-01	1,870529418	5,51E+00	1,89E-02	1,46E-01	Acta2	1,29	No
ENSMUSG00000035785	-8,45E-01	-2,963229433	3,92E-01	5,31E-01	8,05E-01	Cmtm2b	0,56	No
ENSMUSG00000035790	1,66E-01	3,892831836	3,32E+00	6,85E-02	3,29E-01	Cep19	1,12	No
ENSMUSG00000035798	8,10E-02	4,548798933	1,03E+00	3,10E-01	6,09E-01	Zdhhc17	1,06	No
ENSMUSG00000035799	-1,33E+00	-2,8320094	1,11E+00	2,92E-01	5,87E-01	Twist1	0,40	No
ENSMUSG00000035804	9,01E-03	-3,107646452	-5,16E-10	1,00E+00	1,00E+00	Ins1	1,01	No
ENSMUSG00000035818	-6,83E-01	0,586911759	8,74E+00	3,11E-03	3,94E-02	Plekhs1	0,62	Yes
ENSMUSG00000035824	-1,46E-02	4,554713824	3,40E-02	8,54E-01	9,92E-01	Tk2	0,99	No
ENSMUSG00000035828	-4,12E-01	5,06667014	3,13E+01	2,19E-08	1,41E-06	Pim3	0,75	Yes
ENSMUSG00000035829	4,72E-01	0,952299909	5,18E+00	2,28E-02	1,64E-01	Ppp1r26	1,39	No
ENSMUSG00000035834	1,53E-01	3,282180622	2,09E+00	1,48E-01	4,87E-01	Polr3g	1,11	No
ENSMUSG00000035835	4,66E-02	3,774551595	2,49E-01	6,17E-01	8,55E-01	Plppr3	1,03	No
ENSMUSG00000035840	1,39E-01	3,152783247	1,65E+00	1,99E-01	5,50E-01	Lysmd3	1,10	No
ENSMUSG00000035842	1,90E-01	3,712045471	4,09E+00	4,31E-02	2,47E-01	Ddx11	1,14	No
ENSMUSG00000035845	1,74E-01	3,746188542	3,49E+00	6,17E-02	3,09E-01	Alg12	1,13	No
ENSMUSG00000035849	-1,15E+00	-0,980534408	7,05E+00	7,91E-03	7,83E-02	Krt222	0,45	No
ENSMUSG00000035851	-7,90E-02	6,226784996	1,51E+00	2,18E-01	5,50E-01	Ythdc1	0,95	No
ENSMUSG00000035852	6,31E-02	-1,896283452	9,72E-03	9,21E-01	1,00E+00	Misp	1,04	No
ENSMUSG00000035860	-1,11E+00	0,672787201	2,52E+01	5,05E-07	2,40E-05	Cdhr3	0,46	Yes
ENSMUSG00000035863	-2,14E-01	4,209095302	6,29E+00	1,22E-02	1,08E-01	Palm	0,86	No

ENSMUSG00000035864	-9,36E-01	-1,896163663	2,45E+00	1,18E-01	4,26E-01	Syt1	0,52	No
ENSMUSG00000035868	-1,25E-01	2,534361026	1,00E+00	3,16E-01	6,17E-01	Zfp983	0,92	No
ENSMUSG00000035873	2,90E-01	-2,448692547	8,55E-02	7,70E-01	9,42E-01	Pawr	1,22	No
ENSMUSG00000035875	-8,03E-02	-0,816516185	4,21E-02	8,37E-01	9,82E-01	AI182371	0,95	No
ENSMUSG00000035877	1,90E-01	3,719475297	4,10E+00	4,30E-02	2,47E-01	Zhx3	1,14	No
ENSMUSG00000035878	2,34E-01	-2,355630815	6,87E-02	7,93E-01	9,56E-01	Hykk	1,18	No
ENSMUSG00000035885	-6,50E-02	7,490948946	1,20E+00	2,73E-01	5,69E-01	Cox8a	0,96	No
ENSMUSG00000035890	1,28E-01	5,036133805	3,01E+00	8,29E-02	3,70E-01	Rnf126	1,09	No
ENSMUSG00000035891	1,66E-01	7,487893995	7,85E+00	5,08E-03	5,65E-02	Cerk	1,12	No
ENSMUSG00000035896	-3,18E+00	-2,963258684	4,26E+00	3,90E-02	2,34E-01	Rnase1	0,11	No
ENSMUSG00000035898	5,08E-02	4,716872714	4,33E-01	5,10E-01	7,94E-01	Uba6	1,04	No
ENSMUSG00000035900	5,22E-03	6,627749184	7,11E-03	9,33E-01	1,00E+00	Gramd4	1,00	No
ENSMUSG00000035901	-2,38E-01	7,654426019	1,63E+01	5,33E-05	1,43E-03	Dennd5a	0,85	Yes
ENSMUSG00000035914	2,57E-01	2,833241544	4,62E+00	3,16E-02	2,06E-01	Cd276	1,20	No
ENSMUSG00000035919	-9,19E-04	4,074161009	1,11E-04	9,92E-01	1,00E+00	Bbs9	1,00	No
ENSMUSG00000035929	-3,59E-01	6,444079503	3,26E+01	1,11E-08	7,67E-07	H2-Q4	0,78	Yes
ENSMUSG00000035932	-2,63E-02	-3,033653157	2,33E-04	9,88E-01	1,00E+00	Olfr750	0,98	No
ENSMUSG00000035933	1,18E-02	5,768611638	3,10E-02	8,60E-01	9,95E-01	Cog5	1,01	No
ENSMUSG00000035934	4,82E-01	1,866822596	8,67E+00	3,23E-03	4,05E-02	Pknox2	1,40	Yes
ENSMUSG00000035936	-1,66E-01	-0,690143155	1,94E-01	6,60E-01	8,82E-01	Aldh5a1	0,89	No
ENSMUSG00000035941	1,60E-01	6,139696294	6,11E+00	1,34E-02	1,16E-01	Ibtk	1,12	No
ENSMUSG00000035944	-4,94E-02	3,890161448	2,95E-01	5,87E-01	8,40E-01	Ttc38	0,97	No
ENSMUSG00000035946	-1,89E+00	-3,1076441	1,42E+00	2,33E-01	5,50E-01	Gsx2	0,27	No
ENSMUSG00000035948	3,14E-02	-3,107617333	-7,69E-10	1,00E+00	1,00E+00	Acss3	1,02	No
ENSMUSG00000035949	-1,51E-01	6,130910425	5,41E+00	2,00E-02	1,51E-01	Fbxw2	0,90	No
ENSMUSG00000035951	1,91E+00	-2,655148386	2,72E+00	9,92E-02	3,92E-01	Ascl3	3,76	No
ENSMUSG00000035953	5,64E-02	6,685724855	8,32E-01	3,62E-01	6,63E-01	Tmem55b	1,04	No
ENSMUSG00000035954	2,78E-01	5,071197337	1,42E+01	1,65E-04	3,68E-03	Dock4	1,21	Yes
ENSMUSG00000035958	5,56E-02	4,457490563	4,64E-01	4,96E-01	7,81E-01	Tdp2	1,04	No
ENSMUSG00000035960	4,00E-02	5,117372683	3,01E-01	5,83E-01	8,38E-01	Apex1	1,03	No
ENSMUSG00000035969	2,44E-01	6,033400708	1,38E+01	2,00E-04	4,34E-03	Rusc2	1,18	Yes
ENSMUSG00000035983	-6,20E-02	3,07971513	3,21E-01	5,71E-01	8,31E-01	Gm7008	0,96	No

ENSMUSG00000035984	2,57E+00	-2,963293044	2,56E+00	1,10E-01	4,06E-01	Nme5	5,93	No
ENSMUSG00000035992	-8,85E-04	6,748905577	2,05E-04	9,89E-01	1,00E+00	Fnip1	1,00	No
ENSMUSG00000036002	1,06E-01	5,77267373	2,52E+00	1,12E-01	4,13E-01	Fam214b	1,08	No
ENSMUSG00000036006	-3,37E-01	0,46460144	1,93E+00	1,65E-01	5,18E-01	Ripor2	0,79	No
ENSMUSG00000036009	2,35E-01	2,271598146	2,75E+00	9,70E-02	3,89E-01	Mettl25	1,18	No
ENSMUSG00000036019	-3,19E-01	3,817119986	1,24E+01	4,35E-04	8,16E-03	Tmtc2	0,80	Yes
ENSMUSG00000036023	1,09E-01	4,778256773	2,01E+00	1,57E-01	5,01E-01	Parp2	1,08	No
ENSMUSG00000036026	-7,43E-02	4,751273234	9,27E-01	3,36E-01	6,37E-01	Tmem63b	0,95	No
ENSMUSG00000036036	7,81E-01	-1,267998559	2,26E+00	1,33E-01	4,59E-01	Zfp57	1,72	No
ENSMUSG00000036040	1,85E+00	-3,107657832	1,35E+00	2,46E-01	5,50E-01	Adamtsl2	3,61	No
ENSMUSG00000036045	4,27E-04	1,724164142	6,53E-06	9,98E-01	1,00E+00	Gm12221	1,00	No
ENSMUSG00000036046	6,37E-02	8,383171778	1,22E+00	2,69E-01	5,65E-01	5031439G07Rik	1,05	No
ENSMUSG00000036052	1,60E-01	3,591094035	2,70E+00	1,00E-01	3,92E-01	Dnajb5	1,12	No
ENSMUSG00000036053	-2,88E-01	5,809718137	1,90E+01	1,34E-05	4,35E-04	Fmnl2	0,82	Yes
ENSMUSG00000036054	9,89E-02	5,13564887	1,88E+00	1,70E-01	5,26E-01	Sugp2	1,07	No
ENSMUSG00000036057	6,95E-03	6,324609078	1,20E-02	9,13E-01	1,00E+00	Ptpn23	1,00	No
ENSMUSG00000036061	-3,23E-02	2,587363278	7,04E-02	7,91E-01	9,55E-01	Smug1	0,98	No
ENSMUSG00000036062	-9,78E-01	2,99731785	7,40E+01	7,66E-18	1,51E-15	Phf24	0,51	Yes
ENSMUSG00000036067	-3,01E-01	7,288581903	2,54E+01	4,73E-07	2,26E-05	Slc2a6	0,81	Yes
ENSMUSG00000036073	4,26E-02	3,972099441	2,26E-01	6,34E-01	8,66E-01	Galt	1,03	No
ENSMUSG00000036078	2,54E-01	5,922821387	1,46E+01	1,31E-04	3,04E-03	Sigmar1	1,19	Yes
ENSMUSG00000036086	3,98E-01	6,086846654	3,73E+01	1,01E-09	8,27E-08	Zranb3	1,32	Yes
ENSMUSG00000036087	-8,00E-02	6,273469375	1,57E+00	2,10E-01	5,50E-01	Slain2	0,95	No
ENSMUSG00000036091	-1,66E-01	-0,077684653	2,90E-01	5,90E-01	8,41E-01	Hyal3	0,89	No
ENSMUSG00000036093	-2,85E-01	7,711445438	2,36E+01	1,22E-06	5,28E-05	Arl5a	0,82	Yes
ENSMUSG00000036097	1,64E-01	5,740613381	5,95E+00	1,47E-02	1,24E-01	Slf2	1,12	No
ENSMUSG00000036098	2,26E-01	6,537932735	1,30E+01	3,08E-04	6,17E-03	Myrf	1,17	Yes
ENSMUSG00000036099	1,53E-01	4,893827387	4,16E+00	4,13E-02	2,41E-01	Vezt	1,11	No
ENSMUSG00000036103	1,79E-01	3,694031192	3,61E+00	5,73E-02	2,95E-01	Colec12	1,13	No
ENSMUSG00000036104	1,35E-01	5,204874378	3,50E+00	6,14E-02	3,08E-01	Rab3gap1	1,10	No
ENSMUSG00000036106	-1,44E-01	2,203382136	1,08E+00	2,99E-01	5,95E-01	Prr5	0,90	No
ENSMUSG00000036110	9,01E-03	-3,107646452	-5,16E-10	1,00E+00	1,00E+00	Slc17a2	1,01	No

ENSMUSG00000036111	6,83E-01	2,35749316	2,50E+01	5,75E-07	2,70E-05	Lmo1	1,61	Yes
ENSMUSG00000036112	3,63E-02	6,541735785	3,36E-01	5,62E-01	8,25E-01	Metap2	1,03	No
ENSMUSG00000036114	1,61E-01	3,439109779	2,52E+00	1,13E-01	4,13E-01	Rpp25l	1,12	No
ENSMUSG00000036117	2,27E+00	-3,033667073	1,97E+00	1,60E-01	5,09E-01	Il5	4,82	No
ENSMUSG00000036120	-3,34E-02	4,572042629	1,77E-01	6,74E-01	8,90E-01	Rfxank	0,98	No
ENSMUSG00000036123	-8,05E-01	-1,998004314	1,43E+00	2,31E-01	5,50E-01	Slc9a3	0,57	No
ENSMUSG00000036136	-5,39E-01	-0,697221389	1,91E+00	1,67E-01	5,20E-01	Fam110c	0,69	No
ENSMUSG00000036138	3,17E-02	7,017815516	2,73E-01	6,01E-01	8,47E-01	Acaa1a	1,02	No
ENSMUSG00000036151	-6,16E-02	-2,226285992	7,03E-03	9,33E-01	1,00E+00	Tm6sf2	0,96	No
ENSMUSG00000036155	2,16E-01	5,657598403	1,00E+01	1,56E-03	2,30E-02	Mgat5	1,16	Yes
ENSMUSG00000036158	-3,25E-01	1,024087739	2,62E+00	1,05E-01	3,98E-01	Prickle1	0,80	No
ENSMUSG00000036160	1,16E-01	4,634571409	2,22E+00	1,37E-01	4,66E-01	Surf6	1,08	No
ENSMUSG00000036162	-2,27E-02	2,492790664	3,08E-02	8,61E-01	9,96E-01	Fam219aos	0,98	No
ENSMUSG00000036167	-3,03E-02	6,127388659	2,20E-01	6,39E-01	8,69E-01	Pphln1	0,98	No
ENSMUSG00000036168	3,16E-01	0,726683029	1,81E+00	1,79E-01	5,39E-01	Ccdc38	1,25	No
ENSMUSG00000036172	6,00E-01	3,473164103	3,49E+01	3,55E-09	2,68E-07	Cd200r3	1,52	Yes
ENSMUSG00000036180	1,09E-01	6,949096273	3,21E+00	7,33E-02	3,44E-01	Gatad2a	1,08	No
ENSMUSG00000036181	-1,93E-01	8,047057726	1,09E+01	9,43E-04	1,54E-02	Hist1h1c	0,87	Yes
ENSMUSG00000036185	5,66E-01	-1,912573735	6,36E-01	4,25E-01	7,19E-01	Sapcd1	1,48	No
ENSMUSG00000036186	5,83E-03	-0,424953718	2,81E-04	9,87E-01	1,00E+00	Fam69b	1,00	No
ENSMUSG00000036188	1,90E-01	4,263900554	5,15E+00	2,32E-02	1,67E-01	Ankmy2	1,14	No
ENSMUSG00000036196	6,13E-01	-2,448673268	4,20E-01	5,17E-01	7,98E-01	Slc26a8	1,53	No
ENSMUSG00000036197	6,67E-02	6,009278468	1,04E+00	3,08E-01	6,06E-01	Gxylt1	1,05	No
ENSMUSG00000036199	-5,63E-02	5,727582024	7,00E-01	4,03E-01	7,01E-01	Ndufa13	0,96	No
ENSMUSG00000036202	1,54E-01	5,343440466	4,76E+00	2,91E-02	1,94E-01	Rif1	1,11	No
ENSMUSG00000036206	-6,39E-01	5,741273012	9,15E+01	1,14E-21	3,07E-19	Sh3bp4	0,64	Yes
ENSMUSG00000036208	1,52E-02	4,826016235	3,99E-02	8,42E-01	9,84E-01	Nepro	1,01	No
ENSMUSG00000036211	-2,28E+00	-2,497495997	4,25E+00	3,94E-02	2,35E-01	Hist1h1t	0,21	No
ENSMUSG00000036214	8,97E-02	0,028134696	9,96E-02	7,52E-01	9,32E-01	Znrd1as	1,06	No
ENSMUSG00000036216	-7,61E-02	-2,963237265	2,63E-03	9,59E-01	1,00E+00	Leap2	0,95	No
ENSMUSG00000036218	-2,74E+00	-3,033596063	2,99E+00	8,38E-02	3,71E-01	Pdzrn4	0,15	No
ENSMUSG00000036223	2,21E-01	2,739303629	3,18E+00	7,46E-02	3,47E-01	Ska1	1,17	No

ENSMUSG00000036225	1,31E-01	1,22544944	4,60E-01	4,98E-01	7,83E-01	Kctd1	1,10	No
ENSMUSG00000036231	9,01E-03	-3,107646452	-5,16E-10	1,00E+00	1,00E+00	Agr3	1,01	No
ENSMUSG00000036241	-1,51E-01	6,583690096	5,85E+00	1,56E-02	1,29E-01	Ube2r2	0,90	No
ENSMUSG00000036246	4,02E-02	6,913151754	4,34E-01	5,10E-01	7,93E-01	Gmip	1,03	No
ENSMUSG00000036249	1,61E-01	4,628979454	4,17E+00	4,11E-02	2,41E-01	Rbm43	1,12	No
ENSMUSG00000036251	1,22E+00	-3,107637583	6,82E-01	4,09E-01	7,05E-01	Trpm8	2,33	No
ENSMUSG00000036256	-4,81E-02	-0,241703713	2,32E-02	8,79E-01	1,00E+00	Igfbp7	0,97	No
ENSMUSG00000036257	-1,18E-01	6,20681708	3,38E+00	6,62E-02	3,23E-01	Pnpla8	0,92	No
ENSMUSG00000036264	-4,13E-01	0,002077608	1,86E+00	1,73E-01	5,30E-01	Fstl4	0,75	No
ENSMUSG00000036270	-4,44E-02	6,206380032	4,78E-01	4,90E-01	7,76E-01	Edc4	0,97	No
ENSMUSG00000036273	1,55E-01	5,803330575	5,43E+00	1,98E-02	1,50E-01	Lrrk2	1,11	No
ENSMUSG00000036275	-2,15E-02	7,438958533	1,31E-01	7,18E-01	9,14E-01	9530068E07Rik	0,99	No
ENSMUSG00000036278	-9,05E-02	2,221702992	4,07E-01	5,23E-01	8,01E-01	Macrod1	0,94	No
ENSMUSG00000036281	2,42E-01	4,59527058	9,33E+00	2,26E-03	3,08E-02	Snapc4	1,18	Yes
ENSMUSG00000036282	-4,24E-03	4,554921707	2,89E-03	9,57E-01	1,00E+00	Naa30	1,00	No
ENSMUSG00000036285	1,79E-01	3,95951227	4,05E+00	4,41E-02	2,50E-01	Noa1	1,13	No
ENSMUSG00000036291	7,30E-02	4,36419067	7,93E-01	3,73E-01	6,74E-01	Ap5m1	1,05	No
ENSMUSG00000036292	4,22E-02	0,804501506	3,80E-02	8,46E-01	9,87E-01	Gramd1c	1,03	No
ENSMUSG00000036295	1,23E+00	-2,548267169	9,43E-01	3,31E-01	6,32E-01	Lrrn3	2,35	No
ENSMUSG00000036298	-1,72E+00	-2,770605246	2,06E+00	1,51E-01	4,93E-01	Slc2a13	0,30	No
ENSMUSG00000036299	-4,70E-02	5,813376895	4,93E-01	4,83E-01	7,70E-01	BC031181	0,97	No
ENSMUSG00000036304	4,90E-01	-0,574148784	1,62E+00	2,03E-01	5,50E-01	Zdhhc23	1,40	No
ENSMUSG00000036305	-2,51E-01	8,292113827	1,89E+01	1,40E-05	4,52E-04	Rpl39-ps	0,84	Yes
ENSMUSG00000036306	3,46E-01	0,752940535	2,19E+00	1,39E-01	4,71E-01	Lzts1	1,27	No
ENSMUSG00000036309	-4,01E-02	5,802124126	3,60E-01	5,48E-01	8,16E-01	Skp1a	0,97	No
ENSMUSG00000036315	1,11E-01	3,272123731	1,09E+00	2,96E-01	5,92E-01	Znrd1	1,08	No
ENSMUSG00000036322	2,21E-01	-2,311205448	6,13E-02	8,04E-01	9,63E-01	H2-Ea-ps	1,17	No
ENSMUSG00000036323	-2,84E-02	6,93212837	2,17E-01	6,41E-01	8,71E-01	Srp72	0,98	No
ENSMUSG00000036327	2,51E-01	3,372954248	6,08E+00	1,37E-02	1,18E-01	Qsox2	1,19	No
ENSMUSG00000036330	-1,36E-01	2,507047204	1,14E+00	2,85E-01	5,81E-01	Slc18a1	0,91	No
ENSMUSG00000036333	-3,23E-02	7,722772398	3,03E-01	5,82E-01	8,38E-01	Kidins220	0,98	No
ENSMUSG00000036334	-2,55E-01	-0,484991397	5,30E-01	4,67E-01	7,55E-01	Igsf10	0,84	No

ENSMUSG00000036339	1,42E-01	5,201631263	3,89E+00	4,85E-02	2,65E-01	Tmem260	1,10	No
ENSMUSG00000036352	-7,96E-02	4,762279109	1,08E+00	2,99E-01	5,95E-01	Ubac1	0,95	No
ENSMUSG00000036353	1,62E-02	3,117154647	2,16E-02	8,83E-01	1,00E+00	P2ry12	1,01	No
ENSMUSG00000036356	-1,08E+00	1,164396554	3,15E+01	2,04E-08	1,33E-06	Csgalnact1	0,47	Yes
ENSMUSG00000036362	-1,06E-01	4,873543448	1,99E+00	1,59E-01	5,06E-01	P2ry13	0,93	No
ENSMUSG00000036368	-2,85E-01	1,844647622	3,46E+00	6,29E-02	3,13E-01	Rmdn2	0,82	No
ENSMUSG00000036371	-5,60E-03	7,967867603	9,25E-03	9,23E-01	1,00E+00	Serbp1	1,00	No
ENSMUSG00000036372	-2,97E-02	4,996843601	1,62E-01	6,87E-01	8,98E-01	Tmem258	0,98	No
ENSMUSG00000036376	2,34E-02	3,956811566	6,74E-02	7,95E-01	9,57E-01	Abt1	1,02	No
ENSMUSG00000036377	-8,35E-01	-0,740673293	4,85E+00	2,77E-02	1,88E-01	C530008M17Rik	0,56	No
ENSMUSG00000036381	4,85E-02	7,404373611	6,64E-01	4,15E-01	7,10E-01	P2ry14	1,03	No
ENSMUSG00000036390	-2,50E-01	4,289474418	9,02E+00	2,67E-03	3,50E-02	Gadd45a	0,84	Yes
ENSMUSG00000036391	1,24E-01	6,081460804	3,62E+00	5,70E-02	2,94E-01	Sec24a	1,09	No
ENSMUSG00000036395	6,93E-01	-0,621080481	3,53E+00	6,02E-02	3,04E-01	Glb1l2	1,62	No
ENSMUSG00000036398	-5,31E-02	4,943490425	5,05E-01	4,77E-01	7,65E-01	Ppp1r11	0,96	No
ENSMUSG00000036402	6,23E-02	6,949455321	1,05E+00	3,06E-01	6,03E-01	Gng12	1,04	No
ENSMUSG00000036403	-3,99E-02	4,456944399	2,44E-01	6,21E-01	8,57E-01	Cep135	0,97	No
ENSMUSG00000036411	-2,92E-01	-0,131421004	8,10E-01	3,68E-01	6,69E-01	9530077C05Rik	0,82	No
ENSMUSG00000036412	-5,13E-01	-1,146012488	1,07E+00	3,02E-01	5,98E-01	Arsi	0,70	No
ENSMUSG00000036427	1,18E-01	7,919697604	4,06E+00	4,40E-02	2,50E-01	Gpi1	1,08	No
ENSMUSG00000036430	9,63E-02	3,640859666	1,02E+00	3,13E-01	6,13E-01	Tbcc	1,07	No
ENSMUSG00000036432	2,55E-01	5,855240123	1,46E+01	1,34E-04	3,09E-03	Siah2	1,19	Yes
ENSMUSG00000036435	6,27E-02	6,381939497	9,83E-01	3,22E-01	6,22E-01	Exoc1	1,04	No
ENSMUSG00000036437	-1,13E+00	-1,800968412	3,80E+00	5,12E-02	2,75E-01	Npy1r	0,46	No
ENSMUSG00000036438	-3,93E-02	7,626714692	4,44E-01	5,05E-01	7,90E-01	Calm2	0,97	No
ENSMUSG00000036442	6,20E-03	4,745744706	6,54E-03	9,36E-01	1,00E+00	Thap11	1,00	No
ENSMUSG00000036446	-3,22E+00	-2,963202658	4,38E+00	3,63E-02	2,26E-01	Lum	0,11	No
ENSMUSG00000036449	-1,64E+00	-0,485023899	2,13E+01	4,02E-06	1,50E-04	Lcn8	0,32	Yes
ENSMUSG00000036450	2,80E-02	5,875390373	1,79E-01	6,72E-01	8,89E-01	Hif1an	1,02	No
ENSMUSG00000036452	3,12E-01	5,932763827	2,22E+01	2,44E-06	9,67E-05	Arhgap26	1,24	Yes
ENSMUSG00000036459	-3,09E-01	3,676082911	1,07E+01	1,08E-03	1,72E-02	Wtip	0,81	Yes
ENSMUSG00000036461	9,79E-02	6,356048296	2,40E+00	1,21E-01	4,34E-01	Elf1	1,07	No

ENSMUSG00000036466	1,69E-01	1,530196286	9,32E-01	3,34E-01	6,35E-01	Megf11	1,12	No
ENSMUSG00000036469	2,52E-01	7,116207288	1,74E+01	2,98E-05	8,68E-04	March1	1,19	Yes
ENSMUSG00000036473	1,11E-01	5,144055201	2,36E+00	1,24E-01	4,41E-01	Tbc1d24	1,08	No
ENSMUSG00000036478	-2,37E-01	10,4105796	1,78E+01	2,40E-05	7,25E-04	Btg1	0,85	Yes
ENSMUSG00000036480	1,88E+00	-2,963229875	1,37E+00	2,42E-01	5,50E-01	Prss56	3,68	No
ENSMUSG00000036492	-2,03E+00	-2,497594719	3,14E+00	7,65E-02	3,53E-01	Rnf39	0,24	No
ENSMUSG00000036499	1,46E-01	7,107816466	5,86E+00	1,55E-02	1,29E-01	Eea1	1,11	No
ENSMUSG00000036500	-1,94E+00	-3,107619243	1,50E+00	2,21E-01	5,50E-01	Akp3	0,26	No
ENSMUSG00000036501	-4,52E-02	5,765629582	4,57E-01	4,99E-01	7,84E-01	Fam13b	0,97	No
ENSMUSG00000036503	5,75E-02	6,933303591	8,88E-01	3,46E-01	6,47E-01	Rnf13	1,04	No
ENSMUSG00000036504	-9,75E-02	1,778724345	3,63E-01	5,47E-01	8,14E-01	Phpt1	0,93	No
ENSMUSG00000036513	1,07E-01	4,798402247	1,97E+00	1,61E-01	5,09E-01	Commd2	1,08	No
ENSMUSG00000036523	7,18E-01	1,464339456	1,51E+01	1,00E-04	2,42E-03	Greb1	1,64	Yes
ENSMUSG00000036526	-2,71E-02	6,186635965	1,78E-01	6,73E-01	8,90E-01	Card11	0,98	No
ENSMUSG00000036528	-2,94E-01	5,613899588	1,86E+01	1,63E-05	5,17E-04	Ppfibp2	0,82	Yes
ENSMUSG00000036529	2,44E-02	7,100415922	1,64E-01	6,85E-01	8,97E-01	Sbf1	1,02	No
ENSMUSG00000036533	1,40E-01	4,116106034	2,66E+00	1,03E-01	3,95E-01	Cdc42ep3	1,10	No
ENSMUSG00000036534	2,21E-01	5,599269296	1,04E+01	1,25E-03	1,94E-02	Slc38a7	1,17	Yes
ENSMUSG00000036545	-7,46E-02	-1,600583707	1,43E-02	9,05E-01	1,00E+00	Adamts2	0,95	No
ENSMUSG00000036550	-1,93E-03	7,871280118	1,09E-03	9,74E-01	1,00E+00	Cnot1	1,00	No
ENSMUSG00000036552	-9,84E-02	4,385974387	1,47E+00	2,25E-01	5,50E-01	Ermard	0,93	No
ENSMUSG00000036553	3,78E-01	6,904012221	3,80E+01	7,18E-10	6,01E-08	Sh3tc1	1,30	Yes
ENSMUSG00000036555	1,21E-03	4,720520426	2,49E-04	9,87E-01	1,00E+00	Iqce	1,00	No
ENSMUSG00000036557	-1,17E+00	-2,448682833	1,78E+00	1,82E-01	5,43E-01	Stpg4	0,44	No
ENSMUSG00000036560	-3,02E-01	2,457910573	5,66E+00	1,73E-02	1,38E-01	Lgi4	0,81	No
ENSMUSG00000036561	5,24E-02	3,505755155	2,81E-01	5,96E-01	8,45E-01	Ppp6r2	1,04	No
ENSMUSG00000036564	-1,16E+00	2,968187851	1,07E+02	4,14E-25	1,44E-22	Ndrg4	0,45	Yes
ENSMUSG00000036565	-2,28E-01	7,816714893	1,52E+01	9,89E-05	2,40E-03	Ttyh3	0,85	Yes
ENSMUSG00000036568	3,27E-02	4,906989322	1,92E-01	6,62E-01	8,83E-01	Bicral	1,02	No
ENSMUSG00000036570	-1,15E+00	-1,997929821	2,17E+00	1,41E-01	4,75E-01	Fxyd1	0,45	No
ENSMUSG00000036578	-6,33E-01	-2,268074525	5,45E-01	4,60E-01	7,49E-01	Fxyd7	0,64	No
ENSMUSG00000036580	1,12E-01	3,904471366	1,54E+00	2,14E-01	5,50E-01	Spg20	1,08	No

ENSMUSG00000036585	-9,13E-01	1,345307848	2,67E+01	2,39E-07	1,24E-05	Fgf1	0,53	Yes
ENSMUSG00000036586	-1,89E+00	-3,1076441	1,42E+00	2,33E-01	5,50E-01	Grifin	0,27	No
ENSMUSG00000036587	3,37E-03	3,183675269	9,97E-04	9,75E-01	1,00E+00	Fut7	1,00	No
ENSMUSG00000036591	6,06E-02	6,343021754	9,06E-01	3,41E-01	6,42E-01	Arhgap21	1,04	No
ENSMUSG00000036594	-4,93E-02	11,60426104	7,79E-01	3,77E-01	6,78E-01	H2-Aa	0,97	No
ENSMUSG00000036598	-2,71E+00	-3,033620915	2,92E+00	8,75E-02	3,78E-01	Ccdc113	0,15	No
ENSMUSG00000036599	9,87E-02	5,181425375	1,88E+00	1,70E-01	5,26E-01	Chst12	1,07	No
ENSMUSG00000036602	1,85E+00	-3,107657832	1,35E+00	2,46E-01	5,50E-01	Alx1	3,61	No
ENSMUSG00000036606	2,12E-01	7,005315999	1,20E+01	5,19E-04	9,46E-03	Plxnb2	1,16	Yes
ENSMUSG00000036611	-2,36E-02	6,207382994	1,34E-01	7,15E-01	9,12E-01	Eepd1	0,98	No
ENSMUSG00000036613	-1,39E-01	5,579950249	4,19E+00	4,05E-02	2,38E-01	Tssc1	0,91	No
ENSMUSG00000036615	8,25E-02	3,949188455	8,26E-01	3,63E-01	6,64E-01	Rfxap	1,06	No
ENSMUSG00000036617	-2,54E-01	4,110049135	8,83E+00	2,97E-03	3,81E-02	Etl4	0,84	Yes
ENSMUSG00000036620	6,48E-02	6,017906642	9,84E-01	3,21E-01	6,22E-01	Mgat4b	1,05	No
ENSMUSG00000036622	3,45E-01	7,372050576	3,33E+01	7,70E-09	5,46E-07	Atp13a2	1,27	Yes
ENSMUSG00000036632	1,10E-01	4,319554914	1,72E+00	1,89E-01	5,50E-01	Alg5	1,08	No
ENSMUSG00000036634	1,20E-01	3,902146419	1,75E+00	1,86E-01	5,50E-01	Mag	1,09	No
ENSMUSG00000036636	1,87E-01	7,506317485	1,00E+01	1,55E-03	2,29E-02	Clcn7	1,14	Yes
ENSMUSG00000036639	-1,16E-01	3,309511864	1,26E+00	2,61E-01	5,55E-01	Nudt1	0,92	No
ENSMUSG00000036641	3,44E-01	-1,070238865	5,08E-01	4,76E-01	7,64E-01	Ccdc148	1,27	No
ENSMUSG00000036644	3,88E-02	7,049836601	4,12E-01	5,21E-01	8,00E-01	Tbc1d9b	1,03	No
ENSMUSG00000036646	-3,35E-02	6,605532425	2,90E-01	5,90E-01	8,41E-01	Man1b1	0,98	No
ENSMUSG00000036655	1,24E+00	-2,770581513	9,58E-01	3,28E-01	6,29E-01	Colec11	2,37	No
ENSMUSG00000036661	-3,62E-02	6,250321017	3,22E-01	5,71E-01	8,31E-01	Dennd3	0,98	No
ENSMUSG00000036667	2,65E-01	4,391500408	1,03E+01	1,35E-03	2,06E-02	Tcaf1	1,20	Yes
ENSMUSG00000036672	5,79E-02	4,346019508	4,90E-01	4,84E-01	7,71E-01	Cenpt	1,04	No
ENSMUSG00000036676	1,67E-01	4,831057424	4,83E+00	2,80E-02	1,89E-01	Tmtc3	1,12	No
ENSMUSG00000036678	-2,73E-02	4,462810251	1,14E-01	7,36E-01	9,24E-01	Aaas	0,98	No
ENSMUSG00000036686	-4,34E-02	4,99932941	3,48E-01	5,55E-01	8,20E-01	Cc2d1a	0,97	No
ENSMUSG00000036687	9,84E-03	0,25165739	1,23E-03	9,72E-01	1,00E+00	Tmem184a	1,01	No
ENSMUSG00000036693	1,69E-02	4,860610212	4,93E-02	8,24E-01	9,73E-01	Nop14	1,01	No
ENSMUSG00000036698	-5,50E-02	7,862127025	8,87E-01	3,46E-01	6,47E-01	Ago2	0,96	No

ENSMUSG00000036707	-5,84E-02	7,811141109	9,96E-01	3,18E-01	6,19E-01	Cab39	0,96	No
ENSMUSG00000036712	-1,26E-01	6,669742213	4,15E+00	4,16E-02	2,42E-01	Cyld	0,92	No
ENSMUSG00000036718	-1,75E-01	4,161645437	4,12E+00	4,24E-02	2,45E-01	Micall2	0,89	No
ENSMUSG00000036721	-4,42E-01	3,25889007	1,79E+01	2,38E-05	7,22E-04	Zscan12	0,74	Yes
ENSMUSG00000036731	-2,67E+00	-2,963242888	2,84E+00	9,21E-02	3,82E-01	Cysrt1	0,16	No
ENSMUSG00000036733	-3,80E-02	5,564622653	3,09E-01	5,78E-01	8,36E-01	Rbm42	0,97	No
ENSMUSG00000036737	7,55E-02	6,160315443	1,37E+00	2,42E-01	5,50E-01	Oxsr1	1,05	No
ENSMUSG00000036743	8,68E-02	1,441555703	2,36E-01	6,27E-01	8,61E-01	Psma8	1,06	No
ENSMUSG00000036744	-7,02E-02	-2,71172505	3,32E-03	9,54E-01	1,00E+00	Olfr701	0,95	No
ENSMUSG00000036745	1,02E-01	-1,896217054	2,05E-02	8,86E-01	1,00E+00	Ttll7	1,07	No
ENSMUSG00000036748	-4,37E-02	5,608914577	4,09E-01	5,23E-01	8,01E-01	Cuedc2	0,97	No
ENSMUSG00000036751	-3,08E-02	6,692478021	2,47E-01	6,19E-01	8,56E-01	Cox6b1	0,98	No
ENSMUSG00000036752	7,83E-02	7,454332143	1,74E+00	1,88E-01	5,50E-01	Tubb4b	1,06	No
ENSMUSG00000036764	-5,12E-01	3,444766066	2,66E+01	2,55E-07	1,30E-05	Dnajc12	0,70	Yes
ENSMUSG00000036766	-6,88E-01	-1,655149523	1,35E+00	2,45E-01	5,50E-01	Dner	0,62	No
ENSMUSG00000036768	1,97E-01	5,497514965	8,09E+00	4,46E-03	5,12E-02	Kif15	1,15	No
ENSMUSG00000036770	-8,60E-01	-2,896084029	4,06E-01	5,24E-01	8,01E-01	Stpg3	0,55	No
ENSMUSG00000036775	-2,20E-01	4,361556602	7,17E+00	7,43E-03	7,49E-02	Decr2	0,86	No
ENSMUSG00000036777	3,74E-01	5,754820982	3,09E+01	2,78E-08	1,74E-06	Anln	1,30	Yes
ENSMUSG00000036779	5,76E-02	5,772238917	7,41E-01	3,89E-01	6,90E-01	Papd5	1,04	No
ENSMUSG00000036781	-1,99E-01	5,765333938	8,78E+00	3,05E-03	3,87E-02	Rps27l	0,87	Yes
ENSMUSG00000036792	-2,65E-02	4,52745595	1,10E-01	7,40E-01	9,26E-01	Mbd5	0,98	No
ENSMUSG00000036805	-3,16E+00	-2,963246013	4,20E+00	4,04E-02	2,38E-01	Noxa1	0,11	No
ENSMUSG00000036810	4,27E-02	4,82501501	3,16E-01	5,74E-01	8,33E-01	Cnep1r1	1,03	No
ENSMUSG00000036813	-1,92E+00	-3,0336536	1,46E+00	2,27E-01	5,50E-01	Entpd8	0,26	No
ENSMUSG00000036814	-5,67E-01	-0,8585805	1,99E+00	1,58E-01	5,05E-01	Slc6a20a	0,67	No
ENSMUSG00000036815	-9,93E-01	0,345040712	1,52E+01	9,93E-05	2,41E-03	Dpp10	0,50	Yes
ENSMUSG00000036817	8,15E-02	5,654998762	1,44E+00	2,31E-01	5,50E-01	Sun1	1,06	No
ENSMUSG00000036819	-3,00E-02	3,178476971	7,94E-02	7,78E-01	9,47E-01	Jmjd4	0,98	No
ENSMUSG00000036820	1,76E-01	4,9765566	5,55E+00	1,85E-02	1,44E-01	Amdhd2	1,13	No
ENSMUSG00000036822	8,36E-02	5,792695638	1,56E+00	2,11E-01	5,50E-01	Topors	1,06	No
ENSMUSG00000036825	3,99E-02	3,680650289	1,74E-01	6,76E-01	8,91E-01	Ssx2ip	1,03	No

ENSMUSG00000036826	-2,78E-02	4,23825067	1,10E-01	7,40E-01	9,26E-01	Igflr1	0,98	No
ENSMUSG00000036832	4,14E-01	2,280717704	9,19E+00	2,43E-03	3,27E-02	Lpar3	1,33	Yes
ENSMUSG00000036833	1,91E-01	7,18055321	1,01E+01	1,52E-03	2,25E-02	Pnpla7	1,14	Yes
ENSMUSG00000036834	1,49E-01	-1,997928891	3,85E-02	8,44E-01	9,86E-01	Plch1	1,11	No
ENSMUSG00000036835	-5,92E-02	3,33271144	3,31E-01	5,65E-01	8,28E-01	Psenen	0,96	No
ENSMUSG00000036840	-3,14E-01	3,40856722	9,43E+00	2,13E-03	2,94E-02	Siah1a	0,80	Yes
ENSMUSG00000036845	1,77E-01	2,963625361	2,50E+00	1,14E-01	4,17E-01	Lin37	1,13	No
ENSMUSG00000036850	7,60E-02	4,749173819	9,67E-01	3,26E-01	6,26E-01	Mrpl41	1,05	No
ENSMUSG00000036853	4,18E-01	4,429566614	2,61E+01	3,29E-07	1,63E-05	Mcoln3	1,34	Yes
ENSMUSG00000036854	1,71E-02	2,822558937	2,13E-02	8,84E-01	1,00E+00	Hspb6	1,01	No
ENSMUSG00000036855	-2,63E-02	-3,033641358	2,33E-04	9,88E-01	1,00E+00	Gjd4	0,98	No
ENSMUSG00000036856	-1,17E+00	-1,896120686	2,24E+00	1,35E-01	4,62E-01	Wnt4	0,44	No
ENSMUSG00000036858	-8,48E-01	-2,832006653	3,94E-01	5,30E-01	8,05E-01	Ptcra	0,56	No
ENSMUSG00000036860	-1,25E-02	3,579574665	1,64E-02	8,98E-01	1,00E+00	Mrpl55	0,99	No
ENSMUSG00000036862	6,47E-01	0,508491602	7,02E+00	8,04E-03	7,93E-02	Dchs1	1,57	No
ENSMUSG00000036863	6,21E-01	2,953930937	2,98E+01	4,68E-08	2,81E-06	Syde2	1,54	Yes
ENSMUSG00000036864	1,42E-01	2,127719509	1,00E+00	3,17E-01	6,17E-01	Proser3	1,10	No
ENSMUSG00000036867	-4,60E-01	1,160486364	5,10E+00	2,39E-02	1,69E-01	Smad6	0,73	No
ENSMUSG00000036873	3,16E-02	3,928421661	1,22E-01	7,27E-01	9,19E-01	2410004B18Rik	1,02	No
ENSMUSG00000036875	3,59E-01	6,585432869	3,29E+01	9,76E-09	6,80E-07	Dna2	1,28	Yes
ENSMUSG00000036879	-8,74E-02	5,110733518	1,45E+00	2,29E-01	5,50E-01	Phkb	0,94	No
ENSMUSG00000036880	6,22E-02	5,363258779	7,77E-01	3,78E-01	6,79E-01	Acaa2	1,04	No
ENSMUSG00000036882	1,90E-01	2,771825353	2,49E+00	1,15E-01	4,19E-01	Arhgap33	1,14	No
ENSMUSG00000036885	2,09E-01	-1,117043812	1,95E-01	6,59E-01	8,81E-01	Arhgef26	1,16	No
ENSMUSG00000036887	7,71E-01	6,737337556	1,50E+02	1,37E-34	1,08E-31	C1qa	1,71	Yes
ENSMUSG00000036890	-2,13E-01	2,80551291	3,26E+00	7,11E-02	3,38E-01	Gtdc1	0,86	No
ENSMUSG00000036893	-5,26E-02	5,895630913	6,38E-01	4,24E-01	7,19E-01	Ehmt1	0,96	No
ENSMUSG00000036894	-1,60E-01	6,665009249	6,74E+00	9,41E-03	8,93E-02	Rap2b	0,89	No
ENSMUSG00000036896	7,51E-01	7,400990925	1,55E+02	1,53E-35	1,28E-32	C1qc	1,68	Yes
ENSMUSG00000036898	1,21E-01	3,225493621	1,32E+00	2,50E-01	5,50E-01	Zfp157	1,09	No
ENSMUSG00000036902	-4,34E-02	1,497966622	5,88E-02	8,08E-01	9,65E-01	Neto2	0,97	No
ENSMUSG00000036904	7,22E-01	-0,669332633	3,80E+00	5,14E-02	2,76E-01	Fzd8	1,65	No

ENSMUSG00000036905	6,34E-01	8,41246524	1,19E+02	8,62E-28	3,97E-25	C1qb	1,55	Yes
ENSMUSG00000036908	6,12E-03	8,848411029	1,15E-02	9,15E-01	1,00E+00	Unc93b1	1,00	No
ENSMUSG00000036912	-1,07E+00	-0,316251648	1,08E+01	1,01E-03	1,63E-02	Piwil4	0,48	Yes
ENSMUSG00000036915	-2,88E-02	-2,963268425	3,79E-04	9,84E-01	1,00E+00	Kirrel2	0,98	No
ENSMUSG00000036918	1,08E-01	7,112431511	3,22E+00	7,28E-02	3,42E-01	Ttc7	1,08	No
ENSMUSG00000036923	-7,17E-01	-1,448727078	1,94E+00	1,64E-01	5,15E-01	Stox1	0,61	No
ENSMUSG00000036925	4,09E-01	-2,26807888	2,68E-01	6,05E-01	8,49E-01	Mucl2	1,33	No
ENSMUSG00000036928	1,91E-01	0,563307422	6,32E-01	4,26E-01	7,21E-01	Stag3	1,14	No
ENSMUSG00000036931	2,18E-01	4,742090516	8,00E+00	4,69E-03	5,32E-02	Nfkbid	1,16	No
ENSMUSG00000036934	2,46E-01	2,561884058	3,79E+00	5,17E-02	2,77E-01	4921524J17Rik	1,19	No
ENSMUSG00000036940	-6,78E-02	5,669241984	1,01E+00	3,15E-01	6,16E-01	Kdm1a	0,95	No
ENSMUSG00000036941	-7,43E-02	4,824545633	9,63E-01	3,27E-01	6,27E-01	Elac1	0,95	No
ENSMUSG00000036943	-1,56E-01	8,659194002	7,46E+00	6,32E-03	6,67E-02	Rab8b	0,90	No
ENSMUSG00000036944	1,14E-01	3,851135545	1,54E+00	2,15E-01	5,50E-01	Tmem71	1,08	No
ENSMUSG00000036948	1,52E-01	5,574067838	4,94E+00	2,62E-02	1,81E-01	BC037034	1,11	No
ENSMUSG00000036949	3,14E-01	0,032335763	1,04E+00	3,08E-01	6,07E-01	Slc39a12	1,24	No
ENSMUSG00000036951	-1,87E+00	-3,107637761	1,39E+00	2,38E-01	5,50E-01	C130079G13Rik	0,27	No
ENSMUSG00000036955	2,04E-02	4,616753189	6,78E-02	7,95E-01	9,56E-01	Kif1bp	1,01	No
ENSMUSG00000036957	1,85E-01	-2,268058866	5,11E-02	8,21E-01	9,72E-01	Lrfn3	1,14	No
ENSMUSG00000036961	9,11E-01	-1,832029338	2,38E+00	1,23E-01	4,37E-01	Wnt8b	1,88	No
ENSMUSG00000036962	-1,94E+00	-3,107619243	1,50E+00	2,21E-01	5,50E-01	Cfap221	0,26	No
ENSMUSG00000036964	1,16E-01	2,5083201	8,23E-01	3,64E-01	6,65E-01	Trim17	1,08	No
ENSMUSG00000036966	-3,25E-03	5,466486462	2,18E-03	9,63E-01	1,00E+00	Spryd3	1,00	No
ENSMUSG00000036968	4,74E-03	4,024211262	2,86E-03	9,57E-01	1,00E+00	Cnpy4	1,00	No
ENSMUSG00000036975	6,56E-02	3,311328123	4,00E-01	5,27E-01	8,04E-01	Tmem177	1,05	No
ENSMUSG00000036977	-6,02E-02	2,669377564	2,43E-01	6,22E-01	8,58E-01	Anapc10	0,96	No
ENSMUSG00000036980	6,31E-02	4,852064924	6,97E-01	4,04E-01	7,01E-01	Taf6	1,04	No
ENSMUSG00000036983	2,65E-01	2,712629695	4,67E+00	3,07E-02	2,02E-01	Tfb1m	1,20	No
ENSMUSG00000036986	-1,55E-02	5,190320088	4,66E-02	8,29E-01	9,76E-01	Pml	0,99	No
ENSMUSG00000036989	4,76E-02	5,438956716	4,69E-01	4,93E-01	7,79E-01	Trim3	1,03	No
ENSMUSG00000036990	2,80E-02	6,688982207	2,04E-01	6,52E-01	8,77E-01	Otud4	1,02	No
ENSMUSG00000036992	8,27E-02	4,059566567	8,84E-01	3,47E-01	6,48E-01	Nxt1	1,06	No

ENSMUSG00000036995	1,19E-01	0,557314381	2,72E-01	6,02E-01	8,48E-01	Asap3	1,09	No
ENSMUSG00000037001	4,62E-02	2,118682436	1,02E-01	7,49E-01	9,31E-01	Zfp39	1,03	No
ENSMUSG00000037003	2,16E-02	1,525607585	1,59E-02	9,00E-01	1,00E+00	Tns2	1,02	No
ENSMUSG00000037007	3,84E-02	2,449382017	9,24E-02	7,61E-01	9,37E-01	Zfp113	1,03	No
ENSMUSG00000037012	8,29E-02	6,727669229	1,80E+00	1,80E-01	5,41E-01	Hk1	1,06	No
ENSMUSG00000037013	2,48E-01	6,541009846	1,56E+01	7,69E-05	1,95E-03	Ss18	1,19	Yes
ENSMUSG00000037014	7,44E-01	-2,896157547	3,02E-01	5,83E-01	8,38E-01	Sstr4	1,68	No
ENSMUSG00000037016	5,83E-01	-2,146110925	5,75E-01	4,48E-01	7,38E-01	Frem2	1,50	No
ENSMUSG00000037017	1,11E-01	3,741404335	1,46E+00	2,27E-01	5,50E-01	Zscan21	1,08	No
ENSMUSG00000037020	2,58E-01	5,117557318	1,24E+01	4,23E-04	7,98E-03	Wdr62	1,20	Yes
ENSMUSG00000037022	1,58E-01	2,452901784	1,48E+00	2,24E-01	5,50E-01	Mmaa	1,12	No
ENSMUSG00000037029	2,31E-01	4,587710358	8,48E+00	3,59E-03	4,38E-02	Zfp146	1,17	Yes
ENSMUSG00000037031	-1,92E+00	-3,033623267	1,46E+00	2,27E-01	5,50E-01	Tspan15	0,26	No
ENSMUSG00000037032	-1,17E+00	-1,378233942	5,07E+00	2,43E-02	1,71E-01	Apbb1	0,44	No
ENSMUSG00000037035	-2,76E-01	-2,03362296	1,32E-01	7,16E-01	9,13E-01	Inhbb	0,83	No
ENSMUSG00000037049	1,23E-01	4,623326546	2,41E+00	1,20E-01	4,32E-01	Smpd1	1,09	No
ENSMUSG00000037058	-3,82E-02	5,891073301	3,35E-01	5,63E-01	8,26E-01	Paip2	0,97	No
ENSMUSG00000037060	-9,18E-01	-2,522642731	7,39E-01	3,90E-01	6,90E-01	Cavin3	0,53	No
ENSMUSG00000037062	4,19E-04	7,562994895	5,03E-05	9,94E-01	1,00E+00	Sh3glb1	1,00	No
ENSMUSG00000037070	1,00E-02	5,209786971	1,95E-02	8,89E-01	1,00E+00	Rbmxl1	1,01	No
ENSMUSG00000037071	1,92E-01	7,521079833	1,05E+01	1,17E-03	1,84E-02	Scd1	1,14	Yes
ENSMUSG00000037072	4,17E-02	7,62825127	4,99E-01	4,80E-01	7,68E-01	Selenof	1,03	No
ENSMUSG00000037075	9,72E-02	5,635801712	2,01E+00	1,56E-01	5,00E-01	Rnf139	1,07	No
ENSMUSG00000037085	2,93E-01	3,077748387	7,05E+00	7,93E-03	7,85E-02	Trmt12	1,23	No
ENSMUSG00000037089	-5,95E-02	5,824048447	7,99E-01	3,71E-01	6,72E-01	Slc35b2	0,96	No
ENSMUSG00000037095	7,02E-01	-0,946366302	2,54E+00	1,11E-01	4,08E-01	Lrg1	1,63	No
ENSMUSG00000037096	6,58E-03	5,750706567	9,59E-03	9,22E-01	1,00E+00	Gm9762	1,00	No
ENSMUSG00000037098	-5,40E-02	3,656930008	3,31E-01	5,65E-01	8,28E-01	Rab11fip3	0,96	No
ENSMUSG00000037103	-1,07E-02	5,032254787	2,10E-02	8,85E-01	1,00E+00	Dcaf15	0,99	No
ENSMUSG00000037104	1,85E-01	4,005247591	4,51E+00	3,37E-02	2,16E-01	Socs5	1,14	No
ENSMUSG00000037106	7,72E-01	-1,26803517	2,39E+00	1,22E-01	4,36E-01	Fer1l6	1,71	No
ENSMUSG00000037108	6,57E-02	4,459601675	6,49E-01	4,20E-01	7,16E-01	Zcwpw1	1,05	No

ENSMUSG00000037110	1,56E-01	4,967020015	4,32E+00	3,77E-02	2,31E-01	Ralgapa2	1,11	No
ENSMUSG00000037111	4,13E-02	7,405544064	4,83E-01	4,87E-01	7,74E-01	Setd7	1,03	No
ENSMUSG00000037112	-2,17E-02	4,740129222	7,85E-02	7,79E-01	9,48E-01	Sik2	0,99	No
ENSMUSG00000037119	1,93E-01	7,319263299	1,04E+01	1,25E-03	1,93E-02	Fam91a1	1,14	Yes
ENSMUSG00000037126	-4,30E-01	5,289336551	3,73E+01	1,03E-09	8,40E-08	Psd	0,74	Yes
ENSMUSG00000037134	2,48E-01	4,433873843	9,34E+00	2,25E-03	3,07E-02	Prmt9	1,19	Yes
ENSMUSG00000037138	2,20E-01	3,657495079	5,24E+00	2,21E-02	1,61E-01	Aff3	1,16	No
ENSMUSG00000037139	2,81E-02	0,112069178	1,05E-02	9,19E-01	1,00E+00	Myom3	1,02	No
ENSMUSG00000037143	3,14E-02	-3,107617333	-7,69E-10	1,00E+00	1,00E+00	Cfap61	1,02	No
ENSMUSG00000037148	3,14E-01	5,079881775	1,82E+01	1,98E-05	6,15E-04	Arhgap10	1,24	Yes
ENSMUSG00000037149	-2,90E-02	5,510993142	1,78E-01	6,73E-01	8,90E-01	Ddx1	0,98	No
ENSMUSG00000037151	1,84E-01	4,49192903	5,26E+00	2,18E-02	1,59E-01	Lrrc20	1,14	No
ENSMUSG00000037152	1,62E-01	2,952566732	1,93E+00	1,64E-01	5,16E-01	Ndufc1	1,12	No
ENSMUSG00000037157	-1,92E+00	-3,0336536	1,46E+00	2,27E-01	5,50E-01	Il22ra1	0,26	No
ENSMUSG00000037159	1,89E+00	-2,96320077	1,38E+00	2,40E-01	5,50E-01	Wee2	3,71	No
ENSMUSG00000037161	7,83E-01	0,044904991	6,51E+00	1,07E-02	9,83E-02	Mgarp	1,72	No
ENSMUSG00000037166	7,53E-02	0,758372452	1,14E-01	7,36E-01	9,24E-01	Ppp1r14a	1,05	No
ENSMUSG00000037169	-3,19E+00	-2,896088812	4,27E+00	3,87E-02	2,33E-01	Mycn	0,11	No
ENSMUSG00000037171	4,41E-01	-2,832010046	1,52E-01	6,97E-01	9,03E-01	Nodal	1,36	No
ENSMUSG00000037172	-3,41E-02	4,952211214	2,11E-01	6,46E-01	8,74E-01	E330009J07Rik	0,98	No
ENSMUSG00000037174	1,21E-02	6,126180812	3,49E-02	8,52E-01	9,90E-01	Elf2	1,01	No
ENSMUSG00000037185	3,67E-01	-1,333217669	4,36E-01	5,09E-01	7,92E-01	Krt80	1,29	No
ENSMUSG00000037188	1,45E+00	-1,998040779	3,89E+00	4,86E-02	2,65E-01	Grhl3	2,74	No
ENSMUSG00000037190	2,24E-01	3,720493328	5,55E+00	1,85E-02	1,44E-01	Cyb561d2	1,17	No
ENSMUSG00000037196	-6,09E-02	-2,548220308	3,38E-03	9,54E-01	1,00E+00	Pacrg	0,96	No
ENSMUSG00000037197	-2,48E-01	5,310441071	1,23E+01	4,59E-04	8,55E-03	Rbm17	0,84	Yes
ENSMUSG00000037202	-1,86E+00	-2,963227946	1,40E+00	2,36E-01	5,50E-01	Prf1	0,28	No
ENSMUSG00000037204	-7,98E-03	4,540202221	1,00E-02	9,20E-01	1,00E+00	Atg101	0,99	No
ENSMUSG00000037206	1,84E+00	-2,832034594	1,33E+00	2,49E-01	5,50E-01	Islr	3,57	No
ENSMUSG00000037210	-5,95E-02	5,3767887	7,22E-01	3,95E-01	6,96E-01	Fam193a	0,96	No
ENSMUSG00000037211	-2,65E-01	3,147134231	6,07E+00	1,37E-02	1,18E-01	Spry1	0,83	No
ENSMUSG00000037214	1,16E-02	3,298696766	1,21E-02	9,12E-01	1,00E+00	Thap1	1,01	No

ENSMUSG00000037216	-5,28E-02	0,755631381	5,66E-02	8,12E-01	9,67E-01	Lipt1	0,96	No
ENSMUSG00000037221	1,48E-01	4,96334156	3,92E+00	4,77E-02	2,62E-01	Mospd3	1,11	No
ENSMUSG00000037224	-4,54E-01	-0,847842518	1,31E+00	2,52E-01	5,50E-01	Zfyve28	0,73	No
ENSMUSG00000037225	-2,88E-01	1,265182916	2,39E+00	1,22E-01	4,37E-01	Fgf2	0,82	No
ENSMUSG00000037234	8,21E-02	6,718629878	1,77E+00	1,83E-01	5,46E-01	Hook3	1,06	No
ENSMUSG00000037235	1,67E-02	6,62234515	7,22E-02	7,88E-01	9,53E-01	Mxd4	1,01	No
ENSMUSG00000037236	8,94E-02	7,03458553	2,17E+00	1,40E-01	4,73E-01	Matr3	1,06	No
ENSMUSG00000037239	-3,23E-01	3,860115397	1,26E+01	3,86E-04	7,43E-03	Spred3	0,80	Yes
ENSMUSG00000037242	-1,81E-01	8,719235583	1,00E+01	1,56E-03	2,30E-02	Clic4	0,88	Yes
ENSMUSG00000037243	7,56E-02	3,757173004	6,36E-01	4,25E-01	7,19E-01	Zfp692	1,05	No
ENSMUSG00000037247	-5,80E-02	-3,033647069	1,56E-03	9,68E-01	1,00E+00	Pldi	0,96	No
ENSMUSG00000037251	1,13E-01	3,745193481	1,47E+00	2,25E-01	5,50E-01	Pomk	1,08	No
ENSMUSG00000037253	2,34E-02	5,857465946	1,25E-01	7,24E-01	9,17E-01	Mex3c	1,02	No
ENSMUSG00000037254	-3,86E+00	-2,770568777	7,14E+00	7,55E-03	7,58E-02	Itih2	0,07	No
ENSMUSG00000037257	4,92E-02	5,272997312	4,79E-01	4,89E-01	7,76E-01	Aagab	1,03	No
ENSMUSG00000037259	7,41E-01	-2,070195534	9,81E-01	3,22E-01	6,22E-01	Dzank1	1,67	No
ENSMUSG00000037260	8,76E-02	6,13384398	1,83E+00	1,76E-01	5,34E-01	Hgsnat	1,06	No
ENSMUSG00000037262	4,39E-03	3,611813792	2,02E-03	9,64E-01	1,00E+00	Kin	1,00	No
ENSMUSG00000037263	6,85E-01	-2,896150766	2,55E-01	6,14E-01	8,53E-01	Aldh3b3	1,61	No
ENSMUSG00000037266	-2,28E-02	7,874086498	1,53E-01	6,96E-01	9,03E-01	Rsrp1	0,98	No
ENSMUSG00000037270	5,17E-02	6,624644617	6,88E-01	4,07E-01	7,03E-01	4932438A13Rik	1,04	No
ENSMUSG00000037275	7,35E-02	4,550833935	8,67E-01	3,52E-01	6,53E-01	Gemin5	1,05	No
ENSMUSG00000037278	2,19E-01	4,708980915	7,82E+00	5,16E-03	5,72E-02	Tmem97	1,16	No
ENSMUSG00000037279	1,66E-01	0,536557138	4,27E-01	5,13E-01	7,95E-01	Ovol2	1,12	No
ENSMUSG00000037280	2,43E-01	6,883716247	1,56E+01	7,85E-05	1,98E-03	Galnt6	1,18	Yes
ENSMUSG00000037286	6,33E-04	5,612665706	8,60E-05	9,93E-01	1,00E+00	Stag1	1,00	No
ENSMUSG00000037287	1,95E-01	5,141336872	7,14E+00	7,55E-03	7,58E-02	Tbcel	1,14	No
ENSMUSG00000037295	1,44E-01	5,274026095	4,07E+00	4,36E-02	2,49E-01	Ldlrap1	1,11	No
ENSMUSG00000037296	2,58E-02	4,073573368	8,81E-02	7,67E-01	9,40E-01	Lsm1	1,02	No
ENSMUSG00000037300	6,29E-02	5,527690078	8,34E-01	3,61E-01	6,62E-01	Ttc13	1,04	No
ENSMUSG00000037306	2,16E-01	6,753017818	1,22E+01	4,84E-04	8,93E-03	Man1c1	1,16	Yes
ENSMUSG00000037313	9,72E-02	6,012996287	2,20E+00	1,38E-01	4,68E-01	Tacc3	1,07	No

ENSMUSG00000037316	4,43E-02	4,29738067	2,86E-01	5,93E-01	8,42E-01	Bag4	1,03	No
ENSMUSG00000037318	-1,72E-01	4,711948325	4,96E+00	2,59E-02	1,79E-01	Traf3ip3	0,89	No
ENSMUSG00000037321	-1,71E-01	7,44475889	8,29E+00	3,98E-03	4,72E-02	Tap1	0,89	Yes
ENSMUSG00000037325	2,56E-01	1,68604167	2,38E+00	1,23E-01	4,38E-01	Bbs7	1,19	No
ENSMUSG00000037326	1,24E-01	5,820313289	3,48E+00	6,19E-02	3,10E-01	Capn15	1,09	No
ENSMUSG00000037331	5,84E-02	7,66208388	9,84E-01	3,21E-01	6,22E-01	Larp1	1,04	No
ENSMUSG00000037336	-3,86E-01	0,678055108	2,96E+00	8,56E-02	3,76E-01	Mfsd2b	0,77	No
ENSMUSG00000037337	2,38E-02	5,864125148	1,28E-01	7,20E-01	9,16E-01	Map4k1	1,02	No
ENSMUSG00000037339	1,89E-02	4,737511241	6,03E-02	8,06E-01	9,64E-01	Fam53a	1,01	No
ENSMUSG00000037343	9,49E-02	5,473915835	1,87E+00	1,71E-01	5,28E-01	Taf2	1,07	No
ENSMUSG00000037344	2,08E-01	4,954966631	7,89E+00	4,96E-03	5,57E-02	Slc12a9	1,16	No
ENSMUSG00000037346	-2,36E-01	-1,77058281	1,23E-01	7,26E-01	9,18E-01	Hrh4	0,85	No
ENSMUSG00000037348	1,83E-01	5,25725984	6,56E+00	1,04E-02	9,65E-02	Paqr7	1,14	No
ENSMUSG00000037349	-8,37E-03	2,692778761	4,55E-03	9,46E-01	1,00E+00	Nudt22	0,99	No
ENSMUSG00000037351	7,77E-02	6,625431218	1,56E+00	2,12E-01	5,50E-01	Actr1b	1,06	No
ENSMUSG00000037353	3,60E-02	4,623003855	2,10E-01	6,46E-01	8,74E-01	Letmd1	1,03	No
ENSMUSG00000037355	-4,41E-02	4,614852328	3,15E-01	5,75E-01	8,34E-01	Uvssa	0,97	No
ENSMUSG00000037361	-4,81E-02	5,393964112	4,69E-01	4,93E-01	7,79E-01	Sf3b6	0,97	No
ENSMUSG00000037362	9,02E-02	7,273305719	2,24E+00	1,34E-01	4,61E-01	Nov	1,06	No
ENSMUSG00000037363	-2,71E-01	1,760096055	2,97E+00	8,48E-02	3,74E-01	Letm2	0,83	No
ENSMUSG00000037364	6,22E-02	6,929087749	1,04E+00	3,08E-01	6,06E-01	Srrt	1,04	No
ENSMUSG00000037366	1,35E-01	4,272736165	2,57E+00	1,09E-01	4,05E-01	Pafah2	1,10	No
ENSMUSG00000037370	4,98E-01	2,363520691	1,29E+01	3,27E-04	6,50E-03	Enpp1	1,41	Yes
ENSMUSG00000037373	2,66E-02	7,378696786	2,00E-01	6,55E-01	8,79E-01	Ctbp1	1,02	No
ENSMUSG00000037375	3,11E-02	0,8194467	1,85E-02	8,92E-01	1,00E+00	Hhat	1,02	No
ENSMUSG00000037376	7,38E-03	5,298463053	1,08E-02	9,17E-01	1,00E+00	Trmt6	1,01	No
ENSMUSG00000037379	9,50E-02	-1,655055855	1,99E-02	8,88E-01	1,00E+00	Spon2	1,07	No
ENSMUSG00000037386	-6,34E-01	-2,268106367	5,47E-01	4,59E-01	7,48E-01	Rims2	0,64	No
ENSMUSG00000037390	-1,87E+00	-3,107637761	1,39E+00	2,38E-01	5,50E-01	Muc3	0,27	No
ENSMUSG00000037395	1,84E-01	4,586291397	5,55E+00	1,85E-02	1,44E-01	Rcor3	1,14	No
ENSMUSG00000037400	-1,59E-01	7,023836429	6,94E+00	8,42E-03	8,23E-02	Atp11b	0,90	No
ENSMUSG00000037405	-1,45E-01	7,970114969	6,23E+00	1,26E-02	1,10E-01	Icam1	0,90	No

ENSMUSG00000037406	-3,50E-01	1,233338559	3,44E+00	6,35E-02	3,14E-01	Htra4	0,78	No
ENSMUSG00000037408	2,17E-01	4,951260491	8,42E+00	3,71E-03	4,47E-02	Cnnm4	1,16	Yes
ENSMUSG00000037410	4,45E-02	7,321629604	5,54E-01	4,57E-01	7,46E-01	Tbc1d2b	1,03	No
ENSMUSG00000037411	-9,37E-01	-0,56075955	6,85E+00	8,88E-03	8,55E-02	Serpine1	0,52	No
ENSMUSG00000037415	3,49E-02	6,423315174	3,07E-01	5,80E-01	8,36E-01	Ranbp10	1,02	No
ENSMUSG00000037416	4,08E-02	6,219781747	4,02E-01	5,26E-01	8,03E-01	Dmxl1	1,03	No
ENSMUSG00000037418	-2,19E-01	2,681539733	3,00E+00	8,32E-02	3,71E-01	Best1	0,86	No
ENSMUSG00000037419	-4,69E-02	5,601024328	4,76E-01	4,90E-01	7,77E-01	Endod1	0,97	No
ENSMUSG00000037424	-1,86E+00	-3,033612668	1,39E+00	2,38E-01	5,50E-01	Taar9	0,28	No
ENSMUSG00000037426	-1,55E-01	5,034458082	4,43E+00	3,53E-02	2,22E-01	Depdc5	0,90	No
ENSMUSG00000037428	-1,90E+00	-3,033594154	1,44E+00	2,30E-01	5,50E-01	Vgf	0,27	No
ENSMUSG00000037432	2,37E-01	2,860265656	3,99E+00	4,58E-02	2,55E-01	Fer1l5	1,18	No
ENSMUSG00000037434	1,98E-01	5,091529139	7,30E+00	6,89E-03	7,09E-02	Slc30a1	1,15	No
ENSMUSG00000037437	7,16E-01	-2,711741188	2,80E-01	5,97E-01	8,45E-01	Adam32	1,64	No
ENSMUSG00000037438	-7,94E-02	2,033179227	2,77E-01	5,99E-01	8,46E-01	Uqcrh-ps1	0,95	No
ENSMUSG00000037440	9,01E-03	-3,107646452	-5,16E-10	1,00E+00	1,00E+00	Vnn1	1,01	No
ENSMUSG00000037443	-4,82E-02	6,426981367	5,88E-01	4,43E-01	7,35E-01	Cep85	0,97	No
ENSMUSG00000037446	-1,87E+00	-3,107637761	1,39E+00	2,38E-01	5,50E-01	Tulp1	0,27	No
ENSMUSG00000037447	-2,61E-01	5,089288113	1,28E+01	3,52E-04	6,89E-03	Arid5a	0,83	Yes
ENSMUSG00000037451	5,77E-01	-2,185684276	5,64E-01	4,53E-01	7,42E-01	Slc22a20	1,49	No
ENSMUSG00000037455	9,31E-02	0,112129768	1,06E-01	7,45E-01	9,29E-01	Slc18b1	1,07	No
ENSMUSG00000037458	1,64E-01	7,063859942	7,36E+00	6,68E-03	6,94E-02	Azin1	1,12	No
ENSMUSG00000037461	8,36E-02	5,548524622	1,48E+00	2,25E-01	5,50E-01	Ints7	1,06	No
ENSMUSG00000037463	1,65E-01	-2,401372028	4,05E-02	8,41E-01	9,84E-01	Fbxo27	1,12	No
ENSMUSG00000037465	-1,16E-02	5,696497382	2,90E-02	8,65E-01	9,98E-01	Klf10	0,99	No
ENSMUSG00000037466	3,59E-01	4,087175637	1,67E+01	4,42E-05	1,22E-03	4930427A07Rik	1,28	Yes
ENSMUSG00000037470	1,40E-01	7,434412245	5,55E+00	1,84E-02	1,43E-01	Uggt1	1,10	No
ENSMUSG00000037474	2,90E-02	4,58925555	1,32E-01	7,16E-01	9,13E-01	Dtl	1,02	No
ENSMUSG00000037477	-8,21E-01	-2,963266074	3,67E-01	5,45E-01	8,13E-01	Tbx10	0,57	No
ENSMUSG00000037482	-1,81E+00	-1,963152656	5,21E+00	2,25E-02	1,63E-01	Erv3	0,28	No
ENSMUSG00000037486	6,27E-02	6,435915594	9,88E-01	3,20E-01	6,21E-01	Asxl2	1,04	No
ENSMUSG00000037487	-9,02E-02	7,256560194	2,27E+00	1,32E-01	4,57E-01	Ubr5	0,94	No

ENSMUSG00000037490	-4,05E-01	-0,093309128	1,57E+00	2,10E-01	5,50E-01	Slc2a12	0,76	No
ENSMUSG00000037493	2,88E-02	2,514358474	4,84E-02	8,26E-01	9,74E-01	Cib2	1,02	No
ENSMUSG00000037499	1,95E-02	4,061033028	4,99E-02	8,23E-01	9,73E-01	Nenf	1,01	No
ENSMUSG00000037503	-3,43E-02	8,044794545	3,49E-01	5,55E-01	8,20E-01	Fam168b	0,98	No
ENSMUSG00000037509	1,56E-01	3,026776018	1,96E+00	1,62E-01	5,11E-01	Arhgef4	1,11	No
ENSMUSG00000037514	1,19E-02	5,611525884	3,05E-02	8,61E-01	9,96E-01	Pank2	1,01	No
ENSMUSG00000037519	7,08E-02	6,277174097	1,23E+00	2,68E-01	5,64E-01	Ppfia1	1,05	No
ENSMUSG00000037523	1,77E-01	5,763847732	7,00E+00	8,14E-03	8,00E-02	Mavs	1,13	No
ENSMUSG00000037525	1,40E-01	1,535950072	6,37E-01	4,25E-01	7,19E-01	Bcdin3d	1,10	No
ENSMUSG00000037526	-2,74E-02	4,061620845	9,96E-02	7,52E-01	9,32E-01	Atg14	0,98	No
ENSMUSG00000037529	-3,83E-02	-3,107651936	-7,91E-11	1,00E+00	1,00E+00	Prss40	0,97	No
ENSMUSG00000037531	1,13E-01	4,150078263	1,72E+00	1,89E-01	5,50E-01	Mrpl47	1,08	No
ENSMUSG00000037533	-1,56E-01	6,46835543	6,21E+00	1,27E-02	1,11E-01	Rapgef6	0,90	No
ENSMUSG00000037536	1,30E-01	5,057854603	3,10E+00	7,81E-02	3,57E-01	Fbxo34	1,09	No
ENSMUSG00000037541	-1,15E+00	-2,185653567	2,14E+00	1,44E-01	4,79E-01	Shank2	0,45	No
ENSMUSG00000037542	2,61E+00	-2,831999501	2,64E+00	1,04E-01	3,97E-01	Aldh8a1	6,11	No
ENSMUSG00000037544	6,87E-02	4,593298215	7,52E-01	3,86E-01	6,87E-01	Dlgap5	1,05	No
ENSMUSG00000037548	-6,43E-02	8,057834686	1,22E+00	2,69E-01	5,65E-01	H2-DMb2	0,96	No
ENSMUSG00000037552	-4,60E-01	5,103653644	4,07E+01	1,73E-10	1,58E-08	Plekhg2	0,73	Yes
ENSMUSG00000037553	1,13E-01	5,583679912	2,72E+00	9,91E-02	3,92E-01	Zdhhc18	1,08	No
ENSMUSG00000037563	-1,56E-01	4,871642849	4,31E+00	3,78E-02	2,31E-01	Rps16	0,90	No
ENSMUSG00000037568	3,73E-01	0,598389715	2,24E+00	1,34E-01	4,61E-01	Vash2	1,29	No
ENSMUSG00000037570	-2,26E-01	3,721225741	5,67E+00	1,73E-02	1,37E-01	Mcrs1	0,85	No
ENSMUSG00000037572	1,92E-01	5,401236226	7,49E+00	6,21E-03	6,58E-02	Wdhd1	1,14	No
ENSMUSG00000037573	2,54E-01	5,16958882	1,23E+01	4,56E-04	8,51E-03	Tob1	1,19	Yes
ENSMUSG00000037577	-9,49E-01	-2,655184733	7,88E-01	3,75E-01	6,76E-01	Ephx3	0,52	No
ENSMUSG00000037578	2,97E-01	-2,548235639	9,01E-02	7,64E-01	9,39E-01	Pkd2l1	1,23	No
ENSMUSG00000037579	2,74E-01	2,636240553	5,03E+00	2,50E-02	1,75E-01	Kcnh3	1,21	No
ENSMUSG00000037580	2,66E-02	5,021367888	1,29E-01	7,20E-01	9,15E-01	Gch1	1,02	No
ENSMUSG00000037583	7,54E-01	-2,770589857	3,08E-01	5,79E-01	8,36E-01	Nr0b2	1,69	No
ENSMUSG00000037593	2,41E-01	-0,18540477	5,69E-01	4,51E-01	7,41E-01	BC030499	1,18	No
ENSMUSG00000037594	3,30E-01	1,972867412	4,83E+00	2,80E-02	1,89E-01	BC022687	1,26	No

ENSMUSG00000037600	1,61E+00	-2,226211761	1,78E+00	1,82E-01	5,43E-01	Kdf1	3,06	No
ENSMUSG00000037601	-2,35E-02	5,70055899	1,20E-01	7,29E-01	9,20E-01	Nme1	0,98	No
ENSMUSG00000037603	-1,02E+00	-2,311116348	1,61E+00	2,04E-01	5,50E-01	Gm5519	0,49	No
ENSMUSG00000037605	8,54E-01	-1,205955452	2,54E+00	1,11E-01	4,08E-01	Adgrl3	1,81	No
ENSMUSG00000037606	1,68E-01	2,395835258	1,60E+00	2,06E-01	5,50E-01	Osbpl5	1,12	No
ENSMUSG00000037608	1,02E-02	7,25092983	2,91E-02	8,65E-01	9,98E-01	Bclaf1	1,01	No
ENSMUSG00000037613	2,42E-01	4,768167262	9,87E+00	1,68E-03	2,43E-02	Tnfrsf23	1,18	Yes
ENSMUSG00000037617	2,79E-01	1,143717051	1,99E+00	1,58E-01	5,04E-01	Spag1	1,21	No
ENSMUSG00000037621	-3,18E+00	-2,770538059	4,26E+00	3,91E-02	2,35E-01	Atoh8	0,11	No
ENSMUSG00000037622	4,07E-03	5,722528791	3,65E-03	9,52E-01	1,00E+00	Wdtc1	1,00	No
ENSMUSG00000037625	-5,88E-02	-3,033652696	1,62E-03	9,68E-01	1,00E+00	Cldn11	0,96	No
ENSMUSG00000037628	-7,67E-02	2,442047222	3,44E-01	5,58E-01	8,22E-01	Cdkn3	0,95	No
ENSMUSG00000037638	7,84E-02	4,954036911	1,13E+00	2,88E-01	5,83E-01	Zbtb42	1,06	No
ENSMUSG00000037640	1,78E-01	2,325824373	1,92E+00	1,65E-01	5,18E-01	Zfp60	1,13	No
ENSMUSG00000037643	3,58E-02	5,093019801	2,41E-01	6,23E-01	8,58E-01	Prkci	1,03	No
ENSMUSG00000037646	5,81E-02	7,437244675	9,54E-01	3,29E-01	6,29E-01	Vps13b	1,04	No
ENSMUSG00000037649	1,55E-01	9,086274408	7,42E+00	6,45E-03	6,76E-02	H2-DMa	1,11	No
ENSMUSG00000037652	-4,62E-04	6,312332222	5,28E-05	9,94E-01	1,00E+00	Phc3	1,00	No
ENSMUSG00000037656	1,79E-01	3,874464214	3,78E+00	5,20E-02	2,78E-01	Slc20a2	1,13	No
ENSMUSG00000037660	2,60E+00	-2,963275374	2,63E+00	1,05E-01	3,97E-01	Gdf7	6,08	No
ENSMUSG00000037661	2,26E-01	5,737540229	1,13E+01	7,84E-04	1,32E-02	Gpr160	1,17	Yes
ENSMUSG00000037664	-1,17E-01	-0,509963571	1,05E-01	7,46E-01	9,29E-01	Cdkn1c	0,92	No
ENSMUSG00000037669	1,08E-01	5,527639161	2,45E+00	1,17E-01	4,24E-01	Ldah	1,08	No
ENSMUSG00000037674	5,42E-02	6,792535669	7,78E-01	3,78E-01	6,78E-01	Rfx7	1,04	No
ENSMUSG00000037679	-1,48E-01	7,009180392	6,01E+00	1,42E-02	1,21E-01	Inf2	0,90	No
ENSMUSG00000037681	4,39E-01	4,056537538	2,42E+01	8,78E-07	3,98E-05	Esyt3	1,36	Yes
ENSMUSG00000037683	-6,18E-01	-1,088753419	1,93E+00	1,64E-01	5,16E-01	Armc3	0,65	No
ENSMUSG00000037685	1,53E-01	5,716499594	5,13E+00	2,36E-02	1,68E-01	Atp8a1	1,11	No
ENSMUSG00000037686	-1,75E+00	-2,770610262	2,13E+00	1,44E-01	4,80E-01	Aspg	0,30	No
ENSMUSG00000037692	2,03E-01	3,090378098	3,41E+00	6,49E-02	3,19E-01	Ahdc1	1,15	No
ENSMUSG00000037697	-1,19E-01	7,973558109	4,16E+00	4,15E-02	2,42E-01	Ddhd1	0,92	No
ENSMUSG00000037703	2,13E-01	1,641568119	1,56E+00	2,12E-01	5,50E-01	Lzts3	1,16	No

ENSMUSG00000037705	-3,18E+00	-2,83195675	4,25E+00	3,92E-02	2,35E-01	Tecta	0,11	No
ENSMUSG00000037706	5,81E-02	6,587997605	8,63E-01	3,53E-01	6,54E-01	Cd81	1,04	No
ENSMUSG00000037708	1,91E-01	-2,770641786	3,13E-02	8,60E-01	9,95E-01	Spag6	1,14	No
ENSMUSG00000037709	-2,72E-02	-1,015742533	4,06E-03	9,49E-01	1,00E+00	Fam13a	0,98	No
ENSMUSG00000037710	3,08E-02	4,553738775	1,47E-01	7,02E-01	9,05E-01	Cisd1	1,02	No
ENSMUSG00000037712	2,34E-01	-2,311092896	6,89E-02	7,93E-01	9,56E-01	Fermt2	1,18	No
ENSMUSG00000037716	-6,34E-01	-1,755401851	1,13E+00	2,88E-01	5,84E-01	Ccdc33	0,64	No
ENSMUSG00000037720	1,29E-02	6,054333615	3,93E-02	8,43E-01	9,85E-01	Tmem33	1,01	No
ENSMUSG00000037722	-2,17E-01	3,741442983	5,39E+00	2,02E-02	1,52E-01	Gnpnat1	0,86	No
ENSMUSG00000037725	2,44E-01	3,991188919	7,53E+00	6,07E-03	6,48E-02	Ckap2	1,18	No
ENSMUSG00000037730	1,42E-02	4,176961203	2,80E-02	8,67E-01	9,99E-01	Mynn	1,01	No
ENSMUSG00000037731	1,45E-01	7,291935231	5,94E+00	1,48E-02	1,25E-01	Themis2	1,11	No
ENSMUSG00000037736	-5,23E-02	-2,963273906	1,24E-03	9,72E-01	1,00E+00	Limch1	0,96	No
ENSMUSG00000037737	4,24E-02	-3,033592245	-1,17E-09	1,00E+00	1,00E+00	Actrt3	1,03	No
ENSMUSG00000037738	1,26E+00	-2,711710917	9,88E-01	3,20E-01	6,21E-01	Nek5	2,40	No
ENSMUSG00000037740	8,52E-02	4,881222768	1,26E+00	2,61E-01	5,55E-01	Mrps26	1,06	No
ENSMUSG00000037742	-1,90E-01	11,21507975	1,16E+01	6,50E-04	1,14E-02	Eef1a1	0,88	Yes
ENSMUSG00000037750	1,65E-01	4,04924943	3,54E+00	6,00E-02	3,03E-01	Fam222b	1,12	No
ENSMUSG00000037752	-3,81E-02	2,630292219	9,50E-02	7,58E-01	9,36E-01	Xkr8	0,97	No
ENSMUSG00000037754	-3,28E-01	3,025454581	8,62E+00	3,33E-03	4,14E-02	Ppp1r16b	0,80	Yes
ENSMUSG00000037759	1,07E-01	2,833479762	8,00E-01	3,71E-01	6,72E-01	Ptger2	1,08	No
ENSMUSG00000037761	2,27E-01	3,869123196	6,25E+00	1,24E-02	1,10E-01	Actr5	1,17	No
ENSMUSG00000037762	-5,15E-01	-1,107574049	1,26E+00	2,62E-01	5,55E-01	Slc16a9	0,70	No
ENSMUSG00000037771	-1,04E+00	-1,145899309	5,21E+00	2,25E-02	1,63E-01	Slc32a1	0,49	No
ENSMUSG00000037772	-2,20E-01	2,210821839	2,28E+00	1,31E-01	4,55E-01	Mrpl23	0,86	No
ENSMUSG00000037773	-8,35E-02	4,439976843	1,07E+00	3,02E-01	5,98E-01	Pced1a	0,94	No
ENSMUSG00000037784	-1,32E+00	-2,831992953	1,08E+00	2,99E-01	5,95E-01	Dzip1l	0,40	No
ENSMUSG00000037787	-5,22E-02	4,233851396	3,79E-01	5,38E-01	8,09E-01	Apopt1	0,96	No
ENSMUSG00000037788	-5,59E-01	7,550190791	8,94E+01	3,26E-21	8,27E-19	Vopp1	0,68	Yes
ENSMUSG00000037791	8,76E-03	6,167120741	1,86E-02	8,92E-01	1,00E+00	Phf12	1,01	No
ENSMUSG00000037795	4,97E-02	5,425156343	5,08E-01	4,76E-01	7,64E-01	N4bp2	1,04	No
ENSMUSG00000037797	1,87E+00	-3,107639691	1,38E+00	2,40E-01	5,50E-01	Adh4	3,67	No

ENSMUSG00000037801	2,27E+00	-2,47295873	4,26E+00	3,91E-02	2,35E-01	Iqch	4,82	No
ENSMUSG00000037805	-2,99E-02	6,326831936	2,22E-01	6,38E-01	8,68E-01	Rpl10a	0,98	No
ENSMUSG00000037808	-1,42E-02	5,691969624	4,40E-02	8,34E-01	9,79E-01	Fam76b	0,99	No
ENSMUSG00000037813	-2,65E+00	-2,963248364	2,80E+00	9,42E-02	3,84E-01	D630003M21Rik	0,16	No
ENSMUSG00000037815	-5,80E-02	8,620279511	1,02E+00	3,11E-01	6,11E-01	Ctnna1	0,96	No
ENSMUSG00000037816	-6,05E-02	5,263620646	7,29E-01	3,93E-01	6,93E-01	Fbxw17	0,96	No
ENSMUSG00000037818	-2,11E-01	3,505626821	4,65E+00	3,11E-02	2,04E-01	Abhd18	0,86	No
ENSMUSG00000037820	-2,07E-02	6,67097526	1,10E-01	7,40E-01	9,26E-01	Tgm2	0,99	No
ENSMUSG00000037822	-9,73E-02	5,69119774	2,08E+00	1,49E-01	4,90E-01	Smim14	0,93	No
ENSMUSG00000037824	2,26E-01	6,448861838	1,28E+01	3,46E-04	6,80E-03	Tspan14	1,17	Yes
ENSMUSG00000037826	7,64E-03	3,96969289	7,65E-03	9,30E-01	1,00E+00	Ppm1k	1,01	No
ENSMUSG00000037827	1,36E-01	5,56296671	3,89E+00	4,84E-02	2,65E-01	Gm5884	1,10	No
ENSMUSG00000037833	-5,00E-02	-0,226246123	2,57E-02	8,73E-01	1,00E+00	Sh2d4b	0,97	No
ENSMUSG00000037843	-3,83E-02	-3,107651936	-7,91E-11	1,00E+00	1,00E+00	Vstm2l	0,97	No
ENSMUSG00000037845	-1,24E-01	2,954212373	1,19E+00	2,74E-01	5,71E-01	Fdxacb1	0,92	No
ENSMUSG00000037846	9,28E-01	-1,175898028	3,92E+00	4,77E-02	2,62E-01	Rtkn2	1,90	No
ENSMUSG00000037847	-3,30E-01	2,741760116	7,33E+00	6,79E-03	7,03E-02	Nmrk1	0,80	No
ENSMUSG00000037849	2,04E-01	1,664115655	1,51E+00	2,19E-01	5,50E-01	Ifi206	1,15	No
ENSMUSG00000037851	2,50E-01	5,938739717	1,44E+01	1,50E-04	3,40E-03	Iars	1,19	Yes
ENSMUSG00000037852	3,04E-01	-2,600700455	9,41E-02	7,59E-01	9,36E-01	Cpe	1,23	No
ENSMUSG00000037855	-3,24E-01	1,164763072	2,77E+00	9,59E-02	3,87E-01	Zfp365	0,80	No
ENSMUSG00000037857	3,66E-02	6,242493692	3,29E-01	5,66E-01	8,28E-01	Nufip2	1,03	No
ENSMUSG00000037860	-1,12E-01	6,089535408	2,98E+00	8,44E-02	3,73E-01	Aim2	0,93	No
ENSMUSG00000037868	1,27E-01	8,536532017	4,87E+00	2,74E-02	1,87E-01	Egr2	1,09	No
ENSMUSG00000037872	-1,32E+00	-2,268063703	2,70E+00	1,00E-01	3,92E-01	Ackr1	0,40	No
ENSMUSG00000037876	-2,13E-01	7,308047056	1,26E+01	3,89E-04	7,47E-03	Jmjd1c	0,86	Yes
ENSMUSG00000037884	-2,26E-01	-1,929364539	1,13E-01	7,37E-01	9,25E-01	1700017G19Rik	0,85	No
ENSMUSG00000037885	-5,60E-02	5,635075171	6,74E-01	4,12E-01	7,07E-01	Stk35	0,96	No
ENSMUSG00000037887	-3,31E-01	-0,401164698	1,00E+00	3,17E-01	6,18E-01	Dusp8	0,79	No
ENSMUSG00000037890	3,45E-02	2,75695889	8,39E-02	7,72E-01	9,43E-01	Wdr19	1,02	No
ENSMUSG00000037892	-2,88E-02	-2,963268425	3,79E-04	9,84E-01	1,00E+00	Pcdh18	0,98	No
ENSMUSG00000037894	-2,89E-02	7,290660709	2,34E-01	6,28E-01	8,61E-01	H2afz	0,98	No

ENSMUSG00000037896	2,58E-02	6,681967897	1,73E-01	6,77E-01	8,92E-01	Rcor1	1,02	No
ENSMUSG00000037902	1,95E-01	9,868861978	1,20E+01	5,24E-04	9,53E-03	Sirpa	1,15	Yes
ENSMUSG00000037904	8,64E-02	3,19261411	6,58E-01	4,17E-01	7,12E-01	Ankrd9	1,06	No
ENSMUSG00000037905	3,91E-02	6,529854653	3,91E-01	5,32E-01	8,05E-01	Bri3bp	1,03	No
ENSMUSG00000037907	1,54E-01	5,969256671	5,43E+00	1,98E-02	1,50E-01	Ankrd13b	1,11	No
ENSMUSG00000037913	-7,09E-02	3,194061866	4,52E-01	5,01E-01	7,86E-01	Tmem156	0,95	No
ENSMUSG00000037916	-1,88E-02	6,088284223	8,38E-02	7,72E-01	9,43E-01	Ndufv1	0,99	No
ENSMUSG00000037921	-2,83E-02	2,624460317	5,20E-02	8,20E-01	9,71E-01	Ddx60	0,98	No
ENSMUSG00000037922	-3,11E-02	0,592676909	1,68E-02	8,97E-01	1,00E+00	Bank1	0,98	No
ENSMUSG00000037926	1,33E-01	6,320404534	4,34E+00	3,73E-02	2,30E-01	Ssh2	1,10	No
ENSMUSG00000037933	1,05E-01	6,963793031	2,99E+00	8,40E-02	3,71E-01	Bicd2	1,08	No
ENSMUSG00000037935	-1,94E-01	5,461215246	7,80E+00	5,21E-03	5,77E-02	Smarce1	0,87	No
ENSMUSG00000037936	1,79E-01	6,814174933	8,44E+00	3,67E-03	4,44E-02	Scarb1	1,13	Yes
ENSMUSG00000037938	-5,75E-02	3,321146335	3,08E-01	5,79E-01	8,36E-01	Chchd5	0,96	No
ENSMUSG00000037940	6,00E-01	-0,268216983	3,04E+00	8,10E-02	3,65E-01	Inpp4b	1,52	No
ENSMUSG00000037942	7,56E-01	-2,963268003	3,13E-01	5,76E-01	8,34E-01	Crp	1,69	No
ENSMUSG00000037944	-8,95E-01	8,824704396	2,42E+02	1,24E-54	4,64E-51	Ccr7	0,54	Yes
ENSMUSG00000037946	3,39E-01	5,798480674	2,56E+01	4,15E-07	2,02E-05	Fgd3	1,26	Yes
ENSMUSG00000037949	-4,58E-02	4,981727416	3,80E-01	5,37E-01	8,09E-01	Ano10	0,97	No
ENSMUSG00000037957	-1,11E-01	4,255908598	1,77E+00	1,84E-01	5,47E-01	Wdr20	0,93	No
ENSMUSG00000037958	-1,45E-01	4,277784393	3,03E+00	8,18E-02	3,67E-01	Nsrp1	0,90	No
ENSMUSG00000037960	1,11E-01	5,467603711	2,50E+00	1,14E-01	4,16E-01	Card19	1,08	No
ENSMUSG00000037962	6,56E-01	-0,226321505	3,57E+00	5,87E-02	2,99E-01	Rflna	1,58	No
ENSMUSG00000037965	-1,10E-01	7,592004283	3,45E+00	6,32E-02	3,14E-01	Zc3h7a	0,93	No
ENSMUSG00000037966	-9,98E-02	6,332828143	2,46E+00	1,17E-01	4,23E-01	Ninj1	0,93	No
ENSMUSG00000037971	-1,38E-01	3,684999397	2,12E+00	1,45E-01	4,83E-01	1110032A03Rik	0,91	No
ENSMUSG00000037972	-5,98E-01	4,675011594	5,99E+01	1,01E-14	1,49E-12	Snn	0,66	Yes
ENSMUSG00000037974	1,81E+00	-2,963282131	1,30E+00	2,54E-01	5,50E-01	Muc5ac	3,50	No
ENSMUSG00000037977	-7,25E-01	3,325327164	4,38E+01	3,72E-11	3,73E-09	6430571L13Rik	0,60	Yes
ENSMUSG00000037979	2,21E-01	0,087235608	5,98E-01	4,39E-01	7,32E-01	Ccdc92	1,17	No
ENSMUSG00000037982	2,21E-01	2,614009251	3,06E+00	8,00E-02	3,62E-01	Gm9725	1,17	No
ENSMUSG00000037989	-8,06E-02	1,971094529	2,73E-01	6,01E-01	8,47E-01	Wnk2	0,95	No

ENSMUSG00000037990	3,11E+00	-2,83203996	3,94E+00	4,71E-02	2,60E-01	Sh3rf3	8,62	No
ENSMUSG00000037991	1,68E-01	3,272352237	2,59E+00	1,07E-01	4,01E-01	Rmi2	1,12	No
ENSMUSG00000037992	2,46E-01	7,155676998	1,65E+01	4,79E-05	1,31E-03	Rara	1,19	Yes
ENSMUSG00000037993	2,34E-02	6,55591044	1,40E-01	7,08E-01	9,09E-01	Dhx38	1,02	No
ENSMUSG00000037994	3,54E+00	-2,655104838	5,43E+00	1,98E-02	1,50E-01	Slc9b2	11,61	No
ENSMUSG00000037995	-1,75E-01	4,539410687	4,92E+00	2,65E-02	1,82E-01	Igsf9	0,89	No
ENSMUSG00000037997	-1,30E-01	4,548918987	2,75E+00	9,74E-02	3,89E-01	Parp11	0,91	No
ENSMUSG00000037999	-5,70E-01	5,823121473	7,36E+01	9,67E-18	1,89E-15	Arap2	0,67	Yes
ENSMUSG00000038000	1,43E-01	4,811951932	3,47E+00	6,24E-02	3,11E-01	Acd	1,10	No
ENSMUSG00000038002	4,25E-02	6,110297743	4,32E-01	5,11E-01	7,94E-01	Cramp1l	1,03	No
ENSMUSG00000038005	1,23E-01	4,904363151	2,65E+00	1,03E-01	3,95E-01	Hpf1	1,09	No
ENSMUSG00000038007	-5,68E-01	-1,896044848	7,05E-01	4,01E-01	6,99E-01	Acer2	0,67	No
ENSMUSG00000038009	-3,60E-01	-1,548323112	3,60E-01	5,48E-01	8,15E-01	Dnajc22	0,78	No
ENSMUSG00000038010	1,08E-01	1,045279648	2,91E-01	5,90E-01	8,41E-01	Ccdc138	1,08	No
ENSMUSG00000038011	3,68E-01	-0,448799059	1,10E+00	2,95E-01	5,90E-01	Dnah10	1,29	No
ENSMUSG00000038013	-1,95E-02	5,472759963	7,99E-02	7,77E-01	9,47E-01	Wipf2	0,99	No
ENSMUSG00000038014	7,30E-02	8,660148285	1,62E+00	2,02E-01	5,50E-01	Fam120a	1,05	No
ENSMUSG00000038015	-7,80E-01	-1,509946451	1,97E+00	1,60E-01	5,08E-01	Prm2	0,58	No
ENSMUSG00000038020	4,82E-01	-0,424967475	1,96E+00	1,62E-01	5,11E-01	Rapgefl1	1,40	No
ENSMUSG00000038022	1,79E-01	-0,042869465	3,73E-01	5,42E-01	8,11E-01	Mindy4	1,13	No
ENSMUSG00000038023	2,63E-01	6,9765354	1,87E+01	1,55E-05	4,92E-04	Atp6v0a2	1,20	Yes
ENSMUSG00000038024	2,04E-01	6,329265734	1,02E+01	1,41E-03	2,13E-02	Dennd4c	1,15	Yes
ENSMUSG00000038025	1,36E-02	5,881625088	4,22E-02	8,37E-01	9,81E-01	Phf2	1,01	No
ENSMUSG00000038026	5,32E-01	-0,793292546	1,54E+00	2,15E-01	5,50E-01	Kcnj9	1,45	No
ENSMUSG00000038028	5,40E-02	4,964516932	5,31E-01	4,66E-01	7,54E-01	Tigar	1,04	No
ENSMUSG00000038034	2,00E-01	7,025103508	1,08E+01	1,01E-03	1,63E-02	Igsf8	1,15	Yes
ENSMUSG00000038037	-8,72E-02	5,080790185	1,46E+00	2,27E-01	5,50E-01	Socs1	0,94	No
ENSMUSG00000038039	1,12E-01	5,173567817	2,40E+00	1,21E-01	4,34E-01	Gcc2	1,08	No
ENSMUSG00000038042	-1,78E-01	1,672557671	1,14E+00	2,86E-01	5,82E-01	Ptpdc1	0,88	No
ENSMUSG00000038044	-4,13E-02	-3,033659495	7,08E-04	9,79E-01	1,00E+00	Cct8l1	0,97	No
ENSMUSG00000038045	-2,76E-02	0,392641385	1,14E-02	9,15E-01	1,00E+00	Sult6b1	0,98	No
ENSMUSG00000038046	4,29E-01	1,58798499	6,50E+00	1,08E-02	9,87E-02	Mrm3	1,35	No

ENSMUSG00000038047	2,90E-01	4,604303329	1,35E+01	2,36E-04	4,99E-03	Haus6	1,22	Yes
ENSMUSG00000038055	-8,74E-02	5,552596327	1,64E+00	2,01E-01	5,50E-01	Dexi	0,94	No
ENSMUSG00000038056	2,90E-04	6,882359034	2,25E-05	9,96E-01	1,00E+00	Kmt2c	1,00	No
ENSMUSG00000038057	-9,30E-01	-2,711675158	6,14E-01	4,33E-01	7,27E-01	Dbil5	0,52	No
ENSMUSG00000038058	2,60E-01	3,99205221	8,47E+00	3,61E-03	4,39E-02	Nod1	1,20	Yes
ENSMUSG00000038059	-2,27E-01	6,448424317	1,28E+01	3,47E-04	6,81E-03	Smim3	0,85	Yes
ENSMUSG00000038060	1,21E-01	0,409209655	2,40E-01	6,24E-01	8,59E-01	Dlec1	1,09	No
ENSMUSG00000038064	2,27E+00	-2,963288049	1,97E+00	1,60E-01	5,09E-01	Cldn22	4,81	No
ENSMUSG00000038065	-7,61E-02	3,339224411	5,59E-01	4,55E-01	7,44E-01	Mturn	0,95	No
ENSMUSG00000038067	1,59E+00	-2,600675254	1,74E+00	1,88E-01	5,50E-01	Csf3	3,01	No
ENSMUSG00000038068	2,87E-01	4,929045796	1,46E+01	1,30E-04	3,03E-03	Rnf144b	1,22	Yes
ENSMUSG00000038069	-5,20E-02	3,92394781	3,32E-01	5,64E-01	8,27E-01	Cdkn2aip	0,96	No
ENSMUSG00000038070	1,23E-01	3,383008311	1,48E+00	2,24E-01	5,50E-01	Cntln	1,09	No
ENSMUSG00000038072	8,94E-03	5,140888545	1,54E-02	9,01E-01	1,00E+00	Galnt11	1,01	No
ENSMUSG00000038074	4,82E-01	1,017265909	5,25E+00	2,20E-02	1,60E-01	Fkbp14	1,40	No
ENSMUSG00000038077	-1,87E+00	-3,107637761	1,39E+00	2,38E-01	5,50E-01	Kcna6	0,27	No
ENSMUSG00000038079	3,46E-01	2,591608149	7,47E+00	6,26E-03	6,63E-02	Tmem237	1,27	No
ENSMUSG00000038080	-2,72E-02	5,474305907	1,56E-01	6,93E-01	9,01E-01	Kdm1b	0,98	No
ENSMUSG00000038084	-5,83E-03	6,125007131	8,13E-03	9,28E-01	1,00E+00	Opa1	1,00	No
ENSMUSG00000038085	8,30E-02	4,951816334	1,22E+00	2,69E-01	5,65E-01	Cnbd2	1,06	No
ENSMUSG00000038086	4,44E-02	-1,472929592	6,21E-03	9,37E-01	1,00E+00	Hspb2	1,03	No
ENSMUSG00000038094	-1,92E-01	0,897243052	8,75E-01	3,50E-01	6,51E-01	Atp13a4	0,88	No
ENSMUSG00000038095	-4,09E-02	7,241459664	4,66E-01	4,95E-01	7,80E-01	Sbno1	0,97	No
ENSMUSG00000038102	7,38E-02	6,022645915	1,28E+00	2,58E-01	5,50E-01	Trappc11	1,05	No
ENSMUSG00000038112	-1,87E+00	-3,03364178	1,41E+00	2,36E-01	5,50E-01	AW551984	0,27	No
ENSMUSG00000038115	1,24E+00	-0,929368192	7,11E+00	7,66E-03	7,66E-02	Ano2	2,35	No
ENSMUSG00000038116	-1,21E-01	5,194266727	2,85E+00	9,13E-02	3,82E-01	Phf20	0,92	No
ENSMUSG00000038119	-2,19E-01	3,419873356	4,89E+00	2,70E-02	1,84E-01	Cdon	0,86	No
ENSMUSG00000038121	1,79E-01	5,401609613	6,54E+00	1,05E-02	9,72E-02	Fam210a	1,13	No
ENSMUSG00000038122	1,12E-02	3,523185856	1,27E-02	9,10E-01	1,00E+00	Tbc1d32	1,01	No
ENSMUSG00000038126	6,19E-02	4,083956298	5,03E-01	4,78E-01	7,66E-01	Mphosph9	1,04	No
ENSMUSG00000038127	-1,14E-01	7,738602815	3,78E+00	5,20E-02	2,78E-01	Ccdc50	0,92	No

ENSMUSG00000038128	1,67E-01	-0,165861074	2,80E-01	5,97E-01	8,45E-01	Camk4	1,12	No
ENSMUSG00000038132	1,85E+00	-3,107657832	1,35E+00	2,46E-01	5,50E-01	Rbm24	3,61	No
ENSMUSG00000038135	-2,85E-01	-1,548166013	2,47E-01	6,19E-01	8,56E-01	Crygn	0,82	No
ENSMUSG00000038141	1,72E-01	5,275837942	5,85E+00	1,56E-02	1,29E-01	Tmem181a	1,13	No
ENSMUSG00000038143	-3,29E-01	1,432600113	3,52E+00	6,07E-02	3,05E-01	Stox2	0,80	No
ENSMUSG00000038145	8,72E-02	5,172873005	1,48E+00	2,23E-01	5,50E-01	Snrk	1,06	No
ENSMUSG00000038146	-5,82E-03	-0,541887915	2,39E-04	9,88E-01	1,00E+00	Notch3	1,00	No
ENSMUSG00000038147	1,33E-01	8,380634591	5,30E+00	2,13E-02	1,57E-01	Cd84	1,10	No
ENSMUSG00000038150	1,36E-01	3,615798752	2,00E+00	1,57E-01	5,02E-01	Ormdl3	1,10	No
ENSMUSG00000038151	-1,01E-01	5,369898513	2,02E+00	1,55E-01	4,99E-01	Prdm1	0,93	No
ENSMUSG00000038152	-2,54E-01	-1,051727072	3,54E-01	5,52E-01	8,18E-01	5033430I15Rik	0,84	No
ENSMUSG00000038155	-2,06E-01	1,63789072	1,51E+00	2,19E-01	5,50E-01	Gstp2	0,87	No
ENSMUSG00000038156	6,18E-02	1,382439038	1,19E-01	7,31E-01	9,21E-01	Spon1	1,04	No
ENSMUSG00000038160	1,44E-02	5,80693476	4,67E-02	8,29E-01	9,76E-01	Atg5	1,01	No
ENSMUSG00000038167	7,95E-02	1,327686789	1,68E-01	6,81E-01	8,94E-01	Plekhg6	1,06	No
ENSMUSG00000038168	-5,15E-01	1,488429566	9,55E+00	2,00E-03	2,80E-02	P3h2	0,70	Yes
ENSMUSG00000038170	-6,10E-02	5,606585758	8,13E-01	3,67E-01	6,68E-01	Pde4dip	0,96	No
ENSMUSG00000038172	-2,73E-02	6,318282974	1,84E-01	6,68E-01	8,87E-01	Ttc39b	0,98	No
ENSMUSG00000038173	9,95E-01	-0,535587986	5,93E+00	1,49E-02	1,25E-01	Enpp6	1,99	No
ENSMUSG00000038174	2,45E-02	4,09583489	8,03E-02	7,77E-01	9,46E-01	Fam126b	1,02	No
ENSMUSG00000038175	4,37E-01	4,253238624	2,63E+01	2,97E-07	1,49E-05	Mylip	1,35	Yes
ENSMUSG00000038178	2,30E-01	7,891616549	1,55E+01	8,12E-05	2,04E-03	Slc43a2	1,17	Yes
ENSMUSG00000038179	-2,43E-02	7,568090854	1,70E-01	6,81E-01	8,93E-01	Slamf7	0,98	No
ENSMUSG00000038180	8,79E-01	-1,963321155	1,19E+00	2,76E-01	5,73E-01	Spag4	1,84	No
ENSMUSG00000038181	9,15E-02	6,346438396	2,07E+00	1,50E-01	4,91E-01	Chpf2	1,07	No
ENSMUSG00000038187	-4,92E-02	4,622206733	3,93E-01	5,31E-01	8,05E-01	Btbd10	0,97	No
ENSMUSG00000038188	-1,67E-01	4,396132613	4,20E+00	4,05E-02	2,38E-01	Scarf1	0,89	No
ENSMUSG00000038195	1,37E-01	2,887134721	1,41E+00	2,35E-01	5,50E-01	Rilp	1,10	No
ENSMUSG00000038199	2,65E+00	-2,896160752	2,71E+00	9,96E-02	3,92E-01	4931409K22Rik	6,29	No
ENSMUSG00000038201	-5,54E-01	-2,711664916	2,40E-01	6,24E-01	8,59E-01	Kcna7	0,68	No
ENSMUSG00000038204	-7,46E-01	-2,548214788	6,23E-01	4,30E-01	7,23E-01	Asb10	0,60	No
ENSMUSG00000038205	1,70E-01	3,256050013	2,65E+00	1,04E-01	3,96E-01	Prkab2	1,12	No

ENSMUSG00000038206	8,08E-02	4,356375758	9,46E-01	3,31E-01	6,31E-01	Fbxo8	1,06	No
ENSMUSG00000038208	1,47E-01	2,085040148	9,76E-01	3,23E-01	6,23E-01	Pgap3	1,11	No
ENSMUSG00000038209	-1,94E+00	-3,107619243	1,50E+00	2,21E-01	5,50E-01	Itln1	0,26	No
ENSMUSG00000038212	4,11E-02	6,854766409	4,51E-01	5,02E-01	7,87E-01	Mfsd14b	1,03	No
ENSMUSG00000038213	-3,03E-01	4,914196729	1,65E+01	4,77E-05	1,31E-03	Tapbpl	0,81	Yes
ENSMUSG00000038214	2,11E-02	3,303606552	4,01E-02	8,41E-01	9,84E-01	Bend3	1,01	No
ENSMUSG00000038215	9,81E-03	3,436605822	9,46E-03	9,23E-01	1,00E+00	Cep44	1,01	No
ENSMUSG00000038217	4,58E-02	2,511348396	1,28E-01	7,20E-01	9,16E-01	Tlcd2	1,03	No
ENSMUSG00000038224	4,01E-01	-0,816441575	9,23E-01	3,37E-01	6,37E-01	Serpinf2	1,32	No
ENSMUSG00000038225	-1,98E-01	3,554093281	4,17E+00	4,12E-02	2,41E-01	Primpol	0,87	No
ENSMUSG00000038227	-2,70E+00	-2,770604422	2,88E+00	8,96E-02	3,80E-01	Hoxa9	0,15	No
ENSMUSG00000038233	-6,14E-01	2,192217085	2,06E+01	5,76E-06	2,05E-04	Fam198a	0,65	Yes
ENSMUSG00000038235	3,81E-01	4,48445709	2,20E+01	2,66E-06	1,04E-04	F11r	1,30	Yes
ENSMUSG00000038236	4,18E-01	-2,683158522	1,37E-01	7,12E-01	9,11E-01	Hoxa7	1,34	No
ENSMUSG00000038239	6,15E-01	-2,088875291	7,67E-01	3,81E-01	6,83E-01	Hrc	1,53	No
ENSMUSG00000038240	-3,24E-02	2,495776999	6,27E-02	8,02E-01	9,61E-01	Pdss2	0,98	No
ENSMUSG00000038241	1,24E-01	7,022079333	4,18E+00	4,10E-02	2,40E-01	Cep250	1,09	No
ENSMUSG00000038242	1,84E+00	-3,107648531	1,33E+00	2,49E-01	5,50E-01	Aox4	3,58	No
ENSMUSG00000038244	3,83E-01	6,623430976	3,74E+01	9,85E-10	8,07E-08	Mical2	1,30	Yes
ENSMUSG00000038248	-1,36E-01	3,458460032	1,87E+00	1,72E-01	5,28E-01	Sobp	0,91	No
ENSMUSG00000038250	1,73E-01	5,401804289	6,13E+00	1,33E-02	1,15E-01	Usp38	1,13	No
ENSMUSG00000038252	2,30E-01	6,735826954	1,39E+01	1,94E-04	4,24E-03	Ncapd2	1,17	Yes
ENSMUSG00000038253	3,11E-01	-2,070085709	1,78E-01	6,73E-01	8,90E-01	Hoxa5	1,24	No
ENSMUSG00000038256	-1,34E-01	4,20786302	2,50E+00	1,14E-01	4,17E-01	Bcl9	0,91	No
ENSMUSG00000038257	-7,38E-02	-3,033634644	2,66E-03	9,59E-01	1,00E+00	Glra3	0,95	No
ENSMUSG00000038259	-1,87E+00	-3,107637761	1,39E+00	2,38E-01	5,50E-01	Gdf5	0,27	No
ENSMUSG00000038260	2,36E-01	4,537169204	8,61E+00	3,35E-03	4,15E-02	Trpm4	1,18	Yes
ENSMUSG00000038264	-9,38E-01	5,446491792	1,84E+02	6,61E-42	9,48E-39	Sema7a	0,52	Yes
ENSMUSG00000038267	-7,13E-01	5,894877326	1,17E+02	2,67E-27	1,18E-24	Slc22a23	0,61	Yes
ENSMUSG00000038268	3,58E-02	3,890654021	1,56E-01	6,93E-01	9,01E-01	Ovca2	1,03	No
ENSMUSG00000038271	-1,05E-01	5,039067767	2,08E+00	1,49E-01	4,90E-01	Iffo1	0,93	No
ENSMUSG00000038274	-3,34E-01	5,116009307	2,11E+01	4,27E-06	1,58E-04	Fau	0,79	Yes

ENSMUSG00000038276	-2,86E-02	-0,26802302	7,88E-03	9,29E-01	1,00E+00	Asic3	0,98	No
ENSMUSG00000038279	9,77E-02	5,315766777	1,92E+00	1,66E-01	5,19E-01	Nop2	1,07	No
ENSMUSG00000038280	1,08E-01	6,670021328	3,03E+00	8,17E-02	3,67E-01	Ostm1	1,08	No
ENSMUSG00000038286	-2,38E-01	3,228069356	5,21E+00	2,25E-02	1,63E-01	Bphl	0,85	No
ENSMUSG00000038290	-1,77E-01	5,034225003	5,83E+00	1,57E-02	1,30E-01	Smg6	0,88	No
ENSMUSG00000038291	1,29E-01	5,170383387	3,18E+00	7,46E-02	3,47E-01	Snx25	1,09	No
ENSMUSG00000038292	6,08E-01	-2,497668314	4,12E-01	5,21E-01	7,99E-01	Ccdc155	1,52	No
ENSMUSG00000038295	3,19E-01	1,909448404	4,18E+00	4,10E-02	2,40E-01	Atg9b	1,25	No
ENSMUSG00000038296	1,22E+00	-2,770635322	9,26E-01	3,36E-01	6,37E-01	Galnt18	2,33	No
ENSMUSG00000038298	2,01E-01	2,128616718	1,89E+00	1,69E-01	5,24E-01	Pdzk1	1,15	No
ENSMUSG00000038299	1,93E-02	4,75314317	6,20E-02	8,03E-01	9,62E-01	Wdr36	1,01	No
ENSMUSG00000038301	-2,61E-02	6,475700219	1,73E-01	6,78E-01	8,92E-01	Snx10	0,98	No
ENSMUSG00000038302	2,23E-01	3,315212798	4,45E+00	3,49E-02	2,21E-01	Afg1l	1,17	No
ENSMUSG00000038304	-1,41E-01	0,710580932	3,34E-01	5,63E-01	8,26E-01	Cd160	0,91	No
ENSMUSG00000038312	1,00E-01	6,217213665	2,43E+00	1,19E-01	4,30E-01	Edem2	1,07	No
ENSMUSG00000038319	-8,37E-01	-2,832007507	3,84E-01	5,35E-01	8,08E-01	Kcnh2	0,56	No
ENSMUSG00000038323	2,53E-01	3,323965723	6,11E+00	1,34E-02	1,16E-01	1700066M21Rik	1,19	No
ENSMUSG00000038324	-1,86E-02	7,328881407	9,68E-02	7,56E-01	9,34E-01	Trpc4ap	0,99	No
ENSMUSG00000038331	1,37E-01	1,615785664	6,30E-01	4,28E-01	7,21E-01	Satb2	1,10	No
ENSMUSG00000038332	7,84E-02	4,473645284	9,44E-01	3,31E-01	6,32E-01	Sesn1	1,06	No
ENSMUSG00000038335	5,83E-02	5,15167082	6,47E-01	4,21E-01	7,16E-01	Tsr1	1,04	No
ENSMUSG00000038342	-1,09E-02	7,358844037	3,35E-02	8,55E-01	9,92E-01	Mlxip	0,99	No
ENSMUSG00000038346	4,67E-02	6,16547001	5,23E-01	4,69E-01	7,58E-01	Zfp384	1,03	No
ENSMUSG00000038347	1,31E-01	0,206768175	2,22E-01	6,37E-01	8,68E-01	Tcte2	1,09	No
ENSMUSG00000038349	-2,13E-01	2,796422864	3,20E+00	7,38E-02	3,45E-01	Plcl1	0,86	No
ENSMUSG00000038351	-1,95E-01	4,485624443	5,80E+00	1,60E-02	1,32E-01	Sgsm2	0,87	No
ENSMUSG00000038352	-1,92E-01	6,009394518	8,74E+00	3,11E-03	3,94E-02	Arl5c	0,88	Yes
ENSMUSG00000038354	-6,80E-01	0,563415067	7,91E+00	4,92E-03	5,53E-02	Ankrd35	0,62	No
ENSMUSG00000038357	-1,87E+00	-2,448655261	4,10E+00	4,28E-02	2,46E-01	Camp	0,27	No
ENSMUSG00000038365	4,92E-02	3,668948763	2,68E-01	6,05E-01	8,49E-01	Fbxo25	1,03	No
ENSMUSG00000038366	-3,62E-02	8,573422734	3,99E-01	5,27E-01	8,04E-01	Lasp1	0,98	No
ENSMUSG00000038368	1,92E-01	3,747595392	4,14E+00	4,20E-02	2,44E-01	Focad	1,14	No

ENSMUSG00000038369	-3,71E-02	7,056530462	3,76E-01	5,40E-01	8,10E-01	Ncoa6	0,97	No
ENSMUSG00000038370	-5,65E-01	-1,83200669	6,99E-01	4,03E-01	7,01E-01	Pcp4l1	0,68	No
ENSMUSG00000038371	-3,15E-02	6,143746205	2,38E-01	6,26E-01	8,60E-01	Sbf2	0,98	No
ENSMUSG00000038372	1,26E-01	3,03421666	1,27E+00	2,60E-01	5,53E-01	Gmds	1,09	No
ENSMUSG00000038374	-4,10E-02	4,137780254	2,29E-01	6,32E-01	8,64E-01	Rbm8a	0,97	No
ENSMUSG00000038375	9,60E-02	6,761037594	2,43E+00	1,19E-01	4,30E-01	Trp53inp2	1,07	No
ENSMUSG00000038379	1,72E-01	3,291094695	2,78E+00	9,55E-02	3,86E-01	Ttk	1,13	No
ENSMUSG00000038383	1,24E-01	4,323357818	2,22E+00	1,37E-01	4,66E-01	Pigu	1,09	No
ENSMUSG00000038384	1,76E-01	6,109859065	7,46E+00	6,29E-03	6,65E-02	Setd1b	1,13	No
ENSMUSG00000038387	-7,97E-02	4,867085571	1,11E+00	2,92E-01	5,87E-01	Rras	0,95	No
ENSMUSG00000038388	1,41E-01	6,441636899	5,01E+00	2,52E-02	1,76E-01	Mpp6	1,10	No
ENSMUSG00000038390	-2,04E-01	0,848960648	9,03E-01	3,42E-01	6,42E-01	Gpr162	0,87	No
ENSMUSG00000038393	4,83E-02	8,566720815	7,08E-01	4,00E-01	6,99E-01	Txnip	1,03	No
ENSMUSG00000038398	-1,32E-02	4,332175249	2,51E-02	8,74E-01	1,00E+00	Upf3a	0,99	No
ENSMUSG00000038400	3,10E-01	3,278297035	8,76E+00	3,07E-03	3,90E-02	Pmepa1	1,24	Yes
ENSMUSG00000038402	7,50E-01	1,913851432	2,33E+01	1,41E-06	6,00E-05	Foxf2	1,68	Yes
ENSMUSG00000038406	-4,33E-02	6,739638879	4,95E-01	4,82E-01	7,69E-01	Scaf1	0,97	No
ENSMUSG00000038408	1,26E+00	-2,448689246	1,57E+00	2,10E-01	5,50E-01	1700018A04Rik	2,40	No
ENSMUSG00000038412	2,95E-01	3,189572404	7,45E+00	6,35E-03	6,69E-02	Higd1a	1,23	No
ENSMUSG00000038415	-1,04E-01	0,483554364	1,65E-01	6,85E-01	8,97E-01	Foxq1	0,93	No
ENSMUSG00000038416	9,59E-02	4,84592721	1,60E+00	2,06E-01	5,50E-01	Cdc16	1,07	No
ENSMUSG00000038417	1,78E-01	5,431959711	6,44E+00	1,12E-02	1,01E-01	Fig4	1,13	No
ENSMUSG00000038418	5,61E-01	7,776425933	8,88E+01	4,48E-21	1,11E-18	Egr1	1,48	Yes
ENSMUSG00000038421	4,40E-02	1,120288461	4,85E-02	8,26E-01	9,74E-01	Fcrla	1,03	No
ENSMUSG00000038422	1,05E-01	-0,268090964	1,08E-01	7,43E-01	9,28E-01	Hdhd3	1,08	No
ENSMUSG00000038425	1,20E-03	3,270040998	1,38E-04	9,91E-01	1,00E+00	Poli	1,00	No
ENSMUSG00000038429	7,75E-02	6,654232479	1,56E+00	2,11E-01	5,50E-01	Usp5	1,06	No
ENSMUSG00000038437	-2,99E-01	8,2117328	2,67E+01	2,36E-07	1,22E-05	Mllt6	0,81	Yes
ENSMUSG00000038446	1,41E-01	5,041847481	3,67E+00	5,55E-02	2,89E-01	Cdc40	1,10	No
ENSMUSG00000038451	8,56E-02	3,886617837	8,81E-01	3,48E-01	6,49E-01	Spsb2	1,06	No
ENSMUSG00000038453	-3,66E-01	-2,185609931	1,69E-01	6,81E-01	8,94E-01	Srcin1	0,78	No
ENSMUSG00000038456	3,19E-01	5,060993802	1,85E+01	1,70E-05	5,36E-04	Dennd2a	1,25	Yes

ENSMUSG00000038457	7,46E-01	-2,015634632	9,94E-01	3,19E-01	6,19E-01	Tmem255b	1,68	No
ENSMUSG00000038459	2,16E-02	5,430997386	9,47E-02	7,58E-01	9,36E-01	Abhd17c	1,02	No
ENSMUSG00000038462	1,21E-01	7,119554043	4,02E+00	4,50E-02	2,53E-01	Uqcrfs1	1,09	No
ENSMUSG00000038463	8,41E-02	0,789012409	1,46E-01	7,03E-01	9,06E-01	Olfml2b	1,06	No
ENSMUSG00000038467	-1,89E-02	7,731783088	1,03E-01	7,48E-01	9,30E-01	Chmp4b	0,99	No
ENSMUSG00000038473	-1,86E-02	1,912462802	1,49E-02	9,03E-01	1,00E+00	Nos1ap	0,99	No
ENSMUSG00000038481	5,29E-02	5,863142475	6,33E-01	4,26E-01	7,20E-01	Cdk19	1,04	No
ENSMUSG00000038482	3,31E-02	5,900103102	2,52E-01	6,15E-01	8,54E-01	Tfdp1	1,02	No
ENSMUSG00000038485	2,20E-02	5,585580783	1,04E-01	7,47E-01	9,30E-01	Socs7	1,02	No
ENSMUSG00000038486	-9,92E-01	-1,268049738	4,07E+00	4,38E-02	2,49E-01	Sv2a	0,50	No
ENSMUSG00000038489	9,21E-02	3,291671445	7,60E-01	3,83E-01	6,85E-01	Polr2l	1,07	No
ENSMUSG00000038491	-1,49E-01	-0,793157631	1,35E-01	7,13E-01	9,11E-01	Cdk19os	0,90	No
ENSMUSG00000038495	-1,93E-01	4,965296996	6,96E+00	8,32E-03	8,16E-02	Otud7b	0,87	No
ENSMUSG00000038497	1,74E-02	5,104830435	5,66E-02	8,12E-01	9,67E-01	Tmco3	1,01	No
ENSMUSG00000038500	2,64E-02	4,282037884	9,98E-02	7,52E-01	9,32E-01	Prr3	1,02	No
ENSMUSG00000038502	-1,06E-01	5,497642631	2,35E+00	1,26E-01	4,44E-01	Ptov1	0,93	No
ENSMUSG00000038503	2,26E-01	5,413733673	1,05E+01	1,22E-03	1,91E-02	Mesd	1,17	Yes
ENSMUSG00000038506	1,37E-01	4,716857542	3,16E+00	7,56E-02	3,50E-01	Dcun1d2	1,10	No
ENSMUSG00000038507	4,05E-02	5,821084022	3,73E-01	5,42E-01	8,11E-01	Parp12	1,03	No
ENSMUSG00000038508	1,05E-01	4,725044489	1,85E+00	1,74E-01	5,31E-01	Gdf15	1,08	No
ENSMUSG00000038510	3,36E-02	2,656524011	7,20E-02	7,88E-01	9,54E-01	Rpf2	1,02	No
ENSMUSG00000038515	-3,75E-02	6,385351545	3,50E-01	5,54E-01	8,20E-01	Grtp1	0,97	No
ENSMUSG00000038517	1,24E-01	5,681468124	3,33E+00	6,80E-02	3,28E-01	Tbkbp1	1,09	No
ENSMUSG00000038518	-2,61E-01	5,825086762	1,53E+01	9,14E-05	2,25E-03	Jarid2	0,83	Yes
ENSMUSG00000038520	-6,15E-01	7,465269855	1,08E+02	2,79E-25	1,01E-22	Tbc1d17	0,65	Yes
ENSMUSG00000038521	-5,07E-01	-1,278608176	1,07E+00	3,00E-01	5,97E-01	C1s1	0,70	No
ENSMUSG00000038522	2,25E+00	-1,879942223	8,20E+00	4,18E-03	4,89E-02	Mfsd4b1	4,76	Yes
ENSMUSG00000038523	7,09E-01	-1,088840451	2,43E+00	1,19E-01	4,29E-01	1700003F12Rik	1,63	No
ENSMUSG00000038524	-6,31E-02	3,974110637	5,06E-01	4,77E-01	7,65E-01	Fchsd1	0,96	No
ENSMUSG00000038525	-9,33E-02	4,986860908	1,60E+00	2,06E-01	5,50E-01	Armc10	0,94	No
ENSMUSG00000038526	9,83E-02	1,363890081	2,72E-01	6,02E-01	8,48E-01	Car14	1,07	No
ENSMUSG00000038527	-4,69E-02	2,63120277	1,41E-01	7,07E-01	9,08E-01	C1rl	0,97	No

ENSMUSG00000038528	1,44E-01	-1,146190981	1,02E-01	7,49E-01	9,31E-01	Mfsd4b5		1,11	No
ENSMUSG00000038530	-8,32E-01	-2,896143825	3,80E-01	5,38E-01	8,09E-01	Rgs4		0,56	No
ENSMUSG00000038533	9,39E-02	4,718062885	1,46E+00	2,27E-01	5,50E-01	Cbfa2t2		1,07	No
ENSMUSG00000038534	1,02E-01	4,485354313	1,63E+00	2,01E-01	5,50E-01	Osbpl7		1,07	No
ENSMUSG00000038535	-2,14E-02	4,376191083	6,88E-02	7,93E-01	9,56E-01	Zfp280d		0,99	No
ENSMUSG00000038538	5,60E-02	6,884997404	8,42E-01	3,59E-01	6,60E-01	Ubn2		1,04	No
ENSMUSG00000038539	9,82E-03	4,7913965	1,65E-02	8,98E-01	1,00E+00	Atf5		1,01	No
ENSMUSG00000038540	-4,87E-02	-0,824093444	1,48E-02	9,03E-01	1,00E+00	Tmc3		0,97	No
ENSMUSG00000038542	8,89E-02	5,312464094	1,56E+00	2,11E-01	5,50E-01	Pcid2		1,06	No
ENSMUSG00000038543	2,16E-01	3,829361807	5,37E+00	2,05E-02	1,54E-01	BC028528		1,16	No
ENSMUSG00000038544	9,98E-02	5,332134013	2,00E+00	1,57E-01	5,02E-01	Inip		1,07	No
ENSMUSG00000038545	-2,97E-01	4,320221991	1,31E+01	2,96E-04	5,98E-03	Cul7		0,81	Yes
ENSMUSG00000038546	-1,72E-02	6,379624262	7,43E-02	7,85E-01	9,52E-01	Ranbp9		0,99	No
ENSMUSG00000038550	4,44E-01	1,210167085	5,41E+00	2,00E-02	1,51E-01	Ciart		1,36	No
ENSMUSG00000038552	-5,27E-01	-0,587129942	2,11E+00	1,47E-01	4,85E-01	Fndc4		0,69	No
ENSMUSG00000038555	2,44E-01	-1,627653791	2,01E-01	6,54E-01	8,79E-01	Reep2		1,18	No
ENSMUSG00000038560	-6,42E-01	-2,333204796	6,30E-01	4,27E-01	7,21E-01	Sp6		0,64	No
ENSMUSG00000038563	1,24E-01	4,108658505	2,04E+00	1,53E-01	4,96E-01	Efl1		1,09	No
ENSMUSG00000038564	2,70E-02	4,06509154	9,45E-02	7,58E-01	9,36E-01	Ift172		1,02	No
ENSMUSG00000038567	-1,90E+00	-3,033647263	1,44E+00	2,30E-01	5,50E-01	Cyp24a1		0,27	No
ENSMUSG00000038569	1,72E-01	2,468383641	1,73E+00	1,88E-01	5,50E-01	Rad9b		1,13	No
ENSMUSG00000038570	4,17E-01	-1,896146068	3,79E-01	5,38E-01	8,09E-01	Saxo2		1,34	No
ENSMUSG00000038576	2,77E-01	3,988167549	9,47E+00	2,09E-03	2,90E-02	Susd4		1,21	Yes
ENSMUSG00000038578	-1,61E-01	3,747399993	2,98E+00	8,44E-02	3,73E-01	Susd1		0,89	No
ENSMUSG00000038582	4,60E-02	5,422530521	4,38E-01	5,08E-01	7,92E-01	Pptc7		1,03	No
ENSMUSG00000038583	-1,78E+00	-1,497402194	9,04E+00	2,64E-03	3,47E-02	Pln		0,29	Yes
ENSMUSG00000038587	-2,00E-01	0,053756179	5,11E-01	4,75E-01	7,63E-01	Akap12		0,87	No
ENSMUSG00000038593	1,16E-02	7,327661041	3,80E-02	8,45E-01	9,87E-01	Tctn1		1,01	No
ENSMUSG00000038594	-5,56E-01	5,167867216	5,95E+01	1,19E-14	1,76E-12	Cep85l		0,68	Yes
ENSMUSG00000038598	-9,12E-02	-2,355552568	9,46E-03	9,23E-01	1,00E+00	AI481877		0,94	No
ENSMUSG00000038599	-8,78E-02	-2,963255397	3,51E-03	9,53E-01	1,00E+00	Capn8		0,94	No
ENSMUSG00000038600	-1,27E+00	-3,146104694	7,49E-01	3,87E-01	6,87E-01	Atp6v0a4		0,42	No

ENSMUSG00000038604	1,77E-01	5,6880446	6,82E+00	9,01E-03	8,65E-02	Ripor1	1,13	No
ENSMUSG00000038605	-2,48E-01	3,874266853	7,47E+00	6,28E-03	6,64E-02	Samd10	0,84	No
ENSMUSG00000038607	-6,57E-02	5,545666152	9,14E-01	3,39E-01	6,40E-01	Gng10	0,96	No
ENSMUSG00000038608	-5,21E-02	8,571185998	8,26E-01	3,63E-01	6,64E-01	Dock10	0,96	No
ENSMUSG00000038611	7,81E-02	6,232489637	1,48E+00	2,23E-01	5,50E-01	Phrf1	1,06	No
ENSMUSG00000038612	-6,51E-02	9,460840242	1,33E+00	2,49E-01	5,50E-01	Mcl1	0,96	No
ENSMUSG00000038615	-6,69E-02	8,010975698	1,33E+00	2,50E-01	5,50E-01	Nfe2l1	0,95	No
ENSMUSG00000038618	2,37E-01	2,642579497	3,72E+00	5,37E-02	2,83E-01	Rassf7	1,18	No
ENSMUSG00000038619	-1,26E-01	7,221124909	4,46E+00	3,47E-02	2,20E-01	Ensa	0,92	No
ENSMUSG00000038622	-1,63E-01	3,737868065	2,95E+00	8,58E-02	3,77E-01	Med30	0,89	No
ENSMUSG00000038623	2,51E-01	7,417218572	1,77E+01	2,57E-05	7,71E-04	Tm6sf1	1,19	Yes
ENSMUSG00000038628	2,24E-02	4,784019048	8,53E-02	7,70E-01	9,42E-01	Polr3k	1,02	No
ENSMUSG00000038630	-1,89E+00	-3,1076441	1,42E+00	2,33E-01	5,50E-01	Zkscan16	0,27	No
ENSMUSG00000038633	9,02E-02	8,242164096	2,43E+00	1,19E-01	4,28E-01	Degs1	1,06	No
ENSMUSG00000038637	-1,57E-03	2,797946807	1,79E-04	9,89E-01	1,00E+00	Lrrc56	1,00	No
ENSMUSG00000038642	1,52E-01	11,49664179	7,37E+00	6,62E-03	6,90E-02	Ctss	1,11	No
ENSMUSG00000038644	9,83E-02	5,70001428	2,13E+00	1,45E-01	4,81E-01	Pold1	1,07	No
ENSMUSG00000038646	1,27E-02	4,537508956	2,56E-02	8,73E-01	1,00E+00	Fam103a1	1,01	No
ENSMUSG00000038648	1,43E-01	4,431768468	3,06E+00	8,03E-02	3,63E-01	Creb3l2	1,10	No
ENSMUSG00000038650	1,49E-01	9,096773292	6,85E+00	8,85E-03	8,54E-02	Rnh1	1,11	No
ENSMUSG00000038651	8,10E-01	-1,424934318	2,26E+00	1,33E-01	4,59E-01	Sycp2l	1,75	No
ENSMUSG00000038658	-1,10E-01	7,381892313	3,42E+00	6,42E-02	3,17E-01	Ric1	0,93	No
ENSMUSG00000038663	-9,75E-01	-0,668971269	5,73E+00	1,66E-02	1,34E-01	Fsd2	0,51	No
ENSMUSG00000038664	7,66E-02	7,138812741	1,62E+00	2,04E-01	5,50E-01	Herc1	1,05	No
ENSMUSG00000038665	3,53E-01	-1,311216136	5,03E-01	4,78E-01	7,66E-01	Dgki	1,28	No
ENSMUSG00000038668	-2,10E-01	0,575246975	7,02E-01	4,02E-01	7,00E-01	Lpar1	0,86	No
ENSMUSG00000038670	7,75E-02	-1,963209535	1,32E-02	9,08E-01	1,00E+00	Mybpc2	1,06	No
ENSMUSG00000038671	-2,15E-01	4,695618565	7,82E+00	5,18E-03	5,74E-02	Arfrp1	0,86	No
ENSMUSG00000038677	1,73E-01	-1,627710526	8,36E-02	7,72E-01	9,43E-01	Scube3	1,13	No
ENSMUSG00000038679	4,51E-02	5,774053792	4,52E-01	5,01E-01	7,86E-01	Trps1	1,03	No
ENSMUSG00000038683	1,02E-01	5,462901228	2,15E+00	1,42E-01	4,77E-01	Pak1ip1	1,07	No
ENSMUSG00000038685	1,23E-02	4,599246542	2,45E-02	8,76E-01	1,00E+00	Rtel1	1,01	No

ENSMUSG00000038690	-2,30E-01	5,448158478	1,08E+01	9,90E-04	1,60E-02	Atp5j2	0,85	Yes
ENSMUSG00000038692	2,66E-01	1,51422376	2,43E+00	1,19E-01	4,29E-01	Hoxb4	1,20	No
ENSMUSG00000038695	-2,53E-02	4,547488021	1,01E-01	7,50E-01	9,31E-01	Josd2	0,98	No
ENSMUSG00000038696	-2,33E-01	5,990046813	1,27E+01	3,64E-04	7,08E-03	Mapkap1	0,85	Yes
ENSMUSG00000038697	9,66E-02	5,173410774	1,76E+00	1,84E-01	5,48E-01	Taf5l	1,07	No
ENSMUSG00000038702	9,35E-02	3,448633296	8,60E-01	3,54E-01	6,55E-01	Dsel	1,07	No
ENSMUSG00000038704	-8,97E-01	-2,832022722	4,41E-01	5,07E-01	7,91E-01	Aspdh	0,54	No
ENSMUSG00000038705	-2,53E-01	4,957383065	1,14E+01	7,33E-04	1,25E-02	Gmeb2	0,84	Yes
ENSMUSG00000038708	7,53E-02	7,060639204	1,55E+00	2,14E-01	5,50E-01	Golga4	1,05	No
ENSMUSG00000038712	-1,24E-02	6,542662881	3,92E-02	8,43E-01	9,85E-01	Mindy1	0,99	No
ENSMUSG00000038717	-1,12E-01	5,054053481	2,30E+00	1,29E-01	4,52E-01	Atp5l	0,93	No
ENSMUSG00000038718	2,41E-02	3,8284661	6,92E-02	7,92E-01	9,55E-01	Pbx3	1,02	No
ENSMUSG00000038721	1,78E+00	-2,963287612	1,28E+00	2,58E-01	5,50E-01	Hoxb7	3,44	No
ENSMUSG00000038722	-2,52E-02	5,691740829	1,38E-01	7,10E-01	9,09E-01	Bud31	0,98	No
ENSMUSG00000038725	-2,65E+00	-3,033633091	2,78E+00	9,55E-02	3,86E-01	Pkhd1l1	0,16	No
ENSMUSG00000038729	-8,04E-01	3,406311368	6,37E+01	1,41E-15	2,30E-13	Akap2	0,57	Yes
ENSMUSG00000038732	4,76E-01	3,563172367	2,30E+01	1,61E-06	6,74E-05	Mboat1	1,39	Yes
ENSMUSG00000038733	1,42E-03	8,197610126	6,03E-04	9,80E-01	1,00E+00	Wdr26	1,00	No
ENSMUSG00000038736	-1,64E-02	3,826000231	3,20E-02	8,58E-01	9,94E-01	Nudcd1	0,99	No
ENSMUSG00000038738	-1,45E-02	0,53005987	3,70E-03	9,52E-01	1,00E+00	Shank1	0,99	No
ENSMUSG00000038740	-5,50E-02	6,449722941	7,61E-01	3,83E-01	6,84E-01	Mvb12b	0,96	No
ENSMUSG00000038742	-3,24E-02	0,789117359	2,24E-02	8,81E-01	1,00E+00	Angptl6	0,98	No
ENSMUSG00000038745	-8,93E-01	-2,770572856	4,35E-01	5,09E-01	7,93E-01	Nlrp6	0,54	No
ENSMUSG00000038751	-1,51E+00	3,008919547	1,80E+02	3,82E-41	4,77E-38	Ptk6	0,35	Yes
ENSMUSG00000038754	-7,61E-01	-2,548188001	6,50E-01	4,20E-01	7,15E-01	Elovl3	0,59	No
ENSMUSG00000038759	1,85E-01	6,630133166	8,81E+00	2,99E-03	3,83E-02	Nup205	1,14	Yes
ENSMUSG00000038762	1,14E-01	6,570034316	3,32E+00	6,85E-02	3,29E-01	Abcf1	1,08	No
ENSMUSG00000038763	-2,85E-01	-2,355592432	1,21E-01	7,28E-01	9,20E-01	Alpk3	0,82	No
ENSMUSG00000038764	-3,21E-01	4,610493536	1,68E+01	4,12E-05	1,15E-03	Ptpn3	0,80	Yes
ENSMUSG00000038765	-1,34E+00	-2,896122948	1,12E+00	2,89E-01	5,85E-01	Lmx1b	0,39	No
ENSMUSG00000038766	1,19E-01	7,029706615	3,90E+00	4,84E-02	2,65E-01	Gabpb2	1,09	No
ENSMUSG00000038770	3,62E-01	-0,510135279	9,88E-01	3,20E-01	6,21E-01	Kpna7	1,29	No

ENSMUSG00000038773	-2,62E-02	7,06144414	1,89E-01	6,64E-01	8,84E-01	Kdm3b	0,98	No
ENSMUSG00000038774	2,55E-02	5,806713841	1,46E-01	7,03E-01	9,06E-01	Ascc3	1,02	No
ENSMUSG00000038775	-9,52E-03	4,143868224	1,20E-02	9,13E-01	1,00E+00	Vill	0,99	No
ENSMUSG00000038776	6,88E-02	6,564279486	1,21E+00	2,71E-01	5,67E-01	Ephx1	1,05	No
ENSMUSG00000038777	-1,75E+00	-0,573866605	2,14E+01	3,75E-06	1,42E-04	Sema6c	0,30	Yes
ENSMUSG00000038780	-8,20E-02	7,259501052	1,88E+00	1,70E-01	5,26E-01	Smurf1	0,94	No
ENSMUSG00000038781	-5,00E-01	4,727280857	4,29E+01	5,82E-11	5,72E-09	Stap2	0,71	Yes
ENSMUSG00000038782	1,92E-01	2,834002009	2,60E+00	1,07E-01	4,00E-01	1700028J19Rik	1,14	No
ENSMUSG00000038784	1,81E-01	4,546676369	5,29E+00	2,14E-02	1,58E-01	Cnot4	1,13	No
ENSMUSG00000038793	2,01E-01	-0,180645486	3,67E-01	5,45E-01	8,13E-01	Lefty1	1,15	No
ENSMUSG00000038797	-3,30E-01	1,202948883	3,21E+00	7,32E-02	3,44E-01	Zscan2	0,80	No
ENSMUSG00000038801	7,43E-01	-2,963279568	3,02E-01	5,83E-01	8,38E-01	Scgb1c1	1,67	No
ENSMUSG00000038803	6,07E-02	4,974354516	6,64E-01	4,15E-01	7,10E-01	Ost4	1,04	No
ENSMUSG00000038805	1,81E+00	-3,10765737	1,28E+00	2,58E-01	5,50E-01	Six3	3,49	No
ENSMUSG00000038806	-1,38E-02	5,508030951	3,94E-02	8,43E-01	9,85E-01	Sde2	0,99	No
ENSMUSG00000038807	-1,99E-02	2,621858612	2,53E-02	8,74E-01	1,00E+00	Rap1gap2	0,99	No
ENSMUSG00000038811	-3,49E-01	5,559608488	2,61E+01	3,22E-07	1,60E-05	Gngt2	0,79	Yes
ENSMUSG00000038812	-1,87E-01	3,583616508	3,59E+00	5,83E-02	2,98E-01	Trmt112	0,88	No
ENSMUSG00000038816	1,84E-01	1,221924279	9,18E-01	3,38E-01	6,38E-01	Ctnnal1	1,14	No
ENSMUSG00000038822	-4,92E-02	4,049762278	3,12E-01	5,76E-01	8,35E-01	Hace1	0,97	No
ENSMUSG00000038827	1,06E-01	4,547441267	1,79E+00	1,81E-01	5,42E-01	Fam206a	1,08	No
ENSMUSG00000038828	2,15E-01	6,309772346	1,14E+01	7,39E-04	1,26E-02	Tmem214	1,16	Yes
ENSMUSG00000038831	-3,15E-02	2,363989541	5,46E-02	8,15E-01	9,69E-01	Ralgps1	0,98	No
ENSMUSG00000038836	-3,72E-02	0,432359725	2,32E-02	8,79E-01	1,00E+00	Agbl3	0,97	No
ENSMUSG00000038838	2,00E-02	2,928508598	3,09E-02	8,60E-01	9,96E-01	Vars2	1,01	No
ENSMUSG00000038843	1,13E-01	7,075075443	3,49E+00	6,16E-02	3,09E-01	Gcnt1	1,08	No
ENSMUSG00000038844	-3,96E-02	5,705485454	3,43E-01	5,58E-01	8,22E-01	Kif16b	0,97	No
ENSMUSG00000038845	1,61E-01	4,305633544	3,71E+00	5,42E-02	2,85E-01	Phb	1,12	No
ENSMUSG00000038848	4,51E-02	6,003517696	4,75E-01	4,91E-01	7,77E-01	Ythdf1	1,03	No
ENSMUSG00000038855	-1,30E-01	6,621703672	4,40E+00	3,59E-02	2,25E-01	Itpkb	0,91	No
ENSMUSG00000038859	1,54E-01	-0,060836503	2,59E-01	6,11E-01	8,52E-01	Baiap2l1	1,11	No
ENSMUSG00000038860	-9,08E-02	1,419350547	2,59E-01	6,11E-01	8,52E-01	Garnl3	0,94	No

ENSMUSG00000038861	6,48E-02	5,850938673	9,58E-01	3,28E-01	6,29E-01	Pi4kb	1,05	No
ENSMUSG00000038866	-2,92E-02	5,964069135	1,97E-01	6,57E-01	8,80E-01	Zcchc2	0,98	No
ENSMUSG00000038871	5,19E-02	3,510883811	2,72E-01	6,02E-01	8,48E-01	Bpgm	1,04	No
ENSMUSG00000038872	6,44E-02	5,453567003	8,38E-01	3,60E-01	6,61E-01	Zfhx3	1,05	No
ENSMUSG00000038876	-8,91E-02	5,166205472	1,54E+00	2,15E-01	5,50E-01	Rnf146	0,94	No
ENSMUSG00000038880	-1,62E-01	4,097657563	3,49E+00	6,18E-02	3,09E-01	Mrps34	0,89	No
ENSMUSG00000038884	1,32E-01	2,41422841	1,01E+00	3,16E-01	6,16E-01	A230050P20Rik	1,10	No
ENSMUSG00000038886	2,61E-01	7,655605706	1,95E+01	1,02E-05	3,43E-04	Man2a2	1,20	Yes
ENSMUSG00000038888	1,07E-01	3,055136476	9,57E-01	3,28E-01	6,29E-01	Ctu1	1,08	No
ENSMUSG00000038893	-6,67E-02	3,018206494	3,51E-01	5,54E-01	8,19E-01	Fam117a	0,95	No
ENSMUSG00000038894	3,60E-01	6,35184874	3,22E+01	1,40E-08	9,38E-07	Irs2	1,28	Yes
ENSMUSG00000038895	9,00E-03	3,460715683	8,10E-03	9,28E-01	1,00E+00	Zfp653	1,01	No
ENSMUSG00000038900	-5,77E-02	4,171064604	4,41E-01	5,07E-01	7,91E-01	Rpl12	0,96	No
ENSMUSG00000038902	2,70E-02	5,021920915	1,35E-01	7,13E-01	9,11E-01	Pogz	1,02	No
ENSMUSG00000038909	6,15E-03	6,660866324	9,82E-03	9,21E-01	1,00E+00	Kat7	1,00	No
ENSMUSG00000038910	-3,26E-01	6,401136875	2,66E+01	2,44E-07	1,26E-05	Plcl2	0,80	Yes
ENSMUSG00000038914	1,29E-01	6,284295323	4,12E+00	4,23E-02	2,45E-01	Dido1	1,09	No
ENSMUSG00000038917	8,07E-01	-1,770669779	1,81E+00	1,78E-01	5,39E-01	3930402G23Rik	1,75	No
ENSMUSG00000038930	1,31E-01	4,093986094	2,30E+00	1,30E-01	4,52E-01	Rccd1	1,10	No
ENSMUSG00000038932	-1,27E+00	-3,070178353	7,36E-01	3,91E-01	6,91E-01	Tcfl5	0,42	No
ENSMUSG00000038936	-1,03E-01	3,624585112	1,17E+00	2,80E-01	5,76E-01	Sccpdh	0,93	No
ENSMUSG00000038943	2,45E-01	5,820799014	1,36E+01	2,30E-04	4,88E-03	Prc1	1,19	Yes
ENSMUSG00000038949	-4,96E-02	4,884510802	4,39E-01	5,07E-01	7,92E-01	Cnst	0,97	No
ENSMUSG00000038954	-3,02E-01	2,021995483	3,95E+00	4,68E-02	2,59E-01	Supt3	0,81	No
ENSMUSG00000038957	2,75E-02	5,573418109	1,64E-01	6,86E-01	8,97E-01	Edc3	1,02	No
ENSMUSG00000038963	-2,83E-01	3,915644415	9,75E+00	1,79E-03	2,56E-02	Slco4a1	0,82	Yes
ENSMUSG00000038965	-1,93E-02	6,971858428	1,01E-01	7,51E-01	9,32E-01	Ube2l3	0,99	No
ENSMUSG00000038967	-7,07E-03	3,410863079	4,77E-03	9,45E-01	1,00E+00	Pdk2	1,00	No
ENSMUSG00000038968	1,17E-01	-2,070229147	2,69E-02	8,70E-01	1,00E+00	Klk1b16	1,08	No
ENSMUSG00000038970	1,27E-02	5,847883155	3,63E-02	8,49E-01	9,88E-01	Lmtk2	1,01	No
ENSMUSG00000038973	5,25E-01	0,193946532	3,88E+00	4,89E-02	2,66E-01	Cldnd2	1,44	No
ENSMUSG00000038975	4,16E-02	4,913948116	3,06E-01	5,80E-01	8,37E-01	Rabggtb	1,03	No

ENSMUSG00000038976	1,39E-01	7,935745427	5,66E+00	1,73E-02	1,37E-01	Ppp1r9b	1,10	No
ENSMUSG00000038982	2,32E-01	3,860578767	6,41E+00	1,14E-02	1,03E-01	Bloc1s5	1,17	No
ENSMUSG00000038984	1,65E-01	-2,311218732	4,09E-02	8,40E-01	9,83E-01	Tspyl5	1,12	No
ENSMUSG00000038987	2,20E-01	2,139403236	2,29E+00	1,30E-01	4,52E-01	Cfap157	1,16	No
ENSMUSG00000038990	-1,51E-02	4,870073035	4,00E-02	8,42E-01	9,84E-01	Cables2	0,99	No
ENSMUSG00000038991	5,38E-02	6,623476287	7,47E-01	3,87E-01	6,87E-01	Txndc5	1,04	No
ENSMUSG00000038997	2,31E+00	-3,070182045	2,04E+00	1,53E-01	4,96E-01	Asb17	4,95	No
ENSMUSG00000039000	8,59E-02	5,624832735	1,58E+00	2,08E-01	5,50E-01	Ube3c	1,06	No
ENSMUSG00000039001	-9,82E-02	0,763300101	1,87E-01	6,65E-01	8,85E-01	Rps21	0,93	No
ENSMUSG00000039004	-1,51E-01	-2,05176781	4,37E-02	8,34E-01	9,79E-01	Bmp6	0,90	No
ENSMUSG00000039005	1,09E-01	7,665022352	3,44E+00	6,38E-02	3,16E-01	Tlr4	1,08	No
ENSMUSG00000039007	1,93E-01	4,333997558	5,30E+00	2,13E-02	1,57E-01	Cpq	1,14	No
ENSMUSG00000039013	2,86E-01	5,650342589	1,75E+01	2,92E-05	8,56E-04	Siglecf	1,22	Yes
ENSMUSG00000039016	-3,46E-02	4,887352889	2,10E-01	6,47E-01	8,74E-01	Timm8b	0,98	No
ENSMUSG00000039018	6,16E-02	2,826821726	2,74E-01	6,01E-01	8,47E-01	Mtg1	1,04	No
ENSMUSG00000039021	3,61E-02	1,864397618	5,32E-02	8,18E-01	9,70E-01	Ttc16	1,03	No
ENSMUSG00000039031	1,38E-01	5,633691802	4,13E+00	4,21E-02	2,44E-01	Arhgap18	1,10	No
ENSMUSG00000039032	1,85E+00	-3,107657832	1,35E+00	2,46E-01	5,50E-01	Tsga13	3,61	No
ENSMUSG00000039033	2,19E-01	2,06068579	2,09E+00	1,49E-01	4,89E-01	Tasp1	1,16	No
ENSMUSG00000039037	1,81E-02	-1,91261285	6,65E-04	9,79E-01	1,00E+00	St6galnac5	1,01	No
ENSMUSG00000039041	1,22E-02	4,981873983	2,67E-02	8,70E-01	1,00E+00	Adrm1	1,01	No
ENSMUSG00000039043	-4,47E-01	4,58008702	3,32E+01	8,25E-09	5,82E-07	Arpin	0,73	Yes
ENSMUSG00000039046	-1,32E-02	5,009791115	3,15E-02	8,59E-01	9,95E-01	Usp6nl	0,99	No
ENSMUSG00000039047	9,67E-02	5,756192431	2,07E+00	1,50E-01	4,91E-01	Pigk	1,07	No
ENSMUSG00000039048	-5,56E-02	4,867220974	5,44E-01	4,61E-01	7,50E-01	Foxred1	0,96	No
ENSMUSG00000039050	-9,95E-03	4,932273698	1,80E-02	8,93E-01	1,00E+00	Osbpl2	0,99	No
ENSMUSG00000039055	1,64E-01	4,178794318	3,68E+00	5,52E-02	2,88E-01	Eme1	1,12	No
ENSMUSG00000039057	2,94E-01	-2,600644909	8,78E-02	7,67E-01	9,40E-01	Myo16	1,23	No
ENSMUSG00000039058	8,15E-01	-2,333251082	6,88E-01	4,07E-01	7,03E-01	Ak5	1,76	No
ENSMUSG00000039059	-1,89E+00	-3,1076441	1,42E+00	2,33E-01	5,50E-01	Hrh3	0,27	No
ENSMUSG00000039062	1,78E-01	9,230593184	9,80E+00	1,74E-03	2,51E-02	Anpep	1,13	Yes
ENSMUSG00000039063	2,19E-01	0,609783129	9,51E-01	3,30E-01	6,30E-01	Echdc3	1,16	No

ENSMUSG00000039065	-3,28E-02	4,021587471	1,43E-01	7,06E-01	9,07E-01	Fam173b	0,98	No
ENSMUSG00000039067	4,54E-02	5,95920908	4,78E-01	4,89E-01	7,76E-01	Psmd7	1,03	No
ENSMUSG00000039068	6,86E-02	5,73236282	1,04E+00	3,07E-01	6,05E-01	Zzz3	1,05	No
ENSMUSG00000039069	1,48E-01	3,233659233	2,04E+00	1,53E-01	4,96E-01	Mtg2	1,11	No
ENSMUSG00000039079	-1,87E+00	-3,107637761	1,39E+00	2,38E-01	5,50E-01	Trhr2	0,27	No
ENSMUSG00000039081	9,18E-02	1,986320817	3,76E-01	5,40E-01	8,10E-01	Zfp503	1,07	No
ENSMUSG00000039084	4,69E-01	-2,355624058	2,98E-01	5,85E-01	8,39E-01	Chad	1,38	No
ENSMUSG00000039086	-1,53E-01	3,898655201	2,97E+00	8,51E-02	3,74E-01	Ss18l1	0,90	No
ENSMUSG00000039087	2,59E-01	6,639541472	1,73E+01	3,27E-05	9,46E-04	Rreb1	1,20	Yes
ENSMUSG00000039089	1,96E-02	3,198921369	3,39E-02	8,54E-01	9,92E-01	L3mbtl3	1,01	No
ENSMUSG00000039095	-9,94E-01	2,116608024	4,99E+01	1,62E-12	1,93E-10	En2	0,50	Yes
ENSMUSG00000039096	-9,15E-02	2,761009119	5,83E-01	4,45E-01	7,36E-01	Rsad1	0,94	No
ENSMUSG00000039097	-2,43E-01	-2,03362573	1,16E-01	7,33E-01	9,23E-01	Rln1	0,85	No
ENSMUSG00000039099	-1,89E+00	-3,033648118	1,42E+00	2,33E-01	5,50E-01	Wdr93	0,27	No
ENSMUSG00000039100	5,09E-02	6,695076084	6,78E-01	4,10E-01	7,07E-01	March6	1,04	No
ENSMUSG00000039103	-4,73E-01	-1,548075893	7,77E-01	3,78E-01	6,79E-01	Nexn	0,72	No
ENSMUSG00000039105	-3,39E-02	6,826320037	3,06E-01	5,80E-01	8,37E-01	Atp6v1g1	0,98	No
ENSMUSG00000039108	2,09E-02	4,882093048	7,82E-02	7,80E-01	9,48E-01	Lsm14b	1,01	No
ENSMUSG00000039109	1,47E+00	4,621194849	2,84E+02	8,00E-64	5,99E-60	F13a1	2,77	Yes
ENSMUSG00000039110	6,12E-01	-0,770643077	2,42E+00	1,20E-01	4,30E-01	Mycbpap	1,53	No
ENSMUSG00000039114	-1,00E-01	-0,929286145	5,67E-02	8,12E-01	9,67E-01	Nrn1	0,93	No
ENSMUSG00000039115	-7,87E-01	-1,929241623	1,26E+00	2,61E-01	5,54E-01	Itga9	0,58	No
ENSMUSG00000039116	-7,09E-01	6,449020421	1,27E+02	1,65E-29	8,99E-27	Adgrg6	0,61	Yes
ENSMUSG00000039117	9,98E-02	5,410539752	2,05E+00	1,53E-01	4,95E-01	Taf4	1,07	No
ENSMUSG00000039126	3,58E-01	6,902282879	3,40E+01	5,63E-09	4,11E-07	Prune2	1,28	Yes
ENSMUSG00000039128	1,01E-02	5,070182535	1,90E-02	8,90E-01	1,00E+00	Cdc123	1,01	No
ENSMUSG00000039130	-1,60E-01	4,686133974	4,12E+00	4,24E-02	2,45E-01	Zc3hc1	0,90	No
ENSMUSG00000039131	2,02E+00	-1,71175682	7,10E+00	7,70E-03	7,68E-02	Gipc2	4,05	No
ENSMUSG00000039133	5,45E-01	-2,015684773	6,24E-01	4,29E-01	7,23E-01	9330171B17Rik	1,46	No
ENSMUSG00000039137	-6,95E-01	2,500184007	2,81E+01	1,16E-07	6,34E-06	Whrn	0,62	Yes
ENSMUSG00000039145	2,94E-01	5,841223518	1,93E+01	1,10E-05	3,66E-04	Camk1d	1,23	Yes
ENSMUSG00000039148	-3,05E-02	5,865962506	2,11E-01	6,46E-01	8,74E-01	Sart1	0,98	No

ENSMUSG00000039153	-7,72E-02	5,827550742	1,32E+00	2,51E-01	5,50E-01	Runx2	0,95	No
ENSMUSG00000039154	-7,80E-01	-1,57400692	1,80E+00	1,80E-01	5,42E-01	Shd	0,58	No
ENSMUSG00000039155	-1,02E+00	-2,146135971	1,97E+00	1,61E-01	5,09E-01	Cdh26	0,49	No
ENSMUSG00000039156	1,67E-02	5,329912396	5,65E-02	8,12E-01	9,67E-01	Stim2	1,01	No
ENSMUSG00000039157	-5,11E-02	4,159764433	3,63E-01	5,47E-01	8,14E-01	Fam102a	0,97	No
ENSMUSG00000039158	2,72E-02	7,359425979	2,09E-01	6,47E-01	8,75E-01	Akna	1,02	No
ENSMUSG00000039159	3,19E-03	5,756377333	2,27E-03	9,62E-01	1,00E+00	Ube2h	1,00	No
ENSMUSG00000039163	-1,28E-01	3,991673082	2,06E+00	1,51E-01	4,93E-01	Cmc1	0,92	No
ENSMUSG00000039164	1,68E-01	3,489339398	2,85E+00	9,13E-02	3,82E-01	Naif1	1,12	No
ENSMUSG00000039166	5,13E-02	3,396696664	2,52E-01	6,16E-01	8,54E-01	Akap7	1,04	No
ENSMUSG00000039167	-3,16E+00	-2,963246013	4,20E+00	4,04E-02	2,38E-01	Adgrl4	0,11	No
ENSMUSG00000039168	-1,39E-01	7,42988569	5,50E+00	1,90E-02	1,46E-01	Dap	0,91	No
ENSMUSG00000039176	3,69E-01	5,592421272	2,87E+01	8,27E-08	4,70E-06	Polg	1,29	Yes
ENSMUSG00000039178	-1,84E-01	2,478766247	2,06E+00	1,52E-01	4,94E-01	Tbc1d19	0,88	No
ENSMUSG00000039179	-1,27E+00	-2,548213249	1,55E+00	2,12E-01	5,50E-01	Tekt5	0,41	No
ENSMUSG00000039182	1,33E-01	2,699438814	1,19E+00	2,76E-01	5,73E-01	AW209491	1,10	No
ENSMUSG00000039183	5,80E-02	6,045964267	7,95E-01	3,73E-01	6,74E-01	Nubp2	1,04	No
ENSMUSG00000039187	2,90E-01	3,717643008	9,23E+00	2,37E-03	3,21E-02	Fanci	1,22	Yes
ENSMUSG00000039191	-1,50E-01	8,634965896	6,83E+00	8,95E-03	8,60E-02	Rbpj	0,90	No
ENSMUSG00000039193	2,24E-01	4,144888845	6,78E+00	9,21E-03	8,78E-02	Nlrc4	1,17	No
ENSMUSG00000039194	-8,30E-01	-2,896162806	3,77E-01	5,39E-01	8,09E-01	Rlbp1	0,56	No
ENSMUSG00000039195	9,98E-02	5,039958476	1,83E+00	1,76E-01	5,35E-01	1110008P14Rik	1,07	No
ENSMUSG00000039196	-7,47E-02	0,010808198	6,55E-02	7,98E-01	9,59E-01	Orm1	0,95	No
ENSMUSG00000039197	2,55E-01	4,471779076	9,73E+00	1,81E-03	2,58E-02	Adk	1,19	Yes
ENSMUSG00000039198	9,01E-03	-3,107646452	-5,16E-10	1,00E+00	1,00E+00	Ptchd3	1,01	No
ENSMUSG00000039199	3,04E-01	4,675494175	1,51E+01	1,02E-04	2,47E-03	Zdhhc1	1,23	Yes
ENSMUSG00000039200	-8,70E-01	-1,963285748	1,36E+00	2,44E-01	5,50E-01	Atf7ip2	0,55	No
ENSMUSG00000039202	-9,22E-02	6,523520819	2,16E+00	1,41E-01	4,75E-01	Abhd2	0,94	No
ENSMUSG00000039205	1,29E-01	6,097977349	3,97E+00	4,62E-02	2,57E-01	Ciz1	1,09	No
ENSMUSG00000039206	2,48E-01	7,200239537	1,70E+01	3,66E-05	1,04E-03	Daglb	1,19	Yes
ENSMUSG00000039208	5,87E-03	6,386956082	8,58E-03	9,26E-01	1,00E+00	Metrnl	1,00	No
ENSMUSG00000039209	-4,11E-01	-0,711682629	1,19E+00	2,75E-01	5,71E-01	Rpl39l	0,75	No

ENSMUSG00000039210	6,33E-02	4,156111796	5,45E-01	4,60E-01	7,49E-01	Gpatch2	1,04	No
ENSMUSG00000039217	1,51E-01	2,150988885	1,02E+00	3,12E-01	6,12E-01	Il18	1,11	No
ENSMUSG00000039218	-7,42E-02	9,57714487	1,73E+00	1,89E-01	5,50E-01	Srrm2	0,95	No
ENSMUSG00000039219	-4,98E-02	5,583309045	5,29E-01	4,67E-01	7,55E-01	Arid4b	0,97	No
ENSMUSG00000039220	2,08E-01	6,134800143	1,04E+01	1,26E-03	1,95E-02	Ppp1r10	1,16	Yes
ENSMUSG00000039221	-1,08E-01	3,573438512	1,24E+00	2,66E-01	5,61E-01	Rpl22l1	0,93	No
ENSMUSG00000039224	1,86E-02	1,117832414	8,40E-03	9,27E-01	1,00E+00	D1Pas1	1,01	No
ENSMUSG00000039230	-8,80E-02	5,431552677	1,58E+00	2,08E-01	5,50E-01	Tbcd	0,94	No
ENSMUSG00000039232	-6,06E-01	4,454892118	5,84E+01	2,14E-14	3,09E-12	Stx11	0,66	Yes
ENSMUSG00000039233	-2,92E-02	4,743961646	1,44E-01	7,05E-01	9,07E-01	Tbce	0,98	No
ENSMUSG00000039234	2,22E-01	5,295806196	9,71E+00	1,83E-03	2,61E-02	Sec24d	1,17	Yes
ENSMUSG00000039236	-1,04E-02	0,85410249	2,20E-03	9,63E-01	1,00E+00	Isg20	0,99	No
ENSMUSG00000039238	4,94E-01	0,94353533	5,73E+00	1,67E-02	1,35E-01	Zfp750	1,41	No
ENSMUSG00000039239	-9,76E-01	-1,205792236	4,52E+00	3,34E-02	2,14E-01	Tgfb2	0,51	No
ENSMUSG00000039242	1,93E-01	4,252096482	5,26E+00	2,18E-02	1,59E-01	B3galnt2	1,14	No
ENSMUSG00000039244	1,07E-01	5,338467676	2,27E+00	1,32E-01	4,57E-01	E130309D02Rik	1,08	No
ENSMUSG00000039246	8,01E-03	0,062417923	8,17E-04	9,77E-01	1,00E+00	Lyplal1	1,01	No
ENSMUSG00000039253	6,73E-02	2,071283412	2,22E-01	6,37E-01	8,68E-01	Fn3krp	1,05	No
ENSMUSG00000039254	1,07E-01	3,727492845	1,31E+00	2,53E-01	5,50E-01	Pomt1	1,08	No
ENSMUSG00000039262	-2,28E-02	7,328323173	1,47E-01	7,02E-01	9,05E-01	Prrc2b	0,98	No
ENSMUSG00000039263	1,19E-02	5,994870746	3,32E-02	8,55E-01	9,92E-01	Npepl1	1,01	No
ENSMUSG00000039264	-9,92E-01	-2,311121469	1,19E+00	2,75E-01	5,71E-01	Gimap3	0,50	No
ENSMUSG00000039270	1,51E-01	4,64184674	3,67E+00	5,55E-02	2,89E-01	Megf9	1,11	No
ENSMUSG00000039275	1,52E-01	6,254679765	5,68E+00	1,72E-02	1,37E-01	Foxk2	1,11	No
ENSMUSG00000039285	-9,42E-03	5,663640208	1,93E-02	8,90E-01	1,00E+00	Azi2	0,99	No
ENSMUSG00000039286	1,11E-01	6,108581419	2,94E+00	8,62E-02	3,78E-01	Fndc3b	1,08	No
ENSMUSG00000039294	1,72E-01	6,160902156	7,11E+00	7,65E-03	7,65E-02	BC017643	1,13	No
ENSMUSG00000039298	3,64E-02	5,644526045	2,88E-01	5,91E-01	8,42E-01	Cdk5rap2	1,03	No
ENSMUSG00000039304	1,16E-01	0,924664229	3,24E-01	5,69E-01	8,30E-01	Tnfsf10	1,08	No
ENSMUSG00000039307	4,15E-02	2,906445317	1,30E-01	7,18E-01	9,15E-01	Hexdc	1,03	No
ENSMUSG00000039308	1,72E-01	6,494424078	7,52E+00	6,09E-03	6,49E-02	Ndst2	1,13	No
ENSMUSG00000039313	-1,52E-02	-2,831963104	1,73E-04	9,90E-01	1,00E+00	AF529169	0,99	No

ENSMUSG00000039315	1,22E+00	-2,655136616	9,22E-01	3,37E-01	6,38E-01	Clnk	2,33	No
ENSMUSG00000039316	-5,66E-02	7,67933683	9,28E-01	3,35E-01	6,36E-01	Rftn1	0,96	No
ENSMUSG00000039318	8,22E-02	6,203565416	1,64E+00	2,01E-01	5,50E-01	Rab3gap2	1,06	No
ENSMUSG00000039328	4,24E-02	1,179411335	4,68E-02	8,29E-01	9,76E-01	Rnf122	1,03	No
ENSMUSG00000039330	-1,36E+00	-2,896122487	1,16E+00	2,81E-01	5,78E-01	Tsga10ip	0,39	No
ENSMUSG00000039339	2,67E+00	-3,033636951	2,76E+00	9,68E-02	3,88E-01	Mfsd4b2	6,36	No
ENSMUSG00000039342	3,57E-03	-3,165745957	-2,99E-11	1,00E+00	1,00E+00	Ankar	1,00	No
ENSMUSG00000039345	2,36E-01	1,763304811	2,15E+00	1,43E-01	4,77E-01	Mettl22	1,18	No
ENSMUSG00000039347	1,89E-02	-1,088715476	1,54E-03	9,69E-01	1,00E+00	Atp6v0e2	1,01	No
ENSMUSG00000039349	3,16E+00	-2,83198773	4,06E+00	4,38E-02	2,49E-01	C130074G19Rik	8,92	No
ENSMUSG00000039354	6,91E-02	4,764505023	8,25E-01	3,64E-01	6,65E-01	Smarcal1	1,05	No
ENSMUSG00000039356	1,16E-01	3,672089489	1,48E+00	2,24E-01	5,50E-01	Exosc2	1,08	No
ENSMUSG00000039357	-8,82E-03	4,600048969	1,24E-02	9,11E-01	1,00E+00	Fut11	0,99	No
ENSMUSG00000039361	7,60E-02	9,240529848	1,80E+00	1,80E-01	5,42E-01	Picalm	1,05	No
ENSMUSG00000039364	-2,68E+00	-3,033645766	2,84E+00	9,17E-02	3,82E-01	Sectm1b	0,16	No
ENSMUSG00000039367	-3,83E-02	7,386819831	4,15E-01	5,20E-01	7,99E-01	Sec24c	0,97	No
ENSMUSG00000039372	-1,49E+00	-2,497585539	2,31E+00	1,29E-01	4,50E-01	March4	0,35	No
ENSMUSG00000039375	1,21E-02	-3,033650469	7,34E-11	1,00E+00	1,00E+00	Wdr17	1,01	No
ENSMUSG00000039376	-2,89E-01	-1,472955942	2,74E-01	6,01E-01	8,47E-01	Synpo2l	0,82	No
ENSMUSG00000039377	-1,82E-01	7,027192087	8,86E+00	2,91E-03	3,74E-02	Hlx	0,88	Yes
ENSMUSG00000039384	-8,53E-02	2,163731289	3,41E-01	5,59E-01	8,22E-01	Dusp10	0,94	No
ENSMUSG00000039385	1,87E+00	-2,963248007	1,36E+00	2,44E-01	5,50E-01	Cdh6	3,65	No
ENSMUSG00000039391	-1,98E-02	-3,033655952	2,83E-11	1,00E+00	1,00E+00	Ccdc81	0,99	No
ENSMUSG00000039395	-6,21E-01	6,098917627	9,30E+01	5,31E-22	1,47E-19	Mreg	0,65	Yes
ENSMUSG00000039396	3,47E-01	4,690906408	1,98E+01	8,69E-06	2,96E-04	Neil3	1,27	Yes
ENSMUSG00000039405	-2,71E+00	-2,997995711	2,91E+00	8,82E-02	3,79E-01	Prss23	0,15	No
ENSMUSG00000039410	-2,83E-03	1,160120607	2,21E-04	9,88E-01	1,00E+00	Prdm16	1,00	No
ENSMUSG00000039414	8,38E-02	5,722511124	1,55E+00	2,12E-01	5,50E-01	Heatr5b	1,06	No
ENSMUSG00000039419	2,19E-01	-2,600713636	6,02E-02	8,06E-01	9,64E-01	Cntnap2	1,16	No
ENSMUSG00000039427	1,15E-01	4,252002006	1,85E+00	1,74E-01	5,32E-01	Alg1	1,08	No
ENSMUSG00000039428	1,33E-01	5,536665498	3,67E+00	5,56E-02	2,89E-01	Tmem135	1,10	No
ENSMUSG00000039431	2,34E-01	3,457761813	5,56E+00	1,84E-02	1,43E-01	Mtmr7	1,18	No

ENSMUSG00000039438	-6,93E-02	-1,711710433	1,35E-02	9,08E-01	1,00E+00	Ttc36	0,95	No
ENSMUSG00000039449	1,51E-01	5,979287322	5,31E+00	2,12E-02	1,57E-01	Prpf18	1,11	No
ENSMUSG00000039450	-7,02E-02	4,418699396	7,47E-01	3,87E-01	6,87E-01	Dcxr	0,95	No
ENSMUSG00000039452	7,86E-02	6,155222197	1,48E+00	2,23E-01	5,50E-01	Snx22	1,06	No
ENSMUSG00000039456	1,40E-01	6,335079056	4,91E+00	2,67E-02	1,83E-01	Morc3	1,10	No
ENSMUSG00000039457	-7,18E-01	2,209350437	2,86E+01	8,98E-08	5,09E-06	Ppl	0,61	Yes
ENSMUSG00000039458	-1,23E-01	5,560876931	3,24E+00	7,20E-02	3,40E-01	Mtmr12	0,92	No
ENSMUSG00000039461	-2,09E-01	3,755985274	5,19E+00	2,27E-02	1,64E-01	Tcta	0,87	No
ENSMUSG00000039462	4,69E-01	-1,847866028	5,79E-01	4,47E-01	7,37E-01	Col10a1	1,38	No
ENSMUSG00000039463	1,55E-01	5,32893427	4,79E+00	2,86E-02	1,92E-01	Slc9a8	1,11	No
ENSMUSG00000039470	9,50E-02	2,439825614	5,05E-01	4,77E-01	7,65E-01	Zdhhc2	1,07	No
ENSMUSG00000039473	1,81E-02	6,858924397	8,84E-02	7,66E-01	9,40E-01	Ubn1	1,01	No
ENSMUSG00000039474	-3,21E-03	4,173017421	1,42E-03	9,70E-01	1,00E+00	Wfs1	1,00	No
ENSMUSG00000039476	-7,38E-02	-3,033659033	2,66E-03	9,59E-01	1,00E+00	Prrx2	0,95	No
ENSMUSG00000039477	3,02E-02	7,117072047	2,52E-01	6,16E-01	8,54E-01	Tnrc18	1,02	No
ENSMUSG00000039478	-4,12E-02	1,773849655	6,94E-02	7,92E-01	9,55E-01	Micu3	0,97	No
ENSMUSG00000039480	3,77E-02	3,788233579	1,62E-01	6,87E-01	8,98E-01	Nt5dc1	1,03	No
ENSMUSG00000039481	-7,90E-01	-2,268065373	9,08E-01	3,41E-01	6,41E-01	Nrtn	0,58	No
ENSMUSG00000039483	4,58E-02	5,1207974	3,98E-01	5,28E-01	8,04E-01	Asb6	1,03	No
ENSMUSG00000039485	5,34E-02	1,750246338	1,13E-01	7,37E-01	9,25E-01	Tspyl4	1,04	No
ENSMUSG00000039492	3,10E+00	-2,963298937	3,91E+00	4,81E-02	2,64E-01	Ccdc27	8,56	No
ENSMUSG00000039496	1,30E-01	0,036627122	2,26E-01	6,35E-01	8,66E-01	Cdnf	1,09	No
ENSMUSG00000039497	2,26E-01	5,77602501	1,13E+01	7,69E-04	1,30E-02	Dse	1,17	Yes
ENSMUSG00000039501	-2,12E-03	7,061144922	1,23E-03	9,72E-01	1,00E+00	Znfx1	1,00	No
ENSMUSG00000039508	-1,89E+00	-2,896152932	1,43E+00	2,31E-01	5,50E-01	Fam26d	0,27	No
ENSMUSG00000039509	1,45E-01	4,819372958	3,63E+00	5,68E-02	2,93E-01	Nup133	1,11	No
ENSMUSG00000039512	-6,88E-02	5,071134818	8,92E-01	3,45E-01	6,46E-01	Uhrf1bp1	0,95	No
ENSMUSG00000039515	8,91E-02	7,612523934	2,28E+00	1,31E-01	4,55E-01	Ptpa	1,06	No
ENSMUSG00000039518	-2,74E+00	-3,033596063	2,99E+00	8,38E-02	3,71E-01	Cdsn	0,15	No
ENSMUSG00000039519	-5,88E-02	-3,033616507	1,62E-03	9,68E-01	1,00E+00	Cyp7b1	0,96	No
ENSMUSG00000039523	7,52E-02	4,693030689	9,39E-01	3,32E-01	6,33E-01	Cep104	1,05	No
ENSMUSG00000039529	1,23E-01	0,592797149	2,69E-01	6,04E-01	8,49E-01	Atp8b1	1,09	No

ENSMUSG00000039530	1,23E-01	4,978812033	2,71E+00	9,98E-02	3,92E-01	Tusc3	1,09	No
ENSMUSG00000039531	8,66E-03	5,151718133	1,45E-02	9,04E-01	1,00E+00	Zufsp	1,01	No
ENSMUSG00000039536	5,57E-02	5,921278857	7,12E-01	3,99E-01	6,98E-01	Stau1	1,04	No
ENSMUSG00000039539	-2,74E+00	-3,033596063	2,99E+00	8,38E-02	3,71E-01	Sgcz	0,15	No
ENSMUSG00000039542	-6,72E-01	-0,946206507	2,40E+00	1,21E-01	4,34E-01	Ncam1	0,63	No
ENSMUSG00000039543	1,86E+00	-3,033661846	1,35E+00	2,46E-01	5,50E-01	Cfap70	3,62	No
ENSMUSG00000039546	-1,94E+00	-3,107619243	1,50E+00	2,21E-01	5,50E-01	Ajap1	0,26	No
ENSMUSG00000039552	2,06E+00	-2,355663125	5,42E+00	1,99E-02	1,51E-01	Rsph4a	4,18	No
ENSMUSG00000039563	-9,96E-01	-2,448612037	1,20E+00	2,73E-01	5,69E-01	2210406O10Rik	0,50	No
ENSMUSG00000039568	2,61E-01	5,722207238	1,49E+01	1,12E-04	2,67E-03	Ubald1	1,20	Yes
ENSMUSG00000039577	-7,65E-02	1,214444302	1,47E-01	7,02E-01	9,05E-01	Nphp4	0,95	No
ENSMUSG00000039578	4,49E-01	-1,627539196	5,56E-01	4,56E-01	7,45E-01	Ccser1	1,37	No
ENSMUSG00000039579	-5,91E-01	0,288502533	4,51E+00	3,38E-02	2,16E-01	Grin3a	0,66	No
ENSMUSG00000039585	1,45E-01	6,224524397	5,11E+00	2,38E-02	1,69E-01	Myo9a	1,11	No
ENSMUSG00000039599	-1,47E-01	3,912401407	2,75E+00	9,72E-02	3,89E-01	Fam149b	0,90	No
ENSMUSG00000039601	-1,94E+00	-3,107619243	1,50E+00	2,21E-01	5,50E-01	Rcan2	0,26	No
ENSMUSG00000039607	-7,27E-01	-1,401198801	2,00E+00	1,58E-01	5,04E-01	Rbms3	0,60	No
ENSMUSG00000039611	-1,17E+00	-1,246953522	5,56E+00	1,84E-02	1,43E-01	Tmem246	0,44	No
ENSMUSG00000039615	8,33E-02	5,457684967	1,43E+00	2,31E-01	5,50E-01	Stub1	1,06	No
ENSMUSG00000039616	3,57E-02	4,783473423	2,20E-01	6,39E-01	8,69E-01	Mocos	1,03	No
ENSMUSG00000039617	-5,50E-02	5,441462574	6,24E-01	4,29E-01	7,23E-01	Gm7488	0,96	No
ENSMUSG00000039620	-1,89E+00	-3,1076441	1,42E+00	2,33E-01	5,50E-01	6430573F11Rik	0,27	No
ENSMUSG00000039621	-1,28E-01	8,900542417	5,04E+00	2,48E-02	1,74E-01	Prex1	0,92	No
ENSMUSG00000039623	1,27E-01	5,969721987	3,76E+00	5,25E-02	2,79E-01	Ap5z1	1,09	No
ENSMUSG00000039628	-1,84E-01	-1,682977234	9,85E-02	7,54E-01	9,33E-01	Hs3st6	0,88	No
ENSMUSG00000039629	-5,97E-01	5,985880818	8,41E+01	4,65E-20	1,04E-17	Strip2	0,66	Yes
ENSMUSG00000039630	8,31E-02	8,757992996	2,11E+00	1,46E-01	4,84E-01	Hnrnpu	1,06	No
ENSMUSG00000039632	-3,52E-02	-2,711697205	8,68E-04	9,76E-01	1,00E+00	Ccdc151	0,98	No
ENSMUSG00000039633	4,34E-02	3,914641642	2,37E-01	6,26E-01	8,60E-01	Lonrf1	1,03	No
ENSMUSG00000039634	-5,06E-02	0,924997204	6,14E-02	8,04E-01	9,63E-01	Zfp189	0,97	No
ENSMUSG00000039637	1,39E-01	6,824499281	5,10E+00	2,39E-02	1,69E-01	Coro7	1,10	No
ENSMUSG00000039640	1,00E-01	4,836532339	1,74E+00	1,87E-01	5,50E-01	Mrpl12	1,07	No

ENSMUSG00000039646	-6,74E-04	4,869319076	7,84E-05	9,93E-01	1,00E+00	Vasn	1,00	No
ENSMUSG00000039648	2,22E-01	1,206235053	1,29E+00	2,55E-01	5,50E-01	Kyat1	1,17	No
ENSMUSG00000039652	1,19E-01	2,058042236	6,44E-01	4,22E-01	7,18E-01	Cpeb3	1,09	No
ENSMUSG00000039653	-8,38E-01	-2,770640249	3,85E-01	5,35E-01	8,07E-01	Baat	0,56	No
ENSMUSG00000039656	-9,92E-02	5,641735218	2,13E+00	1,44E-01	4,80E-01	Rxrb	0,93	No
ENSMUSG00000039660	6,41E-02	4,053780526	5,27E-01	4,68E-01	7,56E-01	Spout1	1,05	No
ENSMUSG00000039661	-1,87E+00	-3,03364178	1,41E+00	2,36E-01	5,50E-01	Dusp26	0,27	No
ENSMUSG00000039662	-7,77E-04	5,985855537	1,41E-04	9,91E-01	1,00E+00	Icmt	1,00	No
ENSMUSG00000039670	-3,16E-02	1,385896567	3,06E-02	8,61E-01	9,96E-01	Oxld1	0,98	No
ENSMUSG00000039671	1,20E-01	6,730722051	3,76E+00	5,26E-02	2,79E-01	Zmynd8	1,09	No
ENSMUSG00000039672	2,67E+00	-3,033636951	2,76E+00	9,68E-02	3,88E-01	Kcne2	6,36	No
ENSMUSG00000039676	-7,73E-01	-0,535402377	4,93E+00	2,63E-02	1,82E-01	Capsl	0,59	No
ENSMUSG00000039678	4,04E-02	7,313341098	4,59E-01	4,98E-01	7,83E-01	Tbc1d13	1,03	No
ENSMUSG00000039680	-2,28E-01	4,222919672	7,33E+00	6,79E-03	7,03E-02	Mrps6	0,85	No
ENSMUSG00000039682	7,54E-02	6,020375941	1,33E+00	2,48E-01	5,50E-01	Lap3	1,05	No
ENSMUSG00000039683	1,68E-01	-0,711722713	1,63E-01	6,86E-01	8,97E-01	Sdk1	1,12	No
ENSMUSG00000039684	3,44E-02	7,448011621	3,35E-01	5,63E-01	8,26E-01	Gm5422	1,02	No
ENSMUSG00000039686	-1,19E-01	5,011535114	2,65E+00	1,04E-01	3,96E-01	Zer1	0,92	No
ENSMUSG00000039691	-6,17E-01	-0,682967812	2,67E+00	1,02E-01	3,95E-01	Tspan10	0,65	No
ENSMUSG00000039693	1,34E-01	1,866467475	7,10E-01	4,00E-01	6,98E-01	Msantd3	1,10	No
ENSMUSG00000039697	-4,21E-01	5,863073234	4,04E+01	2,12E-10	1,89E-08	Ncoa7	0,75	Yes
ENSMUSG00000039699	-7,27E-02	2,759479662	3,87E-01	5,34E-01	8,07E-01	Batf2	0,95	No
ENSMUSG00000039701	2,74E-03	3,295465754	6,89E-04	9,79E-01	1,00E+00	Usp53	1,00	No
ENSMUSG00000039703	-6,45E-02	6,965482609	1,12E+00	2,89E-01	5,85E-01	Nploc4	0,96	No
ENSMUSG00000039704	2,13E-01	4,527978664	6,95E+00	8,39E-03	8,22E-02	Lmbrd2	1,16	No
ENSMUSG00000039706	3,14E-02	-3,107617333	-7,69E-10	1,00E+00	1,00E+00	Ldb2	1,02	No
ENSMUSG00000039713	2,69E-01	4,03579617	9,31E+00	2,28E-03	3,11E-02	Plekhg5	1,20	Yes
ENSMUSG00000039714	-9,14E-01	-2,65508874	7,30E-01	3,93E-01	6,93E-01	Cplx3	0,53	No
ENSMUSG00000039715	1,36E-01	2,471049267	1,07E+00	3,01E-01	5,98E-01	Wdr34	1,10	No
ENSMUSG00000039716	1,58E-01	-0,607304381	1,72E-01	6,79E-01	8,92E-01	Dock3	1,12	No
ENSMUSG00000039720	-6,79E-01	-2,146032573	7,83E-01	3,76E-01	6,77E-01	Got1l1	0,62	No
ENSMUSG00000039725	2,01E-01	2,712277696	2,86E+00	9,07E-02	3,82E-01	Trp53rka	1,15	No

ENSMUSG00000039728	1,87E+00	-3,107639691	1,38E+00	2,40E-01	5,50E-01	Slc6a5	3,67	No
ENSMUSG00000039735	-5,94E-01	6,148568183	8,58E+01	2,02E-20	4,77E-18	Fnbp1l	0,66	Yes
ENSMUSG00000039737	-4,22E-02	3,66178498	1,93E-01	6,60E-01	8,82E-01	Prkrip1	0,97	No
ENSMUSG00000039738	2,84E-01	4,919786426	1,45E+01	1,42E-04	3,25E-03	Slx4	1,22	Yes
ENSMUSG00000039740	-1,62E-01	5,425680971	5,44E+00	1,97E-02	1,50E-01	Alg2	0,89	No
ENSMUSG00000039741	5,48E-01	2,899670843	2,16E+01	3,36E-06	1,29E-04	Bahcc1	1,46	Yes
ENSMUSG00000039742	-1,32E+00	-2,896135995	1,09E+00	2,97E-01	5,93E-01	Fam71f1	0,40	No
ENSMUSG00000039745	1,30E-02	5,022634023	3,15E-02	8,59E-01	9,95E-01	Htatip2	1,01	No
ENSMUSG00000039747	-1,06E-01	5,778768121	2,52E+00	1,12E-01	4,13E-01	Orai2	0,93	No
ENSMUSG00000039748	3,18E-01	3,236655595	8,93E+00	2,80E-03	3,63E-02	Exo1	1,25	Yes
ENSMUSG00000039753	-5,57E-02	6,344174695	7,73E-01	3,79E-01	6,80E-01	Fbxl5	0,96	No
ENSMUSG00000039754	2,15E-01	2,885510455	3,39E+00	6,57E-02	3,21E-01	Alkbh4	1,16	No
ENSMUSG00000039756	2,84E-03	5,632801727	1,74E-03	9,67E-01	1,00E+00	Dnttip2	1,00	No
ENSMUSG00000039759	-8,02E-02	2,966382511	5,19E-01	4,71E-01	7,60E-01	Thap3	0,95	No
ENSMUSG00000039760	-6,48E-02	-2,963249916	1,90E-03	9,65E-01	1,00E+00	Il22ra2	0,96	No
ENSMUSG00000039763	1,36E-01	1,305695628	5,62E-01	4,53E-01	7,43E-01	Dnajc28	1,10	No
ENSMUSG00000039765	1,69E-01	1,635589507	1,01E+00	3,15E-01	6,15E-01	Cc2d2a	1,12	No
ENSMUSG00000039768	1,27E-01	5,530495667	3,41E+00	6,46E-02	3,19E-01	Dnajc11	1,09	No
ENSMUSG00000039770	-3,64E-02	6,339042009	3,28E-01	5,67E-01	8,29E-01	Ypel5	0,98	No
ENSMUSG00000039771	6,45E-02	4,10685487	5,38E-01	4,63E-01	7,52E-01	Polr2j	1,05	No
ENSMUSG00000039774	-2,01E-01	4,192304403	5,67E+00	1,73E-02	1,37E-01	Galnt12	0,87	No
ENSMUSG00000039781	3,17E-01	4,571196303	1,58E+01	7,02E-05	1,81E-03	Cep131	1,25	Yes
ENSMUSG00000039782	-2,74E-02	5,51764498	1,58E-01	6,91E-01	9,00E-01	Cpeb2	0,98	No
ENSMUSG00000039783	-1,13E-01	5,900262684	3,00E+00	8,35E-02	3,71E-01	Kmo	0,92	No
ENSMUSG00000039787	2,37E-01	4,628637756	9,06E+00	2,61E-03	3,45E-02	Cercam	1,18	Yes
ENSMUSG00000039789	-6,73E-02	4,171270206	6,33E-01	4,26E-01	7,20E-01	Zfp597	0,95	No
ENSMUSG00000039795	-1,47E-01	3,071195649	1,78E+00	1,82E-01	5,44E-01	Zfand1	0,90	No
ENSMUSG00000039798	-4,03E-02	1,322497889	4,48E-02	8,32E-01	9,78E-01	2600006K01Rik	0,97	No
ENSMUSG00000039801	4,08E-02	4,749119553	2,81E-01	5,96E-01	8,44E-01	2410089E03Rik	1,03	No
ENSMUSG00000039804	-2,70E-02	5,282918805	1,46E-01	7,03E-01	9,06E-01	Ncoa5	0,98	No
ENSMUSG00000039809	-9,02E-02	0,56961465	1,32E-01	7,16E-01	9,13E-01	Gabbr2	0,94	No
ENSMUSG00000039810	-4,14E-02	4,087854199	2,30E-01	6,32E-01	8,64E-01	Zc3h10	0,97	No

ENSMUSG00000039813	2,75E-01	5,65358497	1,64E+01	5,26E-05	1,42E-03	Tbc1d2	1,21	Yes
ENSMUSG00000039814	4,22E-01	0,632751933	3,20E+00	7,37E-02	3,45E-01	Xkr5	1,34	No
ENSMUSG00000039824	-2,78E-02	0,316888813	1,05E-02	9,19E-01	1,00E+00	Myl6b	0,98	No
ENSMUSG00000039826	2,47E-01	5,091340952	1,14E+01	7,49E-04	1,28E-02	Trub2	1,19	Yes
ENSMUSG00000039828	6,60E-02	3,272426076	3,83E-01	5,36E-01	8,08E-01	Wdr70	1,05	No
ENSMUSG00000039831	-2,47E-01	-0,051779695	7,00E-01	4,03E-01	7,01E-01	Arhgap29	0,84	No
ENSMUSG00000039834	6,47E-02	5,389752462	8,49E-01	3,57E-01	6,58E-01	Zfp335	1,05	No
ENSMUSG00000039835	-1,17E-01	0,191221172	1,82E-01	6,70E-01	8,88E-01	Nhsl1	0,92	No
ENSMUSG00000039838	1,21E-02	-3,033650469	7,34E-11	1,00E+00	1,00E+00	Slc45a1	1,01	No
ENSMUSG00000039840	1,30E-01	5,440692243	3,51E+00	6,10E-02	3,06E-01	Epg5	1,09	No
ENSMUSG00000039841	-1,28E-01	5,384887222	3,35E+00	6,72E-02	3,26E-01	Zfp800	0,92	No
ENSMUSG00000039842	-1,15E-01	3,760909813	1,50E+00	2,21E-01	5,50E-01	Mcph1	0,92	No
ENSMUSG00000039844	-5,73E-02	6,521603621	8,38E-01	3,60E-01	6,61E-01	Rapgef1	0,96	No
ENSMUSG00000039849	-2,24E-02	5,860212635	1,14E-01	7,36E-01	9,24E-01	Pcif1	0,98	No
ENSMUSG00000039850	-1,51E-01	3,640656403	2,59E+00	1,07E-01	4,01E-01	Endov	0,90	No
ENSMUSG00000039851	-3,47E-01	-0,847739333	8,03E-01	3,70E-01	6,71E-01	4932438H23Rik	0,79	No
ENSMUSG00000039852	1,25E-01	5,874498666	3,54E+00	6,01E-02	3,03E-01	Rere	1,09	No
ENSMUSG00000039853	-4,90E-02	4,801407598	4,21E-01	5,17E-01	7,97E-01	Trim14	0,97	No
ENSMUSG00000039860	-8,94E-02	-2,711742702	5,34E-03	9,42E-01	1,00E+00	Srrm3	0,94	No
ENSMUSG00000039865	1,81E+00	-3,10765737	1,28E+00	2,58E-01	5,50E-01	Slc44a3	3,49	No
ENSMUSG00000039873	1,80E-01	2,246034881	1,65E+00	1,99E-01	5,50E-01	Neurl2	1,13	No
ENSMUSG00000039878	-6,66E-02	-0,300500409	4,11E-02	8,39E-01	9,83E-01	Slc39a5	0,95	No
ENSMUSG00000039879	-1,06E-01	5,412357084	2,29E+00	1,31E-01	4,54E-01	Heca	0,93	No
ENSMUSG00000039883	1,87E+00	-3,107639691	1,38E+00	2,40E-01	5,50E-01	Lrrc17	3,67	No
ENSMUSG00000039886	7,49E-03	3,799571076	6,56E-03	9,35E-01	1,00E+00	Tmem120a	1,01	No
ENSMUSG00000039887	9,03E-02	3,065102089	6,51E-01	4,20E-01	7,15E-01	Alg14	1,06	No
ENSMUSG00000039891	-9,54E-02	2,580659182	6,05E-01	4,37E-01	7,30E-01	Txlnb	0,94	No
ENSMUSG00000039899	3,15E-02	7,208622501	2,75E-01	6,00E-01	8,46E-01	Fgl2	1,02	No
ENSMUSG00000039901	1,65E-01	3,009902777	2,09E+00	1,49E-01	4,89E-01	9130011E15Rik	1,12	No
ENSMUSG00000039903	2,46E-01	-1,448761614	2,03E-01	6,52E-01	8,77E-01	Eva1c	1,19	No
ENSMUSG00000039908	5,57E-01	3,45406408	3,07E+01	2,95E-08	1,84E-06	Slc26a11	1,47	Yes
ENSMUSG00000039910	2,29E-01	6,252743044	1,26E+01	3,77E-04	7,28E-03	Cited2	1,17	Yes

ENSMUSG00000039911	-4,23E-01	3,578628425	1,95E+01	1,00E-05	3,37E-04	Spsb1	0,75	Yes
ENSMUSG00000039914	1,09E-01	4,419476743	1,80E+00	1,80E-01	5,42E-01	Coq10a	1,08	No
ENSMUSG00000039917	2,72E-02	3,839027478	8,88E-02	7,66E-01	9,40E-01	Rhbdd2	1,02	No
ENSMUSG00000039929	2,00E-01	4,063272154	5,26E+00	2,18E-02	1,59E-01	Urb1	1,15	No
ENSMUSG00000039934	3,34E-02	6,699404733	2,92E-01	5,89E-01	8,41E-01	Gsap	1,02	No
ENSMUSG00000039936	3,08E-01	9,460372847	2,96E+01	5,42E-08	3,21E-06	Pik3cd	1,24	Yes
ENSMUSG00000039942	-2,89E-01	6,454634629	2,12E+01	4,07E-06	1,52E-04	Ptger4	0,82	Yes
ENSMUSG00000039943	-1,23E-02	-0,963264624	8,66E-04	9,77E-01	1,00E+00	Plcb4	0,99	No
ENSMUSG00000039952	1,41E-01	6,745839061	5,23E+00	2,23E-02	1,62E-01	Dag1	1,10	No
ENSMUSG00000039953	-1,87E-01	4,651846342	5,71E+00	1,69E-02	1,36E-01	Clstn1	0,88	No
ENSMUSG00000039956	-1,53E+00	-1,401201101	7,09E+00	7,74E-03	7,71E-02	Mrap	0,35	No
ENSMUSG00000039958	-1,23E-01	2,582197134	9,19E-01	3,38E-01	6,38E-01	Etfbkmt	0,92	No
ENSMUSG00000039959	2,69E-01	7,430463531	2,04E+01	6,34E-06	2,22E-04	Hip1	1,20	Yes
ENSMUSG00000039960	-4,19E-01	0,814639592	3,64E+00	5,64E-02	2,92E-01	Rhou	0,75	No
ENSMUSG00000039963	-4,15E-01	0,604635567	3,02E+00	8,25E-02	3,69E-01	Ccdc40	0,75	No
ENSMUSG00000039967	9,22E-03	6,113580314	2,03E-02	8,87E-01	1,00E+00	Zfp292	1,01	No
ENSMUSG00000039968	-2,58E-01	5,731993121	1,48E+01	1,18E-04	2,77E-03	Rsbn1l	0,84	Yes
ENSMUSG00000039976	2,96E-01	5,298370031	1,71E+01	3,60E-05	1,03E-03	Tbc1d16	1,23	Yes
ENSMUSG00000039977	1,86E+00	-2,896125762	1,35E+00	2,46E-01	5,50E-01	Deup1	3,63	No
ENSMUSG00000039981	-2,09E-01	3,465800083	4,61E+00	3,18E-02	2,07E-01	Zc3h12d	0,87	No
ENSMUSG00000039982	-1,08E-01	5,44072583	2,43E+00	1,19E-01	4,30E-01	Dtx4	0,93	No
ENSMUSG00000039983	-4,94E-03	4,329200506	3,50E-03	9,53E-01	1,00E+00	Ccdc32	1,00	No
ENSMUSG00000039985	-4,82E-01	4,411439371	3,62E+01	1,77E-09	1,39E-07	Fam60a	0,72	Yes
ENSMUSG00000039987	-8,48E-02	4,597866411	1,15E+00	2,83E-01	5,79E-01	Phtf2	0,94	No
ENSMUSG00000039988	-9,57E-03	5,438338934	1,89E-02	8,91E-01	1,00E+00	Ankrd13c	0,99	No
ENSMUSG00000039989	1,09E-01	3,563431801	1,25E+00	2,63E-01	5,57E-01	Cbx4	1,08	No
ENSMUSG00000039990	6,74E-02	4,043848851	6,00E-01	4,38E-01	7,31E-01	Edrf1	1,05	No
ENSMUSG00000039994	9,38E-02	4,996746812	1,63E+00	2,02E-01	5,50E-01	Timeless	1,07	No
ENSMUSG00000039997	8,52E-02	5,795450974	1,64E+00	2,00E-01	5,50E-01	Ifi203	1,06	No
ENSMUSG00000040003	-5,39E-02	0,248029232	4,19E-02	8,38E-01	9,82E-01	Magi2	0,96	No
ENSMUSG00000040006	-1,44E-01	4,649105129	3,35E+00	6,73E-02	3,26E-01	Ginm1	0,91	No
ENSMUSG00000040007	-1,50E-01	6,162305652	5,49E+00	1,91E-02	1,47E-01	Bahd1	0,90	No

ENSMUSG00000040009	1,13E-01	-1,070249744	7,03E-02	7,91E-01	9,55E-01	Gnaz	1,08	No
ENSMUSG00000040010	-6,28E-02	7,104669746	1,08E+00	2,98E-01	5,94E-01	Slc7a5	0,96	No
ENSMUSG00000040013	-1,94E+00	-3,107619243	1,50E+00	2,21E-01	5,50E-01	Fkbp6	0,26	No
ENSMUSG00000040016	-8,40E-02	1,752673971	2,69E-01	6,04E-01	8,49E-01	Ptger3	0,94	No
ENSMUSG00000040017	-3,83E-02	-3,107651936	-7,91E-11	1,00E+00	1,00E+00	Saa4	0,97	No
ENSMUSG00000040018	6,44E-02	5,470213616	8,71E-01	3,51E-01	6,52E-01	Cox15	1,05	No
ENSMUSG00000040021	1,15E-01	6,255456108	3,21E+00	7,31E-02	3,43E-01	Lats1	1,08	No
ENSMUSG00000040022	1,29E-01	3,424348257	1,63E+00	2,02E-01	5,50E-01	Rab11fip2	1,09	No
ENSMUSG00000040025	1,42E-02	5,998944935	4,70E-02	8,28E-01	9,76E-01	Ythdf2	1,01	No
ENSMUSG00000040026	1,95E-01	3,005277953	2,83E+00	9,23E-02	3,83E-01	Saa3	1,14	No
ENSMUSG00000040028	1,02E-01	6,33972564	2,62E+00	1,05E-01	3,97E-01	Elavl1	1,07	No
ENSMUSG00000040029	-1,81E-02	6,082231972	7,76E-02	7,81E-01	9,49E-01	Ipo8	0,99	No
ENSMUSG00000040033	8,56E-02	6,709288632	1,94E+00	1,64E-01	5,15E-01	Stat2	1,06	No
ENSMUSG00000040034	1,26E-01	2,485061491	9,49E-01	3,30E-01	6,30E-01	Nup43	1,09	No
ENSMUSG00000040035	-1,09E-02	-2,896111597	8,93E-05	9,92E-01	1,00E+00	Disp2	0,99	No
ENSMUSG00000040037	1,24E+00	-2,49760993	9,48E-01	3,30E-01	6,31E-01	Negr1	2,36	No
ENSMUSG00000040040	2,70E-01	1,769715567	2,79E+00	9,48E-02	3,86E-01	Ift88	1,21	No
ENSMUSG00000040043	1,04E-01	3,571376239	1,11E+00	2,92E-01	5,87E-01	Rbms2	1,07	No
ENSMUSG00000040044	3,04E-02	5,383447953	1,87E-01	6,65E-01	8,85E-01	Orc3	1,02	No
ENSMUSG00000040048	-1,24E-01	5,807521535	3,45E+00	6,31E-02	3,13E-01	Ndufb10	0,92	No
ENSMUSG00000040054	-7,28E-02	7,533097397	1,52E+00	2,18E-01	5,50E-01	Baz2a	0,95	No
ENSMUSG00000040061	1,73E-01	8,642712098	9,15E+00	2,48E-03	3,32E-02	Plcb2	1,13	Yes
ENSMUSG00000040065	-3,83E-02	-3,107651936	-7,91E-11	1,00E+00	1,00E+00	Pfpl	0,97	No
ENSMUSG00000040078	1,32E-01	5,646092511	3,77E+00	5,21E-02	2,78E-01	Ptges3-ps	1,10	No
ENSMUSG00000040084	1,21E-01	6,033200469	3,44E+00	6,38E-02	3,16E-01	Bub1b	1,09	No
ENSMUSG00000040086	3,40E-01	-0,770729953	7,75E-01	3,79E-01	6,79E-01	Tnni3k	1,27	No
ENSMUSG00000040093	-4,44E-01	5,95929513	4,65E+01	9,37E-12	9,93E-10	Bmf	0,74	Yes
ENSMUSG00000040097	-1,51E-01	4,44472299	3,43E+00	6,39E-02	3,16E-01	Flywch1	0,90	No
ENSMUSG00000040102	2,04E-01	2,602859364	2,84E+00	9,20E-02	3,82E-01	Klhl42	1,15	No
ENSMUSG00000040105	1,88E-02	3,763166774	4,00E-02	8,41E-01	9,84E-01	Plpp6	1,01	No
ENSMUSG00000040111	5,12E-03	5,974684871	6,10E-03	9,38E-01	1,00E+00	Gramd1b	1,00	No
ENSMUSG00000040112	-7,33E-02	3,979643448	6,79E-01	4,10E-01	7,06E-01	Mrps35	0,95	No

ENSMUSG00000040118	-1,86E+00	-3,033612668	1,39E+00	2,38E-01	5,50E-01	Cacna2d1	0,28	No
ENSMUSG00000040121	3,09E-01	-2,033742072	1,75E-01	6,76E-01	8,91E-01	Rep15	1,24	No
ENSMUSG00000040123	-7,56E-02	5,33994934	1,17E+00	2,80E-01	5,77E-01	Zmym5	0,95	No
ENSMUSG00000040124	-8,43E-02	3,33092729	6,62E-01	4,16E-01	7,11E-01	Gorab	0,94	No
ENSMUSG00000040128	-1,08E-01	6,363343942	2,91E+00	8,82E-02	3,79E-01	Pnrc1	0,93	No
ENSMUSG00000040132	-1,94E+00	-3,107619243	1,50E+00	2,21E-01	5,50E-01	Svs2	0,26	No
ENSMUSG00000040133	1,09E-01	2,631167319	7,84E-01	3,76E-01	6,77E-01	Gpr176	1,08	No
ENSMUSG00000040136	-5,69E-01	-2,600671505	2,54E-01	6,14E-01	8,53E-01	Abcc8	0,67	No
ENSMUSG00000040139	4,91E-02	3,315628113	2,14E-01	6,43E-01	8,72E-01	9430038I01Rik	1,03	No
ENSMUSG00000040140	3,52E+00	-2,600715964	5,38E+00	2,04E-02	1,53E-01	Tdrd6	11,48	No
ENSMUSG00000040146	-3,14E-01	0,726779012	1,85E+00	1,74E-01	5,32E-01	Rgl3	0,80	No
ENSMUSG00000040151	5,36E-02	5,29753007	5,69E-01	4,51E-01	7,41E-01	Hs2st1	1,04	No
ENSMUSG00000040152	-1,16E-01	10,11424913	4,25E+00	3,92E-02	2,35E-01	Thbs1	0,92	No
ENSMUSG00000040158	-1,21E-01	5,312601265	2,94E+00	8,63E-02	3,78E-01	Tax1bp3	0,92	No
ENSMUSG00000040163	2,60E+00	-2,963258458	2,63E+00	1,05E-01	3,97E-01	1700034J05Rik	6,08	No
ENSMUSG00000040165	3,13E-01	6,289438746	2,38E+01	1,05E-06	4,62E-05	Cd209c	1,24	Yes
ENSMUSG00000040167	2,44E-01	3,48756715	6,02E+00	1,41E-02	1,21E-01	Ikzf5	1,18	No
ENSMUSG00000040174	2,36E-02	4,093212155	7,37E-02	7,86E-01	9,52E-01	Alkbh3	1,02	No
ENSMUSG00000040177	2,84E-01	3,23916561	7,15E+00	7,49E-03	7,54E-02	2310057M21Rik	1,22	No
ENSMUSG00000040181	-9,98E-01	-2,401330623	1,21E+00	2,72E-01	5,69E-01	Fmo1	0,50	No
ENSMUSG00000040183	2,84E-01	-1,146113421	3,18E-01	5,73E-01	8,32E-01	Ankrd6	1,22	No
ENSMUSG00000040187	1,24E-02	0,458269814	2,63E-03	9,59E-01	1,00E+00	Arntl2	1,01	No
ENSMUSG00000040188	-8,15E-02	7,264129969	1,86E+00	1,73E-01	5,30E-01	Scamp2	0,95	No
ENSMUSG00000040189	-2,08E-02	0,950627939	9,79E-03	9,21E-01	1,00E+00	Ccdc114	0,99	No
ENSMUSG00000040195	3,63E-01	4,493162735	2,06E+01	5,74E-06	2,05E-04	Nemp1	1,29	Yes
ENSMUSG00000040197	8,34E-01	1,3790677	1,97E+01	9,12E-06	3,09E-04	Cd209e	1,78	Yes
ENSMUSG00000040204	2,46E-01	6,210368673	1,46E+01	1,35E-04	3,11E-03	Pclaf	1,19	Yes
ENSMUSG00000040205	-3,19E+00	-2,831909156	4,29E+00	3,82E-02	2,32E-01	Cuzd1	0,11	No
ENSMUSG00000040209	8,12E-02	5,852783363	1,50E+00	2,21E-01	5,50E-01	Zfp704	1,06	No
ENSMUSG00000040212	1,59E-01	6,894471478	6,80E+00	9,11E-03	8,71E-02	Emp3	1,12	No
ENSMUSG00000040213	4,60E-02	2,307877555	1,14E-01	7,35E-01	9,24E-01	Kyat3	1,03	No
ENSMUSG00000040219	-6,45E-02	2,472371923	2,46E-01	6,20E-01	8,57E-01	Ttc12	0,96	No

ENSMUSG00000040220	-7,80E-02	3,516992662	6,19E-01	4,31E-01	7,25E-01	Gas8	0,95	No
ENSMUSG00000040225	6,15E-02	8,401276104	1,14E+00	2,85E-01	5,81E-01	Prrc2c	1,04	No
ENSMUSG00000040231	1,13E-01	-1,355449353	5,39E-02	8,16E-01	9,69E-01	Syngr4	1,08	No
ENSMUSG00000040234	9,26E-02	4,779149746	1,45E+00	2,29E-01	5,50E-01	Tm7sf3	1,07	No
ENSMUSG00000040236	1,81E-01	4,798920001	5,68E+00	1,71E-02	1,37E-01	Trappc5	1,13	No
ENSMUSG00000040242	1,08E-01	6,353293066	2,91E+00	8,80E-02	3,79E-01	Fgfr1op2	1,08	No
ENSMUSG00000040247	-5,01E-01	3,412249797	2,54E+01	4,61E-07	2,22E-05	Tbc1d10c	0,71	Yes
ENSMUSG00000040249	2,64E-01	10,10218345	2,19E+01	2,86E-06	1,11E-04	Lrp1	1,20	Yes
ENSMUSG00000040250	-3,87E-04	4,320902882	2,18E-05	9,96E-01	1,00E+00	Ints13	1,00	No
ENSMUSG00000040253	1,38E-01	6,755787225	5,01E+00	2,52E-02	1,76E-01	Gbp7	1,10	No
ENSMUSG00000040254	-8,02E-01	-1,497527953	2,69E+00	1,01E-01	3,92E-01	Sema3d	0,57	No
ENSMUSG00000040258	3,09E+00	-2,896194806	3,89E+00	4,86E-02	2,65E-01	Nxph4	8,51	No
ENSMUSG00000040260	5,99E-02	2,17013927	1,78E-01	6,73E-01	8,90E-01	Daam2	1,04	No
ENSMUSG00000040263	1,66E-01	6,304222732	6,73E+00	9,50E-03	8,98E-02	Klhdc4	1,12	No
ENSMUSG00000040264	-1,96E-01	1,970219955	1,66E+00	1,97E-01	5,50E-01	Gbp2b	0,87	No
ENSMUSG00000040265	-5,55E-01	-0,998017047	1,74E+00	1,87E-01	5,50E-01	Dnm3	0,68	No
ENSMUSG00000040268	-1,14E-01	7,275658232	3,66E+00	5,57E-02	2,89E-01	Plekha1	0,92	No
ENSMUSG00000040269	5,82E-03	3,406915784	3,20E-03	9,55E-01	1,00E+00	Mrps28	1,00	No
ENSMUSG00000040270	3,21E-02	0,816850219	2,04E-02	8,87E-01	1,00E+00	Bach2	1,02	No
ENSMUSG00000040272	2,17E-01	3,683904259	5,31E+00	2,12E-02	1,57E-01	Accs	1,16	No
ENSMUSG00000040274	-3,50E-01	7,09644569	3,36E+01	6,63E-09	4,77E-07	Cdk6	0,78	Yes
ENSMUSG00000040276	-3,25E-01	3,905196629	1,32E+01	2,82E-04	5,77E-03	Pacsin1	0,80	Yes
ENSMUSG00000040280	2,97E-01	-2,740908592	5,39E-02	8,16E-01	9,69E-01	Ndufa4l2	1,23	No
ENSMUSG00000040282	9,95E-02	2,506465657	5,78E-01	4,47E-01	7,38E-01	BC052040	1,07	No
ENSMUSG00000040283	6,47E-01	-1,801111825	1,23E+00	2,67E-01	5,64E-01	Btnl9	1,57	No
ENSMUSG00000040287	-2,58E-02	0,259125401	9,73E-03	9,21E-01	1,00E+00	Stac3	0,98	No
ENSMUSG00000040289	-1,04E+00	-0,289200171	1,01E+01	1,45E-03	2,18E-02	Hey1	0,49	Yes
ENSMUSG00000040296	-1,61E-01	5,043358389	4,84E+00	2,77E-02	1,88E-01	Ddx58	0,89	No
ENSMUSG00000040297	-1,96E-01	5,459625868	7,92E+00	4,89E-03	5,51E-02	Suco	0,87	No
ENSMUSG00000040298	1,20E-01	-1,378238822	5,37E-02	8,17E-01	9,69E-01	Btbd16	1,09	No
ENSMUSG00000040302	-5,02E-02	3,130607205	2,11E-01	6,46E-01	8,74E-01	Rbm48	0,97	No
ENSMUSG00000040310	1,84E+00	-3,033632273	1,33E+00	2,49E-01	5,50E-01	Alx4	3,58	No

ENSMUSG00000040312	6,93E-03	3,787639543	5,60E-03	9,40E-01	1,00E+00	Cchcr1	1,00	No
ENSMUSG00000040314	-8,80E-02	-2,896116238	3,33E-03	9,54E-01	1,00E+00	Ctsg	0,94	No
ENSMUSG00000040321	-2,63E-01	3,405722506	6,78E+00	9,24E-03	8,81E-02	Zfp770	0,83	No
ENSMUSG00000040322	2,04E-01	6,490030359	1,05E+01	1,19E-03	1,88E-02	Slc25a24	1,15	Yes
ENSMUSG00000040323	1,22E-01	6,452704042	3,71E+00	5,40E-02	2,84E-01	Gm15429	1,09	No
ENSMUSG00000040325	-3,91E-02	4,954889269	2,78E-01	5,98E-01	8,45E-01	Vprbp	0,97	No
ENSMUSG00000040327	-2,00E-01	4,916456212	7,16E+00	7,46E-03	7,51E-02	Cul9	0,87	No
ENSMUSG00000040328	-5,80E-01	0,731939334	6,66E+00	9,85E-03	9,22E-02	Olfr56	0,67	No
ENSMUSG00000040329	-5,43E-02	0,578098361	5,23E-02	8,19E-01	9,71E-01	Il7	0,96	No
ENSMUSG00000040331	3,65E-02	4,69306137	2,23E-01	6,37E-01	8,68E-01	Nsmce4a	1,03	No
ENSMUSG00000040339	-5,77E-02	5,863803439	7,50E-01	3,86E-01	6,87E-01	Fam102b	0,96	No
ENSMUSG00000040340	8,05E-01	-2,548287214	5,63E-01	4,53E-01	7,43E-01	Tex45	1,75	No
ENSMUSG00000040345	9,17E-02	6,731078307	2,21E+00	1,38E-01	4,68E-01	Arhgap9	1,07	No
ENSMUSG00000040350	-3,46E-01	5,634130341	2,64E+01	2,80E-07	1,41E-05	Trim7	0,79	Yes
ENSMUSG00000040351	-7,57E-02	6,311251685	1,42E+00	2,33E-01	5,50E-01	Ankib1	0,95	No
ENSMUSG00000040354	1,51E-02	5,633039821	4,93E-02	8,24E-01	9,73E-01	Mars	1,01	No
ENSMUSG00000040356	-4,36E-02	6,564581925	4,89E-01	4,84E-01	7,72E-01	Skiv2l	0,97	No
ENSMUSG00000040359	1,17E-01	4,467283512	2,07E+00	1,50E-01	4,91E-01	Ufl1	1,08	No
ENSMUSG00000040364	-1,31E+00	-2,800961615	1,07E+00	3,00E-01	5,97E-01	Sec1	0,40	No
ENSMUSG00000040365	6,65E-02	5,949036273	1,02E+00	3,11E-01	6,11E-01	Trim41	1,05	No
ENSMUSG00000040367	-5,05E-01	-2,333260509	3,35E-01	5,63E-01	8,26E-01	Lrrd1	0,70	No
ENSMUSG00000040370	-1,20E-01	4,492121994	2,25E+00	1,34E-01	4,60E-01	Etfrf1	0,92	No
ENSMUSG00000040372	-1,87E+00	-3,107637761	1,39E+00	2,38E-01	5,50E-01	Gpr63	0,27	No
ENSMUSG00000040374	4,93E-02	4,902252404	4,25E-01	5,14E-01	7,96E-01	Pex2	1,03	No
ENSMUSG00000040380	-8,03E-01	-1,247093946	3,29E+00	6,95E-02	3,32E-01	Cbln3	0,57	No
ENSMUSG00000040383	8,79E-02	6,096192515	1,84E+00	1,75E-01	5,33E-01	Aqr	1,06	No
ENSMUSG00000040385	-2,07E-02	8,663658831	1,30E-01	7,18E-01	9,15E-01	Ppp1ca	0,99	No
ENSMUSG00000040387	3,63E-01	-1,614233135	3,88E-01	5,33E-01	8,06E-01	Klhl32	1,29	No
ENSMUSG00000040389	2,49E-02	4,306454268	9,05E-02	7,64E-01	9,39E-01	Wdr47	1,02	No
ENSMUSG00000040390	-1,65E-01	4,271625012	3,90E+00	4,83E-02	2,64E-01	Map3k10	0,89	No
ENSMUSG00000040396	1,01E-01	5,408708794	2,07E+00	1,50E-01	4,91E-01	Abhd13	1,07	No
ENSMUSG00000040405	-1,88E+00	-2,448649814	4,13E+00	4,21E-02	2,44E-01	Havcr1	0,27	No

ENSMUSG00000040407	-1,10E-01	6,664080663	3,17E+00	7,51E-02	3,49E-01	Akap9	0,93	No
ENSMUSG00000040410	-6,55E-02	2,353715552	2,36E-01	6,27E-01	8,61E-01	Fbxl4	0,96	No
ENSMUSG00000040412	-1,30E+00	-2,185644543	2,87E+00	9,05E-02	3,81E-01	5330417C22Rik	0,41	No
ENSMUSG00000040413	-1,93E+00	-2,963244023	1,47E+00	2,25E-01	5,50E-01	Timd2	0,26	No
ENSMUSG00000040414	-3,02E-02	4,239737152	1,27E-01	7,21E-01	9,16E-01	Slc25a28	0,98	No
ENSMUSG00000040415	-1,45E-01	6,450561343	5,30E+00	2,14E-02	1,58E-01	Dtx3	0,90	No
ENSMUSG00000040423	1,08E-01	6,504267894	2,99E+00	8,39E-02	3,71E-01	Rc3h1	1,08	No
ENSMUSG00000040424	-5,33E-02	-2,033578303	3,97E-03	9,50E-01	1,00E+00	Hipk4	0,96	No
ENSMUSG00000040428	9,64E-01	-0,30602183	8,53E+00	3,50E-03	4,29E-02	Plekha4	1,95	Yes
ENSMUSG00000040429	-1,90E-01	1,104064198	8,66E-01	3,52E-01	6,53E-01	Mterf1a	0,88	No
ENSMUSG00000040430	7,14E-02	4,329558309	7,29E-01	3,93E-01	6,93E-01	Pitpnc1	1,05	No
ENSMUSG00000040432	3,35E-01	-0,989495213	5,26E-01	4,68E-01	7,56E-01	Ltb4r2	1,26	No
ENSMUSG00000040433	4,24E-02	5,160586567	3,52E-01	5,53E-01	8,19E-01	Zbtb38	1,03	No
ENSMUSG00000040434	-5,31E-02	-2,246972669	4,23E-03	9,48E-01	1,00E+00	Large2	0,96	No
ENSMUSG00000040435	-1,97E-01	6,624659803	1,02E+01	1,43E-03	2,15E-02	Ppp1r15a	0,87	Yes
ENSMUSG00000040441	-2,18E-01	5,616164755	1,03E+01	1,35E-03	2,06E-02	Slc26a10	0,86	Yes
ENSMUSG00000040446	3,33E-02	4,546156535	1,73E-01	6,77E-01	8,92E-01	Rprd1a	1,02	No
ENSMUSG00000040447	-5,32E-02	0,799381199	5,46E-02	8,15E-01	9,69E-01	Spns2	0,96	No
ENSMUSG00000040451	1,61E-02	5,972467773	5,96E-02	8,07E-01	9,64E-01	Sgms1	1,01	No
ENSMUSG00000040455	2,15E-01	5,310451006	9,22E+00	2,39E-03	3,23E-02	Usp45	1,16	Yes
ENSMUSG00000040459	-1,77E-01	6,886525706	8,43E+00	3,70E-03	4,47E-02	Arglu1	0,88	Yes
ENSMUSG00000040462	-4,19E-02	7,304777279	4,91E-01	4,84E-01	7,71E-01	Os9	0,97	No
ENSMUSG00000040463	4,82E-02	6,904543167	6,22E-01	4,30E-01	7,24E-01	Mybbp1a	1,03	No
ENSMUSG00000040464	1,25E-01	3,176891604	1,32E+00	2,51E-01	5,50E-01	Gtpbp10	1,09	No
ENSMUSG00000040466	1,69E-01	6,460812643	7,12E+00	7,63E-03	7,64E-02	Blvrb	1,12	No
ENSMUSG00000040467	-8,48E-01	-2,83197049	3,94E-01	5,30E-01	8,05E-01	4921522P10Rik	0,56	No
ENSMUSG00000040471	1,85E+00	-3,107657832	1,35E+00	2,46E-01	5,50E-01	Ggt6	3,61	No
ENSMUSG00000040472	1,07E-01	4,003685883	1,43E+00	2,32E-01	5,50E-01	Rabggta	1,08	No
ENSMUSG00000040473	3,77E+00	-2,832085847	6,53E+00	1,06E-02	9,77E-02	Cfap69	13,62	No
ENSMUSG00000040478	1,83E+00	-2,831994072	1,32E+00	2,50E-01	5,50E-01	Prdm13	3,55	No
ENSMUSG00000040479	1,78E-01	7,022672297	8,62E+00	3,33E-03	4,14E-02	Dgkz	1,13	Yes
ENSMUSG00000040481	3,95E-02	7,042279255	4,25E-01	5,14E-01	7,96E-01	Bptf	1,03	No

ENSMUSG00000040482	8,63E-02	4,479254612	1,15E+00	2,84E-01	5,80E-01	Dxo	1,06	No
ENSMUSG00000040483	-9,10E-02	3,727000557	9,41E-01	3,32E-01	6,32E-01	Xaf1	0,94	No
ENSMUSG00000040488	-7,14E-02	-1,711793972	1,19E-02	9,13E-01	1,00E+00	Ltbp4	0,95	No
ENSMUSG00000040489	-6,81E-02	-2,896149338	3,18E-03	9,55E-01	1,00E+00	Sox30	0,95	No
ENSMUSG00000040490	1,87E+00	-3,107639691	1,38E+00	2,40E-01	5,50E-01	Lrfn2	3,67	No
ENSMUSG00000040495	2,26E-01	-1,896197535	1,18E-01	7,31E-01	9,22E-01	Chrm4	1,17	No
ENSMUSG00000040498	-1,01E+00	-2,070060498	2,27E+00	1,32E-01	4,56E-01	Igsf23	0,50	No
ENSMUSG00000040502	-1,32E-01	1,310240218	4,80E-01	4,89E-01	7,76E-01	March9	0,91	No
ENSMUSG00000040505	3,14E-02	-3,107617333	-7,69E-10	1,00E+00	1,00E+00	Abcg5	1,02	No
ENSMUSG00000040506	-3,79E-02	5,593089957	3,07E-01	5,79E-01	8,36E-01	Ambra1	0,97	No
ENSMUSG00000040511	-2,05E-01	4,729189752	6,94E+00	8,41E-03	8,23E-02	Pvr	0,87	No
ENSMUSG00000040520	2,17E-01	4,848145095	8,06E+00	4,53E-03	5,18E-02	Manea	1,16	No
ENSMUSG00000040521	7,05E-02	3,869353155	6,04E-01	4,37E-01	7,30E-01	Tsfm	1,05	No
ENSMUSG00000040524	-1,46E-02	6,206636093	5,19E-02	8,20E-01	9,71E-01	Zfp609	0,99	No
ENSMUSG00000040525	-1,90E+00	-3,033594154	1,44E+00	2,30E-01	5,50E-01	Cblc	0,27	No
ENSMUSG00000040528	1,51E-01	5,201565297	4,41E+00	3,56E-02	2,24E-01	Milr1	1,11	No
ENSMUSG00000040532	-7,76E-02	4,148592236	8,07E-01	3,69E-01	6,70E-01	Abhd11	0,95	No
ENSMUSG00000040533	-2,87E-01	-0,175644563	8,16E-01	3,66E-01	6,67E-01	Matn1	0,82	No
ENSMUSG00000040536	1,85E+00	-3,107657832	1,35E+00	2,46E-01	5,50E-01	Necab1	3,61	No
ENSMUSG00000040537	7,88E-02	1,280860602	1,76E-01	6,75E-01	8,91E-01	Adam22	1,06	No
ENSMUSG00000040540	4,08E-02	5,591914125	3,57E-01	5,50E-01	8,17E-01	Gm9770	1,03	No
ENSMUSG00000040543	-8,61E-01	-2,89612532	4,07E-01	5,24E-01	8,01E-01	Pitpnm3	0,55	No
ENSMUSG00000040548	2,06E-01	6,024461558	9,87E+00	1,68E-03	2,43E-02	Tex2	1,15	Yes
ENSMUSG00000040549	8,82E-02	6,799721309	2,06E+00	1,51E-01	4,93E-01	Ckap5	1,06	No
ENSMUSG00000040550	1,42E-01	5,271382889	4,00E+00	4,56E-02	2,55E-01	Otud6b	1,10	No
ENSMUSG00000040552	1,58E-01	7,058913032	6,74E+00	9,42E-03	8,93E-02	C3ar1	1,12	No
ENSMUSG00000040554	2,41E-01	-0,491318818	4,74E-01	4,91E-01	7,77E-01	Aipl1	1,18	No
ENSMUSG00000040557	-8,94E-02	2,825616927	5,96E-01	4,40E-01	7,32E-01	Mettl27	0,94	No
ENSMUSG00000040560	1,31E-01	6,394965669	4,29E+00	3,84E-02	2,33E-01	Wdr7	1,10	No
ENSMUSG00000040562	-2,84E-01	2,092193115	3,77E+00	5,23E-02	2,78E-01	Gstm2	0,82	No
ENSMUSG00000040563	-5,63E-02	1,01081783	7,51E-02	7,84E-01	9,51E-01	Plppr2	0,96	No
ENSMUSG00000040564	-1,94E+00	-3,107619243	1,50E+00	2,21E-01	5,50E-01	Apoc1	0,26	No

ENSMUSG00000040565	5,31E-02	6,508159622	7,16E-01	3,97E-01	6,98E-01	Btaf1	1,04	No
ENSMUSG00000040569	-1,89E+00	-3,1076441	1,42E+00	2,33E-01	5,50E-01	Slc26a7	0,27	No
ENSMUSG00000040570	-1,12E-01	-0,800924337	8,02E-02	7,77E-01	9,46E-01	Rundc3b	0,93	No
ENSMUSG00000040583	1,87E+00	-3,107639691	1,38E+00	2,40E-01	5,50E-01	Cyp2b13	3,67	No
ENSMUSG00000040584	1,51E-01	0,785465556	4,76E-01	4,90E-01	7,76E-01	Abcb1a	1,11	No
ENSMUSG00000040591	-5,25E-02	5,265865837	5,46E-01	4,60E-01	7,49E-01	1110051M20Rik	0,96	No
ENSMUSG00000040592	-1,17E+00	-1,268043724	5,08E+00	2,42E-02	1,71E-01	Cd79b	0,44	No
ENSMUSG00000040594	6,39E-01	-1,711745438	1,20E+00	2,73E-01	5,69E-01	Ranbp17	1,56	No
ENSMUSG00000040596	-1,42E-01	5,376075588	4,14E+00	4,18E-02	2,43E-01	Pogk	0,91	No
ENSMUSG00000040599	2,00E-01	5,192652516	7,86E+00	5,05E-03	5,63E-02	Mis12	1,15	No
ENSMUSG00000040600	4,64E-01	-1,641328372	5,68E-01	4,51E-01	7,41E-01	Eps8l3	1,38	No
ENSMUSG00000040605	-1,19E+00	-0,6550362	1,02E+01	1,44E-03	2,16E-02	Bace2	0,44	Yes
ENSMUSG00000040606	3,49E-01	4,059367564	1,54E+01	8,88E-05	2,20E-03	Kazn	1,27	Yes
ENSMUSG00000040612	-4,90E-01	-2,929309566	1,21E-01	7,28E-01	9,20E-01	Ildr2	0,71	No
ENSMUSG00000040613	-5,84E-02	5,605123162	7,35E-01	3,91E-01	6,91E-01	Apobec1	0,96	No
ENSMUSG00000040616	-2,72E-01	4,016651225	9,67E+00	1,87E-03	2,65E-02	Tmem51	0,83	Yes
ENSMUSG00000040618	1,61E-01	4,288720181	3,72E+00	5,38E-02	2,84E-01	Pck2	1,12	No
ENSMUSG00000040620	4,41E-02	5,609502836	4,20E-01	5,17E-01	7,98E-01	Dhx33	1,03	No
ENSMUSG00000040624	-2,80E-01	6,195224228	1,89E+01	1,35E-05	4,38E-04	Plekhg1	0,82	Yes
ENSMUSG00000040627	-5,12E-02	-2,683172903	1,80E-03	9,66E-01	1,00E+00	Aicda	0,97	No
ENSMUSG00000040631	7,22E-02	2,700624128	3,43E-01	5,58E-01	8,22E-01	Dok4	1,05	No
ENSMUSG00000040632	-1,94E+00	-3,107619243	1,50E+00	2,21E-01	5,50E-01	Nrl	0,26	No
ENSMUSG00000040640	-2,83E-02	-0,195608956	7,36E-03	9,32E-01	1,00E+00	Erc2	0,98	No
ENSMUSG00000040648	1,67E-01	5,199208284	5,43E+00	1,97E-02	1,50E-01	Ppip5k2	1,12	No
ENSMUSG00000040649	-1,94E+00	-3,107619243	1,50E+00	2,21E-01	5,50E-01	Rimklb	0,26	No
ENSMUSG00000040651	-1,05E-01	4,485735207	1,75E+00	1,86E-01	5,50E-01	Fam208a	0,93	No
ENSMUSG00000040652	-1,21E-01	4,2688929	2,05E+00	1,52E-01	4,94E-01	Oaz2	0,92	No
ENSMUSG00000040653	-2,66E+00	-3,033639428	2,81E+00	9,35E-02	3,83E-01	Ppp1r14c	0,16	No
ENSMUSG00000040658	-4,14E-02	2,270227981	8,56E-02	7,70E-01	9,42E-01	Dnph1	0,97	No
ENSMUSG00000040659	1,38E-02	8,719245385	5,83E-02	8,09E-01	9,65E-01	Efhd2	1,01	No
ENSMUSG00000040661	1,31E-02	6,261515902	4,20E-02	8,38E-01	9,82E-01	Rad54l2	1,01	No
ENSMUSG00000040663	3,91E-01	4,359157035	2,21E+01	2,64E-06	1,03E-04	Clcf1	1,31	Yes

ENSMUSG00000040666	-7,38E-02	-3,033659033	2,66E-03	9,59E-01	1,00E+00	Sh3bgr	0,95	No
ENSMUSG00000040667	-1,42E-01	6,320315295	5,04E+00	2,47E-02	1,74E-01	Nup88	0,91	No
ENSMUSG00000040669	-2,88E-01	3,934356671	1,03E+01	1,35E-03	2,06E-02	Phc1	0,82	Yes
ENSMUSG00000040675	3,55E-01	4,069882124	1,63E+01	5,28E-05	1,42E-03	Mthfd1l	1,28	Yes
ENSMUSG00000040680	5,22E-01	-0,697189062	1,63E+00	2,02E-01	5,50E-01	Kremen2	1,44	No
ENSMUSG00000040681	8,62E-02	5,171919607	1,42E+00	2,33E-01	5,50E-01	Hmgn1	1,06	No
ENSMUSG00000040687	3,79E-02	6,057337029	3,40E-01	5,60E-01	8,23E-01	Madd	1,03	No
ENSMUSG00000040688	5,82E-02	4,856203667	5,88E-01	4,43E-01	7,35E-01	Tbl3	1,04	No
ENSMUSG00000040690	-1,58E-02	-2,831999518	1,41E-04	9,91E-01	1,00E+00	Col16a1	0,99	No
ENSMUSG00000040693	-2,05E-01	-2,185553202	9,17E-02	7,62E-01	9,38E-01	Slco4c1	0,87	No
ENSMUSG00000040694	2,37E-01	-1,929274673	1,30E-01	7,19E-01	9,15E-01	Apobec2	1,18	No
ENSMUSG00000040697	1,66E-01	5,420259357	5,70E+00	1,69E-02	1,36E-01	Dnajc16	1,12	No
ENSMUSG00000040699	-3,21E-01	5,384086593	2,11E+01	4,46E-06	1,64E-04	Limd2	0,80	Yes
ENSMUSG00000040701	-3,59E-02	5,533557583	2,72E-01	6,02E-01	8,48E-01	Ap1g2	0,98	No
ENSMUSG00000040703	1,17E-01	2,375023266	7,65E-01	3,82E-01	6,83E-01	Cyp2s1	1,08	No
ENSMUSG00000040705	-9,31E-02	-0,87178677	5,01E-02	8,23E-01	9,73E-01	A930016O22Rik	0,94	No
ENSMUSG00000040706	-1,85E+00	-2,96319884	1,39E+00	2,38E-01	5,50E-01	Agmat	0,28	No
ENSMUSG00000040710	-4,55E-01	6,578605334	5,41E+01	1,92E-13	2,55E-11	St8sia4	0,73	Yes
ENSMUSG00000040711	2,15E-01	9,378536801	1,44E+01	1,45E-04	3,29E-03	Sh3pxd2b	1,16	Yes
ENSMUSG00000040712	5,14E-02	7,069085675	7,24E-01	3,95E-01	6,95E-01	Camta2	1,04	No
ENSMUSG00000040713	1,22E-01	8,266234732	4,45E+00	3,48E-02	2,20E-01	Creg1	1,09	No
ENSMUSG00000040714	-9,08E-02	2,8928822	6,14E-01	4,33E-01	7,27E-01	Klc3	0,94	No
ENSMUSG00000040715	1,06E-01	4,983772331	2,04E+00	1,53E-01	4,96E-01	Rsc1a1	1,08	No
ENSMUSG00000040717	-4,63E-01	-2,311205765	3,94E-01	5,30E-01	8,05E-01	Il17rd	0,73	No
ENSMUSG00000040720	8,09E-03	6,52399711	1,68E-02	8,97E-01	1,00E+00	Virma	1,01	No
ENSMUSG00000040721	8,47E-03	4,251822549	1,01E-02	9,20E-01	1,00E+00	Zfhx2	1,01	No
ENSMUSG00000040722	2,56E-01	5,025642376	1,19E+01	5,69E-04	1,02E-02	Scamp5	1,19	Yes
ENSMUSG00000040723	-2,73E-01	5,48834007	1,58E+01	7,13E-05	1,82E-03	Rcsd1	0,83	Yes
ENSMUSG00000040724	5,86E-02	-1,47301487	1,00E-02	9,20E-01	1,00E+00	Kcna2	1,04	No
ENSMUSG00000040725	4,23E-02	8,006978922	5,29E-01	4,67E-01	7,55E-01	Hnrnpul1	1,03	No
ENSMUSG00000040726	2,60E+00	-2,896177956	2,63E+00	1,05E-01	3,97E-01	Hesx1	6,08	No
ENSMUSG00000040728	-7,35E-02	-2,770561087	3,66E-03	9,52E-01	1,00E+00	Esrp1	0,95	No

ENSMUSG00000040729	-7,24E-01	1,079514031	1,40E+01	1,79E-04	3,96E-03	Cep126	0,61	Yes
ENSMUSG00000040731	-5,37E-02	8,121027562	8,57E-01	3,55E-01	6,56E-01	Eif4h	0,96	No
ENSMUSG00000040732	-2,27E+00	-2,548196127	4,20E+00	4,04E-02	2,38E-01	Erg	0,21	No
ENSMUSG00000040734	3,50E-01	2,741573724	8,38E+00	3,80E-03	4,56E-02	Ppp1r13l	1,27	Yes
ENSMUSG00000040738	-3,95E-02	4,591884285	2,48E-01	6,19E-01	8,56E-01	Ints8	0,97	No
ENSMUSG00000040740	-7,50E-01	-1,37829227	1,99E+00	1,59E-01	5,06E-01	Slc25a34	0,59	No
ENSMUSG00000040746	6,37E-02	4,614669687	6,58E-01	4,17E-01	7,12E-01	Rnf167	1,05	No
ENSMUSG00000040747	1,04E-01	8,96395331	3,33E+00	6,81E-02	3,28E-01	Cd53	1,07	No
ENSMUSG00000040751	1,79E-01	5,965640321	7,43E+00	6,40E-03	6,73E-02	Lat2	1,13	No
ENSMUSG00000040752	-6,07E-01	-2,268138786	7,14E-01	3,98E-01	6,98E-01	Myh6	0,66	No
ENSMUSG00000040760	-7,84E-03	5,971914944	1,44E-02	9,05E-01	1,00E+00	Appl1	0,99	No
ENSMUSG00000040761	6,62E-02	6,620670165	1,14E+00	2,87E-01	5,83E-01	Spen	1,05	No
ENSMUSG00000040767	-3,82E-02	3,91822464	1,77E-01	6,74E-01	8,90E-01	Snrnp25	0,97	No
ENSMUSG00000040771	3,37E-02	3,545173152	1,20E-01	7,29E-01	9,20E-01	Oard1	1,02	No
ENSMUSG00000040774	3,46E-02	5,979045604	2,81E-01	5,96E-01	8,45E-01	Cept1	1,02	No
ENSMUSG00000040775	-2,67E+00	-2,831989413	2,83E+00	9,23E-02	3,83E-01	Gm9772	0,16	No
ENSMUSG00000040782	-9,13E-02	4,582413621	1,32E+00	2,51E-01	5,50E-01	Rfwd2	0,94	No
ENSMUSG00000040785	-1,43E-01	5,697528853	4,48E+00	3,43E-02	2,18E-01	Ttc3	0,91	No
ENSMUSG00000040794	-1,02E+00	-2,107712621	1,61E+00	2,05E-01	5,50E-01	C1qtnf4	0,49	No
ENSMUSG00000040795	5,53E-02	3,11194346	2,70E-01	6,04E-01	8,48E-01	Iqcc	1,04	No
ENSMUSG00000040797	-9,16E-02	-2,600662698	7,64E-03	9,30E-01	1,00E+00	Iqsec3	0,94	No
ENSMUSG00000040809	-2,49E-02	7,534976315	1,76E-01	6,75E-01	8,91E-01	Chil3	0,98	No
ENSMUSG00000040811	5,27E-02	4,588177752	4,36E-01	5,09E-01	7,92E-01	Eml2	1,04	No
ENSMUSG00000040812	2,21E-01	-1,929370576	1,13E-01	7,37E-01	9,25E-01	Agbl2	1,17	No
ENSMUSG00000040813	1,56E-01	6,068135788	5,68E+00	1,72E-02	1,37E-01	Tex264	1,11	No
ENSMUSG00000040818	-2,87E-02	5,152706545	1,59E-01	6,90E-01	8,99E-01	Dennd6a	0,98	No
ENSMUSG00000040820	1,13E-01	4,016707808	1,61E+00	2,04E-01	5,50E-01	Hlcs	1,08	No
ENSMUSG00000040822	8,03E-02	5,079468771	1,20E+00	2,73E-01	5,70E-01	1700123O20Rik	1,06	No
ENSMUSG00000040824	-3,55E-02	4,509390826	1,94E-01	6,60E-01	8,82E-01	Snrpd2	0,98	No
ENSMUSG00000040828	-2,90E-01	-0,015515868	1,04E+00	3,08E-01	6,06E-01	Catsperd	0,82	No
ENSMUSG00000040829	-1,93E-01	5,658514317	8,13E+00	4,34E-03	5,03E-02	Zmynd15	0,88	No
ENSMUSG00000040836	4,35E-01	-1,896142013	4,12E-01	5,21E-01	8,00E-01	Gpr161	1,35	No

ENSMUSG00000040840	6,40E-02	3,463101491	4,13E-01	5,21E-01	7,99E-01	4930579G18Rik	1,05	No
ENSMUSG00000040841	5,13E-02	0,615991624	4,84E-02	8,26E-01	9,74E-01	Six5	1,04	No
ENSMUSG00000040842	2,80E-02	6,800466318	2,07E-01	6,49E-01	8,75E-01	Szrd1	1,02	No
ENSMUSG00000040843	1,92E-02	4,725630035	6,20E-02	8,03E-01	9,62E-01	Tiprl	1,01	No
ENSMUSG00000040848	-2,05E-01	7,777876414	1,23E+01	4,51E-04	8,43E-03	Sft2d2	0,87	Yes
ENSMUSG00000040850	-2,35E-02	6,589465343	1,42E-01	7,06E-01	9,08E-01	Psme4	0,98	No
ENSMUSG00000040852	1,21E+00	-2,655183342	9,04E-01	3,42E-01	6,42E-01	Plekhh2	2,31	No
ENSMUSG00000040857	8,94E-02	4,63772842	1,30E+00	2,54E-01	5,50E-01	Erf	1,06	No
ENSMUSG00000040859	-5,79E-02	5,429357079	6,93E-01	4,05E-01	7,02E-01	Bsdc1	0,96	No
ENSMUSG00000040860	1,89E-01	3,722556498	4,04E+00	4,45E-02	2,51E-01	Crocc	1,14	No
ENSMUSG00000040865	-9,72E-02	6,717643823	2,49E+00	1,14E-01	4,18E-01	Ino80d	0,93	No
ENSMUSG00000040875	-1,08E-02	2,485774707	6,77E-03	9,34E-01	1,00E+00	Osbpl10	0,99	No
ENSMUSG00000040877	-1,68E-01	1,942556265	1,26E+00	2,62E-01	5,55E-01	Wdr25	0,89	No
ENSMUSG00000040883	9,65E-03	3,412962917	9,23E-03	9,23E-01	1,00E+00	Tmem205	1,01	No
ENSMUSG00000040888	-5,14E-02	4,547384827	4,23E-01	5,16E-01	7,97E-01	Gfer	0,96	No
ENSMUSG00000040891	-3,83E-02	-3,107651936	-7,91E-11	1,00E+00	1,00E+00	Foxa3	0,97	No
ENSMUSG00000040896	-4,04E-02	-2,770615379	1,11E-03	9,73E-01	1,00E+00	Kcnd3	0,97	No
ENSMUSG00000040899	-3,25E-01	0,563390326	1,92E+00	1,65E-01	5,18E-01	Ccr6	0,80	No
ENSMUSG00000040901	-8,69E-01	-2,963204587	4,16E-01	5,19E-01	7,98E-01	Kcnk18	0,55	No
ENSMUSG00000040904	6,23E-02	5,587509935	8,27E-01	3,63E-01	6,64E-01	Gm21988	1,04	No
ENSMUSG00000040907	4,66E-02	7,986041981	6,39E-01	4,24E-01	7,19E-01	Atp1a3	1,03	No
ENSMUSG00000040913	-9,07E-02	3,853129439	9,98E-01	3,18E-01	6,18E-01	Fbxw4	0,94	No
ENSMUSG00000040918	1,10E-01	4,911949512	2,16E+00	1,41E-01	4,75E-01	Slc19a2	1,08	No
ENSMUSG00000040928	-8,92E-02	5,163293733	1,56E+00	2,11E-01	5,50E-01	S100pbp	0,94	No
ENSMUSG00000040929	-1,84E-01	2,660173025	2,22E+00	1,36E-01	4,65E-01	Rfx3	0,88	No
ENSMUSG00000040935	7,53E-01	-2,89613942	3,09E-01	5,78E-01	8,36E-01	Padi6	1,69	No
ENSMUSG00000040936	1,66E-01	5,735355789	6,05E+00	1,39E-02	1,19E-01	Ulk4	1,12	No
ENSMUSG00000040938	-2,26E-01	-0,31106841	4,85E-01	4,86E-01	7,73E-01	Slc16a11	0,85	No
ENSMUSG00000040940	6,61E-02	7,783768384	1,27E+00	2,60E-01	5,53E-01	Arhgef1	1,05	No
ENSMUSG00000040943	5,64E-02	7,74403973	9,18E-01	3,38E-01	6,38E-01	Tet2	1,04	No
ENSMUSG00000040945	-5,86E-04	7,080337659	9,41E-05	9,92E-01	1,00E+00	Rcc2	1,00	No
ENSMUSG00000040950	2,56E-01	9,668846909	2,05E+01	5,86E-06	2,08E-04	Mgl2	1,19	Yes

ENSMUSG00000040952	-7,99E-02	5,619365994	1,39E+00	2,39E-01	5,50E-01	Rps19	0,95	No
ENSMUSG00000040957	3,72E-01	2,897918647	9,95E+00	1,61E-03	2,36E-02	Cables1	1,29	Yes
ENSMUSG00000040963	3,15E-01	7,685936823	2,84E+01	1,00E-07	5,61E-06	Asgr2	1,24	Yes
ENSMUSG00000040964	-8,10E-01	0,814576775	1,31E+01	2,90E-04	5,89E-03	Arhgef10l	0,57	Yes
ENSMUSG00000040969	-2,06E+00	-2,770516624	3,22E+00	7,26E-02	3,42E-01	Arhgef38	0,24	No
ENSMUSG00000040978	2,88E-01	-0,980619578	4,04E-01	5,25E-01	8,02E-01	Gm11992	1,22	No
ENSMUSG00000040985	-6,68E-01	-2,497587458	2,97E-01	5,86E-01	8,39E-01	Sun3	0,63	No
ENSMUSG00000040987	-8,87E-01	-0,378095017	6,77E+00	9,27E-03	8,82E-02	Mill2	0,54	No
ENSMUSG00000040997	-5,93E-02	6,730645938	9,28E-01	3,35E-01	6,36E-01	Abhd4	0,96	No
ENSMUSG00000040998	-7,37E-01	-1,146064406	3,03E+00	8,19E-02	3,68E-01	Npnt	0,60	No
ENSMUSG00000041000	-9,06E-02	3,159878507	7,00E-01	4,03E-01	7,01E-01	Trim62	0,94	No
ENSMUSG00000041012	3,06E-01	0,091261174	1,10E+00	2,93E-01	5,89E-01	Cmtm8	1,24	No
ENSMUSG00000041025	-8,17E-02	5,03425439	1,27E+00	2,60E-01	5,53E-01	Iffo2	0,94	No
ENSMUSG00000041028	-1,52E-01	7,83932794	6,80E+00	9,10E-03	8,70E-02	Ghitm	0,90	No
ENSMUSG00000041035	2,69E-02	3,898199756	8,89E-02	7,66E-01	9,40E-01	Gm17018	1,02	No
ENSMUSG00000041037	2,56E-01	4,818299072	1,15E+01	7,10E-04	1,22E-02	Irgq	1,19	Yes
ENSMUSG00000041040	8,57E-02	6,325021124	1,81E+00	1,78E-01	5,38E-01	Fam117b	1,06	No
ENSMUSG00000041046	-1,05E+00	4,06245903	1,43E+02	7,10E-33	4,84E-30	Ramp3	0,48	Yes
ENSMUSG00000041057	-2,69E-02	5,348364926	1,45E-01	7,04E-01	9,06E-01	Wdr43	0,98	No
ENSMUSG00000041058	7,95E-02	4,776707827	1,07E+00	3,02E-01	5,98E-01	Wwp1	1,06	No
ENSMUSG00000041062	8,22E-01	-2,655170814	5,88E-01	4,43E-01	7,35E-01	Mslnl	1,77	No
ENSMUSG00000041064	2,95E-01	3,147411664	7,71E+00	5,51E-03	6,00E-02	Pif1	1,23	No
ENSMUSG00000041073	-2,97E-01	-0,600640977	6,47E-01	4,21E-01	7,16E-01	Nacad	0,81	No
ENSMUSG00000041075	-1,97E-02	4,881465216	6,81E-02	7,94E-01	9,56E-01	Fzd7	0,99	No
ENSMUSG00000041078	7,06E-01	-0,648402495	3,77E+00	5,23E-02	2,78E-01	Grid1	1,63	No
ENSMUSG00000041079	1,35E-01	2,949510975	1,37E+00	2,42E-01	5,50E-01	Rwdd2b	1,10	No
ENSMUSG00000041084	7,13E-02	6,003656442	1,18E+00	2,77E-01	5,74E-01	Ostc	1,05	No
ENSMUSG00000041112	-1,23E-02	5,922926133	3,51E-02	8,51E-01	9,90E-01	Elmo1	0,99	No
ENSMUSG00000041117	3,56E-01	-1,205805989	6,15E-01	4,33E-01	7,26E-01	Ccdc8	1,28	No
ENSMUSG00000041119	1,21E+00	-1,522781551	4,63E+00	3,13E-02	2,05E-01	Pde9a	2,31	No
ENSMUSG00000041120	-6,83E-01	1,091979795	1,19E+01	5,50E-04	9,92E-03	Nbl1	0,62	Yes
ENSMUSG00000041124	9,34E-02	4,970888647	1,59E+00	2,08E-01	5,50E-01	Msantd4	1,07	No

ENSMUSG00000041126	-1,84E-02	4,527746695	5,24E-02	8,19E-01	9,71E-01	H2afv	0,99	No
ENSMUSG00000041130	-2,37E-01	6,04344992	1,34E+01	2,56E-04	5,32E-03	Zfp598	0,85	Yes
ENSMUSG00000041132	-5,80E-01	5,516363283	7,21E+01	2,03E-17	3,95E-15	N4bp2l1	0,67	Yes
ENSMUSG00000041134	-2,16E-01	1,764940898	1,83E+00	1,77E-01	5,36E-01	Cyyr1	0,86	No
ENSMUSG00000041135	-2,81E-03	5,036945603	1,43E-03	9,70E-01	1,00E+00	Ripk2	1,00	No
ENSMUSG00000041138	2,59E+00	-2,963287563	2,60E+00	1,07E-01	3,99E-01	Nme8	6,03	No
ENSMUSG00000041143	4,38E-02	5,036830728	3,53E-01	5,52E-01	8,18E-01	Tmco4	1,03	No
ENSMUSG00000041144	1,87E+00	-3,107639691	1,38E+00	2,40E-01	5,50E-01	Dnah7b	3,67	No
ENSMUSG00000041147	2,12E-01	4,288633	6,40E+00	1,14E-02	1,03E-01	Brca2	1,16	No
ENSMUSG00000041153	-2,17E-01	5,288177747	9,25E+00	2,36E-03	3,20E-02	Osgin2	0,86	Yes
ENSMUSG00000041161	1,46E-02	3,106073929	1,76E-02	8,94E-01	1,00E+00	Otud3	1,01	No
ENSMUSG00000041164	-1,72E-01	8,358271817	8,89E+00	2,86E-03	3,69E-02	Zmiz2	0,89	Yes
ENSMUSG00000041165	-1,87E+00	-3,107637761	1,39E+00	2,38E-01	5,50E-01	Spem1	0,27	No
ENSMUSG00000041168	7,66E-02	5,946726211	1,35E+00	2,45E-01	5,50E-01	Lonp1	1,05	No
ENSMUSG00000041180	-5,43E-02	2,008645598	1,30E-01	7,19E-01	9,15E-01	Hectd2	0,96	No
ENSMUSG00000041187	2,98E-02	4,583668862	1,44E-01	7,05E-01	9,07E-01	Prkd2	1,02	No
ENSMUSG00000041189	-3,44E-01	1,002025687	3,07E+00	7,95E-02	3,61E-01	Chrnb1	0,79	No
ENSMUSG00000041193	-1,78E+00	-2,070103306	6,33E+00	1,19E-02	1,06E-01	Pla2g5	0,29	No
ENSMUSG00000041199	1,67E-02	3,933381801	3,41E-02	8,54E-01	9,91E-01	Rpusd1	1,01	No
ENSMUSG00000041202	-4,48E-01	-1,033671092	1,07E+00	3,02E-01	5,98E-01	Pla2g2d	0,73	No
ENSMUSG00000041203	2,55E-03	6,145884453	1,55E-03	9,69E-01	1,00E+00	Trir	1,00	No
ENSMUSG00000041205	-9,34E-01	-0,682877005	5,19E+00	2,28E-02	1,64E-01	Map6d1	0,52	No
ENSMUSG00000041215	2,12E-01	4,718337482	7,56E+00	5,96E-03	6,38E-02	Yeats2	1,16	No
ENSMUSG00000041216	-1,10E-01	2,233204079	6,08E-01	4,36E-01	7,28E-01	Clvs1	0,93	No
ENSMUSG00000041219	1,60E-01	6,449159542	6,47E+00	1,10E-02	9,99E-02	Arhgap11a	1,12	No
ENSMUSG00000041220	2,00E-01	4,643157935	6,41E+00	1,14E-02	1,02E-01	Elovl6	1,15	No
ENSMUSG00000041225	5,87E-03	4,914631482	6,20E-03	9,37E-01	1,00E+00	Arhgap12	1,00	No
ENSMUSG00000041231	2,68E-01	3,63165293	7,67E+00	5,62E-03	6,09E-02	Ublcp1	1,20	No
ENSMUSG00000041235	-2,41E-01	6,223462809	1,40E+01	1,85E-04	4,07E-03	Chd7	0,85	Yes
ENSMUSG00000041236	3,50E-02	6,023569982	2,90E-01	5,90E-01	8,41E-01	Vps41	1,02	No
ENSMUSG00000041237	-4,82E-01	-2,2895109	3,04E-01	5,81E-01	8,37E-01	Pklr	0,72	No
ENSMUSG00000041238	-1,02E-01	5,528943876	2,20E+00	1,38E-01	4,68E-01	Rbbp8	0,93	No

ENSMUSG00000041241	1,32E-02	4,987315908	3,18E-02	8,59E-01	9,94E-01	Mul1	1,01	No
ENSMUSG00000041247	-2,36E-01	0,471087078	9,07E-01	3,41E-01	6,42E-01	Lamp3	0,85	No
ENSMUSG00000041248	1,87E+00	-3,107639691	1,38E+00	2,40E-01	5,50E-01	Kcnj1	3,67	No
ENSMUSG00000041258	-1,12E-02	6,098358636	2,99E-02	8,63E-01	9,97E-01	Zfp236	0,99	No
ENSMUSG00000041261	1,81E+00	-3,10765737	1,28E+00	2,58E-01	5,50E-01	Car8	3,49	No
ENSMUSG00000041263	1,47E-01	4,159318273	2,91E+00	8,79E-02	3,79E-01	Rusc1	1,11	No
ENSMUSG00000041264	1,39E-01	5,048304376	3,59E+00	5,80E-02	2,97E-01	Uspl1	1,10	No
ENSMUSG00000041268	-5,37E-03	7,215799151	7,98E-03	9,29E-01	1,00E+00	Dmxl2	1,00	No
ENSMUSG00000041272	5,24E-01	2,15696848	1,21E+01	4,93E-04	9,06E-03	Tox	1,44	Yes
ENSMUSG00000041278	1,61E-02	4,81037125	4,44E-02	8,33E-01	9,79E-01	Ttc1	1,01	No
ENSMUSG00000041287	-1,83E-01	-1,535382771	1,13E-01	7,37E-01	9,25E-01	Sox15	0,88	No
ENSMUSG00000041293	-1,74E+00	-2,655119487	2,10E+00	1,47E-01	4,85E-01	Adgrf1	0,30	No
ENSMUSG00000041297	1,55E-03	6,380645516	6,02E-04	9,80E-01	1,00E+00	Cdk13	1,00	No
ENSMUSG00000041298	2,06E-01	3,468937216	4,32E+00	3,78E-02	2,31E-01	Katnal1	1,15	No
ENSMUSG00000041301	-2,92E+00	-2,033599752	1,40E+01	1,78E-04	3,94E-03	Cftr	0,13	Yes
ENSMUSG00000041303	8,62E-02	4,215084238	1,04E+00	3,08E-01	6,06E-01	Gtf3c3	1,06	No
ENSMUSG00000041308	1,68E-01	5,488727364	5,87E+00	1,54E-02	1,28E-01	Sntb2	1,12	No
ENSMUSG00000041309	1,02E-01	0,454937956	1,57E-01	6,92E-01	9,01E-01	Nkx6-2	1,07	No
ENSMUSG00000041313	1,83E-01	6,259378683	8,14E+00	4,33E-03	5,02E-02	Slc7a1	1,14	No
ENSMUSG00000041319	1,50E-01	3,815212807	2,64E+00	1,04E-01	3,97E-01	Thoc6	1,11	No
ENSMUSG00000041323	4,48E-01	-2,448608679	1,57E-01	6,92E-01	9,00E-01	Ak7	1,36	No
ENSMUSG00000041324	-4,99E-03	6,990602588	6,73E-03	9,35E-01	1,00E+00	Inhba	1,00	No
ENSMUSG00000041328	5,14E-02	6,431942747	6,64E-01	4,15E-01	7,10E-01	Pcf11	1,04	No
ENSMUSG00000041329	7,29E-01	-1,333334074	1,79E+00	1,81E-01	5,43E-01	Atp1b2	1,66	No
ENSMUSG00000041341	1,24E-01	5,923046244	3,56E+00	5,93E-02	3,01E-01	Atg2b	1,09	No
ENSMUSG00000041343	-7,82E-02	0,868127549	1,34E-01	7,14E-01	9,12E-01	Ankrd42	0,95	No
ENSMUSG00000041346	6,88E-02	3,275755772	4,20E-01	5,17E-01	7,98E-01	Wrap53	1,05	No
ENSMUSG00000041347	-4,35E-01	-2,268119106	1,93E-01	6,60E-01	8,82E-01	Bdkrb1	0,74	No
ENSMUSG00000041351	-4,28E-01	3,565854232	1,95E+01	1,01E-05	3,40E-04	Rap1gap	0,74	Yes
ENSMUSG00000041354	6,98E-02	6,383695615	1,22E+00	2,69E-01	5,65E-01	Rgl2	1,05	No
ENSMUSG00000041355	-8,77E-03	6,564526197	1,97E-02	8,88E-01	1,00E+00	Ssr2	0,99	No
ENSMUSG00000041358	-6,24E-01	-1,831991978	7,57E-01	3,84E-01	6,86E-01	Nutm1	0,65	No

ENSMUSG00000041360	8,65E-02	5,11490348	1,41E+00	2,35E-01	5,50E-01	Pum3	1,06	No
ENSMUSG00000041361	-7,92E-01	-0,904007832	3,65E+00	5,59E-02	2,90E-01	Myzap	0,58	No
ENSMUSG00000041362	1,56E-01	7,761967819	7,09E+00	7,77E-03	7,72E-02	Shtn1	1,11	No
ENSMUSG00000041372	1,87E+00	-3,03364371	1,36E+00	2,43E-01	5,50E-01	B4galnt3	3,66	No
ENSMUSG00000041375	-4,93E-02	5,48355615	5,09E-01	4,76E-01	7,64E-01	Ccdc9	0,97	No
ENSMUSG00000041377	-1,94E+00	-3,107619243	1,50E+00	2,21E-01	5,50E-01	Ninj2	0,26	No
ENSMUSG00000041390	2,55E-01	6,668208713	1,69E+01	3,96E-05	1,11E-03	Mdfic	1,19	Yes
ENSMUSG00000041396	1,53E-01	1,502367721	7,63E-01	3,82E-01	6,84E-01	Mettl18	1,11	No
ENSMUSG00000041406	2,40E-01	3,463924218	5,75E+00	1,65E-02	1,34E-01	BC055324	1,18	No
ENSMUSG00000041408	-1,66E-03	7,080739169	7,58E-04	9,78E-01	1,00E+00	Wapl	1,00	No
ENSMUSG00000041415	1,82E-02	6,445879243	8,34E-02	7,73E-01	9,44E-01	Dicer1	1,01	No
ENSMUSG00000041417	-2,42E-01	7,525564942	1,67E+01	4,40E-05	1,22E-03	Pik3r1	0,85	Yes
ENSMUSG00000041420	-1,27E-02	3,580283826	1,66E-02	8,97E-01	1,00E+00	Meis3	0,99	No
ENSMUSG00000041423	-9,71E-01	-1,574006454	3,27E+00	7,05E-02	3,36E-01	Paqr6	0,51	No
ENSMUSG00000041426	-9,04E-02	1,044923233	1,87E-01	6,66E-01	8,85E-01	Hibch	0,94	No
ENSMUSG00000041429	-2,31E-01	1,533637141	1,82E+00	1,77E-01	5,36E-01	Nthl1	0,85	No
ENSMUSG00000041431	1,53E-01	4,819765462	4,06E+00	4,39E-02	2,50E-01	Ccnb1	1,11	No
ENSMUSG00000041438	-1,05E-01	4,497959261	1,67E+00	1,96E-01	5,50E-01	Utp4	0,93	No
ENSMUSG00000041439	2,70E-01	5,666064248	1,59E+01	6,84E-05	1,76E-03	Mfsd6	1,21	Yes
ENSMUSG00000041440	2,07E-01	2,795083198	2,93E+00	8,68E-02	3,78E-01	Gk5	1,15	No
ENSMUSG00000041444	6,39E-01	0,45804099	6,72E+00	9,52E-03	8,99E-02	Arhgap32	1,56	No
ENSMUSG00000041445	-2,79E-02	-2,89614703	4,02E-04	9,84E-01	1,00E+00	Mmrn2	0,98	No
ENSMUSG00000041449	-1,34E-01	-1,816509489	5,53E-02	8,14E-01	9,68E-01	Serpina3h	0,91	No
ENSMUSG00000041453	-3,17E-01	4,522209608	1,57E+01	7,44E-05	1,89E-03	Rpl21	0,80	Yes
ENSMUSG00000041459	-4,72E-02	7,31128948	6,25E-01	4,29E-01	7,23E-01	Tardbp	0,97	No
ENSMUSG00000041460	-2,37E-01	-2,146052242	1,10E-01	7,40E-01	9,26E-01	Cacna2d4	0,85	No
ENSMUSG00000041468	-3,83E-02	-3,107651936	-7,91E-11	1,00E+00	1,00E+00	Gpr12	0,97	No
ENSMUSG00000041471	-1,49E-02	2,786856522	1,55E-02	9,01E-01	1,00E+00	Fam35a	0,99	No
ENSMUSG00000041477	1,33E-01	3,041006965	1,45E+00	2,29E-01	5,50E-01	Dcp1b	1,10	No
ENSMUSG00000041479	8,18E-01	-2,448736636	5,83E-01	4,45E-01	7,36E-01	Syt15	1,76	No
ENSMUSG00000041481	-7,19E-01	5,551773865	1,12E+02	3,84E-26	1,47E-23	Serpina3g	0,61	Yes
ENSMUSG00000041482	-8,42E-01	-2,896108869	3,88E-01	5,33E-01	8,06E-01	Piezo2	0,56	No

ENSMUSG00000041483	-2,23E-02	4,429229839	7,52E-02	7,84E-01	9,51E-01	Zfp281	0,98	No
ENSMUSG00000041488	1,51E-01	6,395930031	5,65E+00	1,74E-02	1,38E-01	Stx3	1,11	No
ENSMUSG00000041491	1,51E-01	3,258792583	1,99E+00	1,58E-01	5,05E-01	Cep78	1,11	No
ENSMUSG00000041498	3,26E-01	4,51519373	1,65E+01	4,89E-05	1,34E-03	Kif14	1,25	Yes
ENSMUSG00000041506	3,93E-02	4,059223354	1,98E-01	6,56E-01	8,80E-01	Rrp9	1,03	No
ENSMUSG00000041515	-4,04E-01	8,158595152	4,86E+01	3,10E-12	3,55E-10	Irf8	0,76	Yes
ENSMUSG00000041523	1,82E+00	-3,033661385	1,31E+00	2,52E-01	5,50E-01	Upk2	3,54	No
ENSMUSG00000041528	-7,88E-02	5,395297771	1,27E+00	2,60E-01	5,52E-01	Rnf123	0,95	No
ENSMUSG00000041530	-2,16E-01	6,53830959	1,20E+01	5,21E-04	9,48E-03	Ago1	0,86	Yes
ENSMUSG00000041536	9,01E-03	-3,107646452	-5,16E-10	1,00E+00	1,00E+00	Serpina3a	1,01	No
ENSMUSG00000041538	2,69E-02	5,427314569	1,46E-01	7,02E-01	9,05E-01	H2-Ob	1,02	No
ENSMUSG00000041540	-2,98E-01	-2,226244558	1,33E-01	7,16E-01	9,13E-01	Sox5	0,81	No
ENSMUSG00000041544	-1,95E+00	-3,033628749	1,50E+00	2,21E-01	5,50E-01	Disp3	0,26	No
ENSMUSG00000041548	7,31E-01	-2,963279337	2,91E-01	5,90E-01	8,41E-01	Hspb8	1,66	No
ENSMUSG00000041550	1,82E+00	-3,033661385	1,31E+00	2,52E-01	5,50E-01	Serpina5	3,54	No
ENSMUSG00000041556	1,22E+00	-2,401349888	9,30E-01	3,35E-01	6,36E-01	Fbxo2	2,34	No
ENSMUSG00000041559	2,65E+00	-2,896119006	2,71E+00	9,98E-02	3,92E-01	Fmod	6,28	No
ENSMUSG00000041560	-1,80E-01	5,887288458	7,40E+00	6,54E-03	6,83E-02	Nop53	0,88	No
ENSMUSG00000041566	-3,72E-02	-2,35554854	1,90E-03	9,65E-01	1,00E+00	Tssk1	0,97	No
ENSMUSG00000041567	1,81E+00	-3,10765737	1,28E+00	2,58E-01	5,50E-01	Serpina12	3,49	No
ENSMUSG00000041570	1,33E-01	6,011679937	4,16E+00	4,15E-02	2,42E-01	Camsap2	1,10	No
ENSMUSG00000041571	-1,69E-01	5,295694191	5,63E+00	1,77E-02	1,39E-01	Selenow	0,89	No
ENSMUSG00000041577	-2,71E+00	-2,929237214	2,91E+00	8,81E-02	3,79E-01	Prelp	0,15	No
ENSMUSG00000041583	3,14E-02	-3,107617333	-7,69E-10	1,00E+00	1,00E+00	Obox6	1,02	No
ENSMUSG00000041592	-2,64E-01	0,338007873	1,13E+00	2,87E-01	5,83E-01	Sdk2	0,83	No
ENSMUSG00000041594	-8,68E-03	2,572017222	4,78E-03	9,45E-01	1,00E+00	Tmtc4	0,99	No
ENSMUSG00000041598	2,03E-01	4,409528356	6,10E+00	1,36E-02	1,17E-01	Cdc42ep4	1,15	No
ENSMUSG00000041605	-3,22E+00	-2,963202658	4,38E+00	3,63E-02	2,26E-01	5730559C18Rik	0,11	No
ENSMUSG00000041607	-2,51E-01	6,636025476	1,63E+01	5,31E-05	1,43E-03	Mbp	0,84	Yes
ENSMUSG00000041608	1,08E+00	-0,268623891	1,03E+01	1,33E-03	2,03E-02	Entpd3	2,11	Yes
ENSMUSG00000041609	6,52E-02	2,083156639	2,06E-01	6,50E-01	8,76E-01	Bicdl1	1,05	No
ENSMUSG00000041616	7,27E-01	-1,912649428	1,19E+00	2,76E-01	5,73E-01	Nppa	1,66	No

ENSMUSG00000041617	1,39E-01	1,531350019	6,63E-01	4,16E-01	7,11E-01	Ccdc74a	1,10	No
ENSMUSG00000041620	2,16E-01	2,970602301	3,38E+00	6,61E-02	3,23E-01	Mmp1b	1,16	No
ENSMUSG00000041623	1,05E-01	3,126960123	9,78E-01	3,23E-01	6,23E-01	D11Wsu47e	1,08	No
ENSMUSG00000041624	2,67E+00	-3,033636951	2,76E+00	9,68E-02	3,88E-01	Gucy1a2	6,36	No
ENSMUSG00000041625	-1,46E-01	1,004477242	4,95E-01	4,82E-01	7,69E-01	Ggact	0,90	No
ENSMUSG00000041629	-7,18E-02	5,176167865	9,93E-01	3,19E-01	6,19E-01	Fam104a	0,95	No
ENSMUSG00000041632	5,73E-02	3,745271955	3,70E-01	5,43E-01	8,12E-01	Mrps27	1,04	No
ENSMUSG00000041638	5,51E-02	6,451804702	7,65E-01	3,82E-01	6,83E-01	Gcn1l1	1,04	No
ENSMUSG00000041642	-1,04E-01	6,42904286	2,74E+00	9,82E-02	3,91E-01	Kif21b	0,93	No
ENSMUSG00000041645	-3,29E-03	6,720698931	2,83E-03	9,58E-01	1,00E+00	Ddx24	1,00	No
ENSMUSG00000041650	-4,56E-02	3,754184216	2,35E-01	6,28E-01	8,61E-01	Pcca	0,97	No
ENSMUSG00000041654	8,62E-02	5,797470534	1,66E+00	1,98E-01	5,50E-01	Slc39a11	1,06	No
ENSMUSG00000041669	-3,66E-01	-2,497640097	1,69E-01	6,81E-01	8,94E-01	Prima1	0,78	No
ENSMUSG00000041671	1,48E-01	3,772884325	2,50E+00	1,14E-01	4,16E-01	Pyroxd1	1,11	No
ENSMUSG00000041673	2,83E-01	0,464521699	1,13E+00	2,87E-01	5,83E-01	Lrrc18	1,22	No
ENSMUSG00000041679	-4,64E-01	1,899850071	9,05E+00	2,63E-03	3,46E-02	Lrrc29	0,73	Yes
ENSMUSG00000041681	-1,87E+00	-3,107637761	1,39E+00	2,38E-01	5,50E-01	Iapp	0,27	No
ENSMUSG00000041684	3,97E-02	3,851255578	1,90E-01	6,63E-01	8,83E-01	Bivm	1,03	No
ENSMUSG00000041685	1,27E-01	7,186215281	4,44E+00	3,51E-02	2,22E-01	Fcho2	1,09	No
ENSMUSG00000041695	8,32E-03	2,472777779	3,73E-03	9,51E-01	1,00E+00	Kcnj2	1,01	No
ENSMUSG00000041696	3,75E-01	1,748934293	5,41E+00	2,00E-02	1,51E-01	Rasl12	1,30	No
ENSMUSG00000041697	-5,08E-02	7,166213479	7,13E-01	3,99E-01	6,98E-01	Cox6a1	0,97	No
ENSMUSG00000041702	-8,90E-03	5,85072848	1,80E-02	8,93E-01	1,00E+00	Btbd7	0,99	No
ENSMUSG00000041703	-2,24E+00	-2,600672422	4,07E+00	4,37E-02	2,49E-01	Zic5	0,21	No
ENSMUSG00000041707	-1,05E-01	4,635742058	1,79E+00	1,81E-01	5,43E-01	1810011H11Rik	0,93	No
ENSMUSG00000041708	-1,37E+00	-2,896128821	1,18E+00	2,77E-01	5,74E-01	Mpped1	0,39	No
ENSMUSG00000041712	-7,68E-03	5,62367772	1,27E-02	9,10E-01	1,00E+00	Ubr7	0,99	No
ENSMUSG00000041716	-1,13E-01	3,195062247	1,11E+00	2,92E-01	5,88E-01	Gm20604	0,92	No
ENSMUSG00000041720	8,64E-02	7,72934681	2,16E+00	1,42E-01	4,76E-01	Pi4ka	1,06	No
ENSMUSG00000041722	-2,02E+00	-2,600619696	3,08E+00	7,94E-02	3,60E-01	Khdc1c	0,25	No
ENSMUSG00000041729	1,08E+00	0,720521308	1,79E+01	2,33E-05	7,09E-04	Coro2b	2,12	Yes
ENSMUSG00000041730	-1,87E+00	-3,107637761	1,39E+00	2,38E-01	5,50E-01	Prrxl1	0,27	No

ENSMUSG00000041731	-5,00E-02	-2,655085571	2,26E-03	9,62E-01	1,00E+00	Pgm5	0,97	No
ENSMUSG00000041733	1,05E-01	4,361717561	1,64E+00	2,01E-01	5,50E-01	Coq5	1,08	No
ENSMUSG00000041734	-8,45E-01	-0,300174396	6,49E+00	1,09E-02	9,93E-02	Kirrel	0,56	No
ENSMUSG00000041736	-1,69E-01	7,670148822	8,24E+00	4,09E-03	4,81E-02	Tspo	0,89	Yes
ENSMUSG00000041737	-8,56E-01	-2,832005432	4,01E-01	5,26E-01	8,03E-01	Tmem45b	0,55	No
ENSMUSG00000041740	7,26E-02	7,468825444	1,50E+00	2,21E-01	5,50E-01	Rnf10	1,05	No
ENSMUSG00000041741	-3,19E+00	-2,896117911	4,30E+00	3,81E-02	2,32E-01	Pde3a	0,11	No
ENSMUSG00000041747	1,86E-01	5,442114742	7,09E+00	7,73E-03	7,70E-02	Utp15	1,14	No
ENSMUSG00000041750	-1,32E-01	2,483125162	1,02E+00	3,11E-01	6,11E-01	Cd1d2	0,91	No
ENSMUSG00000041754	-7,44E-02	0,259293045	7,25E-02	7,88E-01	9,53E-01	Trem3	0,95	No
ENSMUSG00000041757	5,93E-02	3,27389973	3,17E-01	5,73E-01	8,33E-01	Plekha6	1,04	No
ENSMUSG00000041762	3,18E-01	4,003337236	1,28E+01	3,44E-04	6,76E-03	Gpr155	1,25	Yes
ENSMUSG00000041763	1,63E-02	6,504637861	6,71E-02	7,96E-01	9,57E-01	Tpp2	1,01	No
ENSMUSG00000041765	-1,27E-02	5,147736596	3,08E-02	8,61E-01	9,96E-01	Ubac2	0,99	No
ENSMUSG00000041769	-4,29E-02	5,105504321	3,48E-01	5,55E-01	8,20E-01	Ppp2r2d	0,97	No
ENSMUSG00000041771	1,28E+00	-2,655085656	1,01E+00	3,15E-01	6,16E-01	Slc24a4	2,42	No
ENSMUSG00000041773	1,91E-01	4,542493886	5,68E+00	1,71E-02	1,37E-01	Enc1	1,14	No
ENSMUSG00000041774	4,69E-02	3,398984453	2,15E-01	6,43E-01	8,71E-01	Ydjc	1,03	No
ENSMUSG00000041775	3,65E-02	3,219597972	1,22E-01	7,27E-01	9,19E-01	Mapk1ip1	1,03	No
ENSMUSG00000041777	-1,67E-03	3,317421669	2,62E-04	9,87E-01	1,00E+00	Cir1	1,00	No
ENSMUSG00000041779	4,16E-01	3,191238636	1,45E+01	1,41E-04	3,23E-03	Tram2	1,33	Yes
ENSMUSG00000041781	2,44E-01	5,96489806	1,39E+01	1,95E-04	4,26E-03	Cpsf2	1,18	Yes
ENSMUSG00000041782	-8,68E-01	3,984860766	9,84E+01	3,44E-23	1,07E-20	Lad1	0,55	Yes
ENSMUSG00000041789	-3,85E+00	-2,548183328	7,08E+00	7,81E-03	7,76E-02	2700046A07Rik	0,07	No
ENSMUSG00000041794	4,51E-01	2,415999973	1,16E+01	6,43E-04	1,13E-02	Myrip	1,37	Yes
ENSMUSG00000041797	5,42E-02	2,008534793	1,33E-01	7,16E-01	9,13E-01	Abca9	1,04	No
ENSMUSG00000041798	6,23E-01	-2,146227321	7,86E-01	3,75E-01	6,76E-01	Gck	1,54	No
ENSMUSG00000041801	1,28E-02	5,740654386	3,61E-02	8,49E-01	9,89E-01	Phlda3	1,01	No
ENSMUSG00000041809	8,58E-02	-0,880007541	5,06E-02	8,22E-01	9,72E-01	Efhc1	1,06	No
ENSMUSG00000041815	-2,54E-02	7,099709611	1,77E-01	6,74E-01	8,90E-01	Poldip3	0,98	No
ENSMUSG00000041817	-1,25E-01	2,959949153	1,20E+00	2,74E-01	5,70E-01	Fam169a	0,92	No
ENSMUSG00000041827	-7,05E-01	1,847299262	1,90E+01	1,30E-05	4,23E-04	Oasl1	0,61	Yes

ENSMUSG00000041828	-1,35E+00	-2,832022066	1,13E+00	2,87E-01	5,83E-01	Abca8a	0,39	No
ENSMUSG00000041831	1,90E+00	-2,185721494	3,53E+00	6,04E-02	3,04E-01	Sytl3	3,73	No
ENSMUSG00000041836	2,62E-01	7,532939508	1,96E+01	9,76E-06	3,29E-04	Ptpre	1,20	Yes
ENSMUSG00000041837	7,34E-02	3,788519032	6,47E-01	4,21E-01	7,16E-01	Pdcd7	1,05	No
ENSMUSG00000041840	3,06E-01	2,602705489	5,92E+00	1,49E-02	1,25E-01	Haus1	1,24	No
ENSMUSG00000041841	-3,03E-01	3,703661652	1,04E+01	1,26E-03	1,95E-02	Rpl37	0,81	Yes
ENSMUSG00000041842	-1,62E-02	-0,107425074	2,84E-03	9,58E-01	1,00E+00	Fhdc1	0,99	No
ENSMUSG00000041845	1,86E-01	1,299170976	9,86E-01	3,21E-01	6,21E-01	Rhod	1,14	No
ENSMUSG00000041846	-1,38E-02	6,617377665	4,95E-02	8,24E-01	9,73E-01	Ppp4r3a	0,99	No
ENSMUSG00000041849	1,32E-01	2,878778542	1,26E+00	2,62E-01	5,57E-01	Card6	1,10	No
ENSMUSG00000041852	-1,29E-01	6,628808496	4,29E+00	3,84E-02	2,32E-01	Tcf20	0,91	No
ENSMUSG00000041857	-7,66E-01	-1,522661918	2,07E+00	1,50E-01	4,91E-01	Oosp1	0,59	No
ENSMUSG00000041859	1,27E-01	6,783861	4,26E+00	3,90E-02	2,34E-01	Mcm3	1,09	No
ENSMUSG00000041870	-7,59E-02	8,197378321	1,72E+00	1,89E-01	5,50E-01	Ankrd13a	0,95	No
ENSMUSG00000041872	1,24E+00	-2,268225212	2,10E+00	1,48E-01	4,87E-01	Il17f	2,36	No
ENSMUSG00000041878	-5,48E-01	-2,185621157	4,46E-01	5,04E-01	7,89E-01	8430432A02Rik	0,68	No
ENSMUSG00000041879	2,04E-01	6,697459019	1,08E+01	9,93E-04	1,61E-02	Ipo9	1,15	Yes
ENSMUSG00000041881	-3,03E-02	4,988609843	1,66E-01	6,84E-01	8,96E-01	Ndufa7	0,98	No
ENSMUSG00000041886	1,32E+00	-2,600704439	1,76E+00	1,84E-01	5,48E-01	Macc1	2,49	No
ENSMUSG00000041889	5,19E-01	0,010766814	3,33E+00	6,82E-02	3,29E-01	Shisa4	1,43	No
ENSMUSG00000041890	2,08E-02	6,740827874	1,15E-01	7,35E-01	9,24E-01	Git2	1,01	No
ENSMUSG00000041891	1,19E-01	6,060126492	3,33E+00	6,80E-02	3,28E-01	Lman1	1,09	No
ENSMUSG00000041895	7,35E-02	3,84815458	6,38E-01	4,24E-01	7,19E-01	Wipi1	1,05	No
ENSMUSG00000041907	-2,71E+00	-3,033620915	2,92E+00	8,75E-02	3,78E-01	Gpr45	0,15	No
ENSMUSG00000041911	1,82E+00	-3,033667329	1,31E+00	2,52E-01	5,50E-01	Dlx1	3,54	No
ENSMUSG00000041912	-1,02E-01	1,891489341	4,54E-01	5,01E-01	7,86E-01	Tdrkh	0,93	No
ENSMUSG00000041915	1,00E-01	6,211921334	2,46E+00	1,17E-01	4,23E-01	Ammecr1l	1,07	No
ENSMUSG00000041920	-1,61E-01	5,226608342	4,91E+00	2,68E-02	1,83E-01	Slc16a6	0,89	No
ENSMUSG00000041921	1,15E-01	3,630120452	1,43E+00	2,32E-01	5,50E-01	Metap1d	1,08	No
ENSMUSG00000041923	8,29E-01	-2,185717273	1,05E+00	3,05E-01	6,02E-01	Nol4	1,78	No
ENSMUSG00000041926	7,11E-02	6,804328726	1,34E+00	2,47E-01	5,50E-01	Rnpep	1,05	No
ENSMUSG00000041930	5,00E-01	-1,378226832	9,15E-01	3,39E-01	6,39E-01	Fam222a	1,41	No

ENSMUSG00000041935	-2,14E-02	6,289755808	1,13E-01	7,37E-01	9,25E-01	AW549877	0,99	No
ENSMUSG00000041936	4,31E-02	4,634851726	2,98E-01	5,85E-01	8,39E-01	Agrn	1,03	No
ENSMUSG00000041939	2,33E-01	4,448829217	8,22E+00	4,14E-03	4,85E-02	Mvk	1,18	Yes
ENSMUSG00000041945	-8,32E-02	1,223887048	1,90E-01	6,63E-01	8,83E-01	Mfsd9	0,94	No
ENSMUSG00000041949	2,74E-01	2,327486266	3,90E+00	4,83E-02	2,64E-01	Tango6	1,21	No
ENSMUSG00000041954	-6,87E-01	3,795882404	5,68E+01	4,81E-14	6,81E-12	Tnfrsf18	0,62	Yes
ENSMUSG00000041957	1,30E-01	3,217016988	1,51E+00	2,18E-01	5,50E-01	Pkp2	1,09	No
ENSMUSG00000041958	1,21E-01	6,492657855	3,73E+00	5,33E-02	2,82E-01	Pigs	1,09	No
ENSMUSG00000041959	-7,48E-02	8,444328565	1,69E+00	1,93E-01	5,50E-01	S100a10	0,95	No
ENSMUSG00000041961	5,20E-01	1,987804789	1,18E+01	5,91E-04	1,05E-02	Znrf3	1,43	Yes
ENSMUSG00000041966	1,17E-01	4,198322409	1,92E+00	1,66E-01	5,19E-01	Dcaf17	1,08	No
ENSMUSG00000041974	8,23E-02	4,475847274	1,04E+00	3,08E-01	6,07E-01	Spidr	1,06	No
ENSMUSG00000041975	9,41E-02	3,277336213	8,22E-01	3,65E-01	6,66E-01	Mettl8	1,07	No
ENSMUSG00000041977	1,19E-01	6,114756509	3,32E+00	6,84E-02	3,29E-01	Arhgef11	1,09	No
ENSMUSG00000041991	-4,53E-01	-2,655134659	2,10E-01	6,47E-01	8,74E-01	Hrnr	0,73	No
ENSMUSG00000041992	-2,49E-02	3,182215031	5,28E-02	8,18E-01	9,70E-01	Rapgef5	0,98	No
ENSMUSG00000041995	3,40E-02	3,739851556	1,28E-01	7,20E-01	9,15E-01	Zbed3	1,02	No
ENSMUSG00000041997	-3,93E-02	6,341885665	3,83E-01	5,36E-01	8,08E-01	Tlk1	0,97	No
ENSMUSG00000042010	2,01E-01	0,997626585	8,14E-01	3,67E-01	6,68E-01	Acacb	1,15	No
ENSMUSG00000042015	1,49E-01	5,38159215	4,48E+00	3,43E-02	2,18E-01	Wdr41	1,11	No
ENSMUSG00000042029	2,84E-01	6,018085515	1,89E+01	1,40E-05	4,51E-04	Ncapg2	1,22	Yes
ENSMUSG00000042032	-8,79E-02	6,029494259	1,82E+00	1,77E-01	5,37E-01	Mat2b	0,94	No
ENSMUSG00000042035	-4,62E-01	1,949933952	9,64E+00	1,90E-03	2,69E-02	Igsf3	0,73	Yes
ENSMUSG00000042041	-3,56E+00	-2,896109226	5,70E+00	1,70E-02	1,36E-01	2010003K11Rik	0,08	No
ENSMUSG00000042042	2,44E-02	5,801705707	1,34E-01	7,15E-01	9,12E-01	Csgalnact2	1,02	No
ENSMUSG00000042043	4,00E-02	3,914274645	1,98E-01	6,57E-01	8,80E-01	Tbca	1,03	No
ENSMUSG00000042046	3,22E-03	5,28671229	2,07E-03	9,64E-01	1,00E+00	Dstyk	1,00	No
ENSMUSG00000042050	1,12E-01	2,187069845	6,04E-01	4,37E-01	7,30E-01	Wdr60	1,08	No
ENSMUSG00000042055	-2,63E-02	5,864351463	1,57E-01	6,92E-01	9,00E-01	Wdr11	0,98	No
ENSMUSG00000042063	2,08E-03	4,747725513	7,33E-04	9,78E-01	1,00E+00	Zfp386	1,00	No
ENSMUSG00000042064	1,55E-01	-2,107565084	4,16E-02	8,38E-01	9,82E-01	Myo3b	1,11	No
ENSMUSG00000042066	-3,19E-02	2,420611884	6,04E-02	8,06E-01	9,63E-01	Tmcc2	0,98	No

ENSMUSG00000042073	-1,15E-01	3,409806258	1,31E+00	2,52E-01	5,50E-01	Abhd14b	0,92	No
ENSMUSG00000042078	4,87E-01	-1,711701943	6,93E-01	4,05E-01	7,02E-01	Svop	1,40	No
ENSMUSG00000042079	3,79E-02	8,284776387	4,32E-01	5,11E-01	7,94E-01	Hnrnpf	1,03	No
ENSMUSG00000042082	3,18E-01	4,77138314	1,68E+01	4,24E-05	1,18E-03	Arsb	1,25	Yes
ENSMUSG00000042087	-4,46E-02	3,680437359	2,16E-01	6,42E-01	8,71E-01	4933440N22Rik	0,97	No
ENSMUSG00000042096	-9,21E-01	-1,401370707	3,71E+00	5,40E-02	2,84E-01	Dao	0,53	No
ENSMUSG00000042097	-7,46E-02	-0,257667494	5,16E-02	8,20E-01	9,71E-01	Zfp239	0,95	No
ENSMUSG00000042099	-2,67E-02	4,771720898	1,21E-01	7,28E-01	9,20E-01	Kank3	0,98	No
ENSMUSG00000042102	3,14E-02	-3,107617333	-7,69E-10	1,00E+00	1,00E+00	Dmgdh	1,02	No
ENSMUSG00000042104	2,77E-01	0,103654973	9,68E-01	3,25E-01	6,26E-01	Uggt2	1,21	No
ENSMUSG00000042105	-3,78E-02	5,683570751	3,13E-01	5,76E-01	8,34E-01	Inpp5f	0,97	No
ENSMUSG00000042106	-1,45E-01	0,844350771	4,87E-01	4,85E-01	7,73E-01	Fam212a	0,90	No
ENSMUSG00000042109	-2,14E+00	-1,831966423	8,13E+00	4,36E-03	5,04E-02	Csdc2	0,23	No
ENSMUSG00000042111	-2,06E-02	5,870293528	9,60E-02	7,57E-01	9,35E-01	Ccdc115	0,99	No
ENSMUSG00000042115	7,05E-01	-2,83200187	2,71E-01	6,03E-01	8,48E-01	Klhdc8a	1,63	No
ENSMUSG00000042116	3,23E-01	-0,793322198	6,67E-01	4,14E-01	7,09E-01	Vwa1	1,25	No
ENSMUSG00000042121	-3,62E-01	6,250828983	3,22E+01	1,37E-08	9,21E-07	Ssh1	0,78	Yes
ENSMUSG00000042129	2,40E-01	9,179184299	1,79E+01	2,35E-05	7,15E-04	Rassf4	1,18	Yes
ENSMUSG00000042133	-5,77E-02	6,105108088	7,85E-01	3,76E-01	6,76E-01	Ppig	0,96	No
ENSMUSG00000042138	1,44E-01	2,97797732	1,67E+00	1,96E-01	5,50E-01	Msantd2	1,11	No
ENSMUSG00000042148	2,69E-01	4,221582011	9,95E+00	1,61E-03	2,35E-02	Cox10	1,21	Yes
ENSMUSG00000042155	7,99E-02	1,399263741	1,92E-01	6,61E-01	8,82E-01	Klhl23	1,06	No
ENSMUSG00000042156	-8,06E-01	0,997397438	1,54E+01	8,54E-05	2,13E-03	Dzip1	0,57	Yes
ENSMUSG00000042165	-7,10E-02	6,110123768	1,20E+00	2,74E-01	5,70E-01	Gm9774	0,95	No
ENSMUSG00000042167	-9,20E-02	5,980571084	1,96E+00	1,61E-01	5,10E-01	Papd4	0,94	No
ENSMUSG00000042178	-8,24E-03	5,360464697	1,35E-02	9,07E-01	1,00E+00	Armc5	0,99	No
ENSMUSG00000042182	3,85E-01	1,786543978	5,28E+00	2,16E-02	1,58E-01	Bend6	1,31	No
ENSMUSG00000042185	4,17E-02	5,21419985	3,40E-01	5,60E-01	8,23E-01	Nfrkb	1,03	No
ENSMUSG00000042189	1,14E+00	-2,600691052	1,24E+00	2,65E-01	5,60E-01	Tekt3	2,21	No
ENSMUSG00000042190	3,49E-01	7,274875952	3,38E+01	6,05E-09	4,38E-07	Cmklr1	1,27	Yes
ENSMUSG00000042195	5,25E-01	-1,655161678	9,43E-01	3,32E-01	6,32E-01	Slc35f2	1,44	No
ENSMUSG00000042197	3,22E-02	5,754229564	2,31E-01	6,31E-01	8,63E-01	Zfp451	1,02	No

ENSMUSG00000042198	-5,06E-02	3,585867972	2,74E-01	6,01E-01	8,47E-01	Chchd7	0,97	No
ENSMUSG00000042202	1,41E-01	4,40432434	3,00E+00	8,31E-02	3,70E-01	Slc35e2	1,10	No
ENSMUSG00000042203	6,54E-02	4,869167319	7,44E-01	3,88E-01	6,88E-01	Tbc1d22b	1,05	No
ENSMUSG00000042207	-1,78E-01	4,395836523	4,78E+00	2,88E-02	1,93E-01	Kdm5b	0,88	No
ENSMUSG00000042208	7,38E-02	5,427453426	1,12E+00	2,90E-01	5,86E-01	0610010F05Rik	1,05	No
ENSMUSG00000042210	8,94E-02	2,616452371	5,01E-01	4,79E-01	7,66E-01	Abhd14a	1,06	No
ENSMUSG00000042211	1,55E-02	6,014012932	5,67E-02	8,12E-01	9,67E-01	Fbxo38	1,01	No
ENSMUSG00000042213	1,83E-01	2,956959547	2,63E+00	1,05E-01	3,97E-01	Zfand4	1,14	No
ENSMUSG00000042215	1,96E-01	2,925493317	2,91E+00	8,80E-02	3,79E-01	Bag2	1,15	No
ENSMUSG00000042216	4,11E-01	1,136099039	4,18E+00	4,09E-02	2,40E-01	Sgsm1	1,33	No
ENSMUSG00000042228	2,00E-01	7,47448762	1,13E+01	7,89E-04	1,33E-02	Lyn	1,15	Yes
ENSMUSG00000042229	-4,26E-02	4,656358372	2,94E-01	5,88E-01	8,40E-01	Rabif	0,97	No
ENSMUSG00000042246	-1,09E-01	-2,71174857	7,86E-03	9,29E-01	1,00E+00	Tmc7	0,93	No
ENSMUSG00000042249	-2,79E-01	7,104015648	2,15E+01	3,56E-06	1,36E-04	Grk3	0,82	Yes
ENSMUSG00000042250	-1,87E+00	-3,107637761	1,39E+00	2,38E-01	5,50E-01	Pglyrp4	0,27	No
ENSMUSG00000042251	-4,86E-01	0,236943162	3,36E+00	6,67E-02	3,25E-01	Pm20d1	0,71	No
ENSMUSG00000042254	1,87E+00	-3,107639691	1,38E+00	2,40E-01	5,50E-01	Cilp	3,67	No
ENSMUSG00000042265	-2,56E-01	5,339575126	1,27E+01	3,66E-04	7,11E-03	Trem1	0,84	Yes
ENSMUSG00000042268	3,42E-01	-1,051861663	6,87E-01	4,07E-01	7,04E-01	Slc26a9	1,27	No
ENSMUSG00000042269	3,86E-01	-2,711735111	1,16E-01	7,34E-01	9,23E-01	Fam92b	1,31	No
ENSMUSG00000042272	8,64E-02	6,141550545	1,80E+00	1,80E-01	5,42E-01	Sestd1	1,06	No
ENSMUSG00000042275	-3,30E-02	3,197803763	9,28E-02	7,61E-01	9,37E-01	Pelo	0,98	No
ENSMUSG00000042279	7,57E-01	-1,44869789	1,93E+00	1,65E-01	5,17E-01	H1foo	1,69	No
ENSMUSG00000042284	9,59E-02	6,848409342	2,44E+00	1,18E-01	4,27E-01	Itga1	1,07	No
ENSMUSG00000042286	3,87E-02	4,048282612	1,90E-01	6,63E-01	8,83E-01	Stab1	1,03	No
ENSMUSG00000042289	-7,10E-02	4,628777466	8,24E-01	3,64E-01	6,65E-01	Hsd3b7	0,95	No
ENSMUSG00000042292	1,33E-03	7,205685259	4,94E-04	9,82E-01	1,00E+00	Mkl1	1,00	No
ENSMUSG00000042293	-1,51E-01	1,935694833	9,44E-01	3,31E-01	6,32E-01	Gm5617	0,90	No
ENSMUSG00000042298	2,64E-02	3,940230223	8,63E-02	7,69E-01	9,42E-01	Ttc19	1,02	No
ENSMUSG00000042302	1,96E-01	4,593386472	6,07E+00	1,37E-02	1,18E-01	Ehbp1	1,15	No
ENSMUSG00000042303	2,47E-01	4,865134435	1,05E+01	1,18E-03	1,86E-02	Sgsm3	1,19	Yes
ENSMUSG00000042305	-1,43E-02	5,833986777	4,65E-02	8,29E-01	9,77E-01	Tmem183a	0,99	No

ENSMUSG00000042306	-7,58E-02	-2,268073675	9,32E-03	9,23E-01	1,00E+00	S100a14	0,95	No
ENSMUSG00000042308	3,86E-03	5,867099618	3,36E-03	9,54E-01	1,00E+00	Setd1a	1,00	No
ENSMUSG00000042312	-5,35E-02	5,208128195	5,57E-01	4,55E-01	7,45E-01	S100a13	0,96	No
ENSMUSG00000042320	7,75E-02	1,585317268	2,14E-01	6,44E-01	8,72E-01	Prox2	1,06	No
ENSMUSG00000042323	6,28E-02	6,83172623	1,05E+00	3,06E-01	6,03E-01	Pbrm1	1,04	No
ENSMUSG00000042328	8,85E-02	4,818609928	1,36E+00	2,43E-01	5,50E-01	Hps4	1,06	No
ENSMUSG00000042331	1,20E-01	6,136371191	3,47E+00	6,26E-02	3,12E-01	Specc1	1,09	No
ENSMUSG00000042333	1,81E-01	3,638860742	3,63E+00	5,68E-02	2,93E-01	Tnfrsf14	1,13	No
ENSMUSG00000042340	7,46E-01	-2,770613093	3,02E-01	5,82E-01	8,38E-01	Ctf1	1,68	No
ENSMUSG00000042345	-9,77E-01	-2,4486688	1,16E+00	2,82E-01	5,79E-01	Ubash3a	0,51	No
ENSMUSG00000042348	1,77E-01	3,277657273	2,72E+00	9,93E-02	3,92E-01	Arl15	1,13	No
ENSMUSG00000042349	-2,36E-01	6,01120554	1,32E+01	2,73E-04	5,63E-03	Ikbke	0,85	Yes
ENSMUSG00000042350	1,66E-02	6,05995829	6,52E-02	7,98E-01	9,59E-01	Arel1	1,01	No
ENSMUSG00000042351	2,99E-01	4,370237089	1,28E+01	3,52E-04	6,89E-03	Grap2	1,23	Yes
ENSMUSG00000042354	5,26E-03	5,410845712	5,59E-03	9,40E-01	1,00E+00	Gnl3	1,00	No
ENSMUSG00000042359	-2,80E-02	-0,436650935	6,45E-03	9,36E-01	1,00E+00	Osbpl6	0,98	No
ENSMUSG00000042363	-7,53E-02	2,882997921	4,25E-01	5,14E-01	7,96E-01	Lgalsl	0,95	No
ENSMUSG00000042364	-4,60E-02	6,267667721	5,16E-01	4,72E-01	7,60E-01	Snx18	0,97	No
ENSMUSG00000042367	-5,88E-02	-3,033616507	1,62E-03	9,68E-01	1,00E+00	Gjb3	0,96	No
ENSMUSG00000042369	-3,05E-02	4,91012391	1,67E-01	6,83E-01	8,95E-01	Rbm45	0,98	No
ENSMUSG00000042371	8,46E-01	-2,401378208	8,60E-01	3,54E-01	6,55E-01	Slc5a10	1,80	No
ENSMUSG00000042372	-1,87E+00	-3,107637761	1,39E+00	2,38E-01	5,50E-01	Dmrt3	0,27	No
ENSMUSG00000042377	7,57E-01	-2,401435727	7,61E-01	3,83E-01	6,85E-01	Fam83g	1,69	No
ENSMUSG00000042380	-7,00E-02	4,521184998	7,54E-01	3,85E-01	6,86E-01	Smim12	0,95	No
ENSMUSG00000042385	-2,65E+00	-3,033633091	2,78E+00	9,55E-02	3,86E-01	Gzmk	0,16	No
ENSMUSG00000042388	-1,38E+00	0,359085868	2,92E+01	6,61E-08	3,83E-06	Dlgap3	0,38	Yes
ENSMUSG00000042389	7,70E-02	2,215706092	3,01E-01	5,83E-01	8,38E-01	Tsen2	1,05	No
ENSMUSG00000042390	-4,86E-02	6,788342128	6,28E-01	4,28E-01	7,22E-01	Gatad2b	0,97	No
ENSMUSG00000042396	2,72E-02	6,41017548	1,86E-01	6,67E-01	8,86E-01	Rbm7	1,02	No
ENSMUSG00000042401	-1,32E+00	-2,896135995	1,09E+00	2,97E-01	5,93E-01	Crtac1	0,40	No
ENSMUSG00000042404	7,54E-02	6,945400329	1,54E+00	2,15E-01	5,50E-01	Dennd4b	1,05	No
ENSMUSG00000042406	-4,55E-02	7,787708594	6,01E-01	4,38E-01	7,31E-01	Atf4	0,97	No

ENSMUSG00000042408	-2,76E-03	4,439353758	1,17E-03	9,73E-01	1,00E+00	Zmym6	1,00	No
ENSMUSG00000042410	4,74E-02	7,194795939	6,24E-01	4,29E-01	7,23E-01	Agps	1,03	No
ENSMUSG00000042414	7,25E-01	-2,896182007	2,86E-01	5,93E-01	8,42E-01	Prdm14	1,65	No
ENSMUSG00000042417	-9,63E-02	1,6554297	3,35E-01	5,63E-01	8,26E-01	Ccno	0,94	No
ENSMUSG00000042419	-2,35E-01	4,014807266	7,20E+00	7,30E-03	7,41E-02	Nfkbil1	0,85	No
ENSMUSG00000042423	-6,31E-03	7,002870999	1,09E-02	9,17E-01	1,00E+00	Fbrs	1,00	No
ENSMUSG00000042426	7,85E-02	5,18551344	1,20E+00	2,74E-01	5,70E-01	Dhx29	1,06	No
ENSMUSG00000042428	-1,37E+00	-0,484924239	1,49E+01	1,15E-04	2,73E-03	Mgat3	0,39	Yes
ENSMUSG00000042429	1,81E+00	-3,10765737	1,28E+00	2,58E-01	5,50E-01	Adora1	3,49	No
ENSMUSG00000042436	-2,78E-01	-0,98036455	4,63E-01	4,96E-01	7,81E-01	Mfap4	0,82	No
ENSMUSG00000042439	2,49E-01	3,800847471	7,10E+00	7,71E-03	7,69E-02	Zfp532	1,19	No
ENSMUSG00000042444	1,94E-01	6,241939717	9,27E+00	2,33E-03	3,16E-02	Mindy2	1,14	Yes
ENSMUSG00000042446	-1,23E-01	4,92427762	2,71E+00	9,96E-02	3,92E-01	Zmym4	0,92	No
ENSMUSG00000042447	-1,27E-02	3,96643183	2,04E-02	8,87E-01	1,00E+00	Mios	0,99	No
ENSMUSG00000042448	9,01E-03	-3,107646452	-5,16E-10	1,00E+00	1,00E+00	Hoxd1	1,01	No
ENSMUSG00000042451	1,87E+00	-3,107639691	1,38E+00	2,40E-01	5,50E-01	Mybph	3,67	No
ENSMUSG00000042453	-7,38E-02	-3,033634644	2,66E-03	9,59E-01	1,00E+00	Reln	0,95	No
ENSMUSG00000042460	1,25E-01	5,608632842	3,34E+00	6,75E-02	3,27E-01	C1galt1	1,09	No
ENSMUSG00000042462	1,78E-01	4,626040975	4,98E+00	2,56E-02	1,78E-01	Dctpp1	1,13	No
ENSMUSG00000042472	4,62E-03	5,173709143	4,12E-03	9,49E-01	1,00E+00	Zfp410	1,00	No
ENSMUSG00000042474	-5,65E-01	-0,997934223	1,58E+00	2,08E-01	5,50E-01	Fcmr	0,68	No
ENSMUSG00000042476	3,86E-01	-0,621009193	1,05E+00	3,05E-01	6,03E-01	Abcb4	1,31	No
ENSMUSG00000042477	-6,18E-01	-2,655166424	2,99E-01	5,85E-01	8,38E-01	Tfap2e	0,65	No
ENSMUSG00000042485	-6,77E-01	-0,998103514	2,62E+00	1,05E-01	3,97E-01	Mustn1	0,63	No
ENSMUSG00000042487	2,60E-02	4,040908378	8,57E-02	7,70E-01	9,42E-01	Leo1	1,02	No
ENSMUSG00000042489	2,71E-01	5,250721263	1,44E+01	1,51E-04	3,41E-03	Clspn	1,21	Yes
ENSMUSG00000042492	1,24E-01	7,093218046	4,21E+00	4,02E-02	2,38E-01	Tbc1d10b	1,09	No
ENSMUSG00000042496	7,72E-02	4,384327649	8,87E-01	3,46E-01	6,47E-01	Prdm10	1,05	No
ENSMUSG00000042500	9,53E-02	4,738922371	1,53E+00	2,16E-01	5,50E-01	Ago4	1,07	No
ENSMUSG00000042501	-5,88E-02	-3,033652696	1,62E-03	9,68E-01	1,00E+00	Cpa6	0,96	No
ENSMUSG00000042502	-1,08E-01	6,085966134	2,76E+00	9,67E-02	3,88E-01	Cd2bp2	0,93	No
ENSMUSG00000042505	1,48E-01	1,752682944	8,25E-01	3,64E-01	6,65E-01	Sdhaf3	1,11	No

ENSMUSG00000042506	1,06E-01	6,457873164	2,83E+00	9,26E-02	3,83E-01	Usp22	1,08	No
ENSMUSG00000042507	-4,21E-02	6,788158814	4,65E-01	4,95E-01	7,81E-01	Elmsan1	0,97	No
ENSMUSG00000042508	-1,59E-01	5,649877898	5,53E+00	1,87E-02	1,45E-01	Dmtf1	0,90	No
ENSMUSG00000042510	-2,80E-01	-0,497672099	6,10E-01	4,35E-01	7,28E-01	AA986860	0,82	No
ENSMUSG00000042514	-2,97E-01	-2,800970853	7,51E-02	7,84E-01	9,51E-01	Klhl14	0,81	No
ENSMUSG00000042520	6,88E-02	7,310991336	1,33E+00	2,50E-01	5,50E-01	Ubap2l	1,05	No
ENSMUSG00000042523	2,54E-01	2,68992642	4,60E+00	3,19E-02	2,07E-01	Dnal1	1,19	No
ENSMUSG00000042524	2,96E-02	7,686646507	2,54E-01	6,14E-01	8,53E-01	Sun2	1,02	No
ENSMUSG00000042529	1,21E-02	-3,033650469	7,34E-11	1,00E+00	1,00E+00	Kcnj12	1,01	No
ENSMUSG00000042532	-9,36E-01	-0,682954634	6,57E+00	1,04E-02	9,60E-02	Golga7b	0,52	No
ENSMUSG00000042535	-1,73E-01	6,50388252	7,68E+00	5,58E-03	6,06E-02	Gtpbp1	0,89	No
ENSMUSG00000042540	-5,88E-02	-3,033652696	1,62E-03	9,68E-01	1,00E+00	Acot5	0,96	No
ENSMUSG00000042541	-9,45E-03	6,686632001	2,33E-02	8,79E-01	1,00E+00	Sem1	0,99	No
ENSMUSG00000042548	5,97E-02	6,432730673	9,00E-01	3,43E-01	6,43E-01	Asxl1	1,04	No
ENSMUSG00000042549	-9,81E-02	-0,503801016	8,22E-02	7,74E-01	9,45E-01	Map2k3os	0,93	No
ENSMUSG00000042557	1,74E-02	5,956375436	7,03E-02	7,91E-01	9,55E-01	Sin3a	1,01	No
ENSMUSG00000042558	4,22E-02	4,947408489	3,19E-01	5,72E-01	8,32E-01	Adprhl2	1,03	No
ENSMUSG00000042564	1,02E-01	-0,216105576	1,09E-01	7,42E-01	9,27E-01	Fam227a	1,07	No
ENSMUSG00000042567	-2,03E-01	-0,980582241	2,32E-01	6,30E-01	8,63E-01	Nek10	0,87	No
ENSMUSG00000042569	1,18E-01	3,849007029	1,66E+00	1,97E-01	5,50E-01	Dhrs7b	1,09	No
ENSMUSG00000042570	2,08E-01	4,791544001	7,43E+00	6,41E-03	6,73E-02	Mier2	1,16	No
ENSMUSG00000042572	-9,72E-02	6,787121709	2,50E+00	1,14E-01	4,16E-01	Ube2q1	0,93	No
ENSMUSG00000042579	1,22E-01	1,965993395	6,99E-01	4,03E-01	7,01E-01	4632404H12Rik	1,09	No
ENSMUSG00000042589	-8,78E-01	-0,963139848	4,26E+00	3,89E-02	2,34E-01	Cux2	0,54	No
ENSMUSG00000042590	1,58E-01	4,43366219	3,80E+00	5,12E-02	2,75E-01	Ipo11	1,12	No
ENSMUSG00000042594	-7,01E-02	7,170803074	1,36E+00	2,43E-01	5,50E-01	Sh2b3	0,95	No
ENSMUSG00000042599	2,69E-01	7,226395914	2,00E+01	7,87E-06	2,72E-04	Kdm7a	1,21	Yes
ENSMUSG00000042605	-2,19E-02	5,603225459	1,02E-01	7,49E-01	9,31E-01	Atxn2	0,98	No
ENSMUSG00000042606	1,04E-01	4,385477477	1,62E+00	2,03E-01	5,50E-01	Hirip3	1,07	No
ENSMUSG00000042607	2,18E-01	2,960025912	3,72E+00	5,36E-02	2,83E-01	Asb4	1,16	No
ENSMUSG00000042608	-1,04E-02	6,051269564	2,57E-02	8,73E-01	1,00E+00	Stk40	0,99	No
ENSMUSG00000042613	-2,59E-01	7,861673346	1,96E+01	9,60E-06	3,25E-04	Pbxip1	0,84	Yes

ENSMUSG00000042616	-1,75E-01	0,451903379	5,03E-01	4,78E-01	7,66E-01	Oscp1	0,89	No
ENSMUSG00000042622	4,38E-02	5,360516245	3,81E-01	5,37E-01	8,09E-01	Maff	1,03	No
ENSMUSG00000042625	-4,12E-03	6,934190499	4,57E-03	9,46E-01	1,00E+00	Safb2	1,00	No
ENSMUSG00000042626	1,49E-01	7,253472673	6,21E+00	1,27E-02	1,11E-01	Shc1	1,11	No
ENSMUSG00000042628	-1,23E-01	5,627593167	3,30E+00	6,93E-02	3,32E-01	Zfyve1	0,92	No
ENSMUSG00000042631	-8,24E-01	-2,831988991	3,73E-01	5,42E-01	8,11E-01	Xkr7	0,56	No
ENSMUSG00000042632	1,13E-01	3,401130078	1,22E+00	2,69E-01	5,65E-01	Pla2g6	1,08	No
ENSMUSG00000042638	-1,42E+00	-1,267886491	6,66E+00	9,84E-03	9,22E-02	Gucy2c	0,37	No
ENSMUSG00000042641	1,38E+00	-1,62775734	4,94E+00	2,62E-02	1,81E-01	Rgsl1	2,61	No
ENSMUSG00000042642	4,60E-02	4,984903627	3,88E-01	5,33E-01	8,06E-01	Flad1	1,03	No
ENSMUSG00000042644	-1,07E-01	5,036061716	2,13E+00	1,44E-01	4,80E-01	Itpr3	0,93	No
ENSMUSG00000042647	7,02E-02	3,615022864	5,33E-01	4,66E-01	7,54E-01	Acad12	1,05	No
ENSMUSG00000042650	1,60E-01	6,879130235	6,84E+00	8,93E-03	8,58E-02	Alkbh5	1,12	No
ENSMUSG00000042655	-2,68E+00	-3,033645766	2,84E+00	9,17E-02	3,82E-01	Fam159b	0,16	No
ENSMUSG00000042659	-3,65E-02	6,023754669	3,12E-01	5,76E-01	8,35E-01	Arrdc4	0,98	No
ENSMUSG00000042660	1,03E-01	4,080932874	1,38E+00	2,39E-01	5,50E-01	Wdr55	1,07	No
ENSMUSG00000042662	-5,77E-01	-2,7706185	2,61E-01	6,09E-01	8,51E-01	Dusp15	0,67	No
ENSMUSG00000042670	-3,25E-02	0,831820586	2,19E-02	8,82E-01	1,00E+00	Immp1l	0,98	No
ENSMUSG00000042671	-3,18E+00	-2,831985843	4,27E+00	3,87E-02	2,33E-01	Rgs8	0,11	No
ENSMUSG00000042672	-2,17E-01	-0,401352225	3,96E-01	5,29E-01	8,05E-01	Dcst1	0,86	No
ENSMUSG00000042675	-1,28E-01	5,363930392	3,35E+00	6,74E-02	3,27E-01	Ypel3	0,91	No
ENSMUSG00000042677	7,06E-02	5,6055767	1,06E+00	3,03E-01	5,99E-01	Zc3h12a	1,05	No
ENSMUSG00000042678	4,37E-01	0,736716305	3,77E+00	5,21E-02	2,78E-01	Myo15	1,35	No
ENSMUSG00000042680	6,59E-01	-1,146235132	2,07E+00	1,51E-01	4,92E-01	Garem1	1,58	No
ENSMUSG00000042682	8,78E-02	4,797440554	1,30E+00	2,54E-01	5,50E-01	Selenok	1,06	No
ENSMUSG00000042684	9,22E-02	1,997571038	3,71E-01	5,42E-01	8,12E-01	Npl	1,07	No
ENSMUSG00000042686	-1,19E+00	-2,311082735	1,83E+00	1,76E-01	5,35E-01	Jph1	0,44	No
ENSMUSG00000042688	1,17E-01	6,721364681	3,58E+00	5,86E-02	2,99E-01	Mapk6	1,08	No
ENSMUSG00000042694	2,15E-01	4,252520841	6,51E+00	1,07E-02	9,83E-02	Stn1	1,16	No
ENSMUSG00000042699	5,52E-02	7,473796459	8,68E-01	3,52E-01	6,53E-01	Dhx9	1,04	No
ENSMUSG00000042700	-2,63E-02	4,392421922	1,04E-01	7,47E-01	9,30E-01	Sipa1l1	0,98	No
ENSMUSG00000042705	1,31E-01	4,69132831	2,82E+00	9,29E-02	3,83E-01	Commd10	1,09	No

ENSMUSG00000042707	1,86E-01	-1,32218157	1,37E-01	7,11E-01	9,11E-01	Dnali1	1,14	No
ENSMUSG00000042708	2,10E-01	2,967473948	3,35E+00	6,74E-02	3,27E-01	Shcbp1l	1,16	No
ENSMUSG00000042709	-2,26E-02	4,303078939	7,28E-02	7,87E-01	9,53E-01	Atpaf2	0,98	No
ENSMUSG00000042719	6,18E-02	4,925953748	6,78E-01	4,10E-01	7,07E-01	Naa25	1,04	No
ENSMUSG00000042724	-1,45E-01	2,488854283	1,20E+00	2,73E-01	5,69E-01	Map3k9	0,90	No
ENSMUSG00000042726	-1,77E-01	7,074025332	8,59E+00	3,38E-03	4,18E-02	Trafd1	0,88	Yes
ENSMUSG00000042729	-1,69E-02	3,036923356	2,27E-02	8,80E-01	1,00E+00	Wdr74	0,99	No
ENSMUSG00000042734	-6,34E-01	0,095986248	5,04E+00	2,48E-02	1,74E-01	Ttc9	0,64	No
ENSMUSG00000042737	2,33E-01	3,109030888	4,41E+00	3,58E-02	2,24E-01	Dpm3	1,18	No
ENSMUSG00000042742	8,22E-02	5,997948473	1,60E+00	2,07E-01	5,50E-01	Bmt2	1,06	No
ENSMUSG00000042743	-1,75E-01	0,638730607	5,56E-01	4,56E-01	7,45E-01	Sgtb	0,89	No
ENSMUSG00000042744	2,29E-03	6,314094783	1,29E-03	9,71E-01	1,00E+00	Hectd4	1,00	No
ENSMUSG00000042745	5,48E-01	3,27121473	2,48E+01	6,22E-07	2,90E-05	Id1	1,46	Yes
ENSMUSG00000042747	7,90E-02	4,680865311	1,02E+00	3,13E-01	6,12E-01	Krtcap2	1,06	No
ENSMUSG00000042751	-3,58E+00	-2,831988971	5,75E+00	1,65E-02	1,34E-01	Nmnat2	0,08	No
ENSMUSG00000042757	-5,42E-01	-2,146095053	4,72E-01	4,92E-01	7,78E-01	Tmem108	0,69	No
ENSMUSG00000042759	1,02E-01	6,822924664	2,76E+00	9,65E-02	3,88E-01	Apobr	1,07	No
ENSMUSG00000042761	-4,12E-01	-1,997982224	3,53E-01	5,53E-01	8,19E-01	Mrap2	0,75	No
ENSMUSG00000042763	-1,01E+00	-0,824009336	5,95E+00	1,47E-02	1,24E-01	Maneal	0,49	No
ENSMUSG00000042766	-3,92E-01	0,977528092	3,76E+00	5,25E-02	2,79E-01	Trim46	0,76	No
ENSMUSG00000042770	1,97E-01	4,385983058	5,70E+00	1,69E-02	1,36E-01	Hebp1	1,15	No
ENSMUSG00000042772	-1,29E-02	6,209721479	4,03E-02	8,41E-01	9,84E-01	Smg7	0,99	No
ENSMUSG00000042784	8,75E-02	-1,770621411	2,05E-02	8,86E-01	1,00E+00	Muc1	1,06	No
ENSMUSG00000042787	2,66E-01	3,544664547	7,53E+00	6,06E-03	6,47E-02	Exog	1,20	No
ENSMUSG00000042788	2,73E-02	-1,497680144	2,35E-03	9,61E-01	1,00E+00	Fam166b	1,02	No
ENSMUSG00000042790	-2,18E-02	4,706492879	7,91E-02	7,79E-01	9,47E-01	Rnf214	0,98	No
ENSMUSG00000042793	-2,02E+00	-2,711712949	3,10E+00	7,81E-02	3,57E-01	Lgr6	0,25	No
ENSMUSG00000042797	-1,87E-01	-0,929353705	1,88E-01	6,65E-01	8,84E-01	Aqp11	0,88	No
ENSMUSG00000042800	-7,11E-03	-1,070157782	2,42E-04	9,88E-01	1,00E+00	Spata46	1,00	No
ENSMUSG00000042801	-1,94E+00	-3,107619243	1,50E+00	2,21E-01	5,50E-01	Olfr769	0,26	No
ENSMUSG00000042804	6,44E-02	1,027675671	9,94E-02	7,53E-01	9,33E-01	Gpr153	1,05	No
ENSMUSG00000042807	2,41E-02	-0,205773156	5,55E-03	9,41E-01	1,00E+00	Hecw2	1,02	No

ENSMUSG00000042810	-7,93E-01	2,267788698	3,48E+01	3,59E-09	2,69E-07	Krba1	0,58	Yes
ENSMUSG00000042812	-5,82E-02	-2,711732971	2,30E-03	9,62E-01	1,00E+00	Foxf1	0,96	No
ENSMUSG00000042814	2,27E-01	2,099313042	2,36E+00	1,25E-01	4,42E-01	Mcts2	1,17	No
ENSMUSG00000042816	5,43E-01	-1,627780184	8,40E-01	3,59E-01	6,61E-01	Gpr151	1,46	No
ENSMUSG00000042817	-3,82E-02	3,615063149	1,53E-01	6,95E-01	9,02E-01	Flt3	0,97	No
ENSMUSG00000042821	-8,42E-02	-1,424784396	2,99E-02	8,63E-01	9,97E-01	Snai1	0,94	No
ENSMUSG00000042826	-1,61E-01	0,957853601	5,70E-01	4,50E-01	7,40E-01	Fgf11	0,89	No
ENSMUSG00000042828	4,11E-01	-1,06089515	7,38E-01	3,90E-01	6,90E-01	Trim72	1,33	No
ENSMUSG00000042831	-2,41E-01	3,973727079	7,34E+00	6,76E-03	7,00E-02	Alkbh6	0,85	No
ENSMUSG00000042834	2,13E-02	4,750218369	7,83E-02	7,80E-01	9,48E-01	Nrep	1,01	No
ENSMUSG00000042842	-1,02E+00	5,649924869	2,27E+02	2,84E-51	6,56E-48	Serpinb6b	0,49	Yes
ENSMUSG00000042851	7,92E-02	1,744458118	2,56E-01	6,13E-01	8,53E-01	Zc3h6	1,06	No
ENSMUSG00000042854	6,59E-02	2,02721226	2,02E-01	6,53E-01	8,78E-01	Trp53rkb	1,05	No
ENSMUSG00000042857	2,01E-01	2,731282631	2,64E+00	1,04E-01	3,97E-01	Gm9776	1,15	No
ENSMUSG00000042870	2,13E-01	3,806877811	5,32E+00	2,11E-02	1,56E-01	Tom1	1,16	No
ENSMUSG00000042873	-8,95E-02	-1,627581278	2,81E-02	8,67E-01	9,99E-01	Lhfpl4	0,94	No
ENSMUSG00000042874	-4,35E-02	1,854099034	7,65E-02	7,82E-01	9,50E-01	D930007J09Rik	0,97	No
ENSMUSG00000042895	4,90E-01	-2,711685798	1,88E-01	6,65E-01	8,84E-01	Abra	1,40	No
ENSMUSG00000042901	1,23E-02	5,640663999	3,25E-02	8,57E-01	9,94E-01	Aida	1,01	No
ENSMUSG00000042909	8,30E-01	-2,497611906	8,27E-01	3,63E-01	6,64E-01	Olfr648	1,78	No
ENSMUSG00000042918	-1,03E+00	0,251921896	1,60E+01	6,48E-05	1,69E-03	Mamstr	0,49	Yes
ENSMUSG00000042938	-1,20E-01	2,847279981	1,07E+00	3,00E-01	5,96E-01	Gm14117	0,92	No
ENSMUSG00000042942	-6,81E-01	0,045512525	5,93E+00	1,49E-02	1,25E-01	Greb1l	0,62	No
ENSMUSG00000042961	-1,89E+00	-3,1076441	1,42E+00	2,33E-01	5,50E-01	Egflam	0,27	No
ENSMUSG00000042962	1,44E-02	6,64370736	5,43E-02	8,16E-01	9,69E-01	Gm5436	1,01	No
ENSMUSG00000042978	2,99E-01	4,901611615	1,55E+01	8,47E-05	2,11E-03	Sbk1	1,23	Yes
ENSMUSG00000042985	-8,45E-01	-2,963229433	3,92E-01	5,31E-01	8,05E-01	Upk3b	0,56	No
ENSMUSG00000042992	1,32E-01	3,794680788	2,03E+00	1,54E-01	4,97E-01	Borcs5	1,10	No
ENSMUSG00000042993	2,71E-01	-0,980528771	3,67E-01	5,45E-01	8,13E-01	Ifnk	1,21	No
ENSMUSG00000042997	1,43E-01	6,006961185	4,78E+00	2,87E-02	1,93E-01	Nhlrc3	1,10	No
ENSMUSG00000043004	1,47E-01	5,984152718	5,00E+00	2,54E-02	1,77E-01	Gng2	1,11	No
ENSMUSG00000043008	1,31E-01	5,363271339	3,46E+00	6,31E-02	3,13E-01	Klhl6	1,09	No

ENSMUSG00000043013	2,06E-02	-3,146103739	-9,26E-10	1,00E+00	1,00E+00	Onecut1	1,01	No
ENSMUSG00000043015	2,54E-01	3,532697579	6,90E+00	8,64E-03	8,38E-02	Nemp2	1,19	No
ENSMUSG00000043017	3,40E-01	6,743068357	3,05E+01	3,35E-08	2,05E-06	Ptgir	1,27	Yes
ENSMUSG00000043019	2,05E-01	5,526962256	8,83E+00	2,97E-03	3,81E-02	Edem3	1,15	Yes
ENSMUSG00000043020	-1,95E-01	-1,54817401	1,19E-01	7,30E-01	9,21E-01	Wdr63	0,87	No
ENSMUSG00000043024	-1,72E-01	5,315449441	5,96E+00	1,47E-02	1,24E-01	Gm16433	0,89	No
ENSMUSG00000043036	1,47E-01	-1,185552034	1,03E-01	7,48E-01	9,30E-01	Ccdc63	1,11	No
ENSMUSG00000043050	-2,71E-01	-1,49759944	2,57E-01	6,12E-01	8,52E-01	Tnp2	0,83	No
ENSMUSG00000043051	2,63E-01	2,592261112	4,06E+00	4,40E-02	2,50E-01	Disc1	1,20	No
ENSMUSG00000043059	-7,78E-02	5,128797144	1,16E+00	2,81E-01	5,78E-01	Zfp513	0,95	No
ENSMUSG00000043061	-2,94E-02	4,696352391	1,46E-01	7,02E-01	9,05E-01	Tmem18	0,98	No
ENSMUSG00000043065	1,82E-01	2,892837646	2,57E+00	1,09E-01	4,04E-01	Spice1	1,13	No
ENSMUSG00000043067	1,91E-01	5,647673307	7,84E+00	5,11E-03	5,67E-02	Dpy19l1	1,14	No
ENSMUSG00000043068	-6,48E-01	1,18385193	1,16E+01	6,59E-04	1,15E-02	Fam89a	0,64	Yes
ENSMUSG00000043073	-1,94E+00	-3,107619243	1,50E+00	2,21E-01	5,50E-01	Usp17le	0,26	No
ENSMUSG00000043079	2,48E-01	6,792855641	1,61E+01	5,97E-05	1,58E-03	Synpo	1,19	Yes
ENSMUSG00000043085	-1,77E-01	2,578251813	1,88E+00	1,70E-01	5,26E-01	Tmem82	0,88	No
ENSMUSG00000043088	-3,83E-01	-0,863816023	9,89E-01	3,20E-01	6,21E-01	Il17re	0,77	No
ENSMUSG00000043090	-1,79E-01	3,082256928	2,74E+00	9,80E-02	3,91E-01	Zfp866	0,88	No
ENSMUSG00000043091	3,22E-02	8,261742175	3,11E-01	5,77E-01	8,35E-01	Tuba1c	1,02	No
ENSMUSG00000043099	-2,73E+00	1,805657321	2,29E+02	1,02E-51	2,77E-48	Hic1	0,15	Yes
ENSMUSG00000043102	-6,96E-01	-2,033666197	1,10E+00	2,93E-01	5,89E-01	Qrfp	0,62	No
ENSMUSG00000043110	-1,98E-02	-3,033655952	2,83E-11	1,00E+00	1,00E+00	Lrrn4	0,99	No
ENSMUSG00000043126	1,86E-02	-1,31097756	1,50E-03	9,69E-01	1,00E+00	D830039M14Rik	1,01	No
ENSMUSG00000043131	-4,06E-02	8,939508458	5,09E-01	4,76E-01	7,64E-01	Mob1a	0,97	No
ENSMUSG00000043140	4,52E-01	3,189586361	1,83E+01	1,91E-05	5,96E-04	Tmem186	1,37	Yes
ENSMUSG00000043145	1,36E+00	-2,497639521	1,90E+00	1,68E-01	5,23E-01	Gm11292	2,57	No
ENSMUSG00000043153	6,33E-02	1,144356746	9,89E-02	7,53E-01	9,33E-01	Ispd	1,04	No
ENSMUSG00000043154	-2,28E-01	3,639493475	5,56E+00	1,83E-02	1,43E-01	Ppp2r3a	0,85	No
ENSMUSG00000043155	8,13E-01	-0,86379372	3,31E+00	6,87E-02	3,30E-01	Hpdl	1,76	No
ENSMUSG00000043157	2,75E-01	5,89459642	1,72E+01	3,38E-05	9,70E-04	Arl11	1,21	Yes
ENSMUSG00000043162	-3,49E-01	3,464215974	1,24E+01	4,31E-04	8,10E-03	Pyurf	0,78	Yes

ENSMUSG00000043168	-6,49E-01	-2,522684812	4,01E-01	5,27E-01	8,03E-01	4930426D05Rik	0,64	No
ENSMUSG00000043183	1,92E-01	3,573853848	3,99E+00	4,58E-02	2,55E-01	Simc1	1,14	No
ENSMUSG00000043190	9,18E-02	2,985304613	6,55E-01	4,18E-01	7,13E-01	Rfesd	1,07	No
ENSMUSG00000043192	9,90E-02	8,937723717	3,02E+00	8,24E-02	3,69E-01	Gm1840	1,07	No
ENSMUSG00000043207	8,05E-02	6,134538686	1,55E+00	2,13E-01	5,50E-01	Zmpste24	1,06	No
ENSMUSG00000043219	-9,86E-01	-2,655074424	8,52E-01	3,56E-01	6,57E-01	Hoxa6	0,50	No
ENSMUSG00000043223	1,43E-01	1,422446279	6,36E-01	4,25E-01	7,20E-01	Gm4835	1,10	No
ENSMUSG00000043230	9,01E-03	-3,107646452	-5,16E-10	1,00E+00	1,00E+00	Fam124b	1,01	No
ENSMUSG00000043241	-1,44E-01	5,548663788	4,47E+00	3,45E-02	2,19E-01	Upf2	0,90	No
ENSMUSG00000043243	-6,07E-01	1,639099587	1,39E+01	1,93E-04	4,23E-03	Fam129c	0,66	Yes
ENSMUSG00000043251	3,06E-01	-0,247084243	8,96E-01	3,44E-01	6,44E-01	Exoc3l	1,24	No
ENSMUSG00000043252	1,26E-01	5,36924351	3,22E+00	7,29E-02	3,43E-01	Tmem64	1,09	No
ENSMUSG00000043257	-1,16E-01	3,095470239	1,16E+00	2,81E-01	5,78E-01	Pigv	0,92	No
ENSMUSG00000043262	6,94E-02	4,400257822	7,25E-01	3,94E-01	6,95E-01	Uevld	1,05	No
ENSMUSG00000043263	-2,07E-01	5,549912924	9,17E+00	2,46E-03	3,29E-02	Ifi209	0,87	Yes
ENSMUSG00000043279	-5,04E-03	5,558554178	5,44E-03	9,41E-01	1,00E+00	Trim56	1,00	No
ENSMUSG00000043282	2,57E+00	-3,0336723	2,56E+00	1,10E-01	4,06E-01	Teddm1b	5,96	No
ENSMUSG00000043284	-5,39E-02	4,55138775	4,56E-01	4,99E-01	7,85E-01	Tmem11	0,96	No
ENSMUSG00000043286	-1,95E+00	-3,033628749	1,50E+00	2,21E-01	5,50E-01	Pnpla1	0,26	No
ENSMUSG00000043290	1,52E-01	0,913495964	5,05E-01	4,77E-01	7,65E-01	Zfp784	1,11	No
ENSMUSG00000043298	1,25E-01	0,979985893	3,63E-01	5,47E-01	8,14E-01	Smco3	1,09	No
ENSMUSG00000043300	-2,42E-01	2,213180026	3,10E+00	7,83E-02	3,58E-01	B3galnt1	0,85	No
ENSMUSG00000043311	-9,70E-02	3,978796777	1,20E+00	2,74E-01	5,70E-01	D17H6S53E	0,93	No
ENSMUSG00000043323	-1,73E-01	6,509784349	7,60E+00	5,84E-03	6,28E-02	Fbrsl1	0,89	No
ENSMUSG00000043329	4,31E-01	0,677847431	3,42E+00	6,43E-02	3,17E-01	Gm8849	1,35	No
ENSMUSG00000043333	-4,01E-02	-2,711726283	1,12E-03	9,73E-01	1,00E+00	Rhbdl2	0,97	No
ENSMUSG00000043336	-3,55E-01	6,232561544	3,06E+01	3,14E-08	1,95E-06	Filip1l	0,78	Yes
ENSMUSG00000043340	-3,72E-01	1,777115962	5,28E+00	2,15E-02	1,58E-01	6530409C15Rik	0,77	No
ENSMUSG00000043346	-1,29E-02	1,697081622	6,12E-03	9,38E-01	1,00E+00	Gm6741	0,99	No
ENSMUSG00000043372	-1,39E-01	1,538149092	6,36E-01	4,25E-01	7,19E-01	Hexim2	0,91	No
ENSMUSG00000043385	-8,91E-01	-2,896142986	4,37E-01	5,09E-01	7,92E-01	Olfr267	0,54	No
ENSMUSG00000043391	-1,30E-01	5,651875793	3,71E+00	5,41E-02	2,85E-01	2510009E07Rik	0,91	No

ENSMUSG00000043398	1,74E+00	-1,896197085	6,16E+00	1,31E-02	1,14E-01	Gpr135	3,34	No
ENSMUSG00000043410	8,82E-01	-1,033570247	3,96E+00	4,65E-02	2,57E-01	Hfm1	1,84	No
ENSMUSG00000043411	7,04E-02	6,804410258	1,32E+00	2,51E-01	5,50E-01	Usp48	1,05	No
ENSMUSG00000043415	2,89E-01	2,762167418	5,72E+00	1,68E-02	1,35E-01	Otud1	1,22	No
ENSMUSG00000043418	1,87E+00	-3,107639691	1,38E+00	2,40E-01	5,50E-01	Lrit2	3,67	No
ENSMUSG00000043419	2,59E-01	1,010368171	1,43E+00	2,32E-01	5,50E-01	Chd3os	1,20	No
ENSMUSG00000043421	-2,10E-01	3,599567951	4,62E+00	3,15E-02	2,06E-01	Hilpda	0,86	No
ENSMUSG00000043424	4,54E-02	5,596445669	4,40E-01	5,07E-01	7,91E-01	Eif3j2	1,03	No
ENSMUSG00000043429	9,01E-03	-3,107646452	-5,16E-10	1,00E+00	1,00E+00	Ccdc185	1,01	No
ENSMUSG00000043432	9,55E-02	3,54514777	9,50E-01	3,30E-01	6,30E-01	Leng9	1,07	No
ENSMUSG00000043439	-1,57E-01	5,082288316	4,56E+00	3,28E-02	2,12E-01	Epop	0,90	No
ENSMUSG00000043441	-7,38E-02	-3,033659033	2,66E-03	9,59E-01	1,00E+00	Gpr149	0,95	No
ENSMUSG00000043445	2,24E-01	5,331827278	9,94E+00	1,62E-03	2,37E-02	Pgp	1,17	Yes
ENSMUSG00000043448	2,74E-02	-3,033621357	9,16E-12	1,00E+00	1,00E+00	Gjc2	1,02	No
ENSMUSG00000043456	-4,87E-02	-2,896122191	1,09E-03	9,74E-01	1,00E+00	Zfp536	0,97	No
ENSMUSG00000043460	3,98E-01	-2,311203016	2,54E-01	6,14E-01	8,53E-01	Elfn2	1,32	No
ENSMUSG00000043461	3,14E-02	-3,107617333	-7,69E-10	1,00E+00	1,00E+00	Sptssb	1,02	No
ENSMUSG00000043467	1,38E-02	4,757176204	3,29E-02	8,56E-01	9,93E-01	Zbtb37	1,01	No
ENSMUSG00000043468	-6,97E-01	-1,963243885	1,11E+00	2,92E-01	5,88E-01	Adam30	0,62	No
ENSMUSG00000043483	-1,33E-01	8,151498809	5,29E+00	2,15E-02	1,58E-01	Gm6863	0,91	No
ENSMUSG00000043484	1,03E-01	5,137706988	2,00E+00	1,57E-01	5,03E-01	Gm5867	1,07	No
ENSMUSG00000043487	2,01E-01	2,591086436	2,72E+00	9,92E-02	3,92E-01	Acot6	1,15	No
ENSMUSG00000043488	4,06E-02	1,684575947	6,28E-02	8,02E-01	9,61E-01	Frmd8os	1,03	No
ENSMUSG00000043496	5,95E-01	-3,070171822	1,40E-01	7,08E-01	9,09E-01	Tril	1,51	No
ENSMUSG00000043501	-1,87E+00	-3,107637761	1,39E+00	2,38E-01	5,50E-01	Lgals2	0,27	No
ENSMUSG00000043505	-2,66E-01	2,415217135	4,18E+00	4,10E-02	2,40E-01	Gimap5	0,83	No
ENSMUSG00000043510	-9,60E-03	2,628119054	6,08E-03	9,38E-01	1,00E+00	Hscb	0,99	No
ENSMUSG00000043529	3,15E+00	-2,963263135	4,04E+00	4,45E-02	2,51E-01	Olfr266	8,85	No
ENSMUSG00000043531	-3,49E-02	-2,497607097	1,38E-03	9,70E-01	1,00E+00	Sorcs1	0,98	No
ENSMUSG00000043535	-1,81E-02	6,945712099	8,85E-02	7,66E-01	9,40E-01	Setx	0,99	No
ENSMUSG00000043541	9,35E-02	1,488315355	2,80E-01	5,97E-01	8,45E-01	Casc1	1,07	No
ENSMUSG00000043542	-1,78E-01	3,236172378	2,84E+00	9,19E-02	3,82E-01	Zc2hc1a	0,88	No

ENSMUSG00000043556	9,01E-03	-3,107646452	-5,16E-10	1,00E+00	1,00E+00	Fbxl7	1,01	No
ENSMUSG00000043557	4,25E-01	2,586248187	1,08E+01	1,01E-03	1,62E-02	Mdga1	1,34	Yes
ENSMUSG00000043572	-1,09E-01	2,16436348	5,90E-01	4,42E-01	7,34E-01	Pars2	0,93	No
ENSMUSG00000043587	2,03E-01	3,432686529	3,94E+00	4,72E-02	2,60E-01	Pxylp1	1,15	No
ENSMUSG00000043592	-1,89E+00	-3,1076441	1,42E+00	2,33E-01	5,50E-01	Unc5cl	0,27	No
ENSMUSG00000043602	4,72E-01	1,447996355	7,50E+00	6,18E-03	6,56E-02	Zfp3	1,39	No
ENSMUSG00000043613	-1,39E+00	-2,896103981	1,22E+00	2,70E-01	5,66E-01	Mmp3	0,38	No
ENSMUSG00000043614	-7,38E-01	-1,600636553	1,43E+00	2,32E-01	5,50E-01	Vps37d	0,60	No
ENSMUSG00000043618	1,72E-02	6,443487654	7,45E-02	7,85E-01	9,51E-01	Eif5al3-ps	1,01	No
ENSMUSG00000043623	-1,76E-01	0,198839056	3,98E-01	5,28E-01	8,04E-01	4933412A08Rik	0,89	No
ENSMUSG00000043629	2,08E+00	-2,355650596	5,53E+00	1,87E-02	1,45E-01	1700019D03Rik	4,24	No
ENSMUSG00000043631	8,33E-02	-1,289574716	2,91E-02	8,65E-01	9,98E-01	Ecm2	1,06	No
ENSMUSG00000043633	-8,89E-02	7,344711779	2,22E+00	1,36E-01	4,65E-01	Fam221b	0,94	No
ENSMUSG00000043635	7,02E-01	-2,311199986	7,16E-01	3,98E-01	6,98E-01	Adamts3	1,63	No
ENSMUSG00000043639	6,14E-01	-0,719335109	2,55E+00	1,10E-01	4,07E-01	Rbm20	1,53	No
ENSMUSG00000043644	-5,20E-01	-0,262778913	2,63E+00	1,05E-01	3,97E-01	0610009L18Rik	0,70	No
ENSMUSG00000043648	3,11E+00	-2,963280805	3,94E+00	4,72E-02	2,60E-01	Pld6	8,63	No
ENSMUSG00000043659	-1,92E+00	-3,033623267	1,46E+00	2,27E-01	5,50E-01	Npsr1	0,26	No
ENSMUSG00000043664	3,49E-01	1,402512839	3,80E+00	5,13E-02	2,75E-01	Tmem221	1,27	No
ENSMUSG00000043668	-8,68E-01	0,804658751	1,64E+01	5,09E-05	1,38E-03	Tox3	0,55	Yes
ENSMUSG00000043670	1,81E+00	-3,10765737	1,28E+00	2,58E-01	5,50E-01	Diras1	3,49	No
ENSMUSG00000043671	-2,01E-01	3,024758491	3,39E+00	6,55E-02	3,21E-01	Dpy19l3	0,87	No
ENSMUSG00000043681	1,81E+00	-3,10765737	1,28E+00	2,58E-01	5,50E-01	Fam25c	3,49	No
ENSMUSG00000043683	8,04E-02	6,044499725	1,53E+00	2,17E-01	5,50E-01	Fem1a	1,06	No
ENSMUSG00000043687	-5,96E-01	-1,2262243	1,53E+00	2,15E-01	5,50E-01	1190005I06Rik	0,66	No
ENSMUSG00000043702	-9,10E-02	5,214855385	1,60E+00	2,05E-01	5,50E-01	Pde12	0,94	No
ENSMUSG00000043705	-1,31E-01	-1,088850072	9,79E-02	7,54E-01	9,34E-01	Capn13	0,91	No
ENSMUSG00000043716	-2,51E-01	6,897270827	1,69E+01	3,84E-05	1,08E-03	Rpl7	0,84	Yes
ENSMUSG00000043727	-1,32E-01	-1,126721625	8,71E-02	7,68E-01	9,41E-01	F830045P16Rik	0,91	No
ENSMUSG00000043733	3,64E-02	7,543253654	3,79E-01	5,38E-01	8,09E-01	Ptpn11	1,03	No
ENSMUSG00000043740	-3,35E-01	6,038800383	2,65E+01	2,66E-07	1,35E-05	B430306N03Rik	0,79	Yes
ENSMUSG00000043747	-2,47E+00	-2,497560991	5,29E+00	2,14E-02	1,58E-01	1520401A03Rik	0,18	No

ENSMUSG00000043753	1,87E+00	-3,107639691	1,38E+00	2,40E-01	5,50E-01	Dmrta1	3,67	No
ENSMUSG00000043760	-1,94E+00	-3,107619243	1,50E+00	2,21E-01	5,50E-01	Pkhd1	0,26	No
ENSMUSG00000043770	-6,91E-02	7,251886527	1,33E+00	2,49E-01	5,50E-01	Gm12481	0,95	No
ENSMUSG00000043773	-2,65E-01	-0,863652439	4,38E-01	5,08E-01	7,92E-01	1700048O20Rik	0,83	No
ENSMUSG00000043782	-8,43E-01	-0,912479762	4,09E+00	4,30E-02	2,47E-01	Bicdl2	0,56	No
ENSMUSG00000043789	1,41E+00	-2,268160015	2,83E+00	9,26E-02	3,83E-01	Vwce	2,65	No
ENSMUSG00000043794	-2,01E-01	-1,436665875	1,68E-01	6,81E-01	8,94E-01	D830025C05Rik	0,87	No
ENSMUSG00000043795	-5,87E-02	3,89720995	4,44E-01	5,05E-01	7,90E-01	Prr33	0,96	No
ENSMUSG00000043800	2,78E-01	1,477163243	2,33E+00	1,27E-01	4,46E-01	Kifc5c-ps	1,21	No
ENSMUSG00000043801	-2,97E-02	8,381512502	2,67E-01	6,06E-01	8,49E-01	Oaz1-ps	0,98	No
ENSMUSG00000043807	-3,52E-02	-0,378374656	9,96E-03	9,21E-01	1,00E+00	Ly6g5b	0,98	No
ENSMUSG00000043811	1,60E-01	2,107898506	1,29E+00	2,56E-01	5,50E-01	Rtn4r	1,12	No
ENSMUSG00000043822	3,06E-01	5,693545589	2,05E+01	6,06E-06	2,14E-04	Adamtsl5	1,24	Yes
ENSMUSG00000043831	2,04E-02	4,007393159	5,25E-02	8,19E-01	9,71E-01	Lysmd4	1,01	No
ENSMUSG00000043832	3,80E-01	5,307131062	2,82E+01	1,09E-07	6,03E-06	Clec4a3	1,30	Yes
ENSMUSG00000043833	6,27E-02	0,483849173	6,80E-02	7,94E-01	9,56E-01	2900005J15Rik	1,04	No
ENSMUSG00000043843	1,87E+00	-3,107639691	1,38E+00	2,40E-01	5,50E-01	Tmem145	3,67	No
ENSMUSG00000043850	1,85E+00	-3,107657832	1,35E+00	2,46E-01	5,50E-01	Clrn1	3,61	No
ENSMUSG00000043857	-3,83E-02	-3,107651936	-7,91E-11	1,00E+00	1,00E+00	Mgat5b	0,97	No
ENSMUSG00000043866	-3,93E-02	6,191475345	3,75E-01	5,40E-01	8,10E-01	Taf10	0,97	No
ENSMUSG00000043870	2,44E-01	3,250359116	5,32E+00	2,11E-02	1,57E-01	Gm5809	1,18	No
ENSMUSG00000043872	2,97E-02	2,804642648	6,40E-02	8,00E-01	9,61E-01	Zmym1	1,02	No
ENSMUSG00000043873	3,28E-01	-1,980550698	2,42E-01	6,23E-01	8,58E-01	Chil5	1,26	No
ENSMUSG00000043881	3,24E-02	3,874713149	1,22E-01	7,27E-01	9,19E-01	Kbtbd7	1,02	No
ENSMUSG00000043885	1,44E-01	7,201805912	5,69E+00	1,71E-02	1,36E-01	Slc36a4	1,10	No
ENSMUSG00000043889	-5,68E-02	9,20125409	1,00E+00	3,16E-01	6,17E-01	Gm8399	0,96	No
ENSMUSG00000043895	5,57E-01	5,197763372	6,08E+01	6,24E-15	9,63E-13	S1pr2	1,47	Yes
ENSMUSG00000043903	6,26E-01	-1,574278862	1,15E+00	2,83E-01	5,80E-01	Zfp469	1,54	No
ENSMUSG00000043909	-7,81E-02	5,482083405	1,28E+00	2,58E-01	5,50E-01	Trp53bp1	0,95	No
ENSMUSG00000043923	-1,64E-01	3,599187963	2,93E+00	8,70E-02	3,78E-01	Ccdc84	0,89	No
ENSMUSG00000043924	-7,33E-01	-1,863607959	1,40E+00	2,36E-01	5,50E-01	Ncmap	0,60	No
ENSMUSG00000043925	-7,66E-01	-1,895977815	1,20E+00	2,74E-01	5,71E-01	Olfr544	0,59	No

ENSMUSG00000043931	-1,86E+00	-3,033612668	1,39E+00	2,38E-01	5,50E-01	Gimap7	0,28	No
ENSMUSG00000043932	-9,94E-02	-0,195567896	1,02E-01	7,50E-01	9,31E-01	Klri2	0,93	No
ENSMUSG00000043939	-1,78E-01	3,81603519	3,73E+00	5,35E-02	2,82E-01	A530064D06Rik	0,88	No
ENSMUSG00000043940	1,31E-01	7,147555274	4,67E+00	3,07E-02	2,02E-01	Wdfy3	1,09	No
ENSMUSG00000043943	-3,43E-01	-1,268079923	4,79E-01	4,89E-01	7,76E-01	Naalad2	0,79	No
ENSMUSG00000043953	1,30E-03	4,271015834	2,51E-04	9,87E-01	1,00E+00	Ccrl2	1,00	No
ENSMUSG00000043962	-3,29E-02	7,586509866	3,10E-01	5,78E-01	8,35E-01	Thrap3	0,98	No
ENSMUSG00000043964	9,27E-02	5,646321831	1,88E+00	1,71E-01	5,26E-01	Orai3	1,07	No
ENSMUSG00000043969	1,87E+00	-3,107639691	1,38E+00	2,40E-01	5,50E-01	Emx2	3,67	No
ENSMUSG00000043986	-3,29E-01	-2,800957559	7,91E-02	7,79E-01	9,47E-01	Spata31d1d	0,80	No
ENSMUSG00000043987	5,10E-02	4,755623469	4,42E-01	5,06E-01	7,91E-01	Cep164	1,04	No
ENSMUSG00000043991	1,83E-01	4,53893966	5,24E+00	2,20E-02	1,61E-01	Pura	1,13	No
ENSMUSG00000043993	-3,30E-02	0,873319913	2,22E-02	8,81E-01	1,00E+00	2900052L18Rik	0,98	No
ENSMUSG00000043998	1,06E-01	6,768833364	2,97E+00	8,48E-02	3,74E-01	Mgat2	1,08	No
ENSMUSG00000043999	-3,51E-01	-2,355581388	1,56E-01	6,93E-01	9,01E-01	Gpr75	0,78	No
ENSMUSG00000044005	-9,11E-01	0,763718725	1,79E+01	2,36E-05	7,18E-04	Gls2	0,53	Yes
ENSMUSG00000044006	1,77E-01	-2,355561141	4,70E-02	8,28E-01	9,76E-01	Cilp2	1,13	No
ENSMUSG00000044014	-2,68E+00	-3,033645766	2,84E+00	9,17E-02	3,82E-01	Npy5r	0,16	No
ENSMUSG00000044017	7,43E-01	-2,963261206	3,01E-01	5,83E-01	8,38E-01	Adgrd1	1,67	No
ENSMUSG00000044018	8,69E-02	4,551254235	1,18E+00	2,77E-01	5,74E-01	Mrpl50	1,06	No
ENSMUSG00000044021	1,06E+00	-2,448773659	1,44E+00	2,31E-01	5,50E-01	Muc19	2,08	No
ENSMUSG00000044022	1,87E+00	-3,107639691	1,38E+00	2,40E-01	5,50E-01	Pcdhb21	3,67	No
ENSMUSG00000044024	-7,04E-02	2,041893025	2,31E-01	6,30E-01	8,63E-01	Rell2	0,95	No
ENSMUSG00000044026	1,60E-01	4,112259064	3,42E+00	6,44E-02	3,18E-01	Slc35g1	1,12	No
ENSMUSG00000044029	-8,80E-02	-2,896116238	3,33E-03	9,54E-01	1,00E+00	Olfr178	0,94	No
ENSMUSG00000044030	1,48E-01	4,226753717	3,14E+00	7,64E-02	3,52E-01	Irf2bp1	1,11	No
ENSMUSG00000044033	-1,31E-01	-0,997958763	7,79E-02	7,80E-01	9,48E-01	Ccdc141	0,91	No
ENSMUSG00000044034	2,46E-01	-1,378369881	2,04E-01	6,52E-01	8,77E-01	Npb	1,19	No
ENSMUSG00000044037	1,67E-02	5,438477159	5,74E-02	8,11E-01	9,66E-01	Als2cl	1,01	No
ENSMUSG00000044042	-3,09E-02	4,816950312	1,64E-01	6,86E-01	8,97E-01	Fmn1	0,98	No
ENSMUSG00000044043	-1,87E+00	-3,107637761	1,39E+00	2,38E-01	5,50E-01	Pcdhb14	0,27	No
ENSMUSG00000044052	3,06E-01	-0,627581621	5,96E-01	4,40E-01	7,32E-01	Ccr10	1,24	No

ENSMUSG00000044055	-2,03E-01	-1,069978359	2,12E-01	6,45E-01	8,73E-01	Otos	0,87	No
ENSMUSG00000044056	2,16E-01	-1,740771754	1,19E-01	7,30E-01	9,21E-01	Efcab9	1,16	No
ENSMUSG00000044058	4,49E-01	0,344880935	3,07E+00	7,95E-02	3,61E-01	Mthfr-ps1	1,37	No
ENSMUSG00000044065	-8,25E-02	-2,54815579	7,85E-03	9,29E-01	1,00E+00	Gm9788	0,94	No
ENSMUSG00000044066	-3,28E-02	4,444693729	1,66E-01	6,84E-01	8,96E-01	Cep68	0,98	No
ENSMUSG00000044067	-7,04E-01	-2,54819638	5,55E-01	4,56E-01	7,46E-01	Gpr22	0,61	No
ENSMUSG00000044068	-3,12E-02	3,345201369	9,39E-02	7,59E-01	9,36E-01	Zrsr1	0,98	No
ENSMUSG00000044072	3,22E-01	4,451625282	1,57E+01	7,50E-05	1,90E-03	Eml6	1,25	Yes
ENSMUSG00000044080	-6,46E-02	6,724458121	1,09E+00	2,97E-01	5,92E-01	S100a1	0,96	No
ENSMUSG00000044081	2,25E-01	-0,73357547	3,52E-01	5,53E-01	8,19E-01	Zfp85os	1,17	No
ENSMUSG00000044083	1,58E-01	-1,548092148	8,13E-02	7,76E-01	9,45E-01	Efcab8	1,12	No
ENSMUSG00000044084	3,14E-02	-3,107617333	-7,69E-10	1,00E+00	1,00E+00	4933402P03Rik	1,02	No
ENSMUSG00000044086	1,24E+00	-0,912733021	8,24E+00	4,10E-03	4,82E-02	Lmod3	2,36	Yes
ENSMUSG00000044092	1,98E-02	4,832987983	6,73E-02	7,95E-01	9,57E-01	C130050O18Rik	1,01	No
ENSMUSG00000044098	3,49E-02	4,550889085	1,98E-01	6,57E-01	8,80E-01	Rsbn1	1,02	No
ENSMUSG00000044103	1,80E-01	3,157984363	2,53E+00	1,12E-01	4,11E-01	Il1f9	1,13	No
ENSMUSG00000044117	-1,89E+00	-3,1076441	1,42E+00	2,33E-01	5,50E-01	2900011O08Rik	0,27	No
ENSMUSG00000044122	-4,36E-01	-0,299894374	1,60E+00	2,06E-01	5,50E-01	Proca1	0,74	No
ENSMUSG00000044125	1,18E-01	-1,412991607	5,24E-02	8,19E-01	9,71E-01	9530080O11Rik	1,09	No
ENSMUSG00000044134	7,85E-02	3,251542906	5,67E-01	4,51E-01	7,41E-01	Fam109a	1,06	No
ENSMUSG00000044139	2,45E-01	0,767456732	1,20E+00	2,74E-01	5,70E-01	Prss53	1,18	No
ENSMUSG00000044141	9,90E-02	3,460239211	9,92E-01	3,19E-01	6,20E-01	E130201H02Rik	1,07	No
ENSMUSG00000044145	-1,04E-02	-0,088870509	1,10E-03	9,74E-01	1,00E+00	1810024B03Rik	0,99	No
ENSMUSG00000044147	1,34E-01	7,657502662	5,14E+00	2,34E-02	1,68E-01	Arf6	1,10	No
ENSMUSG00000044155	2,37E-02	4,38926483	8,40E-02	7,72E-01	9,43E-01	Lsm8	1,02	No
ENSMUSG00000044156	-1,09E-01	6,012545415	2,83E+00	9,27E-02	3,83E-01	Hepacam2	0,93	No
ENSMUSG00000044162	-2,21E-01	6,325003386	1,22E+01	4,84E-04	8,93E-03	Tnip3	0,86	Yes
ENSMUSG00000044164	1,81E+00	-3,10765737	1,28E+00	2,58E-01	5,50E-01	Rnf182	3,49	No
ENSMUSG00000044165	1,07E+00	-2,424884699	1,29E+00	2,56E-01	5,50E-01	Bcl2l15	2,11	No
ENSMUSG00000044167	5,56E-03	4,393964402	4,72E-03	9,45E-01	1,00E+00	Foxo1	1,00	No
ENSMUSG00000044176	1,89E+00	-3,033614598	1,38E+00	2,40E-01	5,50E-01	Spink10	3,70	No
ENSMUSG00000044177	-7,08E-01	-2,18558236	8,53E-01	3,56E-01	6,57E-01	Wfikkn2	0,61	No

ENSMUSG00000044186	-1,87E+00	-3,107637761	1,39E+00	2,38E-01	5,50E-01	Nkx2-6	0,27	No
ENSMUSG00000044197	1,28E-01	6,433655222	4,11E+00	4,25E-02	2,45E-01	Gpr146	1,09	No
ENSMUSG00000044199	1,54E-01	3,560249132	2,50E+00	1,14E-01	4,17E-01	S1pr4	1,11	No
ENSMUSG00000044201	-2,55E-02	2,704213251	4,42E-02	8,33E-01	9,79E-01	Cdc25c	0,98	No
ENSMUSG00000044211	-3,08E-02	4,730386461	1,58E-01	6,91E-01	9,00E-01	Gm7887	0,98	No
ENSMUSG00000044216	1,24E+00	-2,770605879	9,57E-01	3,28E-01	6,29E-01	Kcnj4	2,37	No
ENSMUSG00000044217	-6,07E-02	-2,77056901	2,49E-03	9,60E-01	1,00E+00	Aqp5	0,96	No
ENSMUSG00000044220	1,83E+00	-2,963282593	1,33E+00	2,50E-01	5,50E-01	Nkx2-3	3,56	No
ENSMUSG00000044221	4,26E-02	5,852043911	4,14E-01	5,20E-01	7,99E-01	Grsf1	1,03	No
ENSMUSG00000044224	-2,58E-02	4,401262408	9,86E-02	7,54E-01	9,33E-01	Dnajc21	0,98	No
ENSMUSG00000044229	-5,48E-01	0,152302457	4,13E+00	4,22E-02	2,45E-01	Nxpe4	0,68	No
ENSMUSG00000044231	2,31E-01	1,635880844	1,82E+00	1,77E-01	5,37E-01	Nhlrc1	1,17	No
ENSMUSG00000044243	-2,69E+00	-2,832001224	2,88E+00	8,99E-02	3,80E-01	Bhlha9	0,15	No
ENSMUSG00000044244	9,21E-02	3,432205519	8,15E-01	3,67E-01	6,68E-01	Il20rb	1,07	No
ENSMUSG00000044250	2,31E-01	4,033566372	6,87E+00	8,76E-03	8,47E-02	Pced1b	1,17	No
ENSMUSG00000044252	2,76E-01	4,407988793	1,14E+01	7,42E-04	1,27E-02	Osbpl1a	1,21	Yes
ENSMUSG00000044254	3,32E-01	-1,289571756	4,71E-01	4,93E-01	7,78E-01	Pcsk9	1,26	No
ENSMUSG00000044258	-2,81E+00	-2,318130211	7,56E+00	5,98E-03	6,40E-02	Ctla2a	0,14	No
ENSMUSG00000044268	5,37E-02	4,379743945	4,28E-01	5,13E-01	7,95E-01	Gm4895	1,04	No
ENSMUSG00000044276	1,85E-01	-2,832010644	2,48E-02	8,75E-01	1,00E+00	4933427E11Rik	1,14	No
ENSMUSG00000044279	-7,62E-01	-2,226241736	9,18E-01	3,38E-01	6,38E-01	Crb3	0,59	No
ENSMUSG00000044285	-7,49E-02	9,02099344	1,73E+00	1,88E-01	5,50E-01	Gm1821	0,95	No
ENSMUSG00000044286	3,14E-02	-3,107617333	-7,69E-10	1,00E+00	1,00E+00	Olfr221	1,02	No
ENSMUSG00000044287	-5,71E-01	-2,015677644	5,79E-01	4,47E-01	7,37E-01	Nrn1l	0,67	No
ENSMUSG00000044288	-3,21E+00	-2,896063972	4,33E+00	3,75E-02	2,30E-01	Cnr1	0,11	No
ENSMUSG00000044296	-1,87E+00	-3,033619006	1,41E+00	2,36E-01	5,50E-01	Zfp879	0,27	No
ENSMUSG00000044303	-4,53E-02	-0,098156859	2,30E-02	8,79E-01	1,00E+00	Cdkn2a	0,97	No
ENSMUSG00000044306	1,31E-01	1,714675773	6,48E-01	4,21E-01	7,16E-01	4930500M09Rik	1,09	No
ENSMUSG00000044308	2,01E-02	6,391568876	1,00E-01	7,52E-01	9,32E-01	Ubr3	1,01	No
ENSMUSG00000044309	-1,58E+00	3,801652936	2,93E+02	1,01E-65	1,00E-61	Apol7c	0,33	Yes
ENSMUSG00000044313	-2,76E-01	3,264752355	7,30E+00	6,91E-03	7,10E-02	Mab21l3	0,83	No
ENSMUSG00000044317	4,27E-01	0,508499552	2,62E+00	1,05E-01	3,98E-01	Gpr4	1,34	No

ENSMUSG00000044320	-2,15E-01	-0,2575923	4,38E-01	5,08E-01	7,92E-01	1700001O22Rik	0,86	No
ENSMUSG00000044322	-2,71E+00	-3,033620915	2,92E+00	8,75E-02	3,78E-01	Dsc1	0,15	No
ENSMUSG00000044328	1,55E-01	3,637795117	2,57E+00	1,09E-01	4,05E-01	Trp53i13	1,11	No
ENSMUSG00000044330	8,61E-03	5,052880649	1,38E-02	9,07E-01	1,00E+00	Gm9790	1,01	No
ENSMUSG00000044337	-2,02E+00	-2,65512808	3,10E+00	7,85E-02	3,58E-01	Ackr3	0,25	No
ENSMUSG00000044338	-9,59E-01	-2,600656517	8,07E-01	3,69E-01	6,70E-01	Aplnr	0,51	No
ENSMUSG00000044339	2,43E-01	2,023624443	2,66E+00	1,03E-01	3,95E-01	Alkbh2	1,18	No
ENSMUSG00000044340	-2,14E-01	5,352105766	9,26E+00	2,35E-03	3,19E-02	Phlpp1	0,86	Yes
ENSMUSG00000044345	-1,23E-01	5,62772842	3,32E+00	6,84E-02	3,29E-01	Marveld1	0,92	No
ENSMUSG00000044349	-9,92E-02	2,738491555	6,58E-01	4,17E-01	7,12E-01	Snhg11	0,93	No
ENSMUSG00000044350	-2,09E-02	6,297911835	1,08E-01	7,42E-01	9,27E-01	Lacc1	0,99	No
ENSMUSG00000044352	1,41E+00	-2,863749101	1,30E+00	2,54E-01	5,50E-01	Sowaha	2,66	No
ENSMUSG00000044362	-2,48E-01	-1,126718867	3,53E-01	5,53E-01	8,19E-01	Ccdc89	0,84	No
ENSMUSG00000044364	6,74E-01	-2,226289429	6,58E-01	4,17E-01	7,12E-01	Tmem74b	1,60	No
ENSMUSG00000044367	1,19E-01	3,618852458	1,51E+00	2,20E-01	5,50E-01	Slc16a13	1,09	No
ENSMUSG00000044377	-4,07E-02	2,036438041	7,45E-02	7,85E-01	9,51E-01	Gm13080	0,97	No
ENSMUSG00000044387	-1,18E+00	-2,268009104	1,81E+00	1,78E-01	5,39E-01	2410080I02Rik	0,44	No
ENSMUSG00000044390	-1,80E-05	0,406091652	5,66E-09	1,00E+00	1,00E+00	Tigd3	1,00	No
ENSMUSG00000044393	-3,43E+00	-1,980352302	1,84E+01	1,82E-05	5,69E-04	Dsg2	0,09	Yes
ENSMUSG00000044405	1,92E+00	-2,655135671	2,73E+00	9,83E-02	3,91E-01	Adig	3,77	No
ENSMUSG00000044408	3,22E-02	6,590168402	2,67E-01	6,06E-01	8,49E-01	Sptssa	1,02	No
ENSMUSG00000044424	-2,08E-01	7,445899221	1,23E+01	4,47E-04	8,38E-03	Gm9493	0,87	Yes
ENSMUSG00000044429	8,53E-01	-2,600639625	6,34E-01	4,26E-01	7,20E-01	Cryga	1,81	No
ENSMUSG00000044433	-8,62E-01	-1,02475577	3,40E+00	6,50E-02	3,19E-01	Camsap3	0,55	No
ENSMUSG00000044434	1,32E-01	4,355437396	2,53E+00	1,12E-01	4,11E-01	Gm9791	1,10	No
ENSMUSG00000044442	2,40E-03	3,281615197	5,17E-04	9,82E-01	1,00E+00	N6amt1	1,00	No
ENSMUSG00000044447	1,97E-01	5,697829099	8,39E+00	3,77E-03	4,53E-02	Dock5	1,15	Yes
ENSMUSG00000044452	-3,50E-01	-0,10771093	1,36E+00	2,44E-01	5,50E-01	Zfp507	0,78	No
ENSMUSG00000044453	-2,70E-01	-1,68313162	2,37E-01	6,26E-01	8,60E-01	Ffar1	0,83	No
ENSMUSG00000044456	1,68E-01	7,90467826	8,24E+00	4,10E-03	4,82E-02	Rin3	1,12	Yes
ENSMUSG00000044457	-1,87E+00	-3,107637761	1,39E+00	2,38E-01	5,50E-01	Stk-ps2	0,27	No
ENSMUSG00000044461	-2,63E-02	-3,033653157	2,33E-04	9,88E-01	1,00E+00	Shisa2	0,98	No

ENSMUSG00000044465	5,73E-02	3,279957998	3,03E-01	5,82E-01	8,38E-01	Fam160a2	1,04	No
ENSMUSG00000044468	8,68E-02	5,815030237	1,68E+00	1,95E-01	5,50E-01	Fam46c	1,06	No
ENSMUSG00000044469	2,55E-01	2,898784505	4,75E+00	2,93E-02	1,95E-01	Tnfaip8l1	1,19	No
ENSMUSG00000044471	1,53E-01	0,062310212	2,72E-01	6,02E-01	8,48E-01	Lncpint	1,11	No
ENSMUSG00000044475	1,36E-01	3,358644665	1,75E+00	1,85E-01	5,49E-01	Ascc1	1,10	No
ENSMUSG00000044477	-2,49E-01	6,425566383	1,55E+01	8,07E-05	2,03E-03	Zfand3	0,84	Yes
ENSMUSG00000044485	4,58E-01	7,900217545	6,06E+01	6,95E-15	1,06E-12	Klk1b11	1,37	Yes
ENSMUSG00000044496	1,64E-02	6,810389491	7,15E-02	7,89E-01	9,54E-01	2510039O18Rik	1,01	No
ENSMUSG00000044501	-1,16E-01	2,943750822	1,05E+00	3,05E-01	6,01E-01	Zfp758	0,92	No
ENSMUSG00000044502	1,70E-01	3,503787811	2,94E+00	8,66E-02	3,78E-01	Bod1	1,13	No
ENSMUSG00000044518	1,81E+00	-3,10765737	1,28E+00	2,58E-01	5,50E-01	Foxe3	3,49	No
ENSMUSG00000044519	-4,13E-02	-3,033659495	7,08E-04	9,79E-01	1,00E+00	Zfp488	0,97	No
ENSMUSG00000044522	-5,56E-01	-1,997994108	6,75E-01	4,11E-01	7,07E-01	A730020M07Rik	0,68	No
ENSMUSG00000044528	3,14E-02	-3,107617333	-7,69E-10	1,00E+00	1,00E+00	Tram1l1	1,02	No
ENSMUSG00000044533	-1,59E-02	7,570439694	7,24E-02	7,88E-01	9,53E-01	Rps2	0,99	No
ENSMUSG00000044534	1,08E+00	-2,401372133	1,51E+00	2,20E-01	5,50E-01	Ackr2	2,12	No
ENSMUSG00000044544	3,14E-02	-3,107617333	-7,69E-10	1,00E+00	1,00E+00	4921513I03Rik	1,02	No
ENSMUSG00000044548	-2,21E-01	-1,448636921	1,68E-01	6,82E-01	8,94E-01	Dact1	0,86	No
ENSMUSG00000044551	1,99E-01	1,508097306	1,30E+00	2,54E-01	5,50E-01	9930012K11Rik	1,15	No
ENSMUSG00000044556	-8,67E-02	-2,89613315	3,24E-03	9,55E-01	1,00E+00	Tex38	0,94	No
ENSMUSG00000044562	-5,95E-01	0,31005672	5,41E+00	2,00E-02	1,51E-01	Rasip1	0,66	No
ENSMUSG00000044566	1,26E-01	0,915417498	3,72E-01	5,42E-01	8,11E-01	Cage1	1,09	No
ENSMUSG00000044573	-1,90E-02	4,889921212	6,38E-02	8,01E-01	9,61E-01	Acp1	0,99	No
ENSMUSG00000044576	4,21E-01	-2,800943341	8,81E-02	7,67E-01	9,40E-01	Garem2	1,34	No
ENSMUSG00000044581	-1,87E+00	-3,107637761	1,39E+00	2,38E-01	5,50E-01	4932415D10Rik	0,27	No
ENSMUSG00000044595	2,95E-01	0,388899528	1,27E+00	2,59E-01	5,52E-01	Dnd1	1,23	No
ENSMUSG00000044600	9,81E-02	6,035015468	2,25E+00	1,33E-01	4,59E-01	Smim7	1,07	No
ENSMUSG00000044609	-1,77E-01	6,539799171	8,00E+00	4,67E-03	5,31E-02	Gm9294	0,88	No
ENSMUSG00000044617	-1,68E-01	3,798771869	3,32E+00	6,83E-02	3,29E-01	Zbtb39	0,89	No
ENSMUSG00000044624	3,14E-02	-3,107617333	-7,69E-10	1,00E+00	1,00E+00	Gm4922	1,02	No
ENSMUSG00000044626	-3,02E-01	-0,226431049	9,16E-01	3,39E-01	6,39E-01	Liph	0,81	No
ENSMUSG00000044627	-7,63E-02	5,864312628	1,33E+00	2,50E-01	5,50E-01	Swi5	0,95	No

ENSMUSG00000044628	5,02E-01	-1,18567977	1,21E+00	2,71E-01	5,67E-01	Rnf208	1,42	No
ENSMUSG00000044629	-4,93E-03	-1,522598022	6,98E-05	9,93E-01	1,00E+00	Cnrip1	1,00	No
ENSMUSG00000044636	6,81E-02	2,302175412	2,55E-01	6,13E-01	8,53E-01	Csrnp2	1,05	No
ENSMUSG00000044641	3,27E-01	-0,216216053	1,07E+00	3,00E-01	5,96E-01	Pard6b	1,25	No
ENSMUSG00000044645	-1,32E-01	3,311231639	1,56E+00	2,12E-01	5,50E-01	Gm7334	0,91	No
ENSMUSG00000044646	-6,21E-01	-2,07006663	7,49E-01	3,87E-01	6,87E-01	Zbtb7c	0,65	No
ENSMUSG00000044647	6,93E-02	-0,863648822	2,68E-02	8,70E-01	1,00E+00	Csrnp3	1,05	No
ENSMUSG00000044664	-1,87E+00	-3,03364178	1,41E+00	2,36E-01	5,50E-01	Prss42	0,27	No
ENSMUSG00000044667	-3,94E-01	1,200801787	4,53E+00	3,33E-02	2,14E-01	Plppr4	0,76	No
ENSMUSG00000044674	-1,10E-01	2,180990348	5,81E-01	4,46E-01	7,37E-01	Fzd1	0,93	No
ENSMUSG00000044681	-1,68E+00	-1,831955941	6,61E+00	1,01E-02	9,42E-02	Cnpy1	0,31	No
ENSMUSG00000044694	2,74E-02	-3,033621357	9,16E-12	1,00E+00	1,00E+00	2010007H06Rik	1,02	No
ENSMUSG00000044697	-1,28E-01	1,25536828	4,64E-01	4,96E-01	7,81E-01	Gm5479	0,91	No
ENSMUSG00000044700	3,62E-02	3,859734672	1,57E-01	6,92E-01	9,00E-01	Tmem201	1,03	No
ENSMUSG00000044701	-1,02E-01	1,322413689	2,93E-01	5,88E-01	8,40E-01	Il27	0,93	No
ENSMUSG00000044702	3,40E-01	3,170208672	1,05E+01	1,20E-03	1,88E-02	Palb2	1,27	Yes
ENSMUSG00000044703	-5,14E-01	1,041178655	6,86E+00	8,83E-03	8,52E-02	Phf11a	0,70	No
ENSMUSG00000044707	-1,87E+00	-3,107637761	1,39E+00	2,38E-01	5,50E-01	Ccnjl	0,27	No
ENSMUSG00000044708	-4,76E-02	2,933586697	1,71E-01	6,79E-01	8,93E-01	Kcnj10	0,97	No
ENSMUSG00000044709	-1,36E-01	5,098617274	3,52E+00	6,06E-02	3,05E-01	Gemin7	0,91	No
ENSMUSG00000044712	2,61E-01	5,061511408	1,25E+01	3,97E-04	7,57E-03	Slc38a6	1,20	Yes
ENSMUSG00000044715	5,72E-02	5,042332753	6,12E-01	4,34E-01	7,27E-01	Gskip	1,04	No
ENSMUSG00000044716	-1,43E-01	3,686904108	2,31E+00	1,29E-01	4,50E-01	Dok7	0,91	No
ENSMUSG00000044719	2,97E-01	-2,448745626	9,01E-02	7,64E-01	9,39E-01	E230025N22Rik	1,23	No
ENSMUSG00000044724	-9,31E-01	-2,770598494	7,58E-01	3,84E-01	6,85E-01	Gpr152	0,52	No
ENSMUSG00000044726	-1,35E+00	-2,711637134	1,13E+00	2,87E-01	5,83E-01	Erich5	0,39	No
ENSMUSG00000044730	3,25E-02	4,04796149	1,38E-01	7,11E-01	9,10E-01	9930104L06Rik	1,02	No
ENSMUSG00000044734	-3,92E-01	2,491335229	9,11E+00	2,54E-03	3,37E-02	Serpinb1a	0,76	Yes
ENSMUSG00000044737	2,65E+00	-3,033655087	2,73E+00	9,86E-02	3,91E-01	Klk14	6,30	No
ENSMUSG00000044749	4,20E-01	-0,205926726	1,69E+00	1,94E-01	5,50E-01	Abca6	1,34	No
ENSMUSG00000044751	1,40E-02	6,780308224	5,14E-02	8,21E-01	9,72E-01	Gm12231	1,01	No
ENSMUSG00000044757	6,59E-02	5,635546654	9,36E-01	3,33E-01	6,34E-01	Gm6430	1,05	No

ENSMUSG00000044763	-2,10E-03	4,151950041	5,99E-04	9,80E-01	1,00E+00	Trmt10c	1,00	No
ENSMUSG00000044768	1,30E-01	5,988361566	3,93E+00	4,73E-02	2,60E-01	D1Ertd622e	1,09	No
ENSMUSG00000044770	5,60E-01	-0,574374688	2,35E+00	1,26E-01	4,44E-01	Scml4	1,47	No
ENSMUSG00000044783	2,02E-01	7,242349435	1,14E+01	7,36E-04	1,26E-02	Hjurp	1,15	Yes
ENSMUSG00000044786	4,35E-01	8,477310774	5,56E+01	9,06E-14	1,26E-11	Zfp36	1,35	Yes
ENSMUSG00000044787	-1,41E-01	-1,247046981	1,00E-01	7,52E-01	9,32E-01	Spata32	0,91	No
ENSMUSG00000044788	3,98E-02	2,041130723	7,45E-02	7,85E-01	9,51E-01	Fads6	1,03	No
ENSMUSG00000044791	-6,20E-02	7,00961833	1,05E+00	3,06E-01	6,04E-01	Setd2	0,96	No
ENSMUSG00000044792	1,30E-01	5,116854469	3,17E+00	7,49E-02	3,48E-01	Isca1	1,09	No
ENSMUSG00000044795	9,54E-02	3,177465644	7,93E-01	3,73E-01	6,74E-01	Cyb5d1	1,07	No
ENSMUSG00000044807	-4,54E-01	-2,070172257	3,80E-01	5,38E-01	8,09E-01	Zfp354c	0,73	No
ENSMUSG00000044811	1,28E-01	7,015339709	4,45E+00	3,49E-02	2,21E-01	Cd300c2	1,09	No
ENSMUSG00000044813	-2,06E-01	4,009634406	5,34E+00	2,08E-02	1,55E-01	Shb	0,87	No
ENSMUSG00000044814	4,98E-01	-0,24702186	2,09E+00	1,48E-01	4,88E-01	Olfr543	1,41	No
ENSMUSG00000044816	-4,78E-01	-0,011215248	2,70E+00	1,01E-01	3,92E-01	D630023F18Rik	0,72	No
ENSMUSG00000044819	-1,87E+00	-3,107637761	1,39E+00	2,38E-01	5,50E-01	Oxgr1	0,27	No
ENSMUSG00000044820	-1,92E-02	-0,748231865	2,36E-03	9,61E-01	1,00E+00	AY074887	0,99	No
ENSMUSG00000044824	-1,24E+00	-2,548227111	1,48E+00	2,23E-01	5,50E-01	Olfr545	0,42	No
ENSMUSG00000044827	-5,13E-01	3,318414177	2,43E+01	8,42E-07	3,82E-05	Tlr1	0,70	Yes
ENSMUSG00000044835	1,00E+00	-1,832015556	2,30E+00	1,29E-01	4,51E-01	Ankrd45	2,00	No
ENSMUSG00000044847	1,22E-01	3,281811655	1,36E+00	2,44E-01	5,50E-01	Lsm11	1,09	No
ENSMUSG00000044854	5,53E-02	-0,683061243	1,89E-02	8,91E-01	1,00E+00	1700056E22Rik	1,04	No
ENSMUSG00000044857	2,66E-01	5,978411864	1,66E+01	4,70E-05	1,29E-03	Lemd2	1,20	Yes
ENSMUSG00000044860	3,13E-01	-1,54819901	2,83E-01	5,95E-01	8,44E-01	Gm1123	1,24	No
ENSMUSG00000044864	2,48E-02	5,116253466	1,15E-01	7,34E-01	9,24E-01	Ankrd50	1,02	No
ENSMUSG00000044867	2,44E-01	-0,694925456	4,16E-01	5,19E-01	7,98E-01	Gimap1os	1,18	No
ENSMUSG00000044876	4,95E-02	4,00671084	3,16E-01	5,74E-01	8,33E-01	Zfp444	1,03	No
ENSMUSG00000044881	-4,02E-03	3,430279446	1,64E-03	9,68E-01	1,00E+00	Coa4	1,00	No
ENSMUSG00000044894	-2,71E-02	5,989667791	1,70E-01	6,80E-01	8,93E-01	Uqcrq	0,98	No
ENSMUSG00000044903	9,01E-03	-3,107646452	-5,16E-10	1,00E+00	1,00E+00	Psg22	1,01	No
ENSMUSG00000044906	5,91E-02	5,186879818	6,71E-01	4,13E-01	7,08E-01	4930503L19Rik	1,04	No
ENSMUSG00000044912	-1,94E+00	-3,107619243	1,50E+00	2,21E-01	5,50E-01	Syt16	0,26	No

ENSMUSG00000044916	2,24E-01	0,355018096	7,70E-01	3,80E-01	6,81E-01	1700029I15Rik	1,17	No
ENSMUSG00000044927	-5,49E-01	2,817314033	2,21E+01	2,56E-06	1,00E-04	H1fx	0,68	Yes
ENSMUSG00000044934	1,05E-01	4,937408318	1,96E+00	1,61E-01	5,10E-01	Zfp367	1,08	No
ENSMUSG00000044937	4,25E-01	0,316982388	2,54E+00	1,11E-01	4,09E-01	Ttc41	1,34	No
ENSMUSG00000044948	1,69E-01	4,959708325	5,21E+00	2,25E-02	1,63E-01	Cfap43	1,12	No
ENSMUSG00000044949	-4,69E-03	2,439381408	1,29E-03	9,71E-01	1,00E+00	Ubtd2	1,00	No
ENSMUSG00000044950	-3,34E-02	3,958486443	1,42E-01	7,06E-01	9,08E-01	Pwwp2a	0,98	No
ENSMUSG00000044951	-5,20E+00	-2,401271954	1,88E+01	1,48E-05	4,74E-04	Mylk4	0,03	Yes
ENSMUSG00000044952	2,49E-01	2,487987888	3,83E+00	5,03E-02	2,72E-01	Kctd21	1,19	No
ENSMUSG00000044957	6,75E-02	0,686259338	8,76E-02	7,67E-01	9,40E-01	Pp2d1	1,05	No
ENSMUSG00000044966	3,24E-01	1,943654274	4,66E+00	3,09E-02	2,03E-01	Fbxo48	1,25	No
ENSMUSG00000044968	5,18E-02	2,836668401	2,00E-01	6,55E-01	8,79E-01	Napepld	1,04	No
ENSMUSG00000044976	-1,87E+00	-3,107637761	1,39E+00	2,38E-01	5,50E-01	Wdr72	0,27	No
ENSMUSG00000044982	-2,48E-02	2,997317443	4,69E-02	8,29E-01	9,76E-01	Sft2d3	0,98	No
ENSMUSG00000044986	4,25E-02	2,469238175	1,04E-01	7,48E-01	9,30E-01	Tst	1,03	No
ENSMUSG00000044991	-3,80E-02	2,342684954	7,89E-02	7,79E-01	9,47E-01	1110034G24Rik	0,97	No
ENSMUSG00000044994	-1,98E-02	-3,033655952	2,83E-11	1,00E+00	1,00E+00	Olfr1426	0,99	No
ENSMUSG00000044997	-2,20E-02	-3,107647797	1,16E-04	9,91E-01	1,00E+00	E130304I02Rik	0,98	No
ENSMUSG00000045004	2,94E-01	-1,655196537	2,49E-01	6,17E-01	8,55E-01	Spata21	1,23	No
ENSMUSG00000045005	1,64E-01	3,753112716	3,07E+00	7,99E-02	3,62E-01	Fzd5	1,12	No
ENSMUSG00000045007	3,14E-01	-0,038383968	1,11E+00	2,92E-01	5,87E-01	Tubg2	1,24	No
ENSMUSG00000045009	-1,67E-01	-0,785630292	1,82E-01	6,70E-01	8,88E-01	Prrt3	0,89	No
ENSMUSG00000045027	1,27E+00	-2,896151645	1,00E+00	3,17E-01	6,17E-01	Prss22	2,41	No
ENSMUSG00000045031	-4,75E-01	0,592909157	4,18E+00	4,09E-02	2,40E-01	Cetn4	0,72	No
ENSMUSG00000045034	-6,19E-02	-2,832032244	1,69E-03	9,67E-01	1,00E+00	Ankrd34b	0,96	No
ENSMUSG00000045036	-3,83E-02	-3,107651936	-7,91E-11	1,00E+00	1,00E+00	Tmem232	0,97	No
ENSMUSG00000045038	1,65E-01	3,378820558	2,50E+00	1,14E-01	4,17E-01	Prkce	1,12	No
ENSMUSG00000045039	2,46E-02	5,973479607	1,41E-01	7,07E-01	9,08E-01	Megf8	1,02	No
ENSMUSG00000045045	1,11E-01	3,645663165	1,28E+00	2,57E-01	5,50E-01	Lrfn4	1,08	No
ENSMUSG00000045048	4,17E-01	0,26622847	2,36E+00	1,25E-01	4,42E-01	Gm9795	1,34	No
ENSMUSG00000045052	1,78E+00	-2,963287612	1,28E+00	2,58E-01	5,50E-01	Prlhr	3,44	No
ENSMUSG00000045053	-1,59E+00	-1,683096091	7,80E+00	5,24E-03	5,78E-02	Kcng3	0,33	No

ENSMUSG00000045055	-2,28E-01	8,104886767	1,54E+01	8,59E-05	2,14E-03	Rpsa-ps2	0,85	Yes
ENSMUSG00000045062	-1,91E+00	-2,963238542	1,45E+00	2,29E-01	5,50E-01	Pcdhb7	0,27	No
ENSMUSG00000045064	4,72E-01	0,612859368	3,87E+00	4,92E-02	2,68E-01	Zc2hc1c	1,39	No
ENSMUSG00000045065	-3,14E-01	0,09578906	1,21E+00	2,71E-01	5,67E-01	9930022D16Rik	0,80	No
ENSMUSG00000045071	1,98E-01	3,155811244	3,23E+00	7,25E-02	3,41E-01	E130308A19Rik	1,15	No
ENSMUSG00000045075	9,38E-02	0,493251397	1,40E-01	7,09E-01	9,09E-01	Gm9796	1,07	No
ENSMUSG00000045078	-7,26E-02	6,266796327	1,29E+00	2,56E-01	5,50E-01	Rnf216	0,95	No
ENSMUSG00000045083	-1,98E-02	-3,033655952	2,83E-11	1,00E+00	1,00E+00	Lingo2	0,99	No
ENSMUSG00000045087	-9,30E-01	-2,770634646	7,57E-01	3,84E-01	6,86E-01	S1pr5	0,52	No
ENSMUSG00000045091	-6,11E-01	-2,401328975	5,09E-01	4,76E-01	7,64E-01	Aqp12	0,65	No
ENSMUSG00000045092	-1,03E+00	-0,522500661	8,17E+00	4,25E-03	4,95E-02	S1pr1	0,49	Yes
ENSMUSG00000045094	2,42E-01	0,957076255	1,25E+00	2,63E-01	5,58E-01	Arhgef37	1,18	No
ENSMUSG00000045095	-6,16E-01	-1,998031002	9,58E-01	3,28E-01	6,29E-01	Magi1	0,65	No
ENSMUSG00000045098	9,89E-02	5,8277322	2,22E+00	1,36E-01	4,65E-01	Kmt5b	1,07	No
ENSMUSG00000045100	-3,61E-02	2,308080374	7,07E-02	7,90E-01	9,54E-01	Slc25a26	0,98	No
ENSMUSG00000045102	5,27E-01	-0,466983647	2,05E+00	1,52E-01	4,94E-01	Poln	1,44	No
ENSMUSG00000045104	5,46E-01	2,284772633	1,48E+01	1,22E-04	2,87E-03	Gm5514	1,46	Yes
ENSMUSG00000045106	-2,21E-01	0,894537018	1,04E+00	3,07E-01	6,05E-01	Ccdc73	0,86	No
ENSMUSG00000045107	1,03E-01	2,448909894	5,98E-01	4,39E-01	7,32E-01	Saysd1	1,07	No
ENSMUSG00000045114	1,11E-01	-0,937719447	7,20E-02	7,88E-01	9,54E-01	Prrt2	1,08	No
ENSMUSG00000045126	-3,88E-03	-3,03362684	4,02E-12	1,00E+00	1,00E+00	Olfr1445	1,00	No
ENSMUSG00000045128	-8,89E-02	5,643534737	1,72E+00	1,90E-01	5,50E-01	Rpl18a	0,94	No
ENSMUSG00000045132	9,01E-03	-3,107646452	-5,16E-10	1,00E+00	1,00E+00	Olfr620	1,01	No
ENSMUSG00000045136	-2,34E-01	4,739315057	9,24E+00	2,36E-03	3,20E-02	Tubb2b	0,85	Yes
ENSMUSG00000045140	5,89E-03	2,184819898	1,73E-03	9,67E-01	1,00E+00	Pigw	1,00	No
ENSMUSG00000045160	-3,74E-02	3,624462194	1,50E-01	6,99E-01	9,04E-01	Bola3	0,97	No
ENSMUSG00000045165	1,40E-02	5,001764276	3,58E-02	8,50E-01	9,89E-01	AI467606	1,01	No
ENSMUSG00000045174	-5,23E-02	-2,963267963	1,24E-03	9,72E-01	1,00E+00	Amer3	0,96	No
ENSMUSG00000045176	2,10E-01	4,942517965	7,98E+00	4,72E-03	5,35E-02	Borcs6	1,16	No
ENSMUSG00000045193	-3,00E-01	4,695033747	1,47E+01	1,26E-04	2,95E-03	Cirbp	0,81	Yes
ENSMUSG00000045201	1,81E+00	-3,10765737	1,28E+00	2,58E-01	5,50E-01	Lrrc3b	3,49	No
ENSMUSG00000045205	2,22E-01	5,673871225	1,08E+01	1,04E-03	1,67E-02	Dpy19l4	1,17	Yes

ENSMUSG00000045210	1,19E-01	6,27954704	3,49E+00	6,16E-02	3,09E-01	Vcpip1	1,09	No
ENSMUSG00000045211	-1,67E-01	4,927643772	5,03E+00	2,50E-02	1,75E-01	Nudt18	0,89	No
ENSMUSG00000045215	1,87E+00	-3,107639691	1,38E+00	2,40E-01	5,50E-01	Asxl3	3,67	No
ENSMUSG00000045216	1,02E-01	5,256012277	2,03E+00	1,55E-01	4,98E-01	Hs6st1	1,07	No
ENSMUSG00000045225	9,01E-03	-3,107646452	-5,16E-10	1,00E+00	1,00E+00	Olfr1152	1,01	No
ENSMUSG00000045231	5,75E-01	0,686152762	5,91E+00	1,51E-02	1,26E-01	BC106179	1,49	No
ENSMUSG00000045232	-1,87E+00	-3,107637761	1,39E+00	2,38E-01	5,50E-01	Rln3	0,27	No
ENSMUSG00000045238	-5,47E-01	-1,117179868	1,52E+00	2,17E-01	5,50E-01	A730035I17Rik	0,68	No
ENSMUSG00000045248	1,55E-01	3,454707216	2,41E+00	1,20E-01	4,33E-01	Med26	1,11	No
ENSMUSG00000045251	-1,21E-01	3,267399473	1,34E+00	2,48E-01	5,50E-01	Zfp688	0,92	No
ENSMUSG00000045252	-3,75E-02	3,893893292	1,76E-01	6,75E-01	8,91E-01	Zfp574	0,97	No
ENSMUSG00000045257	6,65E-02	-0,350024734	3,76E-02	8,46E-01	9,87E-01	Morn2	1,05	No
ENSMUSG00000045259	-2,11E-01	1,118108418	1,15E+00	2,84E-01	5,81E-01	Klhdc9	0,86	No
ENSMUSG00000045268	-1,22E-01	1,18131031	4,30E-01	5,12E-01	7,95E-01	Zfp691	0,92	No
ENSMUSG00000045273	6,95E-02	2,502778057	2,85E-01	5,94E-01	8,43E-01	Cenph	1,05	No
ENSMUSG00000045275	-8,36E-02	-2,311171455	6,24E-03	9,37E-01	1,00E+00	Lca5l	0,94	No
ENSMUSG00000045282	5,17E-02	2,838013335	1,94E-01	6,60E-01	8,82E-01	Tmem86b	1,04	No
ENSMUSG00000045287	2,22E-01	1,999030221	2,18E+00	1,40E-01	4,72E-01	Rtn4rl1	1,17	No
ENSMUSG00000045291	1,81E+00	-3,10765737	1,28E+00	2,58E-01	5,50E-01	E330021D16Rik	3,49	No
ENSMUSG00000045294	2,59E-01	6,556803848	1,71E+01	3,63E-05	1,04E-03	Insig1	1,20	Yes
ENSMUSG00000045302	4,42E-02	6,64824211	5,10E-01	4,75E-01	7,63E-01	Preb	1,03	No
ENSMUSG00000045312	2,75E-01	6,944412745	2,04E+01	6,21E-06	2,19E-04	Lhfpl2	1,21	Yes
ENSMUSG00000045314	2,34E-01	-2,497648324	6,91E-02	7,93E-01	9,56E-01	Sowahb	1,18	No
ENSMUSG00000045316	-7,86E-02	1,850188473	2,49E-01	6,18E-01	8,55E-01	Fahd1	0,95	No
ENSMUSG00000045318	-6,38E-02	-0,509892729	2,85E-02	8,66E-01	9,99E-01	Adra2c	0,96	No
ENSMUSG00000045319	1,29E-01	1,814268878	6,32E-01	4,27E-01	7,21E-01	Proser2	1,09	No
ENSMUSG00000045322	2,56E-01	4,972581208	1,19E+01	5,58E-04	1,00E-02	Tlr9	1,19	Yes
ENSMUSG00000045326	6,17E-01	0,16000752	4,74E+00	2,94E-02	1,95E-01	Fndc7	1,53	No
ENSMUSG00000045327	-1,51E-01	3,835998635	2,65E+00	1,04E-01	3,95E-01	6330549D23Rik	0,90	No
ENSMUSG00000045328	3,12E-01	6,082933012	2,30E+01	1,58E-06	6,66E-05	Cenpe	1,24	Yes
ENSMUSG00000045333	-2,66E+00	-3,033639428	2,81E+00	9,35E-02	3,83E-01	Zfp423	0,16	No
ENSMUSG00000045345	-5,09E-02	-2,045623798	5,48E-03	9,41E-01	1,00E+00	9530056K15Rik	0,97	No

ENSMUSG00000045348	1,58E-01	-1,051972488	1,45E-01	7,04E-01	9,06E-01	Nyap1	1,12	No
ENSMUSG00000045349	-7,77E-01	3,494239489	6,42E+01	1,15E-15	1,91E-13	Sh2d5	0,58	Yes
ENSMUSG00000045350	-5,57E-01	-2,832008579	2,42E-01	6,23E-01	8,58E-01	Fam186a	0,68	No
ENSMUSG00000045362	8,07E-02	5,322188864	1,29E+00	2,56E-01	5,50E-01	Tnfrsf26	1,06	No
ENSMUSG00000045374	2,46E-01	7,059739076	1,65E+01	4,92E-05	1,34E-03	Wdr81	1,19	Yes
ENSMUSG00000045377	-4,84E-02	1,836254248	8,96E-02	7,65E-01	9,39E-01	Tmem88	0,97	No
ENSMUSG00000045378	-5,13E-01	-2,832036919	1,32E-01	7,16E-01	9,13E-01	Pxt1	0,70	No
ENSMUSG00000045381	8,36E-02	-1,683071424	1,87E-02	8,91E-01	1,00E+00	Olfr433	1,06	No
ENSMUSG00000045382	-9,50E-02	5,252193015	1,80E+00	1,79E-01	5,40E-01	Cxcr4	0,94	No
ENSMUSG00000045391	1,79E-01	0,932237772	7,08E-01	4,00E-01	6,99E-01	1700120B22Rik	1,13	No
ENSMUSG00000045392	4,74E-01	-2,600680117	1,75E-01	6,76E-01	8,91E-01	Olfr1033	1,39	No
ENSMUSG00000045394	4,07E-01	3,662184281	1,78E+01	2,42E-05	7,32E-04	Epcam	1,33	Yes
ENSMUSG00000045404	1,68E-01	5,246306471	5,48E+00	1,92E-02	1,47E-01	Kcnk13	1,12	No
ENSMUSG00000045409	3,70E-02	4,702755309	2,31E-01	6,31E-01	8,63E-01	Trim39	1,03	No
ENSMUSG00000045410	2,69E-01	3,60397308	7,90E+00	4,93E-03	5,54E-02	Akr1e1	1,21	No
ENSMUSG00000045411	1,22E-01	7,83070347	4,29E+00	3,83E-02	2,32E-01	2410002F23Rik	1,09	No
ENSMUSG00000045414	3,66E-01	3,806037836	1,56E+01	7,62E-05	1,93E-03	1190002N15Rik	1,29	Yes
ENSMUSG00000045435	6,96E-02	3,49992022	4,84E-01	4,87E-01	7,74E-01	Tmem60	1,05	No
ENSMUSG00000045438	7,04E-02	4,223088001	6,90E-01	4,06E-01	7,02E-01	Cox19	1,05	No
ENSMUSG00000045440	-1,14E-02	-3,033635021	1,50E-05	9,97E-01	1,00E+00	Insm2	0,99	No
ENSMUSG00000045441	6,37E-01	1,229229776	1,05E+01	1,20E-03	1,88E-02	Gprin3	1,56	Yes
ENSMUSG00000045455	1,56E-01	3,874629218	2,89E+00	8,93E-02	3,79E-01	Gm9797	1,11	No
ENSMUSG00000045464	4,23E-02	2,050756594	8,18E-02	7,75E-01	9,45E-01	2810002D19Rik	1,03	No
ENSMUSG00000045466	2,80E-02	3,119314338	6,62E-02	7,97E-01	9,58E-01	Zfp956	1,02	No
ENSMUSG00000045467	-2,45E-01	-2,107680555	1,18E-01	7,31E-01	9,22E-01	Ttll13	0,84	No
ENSMUSG00000045471	-2,71E+00	-3,033620915	2,92E+00	8,75E-02	3,78E-01	Hcrt	0,15	No
ENSMUSG00000045479	1,81E+00	-3,10765737	1,28E+00	2,58E-01	5,50E-01	Olfr459	3,49	No
ENSMUSG00000045482	3,24E-02	7,442246679	2,97E-01	5,86E-01	8,39E-01	Trrap	1,02	No
ENSMUSG00000045498	-1,94E+00	-3,107619243	1,50E+00	2,21E-01	5,50E-01	Pcdhb3	0,26	No
ENSMUSG00000045502	2,27E-01	6,745763095	1,35E+01	2,37E-04	5,00E-03	Hcar2	1,17	Yes
ENSMUSG00000045503	2,48E-02	5,064252188	1,14E-01	7,35E-01	9,24E-01	Sys1	1,02	No
ENSMUSG00000045506	-1,89E+00	-3,1076441	1,42E+00	2,33E-01	5,50E-01	A930002H24Rik	0,27	No

ENSMUSG00000045509	4,99E-01	0,867729776	5,23E+00	2,21E-02	1,61E-01	Gpr150		1,41	No
ENSMUSG00000045514	7,18E-01	-1,627580449	1,39E+00	2,38E-01	5,50E-01	Olfr460		1,65	No
ENSMUSG00000045519	1,79E-01	2,347916605	1,75E+00	1,86E-01	5,50E-01	Zfp560		1,13	No
ENSMUSG00000045521	-9,37E-01	-2,548260194	7,69E-01	3,81E-01	6,82E-01	Tssk2		0,52	No
ENSMUSG00000045532	-1,86E+00	-3,033612668	1,39E+00	2,38E-01	5,50E-01	C1ql1		0,28	No
ENSMUSG00000045538	8,47E-02	4,485018353	1,09E+00	2,96E-01	5,92E-01	Ddx28		1,06	No
ENSMUSG00000045551	-3,78E-02	2,678046243	9,05E-02	7,63E-01	9,39E-01	Fpr1		0,97	No
ENSMUSG00000045555	-7,01E-02	-1,600713609	1,61E-02	8,99E-01	1,00E+00	Mettl24		0,95	No
ENSMUSG00000045573	-8,94E-01	1,337605135	2,44E+01	7,68E-07	3,53E-05	Penk		0,54	Yes
ENSMUSG00000045576	3,58E-02	4,103265638	1,71E-01	6,79E-01	8,93E-01	St7l		1,03	No
ENSMUSG00000045589	1,82E+00	-3,033661385	1,31E+00	2,52E-01	5,50E-01	Frrs1l		3,54	No
ENSMUSG00000045591	2,65E+00	-3,033655087	2,73E+00	9,86E-02	3,91E-01	Olig3		6,30	No
ENSMUSG00000045594	5,53E-02	6,168547245	7,36E-01	3,91E-01	6,91E-01	Glb1		1,04	No
ENSMUSG00000045598	-8,55E-02	3,020653482	6,05E-01	4,37E-01	7,29E-01	Zfp553		0,94	No
ENSMUSG00000045609	1,84E-01	-0,669151041	2,31E-01	6,31E-01	8,63E-01	Gm5455		1,14	No
ENSMUSG00000045613	2,13E-01	-1,136415809	1,69E-01	6,81E-01	8,94E-01	Chrm2		1,16	No
ENSMUSG00000045624	2,11E-01	4,179106385	6,04E+00	1,40E-02	1,20E-01	Esf1		1,16	No
ENSMUSG00000045625	4,16E-01	2,358418272	9,18E+00	2,44E-03	3,28E-02	Pigz		1,33	Yes
ENSMUSG00000045629	1,26E+00	-2,832033055	9,81E-01	3,22E-01	6,22E-01	Sh3tc2		2,39	No
ENSMUSG00000045631	1,85E+00	-3,107657832	1,35E+00	2,46E-01	5,50E-01	Tmprss12		3,61	No
ENSMUSG00000045636	-1,28E-01	3,346255201	1,50E+00	2,20E-01	5,50E-01	Mtus1		0,92	No
ENSMUSG00000045639	4,39E-04	3,5930715	2,09E-05	9,96E-01	1,00E+00	Zfp629		1,00	No
ENSMUSG00000045657	9,01E-03	-3,107646452	-5,16E-10	1,00E+00	1,00E+00	Pcdhb10		1,01	No
ENSMUSG00000045658	2,63E-01	6,416983397	1,72E+01	3,36E-05	9,67E-04	Pid1		1,20	Yes
ENSMUSG00000045659	-5,66E-01	-0,195481171	3,08E+00	7,93E-02	3,60E-01	Plekha7		0,68	No
ENSMUSG00000045662	8,50E-02	-1,165742302	3,54E-02	8,51E-01	9,90E-01	Henmt1		1,06	No
ENSMUSG00000045664	-2,87E-01	5,099380168	1,56E+01	7,92E-05	2,00E-03	Cdc42ep2		0,82	Yes
ENSMUSG00000045665	1,66E-01	6,143132209	6,62E+00	1,01E-02	9,39E-02	Mfsd5		1,12	No
ENSMUSG00000045667	8,71E-01	-2,355645367	1,17E+00	2,80E-01	5,77E-01	Smtnl2		1,83	No
ENSMUSG00000045671	-1,08E-01	4,993850983	2,12E+00	1,45E-01	4,82E-01	Spred2		0,93	No
ENSMUSG00000045672	-5,25E-01	3,785017316	3,31E+01	8,56E-09	6,01E-07	Col27a1		0,69	Yes
ENSMUSG00000045679	5,32E-02	6,996111164	7,68E-01	3,81E-01	6,82E-01	Pqlc3		1,04	No

ENSMUSG00000045690	-6,97E-02	8,826458582	1,49E+00	2,22E-01	5,50E-01	Wdr89	0,95	No
ENSMUSG00000045691	-3,89E-02	4,197244803	2,12E-01	6,45E-01	8,73E-01	Thtpa	0,97	No
ENSMUSG00000045699	-1,36E+00	-2,226282811	2,55E+00	1,10E-01	4,06E-01	Gm21411	0,39	No
ENSMUSG00000045709	4,22E-01	-2,033661588	3,87E-01	5,34E-01	8,07E-01	Smkr-ps	1,34	No
ENSMUSG00000045725	2,28E-01	4,453694103	7,80E+00	5,22E-03	5,77E-02	Prr15	1,17	No
ENSMUSG00000045730	4,71E-01	4,51223527	3,49E+01	3,43E-09	2,59E-07	Adrb2	1,39	Yes
ENSMUSG00000045744	1,65E-01	2,528295082	1,70E+00	1,92E-01	5,50E-01	Bricd5	1,12	No
ENSMUSG00000045746	-4,02E-02	-2,711703535	1,12E-03	9,73E-01	1,00E+00	B230317F23Rik	0,97	No
ENSMUSG00000045751	2,19E-01	4,732254383	7,96E+00	4,77E-03	5,40E-02	Mms22l	1,16	No
ENSMUSG00000045752	1,17E-01	4,347551785	1,95E+00	1,63E-01	5,13E-01	Tssc4	1,08	No
ENSMUSG00000045757	2,37E-01	3,095373908	4,64E+00	3,13E-02	2,05E-01	Zfp764	1,18	No
ENSMUSG00000045761	1,83E-01	-2,355595008	4,99E-02	8,23E-01	9,73E-01	Togaram2	1,13	No
ENSMUSG00000045763	-3,56E-01	8,17043497	3,77E+01	8,13E-10	6,77E-08	Basp1	0,78	Yes
ENSMUSG00000045767	5,03E-02	6,40604478	6,36E-01	4,25E-01	7,19E-01	B230219D22Rik	1,04	No
ENSMUSG00000045775	-5,27E-01	-1,051713458	1,44E+00	2,30E-01	5,50E-01	Slc16a5	0,69	No
ENSMUSG00000045777	1,89E-01	-0,185625556	3,46E-01	5,56E-01	8,21E-01	Ifitm10	1,14	No
ENSMUSG00000045780	4,53E-01	-2,711695922	1,60E-01	6,89E-01	8,99E-01	Olfr624	1,37	No
ENSMUSG00000045790	-8,92E-01	-1,424645971	2,69E+00	1,01E-01	3,93E-01	Ccdc149	0,54	No
ENSMUSG00000045794	-1,90E+00	-3,033647263	1,44E+00	2,30E-01	5,50E-01	Lkaaear1	0,27	No
ENSMUSG00000045795	-1,31E-01	4,092928934	2,30E+00	1,30E-01	4,52E-01	Whamm	0,91	No
ENSMUSG00000045799	-2,21E-01	7,368204579	1,37E+01	2,20E-04	4,71E-03	Gm9800	0,86	Yes
ENSMUSG00000045812	7,31E-01	-2,963279337	2,91E-01	5,90E-01	8,41E-01	Olfr984	1,66	No
ENSMUSG00000045817	4,81E-01	8,692474002	7,02E+01	5,34E-17	9,76E-15	Zfp36l2	1,40	Yes
ENSMUSG00000045822	1,77E-01	1,547829867	1,11E+00	2,91E-01	5,87E-01	Zswim3	1,13	No
ENSMUSG00000045826	-1,33E-01	0,502474546	3,02E-01	5,83E-01	8,38E-01	Ptprcap	0,91	No
ENSMUSG00000045827	-8,52E-01	5,889004597	1,67E+02	3,36E-38	3,59E-35	Serpinb9	0,55	Yes
ENSMUSG00000045838	-5,63E-01	-0,997833867	1,58E+00	2,09E-01	5,50E-01	A430105I19Rik	0,68	No
ENSMUSG00000045842	-7,68E-01	-1,015837157	3,29E+00	6,97E-02	3,33E-01	Tgif2-ps2	0,59	No
ENSMUSG00000045854	7,34E-02	2,608291914	3,38E-01	5,61E-01	8,24E-01	Lyrm2	1,05	No
ENSMUSG00000045867	-1,93E-01	3,580006214	3,98E+00	4,59E-02	2,56E-01	Cradd	0,87	No
ENSMUSG00000045868	-1,15E-01	7,731988783	3,85E+00	4,98E-02	2,70E-01	Gvin1	0,92	No
ENSMUSG00000045871	-8,31E-01	-2,831987771	3,79E-01	5,38E-01	8,09E-01	Slitrk6	0,56	No

ENSMUSG00000045886	-5,17E-02	3,437374838	2,65E-01	6,06E-01	8,50E-01	Gm9803	0,96	No
ENSMUSG00000045896	5,75E-02	5,127587053	6,32E-01	4,27E-01	7,21E-01	Paip2b	1,04	No
ENSMUSG00000045903	7,33E-02	0,337689576	8,59E-02	7,69E-01	9,42E-01	Npas4	1,05	No
ENSMUSG00000045912	-4,13E-02	-3,033659495	7,08E-04	9,79E-01	1,00E+00	C2cd4c	0,97	No
ENSMUSG00000045915	-3,84E+00	-2,832005976	7,04E+00	7,96E-03	7,87E-02	Ccdc42	0,07	No
ENSMUSG00000045917	3,10E-02	6,810783512	2,57E-01	6,12E-01	8,52E-01	Tmem268	1,02	No
ENSMUSG00000045929	-1,88E+00	-3,033633562	1,42E+00	2,34E-01	5,50E-01	Gm20715	0,27	No
ENSMUSG00000045932	-3,84E-01	3,439366439	1,49E+01	1,13E-04	2,68E-03	Ifit2	0,77	Yes
ENSMUSG00000045934	-8,38E-02	-0,074814653	7,89E-02	7,79E-01	9,47E-01	Mtmr11	0,94	No
ENSMUSG00000045942	2,34E-02	-1,413060095	2,18E-03	9,63E-01	1,00E+00	BC049762	1,02	No
ENSMUSG00000045948	8,89E-02	4,152461995	1,06E+00	3,04E-01	6,01E-01	Mrps12	1,06	No
ENSMUSG00000045952	-4,15E-02	4,181304666	2,32E-01	6,30E-01	8,62E-01	Gm5548	0,97	No
ENSMUSG00000045954	-1,35E+00	-2,832003544	1,14E+00	2,86E-01	5,82E-01	Cavin2	0,39	No
ENSMUSG00000045962	-1,03E-01	8,932937434	3,26E+00	7,11E-02	3,38E-01	Wnk1	0,93	No
ENSMUSG00000045968	-4,25E-01	1,036222084	4,35E+00	3,70E-02	2,29E-01	Teddm2	0,74	No
ENSMUSG00000045969	-5,26E-02	5,317679501	5,52E-01	4,57E-01	7,46E-01	Ing1	0,96	No
ENSMUSG00000045973	-2,66E-02	6,483640217	1,81E-01	6,70E-01	8,88E-01	Slc25a51	0,98	No
ENSMUSG00000045975	3,41E-01	3,727021721	1,29E+01	3,31E-04	6,56E-03	C2cd2	1,27	Yes
ENSMUSG00000045980	2,21E-01	6,335689269	1,20E+01	5,43E-04	9,82E-03	Tmem104	1,17	Yes
ENSMUSG00000045983	8,49E-02	8,72191594	2,20E+00	1,38E-01	4,68E-01	Eif4g1	1,06	No
ENSMUSG00000045991	1,17E-01	-0,366968731	1,22E-01	7,27E-01	9,19E-01	Onecut2	1,08	No
ENSMUSG00000045994	-1,23E+00	-3,146117124	7,12E-01	3,99E-01	6,98E-01	B3gat1	0,42	No
ENSMUSG00000045996	-3,03E-02	0,943683524	2,02E-02	8,87E-01	1,00E+00	Polr2k	0,98	No
ENSMUSG00000046005	-5,88E-02	-3,033616507	1,62E-03	9,68E-01	1,00E+00	D830044D21Rik	0,96	No
ENSMUSG00000046006	3,27E-02	4,791287278	1,82E-01	6,70E-01	8,88E-01	Gapt	1,02	No
ENSMUSG00000046010	1,07E-01	3,939963158	1,42E+00	2,34E-01	5,50E-01	Zfp830	1,08	No
ENSMUSG00000046016	-3,83E-02	-3,107651936	-7,91E-11	1,00E+00	1,00E+00	Olfr1364	0,97	No
ENSMUSG00000046020	-5,68E-02	5,900919208	7,39E-01	3,90E-01	6,90E-01	Pofut1	0,96	No
ENSMUSG00000046027	2,31E-01	5,1469534	1,02E+01	1,43E-03	2,14E-02	Stard5	1,17	Yes
ENSMUSG00000046031	-6,17E-02	0,551404481	6,55E-02	7,98E-01	9,59E-01	Fam26f	0,96	No
ENSMUSG00000046034	-6,55E-02	5,241679194	8,37E-01	3,60E-01	6,61E-01	Otulin	0,96	No
ENSMUSG00000046056	3,70E-02	0,298915577	1,97E-02	8,88E-01	1,00E+00	Sbsn	1,03	No

ENSMUSG00000046057	2,19E-01	4,560888088	7,43E+00	6,40E-03	6,73E-02	Gm15428	1,16	No
ENSMUSG00000046058	-2,58E-01	2,783540447	4,54E+00	3,31E-02	2,13E-01	Eid2	0,84	No
ENSMUSG00000046062	1,58E-01	7,626776849	7,19E+00	7,34E-03	7,43E-02	Ppp1r15b	1,12	No
ENSMUSG00000046070	2,87E-01	-2,448727156	8,39E-02	7,72E-01	9,43E-01	Igfals	1,22	No
ENSMUSG00000046079	2,46E-02	7,054607834	1,64E-01	6,85E-01	8,97E-01	Lrrc8d	1,02	No
ENSMUSG00000046080	2,04E-01	4,336120817	5,96E+00	1,46E-02	1,23E-01	Clec9a	1,15	No
ENSMUSG00000046082	1,85E+00	-3,107657832	1,35E+00	2,46E-01	5,50E-01	Tmem174	3,61	No
ENSMUSG00000046085	-2,53E-01	1,664432073	2,37E+00	1,24E-01	4,40E-01	4931422A03Rik	0,84	No
ENSMUSG00000046093	-1,13E+00	-1,497634164	4,62E+00	3,16E-02	2,06E-01	Hpcal4	0,46	No
ENSMUSG00000046096	6,61E-02	3,789417224	5,13E-01	4,74E-01	7,62E-01	BC030336	1,05	No
ENSMUSG00000046101	-4,55E-01	-0,305821693	1,88E+00	1,70E-01	5,26E-01	Mcmdc2	0,73	No
ENSMUSG00000046108	-2,97E-01	-0,360936774	7,31E-01	3,92E-01	6,93E-01	Il17c	0,81	No
ENSMUSG00000046110	-5,87E-02	4,839968387	6,06E-01	4,36E-01	7,29E-01	Serinc4	0,96	No
ENSMUSG00000046111	9,78E-02	5,325911428	1,91E+00	1,66E-01	5,19E-01	Cep295	1,07	No
ENSMUSG00000046138	4,30E-02	5,109316546	3,48E-01	5,55E-01	8,20E-01	9930021J03Rik	1,03	No
ENSMUSG00000046139	6,07E-02	6,028584033	8,65E-01	3,52E-01	6,53E-01	Patl1	1,04	No
ENSMUSG00000046145	1,28E-01	2,00311862	7,02E-01	4,02E-01	7,00E-01	1700018M17Rik	1,09	No
ENSMUSG00000046152	2,55E-01	0,609901752	1,11E+00	2,92E-01	5,88E-01	Fut10	1,19	No
ENSMUSG00000046157	1,65E-01	6,014398429	6,43E+00	1,12E-02	1,02E-01	Tmem229b	1,12	No
ENSMUSG00000046159	-2,58E-02	-0,216014988	5,95E-03	9,39E-01	1,00E+00	Chrm3	0,98	No
ENSMUSG00000046160	-3,83E-02	-3,107651936	-7,91E-11	1,00E+00	1,00E+00	Olig1	0,97	No
ENSMUSG00000046167	-5,25E-02	-3,033661434	4,08E-11	1,00E+00	1,00E+00	Gldn	0,96	No
ENSMUSG00000046168	6,62E-01	-1,159369265	2,00E+00	1,57E-01	5,03E-01	Kcnrg	1,58	No
ENSMUSG00000046169	-5,29E-02	0,997647691	6,22E-02	8,03E-01	9,62E-01	Adamts6	0,96	No
ENSMUSG00000046179	2,55E-01	5,665612707	1,41E+01	1,72E-04	3,82E-03	E2f8	1,19	Yes
ENSMUSG00000046182	-3,83E-02	-3,107651936	-7,91E-11	1,00E+00	1,00E+00	Gsg1l	0,97	No
ENSMUSG00000046185	5,57E-02	3,983351436	3,91E-01	5,32E-01	8,06E-01	Zfp84	1,04	No
ENSMUSG00000046186	2,23E-01	2,537757641	3,01E+00	8,30E-02	3,70E-01	Cd109	1,17	No
ENSMUSG00000046201	2,82E-02	5,910195915	1,82E-01	6,70E-01	8,88E-01	Scaf8	1,02	No
ENSMUSG00000046204	-1,87E+00	-3,107637761	1,39E+00	2,38E-01	5,50E-01	Pnma2	0,27	No
ENSMUSG00000046207	-1,49E-02	6,552443892	5,75E-02	8,11E-01	9,66E-01	Pik3r6	0,99	No
ENSMUSG00000046210	-1,89E+00	-3,1076441	1,42E+00	2,33E-01	5,50E-01	Olfr735	0,27	No

ENSMUSG00000046215	1,81E+00	-3,10765737	1,28E+00	2,58E-01	5,50E-01	Rprml	3,49	No
ENSMUSG00000046223	1,52E-01	9,033063281	7,09E+00	7,73E-03	7,70E-02	Plaur	1,11	No
ENSMUSG00000046229	1,92E-01	5,515765414	7,73E+00	5,43E-03	5,94E-02	Scand1	1,14	No
ENSMUSG00000046230	4,85E-02	5,270840704	4,63E-01	4,96E-01	7,81E-01	Vps13a	1,03	No
ENSMUSG00000046240	-5,18E-02	-2,711720429	1,84E-03	9,66E-01	1,00E+00	Hepacam	0,96	No
ENSMUSG00000046242	-2,64E-01	-0,863765099	3,97E-01	5,29E-01	8,04E-01	Nme9	0,83	No
ENSMUSG00000046245	1,46E-02	7,995442204	6,26E-02	8,02E-01	9,62E-01	Pilra	1,01	No
ENSMUSG00000046248	1,85E+00	-3,107657832	1,35E+00	2,46E-01	5,50E-01	Krtap5-3	3,61	No
ENSMUSG00000046272	1,11E+00	-2,448678118	1,18E+00	2,77E-01	5,74E-01	Olfr1444	2,17	No
ENSMUSG00000046275	-5,94E-01	-2,31106433	4,81E-01	4,88E-01	7,75E-01	Tusc5	0,66	No
ENSMUSG00000046280	-1,53E+00	-1,831885481	3,39E+00	6,55E-02	3,21E-01	She	0,35	No
ENSMUSG00000046282	-1,87E+00	-3,107637761	1,39E+00	2,38E-01	5,50E-01	Adam20	0,27	No
ENSMUSG00000046295	1,80E-01	3,76382218	3,77E+00	5,21E-02	2,78E-01	Ankle1	1,13	No
ENSMUSG00000046311	-1,58E-01	5,714969646	5,61E+00	1,78E-02	1,40E-01	Zfp62	0,90	No
ENSMUSG00000046312	3,42E-01	1,240461103	3,05E+00	8,06E-02	3,64E-01	AI464131	1,27	No
ENSMUSG00000046314	-2,55E-01	4,01418414	8,47E+00	3,62E-03	4,40E-02	Stxbp6	0,84	Yes
ENSMUSG00000046318	2,39E-01	3,53826983	5,86E+00	1,55E-02	1,29E-01	Ccbe1	1,18	No
ENSMUSG00000046321	1,85E+00	-3,107657832	1,35E+00	2,46E-01	5,50E-01	Hs3st2	3,61	No
ENSMUSG00000046323	-1,80E+00	-2,268162877	5,10E+00	2,40E-02	1,70E-01	Dppa3	0,29	No
ENSMUSG00000046324	-6,66E-02	5,118589864	8,39E-01	3,60E-01	6,61E-01	Ermp1	0,95	No
ENSMUSG00000046329	1,57E-01	2,242447785	1,23E+00	2,68E-01	5,65E-01	Slc25a23	1,12	No
ENSMUSG00000046330	-5,17E-01	3,294784812	2,40E+01	9,71E-07	4,32E-05	Rpl37a	0,70	Yes
ENSMUSG00000046334	-3,01E-01	2,175953309	4,32E+00	3,77E-02	2,31E-01	Gm6195	0,81	No
ENSMUSG00000046337	2,14E-01	-2,57423717	3,33E-02	8,55E-01	9,92E-01	Fam178b	1,16	No
ENSMUSG00000046338	9,82E-01	-0,160885526	8,62E+00	3,33E-03	4,14E-02	Gpat2	1,98	Yes
ENSMUSG00000046341	1,17E-01	6,507297829	3,46E+00	6,28E-02	3,13E-01	Gm11223	1,08	No
ENSMUSG00000046345	-1,36E+00	-2,896135617	1,16E+00	2,82E-01	5,78E-01	Smco1	0,39	No
ENSMUSG00000046351	1,46E-01	3,591046256	2,33E+00	1,27E-01	4,47E-01	Zfp322a	1,11	No
ENSMUSG00000046352	2,20E-01	-2,401359131	6,09E-02	8,05E-01	9,63E-01	Gjb2	1,16	No
ENSMUSG00000046364	-1,30E-01	6,043423607	3,99E+00	4,57E-02	2,55E-01	Rpl27a	0,91	No
ENSMUSG00000046367	-3,83E-02	-3,107651936	-7,91E-11	1,00E+00	1,00E+00	Mgat4e	0,97	No
ENSMUSG00000046372	-2,93E-01	3,334595805	8,20E+00	4,18E-03	4,89E-02	Gm5590	0,82	Yes

ENSMUSG00000046378	2,06E-02	-1,785565946	9,60E-04	9,75E-01	1,00E+00	Asphd1	1,01	No
ENSMUSG00000046380	2,14E-02	1,320183709	1,42E-02	9,05E-01	1,00E+00	Jrk	1,01	No
ENSMUSG00000046387	-2,64E+00	-2,963219259	2,78E+00	9,55E-02	3,86E-01	Pcdhb17	0,16	No
ENSMUSG00000046388	-1,20E-01	-1,355622578	5,29E-02	8,18E-01	9,70E-01	Gm9806	0,92	No
ENSMUSG00000046402	-7,53E-01	-0,252130698	5,49E+00	1,91E-02	1,47E-01	Rbp1	0,59	No
ENSMUSG00000046404	7,85E-02	3,420402076	5,95E-01	4,40E-01	7,33E-01	Yod1	1,06	No
ENSMUSG00000046408	4,66E-01	-0,863746811	1,39E+00	2,38E-01	5,50E-01	1700067K01Rik	1,38	No
ENSMUSG00000046410	-1,38E-01	7,527971835	5,48E+00	1,92E-02	1,47E-01	Kcnk6	0,91	No
ENSMUSG00000046411	-1,87E+00	-3,033619006	1,41E+00	2,36E-01	5,50E-01	Rps4l-ps	0,27	No
ENSMUSG00000046415	3,68E-01	-0,740965789	8,41E-01	3,59E-01	6,61E-01	B430212C06Rik	1,29	No
ENSMUSG00000046417	1,20E-01	3,089808138	1,16E+00	2,82E-01	5,79E-01	Lrrc75a	1,09	No
ENSMUSG00000046434	-5,55E-02	6,554471581	7,89E-01	3,74E-01	6,75E-01	Hnrnpa1	0,96	No
ENSMUSG00000046440	-5,73E-02	3,835404914	3,92E-01	5,31E-01	8,05E-01	Gm5564	0,96	No
ENSMUSG00000046441	2,20E-01	3,1740878	4,16E+00	4,13E-02	2,41E-01	Cmtr2	1,16	No
ENSMUSG00000046442	2,48E-01	0,152153188	7,59E-01	3,84E-01	6,85E-01	Ppm1e	1,19	No
ENSMUSG00000046447	-6,27E-01	-1,896062818	9,91E-01	3,19E-01	6,20E-01	Camk2n1	0,65	No
ENSMUSG00000046456	3,92E-01	1,945301567	6,84E+00	8,90E-03	8,57E-02	Tmem150b	1,31	No
ENSMUSG00000046460	4,13E-02	-1,51004652	5,18E-03	9,43E-01	1,00E+00	Sh2d7	1,03	No
ENSMUSG00000046463	-1,87E-01	0,563520859	5,96E-01	4,40E-01	7,32E-01	5930403N24Rik	0,88	No
ENSMUSG00000046470	2,45E-01	-2,497589408	7,58E-02	7,83E-01	9,50E-01	Sox18	1,19	No
ENSMUSG00000046480	-1,16E+00	3,820506077	1,61E+02	5,50E-37	5,15E-34	Scn4b	0,45	Yes
ENSMUSG00000046486	1,81E+00	-3,10765737	1,28E+00	2,58E-01	5,50E-01	Olfr231	3,49	No
ENSMUSG00000046487	-5,58E-01	-1,929260836	6,79E-01	4,10E-01	7,06E-01	Mospd4	0,68	No
ENSMUSG00000046490	-3,83E-02	-3,107651936	-7,91E-11	1,00E+00	1,00E+00	Rnf222	0,97	No
ENSMUSG00000046491	-2,63E-02	-3,033653157	2,33E-04	9,88E-01	1,00E+00	C1qtnf2	0,98	No
ENSMUSG00000046516	-2,63E-01	4,800562639	1,20E+01	5,45E-04	9,85E-03	Cox17	0,83	Yes
ENSMUSG00000046519	-4,41E-02	6,188642663	4,78E-01	4,89E-01	7,76E-01	Golph3l	0,97	No
ENSMUSG00000046523	-2,20E+00	-2,033604492	6,26E+00	1,24E-02	1,09E-01	Kctd4	0,22	No
ENSMUSG00000046541	2,71E-01	2,898369243	5,65E+00	1,74E-02	1,38E-01	Zfp526	1,21	No
ENSMUSG00000046546	-3,05E-02	6,006119345	2,18E-01	6,40E-01	8,70E-01	Fam43a	0,98	No
ENSMUSG00000046556	5,95E-02	4,982623615	6,39E-01	4,24E-01	7,18E-01	Zfp319	1,04	No
ENSMUSG00000046562	1,09E-01	6,174032586	2,84E+00	9,19E-02	3,82E-01	Unc119b	1,08	No

ENSMUSG00000046567	6,65E-02	2,434781614	2,55E-01	6,14E-01	8,53E-01	4930430F08Rik	1,05	No
ENSMUSG00000046572	2,13E-02	3,065086407	3,74E-02	8,47E-01	9,87E-01	Zfp518b	1,01	No
ENSMUSG00000046573	7,47E-02	3,237973342	4,95E-01	4,82E-01	7,69E-01	Lyrm4	1,05	No
ENSMUSG00000046574	-7,84E-02	5,524844682	1,31E+00	2,52E-01	5,50E-01	Prr12	0,95	No
ENSMUSG00000046580	9,25E-02	2,524052156	5,31E-01	4,66E-01	7,55E-01	Gm7862	1,07	No
ENSMUSG00000046589	6,18E-01	-2,448735094	4,26E-01	5,14E-01	7,96E-01	Lrrc8e	1,53	No
ENSMUSG00000046591	2,00E-01	4,57886083	6,33E+00	1,19E-02	1,06E-01	Ticrr	1,15	No
ENSMUSG00000046593	-6,40E-01	-0,107667786	3,69E+00	5,48E-02	2,87E-01	Tmem215	0,64	No
ENSMUSG00000046598	7,97E-02	5,881577296	1,45E+00	2,29E-01	5,50E-01	Bdh1	1,06	No
ENSMUSG00000046603	1,55E-01	2,509666632	1,46E+00	2,27E-01	5,50E-01	Tcaim	1,11	No
ENSMUSG00000046605	1,76E-01	3,498671922	3,16E+00	7,55E-02	3,50E-01	B3gntl1	1,13	No
ENSMUSG00000046607	6,95E-01	-2,963260828	2,60E-01	6,10E-01	8,51E-01	Hrk	1,62	No
ENSMUSG00000046610	-8,52E-01	-1,683072168	1,97E+00	1,61E-01	5,09E-01	Oacyl	0,55	No
ENSMUSG00000046613	4,73E-01	-2,424847269	3,04E-01	5,82E-01	8,38E-01	Vwa5b2	1,39	No
ENSMUSG00000046618	-3,17E+00	-2,832010676	4,23E+00	3,97E-02	2,36E-01	Olfml2a	0,11	No
ENSMUSG00000046620	-2,28E-01	0,966369517	1,21E+00	2,72E-01	5,69E-01	Rps11-ps4	0,85	No
ENSMUSG00000046634	9,01E-03	-3,107646452	-5,16E-10	1,00E+00	1,00E+00	Pkd1l1	1,01	No
ENSMUSG00000046636	-3,83E-03	5,38905436	2,98E-03	9,56E-01	1,00E+00	Gm7729	1,00	No
ENSMUSG00000046652	4,15E-01	-2,600737388	1,35E-01	7,14E-01	9,12E-01	Tas2r143	1,33	No
ENSMUSG00000046658	2,96E-01	3,025562059	7,14E+00	7,54E-03	7,58E-02	Zfp316	1,23	No
ENSMUSG00000046667	-5,66E-03	3,086571993	2,74E-03	9,58E-01	1,00E+00	Rbm12b1	1,00	No
ENSMUSG00000046668	1,36E-02	3,196525699	1,64E-02	8,98E-01	1,00E+00	Cxxc5	1,01	No
ENSMUSG00000046671	6,83E-02	6,007553196	1,08E+00	2,98E-01	5,94E-01	Mtfr1l	1,05	No
ENSMUSG00000046675	5,70E-02	4,497427584	4,95E-01	4,82E-01	7,69E-01	Tmem251	1,04	No
ENSMUSG00000046679	1,77E-01	3,37808083	3,02E+00	8,22E-02	3,68E-01	C87436	1,13	No
ENSMUSG00000046687	2,68E-02	9,437481224	2,25E-01	6,36E-01	8,67E-01	Gm5424	1,02	No
ENSMUSG00000046688	-8,09E-02	5,180007026	1,26E+00	2,61E-01	5,55E-01	Tifa	0,95	No
ENSMUSG00000046691	5,00E-02	6,564833671	6,44E-01	4,22E-01	7,18E-01	Chtf8	1,04	No
ENSMUSG00000046694	8,11E-01	-2,655120825	5,72E-01	4,49E-01	7,39E-01	Fam46b	1,75	No
ENSMUSG00000046707	-4,45E-02	5,92758649	4,56E-01	4,99E-01	7,85E-01	Csnk2a2	0,97	No
ENSMUSG00000046709	2,39E-01	-2,497583165	7,18E-02	7,89E-01	9,54E-01	Mapk10	1,18	No
ENSMUSG00000046711	-1,97E-02	7,374552481	1,09E-01	7,41E-01	9,27E-01	Hmga1	0,99	No

ENSMUSG00000046714	-1,89E+00	-3,1076441	1,42E+00	2,33E-01	5,50E-01	Foxc2	0,27	No
ENSMUSG00000046718	-4,03E-01	6,18066271	3,92E+01	3,87E-10	3,39E-08	Bst2	0,76	Yes
ENSMUSG00000046719	3,14E-01	-1,832153908	2,62E-01	6,09E-01	8,51E-01	Nxph3	1,24	No
ENSMUSG00000046721	-1,26E-01	8,53827782	4,79E+00	2,87E-02	1,92E-01	Rpl14-ps1	0,92	No
ENSMUSG00000046722	-5,21E-02	8,101507785	8,06E-01	3,69E-01	6,71E-01	Cdc42se1	0,96	No
ENSMUSG00000046727	-4,19E-01	1,791989165	6,94E+00	8,44E-03	8,24E-02	Cystm1	0,75	No
ENSMUSG00000046731	2,38E-01	3,487960764	5,96E+00	1,46E-02	1,23E-01	Kctd11	1,18	No
ENSMUSG00000046733	-6,79E-01	1,546018026	1,53E+01	9,23E-05	2,26E-03	Gprc5a	0,62	Yes
ENSMUSG00000046743	-3,22E-01	-2,333155942	1,30E-01	7,18E-01	9,15E-01	Fat4	0,80	No
ENSMUSG00000046747	7,64E-02	0,697005485	1,10E-01	7,40E-01	9,26E-01	Gm9812	1,05	No
ENSMUSG00000046748	9,01E-03	-3,107646452	-5,16E-10	1,00E+00	1,00E+00	Tmem45a2	1,01	No
ENSMUSG00000046753	-3,59E-02	3,608351178	1,40E-01	7,08E-01	9,09E-01	Ccdc66	0,98	No
ENSMUSG00000046756	-1,22E-01	5,471282292	3,13E+00	7,70E-02	3,54E-01	Mrps7	0,92	No
ENSMUSG00000046761	6,46E-02	1,713260049	1,59E-01	6,90E-01	8,99E-01	Fam83h	1,05	No
ENSMUSG00000046768	-3,77E-01	0,71058488	2,66E+00	1,03E-01	3,95E-01	Rhoj	0,77	No
ENSMUSG00000046782	-1,89E+00	-3,1076441	1,42E+00	2,33E-01	5,50E-01	Ttc6	0,27	No
ENSMUSG00000046785	-3,26E-02	4,758715951	1,81E-01	6,71E-01	8,89E-01	Epm2aip1	0,98	No
ENSMUSG00000046791	3,27E-02	3,483340439	1,04E-01	7,47E-01	9,30E-01	Riox1	1,02	No
ENSMUSG00000046792	3,01E-02	4,518642689	1,44E-01	7,04E-01	9,06E-01	Zfp787	1,02	No
ENSMUSG00000046793	2,91E-01	-2,655118016	8,60E-02	7,69E-01	9,42E-01	Gpr61	1,22	No
ENSMUSG00000046794	1,27E-01	4,022767023	2,05E+00	1,53E-01	4,95E-01	Ppp1r3b	1,09	No
ENSMUSG00000046798	-1,01E-01	2,595765227	6,37E-01	4,25E-01	7,19E-01	Cldn12	0,93	No
ENSMUSG00000046805	1,16E-01	11,03745125	4,31E+00	3,80E-02	2,31E-01	Mpeg1	1,08	No
ENSMUSG00000046806	2,94E-02	3,833009101	1,05E-01	7,46E-01	9,29E-01	3110062M04Rik	1,02	No
ENSMUSG00000046807	4,34E-01	-2,448684096	1,48E-01	7,01E-01	9,05E-01	Lrrc75b	1,35	No
ENSMUSG00000046808	-2,15E-01	3,971825096	5,81E+00	1,59E-02	1,31E-01	Atp10d	0,86	No
ENSMUSG00000046814	-6,49E-02	-0,816295308	2,55E-02	8,73E-01	1,00E+00	Gchfr	0,96	No
ENSMUSG00000046818	-1,22E-01	1,124341391	3,87E-01	5,34E-01	8,07E-01	Ddit4l	0,92	No
ENSMUSG00000046822	8,84E-02	4,718765515	1,31E+00	2,52E-01	5,50E-01	Slc39a3	1,06	No
ENSMUSG00000046826	3,02E-01	0,337668397	1,25E+00	2,64E-01	5,59E-01	Fam187b	1,23	No
ENSMUSG00000046834	-2,31E+00	-2,35552661	7,16E+00	7,45E-03	7,51E-02	Krt1	0,20	No
ENSMUSG00000046836	7,65E-02	6,350540098	1,45E+00	2,29E-01	5,50E-01	Brox	1,05	No

ENSMUSG00000046841	1,11E-01	5,034113552	2,23E+00	1,36E-01	4,64E-01	Ckap4	1,08	No
ENSMUSG00000046844	-4,38E-01	3,329909581	1,88E+01	1,42E-05	4,56E-04	Vat1l	0,74	Yes
ENSMUSG00000046854	-6,97E-01	-2,770595499	3,23E-01	5,70E-01	8,31E-01	Pip5kl1	0,62	No
ENSMUSG00000046856	-1,87E+00	-3,107637761	1,39E+00	2,38E-01	5,50E-01	Gpr1	0,27	No
ENSMUSG00000046861	1,55E-01	5,767467523	5,34E+00	2,08E-02	1,55E-01	Hectd3	1,11	No
ENSMUSG00000046862	-3,33E-02	5,196361715	2,14E-01	6,44E-01	8,72E-01	Pramef8	0,98	No
ENSMUSG00000046865	1,94E-01	4,183984396	5,15E+00	2,33E-02	1,67E-01	Fbl	1,14	No
ENSMUSG00000046876	1,53E-01	5,268601813	4,55E+00	3,30E-02	2,13E-01	Atxn1	1,11	No
ENSMUSG00000046879	-6,60E-02	5,193489652	8,49E-01	3,57E-01	6,58E-01	Irgm1	0,96	No
ENSMUSG00000046881	1,84E+00	-3,033649193	1,33E+00	2,49E-01	5,50E-01	Olfr374	3,58	No
ENSMUSG00000046897	3,62E-02	5,62056187	2,86E-01	5,93E-01	8,42E-01	Zfp740	1,03	No
ENSMUSG00000046899	-1,87E+00	-3,107637761	1,39E+00	2,38E-01	5,50E-01	Prl7a2	0,27	No
ENSMUSG00000046908	3,12E-01	5,806176399	2,15E+01	3,61E-06	1,37E-04	Ltb4r1	1,24	Yes
ENSMUSG00000046909	2,80E-02	2,281147197	4,16E-02	8,38E-01	9,82E-01	Tefm	1,02	No
ENSMUSG00000046916	-1,91E+00	-2,963268868	1,45E+00	2,29E-01	5,50E-01	Myct1	0,27	No
ENSMUSG00000046934	-2,40E-02	5,292521835	1,14E-01	7,36E-01	9,24E-01	Csl	0,98	No
ENSMUSG00000046947	8,98E-02	3,781453119	9,39E-01	3,32E-01	6,33E-01	Adck2	1,06	No
ENSMUSG00000046949	5,24E-02	5,289034818	5,45E-01	4,60E-01	7,49E-01	Nqo2	1,04	No
ENSMUSG00000046952	1,38E-01	1,868121884	8,33E-01	3,61E-01	6,63E-01	Gm5815	1,10	No
ENSMUSG00000046959	2,55E-01	0,607182401	1,15E+00	2,84E-01	5,81E-01	Slc26a1	1,19	No
ENSMUSG00000046961	-8,89E-01	-2,832016389	4,33E-01	5,11E-01	7,94E-01	Gpr156	0,54	No
ENSMUSG00000046962	3,86E-02	5,296601529	2,92E-01	5,89E-01	8,41E-01	Zbtb21	1,03	No
ENSMUSG00000046971	-4,89E-01	1,957877136	1,13E+01	7,76E-04	1,31E-02	Pla2g4f	0,71	Yes
ENSMUSG00000046975	-1,87E+00	-3,107637761	1,39E+00	2,38E-01	5,50E-01	Olfr1020	0,27	No
ENSMUSG00000046982	1,67E-01	5,334078581	5,51E+00	1,89E-02	1,46E-01	Tshz1	1,12	No
ENSMUSG00000046985	6,85E-02	5,650645169	1,01E+00	3,16E-01	6,16E-01	Tapt1	1,05	No
ENSMUSG00000046991	1,79E+00	-3,033666867	1,28E+00	2,58E-01	5,50E-01	Wdr27	3,46	No
ENSMUSG00000046994	2,01E-01	3,70181115	4,54E+00	3,31E-02	2,13E-01	Mars2	1,15	No
ENSMUSG00000046997	1,79E-01	3,833078976	3,78E+00	5,20E-02	2,78E-01	Spsb4	1,13	No
ENSMUSG00000046999	-5,49E-01	-0,141212289	3,56E+00	5,92E-02	3,01E-01	1110032F04Rik	0,68	No
ENSMUSG00000047003	4,04E-02	2,738360358	1,16E-01	7,34E-01	9,23E-01	Zfp41	1,03	No
ENSMUSG00000047013	2,64E+00	-2,896137133	2,70E+00	1,01E-01	3,92E-01	Fbxo41	6,24	No

ENSMUSG00000047021	1,82E+00	-3,033661385	1,31E+00	2,52E-01	5,50E-01	Cfap65	3,54	No
ENSMUSG00000047022	-1,37E-01	0,324100651	2,76E-01	6,00E-01	8,46E-01	Mipol1	0,91	No
ENSMUSG00000047026	-1,98E-02	-3,033655952	2,83E-11	1,00E+00	1,00E+00	Acsm4	0,99	No
ENSMUSG00000047030	-7,74E-02	5,884602937	1,36E+00	2,43E-01	5,50E-01	Spata2	0,95	No
ENSMUSG00000047036	-1,23E-01	6,81019408	4,00E+00	4,55E-02	2,54E-01	Zfp445	0,92	No
ENSMUSG00000047037	1,40E-01	4,026807216	2,50E+00	1,14E-01	4,16E-01	Nipa1	1,10	No
ENSMUSG00000047040	3,24E-01	-2,448647108	1,07E-01	7,44E-01	9,28E-01	Prr15l	1,25	No
ENSMUSG00000047044	2,00E-01	3,373928637	3,83E+00	5,03E-02	2,72E-01	D030056L22Rik	1,15	No
ENSMUSG00000047048	-5,78E-02	-2,896131211	2,23E-03	9,62E-01	1,00E+00	Olfr432	0,96	No
ENSMUSG00000047053	-1,27E+00	-3,146104694	7,49E-01	3,87E-01	6,87E-01	Teddm1a	0,42	No
ENSMUSG00000047061	-1,15E+00	-2,963255048	5,80E-01	4,46E-01	7,37E-01	Gm9817	0,45	No
ENSMUSG00000047067	1,48E-01	2,091222747	1,01E+00	3,14E-01	6,14E-01	Dusp28	1,11	No
ENSMUSG00000047084	-9,79E-02	3,439655028	9,48E-01	3,30E-01	6,31E-01	Ngrn	0,93	No
ENSMUSG00000047085	-1,60E+00	-1,600630114	7,95E+00	4,81E-03	5,44E-02	Lrrc4b	0,33	No
ENSMUSG00000047090	-6,95E-03	5,440392853	9,82E-03	9,21E-01	1,00E+00	Tmem198b	1,00	No
ENSMUSG00000047098	-3,72E-02	5,2171315	2,75E-01	6,00E-01	8,46E-01	Rnf31	0,97	No
ENSMUSG00000047104	1,87E+00	-3,03364371	1,36E+00	2,43E-01	5,50E-01	Pbp2	3,66	No
ENSMUSG00000047108	-1,52E+00	-2,226207792	3,34E+00	6,77E-02	3,28E-01	Dnajb7	0,35	No
ENSMUSG00000047115	8,17E-02	0,948119829	1,51E-01	6,98E-01	9,04E-01	Fam221a	1,06	No
ENSMUSG00000047117	-2,31E-01	-0,66880713	3,49E-01	5,54E-01	8,20E-01	Ankdd1b	0,85	No
ENSMUSG00000047123	2,84E-01	5,027776505	1,49E+01	1,13E-04	2,69E-03	Ticam1	1,22	Yes
ENSMUSG00000047126	7,23E-02	9,568921943	1,64E+00	2,00E-01	5,50E-01	Cltc	1,05	No
ENSMUSG00000047139	8,57E-02	8,778767272	2,25E+00	1,34E-01	4,60E-01	Cd24a	1,06	No
ENSMUSG00000047141	-4,26E-02	4,876005051	3,21E-01	5,71E-01	8,31E-01	Zfp654	0,97	No
ENSMUSG00000047143	-5,01E-01	3,842638647	3,11E+01	2,47E-08	1,58E-06	Dmrta2	0,71	Yes
ENSMUSG00000047146	5,28E-01	-0,879928814	1,59E+00	2,08E-01	5,50E-01	Tet1	1,44	No
ENSMUSG00000047150	-7,91E-01	-1,963192918	1,38E+00	2,40E-01	5,50E-01	1700001C19Rik	0,58	No
ENSMUSG00000047153	-2,68E-01	5,917430242	1,68E+01	4,18E-05	1,17E-03	Khnyn	0,83	Yes
ENSMUSG00000047155	-3,83E-02	-3,107651936	-7,91E-11	1,00E+00	1,00E+00	Cyp4x1	0,97	No
ENSMUSG00000047180	-1,83E-01	6,889851229	8,97E+00	2,74E-03	3,58E-02	Neurl3	0,88	Yes
ENSMUSG00000047181	-3,02E-01	0,957285487	2,28E+00	1,31E-01	4,55E-01	Samd14	0,81	No
ENSMUSG00000047182	-1,37E-01	-0,126846021	2,09E-01	6,47E-01	8,75E-01	Irs3	0,91	No

ENSMUSG00000047187	5,45E-02	6,054701193	6,99E-01	4,03E-01	7,01E-01	Rab2a	1,04	No
ENSMUSG00000047193	1,70E-01	3,69882361	3,17E+00	7,52E-02	3,49E-01	Dync2h1	1,12	No
ENSMUSG00000047197	-6,17E-02	-2,311147019	6,15E-03	9,37E-01	1,00E+00	Gjd3	0,96	No
ENSMUSG00000047205	1,45E-01	3,02192611	1,64E+00	2,01E-01	5,50E-01	Dusp18	1,11	No
ENSMUSG00000047213	8,16E-02	6,799502238	1,76E+00	1,84E-01	5,48E-01	Ythdf3	1,06	No
ENSMUSG00000047215	-8,37E-02	4,351300407	1,03E+00	3,11E-01	6,11E-01	Rpl9	0,94	No
ENSMUSG00000047220	3,50E+00	-2,655169204	5,32E+00	2,11E-02	1,56E-01	Ccdc36	11,32	No
ENSMUSG00000047221	7,44E-02	2,019499709	2,59E-01	6,10E-01	8,52E-01	Fam185a	1,05	No
ENSMUSG00000047222	4,57E-01	6,474030453	5,16E+01	6,66E-13	8,32E-11	Rnase2a	1,37	Yes
ENSMUSG00000047227	-1,60E-01	0,778824879	5,30E-01	4,67E-01	7,55E-01	Gm527	0,89	No
ENSMUSG00000047228	-3,19E+00	-2,963221167	4,28E+00	3,87E-02	2,33E-01	A2ml1	0,11	No
ENSMUSG00000047246	-2,44E-01	2,417085829	3,45E+00	6,34E-02	3,14E-01	Hist1h2be	0,84	No
ENSMUSG00000047248	1,22E-01	5,250535488	2,91E+00	8,81E-02	3,79E-01	C2cd3	1,09	No
ENSMUSG00000047250	-3,70E-01	7,791427112	3,97E+01	3,01E-10	2,66E-08	Ptgs1	0,77	Yes
ENSMUSG00000047257	-2,70E+00	-2,963266517	2,89E+00	8,90E-02	3,79E-01	Prss45	0,15	No
ENSMUSG00000047260	7,98E-03	5,147202801	1,20E-02	9,13E-01	1,00E+00	Emc6	1,01	No
ENSMUSG00000047264	-6,48E-02	3,891090468	5,08E-01	4,76E-01	7,64E-01	Zfp358	0,96	No
ENSMUSG00000047281	3,42E-03	2,191331897	5,84E-04	9,81E-01	1,00E+00	Sfn	1,00	No
ENSMUSG00000047284	1,06E-01	5,234951661	2,21E+00	1,37E-01	4,68E-01	Neurl4	1,08	No
ENSMUSG00000047286	-1,33E+00	-2,770632423	1,11E+00	2,92E-01	5,87E-01	Olfr370	0,40	No
ENSMUSG00000047293	-2,21E-01	3,995901734	6,25E+00	1,24E-02	1,10E-01	Gpr15	0,86	No
ENSMUSG00000047295	-3,83E-02	-3,107651936	-7,91E-11	1,00E+00	1,00E+00	Smgc	0,97	No
ENSMUSG00000047298	1,87E+00	-3,107639691	1,38E+00	2,40E-01	5,50E-01	Kcnv2	3,67	No
ENSMUSG00000047342	-1,11E+00	-1,485197198	4,31E+00	3,79E-02	2,31E-01	Zfp286	0,46	No
ENSMUSG00000047343	-1,71E-01	-1,522575291	9,24E-02	7,61E-01	9,37E-01	Mettl21c	0,89	No
ENSMUSG00000047347	6,00E-02	2,457765857	2,07E-01	6,49E-01	8,75E-01	Tdg-ps	1,04	No
ENSMUSG00000047361	3,14E-02	-3,107617333	-7,69E-10	1,00E+00	1,00E+00	Gm973	1,02	No
ENSMUSG00000047363	-1,35E+00	-2,832034248	1,15E+00	2,84E-01	5,81E-01	Cstad	0,39	No
ENSMUSG00000047368	4,99E-02	4,855821879	4,33E-01	5,11E-01	7,94E-01	Abhd17b	1,04	No
ENSMUSG00000047369	9,28E-02	2,191435648	4,28E-01	5,13E-01	7,95E-01	Dnah14	1,07	No
ENSMUSG00000047370	2,19E-01	4,589768847	7,49E+00	6,20E-03	6,58E-02	Gm7367	1,16	No
ENSMUSG00000047371	9,22E-02	3,508185982	8,85E-01	3,47E-01	6,48E-01	Zfp768	1,07	No

ENSMUSG00000047379	1,57E-02	4,162194093	3,35E-02	8,55E-01	9,92E-01	B4gat1	1,01	No
ENSMUSG00000047388	-4,79E-01	6,735042065	6,06E+01	7,12E-15	1,08E-12	Atmin	0,72	Yes
ENSMUSG00000047394	1,85E+00	-3,107657832	1,35E+00	2,46E-01	5,50E-01	Odf3b	3,61	No
ENSMUSG00000047407	4,13E-02	6,119436205	4,04E-01	5,25E-01	8,02E-01	Tgif1	1,03	No
ENSMUSG00000047409	1,37E-01	4,077684726	2,36E+00	1,25E-01	4,41E-01	Ctdspl	1,10	No
ENSMUSG00000047412	3,53E-02	5,218439061	2,43E-01	6,22E-01	8,58E-01	Zbtb44	1,02	No
ENSMUSG00000047414	-4,40E-02	3,667856863	2,22E-01	6,38E-01	8,68E-01	Flrt2	0,97	No
ENSMUSG00000047415	2,29E-02	5,637213548	1,16E-01	7,33E-01	9,23E-01	Gpr68	1,02	No
ENSMUSG00000047417	-3,77E-03	6,386527486	3,52E-03	9,53E-01	1,00E+00	Rexo1	1,00	No
ENSMUSG00000047419	-3,92E-01	-2,165734894	3,19E-01	5,72E-01	8,32E-01	Cmya5	0,76	No
ENSMUSG00000047423	-1,39E-01	4,976344393	3,53E+00	6,03E-02	3,04E-01	AI837181	0,91	No
ENSMUSG00000047428	3,94E-01	-1,31114327	6,36E-01	4,25E-01	7,19E-01	Dlk2	1,31	No
ENSMUSG00000047434	2,94E-02	5,152202492	1,65E-01	6,85E-01	8,96E-01	Xxylt1	1,02	No
ENSMUSG00000047443	2,11E-01	-0,954535004	2,00E-01	6,55E-01	8,79E-01	Erfe	1,16	No
ENSMUSG00000047446	1,23E-02	3,079478168	1,26E-02	9,11E-01	1,00E+00	Arl4a	1,01	No
ENSMUSG00000047454	-1,28E-01	4,309686158	2,39E+00	1,22E-01	4,36E-01	Gphn	0,91	No
ENSMUSG00000047459	-4,63E-02	6,019124613	5,03E-01	4,78E-01	7,66E-01	Dynlrb1	0,97	No
ENSMUSG00000047462	8,98E-02	0,666635272	1,39E-01	7,09E-01	9,09E-01	A530099J19Rik	1,06	No
ENSMUSG00000047466	-4,09E-02	4,292382601	2,41E-01	6,24E-01	8,58E-01	8030462N17Rik	0,97	No
ENSMUSG00000047473	-6,81E-02	-1,929261663	9,72E-03	9,21E-01	1,00E+00	Zfp30	0,95	No
ENSMUSG00000047495	-6,03E-02	-2,600713624	1,58E-03	9,68E-01	1,00E+00	Dlgap2	0,96	No
ENSMUSG00000047496	-4,72E-01	3,211066832	2,06E+01	5,68E-06	2,03E-04	Rnf152	0,72	Yes
ENSMUSG00000047497	-1,89E+00	-3,033648118	1,42E+00	2,33E-01	5,50E-01	Adamts12	0,27	No
ENSMUSG00000047501	-1,94E+00	-3,107619243	1,50E+00	2,21E-01	5,50E-01	Cldn4	0,26	No
ENSMUSG00000047502	-1,24E+00	-2,600667663	1,46E+00	2,27E-01	5,50E-01	Mroh7	0,42	No
ENSMUSG00000047507	1,30E-01	0,344983184	2,40E-01	6,25E-01	8,59E-01	Baiap3	1,09	No
ENSMUSG00000047508	4,14E-01	-2,655198619	1,34E-01	7,14E-01	9,12E-01	Mbd3l2	1,33	No
ENSMUSG00000047509	1,99E-01	3,27673066	3,67E+00	5,54E-02	2,88E-01	Gm6776	1,15	No
ENSMUSG00000047511	3,01E-01	-2,401419708	9,23E-02	7,61E-01	9,37E-01	Olfr1396	1,23	No
ENSMUSG00000047514	3,04E-02	6,415785401	2,32E-01	6,30E-01	8,62E-01	Tspyl1	1,02	No
ENSMUSG00000047515	1,42E-01	2,488522006	1,19E+00	2,75E-01	5,71E-01	BC049715	1,10	No
ENSMUSG00000047517	-1,89E+00	-3,1076441	1,42E+00	2,33E-01	5,50E-01	Dmbt1	0,27	No

ENSMUSG00000047518	6,87E-01	-2,896156707	2,56E-01	6,13E-01	8,53E-01	Slfnl1	1,61	No
ENSMUSG00000047528	-1,48E-01	1,783888657	8,56E-01	3,55E-01	6,56E-01	Als2cr12	0,90	No
ENSMUSG00000047534	3,16E-01	4,494388161	1,53E+01	8,94E-05	2,21E-03	Mis18bp1	1,24	Yes
ENSMUSG00000047539	9,35E-02	5,982411215	2,02E+00	1,55E-01	4,99E-01	Fbxo28	1,07	No
ENSMUSG00000047547	1,31E-01	5,608119835	3,69E+00	5,49E-02	2,87E-01	Cltb	1,10	No
ENSMUSG00000047554	1,69E-01	4,765970596	4,78E+00	2,87E-02	1,93E-01	Tmem41b	1,12	No
ENSMUSG00000047557	-6,45E-02	4,942090112	7,55E-01	3,85E-01	6,86E-01	Lxn	0,96	No
ENSMUSG00000047562	1,88E+00	-2,963229875	1,37E+00	2,42E-01	5,50E-01	Mmp10	3,68	No
ENSMUSG00000047565	-5,99E-02	5,532068312	7,56E-01	3,85E-01	6,86E-01	Acot10	0,96	No
ENSMUSG00000047583	1,87E-01	1,511656567	1,17E+00	2,79E-01	5,75E-01	Tyw3	1,14	No
ENSMUSG00000047586	1,55E-01	1,499265726	7,29E-01	3,93E-01	6,93E-01	Nccrp1	1,11	No
ENSMUSG00000047591	5,79E-01	-0,333266586	2,62E+00	1,05E-01	3,97E-01	Mafa	1,49	No
ENSMUSG00000047592	-2,98E-01	1,936920323	3,71E+00	5,40E-02	2,84E-01	Nxpe5	0,81	No
ENSMUSG00000047603	1,76E-01	1,977652035	1,42E+00	2,34E-01	5,50E-01	Zfp235	1,13	No
ENSMUSG00000047604	1,82E-01	2,216892298	1,68E+00	1,95E-01	5,50E-01	Frat2	1,13	No
ENSMUSG00000047606	4,51E-01	-2,600686338	1,58E-01	6,91E-01	8,99E-01	Ankrd34c	1,37	No
ENSMUSG00000047613	3,25E-02	4,229301159	1,46E-01	7,02E-01	9,06E-01	A430005L14Rik	1,02	No
ENSMUSG00000047617	-1,07E-01	2,688066493	7,53E-01	3,86E-01	6,87E-01	Paxx	0,93	No
ENSMUSG00000047631	-2,94E-01	1,199691918	2,27E+00	1,32E-01	4,57E-01	Apof	0,82	No
ENSMUSG00000047632	-1,12E-01	1,371963123	3,75E-01	5,40E-01	8,10E-01	Fgfbp3	0,93	No
ENSMUSG00000047635	2,56E-01	3,256871383	5,97E+00	1,45E-02	1,23E-01	2810006K23Rik	1,19	No
ENSMUSG00000047636	-7,38E-02	-3,033659033	2,66E-03	9,59E-01	1,00E+00	Cdcp2	0,95	No
ENSMUSG00000047638	-5,64E-01	-2,600712266	2,49E-01	6,18E-01	8,55E-01	Nr1h4	0,68	No
ENSMUSG00000047642	6,84E-01	-2,896181084	2,54E-01	6,14E-01	8,53E-01	D930020B18Rik	1,61	No
ENSMUSG00000047643	8,25E-01	0,412248272	9,91E+00	1,64E-03	2,39E-02	Gm5454	1,77	Yes
ENSMUSG00000047648	1,91E-01	4,079835661	4,74E+00	2,95E-02	1,96E-01	Fbxo30	1,14	No
ENSMUSG00000047649	1,88E-01	4,215922371	4,84E+00	2,78E-02	1,88E-01	Cd3eap	1,14	No
ENSMUSG00000047652	3,14E-02	-3,107617333	-7,69E-10	1,00E+00	1,00E+00	Pea15b	1,02	No
ENSMUSG00000047654	1,62E-01	0,105370244	3,53E-01	5,53E-01	8,19E-01	Tssk6	1,12	No
ENSMUSG00000047656	-1,37E-01	2,962052482	1,49E+00	2,22E-01	5,50E-01	Trpt1	0,91	No
ENSMUSG00000047657	7,82E-02	4,645666701	9,94E-01	3,19E-01	6,19E-01	Crry-ps	1,06	No
ENSMUSG00000047669	-1,42E-01	2,534350999	1,14E+00	2,85E-01	5,82E-01	Msl3l2	0,91	No

ENSMUSG00000047671	6,54E-01	0,955259853	8,48E+00	3,59E-03	4,38E-02	Tctex1d4	1,57	Yes
ENSMUSG00000047675	-1,55E-01	5,41455982	4,99E+00	2,55E-02	1,77E-01	Rps8	0,90	No
ENSMUSG00000047676	-2,25E-01	9,511514785	1,59E+01	6,72E-05	1,74E-03	Rpsa-ps10	0,86	Yes
ENSMUSG00000047692	-1,23E+00	-3,146117124	7,12E-01	3,99E-01	6,98E-01	4930533K18Rik	0,42	No
ENSMUSG00000047702	-1,34E+00	-2,831974452	1,12E+00	2,89E-01	5,85E-01	Olfr1388	0,39	No
ENSMUSG00000047710	-4,76E-02	5,10675099	4,27E-01	5,13E-01	7,95E-01	Champ1	0,97	No
ENSMUSG00000047712	2,81E-01	2,486374463	4,66E+00	3,08E-02	2,02E-01	Ust	1,22	No
ENSMUSG00000047714	2,50E-02	5,626935797	1,34E-01	7,14E-01	9,12E-01	Ppp1r2	1,02	No
ENSMUSG00000047716	-5,20E-02	-2,711697956	1,85E-03	9,66E-01	1,00E+00	Olfr736	0,96	No
ENSMUSG00000047719	8,89E-02	3,457333751	7,64E-01	3,82E-01	6,83E-01	Ubiad1	1,06	No
ENSMUSG00000047720	1,87E+00	-3,107639691	1,38E+00	2,40E-01	5,50E-01	4922502D21Rik	3,67	No
ENSMUSG00000047721	7,76E-03	5,27300147	1,18E-02	9,14E-01	1,00E+00	Bola2	1,01	No
ENSMUSG00000047730	-2,98E-01	-2,683143414	7,55E-02	7,83E-01	9,50E-01	Fcgbp	0,81	No
ENSMUSG00000047731	2,08E-02	6,801935906	1,14E-01	7,36E-01	9,24E-01	Wbp1l	1,01	No
ENSMUSG00000047733	-4,22E-01	-1,289483744	7,05E-01	4,01E-01	6,99E-01	Tmem262	0,75	No
ENSMUSG00000047735	4,11E-01	6,493385922	4,26E+01	6,63E-11	6,43E-09	Samd9l	1,33	Yes
ENSMUSG00000047746	-1,53E-01	0,210265965	3,43E-01	5,58E-01	8,22E-01	Fbxo40	0,90	No
ENSMUSG00000047747	4,13E-01	4,718263835	2,81E+01	1,13E-07	6,24E-06	Rnf150	1,33	Yes
ENSMUSG00000047749	-8,66E-02	1,981724698	3,40E-01	5,60E-01	8,23E-01	Zc3hav1l	0,94	No
ENSMUSG00000047751	-1,60E+00	-1,929140222	5,78E+00	1,62E-02	1,32E-01	Utf1	0,33	No
ENSMUSG00000047759	5,93E-01	1,529775441	1,09E+01	9,73E-04	1,58E-02	Hs3st3a1	1,51	Yes
ENSMUSG00000047766	1,15E-01	2,708469689	8,98E-01	3,43E-01	6,44E-01	Lrrc49	1,08	No
ENSMUSG00000047767	-1,46E-01	3,330317457	1,98E+00	1,59E-01	5,07E-01	Atg16l2	0,90	No
ENSMUSG00000047777	4,29E-02	3,457057317	1,93E-01	6,60E-01	8,82E-01	Phf13	1,03	No
ENSMUSG00000047786	-1,26E+00	-2,600695413	1,52E+00	2,18E-01	5,50E-01	Lix1	0,42	No
ENSMUSG00000047787	-2,26E+00	-2,655083267	4,17E+00	4,11E-02	2,40E-01	Flrt1	0,21	No
ENSMUSG00000047789	9,41E-02	5,597794084	1,90E+00	1,68E-01	5,23E-01	Slc38a9	1,07	No
ENSMUSG00000047793	-5,74E-01	4,639288335	5,53E+01	1,03E-13	1,42E-11	Sned1	0,67	Yes
ENSMUSG00000047798	3,29E-01	8,346186453	3,24E+01	1,27E-08	8,66E-07	Cd300lf	1,26	Yes
ENSMUSG00000047804	2,08E-01	5,549923848	9,22E+00	2,39E-03	3,23E-02	Akap10	1,15	Yes
ENSMUSG00000047807	1,78E+00	-2,963287612	1,28E+00	2,58E-01	5,50E-01	9330154K18Rik	3,44	No
ENSMUSG00000047810	-1,66E-01	7,71655487	8,04E+00	4,58E-03	5,23E-02	Ccdc88b	0,89	No

ENSMUSG00000047819	2,38E-01	-2,355508396	7,14E-02	7,89E-01	9,54E-01	Tigd4	1,18	No
ENSMUSG00000047821	4,48E-02	4,044128022	2,62E-01	6,09E-01	8,51E-01	Trim16	1,03	No
ENSMUSG00000047822	-5,69E-01	-1,879929364	8,01E-01	3,71E-01	6,72E-01	Angptl8	0,67	No
ENSMUSG00000047824	-1,06E-02	6,367337643	2,81E-02	8,67E-01	9,99E-01	Pygo2	0,99	No
ENSMUSG00000047832	1,09E-01	5,619541716	2,55E+00	1,10E-01	4,07E-01	Cdca4	1,08	No
ENSMUSG00000047841	-1,04E+00	-1,600521623	3,86E+00	4,95E-02	2,69E-01	Fndc11	0,49	No
ENSMUSG00000047842	4,99E-01	3,179995233	2,14E+01	3,82E-06	1,44E-04	Diras2	1,41	Yes
ENSMUSG00000047843	-3,79E-02	5,647556201	3,13E-01	5,76E-01	8,34E-01	Bri3	0,97	No
ENSMUSG00000047854	6,44E-01	-1,448757595	1,60E+00	2,06E-01	5,50E-01	Stx19	1,56	No
ENSMUSG00000047861	-2,73E-02	-2,963256629	3,40E-04	9,85E-01	1,00E+00	Foxi1	0,98	No
ENSMUSG00000047866	-4,67E-02	5,836644245	4,92E-01	4,83E-01	7,70E-01	Lonp2	0,97	No
ENSMUSG00000047867	-1,48E+00	-0,68299154	1,49E+01	1,16E-04	2,75E-03	Gimap6	0,36	Yes
ENSMUSG00000047875	-3,85E-01	4,392550893	2,28E+01	1,84E-06	7,56E-05	Gpr157	0,77	Yes
ENSMUSG00000047878	-3,23E-01	-0,627579844	6,91E-01	4,06E-01	7,02E-01	A4galt	0,80	No
ENSMUSG00000047879	1,08E-01	6,322158827	2,88E+00	8,99E-02	3,80E-01	Usp14	1,08	No
ENSMUSG00000047880	-4,55E-01	-0,136378602	2,02E+00	1,55E-01	4,98E-01	Cxcr5	0,73	No
ENSMUSG00000047881	1,94E-01	5,487996377	7,74E+00	5,39E-03	5,91E-02	Rell1	1,14	No
ENSMUSG00000047884	4,47E-01	-2,711719153	1,56E-01	6,93E-01	9,01E-01	Klk9	1,36	No
ENSMUSG00000047888	-1,17E-02	5,975662856	3,16E-02	8,59E-01	9,95E-01	Tnrc6b	0,99	No
ENSMUSG00000047889	-1,94E+00	-3,107619243	1,50E+00	2,21E-01	5,50E-01	Serpinb6d	0,26	No
ENSMUSG00000047898	-7,17E-01	0,412595752	8,16E+00	4,28E-03	4,97E-02	Ccr4	0,61	Yes
ENSMUSG00000047904	1,20E-01	-2,146136618	2,85E-02	8,66E-01	9,99E-01	Sstr2	1,09	No
ENSMUSG00000047905	-1,22E-01	6,137533829	3,56E+00	5,90E-02	3,01E-01	Gm8566	0,92	No
ENSMUSG00000047907	-3,83E-02	-3,107651936	-7,91E-11	1,00E+00	1,00E+00	Tshz2	0,97	No
ENSMUSG00000047909	-1,05E-02	5,683568456	2,43E-02	8,76E-01	1,00E+00	Ankrd16	0,99	No
ENSMUSG00000047910	-1,87E+00	-3,107637761	1,39E+00	2,38E-01	5,50E-01	Pcdhb16	0,27	No
ENSMUSG00000047911	3,04E-01	-1,289566545	3,43E-01	5,58E-01	8,22E-01	Npm2	1,23	No
ENSMUSG00000047921	6,71E-02	5,259393196	8,84E-01	3,47E-01	6,48E-01	Trappc9	1,05	No
ENSMUSG00000047938	-1,89E+00	-2,896152932	1,43E+00	2,31E-01	5,50E-01	4930483J18Rik	0,27	No
ENSMUSG00000047940	-1,87E+00	-3,107637761	1,39E+00	2,38E-01	5,50E-01	Stpg2	0,27	No
ENSMUSG00000047945	-6,73E-01	7,329690175	1,27E+02	1,90E-29	9,98E-27	Marcksl1	0,63	Yes
ENSMUSG00000047953	-6,85E-02	-1,770609041	1,21E-02	9,13E-01	1,00E+00	Gp5	0,95	No

ENSMUSG00000047959	4,23E-01	6,165460385	4,17E+01	1,05E-10	9,95E-09	Kcna3	1,34	Yes
ENSMUSG00000047963	-2,64E-01	2,911831823	5,67E+00	1,73E-02	1,37E-01	Stbd1	0,83	No
ENSMUSG00000047965	-1,46E-01	6,823743159	5,63E+00	1,76E-02	1,39E-01	Rpl9-ps7	0,90	No
ENSMUSG00000047976	9,01E-03	-3,107646452	-5,16E-10	1,00E+00	1,00E+00	Kcna1	1,01	No
ENSMUSG00000047977	1,87E+00	-3,03364371	1,36E+00	2,43E-01	5,50E-01	Synb	3,66	No
ENSMUSG00000047986	-4,77E-01	-1,485163983	7,93E-01	3,73E-01	6,74E-01	Palm3	0,72	No
ENSMUSG00000047988	6,18E-01	0,425449926	5,90E+00	1,51E-02	1,27E-01	4933428G20Rik	1,53	No
ENSMUSG00000047989	2,35E-02	5,353549033	1,12E-01	7,38E-01	9,26E-01	Ino80c	1,02	No
ENSMUSG00000047990	1,89E+00	-3,033614598	1,38E+00	2,40E-01	5,50E-01	C2cd4a	3,70	No
ENSMUSG00000047992	-8,15E-01	-2,770593901	3,65E-01	5,45E-01	8,14E-01	Fam69c	0,57	No
ENSMUSG00000048000	4,66E-02	6,100249697	5,17E-01	4,72E-01	7,60E-01	Gigyf2	1,03	No
ENSMUSG00000048001	-2,38E+00	-3,033624832	2,22E+00	1,36E-01	4,66E-01	Hes5	0,19	No
ENSMUSG00000048003	-1,94E+00	-3,107619243	1,50E+00	2,21E-01	5,50E-01	Catsper4	0,26	No
ENSMUSG00000048012	1,81E-01	1,567228184	1,17E+00	2,80E-01	5,77E-01	Zfp473	1,13	No
ENSMUSG00000048022	-3,34E-01	-1,770673236	3,11E-01	5,77E-01	8,35E-01	Tmem229a	0,79	No
ENSMUSG00000048027	-2,03E-01	3,544255979	4,43E+00	3,52E-02	2,22E-01	Rgmb	0,87	No
ENSMUSG00000048029	-1,96E-01	0,952933245	8,19E-01	3,65E-01	6,66E-01	Eno4	0,87	No
ENSMUSG00000048031	-1,41E+00	-1,62751742	6,57E+00	1,04E-02	9,60E-02	Fcrl5	0,38	No
ENSMUSG00000048038	-6,67E-02	-2,655134332	3,00E-03	9,56E-01	1,00E+00	Ccdc187	0,95	No
ENSMUSG00000048039	-1,14E-02	5,268137852	2,56E-02	8,73E-01	1,00E+00	Isg20l2	0,99	No
ENSMUSG00000048058	-7,66E-02	3,608355851	6,32E-01	4,27E-01	7,21E-01	Ldlrad3	0,95	No
ENSMUSG00000048065	-3,89E-01	-1,998028588	3,33E-01	5,64E-01	8,26E-01	Cyb5r2	0,76	No
ENSMUSG00000048076	4,67E-02	8,451878554	6,58E-01	4,17E-01	7,12E-01	Arf1	1,03	No
ENSMUSG00000048078	3,13E-01	1,083238985	2,41E+00	1,21E-01	4,33E-01	Tenm4	1,24	No
ENSMUSG00000048080	1,87E+00	-3,107639691	1,38E+00	2,40E-01	5,50E-01	Olfr731	3,67	No
ENSMUSG00000048087	1,33E-01	5,513510775	3,64E+00	5,63E-02	2,91E-01	Gm4737	1,10	No
ENSMUSG00000048100	2,01E-02	4,27866891	5,75E-02	8,11E-01	9,66E-01	Taf13	1,01	No
ENSMUSG00000048106	6,71E-02	2,681807114	3,15E-01	5,74E-01	8,33E-01	4632415L05Rik	1,05	No
ENSMUSG00000048108	-2,56E-02	-0,697333831	4,67E-03	9,46E-01	1,00E+00	Tmem72	0,98	No
ENSMUSG00000048109	1,12E-01	5,334799129	2,50E+00	1,14E-01	4,17E-01	Rbm15	1,08	No
ENSMUSG00000048118	-1,13E-01	5,736304489	2,83E+00	9,28E-02	3,83E-01	Arid4a	0,92	No
ENSMUSG00000048120	1,96E-01	6,680179681	9,94E+00	1,62E-03	2,37E-02	Entpd1	1,15	Yes

ENSMUSG00000048126	-1,85E+00	-0,895903544	1,75E+01	2,84E-05	8,38E-04	Col6a3	0,28	Yes
ENSMUSG00000048138	1,86E+00	-2,963235356	1,35E+00	2,46E-01	5,50E-01	Dmrt2	3,62	No
ENSMUSG00000048142	-1,06E-01	0,892579915	2,34E-01	6,29E-01	8,62E-01	Nat8l	0,93	No
ENSMUSG00000048148	-5,35E-01	-2,31115529	4,59E-01	4,98E-01	7,83E-01	Nwd1	0,69	No
ENSMUSG00000048153	-3,67E-01	-0,896064608	8,00E-01	3,71E-01	6,72E-01	Olfr49	0,78	No
ENSMUSG00000048154	1,11E-01	7,651178304	3,54E+00	6,00E-02	3,03E-01	Kmt2d	1,08	No
ENSMUSG00000048163	-5,77E-02	9,346983649	1,04E+00	3,08E-01	6,07E-01	Selplg	0,96	No
ENSMUSG00000048164	7,57E-02	7,243934334	1,59E+00	2,08E-01	5,50E-01	Gm7204	1,05	No
ENSMUSG00000048170	1,03E-01	6,38516745	2,66E+00	1,03E-01	3,95E-01	Mcmbp	1,07	No
ENSMUSG00000048174	2,59E-01	2,561389099	4,20E+00	4,05E-02	2,38E-01	Tmem81	1,20	No
ENSMUSG00000048175	1,08E-01	5,299765626	2,32E+00	1,28E-01	4,49E-01	Asb8	1,08	No
ENSMUSG00000048185	-2,65E+00	-3,033633091	2,78E+00	9,55E-02	3,86E-01	Gm7075	0,16	No
ENSMUSG00000048186	-2,70E+00	-3,033614577	2,89E+00	8,93E-02	3,79E-01	Bend7	0,15	No
ENSMUSG00000048188	-1,04E-03	3,750250155	1,22E-04	9,91E-01	1,00E+00	Gm8181	1,00	No
ENSMUSG00000048191	-1,96E-01	0,804171396	6,58E-01	4,17E-01	7,12E-01	Muc6	0,87	No
ENSMUSG00000048200	3,23E-01	4,581749544	1,64E+01	5,23E-05	1,41E-03	Cracr2b	1,25	Yes
ENSMUSG00000048206	-1,90E+00	-3,033594154	1,44E+00	2,30E-01	5,50E-01	Dnajb8	0,27	No
ENSMUSG00000048215	-8,81E-02	-2,832010489	5,36E-03	9,42E-01	1,00E+00	A630023P12Rik	0,94	No
ENSMUSG00000048216	2,81E-01	4,500156753	1,23E+01	4,46E-04	8,37E-03	Gpr85	1,22	Yes
ENSMUSG00000048217	3,37E-01	2,206385274	5,76E+00	1,64E-02	1,33E-01	Nags	1,26	No
ENSMUSG00000048218	1,10E-01	3,89970093	1,44E+00	2,31E-01	5,50E-01	Amigo2	1,08	No
ENSMUSG00000048222	-3,46E-03	5,033815693	2,21E-03	9,63E-01	1,00E+00	Mfap1b	1,00	No
ENSMUSG00000048230	-3,83E-02	-3,107651936	-7,91E-11	1,00E+00	1,00E+00	Fbxo43	0,97	No
ENSMUSG00000048232	8,24E-02	2,104217652	3,35E-01	5,63E-01	8,26E-01	Fbxo10	1,06	No
ENSMUSG00000048234	1,23E-01	7,441551155	4,30E+00	3,82E-02	2,32E-01	Rnf149	1,09	No
ENSMUSG00000048240	-3,60E-01	1,023577523	2,98E+00	8,44E-02	3,73E-01	Gng7	0,78	No
ENSMUSG00000048249	-1,45E-01	5,057030811	4,00E+00	4,54E-02	2,54E-01	Crebrf	0,90	No
ENSMUSG00000048251	-3,19E+00	-2,896117911	4,30E+00	3,81E-02	2,32E-01	Bcl11b	0,11	No
ENSMUSG00000048261	2,75E-01	3,982065959	9,43E+00	2,13E-03	2,94E-02	Gm4879	1,21	Yes
ENSMUSG00000048264	9,01E-02	5,437986113	1,67E+00	1,97E-01	5,50E-01	Dip2c	1,06	No
ENSMUSG00000048271	-2,65E-02	6,594332534	1,82E-01	6,70E-01	8,88E-01	Rbm33	0,98	No
ENSMUSG00000048277	-2,54E-01	8,959571345	1,99E+01	8,16E-06	2,80E-04	Syngr2	0,84	Yes

ENSMUSG00000048279	8,94E-02	5,684504493	1,76E+00	1,85E-01	5,49E-01	Sacs	1,06	No
ENSMUSG00000048280	1,17E-01	4,016366627	1,84E+00	1,75E-01	5,33E-01	Zfp738	1,08	No
ENSMUSG00000048281	-2,95E-01	-2,863708069	6,26E-02	8,03E-01	9,62E-01	Dleu7	0,81	No
ENSMUSG00000048285	1,41E-01	4,124018271	2,61E+00	1,06E-01	3,99E-01	Frmd6	1,10	No
ENSMUSG00000048292	7,06E-01	-1,06112477	2,56E+00	1,10E-01	4,06E-01	Olfr1417	1,63	No
ENSMUSG00000048307	4,21E-02	4,906147078	3,15E-01	5,74E-01	8,34E-01	Ankrd46	1,03	No
ENSMUSG00000048310	-5,60E-02	5,361385544	6,35E-01	4,25E-01	7,20E-01	Pskh1	0,96	No
ENSMUSG00000048316	-5,67E-02	2,349340634	1,78E-01	6,73E-01	8,90E-01	Gm9823	0,96	No
ENSMUSG00000048327	1,43E-01	5,407665492	4,15E+00	4,16E-02	2,42E-01	Ckap2l	1,10	No
ENSMUSG00000048329	-1,41E-01	3,349894052	1,87E+00	1,71E-01	5,28E-01	Mfsd6l	0,91	No
ENSMUSG00000048330	-1,68E-02	-1,344290484	1,07E-03	9,74E-01	1,00E+00	Ric3	0,99	No
ENSMUSG00000048332	2,93E-01	0,685645052	1,63E+00	2,01E-01	5,50E-01	Lhfp	1,23	No
ENSMUSG00000048334	2,52E-02	2,016925046	2,86E-02	8,66E-01	9,99E-01	Gm8258	1,02	No
ENSMUSG00000048337	1,85E+00	-3,107657832	1,35E+00	2,46E-01	5,50E-01	Npy4r	3,61	No
ENSMUSG00000048347	-1,87E+00	-3,107637761	1,39E+00	2,38E-01	5,50E-01	Pcdhb18	0,27	No
ENSMUSG00000048349	-8,63E-01	4,843064389	1,30E+02	3,34E-30	1,92E-27	Pou4f1	0,55	Yes
ENSMUSG00000048351	1,31E-01	3,046620163	1,39E+00	2,38E-01	5,50E-01	Coa7	1,09	No
ENSMUSG00000048368	-4,09E-01	-2,655111062	1,70E-01	6,80E-01	8,93E-01	Omd	0,75	No
ENSMUSG00000048371	2,89E-01	3,43197936	8,24E+00	4,09E-03	4,81E-02	Pdp2	1,22	Yes
ENSMUSG00000048373	3,14E-02	-3,107617333	-7,69E-10	1,00E+00	1,00E+00	Fgfbp1	1,02	No
ENSMUSG00000048376	-7,94E-02	0,036877088	6,97E-02	7,92E-01	9,55E-01	F2r	0,95	No
ENSMUSG00000048379	7,37E-02	5,704427867	1,20E+00	2,73E-01	5,70E-01	Socs4	1,05	No
ENSMUSG00000048385	-5,63E-01	-2,548209898	2,49E-01	6,18E-01	8,55E-01	Scrt1	0,68	No
ENSMUSG00000048388	-6,42E-01	-1,24703555	1,52E+00	2,18E-01	5,50E-01	Fam171b	0,64	No
ENSMUSG00000048398	8,43E-01	-2,401430632	8,55E-01	3,55E-01	6,56E-01	Gm5065	1,79	No
ENSMUSG00000048399	1,87E+00	-3,107639691	1,38E+00	2,40E-01	5,50E-01	Tprg	3,67	No
ENSMUSG00000048402	-1,89E+00	-3,033648118	1,42E+00	2,33E-01	5,50E-01	Gli2	0,27	No
ENSMUSG00000048406	-1,11E-01	-0,522823445	8,77E-02	7,67E-01	9,40E-01	B330016D10Rik	0,93	No
ENSMUSG00000048410	-1,96E-02	4,708562008	6,42E-02	8,00E-01	9,60E-01	Zfp407	0,99	No
ENSMUSG00000048416	5,28E-01	-1,10779654	1,28E+00	2,59E-01	5,51E-01	Mlf1	1,44	No
ENSMUSG00000048424	-5,96E-02	-2,448687971	3,25E-03	9,55E-01	1,00E+00	Ranbp3l	0,96	No
ENSMUSG00000048429	3,30E-02	5,593292818	2,34E-01	6,28E-01	8,61E-01	Timm29	1,02	No

ENSMUSG00000048439	1,29E-01	3,360690397	1,56E+00	2,12E-01	5,50E-01	Nupl2	1,09	No
ENSMUSG00000048440	2,90E-02	6,361079098	2,09E-01	6,48E-01	8,75E-01	Cyp4f16	1,02	No
ENSMUSG00000048442	5,11E-01	3,162497652	2,22E+01	2,45E-06	9,69E-05	Smim5	1,42	Yes
ENSMUSG00000048445	9,63E-02	2,30425333	4,84E-01	4,87E-01	7,74E-01	Ccdc57	1,07	No
ENSMUSG00000048450	3,14E-02	-3,107617333	-7,69E-10	1,00E+00	1,00E+00	Msx1	1,02	No
ENSMUSG00000048458	1,94E-01	3,789899649	4,42E+00	3,54E-02	2,23E-01	Fam212b	1,14	No
ENSMUSG00000048473	-8,06E-01	-2,831995303	3,57E-01	5,50E-01	8,17E-01	Sult6b2	0,57	No
ENSMUSG00000048478	-3,13E-01	-1,574243364	2,97E-01	5,86E-01	8,39E-01	Spata33	0,80	No
ENSMUSG00000048480	-1,23E+00	-1,740841401	3,60E+00	5,79E-02	2,97E-01	Cxcr1	0,43	No
ENSMUSG00000048481	-2,90E-01	1,692090789	3,02E+00	8,23E-02	3,69E-01	Mypop	0,82	No
ENSMUSG00000048483	2,64E+00	-2,896131192	2,70E+00	1,01E-01	3,92E-01	Zdhhc22	6,24	No
ENSMUSG00000048484	-7,79E-02	-2,548233292	6,99E-03	9,33E-01	1,00E+00	Gm7461	0,95	No
ENSMUSG00000048485	-1,94E+00	-3,107619243	1,50E+00	2,21E-01	5,50E-01	Zbtb8b	0,26	No
ENSMUSG00000048486	2,35E-01	2,333057665	3,01E+00	8,30E-02	3,70E-01	Fitm2	1,18	No
ENSMUSG00000048489	1,20E+00	-0,195861061	1,23E+01	4,64E-04	8,63E-03	8430408G22Rik	2,29	Yes
ENSMUSG00000048490	-1,77E-01	6,545439593	8,12E+00	4,39E-03	5,06E-02	Nrip1	0,88	No
ENSMUSG00000048495	-1,72E-01	3,423586758	2,85E+00	9,17E-02	3,82E-01	Tyw5	0,89	No
ENSMUSG00000048497	9,77E-02	4,236157104	1,32E+00	2,50E-01	5,50E-01	Mmgt2	1,07	No
ENSMUSG00000048498	1,01E+00	4,261986873	1,36E+02	2,04E-31	1,30E-28	Cd300e	2,02	Yes
ENSMUSG00000048502	-1,94E+00	-3,107619243	1,50E+00	2,21E-01	5,50E-01	Duxbl1	0,26	No
ENSMUSG00000048503	2,64E+00	-3,033673223	2,69E+00	1,01E-01	3,92E-01	Tmem136	6,23	No
ENSMUSG00000048520	1,37E+00	-2,655162032	1,92E+00	1,66E-01	5,19E-01	Fbxl13	2,58	No
ENSMUSG00000048521	-3,68E-01	1,616443696	5,02E+00	2,51E-02	1,75E-01	Cxcr6	0,77	No
ENSMUSG00000048528	1,22E+00	-3,146114919	6,90E-01	4,06E-01	7,02E-01	Nkx1-2	2,33	No
ENSMUSG00000048534	-3,16E-01	7,201763362	2,78E+01	1,32E-07	7,12E-06	Jaml	0,80	Yes
ENSMUSG00000048537	-1,58E-01	4,313932951	3,71E+00	5,40E-02	2,84E-01	Phldb1	0,90	No
ENSMUSG00000048538	3,54E-03	3,701200557	1,36E-03	9,71E-01	1,00E+00	Gm9826	1,00	No
ENSMUSG00000048544	-3,41E-01	-2,548220383	1,46E-01	7,03E-01	9,06E-01	5930422O12Rik	0,79	No
ENSMUSG00000048546	1,82E-01	5,740406563	7,34E+00	6,73E-03	6,99E-02	Tob2	1,13	No
ENSMUSG00000048550	1,58E-01	2,659055814	1,72E+00	1,90E-01	5,50E-01	Thnsl1	1,12	No
ENSMUSG00000048559	4,65E-01	-1,333295775	8,44E-01	3,58E-01	6,60E-01	4930555K19Rik	1,38	No
ENSMUSG00000048574	1,51E-01	4,885564886	3,97E+00	4,62E-02	2,57E-01	Ccnb1-ps	1,11	No

ENSMUSG00000048578	1,00E-01	8,87714747	3,10E+00	7,82E-02	3,57E-01	Mlec	1,07	No
ENSMUSG00000048581	-3,89E-01	2,358798465	8,74E+00	3,11E-03	3,94E-02	E130311K13Rik	0,76	Yes
ENSMUSG00000048583	-2,68E+00	-2,963200749	2,85E+00	9,14E-02	3,82E-01	Igf2	0,16	No
ENSMUSG00000048602	-1,91E+00	-2,831955981	1,45E+00	2,28E-01	5,50E-01	Morc2b	0,27	No
ENSMUSG00000048603	-2,25E-01	0,840937747	1,11E+00	2,92E-01	5,87E-01	Gm9828	0,86	No
ENSMUSG00000048612	6,38E-02	9,032122682	1,26E+00	2,62E-01	5,56E-01	Myof	1,05	No
ENSMUSG00000048617	2,34E-01	-1,81646717	1,56E-01	6,93E-01	9,01E-01	Rtbdn	1,18	No
ENSMUSG00000048620	-1,89E+00	-3,1076441	1,42E+00	2,33E-01	5,50E-01	Olfr1336	0,27	No
ENSMUSG00000048636	-2,16E+00	-2,896128696	2,57E+00	1,09E-01	4,05E-01	A730049H05Rik	0,22	No
ENSMUSG00000048644	1,99E-01	-0,54834778	3,53E-01	5,52E-01	8,19E-01	Ctxn1	1,15	No
ENSMUSG00000048647	-9,57E-01	0,358927266	1,39E+01	1,93E-04	4,23E-03	Exd1	0,51	Yes
ENSMUSG00000048652	-2,77E-02	-2,929298853	3,59E-04	9,85E-01	1,00E+00	Samd13	0,98	No
ENSMUSG00000048655	2,57E+00	-3,0336723	2,56E+00	1,10E-01	4,06E-01	Ccdc169	5,96	No
ENSMUSG00000048661	1,11E-01	4,846510605	2,12E+00	1,45E-01	4,82E-01	Lemd3	1,08	No
ENSMUSG00000048668	1,03E-01	3,871846588	1,30E+00	2,55E-01	5,50E-01	Rhno1	1,07	No
ENSMUSG00000048677	2,33E-01	5,805820364	1,21E+01	5,03E-04	9,21E-03	Tpcn2	1,18	Yes
ENSMUSG00000048696	1,64E-01	3,389931873	2,59E+00	1,07E-01	4,01E-01	Mex3d	1,12	No
ENSMUSG00000048701	-3,55E-01	6,697812141	3,33E+01	7,80E-09	5,51E-07	Ccdc6	0,78	Yes
ENSMUSG00000048703	1,82E+00	-3,033661385	1,31E+00	2,52E-01	5,50E-01	Speer4b	3,54	No
ENSMUSG00000048707	-4,33E-02	4,351175094	2,74E-01	6,01E-01	8,47E-01	Tprn	0,97	No
ENSMUSG00000048720	5,02E-02	3,004900716	2,06E-01	6,50E-01	8,76E-01	Tbc1d12	1,04	No
ENSMUSG00000048721	-7,43E-01	0,16045746	6,87E+00	8,74E-03	8,46E-02	Fndc9	0,60	No
ENSMUSG00000048731	1,49E-02	4,397559671	3,33E-02	8,55E-01	9,92E-01	Ggnbp1	1,01	No
ENSMUSG00000048732	9,16E-02	3,159787773	7,06E-01	4,01E-01	6,99E-01	Klhl11	1,07	No
ENSMUSG00000048747	1,17E+00	-3,14612376	6,40E-01	4,24E-01	7,18E-01	E130114P18Rik	2,26	No
ENSMUSG00000048752	-4,13E-02	-3,033659495	7,08E-04	9,79E-01	1,00E+00	Prss50	0,97	No
ENSMUSG00000048755	1,46E-01	3,729322848	2,47E+00	1,16E-01	4,21E-01	Mcat	1,11	No
ENSMUSG00000048756	-1,09E-02	6,50439061	3,04E-02	8,61E-01	9,96E-01	Foxo3	0,99	No
ENSMUSG00000048758	-8,14E-02	7,884026866	1,94E+00	1,64E-01	5,15E-01	Rpl29	0,95	No
ENSMUSG00000048763	-2,46E-01	-0,541684301	4,57E-01	4,99E-01	7,84E-01	Hoxb3	0,84	No
ENSMUSG00000048772	-5,97E-02	1,334460657	1,04E-01	7,47E-01	9,30E-01	Tmem53	0,96	No
ENSMUSG00000048775	1,81E+00	-3,10765737	1,28E+00	2,58E-01	5,50E-01	Serpinb13	3,49	No

ENSMUSG00000048779	5,43E-01	6,940511121	7,87E+01	7,16E-19	1,49E-16	P2ry6	1,46	Yes
ENSMUSG00000048782	9,01E-03	-3,107646452	-5,16E-10	1,00E+00	1,00E+00	Insc	1,01	No
ENSMUSG00000048787	1,48E-01	4,074162195	2,79E+00	9,49E-02	3,86E-01	Dcun1d3	1,11	No
ENSMUSG00000048794	2,34E-01	-0,102989671	6,07E-01	4,36E-01	7,29E-01	Cfap100	1,18	No
ENSMUSG00000048796	-5,44E-02	4,983964477	5,36E-01	4,64E-01	7,53E-01	Cyb561d1	0,96	No
ENSMUSG00000048799	1,01E-02	5,662495548	2,24E-02	8,81E-01	1,00E+00	Cep120	1,01	No
ENSMUSG00000048806	7,43E-01	-2,896145754	3,01E-01	5,83E-01	8,38E-01	Ifnb1	1,67	No
ENSMUSG00000048807	1,55E-01	4,797811052	4,08E+00	4,34E-02	2,48E-01	Slc35e4	1,11	No
ENSMUSG00000048824	-5,61E-01	-2,448613537	2,47E-01	6,19E-01	8,56E-01	Gm568	0,68	No
ENSMUSG00000048826	-6,81E-01	-0,344379317	4,62E+00	3,15E-02	2,06E-01	Dact2	0,62	No
ENSMUSG00000048827	-1,21E-02	3,317693171	1,34E-02	9,08E-01	1,00E+00	Pkd1l3	0,99	No
ENSMUSG00000048832	8,21E-02	5,169143366	1,31E+00	2,53E-01	5,50E-01	Vps37c	1,06	No
ENSMUSG00000048833	1,10E-01	6,051616977	2,88E+00	8,96E-02	3,80E-01	Slc39a9	1,08	No
ENSMUSG00000048834	2,71E-01	-2,548181248	7,47E-02	7,85E-01	9,51E-01	Vstm2a	1,21	No
ENSMUSG00000048852	2,20E-01	1,096109296	1,23E+00	2,67E-01	5,64E-01	Gm12185	1,16	No
ENSMUSG00000048856	-4,93E-01	0,545378755	4,41E+00	3,58E-02	2,24E-01	Slc25a47	0,71	No
ENSMUSG00000048865	-1,25E-01	8,646993554	4,80E+00	2,85E-02	1,91E-01	Arhgap30	0,92	No
ENSMUSG00000048874	6,53E-02	6,599793459	1,11E+00	2,93E-01	5,88E-01	Phf3	1,05	No
ENSMUSG00000048878	-1,84E-02	5,468591691	7,05E-02	7,91E-01	9,55E-01	Hexim1	0,99	No
ENSMUSG00000048895	-2,59E-01	1,81188105	2,84E+00	9,22E-02	3,82E-01	Cdk5r1	0,84	No
ENSMUSG00000048897	2,62E-01	7,036687738	1,87E+01	1,50E-05	4,78E-04	Zfp710	1,20	Yes
ENSMUSG00000048899	-1,11E-01	-2,832003778	8,58E-03	9,26E-01	1,00E+00	Rimkla	0,93	No
ENSMUSG00000048905	-4,12E-01	-1,669271814	4,82E-01	4,88E-01	7,75E-01	4930539E08Rik	0,75	No
ENSMUSG00000048911	-2,39E-01	5,195936369	1,08E+01	1,02E-03	1,64E-02	Rnf24	0,85	Yes
ENSMUSG00000048915	2,64E-01	0,757987486	1,32E+00	2,51E-01	5,50E-01	Efna5	1,20	No
ENSMUSG00000048916	-2,95E-01	2,890552667	6,40E+00	1,14E-02	1,03E-01	Gm5540	0,82	No
ENSMUSG00000048920	1,08E-02	5,268853655	2,30E-02	8,79E-01	1,00E+00	Fkrp	1,01	No
ENSMUSG00000048921	-6,82E-02	1,640219443	1,70E-01	6,81E-01	8,93E-01	Zfp689	0,95	No
ENSMUSG00000048922	2,70E-01	4,521086192	1,14E+01	7,18E-04	1,23E-02	Cdca2	1,21	Yes
ENSMUSG00000048924	-1,84E-02	3,116827757	2,83E-02	8,66E-01	9,99E-01	Ccdc125	0,99	No
ENSMUSG00000048930	3,91E-02	4,595187675	2,46E-01	6,20E-01	8,57E-01	Tada3	1,03	No
ENSMUSG00000048939	-1,94E+00	-3,107619243	1,50E+00	2,21E-01	5,50E-01	Atp13a5	0,26	No

ENSMUSG00000048960	1,12E+00	-2,574241512	1,20E+00	2,73E-01	5,69E-01	Prex2	2,18	No
ENSMUSG00000048965	1,82E-01	3,221192897	2,99E+00	8,36E-02	3,71E-01	Mrgpre	1,13	No
ENSMUSG00000048967	-3,40E-01	0,01220383	1,34E+00	2,47E-01	5,50E-01	Yjefn3	0,79	No
ENSMUSG00000048982	-2,65E+00	-3,033633091	2,78E+00	9,55E-02	3,86E-01	Gphb5	0,16	No
ENSMUSG00000048988	-7,93E-02	-2,185690108	1,17E-02	9,14E-01	1,00E+00	Elfn1	0,95	No
ENSMUSG00000048996	1,85E+00	-3,107657832	1,35E+00	2,46E-01	5,50E-01	Olfr1366	3,61	No
ENSMUSG00000048997	-3,21E-02	3,116421795	8,28E-02	7,74E-01	9,44E-01	Atxn7l2	0,98	No
ENSMUSG00000049001	-3,96E-01	2,669555747	1,11E+01	8,44E-04	1,41E-02	Ndnf	0,76	Yes
ENSMUSG00000049008	1,81E+00	-3,10765737	1,28E+00	2,58E-01	5,50E-01	BB014433	3,49	No
ENSMUSG00000049011	9,01E-03	-3,107646452	-5,16E-10	1,00E+00	1,00E+00	Olfr729	1,01	No
ENSMUSG00000049018	1,81E+00	-3,10765737	1,28E+00	2,58E-01	5,50E-01	Olfr368	3,49	No
ENSMUSG00000049036	-3,20E-02	-2,832035763	6,53E-04	9,80E-01	1,00E+00	Tmem121	0,98	No
ENSMUSG00000049037	2,69E-02	2,767692148	4,83E-02	8,26E-01	9,75E-01	Clec4a1	1,02	No
ENSMUSG00000049038	4,57E-01	-1,436713913	8,14E-01	3,67E-01	6,68E-01	Mterf2	1,37	No
ENSMUSG00000049044	-7,02E-01	-2,146082103	8,39E-01	3,60E-01	6,61E-01	Rapgef4	0,61	No
ENSMUSG00000049057	9,01E-03	-3,107646452	-5,16E-10	1,00E+00	1,00E+00	Olfr1257	1,01	No
ENSMUSG00000049076	3,43E-02	7,205575082	3,26E-01	5,68E-01	8,30E-01	Acap2	1,02	No
ENSMUSG00000049086	-3,12E-01	4,206067759	1,36E+01	2,21E-04	4,72E-03	Bmyc	0,81	Yes
ENSMUSG00000049090	6,21E-02	5,944247885	8,90E-01	3,45E-01	6,46E-01	Zadh2	1,04	No
ENSMUSG00000049091	2,10E-01	5,955125629	1,01E+01	1,47E-03	2,20E-02	Sephs2	1,16	Yes
ENSMUSG00000049092	-1,07E+00	-1,561065771	3,33E+00	6,79E-02	3,28E-01	Gpr137c	0,48	No
ENSMUSG00000049093	-6,64E-01	0,112060513	5,95E+00	1,47E-02	1,24E-01	Il23r	0,63	No
ENSMUSG00000049097	1,52E-01	-2,268152104	3,44E-02	8,53E-01	9,91E-01	Ankrd34a	1,11	No
ENSMUSG00000049100	-2,70E+00	-3,033614577	2,89E+00	8,93E-02	3,79E-01	Pcdh10	0,15	No
ENSMUSG00000049103	5,41E-01	9,286671702	9,04E+01	1,92E-21	5,00E-19	Ccr2	1,45	Yes
ENSMUSG00000049106	-8,48E-02	5,617426727	1,56E+00	2,12E-01	5,50E-01	Dcaf5	0,94	No
ENSMUSG00000049109	9,01E-03	-3,107646452	-5,16E-10	1,00E+00	1,00E+00	Themis	1,01	No
ENSMUSG00000049112	-1,33E+00	-2,896140992	1,10E+00	2,93E-01	5,89E-01	Oxtr	0,40	No
ENSMUSG00000049119	2,52E-01	1,445306354	1,88E+00	1,70E-01	5,26E-01	Fam110b	1,19	No
ENSMUSG00000049123	-7,32E-01	0,041183703	6,65E+00	9,93E-03	9,28E-02	Catsperg2	0,60	No
ENSMUSG00000049124	-1,36E-01	5,488675469	3,87E+00	4,93E-02	2,68E-01	Gm8186	0,91	No
ENSMUSG00000049130	-3,08E-01	4,065578747	1,16E+01	6,66E-04	1,16E-02	C5ar1	0,81	Yes

ENSMUSG00000049134	9,29E-01	-2,015758733	1,65E+00	1,99E-01	5,50E-01	Nrap	1,90	No
ENSMUSG00000049152	-5,49E-01	-2,770571758	2,36E-01	6,27E-01	8,61E-01	Ugt3a2	0,68	No
ENSMUSG00000049164	4,31E-02	5,0954889	3,47E-01	5,56E-01	8,21E-01	Zfp518a	1,03	No
ENSMUSG00000049184	-3,10E-01	2,429694023	5,78E+00	1,62E-02	1,32E-01	Purg	0,81	No
ENSMUSG00000049225	2,00E-01	4,685215346	6,55E+00	1,05E-02	9,68E-02	Pdp1	1,15	No
ENSMUSG00000049230	-2,01E-02	3,666364069	4,49E-02	8,32E-01	9,78E-01	Gm9833	0,99	No
ENSMUSG00000049231	-2,78E-01	2,868234142	5,43E+00	1,98E-02	1,50E-01	Gm12497	0,82	No
ENSMUSG00000049232	1,49E-02	4,468047982	3,39E-02	8,54E-01	9,92E-01	Tigd2	1,01	No
ENSMUSG00000049233	3,54E-02	3,377003207	1,18E-01	7,31E-01	9,21E-01	Apoo-ps	1,02	No
ENSMUSG00000049235	3,30E-02	3,836652772	1,27E-01	7,21E-01	9,16E-01	Gm7324	1,02	No
ENSMUSG00000049241	5,58E-01	-0,755613173	1,70E+00	1,92E-01	5,50E-01	Hcar1	1,47	No
ENSMUSG00000049246	-5,64E-02	-2,770641569	2,85E-03	9,57E-01	1,00E+00	Hspe1-ps2	0,96	No
ENSMUSG00000049252	-1,14E-02	-3,033635021	1,50E-05	9,97E-01	1,00E+00	Lrp1b	0,99	No
ENSMUSG00000049265	7,90E-01	-2,770673814	5,40E-01	4,62E-01	7,51E-01	Kcnk3	1,73	No
ENSMUSG00000049280	9,01E-03	-3,107646452	-5,16E-10	1,00E+00	1,00E+00	Olfr509	1,01	No
ENSMUSG00000049281	-4,19E-03	-0,755417479	9,69E-05	9,92E-01	1,00E+00	Scn3b	1,00	No
ENSMUSG00000049285	3,08E-02	1,908825382	4,04E-02	8,41E-01	9,84E-01	Mblac1	1,02	No
ENSMUSG00000049287	5,91E-02	3,123700818	3,04E-01	5,81E-01	8,37E-01	Iba57	1,04	No
ENSMUSG00000049288	-1,86E-01	3,849133426	4,09E+00	4,30E-02	2,47E-01	Lix1l	0,88	No
ENSMUSG00000049291	-1,94E+00	-3,107619243	1,50E+00	2,21E-01	5,50E-01	Prss38	0,26	No
ENSMUSG00000049295	-2,88E-01	5,040497478	1,55E+01	8,17E-05	2,05E-03	Zfp219	0,82	Yes
ENSMUSG00000049299	-5,03E-02	5,105462366	4,75E-01	4,91E-01	7,77E-01	Trappc1	0,97	No
ENSMUSG00000049300	8,99E-02	4,197341891	1,09E+00	2,96E-01	5,92E-01	Prmt6	1,06	No
ENSMUSG00000049303	7,41E-02	-1,669051419	1,47E-02	9,03E-01	1,00E+00	Syt12	1,05	No
ENSMUSG00000049305	-4,43E-02	5,234720522	3,87E-01	5,34E-01	8,07E-01	Ccdc71	0,97	No
ENSMUSG00000049307	-2,85E-01	3,797426004	9,54E+00	2,01E-03	2,81E-02	Fut4	0,82	Yes
ENSMUSG00000049313	-3,00E-01	5,978580833	2,07E+01	5,29E-06	1,90E-04	Sorl1	0,81	Yes
ENSMUSG00000049314	-3,88E-03	-3,03362684	4,02E-12	1,00E+00	1,00E+00	Fbxw13	1,00	No
ENSMUSG00000049321	2,17E-01	-1,800979483	1,21E-01	7,28E-01	9,20E-01	Zfp2	1,16	No
ENSMUSG00000049323	9,23E-02	6,900825235	2,29E+00	1,30E-01	4,53E-01	Smcr8	1,07	No
ENSMUSG00000049327	8,87E-03	6,724692346	2,07E-02	8,86E-01	1,00E+00	Kmt5a	1,01	No
ENSMUSG00000049336	-1,86E+00	-2,963227946	1,40E+00	2,36E-01	5,50E-01	Tenm2	0,28	No

ENSMUSG00000049339	7,20E-02	6,412924317	1,30E+00	2,55E-01	5,50E-01	Retreg2	1,05	No
ENSMUSG00000049350	-1,94E+00	-3,107619243	1,50E+00	2,21E-01	5,50E-01	Zg16	0,26	No
ENSMUSG00000049353	4,15E-01	-1,963284229	3,96E-01	5,29E-01	8,05E-01	Rd3	1,33	No
ENSMUSG00000049354	9,55E-02	7,663091413	2,63E+00	1,05E-01	3,97E-01	Dcaf7	1,07	No
ENSMUSG00000049357	-9,47E-01	-1,289482686	3,63E+00	5,67E-02	2,93E-01	4933408B17Rik	0,52	No
ENSMUSG00000049362	1,81E-01	0,14417751	4,29E-01	5,13E-01	7,95E-01	Olfr173	1,13	No
ENSMUSG00000049382	-3,83E-02	-3,107651936	-7,91E-11	1,00E+00	1,00E+00	Krt8	0,97	No
ENSMUSG00000049396	3,16E-02	2,286022229	5,34E-02	8,17E-01	9,70E-01	Gemin4	1,02	No
ENSMUSG00000049401	-4,20E-02	6,385674986	4,42E-01	5,06E-01	7,91E-01	Ogfr	0,97	No
ENSMUSG00000049404	4,03E-01	1,045030025	3,86E+00	4,96E-02	2,69E-01	Rarres1	1,32	No
ENSMUSG00000049409	3,61E-01	0,508451167	2,16E+00	1,41E-01	4,75E-01	Prokr1	1,28	No
ENSMUSG00000049411	1,84E-01	3,071768483	2,75E+00	9,71E-02	3,89E-01	Tmem241	1,14	No
ENSMUSG00000049414	2,28E-01	2,262474044	2,67E+00	1,02E-01	3,94E-01	Gm5417	1,17	No
ENSMUSG00000049420	9,01E-03	-3,107646452	-5,16E-10	1,00E+00	1,00E+00	Tmem200a	1,01	No
ENSMUSG00000049421	8,09E-02	4,381890366	9,96E-01	3,18E-01	6,19E-01	Zfp260	1,06	No
ENSMUSG00000049422	1,74E-01	4,405781742	4,46E+00	3,46E-02	2,19E-01	Chchd10	1,13	No
ENSMUSG00000049436	7,44E-01	-2,963286135	3,02E-01	5,83E-01	8,38E-01	Upk1b	1,67	No
ENSMUSG00000049439	1,67E-01	4,237657739	3,86E+00	4,94E-02	2,68E-01	Cyp20a1	1,12	No
ENSMUSG00000049460	1,24E-01	3,533595518	1,58E+00	2,08E-01	5,50E-01	Gm8396	1,09	No
ENSMUSG00000049470	2,10E-02	7,413832733	1,24E-01	7,25E-01	9,18E-01	Aff4	1,01	No
ENSMUSG00000049476	1,81E+00	-3,10765737	1,28E+00	2,58E-01	5,50E-01	1700104B16Rik	3,49	No
ENSMUSG00000049477	-1,15E-03	3,259313039	1,16E-04	9,91E-01	1,00E+00	Gm8508	1,00	No
ENSMUSG00000049482	4,57E-02	5,565877377	4,44E-01	5,05E-01	7,90E-01	Ctu2	1,03	No
ENSMUSG00000049488	1,88E-01	2,50629894	2,15E+00	1,43E-01	4,77E-01	Tmem67	1,14	No
ENSMUSG00000049489	-9,03E-02	3,522593957	8,17E-01	3,66E-01	6,67E-01	Fam58b	0,94	No
ENSMUSG00000049491	4,50E-02	-0,079382797	2,28E-02	8,80E-01	1,00E+00	Slc36a3	1,03	No
ENSMUSG00000049493	-2,75E+00	-2,896112716	2,99E+00	8,38E-02	3,71E-01	Pls1	0,15	No
ENSMUSG00000049502	-1,51E-01	5,961723037	5,41E+00	2,00E-02	1,51E-01	Dtx3l	0,90	No
ENSMUSG00000049504	1,01E-01	5,684427618	2,22E+00	1,36E-01	4,65E-01	Proser1	1,07	No
ENSMUSG00000049515	1,03E+00	-2,226369022	1,71E+00	1,91E-01	5,50E-01	Espnl	2,04	No
ENSMUSG00000049516	-1,95E-03	5,943117675	8,76E-04	9,76E-01	1,00E+00	Spty2d1	1,00	No
ENSMUSG00000049517	-2,19E-01	5,359486421	9,69E+00	1,85E-03	2,64E-02	Rps23	0,86	Yes

ENSMUSG00000049521	1,85E+00	-3,107657832	1,35E+00	2,46E-01	5,50E-01	Cdc42ep1	3,61	No
ENSMUSG00000049526	2,26E-01	4,965203573	9,09E+00	2,57E-03	3,40E-02	Tmem202	1,17	Yes
ENSMUSG00000049532	4,05E-01	3,043421902	1,31E+01	3,02E-04	6,09E-03	Sall2	1,32	Yes
ENSMUSG00000049539	-1,20E-01	-0,627700794	1,18E-01	7,31E-01	9,21E-01	Hist1h1a	0,92	No
ENSMUSG00000049550	-6,92E-02	6,090368898	1,14E+00	2,86E-01	5,82E-01	Clip1	0,95	No
ENSMUSG00000049551	-1,36E+00	-2,896086315	1,16E+00	2,81E-01	5,78E-01	Fzd9	0,39	No
ENSMUSG00000049553	1,08E-01	5,589621959	2,48E+00	1,15E-01	4,19E-01	Polr1a	1,08	No
ENSMUSG00000049555	-5,76E-01	0,132372774	4,52E+00	3,36E-02	2,15E-01	Tmie	0,67	No
ENSMUSG00000049556	-1,23E+00	-2,600691255	1,45E+00	2,29E-01	5,50E-01	Lingo1	0,43	No
ENSMUSG00000049562	1,39E-01	4,737892322	3,25E+00	7,12E-02	3,38E-01	Ap5b1	1,10	No
ENSMUSG00000049565	-1,94E+00	-3,107619243	1,50E+00	2,21E-01	5,50E-01	Aknad1	0,26	No
ENSMUSG00000049571	-3,95E-02	-2,770627166	1,06E-03	9,74E-01	1,00E+00	Cfap46	0,97	No
ENSMUSG00000049576	-7,78E-04	1,511472287	1,96E-05	9,96E-01	1,00E+00	Zfa-ps	1,00	No
ENSMUSG00000049577	8,54E-02	3,337029024	6,60E-01	4,17E-01	7,12E-01	Zfpm1	1,06	No
ENSMUSG00000049580	1,48E-02	3,295686972	2,03E-02	8,87E-01	1,00E+00	Tsku	1,01	No
ENSMUSG00000049583	1,87E+00	-3,107639691	1,38E+00	2,40E-01	5,50E-01	Grm5	3,67	No
ENSMUSG00000049588	1,25E-01	3,105577077	1,30E+00	2,53E-01	5,50E-01	Ccdc69	1,09	No
ENSMUSG00000049598	-8,34E-01	0,477827597	1,17E+01	6,29E-04	1,11E-02	Vsig8	0,56	Yes
ENSMUSG00000049600	1,47E-01	4,346277313	3,20E+00	7,36E-02	3,45E-01	Zbtb45	1,11	No
ENSMUSG00000049604	1,85E+00	-3,107657832	1,35E+00	2,46E-01	5,50E-01	Hoxb13	3,61	No
ENSMUSG00000049606	-2,05E-02	4,731404056	7,13E-02	7,89E-01	9,54E-01	Zfp644	0,99	No
ENSMUSG00000049608	-4,12E-01	4,428172498	2,66E+01	2,54E-07	1,30E-05	Gpr55	0,75	Yes
ENSMUSG00000049612	-5,49E-01	-0,596142245	2,23E+00	1,35E-01	4,63E-01	Omg	0,68	No
ENSMUSG00000049620	-1,94E+00	-3,107619243	1,50E+00	2,21E-01	5,50E-01	Prss33	0,26	No
ENSMUSG00000049624	1,42E-01	5,229654406	3,90E+00	4,84E-02	2,64E-01	Slc17a5	1,10	No
ENSMUSG00000049625	3,35E-01	7,788391894	3,26E+01	1,16E-08	8,00E-07	Tifab	1,26	Yes
ENSMUSG00000049630	-1,90E-01	-1,72628322	1,05E-01	7,46E-01	9,29E-01	C1ql3	0,88	No
ENSMUSG00000049643	8,44E-02	4,807514339	1,22E+00	2,69E-01	5,65E-01	2310022A10Rik	1,06	No
ENSMUSG00000049648	-1,87E+00	-3,107637761	1,39E+00	2,38E-01	5,50E-01	Olfr273	0,27	No
ENSMUSG00000049649	4,57E-01	-0,529040222	1,48E+00	2,24E-01	5,50E-01	Gpr3	1,37	No
ENSMUSG00000049653	3,05E-02	-2,247052239	1,56E-03	9,69E-01	1,00E+00	Spatc1	1,02	No
ENSMUSG00000049657	3,47E-01	3,761860196	1,42E+01	1,65E-04	3,69E-03	Zbtb5	1,27	Yes

ENSMUSG00000049658	9,48E-02	5,980750694	2,08E+00	1,49E-01	4,90E-01	Bdp1	1,07	No
ENSMUSG00000049659	-1,80E-01	6,914762527	8,73E+00	3,14E-03	3,96E-02	Aftph	0,88	Yes
ENSMUSG00000049670	-1,73E-01	1,221735889	7,81E-01	3,77E-01	6,78E-01	Morn4	0,89	No
ENSMUSG00000049672	1,79E-02	4,160070271	4,45E-02	8,33E-01	9,78E-01	Zbtb14	1,01	No
ENSMUSG00000049676	-3,57E-01	3,428801366	1,32E+01	2,81E-04	5,75E-03	Catsperg1	0,78	Yes
ENSMUSG00000049680	1,39E-01	5,339894685	3,88E+00	4,89E-02	2,66E-01	Urgcp	1,10	No
ENSMUSG00000049686	1,28E-01	6,728185725	4,28E+00	3,86E-02	2,33E-01	Orai1	1,09	No
ENSMUSG00000049687	7,86E-01	0,612907944	1,10E+01	9,09E-04	1,50E-02	Fam109b	1,72	Yes
ENSMUSG00000049690	-9,00E-01	-1,627624142	3,01E+00	8,29E-02	3,70E-01	Nckap5	0,54	No
ENSMUSG00000049692	4,82E-03	-1,740889391	6,09E-05	9,94E-01	1,00E+00	Tmem239	1,00	No
ENSMUSG00000049694	1,86E+00	-3,033661846	1,35E+00	2,46E-01	5,50E-01	BC048671	3,62	No
ENSMUSG00000049699	1,22E+00	-2,655160971	9,20E-01	3,37E-01	6,38E-01	Ucn2	2,33	No
ENSMUSG00000049709	2,37E-01	3,286107278	5,05E+00	2,46E-02	1,73E-01	Nlrp10	1,18	No
ENSMUSG00000049717	2,33E-01	2,133891355	2,80E+00	9,42E-02	3,84E-01	Lig4	1,18	No
ENSMUSG00000049719	-8,28E-01	-2,070246351	1,00E+00	3,17E-01	6,18E-01	Prss46	0,56	No
ENSMUSG00000049721	-5,58E-01	-0,401199615	2,64E+00	1,04E-01	3,97E-01	Gal3st1	0,68	No
ENSMUSG00000049723	2,21E-01	13,05380738	1,57E+01	7,27E-05	1,86E-03	Mmp12	1,17	Yes
ENSMUSG00000049728	1,06E-01	3,654820511	1,25E+00	2,64E-01	5,59E-01	Zfp668	1,08	No
ENSMUSG00000049734	-7,95E-02	4,374403717	9,56E-01	3,28E-01	6,29E-01	Trex1	0,95	No
ENSMUSG00000049739	8,93E-02	5,715260066	1,77E+00	1,84E-01	5,47E-01	Zfp646	1,06	No
ENSMUSG00000049740	-1,89E+00	-3,070168098	1,43E+00	2,33E-01	5,50E-01	5330417H12Rik	0,27	No
ENSMUSG00000049741	-1,87E+00	-2,963257051	1,41E+00	2,35E-01	5,50E-01	Rxfp4	0,27	No
ENSMUSG00000049744	3,19E-01	4,52723304	1,56E+01	8,03E-05	2,02E-03	Arhgap15	1,25	Yes
ENSMUSG00000049751	-9,35E-02	5,307429442	1,73E+00	1,88E-01	5,50E-01	Rpl36al	0,94	No
ENSMUSG00000049755	3,32E-02	3,799128594	1,34E-01	7,14E-01	9,12E-01	Zfp672	1,02	No
ENSMUSG00000049758	-2,63E-02	-3,033653157	2,33E-04	9,88E-01	1,00E+00	Olfr1318	0,98	No
ENSMUSG00000049760	-7,71E-02	5,031058935	1,10E+00	2,95E-01	5,91E-01	2410015M20Rik	0,95	No
ENSMUSG00000049764	-4,20E-01	4,337560283	2,60E+01	3,45E-07	1,70E-05	Zfp280b	0,75	Yes
ENSMUSG00000049791	5,26E-01	2,81362729	1,91E+01	1,26E-05	4,12E-04	Fzd4	1,44	Yes
ENSMUSG00000049792	5,95E-02	4,378096047	5,25E-01	4,69E-01	7,57E-01	Bag5	1,04	No
ENSMUSG00000049797	1,89E+00	-3,033614598	1,38E+00	2,40E-01	5,50E-01	Olfr642	3,70	No
ENSMUSG00000049799	3,96E-01	-2,226371346	2,50E-01	6,17E-01	8,55E-01	Lrrc19	1,32	No

ENSMUSG00000049800	1,33E-01	6,232100491	4,28E+00	3,86E-02	2,33E-01	Sertad2	1,10	No
ENSMUSG00000049807	2,69E-01	4,770695801	1,21E+01	5,12E-04	9,35E-03	Arhgap23	1,21	Yes
ENSMUSG00000049811	-1,13E-01	1,828936847	5,32E-01	4,66E-01	7,54E-01	Fam161a	0,92	No
ENSMUSG00000049823	-1,39E-01	2,304407906	1,05E+00	3,06E-01	6,03E-01	Zbtb12	0,91	No
ENSMUSG00000049832	-8,95E-02	5,772850966	1,79E+00	1,81E-01	5,43E-01	Rbx1-ps	0,94	No
ENSMUSG00000049848	5,64E-02	0,412418103	4,64E-02	8,29E-01	9,77E-01	Ceacam19	1,04	No
ENSMUSG00000049858	-3,51E-01	4,697176637	2,11E+01	4,41E-06	1,62E-04	Suox	0,78	Yes
ENSMUSG00000049866	-6,61E-03	8,531907405	1,32E-02	9,08E-01	1,00E+00	Arl4c	1,00	No
ENSMUSG00000049871	6,23E-02	1,607215276	1,33E-01	7,16E-01	9,13E-01	Nlrc3	1,04	No
ENSMUSG00000049872	-9,26E-01	-1,896015668	2,11E+00	1,46E-01	4,84E-01	Fam26e	0,53	No
ENSMUSG00000049878	1,62E-02	5,789021922	5,81E-02	8,10E-01	9,65E-01	Rlf	1,01	No
ENSMUSG00000049881	-6,32E-02	3,237687008	3,58E-01	5,49E-01	8,17E-01	2810025M15Rik	0,96	No
ENSMUSG00000049882	-1,69E-02	2,860398124	2,09E-02	8,85E-01	1,00E+00	Vcpkmt	0,99	No
ENSMUSG00000049891	1,01E-01	4,744845426	1,69E+00	1,93E-01	5,50E-01	Gm7984	1,07	No
ENSMUSG00000049892	-4,52E-02	0,087414656	2,70E-02	8,70E-01	1,00E+00	Rasd1	0,97	No
ENSMUSG00000049897	4,44E-02	1,465890531	6,59E-02	7,97E-01	9,58E-01	Stkld1	1,03	No
ENSMUSG00000049902	-4,80E-02	-2,896135597	1,06E-03	9,74E-01	1,00E+00	4921517D22Rik	0,97	No
ENSMUSG00000049904	-7,41E-02	0,229764835	7,30E-02	7,87E-01	9,53E-01	Tmem17	0,95	No
ENSMUSG00000049907	6,29E-01	-0,024847805	4,51E+00	3,36E-02	2,15E-01	Rasl11b	1,55	No
ENSMUSG00000049908	-3,86E+00	-2,71163588	7,12E+00	7,64E-03	7,65E-02	Gja8	0,07	No
ENSMUSG00000049916	-3,15E-02	1,726413904	3,67E-02	8,48E-01	9,88E-01	2610318N02Rik	0,98	No
ENSMUSG00000049922	2,20E-01	6,387668351	1,20E+01	5,20E-04	9,47E-03	Slc35c1	1,16	Yes
ENSMUSG00000049926	7,45E-01	-2,770594121	3,02E-01	5,83E-01	8,38E-01	Olfr921	1,68	No
ENSMUSG00000049928	1,73E-01	1,021227607	6,72E-01	4,12E-01	7,08E-01	Glp2r	1,13	No
ENSMUSG00000049932	1,45E-01	5,904481213	4,76E+00	2,92E-02	1,94E-01	H2afx	1,11	No
ENSMUSG00000049939	-2,05E+00	-1,448470775	9,13E+00	2,51E-03	3,34E-02	Lrrc4	0,24	Yes
ENSMUSG00000049940	2,20E-02	5,463257425	1,01E-01	7,51E-01	9,32E-01	Pgrmc2	1,02	No
ENSMUSG00000049950	1,11E-01	1,146535533	3,34E-01	5,63E-01	8,26E-01	Rpp38	1,08	No
ENSMUSG00000049957	2,22E-01	3,307333356	4,67E+00	3,07E-02	2,02E-01	Ccdc137	1,17	No
ENSMUSG00000049960	-1,32E-01	4,68465877	2,86E+00	9,08E-02	3,82E-01	Mrps16	0,91	No
ENSMUSG00000049969	8,06E-02	6,178452985	1,57E+00	2,10E-01	5,50E-01	Plekhf2	1,06	No
ENSMUSG00000049971	-8,75E-01	-1,600617882	2,34E+00	1,26E-01	4,45E-01	Glt1d1	0,55	No

ENSMUSG00000049985	-8,09E-02	2,682676004	4,50E-01	5,02E-01	7,87E-01	Ankrd55	0,95	No
ENSMUSG00000049988	1,02E-01	6,504343531	2,61E+00	1,06E-01	3,99E-01	Lrrc25	1,07	No
ENSMUSG00000049999	2,79E-01	4,261221521	1,04E+01	1,29E-03	1,98E-02	Ppp1r3d	1,21	Yes
ENSMUSG00000050002	-8,50E-02	4,640097424	1,18E+00	2,77E-01	5,74E-01	Idnk	0,94	No
ENSMUSG00000050010	3,97E-01	0,744945328	2,88E+00	8,96E-02	3,80E-01	Shisa3	1,32	No
ENSMUSG00000050014	-2,03E+00	-1,424745115	1,46E+01	1,36E-04	3,12E-03	Apol10b	0,25	Yes
ENSMUSG00000050017	5,84E-02	5,729194032	7,49E-01	3,87E-01	6,87E-01	Pitpnb	1,04	No
ENSMUSG00000050022	2,31E-01	7,266622421	1,49E+01	1,15E-04	2,73E-03	Amz1	1,17	Yes
ENSMUSG00000050030	-1,94E+00	-3,107619243	1,50E+00	2,21E-01	5,50E-01	Olfr728	0,26	No
ENSMUSG00000050035	6,15E-02	-1,683224165	9,25E-03	9,23E-01	1,00E+00	Fhl4	1,04	No
ENSMUSG00000050043	3,03E-02	4,908130396	1,63E-01	6,86E-01	8,97E-01	Tmx2	1,02	No
ENSMUSG00000050050	-4,54E-01	-0,436655929	1,96E+00	1,61E-01	5,10E-01	Ccdc158	0,73	No
ENSMUSG00000050058	-3,88E-03	-3,03362684	4,02E-12	1,00E+00	1,00E+00	Prm3	1,00	No
ENSMUSG00000050063	-1,89E+00	-3,033648118	1,42E+00	2,33E-01	5,50E-01	Klk6	0,27	No
ENSMUSG00000050064	9,07E-01	-1,165840765	3,18E+00	7,45E-02	3,47E-01	Zfp697	1,88	No
ENSMUSG00000050069	-2,70E+00	-2,896096643	2,88E+00	8,95E-02	3,79E-01	Grem2	0,15	No
ENSMUSG00000050075	3,40E-01	6,666800485	2,99E+01	4,51E-08	2,73E-06	Gpr171	1,27	Yes
ENSMUSG00000050079	-1,18E-01	5,669701453	3,06E+00	8,04E-02	3,63E-01	Rspry1	0,92	No
ENSMUSG00000050087	7,56E-01	-2,963268003	3,13E-01	5,76E-01	8,34E-01	Cby3	1,69	No
ENSMUSG00000050088	9,30E-02	5,181677954	1,67E+00	1,97E-01	5,50E-01	1600012H06Rik	1,07	No
ENSMUSG00000050097	8,26E-01	-2,711748546	5,94E-01	4,41E-01	7,33E-01	Ces2b	1,77	No
ENSMUSG00000050100	-8,40E-01	-2,832005894	3,87E-01	5,34E-01	8,07E-01	Hmx2	0,56	No
ENSMUSG00000050103	-2,19E-01	-0,070310226	5,44E-01	4,61E-01	7,49E-01	Agmo	0,86	No
ENSMUSG00000050106	-2,82E-01	3,429210567	7,81E+00	5,20E-03	5,75E-02	Tmc8	0,82	No
ENSMUSG00000050107	2,44E-01	4,71229645	9,94E+00	1,61E-03	2,36E-02	Haspin	1,18	Yes
ENSMUSG00000050108	3,42E-01	1,461306856	3,58E+00	5,86E-02	2,99E-01	Bpifc	1,27	No
ENSMUSG00000050114	-1,18E-02	3,457419689	1,38E-02	9,07E-01	1,00E+00	Prdx6b	0,99	No
ENSMUSG00000050122	-5,97E-01	-1,088561662	1,89E+00	1,69E-01	5,24E-01	Vwa3b	0,66	No
ENSMUSG00000050132	4,18E-01	-1,770618847	5,23E-01	4,70E-01	7,58E-01	Sarm1	1,34	No
ENSMUSG00000050138	-8,40E-02	-1,740861671	1,64E-02	8,98E-01	1,00E+00	Kcnk12	0,94	No
ENSMUSG00000050144	1,15E-01	5,808381951	2,94E+00	8,65E-02	3,78E-01	Slc25a44	1,08	No
ENSMUSG00000050147	-5,95E-01	-0,847679906	1,88E+00	1,71E-01	5,26E-01	F2rl3	0,66	No

ENSMUSG00000050150	-5,00E-01	-1,863669567	6,07E-01	4,36E-01	7,29E-01	Slc9b1	0,71	No
ENSMUSG00000050157	-2,59E-01	-0,824122753	3,82E-01	5,36E-01	8,08E-01	Gm867	0,84	No
ENSMUSG00000050174	5,44E-02	1,264936393	8,13E-02	7,76E-01	9,45E-01	Nudt6	1,04	No
ENSMUSG00000050179	1,87E+00	-3,107639691	1,38E+00	2,40E-01	5,50E-01	A930002I21Rik	3,67	No
ENSMUSG00000050188	1,07E-01	4,191336142	1,57E+00	2,10E-01	5,50E-01	Lsm10	1,08	No
ENSMUSG00000050189	-8,94E-01	-1,655044417	2,70E+00	1,00E-01	3,92E-01	Etos1	0,54	No
ENSMUSG00000050192	-1,91E-01	3,257584716	3,27E+00	7,06E-02	3,36E-01	Eif5a2	0,88	No
ENSMUSG00000050195	5,88E-01	3,453696325	3,37E+01	6,47E-09	4,67E-07	Scd4	1,50	Yes
ENSMUSG00000050199	-3,01E-01	0,747929472	1,81E+00	1,79E-01	5,39E-01	Lgr4	0,81	No
ENSMUSG00000050212	-2,26E-01	4,737153083	8,62E+00	3,32E-03	4,14E-02	Eva1b	0,85	Yes
ENSMUSG00000050213	1,92E-01	4,644903915	6,05E+00	1,39E-02	1,19E-01	Snip1	1,14	No
ENSMUSG00000050222	-3,16E-02	-0,535339991	8,23E-03	9,28E-01	1,00E+00	Il17d	0,98	No
ENSMUSG00000050229	-7,08E-02	5,895460018	1,14E+00	2,85E-01	5,81E-01	Pigm	0,95	No
ENSMUSG00000050234	7,45E-01	-2,896140634	3,03E-01	5,82E-01	8,38E-01	Gja4	1,68	No
ENSMUSG00000050240	-1,02E-01	2,017510013	4,40E-01	5,07E-01	7,91E-01	Hic2	0,93	No
ENSMUSG00000050241	-6,03E-02	-1,770504975	9,34E-03	9,23E-01	1,00E+00	Klre1	0,96	No
ENSMUSG00000050243	-2,19E-01	4,012264353	6,11E+00	1,35E-02	1,16E-01	Gm5446	0,86	No
ENSMUSG00000050244	3,38E-01	6,666144476	2,94E+01	5,98E-08	3,49E-06	Heatr1	1,26	Yes
ENSMUSG00000050248	-8,32E-01	-2,896143825	3,80E-01	5,38E-01	8,09E-01	Evc2	0,56	No
ENSMUSG00000050270	-1,49E-02	1,195136727	6,10E-03	9,38E-01	1,00E+00	Tmem220	0,99	No
ENSMUSG00000050271	2,57E-01	2,309473542	3,31E+00	6,87E-02	3,30E-01	Prag1	1,19	No
ENSMUSG00000050272	3,57E-01	4,543114397	1,94E+01	1,04E-05	3,48E-04	Dscam	1,28	Yes
ENSMUSG00000050276	-1,90E+00	-2,770607626	1,44E+00	2,31E-01	5,50E-01	Mrgprg	0,27	No
ENSMUSG00000050288	6,91E-01	-2,26821868	6,91E-01	4,06E-01	7,02E-01	Fzd2	1,61	No
ENSMUSG00000050295	3,18E-02	-0,726217468	7,30E-03	9,32E-01	1,00E+00	Foxc1	1,02	No
ENSMUSG00000050296	1,81E+00	-3,10765737	1,28E+00	2,58E-01	5,50E-01	Abca12	3,49	No
ENSMUSG00000050299	-2,06E-01	9,272225373	1,32E+01	2,76E-04	5,67E-03	Gm9843	0,87	Yes
ENSMUSG00000050304	-3,18E-01	-1,683187403	2,83E-01	5,95E-01	8,43E-01	Slc25a2	0,80	No
ENSMUSG00000050310	2,90E-02	5,969582895	1,95E-01	6,58E-01	8,81E-01	Rictor	1,02	No
ENSMUSG00000050312	-4,61E-02	4,190632711	2,92E-01	5,89E-01	8,41E-01	Nsun3	0,97	No
ENSMUSG00000050315	-5,18E-01	0,019412994	3,35E+00	6,72E-02	3,26E-01	Synpo2	0,70	No
ENSMUSG00000050323	-2,95E-02	1,664323803	3,14E-02	8,59E-01	9,95E-01	Ndufaf6	0,98	No

ENSMUSG00000050335	1,70E-01	11,99309251	9,29E+00	2,31E-03	3,15E-02	Lgals3	1,12	Yes
ENSMUSG00000050345	-3,93E-01	-2,219711626	2,09E-01	6,47E-01	8,75E-01	4930486L24Rik	0,76	No
ENSMUSG00000050350	2,63E-01	1,053832727	1,67E+00	1,97E-01	5,50E-01	Gpr18	1,20	No
ENSMUSG00000050357	-1,88E-01	1,154019305	9,52E-01	3,29E-01	6,30E-01	Carmil2	0,88	No
ENSMUSG00000050359	1,82E+00	-3,033661385	1,31E+00	2,52E-01	5,50E-01	Sprr1a	3,54	No
ENSMUSG00000050370	3,35E-01	3,845533587	1,16E+01	6,42E-04	1,13E-02	Ch25h	1,26	Yes
ENSMUSG00000050373	-9,96E-02	4,325567076	1,45E+00	2,28E-01	5,50E-01	Snx21	0,93	No
ENSMUSG00000050377	4,29E-01	2,267978264	9,53E+00	2,02E-03	2,82E-02	Il31ra	1,35	Yes
ENSMUSG00000050382	2,80E-02	-0,140871115	8,15E-03	9,28E-01	1,00E+00	Kif7	1,02	No
ENSMUSG00000050390	1,22E-01	5,692942917	3,24E+00	7,20E-02	3,40E-01	C77080	1,09	No
ENSMUSG00000050395	-6,95E-02	2,067267477	2,35E-01	6,27E-01	8,61E-01	Tnfsf15	0,95	No
ENSMUSG00000050397	-6,81E-02	-2,268126419	6,41E-03	9,36E-01	1,00E+00	Foxl2	0,95	No
ENSMUSG00000050410	1,67E-01	4,937308436	4,93E+00	2,63E-02	1,81E-01	Tcf19	1,12	No
ENSMUSG00000050423	1,40E+00	-1,998093681	2,79E+00	9,49E-02	3,86E-01	Ppp1r3g	2,63	No
ENSMUSG00000050428	7,58E-02	4,040264758	7,79E-01	3,77E-01	6,78E-01	Fbxo46	1,05	No
ENSMUSG00000050439	-1,68E-01	-0,711678812	1,84E-01	6,68E-01	8,87E-01	Enthd1	0,89	No
ENSMUSG00000050440	-4,38E-01	-2,401366126	1,96E-01	6,58E-01	8,81E-01	Hamp	0,74	No
ENSMUSG00000050445	7,96E-01	-2,44864617	5,52E-01	4,58E-01	7,47E-01	Cyp8b1	1,74	No
ENSMUSG00000050447	-2,71E+00	-3,033620915	2,92E+00	8,75E-02	3,78E-01	Lypd6	0,15	No
ENSMUSG00000050463	1,85E+00	-2,963264462	1,34E+00	2,48E-01	5,50E-01	Krt78	3,59	No
ENSMUSG00000050468	-3,83E-02	-3,107651936	-7,91E-11	1,00E+00	1,00E+00	Astl	0,97	No
ENSMUSG00000050471	4,44E-02	4,811415132	3,41E-01	5,59E-01	8,22E-01	Fam118b	1,03	No
ENSMUSG00000050473	-2,83E-01	-2,070209452	1,39E-01	7,09E-01	9,09E-01	Slc35d3	0,82	No
ENSMUSG00000050490	2,22E-02	5,781800603	1,10E-01	7,40E-01	9,26E-01	Gm8394	1,02	No
ENSMUSG00000050493	6,14E-01	-1,20603627	1,66E+00	1,97E-01	5,50E-01	Fam167b	1,53	No
ENSMUSG00000050503	-2,54E-02	1,951746066	2,79E-02	8,67E-01	9,99E-01	Fbxl22	0,98	No
ENSMUSG00000050511	-1,47E+00	0,678465972	3,85E+01	5,43E-10	4,59E-08	Oprd1	0,36	Yes
ENSMUSG00000050526	4,34E-01	-2,711766619	1,47E-01	7,02E-01	9,05E-01	4933406M09Rik	1,35	No
ENSMUSG00000050530	-4,72E-01	-0,236607346	1,95E+00	1,63E-01	5,14E-01	Fam171a1	0,72	No
ENSMUSG00000050533	-1,12E-01	3,662101596	1,40E+00	2,36E-01	5,50E-01	Gm9845	0,93	No
ENSMUSG00000050534	-8,10E-01	-2,963247942	3,56E-01	5,51E-01	8,17E-01	Htr5b	0,57	No
ENSMUSG00000050538	-3,06E-01	1,214516998	2,30E+00	1,30E-01	4,52E-01	B230217C12Rik	0,81	No

ENSMUSG00000050541	-1,75E+00	-2,655094277	2,12E+00	1,45E-01	4,83E-01	Adra1b	0,30	No
ENSMUSG00000050545	-7,33E-01	-1,389783402	1,96E+00	1,61E-01	5,10E-01	Fam228b	0,60	No
ENSMUSG00000050549	1,64E-02	4,306181639	3,89E-02	8,44E-01	9,85E-01	5730508B09Rik	1,01	No
ENSMUSG00000050550	4,29E-02	6,30364809	4,54E-01	5,01E-01	7,86E-01	Gm11868	1,03	No
ENSMUSG00000050552	7,31E-02	4,917396479	9,42E-01	3,32E-01	6,32E-01	Lamtor4	1,05	No
ENSMUSG00000050553	2,70E-01	-0,711778465	4,39E-01	5,08E-01	7,92E-01	Gk2	1,21	No
ENSMUSG00000050555	5,20E-02	3,735829042	3,17E-01	5,73E-01	8,33E-01	Hyls1	1,04	No
ENSMUSG00000050556	-2,55E-01	0,18362027	8,40E-01	3,59E-01	6,61E-01	Kcnb1	0,84	No
ENSMUSG00000050558	-1,14E-02	-3,033635021	1,50E-05	9,97E-01	1,00E+00	Prokr2	0,99	No
ENSMUSG00000050565	-6,55E-02	7,296248262	1,20E+00	2,73E-01	5,69E-01	Tor1aip2	0,96	No
ENSMUSG00000050567	9,72E-02	5,320627393	1,87E+00	1,71E-01	5,27E-01	Maml1	1,07	No
ENSMUSG00000050578	1,96E-01	5,890450628	8,51E+00	3,53E-03	4,32E-02	Mmp13	1,15	Yes
ENSMUSG00000050592	3,20E-01	4,800008437	1,76E+01	2,67E-05	7,97E-04	Fam78a	1,25	Yes
ENSMUSG00000050600	-3,53E-01	-1,472925366	4,80E-01	4,89E-01	7,76E-01	Zfp831	0,78	No
ENSMUSG00000050605	1,82E-01	3,09985379	2,82E+00	9,30E-02	3,83E-01	Zfp61	1,13	No
ENSMUSG00000050608	-1,17E-01	5,787873659	3,07E+00	7,96E-02	3,61E-01	Minos1	0,92	No
ENSMUSG00000050612	-3,83E-01	-2,268065202	1,85E-01	6,67E-01	8,86E-01	Txndc2	0,77	No
ENSMUSG00000050619	4,12E-02	4,920682407	3,08E-01	5,79E-01	8,36E-01	Zscan29	1,03	No
ENSMUSG00000050621	-4,21E-01	7,879847433	5,18E+01	6,02E-13	7,54E-11	Rps27rt	0,75	Yes
ENSMUSG00000050623	2,06E+00	-2,311297568	5,41E+00	2,00E-02	1,51E-01	Catsperz	4,18	No
ENSMUSG00000050627	1,71E-01	6,276742403	7,18E+00	7,37E-03	7,45E-02	Gpd1l	1,13	No
ENSMUSG00000050628	8,98E-02	5,260979218	1,58E+00	2,09E-01	5,50E-01	Ubald2	1,06	No
ENSMUSG00000050640	-6,93E-01	3,315569939	4,53E+01	1,69E-11	1,74E-09	Tmem150c	0,62	Yes
ENSMUSG00000050641	1,84E+00	-3,033632273	1,33E+00	2,49E-01	5,50E-01	BC048562	3,58	No
ENSMUSG00000050650	7,89E-01	-2,71174177	5,39E-01	4,63E-01	7,51E-01	Mrgpra1	1,73	No
ENSMUSG00000050668	5,54E-02	3,602692825	3,24E-01	5,69E-01	8,30E-01	Gpatch11	1,04	No
ENSMUSG00000050671	7,52E-01	-2,655080127	3,07E-01	5,80E-01	8,37E-01	Ism2	1,68	No
ENSMUSG00000050675	-2,64E-01	1,452349497	2,17E+00	1,41E-01	4,75E-01	Gp1ba	0,83	No
ENSMUSG00000050677	1,49E-01	-0,436910773	1,96E-01	6,58E-01	8,81E-01	Ccdc96	1,11	No
ENSMUSG00000050685	-7,07E-01	-2,146111493	8,50E-01	3,57E-01	6,58E-01	Ccdc54	0,61	No
ENSMUSG00000050697	8,54E-02	5,722589012	1,62E+00	2,03E-01	5,50E-01	Prkaa1	1,06	No
ENSMUSG00000050700	-1,92E+00	-2,963214917	1,46E+00	2,27E-01	5,50E-01	Emilin3	0,26	No

ENSMUSG00000050702	-5,76E-01	-2,770648804	2,61E-01	6,10E-01	8,51E-01	4930563M21Rik	0,67	No
ENSMUSG00000050705	-1,59E-01	4,242184965	3,59E+00	5,80E-02	2,97E-01	2310061I04Rik	0,90	No
ENSMUSG00000050708	6,43E-02	11,08753842	1,32E+00	2,50E-01	5,50E-01	Ftl1	1,05	No
ENSMUSG00000050711	1,87E+00	-3,107639691	1,38E+00	2,40E-01	5,50E-01	Scg2	3,67	No
ENSMUSG00000050714	-1,22E-01	1,148322592	4,26E-01	5,14E-01	7,96E-01	Zbtb26	0,92	No
ENSMUSG00000050721	9,78E-02	7,64081717	2,75E+00	9,75E-02	3,90E-01	Plekho2	1,07	No
ENSMUSG00000050730	1,47E-02	0,59883109	3,68E-03	9,52E-01	1,00E+00	Arhgap42	1,01	No
ENSMUSG00000050732	-1,22E-01	6,997578111	4,05E+00	4,43E-02	2,51E-01	Vamp8	0,92	No
ENSMUSG00000050737	-9,07E-02	1,225815712	2,17E-01	6,42E-01	8,71E-01	Ptges	0,94	No
ENSMUSG00000050742	-2,68E+00	-2,831965433	2,85E+00	9,14E-02	3,82E-01	Olfr164	0,16	No
ENSMUSG00000050747	1,08E+00	-2,268049459	1,49E+00	2,22E-01	5,50E-01	Trim15	2,11	No
ENSMUSG00000050751	-7,19E-01	-0,997911087	2,71E+00	9,96E-02	3,92E-01	Pgbd5	0,61	No
ENSMUSG00000050761	5,54E-01	-0,72634539	1,85E+00	1,73E-01	5,30E-01	Gp1bb	1,47	No
ENSMUSG00000050762	-6,85E-02	-1,831997245	1,09E-02	9,17E-01	1,00E+00	Prss27	0,95	No
ENSMUSG00000050772	-1,87E+00	-3,107637761	1,39E+00	2,38E-01	5,50E-01	Olfr1124	0,27	No
ENSMUSG00000050777	4,04E-01	5,66801991	3,46E+01	4,15E-09	3,07E-07	Tmem37	1,32	Yes
ENSMUSG00000050783	-1,36E+00	-2,83197871	1,17E+00	2,80E-01	5,77E-01	Htr1f	0,39	No
ENSMUSG00000050786	-3,36E-02	2,424734933	6,30E-02	8,02E-01	9,61E-01	Ccdc126	0,98	No
ENSMUSG00000050796	2,65E-01	3,069350236	5,66E+00	1,73E-02	1,38E-01	B3galt6	1,20	No
ENSMUSG00000050799	-1,75E-01	-0,051989473	3,16E-01	5,74E-01	8,33E-01	Hist1h2ba	0,89	No
ENSMUSG00000050812	3,38E-03	6,873557654	3,06E-03	9,56E-01	1,00E+00	AI314180	1,00	No
ENSMUSG00000050815	-2,74E+00	-3,033596063	2,99E+00	8,38E-02	3,71E-01	Olfr1441	0,15	No
ENSMUSG00000050821	-2,63E-01	2,856314189	5,27E+00	2,16E-02	1,58E-01	Fam131a	0,83	No
ENSMUSG00000050822	-3,83E-02	-3,107651936	-7,91E-11	1,00E+00	1,00E+00	Slc29a4	0,97	No
ENSMUSG00000050824	-4,20E-01	-1,146025647	8,83E-01	3,47E-01	6,48E-01	Sstr5	0,75	No
ENSMUSG00000050830	1,84E+00	-3,033649193	1,33E+00	2,49E-01	5,50E-01	Vwc2	3,58	No
ENSMUSG00000050844	1,98E-01	-1,289431737	1,59E-01	6,90E-01	8,99E-01	1700020N01Rik	1,15	No
ENSMUSG00000050846	2,51E-01	2,907990976	4,83E+00	2,80E-02	1,89E-01	Zfp623	1,19	No
ENSMUSG00000050854	3,19E-01	-2,497618204	1,03E-01	7,48E-01	9,30E-01	Tmem125	1,25	No
ENSMUSG00000050855	-1,64E-01	0,539334854	4,66E-01	4,95E-01	7,80E-01	Zfp940	0,89	No
ENSMUSG00000050856	-3,33E-01	1,742872244	4,33E+00	3,74E-02	2,30E-01	Atp5k	0,79	No
ENSMUSG00000050860	1,24E-01	4,0474887	1,94E+00	1,64E-01	5,15E-01	Phospho1	1,09	No

ENSMUSG00000050866	-1,91E+00	-2,896105328	1,45E+00	2,28E-01	5,50E-01	Clrn3	0,27	No
ENSMUSG00000050876	-9,23E-01	-1,963165901	2,10E+00	1,47E-01	4,86E-01	Spata31d1a	0,53	No
ENSMUSG00000050890	1,37E-01	3,800338924	2,23E+00	1,36E-01	4,64E-01	Pdik1l	1,10	No
ENSMUSG00000050891	-1,80E-01	1,730606178	1,22E+00	2,69E-01	5,65E-01	Tatdn1	0,88	No
ENSMUSG00000050896	-7,67E-01	0,971441131	1,38E+01	2,06E-04	4,45E-03	Rtn4rl2	0,59	Yes
ENSMUSG00000050908	-4,65E-02	4,098004822	2,88E-01	5,91E-01	8,42E-01	Tvp23a	0,97	No
ENSMUSG00000050910	9,34E-02	-1,472847932	3,66E-02	8,48E-01	9,88E-01	Cdr2l	1,07	No
ENSMUSG00000050912	-6,76E-01	9,955898657	1,43E+02	6,29E-33	4,38E-30	Tmem123	0,63	Yes
ENSMUSG00000050914	6,59E-01	1,35628359	1,32E+01	2,77E-04	5,69E-03	Ankrd37	1,58	Yes
ENSMUSG00000050917	-9,78E-01	-2,548194802	1,16E+00	2,82E-01	5,78E-01	Fgf4	0,51	No
ENSMUSG00000050919	-2,11E-01	5,606439488	9,78E+00	1,77E-03	2,53E-02	Zfp366	0,86	Yes
ENSMUSG00000050930	1,65E-01	1,082867047	6,76E-01	4,11E-01	7,07E-01	Map10	1,12	No
ENSMUSG00000050931	-6,92E-02	3,924343359	5,86E-01	4,44E-01	7,35E-01	Sgms2	0,95	No
ENSMUSG00000050936	-1,60E+00	-3,033631402	1,07E+00	3,00E-01	5,96E-01	Gm42743	0,33	No
ENSMUSG00000050944	7,18E-02	-1,185588933	2,53E-02	8,74E-01	1,00E+00	Efcab5	1,05	No
ENSMUSG00000050945	2,27E-01	1,040979444	1,23E+00	2,67E-01	5,64E-01	Zfp438	1,17	No
ENSMUSG00000050947	-3,36E-01	2,049594668	5,15E+00	2,33E-02	1,67E-01	Amigo1	0,79	No
ENSMUSG00000050953	-1,20E+00	-0,847635891	9,43E+00	2,13E-03	2,94E-02	Gja1	0,43	Yes
ENSMUSG00000050954	1,30E-02	2,61264629	1,08E-02	9,17E-01	1,00E+00	Zfp169	1,01	No
ENSMUSG00000050957	-6,17E-01	4,459324118	5,78E+01	2,96E-14	4,20E-12	Insl6	0,65	Yes
ENSMUSG00000050961	6,75E-02	1,552964907	1,49E-01	7,00E-01	9,04E-01	AY358078	1,05	No
ENSMUSG00000050965	-5,27E-02	4,597383717	4,42E-01	5,06E-01	7,90E-01	Prkca	0,96	No
ENSMUSG00000050966	-9,48E-02	-2,185648962	1,23E-02	9,12E-01	1,00E+00	Lin28a	0,94	No
ENSMUSG00000050967	4,02E-01	3,651873937	1,77E+01	2,55E-05	7,65E-04	Creg2	1,32	Yes
ENSMUSG00000050973	4,25E-01	2,881112224	1,32E+01	2,81E-04	5,75E-03	Gdpgp1	1,34	Yes
ENSMUSG00000050974	-2,51E-01	4,938267896	1,14E+01	7,33E-04	1,25E-02	Gm9847	0,84	Yes
ENSMUSG00000050982	1,87E+00	-3,107639691	1,38E+00	2,40E-01	5,50E-01	Apol10a	3,67	No
ENSMUSG00000050989	9,54E-02	5,751009699	2,01E+00	1,56E-01	5,01E-01	Selenon	1,07	No
ENSMUSG00000050994	3,39E-01	-0,079387363	1,22E+00	2,69E-01	5,66E-01	Adgb	1,26	No
ENSMUSG00000051000	-6,91E-01	-1,448630852	1,92E+00	1,66E-01	5,19E-01	Fam160a1	0,62	No
ENSMUSG00000051007	3,14E-02	4,497735178	1,55E-01	6,94E-01	9,02E-01	Gatd1	1,02	No
ENSMUSG00000051008	-6,13E-01	-0,86357135	1,69E+00	1,94E-01	5,50E-01	4930412M03Rik	0,65	No

ENSMUSG00000051022	-2,02E+00	-2,54822875	3,10E+00	7,82E-02	3,57E-01	Hs3st1	0,25	No
ENSMUSG00000051029	-3,35E-01	0,2067491	1,44E+00	2,30E-01	5,50E-01	Serpinb1b	0,79	No
ENSMUSG00000051034	3,53E-01	1,320149924	3,44E+00	6,36E-02	3,15E-01	Zfp11	1,28	No
ENSMUSG00000051036	1,87E+00	-3,107639691	1,38E+00	2,40E-01	5,50E-01	Ttc24	3,67	No
ENSMUSG00000051037	1,70E-01	1,435154973	9,26E-01	3,36E-01	6,37E-01	Zfp455	1,12	No
ENSMUSG00000051043	4,96E-01	5,145810549	4,64E+01	9,86E-12	1,04E-09	Gprc5c	1,41	Yes
ENSMUSG00000051048	-1,33E+00	-2,711695278	1,10E+00	2,94E-01	5,89E-01	P4ha3	0,40	No
ENSMUSG00000051054	1,25E+00	-2,49762211	9,65E-01	3,26E-01	6,27E-01	1700014D04Rik	2,38	No
ENSMUSG00000051056	-1,89E+00	-3,1076441	1,42E+00	2,33E-01	5,50E-01	Gja10	0,27	No
ENSMUSG00000051065	-3,00E-01	1,419635716	2,61E+00	1,06E-01	3,98E-01	Mb21d2	0,81	No
ENSMUSG00000051074	-1,89E-01	-1,89618463	9,55E-02	7,57E-01	9,35E-01	4930579K19Rik	0,88	No
ENSMUSG00000051076	-8,12E-01	-2,896127374	3,61E-01	5,48E-01	8,15E-01	Vtcn1	0,57	No
ENSMUSG00000051098	-5,33E-02	2,210678213	1,41E-01	7,08E-01	9,08E-01	Mblac2	0,96	No
ENSMUSG00000051107	2,38E-01	1,483702664	1,82E+00	1,77E-01	5,37E-01	Gm15440	1,18	No
ENSMUSG00000051111	-2,71E+00	-2,896162395	2,92E+00	8,76E-02	3,78E-01	Sv2c	0,15	No
ENSMUSG00000051113	-3,70E-01	0,002052461	1,68E+00	1,95E-01	5,50E-01	Fam71e1	0,77	No
ENSMUSG00000051116	-4,82E-02	3,762709251	2,62E-01	6,09E-01	8,51E-01	Gm8121	0,97	No
ENSMUSG00000051118	-1,49E-01	3,98724318	2,81E+00	9,39E-02	3,84E-01	Olfr77	0,90	No
ENSMUSG00000051124	-7,80E-01	-0,448721107	5,02E+00	2,51E-02	1,75E-01	Gimap9	0,58	No
ENSMUSG00000051133	-2,98E-02	0,799330708	1,72E-02	8,96E-01	1,00E+00	Gm6293	0,98	No
ENSMUSG00000051136	3,14E-02	-3,107617333	-7,69E-10	1,00E+00	1,00E+00	Ghsr	1,02	No
ENSMUSG00000051146	4,24E-01	0,019388911	1,86E+00	1,73E-01	5,30E-01	Camk2n2	1,34	No
ENSMUSG00000051147	8,60E-02	2,179622508	3,71E-01	5,42E-01	8,12E-01	Nat2	1,06	No
ENSMUSG00000051149	3,43E-02	5,151195087	2,29E-01	6,32E-01	8,64E-01	Adnp	1,02	No
ENSMUSG00000051154	-1,62E-01	5,627116714	5,67E+00	1,72E-02	1,37E-01	Commd3	0,89	No
ENSMUSG00000051166	-4,08E-01	3,944615857	2,14E+01	3,74E-06	1,41E-04	Eml5	0,75	Yes
ENSMUSG00000051169	2,02E-01	1,173326872	1,09E+00	2,96E-01	5,92E-01	Rpusd3	1,15	No
ENSMUSG00000051177	-3,15E-01	0,323869276	1,45E+00	2,29E-01	5,50E-01	Plcb1	0,80	No
ENSMUSG00000051184	-3,64E-01	3,204244841	1,18E+01	5,77E-04	1,04E-02	Zfp524	0,78	Yes
ENSMUSG00000051185	1,17E-01	5,297589718	2,69E+00	1,01E-01	3,93E-01	Fam174a	1,08	No
ENSMUSG00000051212	2,79E-01	5,261383668	1,53E+01	9,25E-05	2,27E-03	Gpr183	1,21	Yes
ENSMUSG00000051223	-4,39E-02	8,010098907	5,70E-01	4,50E-01	7,40E-01	Bzw1	0,97	No

ENSMUSG00000051225	-1,02E+00	-1,800968341	2,32E+00	1,28E-01	4,49E-01	Fam83a	0,49	No
ENSMUSG00000051232	-3,82E-02	4,427110283	2,18E-01	6,40E-01	8,70E-01	Tmem199	0,97	No
ENSMUSG00000051234	-2,35E-02	5,518079557	1,16E-01	7,33E-01	9,23E-01	Rnf7	0,98	No
ENSMUSG00000051235	2,36E-01	3,9307479	7,01E+00	8,12E-03	7,99E-02	Gen1	1,18	No
ENSMUSG00000051236	-2,23E-01	-1,015692677	2,55E-01	6,14E-01	8,53E-01	Msrb3	0,86	No
ENSMUSG00000051237	1,87E+00	-3,107639691	1,38E+00	2,40E-01	5,50E-01	9230109A22Rik	3,67	No
ENSMUSG00000051238	3,01E-01	2,718253221	6,04E+00	1,40E-02	1,20E-01	Swsap1	1,23	No
ENSMUSG00000051243	-1,35E+00	-2,832022066	1,13E+00	2,87E-01	5,83E-01	Islr2	0,39	No
ENSMUSG00000051246	2,85E-01	1,764978965	2,83E+00	9,28E-02	3,83E-01	Msantd1	1,22	No
ENSMUSG00000051251	-4,00E-02	-2,77057572	1,08E-03	9,74E-01	1,00E+00	Nhlh1	0,97	No
ENSMUSG00000051255	-5,59E-02	5,382356572	6,34E-01	4,26E-01	7,20E-01	Gm6563	0,96	No
ENSMUSG00000051256	-8,34E-03	4,874515056	1,22E-02	9,12E-01	1,00E+00	Jagn1	0,99	No
ENSMUSG00000051262	-1,94E+00	-3,107619243	1,50E+00	2,21E-01	5,50E-01	Nat8f3	0,26	No
ENSMUSG00000051278	2,04E-01	3,997699829	5,17E+00	2,29E-02	1,65E-01	Zgrf1	1,15	No
ENSMUSG00000051279	-1,87E+00	-3,107637761	1,39E+00	2,38E-01	5,50E-01	Gdf6	0,27	No
ENSMUSG00000051285	-9,09E-03	5,794599681	1,86E-02	8,92E-01	1,00E+00	Pcmtd1	0,99	No
ENSMUSG00000051295	7,43E-01	-2,963261206	3,01E-01	5,83E-01	8,38E-01	9630028B13Rik	1,67	No
ENSMUSG00000051306	5,36E-02	3,967320495	3,63E-01	5,47E-01	8,14E-01	Usp42	1,04	No
ENSMUSG00000051314	4,30E-01	3,678604444	1,99E+01	8,31E-06	2,85E-04	Ffar2	1,35	Yes
ENSMUSG00000051316	1,81E-01	3,818772412	3,88E+00	4,89E-02	2,66E-01	Taf7	1,13	No
ENSMUSG00000051319	-3,43E-02	3,826724472	1,35E-01	7,13E-01	9,11E-01	1500011K16Rik	0,98	No
ENSMUSG00000051329	8,86E-02	5,266394192	1,54E+00	2,14E-01	5,50E-01	Nup160	1,06	No
ENSMUSG00000051331	-8,67E-02	-1,627672834	2,64E-02	8,71E-01	1,00E+00	Cacna1c	0,94	No
ENSMUSG00000051335	-2,76E-01	5,261498152	1,47E+01	1,28E-04	2,97E-03	Gfod1	0,83	Yes
ENSMUSG00000051339	3,37E-01	4,255726283	1,56E+01	7,66E-05	1,94E-03	2900026A02Rik	1,26	Yes
ENSMUSG00000051341	2,27E-01	3,71085263	6,20E+00	1,28E-02	1,12E-01	Zfp52	1,17	No
ENSMUSG00000051343	1,25E-01	6,987190511	4,24E+00	3,94E-02	2,36E-01	Rab11fip5	1,09	No
ENSMUSG00000051344	1,95E-01	6,538211873	9,68E+00	1,87E-03	2,65E-02	Plekhm3	1,14	Yes
ENSMUSG00000051346	5,59E-02	3,444828267	3,14E-01	5,75E-01	8,34E-01	Spryd4	1,04	No
ENSMUSG00000051351	1,98E-01	3,648567514	4,36E+00	3,69E-02	2,28E-01	Zfp46	1,15	No
ENSMUSG00000051355	-5,63E-02	4,719631722	5,30E-01	4,66E-01	7,55E-01	Commd1	0,96	No
ENSMUSG00000051359	-1,33E+00	-1,669008195	4,65E+00	3,10E-02	2,03E-01	Ncald	0,40	No

ENSMUSG00000051367	-6,76E-02	3,712153905	5,11E-01	4,75E-01	7,63E-01	Six1	0,95	No
ENSMUSG00000051373	1,99E-02	0,028281444	4,53E-03	9,46E-01	1,00E+00	Plpp7	1,01	No
ENSMUSG00000051375	-5,50E-01	-2,107598669	6,60E-01	4,17E-01	7,12E-01	Pcdh1	0,68	No
ENSMUSG00000051378	3,24E-01	4,546687725	1,64E+01	5,09E-05	1,38E-03	Kif18b	1,25	Yes
ENSMUSG00000051379	-1,28E-01	6,767243123	4,29E+00	3,82E-02	2,32E-01	Flrt3	0,92	No
ENSMUSG00000051390	-3,75E-02	5,543316177	2,95E-01	5,87E-01	8,40E-01	Zbtb22	0,97	No
ENSMUSG00000051391	7,65E-02	8,625873809	1,79E+00	1,81E-01	5,43E-01	Ywhag	1,05	No
ENSMUSG00000051396	2,18E-01	3,990796103	5,90E+00	1,51E-02	1,27E-01	Gm45902	1,16	No
ENSMUSG00000051397	-2,31E-01	1,656032776	1,93E+00	1,65E-01	5,18E-01	Tacstd2	0,85	No
ENSMUSG00000051403	6,90E-02	5,258504354	9,32E-01	3,34E-01	6,35E-01	Ppp1r37	1,05	No
ENSMUSG00000051413	-1,60E-01	6,104142205	6,18E+00	1,29E-02	1,13E-01	Plagl2	0,89	No
ENSMUSG00000051427	-2,70E-01	2,693404169	4,91E+00	2,68E-02	1,83E-01	Ccdc157	0,83	No
ENSMUSG00000051431	1,82E+00	-3,033661385	1,31E+00	2,52E-01	5,50E-01	Gpr87	3,54	No
ENSMUSG00000051435	3,63E-01	-2,289530377	1,72E-01	6,78E-01	8,92E-01	Fhad1	1,29	No
ENSMUSG00000051439	3,67E-02	6,916391537	3,57E-01	5,50E-01	8,17E-01	Cd14	1,03	No
ENSMUSG00000051444	-1,22E-01	1,950656036	7,09E-01	4,00E-01	6,99E-01	Bbs12	0,92	No
ENSMUSG00000051451	4,53E-02	5,207182147	4,04E-01	5,25E-01	8,02E-01	Crebzf	1,03	No
ENSMUSG00000051455	-1,14E+00	-1,896071554	2,97E+00	8,47E-02	3,73E-01	Meioc	0,45	No
ENSMUSG00000051457	1,65E-01	7,023790558	7,43E+00	6,40E-03	6,73E-02	Spn	1,12	No
ENSMUSG00000051469	2,01E-02	4,957466769	7,29E-02	7,87E-01	9,53E-01	Zfp24	1,01	No
ENSMUSG00000051483	8,10E-02	3,642099638	7,02E-01	4,02E-01	7,00E-01	Cbr1	1,06	No
ENSMUSG00000051490	-2,65E+00	-3,033633091	2,78E+00	9,55E-02	3,86E-01	Foxd4	0,16	No
ENSMUSG00000051495	6,15E-02	8,06859387	1,12E+00	2,90E-01	5,86E-01	Irf2bp2	1,04	No
ENSMUSG00000051497	-3,57E+00	-2,89611556	5,73E+00	1,67E-02	1,35E-01	Kcnj16	0,08	No
ENSMUSG00000051498	1,03E-01	4,282030012	1,49E+00	2,21E-01	5,50E-01	Tlr6	1,07	No
ENSMUSG00000051499	4,02E-01	0,244182134	2,38E+00	1,23E-01	4,38E-01	Zfp786	1,32	No
ENSMUSG00000051502	3,79E-02	0,514416257	2,47E-02	8,75E-01	1,00E+00	Ufsp1	1,03	No
ENSMUSG00000051503	3,53E-01	-1,146003202	6,05E-01	4,37E-01	7,29E-01	Gm6583	1,28	No
ENSMUSG00000051504	5,86E-01	0,489920333	5,45E+00	1,96E-02	1,49E-01	Siglech	1,50	No
ENSMUSG00000051506	2,44E-01	8,915076312	1,83E+01	1,86E-05	5,80E-04	Wdfy4	1,18	Yes
ENSMUSG00000051510	6,96E-02	6,724331582	1,27E+00	2,61E-01	5,54E-01	Mafg	1,05	No
ENSMUSG00000051515	-2,91E-01	-0,185486768	7,93E-01	3,73E-01	6,74E-01	Fam181b	0,82	No

ENSMUSG00000051517	3,93E-01	3,125709901	1,32E+01	2,87E-04	5,83E-03	Arhgef39	1,31	Yes
ENSMUSG00000051518	-9,79E-02	4,026079534	1,22E+00	2,69E-01	5,66E-01	Rps19bp1	0,93	No
ENSMUSG00000051527	-2,68E+00	-3,033645766	2,84E+00	9,17E-02	3,82E-01	Usp29	0,16	No
ENSMUSG00000051528	-1,94E+00	-3,107619243	1,50E+00	2,21E-01	5,50E-01	Olfr424	0,26	No
ENSMUSG00000051548	-1,47E-01	3,1687858	1,83E+00	1,76E-01	5,35E-01	Gm6365	0,90	No
ENSMUSG00000051550	-9,50E-02	2,308162772	4,94E-01	4,82E-01	7,69E-01	Zfp579	0,94	No
ENSMUSG00000051557	3,46E-02	4,276304492	1,71E-01	6,79E-01	8,93E-01	Pusl1	1,02	No
ENSMUSG00000051586	-2,80E-01	5,130065133	1,53E+01	9,37E-05	2,29E-03	Mical3	0,82	Yes
ENSMUSG00000051590	-5,08E-01	-1,770590287	6,26E-01	4,29E-01	7,23E-01	Map3k19	0,70	No
ENSMUSG00000051591	-4,01E-02	-2,711715319	1,12E-03	9,73E-01	1,00E+00	Olfr697	0,97	No
ENSMUSG00000051596	-1,89E+00	-3,1076441	1,42E+00	2,33E-01	5,50E-01	Otop1	0,27	No
ENSMUSG00000051599	-1,94E+00	-3,107619243	1,50E+00	2,21E-01	5,50E-01	Pcdhb2	0,26	No
ENSMUSG00000051606	1,57E-01	4,184313769	3,32E+00	6,84E-02	3,29E-01	2010001K21Rik	1,11	No
ENSMUSG00000051611	6,12E-01	1,786258878	1,42E+01	1,64E-04	3,67E-03	Olfr112	1,53	Yes
ENSMUSG00000051615	2,44E-02	7,61889669	1,72E-01	6,79E-01	8,93E-01	Rap2a	1,02	No
ENSMUSG00000051627	-1,26E-01	1,487261235	5,28E-01	4,67E-01	7,56E-01	Hist1h1e	0,92	No
ENSMUSG00000051638	1,85E+00	-3,107657832	1,35E+00	2,46E-01	5,50E-01	Gm9857	3,61	No
ENSMUSG00000051639	5,39E-02	5,552189406	6,09E-01	4,35E-01	7,28E-01	Gm5812	1,04	No
ENSMUSG00000051648	-8,87E-02	1,072807443	1,94E-01	6,60E-01	8,82E-01	Kctd19	0,94	No
ENSMUSG00000051650	2,04E-01	5,47632432	8,58E+00	3,39E-03	4,20E-02	B3gnt2	1,15	Yes
ENSMUSG00000051652	-3,76E-02	-0,998130423	8,09E-03	9,28E-01	1,00E+00	Lrrc3	0,97	No
ENSMUSG00000051669	-1,82E-01	0,48677089	5,50E-01	4,58E-01	7,47E-01	AU021092	0,88	No
ENSMUSG00000051671	-2,02E-01	3,348385353	3,78E+00	5,18E-02	2,77E-01	Coa6	0,87	No
ENSMUSG00000051674	8,86E-02	2,6543016	5,18E-01	4,72E-01	7,60E-01	Dcun1d4	1,06	No
ENSMUSG00000051675	2,48E-01	3,209568118	5,73E+00	1,67E-02	1,35E-01	Trim32	1,19	No
ENSMUSG00000051682	-6,39E-01	0,993411477	9,98E+00	1,58E-03	2,32E-02	Treml4	0,64	Yes
ENSMUSG00000051695	3,77E-02	8,147708841	4,22E-01	5,16E-01	7,97E-01	Pcbp1	1,03	No
ENSMUSG00000051703	1,42E-01	1,433197935	6,08E-01	4,36E-01	7,28E-01	Tmem198	1,10	No
ENSMUSG00000051705	1,64E-01	2,552280385	1,73E+00	1,88E-01	5,50E-01	Senp8	1,12	No
ENSMUSG00000051716	-5,73E-02	-1,77045148	7,67E-03	9,30E-01	1,00E+00	Apon	0,96	No
ENSMUSG00000051721	2,00E-01	2,492098626	2,49E+00	1,14E-01	4,18E-01	Wdcp	1,15	No
ENSMUSG00000051723	-6,40E-02	4,277043312	5,82E-01	4,46E-01	7,37E-01	Rpl31-ps13	0,96	No

ENSMUSG00000051726	-1,94E+00	-3,107619243	1,50E+00	2,21E-01	5,50E-01	Kcnf1	0,26	No
ENSMUSG00000051727	7,78E-02	3,924266643	7,72E-01	3,80E-01	6,81E-01	Kctd14	1,06	No
ENSMUSG00000051728	-1,94E+00	-3,107619243	1,50E+00	2,21E-01	5,50E-01	4930563D23Rik	0,26	No
ENSMUSG00000051730	9,11E-02	3,817705202	9,68E-01	3,25E-01	6,26E-01	Mettl5	1,07	No
ENSMUSG00000051732	1,84E+00	-3,033649193	1,33E+00	2,49E-01	5,50E-01	Pabpc2	3,58	No
ENSMUSG00000051735	4,20E-02	5,943615494	4,11E-01	5,21E-01	8,00E-01	Rinl	1,03	No
ENSMUSG00000051736	-7,03E-02	0,04537418	6,36E-02	8,01E-01	9,61E-01	Fam229b	0,95	No
ENSMUSG00000051747	-2,07E-01	-0,246925354	4,07E-01	5,23E-01	8,01E-01	Ttn	0,87	No
ENSMUSG00000051748	-3,57E-01	5,263789541	2,47E+01	6,78E-07	3,14E-05	Wfdc21	0,78	Yes
ENSMUSG00000051768	4,74E-02	4,606590179	3,61E-01	5,48E-01	8,15E-01	Xrcc1	1,03	No
ENSMUSG00000051769	1,87E+00	-3,107639691	1,38E+00	2,40E-01	5,50E-01	Wfdc15a	3,67	No
ENSMUSG00000051786	1,80E-01	5,06762892	6,12E+00	1,34E-02	1,16E-01	Tubgcp6	1,13	No
ENSMUSG00000051790	-1,52E-01	2,96927859	1,80E+00	1,80E-01	5,42E-01	Nlgn2	0,90	No
ENSMUSG00000051805	-1,74E+00	-2,83197636	2,11E+00	1,47E-01	4,85E-01	A530095I07Rik	0,30	No
ENSMUSG00000051811	2,57E-01	1,070550171	1,50E+00	2,20E-01	5,50E-01	Cox6b2	1,20	No
ENSMUSG00000051817	2,81E-01	1,251699239	2,16E+00	1,42E-01	4,76E-01	Sox12	1,22	No
ENSMUSG00000051832	3,07E-01	5,225688441	1,81E+01	2,05E-05	6,36E-04	E230016K23Rik	1,24	Yes
ENSMUSG00000051839	2,65E+00	-3,033655087	2,73E+00	9,86E-02	3,91E-01	Gypa	6,30	No
ENSMUSG00000051844	-7,34E-02	0,187283117	7,41E-02	7,86E-01	9,52E-01	B230319C09Rik	0,95	No
ENSMUSG00000051853	4,36E-02	8,654512881	5,80E-01	4,46E-01	7,37E-01	Arf3	1,03	No
ENSMUSG00000051855	-1,82E-02	1,246036994	8,50E-03	9,27E-01	1,00E+00	Mest	0,99	No
ENSMUSG00000051860	-1,90E+00	-2,963239763	1,44E+00	2,31E-01	5,50E-01	Samd7	0,27	No
ENSMUSG00000051864	1,15E-01	6,159546467	3,17E+00	7,48E-02	3,48E-01	Tbc1d22a	1,08	No
ENSMUSG00000051890	-1,17E-01	1,726653081	5,17E-01	4,72E-01	7,60E-01	Klhdc1	0,92	No
ENSMUSG00000051896	2,65E+00	-3,033655087	2,73E+00	9,86E-02	3,91E-01	Tex37	6,30	No
ENSMUSG00000051900	-7,38E-02	-3,033659033	2,66E-03	9,59E-01	1,00E+00	Abca16	0,95	No
ENSMUSG00000051910	2,62E+00	-2,963288024	2,65E+00	1,03E-01	3,95E-01	Sox6	6,14	No
ENSMUSG00000051920	1,84E+00	-2,896131242	1,33E+00	2,49E-01	5,50E-01	Rspo2	3,58	No
ENSMUSG00000051934	-1,51E-01	2,701418686	1,59E+00	2,07E-01	5,50E-01	Spats2	0,90	No
ENSMUSG00000051940	1,87E+00	-3,107639691	1,38E+00	2,40E-01	5,50E-01	5031410I06Rik	3,67	No
ENSMUSG00000051950	6,64E-02	2,626991731	2,90E-01	5,90E-01	8,41E-01	B3glct	1,05	No
ENSMUSG00000051965	-1,25E+00	-2,655131058	1,23E+00	2,67E-01	5,64E-01	Nanos2	0,42	No

ENSMUSG00000051969	4,33E-01	3,1987563	1,62E+01	5,61E-05	1,50E-03	Tlr11	1,35	Yes
ENSMUSG00000051977	-8,57E-02	3,618992653	8,19E-01	3,65E-01	6,66E-01	Prdm9	0,94	No
ENSMUSG00000051978	-7,01E-02	3,156306124	4,20E-01	5,17E-01	7,98E-01	Erich1	0,95	No
ENSMUSG00000051980	-1,40E+00	0,358865407	2,84E+01	9,84E-08	5,52E-06	Casr	0,38	Yes
ENSMUSG00000051984	2,52E-01	0,712380554	1,16E+00	2,80E-01	5,77E-01	Sec31b	1,19	No
ENSMUSG00000051985	-3,83E-02	-3,107651936	-7,91E-11	1,00E+00	1,00E+00	Igfn1	0,97	No
ENSMUSG00000051989	-1,39E-01	2,893870069	1,43E+00	2,31E-01	5,50E-01	Smim11	0,91	No
ENSMUSG00000051998	-3,83E-02	-3,107651936	-7,91E-11	1,00E+00	1,00E+00	Lax1	0,97	No
ENSMUSG00000052005	1,55E+00	-2,146128176	3,60E+00	5,78E-02	2,97E-01	Gm9864	2,93	No
ENSMUSG00000052013	-1,37E-02	4,651509762	3,06E-02	8,61E-01	9,96E-01	Btla	0,99	No
ENSMUSG00000052026	-2,74E+00	-3,033596063	2,99E+00	8,38E-02	3,71E-01	Slc6a7	0,15	No
ENSMUSG00000052031	-2,68E-02	4,373842705	1,06E-01	7,44E-01	9,29E-01	Tagap1	0,98	No
ENSMUSG00000052033	2,08E-01	3,608259015	4,63E+00	3,14E-02	2,05E-01	Pfdn4	1,16	No
ENSMUSG00000052040	2,03E-01	7,482451417	1,17E+01	6,28E-04	1,11E-02	Klf13	1,15	Yes
ENSMUSG00000052056	-3,31E-01	7,121697145	3,03E+01	3,71E-08	2,26E-06	Zfp217	0,80	Yes
ENSMUSG00000052062	6,98E-03	-0,121835337	5,37E-04	9,82E-01	1,00E+00	Pard3b	1,00	No
ENSMUSG00000052085	-4,51E-02	8,217456992	6,09E-01	4,35E-01	7,28E-01	Dock8	0,97	No
ENSMUSG00000052087	7,15E-02	5,96111341	1,18E+00	2,77E-01	5,73E-01	Rgs14	1,05	No
ENSMUSG00000052102	1,30E-01	6,551357935	4,32E+00	3,78E-02	2,31E-01	Gnpda1	1,09	No
ENSMUSG00000052105	-6,39E-02	-2,963280242	1,86E-03	9,66E-01	1,00E+00	Mtcl1	0,96	No
ENSMUSG00000052117	-5,28E-01	3,438889335	2,94E+01	5,96E-08	3,49E-06	D630039A03Rik	0,69	Yes
ENSMUSG00000052125	-7,87E-01	5,031203312	1,16E+02	4,05E-27	1,73E-24	F730043M19Rik	0,58	Yes
ENSMUSG00000052131	-1,14E-02	-2,831959937	1,18E-04	9,91E-01	1,00E+00	Akr1b7	0,99	No
ENSMUSG00000052133	4,24E-02	-3,033592245	-1,17E-09	1,00E+00	1,00E+00	Sema5b	1,03	No
ENSMUSG00000052137	1,46E-02	3,648646477	2,41E-02	8,77E-01	1,00E+00	Rbm12b2	1,01	No
ENSMUSG00000052139	-1,65E-02	4,290196992	3,86E-02	8,44E-01	9,86E-01	Babam2	0,99	No
ENSMUSG00000052142	2,13E-01	6,649868062	1,17E+01	6,20E-04	1,10E-02	Rasal3	1,16	Yes
ENSMUSG00000052143	1,84E+00	-3,033649193	1,33E+00	2,49E-01	5,50E-01	Gm9869	3,58	No
ENSMUSG00000052144	-1,71E-02	5,556269123	6,26E-02	8,02E-01	9,62E-01	Ppp4r2	0,99	No
ENSMUSG00000052146	-2,14E-01	4,783902378	7,78E+00	5,30E-03	5,83E-02	Rps10	0,86	No
ENSMUSG00000052151	5,93E-02	5,566652183	7,47E-01	3,87E-01	6,87E-01	Plpp2	1,04	No
ENSMUSG00000052154	-1,63E-02	2,293910649	1,37E-02	9,07E-01	1,00E+00	Gm9870	0,99	No

ENSMUSG00000052155	-1,81E-01	4,247584045	4,48E+00	3,43E-02	2,18E-01	Acvr2a	0,88	No
ENSMUSG00000052160	1,73E-01	8,411123252	9,06E+00	2,61E-03	3,44E-02	Pld4	1,13	Yes
ENSMUSG00000052187	-1,30E+00	0,742829656	3,42E+01	5,01E-09	3,67E-07	Hbb-y	0,41	Yes
ENSMUSG00000052188	4,81E-01	-2,247093602	3,14E-01	5,75E-01	8,34E-01	Gm14964	1,40	No
ENSMUSG00000052192	-3,11E-01	6,682683038	2,53E+01	4,98E-07	2,37E-05	Gm5963	0,81	Yes
ENSMUSG00000052212	-4,21E-01	-0,641221178	1,35E+00	2,45E-01	5,50E-01	Cd177	0,75	No
ENSMUSG00000052214	9,02E-02	5,892942772	1,86E+00	1,73E-01	5,29E-01	Opa3	1,06	No
ENSMUSG00000052221	-3,83E-02	-3,107651936	-7,91E-11	1,00E+00	1,00E+00	Ppp1r36	0,97	No
ENSMUSG00000052229	4,49E-01	-2,522745037	2,15E-01	6,43E-01	8,71E-01	Gpr17	1,37	No
ENSMUSG00000052234	-1,54E+00	0,710275666	4,74E+01	5,79E-12	6,41E-10	Epx	0,34	Yes
ENSMUSG00000052236	-2,63E-02	-3,033641358	2,33E-04	9,88E-01	1,00E+00	Gm9871	0,98	No
ENSMUSG00000052241	8,49E-02	-0,648229819	5,49E-02	8,15E-01	9,68E-01	A930035D04Rik	1,06	No
ENSMUSG00000052248	2,19E-01	3,385379005	4,46E+00	3,47E-02	2,20E-01	Zeb2os	1,16	No
ENSMUSG00000052253	-1,30E-01	6,561742974	4,31E+00	3,79E-02	2,31E-01	Zfp622	0,91	No
ENSMUSG00000052270	-2,90E-02	4,733096938	1,38E-01	7,10E-01	9,09E-01	Fpr2	0,98	No
ENSMUSG00000052271	-3,53E-01	-0,697150599	7,38E-01	3,90E-01	6,90E-01	Bhlha15	0,78	No
ENSMUSG00000052273	-1,46E+00	-2,522701177	2,20E+00	1,38E-01	4,69E-01	Dnah3	0,36	No
ENSMUSG00000052276	-1,89E+00	-3,1076441	1,42E+00	2,33E-01	5,50E-01	Ostn	0,27	No
ENSMUSG00000052281	5,94E-02	-0,548223089	2,75E-02	8,68E-01	1,00E+00	Tspan32os	1,04	No
ENSMUSG00000052291	-1,43E-01	1,109981646	5,10E-01	4,75E-01	7,63E-01	5330438D12Rik	0,91	No
ENSMUSG00000052293	-9,92E-02	4,583948134	1,55E+00	2,13E-01	5,50E-01	Taf9	0,93	No
ENSMUSG00000052295	3,14E-02	-3,107617333	-7,69E-10	1,00E+00	1,00E+00	8030423F21Rik	1,02	No
ENSMUSG00000052296	9,72E-02	7,492230406	2,68E+00	1,01E-01	3,94E-01	Ppp6r1	1,07	No
ENSMUSG00000052298	6,92E-02	7,9729714	1,41E+00	2,35E-01	5,50E-01	Cdc42se2	1,05	No
ENSMUSG00000052299	1,31E-01	6,213834714	4,14E+00	4,19E-02	2,43E-01	Ltn1	1,09	No
ENSMUSG00000052301	1,05E-01	3,286794678	9,97E-01	3,18E-01	6,19E-01	Doc2a	1,08	No
ENSMUSG00000052302	-8,27E-02	1,50233689	2,29E-01	6,32E-01	8,64E-01	Tbc1d30	0,94	No
ENSMUSG00000052303	1,87E+00	-3,107639691	1,38E+00	2,40E-01	5,50E-01	Mrgpra6	3,67	No
ENSMUSG00000052305	-8,45E-01	-0,037843934	7,44E+00	6,39E-03	6,72E-02	Hbb-bs	0,56	No
ENSMUSG00000052310	5,73E-02	7,272342493	9,18E-01	3,38E-01	6,38E-01	Slc39a1	1,04	No
ENSMUSG00000052316	-7,38E-02	-3,033634644	2,66E-03	9,59E-01	1,00E+00	Lrrc15	0,95	No
ENSMUSG00000052331	-2,03E-01	7,418562188	1,17E+01	6,24E-04	1,10E-02	Ankrd44	0,87	Yes

ENSMUSG00000052336	1,03E-02	-0,479146822	9,30E-04	9,76E-01	1,00E+00	Cx3cr1	1,01	No
ENSMUSG00000052337	7,33E-02	6,827983697	1,43E+00	2,32E-01	5,50E-01	Immt	1,05	No
ENSMUSG00000052353	-1,75E+00	-2,770579957	2,13E+00	1,44E-01	4,80E-01	Cemip	0,30	No
ENSMUSG00000052363	1,87E+00	-3,033632735	1,36E+00	2,43E-01	5,50E-01	Zdhhc19	3,66	No
ENSMUSG00000052368	-2,18E+00	-2,355535296	6,16E+00	1,31E-02	1,14E-01	Gm9873	0,22	No
ENSMUSG00000052369	-7,88E-02	4,068748055	8,16E-01	3,66E-01	6,67E-01	Tmem106c	0,95	No
ENSMUSG00000052373	4,35E-01	-2,600703706	1,48E-01	7,01E-01	9,05E-01	Mpp3	1,35	No
ENSMUSG00000052374	7,98E-01	-2,548208861	5,54E-01	4,57E-01	7,46E-01	Actn2	1,74	No
ENSMUSG00000052384	1,24E-01	8,67523828	4,68E+00	3,05E-02	2,01E-01	Nrros	1,09	No
ENSMUSG00000052387	-8,78E-01	-1,600733606	2,60E+00	1,07E-01	4,00E-01	Trpm3	0,54	No
ENSMUSG00000052392	-8,05E-01	-1,401216321	2,32E+00	1,28E-01	4,49E-01	Acot4	0,57	No
ENSMUSG00000052395	8,37E-02	4,434744624	1,07E+00	3,00E-01	5,96E-01	Rft1	1,06	No
ENSMUSG00000052396	-7,17E-02	-2,448702469	5,88E-03	9,39E-01	1,00E+00	Mogat2	0,95	No
ENSMUSG00000052397	7,90E-02	8,022510044	1,84E+00	1,75E-01	5,33E-01	Ezr	1,06	No
ENSMUSG00000052403	-2,08E-01	-0,793441667	2,81E-01	5,96E-01	8,45E-01	Fcnaos	0,87	No
ENSMUSG00000052406	-4,92E-02	5,520762923	5,13E-01	4,74E-01	7,62E-01	Rexo4	0,97	No
ENSMUSG00000052407	1,55E-01	2,101942027	1,13E+00	2,87E-01	5,83E-01	Ccdc171	1,11	No
ENSMUSG00000052414	4,43E-02	2,290640289	1,06E-01	7,44E-01	9,29E-01	Gm28047	1,03	No
ENSMUSG00000052415	3,47E-01	3,115501964	1,02E+01	1,42E-03	2,14E-02	Tchh	1,27	Yes
ENSMUSG00000052419	2,31E-02	6,030962901	1,26E-01	7,23E-01	9,17E-01	2610001J05Rik	1,02	No
ENSMUSG00000052423	2,56E-02	4,519380763	1,03E-01	7,48E-01	9,30E-01	B4galt3	1,02	No
ENSMUSG00000052426	-7,70E-02	-2,8010042	4,04E-03	9,49E-01	1,00E+00	Gm9877	0,95	No
ENSMUSG00000052428	9,45E-02	5,892535225	2,03E+00	1,54E-01	4,97E-01	Tmco1	1,07	No
ENSMUSG00000052430	-1,22E+00	-2,711725084	1,43E+00	2,32E-01	5,50E-01	Bmpr1b	0,43	No
ENSMUSG00000052435	-4,98E-01	0,947733331	5,71E+00	1,69E-02	1,36E-01	Cebpe	0,71	No
ENSMUSG00000052446	8,67E-02	3,247654585	7,05E-01	4,01E-01	6,99E-01	Zfp961	1,06	No
ENSMUSG00000052456	-2,87E-02	6,230622218	2,00E-01	6,55E-01	8,79E-01	Asna1	0,98	No
ENSMUSG00000052459	9,26E-02	8,1750059	2,56E+00	1,10E-01	4,06E-01	Atp6v1a	1,07	No
ENSMUSG00000052468	1,08E+00	-3,033648223	5,01E-01	4,79E-01	7,66E-01	Pmp2	2,11	No
ENSMUSG00000052469	-8,65E-02	-2,497642005	8,60E-03	9,26E-01	1,00E+00	Tcp10c	0,94	No
ENSMUSG00000052471	-2,63E-02	-3,033641358	2,33E-04	9,88E-01	1,00E+00	Gm9881	0,98	No
ENSMUSG00000052477	-5,82E-01	3,135779597	2,83E+01	1,03E-07	5,70E-06	C130026I21Rik	0,67	Yes

ENSMUSG00000052479	2,65E+00	-3,033655087	2,73E+00	9,86E-02	3,91E-01	A330008L17Rik	6,30	No
ENSMUSG00000052482	3,78E-03	-1,655104728	4,47E-05	9,95E-01	1,00E+00	Gm8174	1,00	No
ENSMUSG00000052485	2,76E-01	0,721281317	1,37E+00	2,41E-01	5,50E-01	Tmem171	1,21	No
ENSMUSG00000052488	3,69E-02	6,553222719	3,49E-01	5,55E-01	8,20E-01	Cherp	1,03	No
ENSMUSG00000052504	2,65E+00	-2,831982301	2,71E+00	9,98E-02	3,92E-01	Epha3	6,28	No
ENSMUSG00000052512	-2,37E-01	3,197230269	4,83E+00	2,79E-02	1,89E-01	Nav2	0,85	No
ENSMUSG00000052516	1,24E+00	-2,448693055	9,49E-01	3,30E-01	6,30E-01	Robo2	2,36	No
ENSMUSG00000052525	9,31E-02	0,382587132	1,37E-01	7,11E-01	9,10E-01	Spdya	1,07	No
ENSMUSG00000052533	1,56E-01	5,682824754	5,27E+00	2,16E-02	1,58E-01	Nup188	1,11	No
ENSMUSG00000052534	2,39E-01	3,304133642	5,07E+00	2,44E-02	1,72E-01	Pbx1	1,18	No
ENSMUSG00000052539	1,57E-01	2,454487232	1,41E+00	2,35E-01	5,50E-01	Magi3	1,11	No
ENSMUSG00000052544	-4,66E-01	-0,535287953	1,68E+00	1,95E-01	5,50E-01	St6galnac3	0,72	No
ENSMUSG00000052551	1,59E+00	-2,770645867	1,74E+00	1,87E-01	5,50E-01	Adarb2	3,02	No
ENSMUSG00000052557	8,61E-02	5,515748368	1,57E+00	2,11E-01	5,50E-01	Gan	1,06	No
ENSMUSG00000052560	-2,57E-01	3,757919653	7,52E+00	6,09E-03	6,49E-02	Cpne8	0,84	No
ENSMUSG00000052563	2,56E-01	3,127330639	5,53E+00	1,87E-02	1,45E-01	D930048N14Rik	1,19	No
ENSMUSG00000052565	1,07E-03	2,656802159	7,35E-05	9,93E-01	1,00E+00	Hist1h1d	1,00	No
ENSMUSG00000052566	-2,18E-01	3,403179525	4,66E+00	3,09E-02	2,03E-01	Hook2	0,86	No
ENSMUSG00000052572	-6,45E-02	-2,548244769	3,79E-03	9,51E-01	1,00E+00	Dlg2	0,96	No
ENSMUSG00000052584	1,89E+00	-2,711792085	2,66E+00	1,03E-01	3,95E-01	Serp2	3,70	No
ENSMUSG00000052593	9,41E-02	7,544088519	2,53E+00	1,12E-01	4,11E-01	Adam17	1,07	No
ENSMUSG00000052595	-8,34E-01	-2,963223097	3,81E-01	5,37E-01	8,09E-01	A1cf	0,56	No
ENSMUSG00000052605	2,77E-02	2,29558518	3,98E-02	8,42E-01	9,85E-01	Isoc2b	1,02	No
ENSMUSG00000052609	3,78E-01	5,246992625	2,76E+01	1,50E-07	8,02E-06	Plekhg3	1,30	Yes
ENSMUSG00000052613	8,14E-01	-0,311257626	5,79E+00	1,61E-02	1,32E-01	Pcdh15	1,76	No
ENSMUSG00000052616	-5,79E-01	-2,711686467	2,63E-01	6,08E-01	8,51E-01	Terb1	0,67	No
ENSMUSG00000052631	2,00E-01	-1,35547271	1,12E-01	7,38E-01	9,26E-01	Sh2d6	1,15	No
ENSMUSG00000052632	-1,28E-01	4,704208825	2,78E+00	9,54E-02	3,86E-01	Asap2	0,91	No
ENSMUSG00000052656	7,00E-02	5,094726885	9,35E-01	3,34E-01	6,34E-01	Rnf103	1,05	No
ENSMUSG00000052658	2,24E-01	-2,185665995	6,34E-02	8,01E-01	9,61E-01	5830454E08Rik	1,17	No
ENSMUSG00000052673	9,94E-02	3,832057775	1,16E+00	2,80E-01	5,77E-01	Gm9887	1,07	No
ENSMUSG00000052675	-3,23E-02	1,175768964	2,57E-02	8,73E-01	1,00E+00	Zfp112	0,98	No

ENSMUSG00000052681	-2,71E-03	8,274894269	2,20E-03	9,63E-01	1,00E+00	Rap1b	1,00	No
ENSMUSG00000052684	4,49E-01	6,499197323	5,13E+01	7,97E-13	9,83E-11	Jun	1,37	Yes
ENSMUSG00000052688	3,59E-01	6,364337089	3,18E+01	1,69E-08	1,11E-06	Rab7b	1,28	Yes
ENSMUSG00000052698	3,72E-01	4,917950236	2,39E+01	1,01E-06	4,49E-05	Tln2	1,29	Yes
ENSMUSG00000052707	5,27E-03	6,55937428	7,16E-03	9,33E-01	1,00E+00	Tnrc6a	1,00	No
ENSMUSG00000052712	1,11E-02	5,656092807	2,65E-02	8,71E-01	1,00E+00	BC004004	1,01	No
ENSMUSG00000052713	7,29E-02	5,209002112	9,89E-01	3,20E-01	6,20E-01	Zfp608	1,05	No
ENSMUSG00000052724	-4,20E-02	3,10566043	1,40E-01	7,08E-01	9,09E-01	Gm9888	0,97	No
ENSMUSG00000052727	-3,46E-01	-0,126743029	1,31E+00	2,53E-01	5,50E-01	Map1b	0,79	No
ENSMUSG00000052730	4,60E-01	-2,711710286	1,65E-01	6,84E-01	8,96E-01	Gm5111	1,38	No
ENSMUSG00000052736	1,22E+00	-2,770593231	9,26E-01	3,36E-01	6,37E-01	Klrc2	2,33	No
ENSMUSG00000052738	-6,01E-02	5,742700199	7,98E-01	3,72E-01	6,73E-01	Suclg1	0,96	No
ENSMUSG00000052748	-1,05E-01	3,29641596	1,04E+00	3,08E-01	6,07E-01	Swt1	0,93	No
ENSMUSG00000052749	5,96E-02	2,097482474	1,75E-01	6,76E-01	8,91E-01	Trim30b	1,04	No
ENSMUSG00000052751	2,42E-03	3,436077892	5,96E-04	9,81E-01	1,00E+00	Repin1	1,00	No
ENSMUSG00000052752	1,02E-02	6,343496284	2,60E-02	8,72E-01	1,00E+00	Traf7	1,01	No
ENSMUSG00000052759	1,81E+00	-3,10765737	1,28E+00	2,58E-01	5,50E-01	Gpr25	3,49	No
ENSMUSG00000052760	8,31E-02	4,615225056	1,13E+00	2,88E-01	5,84E-01	A630001G21Rik	1,06	No
ENSMUSG00000052763	4,66E-02	4,391692987	3,27E-01	5,67E-01	8,29E-01	Zfp212	1,03	No
ENSMUSG00000052769	3,14E-02	-3,107617333	-7,69E-10	1,00E+00	1,00E+00	Gm9889	1,02	No
ENSMUSG00000052776	-1,88E-03	5,259662199	6,99E-04	9,79E-01	1,00E+00	Oas1a	1,00	No
ENSMUSG00000052783	4,37E-02	0,856041832	4,33E-02	8,35E-01	9,80E-01	Grk4	1,03	No
ENSMUSG00000052794	4,79E-02	3,701293593	2,58E-01	6,12E-01	8,52E-01	1700030K09Rik	1,03	No
ENSMUSG00000052798	3,62E-02	4,417794582	1,96E-01	6,58E-01	8,81E-01	Nup107	1,03	No
ENSMUSG00000052812	2,44E-02	6,120477385	1,42E-01	7,07E-01	9,08E-01	Atad2b	1,02	No
ENSMUSG00000052819	-2,63E-02	-3,033653157	2,33E-04	9,88E-01	1,00E+00	Best2	0,98	No
ENSMUSG00000052825	-3,18E-02	6,247278267	2,47E-01	6,19E-01	8,56E-01	Gm9892	0,98	No
ENSMUSG00000052833	1,00E-01	6,70317141	2,63E+00	1,05E-01	3,97E-01	Sae1	1,07	No
ENSMUSG00000052837	7,54E-02	8,174782596	1,68E+00	1,94E-01	5,50E-01	Junb	1,05	No
ENSMUSG00000052850	-3,95E-02	-2,963274761	7,08E-04	9,79E-01	1,00E+00	Tas2r137	0,97	No
ENSMUSG00000052852	-7,50E-01	-0,300276367	5,13E+00	2,35E-02	1,68E-01	Reep1	0,59	No
ENSMUSG00000052861	-5,63E-01	-0,038113397	3,67E+00	5,53E-02	2,88E-01	Dnah6	0,68	No

ENSMUSG00000052865	2,65E+00	-3,033664155	2,71E+00	9,96E-02	3,92E-01	Gm13619	6,26	No
ENSMUSG00000052889	-5,54E-03	7,200974668	8,47E-03	9,27E-01	1,00E+00	Prkcb	1,00	No
ENSMUSG00000052906	-8,52E-02	4,085746081	9,56E-01	3,28E-01	6,29E-01	Ubxn8	0,94	No
ENSMUSG00000052909	-3,24E+00	-2,770558382	4,41E+00	3,57E-02	2,24E-01	Gm9894	0,11	No
ENSMUSG00000052911	4,21E-01	0,616226105	2,85E+00	9,13E-02	3,82E-01	Lamb2	1,34	No
ENSMUSG00000052912	2,34E-02	3,721543616	6,19E-02	8,03E-01	9,62E-01	Smarca5-ps	1,02	No
ENSMUSG00000052914	3,42E-01	1,330491608	3,24E+00	7,20E-02	3,40E-01	Cyp2j6	1,27	No
ENSMUSG00000052915	3,79E-02	6,557071158	3,71E-01	5,43E-01	8,12E-01	Msl1	1,03	No
ENSMUSG00000052917	-1,65E-01	4,534051886	4,36E+00	3,67E-02	2,28E-01	Senp7	0,89	No
ENSMUSG00000052920	-2,03E+00	-2,600638559	3,11E+00	7,79E-02	3,56E-01	Prkg1	0,25	No
ENSMUSG00000052921	-4,58E-02	-2,60063022	1,91E-03	9,65E-01	1,00E+00	Arhgef15	0,97	No
ENSMUSG00000052922	-1,87E+00	-3,107637761	1,39E+00	2,38E-01	5,50E-01	Bpi	0,27	No
ENSMUSG00000052926	-1,21E-01	4,539563485	2,30E+00	1,29E-01	4,51E-01	Rnaseh2a	0,92	No
ENSMUSG00000052928	4,55E-02	3,518174431	2,09E-01	6,48E-01	8,75E-01	Ctif	1,03	No
ENSMUSG00000052934	2,13E-01	4,235431866	6,32E+00	1,19E-02	1,06E-01	Fbxo31	1,16	No
ENSMUSG00000052942	-1,02E-01	0,095591224	1,29E-01	7,19E-01	9,15E-01	Glis3	0,93	No
ENSMUSG00000052949	2,62E-01	2,845969709	4,81E+00	2,83E-02	1,91E-01	Rnf157	1,20	No
ENSMUSG00000052955	3,00E-01	-2,497613611	9,14E-02	7,62E-01	9,38E-01	Cpvl	1,23	No
ENSMUSG00000052957	-9,97E-01	-2,088709893	1,70E+00	1,92E-01	5,50E-01	Gas1	0,50	No
ENSMUSG00000052962	-1,01E-01	4,13944812	1,37E+00	2,42E-01	5,50E-01	Mrpl35	0,93	No
ENSMUSG00000052974	3,14E-02	-3,107617333	-7,69E-10	1,00E+00	1,00E+00	Cyp2f2	1,02	No
ENSMUSG00000052981	-7,21E-01	-1,246999339	2,43E+00	1,19E-01	4,30E-01	Ube2ql1	0,61	No
ENSMUSG00000052997	4,37E-02	6,236862907	4,63E-01	4,96E-01	7,82E-01	Uba2	1,03	No
ENSMUSG00000053004	5,15E-01	2,287894519	1,40E+01	1,81E-04	3,99E-03	Hrh1	1,43	Yes
ENSMUSG00000053007	1,52E-01	7,208305448	6,40E+00	1,14E-02	1,03E-01	Creb5	1,11	No
ENSMUSG00000053012	2,77E-02	6,011916976	1,80E-01	6,71E-01	8,89E-01	Krcc1	1,02	No
ENSMUSG00000053024	-8,32E-01	-2,896102534	3,79E-01	5,38E-01	8,09E-01	Cntn2	0,56	No
ENSMUSG00000053025	3,13E-01	1,13803527	2,62E+00	1,05E-01	3,98E-01	Sv2b	1,24	No
ENSMUSG00000053030	6,91E-01	0,742347295	8,54E+00	3,48E-03	4,27E-02	Spink2	1,61	Yes
ENSMUSG00000053038	-1,21E-01	9,269848717	4,58E+00	3,24E-02	2,10E-01	Gm6180	0,92	No
ENSMUSG00000053040	1,05E-01	6,125082571	2,63E+00	1,05E-01	3,97E-01	Aph1c	1,08	No
ENSMUSG00000053046	-5,83E-01	-2,770617645	2,67E-01	6,05E-01	8,49E-01	Brsk2	0,67	No

ENSMUSG00000053049	-3,54E+00	-2,896134066	5,62E+00	1,77E-02	1,39E-01	Gm15413	0,09	No
ENSMUSG00000053062	-2,81E-01	-0,069886797	9,95E-01	3,19E-01	6,19E-01	Jam2	0,82	No
ENSMUSG00000053063	7,06E-02	5,79902297	1,11E+00	2,92E-01	5,87E-01	Clec12a	1,05	No
ENSMUSG00000053070	-2,63E-02	-3,033653157	2,33E-04	9,88E-01	1,00E+00	9230110C19Rik	0,98	No
ENSMUSG00000053080	-2,93E-01	1,394339265	2,84E+00	9,18E-02	3,82E-01	2700081O15Rik	0,82	No
ENSMUSG00000053081	-2,48E-01	-0,808634234	4,05E-01	5,24E-01	8,01E-01	1700069B07Rik	0,84	No
ENSMUSG00000053091	1,41E-01	3,568441676	2,05E+00	1,52E-01	4,94E-01	Lins1	1,10	No
ENSMUSG00000053093	-5,94E-01	-0,309339536	3,38E+00	6,62E-02	3,23E-01	Myh7	0,66	No
ENSMUSG00000053094	-2,02E-02	5,910510905	9,28E-02	7,61E-01	9,37E-01	Tmem248	0,99	No
ENSMUSG00000053101	3,66E-02	6,959182365	3,63E-01	5,47E-01	8,14E-01	Gpr141	1,03	No
ENSMUSG00000053110	3,11E-01	-1,247143371	4,25E-01	5,14E-01	7,96E-01	Yap1	1,24	No
ENSMUSG00000053111	-9,10E-01	-2,770628777	7,21E-01	3,96E-01	6,96E-01	Fank1	0,53	No
ENSMUSG00000053113	5,01E-01	7,123842418	6,83E+01	1,42E-16	2,49E-14	Socs3	1,41	Yes
ENSMUSG00000053117	7,46E-01	-2,770577216	3,02E-01	5,82E-01	8,38E-01	E330013P04Rik	1,68	No
ENSMUSG00000053119	1,11E-02	6,098345231	2,94E-02	8,64E-01	9,97E-01	Chmp3	1,01	No
ENSMUSG00000053121	1,60E+00	-2,71177803	1,75E+00	1,85E-01	5,49E-01	Gm5129	3,03	No
ENSMUSG00000053128	2,11E-01	4,482032685	6,81E+00	9,09E-03	8,70E-02	Rnf26	1,16	No
ENSMUSG00000053134	-5,28E-02	4,696932562	4,65E-01	4,95E-01	7,81E-01	Supt7l	0,96	No
ENSMUSG00000053137	5,03E-01	-0,156097472	2,76E+00	9,64E-02	3,88E-01	Mapk11	1,42	No
ENSMUSG00000053158	1,35E-01	7,971902395	5,36E+00	2,06E-02	1,54E-01	Fes	1,10	No
ENSMUSG00000053164	9,01E-01	-1,49778074	2,60E+00	1,07E-01	4,00E-01	Gpr21	1,87	No
ENSMUSG00000053166	-6,40E-02	-1,401393187	1,82E-02	8,93E-01	1,00E+00	Cdh22	0,96	No
ENSMUSG00000053168	-3,57E+00	-2,711628139	5,73E+00	1,66E-02	1,34E-01	9030619P08Rik	0,08	No
ENSMUSG00000053173	-1,42E-01	7,650331484	5,82E+00	1,58E-02	1,31E-01	Rpl18-ps2	0,91	No
ENSMUSG00000053175	-2,13E-01	4,051712587	6,07E+00	1,37E-02	1,18E-01	Bcl3	0,86	No
ENSMUSG00000053178	-7,65E-01	-0,146047994	6,51E+00	1,07E-02	9,83E-02	Mterf1b	0,59	No
ENSMUSG00000053181	-9,13E-02	-2,226361631	1,15E-02	9,15E-01	1,00E+00	A830005F24Rik	0,94	No
ENSMUSG00000053182	8,28E-02	-0,719064943	4,70E-02	8,28E-01	9,76E-01	Gm609	1,06	No
ENSMUSG00000053184	1,81E+00	-3,10765737	1,28E+00	2,58E-01	5,50E-01	Spaca3	3,49	No
ENSMUSG00000053192	1,49E-01	3,489467559	2,30E+00	1,29E-01	4,51E-01	Mllt11	1,11	No
ENSMUSG00000053198	-1,90E-01	0,464654676	5,75E-01	4,48E-01	7,38E-01	Prx	0,88	No
ENSMUSG00000053199	1,81E+00	-3,10765737	1,28E+00	2,58E-01	5,50E-01	Arhgap20	3,49	No

ENSMUSG00000053205	4,46E-01	0,495967738	2,86E+00	9,10E-02	3,82E-01	Styx	1,36	No
ENSMUSG00000053208	1,85E-02	3,196295326	2,96E-02	8,63E-01	9,97E-01	Kat2b-ps	1,01	No
ENSMUSG00000053216	-8,07E-01	-1,015422256	3,33E+00	6,81E-02	3,28E-01	Btn2a2	0,57	No
ENSMUSG00000053219	1,96E-01	0,306280148	5,09E-01	4,76E-01	7,64E-01	Raet1e	1,15	No
ENSMUSG00000053226	1,31E-01	1,638631771	5,81E-01	4,46E-01	7,37E-01	Dand5	1,09	No
ENSMUSG00000053251	-2,81E-02	-2,963245656	3,59E-04	9,85E-01	1,00E+00	Olfr212	0,98	No
ENSMUSG00000053253	1,55E-01	5,467095166	4,98E+00	2,56E-02	1,78E-01	Ndfip2	1,11	No
ENSMUSG00000053263	7,48E-03	0,959368788	1,32E-03	9,71E-01	1,00E+00	Gm12592	1,01	No
ENSMUSG00000053279	1,85E+00	-2,963253488	1,34E+00	2,48E-01	5,50E-01	Aldh1a1	3,59	No
ENSMUSG00000053286	-4,63E-02	4,617843298	3,56E-01	5,51E-01	8,17E-01	Trmt1l	0,97	No
ENSMUSG00000053289	8,35E-02	4,379423547	1,01E+00	3,15E-01	6,15E-01	Ddx10	1,06	No
ENSMUSG00000053291	2,35E-02	4,438025862	8,34E-02	7,73E-01	9,44E-01	Rab4b	1,02	No
ENSMUSG00000053293	6,01E-02	6,226585938	8,74E-01	3,50E-01	6,51E-01	Pom121	1,04	No
ENSMUSG00000053297	-6,69E-01	-1,497471164	1,54E+00	2,15E-01	5,50E-01	AI854703	0,63	No
ENSMUSG00000053310	-2,58E-01	-1,378329702	2,33E-01	6,29E-01	8,62E-01	Nrgn	0,84	No
ENSMUSG00000053317	-3,78E-01	5,595468564	3,09E+01	2,76E-08	1,74E-06	Sec61b	0,77	Yes
ENSMUSG00000053318	-1,13E-01	4,914376935	2,31E+00	1,29E-01	4,50E-01	Slamf8	0,92	No
ENSMUSG00000053329	6,00E-02	5,05851377	6,62E-01	4,16E-01	7,11E-01	D10Jhu81e	1,04	No
ENSMUSG00000053332	-1,07E-02	4,723058833	1,89E-02	8,91E-01	1,00E+00	Gas5	0,99	No
ENSMUSG00000053333	3,04E-02	4,051134657	1,21E-01	7,28E-01	9,20E-01	Dis3l2	1,02	No
ENSMUSG00000053334	4,88E-02	3,409842441	2,23E-01	6,37E-01	8,68E-01	Ficd	1,03	No
ENSMUSG00000053338	1,94E-01	6,882309378	1,00E+01	1,55E-03	2,29E-02	Tarm1	1,14	Yes
ENSMUSG00000053347	-2,19E-02	3,333011249	4,55E-02	8,31E-01	9,78E-01	Zfp943	0,98	No
ENSMUSG00000053353	5,16E-01	-1,89615453	6,38E-01	4,24E-01	7,19E-01	2310001K24Rik	1,43	No
ENSMUSG00000053358	3,32E-01	1,614482017	3,65E+00	5,61E-02	2,91E-01	Gm9905	1,26	No
ENSMUSG00000053360	1,26E-01	-1,896122591	3,12E-02	8,60E-01	9,95E-01	F730035P03Rik	1,09	No
ENSMUSG00000053368	-1,89E+00	-3,1076441	1,42E+00	2,33E-01	5,50E-01	Rxfp2	0,27	No
ENSMUSG00000053390	9,32E-02	3,548983398	9,21E-01	3,37E-01	6,38E-01	Zfp952	1,07	No
ENSMUSG00000053395	2,85E-01	3,095508218	6,66E+00	9,87E-03	9,24E-02	Cacng8	1,22	No
ENSMUSG00000053398	1,21E-01	3,120305784	1,21E+00	2,72E-01	5,68E-01	Phgdh	1,09	No
ENSMUSG00000053411	-6,93E-01	4,561970414	7,76E+01	1,26E-18	2,58E-16	Cbx7	0,62	Yes
ENSMUSG00000053414	1,85E+00	-2,770563564	1,34E+00	2,46E-01	5,50E-01	Hunk	3,61	No

ENSMUSG00000053436	1,28E-01	7,396101028	4,60E+00	3,20E-02	2,08E-01	Mapk14	1,09	No
ENSMUSG00000053442	5,39E-01	-0,419117366	2,16E+00	1,41E-01	4,75E-01	4930597O21Rik	1,45	No
ENSMUSG00000053453	-1,90E-01	4,336436385	5,24E+00	2,21E-02	1,61E-01	Thoc7	0,88	No
ENSMUSG00000053460	1,91E-01	3,580434576	3,86E+00	4,95E-02	2,69E-01	Ggcx	1,14	No
ENSMUSG00000053461	3,17E+00	-2,963263597	4,11E+00	4,28E-02	2,46E-01	Hhipl2	8,99	No
ENSMUSG00000053465	-1,87E+00	-3,033619006	1,41E+00	2,36E-01	5,50E-01	Hs6st3	0,27	No
ENSMUSG00000053469	3,21E-01	0,214191703	1,22E+00	2,69E-01	5,65E-01	Tg	1,25	No
ENSMUSG00000053470	1,55E-01	5,737288859	5,33E+00	2,09E-02	1,56E-01	Kdm3a	1,11	No
ENSMUSG00000053472	-2,21E-01	0,036682277	5,80E-01	4,46E-01	7,37E-01	Gm13534	0,86	No
ENSMUSG00000053475	2,60E-01	-1,963247151	1,56E-01	6,93E-01	9,01E-01	Tnfaip6	1,20	No
ENSMUSG00000053477	-8,05E-02	5,341788658	1,31E+00	2,53E-01	5,50E-01	Tcf4	0,95	No
ENSMUSG00000053483	-2,31E-03	4,673312353	8,83E-04	9,76E-01	1,00E+00	Usp21	1,00	No
ENSMUSG00000053508	-3,88E-03	-3,03362684	4,02E-12	1,00E+00	1,00E+00	BC048502	1,00	No
ENSMUSG00000053510	6,22E-02	7,113113094	1,06E+00	3,03E-01	5,99E-01	Nrd1	1,04	No
ENSMUSG00000053522	1,60E+00	-2,832054469	1,75E+00	1,86E-01	5,50E-01	Lgals7	3,03	No
ENSMUSG00000053536	-2,50E-02	5,633024081	1,36E-01	7,12E-01	9,11E-01	Cstf2t	0,98	No
ENSMUSG00000053541	3,97E-01	-1,587454969	4,72E-01	4,92E-01	7,78E-01	Gm4759	1,32	No
ENSMUSG00000053550	-6,07E-02	-2,548174731	4,20E-03	9,48E-01	1,00E+00	Shisa7	0,96	No
ENSMUSG00000053552	-1,34E+00	-2,832015733	1,12E+00	2,89E-01	5,85E-01	Ebf4	0,40	No
ENSMUSG00000053553	1,16E-01	5,339567183	2,67E+00	1,02E-01	3,94E-01	3110082I17Rik	1,08	No
ENSMUSG00000053559	1,94E-02	1,432378234	1,09E-02	9,17E-01	1,00E+00	Smagp	1,01	No
ENSMUSG00000053560	3,93E-01	6,055406418	3,64E+01	1,58E-09	1,25E-07	Ier2	1,31	Yes
ENSMUSG00000053565	-1,52E-01	5,915250379	5,29E+00	2,14E-02	1,58E-01	Eif3k	0,90	No
ENSMUSG00000053574	-2,97E-01	-2,165718441	1,42E-01	7,06E-01	9,08E-01	4930563E22Rik	0,81	No
ENSMUSG00000053580	5,67E-03	4,362843916	4,63E-03	9,46E-01	1,00E+00	Tanc2	1,00	No
ENSMUSG00000053581	6,77E-03	6,836789884	1,22E-02	9,12E-01	1,00E+00	Zfand2a	1,00	No
ENSMUSG00000053588	-5,43E-02	-0,131504938	3,25E-02	8,57E-01	9,94E-01	A730061H03Rik	0,96	No
ENSMUSG00000053600	4,81E-03	4,021353103	2,95E-03	9,57E-01	1,00E+00	Zfp472	1,00	No
ENSMUSG00000053603	-5,86E-01	-1,614027396	9,50E-01	3,30E-01	6,30E-01	4930442H23Rik	0,67	No
ENSMUSG00000053604	1,86E-02	4,129880356	4,52E-02	8,32E-01	9,78E-01	Rpia	1,01	No
ENSMUSG00000053617	3,66E-02	4,191321846	1,82E-01	6,70E-01	8,88E-01	Sh3pxd2a	1,03	No
ENSMUSG00000053641	-3,70E-01	6,520938915	3,48E+01	3,58E-09	2,68E-07	Dennd4a	0,77	Yes

ENSMUSG00000053644	-1,20E-03	2,682480253	9,44E-05	9,92E-01	1,00E+00	Aldh7a1	1,00	No
ENSMUSG00000053646	-5,86E-02	-2,448699739	3,14E-03	9,55E-01	1,00E+00	Plxnb1	0,96	No
ENSMUSG00000053647	6,56E-01	-0,366908781	3,20E+00	7,38E-02	3,45E-01	Gper1	1,58	No
ENSMUSG00000053656	-8,01E-01	-0,497341809	4,84E+00	2,78E-02	1,88E-01	Dancr	0,57	No
ENSMUSG00000053675	-7,39E-01	-2,497628296	6,12E-01	4,34E-01	7,27E-01	Tgm5	0,60	No
ENSMUSG00000053684	2,83E-02	3,387895918	7,51E-02	7,84E-01	9,51E-01	BC048403	1,02	No
ENSMUSG00000053687	-3,17E-02	7,884048621	2,93E-01	5,88E-01	8,40E-01	Dpep2	0,98	No
ENSMUSG00000053693	1,48E-01	2,746992866	1,55E+00	2,14E-01	5,50E-01	Mast1	1,11	No
ENSMUSG00000053702	-1,04E+00	-1,226184515	5,24E+00	2,20E-02	1,61E-01	Nebl	0,49	No
ENSMUSG00000053706	-1,57E-01	5,309997557	5,03E+00	2,49E-02	1,74E-01	B430305J03Rik	0,90	No
ENSMUSG00000053714	-6,04E-02	3,74292045	4,29E-01	5,13E-01	7,95E-01	4732471J01Rik	0,96	No
ENSMUSG00000053716	3,13E-01	4,141679846	1,32E+01	2,77E-04	5,69E-03	Dusp7	1,24	Yes
ENSMUSG00000053719	3,60E-01	1,305646157	3,69E+00	5,47E-02	2,86E-01	Klk1b26	1,28	No
ENSMUSG00000053730	1,81E-01	3,028708566	2,66E+00	1,03E-01	3,95E-01	Tmem39b	1,13	No
ENSMUSG00000053740	4,52E-02	4,381873124	3,01E-01	5,83E-01	8,38E-01	Gm6457	1,03	No
ENSMUSG00000053746	1,91E-01	3,056294019	2,88E+00	8,95E-02	3,79E-01	Ptrh1	1,14	No
ENSMUSG00000053749	8,45E-02	1,488538733	2,29E-01	6,33E-01	8,65E-01	Gm9920	1,06	No
ENSMUSG00000053754	-1,24E-02	7,023982637	4,16E-02	8,38E-01	9,82E-01	Chd8	0,99	No
ENSMUSG00000053765	-5,34E-02	-2,600707582	2,60E-03	9,59E-01	1,00E+00	Oas1f	0,96	No
ENSMUSG00000053768	-4,91E-02	5,525469846	5,05E-01	4,77E-01	7,65E-01	Chchd3	0,97	No
ENSMUSG00000053769	6,10E-02	2,671006919	2,45E-01	6,21E-01	8,57E-01	Lysmd1	1,04	No
ENSMUSG00000053774	-1,06E-01	5,568378055	2,41E+00	1,21E-01	4,33E-01	Ubxn7	0,93	No
ENSMUSG00000053799	3,70E-02	4,680493172	2,24E-01	6,36E-01	8,67E-01	Exoc6	1,03	No
ENSMUSG00000053801	1,72E-01	4,019084696	3,71E+00	5,40E-02	2,84E-01	Grwd1	1,13	No
ENSMUSG00000053819	3,21E-01	5,476306436	2,11E+01	4,43E-06	1,63E-04	Camk2d	1,25	Yes
ENSMUSG00000053820	-3,85E-01	3,502100503	1,50E+01	1,10E-04	2,64E-03	Bcl2a1c	0,77	Yes
ENSMUSG00000053821	1,81E+00	-3,10765737	1,28E+00	2,58E-01	5,50E-01	Gm9922	3,49	No
ENSMUSG00000053830	-9,04E-02	3,72312646	9,03E-01	3,42E-01	6,42E-01	Gm9923	0,94	No
ENSMUSG00000053835	-1,45E-02	3,463623453	2,06E-02	8,86E-01	1,00E+00	H2-T24	0,99	No
ENSMUSG00000053838	-7,63E-02	5,975227748	1,35E+00	2,45E-01	5,50E-01	Nudcd3	0,95	No
ENSMUSG00000053841	1,03E-01	6,780257601	2,82E+00	9,30E-02	3,83E-01	Txlna	1,07	No
ENSMUSG00000053846	-7,43E-01	-1,929245477	1,45E+00	2,29E-01	5,50E-01	Lipg	0,60	No

ENSMUSG00000053862	3,86E-01	-2,205885835	1,78E-01	6,74E-01	8,90E-01	Slc51b	1,31	No
ENSMUSG00000053863	2,64E+00	-2,963288486	2,69E+00	1,01E-01	3,92E-01	Mepe	6,24	No
ENSMUSG00000053870	1,45E-02	4,246687528	2,97E-02	8,63E-01	9,97E-01	Fpgt	1,01	No
ENSMUSG00000053877	4,10E-02	6,300167792	4,14E-01	5,20E-01	7,99E-01	Srcap	1,03	No
ENSMUSG00000053886	-1,01E+00	0,650184491	1,94E+01	1,09E-05	3,64E-04	Sh2d4a	0,50	Yes
ENSMUSG00000053889	1,67E+00	-2,311213262	4,37E+00	3,67E-02	2,28E-01	Kirrel3os	3,19	No
ENSMUSG00000053897	-9,50E-02	2,747969918	6,13E-01	4,34E-01	7,27E-01	Slc39a8	0,94	No
ENSMUSG00000053898	-3,76E-02	5,678257361	3,09E-01	5,78E-01	8,35E-01	Ech1	0,97	No
ENSMUSG00000053907	5,01E-02	7,130847498	6,91E-01	4,06E-01	7,02E-01	Mat2a	1,04	No
ENSMUSG00000053914	-4,65E-01	-2,185700439	3,98E-01	5,28E-01	8,04E-01	Kdm4d	0,72	No
ENSMUSG00000053916	1,24E-01	1,384026546	4,71E-01	4,93E-01	7,78E-01	Nanp	1,09	No
ENSMUSG00000053925	4,73E-01	0,87683209	4,81E+00	2,83E-02	1,90E-01	Gm9929	1,39	No
ENSMUSG00000053929	-6,49E-02	6,122065297	1,01E+00	3,14E-01	6,14E-01	Cyhr1	0,96	No
ENSMUSG00000053931	-5,84E-01	0,766062717	7,22E+00	7,22E-03	7,35E-02	Cnn3	0,67	No
ENSMUSG00000053950	2,26E-01	4,12039096	7,14E+00	7,54E-03	7,58E-02	Adnp2	1,17	No
ENSMUSG00000053957	-7,27E-01	-2,1856877	9,00E-01	3,43E-01	6,43E-01	Gm12474	0,60	No
ENSMUSG00000053961	-8,22E-01	-2,831994842	3,71E-01	5,43E-01	8,12E-01	Ang5	0,57	No
ENSMUSG00000053963	-3,57E-01	3,411423279	1,25E+01	4,10E-04	7,76E-03	Stum	0,78	Yes
ENSMUSG00000053964	8,73E-02	1,218048246	1,99E-01	6,56E-01	8,80E-01	Lgals4	1,06	No
ENSMUSG00000053965	-4,53E-01	-0,011175681	2,58E+00	1,08E-01	4,03E-01	Pde5a	0,73	No
ENSMUSG00000053977	4,83E-02	0,721357789	4,88E-02	8,25E-01	9,74E-01	Cd8a	1,03	No
ENSMUSG00000053980	3,14E-02	-3,107617333	-7,69E-10	1,00E+00	1,00E+00	Gm9930	1,02	No
ENSMUSG00000053985	2,13E-01	0,693878225	9,87E-01	3,20E-01	6,21E-01	Zfp14	1,16	No
ENSMUSG00000054000	1,41E-01	4,410224356	2,97E+00	8,47E-02	3,73E-01	Tusc1	1,10	No
ENSMUSG00000054005	-1,89E+00	-3,1076441	1,42E+00	2,33E-01	5,50E-01	Mill1	0,27	No
ENSMUSG00000054006	3,47E-01	1,699378656	4,33E+00	3,75E-02	2,30E-01	D630008O14Rik	1,27	No
ENSMUSG00000054008	1,35E-01	6,266952703	4,47E+00	3,46E-02	2,19E-01	Ndst1	1,10	No
ENSMUSG00000054013	-3,18E+00	-2,832017009	4,25E+00	3,93E-02	2,35E-01	Tmem179	0,11	No
ENSMUSG00000054021	-2,40E-01	2,31702928	3,24E+00	7,19E-02	3,40E-01	Sirt5	0,85	No
ENSMUSG00000054027	1,94E-01	4,758080187	6,37E+00	1,16E-02	1,04E-01	Nt5dc3	1,14	No
ENSMUSG00000054046	-1,87E+00	-3,107637761	1,39E+00	2,38E-01	5,50E-01	Klk13	0,27	No
ENSMUSG00000054051	1,69E-01	5,936425667	6,50E+00	1,08E-02	9,89E-02	Ercc6	1,12	No

ENSMUSG00000054057	6,89E-02	1,083543861	1,19E-01	7,30E-01	9,21E-01	A930004D18Rik	1,05	No
ENSMUSG00000054061	-1,91E-01	-1,051606375	1,85E-01	6,67E-01	8,86E-01	Gm9934	0,88	No
ENSMUSG00000054065	-6,84E-01	3,279171259	4,28E+01	6,06E-11	5,94E-09	Pkp3	0,62	Yes
ENSMUSG00000054069	-5,10E-01	-1,92927015	6,31E-01	4,27E-01	7,21E-01	6030452D12Rik	0,70	No
ENSMUSG00000054072	-3,93E-01	-0,997921705	8,06E-01	3,69E-01	6,71E-01	Iigp1	0,76	No
ENSMUSG00000054074	-1,09E-01	-0,378275065	1,02E-01	7,50E-01	9,31E-01	Skida1	0,93	No
ENSMUSG00000054079	-1,69E-02	5,32459854	5,68E-02	8,12E-01	9,67E-01	Utp18	0,99	No
ENSMUSG00000054083	1,86E-01	2,991735548	2,74E+00	9,80E-02	3,91E-01	Capn12	1,14	No
ENSMUSG00000054091	-8,80E-03	5,181503871	1,47E-02	9,04E-01	1,00E+00	1810037I17Rik	0,99	No
ENSMUSG00000054099	2,69E-01	3,302431512	6,62E+00	1,01E-02	9,38E-02	Slc25a40	1,21	No
ENSMUSG00000054115	5,28E-01	3,247417553	2,56E+01	4,29E-07	2,08E-05	Skp2	1,44	Yes
ENSMUSG00000054116	-2,27E-01	-1,535365654	1,65E-01	6,85E-01	8,97E-01	E130116L18Rik	0,85	No
ENSMUSG00000054128	-3,87E-02	-2,655121351	1,34E-03	9,71E-01	1,00E+00	H2-T3	0,97	No
ENSMUSG00000054134	1,58E+00	-2,711780351	1,71E+00	1,91E-01	5,50E-01	Umodl1	2,99	No
ENSMUSG00000054135	6,20E-01	-1,627645049	1,35E+00	2,45E-01	5,50E-01	A430110L20Rik	1,54	No
ENSMUSG00000054136	3,14E-02	-3,107617333	-7,69E-10	1,00E+00	1,00E+00	Adm2	1,02	No
ENSMUSG00000054150	2,90E-01	4,793268762	1,38E+01	1,99E-04	4,33E-03	Syne3	1,22	Yes
ENSMUSG00000054160	2,57E-01	-0,136571456	7,97E-01	3,72E-01	6,73E-01	Nkx2-4	1,19	No
ENSMUSG00000054161	-7,43E-02	-1,770429767	1,15E-02	9,14E-01	1,00E+00	Fam83e	0,95	No
ENSMUSG00000054169	-1,80E+00	-0,497169084	2,03E+01	6,65E-06	2,33E-04	Ceacam10	0,29	Yes
ENSMUSG00000054178	2,13E-01	2,678534769	2,95E+00	8,61E-02	3,78E-01	Gm9938	1,16	No
ENSMUSG00000054181	2,87E-02	-0,074860406	9,82E-03	9,21E-01	1,00E+00	A930012O16Rik	1,02	No
ENSMUSG00000054191	1,39E-01	-1,697430485	5,82E-02	8,09E-01	9,65E-01	Klf1	1,10	No
ENSMUSG00000054196	6,85E-01	-2,896150766	2,55E-01	6,14E-01	8,53E-01	Cthrc1	1,61	No
ENSMUSG00000054199	5,70E-02	5,951249215	7,55E-01	3,85E-01	6,86E-01	Gon4l	1,04	No
ENSMUSG00000054200	7,73E-02	4,593791716	9,64E-01	3,26E-01	6,27E-01	Ffar4	1,06	No
ENSMUSG00000054203	2,42E-01	3,038753548	4,71E+00	3,00E-02	1,98E-01	Ifi205	1,18	No
ENSMUSG00000054204	-7,53E-02	-2,401408256	6,50E-03	9,36E-01	1,00E+00	Alkal2	0,95	No
ENSMUSG00000054206	-1,54E-01	-0,44883928	2,03E-01	6,52E-01	8,77E-01	Gzmm	0,90	No
ENSMUSG00000054226	1,73E-01	4,303929209	4,32E+00	3,77E-02	2,31E-01	Tprkb	1,13	No
ENSMUSG00000054237	-1,97E-01	2,265133106	2,04E+00	1,53E-01	4,96E-01	Fra10ac1	0,87	No
ENSMUSG00000054247	4,34E-01	1,825303021	7,20E+00	7,28E-03	7,39E-02	Gm9939	1,35	No

ENSMUSG00000054252	-7,02E-01	0,225785425	7,49E+00	6,19E-03	6,57E-02	Fgfr3	0,61	No
ENSMUSG00000054256	9,01E-03	-3,107646452	-5,16E-10	1,00E+00	1,00E+00	Msi1	1,01	No
ENSMUSG00000054258	-3,56E+00	-2,896109226	5,70E+00	1,70E-02	1,36E-01	Gm5082	0,08	No
ENSMUSG00000054263	4,34E-02	4,771625263	3,20E-01	5,72E-01	8,32E-01	Lifr	1,03	No
ENSMUSG00000054277	5,77E-02	5,089464727	6,23E-01	4,30E-01	7,23E-01	Arfgap3	1,04	No
ENSMUSG00000054280	1,85E-01	5,962178315	8,02E+00	4,63E-03	5,27E-02	Prr14l	1,14	No
ENSMUSG00000054302	-2,18E-01	4,592787261	7,61E+00	5,81E-03	6,26E-02	Eapp	0,86	No
ENSMUSG00000054304	4,54E-01	-0,574226933	1,55E+00	2,14E-01	5,50E-01	D130007C19Rik	1,37	No
ENSMUSG00000054309	5,83E-02	5,803166846	7,61E-01	3,83E-01	6,84E-01	Cpsf3	1,04	No
ENSMUSG00000054312	-1,03E-01	4,936751368	1,89E+00	1,70E-01	5,25E-01	Mrps21	0,93	No
ENSMUSG00000054320	7,31E-01	-1,800998207	1,44E+00	2,30E-01	5,50E-01	Lrrc36	1,66	No
ENSMUSG00000054321	1,59E-01	3,606362383	2,63E+00	1,05E-01	3,97E-01	Taf4b	1,12	No
ENSMUSG00000054342	2,71E-01	7,384090227	2,06E+01	5,74E-06	2,05E-04	Kcnn4	1,21	Yes
ENSMUSG00000054360	7,07E-01	-2,963242697	2,70E-01	6,03E-01	8,48E-01	Bsx	1,63	No
ENSMUSG00000054362	-2,70E+00	-3,033614577	2,89E+00	8,93E-02	3,79E-01	Lexm	0,15	No
ENSMUSG00000054364	1,10E-01	6,019658773	2,81E+00	9,35E-02	3,83E-01	Rhob	1,08	No
ENSMUSG00000054381	-7,68E-02	2,598726463	3,77E-01	5,39E-01	8,09E-01	Zfp747	0,95	No
ENSMUSG00000054383	-6,33E-01	-0,210804008	4,10E+00	4,30E-02	2,46E-01	Pnma1	0,64	No
ENSMUSG00000054385	-1,94E-01	3,098838033	3,09E+00	7,88E-02	3,59E-01	Ceacam2	0,87	No
ENSMUSG00000054387	-2,90E-01	7,623818172	2,43E+01	8,38E-07	3,81E-05	Mdm4	0,82	Yes
ENSMUSG00000054400	6,52E-02	4,281835315	6,18E-01	4,32E-01	7,25E-01	Cklf	1,05	No
ENSMUSG00000054404	-7,48E-02	6,223552706	1,37E+00	2,41E-01	5,50E-01	Slfn5	0,95	No
ENSMUSG00000054405	-8,71E-02	5,753026677	1,69E+00	1,94E-01	5,50E-01	Dnajc8	0,94	No
ENSMUSG00000054408	1,54E-01	7,515818526	6,80E+00	9,10E-03	8,70E-02	Spcs3	1,11	No
ENSMUSG00000054409	6,25E-01	-2,497574475	4,36E-01	5,09E-01	7,92E-01	Tmem74	1,54	No
ENSMUSG00000054412	2,10E-01	1,797585509	1,63E+00	2,02E-01	5,50E-01	Gm4793	1,16	No
ENSMUSG00000054414	1,33E-01	6,593902982	4,55E+00	3,28E-02	2,12E-01	Slc30a7	1,10	No
ENSMUSG00000054418	-3,59E-01	-0,740658482	8,98E-01	3,43E-01	6,44E-01	2900041M22Rik	0,78	No
ENSMUSG00000054422	6,97E-01	-0,574341647	3,65E+00	5,60E-02	2,90E-01	Fabp1	1,62	No
ENSMUSG00000054423	-3,77E-01	-0,770580061	9,56E-01	3,28E-01	6,29E-01	Cadps	0,77	No
ENSMUSG00000054426	2,37E-01	0,128059375	7,17E-01	3,97E-01	6,97E-01	A930005H10Rik	1,18	No
ENSMUSG00000054428	-2,38E-01	6,4291056	1,43E+01	1,58E-04	3,55E-03	Atpif1	0,85	Yes

ENSMUSG00000054434	-2,14E-01	4,675838207	7,68E+00	5,57E-03	6,05E-02	Tmem120b	0,86	No
ENSMUSG00000054435	3,85E-01	-2,070171745	2,36E-01	6,27E-01	8,60E-01	Gimap4	1,31	No
ENSMUSG00000054446	-1,87E+00	-3,107637761	1,39E+00	2,38E-01	5,50E-01	Cpa1	0,27	No
ENSMUSG00000054450	1,20E+00	-3,146123991	6,74E-01	4,12E-01	7,07E-01	Gm9945	2,30	No
ENSMUSG00000054452	-8,03E-02	7,822341916	1,88E+00	1,70E-01	5,26E-01	Aes	0,95	No
ENSMUSG00000054455	-2,82E-02	6,745725372	2,10E-01	6,47E-01	8,74E-01	Vapb	0,98	No
ENSMUSG00000054459	6,16E-01	-2,548223342	4,23E-01	5,15E-01	7,97E-01	Vsnl1	1,53	No
ENSMUSG00000054469	9,83E-02	5,241256038	1,88E+00	1,70E-01	5,26E-01	Lclat1	1,07	No
ENSMUSG00000054474	-6,61E-01	0,748192861	8,88E+00	2,88E-03	3,71E-02	Thnsl2	0,63	Yes
ENSMUSG00000054477	-4,70E-01	-2,998027202	1,10E-01	7,41E-01	9,27E-01	Kcnn2	0,72	No
ENSMUSG00000054484	3,30E-03	3,901279219	1,32E-03	9,71E-01	1,00E+00	Tmem62	1,00	No
ENSMUSG00000054488	-1,87E+00	-3,107637761	1,39E+00	2,38E-01	5,50E-01	Gm9946	0,27	No
ENSMUSG00000054493	1,85E+00	-3,107657832	1,35E+00	2,46E-01	5,50E-01	Gm9947	3,61	No
ENSMUSG00000054499	-2,49E-01	4,743219631	1,04E+01	1,25E-03	1,94E-02	Dedd2	0,84	Yes
ENSMUSG00000054509	-1,26E-01	6,472921818	4,01E+00	4,51E-02	2,53E-01	Parp4	0,92	No
ENSMUSG00000054510	-5,81E-01	0,721614673	6,79E+00	9,19E-03	8,77E-02	Gm14461	0,67	No
ENSMUSG00000054514	-8,55E-01	-2,070195968	1,31E+00	2,52E-01	5,50E-01	Atad3aos	0,55	No
ENSMUSG00000054517	2,36E-01	3,257570457	5,12E+00	2,37E-02	1,69E-01	Trim65	1,18	No
ENSMUSG00000054519	-3,86E-02	1,755800298	6,00E-02	8,06E-01	9,64E-01	Zfp867	0,97	No
ENSMUSG00000054520	3,10E-01	6,187599417	2,33E+01	1,39E-06	5,95E-05	Sh3bp2	1,24	Yes
ENSMUSG00000054545	-2,71E-01	3,266932779	6,63E+00	1,00E-02	9,35E-02	Ugt1a6a	0,83	No
ENSMUSG00000054555	5,42E-01	1,652589388	9,74E+00	1,80E-03	2,58E-02	Adam12	1,46	Yes
ENSMUSG00000054556	-2,21E-01	3,521936129	5,09E+00	2,41E-02	1,70E-01	Gm4876	0,86	No
ENSMUSG00000054580	-1,74E+00	-2,033525454	5,34E+00	2,09E-02	1,55E-01	Pla2r1	0,30	No
ENSMUSG00000054582	1,61E-01	0,258888277	2,93E-01	5,88E-01	8,40E-01	Pabpc1l	1,12	No
ENSMUSG00000054589	2,58E-01	0,952587874	1,36E+00	2,44E-01	5,50E-01	Gm9949	1,20	No
ENSMUSG00000054594	-1,25E+00	-1,800847817	3,70E+00	5,45E-02	2,86E-01	Oscar	0,42	No
ENSMUSG00000054598	1,09E-01	1,114226482	2,90E-01	5,90E-01	8,41E-01	9130230L23Rik	1,08	No
ENSMUSG00000054604	-2,32E-03	7,151443671	1,49E-03	9,69E-01	1,00E+00	Cggbp1	1,00	No
ENSMUSG00000054611	5,47E-02	6,984336457	8,14E-01	3,67E-01	6,68E-01	Kdm2a	1,04	No
ENSMUSG00000054612	2,59E-03	2,664695283	4,27E-04	9,84E-01	1,00E+00	Mgmt	1,00	No
ENSMUSG00000054618	8,02E-01	-2,711748167	5,59E-01	4,55E-01	7,44E-01	Gm9951	1,74	No

ENSMUSG00000054619	2,53E-01	5,023013165	1,17E+01	6,36E-04	1,12E-02	Mettl7a1	1,19	Yes
ENSMUSG00000054622	2,26E-01	-2,456919762	5,15E-02	8,21E-01	9,72E-01	D730045B01Rik	1,17	No
ENSMUSG00000054640	-1,18E-01	5,312096297	2,78E+00	9,54E-02	3,86E-01	Slc8a1	0,92	No
ENSMUSG00000054641	-4,50E-02	-2,711766047	1,79E-03	9,66E-01	1,00E+00	Mmrn1	0,97	No
ENSMUSG00000054648	-1,11E-01	4,161595962	1,66E+00	1,98E-01	5,50E-01	Zfp869	0,93	No
ENSMUSG00000054659	-3,77E-01	-2,448548803	1,79E-01	6,72E-01	8,89E-01	Pm20d2	0,77	No
ENSMUSG00000054662	-1,92E+00	-3,033623267	1,46E+00	2,27E-01	5,50E-01	Ano9	0,26	No
ENSMUSG00000054666	-1,87E+00	-3,107637761	1,39E+00	2,38E-01	5,50E-01	Olfr63	0,27	No
ENSMUSG00000054672	9,01E-03	-3,107646452	-5,16E-10	1,00E+00	1,00E+00	5830411N06Rik	1,01	No
ENSMUSG00000054675	-1,51E-02	1,340917136	6,35E-03	9,36E-01	1,00E+00	Tmem119	0,99	No
ENSMUSG00000054676	-8,05E-02	5,784427032	1,47E+00	2,26E-01	5,50E-01	1600014C10Rik	0,95	No
ENSMUSG00000054679	-7,42E-01	-2,497543813	6,17E-01	4,32E-01	7,26E-01	Srsf12	0,60	No
ENSMUSG00000054690	8,60E-01	-2,600645158	6,44E-01	4,22E-01	7,18E-01	Emcn	1,82	No
ENSMUSG00000054693	-1,09E-02	7,493715151	3,41E-02	8,54E-01	9,92E-01	Adam10	0,99	No
ENSMUSG00000054702	1,24E-01	4,397074542	2,33E+00	1,27E-01	4,47E-01	Ap1s3	1,09	No
ENSMUSG00000054708	-7,19E-02	3,621751783	5,50E-01	4,58E-01	7,47E-01	Ankrd24	0,95	No
ENSMUSG00000054715	6,29E-01	2,687031934	2,76E+01	1,51E-07	8,07E-06	Zscan22	1,55	Yes
ENSMUSG00000054716	6,78E-02	3,642850332	5,10E-01	4,75E-01	7,63E-01	Zfp771	1,05	No
ENSMUSG00000054717	1,15E-01	5,611472709	2,83E+00	9,25E-02	3,83E-01	Hmgb2	1,08	No
ENSMUSG00000054720	-3,07E-02	6,952674604	2,54E-01	6,15E-01	8,53E-01	Lrrc8c	0,98	No
ENSMUSG00000054723	-9,13E-02	2,864563671	6,15E-01	4,33E-01	7,26E-01	Vmac	0,94	No
ENSMUSG00000054728	2,10E-01	0,412472747	6,46E-01	4,22E-01	7,17E-01	Phactr1	1,16	No
ENSMUSG00000054733	1,37E-01	3,521535383	1,85E+00	1,73E-01	5,30E-01	Msra	1,10	No
ENSMUSG00000054745	-1,89E+00	-3,1076441	1,42E+00	2,33E-01	5,50E-01	Gm9955	0,27	No
ENSMUSG00000054752	-3,90E-01	3,842550177	1,84E+01	1,81E-05	5,66E-04	Fsd1l	0,76	Yes
ENSMUSG00000054753	1,81E+00	-3,10765737	1,28E+00	2,58E-01	5,50E-01	AU018091	3,49	No
ENSMUSG00000054763	1,87E+00	-3,033632735	1,36E+00	2,43E-01	5,50E-01	Defb42	3,66	No
ENSMUSG00000054766	4,35E-02	6,84531967	5,02E-01	4,79E-01	7,66E-01	Set	1,03	No
ENSMUSG00000054770	-2,18E-03	5,105525016	9,03E-04	9,76E-01	1,00E+00	Kctd18	1,00	No
ENSMUSG00000054779	-2,68E+00	-3,033645766	2,84E+00	9,17E-02	3,82E-01	Fgf2os	0,16	No
ENSMUSG00000054792	1,10E-01	5,167313245	2,34E+00	1,26E-01	4,45E-01	Klhl18	1,08	No
ENSMUSG00000054793	-2,48E-02	-0,267923486	4,66E-03	9,46E-01	1,00E+00	Cadm4	0,98	No

ENSMUSG00000054808	4,52E-02	7,963759072	6,01E-01	4,38E-01	7,31E-01	Actn4	1,03	No
ENSMUSG00000054814	2,10E-01	4,490289685	6,81E+00	9,07E-03	8,69E-02	Usp46	1,16	No
ENSMUSG00000054822	-9,51E-01	-1,847767517	1,97E+00	1,61E-01	5,09E-01	1700041G16Rik	0,52	No
ENSMUSG00000054823	7,38E-03	7,049209296	1,49E-02	9,03E-01	1,00E+00	Nsd3	1,01	No
ENSMUSG00000054836	1,02E-01	2,584477695	6,66E-01	4,14E-01	7,10E-01	Elp6	1,07	No
ENSMUSG00000054843	1,40E-01	6,470573539	4,94E+00	2,63E-02	1,81E-01	Atrnl1	1,10	No
ENSMUSG00000054855	-1,17E-01	0,776300863	2,89E-01	5,91E-01	8,41E-01	Rnd1	0,92	No
ENSMUSG00000054863	-1,94E+00	-3,107619243	1,50E+00	2,21E-01	5,50E-01	Fam19a5	0,26	No
ENSMUSG00000054871	-2,59E-01	0,726584466	1,30E+00	2,54E-01	5,50E-01	Tmem158	0,84	No
ENSMUSG00000054874	5,07E-02	6,804363104	6,83E-01	4,09E-01	7,05E-01	Pcnx3	1,04	No
ENSMUSG00000054889	-6,96E-01	-2,54820941	5,41E-01	4,62E-01	7,51E-01	Dsp	0,62	No
ENSMUSG00000054890	1,87E+00	-3,107639691	1,38E+00	2,40E-01	5,50E-01	Olfr1535	3,67	No
ENSMUSG00000054892	-5,67E-01	-1,711791452	8,89E-01	3,46E-01	6,47E-01	Txk	0,67	No
ENSMUSG00000054893	2,21E-01	0,683178243	9,27E-01	3,36E-01	6,37E-01	Zfp667	1,17	No
ENSMUSG00000054894	-1,74E-02	1,936931079	1,35E-02	9,07E-01	1,00E+00	Atp5s	0,99	No
ENSMUSG00000054901	3,78E-01	-2,088805481	2,87E-01	5,92E-01	8,42E-01	Arhgef33	1,30	No
ENSMUSG00000054905	-6,27E-01	-1,831976508	9,93E-01	3,19E-01	6,19E-01	Stfa3	0,65	No
ENSMUSG00000054910	1,23E+00	-2,424897366	1,50E+00	2,21E-01	5,50E-01	4931415C17Rik	2,35	No
ENSMUSG00000054920	-1,98E-01	6,712137965	1,03E+01	1,30E-03	2,01E-02	Klhl5	0,87	Yes
ENSMUSG00000054931	-3,64E-01	-0,267980734	1,30E+00	2,54E-01	5,50E-01	Zkscan4	0,78	No
ENSMUSG00000054932	-1,44E-02	0,508539065	3,45E-03	9,53E-01	1,00E+00	Afp	0,99	No
ENSMUSG00000054934	-1,89E+00	-2,740792706	2,60E+00	1,07E-01	4,00E-01	Kcnmb4	0,27	No
ENSMUSG00000054939	1,51E-01	0,103737718	3,07E-01	5,79E-01	8,36E-01	Zfp174	1,11	No
ENSMUSG00000054942	-1,38E-01	3,658620323	2,11E+00	1,46E-01	4,85E-01	Miga1	0,91	No
ENSMUSG00000054944	1,81E+00	-3,10765737	1,28E+00	2,58E-01	5,50E-01	5330416C01Rik	3,49	No
ENSMUSG00000054945	1,52E-01	-1,548261553	7,01E-02	7,91E-01	9,55E-01	Gm9958	1,11	No
ENSMUSG00000054951	-7,04E-01	1,792541317	2,04E+01	6,19E-06	2,18E-04	9130008F23Rik	0,61	Yes
ENSMUSG00000054957	-8,36E-01	-2,522659561	6,98E-01	4,03E-01	7,01E-01	Gm9959	0,56	No
ENSMUSG00000054958	-8,45E-01	-2,963265612	3,92E-01	5,31E-01	8,05E-01	Nt5c1a	0,56	No
ENSMUSG00000054966	-2,70E+00	-2,96323619	2,89E+00	8,89E-02	3,79E-01	Lmntd1	0,15	No
ENSMUSG00000054967	1,02E+00	-0,60078341	7,02E+00	8,06E-03	7,95E-02	Zfp647	2,03	No
ENSMUSG00000054976	-7,21E-02	-2,832016801	3,55E-03	9,52E-01	1,00E+00	Nyap2	0,95	No

ENSMUSG00000054978	1,43E-01	-1,47295379	6,63E-02	7,97E-01	9,58E-01	Kbtbd13	1,10	No
ENSMUSG00000054999	2,83E-01	1,112212415	2,01E+00	1,56E-01	5,00E-01	Naaladl1	1,22	No
ENSMUSG00000055003	-2,37E-01	-2,033603789	1,11E-01	7,39E-01	9,26E-01	Lrtm2	0,85	No
ENSMUSG00000055010	-1,94E+00	-3,107619243	1,50E+00	2,21E-01	5,50E-01	A830031A19Rik	0,26	No
ENSMUSG00000055013	-9,73E-02	4,435543189	1,43E+00	2,32E-01	5,50E-01	Agap1	0,93	No
ENSMUSG00000055015	-2,04E+00	-2,600589396	3,14E+00	7,64E-02	3,52E-01	Gm9961	0,24	No
ENSMUSG00000055024	1,32E-01	7,396599192	4,92E+00	2,65E-02	1,82E-01	Ep300	1,10	No
ENSMUSG00000055027	-6,12E-02	1,195521903	1,03E-01	7,48E-01	9,30E-01	Smyd1	0,96	No
ENSMUSG00000055041	7,98E-02	4,007314892	8,01E-01	3,71E-01	6,72E-01	Commd5	1,06	No
ENSMUSG00000055044	1,82E-01	3,356642791	2,95E+00	8,59E-02	3,77E-01	Pdlim1	1,13	No
ENSMUSG00000055053	1,34E-01	5,441172051	3,70E+00	5,43E-02	2,85E-01	Nfic	1,10	No
ENSMUSG00000055061	-3,75E-03	3,741506731	1,60E-03	9,68E-01	1,00E+00	4931431C16Rik	1,00	No
ENSMUSG00000055065	-1,60E-01	8,709824038	7,87E+00	5,04E-03	5,62E-02	Ddx17	0,89	No
ENSMUSG00000055067	-2,12E-02	3,477100792	4,41E-02	8,34E-01	9,79E-01	Smyd3	0,99	No
ENSMUSG00000055069	5,20E-01	0,910911459	6,61E+00	1,02E-02	9,44E-02	Rab39	1,43	No
ENSMUSG00000055093	-1,55E-01	7,74853236	7,02E+00	8,06E-03	7,95E-02	Gm8430	0,90	No
ENSMUSG00000055102	-1,26E+00	0,184144513	1,92E+01	1,18E-05	3,90E-04	Zfp819	0,42	Yes
ENSMUSG00000055108	1,81E+00	-3,10765737	1,28E+00	2,58E-01	5,50E-01	Phxr2	3,49	No
ENSMUSG00000055114	7,05E-01	-2,355641725	7,22E-01	3,96E-01	6,96E-01	Anxa13	1,63	No
ENSMUSG00000055116	1,41E-01	3,68455154	2,16E+00	1,42E-01	4,76E-01	Arntl	1,10	No
ENSMUSG00000055128	2,53E-02	4,153137604	8,48E-02	7,71E-01	9,42E-01	Cgrrf1	1,02	No
ENSMUSG00000055134	-2,35E+00	-2,997971374	2,16E+00	1,42E-01	4,76E-01	9130017K11Rik	0,20	No
ENSMUSG00000055137	2,24E-02	0,499367043	8,61E-03	9,26E-01	1,00E+00	Sugct	1,02	No
ENSMUSG00000055148	-6,83E-02	3,893281608	5,71E-01	4,50E-01	7,40E-01	Klf2	0,95	No
ENSMUSG00000055150	3,07E-01	0,128028966	1,30E+00	2,53E-01	5,50E-01	Zfp78	1,24	No
ENSMUSG00000055159	-1,89E+00	-3,1076441	1,42E+00	2,33E-01	5,50E-01	4930583K01Rik	0,27	No
ENSMUSG00000055172	1,58E-01	3,402744743	2,32E+00	1,28E-01	4,48E-01	C1ra	1,12	No
ENSMUSG00000055177	-1,06E-01	-3,033634182	5,86E-03	9,39E-01	1,00E+00	Cstl1	0,93	No
ENSMUSG00000055184	3,18E-01	1,715412086	3,86E+00	4,94E-02	2,68E-01	Fam72a	1,25	No
ENSMUSG00000055193	4,06E-01	3,335637705	1,46E+01	1,32E-04	3,06E-03	Klk15	1,33	Yes
ENSMUSG00000055200	-3,14E-02	3,363258801	9,32E-02	7,60E-01	9,37E-01	Sertad3	0,98	No
ENSMUSG00000055202	-1,04E+00	-2,033623725	2,03E+00	1,54E-01	4,97E-01	Zfp811	0,49	No

ENSMUSG00000055204	-1,53E-03	7,332092635	6,58E-04	9,80E-01	1,00E+00	Ankrd17	1,00	No
ENSMUSG00000055210	-2,91E-03	-0,126913723	8,24E-05	9,93E-01	1,00E+00	Foxd2	1,00	No
ENSMUSG00000055216	1,26E-01	-0,607656869	1,15E-01	7,35E-01	9,24E-01	9430025C20Rik	1,09	No
ENSMUSG00000055228	-1,37E-02	2,731639126	1,35E-02	9,08E-01	1,00E+00	Zfp935	0,99	No
ENSMUSG00000055235	-1,42E-01	3,635862257	2,28E+00	1,31E-01	4,56E-01	Wdr86	0,91	No
ENSMUSG00000055239	-1,80E-01	6,292729262	8,03E+00	4,60E-03	5,25E-02	Kcmf1	0,88	No
ENSMUSG00000055240	1,89E-01	3,937754213	4,48E+00	3,43E-02	2,18E-01	Zfp101	1,14	No
ENSMUSG00000055254	-3,24E+00	-2,896104886	4,43E+00	3,53E-02	2,22E-01	Ntrk2	0,11	No
ENSMUSG00000055271	2,29E-01	-1,16585687	1,77E-01	6,74E-01	8,90E-01	9330161L09Rik	1,17	No
ENSMUSG00000055296	7,70E-02	7,105168566	1,63E+00	2,02E-01	5,50E-01	Tmem245	1,05	No
ENSMUSG00000055301	-1,94E+00	-3,107619243	1,50E+00	2,21E-01	5,50E-01	Adh7	0,26	No
ENSMUSG00000055302	-1,08E-01	7,646302575	3,35E+00	6,71E-02	3,26E-01	Mrfap1	0,93	No
ENSMUSG00000055305	-1,76E-01	0,554002935	5,85E-01	4,44E-01	7,36E-01	Zfp93	0,89	No
ENSMUSG00000055313	-2,48E-01	-0,126632949	6,19E-01	4,31E-01	7,25E-01	Pgbd1	0,84	No
ENSMUSG00000055319	1,03E-01	5,779377252	2,36E+00	1,25E-01	4,42E-01	Sec23ip	1,07	No
ENSMUSG00000055320	-2,10E-01	1,244224325	1,22E+00	2,70E-01	5,66E-01	Tead1	0,86	No
ENSMUSG00000055322	3,45E-01	8,145270688	3,52E+01	2,96E-09	2,26E-07	Tns1	1,27	Yes
ENSMUSG00000055323	9,99E-02	-0,424837994	8,96E-02	7,65E-01	9,39E-01	Gm9967	1,07	No
ENSMUSG00000055333	1,91E+00	-2,548260003	2,72E+00	9,94E-02	3,92E-01	Fat2	3,75	No
ENSMUSG00000055334	-1,13E-01	3,197192693	1,14E+00	2,86E-01	5,82E-01	Snupn	0,92	No
ENSMUSG00000055341	4,12E-01	-1,412912196	5,58E-01	4,55E-01	7,45E-01	Zfp457	1,33	No
ENSMUSG00000055360	3,67E-01	-2,268140299	2,15E-01	6,43E-01	8,72E-01	Prl2c5	1,29	No
ENSMUSG00000055368	9,01E-03	-3,107646452	-5,16E-10	1,00E+00	1,00E+00	Slc6a2	1,01	No
ENSMUSG00000055370	3,16E+00	-2,831998699	4,06E+00	4,39E-02	2,50E-01	Gm9968	8,91	No
ENSMUSG00000055371	-9,20E-05	5,242272491	1,67E-06	9,99E-01	1,00E+00	Stam2	1,00	No
ENSMUSG00000055401	2,06E-02	5,004766846	7,76E-02	7,81E-01	9,49E-01	Fbxo6	1,01	No
ENSMUSG00000055403	-1,87E+00	-3,107637761	1,39E+00	2,38E-01	5,50E-01	4933427D06Rik	0,27	No
ENSMUSG00000055407	1,25E-01	3,724697905	1,76E+00	1,85E-01	5,49E-01	Map6	1,09	No
ENSMUSG00000055413	-5,35E-01	4,217988352	4,04E+01	2,04E-10	1,84E-08	H2-Q5	0,69	Yes
ENSMUSG00000055415	-2,66E+00	-3,033639428	2,81E+00	9,35E-02	3,83E-01	Atp10b	0,16	No
ENSMUSG00000055416	-2,27E+00	-2,448656627	4,19E+00	4,06E-02	2,39E-01	Fam136b-ps	0,21	No
ENSMUSG00000055424	-1,82E-01	0,221882968	4,65E-01	4,95E-01	7,81E-01	Gm9970	0,88	No

ENSMUSG00000055430	-4,31E-01	-2,185720649	3,87E-01	5,34E-01	8,07E-01	Nap1l5	0,74	No
ENSMUSG00000055435	-9,84E-02	3,27295178	8,52E-01	3,56E-01	6,57E-01	Maf	0,93	No
ENSMUSG00000055436	-4,38E-02	7,54836068	5,48E-01	4,59E-01	7,48E-01	Srsf11	0,97	No
ENSMUSG00000055446	3,14E+00	-2,896171661	4,02E+00	4,50E-02	2,53E-01	A630091E08Rik	8,80	No
ENSMUSG00000055447	-3,46E-01	7,31690759	3,36E+01	6,90E-09	4,93E-07	Cd47	0,79	Yes
ENSMUSG00000055452	-1,40E-02	4,1238935	2,61E-02	8,72E-01	1,00E+00	Gm7353	0,99	No
ENSMUSG00000055471	2,60E+00	-2,77060903	2,63E+00	1,05E-01	3,97E-01	Alk	6,07	No
ENSMUSG00000055480	2,93E-01	1,62579708	3,22E+00	7,28E-02	3,42E-01	Zfp458	1,23	No
ENSMUSG00000055485	1,93E-01	6,907176364	9,94E+00	1,62E-03	2,36E-02	Soga1	1,14	Yes
ENSMUSG00000055491	8,29E-02	6,043598417	1,61E+00	2,05E-01	5,50E-01	Pprc1	1,06	No
ENSMUSG00000055493	-3,70E-02	2,175622441	6,85E-02	7,94E-01	9,56E-01	Epm2a	0,97	No
ENSMUSG00000055494	-6,18E-02	-1,015704741	1,43E-02	9,05E-01	1,00E+00	Gm14168	0,96	No
ENSMUSG00000055497	-2,80E-01	-1,522722147	2,75E-01	6,00E-01	8,46E-01	Gm9974	0,82	No
ENSMUSG00000055523	-3,77E-01	-1,548179395	4,79E-01	4,89E-01	7,76E-01	Gucy2g	0,77	No
ENSMUSG00000055531	3,12E-02	6,377543843	2,42E-01	6,22E-01	8,58E-01	Cpsf6	1,02	No
ENSMUSG00000055538	1,65E-01	3,928226656	3,22E+00	7,27E-02	3,42E-01	Zcchc24	1,12	No
ENSMUSG00000055540	9,01E-03	-3,107646452	-5,16E-10	1,00E+00	1,00E+00	Epha6	1,01	No
ENSMUSG00000055541	2,50E-01	5,311275554	1,22E+01	4,67E-04	8,68E-03	Lair1	1,19	Yes
ENSMUSG00000055546	7,24E-01	-2,896104002	2,86E-01	5,93E-01	8,42E-01	Timd4	1,65	No
ENSMUSG00000055553	-1,18E-01	6,356482822	3,48E+00	6,21E-02	3,10E-01	Kxd1	0,92	No
ENSMUSG00000055560	1,23E-01	-1,300245488	5,68E-02	8,12E-01	9,67E-01	Zfp459	1,09	No
ENSMUSG00000055561	1,85E+00	-3,107657832	1,35E+00	2,46E-01	5,50E-01	Spink5	3,61	No
ENSMUSG00000055567	1,84E+00	-3,033649193	1,33E+00	2,49E-01	5,50E-01	Unc80	3,58	No
ENSMUSG00000055602	-6,90E-01	-1,574234599	1,36E+00	2,43E-01	5,50E-01	Tcp10b	0,62	No
ENSMUSG00000055609	1,87E+00	-3,107639691	1,38E+00	2,40E-01	5,50E-01	Hba-x	3,67	No
ENSMUSG00000055610	-1,89E+00	-3,1076441	1,42E+00	2,33E-01	5,50E-01	Olfr307	0,27	No
ENSMUSG00000055612	2,11E-02	2,524379633	2,77E-02	8,68E-01	1,00E+00	Cdca7	1,01	No
ENSMUSG00000055629	-8,14E-01	-2,311180925	9,66E-01	3,26E-01	6,26E-01	B4galnt4	0,57	No
ENSMUSG00000055632	4,68E-01	3,407108168	2,09E+01	4,93E-06	1,78E-04	Hmcn2	1,38	Yes
ENSMUSG00000055633	-3,21E-01	0,09563633	1,27E+00	2,61E-01	5,54E-01	Zfp580	0,80	No
ENSMUSG00000055639	-3,19E-01	2,126378148	4,69E+00	3,03E-02	2,00E-01	Dach1	0,80	No
ENSMUSG00000055652	3,65E-01	3,76489096	1,58E+01	7,12E-05	1,82E-03	Klhl25	1,29	Yes

ENSMUSG00000055660	1,11E-01	2,736554647	8,61E-01	3,53E-01	6,55E-01	Mettl4	1,08	No
ENSMUSG00000055670	2,39E-03	7,461815117	1,62E-03	9,68E-01	1,00E+00	Zzef1	1,00	No
ENSMUSG00000055675	3,60E-01	2,181630594	6,58E+00	1,03E-02	9,56E-02	Kbtbd11	1,28	No
ENSMUSG00000055676	-2,59E-01	-0,037994491	7,73E-01	3,79E-01	6,80E-01	Gm5069	0,84	No
ENSMUSG00000055681	2,34E-02	6,197941958	1,32E-01	7,16E-01	9,13E-01	Cope	1,02	No
ENSMUSG00000055692	5,01E-02	1,798140672	9,65E-02	7,56E-01	9,35E-01	Tmem191c	1,04	No
ENSMUSG00000055704	-1,89E+00	-3,1076441	1,42E+00	2,33E-01	5,50E-01	Gm9978	0,27	No
ENSMUSG00000055707	-2,80E-02	4,322509691	1,14E-01	7,36E-01	9,24E-01	Klhl26	0,98	No
ENSMUSG00000055717	-6,56E-01	0,768607845	8,78E+00	3,05E-03	3,87E-02	Slain1	0,63	Yes
ENSMUSG00000055720	-2,58E-01	5,282932557	1,33E+01	2,63E-04	5,46E-03	Ubl7	0,84	Yes
ENSMUSG00000055723	-1,86E-02	5,842082842	7,90E-02	7,79E-01	9,47E-01	Rras2	0,99	No
ENSMUSG00000055725	-2,56E-01	5,098913556	1,24E+01	4,21E-04	7,96E-03	Paqr3	0,84	Yes
ENSMUSG00000055730	1,87E+00	-3,107639691	1,38E+00	2,40E-01	5,50E-01	Ces2a	3,67	No
ENSMUSG00000055737	-3,17E-01	-1,35552305	2,81E-01	5,96E-01	8,44E-01	Ghr	0,80	No
ENSMUSG00000055745	1,90E-01	1,872835796	1,55E+00	2,14E-01	5,50E-01	Ldoc1l	1,14	No
ENSMUSG00000055748	-7,88E-02	-2,185701049	1,16E-02	9,14E-01	1,00E+00	Gsdmc4	0,95	No
ENSMUSG00000055760	-1,17E-01	2,166659434	6,60E-01	4,16E-01	7,12E-01	Gemin6	0,92	No
ENSMUSG00000055761	1,85E+00	-3,107657832	1,35E+00	2,46E-01	5,50E-01	Nkain3	3,61	No
ENSMUSG00000055762	4,24E-02	5,555429622	3,82E-01	5,36E-01	8,08E-01	Eef1d	1,03	No
ENSMUSG00000055763	-1,13E-01	2,316808661	6,77E-01	4,11E-01	7,07E-01	Rybp-ps	0,92	No
ENSMUSG00000055771	-1,29E-01	3,868640384	1,98E+00	1,60E-01	5,07E-01	Gm7936	0,91	No
ENSMUSG00000055782	7,23E-02	4,98994052	9,61E-01	3,27E-01	6,28E-01	Abcd2	1,05	No
ENSMUSG00000055795	-4,41E-02	3,485752104	1,97E-01	6,57E-01	8,80E-01	Gm5160	0,97	No
ENSMUSG00000055799	-1,26E-01	-0,150741744	1,81E-01	6,71E-01	8,88E-01	Tcf7l1	0,92	No
ENSMUSG00000055805	-9,83E-02	7,928467612	2,84E+00	9,17E-02	3,82E-01	Fmnl1	0,93	No
ENSMUSG00000055809	-3,10E-01	4,492783228	1,53E+01	9,24E-05	2,26E-03	Dnaaf3	0,81	Yes
ENSMUSG00000055811	-1,89E+00	-3,1076441	1,42E+00	2,33E-01	5,50E-01	Cldn17	0,27	No
ENSMUSG00000055817	-8,50E-02	4,836702791	1,24E+00	2,65E-01	5,61E-01	Mta3	0,94	No
ENSMUSG00000055818	-5,15E-02	0,259500421	4,02E-02	8,41E-01	9,84E-01	A230083G16Rik	0,96	No
ENSMUSG00000055820	-1,94E+00	-3,107619243	1,50E+00	2,21E-01	5,50E-01	Olfr967	0,26	No
ENSMUSG00000055826	-9,44E-01	-2,600722178	7,82E-01	3,77E-01	6,78E-01	Tescl	0,52	No
ENSMUSG00000055833	3,81E-01	1,471992276	4,50E+00	3,40E-02	2,17E-01	1700034H15Rik	1,30	No

ENSMUSG00000055835	-1,34E-01	3,115622655	1,51E+00	2,19E-01	5,50E-01	Zfp1	0,91	No
ENSMUSG00000055839	-1,08E-01	5,049082037	2,18E+00	1,40E-01	4,72E-01	Elob	0,93	No
ENSMUSG00000055850	-7,05E-02	6,549264998	1,28E+00	2,58E-01	5,50E-01	Rnf181	0,95	No
ENSMUSG00000055862	-5,85E-01	3,761162793	4,15E+01	1,16E-10	1,09E-08	Izumo4	0,67	Yes
ENSMUSG00000055865	1,63E-02	1,352101464	8,22E-03	9,28E-01	1,00E+00	Fam19a3	1,01	No
ENSMUSG00000055866	7,37E-02	3,195110966	4,61E-01	4,97E-01	7,82E-01	Per2	1,05	No
ENSMUSG00000055872	1,91E+00	-2,448678443	2,72E+00	9,91E-02	3,92E-01	Gm9985	3,76	No
ENSMUSG00000055874	1,87E+00	-3,107639691	1,38E+00	2,40E-01	5,50E-01	Foxi3	3,67	No
ENSMUSG00000055882	4,14E-02	-1,522686592	5,02E-03	9,44E-01	1,00E+00	Abhd16b	1,03	No
ENSMUSG00000055884	2,42E-01	4,018262138	7,31E+00	6,85E-03	7,06E-02	Fancm	1,18	No
ENSMUSG00000055891	-3,83E-02	-3,107651936	-7,91E-11	1,00E+00	1,00E+00	Ubl4b	0,97	No
ENSMUSG00000055895	-2,66E+00	-3,033639428	2,81E+00	9,35E-02	3,83E-01	Oosp2	0,16	No
ENSMUSG00000055897	-7,30E-02	4,926181736	9,63E-01	3,26E-01	6,27E-01	Ppp4r1l-ps	0,95	No
ENSMUSG00000055900	1,61E-01	3,017953557	2,11E+00	1,46E-01	4,85E-01	Tmem69	1,12	No
ENSMUSG00000055912	-1,53E-01	2,483868863	1,38E+00	2,40E-01	5,50E-01	Tmem150a	0,90	No
ENSMUSG00000055917	3,91E-02	4,165960182	2,09E-01	6,48E-01	8,75E-01	Zfp277	1,03	No
ENSMUSG00000055923	1,83E-01	3,193704446	3,01E+00	8,29E-02	3,70E-01	Aasdh	1,14	No
ENSMUSG00000055926	-4,21E-03	0,672583856	3,35E-04	9,85E-01	1,00E+00	Gm14137	1,00	No
ENSMUSG00000055932	4,84E-02	5,663295545	5,11E-01	4,75E-01	7,63E-01	Fto	1,03	No
ENSMUSG00000055937	-1,94E+00	-3,107619243	1,50E+00	2,21E-01	5,50E-01	Krt28	0,26	No
ENSMUSG00000055943	1,47E-03	5,905910131	4,98E-04	9,82E-01	1,00E+00	Emc7	1,00	No
ENSMUSG00000055945	-4,53E-01	-2,226248983	2,09E-01	6,48E-01	8,75E-01	Prr18	0,73	No
ENSMUSG00000055963	8,44E-01	-0,461102551	4,96E+00	2,59E-02	1,80E-01	Triqk	1,79	No
ENSMUSG00000055968	2,27E-01	0,522879862	8,44E-01	3,58E-01	6,60E-01	Gm9988	1,17	No
ENSMUSG00000055972	-2,25E-02	-2,963247595	1,86E-11	1,00E+00	1,00E+00	2810407A14Rik	0,98	No
ENSMUSG00000055976	6,58E-01	-2,107645747	8,80E-01	3,48E-01	6,49E-01	Cldn23	1,58	No
ENSMUSG00000055978	-2,94E-01	-2,226261287	1,50E-01	6,98E-01	9,04E-01	Fut2	0,82	No
ENSMUSG00000055980	1,35E-01	-2,107590366	3,15E-02	8,59E-01	9,95E-01	Irs1	1,10	No
ENSMUSG00000055991	-1,45E-01	3,143922941	1,83E+00	1,76E-01	5,34E-01	Zkscan5	0,90	No
ENSMUSG00000055994	-2,39E-01	4,217382227	8,08E+00	4,49E-03	5,14E-02	Nod2	0,85	No
ENSMUSG00000056004	-6,89E-02	1,607493098	1,62E-01	6,87E-01	8,98E-01	9330182L06Rik	0,95	No
ENSMUSG00000056008	-2,63E-02	-3,033641358	2,33E-04	9,88E-01	1,00E+00	Gm11541	0,98	No

ENSMUSG00000056014	5,90E-04	1,820713769	1,37E-05	9,97E-01	1,00E+00	A430033K04Rik	1,00	No
ENSMUSG00000056018	-7,53E-02	-2,600750449	3,76E-03	9,51E-01	1,00E+00	Ccdc7b	0,95	No
ENSMUSG00000056019	-5,16E-03	3,740982071	3,10E-03	9,56E-01	1,00E+00	Zfp709	1,00	No
ENSMUSG00000056025	-3,20E+00	-2,963227503	4,31E+00	3,79E-02	2,31E-01	Clca3a1	0,11	No
ENSMUSG00000056031	-1,23E-01	-1,600684126	4,87E-02	8,25E-01	9,74E-01	9330154J02Rik	0,92	No
ENSMUSG00000056043	1,14E-01	-2,185659854	2,58E-02	8,72E-01	1,00E+00	Rgs9bp	1,08	No
ENSMUSG00000056050	7,34E-02	5,993789968	1,25E+00	2,64E-01	5,58E-01	Mia3	1,05	No
ENSMUSG00000056054	-3,38E-01	2,938684163	8,73E+00	3,12E-03	3,95E-02	S100a8	0,79	Yes
ENSMUSG00000056055	1,65E-01	-0,535233566	1,92E-01	6,62E-01	8,83E-01	Sag	1,12	No
ENSMUSG00000056069	1,59E-01	6,53457725	6,48E+00	1,09E-02	9,96E-02	Fam105a	1,12	No
ENSMUSG00000056071	-6,98E-01	2,366518189	2,71E+01	1,90E-07	9,96E-06	S100a9	0,62	Yes
ENSMUSG00000056073	-2,69E+00	-2,831970912	2,88E+00	8,99E-02	3,80E-01	Grik2	0,15	No
ENSMUSG00000056076	1,20E-01	7,46334245	4,09E+00	4,30E-02	2,47E-01	Eif3b	1,09	No
ENSMUSG00000056078	1,24E+00	-1,963290138	3,24E+00	7,17E-02	3,40E-01	Lipm	2,36	No
ENSMUSG00000056089	-3,22E+00	-2,770637488	4,36E+00	3,68E-02	2,28E-01	Gm5468	0,11	No
ENSMUSG00000056091	6,38E-02	5,799973914	9,09E-01	3,40E-01	6,41E-01	St3gal5	1,05	No
ENSMUSG00000056116	-2,04E-01	6,202556512	1,02E+01	1,43E-03	2,15E-02	H2-T22	0,87	Yes
ENSMUSG00000056121	1,82E-01	4,902833609	5,85E+00	1,55E-02	1,29E-01	Fez2	1,13	No
ENSMUSG00000056124	2,12E-01	6,171296579	1,08E+01	1,00E-03	1,62E-02	B4galt6	1,16	Yes
ENSMUSG00000056130	2,66E-01	4,983307915	1,30E+01	3,11E-04	6,24E-03	Ticam2	1,20	Yes
ENSMUSG00000056131	-3,77E-02	3,57933507	1,50E-01	6,99E-01	9,04E-01	Pgm3	0,97	No
ENSMUSG00000056133	5,99E-01	-1,998045256	6,68E-01	4,14E-01	7,09E-01	Gm9992	1,51	No
ENSMUSG00000056144	-2,43E-01	3,570884803	6,55E+00	1,05E-02	9,69E-02	Trim34a	0,85	No
ENSMUSG00000056145	4,18E-01	7,622557572	4,93E+01	2,14E-12	2,48E-10	AI504432	1,34	Yes
ENSMUSG00000056148	1,23E-01	-2,62763472	1,47E-02	9,04E-01	1,00E+00	Rdh9	1,09	No
ENSMUSG00000056153	4,33E-01	7,363488912	5,22E+01	5,10E-13	6,45E-11	Socs6	1,35	Yes
ENSMUSG00000056155	7,73E-01	-2,896122969	3,27E-01	5,68E-01	8,29E-01	Nanos3	1,71	No
ENSMUSG00000056162	1,85E+00	-3,107657832	1,35E+00	2,46E-01	5,50E-01	Cndp1	3,61	No
ENSMUSG00000056167	3,11E-02	5,121417581	1,82E-01	6,70E-01	8,88E-01	Cnot10	1,02	No
ENSMUSG00000056174	3,17E+00	-2,963263597	4,11E+00	4,28E-02	2,46E-01	Col8a2	8,99	No
ENSMUSG00000056184	-1,94E+00	-3,107619243	1,50E+00	2,21E-01	5,50E-01	Olfr283	0,26	No
ENSMUSG00000056185	-1,51E-01	3,211443177	2,09E+00	1,48E-01	4,87E-01	Snx32	0,90	No

ENSMUSG00000056201	-1,31E-02	9,900782855	5,45E-02	8,15E-01	9,69E-01	Cfl1	0,99	No
ENSMUSG00000056203	-7,21E-02	-2,401441207	5,96E-03	9,38E-01	1,00E+00	Tas2r135	0,95	No
ENSMUSG00000056204	3,79E-02	4,695742422	2,45E-01	6,21E-01	8,57E-01	Pgpep1	1,03	No
ENSMUSG00000056209	-5,91E-02	2,051368291	1,59E-01	6,90E-01	8,99E-01	Npm3	0,96	No
ENSMUSG00000056211	1,46E-01	6,303442284	5,23E+00	2,23E-02	1,62E-01	R3hdm1	1,11	No
ENSMUSG00000056214	-8,93E-01	0,087496201	9,72E+00	1,83E-03	2,60E-02	Pard6g	0,54	Yes
ENSMUSG00000056215	-1,87E+00	-3,033619006	1,41E+00	2,36E-01	5,50E-01	Lrguk	0,27	No
ENSMUSG00000056216	1,26E-01	5,69336948	3,51E+00	6,09E-02	3,06E-01	Cebpg	1,09	No
ENSMUSG00000056219	-1,53E-01	0,175903026	2,88E-01	5,91E-01	8,42E-01	Tmem229b-ps	0,90	No
ENSMUSG00000056220	2,00E-01	4,918673007	7,02E+00	8,07E-03	7,95E-02	Pla2g4a	1,15	No
ENSMUSG00000056222	2,64E+00	-3,033673223	2,69E+00	1,01E-01	3,92E-01	Spock1	6,23	No
ENSMUSG00000056228	6,11E-02	4,148525162	5,22E-01	4,70E-01	7,58E-01	Cars2	1,04	No
ENSMUSG00000056234	-2,05E-01	6,544155125	1,08E+01	9,97E-04	1,61E-02	Ncoa4	0,87	Yes
ENSMUSG00000056257	-3,25E-01	-1,655087362	2,95E-01	5,87E-01	8,40E-01	Gm5447	0,80	No
ENSMUSG00000056258	6,63E-01	0,644282409	7,51E+00	6,13E-03	6,51E-02	Kcnq3	1,58	No
ENSMUSG00000056260	1,74E-02	4,777466689	5,19E-02	8,20E-01	9,71E-01	Lrif1	1,01	No
ENSMUSG00000056267	-1,65E-02	4,030900985	3,51E-02	8,51E-01	9,90E-01	Cep70	0,99	No
ENSMUSG00000056268	9,54E-02	6,919126943	2,44E+00	1,18E-01	4,27E-01	Dennd1b	1,07	No
ENSMUSG00000056271	2,89E-01	-1,497628586	2,42E-01	6,23E-01	8,58E-01	Lman1l	1,22	No
ENSMUSG00000056288	-1,89E+00	-3,1076441	1,42E+00	2,33E-01	5,50E-01	Gm11961	0,27	No
ENSMUSG00000056290	5,45E-01	-0,676348197	2,07E+00	1,50E-01	4,91E-01	Ms4a4b	1,46	No
ENSMUSG00000056293	1,81E+00	-3,10765737	1,28E+00	2,58E-01	5,50E-01	Gsdmc2	3,49	No
ENSMUSG00000056300	2,79E-01	-1,366846749	3,07E-01	5,79E-01	8,36E-01	Zfp981	1,21	No
ENSMUSG00000056305	5,58E-02	5,136274632	5,89E-01	4,43E-01	7,34E-01	Usp39	1,04	No
ENSMUSG00000056310	1,19E-02	4,381148222	2,08E-02	8,85E-01	1,00E+00	Tyw1	1,01	No
ENSMUSG00000056313	1,87E+00	-3,107639691	1,38E+00	2,40E-01	5,50E-01	1810011O10Rik	3,67	No
ENSMUSG00000056342	-4,91E-02	7,01857962	6,56E-01	4,18E-01	7,13E-01	Usp34	0,97	No
ENSMUSG00000056366	3,23E-01	0,742117998	1,99E+00	1,59E-01	5,05E-01	Fabp3-ps1	1,25	No
ENSMUSG00000056367	-9,59E-01	-0,070020505	1,06E+01	1,12E-03	1,77E-02	Actr3b	0,51	Yes
ENSMUSG00000056383	9,49E-02	4,687545968	1,54E+00	2,15E-01	5,50E-01	AI987944	1,07	No
ENSMUSG00000056394	1,83E-01	5,164438809	6,37E+00	1,16E-02	1,04E-01	Lig1	1,14	No
ENSMUSG00000056399	-1,04E+00	-0,997888869	5,99E+00	1,44E-02	1,22E-01	Prss34	0,49	No

ENSMUSG00000056411	7,68E-03	2,128117742	2,90E-03	9,57E-01	1,00E+00	Gm12500	1,01	No
ENSMUSG00000056412	-2,83E-02	5,400479069	1,63E-01	6,86E-01	8,97E-01	Gm12396	0,98	No
ENSMUSG00000056413	-1,98E-01	6,262013884	9,63E+00	1,91E-03	2,70E-02	Adap1	0,87	Yes
ENSMUSG00000056418	5,29E-01	-0,697426505	1,76E+00	1,85E-01	5,49E-01	BC043934	1,44	No
ENSMUSG00000056427	1,22E+00	-2,770623536	9,24E-01	3,36E-01	6,37E-01	Slit3	2,33	No
ENSMUSG00000056429	-4,85E-02	7,942575584	6,92E-01	4,05E-01	7,02E-01	Tgoln1	0,97	No
ENSMUSG00000056436	1,85E+00	-3,107657832	1,35E+00	2,46E-01	5,50E-01	Cyct	3,61	No
ENSMUSG00000056445	-1,08E+00	-2,247009345	1,45E+00	2,28E-01	5,50E-01	Hoxaas2	0,47	No
ENSMUSG00000056458	-1,65E-01	-0,220981123	2,66E-01	6,06E-01	8,50E-01	Mok	0,89	No
ENSMUSG00000056459	1,35E-01	2,819250683	1,30E+00	2,55E-01	5,50E-01	Zbtb25	1,10	No
ENSMUSG00000056468	2,62E+00	-3,033654625	2,66E+00	1,03E-01	3,95E-01	5730596B20Rik	6,16	No
ENSMUSG00000056476	-1,27E-01	2,330147258	8,81E-01	3,48E-01	6,49E-01	Med12l	0,92	No
ENSMUSG00000056481	9,01E-03	-3,107646452	-5,16E-10	1,00E+00	1,00E+00	Cd248	1,01	No
ENSMUSG00000056486	-6,99E-01	-2,89609921	1,96E-01	6,58E-01	8,81E-01	Chn1	0,62	No
ENSMUSG00000056487	-1,65E-02	-0,808643921	1,60E-03	9,68E-01	1,00E+00	Mettl7a2	0,99	No
ENSMUSG00000056492	-1,77E-01	-1,770626587	9,92E-02	7,53E-01	9,33E-01	Adgrf5	0,88	No
ENSMUSG00000056493	-5,43E-02	6,132532698	7,13E-01	3,99E-01	6,98E-01	Foxk1	0,96	No
ENSMUSG00000056494	3,11E+00	-2,770631362	3,94E+00	4,72E-02	2,60E-01	Cngb3	8,61	No
ENSMUSG00000056498	9,21E-02	8,58582704	2,58E+00	1,08E-01	4,03E-01	Tmem154	1,07	No
ENSMUSG00000056501	2,76E-01	7,432525355	2,15E+01	3,47E-06	1,33E-04	Cebpb	1,21	Yes
ENSMUSG00000056508	4,50E-02	0,337747431	2,88E-02	8,65E-01	9,98E-01	1700001K19Rik	1,03	No
ENSMUSG00000056515	2,13E-01	6,564947521	1,16E+01	6,67E-04	1,16E-02	Rab31	1,16	Yes
ENSMUSG00000056529	-5,92E-02	6,927785629	9,44E-01	3,31E-01	6,32E-01	Ptafr	0,96	No
ENSMUSG00000056531	3,95E-01	1,889574189	6,32E+00	1,19E-02	1,06E-01	Ccdc18	1,32	No
ENSMUSG00000056536	2,81E-02	5,828121605	1,78E-01	6,73E-01	8,90E-01	Pign	1,02	No
ENSMUSG00000056569	-5,98E-02	-1,711559838	1,08E-02	9,17E-01	1,00E+00	Mpz	0,96	No
ENSMUSG00000056579	-9,13E-02	6,569101229	2,14E+00	1,43E-01	4,78E-01	Tug1	0,94	No
ENSMUSG00000056592	6,85E-01	3,275972018	4,19E+01	9,58E-11	9,08E-09	Zfp658	1,61	Yes
ENSMUSG00000056596	8,64E-01	0,175658626	9,19E+00	2,43E-03	3,26E-02	Trnp1	1,82	Yes
ENSMUSG00000056598	-4,78E-01	-1,424895453	7,95E-01	3,73E-01	6,74E-01	Drc3	0,72	No
ENSMUSG00000056600	1,81E+00	-3,10765737	1,28E+00	2,58E-01	5,50E-01	Olfr90	3,49	No
ENSMUSG00000056602	1,44E-01	4,960884166	3,68E+00	5,50E-02	2,87E-01	Fry	1,11	No

ENSMUSG00000056605	-1,87E+00	-3,107637761	1,39E+00	2,38E-01	5,50E-01	Krt72	0,27	No
ENSMUSG00000056608	1,58E-02	6,572599111	6,37E-02	8,01E-01	9,61E-01	Chd9	1,01	No
ENSMUSG00000056612	1,77E-01	5,403356814	6,40E+00	1,14E-02	1,03E-01	Ppp1r14b	1,13	No
ENSMUSG00000056617	-1,90E+00	-3,033594154	1,44E+00	2,30E-01	5,50E-01	4931429L15Rik	0,27	No
ENSMUSG00000056629	5,89E-02	4,61857142	5,61E-01	4,54E-01	7,44E-01	Fkbp2	1,04	No
ENSMUSG00000056643	-5,36E-01	-1,033717495	1,26E+00	2,62E-01	5,55E-01	Chst13	0,69	No
ENSMUSG00000056648	-5,25E-02	-3,033661434	4,08E-11	1,00E+00	1,00E+00	Hoxb8	0,96	No
ENSMUSG00000056656	2,72E-01	2,743124237	4,68E+00	3,06E-02	2,02E-01	Apol8	1,21	No
ENSMUSG00000056665	-1,06E-01	1,955246088	4,74E-01	4,91E-01	7,77E-01	Them6	0,93	No
ENSMUSG00000056666	2,86E-02	4,151434106	1,08E-01	7,42E-01	9,27E-01	Retsat	1,02	No
ENSMUSG00000056671	-4,43E-02	2,604468596	1,27E-01	7,22E-01	9,16E-01	Prelid2	0,97	No
ENSMUSG00000056682	-1,39E+00	-2,311230225	1,91E+00	1,67E-01	5,20E-01	4930465K10Rik	0,38	No
ENSMUSG00000056687	3,57E-02	3,242759082	1,12E-01	7,38E-01	9,25E-01	Gm11696	1,03	No
ENSMUSG00000056692	-1,46E-01	7,898065608	6,25E+00	1,24E-02	1,09E-01	D17Wsu92e	0,90	No
ENSMUSG00000056698	-6,31E-02	2,85634298	2,94E-01	5,88E-01	8,40E-01	Elmod3	0,96	No
ENSMUSG00000056699	-1,87E+00	-3,107637761	1,39E+00	2,38E-01	5,50E-01	Gm5533	0,27	No
ENSMUSG00000056708	2,93E-02	7,480644208	2,45E-01	6,21E-01	8,57E-01	Ier5	1,02	No
ENSMUSG00000056716	-1,86E+00	-2,448676679	4,05E+00	4,41E-02	2,50E-01	Gm5420	0,27	No
ENSMUSG00000056718	4,57E-01	0,615841604	3,71E+00	5,42E-02	2,85E-01	Gm13199	1,37	No
ENSMUSG00000056724	2,25E-01	7,226982142	1,40E+01	1,79E-04	3,95E-03	Nbeal2	1,17	Yes
ENSMUSG00000056735	-4,95E-01	0,499507679	4,63E+00	3,13E-02	2,05E-01	A930024E05Rik	0,71	No
ENSMUSG00000056737	1,47E-01	9,501531301	6,75E+00	9,37E-03	8,90E-02	Capg	1,11	No
ENSMUSG00000056738	-2,16E+00	-2,711711741	3,68E+00	5,51E-02	2,88E-01	A730036I17Rik	0,22	No
ENSMUSG00000056749	-3,14E-01	6,006243181	2,32E+01	1,45E-06	6,14E-05	Nfil3	0,80	Yes
ENSMUSG00000056752	-2,95E-01	-1,522576982	2,85E-01	5,94E-01	8,43E-01	Dnah9	0,81	No
ENSMUSG00000056753	1,37E-01	0,221893765	2,81E-01	5,96E-01	8,45E-01	C330011M18Rik	1,10	No
ENSMUSG00000056758	-2,89E-01	-2,627597878	7,10E-02	7,90E-01	9,54E-01	Hmga2	0,82	No
ENSMUSG00000056763	-1,01E-01	4,534738411	1,58E+00	2,08E-01	5,50E-01	Cspp1	0,93	No
ENSMUSG00000056770	1,31E-02	6,725889512	4,54E-02	8,31E-01	9,78E-01	Setd3	1,01	No
ENSMUSG00000056771	-5,88E-02	-3,033652696	1,62E-03	9,68E-01	1,00E+00	Gm10010	0,96	No
ENSMUSG00000056772	1,03E-01	-0,726240632	6,54E-02	7,98E-01	9,59E-01	Rps6-ps2	1,07	No
ENSMUSG00000056777	-6,07E-01	-2,448636781	5,03E-01	4,78E-01	7,66E-01	Rpl21-ps13	0,66	No

ENSMUSG00000056820	3,22E-02	5,726254822	2,29E-01	6,32E-01	8,64E-01	Tsnax	1,02	No
ENSMUSG00000056821	-1,72E+00	-2,448652536	3,26E+00	7,12E-02	3,38E-01	1700028I16Rik	0,30	No
ENSMUSG00000056824	-2,71E+00	-3,033620915	2,92E+00	8,75E-02	3,78E-01	Zfp663	0,15	No
ENSMUSG00000056829	3,51E+00	-2,83205728	5,36E+00	2,06E-02	1,54E-01	Foxb2	11,42	No
ENSMUSG00000056832	1,13E+00	-1,801042781	2,60E+00	1,07E-01	4,00E-01	Ttc26	2,19	No
ENSMUSG00000056836	-8,99E-02	3,928088442	1,01E+00	3,15E-01	6,15E-01	Gm6851	0,94	No
ENSMUSG00000056849	-1,21E+00	-2,448597519	1,89E+00	1,69E-01	5,24E-01	Gm16415	0,43	No
ENSMUSG00000056851	-5,50E-02	7,370161889	8,52E-01	3,56E-01	6,57E-01	Pcbp2	0,96	No
ENSMUSG00000056856	1,88E+00	-2,896170024	1,82E+00	1,77E-01	5,37E-01	Jakmip3	3,67	No
ENSMUSG00000056863	-8,41E-01	-2,89611984	3,88E-01	5,33E-01	8,06E-01	Olfr702	0,56	No
ENSMUSG00000056870	-1,83E+00	-2,226255286	5,29E+00	2,15E-02	1,58E-01	Gulp1	0,28	No
ENSMUSG00000056880	-3,11E-01	6,360566367	2,40E+01	9,48E-07	4,24E-05	Gadl1	0,81	Yes
ENSMUSG00000056888	-2,86E-01	7,327502746	2,28E+01	1,76E-06	7,27E-05	Glipr1	0,82	Yes
ENSMUSG00000056895	2,54E-01	-0,963340957	3,52E-01	5,53E-01	8,19E-01	Hist3h2ba	1,19	No
ENSMUSG00000056899	-2,29E-01	-0,711491781	3,49E-01	5,55E-01	8,20E-01	Immp2l	0,85	No
ENSMUSG00000056900	-5,47E-03	-2,963221188	1,50E-05	9,97E-01	1,00E+00	Usp13	1,00	No
ENSMUSG00000056904	4,08E-02	6,398339459	4,15E-01	5,20E-01	7,99E-01	Gm5620	1,03	No
ENSMUSG00000056917	1,13E-01	6,703448455	3,32E+00	6,84E-02	3,29E-01	Sipa1	1,08	No
ENSMUSG00000056919	5,50E-02	4,44729641	4,66E-01	4,95E-01	7,81E-01	Cep162	1,04	No
ENSMUSG00000056938	-3,45E-02	2,598725541	7,36E-02	7,86E-01	9,52E-01	Acbd4	0,98	No
ENSMUSG00000056941	-3,27E-03	5,492298828	2,25E-03	9,62E-01	1,00E+00	Commd7	1,00	No
ENSMUSG00000056943	-1,14E-01	1,80898077	5,23E-01	4,70E-01	7,58E-01	Gm10015	0,92	No
ENSMUSG00000056952	-7,43E-03	6,890649298	1,47E-02	9,03E-01	1,00E+00	Tatdn2	0,99	No
ENSMUSG00000056962	5,18E-02	5,033769236	4,96E-01	4,81E-01	7,69E-01	Jmjd6	1,04	No
ENSMUSG00000056966	-2,66E+00	-3,033639428	2,81E+00	9,35E-02	3,83E-01	Gjc3	0,16	No
ENSMUSG00000056978	-4,14E-01	-1,885259429	4,63E-01	4,96E-01	7,82E-01	Hamp2	0,75	No
ENSMUSG00000056987	-2,71E+00	-3,033620915	2,92E+00	8,75E-02	3,78E-01	Fam71d	0,15	No
ENSMUSG00000056999	1,02E-01	5,779204483	2,31E+00	1,28E-01	4,49E-01	Ide	1,07	No
ENSMUSG00000057036	-1,04E-01	8,761183799	3,34E+00	6,77E-02	3,28E-01	Gm7536	0,93	No
ENSMUSG00000057047	-1,94E+00	-3,107619243	1,50E+00	2,21E-01	5,50E-01	1700010B08Rik	0,26	No
ENSMUSG00000057050	-1,87E+00	-3,107637761	1,39E+00	2,38E-01	5,50E-01	Olfr397	0,27	No
ENSMUSG00000057054	-6,52E-01	0,075075474	5,14E+00	2,34E-02	1,68E-01	Inca1	0,64	No

ENSMUSG00000057058	-8,49E-01	-2,896162345	3,95E-01	5,30E-01	8,05E-01	Skap1	0,56	No
ENSMUSG00000057060	-3,88E-03	-3,03362684	4,02E-12	1,00E+00	1,00E+00	Slc35f3	1,00	No
ENSMUSG00000057068	1,87E+00	-3,107639691	1,38E+00	2,40E-01	5,50E-01	Fam47e	3,67	No
ENSMUSG00000057069	-1,77E-02	5,992648179	7,35E-02	7,86E-01	9,52E-01	Ero1lb	0,99	No
ENSMUSG00000057072	6,69E-01	-1,68319466	1,46E+00	2,26E-01	5,50E-01	Spata45	1,59	No
ENSMUSG00000057092	-4,05E-01	1,368924869	5,27E+00	2,17E-02	1,59E-01	Fxyd3	0,76	No
ENSMUSG00000057093	2,79E-01	-0,065710314	9,04E-01	3,42E-01	6,42E-01	Zfp607b	1,21	No
ENSMUSG00000057098	-4,15E-01	-2,655106428	1,75E-01	6,76E-01	8,91E-01	Ebf1	0,75	No
ENSMUSG00000057101	1,73E-02	4,90353205	5,32E-02	8,18E-01	9,70E-01	Zfp180	1,01	No
ENSMUSG00000057103	-3,76E-01	-0,0748604	1,62E+00	2,04E-01	5,50E-01	Nat8f1	0,77	No
ENSMUSG00000057110	-4,76E-02	6,647790045	5,92E-01	4,42E-01	7,34E-01	Cntrl	0,97	No
ENSMUSG00000057113	-7,01E-02	6,273314582	1,21E+00	2,71E-01	5,67E-01	Npm1	0,95	No
ENSMUSG00000057123	2,97E-01	3,525852954	8,92E+00	2,82E-03	3,66E-02	Gja5	1,23	Yes
ENSMUSG00000057130	-6,98E-02	4,389687588	7,20E-01	3,96E-01	6,96E-01	Txnl4a	0,95	No
ENSMUSG00000057132	1,34E-01	4,32491257	2,59E+00	1,07E-01	4,01E-01	Rpgrip1	1,10	No
ENSMUSG00000057133	-1,82E-02	5,320756309	6,63E-02	7,97E-01	9,58E-01	Chd6	0,99	No
ENSMUSG00000057134	-5,07E-02	6,528371343	6,52E-01	4,19E-01	7,15E-01	Ado	0,97	No
ENSMUSG00000057135	2,62E-01	6,594278704	1,76E+01	2,78E-05	8,23E-04	Scimp	1,20	Yes
ENSMUSG00000057137	-4,98E-02	3,969753694	3,21E-01	5,71E-01	8,32E-01	Tmem140	0,97	No
ENSMUSG00000057143	2,89E-02	5,194453297	1,64E-01	6,86E-01	8,97E-01	Trim12c	1,02	No
ENSMUSG00000057147	2,25E-01	3,439241994	4,93E+00	2,64E-02	1,82E-01	Dph6	1,17	No
ENSMUSG00000057156	-1,28E-01	3,203693425	1,50E+00	2,21E-01	5,50E-01	Homez	0,91	No
ENSMUSG00000057157	-9,11E-03	2,932353616	6,20E-03	9,37E-01	1,00E+00	Gm6054	0,99	No
ENSMUSG00000057160	2,71E-02	4,135681633	9,52E-02	7,58E-01	9,35E-01	Gm16372	1,02	No
ENSMUSG00000057163	-9,11E-01	-2,548213869	7,27E-01	3,94E-01	6,94E-01	Prss2	0,53	No
ENSMUSG00000057173	1,86E+00	-3,033661846	1,35E+00	2,46E-01	5,50E-01	Rfx8	3,62	No
ENSMUSG00000057176	4,63E-02	3,638095722	2,31E-01	6,30E-01	8,63E-01	Ccdc189	1,03	No
ENSMUSG00000057177	1,34E-02	5,670793482	3,92E-02	8,43E-01	9,85E-01	Gsk3a	1,01	No
ENSMUSG00000057181	9,88E-02	4,07208763	1,28E+00	2,58E-01	5,50E-01	5730455P16Rik	1,07	No
ENSMUSG00000057182	8,57E-01	4,88600804	1,20E+02	5,47E-28	2,65E-25	Scn3a	1,81	Yes
ENSMUSG00000057191	1,31E-02	5,124649142	3,25E-02	8,57E-01	9,94E-01	AB124611	1,01	No
ENSMUSG00000057193	-8,13E-03	5,931332876	1,52E-02	9,02E-01	1,00E+00	Slc44a2	0,99	No

ENSMUSG00000057219	8,66E-02	4,036422632	9,61E-01	3,27E-01	6,28E-01	Armc7	1,06	No
ENSMUSG00000057223	-1,83E-01	0,152391223	4,10E-01	5,22E-01	8,00E-01	Gm6578	0,88	No
ENSMUSG00000057229	1,17E-01	4,630572418	2,18E+00	1,40E-01	4,73E-01	Atp5sl	1,08	No
ENSMUSG00000057230	5,51E-02	6,50686755	7,71E-01	3,80E-01	6,81E-01	Aak1	1,04	No
ENSMUSG00000057234	7,70E-02	1,602721374	1,96E-01	6,58E-01	8,81E-01	Mettl15	1,05	No
ENSMUSG00000057236	-4,13E-02	6,946134007	4,60E-01	4,97E-01	7,83E-01	Rbbp4	0,97	No
ENSMUSG00000057244	-1,58E-02	5,81857918	5,58E-02	8,13E-01	9,67E-01	Gm6139	0,99	No
ENSMUSG00000057246	4,90E-01	-1,627637713	7,43E-01	3,89E-01	6,89E-01	BC051142	1,40	No
ENSMUSG00000057262	-2,61E-01	6,097412629	1,62E+01	5,73E-05	1,53E-03	Gm10020	0,83	Yes
ENSMUSG00000057265	-2,70E-03	0,266697682	1,04E-04	9,92E-01	1,00E+00	Bbof1	1,00	No
ENSMUSG00000057278	1,19E-02	2,72747741	9,93E-03	9,21E-01	1,00E+00	Snrpg	1,01	No
ENSMUSG00000057280	-5,24E-02	-2,963220811	1,25E-03	9,72E-01	1,00E+00	Musk	0,96	No
ENSMUSG00000057286	-9,39E-02	1,054052342	1,97E-01	6,58E-01	8,81E-01	St6galnac2	0,94	No
ENSMUSG00000057315	2,13E-01	3,874229762	5,34E+00	2,09E-02	1,56E-01	Arhgap24	1,16	No
ENSMUSG00000057321	-1,55E+00	-2,185543571	3,49E+00	6,17E-02	3,09E-01	Usp17ld	0,34	No
ENSMUSG00000057322	-1,82E-01	1,844436795	1,33E+00	2,49E-01	5,50E-01	Rpl38	0,88	No
ENSMUSG00000057329	2,21E-01	5,547657541	1,03E+01	1,32E-03	2,03E-02	Bcl2	1,17	Yes
ENSMUSG00000057335	1,64E-01	6,254666766	6,63E+00	1,00E-02	9,36E-02	Cep170	1,12	No
ENSMUSG00000057337	-6,38E-01	1,341586111	1,29E+01	3,27E-04	6,50E-03	Chst3	0,64	Yes
ENSMUSG00000057342	8,64E-02	6,285369449	1,84E+00	1,75E-01	5,33E-01	Sphk2	1,06	No
ENSMUSG00000057346	-5,77E-01	-2,548238494	2,60E-01	6,10E-01	8,51E-01	Apol9a	0,67	No
ENSMUSG00000057359	-2,81E-02	6,520587432	2,02E-01	6,53E-01	8,78E-01	Gm17494	0,98	No
ENSMUSG00000057363	-8,53E-02	4,647801025	1,20E+00	2,73E-01	5,69E-01	Uxs1	0,94	No
ENSMUSG00000057367	-5,15E-01	6,229866604	6,53E+01	6,36E-16	1,06E-13	Birc2	0,70	Yes
ENSMUSG00000057370	-8,33E-01	-2,963259277	3,79E-01	5,38E-01	8,09E-01	Gm8724	0,56	No
ENSMUSG00000057375	-8,14E-02	4,937022432	1,19E+00	2,76E-01	5,72E-01	Yipf1	0,95	No
ENSMUSG00000057378	-2,21E-01	-0,91267096	2,76E-01	5,99E-01	8,46E-01	Ryr3	0,86	No
ENSMUSG00000057388	1,32E-03	4,999775625	3,16E-04	9,86E-01	1,00E+00	Mrpl18	1,00	No
ENSMUSG00000057396	5,00E-01	0,891978407	6,04E+00	1,40E-02	1,20E-01	Zfp759	1,41	No
ENSMUSG00000057406	1,58E-01	7,048154012	6,80E+00	9,09E-03	8,70E-02	Nsd2	1,12	No
ENSMUSG00000057409	2,94E-01	3,068431941	7,17E+00	7,43E-03	7,49E-02	Zfp53	1,23	No
ENSMUSG00000057411	-1,70E-02	4,759760373	4,90E-02	8,25E-01	9,74E-01	Fam173a	0,99	No

ENSMUSG00000057440	1,26E-01	3,148439318	1,37E+00	2,41E-01	5,50E-01	Mpp7		1,09	No
ENSMUSG00000057465	1,79E+00	-3,033666867	1,28E+00	2,58E-01	5,50E-01	Saa2		3,46	No
ENSMUSG00000057469	2,11E-01	4,680856045	7,26E+00	7,05E-03	7,22E-02	E2f6		1,16	No
ENSMUSG00000057497	5,01E-02	4,198443551	3,46E-01	5,56E-01	8,21E-01	Fam136a		1,04	No
ENSMUSG00000057499	-9,59E-02	-2,832028611	6,37E-03	9,36E-01	1,00E+00	Gm6177		0,94	No
ENSMUSG00000057503	-2,02E+00	-2,655093162	3,08E+00	7,93E-02	3,60E-01	Olfr1459		0,25	No
ENSMUSG00000057506	-1,48E-01	4,384866376	3,29E+00	6,97E-02	3,33E-01	Bloc1s2		0,90	No
ENSMUSG00000057522	-4,16E-03	7,256179841	4,85E-03	9,44E-01	1,00E+00	Spop		1,00	No
ENSMUSG00000057530	1,04E-01	6,40327762	2,70E+00	1,00E-01	3,92E-01	Ece1		1,07	No
ENSMUSG00000057531	1,20E-01	6,042044986	3,39E+00	6,57E-02	3,21E-01	Dtnbp1		1,09	No
ENSMUSG00000057534	-1,90E+00	-3,033594154	1,44E+00	2,30E-01	5,50E-01	Elobl		0,27	No
ENSMUSG00000057541	6,96E-02	3,641332923	5,16E-01	4,72E-01	7,60E-01	Pus7		1,05	No
ENSMUSG00000057551	1,51E-02	4,308923324	3,34E-02	8,55E-01	9,92E-01	Zfp317		1,01	No
ENSMUSG00000057554	1,92E-01	6,353178231	9,10E+00	2,55E-03	3,38E-02	Lgals8		1,14	Yes
ENSMUSG00000057561	2,76E-02	6,657347826	1,98E-01	6,56E-01	8,80E-01	Eif1a		1,02	No
ENSMUSG00000057564	-2,63E-02	-3,033641358	2,33E-04	9,88E-01	1,00E+00	Olfr1508		0,98	No
ENSMUSG00000057572	1,11E-01	3,201143241	1,06E+00	3,04E-01	6,01E-01	Zbtb8os		1,08	No
ENSMUSG00000057580	-5,35E-02	2,404156418	1,65E-01	6,85E-01	8,97E-01	Gm10012		0,96	No
ENSMUSG00000057594	3,72E-02	2,947622252	1,07E-01	7,44E-01	9,28E-01	Arl16		1,03	No
ENSMUSG00000057596	-9,17E-02	5,326834798	1,71E+00	1,90E-01	5,50E-01	Trim30d		0,94	No
ENSMUSG00000057604	-8,79E-01	-2,89610048	4,25E-01	5,15E-01	7,96E-01	Lmcd1		0,54	No
ENSMUSG00000057605	-1,42E-01	5,068452666	3,78E+00	5,17E-02	2,77E-01	Gm6807		0,91	No
ENSMUSG00000057606	-1,02E+00	-1,816395684	2,15E+00	1,42E-01	4,77E-01	Colq		0,49	No
ENSMUSG00000057614	-2,66E+00	-3,033639428	2,81E+00	9,35E-02	3,83E-01	Gnai1		0,16	No
ENSMUSG00000057637	-4,13E-02	6,533197626	4,38E-01	5,08E-01	7,92E-01	Prdm2		0,97	No
ENSMUSG00000057649	-3,25E-02	5,488667999	2,21E-01	6,39E-01	8,69E-01	Brd9		0,98	No
ENSMUSG00000057654	3,14E-02	-3,107617333	-7,69E-10	1,00E+00	1,00E+00	Olfr328		1,02	No
ENSMUSG00000057657	-1,54E-01	7,525205129	6,79E+00	9,19E-03	8,77E-02	Rps18-ps3		0,90	No
ENSMUSG00000057666	8,33E-02	6,913974839	1,87E+00	1,72E-01	5,28E-01	Gapdh		1,06	No
ENSMUSG00000057667	1,08E-01	4,048984869	1,53E+00	2,16E-01	5,50E-01	Bloc1s3		1,08	No
ENSMUSG00000057672	2,60E-02	7,92686888	1,98E-01	6,56E-01	8,80E-01	Pkn1		1,02	No
ENSMUSG00000057685	1,87E+00	-3,107639691	1,38E+00	2,40E-01	5,50E-01	Gm8526		3,67	No

ENSMUSG00000057691	1,21E-01	4,897302878	2,59E+00	1,07E-01	4,01E-01	Zfp746	1,09	No
ENSMUSG00000057696	-3,68E-01	3,592287463	1,45E+01	1,43E-04	3,26E-03	Rpl30-ps1	0,77	Yes
ENSMUSG00000057706	-2,16E-01	4,340681735	6,73E+00	9,49E-03	8,98E-02	Mex3b	0,86	No
ENSMUSG00000057716	-3,83E-02	-3,107651936	-7,91E-11	1,00E+00	1,00E+00	Tmem178b	0,97	No
ENSMUSG00000057722	4,30E-01	4,361895554	2,62E+01	3,12E-07	1,56E-05	Lepr	1,35	Yes
ENSMUSG00000057726	-2,63E-02	-3,033653157	2,33E-04	9,88E-01	1,00E+00	Serpinb9g	0,98	No
ENSMUSG00000057729	-1,00E+00	-0,871746024	5,63E+00	1,77E-02	1,39E-01	Prtn3	0,50	No
ENSMUSG00000057738	-1,45E-02	8,714313116	6,40E-02	8,00E-01	9,61E-01	Sptan1	0,99	No
ENSMUSG00000057751	-1,87E+00	-3,03364178	1,41E+00	2,36E-01	5,50E-01	Megf6	0,27	No
ENSMUSG00000057762	1,58E-01	7,347596081	7,00E+00	8,14E-03	8,00E-02	Gm6169	1,12	No
ENSMUSG00000057766	3,22E-01	-2,600712337	1,06E-01	7,45E-01	9,29E-01	Ankrd29	1,25	No
ENSMUSG00000057777	-7,02E-01	-2,800981932	1,97E-01	6,57E-01	8,80E-01	Mab21l2	0,61	No
ENSMUSG00000057778	-7,16E-02	2,931679614	4,03E-01	5,26E-01	8,03E-01	Cyb5d2	0,95	No
ENSMUSG00000057788	4,14E-02	5,086012499	3,19E-01	5,72E-01	8,32E-01	Ddx49	1,03	No
ENSMUSG00000057789	5,98E-02	7,254920443	9,97E-01	3,18E-01	6,19E-01	Bak1	1,04	No
ENSMUSG00000057802	9,01E-03	-3,107646452	-5,16E-10	1,00E+00	1,00E+00	Gm10030	1,01	No
ENSMUSG00000057817	2,62E+00	-2,963288024	2,65E+00	1,03E-01	3,95E-01	Olfr1446	6,14	No
ENSMUSG00000057818	1,87E+00	-3,03364371	1,36E+00	2,43E-01	5,50E-01	1600029O15Rik	3,66	No
ENSMUSG00000057835	-1,21E-01	1,783922989	5,82E-01	4,46E-01	7,36E-01	Zfp119a	0,92	No
ENSMUSG00000057841	-2,26E-01	6,850578285	1,37E+01	2,19E-04	4,69E-03	Rpl32	0,85	Yes
ENSMUSG00000057842	2,16E-01	2,570900053	2,88E+00	8,95E-02	3,79E-01	Zfp595	1,16	No
ENSMUSG00000057858	8,88E-02	3,012948987	6,02E-01	4,38E-01	7,30E-01	Fam204a	1,06	No
ENSMUSG00000057863	-2,12E-01	3,422274933	4,33E+00	3,75E-02	2,30E-01	Rpl36	0,86	No
ENSMUSG00000057880	-3,24E-01	-2,268089998	1,57E-01	6,92E-01	9,00E-01	Abat	0,80	No
ENSMUSG00000057886	5,95E-02	4,539822513	5,58E-01	4,55E-01	7,45E-01	Cbx3-ps6	1,04	No
ENSMUSG00000057894	-1,06E-01	2,883334096	8,97E-01	3,44E-01	6,44E-01	Zfp329	0,93	No
ENSMUSG00000057895	1,36E-01	1,316818549	5,40E-01	4,62E-01	7,51E-01	Zfp105	1,10	No
ENSMUSG00000057897	-7,79E-01	-0,378124734	5,74E+00	1,65E-02	1,34E-01	Camk2b	0,58	No
ENSMUSG00000057899	-7,08E-02	-2,49758507	6,95E-03	9,34E-01	1,00E+00	Adgrf2	0,95	No
ENSMUSG00000057914	9,10E-02	0,171827032	1,03E-01	7,48E-01	9,30E-01	Cacnb2	1,07	No
ENSMUSG00000057933	3,14E-02	-3,107617333	-7,69E-10	1,00E+00	1,00E+00	Gsta2	1,02	No
ENSMUSG00000057945	-4,26E-01	-2,929298692	1,13E-01	7,37E-01	9,25E-01	Gm17136	0,74	No

ENSMUSG00000057948	-1,96E-01	5,654982586	8,41E+00	3,73E-03	4,48E-02	Unc13d	0,87	Yes
ENSMUSG00000057963	2,96E-02	6,169889149	2,10E-01	6,47E-01	8,74E-01	Itpk1	1,02	No
ENSMUSG00000057967	-8,11E-02	-2,355510369	5,89E-03	9,39E-01	1,00E+00	Fgf18	0,95	No
ENSMUSG00000057969	1,54E+00	-1,655058537	5,51E+00	1,89E-02	1,46E-01	Sema3b	2,91	No
ENSMUSG00000057982	-1,32E-01	4,243266161	2,50E+00	1,14E-01	4,17E-01	Zfp809	0,91	No
ENSMUSG00000057990	-3,62E-01	3,479290323	1,33E+01	2,66E-04	5,49E-03	E030024N20Rik	0,78	Yes
ENSMUSG00000058006	2,06E-01	5,867176891	9,53E+00	2,02E-03	2,82E-02	Mdn1	1,15	Yes
ENSMUSG00000058013	1,35E-01	7,327852465	5,08E+00	2,42E-02	1,71E-01	40787	1,10	No
ENSMUSG00000058019	1,85E+00	-3,107657832	1,35E+00	2,46E-01	5,50E-01	Ces5a	3,61	No
ENSMUSG00000058022	-9,32E-01	0,144252859	1,15E+01	6,78E-04	1,17E-02	Adtrp	0,52	Yes
ENSMUSG00000058046	3,16E-01	2,023585807	4,39E+00	3,61E-02	2,25E-01	4933430I17Rik	1,24	No
ENSMUSG00000058050	-2,81E-01	2,649237747	5,16E+00	2,31E-02	1,66E-01	Gm9234	0,82	No
ENSMUSG00000058052	-1,94E+00	-3,107619243	1,50E+00	2,21E-01	5,50E-01	Defb35	0,26	No
ENSMUSG00000058056	-3,62E-01	5,63883826	2,88E+01	8,16E-08	4,65E-06	Palld	0,78	Yes
ENSMUSG00000058063	1,81E+00	-3,10765737	1,28E+00	2,58E-01	5,50E-01	Trim31	3,49	No
ENSMUSG00000058064	-3,02E-01	6,226616429	2,21E+01	2,58E-06	1,01E-04	Gm10036	0,81	Yes
ENSMUSG00000058070	1,61E+00	-2,522656062	1,77E+00	1,83E-01	5,46E-01	Eml1	3,05	No
ENSMUSG00000058073	-2,52E-01	2,80687039	4,49E+00	3,41E-02	2,18E-01	Gm11189	0,84	No
ENSMUSG00000058076	9,11E-02	6,39969266	2,07E+00	1,50E-01	4,91E-01	Sdhc	1,07	No
ENSMUSG00000058093	9,03E-02	3,510784469	8,27E-01	3,63E-01	6,64E-01	Zfp729b	1,06	No
ENSMUSG00000058099	1,15E-01	8,442932589	3,98E+00	4,61E-02	2,57E-01	Nfam1	1,08	No
ENSMUSG00000058101	8,12E-01	-0,980688368	3,30E+00	6,92E-02	3,31E-01	1700084J12Rik	1,76	No
ENSMUSG00000058114	1,81E+00	-3,10765737	1,28E+00	2,58E-01	5,50E-01	Olfr127	3,49	No
ENSMUSG00000058126	-4,56E-03	8,286825385	6,23E-03	9,37E-01	1,00E+00	Tpm3-rs7	1,00	No
ENSMUSG00000058135	-7,53E-02	5,77151828	1,26E+00	2,61E-01	5,55E-01	Gstm1	0,95	No
ENSMUSG00000058152	3,66E-01	1,56915882	4,67E+00	3,06E-02	2,02E-01	Chsy3	1,29	No
ENSMUSG00000058153	1,20E+00	-2,226341615	2,45E+00	1,18E-01	4,26E-01	Sez6l	2,30	No
ENSMUSG00000058159	-1,27E+00	-3,146104694	7,49E-01	3,87E-01	6,87E-01	T2	0,42	No
ENSMUSG00000058163	2,19E-01	4,412562971	7,30E+00	6,90E-03	7,10E-02	Gm5431	1,16	No
ENSMUSG00000058173	1,60E-01	2,259088355	1,28E+00	2,57E-01	5,50E-01	Smco4	1,12	No
ENSMUSG00000058174	-1,96E-01	7,68949154	1,11E+01	8,52E-04	1,42E-02	Gm5148	0,87	Yes
ENSMUSG00000058183	-1,26E+00	-1,726045096	3,50E+00	6,13E-02	3,08E-01	Mmel1	0,42	No

ENSMUSG00000058186	-1,24E+00	-2,655103303	1,47E+00	2,25E-01	5,50E-01	Zfp980	0,42	No
ENSMUSG00000058192	1,38E-01	2,765834824	1,42E+00	2,34E-01	5,50E-01	Zfp846	1,10	No
ENSMUSG00000058216	-4,13E-02	-3,033659495	7,08E-04	9,79E-01	1,00E+00	Gstp3	0,97	No
ENSMUSG00000058230	-1,36E-01	4,634987589	3,01E+00	8,28E-02	3,70E-01	Arhgap35	0,91	No
ENSMUSG00000058239	8,58E-02	6,318232711	1,81E+00	1,79E-01	5,39E-01	Usf2	1,06	No
ENSMUSG00000058240	3,80E-02	3,979084034	1,81E-01	6,71E-01	8,88E-01	Cryzl1	1,03	No
ENSMUSG00000058246	4,99E-02	-1,627658342	7,26E-03	9,32E-01	1,00E+00	Gm10037	1,04	No
ENSMUSG00000058248	-2,24E+00	-2,548215921	4,09E+00	4,32E-02	2,47E-01	Kcnh1	0,21	No
ENSMUSG00000058250	2,65E+00	-2,963270355	2,72E+00	9,93E-02	3,92E-01	Tas2r138	6,29	No
ENSMUSG00000058258	-1,47E-04	5,875575663	5,00E-06	9,98E-01	1,00E+00	Idi1	1,00	No
ENSMUSG00000058267	9,67E-02	4,677383784	1,53E+00	2,16E-01	5,50E-01	Mrps14	1,07	No
ENSMUSG00000058281	-7,88E-02	-2,832049512	2,63E-03	9,59E-01	1,00E+00	Gm10038	0,95	No
ENSMUSG00000058290	2,39E-01	5,448181573	1,18E+01	5,91E-04	1,05E-02	Espl1	1,18	Yes
ENSMUSG00000058291	-8,33E-02	4,212902225	9,68E-01	3,25E-01	6,26E-01	Zfp68	0,94	No
ENSMUSG00000058297	-5,48E-01	-1,998055333	6,55E-01	4,18E-01	7,13E-01	Spock2	0,68	No
ENSMUSG00000058298	-7,25E-02	4,532319281	8,19E-01	3,65E-01	6,66E-01	Mcm9	0,95	No
ENSMUSG00000058301	1,12E-01	6,292838244	3,10E+00	7,84E-02	3,58E-01	Upf1	1,08	No
ENSMUSG00000058317	-1,12E-01	2,100286275	5,85E-01	4,44E-01	7,36E-01	Ube2e2	0,93	No
ENSMUSG00000058318	-3,46E-01	5,225880795	2,34E+01	1,34E-06	5,76E-05	Phf21a	0,79	Yes
ENSMUSG00000058325	3,82E-03	5,614838049	3,11E-03	9,56E-01	1,00E+00	Dock1	1,00	No
ENSMUSG00000058331	-7,00E-02	1,107082782	1,27E-01	7,21E-01	9,16E-01	Zfp85	0,95	No
ENSMUSG00000058351	1,27E-01	2,786991865	1,12E+00	2,90E-01	5,86E-01	Smim4	1,09	No
ENSMUSG00000058355	7,85E-02	6,546785886	1,58E+00	2,09E-01	5,50E-01	Abce1	1,06	No
ENSMUSG00000058357	-1,13E+00	-2,497614886	7,53E-01	3,85E-01	6,87E-01	Gm6612	0,46	No
ENSMUSG00000058360	-1,33E+00	-2,831968119	1,11E+00	2,92E-01	5,88E-01	Gm10040	0,40	No
ENSMUSG00000058385	-1,35E-01	3,526004137	1,86E+00	1,73E-01	5,30E-01	Hist1h2bg	0,91	No
ENSMUSG00000058388	8,40E-02	4,467047521	1,08E+00	2,98E-01	5,94E-01	Phtf1	1,06	No
ENSMUSG00000058392	1,35E-01	4,474557262	2,77E+00	9,58E-02	3,86E-01	Rrp1b	1,10	No
ENSMUSG00000058396	1,02E-01	-1,107631585	5,04E-02	8,22E-01	9,72E-01	Gpr182	1,07	No
ENSMUSG00000058400	-8,86E-02	-2,963249454	3,57E-03	9,52E-01	1,00E+00	Qrfpr	0,94	No
ENSMUSG00000058402	1,25E-01	1,010529429	3,81E-01	5,37E-01	8,09E-01	Zfp420	1,09	No
ENSMUSG00000058407	-2,44E-02	5,630834705	1,28E-01	7,20E-01	9,16E-01	Txndc9	0,98	No

ENSMUSG00000058420	2,84E+00	-2,355705877	7,98E+00	4,74E-03	5,37E-02	Syt17	7,16	No
ENSMUSG00000058427	-2,23E-01	6,320636055	1,19E+01	5,69E-04	1,02E-02	Cxcl2	0,86	Yes
ENSMUSG00000058430	9,01E-03	-3,107646452	-5,16E-10	1,00E+00	1,00E+00	Rpl27a-ps2	1,01	No
ENSMUSG00000058435	-8,51E-01	-2,896137967	3,97E-01	5,28E-01	8,04E-01	Btnl4	0,55	No
ENSMUSG00000058440	2,30E-01	4,77817411	9,01E+00	2,68E-03	3,51E-02	Nrf1	1,17	Yes
ENSMUSG00000058441	-3,77E-01	-2,497572405	1,79E-01	6,72E-01	8,89E-01	Panx2	0,77	No
ENSMUSG00000058443	-2,31E-01	8,110247575	1,59E+01	6,64E-05	1,72E-03	Rpl10-ps3	0,85	Yes
ENSMUSG00000058444	-3,40E-02	3,870985047	1,39E-01	7,09E-01	9,09E-01	Map2k5	0,98	No
ENSMUSG00000058446	3,04E-02	8,34714363	2,79E-01	5,98E-01	8,45E-01	Znrf2	1,02	No
ENSMUSG00000058447	-8,97E-03	0,486641207	1,51E-03	9,69E-01	1,00E+00	Gm26920	0,99	No
ENSMUSG00000058454	2,86E-01	5,560312303	1,73E+01	3,22E-05	9,32E-04	Dhcr7	1,22	Yes
ENSMUSG00000058463	-1,87E+00	-3,033619006	1,41E+00	2,36E-01	5,50E-01	Gm5471	0,27	No
ENSMUSG00000058470	4,62E-01	-1,740802206	5,89E-01	4,43E-01	7,35E-01	Gm8369	1,38	No
ENSMUSG00000058486	1,67E-02	6,866264813	7,42E-02	7,85E-01	9,52E-01	Wdr91	1,01	No
ENSMUSG00000058492	-7,14E-02	5,856890726	1,16E+00	2,82E-01	5,79E-01	Scp2-ps2	0,95	No
ENSMUSG00000058498	-1,02E+00	-1,278586247	3,97E+00	4,63E-02	2,57E-01	Rnf207	0,49	No
ENSMUSG00000058503	-1,71E-01	3,873501946	3,61E+00	5,74E-02	2,95E-01	Fam133b	0,89	No
ENSMUSG00000058537	-1,89E+00	-3,1076441	1,42E+00	2,33E-01	5,50E-01	AW822073	0,27	No
ENSMUSG00000058542	2,13E-02	9,13447097	1,40E-01	7,08E-01	9,09E-01	Gm15590	1,01	No
ENSMUSG00000058546	-1,80E-01	4,532690094	5,11E+00	2,38E-02	1,69E-01	Rpl23a	0,88	No
ENSMUSG00000058558	-1,36E-01	5,992193649	4,35E+00	3,70E-02	2,28E-01	Rpl5	0,91	No
ENSMUSG00000058567	-2,49E-01	0,385670182	8,74E-01	3,50E-01	6,51E-01	Gm2531	0,84	No
ENSMUSG00000058569	1,49E-02	7,087458191	6,07E-02	8,05E-01	9,63E-01	Tmed9	1,01	No
ENSMUSG00000058571	2,07E-01	-2,355593794	5,38E-02	8,17E-01	9,69E-01	Gpc6	1,15	No
ENSMUSG00000058579	8,68E-02	0,516793528	1,25E-01	7,24E-01	9,17E-01	Cela2a	1,06	No
ENSMUSG00000058581	-1,14E-01	4,964055783	2,34E+00	1,26E-01	4,45E-01	Gm5801	0,92	No
ENSMUSG00000058586	1,08E-03	4,727559938	1,95E-04	9,89E-01	1,00E+00	Serhl	1,00	No
ENSMUSG00000058587	-4,35E-02	7,314753543	5,31E-01	4,66E-01	7,54E-01	Tmod3	0,97	No
ENSMUSG00000058588	-1,88E+00	-2,655099755	2,56E+00	1,09E-01	4,05E-01	Vmn1r21	0,27	No
ENSMUSG00000058589	-2,65E+00	-2,963225595	2,80E+00	9,41E-02	3,84E-01	Anks1b	0,16	No
ENSMUSG00000058594	-2,49E-02	6,549422023	1,60E-01	6,89E-01	8,99E-01	Fbxo18	0,98	No
ENSMUSG00000058600	-1,14E-01	5,261978935	2,56E+00	1,09E-01	4,06E-01	Rpl30	0,92	No

ENSMUSG00000058603	-1,27E-01	8,299617447	4,82E+00	2,81E-02	1,89E-01	Rpl28-ps1	0,92	No
ENSMUSG00000058620	2,59E+00	-2,832063622	2,61E+00	1,06E-01	3,98E-01	Adra2b	6,04	No
ENSMUSG00000058622	7,66E-01	-2,963249872	3,22E-01	5,70E-01	8,31E-01	Gm5265	1,70	No
ENSMUSG00000058624	-4,09E-02	7,763643209	4,84E-01	4,87E-01	7,74E-01	Gda	0,97	No
ENSMUSG00000058625	5,73E-02	6,688466684	8,56E-01	3,55E-01	6,56E-01	Gm17383	1,04	No
ENSMUSG00000058626	4,10E-02	0,062484703	2,05E-02	8,86E-01	1,00E+00	Capn11	1,03	No
ENSMUSG00000058638	-1,63E-02	5,20678086	5,18E-02	8,20E-01	9,71E-01	Zfp110	0,99	No
ENSMUSG00000058655	-6,90E-02	8,305306055	1,43E+00	2,32E-01	5,50E-01	Eif4b	0,95	No
ENSMUSG00000058656	-6,85E-02	-2,896124416	3,23E-03	9,55E-01	1,00E+00	Samd12	0,95	No
ENSMUSG00000058669	1,84E+00	-3,033649193	1,33E+00	2,49E-01	5,50E-01	Nkx2-9	3,58	No
ENSMUSG00000058672	-1,31E-01	6,744920145	4,49E+00	3,41E-02	2,17E-01	Tubb2a	0,91	No
ENSMUSG00000058690	-5,10E-01	6,391331166	6,53E+01	6,33E-16	1,06E-13	Ccser2	0,70	Yes
ENSMUSG00000058700	1,11E-02	-1,24712204	5,07E-04	9,82E-01	1,00E+00	Rpl21-ps12	1,01	No
ENSMUSG00000058704	5,71E-02	4,631853687	5,19E-01	4,71E-01	7,60E-01	Memo1	1,04	No
ENSMUSG00000058706	8,22E-02	4,96255991	1,24E+00	2,65E-01	5,60E-01	0610030E20Rik	1,06	No
ENSMUSG00000058709	1,17E-01	5,699594487	2,99E+00	8,38E-02	3,71E-01	Egln2	1,08	No
ENSMUSG00000058715	1,43E-01	8,486607599	6,22E+00	1,26E-02	1,11E-01	Fcer1g	1,10	No
ENSMUSG00000058717	-1,94E+00	-3,107619243	1,50E+00	2,21E-01	5,50E-01	Gm4763	0,26	No
ENSMUSG00000058728	-4,52E-01	1,432541667	6,50E+00	1,08E-02	9,89E-02	Cd300c	0,73	No
ENSMUSG00000058729	1,72E-01	3,418316267	2,84E+00	9,17E-02	3,82E-01	Lin9	1,13	No
ENSMUSG00000058740	-1,03E+00	-0,14596827	1,06E+01	1,10E-03	1,75E-02	Kcnt1	0,49	Yes
ENSMUSG00000058741	1,24E+00	-2,832043562	9,59E-01	3,28E-01	6,28E-01	Prr19	2,37	No
ENSMUSG00000058743	3,83E-01	-2,185605758	2,35E-01	6,28E-01	8,61E-01	Kcnj14	1,30	No
ENSMUSG00000058745	-3,24E-03	2,500999357	6,01E-04	9,80E-01	1,00E+00	Gm9174	1,00	No
ENSMUSG00000058748	5,15E-02	3,073631252	2,23E-01	6,37E-01	8,68E-01	Zfp958	1,04	No
ENSMUSG00000058755	1,41E-01	5,905960355	4,55E+00	3,30E-02	2,13E-01	Osm	1,10	No
ENSMUSG00000058756	1,35E-01	3,043826217	1,43E+00	2,32E-01	5,50E-01	Thra	1,10	No
ENSMUSG00000058761	4,98E-02	6,239799458	6,10E-01	4,35E-01	7,28E-01	Rnf169	1,04	No
ENSMUSG00000058773	3,53E-01	0,432112722	2,03E+00	1,54E-01	4,98E-01	Hist1h1b	1,28	No
ENSMUSG00000058793	2,70E-02	8,450677663	2,20E-01	6,39E-01	8,69E-01	Cds2	1,02	No
ENSMUSG00000058794	3,10E-01	3,876426762	1,14E+01	7,28E-04	1,25E-02	Nfe2	1,24	Yes
ENSMUSG00000058799	-2,90E-01	7,265104516	2,35E+01	1,26E-06	5,45E-05	Nap1l1	0,82	Yes

ENSMUSG00000058809	8,42E-02	6,96359059	1,91E+00	1,67E-01	5,20E-01	Hspd1-ps3	1,06	No
ENSMUSG00000058812	-5,01E-02	1,102089682	6,11E-02	8,05E-01	9,63E-01	0610039K10Rik	0,97	No
ENSMUSG00000058816	1,10E-01	1,244476161	3,25E-01	5,69E-01	8,30E-01	Ppp1r2-ps3	1,08	No
ENSMUSG00000058818	-3,68E-02	8,093293048	4,02E-01	5,26E-01	8,03E-01	Pirb	0,97	No
ENSMUSG00000058831	3,93E-01	-2,929263107	7,75E-02	7,81E-01	9,49E-01	Opn1sw	1,31	No
ENSMUSG00000058833	6,62E-02	4,110469929	5,79E-01	4,47E-01	7,37E-01	2810428I15Rik	1,05	No
ENSMUSG00000058835	-7,91E-02	7,530311565	1,79E+00	1,81E-01	5,43E-01	Abi1	0,95	No
ENSMUSG00000058838	-1,43E-01	7,285921763	5,70E+00	1,69E-02	1,36E-01	Rps27a-ps2	0,91	No
ENSMUSG00000058869	-1,06E-01	-3,033634182	5,86E-03	9,39E-01	1,00E+00	Gm4832	0,93	No
ENSMUSG00000058873	1,51E-01	2,051524082	1,01E+00	3,16E-01	6,16E-01	Gm5582	1,11	No
ENSMUSG00000058881	7,58E-02	6,639319608	1,49E+00	2,22E-01	5,50E-01	Zfp516	1,05	No
ENSMUSG00000058883	7,64E-01	-1,076343143	3,06E+00	8,05E-02	3,64E-01	Zfp708	1,70	No
ENSMUSG00000058886	2,71E-01	4,121686376	9,82E+00	1,72E-03	2,48E-02	Deaf1	1,21	Yes
ENSMUSG00000058897	-1,95E+00	-3,033628749	1,50E+00	2,21E-01	5,50E-01	Col25a1	0,26	No
ENSMUSG00000058900	9,21E-02	2,342694242	4,77E-01	4,90E-01	7,76E-01	Rsl1	1,07	No
ENSMUSG00000058905	-2,13E-01	7,046135796	1,24E+01	4,24E-04	8,00E-03	Gm10051	0,86	Yes
ENSMUSG00000058914	4,70E-01	-2,770578322	1,73E-01	6,78E-01	8,92E-01	C1qtnf3	1,38	No
ENSMUSG00000058921	-1,92E+00	-2,963268013	1,46E+00	2,27E-01	5,50E-01	Slc10a5	0,26	No
ENSMUSG00000058922	1,17E-02	7,773555812	3,96E-02	8,42E-01	9,85E-01	Gm10052	1,01	No
ENSMUSG00000058927	-8,62E-02	5,968418863	1,72E+00	1,90E-01	5,50E-01	Gm10053	0,94	No
ENSMUSG00000058934	-3,21E-01	-0,997902971	5,76E-01	4,48E-01	7,38E-01	Igf1os	0,80	No
ENSMUSG00000058935	-3,24E-02	-2,401332317	1,23E-03	9,72E-01	1,00E+00	Tex48	0,98	No
ENSMUSG00000058952	-3,19E+00	-2,896088812	4,27E+00	3,87E-02	2,33E-01	Cfi	0,11	No
ENSMUSG00000058966	1,36E-01	5,33341584	3,68E+00	5,51E-02	2,88E-01	Fam57b	1,10	No
ENSMUSG00000058975	-2,66E+00	-2,896122337	2,83E+00	9,26E-02	3,83E-01	Kcnc1	0,16	No
ENSMUSG00000058976	-5,25E-01	-2,226322912	4,42E-01	5,06E-01	7,91E-01	Usp17lc	0,69	No
ENSMUSG00000058979	1,87E-01	3,869989645	4,22E+00	4,00E-02	2,37E-01	Hdhd5	1,14	No
ENSMUSG00000058997	1,02E-01	5,05629919	1,92E+00	1,65E-01	5,18E-01	Vwa8	1,07	No
ENSMUSG00000059005	4,27E-02	6,330955436	4,50E-01	5,02E-01	7,87E-01	Hnrnpa3	1,03	No
ENSMUSG00000059013	3,14E-01	4,378153859	1,48E+01	1,19E-04	2,81E-03	Sh2d3c	1,24	Yes
ENSMUSG00000059022	-5,08E-01	5,858309314	5,94E+01	1,28E-14	1,88E-12	Kcp	0,70	Yes
ENSMUSG00000059027	-5,13E-01	-0,378128443	2,32E+00	1,28E-01	4,49E-01	9630013D21Rik	0,70	No

ENSMUSG00000059031	-2,09E-01	2,166422254	2,19E+00	1,39E-01	4,71E-01	Olfr482		0,87	No
ENSMUSG00000059033	-1,73E-01	1,210951938	7,80E-01	3,77E-01	6,78E-01	Rpl18a-ps1		0,89	No
ENSMUSG00000059040	1,80E-01	9,24440717	1,00E+01	1,55E-03	2,29E-02	Eno1b		1,13	Yes
ENSMUSG00000059042	6,18E-01	4,49360339	5,57E+01	8,60E-14	1,20E-11	Klk1b9		1,53	Yes
ENSMUSG00000059049	-2,80E-01	-2,35556794	1,36E-01	7,13E-01	9,11E-01	Frem1		0,82	No
ENSMUSG00000059058	-1,43E-01	5,899395912	4,70E+00	3,01E-02	1,99E-01	Tma7-ps		0,91	No
ENSMUSG00000059060	-1,97E-01	0,844047054	8,16E-01	3,66E-01	6,67E-01	Rad51b		0,87	No
ENSMUSG00000059064	-7,60E-01	-2,185570254	1,18E+00	2,78E-01	5,74E-01	Gm10059		0,59	No
ENSMUSG00000059070	-1,24E-01	5,715209962	3,39E+00	6,55E-02	3,21E-01	Rpl18		0,92	No
ENSMUSG00000059087	2,56E+00	-2,896188915	2,56E+00	1,10E-01	4,06E-01	Olfr698		5,91	No
ENSMUSG00000059089	-8,67E-01	2,503723727	4,15E+01	1,20E-10	1,12E-08	Fcgr4		0,55	Yes
ENSMUSG00000059106	-1,89E+00	-3,1076441	1,42E+00	2,33E-01	5,50E-01	Olfr961		0,27	No
ENSMUSG00000059108	-1,79E+00	2,032521522	1,32E+02	1,81E-30	1,09E-27	Ifitm6		0,29	Yes
ENSMUSG00000059119	5,64E-02	6,446010687	8,02E-01	3,71E-01	6,72E-01	Nap1l4		1,04	No
ENSMUSG00000059134	-5,25E-02	-3,033661434	4,08E-11	1,00E+00	1,00E+00	Olfr814		0,96	No
ENSMUSG00000059136	9,01E-03	-3,107646452	-5,16E-10	1,00E+00	1,00E+00	Olfr539		1,01	No
ENSMUSG00000059142	-3,39E-01	4,435787404	1,80E+01	2,26E-05	6,92E-04	Zfp945		0,79	Yes
ENSMUSG00000059149	1,11E-01	4,289962813	1,73E+00	1,88E-01	5,50E-01	Mfsd4a		1,08	No
ENSMUSG00000059150	7,50E-01	-1,574291434	1,89E+00	1,69E-01	5,24E-01	Rpl26-ps4		1,68	No
ENSMUSG00000059159	-1,98E-01	5,87182233	8,97E+00	2,75E-03	3,58E-02	Gm8129		0,87	Yes
ENSMUSG00000059173	9,01E-03	-3,107646452	-5,16E-10	1,00E+00	1,00E+00	Pde1a		1,01	No
ENSMUSG00000059179	-4,97E-02	4,268886065	3,41E-01	5,60E-01	8,22E-01	Gm8991		0,97	No
ENSMUSG00000059182	3,54E-02	7,188729447	3,47E-01	5,56E-01	8,21E-01	Skap2		1,02	No
ENSMUSG00000059183	1,31E-01	3,912745652	2,13E+00	1,44E-01	4,80E-01	Mtfmt		1,10	No
ENSMUSG00000059195	-5,09E-02	11,13064523	8,29E-01	3,63E-01	6,64E-01	Gm12715		0,97	No
ENSMUSG00000059208	5,31E-02	7,636646621	8,10E-01	3,68E-01	6,69E-01	Hnrnpm		1,04	No
ENSMUSG00000059213	1,12E+00	-2,655145496	1,18E+00	2,77E-01	5,73E-01	Ddn		2,17	No
ENSMUSG00000059237	-1,09E-01	3,82340988	1,39E+00	2,39E-01	5,50E-01	Ppp1r2-ps4		0,93	No
ENSMUSG00000059244	2,87E-01	-2,83201524	5,07E-02	8,22E-01	9,72E-01	Gm10062		1,22	No
ENSMUSG00000059248	1,71E-01	8,765347035	8,99E+00	2,71E-03	3,54E-02	40057		1,13	Yes
ENSMUSG00000059252	-1,37E-01	3,278653619	1,71E+00	1,90E-01	5,50E-01	Tpt1-ps5		0,91	No
ENSMUSG00000059263	1,58E-01	6,923813132	6,72E+00	9,53E-03	8,99E-02	Usp47		1,12	No

ENSMUSG00000059273	7,46E-02	6,581975834	1,44E+00	2,31E-01	5,50E-01	Zc3h4	1,05	No
ENSMUSG00000059277	9,46E-03	2,530135877	5,23E-03	9,42E-01	1,00E+00	R74862	1,01	No
ENSMUSG00000059278	-6,54E-02	4,042077516	5,48E-01	4,59E-01	7,48E-01	Naa38	0,96	No
ENSMUSG00000059279	-3,83E-02	-3,107651936	-7,91E-11	1,00E+00	1,00E+00	Olfr224	0,97	No
ENSMUSG00000059288	6,00E-02	4,219975045	4,94E-01	4,82E-01	7,70E-01	Cdyl	1,04	No
ENSMUSG00000059291	-2,51E-01	4,634711776	1,03E+01	1,33E-03	2,03E-02	Rpl11	0,84	Yes
ENSMUSG00000059301	-1,06E-01	1,566271629	3,68E-01	5,44E-01	8,13E-01	Gm5434	0,93	No
ENSMUSG00000059305	-7,64E-01	-1,879748081	1,19E+00	2,75E-01	5,71E-01	Vpreb1	0,59	No
ENSMUSG00000059316	2,00E-01	5,60953676	8,49E+00	3,57E-03	4,36E-02	Slc27a4	1,15	Yes
ENSMUSG00000059323	-4,42E-02	5,655002393	4,28E-01	5,13E-01	7,95E-01	Tonsl	0,97	No
ENSMUSG00000059325	8,90E-02	4,354397912	1,15E+00	2,84E-01	5,81E-01	Hopx	1,06	No
ENSMUSG00000059326	1,65E-01	8,739706999	8,30E+00	3,97E-03	4,71E-02	Csf2ra	1,12	Yes
ENSMUSG00000059336	-4,41E-02	1,896938007	8,12E-02	7,76E-01	9,45E-01	Slc14a1	0,97	No
ENSMUSG00000059343	9,04E-02	7,24076344	2,27E+00	1,32E-01	4,56E-01	Aldoart1	1,06	No
ENSMUSG00000059352	-1,95E+00	-3,033628749	1,50E+00	2,21E-01	5,50E-01	Gm10064	0,26	No
ENSMUSG00000059355	-9,99E-03	3,919287421	1,21E-02	9,12E-01	1,00E+00	Wdr83os	0,99	No
ENSMUSG00000059363	3,82E-01	0,550981906	2,62E+00	1,06E-01	3,98E-01	Fxn	1,30	No
ENSMUSG00000059395	8,50E-02	-0,669281562	4,62E-02	8,30E-01	9,77E-01	Nkapl	1,06	No
ENSMUSG00000059397	-1,87E+00	-3,107637761	1,39E+00	2,38E-01	5,50E-01	Olfr54	0,27	No
ENSMUSG00000059406	5,83E-01	-1,998083955	7,31E-01	3,92E-01	6,93E-01	Tmprss9	1,50	No
ENSMUSG00000059408	-3,83E-02	-3,107651936	-7,91E-11	1,00E+00	1,00E+00	Mrgprh	0,97	No
ENSMUSG00000059409	-1,23E-03	6,30655525	3,74E-04	9,85E-01	1,00E+00	Ppp2r5d	1,00	No
ENSMUSG00000059412	1,81E-01	-0,641329398	2,16E-01	6,42E-01	8,71E-01	Fxyd2	1,13	No
ENSMUSG00000059422	1,48E-01	3,980701898	2,75E+00	9,74E-02	3,89E-01	Gm8116	1,11	No
ENSMUSG00000059423	-3,93E-01	3,690201632	1,75E+01	2,86E-05	8,42E-04	Zfp933	0,76	Yes
ENSMUSG00000059429	-1,94E+00	-3,107619243	1,50E+00	2,21E-01	5,50E-01	Olfr365	0,26	No
ENSMUSG00000059430	3,43E-01	-1,54841855	3,79E-01	5,38E-01	8,09E-01	Actg2	1,27	No
ENSMUSG00000059434	3,14E-02	-3,107617333	-7,69E-10	1,00E+00	1,00E+00	Gckr	1,02	No
ENSMUSG00000059436	4,73E-02	6,238716272	5,45E-01	4,60E-01	7,49E-01	Max	1,03	No
ENSMUSG00000059439	3,19E-02	4,531534675	1,58E-01	6,91E-01	9,00E-01	Bcas3	1,02	No
ENSMUSG00000059447	-9,35E-03	4,346793392	1,27E-02	9,10E-01	1,00E+00	Hadhb	0,99	No
ENSMUSG00000059456	-7,29E-02	6,931969376	1,43E+00	2,32E-01	5,50E-01	Ptk2b	0,95	No

ENSMUSG00000059459	-6,64E-02	-2,247078195	7,14E-03	9,33E-01	1,00E+00	Gm10065	0,96	No
ENSMUSG00000059460	-3,83E-02	-3,107651936	-7,91E-11	1,00E+00	1,00E+00	Olfr286	0,97	No
ENSMUSG00000059463	-1,60E+00	-2,185539083	4,30E+00	3,81E-02	2,32E-01	Spag11b	0,33	No
ENSMUSG00000059474	-1,20E-01	6,344020265	3,57E+00	5,87E-02	3,00E-01	Mbtd1	0,92	No
ENSMUSG00000059475	9,50E-02	4,216067466	1,30E+00	2,54E-01	5,50E-01	Zfp426	1,07	No
ENSMUSG00000059479	1,27E-01	5,384839618	3,23E+00	7,22E-02	3,41E-01	B3gnt8	1,09	No
ENSMUSG00000059481	1,87E+00	-3,107639691	1,38E+00	2,40E-01	5,50E-01	Plg	3,67	No
ENSMUSG00000059482	1,16E-01	3,385377606	1,29E+00	2,56E-01	5,50E-01	2610301B20Rik	1,08	No
ENSMUSG00000059486	1,38E-01	5,510015917	3,98E+00	4,60E-02	2,56E-01	Kbtbd2	1,10	No
ENSMUSG00000059495	-1,14E-01	5,653568279	2,82E+00	9,31E-02	3,83E-01	Arhgef12	0,92	No
ENSMUSG00000059498	1,78E-01	9,460093837	9,88E+00	1,67E-03	2,43E-02	Fcgr3	1,13	Yes
ENSMUSG00000059518	2,95E-02	4,832608369	1,48E-01	7,01E-01	9,05E-01	Znhit1	1,02	No
ENSMUSG00000059534	-7,81E-02	6,136698657	1,47E+00	2,26E-01	5,50E-01	Uqcr10	0,95	No
ENSMUSG00000059540	-2,19E-01	1,897652881	1,97E+00	1,60E-01	5,08E-01	Tcea2	0,86	No
ENSMUSG00000059552	3,81E-02	5,905528397	3,34E-01	5,63E-01	8,26E-01	Trp53	1,03	No
ENSMUSG00000059554	-8,42E-03	2,995801549	5,65E-03	9,40E-01	1,00E+00	Ccdc28a	0,99	No
ENSMUSG00000059555	2,42E-01	6,014585612	1,39E+01	1,88E-04	4,13E-03	Tor4a	1,18	Yes
ENSMUSG00000059562	-2,80E-01	-1,107613771	3,75E-01	5,40E-01	8,10E-01	Ccdc154	0,82	No
ENSMUSG00000059565	-6,81E-02	-2,896149338	3,18E-03	9,55E-01	1,00E+00	Gm5292	0,95	No
ENSMUSG00000059571	-1,89E+00	-3,1076441	1,42E+00	2,33E-01	5,50E-01	Gm16218	0,27	No
ENSMUSG00000059585	-7,45E-02	1,455300964	1,67E-01	6,83E-01	8,95E-01	Ube2nl	0,95	No
ENSMUSG00000059586	1,53E-01	4,286048899	3,32E+00	6,86E-02	3,29E-01	Nsmce2	1,11	No
ENSMUSG00000059588	3,04E-01	3,188333506	8,18E+00	4,24E-03	4,94E-02	Calcrl	1,23	Yes
ENSMUSG00000059602	-9,40E-01	1,57736087	3,35E+01	7,04E-09	5,03E-07	Syn3	0,52	Yes
ENSMUSG00000059606	4,96E-01	-0,522710821	1,80E+00	1,80E-01	5,41E-01	Rnase2b	1,41	No
ENSMUSG00000059610	-1,90E+00	-2,963239763	1,44E+00	2,31E-01	5,50E-01	Olfr222	0,27	No
ENSMUSG00000059625	-1,87E+00	-3,107637761	1,39E+00	2,38E-01	5,50E-01	Sohlh1	0,27	No
ENSMUSG00000059631	-1,87E+00	-3,033619006	1,41E+00	2,36E-01	5,50E-01	1500035N22Rik	0,27	No
ENSMUSG00000059639	2,44E-01	3,071157241	4,66E+00	3,09E-02	2,03E-01	Clec4a4	1,18	No
ENSMUSG00000059647	5,80E-02	3,191244013	2,93E-01	5,89E-01	8,41E-01	Cbx3-ps7	1,04	No
ENSMUSG00000059657	1,44E-01	-1,548287492	6,79E-02	7,94E-01	9,56E-01	Stfa2l1	1,11	No
ENSMUSG00000059658	1,74E-01	2,533882937	1,84E+00	1,75E-01	5,33E-01	Gm16379	1,13	No

ENSMUSG00000059659	1,06E-01	1,935301921	4,76E-01	4,90E-01	7,76E-01	Gm10069	1,08	No
ENSMUSG00000059668	-1,94E+00	-3,107619243	1,50E+00	2,21E-01	5,50E-01	Krt4	0,26	No
ENSMUSG00000059669	-2,81E-01	3,274552351	7,21E+00	7,27E-03	7,38E-02	Taf1b	0,82	No
ENSMUSG00000059674	1,36E-01	0,707652342	3,42E-01	5,59E-01	8,22E-01	Cdh24	1,10	No
ENSMUSG00000059687	4,43E-01	-2,497595722	1,53E-01	6,95E-01	9,02E-01	Olfr108	1,36	No
ENSMUSG00000059689	-4,53E-02	3,506686054	2,12E-01	6,45E-01	8,73E-01	Zfp637	0,97	No
ENSMUSG00000059706	-1,94E+00	-3,107619243	1,50E+00	2,21E-01	5,50E-01	A830035A12Rik	0,26	No
ENSMUSG00000059713	8,79E-02	6,218380333	1,89E+00	1,69E-01	5,25E-01	Rcan3	1,06	No
ENSMUSG00000059714	-6,27E-02	5,792125807	8,79E-01	3,48E-01	6,49E-01	Flot1	0,96	No
ENSMUSG00000059729	-8,41E-01	-2,89611984	3,88E-01	5,33E-01	8,06E-01	Olfr1385	0,56	No
ENSMUSG00000059734	-6,06E-02	4,861598722	6,38E-01	4,25E-01	7,19E-01	Ndufs8	0,96	No
ENSMUSG00000059742	-6,25E-01	1,140404956	1,01E+01	1,46E-03	2,19E-02	Kcnh7	0,65	Yes
ENSMUSG00000059743	2,55E-01	3,860301429	7,73E+00	5,44E-03	5,94E-02	Fdps	1,19	No
ENSMUSG00000059751	-2,04E-01	7,23724169	1,16E+01	6,59E-04	1,15E-02	Rps3a3	0,87	Yes
ENSMUSG00000059772	-2,22E-02	3,350112942	4,80E-02	8,27E-01	9,75E-01	Slx1b	0,98	No
ENSMUSG00000059775	-1,83E-01	8,55130276	1,01E+01	1,45E-03	2,18E-02	Rps26-ps1	0,88	Yes
ENSMUSG00000059776	-1,35E-01	7,242263448	5,08E+00	2,41E-02	1,71E-01	Rpl13-ps6	0,91	No
ENSMUSG00000059791	2,30E-01	4,002292397	6,61E+00	1,02E-02	9,44E-02	Nrm	1,17	No
ENSMUSG00000059796	2,79E-02	8,030882882	2,30E-01	6,32E-01	8,64E-01	Eif4a1	1,02	No
ENSMUSG00000059810	-1,26E-01	4,559655497	2,57E+00	1,09E-01	4,04E-01	Rgs3	0,92	No
ENSMUSG00000059811	-1,13E-02	4,884846271	2,28E-02	8,80E-01	1,00E+00	Atl2	0,99	No
ENSMUSG00000059816	-8,45E-01	-2,963229433	3,92E-01	5,31E-01	8,05E-01	Fam159a	0,56	No
ENSMUSG00000059820	-9,75E-02	5,112190963	1,79E+00	1,81E-01	5,42E-01	AU019823	0,93	No
ENSMUSG00000059824	-1,20E-01	1,737150025	5,58E-01	4,55E-01	7,45E-01	Dbp	0,92	No
ENSMUSG00000059827	-1,98E-02	-3,033655952	2,83E-11	1,00E+00	1,00E+00	Gm5670	0,99	No
ENSMUSG00000059830	4,33E-02	2,411592278	1,09E-01	7,41E-01	9,27E-01	Gm16380	1,03	No
ENSMUSG00000059834	7,21E-03	2,881747166	3,83E-03	9,51E-01	1,00E+00	Sclt1	1,01	No
ENSMUSG00000059835	-2,85E-01	4,16773509	1,09E+01	9,83E-04	1,60E-02	Rpl13-ps3	0,82	Yes
ENSMUSG00000059839	2,00E-01	1,774318202	1,58E+00	2,09E-01	5,50E-01	Zfp874b	1,15	No
ENSMUSG00000059842	3,98E-02	2,169337973	8,34E-02	7,73E-01	9,44E-01	Zfp341	1,03	No
ENSMUSG00000059851	5,71E-02	6,417724138	8,24E-01	3,64E-01	6,65E-01	Kmt5c	1,04	No
ENSMUSG00000059852	2,29E-02	-0,289418706	5,19E-03	9,43E-01	1,00E+00	Kcng2	1,02	No

ENSMUSG00000059854	-1,38E+00	-2,831984188	1,20E+00	2,74E-01	5,71E-01	Hydin	0,38	No
ENSMUSG00000059857	1,87E+00	-3,107639691	1,38E+00	2,40E-01	5,50E-01	Ntng1	3,67	No
ENSMUSG00000059864	-1,77E-01	-0,510167502	2,33E-01	6,29E-01	8,62E-01	Olfr1393	0,88	No
ENSMUSG00000059866	9,66E-03	5,850702771	2,12E-02	8,84E-01	1,00E+00	Tnip2	1,01	No
ENSMUSG00000059873	1,87E+00	-3,107639691	1,38E+00	2,40E-01	5,50E-01	Olfr1029	3,67	No
ENSMUSG00000059878	-1,51E-02	4,654846605	3,77E-02	8,46E-01	9,87E-01	Zfp422	0,99	No
ENSMUSG00000059883	2,20E-02	5,673364235	1,06E-01	7,45E-01	9,29E-01	Irak4	1,02	No
ENSMUSG00000059887	-3,83E-02	-3,107651936	-7,91E-11	1,00E+00	1,00E+00	Olfr1507	0,97	No
ENSMUSG00000059890	-3,51E-03	7,010265495	3,35E-03	9,54E-01	1,00E+00	Ube4a	1,00	No
ENSMUSG00000059891	1,72E-01	-2,448739611	4,40E-02	8,34E-01	9,79E-01	Tsks	1,13	No
ENSMUSG00000059895	8,85E-03	4,832784941	1,35E-02	9,07E-01	1,00E+00	Ptp4a3	1,01	No
ENSMUSG00000059897	3,82E-02	2,411676389	8,54E-02	7,70E-01	9,42E-01	Zfp930	1,03	No
ENSMUSG00000059900	1,77E-01	0,742231912	6,34E-01	4,26E-01	7,20E-01	Tmem40	1,13	No
ENSMUSG00000059901	-7,98E-01	-1,627582166	1,88E+00	1,70E-01	5,26E-01	Adamts14	0,58	No
ENSMUSG00000059912	3,43E-02	1,152067128	3,12E-02	8,60E-01	9,95E-01	Rpl21-ps6	1,02	No
ENSMUSG00000059920	6,22E-02	4,64352825	6,25E-01	4,29E-01	7,23E-01	4930453N24Rik	1,04	No
ENSMUSG00000059921	-2,41E-01	-0,832149776	3,58E-01	5,50E-01	8,17E-01	Unc5c	0,85	No
ENSMUSG00000059923	-7,01E-02	7,637972109	1,42E+00	2,34E-01	5,50E-01	Grb2	0,95	No
ENSMUSG00000059939	-3,30E-02	4,345730428	1,62E-01	6,87E-01	8,98E-01	9430015G10Rik	0,98	No
ENSMUSG00000059956	5,67E-01	1,968444322	1,31E+01	3,01E-04	6,07E-03	Serpinb12	1,48	Yes
ENSMUSG00000059970	1,97E-01	1,829310847	1,46E+00	2,27E-01	5,50E-01	Hspa2	1,15	No
ENSMUSG00000059975	1,03E-02	2,784846159	7,61E-03	9,30E-01	1,00E+00	Zfp74	1,01	No
ENSMUSG00000059981	3,45E-02	6,521506794	3,06E-01	5,80E-01	8,37E-01	Taok2	1,02	No
ENSMUSG00000059991	-1,60E+00	-1,472837707	8,96E+00	2,76E-03	3,58E-02	Nptx2	0,33	Yes
ENSMUSG00000059994	-4,98E-02	0,52104397	3,99E-02	8,42E-01	9,85E-01	Fcrl1	0,97	No
ENSMUSG00000059995	-3,74E-02	6,489120589	3,57E-01	5,50E-01	8,17E-01	Atxn7l3	0,97	No
ENSMUSG00000060002	-6,60E-02	4,377193896	6,40E-01	4,24E-01	7,18E-01	Chpt1	0,96	No
ENSMUSG00000060012	-1,20E-01	5,720774396	3,18E+00	7,46E-02	3,47E-01	Kif13b	0,92	No
ENSMUSG00000060019	-9,63E-02	8,653546162	2,82E+00	9,30E-02	3,83E-01	Gm10073	0,94	No
ENSMUSG00000060029	-2,24E-02	2,789939572	3,54E-02	8,51E-01	9,90E-01	4930473A02Rik	0,98	No
ENSMUSG00000060032	4,12E-02	5,339858819	3,36E-01	5,62E-01	8,25E-01	H2afj	1,03	No
ENSMUSG00000060034	-1,06E+00	-0,711452475	6,93E+00	8,47E-03	8,27E-02	Ctf2	0,48	No

ENSMUSG00000060036	-1,16E-02	6,901695915	3,63E-02	8,49E-01	9,88E-01	Rpl3	0,99	No
ENSMUSG00000060038	-2,82E-02	4,265383656	1,12E-01	7,38E-01	9,25E-01	Dhps	0,98	No
ENSMUSG00000060044	-1,15E+00	-0,963290706	7,09E+00	7,74E-03	7,71E-02	Tmem26	0,45	No
ENSMUSG00000060049	-1,95E+00	-3,033628749	1,50E+00	2,21E-01	5,50E-01	Olfr235	0,26	No
ENSMUSG00000060063	1,95E-01	7,719659244	1,09E+01	9,52E-04	1,56E-02	Alox5ap	1,14	Yes
ENSMUSG00000060068	2,05E-01	2,633575993	2,61E+00	1,06E-01	3,99E-01	Gm12222	1,15	No
ENSMUSG00000060073	-2,17E-02	3,31486385	4,36E-02	8,35E-01	9,80E-01	Psma3	0,99	No
ENSMUSG00000060093	-7,41E-01	-1,424897595	1,76E+00	1,85E-01	5,49E-01	Hist1h4a	0,60	No
ENSMUSG00000060096	2,75E-01	3,661839252	8,18E+00	4,24E-03	4,94E-02	Amd-ps3	1,21	Yes
ENSMUSG00000060098	4,27E-02	4,154944678	2,49E-01	6,18E-01	8,55E-01	Prmt7	1,03	No
ENSMUSG00000060121	1,66E-01	2,439958394	1,60E+00	2,06E-01	5,50E-01	Gemin2	1,12	No
ENSMUSG00000060126	-1,45E-01	7,290472508	5,90E+00	1,51E-02	1,27E-01	Tpt1	0,90	No
ENSMUSG00000060128	-1,42E-01	-0,074645069	2,11E-01	6,46E-01	8,74E-01	Gm2710	0,91	No
ENSMUSG00000060131	2,89E-01	7,398162868	2,35E+01	1,27E-06	5,48E-05	Atp8b4	1,22	Yes
ENSMUSG00000060143	-2,02E-01	7,007846712	1,10E+01	9,12E-04	1,50E-02	Gm10076	0,87	Yes
ENSMUSG00000060147	-1,75E-02	6,808308042	8,12E-02	7,76E-01	9,45E-01	Serpinb6a	0,99	No
ENSMUSG00000060149	1,77E-01	2,586759656	1,98E+00	1,60E-01	5,07E-01	BC002059	1,13	No
ENSMUSG00000060152	-3,82E-02	4,396927063	2,16E-01	6,42E-01	8,71E-01	Pop5	0,97	No
ENSMUSG00000060161	-6,64E-01	-1,448651582	1,51E+00	2,18E-01	5,50E-01	Klk1b7-ps	0,63	No
ENSMUSG00000060166	5,62E-02	5,491693005	6,58E-01	4,17E-01	7,12E-01	Zdhhc8	1,04	No
ENSMUSG00000060170	9,01E-03	-3,107646452	-5,16E-10	1,00E+00	1,00E+00	Olfr1371	1,01	No
ENSMUSG00000060176	-7,66E-01	-1,322017726	2,51E+00	1,13E-01	4,15E-01	Kif27	0,59	No
ENSMUSG00000060180	-7,38E-02	-3,033659033	2,66E-03	9,59E-01	1,00E+00	Myh13	0,95	No
ENSMUSG00000060181	2,01E-01	3,435130624	4,10E+00	4,28E-02	2,46E-01	Slc35e3	1,15	No
ENSMUSG00000060183	4,94E-03	-2,896132518	-4,80E-12	1,00E+00	1,00E+00	Cxcl11	1,00	No
ENSMUSG00000060187	1,81E+00	-3,10765737	1,28E+00	2,58E-01	5,50E-01	Lrrc10	3,49	No
ENSMUSG00000060188	-1,92E+00	-3,033623267	1,46E+00	2,27E-01	5,50E-01	Cxcl17	0,26	No
ENSMUSG00000060198	-7,08E-02	6,943753257	1,35E+00	2,45E-01	5,50E-01	Gm11353	0,95	No
ENSMUSG00000060206	-2,19E-01	-1,997962988	1,06E-01	7,45E-01	9,29E-01	Zfp462	0,86	No
ENSMUSG00000060212	-2,68E-01	-0,929272359	4,09E-01	5,23E-01	8,01E-01	Pcnx2	0,83	No
ENSMUSG00000060216	-1,69E-01	6,954053925	7,68E+00	5,57E-03	6,05E-02	Arrb2	0,89	No
ENSMUSG00000060224	2,48E-01	-0,136537112	6,02E-01	4,38E-01	7,31E-01	Pyroxd2	1,19	No

ENSMUSG00000060227	-5,34E-02	-1,998071876	5,95E-03	9,39E-01	1,00E+00	Casc4	0,96	No
ENSMUSG00000060240	-1,50E-01	-1,424816235	9,76E-02	7,55E-01	9,34E-01	Cend1	0,90	No
ENSMUSG00000060244	-1,37E-01	1,750525675	6,87E-01	4,07E-01	7,04E-01	Alyref2	0,91	No
ENSMUSG00000060256	-1,89E+00	-3,1076441	1,42E+00	2,33E-01	5,50E-01	Tdpoz4	0,27	No
ENSMUSG00000060257	-1,89E+00	-3,1076441	1,42E+00	2,33E-01	5,50E-01	Scrt2	0,27	No
ENSMUSG00000060260	2,74E-01	3,829263489	9,04E+00	2,64E-03	3,47E-02	Pwwp2b	1,21	Yes
ENSMUSG00000060261	6,12E-02	5,970923136	8,68E-01	3,51E-01	6,53E-01	Gtf2i	1,04	No
ENSMUSG00000060268	3,18E-01	-1,896147098	2,28E-01	6,33E-01	8,65E-01	Gm1661	1,25	No
ENSMUSG00000060275	1,98E-01	-0,770547565	2,49E-01	6,18E-01	8,55E-01	Nrg2	1,15	No
ENSMUSG00000060279	9,08E-02	6,664365833	2,14E+00	1,44E-01	4,79E-01	Ap2a1	1,06	No
ENSMUSG00000060284	-5,85E-01	-1,740762404	9,47E-01	3,31E-01	6,31E-01	Sp7	0,67	No
ENSMUSG00000060288	1,19E-02	2,819109156	9,85E-03	9,21E-01	1,00E+00	Ppih	1,01	No
ENSMUSG00000060301	6,43E-02	2,593459618	2,85E-01	5,94E-01	8,43E-01	2610008E11Rik	1,05	No
ENSMUSG00000060311	4,52E-01	3,751052616	2,27E+01	1,90E-06	7,77E-05	Mucl1	1,37	Yes
ENSMUSG00000060314	-4,21E-01	-0,733281205	1,14E+00	2,85E-01	5,82E-01	Zfp941	0,75	No
ENSMUSG00000060332	-1,33E+00	-2,83201525	1,11E+00	2,93E-01	5,88E-01	Tmc2	0,40	No
ENSMUSG00000060336	1,73E-01	0,40581333	4,93E-01	4,83E-01	7,70E-01	Zfp937	1,13	No
ENSMUSG00000060371	9,01E-03	-3,107646452	-5,16E-10	1,00E+00	1,00E+00	Caln1	1,01	No
ENSMUSG00000060373	-1,12E-01	7,171694344	3,47E+00	6,25E-02	3,11E-01	Hnrnpc	0,93	No
ENSMUSG00000060376	-1,61E-02	4,38335229	3,84E-02	8,45E-01	9,86E-01	Bckdha	0,99	No
ENSMUSG00000060377	-1,34E-01	5,038388505	3,34E+00	6,78E-02	3,28E-01	Rpl36a-ps1	0,91	No
ENSMUSG00000060380	-2,10E-01	0,0240095	4,92E-01	4,83E-01	7,70E-01	C030014I23Rik	0,86	No
ENSMUSG00000060397	1,01E-01	1,395380557	3,53E-01	5,52E-01	8,19E-01	Zfp128	1,07	No
ENSMUSG00000060402	-6,81E-02	-2,711721797	4,24E-03	9,48E-01	1,00E+00	Chst8	0,95	No
ENSMUSG00000060404	-1,94E+00	-3,107619243	1,50E+00	2,21E-01	5,50E-01	Olfr1369-ps1	0,26	No
ENSMUSG00000060416	1,24E+00	-2,770618059	9,48E-01	3,30E-01	6,31E-01	Gm839	2,36	No
ENSMUSG00000060417	-8,93E-02	-2,96327978	3,63E-03	9,52E-01	1,00E+00	Gm8603	0,94	No
ENSMUSG00000060419	-7,60E-02	9,14520855	1,79E+00	1,81E-01	5,42E-01	Rps16-ps2	0,95	No
ENSMUSG00000060427	-1,18E-01	3,711658889	1,56E+00	2,12E-01	5,50E-01	Zfp868	0,92	No
ENSMUSG00000060429	-3,27E-01	0,794541792	2,18E+00	1,40E-01	4,72E-01	Sntb1	0,80	No
ENSMUSG00000060438	-1,11E-01	7,053564469	3,36E+00	6,70E-02	3,26E-01	Rps10-ps1	0,93	No
ENSMUSG00000060441	-3,73E-02	3,610353564	1,54E-01	6,95E-01	9,02E-01	Trim5	0,97	No

ENSMUSG00000060445	3,38E-01	0,882973969	2,35E+00	1,25E-01	4,43E-01	Sycp2	1,26	No
ENSMUSG00000060450	-5,03E-02	6,134471441	6,08E-01	4,35E-01	7,28E-01	Rnf14	0,97	No
ENSMUSG00000060459	-5,25E-02	-3,033661434	4,08E-11	1,00E+00	1,00E+00	Kng2	0,96	No
ENSMUSG00000060467	-1,27E-01	6,612930053	4,15E+00	4,17E-02	2,43E-01	Gm10080	0,92	No
ENSMUSG00000060470	1,94E-03	1,91056361	1,60E-04	9,90E-01	1,00E+00	Adgrg3	1,00	No
ENSMUSG00000060475	-8,86E-02	6,310969972	1,95E+00	1,63E-01	5,13E-01	Wtap	0,94	No
ENSMUSG00000060477	-9,13E-02	6,456259479	2,10E+00	1,47E-01	4,85E-01	Irak2	0,94	No
ENSMUSG00000060487	2,66E-01	3,64856955	7,46E+00	6,31E-03	6,66E-02	Samd5	1,20	No
ENSMUSG00000060508	-1,87E+00	-3,033619006	1,41E+00	2,36E-01	5,50E-01	Nlrp9b	0,27	No
ENSMUSG00000060509	1,14E+00	1,64399235	4,20E+01	9,24E-11	8,79E-09	Xcr1	2,21	Yes
ENSMUSG00000060510	3,30E-02	4,533863343	1,74E-01	6,77E-01	8,91E-01	Zfp266	1,02	No
ENSMUSG00000060512	4,21E-01	0,045562793	1,93E+00	1,65E-01	5,17E-01	0610040J01Rik	1,34	No
ENSMUSG00000060519	2,22E-01	6,05682116	1,18E+01	6,02E-04	1,07E-02	Tor3a	1,17	Yes
ENSMUSG00000060538	-8,58E-02	5,019536979	1,36E+00	2,43E-01	5,50E-01	Tmem219	0,94	No
ENSMUSG00000060548	1,57E+00	-2,497658921	2,69E+00	1,01E-01	3,92E-01	Tnfrsf19	2,97	No
ENSMUSG00000060550	-6,33E-01	6,039901381	9,49E+01	1,98E-22	5,88E-20	H2-Q7	0,64	Yes
ENSMUSG00000060556	1,63E+00	-2,711739573	1,82E+00	1,77E-01	5,36E-01	Olfr1418	3,09	No
ENSMUSG00000060566	-2,88E-01	-2,355549477	1,44E-01	7,04E-01	9,06E-01	Rpl21-ps3	0,82	No
ENSMUSG00000060568	2,20E-01	2,36764198	2,67E+00	1,02E-01	3,94E-01	Fam78b	1,16	No
ENSMUSG00000060572	-7,10E-02	-1,863713643	1,17E-02	9,14E-01	1,00E+00	Mfap2	0,95	No
ENSMUSG00000060579	1,55E+00	-2,355680901	2,62E+00	1,06E-01	3,98E-01	Fhit	2,93	No
ENSMUSG00000060586	-1,16E-01	11,28142136	4,32E+00	3,76E-02	2,31E-01	H2-Eb1	0,92	No
ENSMUSG00000060591	-2,43E-01	5,242982463	1,14E+01	7,27E-04	1,25E-02	Ifitm2	0,85	Yes
ENSMUSG00000060594	-3,41E-01	2,024348438	5,30E+00	2,13E-02	1,57E-01	Layn	0,79	No
ENSMUSG00000060600	-5,42E-01	4,005931723	3,88E+01	4,58E-10	3,95E-08	Eno3	0,69	Yes
ENSMUSG00000060601	-1,78E-02	6,674555148	8,26E-02	7,74E-01	9,44E-01	Nr1h2	0,99	No
ENSMUSG00000060615	-7,38E-02	-3,033634644	2,66E-03	9,59E-01	1,00E+00	Ang4	0,95	No
ENSMUSG00000060621	1,23E+00	-1,963281506	3,19E+00	7,41E-02	3,46E-01	Nkpd1	2,35	No
ENSMUSG00000060636	-1,97E-01	4,913570654	6,95E+00	8,38E-03	8,21E-02	Rpl35a	0,87	No
ENSMUSG00000060639	-1,75E-01	2,150960927	1,38E+00	2,40E-01	5,50E-01	Hist1h4i	0,89	No
ENSMUSG00000060647	-1,19E-01	4,619626181	2,32E+00	1,27E-01	4,48E-01	Gm7099	0,92	No
ENSMUSG00000060657	-7,57E-02	6,482713655	1,47E+00	2,25E-01	5,50E-01	Marf1	0,95	No

ENSMUSG00000060663	-8,44E-01	-2,770627609	3,91E-01	5,32E-01	8,06E-01	Olfr175-ps1	0,56	No
ENSMUSG00000060671	1,12E-01	5,91522318	2,85E+00	9,11E-02	3,82E-01	Atp8b2	1,08	No
ENSMUSG00000060675	-6,46E-01	3,75354083	4,71E+01	6,62E-12	7,27E-10	Pla2g16	0,64	Yes
ENSMUSG00000060678	-2,13E-01	-1,831969383	1,11E-01	7,39E-01	9,26E-01	Hist1h4c	0,86	No
ENSMUSG00000060679	3,07E-02	4,593511629	1,49E-01	7,00E-01	9,04E-01	Mrps9	1,02	No
ENSMUSG00000060680	-1,89E-01	7,085771049	9,84E+00	1,71E-03	2,46E-02	Gm8894	0,88	Yes
ENSMUSG00000060703	-1,04E-02	5,839674684	2,46E-02	8,75E-01	1,00E+00	Cd302	0,99	No
ENSMUSG00000060708	1,51E-02	4,206753686	3,15E-02	8,59E-01	9,95E-01	Bloc1s4	1,01	No
ENSMUSG00000060715	-1,88E+00	-2,896101069	1,42E+00	2,33E-01	5,50E-01	1700019A02Rik	0,27	No
ENSMUSG00000060716	1,44E-01	1,238553621	5,78E-01	4,47E-01	7,37E-01	Plekhh1	1,10	No
ENSMUSG00000060727	-4,06E-02	2,945086808	1,28E-01	7,21E-01	9,16E-01	Gm12571	0,97	No
ENSMUSG00000060730	-2,92E-01	1,045129324	2,15E+00	1,42E-01	4,77E-01	Gm10086	0,82	No
ENSMUSG00000060733	1,26E-01	6,161299891	3,82E+00	5,05E-02	2,72E-01	Ipmk	1,09	No
ENSMUSG00000060739	-5,37E-02	5,231279964	5,64E-01	4,53E-01	7,42E-01	Nsa2	0,96	No
ENSMUSG00000060743	9,17E-02	6,615637327	2,18E+00	1,40E-01	4,73E-01	H3f3a	1,07	No
ENSMUSG00000060771	2,44E-01	2,928977051	4,51E+00	3,37E-02	2,16E-01	Tsga10	1,18	No
ENSMUSG00000060777	1,28E-01	0,237057732	2,02E-01	6,53E-01	8,78E-01	Gm10087	1,09	No
ENSMUSG00000060791	2,18E-02	3,240204007	4,18E-02	8,38E-01	9,82E-01	Gmfg	1,02	No
ENSMUSG00000060794	1,81E+00	-3,10765737	1,28E+00	2,58E-01	5,50E-01	Tssk5	3,49	No
ENSMUSG00000060795	2,75E-01	2,16736685	3,69E+00	5,47E-02	2,87E-01	Gm13363	1,21	No
ENSMUSG00000060798	-7,86E-02	1,758581118	2,34E-01	6,29E-01	8,62E-01	Intu	0,95	No
ENSMUSG00000060802	-1,38E-01	10,78981826	6,06E+00	1,38E-02	1,19E-01	B2m	0,91	No
ENSMUSG00000060803	-7,54E-03	2,52247852	3,36E-03	9,54E-01	1,00E+00	Gstp1	0,99	No
ENSMUSG00000060860	1,64E-01	5,130269031	5,10E+00	2,40E-02	1,70E-01	Ube2s	1,12	No
ENSMUSG00000060862	1,30E-01	4,48484542	2,62E+00	1,05E-01	3,97E-01	Zbtb40	1,09	No
ENSMUSG00000060863	9,01E-03	-3,107646452	-5,16E-10	1,00E+00	1,00E+00	Tcl1b2	1,01	No
ENSMUSG00000060878	4,81E-01	-2,311213769	4,47E-01	5,04E-01	7,88E-01	Olfr1420	1,40	No
ENSMUSG00000060879	1,81E+00	-3,10765737	1,28E+00	2,58E-01	5,50E-01	Gm6564	3,49	No
ENSMUSG00000060904	1,57E-02	6,344173425	6,09E-02	8,05E-01	9,63E-01	Arl1	1,01	No
ENSMUSG00000060923	-3,50E-02	-0,522615459	9,57E-03	9,22E-01	1,00E+00	Acyp2	0,98	No
ENSMUSG00000060924	-1,22E+00	-2,49754544	1,43E+00	2,31E-01	5,50E-01	Csmd1	0,43	No
ENSMUSG00000060935	-5,92E-03	2,905616966	2,73E-03	9,58E-01	1,00E+00	Tmem263	1,00	No

ENSMUSG00000060938	-2,65E-01	5,834035174	1,60E+01	6,32E-05	1,66E-03	Rpl26	0,83	Yes
ENSMUSG00000060950	1,59E-01	2,944447018	1,98E+00	1,60E-01	5,07E-01	Trmt61a	1,12	No
ENSMUSG00000060961	-7,27E-01	1,285085041	1,61E+01	5,87E-05	1,56E-03	Slc4a4	0,60	Yes
ENSMUSG00000060962	7,35E-01	-2,896163881	2,94E-01	5,87E-01	8,40E-01	Dmkn	1,66	No
ENSMUSG00000060969	-5,25E-02	-3,033661434	4,08E-11	1,00E+00	1,00E+00	Irx1	0,96	No
ENSMUSG00000060981	-1,81E-01	-1,18560079	1,18E-01	7,31E-01	9,22E-01	Hist1h4h	0,88	No
ENSMUSG00000060985	-3,45E-01	-0,937763082	7,34E-01	3,92E-01	6,92E-01	Tdrd5	0,79	No
ENSMUSG00000060989	5,79E-02	6,51348571	8,52E-01	3,56E-01	6,57E-01	Gm11847	1,04	No
ENSMUSG00000060992	-4,08E-02	7,117615278	4,58E-01	4,99E-01	7,84E-01	Copz1	0,97	No
ENSMUSG00000061013	-5,68E-01	-1,770693239	7,05E-01	4,01E-01	6,99E-01	Mkx	0,67	No
ENSMUSG00000061024	1,12E-01	5,230276951	2,41E+00	1,20E-01	4,32E-01	Rrs1	1,08	No
ENSMUSG00000061028	1,45E-01	4,480190465	3,26E+00	7,11E-02	3,38E-01	Clasrp	1,11	No
ENSMUSG00000061032	4,95E-03	6,650488602	6,37E-03	9,36E-01	1,00E+00	Rrp1	1,00	No
ENSMUSG00000061039	-7,36E-01	2,39051218	2,95E+01	5,49E-08	3,23E-06	Olfr920	0,60	Yes
ENSMUSG00000061046	-1,05E-01	3,790640242	1,32E+00	2,51E-01	5,50E-01	Haghl	0,93	No
ENSMUSG00000061048	5,71E-01	-2,107731269	5,52E-01	4,58E-01	7,47E-01	Cdh3	1,49	No
ENSMUSG00000061062	-1,49E-01	6,495802374	5,68E+00	1,72E-02	1,37E-01	Gm10093	0,90	No
ENSMUSG00000061079	1,66E-01	4,006123064	3,58E+00	5,86E-02	2,99E-01	Zfp143	1,12	No
ENSMUSG00000061086	-6,90E-01	-1,963284695	1,09E+00	2,97E-01	5,93E-01	Myl4	0,62	No
ENSMUSG00000061099	-4,77E-01	0,087474163	2,99E+00	8,39E-02	3,71E-01	Gapdhs	0,72	No
ENSMUSG00000061100	-1,86E+00	-0,669111663	2,07E+01	5,41E-06	1,95E-04	Retnla	0,27	Yes
ENSMUSG00000061104	-3,26E-02	5,93742532	2,44E-01	6,21E-01	8,57E-01	Sap18b	0,98	No
ENSMUSG00000061111	-2,41E-02	5,429949622	1,21E-01	7,28E-01	9,20E-01	Mcrip1	0,98	No
ENSMUSG00000061115	1,40E-01	-2,146084808	3,41E-02	8,54E-01	9,91E-01	Gm17113	1,10	No
ENSMUSG00000061118	-3,36E-02	3,773113205	1,32E-01	7,17E-01	9,13E-01	Dnajc30	0,98	No
ENSMUSG00000061119	-7,42E-02	7,843287099	1,61E+00	2,04E-01	5,50E-01	Prcp	0,95	No
ENSMUSG00000061126	-2,29E-01	-1,800893725	1,16E-01	7,33E-01	9,23E-01	Cyp4f39	0,85	No
ENSMUSG00000061130	-4,86E-02	5,645681916	5,14E-01	4,73E-01	7,62E-01	Ppm1b	0,97	No
ENSMUSG00000061132	6,36E-04	4,918589997	7,26E-05	9,93E-01	1,00E+00	Blnk	1,00	No
ENSMUSG00000061136	5,00E-02	6,766789241	6,57E-01	4,18E-01	7,12E-01	Prpf40a	1,04	No
ENSMUSG00000061143	3,93E-01	3,054853891	1,19E+01	5,74E-04	1,03E-02	Maml3	1,31	Yes
ENSMUSG00000061167	-2,26E-01	7,410498996	1,45E+01	1,43E-04	3,26E-03	Rpl15-ps3	0,85	Yes

ENSMUSG00000061171	2,62E+00	-3,033654625	2,66E+00	1,03E-01	3,95E-01	Slc38a11	6,16	No
ENSMUSG00000061175	2,32E-01	7,500369952	1,53E+01	9,21E-05	2,26E-03	Fnip2	1,17	Yes
ENSMUSG00000061186	2,00E-01	1,347951296	1,17E+00	2,79E-01	5,75E-01	Sfmbt2	1,15	No
ENSMUSG00000061207	-1,16E-01	5,103468316	2,56E+00	1,09E-01	4,05E-01	Stk19	0,92	No
ENSMUSG00000061232	-2,51E-01	10,0179342	2,00E+01	7,92E-06	2,73E-04	H2-K1	0,84	Yes
ENSMUSG00000061242	-6,37E-02	3,844929411	4,91E-01	4,83E-01	7,71E-01	Gm9104	0,96	No
ENSMUSG00000061244	1,03E-01	6,556927142	2,71E+00	1,00E-01	3,92E-01	Exoc5	1,07	No
ENSMUSG00000061272	-1,63E-01	0,755814722	5,35E-01	4,65E-01	7,53E-01	Gm14173	0,89	No
ENSMUSG00000061286	9,07E-02	4,659740629	1,35E+00	2,45E-01	5,50E-01	Exosc5	1,06	No
ENSMUSG00000061288	7,21E-02	7,362927997	1,46E+00	2,27E-01	5,50E-01	Taok3	1,05	No
ENSMUSG00000061306	1,01E-01	7,452883754	2,89E+00	8,90E-02	3,79E-01	Slc38a10	1,07	No
ENSMUSG00000061311	-3,45E-01	-2,497598046	1,50E-01	6,99E-01	9,04E-01	Rag1	0,79	No
ENSMUSG00000061313	9,80E-02	5,700962827	2,12E+00	1,46E-01	4,83E-01	Ddhd2	1,07	No
ENSMUSG00000061315	-2,84E-01	3,964991191	1,02E+01	1,41E-03	2,12E-02	Naca	0,82	Yes
ENSMUSG00000061322	-4,11E-02	-1,627679893	5,88E-03	9,39E-01	1,00E+00	Dnaic1	0,97	No
ENSMUSG00000061330	2,15E-01	-1,428864787	1,97E-01	6,57E-01	8,80E-01	Gm11361	1,16	No
ENSMUSG00000061331	6,95E-01	-2,770658518	2,64E-01	6,07E-01	8,50E-01	Gm17132	1,62	No
ENSMUSG00000061336	9,50E-01	-0,648424254	6,65E+00	9,91E-03	9,26E-02	Olfr116	1,93	No
ENSMUSG00000061353	-4,56E-01	-0,816346778	1,44E+00	2,30E-01	5,50E-01	Cxcl12	0,73	No
ENSMUSG00000061356	2,59E+00	-2,963287563	2,60E+00	1,07E-01	3,99E-01	Nuggc	6,03	No
ENSMUSG00000061360	-1,35E-01	5,141538017	3,45E+00	6,31E-02	3,13E-01	Phf5a	0,91	No
ENSMUSG00000061371	-2,33E-01	1,685754524	2,13E+00	1,45E-01	4,81E-01	Zfp873	0,85	No
ENSMUSG00000061374	4,87E-02	4,808308457	4,06E-01	5,24E-01	8,01E-01	Fiz1	1,03	No
ENSMUSG00000061390	-1,62E-01	5,605683423	5,63E+00	1,76E-02	1,39E-01	Gm7866	0,89	No
ENSMUSG00000061393	1,91E-01	1,473875284	1,17E+00	2,78E-01	5,75E-01	Acvr2b	1,14	No
ENSMUSG00000061397	-8,01E-01	-2,711640323	3,54E-01	5,52E-01	8,18E-01	Krt79	0,57	No
ENSMUSG00000061410	5,55E-01	0,809296067	5,92E+00	1,50E-02	1,26E-01	Zcchc14	1,47	No
ENSMUSG00000061411	-8,95E-02	5,493585925	1,66E+00	1,97E-01	5,50E-01	Nol4l	0,94	No
ENSMUSG00000061414	-1,30E-02	3,74482191	1,90E-02	8,90E-01	1,00E+00	Cracr2a	0,99	No
ENSMUSG00000061436	1,53E-02	7,06730803	6,36E-02	8,01E-01	9,61E-01	Hipk2	1,01	No
ENSMUSG00000061451	6,45E-02	3,866512123	4,95E-01	4,82E-01	7,69E-01	Tmem151a	1,05	No
ENSMUSG00000061455	-1,27E-02	4,969999523	2,94E-02	8,64E-01	9,97E-01	Stx17	0,99	No

ENSMUSG00000061458	1,19E-01	3,58007981	1,50E+00	2,21E-01	5,50E-01	Nol10	1,09	No
ENSMUSG00000061461	-1,54E-01	4,676977142	3,88E+00	4,88E-02	2,66E-01	Smim20	0,90	No
ENSMUSG00000061462	-4,59E-01	-1,497515715	7,82E-01	3,76E-01	6,77E-01	Obscn	0,73	No
ENSMUSG00000061474	-3,00E-01	2,420291712	4,97E+00	2,58E-02	1,79E-01	Mrps36	0,81	No
ENSMUSG00000061477	-2,29E-01	4,316400433	7,53E+00	6,08E-03	6,48E-02	Rps7	0,85	No
ENSMUSG00000061479	8,71E-02	4,89039588	1,33E+00	2,50E-01	5,50E-01	Snrpa	1,06	No
ENSMUSG00000061482	-2,44E-01	-0,963224995	3,41E-01	5,59E-01	8,22E-01	Hist1h4d	0,84	No
ENSMUSG00000061486	-1,08E-01	1,581397803	4,12E-01	5,21E-01	8,00E-01	Gm5161	0,93	No
ENSMUSG00000061488	-2,76E-01	-0,117170624	7,58E-01	3,84E-01	6,85E-01	Rpl27a-ps1	0,83	No
ENSMUSG00000061490	1,36E+00	-2,226331283	1,90E+00	1,68E-01	5,23E-01	Ube2d4	2,57	No
ENSMUSG00000061510	-9,57E-01	-1,098049784	4,04E+00	4,43E-02	2,51E-01	Gm10101	0,52	No
ENSMUSG00000061518	-2,70E-02	3,739450005	8,24E-02	7,74E-01	9,45E-01	Cox5b	0,98	No
ENSMUSG00000061524	2,99E-01	-2,600699326	9,12E-02	7,63E-01	9,38E-01	Zic2	1,23	No
ENSMUSG00000061525	-1,14E-02	-3,033635021	1,50E-05	9,97E-01	1,00E+00	4921509C19Rik	0,99	No
ENSMUSG00000061527	-1,94E+00	-3,107619243	1,50E+00	2,21E-01	5,50E-01	Krt5	0,26	No
ENSMUSG00000061531	4,62E-01	-1,355514107	7,11E-01	3,99E-01	6,98E-01	Tmem236	1,38	No
ENSMUSG00000061533	1,56E-01	4,458016781	3,69E+00	5,48E-02	2,87E-01	Cep128	1,11	No
ENSMUSG00000061536	2,46E-01	4,7603571	1,03E+01	1,37E-03	2,07E-02	Sec22c	1,19	Yes
ENSMUSG00000061540	-1,28E-01	1,680621096	6,35E-01	4,26E-01	7,20E-01	Orm2	0,91	No
ENSMUSG00000061544	-1,32E-01	1,541857377	6,33E-01	4,26E-01	7,21E-01	Zfp229	0,91	No
ENSMUSG00000061559	-1,12E-01	4,489643199	1,93E+00	1,65E-01	5,17E-01	Wdr61	0,93	No
ENSMUSG00000061561	1,26E+00	-2,770612189	9,82E-01	3,22E-01	6,22E-01	Olfr373	2,39	No
ENSMUSG00000061576	-3,57E-01	-1,548165455	4,59E-01	4,98E-01	7,83E-01	Dpp6	0,78	No
ENSMUSG00000061577	1,88E-01	3,527692644	3,64E+00	5,63E-02	2,91E-01	Adgrg5	1,14	No
ENSMUSG00000061578	4,29E-01	4,044109071	2,28E+01	1,84E-06	7,56E-05	Ksr2	1,35	Yes
ENSMUSG00000061589	9,03E-02	6,249048798	1,98E+00	1,59E-01	5,06E-01	Dot1l	1,06	No
ENSMUSG00000061603	4,70E-01	0,997535234	4,96E+00	2,59E-02	1,79E-01	Akap6	1,38	No
ENSMUSG00000061607	1,84E-01	5,871238189	7,74E+00	5,41E-03	5,93E-02	Mdc1	1,14	No
ENSMUSG00000061613	6,69E-02	3,524558191	4,66E-01	4,95E-01	7,80E-01	U2af1	1,05	No
ENSMUSG00000061615	1,63E+00	-1,816504062	5,82E+00	1,59E-02	1,31E-01	Hist1h2ab	3,10	No
ENSMUSG00000061616	1,86E+00	-2,963277112	1,35E+00	2,46E-01	5,50E-01	Olfr12	3,62	No
ENSMUSG00000061619	2,40E-01	1,644323391	2,01E+00	1,56E-01	5,00E-01	Vma21-ps	1,18	No

ENSMUSG00000061650	1,15E-01	4,661950531	2,18E+00	1,40E-01	4,72E-01	Med9	1,08	No
ENSMUSG00000061665	-6,30E-03	6,021975822	9,34E-03	9,23E-01	1,00E+00	Cd2ap	1,00	No
ENSMUSG00000061666	-9,31E-02	3,300621401	7,82E-01	3,77E-01	6,78E-01	Gdpd1	0,94	No
ENSMUSG00000061669	-1,22E-01	2,842564104	1,10E+00	2,93E-01	5,89E-01	Rpl34-ps2	0,92	No
ENSMUSG00000061684	-9,66E-02	4,165995932	1,27E+00	2,60E-01	5,53E-01	Rpl21-ps8	0,94	No
ENSMUSG00000061689	2,92E-03	6,150435897	2,06E-03	9,64E-01	1,00E+00	Dlgap4	1,00	No
ENSMUSG00000061701	-3,83E-02	-3,107651936	-7,91E-11	1,00E+00	1,00E+00	Fbxw20	0,97	No
ENSMUSG00000061702	-2,38E-01	-0,378293422	4,47E-01	5,04E-01	7,88E-01	Tmem91	0,85	No
ENSMUSG00000061718	1,86E+00	-2,963277112	1,35E+00	2,46E-01	5,50E-01	Ppp1r1b	3,62	No
ENSMUSG00000061723	2,11E-01	2,75327032	2,96E+00	8,51E-02	3,75E-01	Tnnt3	1,16	No
ENSMUSG00000061724	-9,26E-04	5,691480866	1,88E-04	9,89E-01	1,00E+00	Gm2423	1,00	No
ENSMUSG00000061728	-1,19E+00	-2,548203955	1,83E+00	1,77E-01	5,36E-01	Btnl7-ps	0,44	No
ENSMUSG00000061731	-2,86E-01	5,557073725	1,75E+01	2,83E-05	8,36E-04	Ext1	0,82	Yes
ENSMUSG00000061740	-1,89E+00	-3,1076441	1,42E+00	2,33E-01	5,50E-01	Cyp2d22	0,27	No
ENSMUSG00000061751	3,58E-01	1,480165235	4,28E+00	3,85E-02	2,33E-01	Kalrn	1,28	No
ENSMUSG00000061755	1,11E-01	6,103079549	2,97E+00	8,47E-02	3,73E-01	Bod1l	1,08	No
ENSMUSG00000061758	-1,97E-02	4,539788979	6,04E-02	8,06E-01	9,63E-01	Akr1b10	0,99	No
ENSMUSG00000061759	9,20E-02	4,90486506	1,51E+00	2,19E-01	5,50E-01	Armt1	1,07	No
ENSMUSG00000061787	-1,51E-01	5,522779663	4,83E+00	2,79E-02	1,89E-01	Rps17	0,90	No
ENSMUSG00000061802	-2,80E-01	-1,740882057	2,55E-01	6,13E-01	8,53E-01	Armc4	0,82	No
ENSMUSG00000061808	3,14E-02	-3,107617333	-7,69E-10	1,00E+00	1,00E+00	Ttr	1,02	No
ENSMUSG00000061809	6,65E-01	-2,896156246	2,39E-01	6,25E-01	8,59E-01	Tbpl2	1,59	No
ENSMUSG00000061815	9,84E-04	-0,300098264	8,93E-06	9,98E-01	1,00E+00	Rufy4	1,00	No
ENSMUSG00000061825	9,93E-01	-1,847796402	1,92E+00	1,65E-01	5,18E-01	Ces2c	1,99	No
ENSMUSG00000061833	-3,88E-02	5,942938728	3,47E-01	5,56E-01	8,21E-01	Gm6311	0,97	No
ENSMUSG00000061838	-1,69E-02	2,742273961	1,93E-02	8,89E-01	1,00E+00	Suclg2	0,99	No
ENSMUSG00000061848	-2,12E-01	7,568320704	1,29E+01	3,31E-04	6,56E-03	Gm5805	0,86	Yes
ENSMUSG00000061852	8,06E-02	0,863674047	1,42E-01	7,06E-01	9,08E-01	Gm7582	1,06	No
ENSMUSG00000061859	-1,64E-03	0,214047742	4,10E-05	9,95E-01	1,00E+00	Patj	1,00	No
ENSMUSG00000061863	-4,27E-01	-2,497604114	1,87E-01	6,66E-01	8,85E-01	Gm6822	0,74	No
ENSMUSG00000061878	2,18E-01	3,842498832	5,65E+00	1,74E-02	1,38E-01	Sphk1	1,16	No
ENSMUSG00000061882	1,72E-01	0,768408908	5,87E-01	4,44E-01	7,35E-01	Ccdc62	1,13	No

ENSMUSG00000061887	-1,51E-01	3,991483463	2,91E+00	8,80E-02	3,79E-01	Ssbp3	0,90	No
ENSMUSG00000061894	-2,13E-01	2,569056738	3,01E+00	8,30E-02	3,70E-01	Zscan20	0,86	No
ENSMUSG00000061897	-1,63E-01	5,052542642	4,97E+00	2,59E-02	1,79E-01	Gm14292	0,89	No
ENSMUSG00000061898	7,89E-02	3,919050276	7,65E-01	3,82E-01	6,83E-01	Rbak	1,06	No
ENSMUSG00000061904	-2,45E-04	8,540139476	1,83E-05	9,97E-01	1,00E+00	Slc25a3	1,00	No
ENSMUSG00000061911	-2,66E+00	-3,033639428	2,81E+00	9,35E-02	3,83E-01	Myt1l	0,16	No
ENSMUSG00000061928	-1,89E+00	-3,1076441	1,42E+00	2,33E-01	5,50E-01	Dsg1b	0,27	No
ENSMUSG00000061950	8,85E-02	5,996658462	1,83E+00	1,76E-01	5,35E-01	Ppp4r1	1,06	No
ENSMUSG00000061969	-1,37E+00	-2,89609265	1,18E+00	2,78E-01	5,74E-01	Gm5581	0,39	No
ENSMUSG00000061972	-1,62E-01	0,372240442	4,22E-01	5,16E-01	7,97E-01	Olfr99	0,89	No
ENSMUSG00000061979	-5,95E-02	3,458716948	3,48E-01	5,55E-01	8,20E-01	Rcc1l	0,96	No
ENSMUSG00000061981	3,41E-03	6,827197614	3,09E-03	9,56E-01	1,00E+00	Flot2	1,00	No
ENSMUSG00000061983	-1,37E-01	4,145412185	2,51E+00	1,13E-01	4,14E-01	Rps12	0,91	No
ENSMUSG00000061988	1,89E-01	-1,800986829	9,98E-02	7,52E-01	9,32E-01	Rpl10a-ps2	1,14	No
ENSMUSG00000061991	-3,33E-01	-2,600661269	8,11E-02	7,76E-01	9,45E-01	Hist1h2af	0,79	No
ENSMUSG00000061992	-8,57E-02	-2,070200636	1,36E-02	9,07E-01	1,00E+00	Gm4953	0,94	No
ENSMUSG00000062006	-3,12E-01	3,995433346	1,25E+01	4,02E-04	7,65E-03	Rpl34	0,81	Yes
ENSMUSG00000062007	-2,56E-01	1,536178583	2,46E+00	1,17E-01	4,23E-01	Hsh2d	0,84	No
ENSMUSG00000062012	4,93E-01	-0,896367153	1,44E+00	2,29E-01	5,50E-01	Zfp13	1,41	No
ENSMUSG00000062014	1,46E-01	7,087203236	5,86E+00	1,55E-02	1,29E-01	Gmfb	1,11	No
ENSMUSG00000062028	5,00E-01	4,029567071	3,12E+01	2,35E-08	1,51E-06	Irgc1	1,41	Yes
ENSMUSG00000062031	1,99E-01	3,648277175	4,36E+00	3,68E-02	2,28E-01	Pgghg	1,15	No
ENSMUSG00000062038	3,78E-02	4,683122825	2,34E-01	6,29E-01	8,62E-01	Gm10108	1,03	No
ENSMUSG00000062040	4,52E-01	2,603952723	1,31E+01	2,94E-04	5,97E-03	Zfp27	1,37	Yes
ENSMUSG00000062044	-2,15E-01	-2,311134572	5,57E-02	8,13E-01	9,68E-01	Lmtk3	0,86	No
ENSMUSG00000062054	1,20E-01	5,231437385	2,80E+00	9,41E-02	3,84E-01	Iah1	1,09	No
ENSMUSG00000062061	-2,71E+00	-3,033620915	2,92E+00	8,75E-02	3,78E-01	Obp2a	0,15	No
ENSMUSG00000062064	1,21E-02	-3,033650469	7,34E-11	1,00E+00	1,00E+00	Slc2a7	1,01	No
ENSMUSG00000062073	1,17E+00	-0,355735301	1,00E+01	1,53E-03	2,27E-02	Gm10109	2,24	Yes
ENSMUSG00000062074	-1,69E-01	-1,60057942	8,97E-02	7,64E-01	9,39E-01	Wisp3	0,89	No
ENSMUSG00000062075	2,26E-01	5,29268384	1,01E+01	1,51E-03	2,24E-02	Lmnb2	1,17	Yes
ENSMUSG00000062077	2,01E+00	-2,71176413	3,10E+00	7,81E-02	3,57E-01	Trim54	4,02	No

ENSMUSG00000062078	1,77E-01	7,623451373	9,04E+00	2,64E-03	3,47E-02	Qk	1,13	Yes
ENSMUSG00000062081	-4,60E-02	6,041897528	4,95E-01	4,82E-01	7,69E-01	Gm6055	0,97	No
ENSMUSG00000062082	3,29E-01	7,710390958	3,10E+01	2,54E-08	1,62E-06	Cd200r4	1,26	Yes
ENSMUSG00000062083	-2,09E-01	6,361913989	1,08E+01	1,01E-03	1,63E-02	Rpl13a-ps1	0,87	Yes
ENSMUSG00000062093	-5,22E-02	5,848518505	6,16E-01	4,33E-01	7,26E-01	Gm10110	0,96	No
ENSMUSG00000062098	1,74E-01	3,18918159	2,65E+00	1,04E-01	3,95E-01	Btbd3	1,13	No
ENSMUSG00000062101	-4,27E-03	1,480198964	5,92E-04	9,81E-01	1,00E+00	Zfp119b	1,00	No
ENSMUSG00000062110	4,88E-02	4,006358151	3,02E-01	5,83E-01	8,38E-01	Scfd2	1,03	No
ENSMUSG00000062115	1,33E-01	6,459648149	4,50E+00	3,38E-02	2,16E-01	Rai1	1,10	No
ENSMUSG00000062116	-5,96E-02	3,687241858	3,97E-01	5,29E-01	8,04E-01	Zfp954	0,96	No
ENSMUSG00000062127	7,70E-02	6,016274009	1,39E+00	2,39E-01	5,50E-01	Cttnbp2nl	1,05	No
ENSMUSG00000062132	1,17E+00	-3,14612376	6,40E-01	4,24E-01	7,18E-01	Arhgap33os	2,26	No
ENSMUSG00000062148	2,84E-01	3,959000715	9,78E+00	1,76E-03	2,52E-02	Ear6	1,22	Yes
ENSMUSG00000062151	1,81E+00	-3,10765737	1,28E+00	2,58E-01	5,50E-01	Unc13c	3,49	No
ENSMUSG00000062152	-1,41E+00	-1,497409473	5,68E+00	1,71E-02	1,37E-01	Rpl21-ps14	0,38	No
ENSMUSG00000062157	-6,94E-01	2,333026741	2,82E+01	1,10E-07	6,09E-06	Ifnlr1	0,62	Yes
ENSMUSG00000062169	-5,67E-03	4,51000109	4,98E-03	9,44E-01	1,00E+00	Cnih4	1,00	No
ENSMUSG00000062175	-4,69E-01	1,266118257	6,48E+00	1,09E-02	9,96E-02	Tgif2	0,72	No
ENSMUSG00000062190	5,94E-02	3,892163032	4,33E-01	5,11E-01	7,94E-01	Lancl2	1,04	No
ENSMUSG00000062198	8,68E-02	2,303889089	4,22E-01	5,16E-01	7,97E-01	2700097O09Rik	1,06	No
ENSMUSG00000062202	-7,13E-02	5,458392745	1,05E+00	3,05E-01	6,02E-01	Btbd9	0,95	No
ENSMUSG00000062203	3,12E-02	6,933869348	2,61E-01	6,09E-01	8,51E-01	Gspt1	1,02	No
ENSMUSG00000062204	-4,28E-01	-1,401295251	5,31E-01	4,66E-01	7,54E-01	Olfr1373	0,74	No
ENSMUSG00000062210	-1,71E-01	6,678306444	7,69E+00	5,55E-03	6,04E-02	Tnfaip8	0,89	No
ENSMUSG00000062232	1,38E-02	5,249388279	3,68E-02	8,48E-01	9,88E-01	Rapgef2	1,01	No
ENSMUSG00000062234	6,89E-02	7,506635886	1,35E+00	2,45E-01	5,50E-01	Gak	1,05	No
ENSMUSG00000062235	-5,78E-02	-2,896167382	2,23E-03	9,62E-01	1,00E+00	Gm5341	0,96	No
ENSMUSG00000062248	1,58E-01	3,447687375	2,47E+00	1,16E-01	4,22E-01	Cks2	1,12	No
ENSMUSG00000062257	-1,90E+00	-3,033647263	1,44E+00	2,30E-01	5,50E-01	Opcml	0,27	No
ENSMUSG00000062270	-9,27E-03	5,653714188	1,87E-02	8,91E-01	1,00E+00	Morf4l1	0,99	No
ENSMUSG00000062279	-7,60E-01	-0,528828629	4,16E+00	4,13E-02	2,41E-01	Gm10237	0,59	No
ENSMUSG00000062284	-2,66E-01	2,972289971	5,41E+00	2,00E-02	1,51E-01	Gm6030	0,83	No

ENSMUSG00000062296	-1,49E+00	-2,497512835	2,30E+00	1,30E-01	4,52E-01	Trank1	0,36	No
ENSMUSG00000062300	-1,76E-01	5,101670306	5,96E+00	1,46E-02	1,24E-01	Nectin2	0,89	No
ENSMUSG00000062309	6,35E-01	1,090954446	9,36E+00	2,22E-03	3,03E-02	Rpp25	1,55	Yes
ENSMUSG00000062310	-3,40E-01	0,783949013	2,54E+00	1,11E-01	4,09E-01	Glrp1	0,79	No
ENSMUSG00000062312	-1,02E+00	-1,785671985	2,12E+00	1,45E-01	4,83E-01	Erbb2	0,49	No
ENSMUSG00000062327	9,01E-03	-3,107646452	-5,16E-10	1,00E+00	1,00E+00	T	1,01	No
ENSMUSG00000062328	-1,80E-01	4,225504527	4,53E+00	3,32E-02	2,14E-01	Rpl17	0,88	No
ENSMUSG00000062345	-1,45E+00	4,26136652	2,70E+02	1,20E-60	6,00E-57	Serpinb2	0,36	Yes
ENSMUSG00000062352	1,20E-01	5,064604754	2,69E+00	1,01E-01	3,93E-01	Itgb1bp1	1,09	No
ENSMUSG00000062353	-1,70E-01	8,507981838	8,76E+00	3,07E-03	3,90E-02	Gm15772	0,89	Yes
ENSMUSG00000062365	-1,69E+00	-2,401298528	3,15E+00	7,61E-02	3,52E-01	Gm4968	0,31	No
ENSMUSG00000062372	-6,31E-01	-1,740837581	1,00E+00	3,16E-01	6,17E-01	Otof	0,65	No
ENSMUSG00000062373	2,59E-01	6,913939989	1,80E+01	2,24E-05	6,88E-04	Tmem65	1,20	Yes
ENSMUSG00000062376	7,00E-02	3,122269287	4,14E-01	5,20E-01	7,99E-01	Borcs7	1,05	No
ENSMUSG00000062380	7,05E-01	-0,785894315	3,30E+00	6,94E-02	3,32E-01	Tubb3	1,63	No
ENSMUSG00000062382	5,03E-02	11,16989362	8,11E-01	3,68E-01	6,69E-01	Ftl1-ps1	1,04	No
ENSMUSG00000062397	4,39E-02	6,52299941	4,92E-01	4,83E-01	7,70E-01	Zfp706	1,03	No
ENSMUSG00000062410	-3,83E-02	-3,107651936	-7,91E-11	1,00E+00	1,00E+00	Hsd3b3	0,97	No
ENSMUSG00000062421	7,75E-02	5,463332027	1,25E+00	2,64E-01	5,59E-01	Arf2	1,06	No
ENSMUSG00000062432	-5,56E-01	-2,71172308	2,42E-01	6,23E-01	8,58E-01	Cyp26c1	0,68	No
ENSMUSG00000062438	4,09E-01	-0,954629677	9,13E-01	3,39E-01	6,40E-01	Adam1b	1,33	No
ENSMUSG00000062456	-2,21E-01	7,803019819	1,43E+01	1,59E-04	3,57E-03	Rpl9-ps6	0,86	Yes
ENSMUSG00000062458	-8,41E-02	4,789806227	1,21E+00	2,71E-01	5,67E-01	Gm8623	0,94	No
ENSMUSG00000062461	-2,01E-01	5,74770076	9,02E+00	2,67E-03	3,50E-02	Gm5453	0,87	Yes
ENSMUSG00000062464	2,27E-02	2,111239891	2,42E-02	8,76E-01	1,00E+00	Cyp4f37	1,02	No
ENSMUSG00000062470	-7,80E-02	-0,300307193	5,81E-02	8,10E-01	9,65E-01	Fbxl12os	0,95	No
ENSMUSG00000062472	4,78E-01	-2,401424486	3,10E-01	5,78E-01	8,35E-01	Gm4945	1,39	No
ENSMUSG00000062477	-3,83E-02	-3,107651936	-7,91E-11	1,00E+00	1,00E+00	Hmgb1l	0,97	No
ENSMUSG00000062478	6,19E-02	0,547246476	6,58E-02	7,98E-01	9,58E-01	Ctrc	1,04	No
ENSMUSG00000062480	-1,34E-01	5,237882246	3,58E+00	5,84E-02	2,98E-01	Acat3	0,91	No
ENSMUSG00000062488	-3,36E-01	-0,010971018	1,33E+00	2,49E-01	5,50E-01	Ifit3b	0,79	No
ENSMUSG00000062510	2,06E-01	3,911656381	5,33E+00	2,09E-02	1,56E-01	Nsl1	1,15	No

ENSMUSG00000062515	-5,71E-02	4,285858327	4,38E-01	5,08E-01	7,92E-01	Fabp4	0,96	No
ENSMUSG00000062518	-3,56E+00	-2,600652749	5,71E+00	1,69E-02	1,36E-01	Zfp534	0,08	No
ENSMUSG00000062519	-3,16E-02	5,277101695	2,00E-01	6,55E-01	8,79E-01	Zfp398	0,98	No
ENSMUSG00000062526	6,99E-02	3,896657496	5,95E-01	4,40E-01	7,32E-01	Mppe1	1,05	No
ENSMUSG00000062542	1,25E-01	2,826156754	1,11E+00	2,93E-01	5,88E-01	Syt9	1,09	No
ENSMUSG00000062543	-2,66E+00	-3,033639428	2,81E+00	9,35E-02	3,83E-01	Gsdmcl1	0,16	No
ENSMUSG00000062545	2,19E-01	2,517420203	2,81E+00	9,39E-02	3,84E-01	Tlr12	1,16	No
ENSMUSG00000062554	-5,46E-02	4,525394891	4,59E-01	4,98E-01	7,83E-01	Gm12751	0,96	No
ENSMUSG00000062563	9,01E-03	-3,107646452	-5,16E-10	1,00E+00	1,00E+00	Cys1	1,01	No
ENSMUSG00000062580	-4,98E-02	4,255101401	3,47E-01	5,56E-01	8,21E-01	Timm17a	0,97	No
ENSMUSG00000062582	-1,89E-01	4,193501788	5,04E+00	2,48E-02	1,74E-01	Rpl30-ps8	0,88	No
ENSMUSG00000062585	3,90E-01	5,207352965	2,98E+01	4,80E-08	2,88E-06	Cnr2	1,31	Yes
ENSMUSG00000062588	1,64E-01	2,867122098	1,97E+00	1,61E-01	5,09E-01	Gm6104	1,12	No
ENSMUSG00000062590	3,67E-01	2,918225704	9,96E+00	1,60E-03	2,34E-02	Armc9	1,29	Yes
ENSMUSG00000062591	-8,93E-02	1,030250232	1,91E-01	6,62E-01	8,83E-01	Tubb4a	0,94	No
ENSMUSG00000062593	1,67E-01	9,812412257	8,80E+00	3,01E-03	3,84E-02	Lilrb4a	1,12	Yes
ENSMUSG00000062604	7,01E-02	5,561648611	1,04E+00	3,07E-01	6,05E-01	Srpk2	1,05	No
ENSMUSG00000062611	-9,02E-02	8,672600892	2,48E+00	1,15E-01	4,20E-01	Rps3a2	0,94	No
ENSMUSG00000062619	1,95E-02	2,563572223	2,27E-02	8,80E-01	1,00E+00	2310039H08Rik	1,01	No
ENSMUSG00000062627	-2,16E-02	5,695382229	1,02E-01	7,49E-01	9,31E-01	Mysm1	0,99	No
ENSMUSG00000062629	3,09E-01	0,183735617	1,12E+00	2,89E-01	5,85E-01	Olfr114	1,24	No
ENSMUSG00000062638	2,58E-01	-0,247177334	6,05E-01	4,37E-01	7,29E-01	Btnl1	1,20	No
ENSMUSG00000062646	-8,81E-02	4,511498435	1,22E+00	2,69E-01	5,65E-01	Ganc	0,94	No
ENSMUSG00000062647	-1,45E-01	5,146564354	4,05E+00	4,42E-02	2,50E-01	Rpl7a	0,90	No
ENSMUSG00000062651	-1,94E+00	-3,107619243	1,50E+00	2,21E-01	5,50E-01	H2al2a	0,26	No
ENSMUSG00000062661	-4,65E-02	3,584206554	2,26E-01	6,34E-01	8,66E-01	Ncs1	0,97	No
ENSMUSG00000062683	-6,18E-02	4,405879197	5,79E-01	4,47E-01	7,37E-01	Atp5g2	0,96	No
ENSMUSG00000062687	6,06E-01	-2,311154102	4,10E-01	5,22E-01	8,00E-01	Cks1brt	1,52	No
ENSMUSG00000062691	-1,04E-01	2,716141061	7,20E-01	3,96E-01	6,96E-01	Cebpzos	0,93	No
ENSMUSG00000062694	-8,45E-01	-2,963241229	3,92E-01	5,31E-01	8,05E-01	Cav3	0,56	No
ENSMUSG00000062704	3,35E-01	-1,929267087	2,06E-01	6,50E-01	8,76E-01	9430002A10Rik	1,26	No
ENSMUSG00000062727	-3,37E-01	-0,112305393	1,28E+00	2,57E-01	5,50E-01	Hist1h2bk	0,79	No

ENSMUSG00000062729	6,83E-02	5,131796565	8,98E-01	3,43E-01	6,44E-01	Ppox	1,05	No
ENSMUSG00000062732	-1,89E+00	-3,1076441	1,42E+00	2,33E-01	5,50E-01	Lypd4	0,27	No
ENSMUSG00000062742	-1,35E+00	-2,548168267	1,14E+00	2,86E-01	5,82E-01	Gm5239	0,39	No
ENSMUSG00000062743	-1,11E-01	2,789210109	8,83E-01	3,47E-01	6,48E-01	Zfp677	0,93	No
ENSMUSG00000062753	4,27E-02	4,881091742	3,21E-01	5,71E-01	8,31E-01	AI413582	1,03	No
ENSMUSG00000062758	-1,88E-01	5,682284826	7,71E+00	5,48E-03	5,98E-02	Gm16477	0,88	No
ENSMUSG00000062760	-1,73E+00	-2,770551333	2,08E+00	1,49E-01	4,90E-01	1810041L15Rik	0,30	No
ENSMUSG00000062761	1,79E-01	4,468330773	4,94E+00	2,63E-02	1,81E-01	Zfp512	1,13	No
ENSMUSG00000062762	1,50E-03	6,319982	5,54E-04	9,81E-01	1,00E+00	Ei24	1,00	No
ENSMUSG00000062773	4,69E-01	-2,448675959	2,98E-01	5,85E-01	8,39E-01	Tex101	1,38	No
ENSMUSG00000062778	1,87E+00	-3,107639691	1,38E+00	2,40E-01	5,50E-01	Chia1	3,67	No
ENSMUSG00000062785	1,10E-01	3,284169022	1,14E+00	2,87E-01	5,83E-01	Kcnc3	1,08	No
ENSMUSG00000062794	-8,76E-02	1,015005515	1,78E-01	6,73E-01	8,90E-01	Zfp599	0,94	No
ENSMUSG00000062797	1,74E-02	3,929930597	3,74E-02	8,47E-01	9,87E-01	Hikeshi	1,01	No
ENSMUSG00000062822	-1,04E-01	5,606624893	2,37E+00	1,24E-01	4,40E-01	4833420G17Rik	0,93	No
ENSMUSG00000062825	-7,58E-02	11,03281471	1,84E+00	1,75E-01	5,33E-01	Actg1	0,95	No
ENSMUSG00000062826	-8,16E-01	-2,770547056	3,67E-01	5,45E-01	8,13E-01	Ces2f	0,57	No
ENSMUSG00000062846	-9,14E-02	5,997258373	1,94E+00	1,63E-01	5,14E-01	Gm14176	0,94	No
ENSMUSG00000062859	2,79E-02	-1,289431983	2,82E-03	9,58E-01	1,00E+00	Tcp11	1,02	No
ENSMUSG00000062861	1,00E-01	3,603608549	1,07E+00	3,00E-01	5,97E-01	Zfp28	1,07	No
ENSMUSG00000062866	-1,33E-02	5,953905687	4,12E-02	8,39E-01	9,83E-01	Phactr2	0,99	No
ENSMUSG00000062867	3,15E-02	3,541387045	1,03E-01	7,48E-01	9,30E-01	Impdh2	1,02	No
ENSMUSG00000062874	-8,25E-01	-2,655061026	3,75E-01	5,40E-01	8,10E-01	Gm4978	0,56	No
ENSMUSG00000062896	-5,18E-01	4,267744894	3,78E+01	7,92E-10	6,61E-08	Rpl31-ps11	0,70	Yes
ENSMUSG00000062901	1,57E-02	5,695535764	5,48E-02	8,15E-01	9,69E-01	Klhl24	1,01	No
ENSMUSG00000062905	8,34E-01	-2,600739033	6,05E-01	4,37E-01	7,30E-01	Vmn1r32	1,78	No
ENSMUSG00000062906	5,65E-02	2,530241868	1,95E-01	6,59E-01	8,81E-01	Hdac10	1,04	No
ENSMUSG00000062908	4,60E-02	5,427007462	4,37E-01	5,09E-01	7,92E-01	Acadm	1,03	No
ENSMUSG00000062929	-8,13E-02	4,33624382	9,66E-01	3,26E-01	6,26E-01	Cfl2	0,95	No
ENSMUSG00000062931	1,61E-01	1,911257896	1,11E+00	2,92E-01	5,87E-01	Zfp938	1,12	No
ENSMUSG00000062933	-7,14E-02	9,682455771	1,60E+00	2,06E-01	5,50E-01	Gm10123	0,95	No
ENSMUSG00000062937	2,81E-01	4,610439132	1,25E+01	4,09E-04	7,76E-03	Mtap	1,21	Yes

ENSMUSG00000062939	-9,29E-01	4,196718584	1,23E+02	1,41E-28	7,05E-26	Stat4	0,53	Yes
ENSMUSG00000062944	1,64E-01	2,763169398	1,86E+00	1,73E-01	5,29E-01	9130023H24Rik	1,12	No
ENSMUSG00000062960	3,19E-01	4,91735644	1,71E+01	3,51E-05	1,01E-03	Kdr	1,25	Yes
ENSMUSG00000062961	-2,66E+00	-3,033639428	2,81E+00	9,35E-02	3,83E-01	Ccdc177	0,16	No
ENSMUSG00000062962	-8,61E-01	-2,89612532	4,07E-01	5,24E-01	8,01E-01	Gm6378	0,55	No
ENSMUSG00000062963	-5,34E-03	5,510400445	5,96E-03	9,38E-01	1,00E+00	Ufc1	1,00	No
ENSMUSG00000062980	-3,05E-01	1,060387834	2,21E+00	1,37E-01	4,68E-01	Cped1	0,81	No
ENSMUSG00000062981	-4,57E-02	4,179288795	2,87E-01	5,92E-01	8,42E-01	Mrpl42	0,97	No
ENSMUSG00000062991	-7,29E-01	5,103006	9,97E+01	1,76E-23	5,61E-21	Nrg1	0,60	Yes
ENSMUSG00000062995	2,40E-01	3,832754287	6,70E+00	9,62E-03	9,06E-02	Ica1	1,18	No
ENSMUSG00000062997	-1,26E-01	4,345129637	2,31E+00	1,29E-01	4,50E-01	Rpl35	0,92	No
ENSMUSG00000063001	-1,69E+00	-2,107431223	4,34E+00	3,71E-02	2,29E-01	Gm9701	0,31	No
ENSMUSG00000063011	1,85E+00	-3,107657832	1,35E+00	2,46E-01	5,50E-01	Msln	3,61	No
ENSMUSG00000063015	-1,32E-01	7,895144358	5,15E+00	2,33E-02	1,67E-01	Ccni	0,91	No
ENSMUSG00000063019	-1,31E-01	5,141662818	3,26E+00	7,09E-02	3,37E-01	Manbal	0,91	No
ENSMUSG00000063021	-3,11E-01	-2,750914122	7,09E-02	7,90E-01	9,54E-01	Hist1h2ak	0,81	No
ENSMUSG00000063047	2,68E-02	2,740108358	5,19E-02	8,20E-01	9,71E-01	Zfp780b	1,02	No
ENSMUSG00000063049	-3,02E-02	3,611648165	9,99E-02	7,52E-01	9,32E-01	Ing2	0,98	No
ENSMUSG00000063052	1,49E-01	4,343080229	3,22E+00	7,26E-02	3,42E-01	Lrrc40	1,11	No
ENSMUSG00000063060	-2,57E-01	-0,033675665	7,92E-01	3,73E-01	6,74E-01	Sox7	0,84	No
ENSMUSG00000063065	-4,59E-02	7,986176431	6,22E-01	4,30E-01	7,24E-01	Mapk3	0,97	No
ENSMUSG00000063077	-8,46E-02	7,135259427	1,98E+00	1,59E-01	5,07E-01	Kif1b	0,94	No
ENSMUSG00000063087	-2,99E-01	-0,59400296	7,61E-01	3,83E-01	6,85E-01	Gm10125	0,81	No
ENSMUSG00000063089	1,85E-01	0,799141524	6,50E-01	4,20E-01	7,15E-01	Klk1b8	1,14	No
ENSMUSG00000063106	1,87E+00	-3,107639691	1,38E+00	2,40E-01	5,50E-01	Olfr1510	3,67	No
ENSMUSG00000063108	-1,30E-01	5,023359063	3,14E+00	7,63E-02	3,52E-01	Zfp26	0,91	No
ENSMUSG00000063109	-2,66E+00	-2,96323193	2,82E+00	9,29E-02	3,83E-01	Dgkeos	0,16	No
ENSMUSG00000063129	6,95E-02	6,140045885	1,16E+00	2,82E-01	5,79E-01	Aldoart2	1,05	No
ENSMUSG00000063130	-1,92E+00	-3,0336536	1,46E+00	2,27E-01	5,50E-01	Calml3	0,26	No
ENSMUSG00000063133	6,54E-01	5,739269698	8,92E+01	3,63E-21	9,13E-19	Klk1b1	1,57	Yes
ENSMUSG00000063137	1,83E+00	-2,963282593	1,33E+00	2,50E-01	5,50E-01	Olfr177	3,56	No
ENSMUSG00000063142	1,83E+00	-2,89616034	1,32E+00	2,50E-01	5,50E-01	Kcnma1	3,56	No

ENSMUSG00000063145	4,11E-03	1,066361004	4,11E-04	9,84E-01	1,00E+00	Bbs5	1,00	No
ENSMUSG00000063146	-2,04E-01	5,913045276	9,62E+00	1,92E-03	2,72E-02	Clip2	0,87	Yes
ENSMUSG00000063157	1,74E+00	-2,226340772	3,47E+00	6,24E-02	3,11E-01	Csn2	3,33	No
ENSMUSG00000063160	2,16E-01	3,073163961	3,73E+00	5,35E-02	2,82E-01	Numbl	1,16	No
ENSMUSG00000063166	-7,02E-01	-0,165594607	4,97E+00	2,58E-02	1,79E-01	Gm5449	0,61	No
ENSMUSG00000063171	-1,33E-01	2,268534045	9,13E-01	3,39E-01	6,40E-01	Rps4l	0,91	No
ENSMUSG00000063172	-3,26E-01	1,442075986	3,42E+00	6,43E-02	3,18E-01	Hspb11	0,80	No
ENSMUSG00000063177	1,30E-01	2,638646294	1,03E+00	3,10E-01	6,09E-01	Klk1b27	1,09	No
ENSMUSG00000063179	3,85E-02	3,997167785	1,89E-01	6,64E-01	8,84E-01	Pstk	1,03	No
ENSMUSG00000063188	1,36E-01	-1,726265586	5,56E-02	8,14E-01	9,68E-01	Olfr107	1,10	No
ENSMUSG00000063193	9,85E-02	5,598653469	2,07E+00	1,50E-01	4,91E-01	Cd300lb	1,07	No
ENSMUSG00000063200	-2,26E-02	4,810273475	8,70E-02	7,68E-01	9,41E-01	Nol7	0,98	No
ENSMUSG00000063229	1,81E-01	8,970619211	1,01E+01	1,50E-03	2,23E-02	Ldha	1,13	Yes
ENSMUSG00000063232	-6,79E-02	-2,548241956	4,75E-03	9,45E-01	1,00E+00	Serpina11	0,95	No
ENSMUSG00000063234	2,70E-01	2,848305234	5,46E+00	1,95E-02	1,49E-01	Gpr84	1,21	No
ENSMUSG00000063235	5,54E-03	5,170065296	5,89E-03	9,39E-01	1,00E+00	Ptpmt1	1,00	No
ENSMUSG00000063236	-1,81E-02	4,107429693	4,34E-02	8,35E-01	9,80E-01	1110038F14Rik	0,99	No
ENSMUSG00000063239	2,64E+00	-2,655094739	2,70E+00	1,01E-01	3,92E-01	Grm4	6,25	No
ENSMUSG00000063245	1,11E-01	4,188112942	1,69E+00	1,94E-01	5,50E-01	Zfp993	1,08	No
ENSMUSG00000063253	1,47E-01	3,604125825	2,25E+00	1,33E-01	4,60E-01	Scoc	1,11	No
ENSMUSG00000063268	6,41E-03	5,184002077	7,98E-03	9,29E-01	1,00E+00	Parp10	1,00	No
ENSMUSG00000063273	1,62E-01	6,256027152	6,41E+00	1,13E-02	1,02E-01	Naa15	1,12	No
ENSMUSG00000063275	-4,50E-02	0,21828354	2,49E-02	8,75E-01	1,00E+00	Hacd1	0,97	No
ENSMUSG00000063281	1,03E-01	4,102419243	1,41E+00	2,35E-01	5,50E-01	Zfp35	1,07	No
ENSMUSG00000063286	1,57E-01	8,206700845	7,33E+00	6,79E-03	7,03E-02	Gm8995	1,11	No
ENSMUSG00000063314	-8,75E-02	5,568952992	1,62E+00	2,03E-01	5,50E-01	Gm12657	0,94	No
ENSMUSG00000063316	-1,54E-01	4,34332873	3,49E+00	6,18E-02	3,09E-01	Rpl27	0,90	No
ENSMUSG00000063317	1,04E-01	5,226970654	2,14E+00	1,43E-01	4,79E-01	Usp31	1,08	No
ENSMUSG00000063320	1,47E-03	1,187490473	5,54E-05	9,94E-01	1,00E+00	1190007I07Rik	1,00	No
ENSMUSG00000063328	-2,26E-01	6,123801231	1,22E+01	4,80E-04	8,89E-03	Gm9396	0,86	Yes
ENSMUSG00000063334	-1,87E-02	5,991006718	8,12E-02	7,76E-01	9,45E-01	Krr1	0,99	No
ENSMUSG00000063354	-2,25E-01	3,289341335	4,70E+00	3,02E-02	2,00E-01	Slc39a4	0,86	No

ENSMUSG00000063358	-1,51E-02	7,525861229	6,52E-02	7,98E-01	9,59E-01	Mapk1	0,99	No
ENSMUSG00000063362	1,94E-01	4,069403593	4,91E+00	2,67E-02	1,83E-01	Alg11	1,14	No
ENSMUSG00000063364	9,81E-02	0,351655459	1,60E-01	6,89E-01	8,99E-01	3300002I08Rik	1,07	No
ENSMUSG00000063376	-1,87E+00	-3,107637761	1,39E+00	2,38E-01	5,50E-01	Ifna13	0,27	No
ENSMUSG00000063382	-3,12E-01	5,890660822	2,22E+01	2,46E-06	9,73E-05	Bcl9l	0,81	Yes
ENSMUSG00000063383	3,12E-01	0,950279125	2,17E+00	1,41E-01	4,74E-01	Zfp947	1,24	No
ENSMUSG00000063388	-1,94E+00	-3,107619243	1,50E+00	2,21E-01	5,50E-01	BC023105	0,26	No
ENSMUSG00000063406	1,22E-01	7,09470547	4,06E+00	4,40E-02	2,50E-01	Tmed5	1,09	No
ENSMUSG00000063410	-8,85E-02	7,645551718	2,26E+00	1,33E-01	4,58E-01	Stk24	0,94	No
ENSMUSG00000063412	-4,42E-02	5,569683584	4,15E-01	5,19E-01	7,98E-01	Gm10131	0,97	No
ENSMUSG00000063415	-5,85E-01	1,484348853	9,91E+00	1,64E-03	2,39E-02	Cyp26b1	0,67	Yes
ENSMUSG00000063430	9,34E-02	-0,413070997	5,39E-02	8,16E-01	9,69E-01	Wscd2	1,07	No
ENSMUSG00000063439	6,90E-03	4,491636513	7,23E-03	9,32E-01	1,00E+00	B9d2	1,00	No
ENSMUSG00000063445	1,78E-01	3,488949578	3,21E+00	7,33E-02	3,44E-01	Nmral1	1,13	No
ENSMUSG00000063447	1,22E+00	-2,770593231	9,26E-01	3,36E-01	6,37E-01	Ube2d2b	2,33	No
ENSMUSG00000063450	6,57E-02	5,448559503	9,02E-01	3,42E-01	6,43E-01	Syne2	1,05	No
ENSMUSG00000063455	-5,61E-01	0,480918798	5,34E+00	2,09E-02	1,56E-01	D630045J12Rik	0,68	No
ENSMUSG00000063457	-7,43E-02	7,103378404	1,51E+00	2,19E-01	5,50E-01	Rps15	0,95	No
ENSMUSG00000063458	-1,28E-01	2,384085125	9,28E-01	3,35E-01	6,36E-01	Lrmda	0,91	No
ENSMUSG00000063480	-1,37E-02	5,316868357	3,76E-02	8,46E-01	9,87E-01	Snu13	0,99	No
ENSMUSG00000063488	-1,92E-02	1,776265297	1,53E-02	9,01E-01	1,00E+00	Zkscan7	0,99	No
ENSMUSG00000063506	-4,17E-01	3,178927901	1,55E+01	8,12E-05	2,04E-03	Arhgap22	0,75	Yes
ENSMUSG00000063511	-1,71E-01	7,78177452	8,54E+00	3,47E-03	4,27E-02	Snrnp70	0,89	Yes
ENSMUSG00000063522	6,33E-01	-1,448698195	1,18E+00	2,78E-01	5,74E-01	2010109I03Rik	1,55	No
ENSMUSG00000063524	1,81E-01	8,065440053	9,68E+00	1,86E-03	2,64E-02	Eno1	1,13	Yes
ENSMUSG00000063531	-3,59E+00	-2,770568198	5,79E+00	1,61E-02	1,32E-01	Sema3e	0,08	No
ENSMUSG00000063535	6,69E-01	-0,697434256	3,08E+00	7,95E-02	3,61E-01	Zfp773	1,59	No
ENSMUSG00000063543	-8,96E-02	3,19121848	6,94E-01	4,05E-01	7,02E-01	Gm5616	0,94	No
ENSMUSG00000063548	-1,87E+00	-3,107637761	1,39E+00	2,38E-01	5,50E-01	Gm4825	0,27	No
ENSMUSG00000063550	-6,64E-03	6,664460035	1,15E-02	9,15E-01	1,00E+00	Nup98	1,00	No
ENSMUSG00000063556	-1,61E-01	7,106715239	7,14E+00	7,52E-03	7,57E-02	Gm10132	0,89	No
ENSMUSG00000063558	-7,00E-01	-1,929220859	1,12E+00	2,90E-01	5,86E-01	Aox1	0,62	No

ENSMUSG00000063564	3,53E-02	1,7788394	4,64E-02	8,29E-01	9,77E-01	Col23a1	1,02	No
ENSMUSG00000063568	-1,01E-01	3,749986627	1,20E+00	2,74E-01	5,70E-01	Jazf1	0,93	No
ENSMUSG00000063576	4,51E-02	5,736489524	4,49E-01	5,03E-01	7,88E-01	Klhdc3	1,03	No
ENSMUSG00000063583	-4,74E-02	-2,832029451	1,49E-03	9,69E-01	1,00E+00	Olfr1410	0,97	No
ENSMUSG00000063586	-7,07E-01	-1,574195491	1,72E+00	1,90E-01	5,50E-01	Gm5513	0,61	No
ENSMUSG00000063594	-6,35E-01	-1,311155457	1,49E+00	2,23E-01	5,50E-01	Gng8	0,64	No
ENSMUSG00000063600	3,24E-01	-2,497561456	1,07E-01	7,44E-01	9,29E-01	Egfem1	1,25	No
ENSMUSG00000063605	-3,40E-02	3,735073857	1,34E-01	7,14E-01	9,12E-01	Ccdc102a	0,98	No
ENSMUSG00000063611	4,29E-01	2,800464558	1,28E+01	3,40E-04	6,70E-03	Gm10134	1,35	Yes
ENSMUSG00000063623	2,84E-01	5,160366492	1,52E+01	9,44E-05	2,31E-03	C230062I16Rik	1,22	Yes
ENSMUSG00000063626	1,82E+00	-2,963253027	1,31E+00	2,52E-01	5,50E-01	Unc5d	3,53	No
ENSMUSG00000063628	-2,29E-01	7,564076766	1,50E+01	1,10E-04	2,63E-03	Gm7665	0,85	Yes
ENSMUSG00000063632	-1,87E+00	-3,03364178	1,41E+00	2,36E-01	5,50E-01	Sox11	0,27	No
ENSMUSG00000063646	-4,78E-02	-2,655150386	2,06E-03	9,64E-01	1,00E+00	Jakmip1	0,97	No
ENSMUSG00000063651	-1,87E+00	-3,107637761	1,39E+00	2,38E-01	5,50E-01	Cnfn	0,27	No
ENSMUSG00000063652	4,11E-01	3,251210624	1,43E+01	1,52E-04	3,44E-03	Slc22a21	1,33	Yes
ENSMUSG00000063656	-1,23E-01	1,961832903	6,56E-01	4,18E-01	7,13E-01	Gm10135	0,92	No
ENSMUSG00000063659	-9,51E-02	7,319753953	2,55E+00	1,10E-01	4,07E-01	Zbtb18	0,94	No
ENSMUSG00000063660	-2,91E-01	-1,535336054	3,10E-01	5,78E-01	8,35E-01	Olfr98	0,82	No
ENSMUSG00000063672	1,87E+00	-3,107639691	1,38E+00	2,40E-01	5,50E-01	Nkx6-3	3,67	No
ENSMUSG00000063681	1,87E+00	-3,03364371	1,36E+00	2,43E-01	5,50E-01	Crb1	3,66	No
ENSMUSG00000063683	1,85E+00	-3,107657832	1,35E+00	2,46E-01	5,50E-01	Glyat	3,61	No
ENSMUSG00000063684	-1,69E-03	6,377682381	7,10E-04	9,79E-01	1,00E+00	Gm13910	1,00	No
ENSMUSG00000063689	1,05E+00	-2,929330032	4,72E-01	4,92E-01	7,78E-01	Hist2h2ab	2,07	No
ENSMUSG00000063694	7,57E-02	4,815984791	9,77E-01	3,23E-01	6,23E-01	Cycs	1,05	No
ENSMUSG00000063696	-1,47E-01	9,468958036	6,78E+00	9,21E-03	8,78E-02	Gm8730	0,90	No
ENSMUSG00000063698	-6,12E-01	-0,831947941	2,32E+00	1,28E-01	4,49E-01	Sfxn4	0,65	No
ENSMUSG00000063704	-7,33E-01	-2,497533592	6,03E-01	4,38E-01	7,30E-01	Mapk15	0,60	No
ENSMUSG00000063713	1,87E+00	-2,548299451	2,62E+00	1,06E-01	3,98E-01	Klk1b24	3,67	No
ENSMUSG00000063714	-1,92E-01	1,25902877	1,06E+00	3,04E-01	6,01E-01	Sp3os	0,88	No
ENSMUSG00000063727	-6,15E-01	-0,205662795	3,58E+00	5,83E-02	2,98E-01	Tnfrsf11b	0,65	No
ENSMUSG00000063739	-6,08E-02	5,303049576	7,33E-01	3,92E-01	6,92E-01	Gm4963	0,96	No

ENSMUSG00000063751	-3,00E-01	-2,311127766	1,35E-01	7,14E-01	9,12E-01	Platr8	0,81	No
ENSMUSG00000063754	-3,68E-01	6,058818072	3,20E+01	1,55E-08	1,03E-06	Gm10136	0,77	Yes
ENSMUSG00000063760	7,87E-02	5,581445224	1,32E+00	2,51E-01	5,50E-01	Rnf217	1,06	No
ENSMUSG00000063765	1,60E-01	1,902607048	1,08E+00	2,99E-01	5,95E-01	Chadl	1,12	No
ENSMUSG00000063767	-1,41E+00	-2,770599592	1,25E+00	2,64E-01	5,59E-01	S100a7a	0,38	No
ENSMUSG00000063779	1,80E-01	1,583892011	1,03E+00	3,10E-01	6,09E-01	Chil4	1,13	No
ENSMUSG00000063787	-1,68E-01	4,493716949	4,34E+00	3,73E-02	2,30E-01	Chchd1	0,89	No
ENSMUSG00000063796	-1,14E-02	-3,033635021	1,50E-05	9,97E-01	1,00E+00	Slc22a8	0,99	No
ENSMUSG00000063800	-5,55E-02	4,070908454	4,00E-01	5,27E-01	8,04E-01	Prpf38a	0,96	No
ENSMUSG00000063801	3,77E-02	5,460798754	2,94E-01	5,87E-01	8,40E-01	Ap3s2	1,03	No
ENSMUSG00000063802	-6,87E-02	4,912810264	8,38E-01	3,60E-01	6,61E-01	Hspbp1	0,95	No
ENSMUSG00000063808	2,19E-01	3,368671448	4,61E+00	3,18E-02	2,07E-01	Gpatch1	1,16	No
ENSMUSG00000063810	3,56E-01	4,1818695	1,69E+01	3,94E-05	1,11E-03	Alms1	1,28	Yes
ENSMUSG00000063820	3,11E-01	-1,205992408	4,10E-01	5,22E-01	8,00E-01	Arl9	1,24	No
ENSMUSG00000063821	1,85E+00	-3,107657832	1,35E+00	2,46E-01	5,50E-01	Dupd1	3,61	No
ENSMUSG00000063830	-1,40E+00	-2,896110316	1,23E+00	2,67E-01	5,63E-01	Gm6065	0,38	No
ENSMUSG00000063838	1,84E+00	-3,033632273	1,33E+00	2,49E-01	5,50E-01	Cdc42ep5	3,58	No
ENSMUSG00000063849	8,71E-02	4,26829724	1,07E+00	3,01E-01	5,97E-01	Ppcdc	1,06	No
ENSMUSG00000063851	1,60E+00	-2,65522043	1,76E+00	1,85E-01	5,49E-01	Rnf183	3,04	No
ENSMUSG00000063856	-3,66E-02	9,985712774	4,23E-01	5,15E-01	7,97E-01	Gpx1	0,97	No
ENSMUSG00000063870	1,01E-01	8,592764922	3,11E+00	7,78E-02	3,56E-01	Chd4	1,07	No
ENSMUSG00000063873	-7,45E-01	-0,268101299	4,22E+00	3,99E-02	2,37E-01	Slc24a3	0,60	No
ENSMUSG00000063875	-3,02E-01	3,320686844	8,27E+00	4,02E-03	4,75E-02	Rps6-ps1	0,81	Yes
ENSMUSG00000063882	-1,98E-01	6,257879305	9,59E+00	1,95E-03	2,75E-02	Uqcrh	0,87	Yes
ENSMUSG00000063884	3,34E-02	4,563827625	1,77E-01	6,74E-01	8,90E-01	Ptcd3	1,02	No
ENSMUSG00000063885	9,20E-02	-0,847847571	5,22E-02	8,19E-01	9,71E-01	Gm6498	1,07	No
ENSMUSG00000063887	1,60E+00	-2,832072821	1,75E+00	1,86E-01	5,50E-01	Nlgn1	3,03	No
ENSMUSG00000063888	2,24E-02	5,975527425	1,17E-01	7,32E-01	9,22E-01	Rpl7l1	1,02	No
ENSMUSG00000063889	-2,99E-01	4,557180346	1,44E+01	1,47E-04	3,32E-03	Crem	0,81	Yes
ENSMUSG00000063894	2,45E-01	3,804865028	7,24E+00	7,13E-03	7,28E-02	Zkscan8	1,18	No
ENSMUSG00000063895	2,47E-01	4,261559427	8,57E+00	3,42E-03	4,23E-02	Nupl1	1,19	Yes
ENSMUSG00000063900	-2,70E+00	-3,033614577	2,89E+00	8,93E-02	3,79E-01	Adam26b	0,15	No

ENSMUSG00000063902	1,71E-02	8,288203321	8,77E-02	7,67E-01	9,40E-01	Gm7964	1,01	No
ENSMUSG00000063903	4,12E-02	-1,448655842	5,75E-03	9,40E-01	1,00E+00	Klk1	1,03	No
ENSMUSG00000063904	6,47E-02	6,489335362	1,06E+00	3,03E-01	5,99E-01	4930533O14Rik	1,05	No
ENSMUSG00000063911	1,85E+00	-2,963264462	1,34E+00	2,48E-01	5,50E-01	Tdgf1-ps2	3,59	No
ENSMUSG00000063931	1,44E-01	6,126051736	4,89E+00	2,70E-02	1,85E-01	Pepd	1,10	No
ENSMUSG00000063952	-1,81E-01	5,252095142	6,55E+00	1,05E-02	9,66E-02	Brpf3	0,88	No
ENSMUSG00000063953	9,27E-02	4,440110979	1,30E+00	2,54E-01	5,50E-01	Amd2	1,07	No
ENSMUSG00000063954	1,70E-02	1,791563993	1,12E-02	9,16E-01	1,00E+00	Hist2h2aa2	1,01	No
ENSMUSG00000063972	-4,42E-01	2,825741356	1,39E+01	1,91E-04	4,18E-03	Nr6a1	0,74	Yes
ENSMUSG00000063975	-8,10E-01	-2,963247942	3,56E-01	5,51E-01	8,17E-01	Slco1a5	0,57	No
ENSMUSG00000064023	3,42E-01	0,661334363	2,16E+00	1,41E-01	4,75E-01	Klk8	1,27	No
ENSMUSG00000064030	-9,51E-02	4,461202806	1,38E+00	2,41E-01	5,50E-01	Pym1	0,94	No
ENSMUSG00000064032	1,78E-01	5,475849305	6,59E+00	1,03E-02	9,52E-02	Gm10143	1,13	No
ENSMUSG00000064037	1,47E-02	4,219882332	3,06E-02	8,61E-01	9,96E-01	Gpn1	1,01	No
ENSMUSG00000064039	8,32E-01	-1,107691459	3,24E+00	7,19E-02	3,40E-01	Ccr1l1	1,78	No
ENSMUSG00000064043	2,05E-01	7,390110613	1,18E+01	6,03E-04	1,07E-02	Trerf1	1,15	Yes
ENSMUSG00000064057	7,46E-01	-2,832025862	3,03E-01	5,82E-01	8,38E-01	Scgb3a1	1,68	No
ENSMUSG00000064061	5,22E-03	3,402042934	2,68E-03	9,59E-01	1,00E+00	Dzip3	1,00	No
ENSMUSG00000064063	-2,17E-01	-1,246884461	2,20E-01	6,39E-01	8,69E-01	BC048507	0,86	No
ENSMUSG00000064065	2,17E-01	3,740849488	5,27E+00	2,16E-02	1,58E-01	Ipcef1	1,16	No
ENSMUSG00000064068	1,63E-02	4,711565387	4,37E-02	8,34E-01	9,79E-01	Mtx1	1,01	No
ENSMUSG00000064080	-1,13E+00	-1,963104737	2,50E+00	1,14E-01	4,17E-01	Fbln2	0,46	No
ENSMUSG00000064081	-1,71E+00	-2,8320129	2,02E+00	1,55E-01	4,99E-01	Gm8973	0,31	No
ENSMUSG00000064090	-6,96E-02	4,139506915	6,69E-01	4,13E-01	7,09E-01	Vrk2	0,95	No
ENSMUSG00000064105	2,22E-01	3,754796075	5,43E+00	1,98E-02	1,50E-01	Cnnm2	1,17	No
ENSMUSG00000064109	-1,01E-01	1,880569261	4,25E-01	5,14E-01	7,96E-01	Hcst	0,93	No
ENSMUSG00000064115	1,81E+00	-3,10765737	1,28E+00	2,58E-01	5,50E-01	Cadm2	3,49	No
ENSMUSG00000064120	-2,76E-02	5,177923782	1,47E-01	7,01E-01	9,05E-01	Mocs1	0,98	No
ENSMUSG00000064125	-6,79E-02	-0,879752458	2,80E-02	8,67E-01	9,99E-01	Prr36	0,95	No
ENSMUSG00000064128	9,97E-02	3,785535264	1,15E+00	2,83E-01	5,79E-01	Cenpj	1,07	No
ENSMUSG00000064138	5,23E-02	5,307892512	5,42E-01	4,62E-01	7,50E-01	Fam172a	1,04	No
ENSMUSG00000064140	-1,50E+00	-2,355621574	2,31E+00	1,29E-01	4,50E-01	Trim38	0,35	No

ENSMUSG00000064141	-5,80E-01	1,527603006	1,06E+01	1,14E-03	1,79E-02	Zfp69	0,67	Yes
ENSMUSG00000064145	-2,18E-01	5,932624444	1,08E+01	1,04E-03	1,67E-02	Arih2	0,86	Yes
ENSMUSG00000064147	-3,13E-01	5,146680073	1,89E+01	1,41E-05	4,53E-04	Rab44	0,80	Yes
ENSMUSG00000064158	-3,91E-01	-2,863714745	9,44E-02	7,59E-01	9,36E-01	Izumo1	0,76	No
ENSMUSG00000064177	9,39E-02	1,592394795	2,94E-01	5,88E-01	8,40E-01	Ghrl	1,07	No
ENSMUSG00000064179	3,04E-01	-0,510154818	6,81E-01	4,09E-01	7,06E-01	Tnnt1	1,23	No
ENSMUSG00000064181	2,09E-01	2,807092266	3,12E+00	7,72E-02	3,54E-01	Rab3ip	1,16	No
ENSMUSG00000064193	1,70E-01	9,486413771	9,06E+00	2,61E-03	3,45E-02	Gm4735	1,13	Yes
ENSMUSG00000064194	2,65E+00	-2,831982301	2,71E+00	9,98E-02	3,92E-01	Zfp936	6,28	No
ENSMUSG00000064202	-3,35E-02	-0,252266777	1,16E-02	9,14E-01	1,00E+00	4430402I18Rik	0,98	No
ENSMUSG00000064208	-9,76E-02	5,089351023	1,78E+00	1,82E-01	5,44E-01	Gm10145	0,93	No
ENSMUSG00000064210	8,56E-02	6,502545792	1,85E+00	1,73E-01	5,31E-01	Ano6	1,06	No
ENSMUSG00000064215	-2,55E-01	4,400677247	9,68E+00	1,86E-03	2,64E-02	Ifi27	0,84	Yes
ENSMUSG00000064220	1,14E-01	1,161882363	3,27E-01	5,68E-01	8,29E-01	Hist2h2aa1	1,08	No
ENSMUSG00000064225	-1,31E+00	-2,070107598	2,94E+00	8,63E-02	3,78E-01	Paqr9	0,40	No
ENSMUSG00000064231	-3,20E-01	-2,146063398	1,54E-01	6,95E-01	9,02E-01	Gm5321	0,80	No
ENSMUSG00000064246	-1,66E+00	-1,831927078	5,30E+00	2,13E-02	1,57E-01	Chil1	0,32	No
ENSMUSG00000064247	-6,82E-02	2,681983301	3,10E-01	5,78E-01	8,35E-01	Plcxd1	0,95	No
ENSMUSG00000064254	2,77E-02	3,556281623	7,74E-02	7,81E-01	9,49E-01	Ethe1	1,02	No
ENSMUSG00000064262	-1,38E+00	-1,831985887	5,51E+00	1,89E-02	1,46E-01	Gimap8	0,38	No
ENSMUSG00000064264	3,93E-01	0,539386134	2,62E+00	1,05E-01	3,97E-01	Zfp428	1,31	No
ENSMUSG00000064267	1,63E-02	8,071740511	7,92E-02	7,78E-01	9,47E-01	Hvcn1	1,01	No
ENSMUSG00000064272	9,88E-01	-0,801093822	5,53E+00	1,87E-02	1,45E-01	Gpbar1	1,98	No
ENSMUSG00000064280	-7,93E-02	2,668124815	3,79E-01	5,38E-01	8,09E-01	Ccdc146	0,95	No
ENSMUSG00000064281	-1,35E-01	6,217193753	4,42E+00	3,55E-02	2,23E-01	Rpl19-ps1	0,91	No
ENSMUSG00000064284	6,87E-02	3,072207333	3,90E-01	5,32E-01	8,06E-01	Cdpf1	1,05	No
ENSMUSG00000064288	-1,92E-01	-0,556860474	2,80E-01	5,97E-01	8,45E-01	Hist1h4k	0,88	No
ENSMUSG00000064289	-3,59E-02	6,555077951	3,30E-01	5,66E-01	8,28E-01	Tank	0,98	No
ENSMUSG00000064294	-2,03E+00	-2,401390559	5,09E+00	2,41E-02	1,70E-01	Aox3	0,24	No
ENSMUSG00000064299	-5,30E-01	1,813675228	1,20E+01	5,23E-04	9,52E-03	4921528I07Rik	0,69	Yes
ENSMUSG00000064302	9,93E-03	5,675222549	2,15E-02	8,83E-01	1,00E+00	Clasp1	1,01	No
ENSMUSG00000064307	-1,54E-01	1,091567781	5,89E-01	4,43E-01	7,34E-01	Lrrc51	0,90	No

ENSMUSG00000064308	1,87E+00	-3,107639691	1,38E+00	2,40E-01	5,50E-01	2410137M14Rik	3,67	No
ENSMUSG00000064317	1,58E-02	3,712780275	2,76E-02	8,68E-01	1,00E+00	Gm10146	1,01	No
ENSMUSG00000064325	-9,13E-01	0,909222229	2,02E+01	6,83E-06	2,38E-04	Hhip	0,53	Yes
ENSMUSG00000064326	3,66E-02	3,63465713	1,45E-01	7,03E-01	9,06E-01	Siva1	1,03	No
ENSMUSG00000064329	-1,23E+00	-2,448652601	1,44E+00	2,30E-01	5,50E-01	Scn1a	0,43	No
ENSMUSG00000064330	3,02E-02	6,539928982	2,33E-01	6,30E-01	8,62E-01	Pde6h	1,02	No
ENSMUSG00000064373	9,76E-02	6,572234453	2,44E+00	1,19E-01	4,28E-01	Selenop	1,07	No
ENSMUSG00000064380	4,69E-01	-0,430747686	2,01E+00	1,56E-01	5,01E-01	Gm26448	1,38	No
ENSMUSG00000064382	-4,58E-01	0,896545451	4,54E+00	3,31E-02	2,13E-01	Gm26447	0,73	No
ENSMUSG00000064387	-2,54E-01	-0,998004409	3,41E-01	5,59E-01	8,22E-01	Snora73a	0,84	No
ENSMUSG00000064389	1,89E-01	-1,997987319	5,31E-02	8,18E-01	9,70E-01	Gm26446	1,14	No
ENSMUSG00000064390	-2,98E-01	-0,205774779	8,04E-01	3,70E-01	6,71E-01	Rnu73b	0,81	No
ENSMUSG00000064399	2,65E+00	-3,033655087	2,73E+00	9,86E-02	3,91E-01	Gm23629	6,30	No
ENSMUSG00000064405	-4,16E-01	-0,943234464	9,00E-01	3,43E-01	6,43E-01	Gm23925	0,75	No
ENSMUSG00000064409	9,29E-01	-1,946172527	1,79E+00	1,81E-01	5,42E-01	n-R5s40	1,90	No
ENSMUSG00000064410	3,55E-01	-2,233298471	1,94E-01	6,59E-01	8,81E-01	Gm22247	1,28	No
ENSMUSG00000064412	1,08E+00	-2,548266829	1,11E+00	2,92E-01	5,87E-01	Gm22246	2,12	No
ENSMUSG00000064414	-2,68E-02	-1,289540813	2,65E-03	9,59E-01	1,00E+00	n-R5s215	0,98	No
ENSMUSG00000064419	-5,42E-01	0,409661081	4,29E+00	3,82E-02	2,32E-01	Gm22249	0,69	No
ENSMUSG00000064422	-7,79E-02	-2,831996442	2,57E-03	9,60E-01	1,00E+00	Gm22502	0,95	No
ENSMUSG00000064427	-2,13E-01	0,839147171	9,15E-01	3,39E-01	6,39E-01	Gm22748	0,86	No
ENSMUSG00000064435	-1,89E-01	-1,205793515	1,69E-01	6,81E-01	8,94E-01	Gm25588	0,88	No
ENSMUSG00000064437	6,21E-02	-0,061167877	4,38E-02	8,34E-01	9,79E-01	Snord49b	1,04	No
ENSMUSG00000064440	2,35E-01	-0,529067482	3,95E-01	5,30E-01	8,05E-01	Gm26224	1,18	No
ENSMUSG00000064441	3,34E-01	-0,634421231	7,46E-01	3,88E-01	6,88E-01	Snord37	1,26	No
ENSMUSG00000064443	2,74E-02	-3,033621357	9,16E-12	1,00E+00	1,00E+00	Gm26226	1,02	No
ENSMUSG00000064450	2,74E-01	-1,54824226	2,36E-01	6,27E-01	8,61E-01	Snord68	1,21	No
ENSMUSG00000064451	2,63E-01	-1,322251273	2,88E-01	5,91E-01	8,42E-01	Snora23	1,20	No
ENSMUSG00000064453	-1,91E-01	-0,09805557	3,80E-01	5,37E-01	8,09E-01	Snord21	0,88	No
ENSMUSG00000064468	1,87E+00	-3,107639691	1,38E+00	2,40E-01	5,50E-01	Gm25081	3,67	No
ENSMUSG00000064489	1,87E+00	-3,03364371	1,36E+00	2,43E-01	5,50E-01	Gm23296	3,66	No
ENSMUSG00000064491	1,33E-02	-1,401308461	6,37E-04	9,80E-01	1,00E+00	Gm22061	1,01	No

ENSMUSG00000064492	-3,55E-01	-2,311039685	1,59E-01	6,90E-01	8,99E-01	Gm22060	0,78	No
ENSMUSG00000064493	3,27E-01	1,952957763	4,38E+00	3,63E-02	2,26E-01	Snora28	1,25	No
ENSMUSG00000064494	-5,20E-01	-1,205831944	1,10E+00	2,95E-01	5,91E-01	Gm22063	0,70	No
ENSMUSG00000064495	1,14E+00	-1,740953158	3,55E+00	5,96E-02	3,02E-01	Gm24313	2,20	No
ENSMUSG00000064500	-5,22E-01	-0,896139393	1,64E+00	2,01E-01	5,50E-01	Gm25296	0,70	No
ENSMUSG00000064508	-1,89E+00	-3,1076441	1,42E+00	2,33E-01	5,50E-01	n-R5s96	0,27	No
ENSMUSG00000064513	-1,32E-01	-0,015688825	2,00E-01	6,55E-01	8,79E-01	Snora9	0,91	No
ENSMUSG00000064514	1,78E-01	-0,093439553	3,08E-01	5,79E-01	8,36E-01	Gm22455	1,13	No
ENSMUSG00000064525	1,17E+00	-1,801057213	3,04E+00	8,12E-02	3,66E-01	Gm26440	2,24	No
ENSMUSG00000064540	-4,90E-01	-2,472393727	2,49E-01	6,17E-01	8,55E-01	Snord42a	0,71	No
ENSMUSG00000064542	1,73E+00	-1,946236371	5,41E+00	2,01E-02	1,51E-01	Gm22980	3,32	No
ENSMUSG00000064550	-4,10E-01	-2,548172971	1,72E-01	6,78E-01	8,92E-01	Gm24623	0,75	No
ENSMUSG00000064558	1,84E-01	-2,311233088	5,03E-02	8,22E-01	9,72E-01	Gm24620	1,14	No
ENSMUSG00000064563	-1,09E+00	-0,136162056	1,29E+01	3,20E-04	6,39E-03	Snora7a	0,47	Yes
ENSMUSG00000064565	-4,13E-01	-2,355563159	1,74E-01	6,76E-01	8,91E-01	Gm24145	0,75	No
ENSMUSG00000064566	-6,43E-02	-2,83202899	2,81E-03	9,58E-01	1,00E+00	Gm24144	0,96	No
ENSMUSG00000064567	1,81E+00	-3,10765737	1,28E+00	2,58E-01	5,50E-01	Gm24143	3,49	No
ENSMUSG00000064570	-2,68E+00	-2,963200749	2,85E+00	9,14E-02	3,82E-01	Gm25800	0,16	No
ENSMUSG00000064573	-1,94E+00	-3,107619243	1,50E+00	2,21E-01	5,50E-01	Gm25802	0,26	No
ENSMUSG00000064580	1,89E+00	-3,033614598	1,38E+00	2,40E-01	5,50E-01	Gm25134	3,70	No
ENSMUSG00000064585	-8,55E-01	-2,963271948	4,02E-01	5,26E-01	8,03E-01	Gm25129	0,55	No
ENSMUSG00000064587	-1,02E+00	-2,107625456	1,95E+00	1,62E-01	5,12E-01	Gm25131	0,49	No
ENSMUSG00000064590	2,18E-01	-2,401380721	5,98E-02	8,07E-01	9,64E-01	Gm22301	1,16	No
ENSMUSG00000064595	-7,17E-01	-2,146166148	8,73E-01	3,50E-01	6,51E-01	Gm22300	0,61	No
ENSMUSG00000064597	1,88E+00	-2,963229875	1,37E+00	2,42E-01	5,50E-01	Gm22299	3,68	No
ENSMUSG00000064600	4,27E-01	-2,655157011	1,42E-01	7,06E-01	9,08E-01	Gm25636	1,34	No
ENSMUSG00000064602	-2,53E-01	-0,099716251	6,74E-01	4,12E-01	7,07E-01	Snora41	0,84	No
ENSMUSG00000064604	-5,88E-01	-1,189204024	1,64E+00	2,00E-01	5,50E-01	Snora44	0,67	No
ENSMUSG00000064606	1,89E+00	-3,033614598	1,38E+00	2,40E-01	5,50E-01	Gm25899	3,70	No
ENSMUSG00000064612	-9,36E-02	-1,098177709	4,20E-02	8,38E-01	9,82E-01	Gm26457	0,94	No
ENSMUSG00000064620	-1,90E+00	-3,033594154	1,44E+00	2,30E-01	5,50E-01	Gm22303	0,27	No
ENSMUSG00000064622	1,36E+00	-2,600713657	1,91E+00	1,67E-01	5,21E-01	Gm22304	2,57	No

ENSMUSG00000064624	-5,78E-02	-2,896167382	2,23E-03	9,62E-01	1,00E+00	Gm22305	0,96	No
ENSMUSG00000064634	-3,22E-01	-1,170664483	4,75E-01	4,90E-01	7,77E-01	Gm22620	0,80	No
ENSMUSG00000064637	-4,60E-01	-0,344274392	1,77E+00	1,83E-01	5,46E-01	Snora20	0,73	No
ENSMUSG00000064644	1,62E-03	-2,963242114	-1,56E-12	1,00E+00	1,00E+00	Gm23302	1,00	No
ENSMUSG00000064646	-7,59E-02	-2,71173022	3,14E-03	9,55E-01	1,00E+00	Gm23300	0,95	No
ENSMUSG00000064647	-2,10E-01	0,169476136	5,50E-01	4,58E-01	7,47E-01	Gm23301	0,86	No
ENSMUSG00000064648	7,60E-01	-2,831984587	3,14E-01	5,75E-01	8,34E-01	Gm23303	1,69	No
ENSMUSG00000064652	-1,90E+00	-3,033647263	1,44E+00	2,30E-01	5,50E-01	Gm24165	0,27	No
ENSMUSG00000064653	-5,88E-02	-3,033652696	1,62E-03	9,68E-01	1,00E+00	Gm26129	0,96	No
ENSMUSG00000064655	3,54E-01	-1,816439372	2,66E-01	6,06E-01	8,49E-01	Gm25788	1,28	No
ENSMUSG00000064658	-1,88E+00	-2,832021709	1,42E+00	2,34E-01	5,50E-01	Gm24166	0,27	No
ENSMUSG00000064663	-5,84E-01	-2,711698733	2,68E-01	6,05E-01	8,49E-01	Gm24859	0,67	No
ENSMUSG00000064666	-1,84E-01	-0,389799284	3,07E-01	5,80E-01	8,36E-01	Snora52	0,88	No
ENSMUSG00000064672	3,69E-02	-0,024705692	1,63E-02	8,99E-01	1,00E+00	Gm22806	1,03	No
ENSMUSG00000064696	1,04E+00	-2,152404516	1,88E+00	1,71E-01	5,26E-01	Gm24148	2,05	No
ENSMUSG00000064702	-5,71E-02	-2,655159966	2,22E-03	9,62E-01	1,00E+00	Gm24950	0,96	No
ENSMUSG00000064704	4,74E-01	-2,107634546	3,05E-01	5,81E-01	8,37E-01	n-R5s180	1,39	No
ENSMUSG00000064721	-9,08E-02	-0,417034053	6,73E-02	7,95E-01	9,57E-01	Gm25855	0,94	No
ENSMUSG00000064722	9,01E-03	-3,107646452	-5,16E-10	1,00E+00	1,00E+00	Gm25857	1,01	No
ENSMUSG00000064725	-8,05E-02	1,183628591	1,67E-01	6,83E-01	8,95E-01	Snord96a	0,95	No
ENSMUSG00000064731	-2,57E-01	-0,697297472	4,43E-01	5,06E-01	7,90E-01	Snord45b	0,84	No
ENSMUSG00000064741	1,84E-01	0,03531947	4,19E-01	5,17E-01	7,98E-01	Snord14a	1,14	No
ENSMUSG00000064751	1,02E-01	-1,051828613	5,03E-02	8,22E-01	9,72E-01	Gm26330	1,07	No
ENSMUSG00000064758	-1,71E+00	-2,655089183	2,04E+00	1,53E-01	4,96E-01	Gm26331	0,30	No
ENSMUSG00000064765	1,76E-01	-2,683122004	2,64E-02	8,71E-01	1,00E+00	Gm26456	1,13	No
ENSMUSG00000064767	1,45E-01	-1,664274041	6,50E-02	7,99E-01	9,59E-01	Snord35b	1,11	No
ENSMUSG00000064768	-1,17E+00	-2,963257985	5,98E-01	4,39E-01	7,32E-01	Snord60	0,44	No
ENSMUSG00000064769	-1,94E+00	-3,107619243	1,50E+00	2,21E-01	5,50E-01	Gm23518	0,26	No
ENSMUSG00000064770	9,77E-01	-0,98939522	5,22E+00	2,24E-02	1,62E-01	n-R5s29	1,97	No
ENSMUSG00000064778	-4,28E-01	-0,131565267	1,82E+00	1,77E-01	5,37E-01	Snord59a	0,74	No
ENSMUSG00000064780	3,44E-01	-0,763200302	7,32E-01	3,92E-01	6,92E-01	Snord95	1,27	No
ENSMUSG00000064791	4,69E-01	-1,378268529	7,95E-01	3,73E-01	6,74E-01	Snord14e	1,38	No

ENSMUSG00000064792	-2,35E+00	-2,896127955	2,16E+00	1,41E-01	4,75E-01	Gm24355	0,20	No
ENSMUSG00000064796	-7,96E-02	-2,655170372	7,35E-03	9,32E-01	1,00E+00	Terc	0,95	No
ENSMUSG00000064797	-2,25E-01	0,053928007	5,48E-01	4,59E-01	7,48E-01	Gm24357	0,86	No
ENSMUSG00000064805	1,85E+00	-3,107657832	1,35E+00	2,46E-01	5,50E-01	Gm24037	3,61	No
ENSMUSG00000064811	5,03E-01	-2,070253202	4,90E-01	4,84E-01	7,71E-01	Gm22358	1,42	No
ENSMUSG00000064814	1,87E+00	-3,107639691	1,38E+00	2,40E-01	5,50E-01	Gm23939	3,67	No
ENSMUSG00000064816	1,32E-01	1,088651528	4,32E-01	5,11E-01	7,94E-01	Gm22357	1,10	No
ENSMUSG00000064823	6,51E-01	-2,740902294	3,51E-01	5,54E-01	8,19E-01	Snord82	1,57	No
ENSMUSG00000064829	-3,66E-01	-0,632087833	9,79E-01	3,22E-01	6,23E-01	Gm22879	0,78	No
ENSMUSG00000064833	-3,57E+00	-2,831989825	5,72E+00	1,68E-02	1,35E-01	Gm25926	0,08	No
ENSMUSG00000064837	-2,68E-01	-0,34432397	6,23E-01	4,30E-01	7,23E-01	Snora75	0,83	No
ENSMUSG00000064841	-6,33E-01	-0,855601695	2,49E+00	1,15E-01	4,19E-01	Gm26205	0,64	No
ENSMUSG00000064844	8,08E-03	0,028180656	8,47E-04	9,77E-01	1,00E+00	Gm26202	1,01	No
ENSMUSG00000064845	1,79E+00	-1,711732058	8,02E+00	4,64E-03	5,28E-02	Gm26203	3,45	No
ENSMUSG00000064850	-2,68E+00	-3,033645766	2,84E+00	9,17E-02	3,82E-01	Gm23443	0,16	No
ENSMUSG00000064853	-1,61E+00	-1,497402448	8,52E+00	3,52E-03	4,31E-02	Gm23442	0,33	Yes
ENSMUSG00000064856	1,23E+00	-2,711717536	9,32E-01	3,34E-01	6,35E-01	Gm23444	2,34	No
ENSMUSG00000064858	6,23E-01	-1,963311701	9,01E-01	3,43E-01	6,43E-01	Snora43	1,54	No
ENSMUSG00000064859	-1,24E+00	-2,448683335	1,47E+00	2,25E-01	5,50E-01	Gm24524	0,42	No
ENSMUSG00000064867	1,81E+00	-3,10765737	1,28E+00	2,58E-01	5,50E-01	Gm25039	3,49	No
ENSMUSG00000064871	-5,54E-01	-0,387852924	2,76E+00	9,69E-02	3,88E-01	Snord58b	0,68	No
ENSMUSG00000064880	-1,82E-01	1,757074593	1,25E+00	2,63E-01	5,58E-01	Gm24201	0,88	No
ENSMUSG00000064887	1,92E+00	-2,711774895	2,75E+00	9,72E-02	3,89E-01	Gm24205	3,78	No
ENSMUSG00000064890	-5,23E-02	-2,963273906	1,24E-03	9,72E-01	1,00E+00	Gm22505	0,96	No
ENSMUSG00000064897	-6,51E-03	-3,033622701	4,10E-05	9,95E-01	1,00E+00	Gm22504	1,00	No
ENSMUSG00000064899	-3,18E+00	-2,963258684	4,26E+00	3,90E-02	2,34E-01	Snord118	0,11	No
ENSMUSG00000064900	9,01E-03	-3,107646452	-5,16E-10	1,00E+00	1,00E+00	Gm25402	1,01	No
ENSMUSG00000064901	-3,19E-01	-0,725875113	5,55E-01	4,56E-01	7,45E-01	Snora21	0,80	No
ENSMUSG00000064906	6,94E-01	-2,89617475	2,61E-01	6,09E-01	8,51E-01	n-R5s204	1,62	No
ENSMUSG00000064907	-1,26E+00	-2,497569017	1,51E+00	2,19E-01	5,50E-01	Gm25405	0,42	No
ENSMUSG00000064916	-8,19E-01	-2,033601249	9,78E-01	3,23E-01	6,23E-01	Gm22573	0,57	No
ENSMUSG00000064917	-2,07E-02	-0,430839664	3,58E-03	9,52E-01	1,00E+00	Gm22574	0,99	No

ENSMUSG00000064918	3,21E-01	0,915851923	2,15E+00	1,42E-01	4,77E-01	Gm22571	1,25	No
ENSMUSG00000064919	-7,54E-01	-1,070173758	2,84E+00	9,18E-02	3,82E-01	Gm22572	0,59	No
ENSMUSG00000064923	-1,94E+00	-3,107619243	1,50E+00	2,21E-01	5,50E-01	Gm22042	0,26	No
ENSMUSG00000064925	-1,57E-02	-1,509959367	9,57E-04	9,75E-01	1,00E+00	Snora62	0,99	No
ENSMUSG00000064927	-7,53E-02	-2,770585454	3,83E-03	9,51E-01	1,00E+00	Gm22043	0,95	No
ENSMUSG00000064930	1,75E-01	-2,683175171	2,26E-02	8,80E-01	1,00E+00	Gm23723	1,13	No
ENSMUSG00000064936	-3,26E-01	0,095818316	1,28E+00	2,58E-01	5,50E-01	Gm23722	0,80	No
ENSMUSG00000064938	4,28E-02	-1,692613142	4,39E-03	9,47E-01	1,00E+00	Mir3068	1,03	No
ENSMUSG00000064941	-1,86E+00	-3,033612668	1,39E+00	2,38E-01	5,50E-01	Gm23238	0,28	No
ENSMUSG00000064945	-3,83E-02	-3,107651936	-7,91E-11	1,00E+00	1,00E+00	Rny3	0,97	No
ENSMUSG00000064949	2,17E-01	-0,949224386	2,60E-01	6,10E-01	8,51E-01	Snora61	1,16	No
ENSMUSG00000064952	-2,71E+00	-2,89615606	2,90E+00	8,84E-02	3,79E-01	Gm24920	0,15	No
ENSMUSG00000064956	-8,80E-01	-2,963222719	4,28E-01	5,13E-01	7,95E-01	Gm24918	0,54	No
ENSMUSG00000064959	-2,63E-02	-3,033641358	2,33E-04	9,88E-01	1,00E+00	n-R5s41	0,98	No
ENSMUSG00000064960	-3,71E-01	-2,448645529	1,73E-01	6,78E-01	8,92E-01	Gm24406	0,77	No
ENSMUSG00000064964	-6,21E-01	-2,448646108	5,25E-01	4,69E-01	7,57E-01	Gm25147	0,65	No
ENSMUSG00000064966	-3,37E-01	-0,824076719	7,51E-01	3,86E-01	6,87E-01	Snord15b	0,79	No
ENSMUSG00000064968	-1,77E-01	-0,26874873	2,74E-01	6,01E-01	8,47E-01	Snord47	0,88	No
ENSMUSG00000064970	1,18E+00	-2,998025243	6,58E-01	4,17E-01	7,12E-01	Gm23690	2,26	No
ENSMUSG00000064984	5,40E-01	-0,929340753	1,63E+00	2,01E-01	5,50E-01	Snord73a	1,45	No
ENSMUSG00000064987	-5,88E-02	-3,033616507	1,62E-03	9,68E-01	1,00E+00	Gm25260	0,96	No
ENSMUSG00000064994	-4,67E-02	-2,711733432	1,50E-03	9,69E-01	1,00E+00	Gm22422	0,97	No
ENSMUSG00000064995	-1,69E-01	-0,221024421	2,71E-01	6,03E-01	8,48E-01	Gm26003	0,89	No
ENSMUSG00000065006	7,14E-01	-2,863747814	2,78E-01	5,98E-01	8,45E-01	Gm26325	1,64	No
ENSMUSG00000065016	-1,24E-01	0,222195326	2,05E-01	6,51E-01	8,76E-01	Snora3	0,92	No
ENSMUSG00000065034	-1,89E+00	-3,033648118	1,42E+00	2,33E-01	5,50E-01	Gm22363	0,27	No
ENSMUSG00000065036	-7,59E-01	0,136821453	6,72E+00	9,54E-03	9,00E-02	Gm22362	0,59	No
ENSMUSG00000065037	-8,44E-01	-0,378268058	5,62E+00	1,78E-02	1,40E-01	Rn7sk	0,56	No
ENSMUSG00000065041	1,39E-01	0,218100209	2,67E-01	6,05E-01	8,49E-01	Gm24044	1,10	No
ENSMUSG00000065044	1,21E+00	-2,998005207	6,74E-01	4,12E-01	7,07E-01	n-R5s169	2,32	No
ENSMUSG00000065047	-8,69E-01	-2,963204587	4,16E-01	5,19E-01	7,98E-01	Gm24045	0,55	No
ENSMUSG00000065049	-4,61E-02	-2,831979911	9,96E-04	9,75E-01	1,00E+00	Gm24041	0,97	No

ENSMUSG00000065057	-3,19E+00	-2,770490475	4,29E+00	3,83E-02	2,32E-01	Gm25703	0,11	No
ENSMUSG00000065061	-8,39E-02	-2,600654908	4,63E-03	9,46E-01	1,00E+00	Gm22884	0,94	No
ENSMUSG00000065072	-1,87E+00	-3,107637761	1,39E+00	2,38E-01	5,50E-01	Gm24533	0,27	No
ENSMUSG00000065078	1,17E+00	-3,14612376	6,40E-01	4,24E-01	7,18E-01	Gm24536	2,26	No
ENSMUSG00000065080	-1,89E+00	-3,1076441	1,42E+00	2,33E-01	5,50E-01	Gm26497	0,27	No
ENSMUSG00000065083	1,85E+00	-3,107657832	1,35E+00	2,46E-01	5,50E-01	n-R5s205	3,61	No
ENSMUSG00000065084	-6,03E-02	-2,548233673	4,16E-03	9,49E-01	1,00E+00	Gm26496	0,96	No
ENSMUSG00000065087	3,21E-01	0,815078896	2,14E+00	1,44E-01	4,79E-01	Snord22	1,25	No
ENSMUSG00000065089	-6,97E-01	-0,99809249	2,67E+00	1,02E-01	3,95E-01	Gm26493	0,62	No
ENSMUSG00000065094	-2,05E-01	-0,896037294	2,25E-01	6,35E-01	8,67E-01	Snord1a	0,87	No
ENSMUSG00000065097	-6,79E-01	-1,869451355	9,55E-01	3,29E-01	6,29E-01	Snora16a	0,62	No
ENSMUSG00000065105	-4,39E-01	0,804528467	3,83E+00	5,04E-02	2,72E-01	Gm26130	0,74	No
ENSMUSG00000065107	9,01E-03	-3,107646452	-5,16E-10	1,00E+00	1,00E+00	n-R5s88	1,01	No
ENSMUSG00000065118	2,36E-01	-2,033521005	7,00E-02	7,91E-01	9,55E-01	Gm23297	1,18	No
ENSMUSG00000065124	-3,63E-01	0,758491479	2,38E+00	1,23E-01	4,39E-01	Snora65	0,78	No
ENSMUSG00000065126	-5,49E-01	-0,088495588	3,36E+00	6,66E-02	3,25E-01	Snord104	0,68	No
ENSMUSG00000065127	-5,88E-02	-3,033616507	1,62E-03	9,68E-01	1,00E+00	Gm26127	0,96	No
ENSMUSG00000065130	-4,84E-01	-1,26811372	1,00E+00	3,16E-01	6,17E-01	Gm24457	0,72	No
ENSMUSG00000065132	-3,83E-02	-3,107651936	-7,91E-11	1,00E+00	1,00E+00	Gm24456	0,97	No
ENSMUSG00000065138	4,45E-01	-2,600667498	1,54E-01	6,94E-01	9,02E-01	Gm24458	1,36	No
ENSMUSG00000065145	-9,34E-01	-2,600635012	7,64E-01	3,82E-01	6,83E-01	Vaultrc5	0,52	No
ENSMUSG00000065147	4,13E-01	0,563791237	2,85E+00	9,16E-02	3,82E-01	Snora31	1,33	No
ENSMUSG00000065158	-8,86E-01	-1,14613865	3,37E+00	6,65E-02	3,24E-01	Gm25483	0,54	No
ENSMUSG00000065159	-1,89E+00	-3,1076441	1,42E+00	2,33E-01	5,50E-01	Gm25482	0,27	No
ENSMUSG00000065160	4,74E-01	-0,351959469	1,77E+00	1,83E-01	5,46E-01	Mir5117	1,39	No
ENSMUSG00000065162	-5,23E-01	-1,929335812	4,38E-01	5,08E-01	7,92E-01	Gm23645	0,70	No
ENSMUSG00000065164	-7,38E-02	-3,033634644	2,66E-03	9,59E-01	1,00E+00	Gm23814	0,95	No
ENSMUSG00000065165	1,85E+00	-3,107657832	1,35E+00	2,46E-01	5,50E-01	Gm23815	3,61	No
ENSMUSG00000065176	1,79E+00	-3,033666867	1,28E+00	2,58E-01	5,50E-01	Rnu12	3,46	No
ENSMUSG00000065191	-9,49E-02	-2,448698919	8,06E-03	9,28E-01	1,00E+00	Gm23458	0,94	No
ENSMUSG00000065196	4,61E+00	-2,497672957	1,20E+01	5,26E-04	9,55E-03	Snord85	24,38	Yes
ENSMUSG00000065200	1,17E+00	-3,14612376	6,40E-01	4,24E-01	7,18E-01	Gm24613	2,26	No

ENSMUSG00000065202	-2,71E+00	-3,033620915	2,92E+00	8,75E-02	3,78E-01	Gm24615	0,15	No
ENSMUSG00000065204	-1,51E+00	-1,863510226	5,00E+00	2,54E-02	1,77E-01	Gm24610	0,35	No
ENSMUSG00000065206	3,14E-02	-3,107617333	-7,69E-10	1,00E+00	1,00E+00	Gm24611	1,02	No
ENSMUSG00000065208	-2,83E-01	0,392835235	1,23E+00	2,67E-01	5,63E-01	Gm24616	0,82	No
ENSMUSG00000065211	-1,37E+00	-1,785633929	4,00E+00	4,54E-02	2,54E-01	Gm26265	0,39	No
ENSMUSG00000065219	-5,42E-01	-1,027641447	1,53E+00	2,15E-01	5,50E-01	Snord32a	0,69	No
ENSMUSG00000065226	-1,12E-01	-0,352746163	1,14E-01	7,35E-01	9,24E-01	Gm25791	0,93	No
ENSMUSG00000065227	-9,76E-01	-2,548197577	8,35E-01	3,61E-01	6,62E-01	Gm25790	0,51	No
ENSMUSG00000065228	-4,34E-01	-0,912321046	9,55E-01	3,28E-01	6,29E-01	Gm25789	0,74	No
ENSMUSG00000065231	-2,27E-01	1,030615332	1,16E+00	2,81E-01	5,78E-01	Gm22972	0,85	No
ENSMUSG00000065236	4,51E-01	-1,226409076	9,59E-01	3,27E-01	6,28E-01	n-R5s197	1,37	No
ENSMUSG00000065245	-4,62E-02	-2,711661418	1,47E-03	9,69E-01	1,00E+00	Gm22295	0,97	No
ENSMUSG00000065246	-7,59E-01	-2,31116704	6,45E-01	4,22E-01	7,17E-01	Gm22297	0,59	No
ENSMUSG00000065251	-5,88E-01	-2,655173593	2,71E-01	6,03E-01	8,48E-01	Gm23971	0,67	No
ENSMUSG00000065258	-2,64E-01	-0,252259881	7,08E-01	4,00E-01	6,99E-01	Gm23969	0,83	No
ENSMUSG00000065259	2,62E-01	-1,126860467	2,86E-01	5,93E-01	8,42E-01	Snora30	1,20	No
ENSMUSG00000065262	-3,20E+00	-2,711686274	4,32E+00	3,77E-02	2,31E-01	Gm23451	0,11	No
ENSMUSG00000065265	-5,29E-01	0,484790934	4,54E+00	3,32E-02	2,13E-01	Gm23455	0,69	No
ENSMUSG00000065270	-5,63E-01	-2,770594051	2,48E-01	6,19E-01	8,56E-01	Gm25127	0,68	No
ENSMUSG00000065272	-9,10E-02	-0,638965205	5,84E-02	8,09E-01	9,65E-01	Snord57	0,94	No
ENSMUSG00000065273	-5,25E-01	-1,333113746	1,05E+00	3,05E-01	6,01E-01	Gm25128	0,69	No
ENSMUSG00000065280	1,47E-01	-0,702169532	1,47E-01	7,01E-01	9,05E-01	Gm24453	1,11	No
ENSMUSG00000065281	1,37E-01	0,61599306	3,29E-01	5,66E-01	8,28E-01	Gm24452	1,10	No
ENSMUSG00000065282	-4,43E-01	0,015066227	2,13E+00	1,44E-01	4,80E-01	Gm24455	0,74	No
ENSMUSG00000065287	-3,52E-01	0,095801514	1,47E+00	2,25E-01	5,50E-01	Gm24451	0,78	No
ENSMUSG00000065289	1,65E-01	-0,19397428	2,56E-01	6,13E-01	8,53E-01	Gm23650	1,12	No
ENSMUSG00000065295	-3,63E-01	-0,063886197	1,49E+00	2,22E-01	5,50E-01	Gm24134	0,78	No
ENSMUSG00000065296	-8,21E-01	-2,963266074	3,67E-01	5,45E-01	8,13E-01	Gm26122	0,57	No
ENSMUSG00000065298	-1,92E+00	-3,0336536	1,46E+00	2,27E-01	5,50E-01	Gm26121	0,26	No
ENSMUSG00000065299	-4,08E-02	-2,963255775	7,59E-04	9,78E-01	1,00E+00	Gm23138	0,97	No
ENSMUSG00000065301	4,48E-01	-2,655112357	1,57E-01	6,92E-01	9,00E-01	Gm23248	1,36	No
ENSMUSG00000065304	-2,69E-01	-0,580660564	5,65E-01	4,52E-01	7,42E-01	Gm23245	0,83	No

ENSMUSG00000065305	-8,09E-02	-0,26449972	5,87E-02	8,09E-01	9,65E-01	Gm23246	0,95	No
ENSMUSG00000065307	1,61E+00	-2,832036347	1,77E+00	1,84E-01	5,47E-01	Gm23247	3,04	No
ENSMUSG00000065311	1,85E+00	-3,107657832	1,35E+00	2,46E-01	5,50E-01	n-R5s156	3,61	No
ENSMUSG00000065315	-6,68E-02	-1,126646749	1,98E-02	8,88E-01	1,00E+00	Gm26064	0,95	No
ENSMUSG00000065331	3,14E-02	-3,107617333	-7,69E-10	1,00E+00	1,00E+00	Gm24927	1,02	No
ENSMUSG00000065336	8,67E-03	-1,556961207	2,22E-04	9,88E-01	1,00E+00	Snora34	1,01	No
ENSMUSG00000065341	3,95E-01	-2,311230521	2,49E-01	6,18E-01	8,55E-01	Gm25579	1,31	No
ENSMUSG00000065343	5,01E-01	-1,683157186	6,30E-01	4,27E-01	7,21E-01	Gm25580	1,41	No
ENSMUSG00000065347	2,25E-01	-2,497600741	6,38E-02	8,01E-01	9,61E-01	Gm25583	1,17	No
ENSMUSG00000065348	1,84E+00	-3,033649193	1,33E+00	2,49E-01	5,50E-01	Gm25577	3,58	No
ENSMUSG00000065353	-1,20E-01	-0,349985415	1,21E-01	7,28E-01	9,20E-01	Snora73b	0,92	No
ENSMUSG00000065360	-3,86E-01	-2,070189232	3,46E-01	5,57E-01	8,21E-01	Gm24412	0,77	No
ENSMUSG00000065362	-6,88E-02	-1,268109869	1,83E-02	8,93E-01	1,00E+00	Gm24411	0,95	No
ENSMUSG00000065368	8,09E-01	-2,600670938	5,70E-01	4,50E-01	7,40E-01	Gm24414	1,75	No
ENSMUSG00000065374	3,13E+00	-2,770609441	4,01E+00	4,53E-02	2,54E-01	Gm22738	8,78	No
ENSMUSG00000065378	4,12E-01	-0,159383033	1,60E+00	2,06E-01	5,50E-01	Gm22744	1,33	No
ENSMUSG00000065387	-3,84E-01	-2,40130776	1,86E-01	6,66E-01	8,86E-01	n-R5s85	0,77	No
ENSMUSG00000065389	2,27E-01	-1,205853298	2,39E-01	6,25E-01	8,59E-01	Gm23100	1,17	No
ENSMUSG00000065392	-3,15E-01	-0,214203797	9,69E-01	3,25E-01	6,26E-01	Gm25894	0,80	No
ENSMUSG00000065394	1,47E-02	-1,464901493	6,71E-04	9,79E-01	1,00E+00	Mir25	1,01	No
ENSMUSG00000065395	1,82E+00	-3,033661385	1,31E+00	2,52E-01	5,50E-01	Mir193a	3,54	No
ENSMUSG00000065396	-1,89E+00	-3,1076441	1,42E+00	2,33E-01	5,50E-01	Mir99b	0,27	No
ENSMUSG00000065397	-1,01E+00	-1,257318426	4,11E+00	4,26E-02	2,45E-01	Mir155	0,50	No
ENSMUSG00000065399	2,49E-01	-2,548215523	7,81E-02	7,80E-01	9,48E-01	Mir133a-1	1,19	No
ENSMUSG00000065401	-1,94E+00	-3,107619243	1,50E+00	2,21E-01	5,50E-01	Mir144	0,26	No
ENSMUSG00000065403	-1,08E+00	-2,514598921	1,34E+00	2,47E-01	5,50E-01	Mir18	0,47	No
ENSMUSG00000065406	2,93E-01	-2,929296909	5,30E-02	8,18E-01	9,70E-01	Mirlet7i	1,22	No
ENSMUSG00000065407	8,73E-01	-2,28949091	1,04E+00	3,07E-01	6,05E-01	Mir135a-1	1,83	No
ENSMUSG00000065409	1,22E+00	-3,146114919	6,90E-01	4,06E-01	7,02E-01	Mir30e	2,33	No
ENSMUSG00000065412	5,06E-01	-2,769993243	1,80E-01	6,71E-01	8,89E-01	Mir29b-2	1,42	No
ENSMUSG00000065413	-5,90E-02	-2,97498172	1,59E-03	9,68E-01	1,00E+00	Mir367	0,96	No
ENSMUSG00000065414	1,66E+00	-2,896170739	1,34E+00	2,46E-01	5,50E-01	Mir138-1	3,15	No

ENSMUSG00000065416	-1,42E+00	-2,619474321	1,65E+00	1,99E-01	5,50E-01	Mir19a	0,37	No
ENSMUSG00000065417	-4,17E-02	-2,963202678	7,92E-04	9,78E-01	1,00E+00	Mir340	0,97	No
ENSMUSG00000065420	-7,36E-01	2,20463804	2,72E+01	1,84E-07	9,69E-06	Mir142b	0,60	Yes
ENSMUSG00000065429	2,25E+00	-2,929330971	1,96E+00	1,62E-01	5,11E-01	Mir345	4,77	No
ENSMUSG00000065430	1,25E+00	-2,740858078	9,66E-01	3,26E-01	6,26E-01	Mir26a-2	2,38	No
ENSMUSG00000065431	-1,89E-01	-1,015459572	1,83E-01	6,69E-01	8,87E-01	Mir186	0,88	No
ENSMUSG00000065434	2,91E-01	0,635553179	1,58E+00	2,09E-01	5,50E-01	Mir7-1	1,22	No
ENSMUSG00000065437	1,43E+00	-3,058381022	8,72E-01	3,50E-01	6,52E-01	Mir30d	2,69	No
ENSMUSG00000065439	1,20E+00	-2,378376038	1,66E+00	1,98E-01	5,50E-01	Mir140	2,29	No
ENSMUSG00000065440	1,81E+00	-3,10765737	1,28E+00	2,58E-01	5,50E-01	Mirlet7g	3,49	No
ENSMUSG00000065442	-1,12E+00	-2,429079041	1,52E+00	2,18E-01	5,50E-01	Mir20a	0,46	No
ENSMUSG00000065444	1,11E+00	-0,588503617	8,83E+00	2,97E-03	3,81E-02	Mir27a	2,16	Yes
ENSMUSG00000065445	1,87E+00	-3,107639691	1,38E+00	2,40E-01	5,50E-01	Mir143	3,67	No
ENSMUSG00000065447	-5,23E-01	-2,94075836	1,38E-01	7,10E-01	9,09E-01	Mir302d	0,70	No
ENSMUSG00000065449	1,85E+00	-3,107657832	1,35E+00	2,46E-01	5,50E-01	Mir365-1	3,61	No
ENSMUSG00000065451	3,82E-01	-2,832040853	7,34E-02	7,86E-01	9,52E-01	Mir101a	1,30	No
ENSMUSG00000065452	-2,88E-01	-2,015725494	1,44E-01	7,04E-01	9,06E-01	Mir207	0,82	No
ENSMUSG00000065453	9,55E-01	-2,333224214	1,13E+00	2,87E-01	5,83E-01	Mirlet7d	1,94	No
ENSMUSG00000065455	3,32E-02	-1,522718864	4,01E-03	9,50E-01	1,00E+00	Mir21a	1,02	No
ENSMUSG00000065458	1,44E+00	-2,907340105	1,22E+00	2,68E-01	5,65E-01	Mir181b-1	2,72	No
ENSMUSG00000065463	2,68E-01	-1,355719613	2,45E-01	6,20E-01	8,57E-01	Snord45c	1,20	No
ENSMUSG00000065464	-1,87E+00	-3,107637761	1,39E+00	2,38E-01	5,50E-01	Mir185	0,27	No
ENSMUSG00000065468	-3,61E-01	-2,226330568	1,64E-01	6,85E-01	8,97E-01	Mir26b	0,78	No
ENSMUSG00000065470	1,81E+00	-3,10765737	1,28E+00	2,58E-01	5,50E-01	Mir149	3,49	No
ENSMUSG00000065475	8,69E-01	-1,216142242	2,80E+00	9,40E-02	3,84E-01	Mir27b	1,83	No
ENSMUSG00000065479	1,21E+00	-3,07015885	6,74E-01	4,12E-01	7,07E-01	Mir125a	2,31	No
ENSMUSG00000065482	-1,24E+00	-2,963267303	7,18E-01	3,97E-01	6,97E-01	Mir302c	0,42	No
ENSMUSG00000065483	2,61E+00	-3,033672762	2,63E+00	1,05E-01	3,97E-01	Mir181c	6,10	No
ENSMUSG00000065489	4,79E-01	-2,226324542	3,11E-01	5,77E-01	8,35E-01	Mir365-2	1,39	No
ENSMUSG00000065490	7,63E-01	-2,896152067	3,18E-01	5,73E-01	8,32E-01	Mir30c-1	1,70	No
ENSMUSG00000065493	5,93E-01	-2,800991485	1,41E-01	7,07E-01	9,08E-01	Mir34a	1,51	No
ENSMUSG00000065495	-1,10E+00	-2,497578824	1,10E+00	2,94E-01	5,89E-01	Mir150	0,47	No

ENSMUSG00000065501	9,01E-03	-3,107646452	-5,16E-10	1,00E+00	1,00E+00	Mir335	1,01	No
ENSMUSG00000065502	-2,19E-01	-2,750907144	3,97E-02	8,42E-01	9,85E-01	Mir202	0,86	No
ENSMUSG00000065508	-9,04E-01	-2,614584237	7,10E-01	4,00E-01	6,98E-01	Mir17	0,53	No
ENSMUSG00000065512	-1,23E+00	-3,146117124	7,12E-01	3,99E-01	6,98E-01	Mir138-2	0,42	No
ENSMUSG00000065513	-5,62E-01	-2,548245651	2,48E-01	6,19E-01	8,56E-01	Mir26a-1	0,68	No
ENSMUSG00000065514	8,65E-01	-2,673603807	5,38E-01	4,63E-01	7,52E-01	Mir106b	1,82	No
ENSMUSG00000065518	-4,90E-01	-2,998036706	1,20E-01	7,29E-01	9,20E-01	Mir423	0,71	No
ENSMUSG00000065519	8,07E-01	-2,60071452	5,67E-01	4,52E-01	7,41E-01	Mir10a	1,75	No
ENSMUSG00000065520	-2,70E+00	-2,896126596	2,88E+00	8,95E-02	3,79E-01	Mir128-1	0,15	No
ENSMUSG00000065521	-2,63E-02	-3,033641358	2,33E-04	9,88E-01	1,00E+00	Mir296	0,98	No
ENSMUSG00000065523	-1,06E-01	-3,033634182	5,86E-03	9,39E-01	1,00E+00	Mir192	0,93	No
ENSMUSG00000065527	2,14E-01	-2,481311834	4,96E-02	8,24E-01	9,73E-01	Mir93	1,16	No
ENSMUSG00000065529	-2,36E-01	0,432347385	8,31E-01	3,62E-01	6,64E-01	Mir22	0,85	No
ENSMUSG00000065532	9,01E-03	-3,107646452	-5,16E-10	1,00E+00	1,00E+00	Mir187	1,01	No
ENSMUSG00000065534	2,97E-01	-2,601223874	7,76E-02	7,81E-01	9,49E-01	Mir324	1,23	No
ENSMUSG00000065537	-2,10E-01	-2,424811305	5,30E-02	8,18E-01	9,70E-01	Mir132	0,86	No
ENSMUSG00000065541	4,49E-01	-0,384920592	1,84E+00	1,75E-01	5,33E-01	Mir24-2	1,36	No
ENSMUSG00000065543	-4,81E-02	-2,998010904	1,04E-03	9,74E-01	1,00E+00	Mir330	0,97	No
ENSMUSG00000065544	-2,71E+00	-2,963235336	2,92E+00	8,75E-02	3,78E-01	Mir32	0,15	No
ENSMUSG00000065547	-1,35E-01	-0,879702383	1,08E-01	7,43E-01	9,28E-01	Mir199a-1	0,91	No
ENSMUSG00000065548	-1,39E+00	-2,812053682	1,20E+00	2,73E-01	5,69E-01	Mir29c	0,38	No
ENSMUSG00000065551	2,64E+00	-3,033673223	2,69E+00	1,01E-01	3,92E-01	Mir210	6,23	No
ENSMUSG00000065552	-1,16E+00	-2,929324749	5,83E-01	4,45E-01	7,36E-01	Mir302a	0,45	No
ENSMUSG00000065553	1,81E+00	-3,10765737	1,28E+00	2,58E-01	5,50E-01	Mir103-1	3,49	No
ENSMUSG00000065555	-1,68E+00	-3,120604875	1,15E+00	2,83E-01	5,79E-01	Mir219a-1	0,31	No
ENSMUSG00000065562	6,51E-01	-2,740869876	3,51E-01	5,54E-01	8,19E-01	Mir215	1,57	No
ENSMUSG00000065563	-6,64E-02	-2,600678392	5,05E-03	9,43E-01	1,00E+00	Mir103-2	0,96	No
ENSMUSG00000065564	2,36E-01	-2,548233162	7,01E-02	7,91E-01	9,55E-01	Mirlet7b	1,18	No
ENSMUSG00000065565	-9,11E-01	-3,159433134	4,59E-01	4,98E-01	7,83E-01	Mir181a-1	0,53	No
ENSMUSG00000065571	-8,86E-02	-2,963249454	3,57E-03	9,52E-01	1,00E+00	Mir326	0,94	No
ENSMUSG00000065572	3,53E+00	-2,711725021	5,41E+00	2,01E-02	1,51E-01	Mir130b	11,54	No
ENSMUSG00000065573	3,12E+00	-2,8961712	3,97E+00	4,64E-02	2,57E-01	Mir350	8,68	No

ENSMUSG00000065578	-1,22E+00	-3,146113954	6,96E-01	4,04E-01	7,01E-01	Mir181b-2	0,43	No
ENSMUSG00000065579	3,29E-01	-1,079614688	5,80E-01	4,46E-01	7,37E-01	Mir425	1,26	No
ENSMUSG00000065580	9,01E-03	-3,107646452	-5,16E-10	1,00E+00	1,00E+00	Mir15b	1,01	No
ENSMUSG00000065581	-1,74E-01	-2,205804177	5,06E-02	8,22E-01	9,72E-01	Mir194-1	0,89	No
ENSMUSG00000065582	7,43E-01	-2,963261206	3,01E-01	5,83E-01	8,38E-01	Mir194-2	1,67	No
ENSMUSG00000065586	5,97E-03	-3,1461183	-3,40E-10	1,00E+00	1,00E+00	Mir96	1,00	No
ENSMUSG00000065589	-1,03E-01	-2,831985655	7,40E-03	9,31E-01	1,00E+00	Mir301	0,93	No
ENSMUSG00000065590	-7,95E-01	-2,015640706	1,10E+00	2,93E-01	5,89E-01	Mir212	0,58	No
ENSMUSG00000065591	2,64E-02	-0,669227731	4,72E-03	9,45E-01	1,00E+00	Gm22753	1,02	No
ENSMUSG00000065593	5,83E-01	-2,107653441	7,73E-01	3,79E-01	6,80E-01	Mir339	1,50	No
ENSMUSG00000065594	-1,89E+00	-3,1076441	1,42E+00	2,33E-01	5,50E-01	Mir107	0,27	No
ENSMUSG00000065599	5,64E-01	-0,793523432	1,94E+00	1,64E-01	5,15E-01	Mir23b	1,48	No
ENSMUSG00000065600	6,16E-01	-3,03366067	1,51E-01	6,97E-01	9,03E-01	Mir338	1,53	No
ENSMUSG00000065601	1,84E+00	-3,033649193	1,33E+00	2,49E-01	5,50E-01	Mir146	3,58	No
ENSMUSG00000065604	2,06E-02	-3,146103739	-9,26E-10	1,00E+00	1,00E+00	Mir29b-1	1,01	No
ENSMUSG00000065607	1,18E+00	-3,107651911	6,57E-01	4,17E-01	7,12E-01	Mir331	2,27	No
ENSMUSG00000065608	2,64E+00	-3,033673223	2,69E+00	1,01E-01	3,92E-01	Mirlet7c-2	6,23	No
ENSMUSG00000065610	-4,08E-02	-2,963255775	7,59E-04	9,78E-01	1,00E+00	Mir29a	0,97	No
ENSMUSG00000065611	1,34E+00	-0,7696194	1,05E+01	1,21E-03	1,89E-02	Mir23a	2,54	Yes
ENSMUSG00000065619	5,97E-03	-3,1461183	-3,40E-10	1,00E+00	1,00E+00	Mir183	1,00	No
ENSMUSG00000065623	-2,41E-01	-1,92923942	1,15E-01	7,35E-01	9,24E-01	Gm24762	0,85	No
ENSMUSG00000065627	7,53E-01	-2,711659049	3,07E-01	5,79E-01	8,36E-01	Gm24803	1,68	No
ENSMUSG00000065628	-1,63E-01	-1,571851145	8,22E-02	7,74E-01	9,45E-01	Snord33	0,89	No
ENSMUSG00000065634	1,31E+00	-1,929345509	3,05E+00	8,09E-02	3,65E-01	Gm24252	2,48	No
ENSMUSG00000065637	-2,96E-01	0,352105175	1,32E+00	2,51E-01	5,50E-01	Gm26397	0,81	No
ENSMUSG00000065640	-9,05E-02	-1,912584167	1,71E-02	8,96E-01	1,00E+00	Snord1c	0,94	No
ENSMUSG00000065643	-1,75E+00	-2,548171965	3,38E+00	6,58E-02	3,22E-01	Gm25897	0,30	No
ENSMUSG00000065649	1,14E+00	-2,760442601	7,89E-01	3,74E-01	6,75E-01	Snora74a	2,20	No
ENSMUSG00000065651	-7,39E-01	-1,711612729	1,43E+00	2,32E-01	5,50E-01	Gm25973	0,60	No
ENSMUSG00000065657	1,84E+00	-3,033649193	1,33E+00	2,49E-01	5,50E-01	Gm25337	3,58	No
ENSMUSG00000065658	9,01E-03	-3,107646452	-5,16E-10	1,00E+00	1,00E+00	Gm23102	1,01	No
ENSMUSG00000065662	-4,87E-02	-2,896149255	1,54E-03	9,69E-01	1,00E+00	n-R5s161	0,97	No

ENSMUSG00000065663	-2,92E-01	-0,235000795	8,87E-01	3,46E-01	6,47E-01	Gm22579	0,82	No
ENSMUSG00000065669	-6,88E-01	0,22186807	6,25E+00	1,24E-02	1,10E-01	Gm22581	0,62	No
ENSMUSG00000065673	-5,51E-02	-2,832041634	2,04E-03	9,64E-01	1,00E+00	Gm24258	0,96	No
ENSMUSG00000065674	-4,38E-01	-2,655086267	1,96E-01	6,58E-01	8,81E-01	Gm24259	0,74	No
ENSMUSG00000065675	4,44E-01	-2,770627496	1,54E-01	6,95E-01	9,02E-01	Gm24260	1,36	No
ENSMUSG00000065676	-1,27E+00	-1,755656655	4,19E+00	4,08E-02	2,39E-01	Snord42b	0,41	No
ENSMUSG00000065678	3,50E+00	-2,711742211	5,31E+00	2,12E-02	1,57E-01	Gm24261	11,29	No
ENSMUSG00000065680	-1,57E+00	-2,863674799	1,64E+00	2,00E-01	5,50E-01	Snord38a	0,34	No
ENSMUSG00000065683	-3,55E+00	-2,83197716	5,67E+00	1,73E-02	1,37E-01	Gm23731	0,09	No
ENSMUSG00000065685	-1,94E+00	-3,107619243	1,50E+00	2,21E-01	5,50E-01	Gm23732	0,26	No
ENSMUSG00000065686	-7,25E-02	-0,831935731	2,76E-02	8,68E-01	1,00E+00	Snora5c	0,95	No
ENSMUSG00000065687	2,62E+00	-3,033654625	2,66E+00	1,03E-01	3,95E-01	Gm23734	6,16	No
ENSMUSG00000065692	-1,89E+00	-3,1076441	1,42E+00	2,33E-01	5,50E-01	Gm25410	0,27	No
ENSMUSG00000065701	6,78E-01	-2,832060811	2,51E-01	6,17E-01	8,55E-01	Rny1	1,60	No
ENSMUSG00000065715	-2,76E-02	-1,344338795	2,90E-03	9,57E-01	1,00E+00	Snord7	0,98	No
ENSMUSG00000065725	3,86E-01	-0,541941829	1,16E+00	2,82E-01	5,78E-01	Gm26165	1,31	No
ENSMUSG00000065728	6,22E-02	-1,740734279	1,04E-02	9,19E-01	1,00E+00	Gm26175	1,04	No
ENSMUSG00000065732	-1,87E+00	-3,03364178	1,41E+00	2,36E-01	5,50E-01	Gm24492	0,27	No
ENSMUSG00000065734	-7,32E-02	0,195379646	7,31E-02	7,87E-01	9,53E-01	Snord49a	0,95	No
ENSMUSG00000065735	8,31E-02	-1,183727399	3,22E-02	8,58E-01	9,94E-01	Snord80	1,06	No
ENSMUSG00000065738	-3,59E-01	0,439036965	1,83E+00	1,76E-01	5,35E-01	Gm24494	0,78	No
ENSMUSG00000065743	3,14E-02	-3,107617333	-7,69E-10	1,00E+00	1,00E+00	Gm25008	1,02	No
ENSMUSG00000065750	1,23E+00	-2,832024669	9,31E-01	3,35E-01	6,35E-01	Gm23346	2,34	No
ENSMUSG00000065752	3,17E-01	-1,095090673	4,53E-01	5,01E-01	7,86E-01	Gm23344	1,25	No
ENSMUSG00000065765	6,58E-01	-1,879985217	1,15E+00	2,84E-01	5,80E-01	Gm23851	1,58	No
ENSMUSG00000065767	-2,30E-01	-2,600693934	5,76E-02	8,10E-01	9,66E-01	Gm23849	0,85	No
ENSMUSG00000065771	1,82E+00	-3,033661385	1,31E+00	2,52E-01	5,50E-01	n-R5s54	3,54	No
ENSMUSG00000065773	1,81E+00	-3,10765737	1,28E+00	2,58E-01	5,50E-01	Rnu1b6	3,49	No
ENSMUSG00000065778	-1,03E+00	-1,697185198	3,43E+00	6,41E-02	3,17E-01	Gm22154	0,49	No
ENSMUSG00000065782	-4,22E-01	-0,403240587	1,39E+00	2,38E-01	5,50E-01	Gm22680	0,75	No
ENSMUSG00000065788	-2,71E+00	-2,896102977	2,90E+00	8,85E-02	3,79E-01	Gm22682	0,15	No
ENSMUSG00000065789	2,82E-01	-2,378362191	1,23E-01	7,26E-01	9,19E-01	Gm25869	1,22	No

ENSMUSG00000065798	-1,19E-01	-2,770637672	9,55E-03	9,22E-01	1,00E+00	Gm25518	0,92	No
ENSMUSG00000065799	-1,89E+00	-3,1076441	1,42E+00	2,33E-01	5,50E-01	Gm25517	0,27	No
ENSMUSG00000065800	1,82E+00	-3,033661385	1,31E+00	2,52E-01	5,50E-01	Gm23042	3,54	No
ENSMUSG00000065808	-6,19E-01	-1,165644343	1,94E+00	1,63E-01	5,14E-01	Gm23037	0,65	No
ENSMUSG00000065817	-6,48E-01	-2,600633321	4,00E-01	5,27E-01	8,04E-01	Gm24698	0,64	No
ENSMUSG00000065818	3,10E-01	-1,136472695	4,56E-01	5,00E-01	7,85E-01	Snord35a	1,24	No
ENSMUSG00000065819	4,47E-01	-2,711702252	1,56E-01	6,93E-01	9,01E-01	Gm24693	1,36	No
ENSMUSG00000065820	-5,83E-01	-2,770564587	2,67E-01	6,05E-01	8,49E-01	Gm26316	0,67	No
ENSMUSG00000065822	-2,98E-01	-1,785715074	2,59E-01	6,11E-01	8,52E-01	Snord15a	0,81	No
ENSMUSG00000065824	-6,65E-02	-1,289541112	1,76E-02	8,95E-01	1,00E+00	Gm26315	0,95	No
ENSMUSG00000065836	2,62E+00	-2,963293967	2,65E+00	1,04E-01	3,95E-01	Gm23502	6,13	No
ENSMUSG00000065851	-4,01E-02	-2,548210308	1,49E-03	9,69E-01	1,00E+00	Gm22352	0,97	No
ENSMUSG00000065852	-2,65E+00	-3,033633091	2,78E+00	9,55E-02	3,86E-01	Gm22353	0,16	No
ENSMUSG00000065853	9,01E-03	-3,107646452	-5,16E-10	1,00E+00	1,00E+00	Gm22354	1,01	No
ENSMUSG00000065862	5,39E-02	1,788210684	1,13E-01	7,37E-01	9,25E-01	Gm24029	1,04	No
ENSMUSG00000065864	-3,17E+00	-2,896142751	4,24E+00	3,94E-02	2,35E-01	Gm24031	0,11	No
ENSMUSG00000065870	-4,28E-02	-2,740848597	1,26E-03	9,72E-01	1,00E+00	Rnu3a	0,97	No
ENSMUSG00000065873	-7,95E-02	-1,863748942	1,93E-02	8,90E-01	1,00E+00	n-R5s183	0,95	No
ENSMUSG00000065874	-2,68E+00	-2,963200749	2,85E+00	9,14E-02	3,82E-01	Gm25683	0,16	No
ENSMUSG00000065878	1,59E-01	-1,128308819	1,14E-01	7,36E-01	9,24E-01	Snord34	1,12	No
ENSMUSG00000065883	2,33E-01	-1,770652625	1,25E-01	7,23E-01	9,17E-01	Gm22865	1,18	No
ENSMUSG00000065887	-1,30E-02	-0,241720182	1,43E-03	9,70E-01	1,00E+00	n-R5s185	0,99	No
ENSMUSG00000065899	2,48E-01	-0,627704851	4,33E-01	5,11E-01	7,94E-01	Gm24523	1,19	No
ENSMUSG00000065904	-2,91E-01	-0,963211801	4,06E-01	5,24E-01	8,01E-01	Gm26109	0,82	No
ENSMUSG00000065905	4,24E-02	-3,033592245	-1,17E-09	1,00E+00	1,00E+00	Gm26110	1,03	No
ENSMUSG00000065911	-9,46E-01	-1,448671775	3,10E+00	7,83E-02	3,57E-01	Gm24447	0,52	No
ENSMUSG00000065913	1,34E+00	-2,497645752	1,83E+00	1,76E-01	5,34E-01	Gm24448	2,53	No
ENSMUSG00000065919	-8,57E-01	-2,963247565	4,04E-01	5,25E-01	8,02E-01	Gm24449	0,55	No
ENSMUSG00000065926	5,95E-01	-2,448643382	3,95E-01	5,30E-01	8,05E-01	Gm22798	1,51	No
ENSMUSG00000065934	-1,93E+00	-2,963244023	1,47E+00	2,25E-01	5,50E-01	Gm25630	0,26	No
ENSMUSG00000065936	-1,67E-02	-2,963250293	1,29E-04	9,91E-01	1,00E+00	Gm25628	0,99	No
ENSMUSG00000065939	-9,60E-01	-0,816211653	4,79E+00	2,85E-02	1,92E-01	Snora2b	0,51	No

ENSMUSG00000065944	-3,02E-01	-2,268053088	1,36E-01	7,12E-01	9,11E-01	Rnu2-10	0,81	No
ENSMUSG00000065952	-2,26E-01	5,491662926	1,07E+01	1,08E-03	1,72E-02	C330021F23Rik	0,86	Yes
ENSMUSG00000065954	9,90E-02	7,73273441	2,84E+00	9,18E-02	3,82E-01	Tacc1	1,07	No
ENSMUSG00000065968	-7,38E-02	-3,033659033	2,66E-03	9,59E-01	1,00E+00	Ifitm7	0,95	No
ENSMUSG00000065979	3,62E-02	5,580606068	2,79E-01	5,97E-01	8,45E-01	Cpped1	1,03	No
ENSMUSG00000065987	5,75E-01	-1,832018657	7,52E-01	3,86E-01	6,87E-01	Cd209b	1,49	No
ENSMUSG00000065990	-2,70E-02	5,730929421	1,61E-01	6,88E-01	8,98E-01	Aurkaip1	0,98	No
ENSMUSG00000065999	-8,59E-01	-0,980369139	4,38E+00	3,65E-02	2,27E-01	Zfp985	0,55	No
ENSMUSG00000066000	3,23E-01	1,269699989	2,98E+00	8,43E-02	3,72E-01	Zfp979	1,25	No
ENSMUSG00000066007	3,39E-01	-2,355620086	1,64E-01	6,85E-01	8,97E-01	Zfp600	1,26	No
ENSMUSG00000066009	-3,76E-01	-0,57416539	1,11E+00	2,91E-01	5,87E-01	Zfp987	0,77	No
ENSMUSG00000066026	1,97E-01	5,67731001	8,44E+00	3,66E-03	4,44E-02	Dhrs3	1,15	Yes
ENSMUSG00000066027	1,87E+00	-3,107639691	1,38E+00	2,40E-01	5,50E-01	Gm10436	3,67	No
ENSMUSG00000066036	-3,13E-02	8,210621907	2,92E-01	5,89E-01	8,41E-01	Ubr4	0,98	No
ENSMUSG00000066037	-9,81E-02	5,88126472	2,19E+00	1,38E-01	4,70E-01	Hnrnpr	0,93	No
ENSMUSG00000066042	1,38E-01	1,604474847	6,24E-01	4,29E-01	7,23E-01	Med18	1,10	No
ENSMUSG00000066043	1,02E-01	5,525017338	2,18E+00	1,40E-01	4,73E-01	Phactr4	1,07	No
ENSMUSG00000066057	2,09E-01	1,462748546	1,45E+00	2,29E-01	5,50E-01	Gm1976	1,16	No
ENSMUSG00000066058	-1,94E+00	-3,107619243	1,50E+00	2,21E-01	5,50E-01	Cldn19	0,26	No
ENSMUSG00000066060	1,61E+00	-1,627729446	6,19E+00	1,29E-02	1,12E-01	Gm12866	3,06	No
ENSMUSG00000066068	-1,59E-01	5,692332982	5,55E+00	1,85E-02	1,44E-01	Gm13611	0,90	No
ENSMUSG00000066071	4,50E-01	2,399188382	9,61E+00	1,93E-03	2,72E-02	Cyp4a12a	1,37	Yes
ENSMUSG00000066072	-6,39E-02	-2,831998678	2,77E-03	9,58E-01	1,00E+00	Cyp4a10	0,96	No
ENSMUSG00000066107	-1,13E-02	0,4059689	2,09E-03	9,64E-01	1,00E+00	Gm12666	0,99	No
ENSMUSG00000066108	4,46E+00	-2,268168792	1,07E+01	1,05E-03	1,68E-02	Muc5b	21,94	Yes
ENSMUSG00000066113	-1,89E+00	-3,1076441	1,42E+00	2,33E-01	5,50E-01	Adamtsl1	0,27	No
ENSMUSG00000066116	-2,76E-01	6,395450617	1,91E+01	1,24E-05	4,07E-04	Gm10154	0,83	Yes
ENSMUSG00000066129	-1,01E+00	-0,895974874	6,24E+00	1,25E-02	1,10E-01	Kndc1	0,50	No
ENSMUSG00000066141	-1,92E+00	-3,0336536	1,46E+00	2,27E-01	5,50E-01	Gm11232	0,26	No
ENSMUSG00000066148	3,33E-02	4,400204488	1,67E-01	6,83E-01	8,95E-01	Prpf4	1,02	No
ENSMUSG00000066149	4,47E-02	3,880681163	2,44E-01	6,21E-01	8,57E-01	Cdc26	1,03	No
ENSMUSG00000066150	2,02E-01	6,375061937	1,01E+01	1,50E-03	2,23E-02	Slc31a1	1,15	Yes

ENSMUSG00000066151	8,88E-02	6,847720153	2,10E+00	1,47E-01	4,86E-01	Fkbp15	1,06	No
ENSMUSG00000066152	2,39E-01	5,980580959	1,33E+01	2,62E-04	5,45E-03	Slc31a2	1,18	Yes
ENSMUSG00000066170	6,03E-01	-2,070265394	7,36E-01	3,91E-01	6,91E-01	E230001N04Rik	1,52	No
ENSMUSG00000066175	-3,27E-01	-0,200604175	1,04E+00	3,07E-01	6,05E-01	2510046G10Rik	0,80	No
ENSMUSG00000066176	-1,44E+00	-2,424798597	2,51E+00	1,13E-01	4,15E-01	Gm12511	0,37	No
ENSMUSG00000066178	-1,45E+00	-2,99801717	9,78E-01	3,23E-01	6,23E-01	6030445D17Rik	0,37	No
ENSMUSG00000066180	-3,25E-01	4,453496618	1,60E+01	6,41E-05	1,68E-03	Gm10155	0,80	Yes
ENSMUSG00000066189	-3,83E-02	-3,107651936	-7,91E-11	1,00E+00	1,00E+00	Cacng3	0,97	No
ENSMUSG00000066191	2,34E-02	0,295679046	7,99E-03	9,29E-01	1,00E+00	Anks6	1,02	No
ENSMUSG00000066196	-4,41E-01	-2,401306736	1,99E-01	6,55E-01	8,79E-01	Spag8	0,74	No
ENSMUSG00000066224	9,01E-03	-3,107646452	-5,16E-10	1,00E+00	1,00E+00	Arid3c	1,01	No
ENSMUSG00000066232	9,69E-02	6,70667224	2,47E+00	1,16E-01	4,22E-01	Ipo7	1,07	No
ENSMUSG00000066233	-1,77E-02	1,83449165	1,20E-02	9,13E-01	1,00E+00	Tmem42	0,99	No
ENSMUSG00000066235	-1,13E-01	0,952688468	2,96E-01	5,86E-01	8,39E-01	Pomgnt2	0,92	No
ENSMUSG00000066245	9,67E-03	5,325041871	1,86E-02	8,91E-01	1,00E+00	Gm10156	1,01	No
ENSMUSG00000066258	-6,90E-02	4,72795607	8,09E-01	3,69E-01	6,70E-01	Trim12a	0,95	No
ENSMUSG00000066262	1,85E+00	-3,107657832	1,35E+00	2,46E-01	5,50E-01	Olfr640	3,61	No
ENSMUSG00000066263	-2,70E+00	-3,033614577	2,89E+00	8,93E-02	3,79E-01	Olfr639	0,15	No
ENSMUSG00000066269	-1,89E+00	-3,1076441	1,42E+00	2,33E-01	5,50E-01	Olfr575	0,27	No
ENSMUSG00000066270	-1,42E-01	5,307116505	4,00E+00	4,56E-02	2,55E-01	Gm10157	0,91	No
ENSMUSG00000066278	3,17E-02	4,594057073	1,59E-01	6,90E-01	8,99E-01	Vps37b	1,02	No
ENSMUSG00000066279	3,80E-02	-3,070130373	-9,83E-10	1,00E+00	1,00E+00	Chrna10	1,03	No
ENSMUSG00000066306	-2,80E-02	7,730021244	2,28E-01	6,33E-01	8,65E-01	Numa1	0,98	No
ENSMUSG00000066315	-1,07E-01	8,418464037	3,46E+00	6,29E-02	3,13E-01	Gm12918	0,93	No
ENSMUSG00000066324	3,13E-02	6,267239521	2,40E-01	6,24E-01	8,59E-01	Impad1	1,02	No
ENSMUSG00000066357	-2,18E-01	5,400066917	9,87E+00	1,68E-03	2,43E-02	Wdr6	0,86	Yes
ENSMUSG00000066361	-3,21E+00	-2,896117057	4,33E+00	3,75E-02	2,30E-01	Serpina3c	0,11	No
ENSMUSG00000066362	-1,67E-01	6,759318934	7,37E+00	6,64E-03	6,91E-02	Rps13-ps1	0,89	No
ENSMUSG00000066363	-1,53E+00	-1,574183702	7,02E+00	8,07E-03	7,95E-02	Serpina3f	0,35	No
ENSMUSG00000066364	-3,16E+00	-2,963246013	4,20E+00	4,04E-02	2,38E-01	Serpina3b	0,11	No
ENSMUSG00000066378	1,29E-01	-2,185672321	2,89E-02	8,65E-01	9,98E-01	Gm10160	1,09	No
ENSMUSG00000066382	-1,89E+00	-3,1076441	1,42E+00	2,33E-01	5,50E-01	Iqcf5	0,27	No

ENSMUSG00000066389	-2,74E-01	-0,205733382	7,65E-01	3,82E-01	6,83E-01	Rpl31-ps1	0,83	No
ENSMUSG00000066392	-1,94E+00	-3,107619243	1,50E+00	2,21E-01	5,50E-01	Nrxn3	0,26	No
ENSMUSG00000066406	-1,16E-01	8,786914957	4,11E+00	4,26E-02	2,45E-01	Akap13	0,92	No
ENSMUSG00000066407	-2,37E-01	6,369925071	1,40E+01	1,82E-04	4,02E-03	Gm10263	0,85	Yes
ENSMUSG00000066415	1,52E-01	5,996313094	5,39E+00	2,03E-02	1,53E-01	Msl2	1,11	No
ENSMUSG00000066438	9,01E-03	-3,107646452	-5,16E-10	1,00E+00	1,00E+00	Plekhd1	1,01	No
ENSMUSG00000066440	8,31E-02	6,680917675	1,80E+00	1,79E-01	5,40E-01	Zfyve26	1,06	No
ENSMUSG00000066441	7,05E-02	5,283515492	9,75E-01	3,23E-01	6,24E-01	Rdh11	1,05	No
ENSMUSG00000066442	-2,24E-01	3,985292283	6,40E+00	1,14E-02	1,03E-01	Mthfs	0,86	No
ENSMUSG00000066443	-2,27E-01	6,052655469	1,22E+01	4,83E-04	8,92E-03	Gm10163	0,85	Yes
ENSMUSG00000066456	-1,07E+00	3,250500871	1,04E+02	1,97E-24	6,65E-22	Hmgn3	0,48	Yes
ENSMUSG00000066475	-2,90E-01	2,032790735	3,92E+00	4,77E-02	2,62E-01	Gm10268	0,82	No
ENSMUSG00000066477	-1,94E+00	-3,107619243	1,50E+00	2,21E-01	5,50E-01	Gm16551	0,26	No
ENSMUSG00000066478	1,79E+00	-3,033666867	1,28E+00	2,58E-01	5,50E-01	Gm5745	3,46	No
ENSMUSG00000066487	3,77E-02	5,748185959	3,17E-01	5,74E-01	8,33E-01	Gm5786	1,03	No
ENSMUSG00000066491	-8,58E-02	5,985249392	1,71E+00	1,91E-01	5,50E-01	Gm6265	0,94	No
ENSMUSG00000066510	2,26E-01	-0,800799738	2,38E-01	6,26E-01	8,60E-01	Ankdd1a	1,17	No
ENSMUSG00000066513	-6,79E-02	-2,548268043	4,19E-03	9,48E-01	1,00E+00	Klk1b4	0,95	No
ENSMUSG00000066515	7,31E-01	-2,963279337	2,91E-01	5,90E-01	8,41E-01	Klk1b3	1,66	No
ENSMUSG00000066516	9,08E-02	-1,929238831	1,92E-02	8,90E-01	1,00E+00	Klk1b21	1,06	No
ENSMUSG00000066537	9,94E-01	-2,80099769	5,76E-01	4,48E-01	7,38E-01	Vmn2r57	1,99	No
ENSMUSG00000066538	-3,37E-02	4,605415029	1,82E-01	6,69E-01	8,88E-01	Gm6254	0,98	No
ENSMUSG00000066543	-3,14E-01	6,334240186	2,45E+01	7,54E-07	3,47E-05	Rpl17-ps9	0,80	Yes
ENSMUSG00000066551	3,69E-02	6,352117019	3,38E-01	5,61E-01	8,24E-01	Hmgb1	1,03	No
ENSMUSG00000066553	-1,47E-01	5,816683632	4,87E+00	2,73E-02	1,86E-01	Gm6969	0,90	No
ENSMUSG00000066554	-5,04E-02	6,839489674	6,77E-01	4,11E-01	7,07E-01	Gm10167	0,97	No
ENSMUSG00000066568	5,63E-02	6,758741566	8,34E-01	3,61E-01	6,62E-01	Lsm14a	1,04	No
ENSMUSG00000066571	1,60E-01	5,165493408	4,85E+00	2,76E-02	1,87E-01	4931406P16Rik	1,12	No
ENSMUSG00000066592	-1,17E-01	2,274118366	7,48E-01	3,87E-01	6,87E-01	Gm7589	0,92	No
ENSMUSG00000066595	9,22E-02	5,328857985	1,71E+00	1,91E-01	5,50E-01	Mfsd7b	1,07	No
ENSMUSG00000066607	-1,38E+00	-1,574036049	5,48E+00	1,92E-02	1,47E-01	6030419C18Rik	0,38	No
ENSMUSG00000066613	4,32E-02	3,069968397	1,53E-01	6,96E-01	9,03E-01	Zfp932	1,03	No

ENSMUSG00000066621	-1,02E-01	7,004753925	2,81E+00	9,36E-02	3,83E-01	Tecpr1	0,93	No
ENSMUSG00000066626	-4,88E-01	1,661739914	8,69E+00	3,20E-03	4,02E-02	Gm5121	0,71	Yes
ENSMUSG00000066629	-3,39E-01	3,361414542	1,10E+01	9,19E-04	1,51E-02	Rpl36-ps3	0,79	Yes
ENSMUSG00000066632	1,40E-01	8,385744631	5,88E+00	1,53E-02	1,28E-01	Pgk1-rs7	1,10	No
ENSMUSG00000066637	1,31E-01	1,086843544	4,13E-01	5,20E-01	7,99E-01	Ttc32	1,10	No
ENSMUSG00000066640	-9,27E-02	4,955633658	1,57E+00	2,10E-01	5,50E-01	Fbxl18	0,94	No
ENSMUSG00000066643	-1,29E-03	0,948146293	3,76E-05	9,95E-01	1,00E+00	Wdr35	1,00	No
ENSMUSG00000066647	5,27E-01	1,649315628	1,04E+01	1,23E-03	1,92E-02	Gm5113	1,44	Yes
ENSMUSG00000066652	-3,13E-01	-2,740768279	7,17E-02	7,89E-01	9,54E-01	Lefty2	0,80	No
ENSMUSG00000066671	-3,83E-02	-3,107651936	-7,91E-11	1,00E+00	1,00E+00	Olfr220	0,97	No
ENSMUSG00000066672	2,85E-01	-2,54820566	8,25E-02	7,74E-01	9,44E-01	Olfr417	1,22	No
ENSMUSG00000066677	-3,33E-01	-0,627605077	8,48E-01	3,57E-01	6,58E-01	Ifi208	0,79	No
ENSMUSG00000066682	-2,79E-01	4,472098132	1,18E+01	5,90E-04	1,05E-02	Pilrb2	0,82	Yes
ENSMUSG00000066684	-3,00E-01	4,926649941	1,60E+01	6,22E-05	1,63E-03	Pilrb1	0,81	Yes
ENSMUSG00000066687	-4,78E-01	-1,088742076	1,11E+00	2,92E-01	5,87E-01	Zbtb16	0,72	No
ENSMUSG00000066693	3,24E-01	-1,561217675	3,65E-01	5,46E-01	8,14E-01	Gm7493	1,25	No
ENSMUSG00000066705	-3,68E-01	-2,35557	1,70E-01	6,80E-01	8,93E-01	Fxyd6	0,78	No
ENSMUSG00000066714	1,81E+00	-3,10765737	1,28E+00	2,58E-01	5,50E-01	Gm8062	3,49	No
ENSMUSG00000066720	-1,96E-01	-1,448702888	1,22E-01	7,27E-01	9,19E-01	Cldn9	0,87	No
ENSMUSG00000066721	7,05E-01	-2,832012839	2,71E-01	6,03E-01	8,48E-01	Zfp575	1,63	No
ENSMUSG00000066724	-1,96E-02	6,283539376	9,41E-02	7,59E-01	9,36E-01	Gm10175	0,99	No
ENSMUSG00000066735	1,03E-01	5,535784462	2,26E+00	1,33E-01	4,58E-01	Vkorc1l1	1,07	No
ENSMUSG00000066749	-1,89E+00	-3,1076441	1,42E+00	2,33E-01	5,50E-01	Olfr877	0,27	No
ENSMUSG00000066752	-2,80E-02	-2,963263871	3,49E-04	9,85E-01	1,00E+00	Gm10176	0,98	No
ENSMUSG00000066755	-1,38E-01	1,079104368	4,82E-01	4,88E-01	7,75E-01	Tnfsf18	0,91	No
ENSMUSG00000066760	-2,66E+00	-2,896093238	2,81E+00	9,35E-02	3,83E-01	Psg16	0,16	No
ENSMUSG00000066771	-3,83E-02	-3,107651936	-7,91E-11	1,00E+00	1,00E+00	Gm5614	0,97	No
ENSMUSG00000066798	-2,90E-02	4,350434122	1,22E-01	7,27E-01	9,19E-01	Zbtb6	0,98	No
ENSMUSG00000066800	2,14E-01	6,586534041	1,17E+01	6,30E-04	1,11E-02	Rnasel	1,16	Yes
ENSMUSG00000066807	1,07E-01	3,340736367	1,06E+00	3,03E-01	5,99E-01	Gm10179	1,08	No
ENSMUSG00000066809	-1,78E-01	4,980943822	5,65E+00	1,75E-02	1,38E-01	Gm10180	0,88	No
ENSMUSG00000066829	-8,05E-03	3,979094547	8,29E-03	9,27E-01	1,00E+00	Zfp810	0,99	No

ENSMUSG00000066838	6,39E-03	1,750239416	1,59E-03	9,68E-01	1,00E+00	Zfp772	1,00	No
ENSMUSG00000066839	-1,01E-02	3,628794819	1,06E-02	9,18E-01	1,00E+00	Ecsit	0,99	No
ENSMUSG00000066842	-8,55E-01	-2,831988613	4,01E-01	5,27E-01	8,03E-01	Hmcn1	0,55	No
ENSMUSG00000066861	-6,45E-02	4,173404472	5,72E-01	4,49E-01	7,40E-01	Oas1g	0,96	No
ENSMUSG00000066867	1,87E+00	-3,107639691	1,38E+00	2,40E-01	5,50E-01	Oas1e	3,67	No
ENSMUSG00000066877	-5,58E-01	4,614328997	5,02E+01	1,41E-12	1,70E-10	Nck2	0,68	Yes
ENSMUSG00000066878	1,69E-01	6,610648414	7,39E+00	6,54E-03	6,83E-02	Gm10184	1,12	No
ENSMUSG00000066880	7,82E-02	3,540922297	6,37E-01	4,25E-01	7,19E-01	Zfp617	1,06	No
ENSMUSG00000066892	-1,51E-01	4,455541611	3,49E+00	6,17E-02	3,09E-01	Fbxl12	0,90	No
ENSMUSG00000066894	-5,92E-01	3,681160001	3,99E+01	2,71E-10	2,40E-08	Vsig10	0,66	Yes
ENSMUSG00000066896	1,87E+00	-3,033632735	1,36E+00	2,43E-01	5,50E-01	Olfr18	3,66	No
ENSMUSG00000066900	-1,46E-01	6,161696717	5,16E+00	2,31E-02	1,66E-01	Suds3	0,90	No
ENSMUSG00000066902	-2,26E-01	5,491662926	1,07E+01	1,08E-03	1,72E-02	Rps23-ps2	0,86	Yes
ENSMUSG00000066912	9,01E-03	-3,107646452	-5,16E-10	1,00E+00	1,00E+00	Olfr833-ps1	1,01	No
ENSMUSG00000066938	1,39E+00	-2,998019645	8,86E-01	3,47E-01	6,47E-01	Gm10190	2,61	No
ENSMUSG00000066952	9,42E-02	5,22020047	1,72E+00	1,90E-01	5,50E-01	Myo1h	1,07	No
ENSMUSG00000066958	-9,43E-01	-2,548210112	7,79E-01	3,78E-01	6,78E-01	Gm16391	0,52	No
ENSMUSG00000066975	1,07E-01	-1,831986194	2,51E-02	8,74E-01	1,00E+00	Cryba4	1,08	No
ENSMUSG00000066979	2,45E-02	6,16751089	1,44E-01	7,04E-01	9,06E-01	Bub3	1,02	No
ENSMUSG00000066983	-5,88E-02	-3,033652696	1,62E-03	9,68E-01	1,00E+00	Gm16519	0,96	No
ENSMUSG00000067001	-7,04E-01	-2,497500393	5,54E-01	4,57E-01	7,46E-01	Serpinb7	0,61	No
ENSMUSG00000067017	1,66E-03	7,755789411	8,02E-04	9,77E-01	1,00E+00	Gm3608	1,00	No
ENSMUSG00000067038	-1,24E-01	7,857601971	4,48E+00	3,43E-02	2,18E-01	Rps12-ps3	0,92	No
ENSMUSG00000067049	1,56E-01	-1,627547145	7,32E-02	7,87E-01	9,53E-01	Unc93a	1,11	No
ENSMUSG00000067058	-2,86E-01	6,94474124	2,22E+01	2,49E-06	9,81E-05	Rps15a-ps5	0,82	Yes
ENSMUSG00000067063	1,81E+00	-3,10765737	1,28E+00	2,58E-01	5,50E-01	Gm6990	3,49	No
ENSMUSG00000067071	8,06E-02	4,940913575	1,18E+00	2,78E-01	5,75E-01	Hes6	1,06	No
ENSMUSG00000067085	-8,11E-02	1,644722846	2,32E-01	6,30E-01	8,62E-01	Gm10197	0,95	No
ENSMUSG00000067106	9,35E-02	5,649676699	1,88E+00	1,70E-01	5,26E-01	Gm5529	1,07	No
ENSMUSG00000067121	-1,64E-01	5,849707869	6,11E+00	1,34E-02	1,16E-01	Gm7027	0,89	No
ENSMUSG00000067144	3,14E-02	-3,107617333	-7,69E-10	1,00E+00	1,00E+00	Slc22a7	1,02	No
ENSMUSG00000067148	-4,61E-02	4,253497619	3,02E-01	5,82E-01	8,38E-01	Polr1c	0,97	No

ENSMUSG00000067149	-5,07E-01	-1,929354894	6,22E-01	4,30E-01	7,24E-01	Jchain	0,70	No
ENSMUSG00000067150	2,14E-01	4,961220936	8,27E+00	4,03E-03	4,75E-02	Xpo5	1,16	Yes
ENSMUSG00000067156	1,85E+00	-3,107657832	1,35E+00	2,46E-01	5,50E-01	Gm7337	3,61	No
ENSMUSG00000067158	2,50E-01	-0,847863632	3,27E-01	5,68E-01	8,29E-01	Col4a4	1,19	No
ENSMUSG00000067161	4,84E-02	7,029325024	6,35E-01	4,25E-01	7,20E-01	Gm5560	1,03	No
ENSMUSG00000067186	-5,88E-02	-3,033652696	1,62E-03	9,68E-01	1,00E+00	Olfr132	0,96	No
ENSMUSG00000067189	9,40E-02	1,569299653	2,96E-01	5,86E-01	8,39E-01	Gm7335	1,07	No
ENSMUSG00000067199	1,33E-01	2,005940885	7,94E-01	3,73E-01	6,74E-01	Frat1	1,10	No
ENSMUSG00000067203	-2,00E-01	3,36792696	3,95E+00	4,69E-02	2,59E-01	H2-K2	0,87	No
ENSMUSG00000067206	6,86E-02	-0,831885979	2,63E-02	8,71E-01	1,00E+00	Lrrc66	1,05	No
ENSMUSG00000067212	-2,55E-01	7,93293569	1,92E+01	1,21E-05	3,97E-04	H2-T23	0,84	Yes
ENSMUSG00000067219	-3,66E-01	2,026697129	6,69E+00	9,72E-03	9,14E-02	Nipal1	0,78	No
ENSMUSG00000067224	-1,13E-01	-0,367041043	1,17E-01	7,32E-01	9,22E-01	Gm3695	0,92	No
ENSMUSG00000067235	-4,46E-01	1,410894713	6,44E+00	1,12E-02	1,01E-01	H2-Q10	0,73	No
ENSMUSG00000067274	-1,72E-01	9,017943648	9,17E+00	2,46E-03	3,29E-02	Rplp0	0,89	Yes
ENSMUSG00000067279	2,57E+00	-3,0336723	2,56E+00	1,10E-01	4,06E-01	Ppp1r3c	5,96	No
ENSMUSG00000067288	-2,42E-01	3,112207408	5,02E+00	2,50E-02	1,75E-01	Rps28	0,85	No
ENSMUSG00000067297	4,27E-01	-1,770551402	3,97E-01	5,29E-01	8,04E-01	Ifit1bl2	1,34	No
ENSMUSG00000067321	-3,26E-02	3,062445109	8,50E-02	7,71E-01	9,42E-01	Gm7931	0,98	No
ENSMUSG00000067336	1,12E-01	6,340016156	3,12E+00	7,71E-02	3,54E-01	Bmpr2	1,08	No
ENSMUSG00000067338	7,31E-01	-2,963267542	2,91E-01	5,90E-01	8,41E-01	Tuba3b	1,66	No
ENSMUSG00000067341	-4,26E-01	4,032680885	2,43E+01	8,36E-07	3,81E-05	H2-Eb2	0,74	Yes
ENSMUSG00000067344	-1,43E-01	7,281114431	5,69E+00	1,70E-02	1,36E-01	Rps25-ps1	0,91	No
ENSMUSG00000067351	-3,94E-01	1,805405462	6,00E+00	1,43E-02	1,22E-01	Rps15a-ps2	0,76	No
ENSMUSG00000067352	-2,70E+00	-2,96323619	2,89E+00	8,89E-02	3,79E-01	Gm14149	0,15	No
ENSMUSG00000067356	-3,18E-02	-0,683053837	7,42E-03	9,31E-01	1,00E+00	B430203G13Rik	0,98	No
ENSMUSG00000067365	-1,45E-01	5,250411219	4,13E+00	4,21E-02	2,44E-01	Tmem128	0,90	No
ENSMUSG00000067367	1,45E-01	3,928758544	2,59E+00	1,08E-01	4,02E-01	Lyar	1,11	No
ENSMUSG00000067370	1,03E-01	2,73848757	7,31E-01	3,93E-01	6,93E-01	B3galt4	1,07	No
ENSMUSG00000067389	1,84E-01	3,010795413	2,59E+00	1,08E-01	4,02E-01	Gm17080	1,14	No
ENSMUSG00000067399	-4,04E-01	0,40977336	2,78E+00	9,57E-02	3,86E-01	Trim43c	0,76	No
ENSMUSG00000067424	-2,02E-01	1,890190966	1,83E+00	1,77E-01	5,36E-01	Zfp563	0,87	No

ENSMUSG00000067430	2,66E-01	1,388794783	2,19E+00	1,39E-01	4,70E-01	Zfp763	1,20	No
ENSMUSG00000067455	1,32E+00	-1,497665348	5,11E+00	2,38E-02	1,69E-01	Hist1h4j	2,50	No
ENSMUSG00000067522	3,10E+00	-2,896176679	3,91E+00	4,79E-02	2,63E-01	Olfr76	8,56	No
ENSMUSG00000067525	-1,94E+00	-3,107619243	1,50E+00	2,21E-01	5,50E-01	Olfr1427	0,26	No
ENSMUSG00000067528	-7,94E-01	-2,896102912	3,44E-01	5,57E-01	8,21E-01	Olfr1424	0,58	No
ENSMUSG00000067529	-1,96E-02	-2,600667835	3,82E-04	9,84E-01	1,00E+00	Olfr1423	0,99	No
ENSMUSG00000067547	-1,04E-02	5,438354673	2,23E-02	8,81E-01	1,00E+00	Gm7666	0,99	No
ENSMUSG00000067571	-2,71E+00	-3,033620915	2,92E+00	8,75E-02	3,78E-01	Ms4a15	0,15	No
ENSMUSG00000067575	-2,25E-01	5,974471063	1,18E+01	6,08E-04	1,08E-02	Rpl35a-ps3	0,86	Yes
ENSMUSG00000067577	-3,33E-01	-0,800778477	7,59E-01	3,84E-01	6,85E-01	A430093F15Rik	0,79	No
ENSMUSG00000067586	-4,68E-01	4,897151521	3,86E+01	5,08E-10	4,35E-08	S1pr3	0,72	Yes
ENSMUSG00000067591	5,27E-01	1,385568298	7,75E+00	5,37E-03	5,89E-02	Klra3	1,44	No
ENSMUSG00000067594	1,82E+00	-2,963253027	1,31E+00	2,52E-01	5,50E-01	Krt77	3,53	No
ENSMUSG00000067608	1,93E-01	7,343238339	1,05E+01	1,20E-03	1,88E-02	Pcna-ps2	1,14	Yes
ENSMUSG00000067610	1,28E+00	-0,401615651	1,50E+01	1,07E-04	2,58E-03	Klri1	2,43	Yes
ENSMUSG00000067614	-1,94E+00	-3,107619243	1,50E+00	2,21E-01	5,50E-01	Krt86	0,26	No
ENSMUSG00000067615	6,96E-01	-2,832055333	2,64E-01	6,07E-01	8,50E-01	Krt81	1,62	No
ENSMUSG00000067629	2,22E-01	-0,289524439	4,67E-01	4,94E-01	7,80E-01	Syngap1	1,17	No
ENSMUSG00000067642	1,04E-01	-0,098152348	1,22E-01	7,27E-01	9,19E-01	Adgrf3	1,07	No
ENSMUSG00000067653	9,87E-02	1,825162973	3,91E-01	5,32E-01	8,05E-01	Ankrd23	1,07	No
ENSMUSG00000067704	1,22E+00	-2,77065262	9,15E-01	3,39E-01	6,39E-01	Wfdc13	2,32	No
ENSMUSG00000067713	-4,60E-02	5,457575439	4,36E-01	5,09E-01	7,92E-01	Prkag1	0,97	No
ENSMUSG00000067714	5,54E-01	2,841094408	2,33E+01	1,41E-06	6,02E-05	Lpar5	1,47	Yes
ENSMUSG00000067719	-8,27E-02	7,060967634	1,87E+00	1,72E-01	5,28E-01	Gm10221	0,94	No
ENSMUSG00000067722	1,13E-03	4,087011108	1,69E-04	9,90E-01	1,00E+00	BC003965	1,00	No
ENSMUSG00000067724	5,81E-01	-2,268120066	5,73E-01	4,49E-01	7,39E-01	Gbx1	1,50	No
ENSMUSG00000067736	7,98E-02	8,285143663	1,91E+00	1,67E-01	5,20E-01	Gm10222	1,06	No
ENSMUSG00000067750	-1,03E+00	-1,800969748	2,71E+00	9,95E-02	3,92E-01	Khdc1a	0,49	No
ENSMUSG00000067767	1,83E+00	-1,655092009	7,02E+00	8,05E-03	7,94E-02	Clec4b2	3,56	No
ENSMUSG00000067780	-2,71E+00	-2,832006336	2,90E+00	8,84E-02	3,79E-01	Pi15	0,15	No
ENSMUSG00000067786	2,94E-01	1,867235782	3,19E+00	7,43E-02	3,47E-01	Nnat	1,23	No
ENSMUSG00000067787	4,05E-02	5,405878936	3,36E-01	5,62E-01	8,25E-01	Blcap	1,03	No

ENSMUSG00000067813	1,85E+00	-3,107657832	1,35E+00	2,46E-01	5,50E-01	Xkr9	3,61	No
ENSMUSG00000067818	-3,83E-02	-3,107651936	-7,91E-11	1,00E+00	1,00E+00	Myl9	0,97	No
ENSMUSG00000067825	-9,88E-02	4,409611625	1,51E+00	2,19E-01	5,50E-01	Pex26	0,93	No
ENSMUSG00000067847	-1,26E-01	4,044385128	2,03E+00	1,54E-01	4,97E-01	Romo1	0,92	No
ENSMUSG00000067851	-9,02E-02	7,228674423	2,27E+00	1,32E-01	4,57E-01	Arfgef1	0,94	No
ENSMUSG00000067869	-1,05E-01	5,518221296	2,33E+00	1,27E-01	4,47E-01	Tcea1-ps1	0,93	No
ENSMUSG00000067870	-2,12E-01	8,149914916	1,34E+01	2,47E-04	5,19E-03	Rpl31-ps8	0,86	Yes
ENSMUSG00000067872	5,41E-01	-2,424882313	2,77E-01	5,99E-01	8,46E-01	Ccdc87	1,45	No
ENSMUSG00000067879	-2,57E-01	-2,52270685	7,17E-02	7,89E-01	9,54E-01	3110035E14Rik	0,84	No
ENSMUSG00000067882	2,61E+00	-3,033672762	2,63E+00	1,05E-01	3,97E-01	Casp16-ps	6,10	No
ENSMUSG00000067889	-4,66E-02	1,335884385	6,66E-02	7,96E-01	9,58E-01	Sptbn2	0,97	No
ENSMUSG00000067916	1,15E-01	5,820576408	2,97E+00	8,47E-02	3,73E-01	Zfp991	1,08	No
ENSMUSG00000067919	-1,74E+00	-2,548120733	2,10E+00	1,47E-01	4,86E-01	Rex2	0,30	No
ENSMUSG00000067928	8,60E-02	1,472019091	2,51E-01	6,16E-01	8,55E-01	Zfp760	1,06	No
ENSMUSG00000067929	2,06E-01	0,556956471	7,32E-01	3,92E-01	6,93E-01	Gm10226	1,15	No
ENSMUSG00000067931	1,17E-01	4,114684675	1,79E+00	1,81E-01	5,42E-01	Zfp948	1,08	No
ENSMUSG00000067941	-1,06E-01	-3,033634182	5,86E-03	9,39E-01	1,00E+00	Gm7168	0,93	No
ENSMUSG00000067942	-1,72E-01	3,504491614	3,26E+00	7,10E-02	3,37E-01	Zfp160	0,89	No
ENSMUSG00000067949	-1,94E+00	-3,107619243	1,50E+00	2,21E-01	5,50E-01	Vmn1r-ps144	0,26	No
ENSMUSG00000067995	-5,57E-02	4,684327376	5,08E-01	4,76E-01	7,64E-01	Gtf2f2	0,96	No
ENSMUSG00000067996	1,56E+00	-2,711767707	1,67E+00	1,96E-01	5,50E-01	Bpifb9b	2,95	No
ENSMUSG00000067998	-1,94E+00	-3,107619243	1,50E+00	2,21E-01	5,50E-01	Bpifb9a	0,26	No
ENSMUSG00000068011	6,94E-01	-2,89617475	2,61E-01	6,09E-01	8,51E-01	Mkrn2os	1,62	No
ENSMUSG00000068015	3,28E-02	5,204108014	2,06E-01	6,50E-01	8,76E-01	Lrch1	1,02	No
ENSMUSG00000068036	1,31E-01	7,082351782	4,67E+00	3,06E-02	2,02E-01	Afdn	1,09	No
ENSMUSG00000068037	1,25E+00	-2,770629487	9,72E-01	3,24E-01	6,25E-01	Mas1	2,38	No
ENSMUSG00000068039	-1,41E-01	7,970767558	5,90E+00	1,52E-02	1,27E-01	Tcp1	0,91	No
ENSMUSG00000068040	4,60E-02	6,236158273	5,18E-01	4,72E-01	7,60E-01	Tm9sf4	1,03	No
ENSMUSG00000068062	2,65E+00	-3,033655087	2,73E+00	9,86E-02	3,91E-01	Gm14164	6,30	No
ENSMUSG00000068079	1,85E+00	-3,107657832	1,35E+00	2,46E-01	5,50E-01	Tcf15	3,61	No
ENSMUSG00000068099	-3,83E-02	-3,107651936	-7,91E-11	1,00E+00	1,00E+00	1500009C09Rik	0,97	No
ENSMUSG00000068101	3,62E-01	2,475451744	7,53E+00	6,07E-03	6,47E-02	Cenpm	1,29	No

ENSMUSG00000068105	-2,83E-01	0,214443115	1,16E+00	2,81E-01	5,77E-01	Tnfrsf13c	0,82	No
ENSMUSG00000068114	2,25E-01	3,981904228	6,26E+00	1,23E-02	1,09E-01	Ccdc134	1,17	No
ENSMUSG00000068115	2,25E-01	1,676358981	1,84E+00	1,75E-01	5,33E-01	Ninl	1,17	No
ENSMUSG00000068120	-4,89E-02	6,298390396	5,90E-01	4,42E-01	7,34E-01	Gm10231	0,97	No
ENSMUSG00000068129	-2,31E-01	0,966582609	1,22E+00	2,69E-01	5,65E-01	Cst7	0,85	No
ENSMUSG00000068130	-5,72E-02	0,76821088	6,78E-02	7,95E-01	9,56E-01	Zfp442	0,96	No
ENSMUSG00000068134	1,02E-01	4,597775948	1,67E+00	1,96E-01	5,50E-01	Zfp120	1,07	No
ENSMUSG00000068141	1,03E-02	5,59909193	2,27E-02	8,80E-01	1,00E+00	Gm10232	1,01	No
ENSMUSG00000068154	-7,68E-01	1,56354401	2,13E+01	3,98E-06	1,49E-04	Insm1	0,59	Yes
ENSMUSG00000068165	1,39E-02	4,551746786	2,99E-02	8,63E-01	9,97E-01	Gm10233	1,01	No
ENSMUSG00000068167	1,81E+00	-3,10765737	1,28E+00	2,58E-01	5,50E-01	Csnka2ip	3,49	No
ENSMUSG00000068184	-1,09E-01	2,426938948	6,77E-01	4,11E-01	7,07E-01	Ndufaf2	0,93	No
ENSMUSG00000068196	-3,57E+00	-2,711681187	5,73E+00	1,66E-02	1,34E-01	Col8a1	0,08	No
ENSMUSG00000068205	-2,96E-02	0,379507687	1,39E-02	9,06E-01	1,00E+00	Macrod2	0,98	No
ENSMUSG00000068206	6,06E-02	3,567229962	3,84E-01	5,36E-01	8,08E-01	Pick1	1,04	No
ENSMUSG00000068220	1,31E-01	7,990804128	5,04E+00	2,48E-02	1,74E-01	Lgals1	1,09	No
ENSMUSG00000068226	1,50E-01	1,514765136	7,23E-01	3,95E-01	6,96E-01	Gm6723	1,11	No
ENSMUSG00000068227	1,60E-01	-1,574179011	8,35E-02	7,73E-01	9,44E-01	Il2rb	1,12	No
ENSMUSG00000068240	-2,04E-01	8,645458695	1,27E+01	3,64E-04	7,08E-03	Gm11808	0,87	Yes
ENSMUSG00000068245	-5,50E-02	1,705290742	1,12E-01	7,37E-01	9,25E-01	Phf11d	0,96	No
ENSMUSG00000068246	-1,69E+00	-2,401240471	3,16E+00	7,53E-02	3,49E-01	Apol9b	0,31	No
ENSMUSG00000068250	-1,59E-01	4,144078639	3,51E+00	6,11E-02	3,07E-01	Amn1	0,90	No
ENSMUSG00000068252	-3,83E-02	-3,107651936	-7,91E-11	1,00E+00	1,00E+00	Apol7b	0,97	No
ENSMUSG00000068259	-2,51E-01	-1,997921343	1,24E-01	7,25E-01	9,18E-01	Olfr461	0,84	No
ENSMUSG00000068262	-9,96E-02	6,554771382	2,54E+00	1,11E-01	4,08E-01	Gm5879	0,93	No
ENSMUSG00000068263	-6,59E-01	-0,170584437	4,44E+00	3,51E-02	2,22E-01	Efcc1	0,63	No
ENSMUSG00000068264	3,04E-03	4,370293172	1,36E-03	9,71E-01	1,00E+00	Ap5s1	1,00	No
ENSMUSG00000068267	1,10E-01	7,809706074	3,49E+00	6,18E-02	3,09E-01	Cenpb	1,08	No
ENSMUSG00000068284	1,42E-03	6,407348797	5,11E-04	9,82E-01	1,00E+00	Usf3	1,00	No
ENSMUSG00000068289	-7,69E-02	-2,963220349	2,69E-03	9,59E-01	1,00E+00	Cma2	0,95	No
ENSMUSG00000068290	1,10E-01	4,819465319	2,08E+00	1,49E-01	4,90E-01	Ddrgk1	1,08	No
ENSMUSG00000068299	1,44E-01	0,776175005	4,31E-01	5,12E-01	7,94E-01	Nat8f4	1,11	No

ENSMUSG00000068303	1,57E+00	-2,226323696	2,70E+00	1,01E-01	3,92E-01	Spr-ps1	2,97	No
ENSMUSG00000068314	1,36E-01	-1,070151694	9,30E-02	7,60E-01	9,37E-01	Gm6899	1,10	No
ENSMUSG00000068323	1,86E-01	-0,257666459	3,49E-01	5,54E-01	8,20E-01	Slc4a5	1,14	No
ENSMUSG00000068324	-2,59E-01	4,204835274	9,17E+00	2,46E-03	3,29E-02	Gm10029	0,84	Yes
ENSMUSG00000068327	-7,45E-01	1,192042769	1,60E+01	6,18E-05	1,62E-03	Tlx2	0,60	Yes
ENSMUSG00000068328	-6,41E-02	6,543720636	1,06E+00	3,04E-01	6,01E-01	Aup1	0,96	No
ENSMUSG00000068329	-2,45E-01	6,233952624	1,47E+01	1,26E-04	2,95E-03	Htra2	0,84	Yes
ENSMUSG00000068335	-4,29E-01	6,044743189	4,39E+01	3,47E-11	3,49E-09	Dok1	0,74	Yes
ENSMUSG00000068373	-1,89E+00	-3,1076441	1,42E+00	2,33E-01	5,50E-01	D430041D05Rik	0,27	No
ENSMUSG00000068391	-1,36E-01	4,766924508	3,14E+00	7,62E-02	3,52E-01	Chrac1	0,91	No
ENSMUSG00000068394	1,55E-01	4,737363077	4,03E+00	4,48E-02	2,52E-01	Cep152	1,11	No
ENSMUSG00000068396	-2,42E-01	7,785977214	1,70E+01	3,75E-05	1,06E-03	Rpl34-ps1	0,85	Yes
ENSMUSG00000068397	-2,59E-01	5,935284346	1,55E+01	8,25E-05	2,06E-03	Gm10240	0,84	Yes
ENSMUSG00000068417	-7,77E-02	5,057492793	1,13E+00	2,88E-01	5,84E-01	Pnp2	0,95	No
ENSMUSG00000068463	3,18E-01	-1,614120085	3,22E-01	5,70E-01	8,31E-01	B630019A10Rik	1,25	No
ENSMUSG00000068466	-7,61E-02	4,503144189	8,89E-01	3,46E-01	6,47E-01	Gm5518	0,95	No
ENSMUSG00000068479	3,60E-02	5,467946191	2,72E-01	6,02E-01	8,48E-01	Mfap1a	1,03	No
ENSMUSG00000068480	9,00E-02	4,395725997	1,18E+00	2,77E-01	5,73E-01	Gm7551	1,06	No
ENSMUSG00000068487	-4,50E-02	3,719387691	2,26E-01	6,34E-01	8,66E-01	Gm4943	0,97	No
ENSMUSG00000068522	-1,35E+00	-1,107420821	9,14E+00	2,50E-03	3,33E-02	Aard	0,39	Yes
ENSMUSG00000068523	7,86E-03	4,938341624	1,11E-02	9,16E-01	1,00E+00	Gng5	1,01	No
ENSMUSG00000068536	-1,89E+00	-3,1076441	1,42E+00	2,33E-01	5,50E-01	Doxl2	0,27	No
ENSMUSG00000068551	-1,54E-01	4,200646875	3,40E+00	6,54E-02	3,21E-01	Zfp467	0,90	No
ENSMUSG00000068566	2,69E-01	8,990077186	2,24E+01	2,24E-06	8,98E-05	Myadm	1,21	Yes
ENSMUSG00000068579	-1,76E-01	3,548031847	3,22E+00	7,26E-02	3,42E-01	Rpl7a-ps3	0,88	No
ENSMUSG00000068580	1,21E-01	4,439701652	2,22E+00	1,36E-01	4,65E-01	Zfyve19	1,09	No
ENSMUSG00000068587	-2,63E-01	0,489897454	1,29E+00	2,56E-01	5,50E-01	Mgam	0,83	No
ENSMUSG00000068601	-2,21E-02	3,099743998	3,91E-02	8,43E-01	9,85E-01	Gm10244	0,98	No
ENSMUSG00000068604	-5,00E-02	4,085976686	3,31E-01	5,65E-01	8,28E-01	Gm8225	0,97	No
ENSMUSG00000068606	1,83E+00	-2,96327665	1,33E+00	2,49E-01	5,50E-01	Gm4841	3,57	No
ENSMUSG00000068629	1,57E-01	-0,800833654	1,59E-01	6,90E-01	8,99E-01	Gm6155	1,12	No
ENSMUSG00000068631	-8,14E-01	6,208854092	1,57E+02	5,60E-36	4,94E-33	Gm7676	0,57	Yes

ENSMUSG00000068663	-3,10E-02	5,752985519	2,13E-01	6,44E-01	8,72E-01	Clec16a	0,98	No
ENSMUSG00000068674	-2,02E-01	3,740057384	4,70E+00	3,02E-02	1,99E-01	Gm10247	0,87	No
ENSMUSG00000068680	6,46E-01	-2,26819634	6,03E-01	4,37E-01	7,30E-01	Gm10248	1,56	No
ENSMUSG00000068686	4,64E-01	-1,460812195	7,79E-01	3,77E-01	6,78E-01	Cd59b	1,38	No
ENSMUSG00000068689	-1,38E+00	-2,896110777	1,20E+00	2,74E-01	5,71E-01	Rps19-ps9	0,38	No
ENSMUSG00000068697	-2,38E-01	-0,726206108	3,49E-01	5,54E-01	8,20E-01	Myoz1	0,85	No
ENSMUSG00000068699	4,47E-01	4,881282953	3,42E+01	4,87E-09	3,58E-07	Flnc	1,36	Yes
ENSMUSG00000068706	-1,14E-01	6,898384032	3,51E+00	6,11E-02	3,07E-01	Gm10250	0,92	No
ENSMUSG00000068732	-5,35E-02	5,94614792	6,62E-01	4,16E-01	7,11E-01	Tmem167b	0,96	No
ENSMUSG00000068735	7,61E-02	6,97339337	1,56E+00	2,11E-01	5,50E-01	Trp53i11	1,05	No
ENSMUSG00000068739	2,96E-02	6,858746987	2,34E-01	6,29E-01	8,62E-01	Sars	1,02	No
ENSMUSG00000068740	1,19E-01	-0,175499611	1,28E-01	7,20E-01	9,15E-01	Celsr2	1,09	No
ENSMUSG00000068742	-4,28E-02	3,547119133	1,86E-01	6,66E-01	8,86E-01	Cry2	0,97	No
ENSMUSG00000068744	-9,56E-02	4,580322289	1,46E+00	2,28E-01	5,50E-01	Psrc1	0,94	No
ENSMUSG00000068745	-4,86E-02	-2,896141539	1,09E-03	9,74E-01	1,00E+00	Mybphl	0,97	No
ENSMUSG00000068747	-1,99E-02	7,20812415	1,09E-01	7,41E-01	9,27E-01	Sort1	0,99	No
ENSMUSG00000068749	-8,09E-02	3,338648018	6,21E-01	4,31E-01	7,24E-01	Psma5	0,95	No
ENSMUSG00000068758	9,95E-04	4,432382762	1,49E-04	9,90E-01	1,00E+00	Il3ra	1,00	No
ENSMUSG00000068762	-7,76E-02	-2,89613075	4,21E-03	9,48E-01	1,00E+00	Gstm6	0,95	No
ENSMUSG00000068794	-2,66E+00	-2,96323193	2,82E+00	9,29E-02	3,83E-01	Col28a1	0,16	No
ENSMUSG00000068798	3,49E-02	5,785954985	2,71E-01	6,03E-01	8,48E-01	Rap1a	1,02	No
ENSMUSG00000068823	-6,79E-02	7,763130418	1,34E+00	2,47E-01	5,50E-01	Csde1	0,95	No
ENSMUSG00000068854	6,02E-02	3,193209454	3,20E-01	5,71E-01	8,32E-01	Hist2h2be	1,04	No
ENSMUSG00000068855	4,68E-01	-2,355611402	2,98E-01	5,85E-01	8,39E-01	Hist2h2ac	1,38	No
ENSMUSG00000068856	4,82E-02	5,159891606	4,41E-01	5,07E-01	7,91E-01	Sf3b4	1,03	No
ENSMUSG00000068859	7,33E-02	1,604129608	1,89E-01	6,64E-01	8,84E-01	Sp9	1,05	No
ENSMUSG00000068860	-3,85E-01	-1,006905468	6,73E-01	4,12E-01	7,07E-01	Gm128	0,77	No
ENSMUSG00000068874	3,32E-01	3,381152287	1,01E+01	1,49E-03	2,22E-02	Selenbp1	1,26	Yes
ENSMUSG00000068876	1,75E+00	-2,355712016	3,54E+00	5,98E-02	3,03E-01	Cgn	3,37	No
ENSMUSG00000068877	7,06E-01	0,425377227	7,47E+00	6,29E-03	6,65E-02	Selenbp2	1,63	No
ENSMUSG00000068882	4,91E-02	6,467028102	6,08E-01	4,35E-01	7,28E-01	Ssb	1,03	No
ENSMUSG00000068890	-8,45E-01	-2,963241229	3,92E-01	5,31E-01	8,05E-01	Lce1a2	0,56	No

ENSMUSG00000068917	8,20E-02	5,347062725	1,36E+00	2,43E-01	5,50E-01	Clk2	1,06	No
ENSMUSG00000068921	7,73E-02	5,000137884	1,09E+00	2,96E-01	5,92E-01	Dap3	1,06	No
ENSMUSG00000068922	2,95E-02	4,272094012	1,25E-01	7,24E-01	9,17E-01	Msto1	1,02	No
ENSMUSG00000068923	2,10E-01	3,64039162	4,74E+00	2,95E-02	1,96E-01	Syt11	1,16	No
ENSMUSG00000068957	-2,41E-01	-0,755559597	3,58E-01	5,49E-01	8,17E-01	4930589L23Rik	0,85	No
ENSMUSG00000068959	-1,27E-01	3,004249544	1,27E+00	2,61E-01	5,54E-01	Zfp619	0,92	No
ENSMUSG00000068962	-6,48E-02	-1,522751357	1,37E-02	9,07E-01	1,00E+00	Zfp114	0,96	No
ENSMUSG00000068964	-9,27E-02	2,315417185	4,58E-01	4,99E-01	7,84E-01	Gm6361	0,94	No
ENSMUSG00000068966	-6,98E-02	4,442310379	7,66E-01	3,82E-01	6,83E-01	Zbtb34	0,95	No
ENSMUSG00000068969	-1,65E-01	0,866296934	5,76E-01	4,48E-01	7,38E-01	Rpl32-ps	0,89	No
ENSMUSG00000069008	1,08E-01	8,301974006	3,47E+00	6,24E-02	3,11E-01	Gm5537	1,08	No
ENSMUSG00000069011	9,31E-03	5,955588151	2,00E-02	8,88E-01	1,00E+00	Gm10254	1,01	No
ENSMUSG00000069014	6,37E-02	6,664221366	1,05E+00	3,04E-01	6,01E-01	Gm5641	1,05	No
ENSMUSG00000069020	5,03E-02	4,991791065	4,57E-01	4,99E-01	7,84E-01	Urm1	1,04	No
ENSMUSG00000069041	-2,00E-02	-2,896117932	2,29E-04	9,88E-01	1,00E+00	Slc25a31	0,99	No
ENSMUSG00000069072	-8,86E-02	-2,963249454	3,57E-03	9,52E-01	1,00E+00	Slc7a14	0,94	No
ENSMUSG00000069080	1,81E+00	-3,10765737	1,28E+00	2,58E-01	5,50E-01	Lcn11	3,49	No
ENSMUSG00000069083	2,20E-01	6,141481424	1,15E+01	6,95E-04	1,20E-02	Gm10259	1,16	Yes
ENSMUSG00000069089	6,39E-02	3,895787452	4,99E-01	4,80E-01	7,68E-01	Cdk7	1,05	No
ENSMUSG00000069094	-1,01E-01	4,543783801	1,60E+00	2,06E-01	5,50E-01	Pde7a	0,93	No
ENSMUSG00000069114	-4,11E-01	2,942949437	1,36E+01	2,22E-04	4,72E-03	Zbtb10	0,75	Yes
ENSMUSG00000069117	-2,28E-01	6,277234392	1,28E+01	3,51E-04	6,88E-03	Gm10260	0,85	Yes
ENSMUSG00000069125	-4,72E-01	-2,185687377	4,11E-01	5,21E-01	8,00E-01	Rps24-ps2	0,72	No
ENSMUSG00000069135	4,29E-03	4,613472587	2,91E-03	9,57E-01	1,00E+00	Fgfr1op	1,00	No
ENSMUSG00000069136	5,45E-01	-1,195773491	1,32E+00	2,50E-01	5,50E-01	A130006I12Rik	1,46	No
ENSMUSG00000069170	-5,88E-02	-2,770553291	1,53E-03	9,69E-01	1,00E+00	Adgrv1	0,96	No
ENSMUSG00000069171	-2,66E+00	-2,9632547	2,82E+00	9,28E-02	3,83E-01	Nr2f1	0,16	No
ENSMUSG00000069184	3,61E-01	1,22493967	3,54E+00	6,00E-02	3,03E-01	Zfp72	1,28	No
ENSMUSG00000069188	-1,34E-01	5,388180091	3,70E+00	5,43E-02	2,85E-01	Gm13192	0,91	No
ENSMUSG00000069206	3,83E-02	2,878817458	1,12E-01	7,37E-01	9,25E-01	Zfp874a	1,03	No
ENSMUSG00000069208	2,62E-01	2,320576461	3,73E+00	5,34E-02	2,82E-01	Zfp825	1,20	No
ENSMUSG00000069223	1,03E+00	-1,322331289	4,07E+00	4,36E-02	2,49E-01	4930451E10Rik	2,04	No

ENSMUSG00000069236	-5,74E-01	-1,683232227	9,11E-01	3,40E-01	6,41E-01	Gm7251	0,67	No
ENSMUSG00000069237	5,64E-02	4,540507098	4,96E-01	4,81E-01	7,69E-01	Fam8a1	1,04	No
ENSMUSG00000069248	-1,95E+00	-3,033628749	1,50E+00	2,21E-01	5,50E-01	Serpinb6e	0,26	No
ENSMUSG00000069255	1,77E-01	5,12710322	5,96E+00	1,47E-02	1,24E-01	Dusp22	1,13	No
ENSMUSG00000069265	-2,20E-01	-1,126803979	2,25E-01	6,35E-01	8,67E-01	Hist1h3a	0,86	No
ENSMUSG00000069266	4,85E-01	-2,318647281	3,54E-01	5,52E-01	8,18E-01	Hist1h4b	1,40	No
ENSMUSG00000069267	8,05E-01	-1,49762964	1,81E+00	1,79E-01	5,40E-01	Hist1h3b	1,75	No
ENSMUSG00000069268	-1,34E-01	0,511845113	3,00E-01	5,84E-01	8,38E-01	Hist1h2bf	0,91	No
ENSMUSG00000069270	2,05E-01	0,443845754	6,53E-01	4,19E-01	7,14E-01	Hist1h2ac	1,15	No
ENSMUSG00000069272	2,69E-01	-0,355480053	5,99E-01	4,39E-01	7,32E-01	Hist1h2ae	1,20	No
ENSMUSG00000069273	-3,60E-01	-1,205840439	6,65E-01	4,15E-01	7,10E-01	Hist1h3e	0,78	No
ENSMUSG00000069274	-1,35E+00	-2,770624777	1,13E+00	2,87E-01	5,83E-01	Hist1h4f	0,39	No
ENSMUSG00000069300	1,91E-02	1,21815769	9,52E-03	9,22E-01	1,00E+00	Hist1h2bj	1,01	No
ENSMUSG00000069301	-6,71E-01	-1,262635013	2,03E+00	1,54E-01	4,98E-01	Hist1h2ag	0,63	No
ENSMUSG00000069302	1,63E+00	-1,879964881	5,79E+00	1,62E-02	1,32E-01	Hist1h2ah	3,10	No
ENSMUSG00000069303	-5,41E-01	1,887642061	1,22E+01	4,77E-04	8,83E-03	Hist1h2br	0,69	Yes
ENSMUSG00000069305	-5,63E-01	-0,574004014	2,61E+00	1,06E-01	3,98E-01	Hist1h4n	0,68	No
ENSMUSG00000069306	-4,09E-03	-1,070138684	7,98E-05	9,93E-01	1,00E+00	Hist1h4m	1,00	No
ENSMUSG00000069307	-1,39E-01	1,842603405	7,99E-01	3,71E-01	6,73E-01	Hist1h2bq	0,91	No
ENSMUSG00000069308	1,39E-01	0,116236392	2,40E-01	6,24E-01	8,59E-01	Hist1h2bp	1,10	No
ENSMUSG00000069309	1,02E+00	-2,165776942	1,85E+00	1,74E-01	5,31E-01	Hist1h2an	2,02	No
ENSMUSG00000069310	-1,97E-01	-0,413246447	3,28E-01	5,67E-01	8,29E-01	Hist1h3c	0,87	No
ENSMUSG00000069376	6,00E-02	2,57771778	2,17E-01	6,42E-01	8,71E-01	Gm5507	1,04	No
ENSMUSG00000069378	1,81E+00	-3,10765737	1,28E+00	2,58E-01	5,50E-01	Prdm6	3,49	No
ENSMUSG00000069379	3,93E-02	4,730499596	2,57E-01	6,12E-01	8,52E-01	Gm4950	1,03	No
ENSMUSG00000069439	-7,38E-02	-3,033659033	2,66E-03	9,59E-01	1,00E+00	Gm8444	0,95	No
ENSMUSG00000069456	-1,23E+00	-3,146117124	7,12E-01	3,99E-01	6,98E-01	Rdh16	0,42	No
ENSMUSG00000069475	2,40E-01	-2,35560678	7,23E-02	7,88E-01	9,53E-01	Gm6020	1,18	No
ENSMUSG00000069495	1,47E-01	5,116409778	4,05E+00	4,42E-02	2,50E-01	Epc2	1,11	No
ENSMUSG00000069515	6,80E-02	3,654887257	4,95E-01	4,82E-01	7,69E-01	Lyz1	1,05	No
ENSMUSG00000069516	1,80E-02	14,02226704	1,05E-01	7,46E-01	9,30E-01	Lyz2	1,01	No
ENSMUSG00000069518	-1,86E+00	-2,963227946	1,40E+00	2,36E-01	5,50E-01	Gm10271	0,28	No

ENSMUSG00000069520	-8,97E-02	5,964725381	1,88E+00	1,71E-01	5,26E-01	Tmem19	0,94	No
ENSMUSG00000069539	-7,85E-02	6,416596664	1,56E+00	2,12E-01	5,50E-01	Scyl2	0,95	No
ENSMUSG00000069540	-1,03E-02	-1,600678674	3,25E-04	9,86E-01	1,00E+00	Gm4925	0,99	No
ENSMUSG00000069554	3,14E-02	-3,107617333	-7,69E-10	1,00E+00	1,00E+00	I830134H01Rik	1,02	No
ENSMUSG00000069565	3,42E-02	6,332934588	2,91E-01	5,90E-01	8,41E-01	Dazap1	1,02	No
ENSMUSG00000069581	-1,89E+00	-3,1076441	1,42E+00	2,33E-01	5,50E-01	Tspear	0,27	No
ENSMUSG00000069588	-2,68E+00	-3,033645766	2,84E+00	9,17E-02	3,82E-01	Gm11733	0,16	No
ENSMUSG00000069601	-1,77E+00	-2,655122034	2,18E+00	1,40E-01	4,72E-01	Ank3	0,29	No
ENSMUSG00000069607	7,60E-01	2,23727868	2,66E+01	2,47E-07	1,27E-05	Cd300ld3	1,69	Yes
ENSMUSG00000069609	7,44E-01	1,240266645	1,45E+01	1,40E-04	3,21E-03	Cd300ld4	1,68	Yes
ENSMUSG00000069622	1,85E-02	4,901643634	6,04E-02	8,06E-01	9,63E-01	Gm10273	1,01	No
ENSMUSG00000069631	1,88E-02	4,946504884	6,40E-02	8,00E-01	9,61E-01	Strada	1,01	No
ENSMUSG00000069633	-3,60E-02	2,000013391	5,81E-02	8,10E-01	9,65E-01	Pex11g	0,98	No
ENSMUSG00000069662	-1,77E-01	8,409251047	9,54E+00	2,01E-03	2,82E-02	Marcks	0,88	Yes
ENSMUSG00000069670	-2,71E+00	-3,033620915	2,92E+00	8,75E-02	3,78E-01	Nkain2	0,15	No
ENSMUSG00000069678	-3,01E-01	2,119559778	4,22E+00	4,00E-02	2,38E-01	Pcgf1	0,81	No
ENSMUSG00000069682	-1,09E-01	8,776957283	3,68E+00	5,52E-02	2,88E-01	Gm10275	0,93	No
ENSMUSG00000069712	-1,90E+00	-3,033647263	1,44E+00	2,30E-01	5,50E-01	4930444G20Rik	0,27	No
ENSMUSG00000069713	4,01E-01	-1,247144278	4,81E-01	4,88E-01	7,75E-01	4933406P04Rik	1,32	No
ENSMUSG00000069727	5,56E-02	1,569024704	1,10E-01	7,40E-01	9,26E-01	Zfp975	1,04	No
ENSMUSG00000069729	-1,25E-01	6,520306604	3,98E+00	4,61E-02	2,57E-01	Arid1b	0,92	No
ENSMUSG00000069743	8,40E-02	-0,436824797	6,61E-02	7,97E-01	9,58E-01	Zfp820	1,06	No
ENSMUSG00000069744	-1,31E-01	4,445846535	2,61E+00	1,06E-01	3,99E-01	Psmb3	0,91	No
ENSMUSG00000069755	-9,29E-02	2,457290358	5,15E-01	4,73E-01	7,61E-01	Zfp125	0,94	No
ENSMUSG00000069763	-1,89E+00	-3,033648118	1,42E+00	2,33E-01	5,50E-01	Tmem100	0,27	No
ENSMUSG00000069769	-4,68E-01	2,473603951	1,34E+01	2,55E-04	5,31E-03	Msi2	0,72	Yes
ENSMUSG00000069792	2,03E-02	9,564908452	1,29E-01	7,19E-01	9,15E-01	Wfdc17	1,01	No
ENSMUSG00000069793	2,19E-01	5,622432027	1,04E+01	1,27E-03	1,96E-02	Slfn9	1,16	Yes
ENSMUSG00000069804	-1,03E+00	-1,472818242	3,40E+00	6,52E-02	3,20E-01	Gm10277	0,49	No
ENSMUSG00000069805	1,21E+00	-1,44883177	4,64E+00	3,13E-02	2,05E-01	Fbp1	2,31	No
ENSMUSG00000069806	-2,34E+00	-1,682954528	1,03E+01	1,32E-03	2,03E-02	Cacng7	0,20	Yes
ENSMUSG00000069808	8,56E-02	3,919226472	9,13E-01	3,39E-01	6,40E-01	Fam57a	1,06	No

ENSMUSG00000069814	6,45E-01	-1,165869271	1,60E+00	2,05E-01	5,50E-01	Ccdc92b	1,56	No
ENSMUSG00000069830	3,08E-01	4,590306718	1,51E+01	1,01E-04	2,44E-03	Nlrp1a	1,24	Yes
ENSMUSG00000069833	3,02E-01	10,3520173	2,90E+01	7,08E-08	4,08E-06	Ahnak	1,23	Yes
ENSMUSG00000069835	1,12E-01	-0,070287597	1,37E-01	7,12E-01	9,11E-01	Sat2	1,08	No
ENSMUSG00000069844	-2,42E-02	4,000997031	7,30E-02	7,87E-01	9,53E-01	Sco1	0,98	No
ENSMUSG00000069855	3,13E+00	-2,832064034	4,00E+00	4,55E-02	2,54E-01	Slc47a2	8,77	No
ENSMUSG00000069862	-1,55E-01	6,156388001	5,80E+00	1,60E-02	1,31E-01	Rps12-ps9	0,90	No
ENSMUSG00000069867	8,41E-01	-2,401437283	8,51E-01	3,56E-01	6,58E-01	Pabpn1l	1,79	No
ENSMUSG00000069873	-6,51E-01	-0,535278462	3,48E+00	6,22E-02	3,10E-01	4930438A08Rik	0,64	No
ENSMUSG00000069874	6,48E-02	3,933609689	5,43E-01	4,61E-01	7,50E-01	Irgm2	1,05	No
ENSMUSG00000069890	-1,89E+00	-3,1076441	1,42E+00	2,33E-01	5,50E-01	1700024J04Rik	0,27	No
ENSMUSG00000069892	3,76E-01	5,737520732	3,16E+01	1,87E-08	1,22E-06	9930111J21Rik2	1,30	Yes
ENSMUSG00000069893	3,58E-01	5,084909194	2,46E+01	7,17E-07	3,31E-05	9930111J21Rik1	1,28	Yes
ENSMUSG00000069895	3,74E-02	6,439055593	3,51E-01	5,54E-01	8,19E-01	Atxn1l	1,03	No
ENSMUSG00000069899	-2,99E-02	5,273868402	1,75E-01	6,76E-01	8,91E-01	Gm12166	0,98	No
ENSMUSG00000069910	1,19E-01	4,395005005	2,12E+00	1,45E-01	4,82E-01	Spdl1	1,09	No
ENSMUSG00000069911	-1,35E+00	-0,682973486	1,05E+01	1,22E-03	1,90E-02	Fam196b	0,39	Yes
ENSMUSG00000069913	1,55E-01	3,738998087	2,74E+00	9,76E-02	3,90E-01	Anp32-ps	1,11	No
ENSMUSG00000069917	-1,73E+00	-2,600609768	2,09E+00	1,48E-01	4,88E-01	Hba-a2	0,30	No
ENSMUSG00000069919	-4,10E-02	-2,497611554	1,57E-03	9,68E-01	1,00E+00	Hba-a1	0,97	No
ENSMUSG00000069920	2,06E-01	1,200769428	1,18E+00	2,76E-01	5,73E-01	B3gnt9	1,15	No
ENSMUSG00000069925	-7,98E-01	-2,963241606	3,44E-01	5,57E-01	8,21E-01	4932416K20Rik	0,58	No
ENSMUSG00000069939	1,05E-01	7,327919932	3,11E+00	7,76E-02	3,56E-01	Gm12070	1,08	No
ENSMUSG00000069962	5,34E-01	-0,980521428	1,30E+00	2,54E-01	5,50E-01	Gm5356	1,45	No
ENSMUSG00000069972	-1,52E-01	7,438082361	6,54E+00	1,05E-02	9,70E-02	Rps13-ps2	0,90	No
ENSMUSG00000069986	-4,34E-02	1,755884819	7,50E-02	7,84E-01	9,51E-01	Gm8054	0,97	No
ENSMUSG00000069996	7,20E-01	-2,963285673	2,81E-01	5,96E-01	8,44E-01	Gm7125	1,65	No
ENSMUSG00000070000	8,74E-02	5,296928801	1,51E+00	2,19E-01	5,50E-01	Fcho1	1,06	No
ENSMUSG00000070002	-1,03E-01	5,333902521	2,11E+00	1,46E-01	4,84E-01	Ell	0,93	No
ENSMUSG00000070003	1,55E-01	5,531369642	5,01E+00	2,52E-02	1,76E-01	Ssbp4	1,11	No
ENSMUSG00000070025	1,36E-01	5,240753664	3,60E+00	5,78E-02	2,97E-01	Gm10284	1,10	No
ENSMUSG00000070031	-2,97E-01	5,224677149	1,73E+01	3,15E-05	9,14E-04	Sp140	0,81	Yes

ENSMUSG00000070034	-2,08E-01	5,172184388	8,39E+00	3,77E-03	4,53E-02	Sp110	0,87	Yes
ENSMUSG00000070044	4,63E-01	3,229683653	1,81E+01	2,14E-05	6,62E-04	Fam149a	1,38	Yes
ENSMUSG00000070047	-4,51E-01	1,075154622	5,44E+00	1,96E-02	1,49E-01	Fat1	0,73	No
ENSMUSG00000070056	-5,86E-02	6,522143987	8,76E-01	3,49E-01	6,51E-01	Mfhas1	0,96	No
ENSMUSG00000070063	-7,84E-03	-1,543875005	2,04E-04	9,89E-01	1,00E+00	Snora33	0,99	No
ENSMUSG00000070065	2,06E-02	-3,146103739	-9,26E-10	1,00E+00	1,00E+00	Mir451a	1,01	No
ENSMUSG00000070074	1,87E+00	-3,107639691	1,38E+00	2,40E-01	5,50E-01	Mir484	3,67	No
ENSMUSG00000070077	-1,87E+00	-3,03364178	1,41E+00	2,36E-01	5,50E-01	Mir491	0,27	No
ENSMUSG00000070127	-7,00E-02	-2,600667548	3,27E-03	9,54E-01	1,00E+00	Mir146b	0,95	No
ENSMUSG00000070130	1,43E-01	-2,68311995	1,97E-02	8,88E-01	1,00E+00	Mir328	1,10	No
ENSMUSG00000070166	-6,30E-01	-2,268049019	7,73E-01	3,79E-01	6,80E-01	Gm25821	0,65	No
ENSMUSG00000070167	-5,78E-01	-2,226276809	5,92E-01	4,42E-01	7,34E-01	Snora57	0,67	No
ENSMUSG00000070209	-1,89E+00	-3,1076441	1,42E+00	2,33E-01	5,50E-01	n-R5s27	0,27	No
ENSMUSG00000070214	1,81E+00	-3,10765737	1,28E+00	2,58E-01	5,50E-01	Gm23058	3,49	No
ENSMUSG00000070225	-2,66E+00	-2,896116003	2,81E+00	9,35E-02	3,83E-01	n-R5s33	0,16	No
ENSMUSG00000070249	9,01E-03	-3,107646452	-5,16E-10	1,00E+00	1,00E+00	Gm23520	1,01	No
ENSMUSG00000070271	-4,09E-01	3,011404501	1,27E+01	3,65E-04	7,08E-03	Gm13268	0,75	Yes
ENSMUSG00000070280	-1,94E+00	-3,107619243	1,50E+00	2,21E-01	5,50E-01	Slc22a14	0,26	No
ENSMUSG00000070282	4,46E-01	0,119899031	2,55E+00	1,10E-01	4,06E-01	3000002C10Rik	1,36	No
ENSMUSG00000070283	-9,93E-02	3,388177898	9,22E-01	3,37E-01	6,38E-01	Ndufaf3	0,93	No
ENSMUSG00000070284	1,60E-01	4,64256233	4,16E+00	4,14E-02	2,42E-01	Gmppb	1,12	No
ENSMUSG00000070287	-6,60E-01	-0,912481106	2,25E+00	1,34E-01	4,60E-01	Slc35g2	0,63	No
ENSMUSG00000070291	-2,49E-02	-1,247141665	3,01E-03	9,56E-01	1,00E+00	Ddx43	0,98	No
ENSMUSG00000070298	-5,13E-01	-1,289611525	8,52E-01	3,56E-01	6,57E-01	Trcg1	0,70	No
ENSMUSG00000070304	-1,01E+00	2,810565049	7,89E+01	6,54E-19	1,38E-16	Scn2b	0,49	Yes
ENSMUSG00000070305	-2,45E-01	4,892305175	1,07E+01	1,05E-03	1,69E-02	Mpzl3	0,84	Yes
ENSMUSG00000070306	-2,73E-02	-2,963256629	3,40E-04	9,85E-01	1,00E+00	Ccdc153	0,98	No
ENSMUSG00000070315	-1,95E-01	2,887269794	2,98E+00	8,42E-02	3,72E-01	4930581F22Rik	0,87	No
ENSMUSG00000070319	-9,76E-02	5,44148172	1,96E+00	1,62E-01	5,11E-01	Eif3g	0,93	No
ENSMUSG00000070323	-2,72E-02	0,378887087	1,07E-02	9,18E-01	1,00E+00	Mmp27	0,98	No
ENSMUSG00000070327	-4,11E-04	8,057611619	5,01E-05	9,94E-01	1,00E+00	Rnf213	1,00	No
ENSMUSG00000070330	-8,52E-02	-2,311172974	6,47E-03	9,36E-01	1,00E+00	Tmem235	0,94	No

ENSMUSG00000070331	-8,60E-02	-2,448724417	8,47E-03	9,27E-01	1,00E+00	Qrich2	0,94	No
ENSMUSG00000070332	-1,94E+00	-3,107619243	1,50E+00	2,21E-01	5,50E-01	Trim80	0,26	No
ENSMUSG00000070336	-7,33E-02	-0,641435352	4,12E-02	8,39E-01	9,83E-01	Fbxo47	0,95	No
ENSMUSG00000070337	-2,50E-01	0,087553374	7,03E-01	4,02E-01	7,00E-01	Gpr179	0,84	No
ENSMUSG00000070342	1,23E+00	-2,83200776	9,30E-01	3,35E-01	6,36E-01	Gm10287	2,34	No
ENSMUSG00000070343	-1,67E-01	8,711766124	8,53E+00	3,49E-03	4,28E-02	Gm10288	0,89	Yes
ENSMUSG00000070345	2,98E-01	-0,355600439	7,47E-01	3,87E-01	6,87E-01	Hsf5	1,23	No
ENSMUSG00000070348	3,35E-01	6,916759664	2,99E+01	4,59E-08	2,78E-06	Ccnd1	1,26	Yes
ENSMUSG00000070354	1,15E-01	6,831573132	3,51E+00	6,12E-02	3,07E-01	Evi2	1,08	No
ENSMUSG00000070368	1,21E+00	-2,896186976	9,04E-01	3,42E-01	6,42E-01	Prok1	2,31	No
ENSMUSG00000070369	1,96E-01	5,771128694	8,52E+00	3,52E-03	4,31E-02	Itgad	1,15	Yes
ENSMUSG00000070371	1,47E-02	0,432193141	3,71E-03	9,51E-01	1,00E+00	Prss36	1,01	No
ENSMUSG00000070372	2,94E-02	7,830965135	2,53E-01	6,15E-01	8,54E-01	Capza1	1,02	No
ENSMUSG00000070385	-1,49E+00	-0,573961916	1,59E+01	6,61E-05	1,72E-03	Ampd1	0,36	Yes
ENSMUSG00000070388	-1,59E-02	-2,96323932	1,18E-04	9,91E-01	1,00E+00	Fbxo39	0,99	No
ENSMUSG00000070390	3,60E-01	5,170678509	2,44E+01	7,68E-07	3,53E-05	Nlrp1b	1,28	Yes
ENSMUSG00000070392	1,76E-01	2,15979741	1,48E+00	2,24E-01	5,50E-01	Gm20634	1,13	No
ENSMUSG00000070394	-1,76E-02	5,012328396	5,65E-02	8,12E-01	9,67E-01	Tmem256	0,99	No
ENSMUSG00000070407	2,22E-01	6,73590478	1,27E+01	3,65E-04	7,08E-03	Hs3st3b1	1,17	Yes
ENSMUSG00000070420	2,06E-02	3,898405065	5,11E-02	8,21E-01	9,72E-01	Zscan25	1,01	No
ENSMUSG00000070421	7,67E-01	-2,963261668	3,24E-01	5,69E-01	8,30E-01	Olfr658	1,70	No
ENSMUSG00000070424	-1,48E+00	-2,548199946	2,26E+00	1,33E-01	4,58E-01	Art5	0,36	No
ENSMUSG00000070425	-3,52E-02	2,744550535	8,91E-02	7,65E-01	9,40E-01	Xntrpc	0,98	No
ENSMUSG00000070426	2,34E-02	4,370323909	8,09E-02	7,76E-01	9,46E-01	Rnf121	1,02	No
ENSMUSG00000070427	-1,20E-02	3,158932277	1,25E-02	9,11E-01	1,00E+00	Il18bp	0,99	No
ENSMUSG00000070436	-9,29E-01	-0,83163301	5,12E+00	2,37E-02	1,69E-01	Serpinh1	0,53	No
ENSMUSG00000070443	1,46E-01	3,791589823	2,50E+00	1,14E-01	4,16E-01	Gm10291	1,11	No
ENSMUSG00000070461	-1,42E-02	3,044723491	1,63E-02	8,98E-01	1,00E+00	9230112E08Rik	0,99	No
ENSMUSG00000070462	1,86E-01	6,644096313	8,94E+00	2,80E-03	3,63E-02	Tlnrd1	1,14	Yes
ENSMUSG00000070469	9,01E-03	-3,107646452	-5,16E-10	1,00E+00	1,00E+00	Adamtsl3	1,01	No
ENSMUSG00000070471	-1,02E+00	-1,62751781	2,30E+00	1,30E-01	4,52E-01	Erich6	0,49	No
ENSMUSG00000070476	2,37E-01	5,322415795	1,11E+01	8,43E-04	1,40E-02	Fam217b	1,18	Yes

ENSMUSG00000070490	1,41E-01	4,576596007	3,16E+00	7,55E-02	3,50E-01	Gm10293	1,10	No
ENSMUSG00000070493	-6,27E-02	5,786002073	8,83E-01	3,47E-01	6,48E-01	Chchd2	0,96	No
ENSMUSG00000070495	-5,88E-02	-3,033616507	1,62E-03	9,68E-01	1,00E+00	Ctcfl	0,96	No
ENSMUSG00000070498	-2,70E+00	-2,96323619	2,89E+00	8,89E-02	3,79E-01	Tmem132b	0,15	No
ENSMUSG00000070501	-2,56E-01	2,041179072	3,13E+00	7,67E-02	3,53E-01	Ifi214	0,84	No
ENSMUSG00000070504	-6,57E-02	-2,655178995	3,94E-03	9,50E-01	1,00E+00	Fcrl6	0,96	No
ENSMUSG00000070509	-1,87E+00	-3,107637761	1,39E+00	2,38E-01	5,50E-01	Rgma	0,27	No
ENSMUSG00000070520	-1,15E-01	4,324020686	1,93E+00	1,64E-01	5,16E-01	Nsmce3	0,92	No
ENSMUSG00000070522	3,67E-01	1,019199767	3,14E+00	7,63E-02	3,52E-01	Gm6505	1,29	No
ENSMUSG00000070524	4,60E-01	3,61508213	2,22E+01	2,48E-06	9,76E-05	Fcrlb	1,38	Yes
ENSMUSG00000070526	6,01E-01	-0,847730971	2,01E+00	1,56E-01	5,01E-01	Peg12	1,52	No
ENSMUSG00000070527	-2,61E-01	-2,146174912	1,34E-01	7,14E-01	9,12E-01	Mkrn3	0,83	No
ENSMUSG00000070544	-1,25E-01	6,880939603	4,20E+00	4,04E-02	2,38E-01	Top1	0,92	No
ENSMUSG00000070547	1,81E+00	-3,10765737	1,28E+00	2,58E-01	5,50E-01	Mrgprb1	3,49	No
ENSMUSG00000070563	-5,45E-02	-2,832009003	1,34E-03	9,71E-01	1,00E+00	Spaca4	0,96	No
ENSMUSG00000070564	-1,25E+00	-2,333205398	1,49E+00	2,22E-01	5,50E-01	Ntn5	0,42	No
ENSMUSG00000070565	-9,52E-02	4,496540658	1,40E+00	2,36E-01	5,50E-01	Rasal2	0,94	No
ENSMUSG00000070568	-8,50E-02	-2,548242778	6,54E-03	9,36E-01	1,00E+00	Slc6a21	0,94	No
ENSMUSG00000070570	-9,20E-02	-1,77057307	1,97E-02	8,88E-01	1,00E+00	Slc17a7	0,94	No
ENSMUSG00000070572	-2,64E-02	4,184972443	9,69E-02	7,56E-01	9,34E-01	Trmt112-ps2	0,98	No
ENSMUSG00000070576	2,37E+00	-1,801085262	1,10E+01	9,14E-04	1,50E-02	Mn1	5,17	Yes
ENSMUSG00000070583	-7,20E-02	0,875762678	1,21E-01	7,28E-01	9,20E-01	Fv1	0,95	No
ENSMUSG00000070594	-5,51E-02	-2,998031084	1,38E-03	9,70E-01	1,00E+00	Gm4788	0,96	No
ENSMUSG00000070601	1,35E+00	-2,401411377	1,86E+00	1,73E-01	5,30E-01	Vmn2r84	2,54	No
ENSMUSG00000070604	2,33E-01	2,227116284	2,69E+00	1,01E-01	3,92E-01	Vsig10l	1,18	No
ENSMUSG00000070605	7,40E-02	5,632323806	1,18E+00	2,77E-01	5,73E-01	Zfp992	1,05	No
ENSMUSG00000070606	8,42E-01	-2,711677848	6,18E-01	4,32E-01	7,25E-01	Gm10298	1,79	No
ENSMUSG00000070610	9,59E-03	5,216255388	1,78E-02	8,94E-01	1,00E+00	Gm13127	1,01	No
ENSMUSG00000070637	-1,14E-02	-3,033635021	1,50E-05	9,97E-01	1,00E+00	Gm694	0,99	No
ENSMUSG00000070639	1,04E-01	5,874568542	2,42E+00	1,20E-01	4,30E-01	Lrrc8b	1,07	No
ENSMUSG00000070643	8,96E-01	-1,963178469	1,77E+00	1,83E-01	5,46E-01	Sox13	1,86	No
ENSMUSG00000070644	-4,28E-01	-2,448604448	1,87E-01	6,65E-01	8,85E-01	Etnk2	0,74	No

ENSMUSG00000070645	-3,19E+00	-2,963221167	4,28E+00	3,87E-02	2,33E-01	Ren1	0,11	No
ENSMUSG00000070661	-3,51E-01	-1,963178054	2,86E-01	5,93E-01	8,42E-01	Rnf186	0,78	No
ENSMUSG00000070667	-4,23E-01	-0,460559109	1,41E+00	2,36E-01	5,50E-01	Rpl31-ps10	0,75	No
ENSMUSG00000070683	1,24E+00	-2,770564607	9,59E-01	3,28E-01	6,28E-01	Lactbl1	2,37	No
ENSMUSG00000070690	1,50E-01	-1,627598021	6,83E-02	7,94E-01	9,56E-01	5830473C10Rik	1,11	No
ENSMUSG00000070691	-1,68E-01	6,688526369	7,39E+00	6,56E-03	6,85E-02	Runx3	0,89	No
ENSMUSG00000070692	-2,42E-01	0,900089984	1,28E+00	2,57E-01	5,50E-01	Rps12-ps10	0,85	No
ENSMUSG00000070697	-7,08E-02	5,739907616	1,11E+00	2,92E-01	5,88E-01	Utp3	0,95	No
ENSMUSG00000070699	1,33E-01	2,854242944	1,22E+00	2,70E-01	5,66E-01	Sars2	1,10	No
ENSMUSG00000070702	-1,87E+00	-3,107637761	1,39E+00	2,38E-01	5,50E-01	Csn1s1	0,27	No
ENSMUSG00000070705	-3,54E-02	2,411097566	7,18E-02	7,89E-01	9,54E-01	Eid2b	0,98	No
ENSMUSG00000070709	-5,99E-02	2,247120041	1,95E-01	6,58E-01	8,81E-01	Zfp974	0,96	No
ENSMUSG00000070713	1,37E-01	5,887487976	4,27E+00	3,88E-02	2,33E-01	Gm10282	1,10	No
ENSMUSG00000070717	-2,52E-02	-0,454664272	4,81E-03	9,45E-01	1,00E+00	Gm10300	0,98	No
ENSMUSG00000070719	-5,35E-01	1,650419227	1,10E+01	9,10E-04	1,50E-02	Pla2g4d	0,69	Yes
ENSMUSG00000070720	-7,41E-01	-2,548136224	6,15E-01	4,33E-01	7,26E-01	Tmem200b	0,60	No
ENSMUSG00000070729	-1,48E-01	7,4904196	6,24E+00	1,25E-02	1,10E-01	Gm12966	0,90	No
ENSMUSG00000070730	-5,78E-02	4,570083911	5,28E-01	4,68E-01	7,56E-01	Rmdn3	0,96	No
ENSMUSG00000070732	-1,15E-01	0,458157754	2,30E-01	6,31E-01	8,64E-01	Rbm44	0,92	No
ENSMUSG00000070733	-2,32E-02	6,869078623	1,44E-01	7,05E-01	9,07E-01	Fryl	0,98	No
ENSMUSG00000070737	-9,77E-02	3,670011383	1,06E+00	3,03E-01	5,99E-01	Tmem35b	0,93	No
ENSMUSG00000070738	7,44E-02	6,369863933	1,38E+00	2,41E-01	5,50E-01	Dgkd	1,05	No
ENSMUSG00000070778	2,65E+00	-2,896119006	2,71E+00	9,98E-02	3,92E-01	Rpl7a-ps8	6,28	No
ENSMUSG00000070780	1,04E-01	5,353960341	2,18E+00	1,40E-01	4,73E-01	Rbm47	1,07	No
ENSMUSG00000070797	-2,74E+00	-3,033596063	2,99E+00	8,38E-02	3,71E-01	Psg27	0,15	No
ENSMUSG00000070803	8,77E-02	1,74478111	3,00E-01	5,84E-01	8,38E-01	Cited4	1,06	No
ENSMUSG00000070806	-6,06E-02	-2,770638974	2,49E-03	9,60E-01	1,00E+00	Zmynd12	0,96	No
ENSMUSG00000070808	6,82E-02	5,141218239	8,90E-01	3,46E-01	6,47E-01	Bicra	1,05	No
ENSMUSG00000070814	2,55E-02	1,60283841	2,44E-02	8,76E-01	1,00E+00	6330408A02Rik	1,02	No
ENSMUSG00000070821	1,85E+00	-3,107657832	1,35E+00	2,46E-01	5,50E-01	Olfr1340	3,61	No
ENSMUSG00000070822	-6,44E-01	-0,006499409	4,66E+00	3,10E-02	2,03E-01	Zscan18	0,64	No
ENSMUSG00000070834	4,33E-01	-0,641326375	1,29E+00	2,56E-01	5,50E-01	Gm13015	1,35	No

ENSMUSG00000070844	-1,87E+00	-3,107637761	1,39E+00	2,38E-01	5,50E-01	Vmn2r42	0,27	No
ENSMUSG00000070847	-8,15E-01	-2,863692811	3,64E-01	5,46E-01	8,14E-01	Vmn2r30	0,57	No
ENSMUSG00000070858	-8,07E-02	-1,655230023	1,67E-02	8,97E-01	1,00E+00	Gm1673	0,95	No
ENSMUSG00000070867	2,64E+00	-3,033673223	2,69E+00	1,01E-01	3,92E-01	Trabd2b	6,23	No
ENSMUSG00000070868	7,36E-01	0,330590726	6,87E+00	8,77E-03	8,48E-02	Skint3	1,67	No
ENSMUSG00000070870	-1,89E+00	-3,1076441	1,42E+00	2,33E-01	5,50E-01	Cryge	0,27	No
ENSMUSG00000070871	7,49E-02	3,874688784	6,74E-01	4,12E-01	7,07E-01	Ccnyl1	1,05	No
ENSMUSG00000070873	3,47E-01	2,435762473	6,58E+00	1,03E-02	9,56E-02	Lilra5	1,27	No
ENSMUSG00000070877	1,17E+00	-1,863839301	2,31E+00	1,29E-01	4,50E-01	Ldlrad1	2,24	No
ENSMUSG00000070880	-2,63E-02	-3,033641358	2,33E-04	9,88E-01	1,00E+00	Gad1	0,98	No
ENSMUSG00000070883	-1,96E-01	-0,006958646	4,24E-01	5,15E-01	7,96E-01	Ccdc173	0,87	No
ENSMUSG00000070891	-1,18E-01	-0,64153748	9,96E-02	7,52E-01	9,32E-01	Gm12689	0,92	No
ENSMUSG00000070896	3,14E-02	-3,107617333	-7,69E-10	1,00E+00	1,00E+00	Rpl23a-ps1	1,02	No
ENSMUSG00000070900	3,58E-01	-0,41919954	1,08E+00	2,98E-01	5,94E-01	Gm10306	1,28	No
ENSMUSG00000070904	1,85E+00	-3,107657832	1,35E+00	2,46E-01	5,50E-01	Ifna4	3,61	No
ENSMUSG00000070908	-7,13E-01	-2,655115042	5,68E-01	4,51E-01	7,41E-01	Gm13288	0,61	No
ENSMUSG00000070923	2,55E-01	5,807640898	1,48E+01	1,22E-04	2,86E-03	Klhl9	1,19	Yes
ENSMUSG00000070934	1,73E-01	5,93622456	6,83E+00	8,95E-03	8,60E-02	Rraga	1,13	No
ENSMUSG00000070939	3,78E-02	6,154461256	3,43E-01	5,58E-01	8,22E-01	Tgfbrap1	1,03	No
ENSMUSG00000070942	1,95E-01	6,199165595	9,20E+00	2,42E-03	3,26E-02	Il1rl2	1,14	Yes
ENSMUSG00000070953	-6,39E-02	3,535399736	4,11E-01	5,21E-01	8,00E-01	Rabepk	0,96	No
ENSMUSG00000070960	3,84E-01	3,185675598	1,25E+01	4,14E-04	7,84E-03	Gm19680	1,30	Yes
ENSMUSG00000070972	1,45E-01	4,043618857	2,64E+00	1,04E-01	3,96E-01	Dnajc25	1,11	No
ENSMUSG00000070979	8,94E-01	-1,770558319	1,76E+00	1,84E-01	5,48E-01	Actl7a	1,86	No
ENSMUSG00000070980	8,10E-01	-2,600690781	5,71E-01	4,50E-01	7,40E-01	Actl7b	1,75	No
ENSMUSG00000070990	1,85E+00	-3,107657832	1,35E+00	2,46E-01	5,50E-01	Foxe1	3,61	No
ENSMUSG00000070997	-3,95E-02	-0,473098436	1,28E-02	9,10E-01	1,00E+00	1700055D18Rik	0,97	No
ENSMUSG00000070999	-8,88E-01	1,013396574	1,98E+01	8,39E-06	2,87E-04	Ccin	0,54	Yes
ENSMUSG00000071014	-1,33E-01	4,507989852	2,74E+00	9,78E-02	3,90E-01	Ndufb6	0,91	No
ENSMUSG00000071033	-4,85E-01	-2,974942494	9,25E-02	7,61E-01	9,37E-01	Gm10308	0,71	No
ENSMUSG00000071035	5,49E-03	4,752814594	5,05E-03	9,43E-01	1,00E+00	Gm5499	1,00	No
ENSMUSG00000071036	-6,44E-02	-2,770648468	1,80E-03	9,66E-01	1,00E+00	Gm10309	0,96	No

ENSMUSG00000071037	4,53E-02	0,694369611	3,49E-02	8,52E-01	9,90E-01	Camkmt	1,03	No
ENSMUSG00000071040	-6,19E-01	-2,033744279	7,44E-01	3,88E-01	6,88E-01	Gm9386	0,65	No
ENSMUSG00000071041	9,07E-03	6,534882297	2,10E-02	8,85E-01	1,00E+00	Impdh2-ps	1,01	No
ENSMUSG00000071042	-8,00E-02	2,014719978	2,94E-01	5,88E-01	8,40E-01	Rasgrp3	0,95	No
ENSMUSG00000071052	-1,82E-01	4,559554825	5,26E+00	2,18E-02	1,59E-01	Rpl7a-ps5	0,88	No
ENSMUSG00000071054	-1,40E-02	6,491080843	4,95E-02	8,24E-01	9,73E-01	Safb	0,99	No
ENSMUSG00000071064	-9,73E-01	1,753415484	3,86E+01	5,14E-10	4,39E-08	Zfp827	0,51	Yes
ENSMUSG00000071068	-7,25E-01	6,211008669	1,27E+02	1,77E-29	9,49E-27	Treml2	0,61	Yes
ENSMUSG00000071072	2,08E-01	5,816586185	9,78E+00	1,76E-03	2,52E-02	Ptges3	1,16	Yes
ENSMUSG00000071073	1,56E-01	-0,413144309	2,08E-01	6,48E-01	8,75E-01	Lrrc73	1,11	No
ENSMUSG00000071074	6,56E-02	5,85978794	9,77E-01	3,23E-01	6,23E-01	Yipf3	1,05	No
ENSMUSG00000071076	1,97E-01	7,789362621	1,12E+01	8,02E-04	1,35E-02	Jund	1,15	Yes
ENSMUSG00000071078	4,02E-03	4,19520528	2,23E-03	9,62E-01	1,00E+00	Nr2c2ap	1,00	No
ENSMUSG00000071089	-3,20E+00	-2,655163116	4,32E+00	3,76E-02	2,30E-01	Trim75	0,11	No
ENSMUSG00000071102	-7,25E-02	-0,006877568	5,47E-02	8,15E-01	9,69E-01	Gm10313	0,95	No
ENSMUSG00000071103	6,64E-01	-1,37836308	1,70E+00	1,93E-01	5,50E-01	1700029J07Rik	1,58	No
ENSMUSG00000071112	-6,91E-02	-0,755475743	2,57E-02	8,73E-01	1,00E+00	Spx	0,95	No
ENSMUSG00000071113	-1,40E+00	-2,896110316	1,23E+00	2,67E-01	5,63E-01	Mboat4	0,38	No
ENSMUSG00000071141	-1,56E-01	6,16283634	5,87E+00	1,54E-02	1,28E-01	Rpl36a-ps3	0,90	No
ENSMUSG00000071151	-7,82E-02	5,627584609	1,32E+00	2,50E-01	5,50E-01	Gm4799	0,95	No
ENSMUSG00000071172	5,54E-02	7,372813119	8,66E-01	3,52E-01	6,53E-01	Srsf3	1,04	No
ENSMUSG00000071176	-4,34E-01	-0,718767026	1,22E+00	2,70E-01	5,66E-01	Arhgef10	0,74	No
ENSMUSG00000071180	1,28E-01	5,876452435	3,72E+00	5,39E-02	2,84E-01	Smim15	1,09	No
ENSMUSG00000071181	-3,81E-01	0,010936691	1,72E+00	1,90E-01	5,50E-01	3830408C21Rik	0,77	No
ENSMUSG00000071192	5,78E-02	2,734881157	2,16E-01	6,42E-01	8,71E-01	Wfikkn1	1,04	No
ENSMUSG00000071202	5,98E-02	0,240465859	5,00E-02	8,23E-01	9,73E-01	Ccdc78	1,04	No
ENSMUSG00000071203	1,45E-01	5,807463173	4,76E+00	2,91E-02	1,94E-01	Naip5	1,11	No
ENSMUSG00000071207	1,00E-01	0,560459322	1,75E-01	6,75E-01	8,91E-01	Gm8587	1,07	No
ENSMUSG00000071226	-8,23E-02	-0,485318362	5,04E-02	8,22E-01	9,72E-01	Cecr2	0,94	No
ENSMUSG00000071229	-9,38E-01	-1,896068382	2,46E+00	1,16E-01	4,23E-01	Timm8a2	0,52	No
ENSMUSG00000071230	1,81E+00	-3,10765737	1,28E+00	2,58E-01	5,50E-01	Npw	3,49	No
ENSMUSG00000071237	2,02E-01	-1,333315464	1,66E-01	6,84E-01	8,96E-01	Gemin6-ps	1,15	No

ENSMUSG00000071252	1,02E-01	0,932084959	2,24E-01	6,36E-01	8,67E-01	2210408I21Rik	1,07	No
ENSMUSG00000071253	1,18E-01	4,530427063	2,16E+00	1,42E-01	4,76E-01	Slc25a16	1,09	No
ENSMUSG00000071256	-4,72E-02	2,500758964	1,33E-01	7,16E-01	9,13E-01	Zfp213	0,97	No
ENSMUSG00000071262	-1,38E-01	0,221787533	2,61E-01	6,09E-01	8,51E-01	Zfp957	0,91	No
ENSMUSG00000071266	-2,59E-02	3,404014537	6,41E-02	8,00E-01	9,60E-01	Zfp946	0,98	No
ENSMUSG00000071267	-7,28E-02	4,263661488	7,58E-01	3,84E-01	6,85E-01	Zfp942	0,95	No
ENSMUSG00000071273	8,56E-02	2,843470342	5,27E-01	4,68E-01	7,56E-01	Gm5145	1,06	No
ENSMUSG00000071280	-1,94E+00	-3,107619243	1,50E+00	2,21E-01	5,50E-01	Gm10322	0,26	No
ENSMUSG00000071281	3,22E-01	3,563514155	1,11E+01	8,41E-04	1,40E-02	Zfp65	1,25	Yes
ENSMUSG00000071291	3,52E-01	1,553725766	4,31E+00	3,79E-02	2,31E-01	Zfp58	1,28	No
ENSMUSG00000071294	-2,99E+00	-2,655119895	3,63E+00	5,67E-02	2,93E-01	Cbx3-ps4	0,13	No
ENSMUSG00000071302	4,93E-02	2,390598356	1,46E-01	7,03E-01	9,06E-01	2610044O15Rik8	1,03	No
ENSMUSG00000071303	4,98E-02	4,749520936	4,20E-01	5,17E-01	7,98E-01	Rps8-ps1	1,04	No
ENSMUSG00000071311	-6,48E-01	-1,770597373	1,06E+00	3,03E-01	6,00E-01	Gpr31b	0,64	No
ENSMUSG00000071317	-8,71E-01	-2,831957839	4,16E-01	5,19E-01	7,98E-01	Bves	0,55	No
ENSMUSG00000071322	-5,57E-02	-2,0701431	5,76E-03	9,40E-01	1,00E+00	Tcp10a	0,96	No
ENSMUSG00000071324	-1,31E-01	0,934373943	3,99E-01	5,28E-01	8,04E-01	Armc2	0,91	No
ENSMUSG00000071332	1,17E+00	-2,033713631	2,33E+00	1,27E-01	4,47E-01	E230015B07Rik	2,25	No
ENSMUSG00000071335	-1,90E-01	-0,006882198	4,53E-01	5,01E-01	7,86E-01	Mfsd4b3	0,88	No
ENSMUSG00000071337	-3,31E-02	5,122289271	2,13E-01	6,45E-01	8,73E-01	Tia1	0,98	No
ENSMUSG00000071340	-7,61E-02	-2,401462629	5,22E-03	9,42E-01	1,00E+00	Trappc3l	0,95	No
ENSMUSG00000071341	-4,16E+00	-2,600560606	8,81E+00	3,00E-03	3,83E-02	Egr4	0,06	Yes
ENSMUSG00000071342	8,10E-01	-2,355574068	5,72E-01	4,49E-01	7,40E-01	Lsmem1	1,75	No
ENSMUSG00000071343	-1,86E-02	3,52617539	3,56E-02	8,50E-01	9,90E-01	Gm10327	0,99	No
ENSMUSG00000071347	-1,36E+00	-2,896086315	1,16E+00	2,81E-01	5,78E-01	C1qtnf9	0,39	No
ENSMUSG00000071350	-1,40E-01	4,356572219	2,90E+00	8,84E-02	3,79E-01	Setdb2	0,91	No
ENSMUSG00000071359	-8,96E-02	4,41386094	1,21E+00	2,72E-01	5,68E-01	Tbpl1	0,94	No
ENSMUSG00000071369	2,82E-01	4,11002359	1,05E+01	1,21E-03	1,90E-02	Map3k5	1,22	Yes
ENSMUSG00000071379	1,37E-01	6,950375612	5,07E+00	2,43E-02	1,72E-01	Hpcal1	1,10	No
ENSMUSG00000071392	3,35E-01	-2,65513271	1,15E-01	7,35E-01	9,24E-01	Ect2l	1,26	No
ENSMUSG00000071398	-5,75E-04	0,705199053	5,77E-06	9,98E-01	1,00E+00	2410004P03Rik	1,00	No
ENSMUSG00000071414	7,92E-02	4,819732356	1,07E+00	3,00E-01	5,96E-01	Gm6736	1,06	No

ENSMUSG00000071415	-1,34E-01	5,103807171	3,39E+00	6,58E-02	3,22E-01	Rpl23	0,91	No
ENSMUSG00000071419	-2,16E-01	7,908375701	1,36E+01	2,22E-04	4,72E-03	Rps15-ps2	0,86	Yes
ENSMUSG00000071424	1,81E+00	-3,10765737	1,28E+00	2,58E-01	5,50E-01	Grid2	3,49	No
ENSMUSG00000071434	-1,87E+00	-3,107637761	1,39E+00	2,38E-01	5,50E-01	9230019H11Rik	0,27	No
ENSMUSG00000071451	-2,54E-01	3,666713698	7,13E+00	7,58E-03	7,60E-02	Psmg4	0,84	No
ENSMUSG00000071454	-2,72E-01	2,062670654	3,53E+00	6,03E-02	3,04E-01	Dtnb	0,83	No
ENSMUSG00000071456	1,10E-02	2,082479874	5,62E-03	9,40E-01	1,00E+00	1110002L01Rik	1,01	No
ENSMUSG00000071477	-2,51E-02	4,184513922	8,63E-02	7,69E-01	9,42E-01	Zfp777	0,98	No
ENSMUSG00000071478	-2,92E-01	-0,980410919	4,72E-01	4,92E-01	7,78E-01	Hist1h2ad	0,82	No
ENSMUSG00000071489	2,67E+00	-3,033636951	2,76E+00	9,68E-02	3,88E-01	Ptgdr	6,36	No
ENSMUSG00000071494	1,81E+00	-3,10765737	1,28E+00	2,58E-01	5,50E-01	Olfr455	3,49	No
ENSMUSG00000071497	5,72E-02	4,846473197	5,64E-01	4,52E-01	7,42E-01	Nutf2-ps1	1,04	No
ENSMUSG00000071506	7,67E-01	-2,963261668	3,24E-01	5,69E-01	8,30E-01	Tmem139	1,70	No
ENSMUSG00000071510	1,12E+00	-2,311104714	1,19E+00	2,76E-01	5,73E-01	Olfr172	2,17	No
ENSMUSG00000071516	-4,15E-01	-1,785618436	4,29E-01	5,12E-01	7,95E-01	Hist1h2ai	0,75	No
ENSMUSG00000071528	-1,10E-01	1,021364434	2,92E-01	5,89E-01	8,41E-01	Usmg5	0,93	No
ENSMUSG00000071531	-2,08E+00	-2,655102051	3,28E+00	7,02E-02	3,35E-01	Gprin2	0,24	No
ENSMUSG00000071532	-1,33E-01	7,114051681	4,84E+00	2,78E-02	1,88E-01	Gm10335	0,91	No
ENSMUSG00000071533	-1,15E-01	5,895050756	3,03E+00	8,20E-02	3,68E-01	Pcnp	0,92	No
ENSMUSG00000071537	4,20E-01	3,840397359	1,97E+01	8,88E-06	3,02E-04	Klrg2	1,34	Yes
ENSMUSG00000071547	2,67E-01	3,869309975	8,30E+00	3,96E-03	4,70E-02	Nt5dc2	1,20	Yes
ENSMUSG00000071550	-1,89E+00	-3,1076441	1,42E+00	2,33E-01	5,50E-01	Cfap44	0,27	No
ENSMUSG00000071551	-2,70E+00	-2,963266517	2,89E+00	8,90E-02	3,79E-01	Akr1c19	0,15	No
ENSMUSG00000071552	-7,08E-02	-1,832002152	1,40E-02	9,06E-01	1,00E+00	Tigit	0,95	No
ENSMUSG00000071553	-2,07E-01	-1,655016721	1,25E-01	7,24E-01	9,17E-01	Cpa2	0,87	No
ENSMUSG00000071561	-2,82E+00	-2,711642336	5,19E+00	2,27E-02	1,64E-01	BC100530	0,14	No
ENSMUSG00000071562	-2,11E-01	-1,800899902	1,09E-01	7,42E-01	9,27E-01	Stfa1	0,86	No
ENSMUSG00000071568	6,40E-01	1,48611016	1,28E+01	3,50E-04	6,88E-03	Gm5874	1,56	Yes
ENSMUSG00000071573	-2,91E-02	1,976903731	3,80E-02	8,46E-01	9,87E-01	Rnls	0,98	No
ENSMUSG00000071586	3,04E-01	-1,401476339	3,43E-01	5,58E-01	8,22E-01	Gm10337	1,23	No
ENSMUSG00000071591	-1,87E+00	-3,107637761	1,39E+00	2,38E-01	5,50E-01	Gm10339	0,27	No
ENSMUSG00000071604	5,93E-02	-0,561149272	2,33E-02	8,79E-01	1,00E+00	Fam189a2	1,04	No

ENSMUSG00000071613	3,14E-02	-3,107617333	-7,69E-10	1,00E+00	1,00E+00	Gm10340	1,02	No
ENSMUSG00000071632	-1,27E-01	2,528530983	9,83E-01	3,21E-01	6,22E-01	2510002D24Rik	0,92	No
ENSMUSG00000071633	3,16E+00	-2,770590572	4,07E+00	4,37E-02	2,49E-01	Gm4952	8,94	No
ENSMUSG00000071636	-1,75E-01	-1,548228939	1,04E-01	7,47E-01	9,30E-01	Rimbp3	0,89	No
ENSMUSG00000071637	4,66E-01	4,698298835	3,61E+01	1,84E-09	1,45E-07	Cebpd	1,38	Yes
ENSMUSG00000071640	-2,00E-01	4,706111867	6,75E+00	9,37E-03	8,90E-02	Stxbp3-ps	0,87	No
ENSMUSG00000071644	3,26E-02	6,287223547	2,61E-01	6,10E-01	8,51E-01	Eef1g	1,02	No
ENSMUSG00000071645	8,33E-02	4,11484426	9,31E-01	3,35E-01	6,35E-01	Tut1	1,06	No
ENSMUSG00000071646	4,38E-02	7,181418612	5,30E-01	4,67E-01	7,55E-01	Mta2	1,03	No
ENSMUSG00000071647	1,01E-01	6,054585528	2,42E+00	1,20E-01	4,30E-01	Eml3	1,07	No
ENSMUSG00000071648	-4,04E-01	2,091668282	7,79E+00	5,27E-03	5,80E-02	Rom1	0,76	No
ENSMUSG00000071649	7,64E-03	5,244647089	1,15E-02	9,15E-01	1,00E+00	B3gat3	1,01	No
ENSMUSG00000071650	6,50E-02	7,652286012	1,22E+00	2,69E-01	5,66E-01	Ganab	1,05	No
ENSMUSG00000071652	8,51E-02	5,30253076	1,46E+00	2,28E-01	5,50E-01	Ints5	1,06	No
ENSMUSG00000071653	3,39E-02	2,529000708	6,88E-02	7,93E-01	9,56E-01	1810009A15Rik	1,02	No
ENSMUSG00000071654	-1,04E-01	4,353321806	1,59E+00	2,07E-01	5,50E-01	Uqcc3	0,93	No
ENSMUSG00000071655	-1,35E-01	6,214300204	4,43E+00	3,53E-02	2,22E-01	Ubxn1	0,91	No
ENSMUSG00000071656	-7,09E-01	-1,088616669	2,19E+00	1,39E-01	4,71E-01	Lrrn4cl	0,61	No
ENSMUSG00000071657	1,56E-01	5,339974619	4,90E+00	2,68E-02	1,84E-01	Bscl2	1,11	No
ENSMUSG00000071658	2,97E-01	0,652571046	1,61E+00	2,04E-01	5,50E-01	Gng3	1,23	No
ENSMUSG00000071659	2,99E-02	8,119772848	2,67E-01	6,06E-01	8,49E-01	Hnrnpul2	1,02	No
ENSMUSG00000071660	-1,12E-01	5,55799054	2,72E+00	9,90E-02	3,92E-01	Ttc9c	0,93	No
ENSMUSG00000071661	5,49E-01	0,526652801	5,30E+00	2,14E-02	1,58E-01	Zbtb3	1,46	No
ENSMUSG00000071662	-1,34E-01	5,001773768	3,29E+00	6,95E-02	3,32E-01	Polr2g	0,91	No
ENSMUSG00000071669	2,26E-01	3,167787806	4,34E+00	3,72E-02	2,29E-01	Snx29	1,17	No
ENSMUSG00000071671	-5,69E-01	0,281316238	4,67E+00	3,07E-02	2,02E-01	Gm10343	0,67	No
ENSMUSG00000071691	-4,47E-01	-2,355577026	2,04E-01	6,52E-01	8,77E-01	Gm960	0,73	No
ENSMUSG00000071711	5,71E-02	4,590089985	5,18E-01	4,72E-01	7,60E-01	Mpst	1,04	No
ENSMUSG00000071713	-1,51E-01	11,74703014	7,35E+00	6,72E-03	6,98E-02	Csf2rb	0,90	No
ENSMUSG00000071714	-2,45E-01	10,67180788	1,91E+01	1,25E-05	4,09E-04	Csf2rb2	0,84	Yes
ENSMUSG00000071715	-1,52E-01	5,971349899	5,37E+00	2,05E-02	1,54E-01	Ncf4	0,90	No
ENSMUSG00000071716	-1,87E+00	-3,107637761	1,39E+00	2,38E-01	5,50E-01	Apol7e	0,27	No

ENSMUSG00000071724	-6,81E-01	0,024204048	5,67E+00	1,72E-02	1,37E-01	Smpd5		0,62	No
ENSMUSG00000071757	-3,16E-01	2,984359446	8,09E+00	4,45E-03	5,11E-02	Zhx2		0,80	No
ENSMUSG00000071793	-1,04E-01	4,340225165	1,59E+00	2,07E-01	5,50E-01	2610005L07Rik		0,93	No
ENSMUSG00000071796	-1,65E-01	5,193742523	5,24E+00	2,21E-02	1,61E-01	6820431F20Rik		0,89	No
ENSMUSG00000071847	-7,04E-01	-0,831925123	3,45E+00	6,34E-02	3,14E-01	Apcdd1		0,61	No
ENSMUSG00000071855	2,57E-03	2,055618379	3,12E-04	9,86E-01	1,00E+00	Ccdc112		1,00	No
ENSMUSG00000071856	2,25E-01	-2,522733744	6,37E-02	8,01E-01	9,61E-01	Mcc		1,17	No
ENSMUSG00000071862	-3,40E-01	-1,107535215	5,91E-01	4,42E-01	7,34E-01	Lrrtm2		0,79	No
ENSMUSG00000071866	-7,57E-02	7,604214563	1,64E+00	2,00E-01	5,50E-01	Ppia		0,95	No
ENSMUSG00000071867	9,23E-03	4,013797456	1,08E-02	9,17E-01	1,00E+00	Gm6304		1,01	No
ENSMUSG00000071893	2,17E-01	-2,548185163	5,87E-02	8,09E-01	9,65E-01	Vmn1r4		1,16	No
ENSMUSG00000071984	-2,70E+00	-3,033614577	2,89E+00	8,93E-02	3,79E-01	Fndc1		0,15	No
ENSMUSG00000072066	1,44E-01	2,697137427	1,44E+00	2,31E-01	5,50E-01	6720489N17Rik		1,10	No
ENSMUSG00000072082	2,33E-01	5,021976433	1,00E+01	1,53E-03	2,26E-02	Ccnf		1,18	Yes
ENSMUSG00000072109	-5,53E-01	4,361337039	4,48E+01	2,18E-11	2,22E-09	A530040E14Rik		0,68	Yes
ENSMUSG00000072115	3,57E-01	1,549587001	3,81E+00	5,09E-02	2,74E-01	Ang		1,28	No
ENSMUSG00000072214	2,51E-01	1,347877858	1,84E+00	1,75E-01	5,33E-01	38596		1,19	No
ENSMUSG00000072235	-6,84E-02	7,763554522	1,36E+00	2,43E-01	5,50E-01	Tuba1a		0,95	No
ENSMUSG00000072244	-2,57E-01	3,255226187	5,94E+00	1,48E-02	1,25E-01	Trim6		0,84	No
ENSMUSG00000072255	1,25E+00	-1,92936973	2,12E+00	1,45E-01	4,82E-01	Rpl31-ps17		2,38	No
ENSMUSG00000072258	4,15E-02	2,700018205	1,11E-01	7,39E-01	9,26E-01	Taf1a		1,03	No
ENSMUSG00000072294	-1,69E-01	-1,024372641	1,49E-01	7,00E-01	9,04E-01	Klf12		0,89	No
ENSMUSG00000072324	1,87E+00	-2,963248007	1,36E+00	2,44E-01	5,50E-01	Gm8420		3,65	No
ENSMUSG00000072381	3,98E-01	0,948012455	3,25E+00	7,14E-02	3,38E-01	Gm10355		1,32	No
ENSMUSG00000072387	2,30E-01	1,917954108	2,28E+00	1,31E-01	4,55E-01	Gm10356		1,17	No
ENSMUSG00000072407	-2,12E-02	4,004406646	5,71E-02	8,11E-01	9,66E-01	Gm6419		0,99	No
ENSMUSG00000072419	1,81E+00	-3,10765737	1,28E+00	2,58E-01	5,50E-01	Dppa2		3,49	No
ENSMUSG00000072421	2,25E-02	2,888861226	3,71E-02	8,47E-01	9,87E-01	Gm10357		1,02	No
ENSMUSG00000072423	1,62E-03	-2,963242114	-1,56E-12	1,00E+00	1,00E+00	Psmb11		1,00	No
ENSMUSG00000072437	3,41E-01	5,362802395	2,30E+01	1,64E-06	6,86E-05	Nanos1		1,27	Yes
ENSMUSG00000072460	6,40E-03	3,226507687	3,61E-03	9,52E-01	1,00E+00	Atp5l-ps1		1,00	No
ENSMUSG00000072476	1,24E-01	1,454899136	4,70E-01	4,93E-01	7,79E-01	Gm9008		1,09	No

ENSMUSG00000072487	-1,89E+00	-3,1076441	1,42E+00	2,33E-01	5,50E-01	Mroh5	0,27	No
ENSMUSG00000072492	-1,25E+00	-2,600641909	1,51E+00	2,20E-01	5,50E-01	Gm10364	0,42	No
ENSMUSG00000072494	2,22E-01	3,552993468	5,12E+00	2,36E-02	1,69E-01	Ppp1r3e	1,17	No
ENSMUSG00000072501	7,41E-02	6,467068111	1,39E+00	2,38E-01	5,50E-01	Phf20l1	1,05	No
ENSMUSG00000072541	2,34E-01	3,721860768	6,04E+00	1,40E-02	1,20E-01	Gm5046	1,18	No
ENSMUSG00000072553	-2,00E+00	-2,770572116	3,02E+00	8,23E-02	3,69E-01	Gm525	0,25	No
ENSMUSG00000072568	-4,69E-01	4,670766781	3,58E+01	2,20E-09	1,69E-07	Fam84b	0,72	Yes
ENSMUSG00000072571	2,19E-01	-0,683271252	3,63E-01	5,47E-01	8,15E-01	Tmem253	1,16	No
ENSMUSG00000072572	2,95E-01	5,382544008	1,76E+01	2,72E-05	8,08E-04	Slc39a2	1,23	Yes
ENSMUSG00000072573	-1,32E+00	-0,436559587	1,30E+01	3,07E-04	6,17E-03	Gm10369	0,40	Yes
ENSMUSG00000072575	-2,63E-02	-3,033653157	2,33E-04	9,88E-01	1,00E+00	Eddm3b	0,98	No
ENSMUSG00000072576	1,47E+00	-2,627629394	1,86E+00	1,73E-01	5,30E-01	Gm16020	2,78	No
ENSMUSG00000072582	-1,58E-03	3,904755951	3,02E-04	9,86E-01	1,00E+00	Ptrh2	1,00	No
ENSMUSG00000072584	1,81E+00	-3,10765737	1,28E+00	2,58E-01	5,50E-01	Gm7489	3,49	No
ENSMUSG00000072589	-3,83E-02	-3,107651936	-7,91E-11	1,00E+00	1,00E+00	Gm10371	0,97	No
ENSMUSG00000072591	2,64E+00	-2,896137133	2,70E+00	1,01E-01	3,92E-01	5930412G12Rik	6,24	No
ENSMUSG00000072592	2,60E+00	-2,963258458	2,63E+00	1,05E-01	3,97E-01	Gm10373	6,08	No
ENSMUSG00000072594	4,07E-01	3,590813574	1,68E+01	4,07E-05	1,14E-03	Gm16439	1,33	Yes
ENSMUSG00000072596	6,38E-01	6,512228729	1,00E+02	1,28E-23	4,12E-21	Ear2	1,56	Yes
ENSMUSG00000072598	1,79E+00	-3,033666867	1,28E+00	2,58E-01	5,50E-01	Ang6	3,46	No
ENSMUSG00000072599	6,18E-01	-1,980512631	7,73E-01	3,79E-01	6,80E-01	Ear-ps2	1,53	No
ENSMUSG00000072600	-2,34E+00	-2,929293556	2,15E+00	1,42E-01	4,77E-01	Ear-ps9	0,20	No
ENSMUSG00000072601	-1,22E+00	-0,641365038	1,03E+01	1,31E-03	2,01E-02	Ear1	0,43	Yes
ENSMUSG00000072612	-4,59E-01	2,75052573	1,36E+01	2,27E-04	4,82E-03	Gm10382	0,73	Yes
ENSMUSG00000072618	4,17E-01	0,087260143	2,12E+00	1,45E-01	4,83E-01	Gm10384	1,33	No
ENSMUSG00000072620	2,75E-02	8,314859042	2,26E-01	6,34E-01	8,66E-01	Slfn2	1,02	No
ENSMUSG00000072621	1,10E-01	4,184364774	1,69E+00	1,94E-01	5,50E-01	Slfn10-ps	1,08	No
ENSMUSG00000072623	3,62E-01	-0,070738602	1,51E+00	2,20E-01	5,50E-01	Zfp9	1,29	No
ENSMUSG00000072640	1,18E-01	2,601490699	9,22E-01	3,37E-01	6,37E-01	Lyrm9	1,09	No
ENSMUSG00000072647	1,35E-01	0,941404951	4,07E-01	5,23E-01	8,01E-01	Adam1a	1,10	No
ENSMUSG00000072653	3,55E-01	1,764632783	4,88E+00	2,72E-02	1,86E-01	Zfp783	1,28	No
ENSMUSG00000072660	-1,90E-01	-0,634488009	2,63E-01	6,08E-01	8,51E-01	Gm6288	0,88	No

ENSMUSG00000072662	-1,03E+00	-1,896010355	2,35E+00	1,25E-01	4,43E-01	Mansc4	0,49	No
ENSMUSG00000072663	2,20E-01	1,744763774	1,85E+00	1,74E-01	5,31E-01	Spef2	1,16	No
ENSMUSG00000072673	4,08E-01	1,239074627	4,40E+00	3,59E-02	2,25E-01	Gm10392	1,33	No
ENSMUSG00000072674	1,81E+00	-3,10765737	1,28E+00	2,58E-01	5,50E-01	Plac9b	3,49	No
ENSMUSG00000072675	1,81E+00	-3,10765737	1,28E+00	2,58E-01	5,50E-01	Duxbl2	3,49	No
ENSMUSG00000072676	-7,26E-02	2,464448394	3,06E-01	5,80E-01	8,37E-01	Tmem254a	0,95	No
ENSMUSG00000072679	1,87E+00	-3,033632735	1,36E+00	2,43E-01	5,50E-01	D6Ertd474e	3,66	No
ENSMUSG00000072680	-1,19E-01	2,532299722	8,45E-01	3,58E-01	6,59E-01	Tmem254c	0,92	No
ENSMUSG00000072683	7,20E-01	-1,669170076	1,57E+00	2,10E-01	5,50E-01	Gm7457	1,65	No
ENSMUSG00000072688	1,33E+00	-2,574230047	1,44E+00	2,30E-01	5,50E-01	Gm10399	2,51	No
ENSMUSG00000072692	-3,22E-01	8,47306789	3,14E+01	2,07E-08	1,34E-06	Rpl37rt	0,80	Yes
ENSMUSG00000072693	6,27E-03	2,725898475	2,65E-03	9,59E-01	1,00E+00	Gm10401	1,00	No
ENSMUSG00000072694	-8,52E-02	2,915522883	5,55E-01	4,56E-01	7,46E-01	1500011B03Rik	0,94	No
ENSMUSG00000072704	2,89E-02	5,431360627	1,73E-01	6,78E-01	8,92E-01	Smim10l1	1,02	No
ENSMUSG00000072707	1,81E+00	-2,963282131	1,30E+00	2,54E-01	5,50E-01	Olfr31	3,50	No
ENSMUSG00000072714	-4,54E-02	-2,107647014	4,27E-03	9,48E-01	1,00E+00	Rpl21-ps4	0,97	No
ENSMUSG00000072717	3,14E-02	-3,107617333	-7,69E-10	1,00E+00	1,00E+00	Klra11-ps	1,02	No
ENSMUSG00000072718	3,51E+00	-2,77064274	5,34E+00	2,08E-02	1,55E-01	Klra10	11,37	No
ENSMUSG00000072720	9,43E-02	-1,863852447	2,41E-02	8,77E-01	1,00E+00	Myo18b	1,07	No
ENSMUSG00000072721	-1,94E+00	-3,107619243	1,50E+00	2,21E-01	5,50E-01	Klra14-ps	0,26	No
ENSMUSG00000072722	-3,83E-02	-3,107651936	-7,91E-11	1,00E+00	1,00E+00	Gm6588	0,97	No
ENSMUSG00000072731	-7,90E-01	-2,574197874	8,04E-01	3,70E-01	6,71E-01	Gm3715	0,58	No
ENSMUSG00000072754	-2,69E+00	-2,963207085	2,87E+00	9,01E-02	3,80E-01	Lrcol1	0,16	No
ENSMUSG00000072761	-3,36E-01	0,575367624	1,99E+00	1,58E-01	5,05E-01	Gm6712	0,79	No
ENSMUSG00000072762	-1,92E-01	1,399740185	1,14E+00	2,85E-01	5,81E-01	4930522L14Rik	0,88	No
ENSMUSG00000072763	-3,71E-02	0,382248992	2,22E-02	8,82E-01	1,00E+00	5430403G16Rik	0,97	No
ENSMUSG00000072768	-7,38E-02	1,16610886	1,44E-01	7,04E-01	9,06E-01	Gm10418	0,95	No
ENSMUSG00000072769	-6,45E-02	-1,070125177	2,43E-02	8,76E-01	1,00E+00	Gm10419	0,96	No
ENSMUSG00000072770	5,27E-02	1,452891043	9,45E-02	7,59E-01	9,36E-01	Acrbp	1,04	No
ENSMUSG00000072772	-1,61E-01	4,251460895	3,68E+00	5,52E-02	2,88E-01	Grcc10	0,89	No
ENSMUSG00000072774	2,87E-01	2,103097357	4,14E+00	4,20E-02	2,44E-01	Zfp951	1,22	No
ENSMUSG00000072778	9,01E-03	-3,107646452	-5,16E-10	1,00E+00	1,00E+00	Vmn2r27	1,01	No

ENSMUSG00000072789	4,31E-03	4,666908695	3,01E-03	9,56E-01	1,00E+00	Gm10420	1,00	No
ENSMUSG00000072809	1,39E-01	3,074519117	1,56E+00	2,11E-01	5,50E-01	9330160F10Rik	1,10	No
ENSMUSG00000072812	3,16E-01	7,205236711	2,76E+01	1,53E-07	8,13E-06	Ahnak2	1,25	Yes
ENSMUSG00000072825	-5,71E-02	5,376944203	6,60E-01	4,17E-01	7,12E-01	Cep170b	0,96	No
ENSMUSG00000072837	1,01E-02	2,872313164	7,58E-03	9,31E-01	1,00E+00	Mir546	1,01	No
ENSMUSG00000072847	2,90E-01	3,259826999	7,56E+00	5,96E-03	6,38E-02	A530017D24Rik	1,22	No
ENSMUSG00000072852	-7,77E-02	-2,355645327	8,35E-03	9,27E-01	1,00E+00	2310040G07Rik	0,95	No
ENSMUSG00000072872	-1,75E-01	3,706348948	3,42E+00	6,46E-02	3,18E-01	Rybp	0,89	No
ENSMUSG00000072874	1,85E-01	-0,061002069	4,02E-01	5,26E-01	8,03E-01	Gm6116	1,14	No
ENSMUSG00000072875	7,29E-01	-2,832014431	2,89E-01	5,91E-01	8,41E-01	Gpr27	1,66	No
ENSMUSG00000072884	-1,04E+00	-1,770667114	2,75E+00	9,75E-02	3,90E-01	Gm10433	0,49	No
ENSMUSG00000072889	1,76E-01	4,031081244	4,03E+00	4,48E-02	2,52E-01	Nfxl1	1,13	No
ENSMUSG00000072893	9,33E-02	3,474115483	8,46E-01	3,58E-01	6,59E-01	4933439C10Rik	1,07	No
ENSMUSG00000072898	-3,00E-02	0,705177174	1,70E-02	8,96E-01	1,00E+00	Gm10434	0,98	No
ENSMUSG00000072902	-2,70E+00	-3,033614577	2,89E+00	8,93E-02	3,79E-01	Gm10435	0,15	No
ENSMUSG00000072905	-2,47E+00	-2,548242653	5,27E+00	2,17E-02	1,59E-01	Gm2016	0,18	No
ENSMUSG00000072915	-2,12E-01	0,569211556	8,35E-01	3,61E-01	6,62E-01	Gm12258	0,86	No
ENSMUSG00000072919	-2,21E-01	-0,541895141	4,01E-01	5,27E-01	8,03E-01	Noxred1	0,86	No
ENSMUSG00000072940	-1,82E-01	7,550595877	9,46E+00	2,10E-03	2,90E-02	Gm10443	0,88	Yes
ENSMUSG00000072941	-1,14E+00	0,152721795	1,86E+01	1,62E-05	5,14E-04	Sod3	0,45	Yes
ENSMUSG00000072946	-1,53E-02	4,807779552	4,04E-02	8,41E-01	9,84E-01	Ptgr2	0,99	No
ENSMUSG00000072949	7,26E-02	2,055928325	2,40E-01	6,24E-01	8,59E-01	Acot1	1,05	No
ENSMUSG00000072950	-1,92E+00	-3,033623267	1,46E+00	2,27E-01	5,50E-01	Gm10444	0,26	No
ENSMUSG00000072952	-3,83E-02	-3,107651936	-7,91E-11	1,00E+00	1,00E+00	Gm5878	0,97	No
ENSMUSG00000072962	1,20E+00	-3,146123991	6,74E-01	4,12E-01	7,07E-01	Gm16401	2,30	No
ENSMUSG00000072963	-1,92E+00	-2,963214917	1,46E+00	2,27E-01	5,50E-01	Gm10447	0,26	No
ENSMUSG00000072968	4,12E-01	-0,311359154	1,28E+00	2,58E-01	5,50E-01	Gm17728	1,33	No
ENSMUSG00000072972	5,32E-01	-0,627589987	1,93E+00	1,64E-01	5,16E-01	Adam4	1,45	No
ENSMUSG00000072974	3,53E-01	-0,801156946	8,04E-01	3,70E-01	6,71E-01	Gm4787	1,28	No
ENSMUSG00000072978	-1,26E-01	3,832361695	1,92E+00	1,66E-01	5,18E-01	Gm5830	0,92	No
ENSMUSG00000072980	3,34E-01	2,891036315	8,32E+00	3,92E-03	4,66E-02	Oip5	1,26	Yes
ENSMUSG00000072999	-6,83E-01	-2,627624449	4,45E-01	5,05E-01	7,89E-01	Gm15401	0,62	No

ENSMUSG00000073000	1,86E-01	0,214382889	4,84E-01	4,86E-01	7,74E-01	Gm10451	1,14	No
ENSMUSG00000073002	-1,13E-01	2,071121733	5,99E-01	4,39E-01	7,32E-01	Vamp5	0,92	No
ENSMUSG00000073018	-6,71E-01	-0,697254944	3,34E+00	6,77E-02	3,28E-01	Gm1070	0,63	No
ENSMUSG00000073067	2,08E-01	2,553746363	2,63E+00	1,05E-01	3,97E-01	9130019P16Rik	1,16	No
ENSMUSG00000073073	-3,36E-01	-1,946110422	2,88E-01	5,91E-01	8,42E-01	Gm8098	0,79	No
ENSMUSG00000073079	-6,94E-02	5,315572734	9,59E-01	3,28E-01	6,28E-01	Srp54a	0,95	No
ENSMUSG00000073096	3,06E-02	3,849183274	1,14E-01	7,36E-01	9,24E-01	Lrrc61	1,02	No
ENSMUSG00000073102	3,83E-01	-2,998009655	7,32E-02	7,87E-01	9,53E-01	Drc1	1,30	No
ENSMUSG00000073111	-1,87E+00	-3,107637761	1,39E+00	2,38E-01	5,50E-01	Olfr446	0,27	No
ENSMUSG00000073117	1,79E+00	-3,033666867	1,28E+00	2,58E-01	5,50E-01	Gm7347	3,46	No
ENSMUSG00000073119	1,87E+00	-3,107639691	1,38E+00	2,40E-01	5,50E-01	Speer4a	3,67	No
ENSMUSG00000073144	1,47E-01	2,612790154	1,32E+00	2,51E-01	5,50E-01	4930599N23Rik	1,11	No
ENSMUSG00000073145	-1,15E-01	-0,683027528	9,48E-02	7,58E-01	9,36E-01	Gm12000	0,92	No
ENSMUSG00000073147	-1,47E-01	3,382032517	2,07E+00	1,50E-01	4,92E-01	5031425E22Rik	0,90	No
ENSMUSG00000073154	1,04E+00	-2,929311517	4,67E-01	4,94E-01	7,80E-01	9330158H04Rik	2,06	No
ENSMUSG00000073155	-7,11E-02	6,174857982	1,22E+00	2,70E-01	5,66E-01	1810058I24Rik	0,95	No
ENSMUSG00000073158	-7,34E-01	-2,497593856	6,04E-01	4,37E-01	7,30E-01	9030624G23Rik	0,60	No
ENSMUSG00000073164	-3,68E-01	-2,800979556	8,35E-02	7,73E-01	9,44E-01	2410018L13Rik	0,77	No
ENSMUSG00000073174	1,25E+00	-2,37819723	1,53E+00	2,16E-01	5,50E-01	Gm29254	2,37	No
ENSMUSG00000073179	-4,49E-01	-1,311042927	9,12E-01	3,40E-01	6,40E-01	Gm10478	0,73	No
ENSMUSG00000073197	-5,38E-02	-1,896140246	6,74E-03	9,35E-01	1,00E+00	5730507C01Rik	0,96	No
ENSMUSG00000073198	4,78E-02	5,851550813	5,18E-01	4,72E-01	7,60E-01	Bnip3l-ps	1,03	No
ENSMUSG00000073209	-1,91E+00	-2,963238542	1,45E+00	2,29E-01	5,50E-01	Klf14	0,27	No
ENSMUSG00000073226	-1,75E-02	1,572674369	1,01E-02	9,20E-01	1,00E+00	Gm10482	0,99	No
ENSMUSG00000073233	9,33E-02	4,477371542	1,33E+00	2,49E-01	5,50E-01	Gm9144	1,07	No
ENSMUSG00000073234	-7,77E-01	0,160268829	8,65E+00	3,27E-03	4,09E-02	Gm8773	0,58	Yes
ENSMUSG00000073236	-2,18E-02	0,560433373	7,94E-03	9,29E-01	1,00E+00	2500004C02Rik	0,99	No
ENSMUSG00000073242	-2,66E-01	2,524213273	4,43E+00	3,53E-02	2,23E-01	Dnmt3aos	0,83	No
ENSMUSG00000073371	-1,92E+00	-3,0336536	1,46E+00	2,27E-01	5,50E-01	Gm6594	0,26	No
ENSMUSG00000073374	-1,60E-01	0,348440366	3,81E-01	5,37E-01	8,09E-01	C030034I22Rik	0,90	No
ENSMUSG00000073386	-2,97E-01	2,091552992	4,23E+00	3,96E-02	2,36E-01	9830107B12Rik	0,81	No
ENSMUSG00000073394	3,63E-01	1,47891449	3,77E+00	5,21E-02	2,78E-01	Runx2os1	1,29	No

ENSMUSG00000073399	1,63E+00	-2,655125983	1,81E+00	1,78E-01	5,39E-01	Trim40	3,09	No
ENSMUSG00000073400	-2,64E+00	-2,963219259	2,78E+00	9,55E-02	3,86E-01	Trim10	0,16	No
ENSMUSG00000073402	-3,37E-01	2,318158795	6,01E+00	1,42E-02	1,21E-01	Gm8909	0,79	No
ENSMUSG00000073403	-5,41E-01	-2,448641072	3,49E-01	5,54E-01	8,20E-01	Gm10499	0,69	No
ENSMUSG00000073405	2,90E-01	3,277130499	7,65E+00	5,68E-03	6,14E-02	H2-T-ps	1,22	No
ENSMUSG00000073406	1,31E-01	-1,980524426	4,31E-02	8,36E-01	9,80E-01	H2-Bl	1,10	No
ENSMUSG00000073407	-3,47E-02	-2,318585597	2,07E-03	9,64E-01	1,00E+00	Gm6034	0,98	No
ENSMUSG00000073409	-6,42E-01	6,3125018	1,02E+02	4,64E-24	1,51E-21	H2-Q6	0,64	Yes
ENSMUSG00000073411	-2,11E-01	10,44232098	1,42E+01	1,64E-04	3,66E-03	H2-D1	0,86	Yes
ENSMUSG00000073412	-1,16E-01	2,868152857	1,00E+00	3,17E-01	6,18E-01	Lst1	0,92	No
ENSMUSG00000073413	1,08E+00	-2,574265681	1,11E+00	2,92E-01	5,88E-01	Ly6g6d	2,12	No
ENSMUSG00000073414	-1,51E+00	-2,146065661	4,17E+00	4,12E-02	2,41E-01	G6b	0,35	No
ENSMUSG00000073415	-4,59E-01	-0,355474275	1,76E+00	1,85E-01	5,48E-01	Gm10501	0,73	No
ENSMUSG00000073418	-1,80E-01	-1,655102751	1,02E-01	7,50E-01	9,31E-01	C4b	0,88	No
ENSMUSG00000073421	-3,86E-02	11,43246137	4,78E-01	4,89E-01	7,76E-01	H2-Ab1	0,97	No
ENSMUSG00000073422	4,23E-02	2,394427826	9,78E-02	7,54E-01	9,34E-01	H2-Ke6	1,03	No
ENSMUSG00000073423	-9,65E-02	4,39869652	1,38E+00	2,40E-01	5,50E-01	Zfp414	0,94	No
ENSMUSG00000073424	-1,87E+00	-3,107637761	1,39E+00	2,38E-01	5,50E-01	Cyp4f15	0,27	No
ENSMUSG00000073427	-7,06E-02	1,54813673	1,75E-01	6,76E-01	8,91E-01	Gm4924	0,95	No
ENSMUSG00000073430	-9,26E-01	-2,60066999	7,51E-01	3,86E-01	6,87E-01	Gm10505	0,53	No
ENSMUSG00000073433	-6,03E-02	-0,855789142	2,07E-02	8,85E-01	1,00E+00	Arhgdig	0,96	No
ENSMUSG00000073434	8,42E-02	4,87489373	1,23E+00	2,67E-01	5,63E-01	Wdr90	1,06	No
ENSMUSG00000073435	-7,15E-02	0,749319525	1,00E-01	7,51E-01	9,32E-01	Nme3	0,95	No
ENSMUSG00000073436	6,60E-02	4,82451982	7,56E-01	3,85E-01	6,86E-01	Eme2	1,05	No
ENSMUSG00000073437	3,10E-02	4,107554805	1,28E-01	7,20E-01	9,15E-01	D330041H03Rik	1,02	No
ENSMUSG00000073448	-3,06E-02	0,502151233	1,59E-02	9,00E-01	1,00E+00	Gm10509	0,98	No
ENSMUSG00000073457	-1,89E+00	-3,1076441	1,42E+00	2,33E-01	5,50E-01	Smok2b	0,27	No
ENSMUSG00000073460	-3,68E-01	-0,366772885	1,22E+00	2,69E-01	5,65E-01	Pnldc1	0,77	No
ENSMUSG00000073468	4,13E-02	4,916264364	3,02E-01	5,83E-01	8,38E-01	Sft2d1	1,03	No
ENSMUSG00000073471	-2,54E-01	4,010587463	8,28E+00	4,01E-03	4,74E-02	Rsph3a	0,84	Yes
ENSMUSG00000073478	-3,71E-02	0,175773481	1,79E-02	8,93E-01	1,00E+00	D730003I15Rik	0,97	No
ENSMUSG00000073481	1,70E-01	5,348329299	5,82E+00	1,58E-02	1,31E-01	Marc2	1,13	No

ENSMUSG00000073482	-2,70E+00	-2,896043557	2,88E+00	8,95E-02	3,79E-01	Gm10517	0,15	No
ENSMUSG00000073485	-8,24E-02	-1,548284736	2,30E-02	8,79E-01	1,00E+00	H3f3aos	0,94	No
ENSMUSG00000073486	1,16E-01	0,71854832	2,68E-01	6,05E-01	8,49E-01	Gm10518	1,08	No
ENSMUSG00000073489	4,23E-03	5,181283448	3,40E-03	9,53E-01	1,00E+00	Ifi204	1,00	No
ENSMUSG00000073490	3,00E-02	6,622318957	2,33E-01	6,29E-01	8,62E-01	Ifi207	1,02	No
ENSMUSG00000073491	-2,66E-01	0,338069498	1,05E+00	3,05E-01	6,02E-01	Ifi213	0,83	No
ENSMUSG00000073492	2,94E-01	4,598682688	1,38E+01	2,07E-04	4,47E-03	Gm10521	1,23	Yes
ENSMUSG00000073494	2,93E-01	1,986012308	3,65E+00	5,62E-02	2,91E-01	Sh2d1b2	1,22	No
ENSMUSG00000073497	-5,25E-02	-3,033661434	4,08E-11	1,00E+00	1,00E+00	AA792892	0,96	No
ENSMUSG00000073514	3,14E-02	-3,107617333	-7,69E-10	1,00E+00	1,00E+00	Dok6	1,02	No
ENSMUSG00000073529	-1,57E-01	2,153418036	1,17E+00	2,79E-01	5,76E-01	F830208F22Rik	0,90	No
ENSMUSG00000073530	-4,90E-01	-1,770473795	5,81E-01	4,46E-01	7,37E-01	Pappa2	0,71	No
ENSMUSG00000073535	4,92E-01	-2,7116559	1,89E-01	6,64E-01	8,84E-01	Gm5532	1,41	No
ENSMUSG00000073538	-2,95E-02	3,373908657	8,64E-02	7,69E-01	9,42E-01	E330020D12Rik	0,98	No
ENSMUSG00000073542	1,31E-01	3,939169099	2,10E+00	1,47E-01	4,86E-01	Cep76	1,10	No
ENSMUSG00000073551	-1,87E+00	-3,107637761	1,39E+00	2,38E-01	5,50E-01	Spink13	0,27	No
ENSMUSG00000073554	8,56E-02	1,115741566	1,93E-01	6,61E-01	8,82E-01	Gm6960	1,06	No
ENSMUSG00000073555	2,13E-02	3,269274014	4,11E-02	8,39E-01	9,83E-01	Gm4951	1,01	No
ENSMUSG00000073557	5,60E-02	4,334973487	4,55E-01	5,00E-01	7,85E-01	Ppp1r12b	1,04	No
ENSMUSG00000073559	2,66E+00	-2,896142625	2,73E+00	9,86E-02	3,91E-01	Gm8618	6,32	No
ENSMUSG00000073563	5,69E-02	5,360850476	6,58E-01	4,17E-01	7,12E-01	Csnk1g3	1,04	No
ENSMUSG00000073565	1,81E+00	-3,10765737	1,28E+00	2,58E-01	5,50E-01	Prr16	3,49	No
ENSMUSG00000073568	-1,89E+00	-3,1076441	1,42E+00	2,33E-01	5,50E-01	Arl14epl	0,27	No
ENSMUSG00000073569	-6,37E-01	-1,424766502	1,49E+00	2,22E-01	5,50E-01	Gm10540	0,64	No
ENSMUSG00000073574	2,62E+00	-3,033654625	2,66E+00	1,03E-01	3,95E-01	Grxcr2	6,16	No
ENSMUSG00000073590	3,83E-01	0,458093068	2,33E+00	1,27E-01	4,46E-01	3222401L13Rik	1,30	No
ENSMUSG00000073591	-1,87E+00	-3,03364178	1,41E+00	2,36E-01	5,50E-01	Pcdhb22	0,27	No
ENSMUSG00000073598	5,19E-01	-2,051856889	5,67E-01	4,51E-01	7,41E-01	1700066B19Rik	1,43	No
ENSMUSG00000073599	-1,12E+00	-2,574205579	1,15E+00	2,84E-01	5,80E-01	Ecscr	0,46	No
ENSMUSG00000073600	-8,51E-01	-1,289354103	2,87E+00	9,04E-02	3,81E-01	Prob1	0,55	No
ENSMUSG00000073607	2,85E-02	-0,920956747	4,88E-03	9,44E-01	1,00E+00	Gm10548	1,02	No
ENSMUSG00000073608	-8,72E-01	-0,448515547	6,73E+00	9,49E-03	8,98E-02	Gal3st2c	0,55	No

ENSMUSG00000073609	1,10E-02	4,094507575	1,60E-02	8,99E-01	1,00E+00	D2hgdh	1,01	No
ENSMUSG00000073610	-1,87E+00	-3,107637761	1,39E+00	2,38E-01	5,50E-01	Gm10549	0,27	No
ENSMUSG00000073616	-1,63E-01	5,345910874	5,34E+00	2,08E-02	1,55E-01	Cops9	0,89	No
ENSMUSG00000073627	1,14E-02	-0,136408653	1,44E-03	9,70E-01	1,00E+00	C130036L24Rik	1,01	No
ENSMUSG00000073628	-3,84E-01	-0,278605926	1,52E+00	2,17E-01	5,50E-01	Gm10552	0,77	No
ENSMUSG00000073631	1,37E+00	-2,548229673	1,92E+00	1,66E-01	5,18E-01	Gm10553	2,58	No
ENSMUSG00000073633	2,43E-01	0,598614877	9,74E-01	3,24E-01	6,24E-01	Fbxo36	1,18	No
ENSMUSG00000073639	7,65E-02	6,376542252	1,46E+00	2,28E-01	5,50E-01	Rab18	1,05	No
ENSMUSG00000073640	-2,83E-01	7,321705979	2,24E+01	2,17E-06	8,74E-05	Rpl27-ps3	0,82	Yes
ENSMUSG00000073643	1,99E-02	5,97764757	9,21E-02	7,62E-01	9,38E-01	Wdfy1	1,01	No
ENSMUSG00000073647	-1,27E-01	5,117174986	3,02E+00	8,23E-02	3,69E-01	Gm10557	0,92	No
ENSMUSG00000073650	2,89E-01	-2,070213716	1,73E-01	6,77E-01	8,92E-01	Catip	1,22	No
ENSMUSG00000073652	3,21E-01	4,156789185	1,30E+01	3,04E-04	6,12E-03	Apol7d	1,25	Yes
ENSMUSG00000073664	-1,22E-02	5,538070808	3,11E-02	8,60E-01	9,95E-01	Nbeal1	0,99	No
ENSMUSG00000073676	-1,77E-01	4,655571433	5,15E+00	2,32E-02	1,67E-01	Hspe1	0,88	No
ENSMUSG00000073678	2,72E-01	6,073354792	1,74E+01	2,96E-05	8,65E-04	Pgap1	1,21	Yes
ENSMUSG00000073679	4,27E-01	-2,740810279	9,00E-02	7,64E-01	9,39E-01	Mxra8os	1,34	No
ENSMUSG00000073680	-7,08E-01	-2,31109151	8,51E-01	3,56E-01	6,57E-01	Tmem88b	0,61	No
ENSMUSG00000073682	-4,28E-01	-1,80105746	3,81E-01	5,37E-01	8,09E-01	Gm10563	0,74	No
ENSMUSG00000073684	-5,84E-02	4,023486757	4,45E-01	5,05E-01	7,89E-01	Faap20	0,96	No
ENSMUSG00000073700	2,61E-01	4,634213574	1,13E+01	7,64E-04	1,30E-02	Klhl21	1,20	Yes
ENSMUSG00000073702	-2,58E-01	3,211107344	5,89E+00	1,53E-02	1,27E-01	Rpl31	0,84	No
ENSMUSG00000073705	1,34E-01	2,19530012	8,69E-01	3,51E-01	6,53E-01	Apitd1	1,10	No
ENSMUSG00000073716	-1,44E-02	2,775752751	1,44E-02	9,04E-01	1,00E+00	Gm13241	0,99	No
ENSMUSG00000073722	-3,95E-02	-2,770598474	1,06E-03	9,74E-01	1,00E+00	4931408C20Rik	0,97	No
ENSMUSG00000073725	1,04E-01	5,705803776	2,35E+00	1,25E-01	4,43E-01	Lmbrd1	1,07	No
ENSMUSG00000073728	-2,80E-01	-0,157437922	8,68E-01	3,52E-01	6,53E-01	Tmem51os1	0,82	No
ENSMUSG00000073731	-6,50E-02	0,993140457	9,79E-02	7,54E-01	9,34E-01	AI507597	0,96	No
ENSMUSG00000073733	-1,32E-01	-0,929319642	1,08E-01	7,43E-01	9,28E-01	Rsg1	0,91	No
ENSMUSG00000073737	1,15E-01	4,367511718	1,97E+00	1,61E-01	5,09E-01	Gm10566	1,08	No
ENSMUSG00000073739	9,65E-02	0,937013822	2,09E-01	6,48E-01	8,75E-01	Gm16287	1,07	No
ENSMUSG00000073752	-5,69E-01	-2,627686938	2,54E-01	6,15E-01	8,53E-01	Gm10570	0,67	No

ENSMUSG00000073753	2,25E-01	-2,40141646	6,34E-02	8,01E-01	9,61E-01	Gm10571	1,17	No
ENSMUSG00000073755	-1,37E-01	3,727958439	2,15E+00	1,42E-01	4,77E-01	5730409E04Rik	0,91	No
ENSMUSG00000073758	-5,53E-01	1,959979759	1,36E+01	2,24E-04	4,77E-03	Sh3d21	0,68	Yes
ENSMUSG00000073761	-1,99E+00	-2,770603275	2,99E+00	8,38E-02	3,71E-01	4933427I04Rik	0,25	No
ENSMUSG00000073771	2,57E-01	3,935799473	7,81E+00	5,18E-03	5,74E-02	Btbd19	1,20	No
ENSMUSG00000073774	-3,60E+00	-2,448584818	5,82E+00	1,59E-02	1,31E-01	Kncn	0,08	No
ENSMUSG00000073775	-2,15E-02	3,805440617	5,33E-02	8,17E-01	9,70E-01	Kti12	0,99	No
ENSMUSG00000073778	7,46E-02	1,297278263	1,60E-01	6,90E-01	8,99E-01	Faddos	1,05	No
ENSMUSG00000073779	1,04E+00	-2,311269694	1,73E+00	1,88E-01	5,50E-01	Lrp8os2	2,05	No
ENSMUSG00000073787	2,45E-01	2,94332557	4,55E+00	3,29E-02	2,12E-01	Gm10575	1,19	No
ENSMUSG00000073791	3,48E-01	-0,024985448	1,35E+00	2,45E-01	5,50E-01	Efcab7	1,27	No
ENSMUSG00000073792	-6,08E-03	3,319875727	3,47E-03	9,53E-01	1,00E+00	Alg6	1,00	No
ENSMUSG00000073795	-1,22E-01	1,110486351	3,65E-01	5,46E-01	8,14E-01	6430531B16Rik	0,92	No
ENSMUSG00000073797	-8,56E-01	-2,832011372	4,01E-01	5,26E-01	8,03E-01	Gm12650	0,55	No
ENSMUSG00000073802	-5,41E-01	1,974844313	1,37E+01	2,09E-04	4,51E-03	Cdkn2b	0,69	Yes
ENSMUSG00000073803	-1,87E+00	-3,107637761	1,39E+00	2,38E-01	5,50E-01	4930544L04Rik	0,27	No
ENSMUSG00000073805	-2,81E-01	-1,574274012	2,57E-01	6,12E-01	8,52E-01	Fam196a	0,82	No
ENSMUSG00000073807	1,87E+00	-3,033632735	1,36E+00	2,43E-01	5,50E-01	Gm10581	3,66	No
ENSMUSG00000073809	-7,01E-02	-2,998026428	1,57E-03	9,68E-01	1,00E+00	Gm10583	0,95	No
ENSMUSG00000073821	-5,15E-01	-1,548193266	9,36E-01	3,33E-01	6,34E-01	8030451A03Rik	0,70	No
ENSMUSG00000073838	1,67E-02	3,887875235	3,40E-02	8,54E-01	9,92E-01	Tufm	1,01	No
ENSMUSG00000073843	2,52E-01	-0,195810141	6,69E-01	4,13E-01	7,09E-01	Gm12542	1,19	No
ENSMUSG00000073846	9,01E-03	-3,107646452	-5,16E-10	1,00E+00	1,00E+00	Gm12526	1,01	No
ENSMUSG00000073856	3,63E-01	-0,485356684	1,17E+00	2,79E-01	5,76E-01	Iqck	1,29	No
ENSMUSG00000073860	3,14E-02	-3,107617333	-7,69E-10	1,00E+00	1,00E+00	Gm829	1,02	No
ENSMUSG00000073867	-6,56E-01	-0,175615656	4,01E+00	4,51E-02	2,53E-01	AA474408	0,63	No
ENSMUSG00000073876	4,32E-01	2,541171791	1,11E+01	8,55E-04	1,42E-02	Gm13305	1,35	Yes
ENSMUSG00000073877	1,24E+00	-2,770593692	9,48E-01	3,30E-01	6,31E-01	Gm13306	2,36	No
ENSMUSG00000073888	1,09E-01	-1,015823106	6,93E-02	7,92E-01	9,55E-01	Ccl27a	1,08	No
ENSMUSG00000073889	2,45E-01	5,650643227	1,29E+01	3,27E-04	6,50E-03	Il11ra1	1,19	Yes
ENSMUSG00000073893	3,14E-02	-3,107617333	-7,69E-10	1,00E+00	1,00E+00	Olfr472	1,02	No
ENSMUSG00000073901	-3,83E-02	-3,107651936	-7,91E-11	1,00E+00	1,00E+00	Olfr703	0,97	No

ENSMUSG00000073902	4,11E-01	6,395019733	4,21E+01	8,77E-11	8,37E-09	Gm1966	1,33	Yes
ENSMUSG00000073910	1,73E-01	5,604443647	6,34E+00	1,18E-02	1,05E-01	Mob3b	1,13	No
ENSMUSG00000073926	9,01E-03	-3,107646452	-5,16E-10	1,00E+00	1,00E+00	Olfr653	1,01	No
ENSMUSG00000073927	1,21E-02	-3,033650469	7,34E-11	1,00E+00	1,00E+00	Olfr652	1,01	No
ENSMUSG00000073940	-8,48E-01	1,469979191	2,52E+01	5,09E-07	2,42E-05	Hbb-bt	0,56	Yes
ENSMUSG00000073968	5,28E-02	3,529640535	2,92E-01	5,89E-01	8,41E-01	Trim68	1,04	No
ENSMUSG00000073969	-1,87E+00	-3,03364178	1,41E+00	2,36E-01	5,50E-01	Olfr556	0,27	No
ENSMUSG00000073973	-2,63E-02	-3,033641358	2,33E-04	9,88E-01	1,00E+00	Olfr552	0,98	No
ENSMUSG00000073975	-1,89E+00	-3,1076441	1,42E+00	2,33E-01	5,50E-01	Olfr550	0,27	No
ENSMUSG00000073980	5,29E-01	-0,929280608	1,60E+00	2,06E-01	5,50E-01	Gm9574	1,44	No
ENSMUSG00000073982	-3,37E-02	7,807723888	3,31E-01	5,65E-01	8,27E-01	Rhog	0,98	No
ENSMUSG00000073985	-2,54E-01	2,308041078	3,43E+00	6,40E-02	3,16E-01	Gm10602	0,84	No
ENSMUSG00000073987	4,44E-02	5,837291777	4,43E-01	5,05E-01	7,90E-01	Ggh	1,03	No
ENSMUSG00000073988	4,91E-01	1,51440354	7,76E+00	5,34E-03	5,87E-02	Ttpa	1,41	No
ENSMUSG00000073995	1,52E-01	-2,655160083	2,24E-02	8,81E-01	1,00E+00	Gm10604	1,11	No
ENSMUSG00000074001	-1,87E+00	-3,107637761	1,39E+00	2,38E-01	5,50E-01	Klhl40	0,27	No
ENSMUSG00000074004	1,04E-01	-1,401381167	4,58E-02	8,31E-01	9,77E-01	B3gnt6	1,08	No
ENSMUSG00000074006	6,60E-01	0,480431317	7,16E+00	7,45E-03	7,51E-02	Omp	1,58	No
ENSMUSG00000074024	-2,17E-01	0,078947749	6,38E-01	4,24E-01	7,19E-01	4632427E13Rik	0,86	No
ENSMUSG00000074026	1,51E-01	2,738673225	1,52E+00	2,18E-01	5,50E-01	Gm6091	1,11	No
ENSMUSG00000074028	2,82E-01	-2,574280063	8,09E-02	7,76E-01	9,46E-01	Slc22a13	1,22	No
ENSMUSG00000074030	2,06E-01	4,22201953	6,07E+00	1,38E-02	1,18E-01	Exoc8	1,15	No
ENSMUSG00000074034	2,84E-02	5,046826943	1,49E-01	7,00E-01	9,04E-01	Gm5921	1,02	No
ENSMUSG00000074037	4,31E-01	-2,77058342	1,45E-01	7,03E-01	9,06E-01	Mc1r	1,35	No
ENSMUSG00000074039	-7,23E-01	4,156795779	7,57E+01	3,27E-18	6,49E-16	4930520O04Rik	0,61	Yes
ENSMUSG00000074052	-1,53E+00	-2,711675362	1,54E+00	2,15E-01	5,50E-01	BC048644	0,35	No
ENSMUSG00000074063	-3,03E-01	4,788972328	1,44E+01	1,45E-04	3,30E-03	Osgin1	0,81	Yes
ENSMUSG00000074064	1,18E-01	4,276481586	1,99E+00	1,59E-01	5,06E-01	Mlycd	1,08	No
ENSMUSG00000074065	1,87E+00	-3,033632735	1,36E+00	2,43E-01	5,50E-01	Gm10617	3,66	No
ENSMUSG00000074071	-8,04E-01	2,320297629	3,68E+01	1,34E-09	1,07E-07	Fam169b	0,57	Yes
ENSMUSG00000074088	7,05E-03	5,003629268	8,96E-03	9,25E-01	1,00E+00	Snrnp40	1,00	No
ENSMUSG00000074092	-2,39E-01	3,68596279	6,25E+00	1,24E-02	1,10E-01	Gm6181	0,85	No

ENSMUSG00000074093	-1,83E-02	3,285476212	3,14E-02	8,59E-01	9,95E-01	Svip	0,99	No
ENSMUSG00000074102	7,95E-03	5,778281123	1,41E-02	9,06E-01	1,00E+00	Rbm15b	1,01	No
ENSMUSG00000074108	-2,81E-01	3,27291008	7,19E+00	7,31E-03	7,41E-02	Rpl10-ps2	0,82	No
ENSMUSG00000074111	-5,51E-02	-2,831986244	1,37E-03	9,71E-01	1,00E+00	Mrgpra9	0,96	No
ENSMUSG00000074113	4,79E-02	-2,963178421	-8,19E-10	1,00E+00	1,00E+00	Gm10629	1,03	No
ENSMUSG00000074115	-1,87E+00	-3,107637761	1,39E+00	2,38E-01	5,50E-01	Saa1	0,27	No
ENSMUSG00000074121	-2,69E+00	-2,831970912	2,88E+00	8,99E-02	3,80E-01	Ntf5	0,15	No
ENSMUSG00000074127	1,16E+00	-2,514138736	1,31E+00	2,53E-01	5,50E-01	Cmtm2a	2,24	No
ENSMUSG00000074129	-7,62E-02	5,040219309	1,07E+00	3,02E-01	5,98E-01	Rpl13a	0,95	No
ENSMUSG00000074136	1,24E-02	1,165712942	3,89E-03	9,50E-01	1,00E+00	4930513N10Rik	1,01	No
ENSMUSG00000074141	-6,98E-01	7,175262399	1,35E+02	2,95E-31	1,80E-28	Il4i1	0,62	Yes
ENSMUSG00000074146	-3,75E-02	0,760830297	2,85E-02	8,66E-01	9,99E-01	4930579C12Rik	0,97	No
ENSMUSG00000074149	-4,01E-01	1,4421551	5,03E+00	2,49E-02	1,74E-01	Gm10634	0,76	No
ENSMUSG00000074151	-8,39E-02	6,233032929	1,73E+00	1,88E-01	5,50E-01	Nlrc5	0,94	No
ENSMUSG00000074158	-4,03E-02	1,360669794	4,79E-02	8,27E-01	9,75E-01	Zfp976	0,97	No
ENSMUSG00000074164	2,29E+00	-2,998039595	2,01E+00	1,56E-01	5,01E-01	Vmn2r-ps54	4,91	No
ENSMUSG00000074165	-1,04E-01	3,468618814	1,08E+00	2,98E-01	5,94E-01	Zfp788	0,93	No
ENSMUSG00000074166	-2,96E-01	3,036519038	7,17E+00	7,43E-03	7,49E-02	AW146154	0,81	No
ENSMUSG00000074170	1,37E-01	3,272315799	1,77E+00	1,84E-01	5,47E-01	Plekhf1	1,10	No
ENSMUSG00000074171	-9,85E-01	-0,816305159	6,17E+00	1,30E-02	1,13E-01	Gm6658	0,51	No
ENSMUSG00000074178	2,03E-01	-1,697398857	1,05E-01	7,45E-01	9,29E-01	Gm10638	1,15	No
ENSMUSG00000074182	7,44E-02	2,447063229	3,27E-01	5,67E-01	8,29E-01	Znhit6	1,05	No
ENSMUSG00000074183	2,64E+00	-3,033673223	2,69E+00	1,01E-01	3,92E-01	Gsta1	6,23	No
ENSMUSG00000074184	3,14E-02	-3,107617333	-7,69E-10	1,00E+00	1,00E+00	Smt3h2-ps2	1,02	No
ENSMUSG00000074194	1,33E+00	-1,33323684	5,16E+00	2,31E-02	1,66E-01	Zfp791	2,51	No
ENSMUSG00000074196	-2,71E+00	-3,033620915	2,92E+00	8,75E-02	3,78E-01	Clca4c-ps	0,15	No
ENSMUSG00000074203	5,40E-01	1,238311454	7,64E+00	5,71E-03	6,17E-02	G430095P16Rik	1,45	No
ENSMUSG00000074207	-4,85E-01	-2,896130679	1,19E-01	7,31E-01	9,21E-01	Adh1	0,71	No
ENSMUSG00000074210	-3,79E-01	2,987578511	1,16E+01	6,76E-04	1,17E-02	E130208F15Rik	0,77	Yes
ENSMUSG00000074211	-3,81E-01	3,661583109	1,65E+01	4,95E-05	1,35E-03	Sdhaf1	0,77	Yes
ENSMUSG00000074212	1,66E-01	7,051898455	7,51E+00	6,15E-03	6,53E-02	Dnajb14	1,12	No
ENSMUSG00000074213	2,23E-01	2,507881378	3,03E+00	8,18E-02	3,67E-01	Gm10642	1,17	No

ENSMUSG00000074215	1,94E-01	-0,778164668	2,16E-01	6,42E-01	8,71E-01	Gm10643	1,14	No
ENSMUSG00000074217	3,13E-01	-1,165785716	4,43E-01	5,06E-01	7,90E-01	2210011C24Rik	1,24	No
ENSMUSG00000074218	1,32E-01	0,167870883	2,25E-01	6,35E-01	8,67E-01	Cox7a1	1,10	No
ENSMUSG00000074219	1,37E-01	-0,740852096	1,09E-01	7,41E-01	9,27E-01	Gm10644	1,10	No
ENSMUSG00000074220	2,61E-01	3,33088392	6,21E+00	1,27E-02	1,11E-01	Zfp382	1,20	No
ENSMUSG00000074221	1,02E-01	5,797375739	2,34E+00	1,26E-01	4,44E-01	Zfp568	1,07	No
ENSMUSG00000074224	-1,87E+00	-3,107637761	1,39E+00	2,38E-01	5,50E-01	4932431P20Rik	0,27	No
ENSMUSG00000074227	-6,98E-01	5,092142887	9,23E+01	7,30E-22	2,01E-19	Spint2	0,62	Yes
ENSMUSG00000074228	3,34E-01	2,974986486	8,39E+00	3,77E-03	4,53E-02	Gm10645	1,26	Yes
ENSMUSG00000074232	-3,05E-01	-2,268115507	1,39E-01	7,09E-01	9,09E-01	Gm10647	0,81	No
ENSMUSG00000074236	-6,92E-03	3,701359924	5,38E-03	9,42E-01	1,00E+00	Gm9387	1,00	No
ENSMUSG00000074238	8,34E-02	5,314972173	1,40E+00	2,37E-01	5,50E-01	Ap1ar	1,06	No
ENSMUSG00000074240	-4,86E-01	-0,980695198	1,39E+00	2,38E-01	5,50E-01	Cib3	0,71	No
ENSMUSG00000074243	-1,95E-01	3,494953947	3,86E+00	4,93E-02	2,68E-01	Gm10651	0,87	No
ENSMUSG00000074247	1,31E-02	5,921131502	3,93E-02	8,43E-01	9,85E-01	Dda1	1,01	No
ENSMUSG00000074248	1,89E+00	-3,033614598	1,38E+00	2,40E-01	5,50E-01	4930432M17Rik	3,70	No
ENSMUSG00000074250	-3,39E-02	2,132076502	5,45E-02	8,15E-01	9,69E-01	Gm10653	0,98	No
ENSMUSG00000074252	1,56E-01	0,979803875	5,05E-01	4,77E-01	7,65E-01	Gm10654	1,11	No
ENSMUSG00000074254	-1,34E+00	-2,896147326	1,12E+00	2,89E-01	5,85E-01	Cyp2a4	0,39	No
ENSMUSG00000074256	-6,34E-01	-2,107603829	7,81E-01	3,77E-01	6,78E-01	Gm10655	0,64	No
ENSMUSG00000074259	-4,63E-01	2,28704397	1,22E+01	4,83E-04	8,92E-03	Gramd2	0,73	Yes
ENSMUSG00000074264	-6,11E-02	0,001912162	4,24E-02	8,37E-01	9,81E-01	Amy1	0,96	No
ENSMUSG00000074269	2,37E-01	2,036358579	2,41E+00	1,21E-01	4,33E-01	Rec114	1,18	No
ENSMUSG00000074272	-1,68E-01	5,816831062	6,32E+00	1,19E-02	1,06E-01	Ceacam1	0,89	No
ENSMUSG00000074274	-4,13E-02	-3,033659495	7,08E-04	9,79E-01	1,00E+00	D930028M14Rik	0,97	No
ENSMUSG00000074277	1,26E-01	1,217911259	4,31E-01	5,11E-01	7,94E-01	Phldb3	1,09	No
ENSMUSG00000074280	-1,93E-01	8,429614777	1,13E+01	7,84E-04	1,32E-02	Gm6166	0,87	Yes
ENSMUSG00000074281	-4,91E-01	-2,015665257	4,15E-01	5,20E-01	7,99E-01	Gm12522	0,71	No
ENSMUSG00000074282	6,85E-01	0,621325951	8,63E+00	3,31E-03	4,13E-02	Zfp94	1,61	Yes
ENSMUSG00000074283	7,38E-02	1,607012778	2,02E-01	6,53E-01	8,78E-01	Zfp109	1,05	No
ENSMUSG00000074284	-3,70E-01	2,303734681	7,46E+00	6,32E-03	6,67E-02	Gm10658	0,77	No
ENSMUSG00000074292	-1,89E+00	-3,1076441	1,42E+00	2,33E-01	5,50E-01	Gm10660	0,27	No

ENSMUSG00000074305	-3,30E-01	5,745644984	2,44E+01	7,94E-07	3,62E-05	Peak1	0,80	Yes
ENSMUSG00000074340	-1,49E-01	-0,233010272	2,18E-01	6,40E-01	8,70E-01	Ovgp1	0,90	No
ENSMUSG00000074342	7,95E-02	4,965973764	1,14E+00	2,85E-01	5,82E-01	I830077J02Rik	1,06	No
ENSMUSG00000074344	1,48E-01	2,402859585	1,23E+00	2,68E-01	5,64E-01	Tmigd3	1,11	No
ENSMUSG00000074345	6,78E-01	5,033150228	8,32E+01	7,27E-20	1,61E-17	Tnfaip8l3	1,60	Yes
ENSMUSG00000074354	2,34E-01	0,358729848	8,33E-01	3,61E-01	6,63E-01	Arhgap20os	1,18	No
ENSMUSG00000074355	-2,21E-01	1,578559406	1,72E+00	1,90E-01	5,50E-01	Gm10676	0,86	No
ENSMUSG00000074357	-5,81E-01	-0,748123553	2,06E+00	1,51E-01	4,93E-01	AA386476	0,67	No
ENSMUSG00000074358	5,74E-02	3,221052063	2,93E-01	5,88E-01	8,41E-01	Ccdc61	1,04	No
ENSMUSG00000074359	-3,83E-02	-3,107651936	-7,91E-11	1,00E+00	1,00E+00	Psg23	0,97	No
ENSMUSG00000074361	4,41E-01	1,273137152	5,12E+00	2,36E-02	1,68E-01	C5ar2	1,36	No
ENSMUSG00000074364	-1,55E+00	-2,107605586	3,46E+00	6,29E-02	3,13E-01	Ehd2	0,34	No
ENSMUSG00000074365	-1,38E+00	-1,378244054	6,90E+00	8,61E-03	8,36E-02	Crxos	0,38	No
ENSMUSG00000074373	-1,23E+00	-3,146117124	7,12E-01	3,99E-01	6,98E-01	Gm10680	0,42	No
ENSMUSG00000074384	4,89E-01	-0,561154575	1,88E+00	1,71E-01	5,26E-01	AI429214	1,40	No
ENSMUSG00000074385	-2,17E-01	4,451978238	7,27E+00	7,01E-03	7,19E-02	Gm10684	0,86	No
ENSMUSG00000074388	-4,71E-01	-1,574226725	7,71E-01	3,80E-01	6,81E-01	Gm5544	0,72	No
ENSMUSG00000074397	-6,01E-01	-2,18570088	4,92E-01	4,83E-01	7,70E-01	Foxr1	0,66	No
ENSMUSG00000074398	3,98E-04	6,193498327	3,82E-05	9,95E-01	1,00E+00	Gm15441	1,00	No
ENSMUSG00000074403	1,95E-02	-1,963243766	7,33E-04	9,78E-01	1,00E+00	Hist2h3b	1,01	No
ENSMUSG00000074405	-6,29E-02	5,064130588	7,42E-01	3,89E-01	6,89E-01	Zfp865	0,96	No
ENSMUSG00000074406	-3,29E-02	4,406682713	1,58E-01	6,91E-01	9,00E-01	Zfp628	0,98	No
ENSMUSG00000074412	-2,26E-01	1,17820702	1,33E+00	2,48E-01	5,50E-01	Gm10689	0,85	No
ENSMUSG00000074415	-4,39E-01	-2,655033231	1,96E-01	6,58E-01	8,81E-01	2610203C20Rik	0,74	No
ENSMUSG00000074417	2,45E-01	5,790283995	1,34E+01	2,52E-04	5,27E-03	Gm14548	1,19	Yes
ENSMUSG00000074418	2,25E-01	1,046986145	1,26E+00	2,61E-01	5,55E-01	Gm15930	1,17	No
ENSMUSG00000074419	4,81E-02	4,306027131	3,37E-01	5,62E-01	8,25E-01	Gm15448	1,03	No
ENSMUSG00000074420	1,81E-01	1,494020748	1,01E+00	3,15E-01	6,15E-01	Gm10693	1,13	No
ENSMUSG00000074424	-3,83E-02	-3,107651936	-7,91E-11	1,00E+00	1,00E+00	Gm10696	0,97	No
ENSMUSG00000074449	7,67E-01	-2,963261668	3,24E-01	5,69E-01	8,30E-01	Gm15319	1,70	No
ENSMUSG00000074452	-4,91E-01	-1,322143982	9,09E-01	3,40E-01	6,41E-01	Pate2	0,71	No
ENSMUSG00000074457	-8,47E-01	-2,831940177	3,94E-01	5,30E-01	8,05E-01	S100a16	0,56	No

ENSMUSG00000074461	-1,90E+00	-3,033594154	1,44E+00	2,30E-01	5,50E-01	Gm10699	0,27	No
ENSMUSG00000074465	1,42E+00	-1,574284917	6,84E+00	8,93E-03	8,58E-02	Gm10701	2,67	No
ENSMUSG00000074466	3,76E-01	1,420916667	4,20E+00	4,04E-02	2,38E-01	Gm15417	1,30	No
ENSMUSG00000074467	3,63E-01	-2,574216556	1,18E-01	7,31E-01	9,21E-01	Gm10702	1,29	No
ENSMUSG00000074472	-1,43E+00	-0,634193624	1,61E+01	6,01E-05	1,58E-03	Zfp872	0,37	Yes
ENSMUSG00000074476	1,74E-02	3,595949476	3,24E-02	8,57E-01	9,94E-01	Spc24	1,01	No
ENSMUSG00000074479	-1,64E-01	6,111807871	6,41E+00	1,13E-02	1,02E-01	Gm10704	0,89	No
ENSMUSG00000074480	-4,73E-01	-0,879874463	1,55E+00	2,13E-01	5,50E-01	Mex3a	0,72	No
ENSMUSG00000074482	1,49E-02	4,532572748	3,45E-02	8,53E-01	9,91E-01	Gm16589	1,01	No
ENSMUSG00000074483	-3,18E+00	-2,655092806	4,26E+00	3,90E-02	2,34E-01	Bglap	0,11	No
ENSMUSG00000074485	3,71E-01	-1,863762564	3,33E-01	5,64E-01	8,26E-01	Gm7381	1,29	No
ENSMUSG00000074486	-5,24E-02	-2,963245194	1,25E-03	9,72E-01	1,00E+00	Bglap2	0,96	No
ENSMUSG00000074489	3,14E-02	-3,107617333	-7,69E-10	1,00E+00	1,00E+00	Bglap3	1,02	No
ENSMUSG00000074491	6,33E-01	-1,366826168	1,48E+00	2,24E-01	5,50E-01	Clec4g	1,55	No
ENSMUSG00000074497	2,17E-01	1,152051475	1,23E+00	2,68E-01	5,65E-01	A430078G23Rik	1,16	No
ENSMUSG00000074500	-1,37E-01	-0,079634321	2,13E-01	6,44E-01	8,73E-01	Zfp558	0,91	No
ENSMUSG00000074501	-1,94E+00	-3,107619243	1,50E+00	2,21E-01	5,50E-01	Gm5612	0,26	No
ENSMUSG00000074502	-3,83E-02	-3,107651936	-7,91E-11	1,00E+00	1,00E+00	Ubtfl1	0,97	No
ENSMUSG00000074505	1,23E+00	2,247549397	6,73E+01	2,37E-16	4,13E-14	Fat3	2,35	Yes
ENSMUSG00000074506	-1,55E-01	5,330809937	4,78E+00	2,88E-02	1,93E-01	Gm10705	0,90	No
ENSMUSG00000074508	-4,42E+00	-2,333198126	1,07E+01	1,06E-03	1,70E-02	Gm10706	0,05	Yes
ENSMUSG00000074513	1,41E-01	4,825043035	3,44E+00	6,37E-02	3,15E-01	Arfip1	1,10	No
ENSMUSG00000074516	-2,64E-01	6,234751646	1,69E+01	3,91E-05	1,10E-03	Gm10709	0,83	Yes
ENSMUSG00000074519	-1,37E-01	3,638126402	2,08E+00	1,50E-01	4,91E-01	Zfp971	0,91	No
ENSMUSG00000074521	1,15E-01	-0,0200711	1,55E-01	6,94E-01	9,02E-01	Gm14327	1,08	No
ENSMUSG00000074527	3,38E-01	2,048105398	5,18E+00	2,28E-02	1,65E-01	Gm14296	1,26	No
ENSMUSG00000074529	-2,16E-01	-0,662118302	3,43E-01	5,58E-01	8,22E-01	Zfp972	0,86	No
ENSMUSG00000074547	-1,22E-01	4,774803781	2,54E+00	1,11E-01	4,08E-01	Rps8-ps4	0,92	No
ENSMUSG00000074553	-1,49E-01	4,218950375	3,09E+00	7,90E-02	3,60E-01	Eif1-ps1	0,90	No
ENSMUSG00000074555	5,92E-01	-2,65518408	3,89E-01	5,33E-01	8,06E-01	Gm10714	1,51	No
ENSMUSG00000074564	7,63E-01	-2,896140274	3,18E-01	5,73E-01	8,32E-01	Gm10720	1,70	No
ENSMUSG00000074569	-2,83E-01	-0,190721013	8,52E-01	3,56E-01	6,57E-01	Gcnt7	0,82	No

ENSMUSG00000074570	-1,51E-01	5,529302392	4,63E+00	3,14E-02	2,05E-01	Cass4	0,90	No
ENSMUSG00000074575	-9,43E-01	-2,711697975	7,78E-01	3,78E-01	6,78E-01	Kcng1	0,52	No
ENSMUSG00000074576	1,12E-01	3,026717908	1,01E+00	3,16E-01	6,16E-01	Mocs3	1,08	No
ENSMUSG00000074577	-9,57E-01	-0,655105255	6,33E+00	1,18E-02	1,06E-01	Ripor3	0,52	No
ENSMUSG00000074578	-4,98E-01	3,26026481	2,22E+01	2,44E-06	9,67E-05	Zfas1	0,71	Yes
ENSMUSG00000074579	-2,96E-01	0,496750594	1,40E+00	2,37E-01	5,50E-01	Lekr1	0,81	No
ENSMUSG00000074580	-3,84E-02	2,97232466	1,10E-01	7,40E-01	9,26E-01	4931440P22Rik	0,97	No
ENSMUSG00000074582	4,44E-02	6,822352565	5,23E-01	4,70E-01	7,58E-01	Arfgef2	1,03	No
ENSMUSG00000074591	-8,86E-02	-2,963249454	3,57E-03	9,52E-01	1,00E+00	Ankub1	0,94	No
ENSMUSG00000074594	-7,88E-02	-2,89616692	4,36E-03	9,47E-01	1,00E+00	Wfdc9	0,95	No
ENSMUSG00000074603	6,23E-01	-1,998031529	7,87E-01	3,75E-01	6,76E-01	Gm10729	1,54	No
ENSMUSG00000074604	4,19E-01	2,589114002	1,06E+01	1,10E-03	1,75E-02	Mgst2	1,34	Yes
ENSMUSG00000074607	-3,65E-01	-1,401372859	5,87E-01	4,44E-01	7,35E-01	Tox2	0,78	No
ENSMUSG00000074619	-7,13E-02	-1,627680916	1,66E-02	8,97E-01	1,00E+00	1700034I23Rik	0,95	No
ENSMUSG00000074620	-1,87E+00	-3,033619006	1,41E+00	2,36E-01	5,50E-01	Gm10731	0,27	No
ENSMUSG00000074622	4,54E-01	6,659191049	5,24E+01	4,55E-13	5,78E-11	Mafb	1,37	Yes
ENSMUSG00000074623	1,82E-01	-1,497591875	1,00E-01	7,52E-01	9,32E-01	Gm826	1,13	No
ENSMUSG00000074627	2,58E-01	1,202721677	1,70E+00	1,92E-01	5,50E-01	Mroh8	1,20	No
ENSMUSG00000074628	5,00E-01	-1,627730406	7,74E-01	3,79E-01	6,80E-01	Tldc2	1,41	No
ENSMUSG00000074629	1,92E-01	-0,436708893	3,36E-01	5,62E-01	8,25E-01	4930518I15Rik	1,14	No
ENSMUSG00000074634	1,80E-01	2,867318961	2,40E+00	1,21E-01	4,34E-01	Tmem267	1,13	No
ENSMUSG00000074635	-1,68E-01	-0,929198874	1,61E-01	6,89E-01	8,98E-01	3110070M22Rik	0,89	No
ENSMUSG00000074643	4,79E-02	5,056041276	4,30E-01	5,12E-01	7,95E-01	Cpne1	1,03	No
ENSMUSG00000074646	2,02E-01	2,682403126	2,71E+00	9,94E-02	3,92E-01	6430550D23Rik	1,15	No
ENSMUSG00000074647	-6,62E-01	-1,448615974	1,56E+00	2,12E-01	5,50E-01	Fam83c	0,63	No
ENSMUSG00000074649	2,53E-02	4,621312577	1,03E-01	7,49E-01	9,30E-01	BC029722	1,02	No
ENSMUSG00000074651	3,12E+00	-2,83205733	3,98E+00	4,60E-02	2,56E-01	Mcidas	8,72	No
ENSMUSG00000074652	2,20E-02	0,010779819	5,77E-03	9,39E-01	1,00E+00	Myh7b	1,02	No
ENSMUSG00000074653	-2,65E+00	-3,033633091	2,78E+00	9,55E-02	3,86E-01	Lrrc31	0,16	No
ENSMUSG00000074656	-7,57E-02	5,673524727	1,24E+00	2,66E-01	5,61E-01	Eif2s2	0,95	No
ENSMUSG00000074657	1,70E-01	2,651304375	1,98E+00	1,59E-01	5,06E-01	Kif5a	1,12	No
ENSMUSG00000074664	1,84E+00	-3,033661615	1,33E+00	2,48E-01	5,50E-01	A830092H15Rik	3,58	No

ENSMUSG00000074671	4,90E-02	3,210272869	2,18E-01	6,40E-01	8,70E-01	Tspyl3	1,03	No
ENSMUSG00000074673	-8,63E-02	-2,070194068	1,20E-02	9,13E-01	1,00E+00	Ttll9	0,94	No
ENSMUSG00000074677	5,34E-01	5,244071341	5,43E+01	1,70E-13	2,27E-11	Sirpb1c	1,45	Yes
ENSMUSG00000074680	1,84E+00	-3,033649193	1,33E+00	2,49E-01	5,50E-01	Defb26	3,58	No
ENSMUSG00000074682	-1,96E-01	2,57079295	2,36E+00	1,24E-01	4,41E-01	Zcchc3	0,87	No
ENSMUSG00000074695	-2,68E+00	-2,963261035	2,84E+00	9,17E-02	3,82E-01	Il22	0,16	No
ENSMUSG00000074698	-1,27E-01	6,398603547	4,05E+00	4,41E-02	2,50E-01	Csnk2a1	0,92	No
ENSMUSG00000074704	1,19E+00	-1,800981744	3,44E+00	6,38E-02	3,16E-01	Rad21l	2,28	No
ENSMUSG00000074715	-7,10E-02	-2,6551508	4,60E-03	9,46E-01	1,00E+00	Ccl28	0,95	No
ENSMUSG00000074731	8,87E-01	-2,268098875	1,21E+00	2,71E-01	5,68E-01	Zfp345	1,85	No
ENSMUSG00000074733	-7,90E-02	4,023410562	8,22E-01	3,65E-01	6,66E-01	Zfp950	0,95	No
ENSMUSG00000074734	-2,49E-02	-3,146121042	-4,44E-11	1,00E+00	1,00E+00	4933416C03Rik	0,98	No
ENSMUSG00000074735	5,57E-01	-0,413215671	2,84E+00	9,20E-02	3,82E-01	Gm21994	1,47	No
ENSMUSG00000074738	5,07E-02	2,509408131	1,52E-01	6,96E-01	9,03E-01	Fndc10	1,04	No
ENSMUSG00000074743	5,20E-01	2,023240104	1,18E+01	5,97E-04	1,06E-02	Thbd	1,43	Yes
ENSMUSG00000074746	-7,56E-03	6,379575565	1,42E-02	9,05E-01	1,00E+00	Pdzd8	0,99	No
ENSMUSG00000074748	-1,18E-02	6,272418709	3,42E-02	8,53E-01	9,91E-01	Atxn7l3b	0,99	No
ENSMUSG00000074749	-7,33E-02	2,817105561	3,93E-01	5,30E-01	8,05E-01	Kiz	0,95	No
ENSMUSG00000074754	-1,91E-01	1,986806185	1,58E+00	2,08E-01	5,50E-01	Smim26	0,88	No
ENSMUSG00000074758	7,49E-01	-1,448747098	1,70E+00	1,92E-01	5,50E-01	Gm5535	1,68	No
ENSMUSG00000074763	1,81E+00	-3,10765737	1,28E+00	2,58E-01	5,50E-01	Macrod2os2	3,49	No
ENSMUSG00000074768	1,87E+00	-3,107639691	1,38E+00	2,40E-01	5,50E-01	Bhmt	3,67	No
ENSMUSG00000074780	4,25E-02	3,558363429	1,87E-01	6,66E-01	8,85E-01	Anapc15-ps	1,03	No
ENSMUSG00000074781	3,98E-02	5,736528986	3,49E-01	5,55E-01	8,20E-01	Ube2n	1,03	No
ENSMUSG00000074782	-4,48E-03	-1,448725155	6,31E-05	9,94E-01	1,00E+00	4833422C13Rik	1,00	No
ENSMUSG00000074783	-1,37E+00	-2,146017442	2,59E+00	1,08E-01	4,02E-01	AU019990	0,39	No
ENSMUSG00000074785	-6,25E-02	7,82115973	1,14E+00	2,85E-01	5,82E-01	Plxnc1	0,96	No
ENSMUSG00000074793	3,46E-01	-0,55481914	8,70E-01	3,51E-01	6,52E-01	Hspa12b	1,27	No
ENSMUSG00000074794	1,50E-02	5,590023709	4,80E-02	8,27E-01	9,75E-01	Arrdc3	1,01	No
ENSMUSG00000074796	-2,32E-02	2,514319792	3,21E-02	8,58E-01	9,94E-01	Slc4a11	0,98	No
ENSMUSG00000074797	7,50E-02	4,022134316	7,22E-01	3,95E-01	6,96E-01	Itpa	1,05	No
ENSMUSG00000074800	-1,80E-01	7,812381871	9,43E+00	2,14E-03	2,94E-02	Gm4149	0,88	Yes

ENSMUSG00000074802	4,43E-01	6,180146989	4,70E+01	7,13E-12	7,69E-10	Gas2l3	1,36	Yes
ENSMUSG00000074804	-9,56E-01	-2,711709022	8,00E-01	3,71E-01	6,72E-01	AU015228	0,52	No
ENSMUSG00000074805	-3,78E-01	-1,006693149	7,22E-01	3,95E-01	6,96E-01	Il1bos	0,77	No
ENSMUSG00000074807	3,07E-01	0,520664618	1,56E+00	2,12E-01	5,50E-01	Gm10762	1,24	No
ENSMUSG00000074811	-1,21E-02	4,395436092	2,18E-02	8,83E-01	1,00E+00	Hps6	0,99	No
ENSMUSG00000074812	7,16E-01	-2,896139042	2,79E-01	5,97E-01	8,45E-01	Spdye4c	1,64	No
ENSMUSG00000074813	2,62E-01	3,127892302	5,37E+00	2,05E-02	1,54E-01	Gm14005	1,20	No
ENSMUSG00000074817	1,18E-01	1,995157616	6,09E-01	4,35E-01	7,28E-01	Papolb	1,08	No
ENSMUSG00000074818	1,53E-01	-0,126790857	2,45E-01	6,21E-01	8,57E-01	Pdzd7	1,11	No
ENSMUSG00000074824	9,35E-01	-0,438984632	7,43E+00	6,42E-03	6,74E-02	Rslcan18	1,91	No
ENSMUSG00000074825	2,35E-01	5,114234539	1,06E+01	1,13E-03	1,79E-02	Itpripl1	1,18	Yes
ENSMUSG00000074826	-8,97E-02	0,952554152	1,77E-01	6,74E-01	8,90E-01	Gm10767	0,94	No
ENSMUSG00000074829	-1,17E-01	4,032475694	1,79E+00	1,81E-01	5,42E-01	2010315B03Rik	0,92	No
ENSMUSG00000074841	3,14E-02	-3,107617333	-7,69E-10	1,00E+00	1,00E+00	Cbx3-ps1	1,02	No
ENSMUSG00000074846	1,13E-01	3,563332657	1,31E+00	2,53E-01	5,50E-01	Gm10774	1,08	No
ENSMUSG00000074847	-5,72E-01	-2,831971956	2,57E-01	6,12E-01	8,52E-01	Gm10775	0,67	No
ENSMUSG00000074852	-4,13E-02	-3,033659495	7,08E-04	9,79E-01	1,00E+00	Hpse2	0,97	No
ENSMUSG00000074862	-7,63E-02	-0,065523754	6,48E-02	7,99E-01	9,60E-01	BC025920	0,95	No
ENSMUSG00000074863	1,71E-01	3,443924149	3,01E+00	8,30E-02	3,70E-01	Platr25	1,13	No
ENSMUSG00000074865	1,01E-01	2,705508631	6,97E-01	4,04E-01	7,01E-01	Zfp934	1,07	No
ENSMUSG00000074867	2,67E-01	1,473616989	2,28E+00	1,31E-01	4,55E-01	Zfp808	1,20	No
ENSMUSG00000074868	-8,18E-02	1,11014883	1,72E-01	6,78E-01	8,92E-01	Gm6478	0,94	No
ENSMUSG00000074873	1,04E-01	2,932305107	8,13E-01	3,67E-01	6,68E-01	AI606181	1,07	No
ENSMUSG00000074874	3,09E-01	0,795911878	1,92E+00	1,65E-01	5,18E-01	Ctla2b	1,24	No
ENSMUSG00000074876	3,15E-01	3,140324934	8,24E+00	4,11E-03	4,82E-02	Spata5l1	1,24	Yes
ENSMUSG00000074881	-5,90E-01	-2,497581264	3,74E-01	5,41E-01	8,11E-01	Mageb3	0,66	No
ENSMUSG00000074884	-3,39E-02	7,098736686	3,16E-01	5,74E-01	8,33E-01	Serf2	0,98	No
ENSMUSG00000074885	-7,38E-02	-3,033659033	2,66E-03	9,59E-01	1,00E+00	Gm10782	0,95	No
ENSMUSG00000074886	-5,81E-02	6,190420547	8,15E-01	3,67E-01	6,68E-01	Grk6	0,96	No
ENSMUSG00000074887	-1,87E+00	-3,107637761	1,39E+00	2,38E-01	5,50E-01	EU599041	0,27	No
ENSMUSG00000074890	8,17E-02	3,319573574	6,11E-01	4,34E-01	7,27E-01	Lcmt2	1,06	No
ENSMUSG00000074892	7,47E-01	-2,146070568	9,97E-01	3,18E-01	6,19E-01	B3galt5	1,68	No

ENSMUSG00000074896	-3,49E-01	1,770600774	4,98E+00	2,57E-02	1,78E-01	Ifit3	0,79	No
ENSMUSG00000074899	-1,92E-01	1,36018635	9,11E-01	3,40E-01	6,41E-01	Sptbn5	0,88	No
ENSMUSG00000074903	-6,27E-02	2,363229721	2,17E-01	6,42E-01	8,71E-01	Gm2058	0,96	No
ENSMUSG00000074909	-3,63E-02	4,331074391	1,92E-01	6,61E-01	8,82E-01	Ranbp6	0,98	No
ENSMUSG00000074912	-1,20E+00	-1,863536666	2,87E+00	9,00E-02	3,80E-01	Gm14207	0,44	No
ENSMUSG00000074916	-7,77E-02	3,834815477	7,19E-01	3,97E-01	6,97E-01	Chst14	0,95	No
ENSMUSG00000074918	-3,14E-02	4,449906426	1,49E-01	6,99E-01	9,04E-01	Inafm2	0,98	No
ENSMUSG00000074922	1,07E-01	3,438754895	1,13E+00	2,88E-01	5,84E-01	Fam122a	1,08	No
ENSMUSG00000074923	-3,09E-01	-0,155680921	9,62E-01	3,27E-01	6,28E-01	Pak6	0,81	No
ENSMUSG00000074925	2,12E-01	2,838483219	3,20E+00	7,36E-02	3,45E-01	Ptar1	1,16	No
ENSMUSG00000074930	6,60E-01	-1,355742889	1,42E+00	2,33E-01	5,50E-01	Gm13981	1,58	No
ENSMUSG00000074934	-4,66E-01	-1,740843968	5,78E-01	4,47E-01	7,37E-01	Grem1	0,72	No
ENSMUSG00000074939	7,06E-01	-2,832018779	2,72E-01	6,02E-01	8,48E-01	Chrm5	1,63	No
ENSMUSG00000074955	1,87E+00	-3,107639691	1,38E+00	2,40E-01	5,50E-01	Olfr1305	3,67	No
ENSMUSG00000074968	-8,45E-01	-2,963241229	3,92E-01	5,31E-01	8,05E-01	Ano3	0,56	No
ENSMUSG00000074978	-1,06E-01	5,040654777	2,08E+00	1,49E-01	4,90E-01	Actg-ps1	0,93	No
ENSMUSG00000074987	-1,89E+00	-3,1076441	1,42E+00	2,33E-01	5,50E-01	Wt1os	0,27	No
ENSMUSG00000074994	-6,02E-02	5,253867339	7,24E-01	3,95E-01	6,95E-01	Qser1	0,96	No
ENSMUSG00000074997	-5,17E-04	2,238425092	1,41E-05	9,97E-01	1,00E+00	Pin1rt1	1,00	No
ENSMUSG00000074999	-4,95E-01	0,187840918	2,91E+00	8,79E-02	3,79E-01	Gm10797	0,71	No
ENSMUSG00000075000	-7,93E-02	4,328774258	9,20E-01	3,38E-01	6,38E-01	Nrbf2	0,95	No
ENSMUSG00000075010	-8,36E-01	6,214277545	1,69E+02	1,06E-38	1,18E-35	AW112010	0,56	Yes
ENSMUSG00000075012	2,72E-01	-2,497577868	7,51E-02	7,84E-01	9,51E-01	Fjx1	1,21	No
ENSMUSG00000075014	2,70E-01	0,56366804	1,43E+00	2,32E-01	5,50E-01	Gm10800	1,21	No
ENSMUSG00000075015	2,68E-01	-2,355555165	9,05E-02	7,64E-01	9,39E-01	Gm10801	1,20	No
ENSMUSG00000075020	9,01E-03	-3,107646452	-5,16E-10	1,00E+00	1,00E+00	Mir670hg	1,01	No
ENSMUSG00000075023	2,62E+00	-3,033654625	2,66E+00	1,03E-01	3,95E-01	Accsl	6,16	No
ENSMUSG00000075025	-2,52E-01	-1,333268932	2,52E-01	6,16E-01	8,54E-01	Gm10804	0,84	No
ENSMUSG00000075027	-4,44E-01	-1,963165853	3,63E-01	5,47E-01	8,14E-01	4631405J19Rik	0,74	No
ENSMUSG00000075028	-1,07E-01	2,665324602	7,83E-01	3,76E-01	6,77E-01	Prdm11	0,93	No
ENSMUSG00000075029	4,35E-05	-2,318609281	3,92E-09	1,00E+00	1,00E+00	4930558J22Rik	1,00	No
ENSMUSG00000075031	5,51E-02	-1,107673378	1,30E-02	9,09E-01	1,00E+00	Hist1h2bb	1,04	No

ENSMUSG00000075033	-8,39E-02	0,638370497	1,34E-01	7,14E-01	9,12E-01	Nxpe3	0,94	No
ENSMUSG00000075036	-7,21E-02	-2,832041172	3,55E-03	9,52E-01	1,00E+00	Gm10805	0,95	No
ENSMUSG00000075040	-1,53E-01	4,797170299	3,99E+00	4,56E-02	2,55E-01	Zfp408	0,90	No
ENSMUSG00000075042	6,90E-01	-2,226342	6,90E-01	4,06E-01	7,02E-01	4930431P03Rik	1,61	No
ENSMUSG00000075044	-1,87E+00	-3,107637761	1,39E+00	2,38E-01	5,50E-01	Slc22a29	0,27	No
ENSMUSG00000075053	6,66E-02	6,052410261	1,04E+00	3,07E-01	6,05E-01	Vdac3-ps1	1,05	No
ENSMUSG00000075054	1,26E-01	2,311838502	8,71E-01	3,51E-01	6,52E-01	Yae1d1	1,09	No
ENSMUSG00000075061	1,87E+00	-3,107639691	1,38E+00	2,40E-01	5,50E-01	Olfr1272	3,67	No
ENSMUSG00000075065	-1,31E+00	-2,03371007	3,59E+00	5,80E-02	2,97E-01	Olfr1270	0,40	No
ENSMUSG00000075069	1,87E+00	-3,107639691	1,38E+00	2,40E-01	5,50E-01	Olfr1264	3,67	No
ENSMUSG00000075122	-4,42E-01	6,577245624	5,00E+01	1,56E-12	1,88E-10	Cd80	0,74	Yes
ENSMUSG00000075162	2,36E-01	0,047992418	6,29E-01	4,28E-01	7,22E-01	Gm10812	1,18	No
ENSMUSG00000075184	1,17E+00	-1,655282729	3,78E+00	5,20E-02	2,78E-01	F930017D23Rik	2,24	No
ENSMUSG00000075224	-1,87E+00	-3,107637761	1,39E+00	2,38E-01	5,50E-01	Lrrc55	0,27	No
ENSMUSG00000075225	-6,45E-01	1,013130939	1,07E+01	1,06E-03	1,69E-02	Ccdc162	0,64	Yes
ENSMUSG00000075227	8,56E-02	4,667120549	1,20E+00	2,74E-01	5,71E-01	Znhit2	1,06	No
ENSMUSG00000075229	8,71E-02	2,880204444	5,51E-01	4,58E-01	7,47E-01	Ccdc58	1,06	No
ENSMUSG00000075232	2,12E-01	5,071446685	8,41E+00	3,72E-03	4,48E-02	Amd1	1,16	Yes
ENSMUSG00000075245	-6,73E-01	-0,879731266	2,60E+00	1,07E-01	4,01E-01	Gm6043	0,63	No
ENSMUSG00000075254	1,37E-01	4,802913451	3,25E+00	7,16E-02	3,39E-01	Heg1	1,10	No
ENSMUSG00000075256	-4,40E-01	3,465951293	1,95E+01	1,01E-05	3,41E-04	Cerkl	0,74	Yes
ENSMUSG00000075265	-1,21E+00	-2,600669775	1,66E+00	1,98E-01	5,50E-01	Tnk2os	0,43	No
ENSMUSG00000075266	-1,41E-01	2,292582487	1,04E+00	3,09E-01	6,07E-01	Cenpw	0,91	No
ENSMUSG00000075267	-3,15E-01	-1,574129194	2,78E-01	5,98E-01	8,45E-01	Dfnb59	0,80	No
ENSMUSG00000075268	1,07E-01	4,935800154	2,03E+00	1,54E-01	4,97E-01	Gm10819	1,08	No
ENSMUSG00000075269	1,68E-01	2,680296561	1,80E+00	1,79E-01	5,40E-01	Bex6	1,12	No
ENSMUSG00000075270	2,13E-01	-1,627706738	1,63E-01	6,86E-01	8,97E-01	Pde11a	1,16	No
ENSMUSG00000075271	2,27E-01	1,706574039	2,01E+00	1,56E-01	5,01E-01	Ttc30a1	1,17	No
ENSMUSG00000075272	9,80E-02	-0,436747634	7,36E-02	7,86E-01	9,52E-01	Ttc30a2	1,07	No
ENSMUSG00000075273	9,87E-02	1,813356331	3,75E-01	5,40E-01	8,10E-01	Ttc30b	1,07	No
ENSMUSG00000075276	-4,13E-02	1,704871442	6,29E-02	8,02E-01	9,61E-01	Gm13653	0,97	No
ENSMUSG00000075279	-1,46E-02	4,446442693	3,24E-02	8,57E-01	9,94E-01	Mrpl23-ps1	0,99	No

ENSMUSG00000075284	-4,11E-04	7,760762019	4,91E-05	9,94E-01	1,00E+00	Wipf1	1,00	No
ENSMUSG00000075286	-2,65E+00	-3,033633091	2,78E+00	9,55E-02	3,86E-01	Gm1968	0,16	No
ENSMUSG00000075289	-2,80E-01	2,680850292	5,32E+00	2,11E-02	1,57E-01	Carns1	0,82	No
ENSMUSG00000075296	1,85E+00	-3,107657832	1,35E+00	2,46E-01	5,50E-01	Aldh3b2	3,61	No
ENSMUSG00000075297	-2,27E-02	-0,097952744	5,63E-03	9,40E-01	1,00E+00	H60b	0,98	No
ENSMUSG00000075304	-1,86E+00	-3,033612668	1,39E+00	2,38E-01	5,50E-01	Sp5	0,28	No
ENSMUSG00000075307	-6,22E-02	-2,655111106	2,61E-03	9,59E-01	1,00E+00	Klhl41	0,96	No
ENSMUSG00000075312	-1,11E-01	4,949030473	2,22E+00	1,36E-01	4,65E-01	Gm13597	0,93	No
ENSMUSG00000075316	-1,98E-01	-1,702240106	9,93E-02	7,53E-01	9,33E-01	Scn9a	0,87	No
ENSMUSG00000075318	1,77E+00	-2,268128223	3,61E+00	5,75E-02	2,95E-01	Scn2a	3,40	No
ENSMUSG00000075325	1,21E-02	-3,033650469	7,34E-11	1,00E+00	1,00E+00	Gm13582	1,01	No
ENSMUSG00000075327	3,02E-03	4,50807584	1,42E-03	9,70E-01	1,00E+00	Zbtb2	1,00	No
ENSMUSG00000075334	-2,45E-01	-1,268059685	2,39E-01	6,25E-01	8,59E-01	Rprm	0,84	No
ENSMUSG00000075370	3,14E-02	-3,107617333	-7,69E-10	1,00E+00	1,00E+00	Igll1	1,02	No
ENSMUSG00000075376	1,43E-02	5,59440484	4,43E-02	8,33E-01	9,79E-01	Rc3h2	1,01	No
ENSMUSG00000075389	2,42E-01	-2,311129433	7,34E-02	7,86E-01	9,52E-01	2810410L24Rik	1,18	No
ENSMUSG00000075391	8,80E-02	4,185001208	1,05E+00	3,05E-01	6,01E-01	Gm13443	1,06	No
ENSMUSG00000075402	-1,13E-01	-2,963254935	5,86E-03	9,39E-01	1,00E+00	Krt76	0,92	No
ENSMUSG00000075405	1,64E+00	-2,963261751	1,31E+00	2,52E-01	5,50E-01	9430097D07Rik	3,11	No
ENSMUSG00000075408	-1,64E-01	-2,378253246	3,86E-02	8,44E-01	9,86E-01	6030408B16Rik	0,89	No
ENSMUSG00000075410	-6,19E-01	-1,980485984	5,22E-01	4,70E-01	7,58E-01	Prcd	0,65	No
ENSMUSG00000075415	-2,92E-01	8,640634302	2,59E+01	3,51E-07	1,73E-05	Fnbp1	0,82	Yes
ENSMUSG00000075416	4,45E-01	-2,600656537	1,54E-01	6,94E-01	9,02E-01	Gm14488	1,36	No
ENSMUSG00000075419	1,05E-01	4,018602814	1,40E+00	2,37E-01	5,50E-01	Dolk	1,08	No
ENSMUSG00000075420	2,36E-01	0,302553744	8,57E-01	3,55E-01	6,56E-01	Smim6	1,18	No
ENSMUSG00000075425	1,30E-01	-2,289458305	3,14E-02	8,59E-01	9,95E-01	Gm13547	1,09	No
ENSMUSG00000075463	-3,83E-01	-0,36118905	1,18E+00	2,77E-01	5,74E-01	4930594M22Rik	0,77	No
ENSMUSG00000075467	-1,72E-02	4,940076524	5,29E-02	8,18E-01	9,70E-01	Dnlz	0,99	No
ENSMUSG00000075469	-3,83E-02	-3,107651936	-7,91E-11	1,00E+00	1,00E+00	Gm17205	0,97	No
ENSMUSG00000075470	9,97E-03	6,109313355	2,37E-02	8,78E-01	1,00E+00	Alg10b	1,01	No
ENSMUSG00000075480	-6,43E-01	-2,711680239	2,76E-01	5,99E-01	8,46E-01	Gm10840	0,64	No
ENSMUSG00000075485	1,81E+00	-3,10765737	1,28E+00	2,58E-01	5,50E-01	Gm10842	3,49	No

ENSMUSG00000075486	-2,52E-02	4,646693848	1,04E-01	7,47E-01	9,30E-01	Commd6	0,98	No
ENSMUSG00000075502	6,02E-01	0,977515645	8,32E+00	3,91E-03	4,66E-02	Kbtbd6	1,52	Yes
ENSMUSG00000075511	1,01E-01	1,907315471	4,23E-01	5,16E-01	7,97E-01	1700001L05Rik	1,07	No
ENSMUSG00000075514	2,03E-01	1,319930444	1,20E+00	2,74E-01	5,71E-01	Gm13375	1,15	No
ENSMUSG00000075520	6,22E-01	-2,31107493	4,31E-01	5,11E-01	7,94E-01	Malrd1	1,54	No
ENSMUSG00000075525	1,04E+00	-2,963280965	4,69E-01	4,94E-01	7,79E-01	Gm10849	2,06	No
ENSMUSG00000075528	9,97E-02	2,959294389	7,96E-01	3,72E-01	6,74E-01	Aarsd1	1,07	No
ENSMUSG00000075534	7,56E-01	-2,800966083	3,11E-01	5,77E-01	8,35E-01	Gm13262	1,69	No
ENSMUSG00000075552	1,87E+00	-3,107639691	1,38E+00	2,40E-01	5,50E-01	Cyp3a41b	3,67	No
ENSMUSG00000075553	-6,80E-01	-2,165716449	7,08E-01	4,00E-01	6,99E-01	Gm5464	0,62	No
ENSMUSG00000075555	3,13E-01	0,539033456	1,74E+00	1,87E-01	5,50E-01	Gm10863	1,24	No
ENSMUSG00000075558	-3,53E+00	-2,831972902	5,58E+00	1,81E-02	1,42E-01	Gm17151	0,09	No
ENSMUSG00000075569	5,56E-02	-1,493441327	1,14E-02	9,15E-01	1,00E+00	Rsph10b	1,04	No
ENSMUSG00000075571	-7,38E-02	-3,033659033	2,66E-03	9,59E-01	1,00E+00	Defb30	0,95	No
ENSMUSG00000075573	-1,89E+00	-3,1076441	1,42E+00	2,33E-01	5,50E-01	Defb47	0,27	No
ENSMUSG00000075574	-1,90E+00	-3,033594154	1,44E+00	2,30E-01	5,50E-01	Defb48	0,27	No
ENSMUSG00000075576	6,76E-02	2,440637228	2,64E-01	6,07E-01	8,50E-01	Gm12359	1,05	No
ENSMUSG00000075581	-1,95E-01	5,651670049	8,23E+00	4,12E-03	4,84E-02	Gm16409	0,87	Yes
ENSMUSG00000075585	-1,12E-01	2,626912856	8,20E-01	3,65E-01	6,66E-01	6330403L08Rik	0,93	No
ENSMUSG00000075587	-5,82E-01	-2,770624831	2,66E-01	6,06E-01	8,49E-01	Gm11531	0,67	No
ENSMUSG00000075588	-1,90E+00	-3,107628502	1,44E+00	2,29E-01	5,50E-01	Hoxb2	0,27	No
ENSMUSG00000075589	-8,83E-02	-2,770632657	5,28E-03	9,42E-01	1,00E+00	Gm11536	0,94	No
ENSMUSG00000075590	6,52E-01	-0,522916013	3,02E+00	8,24E-02	3,69E-01	Nrbp2	1,57	No
ENSMUSG00000075591	-1,42E-01	5,359613583	4,09E+00	4,32E-02	2,47E-01	Gm10874	0,91	No
ENSMUSG00000075592	1,58E-01	0,399257798	3,70E-01	5,43E-01	8,12E-01	Nynrin	1,12	No
ENSMUSG00000075593	3,36E-01	-0,673595902	8,40E-01	3,59E-01	6,61E-01	Gal3st4	1,26	No
ENSMUSG00000075595	2,10E-01	6,822096428	1,17E+01	6,10E-04	1,08E-02	Zfp652	1,16	Yes
ENSMUSG00000075596	-1,05E+00	-0,573969932	6,42E+00	1,13E-02	1,02E-01	B130006D01Rik	0,48	No
ENSMUSG00000075600	2,20E-01	3,699109059	5,36E+00	2,06E-02	1,54E-01	Zc3h3	1,16	No
ENSMUSG00000075602	-6,13E-01	2,980749693	2,83E+01	1,02E-07	5,67E-06	Ly6a	0,65	Yes
ENSMUSG00000075609	1,68E-01	5,577393302	5,94E+00	1,48E-02	1,25E-01	Gm7935	1,12	No
ENSMUSG00000075611	6,38E-01	2,335861615	2,09E+01	4,90E-06	1,77E-04	Gm11545	1,56	Yes

ENSMUSG00000075700	1,97E-01	7,882249397	1,13E+01	7,57E-04	1,29E-02	Selenot	1,15	Yes
ENSMUSG00000075701	2,50E-02	5,789600087	1,40E-01	7,09E-01	9,09E-01	Selenos	1,02	No
ENSMUSG00000075702	-3,10E-01	1,416328915	2,82E+00	9,30E-02	3,83E-01	Selenom	0,81	No
ENSMUSG00000075703	2,31E-01	5,259595131	1,06E+01	1,16E-03	1,83E-02	Selenoi	1,17	Yes
ENSMUSG00000075704	2,07E-01	3,544104705	4,35E+00	3,71E-02	2,29E-01	Txnrd2	1,15	No
ENSMUSG00000075705	2,24E-02	7,96826769	1,48E-01	7,01E-01	9,05E-01	Msrb1	1,02	No
ENSMUSG00000075706	-5,70E-02	5,059121962	6,06E-01	4,36E-01	7,29E-01	Gpx4	0,96	No
ENSMUSG00000075770	1,80E-01	-0,472964648	2,38E-01	6,26E-01	8,60E-01	Gm22589	1,13	No
ENSMUSG00000075826	3,14E-02	-3,107617333	-7,69E-10	1,00E+00	1,00E+00	Gm23691	1,02	No
ENSMUSG00000075829	-1,89E+00	-3,1076441	1,42E+00	2,33E-01	5,50E-01	Gm23692	0,27	No
ENSMUSG00000075837	5,02E-01	-1,997988307	4,87E-01	4,85E-01	7,72E-01	Gm22005	1,42	No
ENSMUSG00000075903	8,58E-01	-2,548282979	8,86E-01	3,47E-01	6,47E-01	Gm23941	1,81	No
ENSMUSG00000075908	-1,86E+00	-3,033612668	1,39E+00	2,38E-01	5,50E-01	Gm23940	0,28	No
ENSMUSG00000076009	-2,20E-02	-3,107641896	1,16E-04	9,91E-01	1,00E+00	Mir688	0,98	No
ENSMUSG00000076012	5,69E-02	1,576556573	1,11E-01	7,39E-01	9,26E-01	Mir705	1,04	No
ENSMUSG00000076031	2,36E-02	2,811768367	3,92E-02	8,43E-01	9,85E-01	Gm22751	1,02	No
ENSMUSG00000076033	8,65E-02	7,680376894	2,15E+00	1,42E-01	4,77E-01	Mir692-3	1,06	No
ENSMUSG00000076036	1,01E-01	9,54973279	3,17E+00	7,52E-02	3,49E-01	Gm22133	1,07	No
ENSMUSG00000076038	5,67E-02	6,867616703	8,55E-01	3,55E-01	6,56E-01	Gm22774	1,04	No
ENSMUSG00000076051	6,58E-01	-2,289491197	5,55E-01	4,56E-01	7,46E-01	Mir707	1,58	No
ENSMUSG00000076060	2,14E-01	2,45473794	2,58E+00	1,08E-01	4,03E-01	Mir704	1,16	No
ENSMUSG00000076062	-1,34E-01	-2,094763402	3,00E-02	8,63E-01	9,97E-01	Mir92-1	0,91	No
ENSMUSG00000076068	-2,31E-02	0,464549054	8,87E-03	9,25E-01	1,00E+00	Mir678	0,98	No
ENSMUSG00000076119	-2,54E+00	-2,68312013	3,92E+00	4,76E-02	2,61E-01	Mir698	0,17	No
ENSMUSG00000076121	-7,57E-01	-2,448652175	6,42E-01	4,23E-01	7,18E-01	Mir687	0,59	No
ENSMUSG00000076123	7,44E-01	-0,19589721	4,99E+00	2,55E-02	1,77E-01	Mir700	1,68	No
ENSMUSG00000076128	2,93E-02	3,645425135	9,14E-02	7,62E-01	9,38E-01	Mir686	1,02	No
ENSMUSG00000076129	-3,27E-01	-2,088767788	2,29E-01	6,32E-01	8,64E-01	Gm22920	0,80	No
ENSMUSG00000076135	-1,91E-01	-2,226309059	5,25E-02	8,19E-01	9,71E-01	Gm24276	0,88	No
ENSMUSG00000076136	-9,41E-01	-1,522494595	2,48E+00	1,15E-01	4,20E-01	Gm24951	0,52	No
ENSMUSG00000076137	1,25E-01	2,668859016	1,03E+00	3,10E-01	6,09E-01	Gm26384	1,09	No
ENSMUSG00000076138	-1,58E-01	7,68105669	7,23E+00	7,19E-03	7,33E-02	Gm26377	0,90	No

ENSMUSG00000076141	-4,80E-02	-2,896135597	1,06E-03	9,74E-01	1,00E+00	Mir693	0,97	No
ENSMUSG00000076142	1,17E+00	-3,14612376	6,40E-01	4,24E-01	7,18E-01	Mir711	2,26	No
ENSMUSG00000076144	-6,33E-01	-2,311077812	5,45E-01	4,60E-01	7,49E-01	Mir709	0,64	No
ENSMUSG00000076163	-3,83E-02	-3,107651936	-7,91E-11	1,00E+00	1,00E+00	Mir701	0,97	No
ENSMUSG00000076212	9,01E-03	-3,107646452	-5,16E-10	1,00E+00	1,00E+00	Mir680-1	1,01	No
ENSMUSG00000076216	3,17E+00	-2,963263597	4,11E+00	4,28E-02	2,46E-01	Mir695	8,99	No
ENSMUSG00000076218	3,69E-01	-2,033663439	3,28E-01	5,67E-01	8,29E-01	Mir692-1	1,29	No
ENSMUSG00000076228	2,83E-01	-1,59641435	2,56E-01	6,13E-01	8,53E-01	Gm25595	1,22	No
ENSMUSG00000076230	-1,80E-01	0,745333394	6,50E-01	4,20E-01	7,16E-01	Gm22758	0,88	No
ENSMUSG00000076231	-3,27E-01	-1,226142935	5,03E-01	4,78E-01	7,66E-01	Gm22759	0,80	No
ENSMUSG00000076232	1,22E+00	-3,146114919	6,90E-01	4,06E-01	7,02E-01	Gm22760	2,33	No
ENSMUSG00000076236	-1,43E-01	7,216682476	5,66E+00	1,74E-02	1,38E-01	Mir682	0,91	No
ENSMUSG00000076237	-6,61E-01	-3,070161976	1,72E-01	6,78E-01	8,92E-01	Gm26300	0,63	No
ENSMUSG00000076240	3,03E-01	1,484949882	3,01E+00	8,28E-02	3,70E-01	Mir702	1,23	No
ENSMUSG00000076246	1,85E+00	-3,107657832	1,35E+00	2,46E-01	5,50E-01	Gm22256	3,61	No
ENSMUSG00000076252	-2,63E-02	-3,033653157	2,33E-04	9,88E-01	1,00E+00	Mir302b	0,98	No
ENSMUSG00000076256	-1,51E+00	-2,627611361	1,50E+00	2,20E-01	5,50E-01	Mir19b-1	0,35	No
ENSMUSG00000076258	-5,67E-01	8,437934058	9,45E+01	2,41E-22	7,07E-20	Gm23935	0,67	Yes
ENSMUSG00000076270	9,69E-02	1,39681551	2,74E-01	6,00E-01	8,47E-01	Mir719	1,07	No
ENSMUSG00000076271	-9,81E-01	-2,145996491	1,49E+00	2,23E-01	5,50E-01	Mir697	0,51	No
ENSMUSG00000076281	-6,94E-01	6,591668279	1,20E+02	7,70E-28	3,60E-25	Gm24270	0,62	Yes
ENSMUSG00000076284	1,85E+00	-3,107657832	1,35E+00	2,46E-01	5,50E-01	Gm24272	3,61	No
ENSMUSG00000076288	7,06E-01	-2,896181546	2,71E-01	6,03E-01	8,48E-01	Mir301b	1,63	No
ENSMUSG00000076300	1,81E+00	-3,10765737	1,28E+00	2,58E-01	5,50E-01	Gm23060	3,49	No
ENSMUSG00000076303	1,84E+00	-3,033632273	1,33E+00	2,49E-01	5,50E-01	Gm23061	3,58	No
ENSMUSG00000076304	-2,66E+00	-3,033639428	2,81E+00	9,35E-02	3,83E-01	Gm23062	0,16	No
ENSMUSG00000076309	6,34E-02	6,10240796	9,48E-01	3,30E-01	6,31E-01	Mir147	1,04	No
ENSMUSG00000076315	4,89E-01	-0,946233064	1,31E+00	2,52E-01	5,50E-01	Mir343	1,40	No
ENSMUSG00000076333	-2,71E+00	-2,863668034	2,90E+00	8,84E-02	3,79E-01	Mir599	0,15	No
ENSMUSG00000076337	-4,17E-02	-2,963202678	7,92E-04	9,78E-01	1,00E+00	Gm24714	0,97	No
ENSMUSG00000076338	-1,89E+00	-3,1076441	1,42E+00	2,33E-01	5,50E-01	Mir181d	0,27	No
ENSMUSG00000076364	-1,87E+00	-3,107637761	1,39E+00	2,38E-01	5,50E-01	Gm24049	0,27	No

ENSMUSG00000076370	1,87E+00	-3,03364371	1,36E+00	2,43E-01	5,50E-01	Gm22369	3,66	No
ENSMUSG00000076372	4,17E-01	-2,333169735	2,07E-01	6,49E-01	8,75E-01	Mir568	1,34	No
ENSMUSG00000076376	-6,33E-01	-1,57409235	1,24E+00	2,65E-01	5,60E-01	Mir674	0,64	No
ENSMUSG00000076379	-1,87E+00	-3,107637761	1,39E+00	2,38E-01	5,50E-01	Mir190a	0,27	No
ENSMUSG00000076382	1,82E+00	-3,033667329	1,31E+00	2,52E-01	5,50E-01	Gm23193	3,54	No
ENSMUSG00000076387	-5,23E-02	1,195305346	7,20E-02	7,88E-01	9,54E-01	Mir671	0,96	No
ENSMUSG00000076392	1,62E+00	-2,711751167	1,79E+00	1,80E-01	5,42E-01	Gm25992	3,07	No
ENSMUSG00000076424	-3,20E+00	-2,896154564	4,32E+00	3,77E-02	2,31E-01	Gm25986	0,11	No
ENSMUSG00000076430	6,17E-01	-1,863743489	9,94E-01	3,19E-01	6,19E-01	Hus1b	1,53	No
ENSMUSG00000076431	-4,99E-01	3,422190837	2,55E+01	4,35E-07	2,11E-05	Sox4	0,71	Yes
ENSMUSG00000076432	-8,86E-02	6,240307294	1,92E+00	1,66E-01	5,18E-01	Ywhaq	0,94	No
ENSMUSG00000076433	5,54E-01	-1,05190487	1,68E+00	1,95E-01	5,50E-01	Cep295nl	1,47	No
ENSMUSG00000076434	7,54E-01	-2,896151836	3,10E-01	5,77E-01	8,35E-01	Wfdc3	1,69	No
ENSMUSG00000076435	9,18E-02	2,395020888	4,65E-01	4,95E-01	7,81E-01	Acsf2	1,07	No
ENSMUSG00000076436	4,73E-01	-2,548255829	3,04E-01	5,81E-01	8,38E-01	Oxct2a	1,39	No
ENSMUSG00000076437	8,10E-02	4,870047953	1,12E+00	2,90E-01	5,85E-01	Selenoh	1,06	No
ENSMUSG00000076438	-1,45E-02	-3,107649194	3,72E-11	1,00E+00	1,00E+00	Oxct2b	0,99	No
ENSMUSG00000076441	1,46E-01	5,123756448	4,07E+00	4,36E-02	2,49E-01	Ass1	1,11	No
ENSMUSG00000076444	5,88E-01	-1,311239065	1,36E+00	2,44E-01	5,50E-01	Mir761	1,50	No
ENSMUSG00000076445	-7,93E-02	3,32605489	5,83E-01	4,45E-01	7,36E-01	Gm24865	0,95	No
ENSMUSG00000076454	-1,85E-01	1,030764833	8,29E-01	3,63E-01	6,64E-01	Mir762	0,88	No
ENSMUSG00000076456	-2,22E+00	-3,010026869	1,93E+00	1,65E-01	5,17E-01	Mir760	0,21	No
ENSMUSG00000076460	-1,98E-02	-3,033655952	2,83E-11	1,00E+00	1,00E+00	Mir744	0,99	No
ENSMUSG00000076461	3,52E+00	-2,896164906	5,38E+00	2,03E-02	1,53E-01	Trbv1	11,47	No
ENSMUSG00000076463	9,01E-03	-3,107646452	-5,16E-10	1,00E+00	1,00E+00	Trbv3	1,01	No
ENSMUSG00000076465	-1,22E+00	-3,146113954	6,96E-01	4,04E-01	7,01E-01	Trbv5	0,43	No
ENSMUSG00000076474	3,14E-02	-3,107617333	-7,69E-10	1,00E+00	1,00E+00	Trbv17	1,02	No
ENSMUSG00000076476	-1,89E+00	-3,1076441	1,42E+00	2,33E-01	5,50E-01	Trbv20	0,27	No
ENSMUSG00000076479	-1,89E+00	-3,1076441	1,42E+00	2,33E-01	5,50E-01	Trbv26	0,27	No
ENSMUSG00000076490	-1,87E+00	-3,107637761	1,39E+00	2,38E-01	5,50E-01	Trbc1	0,27	No
ENSMUSG00000076498	-1,94E+00	-3,107619243	1,50E+00	2,21E-01	5,50E-01	Trbc2	0,26	No
ENSMUSG00000076501	-1,20E+00	-3,070140586	6,96E-01	4,04E-01	7,01E-01	Igkv2-137	0,43	No

ENSMUSG00000076508	-1,89E+00	-3,1076441	1,42E+00	2,33E-01	5,50E-01	Igkv17-127	0,27	No
ENSMUSG00000076514	-5,88E-02	-3,033616507	1,62E-03	9,68E-01	1,00E+00	Igkv17-121	0,96	No
ENSMUSG00000076523	-1,87E+00	-3,107637761	1,39E+00	2,38E-01	5,50E-01	Igkv15-103	0,27	No
ENSMUSG00000076534	-3,83E-02	-3,107651936	-7,91E-11	1,00E+00	1,00E+00	Igkv12-89	0,97	No
ENSMUSG00000076536	-1,14E-02	-3,033635021	1,50E-05	9,97E-01	1,00E+00	Igkv4-86	0,99	No
ENSMUSG00000076540	-1,87E+00	-3,107637761	1,39E+00	2,38E-01	5,50E-01	Igkv4-80	0,27	No
ENSMUSG00000076545	-3,54E+00	-2,896134066	5,62E+00	1,77E-02	1,39E-01	Igkv4-72	0,09	No
ENSMUSG00000076549	1,85E+00	-3,107657832	1,35E+00	2,46E-01	5,50E-01	Igkv4-68	3,61	No
ENSMUSG00000076555	-5,88E-02	-3,033616507	1,62E-03	9,68E-01	1,00E+00	Igkv4-57-1	0,96	No
ENSMUSG00000076562	-1,94E+00	-3,107619243	1,50E+00	2,21E-01	5,50E-01	Igkv4-50	0,26	No
ENSMUSG00000076569	-3,26E+00	-2,963184148	4,48E+00	3,43E-02	2,18E-01	Igkv5-39	0,10	No
ENSMUSG00000076576	3,14E-02	-3,107617333	-7,69E-10	1,00E+00	1,00E+00	Igkv6-32	1,02	No
ENSMUSG00000076577	-2,65E+00	-2,831983935	2,81E+00	9,39E-02	3,84E-01	Igkv8-30	0,16	No
ENSMUSG00000076583	-1,86E+00	-3,033612668	1,39E+00	2,38E-01	5,50E-01	Igkv8-24	0,28	No
ENSMUSG00000076586	-1,94E+00	-3,107619243	1,50E+00	2,21E-01	5,50E-01	Igkv8-21	0,26	No
ENSMUSG00000076598	-3,83E-02	-3,107651936	-7,91E-11	1,00E+00	1,00E+00	Igkv3-7	0,97	No
ENSMUSG00000076608	2,06E-02	-3,146103739	-9,26E-10	1,00E+00	1,00E+00	Igkj5	1,01	No
ENSMUSG00000076609	-1,09E+00	-0,704219365	7,40E+00	6,53E-03	6,82E-02	Igkc	0,47	No
ENSMUSG00000076612	3,17E+00	-2,896135869	4,11E+00	4,26E-02	2,46E-01	Ighg2c	9,02	No
ENSMUSG00000076613	4,10E+00	-2,711675753	8,25E+00	4,09E-03	4,81E-02	Ighg2b	17,18	Yes
ENSMUSG00000076614	-4,84E+00	-2,311162665	1,45E+01	1,43E-04	3,25E-03	Ighg1	0,03	Yes
ENSMUSG00000076615	-1,24E+00	-2,548190874	1,48E+00	2,24E-01	5,50E-01	Ighg3	0,42	No
ENSMUSG00000076617	-3,45E-02	4,077407162	1,55E-01	6,93E-01	9,01E-01	Ighm	0,98	No
ENSMUSG00000076618	-1,76E+00	-2,831982231	2,17E+00	1,41E-01	4,74E-01	Ighj4	0,29	No
ENSMUSG00000076619	3,14E-02	-3,107617333	-7,69E-10	1,00E+00	1,00E+00	Ighj3	1,02	No
ENSMUSG00000076620	-1,14E-02	-3,033635021	1,50E-05	9,97E-01	1,00E+00	Ighj2	0,99	No
ENSMUSG00000076621	-5,88E-02	-3,033616507	1,62E-03	9,68E-01	1,00E+00	Ighj1	0,96	No
ENSMUSG00000076652	-1,90E+00	-3,033647263	1,44E+00	2,30E-01	5,50E-01	Ighv7-3	0,27	No
ENSMUSG00000076655	9,01E-03	-3,107646452	-5,16E-10	1,00E+00	1,00E+00	Ighv4-1	1,01	No
ENSMUSG00000076665	-5,88E-02	-3,033652696	1,62E-03	9,68E-01	1,00E+00	Ighv7-1	0,96	No
ENSMUSG00000076672	1,88E+00	-2,963229875	1,37E+00	2,42E-01	5,50E-01	Ighv3-6	3,68	No
ENSMUSG00000076674	-1,87E+00	-3,107637761	1,39E+00	2,38E-01	5,50E-01	Ighv3-8	0,27	No

ENSMUSG00000076677	-4,79E-01	-2,997999812	1,14E-01	7,35E-01	9,24E-01	Ighv6-3	0,72	No
ENSMUSG00000076695	-1,87E+00	-3,033619006	1,41E+00	2,36E-01	5,50E-01	Ighv1-18	0,27	No
ENSMUSG00000076731	-3,16E+00	-2,963246013	4,20E+00	4,04E-02	2,38E-01	Ighv8-12	0,11	No
ENSMUSG00000076749	4,05E+00	-2,711782297	8,02E+00	4,63E-03	5,27E-02	Tcrg-C1	16,58	No
ENSMUSG00000076752	-2,02E+00	-2,71173098	3,08E+00	7,92E-02	3,60E-01	Tcrg-C2	0,25	No
ENSMUSG00000076824	4,24E-02	-3,033592245	-1,17E-09	1,00E+00	1,00E+00	Trav14n-3	1,03	No
ENSMUSG00000076892	9,01E-03	-3,107646452	-5,16E-10	1,00E+00	1,00E+00	Traj38	1,01	No
ENSMUSG00000076915	-3,83E-02	-3,107651936	-7,91E-11	1,00E+00	1,00E+00	Traj14	0,97	No
ENSMUSG00000076928	-1,98E-02	-3,033655952	2,83E-11	1,00E+00	1,00E+00	Trac	0,99	No
ENSMUSG00000076934	1,20E+00	-2,832060761	8,90E-01	3,46E-01	6,46E-01	Iglv1	2,30	No
ENSMUSG00000076937	3,13E-01	-2,448715837	9,99E-02	7,52E-01	9,32E-01	Iglc2	1,24	No
ENSMUSG00000076940	-2,71E+00	-2,963235336	2,92E+00	8,75E-02	3,78E-01	Iglv2	0,15	No
ENSMUSG00000076941	3,51E+00	-2,83205728	5,36E+00	2,06E-02	1,54E-01	Gm22065	11,42	No
ENSMUSG00000076944	-3,29E-01	-2,070138085	2,19E-01	6,39E-01	8,69E-01	Gm22064	0,80	No
ENSMUSG00000076949	-3,83E-02	-3,107651936	-7,91E-11	1,00E+00	1,00E+00	Gm22066	0,97	No
ENSMUSG00000076961	-1,74E+00	-2,71169766	2,10E+00	1,47E-01	4,86E-01	Gm23251	0,30	No
ENSMUSG00000076972	-1,87E+00	-3,107637761	1,39E+00	2,38E-01	5,50E-01	Mir193b	0,27	No
ENSMUSG00000076987	1,85E+00	-3,107657832	1,35E+00	2,46E-01	5,50E-01	Gm24115	3,61	No
ENSMUSG00000076999	-3,29E-01	-2,711686354	7,95E-02	7,78E-01	9,47E-01	Gm22426	0,80	No
ENSMUSG00000077004	-2,68E+00	-2,963261035	2,84E+00	9,17E-02	3,82E-01	Gm23876	0,16	No
ENSMUSG00000077019	3,14E-02	-3,107617333	-7,69E-10	1,00E+00	1,00E+00	Gm22191	1,02	No
ENSMUSG00000077090	3,14E-02	-3,107617333	-7,69E-10	1,00E+00	1,00E+00	Gm22117	1,02	No
ENSMUSG00000077148	-8,79E-01	-2,832023183	4,23E-01	5,15E-01	7,97E-01	Gm22935	0,54	No
ENSMUSG00000077150	-3,83E-02	-3,107651936	-7,91E-11	1,00E+00	1,00E+00	Gm24581	0,97	No
ENSMUSG00000077155	3,14E-02	-3,107617333	-7,69E-10	1,00E+00	1,00E+00	Gm24582	1,02	No
ENSMUSG00000077158	1,87E+00	-3,107639691	1,38E+00	2,40E-01	5,50E-01	Gm24578	3,67	No
ENSMUSG00000077167	-2,45E-01	0,527113049	1,06E+00	3,04E-01	6,00E-01	Gm24119	0,84	No
ENSMUSG00000077185	6,59E-01	-2,289445639	5,58E-01	4,55E-01	7,45E-01	Gm25091	1,58	No
ENSMUSG00000077192	-1,50E+00	-1,378058792	8,44E+00	3,67E-03	4,44E-02	Snora17	0,35	Yes
ENSMUSG00000077200	-1,94E+00	-3,107619243	1,50E+00	2,21E-01	5,50E-01	Gm25613	0,26	No
ENSMUSG00000077202	3,87E-02	-2,289456653	2,71E-03	9,59E-01	1,00E+00	Gm25612	1,03	No
ENSMUSG00000077212	7,94E-03	1,195312619	1,60E-03	9,68E-01	1,00E+00	Snord69	1,01	No

ENSMUSG00000077217	-1,03E+00	-1,770569825	3,08E+00	7,94E-02	3,60E-01	Gm23951	0,49	No
ENSMUSG00000077220	1,48E-02	1,488973732	6,90E-03	9,34E-01	1,00E+00	Snord78	1,01	No
ENSMUSG00000077221	-3,91E-01	-2,896165094	9,42E-02	7,59E-01	9,36E-01	Snord67	0,76	No
ENSMUSG00000077222	-3,00E-01	-0,042674893	9,65E-01	3,26E-01	6,27E-01	Gm22270	0,81	No
ENSMUSG00000077223	-2,68E+00	-2,896121483	2,85E+00	9,14E-02	3,82E-01	Gm22271	0,16	No
ENSMUSG00000077227	-3,09E-01	-2,497588693	8,09E-02	7,76E-01	9,46E-01	Gm22272	0,81	No
ENSMUSG00000077239	8,39E-01	-1,300292139	2,31E+00	1,28E-01	4,49E-01	Snord66	1,79	No
ENSMUSG00000077254	1,21E+00	-2,99802822	6,71E-01	4,13E-01	7,08E-01	Gm26079	2,31	No
ENSMUSG00000077258	1,62E+00	-2,832048598	1,81E+00	1,78E-01	5,39E-01	Gm26082	3,08	No
ENSMUSG00000077265	-2,36E-01	-0,912582699	3,56E-01	5,51E-01	8,17E-01	Gm24430	0,85	No
ENSMUSG00000077271	-4,39E-01	-1,863590698	3,55E-01	5,51E-01	8,18E-01	Gm22781	0,74	No
ENSMUSG00000077274	7,39E-01	-2,77055918	2,97E-01	5,86E-01	8,39E-01	Gm22786	1,67	No
ENSMUSG00000077285	-3,24E-01	-1,033600362	5,73E-01	4,49E-01	7,39E-01	Gm26394	0,80	No
ENSMUSG00000077293	-1,87E+00	-3,03364178	1,41E+00	2,36E-01	5,50E-01	Gm24125	0,27	No
ENSMUSG00000077302	6,65E-01	-2,896156246	2,39E-01	6,25E-01	8,59E-01	Gm22473	1,59	No
ENSMUSG00000077305	-1,87E+00	-3,107637761	1,39E+00	2,38E-01	5,50E-01	Gm22470	0,27	No
ENSMUSG00000077323	-4,05E-02	-2,963228491	6,10E-04	9,80E-01	1,00E+00	Rnu11	0,97	No
ENSMUSG00000077339	4,16E-01	-2,600719678	1,35E-01	7,13E-01	9,11E-01	Gm23010	1,33	No
ENSMUSG00000077345	8,16E-02	-1,863711737	1,63E-02	8,98E-01	1,00E+00	Snord70	1,06	No
ENSMUSG00000077347	-3,83E-02	-3,107651936	-7,91E-11	1,00E+00	1,00E+00	Gm24671	0,97	No
ENSMUSG00000077349	2,66E+00	-2,96324125	2,74E+00	9,79E-02	3,91E-01	Gm24867	6,34	No
ENSMUSG00000077350	-3,83E-02	-3,107651936	-7,91E-11	1,00E+00	1,00E+00	n-R5s31	0,97	No
ENSMUSG00000077358	4,00E-01	-2,268121243	1,90E-01	6,63E-01	8,83E-01	Gm26293	1,32	No
ENSMUSG00000077360	-3,83E-02	-3,107651936	-7,91E-11	1,00E+00	1,00E+00	Gm23482	0,97	No
ENSMUSG00000077367	6,93E-01	-2,22631947	6,97E-01	4,04E-01	7,01E-01	Gm23483	1,62	No
ENSMUSG00000077380	-1,33E-01	-1,033677105	1,01E-01	7,50E-01	9,31E-01	Gm22661	0,91	No
ENSMUSG00000077381	-1,87E+00	-3,107637761	1,39E+00	2,38E-01	5,50E-01	Gm22660	0,27	No
ENSMUSG00000077382	1,22E+00	-1,522636966	4,74E+00	2,95E-02	1,96E-01	n-R5s200	2,33	No
ENSMUSG00000077385	-4,13E-02	-3,033659495	7,08E-04	9,79E-01	1,00E+00	Gm22657	0,97	No
ENSMUSG00000077387	1,87E+00	-3,107639691	1,38E+00	2,40E-01	5,50E-01	Gm22656	3,67	No
ENSMUSG00000077391	8,71E-01	-2,247080733	1,04E+00	3,08E-01	6,06E-01	Gm24336	1,83	No
ENSMUSG00000077394	-2,42E-01	-2,522692928	7,06E-02	7,90E-01	9,55E-01	Gm24339	0,85	No

ENSMUSG00000077416	-6,39E-01	-2,146102866	7,94E-01	3,73E-01	6,74E-01	Gm23747	0,64	No
ENSMUSG00000077419	1,87E+00	-3,033632735	1,36E+00	2,43E-01	5,50E-01	Gm23744	3,66	No
ENSMUSG00000077420	2,63E+00	-2,770656706	2,67E+00	1,02E-01	3,94E-01	Gm24282	6,19	No
ENSMUSG00000077421	-1,89E+00	-3,1076441	1,42E+00	2,33E-01	5,50E-01	Gm24283	0,27	No
ENSMUSG00000077426	1,74E-01	-0,785739175	1,88E-01	6,64E-01	8,84E-01	Gm26387	1,13	No
ENSMUSG00000077431	2,45E+00	-2,71172088	3,55E+00	5,95E-02	3,02E-01	Gm22591	5,45	No
ENSMUSG00000077440	8,26E-02	0,915862894	1,50E-01	6,98E-01	9,04E-01	Gm23130	1,06	No
ENSMUSG00000077448	-6,93E-01	-2,070048892	8,18E-01	3,66E-01	6,67E-01	Gm23126	0,62	No
ENSMUSG00000077449	-1,16E+00	-3,033636805	5,90E-01	4,42E-01	7,34E-01	Gm23127	0,45	No
ENSMUSG00000077450	1,15E-01	6,592229778	3,40E+00	6,53E-02	3,20E-01	Rab11b	1,08	No
ENSMUSG00000077454	-6,48E-02	-2,963249916	1,90E-03	9,65E-01	1,00E+00	Gm25918	0,96	No
ENSMUSG00000077457	-4,37E-02	-0,600427182	1,30E-02	9,09E-01	1,00E+00	Snord65	0,97	No
ENSMUSG00000077460	-1,23E+00	-1,800931002	4,11E+00	4,27E-02	2,46E-01	Gm26419	0,43	No
ENSMUSG00000077465	-5,78E-02	-2,896167382	2,23E-03	9,62E-01	1,00E+00	Gm26423	0,96	No
ENSMUSG00000077476	3,14E-02	-3,107617333	-7,69E-10	1,00E+00	1,00E+00	Gm24794	1,02	No
ENSMUSG00000077484	1,85E+00	-3,107657832	1,35E+00	2,46E-01	5,50E-01	Gm25274	3,61	No
ENSMUSG00000077488	-1,94E+00	-3,107619243	1,50E+00	2,21E-01	5,50E-01	Gm25276	0,26	No
ENSMUSG00000077493	-2,56E-01	-0,350043051	5,81E-01	4,46E-01	7,37E-01	Snord91a	0,84	No
ENSMUSG00000077505	-1,73E+00	-2,655070694	2,08E+00	1,49E-01	4,90E-01	Gm24233	0,30	No
ENSMUSG00000077506	-2,17E+00	-2,831952198	2,62E+00	1,06E-01	3,98E-01	Scarna9	0,22	No
ENSMUSG00000077515	-5,12E-01	-2,070152775	4,21E-01	5,17E-01	7,97E-01	Gm25878	0,70	No
ENSMUSG00000077524	-9,51E-01	-2,448643241	7,92E-01	3,74E-01	6,74E-01	n-R5s67	0,52	No
ENSMUSG00000077526	8,20E-01	-2,54823876	5,85E-01	4,44E-01	7,36E-01	Gm25379	1,76	No
ENSMUSG00000077527	-4,64E-02	-2,963261869	9,78E-04	9,75E-01	1,00E+00	Gm25506	0,97	No
ENSMUSG00000077549	-2,44E-01	0,24420321	8,53E-01	3,56E-01	6,57E-01	Snord71	0,84	No
ENSMUSG00000077554	-2,19E+00	-2,770579875	2,66E+00	1,03E-01	3,95E-01	Gm23707	0,22	No
ENSMUSG00000077557	-7,83E-01	-1,929237216	1,45E+00	2,28E-01	5,50E-01	Gm23706	0,58	No
ENSMUSG00000077561	-4,13E-02	-3,033659495	7,08E-04	9,79E-01	1,00E+00	Gm24607	0,97	No
ENSMUSG00000077563	1,18E-01	-0,131740564	1,43E-01	7,05E-01	9,07E-01	Snora68	1,09	No
ENSMUSG00000077564	4,75E-01	-2,355543375	3,06E-01	5,80E-01	8,37E-01	Gm23202	1,39	No
ENSMUSG00000077565	-2,64E-01	-2,192540172	1,11E-01	7,38E-01	9,26E-01	Gm23201	0,83	No
ENSMUSG00000077569	6,34E-01	-1,522659776	1,30E+00	2,55E-01	5,50E-01	Gm24608	1,55	No

ENSMUSG00000077571	-6,92E-01	-2,963232606	1,93E-01	6,61E-01	8,82E-01	Snora36b	0,62	No
ENSMUSG00000077575	4,63E-01	-0,238325002	2,09E+00	1,48E-01	4,88E-01	Gm24888	1,38	No
ENSMUSG00000077577	9,01E-03	-3,107646452	-5,16E-10	1,00E+00	1,00E+00	Gm24890	1,01	No
ENSMUSG00000077578	-1,74E+00	-2,655082014	2,11E+00	1,47E-01	4,85E-01	Gm25631	0,30	No
ENSMUSG00000077591	1,82E+00	-3,033661385	1,31E+00	2,52E-01	5,50E-01	Gm26016	3,54	No
ENSMUSG00000077592	-1,89E+00	-3,1076441	1,42E+00	2,33E-01	5,50E-01	Gm26013	0,27	No
ENSMUSG00000077595	-7,38E-02	-3,033659033	2,66E-03	9,59E-01	1,00E+00	Gm26019	0,95	No
ENSMUSG00000077604	-1,18E+00	-2,070016948	2,03E+00	1,54E-01	4,98E-01	Snord1b	0,44	No
ENSMUSG00000077611	-5,35E-01	-2,472924593	3,41E-01	5,59E-01	8,22E-01	Gm23946	0,69	No
ENSMUSG00000077614	4,37E-01	-1,530796496	5,86E-01	4,44E-01	7,35E-01	Gm25500	1,35	No
ENSMUSG00000077621	2,43E-01	-1,832023136	1,36E-01	7,12E-01	9,11E-01	Gm23772	1,18	No
ENSMUSG00000077625	-7,95E-01	-1,726421146	1,71E+00	1,91E-01	5,50E-01	Snord4a	0,58	No
ENSMUSG00000077637	-5,35E-01	-0,614001933	2,04E+00	1,53E-01	4,96E-01	Gm22771	0,69	No
ENSMUSG00000077639	-5,90E-02	-1,226347777	1,47E-02	9,04E-01	1,00E+00	Gm22772	0,96	No
ENSMUSG00000077667	-2,73E-02	-2,963256629	3,40E-04	9,85E-01	1,00E+00	Gm26147	0,98	No
ENSMUSG00000077668	-1,12E-01	-2,770567961	1,20E-02	9,13E-01	1,00E+00	Gm26143	0,93	No
ENSMUSG00000077685	-1,84E-02	-3,033644089	8,41E-05	9,93E-01	1,00E+00	Gm22498	0,99	No
ENSMUSG00000077697	3,14E-02	-3,107617333	-7,69E-10	1,00E+00	1,00E+00	Gm25346	1,02	No
ENSMUSG00000077698	-9,02E-01	-1,212717335	3,13E+00	7,68E-02	3,53E-01	Snord12	0,54	No
ENSMUSG00000077702	-5,98E-01	-0,64800853	2,24E+00	1,34E-01	4,61E-01	Gm24067	0,66	No
ENSMUSG00000077704	-1,34E-01	-0,14623152	1,60E-01	6,89E-01	8,99E-01	Snord89	0,91	No
ENSMUSG00000077705	-1,53E-01	-1,267999574	9,33E-02	7,60E-01	9,37E-01	n-R5s202	0,90	No
ENSMUSG00000077709	-4,07E-01	0,786889236	3,08E+00	7,93E-02	3,60E-01	Snora64	0,75	No
ENSMUSG00000077711	-4,20E-01	-0,627585337	1,24E+00	2,65E-01	5,60E-01	AF357399	0,75	No
ENSMUSG00000077714	9,43E-02	0,288105893	1,25E-01	7,24E-01	9,17E-01	Snord17	1,07	No
ENSMUSG00000077717	-3,83E-02	-3,107651936	-7,91E-11	1,00E+00	1,00E+00	Gm25726	0,97	No
ENSMUSG00000077730	3,14E-02	-3,107617333	-7,69E-10	1,00E+00	1,00E+00	Gm23609	1,02	No
ENSMUSG00000077733	-7,12E-01	-2,033565109	8,64E-01	3,53E-01	6,54E-01	Gm23608	0,61	No
ENSMUSG00000077734	1,88E-01	-0,211000877	3,43E-01	5,58E-01	8,22E-01	Snord83b	1,14	No
ENSMUSG00000077737	-1,80E-01	0,604398567	5,97E-01	4,40E-01	7,32E-01	Snord72	0,88	No
ENSMUSG00000077744	9,01E-03	-3,107646452	-5,16E-10	1,00E+00	1,00E+00	Gm26343	1,01	No
ENSMUSG00000077756	7,37E-01	-2,963270272	2,96E-01	5,86E-01	8,39E-01	Snord90	1,67	No

ENSMUSG00000077758	4,24E-02	-3,033592245	-1,17E-09	1,00E+00	1,00E+00	Gm23527	1,03	No
ENSMUSG00000077759	-1,87E+00	-3,03364178	1,41E+00	2,36E-01	5,50E-01	Gm23528	0,27	No
ENSMUSG00000077761	3,14E-02	-3,107617333	-7,69E-10	1,00E+00	1,00E+00	Gm25214	1,02	No
ENSMUSG00000077780	-3,88E-03	-3,03362684	4,02E-12	1,00E+00	1,00E+00	Gm24161	1,00	No
ENSMUSG00000077785	-1,02E-01	-2,770607829	7,01E-03	9,33E-01	1,00E+00	Gm23910	0,93	No
ENSMUSG00000077787	-7,10E-02	-1,424798701	1,89E-02	8,91E-01	1,00E+00	Gm23908	0,95	No
ENSMUSG00000077789	1,87E+00	-3,107639691	1,38E+00	2,40E-01	5,50E-01	n-R5s26	3,67	No
ENSMUSG00000077796	-1,39E+00	-2,831984581	1,21E+00	2,71E-01	5,68E-01	Gm25562	0,38	No
ENSMUSG00000077797	1,31E-01	0,505540237	2,63E-01	6,08E-01	8,51E-01	Snord19	1,09	No
ENSMUSG00000077799	1,23E+00	-2,831989638	9,43E-01	3,31E-01	6,32E-01	Gm25148	2,35	No
ENSMUSG00000077802	-2,74E+00	-3,033596063	2,99E+00	8,38E-02	3,71E-01	n-R5s52	0,15	No
ENSMUSG00000077804	1,87E+00	-3,107639691	1,38E+00	2,40E-01	5,50E-01	Gm24403	3,67	No
ENSMUSG00000077844	1,85E+00	-3,107657832	1,35E+00	2,46E-01	5,50E-01	Gm22231	3,61	No
ENSMUSG00000077846	7,72E-01	-2,89613394	3,26E-01	5,68E-01	8,30E-01	Gm22232	1,71	No
ENSMUSG00000077884	-4,25E-01	-2,54821128	1,84E-01	6,68E-01	8,86E-01	Gm24557	0,74	No
ENSMUSG00000077890	-1,33E+00	-2,800928982	1,10E+00	2,94E-01	5,89E-01	Mir875	0,40	No
ENSMUSG00000077897	3,02E-01	-2,311201326	9,30E-02	7,60E-01	9,37E-01	Mir511	1,23	No
ENSMUSG00000077931	3,92E-02	1,106025454	3,68E-02	8,48E-01	9,88E-01	Mir877	1,03	No
ENSMUSG00000077962	1,63E+00	-2,711734455	1,83E+00	1,77E-01	5,36E-01	Mir874	3,10	No
ENSMUSG00000077965	-1,90E+00	-3,107628502	1,44E+00	2,29E-01	5,50E-01	Gm24605	0,27	No
ENSMUSG00000077990	4,43E-01	-1,378411122	7,34E-01	3,92E-01	6,92E-01	Gm23134	1,36	No
ENSMUSG00000078087	-9,45E-02	6,272877106	2,20E+00	1,38E-01	4,69E-01	Rps12l1	0,94	No
ENSMUSG00000078091	2,61E+00	-2,711743537	2,65E+00	1,04E-01	3,96E-01	Gm10912	6,12	No
ENSMUSG00000078097	-3,29E-01	-2,070187495	1,62E-01	6,87E-01	8,98E-01	Gm10913	0,80	No
ENSMUSG00000078125	-4,37E-01	1,432771299	5,82E+00	1,59E-02	1,31E-01	Gm10916	0,74	No
ENSMUSG00000078126	-1,16E-01	7,739553551	3,93E+00	4,75E-02	2,61E-01	Rpl23a-ps3	0,92	No
ENSMUSG00000078127	2,00E+00	-1,627772392	8,89E+00	2,86E-03	3,69E-02	Fam170b	4,01	Yes
ENSMUSG00000078128	-2,68E+00	-2,896144247	2,85E+00	9,15E-02	3,82E-01	Gm2178	0,16	No
ENSMUSG00000078129	8,19E-01	-2,311207645	5,84E-01	4,45E-01	7,36E-01	Actl10	1,76	No
ENSMUSG00000078134	3,59E-02	5,523990037	2,71E-01	6,03E-01	8,48E-01	Gm12355	1,03	No
ENSMUSG00000078137	1,72E-01	4,492541791	4,57E+00	3,25E-02	2,10E-01	Ankrd63	1,13	No
ENSMUSG00000078139	3,22E-02	3,782815852	1,16E-01	7,34E-01	9,23E-01	AK157302	1,02	No

ENSMUSG00000078141	-1,94E+00	-3,107619243	1,50E+00	2,21E-01	5,50E-01	Gm2399	0,26	No
ENSMUSG00000078143	1,67E-01	4,298913867	4,01E+00	4,53E-02	2,54E-01	Gm17344	1,12	No
ENSMUSG00000078144	3,01E-01	0,873118476	1,78E+00	1,82E-01	5,44E-01	Capns2	1,23	No
ENSMUSG00000078151	-1,39E-02	4,311574723	2,82E-02	8,67E-01	9,99E-01	Gm12226	0,99	No
ENSMUSG00000078153	-4,37E-01	7,812166236	5,57E+01	8,43E-14	1,18E-11	Psme2b	0,74	Yes
ENSMUSG00000078154	-3,76E-02	5,921830444	3,26E-01	5,68E-01	8,30E-01	Gm12184	0,97	No
ENSMUSG00000078157	-8,51E-01	-1,033506436	3,53E+00	6,04E-02	3,04E-01	4931440F15Rik	0,55	No
ENSMUSG00000078160	2,43E-01	-1,740824723	1,36E-01	7,12E-01	9,11E-01	Gm16503	1,18	No
ENSMUSG00000078162	8,67E-02	6,356547081	1,86E+00	1,72E-01	5,29E-01	Gm2574	1,06	No
ENSMUSG00000078173	1,44E-01	1,16596145	5,52E-01	4,58E-01	7,47E-01	Lenep	1,11	No
ENSMUSG00000078180	-3,39E-01	2,902681262	8,39E+00	3,78E-03	4,53E-02	Gm20900	0,79	Yes
ENSMUSG00000078182	1,81E+00	-3,10765737	1,28E+00	2,58E-01	5,50E-01	Gm6083	3,49	No
ENSMUSG00000078183	-9,47E-02	3,101440586	7,38E-01	3,90E-01	6,90E-01	Gm15610	0,94	No
ENSMUSG00000078184	-1,97E-01	4,828800508	6,68E+00	9,77E-03	9,18E-02	Rbm8a2	0,87	No
ENSMUSG00000078185	2,56E-02	5,147768579	1,25E-01	7,24E-01	9,18E-01	Chml	1,02	No
ENSMUSG00000078190	-1,90E+00	-2,963262532	1,44E+00	2,31E-01	5,50E-01	Dnm3os	0,27	No
ENSMUSG00000078192	-1,25E+00	-2,497666241	1,49E+00	2,21E-01	5,50E-01	Gm7290	0,42	No
ENSMUSG00000078193	-1,69E-01	7,992556373	8,42E+00	3,72E-03	4,48E-02	Gm2000	0,89	Yes
ENSMUSG00000078197	-4,69E-02	-2,832012543	1,46E-03	9,70E-01	1,00E+00	Gm17374	0,97	No
ENSMUSG00000078201	3,44E-03	2,602329504	7,35E-04	9,78E-01	1,00E+00	Tmem203	1,00	No
ENSMUSG00000078202	-6,35E-01	-1,627681078	1,37E+00	2,42E-01	5,50E-01	Nrarp	0,64	No
ENSMUSG00000078234	-1,92E+00	-3,033623267	1,46E+00	2,27E-01	5,50E-01	Klhdc7a	0,26	No
ENSMUSG00000078238	-1,73E-01	7,821142658	8,71E+00	3,16E-03	3,99E-02	Gm12854	0,89	Yes
ENSMUSG00000078240	-5,64E-02	1,528465741	1,05E-01	7,46E-01	9,29E-01	Gm3550	0,96	No
ENSMUSG00000078247	5,12E-01	1,411764692	7,88E+00	4,99E-03	5,59E-02	Airn	1,43	No
ENSMUSG00000078249	-6,96E-02	6,914790256	1,30E+00	2,55E-01	5,50E-01	Hmga1-rs1	0,95	No
ENSMUSG00000078251	-5,47E-03	-2,963221188	1,50E-05	9,97E-01	1,00E+00	Gm16487	1,00	No
ENSMUSG00000078289	-8,31E-01	-2,896155618	3,78E-01	5,38E-01	8,09E-01	Gm10923	0,56	No
ENSMUSG00000078291	-3,51E-01	-0,863777044	7,29E-01	3,93E-01	6,93E-01	Gm3940	0,78	No
ENSMUSG00000078300	4,64E-02	7,298302758	6,01E-01	4,38E-01	7,31E-01	Gm2606	1,03	No
ENSMUSG00000078302	-3,83E-02	-3,107651936	-7,91E-11	1,00E+00	1,00E+00	Foxd1	0,97	No
ENSMUSG00000078307	9,01E-03	-3,107646452	-5,16E-10	1,00E+00	1,00E+00	AI593442	1,01	No

ENSMUSG00000078308	-1,16E+00	-1,333045739	5,27E+00	2,17E-02	1,59E-01	Gm7293		0,45	No
ENSMUSG00000078348	4,38E-02	5,579966762	4,08E-01	5,23E-01	8,01E-01	Sf3b5		1,03	No
ENSMUSG00000078349	1,65E-01	-0,367054285	2,33E-01	6,30E-01	8,62E-01	AW011738		1,12	No
ENSMUSG00000078350	-7,58E-03	3,626201509	6,12E-03	9,38E-01	1,00E+00	Smim1		0,99	No
ENSMUSG00000078365	2,65E+00	-3,033655087	2,73E+00	9,86E-02	3,91E-01	Mos		6,30	No
ENSMUSG00000078370	-8,30E-02	2,693850274	4,70E-01	4,93E-01	7,79E-01	Gm6316		0,94	No
ENSMUSG00000078377	-1,99E-01	7,271655365	1,10E+01	9,02E-04	1,49E-02	Gm4294		0,87	Yes
ENSMUSG00000078380	-6,20E-02	-2,268024747	5,76E-03	9,39E-01	1,00E+00	Gm6096		0,96	No
ENSMUSG00000078427	8,80E-02	3,284380336	6,94E-01	4,05E-01	7,02E-01	Sarnp		1,06	No
ENSMUSG00000078429	-2,95E-02	7,408859464	2,46E-01	6,20E-01	8,57E-01	Ctdsp2		0,98	No
ENSMUSG00000078430	-1,89E+00	-3,1076441	1,42E+00	2,33E-01	5,50E-01	Gm10936		0,27	No
ENSMUSG00000078435	-9,83E-03	1,041267894	2,42E-03	9,61E-01	1,00E+00	AU041133		0,99	No
ENSMUSG00000078439	-4,04E-02	2,779833835	1,12E-01	7,38E-01	9,26E-01	Smim24		0,97	No
ENSMUSG00000078440	-9,32E-03	4,545736061	1,35E-02	9,08E-01	1,00E+00	Dohh		0,99	No
ENSMUSG00000078444	8,16E-01	-2,548251016	5,79E-01	4,47E-01	7,37E-01	Gm10941		1,76	No
ENSMUSG00000078451	1,31E+00	-2,311202244	2,03E+00	1,54E-01	4,97E-01	Ppil6		2,47	No
ENSMUSG00000078452	-3,59E-02	-0,479039217	9,76E-03	9,21E-01	1,00E+00	Raet1d		0,98	No
ENSMUSG00000078453	-3,01E-02	5,464872193	1,89E-01	6,64E-01	8,84E-01	Abracl		0,98	No
ENSMUSG00000078480	-4,28E-02	4,153768119	2,44E-01	6,21E-01	8,57E-01	Mrpl48-ps		0,97	No
ENSMUSG00000078485	2,68E-02	5,572409488	1,53E-01	6,95E-01	9,02E-01	Plekhn1		1,02	No
ENSMUSG00000078486	-1,45E-01	3,127092766	1,78E+00	1,82E-01	5,45E-01	Perm1		0,90	No
ENSMUSG00000078487	-2,70E+00	-3,033614577	2,89E+00	8,93E-02	3,79E-01	Ankrd65		0,15	No
ENSMUSG00000078489	-2,41E-01	-2,574228134	6,31E-02	8,02E-01	9,61E-01	Gm17106		0,85	No
ENSMUSG00000078490	-4,45E-01	1,41631653	6,61E+00	1,02E-02	9,45E-02	Cfap74		0,73	No
ENSMUSG00000078492	1,85E+00	-3,107657832	1,35E+00	2,46E-01	5,50E-01	1700045H11Rik		3,61	No
ENSMUSG00000078494	7,27E-01	-2,107725672	9,42E-01	3,32E-01	6,32E-01	Gm13201		1,65	No
ENSMUSG00000078495	-4,86E-02	5,305414209	4,79E-01	4,89E-01	7,76E-01	Zfp984		0,97	No
ENSMUSG00000078496	-8,42E-02	0,372054302	1,10E-01	7,40E-01	9,26E-01	Zfp982		0,94	No
ENSMUSG00000078497	5,07E-01	-2,497612096	3,50E-01	5,54E-01	8,20E-01	Zfp978		1,42	No
ENSMUSG00000078498	-4,62E-01	-1,627559535	6,80E-01	4,10E-01	7,06E-01	Zfp988		0,73	No
ENSMUSG00000078500	-1,22E+00	-2,750899356	1,15E+00	2,83E-01	5,79E-01	Zfp986		0,43	No
ENSMUSG00000078502	-1,10E-01	5,125925164	2,33E+00	1,27E-01	4,46E-01	Gm13212		0,93	No

ENSMUSG00000078503	8,52E-01	-2,179288738	1,23E+00	2,68E-01	5,65E-01	Zfp990	1,80	No
ENSMUSG00000078515	1,11E-01	7,064893207	3,41E+00	6,50E-02	3,19E-01	Ddi2	1,08	No
ENSMUSG00000078517	1,27E-01	5,604294917	3,48E+00	6,23E-02	3,11E-01	Emc1	1,09	No
ENSMUSG00000078519	6,07E-01	-1,627728985	9,61E-01	3,27E-01	6,28E-01	2310026L22Rik	1,52	No
ENSMUSG00000078521	4,02E-01	1,574548346	5,44E+00	1,96E-02	1,49E-01	Aunip	1,32	No
ENSMUSG00000078528	-1,71E+00	-2,548184036	2,03E+00	1,54E-01	4,98E-01	Gm17068	0,31	No
ENSMUSG00000078532	2,85E-02	2,808957702	5,81E-02	8,09E-01	9,65E-01	Nkain1	1,02	No
ENSMUSG00000078546	-9,88E-02	3,041879474	8,02E-01	3,70E-01	6,72E-01	Zfp995	0,93	No
ENSMUSG00000078552	-1,25E-01	3,122348626	1,30E+00	2,54E-01	5,50E-01	Dcdc2b	0,92	No
ENSMUSG00000078554	7,05E-01	-2,83200187	2,71E-01	6,03E-01	8,48E-01	Fam229a	1,63	No
ENSMUSG00000078566	4,50E-01	4,120247323	2,57E+01	3,93E-07	1,91E-05	Bnip3	1,37	Yes
ENSMUSG00000078570	-3,33E-03	3,322083733	1,01E-03	9,75E-01	1,00E+00	1110065P20Rik	1,00	No
ENSMUSG00000078572	4,35E-02	3,385719525	1,76E-01	6,75E-01	8,91E-01	Ndufaf8	1,03	No
ENSMUSG00000078578	-9,12E-02	8,182623487	2,48E+00	1,15E-01	4,20E-01	Ube2d3	0,94	No
ENSMUSG00000078580	3,78E-01	1,814068523	5,78E+00	1,62E-02	1,32E-01	E430018J23Rik	1,30	No
ENSMUSG00000078584	-5,22E-02	4,059332795	3,57E-01	5,50E-01	8,17E-01	AU022252	0,96	No
ENSMUSG00000078588	3,81E-01	-1,880147415	3,45E-01	5,57E-01	8,21E-01	Ccdc24	1,30	No
ENSMUSG00000078590	3,54E+00	-2,896165367	5,45E+00	1,96E-02	1,49E-01	Gm10959	11,62	No
ENSMUSG00000078592	8,89E-02	5,960948252	1,83E+00	1,77E-01	5,36E-01	Gm4609	1,06	No
ENSMUSG00000078597	2,75E-01	1,206684872	1,84E+00	1,75E-01	5,33E-01	Cyp4a12b	1,21	No
ENSMUSG00000078601	-1,35E+00	-2,574195026	1,14E+00	2,85E-01	5,82E-01	Gm12525	0,39	No
ENSMUSG00000078604	9,27E-02	-2,069990578	1,12E-02	9,16E-01	1,00E+00	Gm10961	1,07	No
ENSMUSG00000078606	-1,53E-01	7,710805494	6,76E+00	9,34E-03	8,87E-02	Gm4070	0,90	No
ENSMUSG00000078607	-4,32E-01	-0,726183182	1,23E+00	2,68E-01	5,64E-01	1810010H24Rik	0,74	No
ENSMUSG00000078611	7,95E-01	-1,946322427	1,55E+00	2,13E-01	5,50E-01	Gm5901	1,74	No
ENSMUSG00000078612	2,36E-01	3,194036342	4,81E+00	2,83E-02	1,91E-01	Fyb2	1,18	No
ENSMUSG00000078616	3,44E-01	1,915378664	5,00E+00	2,54E-02	1,76E-01	Trim30c	1,27	No
ENSMUSG00000078619	5,25E-02	6,552133269	7,06E-01	4,01E-01	6,99E-01	Smarcd2	1,04	No
ENSMUSG00000078620	1,41E-01	-0,997994629	9,92E-02	7,53E-01	9,33E-01	Gm10964	1,10	No
ENSMUSG00000078622	1,79E-01	6,009741207	7,47E+00	6,26E-03	6,63E-02	Ccdc47	1,13	No
ENSMUSG00000078626	2,65E+00	-3,033655087	2,73E+00	9,86E-02	3,91E-01	Gm12790	6,30	No
ENSMUSG00000078627	1,85E+00	-3,107657832	1,35E+00	2,46E-01	5,50E-01	March10	3,61	No

ENSMUSG00000078630	5,72E-02	2,90962331	2,47E-01	6,19E-01	8,56E-01	Tomt	1,04	No
ENSMUSG00000078632	-6,22E-01	-1,600721384	1,42E+00	2,33E-01	5,50E-01	Lrrc37a	0,65	No
ENSMUSG00000078635	-6,05E-01	-0,088688832	4,35E+00	3,69E-02	2,28E-01	1700028N14Rik	0,66	No
ENSMUSG00000078636	9,33E-02	7,776145624	2,53E+00	1,12E-01	4,11E-01	Gm7336	1,07	No
ENSMUSG00000078650	1,89E+00	-3,033614598	1,38E+00	2,40E-01	5,50E-01	G6pc	3,70	No
ENSMUSG00000078651	3,21E-02	2,475905829	6,07E-02	8,05E-01	9,63E-01	Aoc2	1,02	No
ENSMUSG00000078652	4,71E-02	6,739445756	5,82E-01	4,45E-01	7,36E-01	Psme3	1,03	No
ENSMUSG00000078653	-3,51E-02	4,607752829	1,98E-01	6,56E-01	8,80E-01	Cntd1	0,98	No
ENSMUSG00000078655	-1,14E-02	-3,033635021	1,50E-05	9,97E-01	1,00E+00	Gm10972	0,99	No
ENSMUSG00000078656	4,14E-03	4,615899168	2,76E-03	9,58E-01	1,00E+00	Vps25	1,00	No
ENSMUSG00000078668	3,14E-02	-3,107617333	-7,69E-10	1,00E+00	1,00E+00	Gm11595	1,02	No
ENSMUSG00000078670	-1,12E+00	-2,831960619	7,39E-01	3,90E-01	6,90E-01	Fam174b	0,46	No
ENSMUSG00000078671	-1,98E-01	6,857332203	1,05E+01	1,22E-03	1,91E-02	Chd2	0,87	Yes
ENSMUSG00000078676	-2,79E-02	5,963135151	1,82E-01	6,70E-01	8,88E-01	Casc3	0,98	No
ENSMUSG00000078677	-5,73E-01	-2,711646423	2,57E-01	6,12E-01	8,52E-01	Gm10974	0,67	No
ENSMUSG00000078681	1,39E-01	3,921622072	2,34E+00	1,26E-01	4,44E-01	Tm2d3	1,10	No
ENSMUSG00000078684	1,59E-01	5,476664999	5,29E+00	2,15E-02	1,58E-01	5830417I10Rik	1,12	No
ENSMUSG00000078691	-2,63E-02	-3,033653157	2,33E-04	9,88E-01	1,00E+00	Gm6821	0,98	No
ENSMUSG00000078695	-4,49E-02	2,91227306	1,53E-01	6,95E-01	9,02E-01	Cisd3	0,97	No
ENSMUSG00000078700	7,22E-02	2,352461046	2,81E-01	5,96E-01	8,44E-01	D030028A08Rik	1,05	No
ENSMUSG00000078713	-4,15E-03	4,847659197	2,99E-03	9,56E-01	1,00E+00	Tomm5	1,00	No
ENSMUSG00000078716	-3,19E-01	2,030341537	4,90E+00	2,68E-02	1,84E-01	Tmem8b	0,80	No
ENSMUSG00000078719	5,38E-03	3,052494462	2,38E-03	9,61E-01	1,00E+00	Msmp	1,00	No
ENSMUSG00000078722	-1,87E+00	-3,107637761	1,39E+00	2,38E-01	5,50E-01	Gm12394	0,27	No
ENSMUSG00000078735	1,24E-01	2,59562031	9,66E-01	3,26E-01	6,26E-01	Il11ra2	1,09	No
ENSMUSG00000078747	-2,34E+00	-2,997968205	2,15E+00	1,43E-01	4,77E-01	Gm20878	0,20	No
ENSMUSG00000078752	4,76E-01	-2,711673553	1,77E-01	6,74E-01	8,90E-01	Scgb1b30	1,39	No
ENSMUSG00000078762	-4,52E-02	3,211402438	1,84E-01	6,68E-01	8,86E-01	Haus5	0,97	No
ENSMUSG00000078763	-1,13E-01	4,262164887	1,80E+00	1,79E-01	5,40E-01	Slfn1	0,92	No
ENSMUSG00000078765	-5,52E-02	3,247572048	2,72E-01	6,02E-01	8,48E-01	U2af1l4	0,96	No
ENSMUSG00000078768	1,57E-01	0,529866566	4,31E-01	5,12E-01	7,94E-01	Zfp566	1,11	No
ENSMUSG00000078771	-3,20E-03	7,176810961	2,83E-03	9,58E-01	1,00E+00	Evi2a	1,00	No

ENSMUSG00000078772	-8,31E-02	0,530112464	1,16E-01	7,34E-01	9,23E-01	Gm12353	0,94	No
ENSMUSG00000078773	1,91E-01	3,142512904	3,13E+00	7,70E-02	3,54E-01	Rad54b	1,14	No
ENSMUSG00000078776	-8,20E-02	0,327810896	1,07E-01	7,44E-01	9,29E-01	9530053A07Rik	0,94	No
ENSMUSG00000078779	2,30E-01	1,946539508	2,41E+00	1,21E-01	4,34E-01	Zfp59	1,17	No
ENSMUSG00000078780	-1,07E-01	0,975411923	2,47E-01	6,19E-01	8,56E-01	Gm5150	0,93	No
ENSMUSG00000078783	2,74E-01	-0,201019989	6,65E-01	4,15E-01	7,10E-01	Gm9733	1,21	No
ENSMUSG00000078784	-3,94E-01	2,041122665	6,84E+00	8,92E-03	8,58E-02	1810022K09Rik	0,76	No
ENSMUSG00000078786	-8,24E-02	3,563112413	7,10E-01	3,99E-01	6,98E-01	BC024978	0,94	No
ENSMUSG00000078787	3,30E-01	0,17940315	1,46E+00	2,26E-01	5,50E-01	Cyp2t4	1,26	No
ENSMUSG00000078789	4,11E-02	3,955258206	2,10E-01	6,47E-01	8,74E-01	Dph1	1,03	No
ENSMUSG00000078794	-4,69E-01	0,683645939	4,20E+00	4,03E-02	2,38E-01	Dact3	0,72	No
ENSMUSG00000078795	-1,40E+00	2,045490397	8,44E+01	4,03E-20	9,14E-18	Ceacam15	0,38	Yes
ENSMUSG00000078796	5,92E-01	-0,755712127	2,14E+00	1,43E-01	4,78E-01	Zfp541	1,51	No
ENSMUSG00000078810	-7,13E-02	-2,711744017	3,42E-03	9,53E-01	1,00E+00	Gp6	0,95	No
ENSMUSG00000078812	4,69E-02	8,794509327	6,75E-01	4,11E-01	7,07E-01	Eif5a	1,03	No
ENSMUSG00000078813	-1,17E-01	3,535194887	1,48E+00	2,24E-01	5,50E-01	Leng1	0,92	No
ENSMUSG00000078815	-7,52E-01	-1,801037999	1,48E+00	2,24E-01	5,50E-01	Cacng6	0,59	No
ENSMUSG00000078816	-2,64E-01	3,040960639	5,26E+00	2,18E-02	1,59E-01	Prkcg	0,83	No
ENSMUSG00000078817	-5,38E-02	-2,600718632	2,63E-03	9,59E-01	1,00E+00	Nlrp12	0,96	No
ENSMUSG00000078838	7,54E-01	-2,89614743	3,10E-01	5,78E-01	8,35E-01	Gm17382	1,69	No
ENSMUSG00000078850	6,69E-03	-0,980420129	2,34E-04	9,88E-01	1,00E+00	Gm2348	1,00	No
ENSMUSG00000078851	6,86E-04	2,901625353	3,59E-05	9,95E-01	1,00E+00	Hist3h2a	1,00	No
ENSMUSG00000078853	-1,61E-01	3,620709831	2,81E+00	9,39E-02	3,84E-01	Igtp	0,89	No
ENSMUSG00000078861	-3,12E-01	1,630036571	3,45E+00	6,33E-02	3,14E-01	Zfp931	0,81	No
ENSMUSG00000078862	-8,65E-02	2,81581342	5,32E-01	4,66E-01	7,54E-01	Gm14326	0,94	No
ENSMUSG00000078864	2,59E-01	-1,015787184	3,23E-01	5,70E-01	8,31E-01	Gm14322	1,20	No
ENSMUSG00000078865	1,81E+00	-3,10765737	1,28E+00	2,58E-01	5,50E-01	Gm14406	3,49	No
ENSMUSG00000078866	1,45E-01	2,015185392	1,01E+00	3,15E-01	6,16E-01	Zfp970	1,11	No
ENSMUSG00000078867	3,87E-01	-0,305944017	1,32E+00	2,50E-01	5,50E-01	Gm14418	1,31	No
ENSMUSG00000078868	-1,34E+00	-2,770542632	1,12E+00	2,89E-01	5,85E-01	Gm14412	0,40	No
ENSMUSG00000078869	-7,07E-01	-2,997998279	2,00E-01	6,54E-01	8,79E-01	Gm14409	0,61	No
ENSMUSG00000078870	7,09E-03	1,988686749	2,24E-03	9,62E-01	1,00E+00	Gm14410	1,00	No

ENSMUSG00000078872	1,20E+00	-3,146123991	6,74E-01	4,12E-01	7,07E-01	Gm14401	2,30	No
ENSMUSG00000078875	-1,01E-01	1,295404193	2,94E-01	5,88E-01	8,40E-01	Gm14419	0,93	No
ENSMUSG00000078877	-2,48E-02	2,083914743	2,85E-02	8,66E-01	9,99E-01	Gm14295	0,98	No
ENSMUSG00000078878	7,43E-02	2,420205834	3,19E-01	5,72E-01	8,32E-01	Gm14305	1,05	No
ENSMUSG00000078879	-1,19E-01	2,303662197	7,45E-01	3,88E-01	6,88E-01	Zfp973	0,92	No
ENSMUSG00000078880	-1,12E-01	1,825499142	5,17E-01	4,72E-01	7,60E-01	Gm14308	0,93	No
ENSMUSG00000078881	7,13E-02	1,679553352	1,86E-01	6,67E-01	8,86E-01	Gm14434	1,05	No
ENSMUSG00000078886	4,17E-02	1,889826325	7,11E-02	7,90E-01	9,54E-01	Gm2026	1,03	No
ENSMUSG00000078887	1,84E-01	1,803775301	1,35E+00	2,45E-01	5,50E-01	Gm6710	1,14	No
ENSMUSG00000078889	9,39E-02	1,587034456	3,11E-01	5,77E-01	8,35E-01	Gm14288	1,07	No
ENSMUSG00000078891	2,37E-01	-0,912513731	3,18E-01	5,73E-01	8,32E-01	Gm11008	1,18	No
ENSMUSG00000078894	1,09E-01	1,895644719	4,89E-01	4,85E-01	7,72E-01	2210418O10Rik	1,08	No
ENSMUSG00000078895	-1,87E+00	-3,107637761	1,39E+00	2,38E-01	5,50E-01	Gm11009	0,27	No
ENSMUSG00000078896	-2,68E-01	2,14809071	3,57E+00	5,90E-02	3,00E-01	Zfp965	0,83	No
ENSMUSG00000078897	8,46E-02	1,71421964	2,67E-01	6,06E-01	8,49E-01	Gm4724	1,06	No
ENSMUSG00000078898	5,86E-02	1,382800084	1,07E-01	7,44E-01	9,29E-01	Zfp968	1,04	No
ENSMUSG00000078899	4,80E-02	1,353145844	7,02E-02	7,91E-01	9,55E-01	Gm4631	1,03	No
ENSMUSG00000078901	1,83E-01	1,731408591	1,26E+00	2,61E-01	5,55E-01	Gm14440	1,14	No
ENSMUSG00000078902	-2,17E-01	-1,389697477	1,84E-01	6,68E-01	8,87E-01	Gm14443	0,86	No
ENSMUSG00000078903	-1,39E-02	2,100139566	9,31E-03	9,23E-01	1,00E+00	Gm14391	0,99	No
ENSMUSG00000078905	1,03E+00	-1,641293458	3,53E+00	6,03E-02	3,04E-01	Gm14393	2,04	No
ENSMUSG00000078906	6,13E-01	-3,070153683	1,50E-01	6,98E-01	9,04E-01	Gm14444	1,53	No
ENSMUSG00000078907	-7,13E-01	-2,548239305	5,68E-01	4,51E-01	7,41E-01	Fam186b	0,61	No
ENSMUSG00000078908	-7,78E-02	5,247444275	1,21E+00	2,71E-01	5,67E-01	Mon1b	0,95	No
ENSMUSG00000078915	-1,15E+00	-2,963255048	5,80E-01	4,46E-01	7,37E-01	Hsp25-ps1	0,45	No
ENSMUSG00000078917	4,65E-01	-2,711720744	1,69E-01	6,81E-01	8,94E-01	Gm11281	1,38	No
ENSMUSG00000078919	-1,67E-01	4,197633073	3,87E+00	4,92E-02	2,68E-01	Dpm1	0,89	No
ENSMUSG00000078920	-7,42E-02	4,543088271	8,69E-01	3,51E-01	6,53E-01	Ifi47	0,95	No
ENSMUSG00000078921	-4,99E-01	1,379789025	7,73E+00	5,45E-03	5,95E-02	Tgtp2	0,71	No
ENSMUSG00000078922	-4,37E-01	0,599768372	3,48E+00	6,23E-02	3,11E-01	Tgtp1	0,74	No
ENSMUSG00000078923	-7,75E-02	5,236452016	1,17E+00	2,79E-01	5,75E-01	Ube2v1	0,95	No
ENSMUSG00000078925	1,84E+00	-3,033649193	1,33E+00	2,49E-01	5,50E-01	D030018L15Rik	3,58	No

ENSMUSG00000078926	1,85E+00	-3,107657832	1,35E+00	2,46E-01	5,50E-01	Cdc20b	3,61	No
ENSMUSG00000078931	1,99E-01	3,68815107	4,31E+00	3,78E-02	2,31E-01	Pdf	1,15	No
ENSMUSG00000078937	2,69E-01	1,20284327	1,90E+00	1,68E-01	5,23E-01	Cpt1b	1,20	No
ENSMUSG00000078941	-4,36E-02	5,079696276	3,51E-01	5,53E-01	8,19E-01	Ak6	0,97	No
ENSMUSG00000078942	9,57E-02	5,959511211	2,12E+00	1,45E-01	4,82E-01	Naip6	1,07	No
ENSMUSG00000078945	1,20E-01	6,521222174	3,69E+00	5,46E-02	2,86E-01	Naip2	1,09	No
ENSMUSG00000078952	2,00E-01	-2,311145575	5,99E-02	8,07E-01	9,64E-01	Lncenc1	1,15	No
ENSMUSG00000078953	-1,98E-02	-3,033655952	2,83E-11	1,00E+00	1,00E+00	Gm11020	0,99	No
ENSMUSG00000078954	-8,02E-01	-1,107560534	2,70E+00	1,00E-01	3,92E-01	Arhgap8	0,57	No
ENSMUSG00000078956	9,37E-02	1,156152284	2,50E-01	6,17E-01	8,55E-01	Gm14221	1,07	No
ENSMUSG00000078963	-1,52E+00	-2,548192793	2,41E+00	1,21E-01	4,33E-01	Hsbp1l1	0,35	No
ENSMUSG00000078965	8,31E-02	7,964487931	2,03E+00	1,54E-01	4,97E-01	Gm12033	1,06	No
ENSMUSG00000078967	3,69E-02	5,998437918	3,16E-01	5,74E-01	8,33E-01	Gm3272	1,03	No
ENSMUSG00000078970	8,71E-03	4,899976334	1,34E-02	9,08E-01	1,00E+00	Wdr92	1,01	No
ENSMUSG00000078974	-9,85E-02	1,262767321	2,78E-01	5,98E-01	8,45E-01	Sec61g	0,93	No
ENSMUSG00000078984	1,81E+00	-3,10765737	1,28E+00	2,58E-01	5,50E-01	Gm11027	3,49	No
ENSMUSG00000078994	-6,94E-02	2,764953788	3,37E-01	5,62E-01	8,25E-01	Zfp429	0,95	No
ENSMUSG00000078995	1,27E-01	1,737891647	6,04E-01	4,37E-01	7,30E-01	Zfp456	1,09	No
ENSMUSG00000079001	-3,22E+00	-2,770613515	4,38E+00	3,65E-02	2,27E-01	4930404H24Rik	0,11	No
ENSMUSG00000079003	1,59E-01	5,611981551	5,46E+00	1,94E-02	1,48E-01	Samd1	1,12	No
ENSMUSG00000079008	-2,27E+00	-2,497567472	4,19E+00	4,05E-02	2,38E-01	Gm14124	0,21	No
ENSMUSG00000079009	1,38E+00	-2,600725539	1,97E+00	1,61E-01	5,09E-01	Gm14139	2,61	No
ENSMUSG00000079014	-6,89E-01	-1,116986214	2,46E+00	1,17E-01	4,23E-01	Serpina3i	0,62	No
ENSMUSG00000079016	1,85E+00	-3,107657832	1,35E+00	2,46E-01	5,50E-01	Gm11034	3,61	No
ENSMUSG00000079017	2,72E-04	5,318110714	1,49E-05	9,97E-01	1,00E+00	Ifi27l2a	1,00	No
ENSMUSG00000079018	4,66E-01	0,755319941	4,12E+00	4,25E-02	2,45E-01	Ly6c1	1,38	No
ENSMUSG00000079019	-4,57E-01	0,474434669	3,50E+00	6,14E-02	3,08E-01	Insl3	0,73	No
ENSMUSG00000079020	1,73E-01	6,135752523	7,17E+00	7,42E-03	7,49E-02	Slc45a4	1,13	No
ENSMUSG00000079024	1,81E+00	-3,10765737	1,28E+00	2,58E-01	5,50E-01	Gm21961	3,49	No
ENSMUSG00000079029	-2,76E+00	-1,655078482	2,10E+01	4,51E-06	1,65E-04	Gm5662	0,15	Yes
ENSMUSG00000079031	-2,70E+00	-3,033614577	2,89E+00	8,93E-02	3,79E-01	BB287469	0,15	No
ENSMUSG00000079033	-8,15E-01	-2,655140165	6,62E-01	4,16E-01	7,11E-01	Mef2b	0,57	No

ENSMUSG00000079034	-1,74E+00	-1,863691678	7,31E+00	6,84E-03	7,05E-02	Gm8300	0,30	No
ENSMUSG00000079036	-7,32E-02	5,174512541	1,02E+00	3,12E-01	6,12E-01	Alkbh1	0,95	No
ENSMUSG00000079037	-5,58E-01	4,454179554	4,79E+01	4,45E-12	5,05E-10	Prnp	0,68	Yes
ENSMUSG00000079038	-7,22E-01	3,103368103	4,42E+01	2,96E-11	2,98E-09	D130040H23Rik	0,61	Yes
ENSMUSG00000079043	2,02E-01	1,924797189	1,83E+00	1,77E-01	5,36E-01	Fastkd5	1,15	No
ENSMUSG00000079049	-3,66E-01	-2,311136858	1,69E-01	6,81E-01	8,94E-01	Serpinb1c	0,78	No
ENSMUSG00000079051	-1,25E+00	-2,655145306	1,51E+00	2,19E-01	5,50E-01	Gm14025	0,42	No
ENSMUSG00000079053	3,91E-01	-0,896101573	7,06E-01	4,01E-01	6,99E-01	Gm14010	1,31	No
ENSMUSG00000079055	-4,87E-02	-2,896124877	1,54E-03	9,69E-01	1,00E+00	Slc8a3	0,97	No
ENSMUSG00000079056	5,75E-02	2,302689164	1,79E-01	6,72E-01	8,89E-01	Kcnip3	1,04	No
ENSMUSG00000079057	7,59E-02	5,499465906	1,21E+00	2,72E-01	5,69E-01	Cyp4v3	1,05	No
ENSMUSG00000079061	1,82E+00	-2,963269943	1,32E+00	2,51E-01	5,50E-01	Gm11042	3,54	No
ENSMUSG00000079065	8,50E-02	3,013370115	5,71E-01	4,50E-01	7,40E-01	BC005561	1,06	No
ENSMUSG00000079067	1,70E-02	3,754823866	3,20E-02	8,58E-01	9,94E-01	Hmgn2-ps1	1,01	No
ENSMUSG00000079069	4,99E-01	-2,107675527	4,82E-01	4,87E-01	7,74E-01	8430423G03Rik	1,41	No
ENSMUSG00000079071	-1,24E-01	-0,963169262	8,39E-02	7,72E-01	9,43E-01	Gm14085	0,92	No
ENSMUSG00000079076	4,89E-01	1,005434985	5,93E+00	1,49E-02	1,25E-01	Gm3086	1,40	No
ENSMUSG00000079083	2,28E-01	3,210445785	4,70E+00	3,02E-02	1,99E-01	Jrkl	1,17	No
ENSMUSG00000079084	-1,70E-02	6,258474232	7,10E-02	7,90E-01	9,54E-01	Ccdc82	0,99	No
ENSMUSG00000079101	1,72E-02	5,070847459	5,45E-02	8,15E-01	9,69E-01	Gm2904	1,01	No
ENSMUSG00000079103	-5,05E-02	-2,77063389	1,73E-03	9,67E-01	1,00E+00	Tgm7	0,97	No
ENSMUSG00000079104	-1,96E-02	4,715130039	6,46E-02	7,99E-01	9,60E-01	Prps1l3	0,99	No
ENSMUSG00000079105	-7,38E-02	-3,033659033	2,66E-03	9,59E-01	1,00E+00	C7	0,95	No
ENSMUSG00000079108	-6,84E-02	5,261653257	9,24E-01	3,36E-01	6,37E-01	Srp54b	0,95	No
ENSMUSG00000079109	-2,26E-02	3,639824016	5,57E-02	8,13E-01	9,68E-01	Pms2	0,98	No
ENSMUSG00000079110	-2,80E-01	0,563372645	1,35E+00	2,45E-01	5,50E-01	Capn3	0,82	No
ENSMUSG00000079111	1,82E-01	5,793116188	7,42E+00	6,45E-03	6,76E-02	Kdelr2	1,13	No
ENSMUSG00000079137	-2,70E-01	3,962500824	9,15E+00	2,49E-03	3,32E-02	Rpl27-ps1	0,83	Yes
ENSMUSG00000079138	-7,67E-02	1,148515746	1,52E-01	6,97E-01	9,03E-01	Gm8818	0,95	No
ENSMUSG00000079139	-3,48E-01	8,336761434	3,64E+01	1,61E-09	1,27E-07	Gm4204	0,79	Yes
ENSMUSG00000079140	9,96E-02	2,112116973	4,78E-01	4,89E-01	7,76E-01	Gm6374	1,07	No
ENSMUSG00000079143	-6,16E-01	-1,185566647	1,70E+00	1,92E-01	5,50E-01	Atxn7l1os1	0,65	No

ENSMUSG00000079144	-1,28E-01	2,627225286	1,07E+00	3,00E-01	5,96E-01	A130010J15Rik	0,91	No
ENSMUSG00000079146	-5,69E-02	-3,107638538	1,33E-03	9,71E-01	1,00E+00	Gm15353	0,96	No
ENSMUSG00000079162	1,40E-01	-2,107508982	3,41E-02	8,53E-01	9,91E-01	Trim43b	1,10	No
ENSMUSG00000079164	-2,98E-01	0,087243463	1,17E+00	2,80E-01	5,77E-01	Tlr5	0,81	No
ENSMUSG00000079165	-5,70E-02	4,245619417	4,63E-01	4,96E-01	7,81E-01	Sap25	0,96	No
ENSMUSG00000079173	-5,04E-01	-1,863527134	6,16E-01	4,32E-01	7,26E-01	Zan	0,70	No
ENSMUSG00000079177	-3,76E-02	0,266474509	1,92E-02	8,90E-01	1,00E+00	Fam228a	0,97	No
ENSMUSG00000079179	-1,63E-01	5,009361291	4,81E+00	2,82E-02	1,90E-01	Rab10os	0,89	No
ENSMUSG00000079184	6,73E-02	4,423507243	6,96E-01	4,04E-01	7,01E-01	Mphosph8	1,05	No
ENSMUSG00000079186	-8,31E-01	-2,96327241	3,77E-01	5,39E-01	8,09E-01	Gzmc	0,56	No
ENSMUSG00000079197	-4,92E-01	2,265724974	1,34E+01	2,51E-04	5,26E-03	Psme2	0,71	Yes
ENSMUSG00000079215	1,09E-01	5,742168492	2,63E+00	1,05E-01	3,97E-01	Zfp664	1,08	No
ENSMUSG00000079224	-9,36E-02	2,881805387	6,51E-01	4,20E-01	7,15E-01	Gm6565	0,94	No
ENSMUSG00000079225	6,86E-02	7,040498666	1,28E+00	2,57E-01	5,50E-01	Gm9531	1,05	No
ENSMUSG00000079227	2,63E-01	9,205464805	2,14E+01	3,75E-06	1,42E-04	Ccr5	1,20	Yes
ENSMUSG00000079235	1,34E-02	-2,963213009	-8,25E-12	1,00E+00	1,00E+00	Ccdc13	1,01	No
ENSMUSG00000079242	-2,77E-01	2,111353187	3,55E+00	5,95E-02	3,02E-01	C730034F03Rik	0,83	No
ENSMUSG00000079243	3,50E+00	-2,832056819	5,31E+00	2,12E-02	1,57E-01	Xirp1	11,29	No
ENSMUSG00000079247	2,86E-01	1,36034729	2,48E+00	1,15E-01	4,21E-01	Gm13691	1,22	No
ENSMUSG00000079258	-1,94E+00	-3,107619243	1,50E+00	2,21E-01	5,50E-01	Calhm1	0,26	No
ENSMUSG00000079259	1,85E+00	-3,107657832	1,35E+00	2,46E-01	5,50E-01	Trim71	3,61	No
ENSMUSG00000079260	4,46E-02	4,090724683	2,64E-01	6,07E-01	8,50E-01	Tmppe	1,03	No
ENSMUSG00000079262	-1,87E+00	-3,107637761	1,39E+00	2,38E-01	5,50E-01	Slco1a6	0,27	No
ENSMUSG00000079264	-1,94E+00	-3,107619243	1,50E+00	2,21E-01	5,50E-01	Gm11077	0,26	No
ENSMUSG00000079283	-1,93E-01	1,84921206	1,53E+00	2,16E-01	5,50E-01	2310009B15Rik	0,87	No
ENSMUSG00000079286	-6,20E-01	-1,088751655	1,95E+00	1,63E-01	5,13E-01	Gm11084	0,65	No
ENSMUSG00000079293	1,04E-01	9,934226706	3,40E+00	6,51E-02	3,20E-01	Clec7a	1,07	No
ENSMUSG00000079295	-2,71E+00	-3,033620915	2,92E+00	8,75E-02	3,78E-01	Klrb1-ps1	0,15	No
ENSMUSG00000079298	1,77E-01	5,014113162	5,55E+00	1,85E-02	1,44E-01	Klrb1b	1,13	No
ENSMUSG00000079304	1,27E-01	0,284593025	2,29E-01	6,33E-01	8,65E-01	4933413G19Rik	1,09	No
ENSMUSG00000079311	-1,79E-02	3,934497318	3,90E-02	8,43E-01	9,85E-01	Gm3222	0,99	No
ENSMUSG00000079323	-2,28E-01	1,150356412	1,42E+00	2,33E-01	5,50E-01	Gm20661	0,85	No

ENSMUSG00000079330	2,65E+00	-3,033655087	2,73E+00	9,86E-02	3,91E-01	Lemd1	6,30	No
ENSMUSG00000079333	1,21E+00	-3,07015885	6,74E-01	4,12E-01	7,07E-01	Gm11096	2,31	No
ENSMUSG00000079334	9,84E-02	3,422375853	9,64E-01	3,26E-01	6,27E-01	Nat6	1,07	No
ENSMUSG00000079339	-3,62E-01	0,28136595	1,96E+00	1,62E-01	5,11E-01	Ifit1bl1	0,78	No
ENSMUSG00000079343	4,92E-01	-2,311231094	3,28E-01	5,67E-01	8,29E-01	C1s2	1,41	No
ENSMUSG00000079344	-1,89E+00	-3,033648118	1,42E+00	2,33E-01	5,50E-01	Lipo4	0,27	No
ENSMUSG00000079346	1,81E+00	-3,10765737	1,28E+00	2,58E-01	5,50E-01	1700013D24Rik	3,49	No
ENSMUSG00000079355	6,08E-02	1,473916685	1,16E-01	7,33E-01	9,23E-01	Ackr4	1,04	No
ENSMUSG00000079356	4,22E-01	-2,96325956	9,01E-02	7,64E-01	9,39E-01	Mettl4-ps1	1,34	No
ENSMUSG00000079357	-1,22E-01	2,233328328	7,46E-01	3,88E-01	6,88E-01	Gm11100	0,92	No
ENSMUSG00000079362	-7,14E-02	1,71979324	1,95E-01	6,59E-01	8,81E-01	Gm43302	0,95	No
ENSMUSG00000079363	-1,71E-01	4,293364194	4,25E+00	3,93E-02	2,35E-01	Gbp4	0,89	No
ENSMUSG00000079364	7,67E-01	-2,963261668	3,24E-01	5,69E-01	8,30E-01	Gm3558	1,70	No
ENSMUSG00000079382	-5,88E-02	-3,033652696	1,62E-03	9,68E-01	1,00E+00	Gm3443	0,96	No
ENSMUSG00000079383	1,85E+00	-3,107657832	1,35E+00	2,46E-01	5,50E-01	Gm3298	3,61	No
ENSMUSG00000079386	2,61E+00	-3,033672762	2,63E+00	1,05E-01	3,97E-01	Gm3173	6,10	No
ENSMUSG00000079388	-1,89E+00	-3,1076441	1,42E+00	2,33E-01	5,50E-01	2610042L04Rik	0,27	No
ENSMUSG00000079391	-1,87E+00	-2,963234282	1,41E+00	2,35E-01	5,50E-01	Gm2974	0,27	No
ENSMUSG00000079396	1,81E+00	-2,963282131	1,30E+00	2,54E-01	5,50E-01	Gm3411	3,50	No
ENSMUSG00000079402	-2,66E+00	-3,033639428	2,81E+00	9,35E-02	3,83E-01	Gm3020	0,16	No
ENSMUSG00000079407	1,86E+00	-3,033661846	1,35E+00	2,46E-01	5,50E-01	1700110I01Rik	3,62	No
ENSMUSG00000079410	-8,63E-01	-2,831945656	4,08E-01	5,23E-01	8,01E-01	Gm2897	0,55	No
ENSMUSG00000079414	-2,60E-01	1,497579692	2,15E+00	1,43E-01	4,78E-01	Gm11110	0,84	No
ENSMUSG00000079415	1,20E-01	0,179581229	1,77E-01	6,74E-01	8,90E-01	Cntf	1,09	No
ENSMUSG00000079419	2,37E-01	3,44047099	5,36E+00	2,06E-02	1,54E-01	Ms4a6c	1,18	No
ENSMUSG00000079421	-1,87E+00	-3,033619006	1,41E+00	2,36E-01	5,50E-01	4930402F06Rik	0,27	No
ENSMUSG00000079426	-5,03E-02	8,198259966	7,57E-01	3,84E-01	6,86E-01	Arpc4	0,97	No
ENSMUSG00000079427	-2,13E-01	4,795019869	7,86E+00	5,04E-03	5,63E-02	Mthfsl	0,86	No
ENSMUSG00000079429	1,96E-02	3,479940348	3,74E-02	8,47E-01	9,87E-01	Mroh2a	1,01	No
ENSMUSG00000079433	9,01E-03	-3,107646452	-5,16E-10	1,00E+00	1,00E+00	Gm11114	1,01	No
ENSMUSG00000079434	-3,83E-02	-3,107651936	-7,91E-11	1,00E+00	1,00E+00	Neu2	0,97	No
ENSMUSG00000079436	1,53E-01	-1,614194475	7,31E-02	7,87E-01	9,53E-01	Kcnj13	1,11	No

ENSMUSG00000079442	1,45E-01	5,889359444	4,75E+00	2,92E-02	1,95E-01	St6galnac4	1,11	No
ENSMUSG00000079444	3,54E-02	4,890144343	2,19E-01	6,40E-01	8,70E-01	Gm21981	1,02	No
ENSMUSG00000079445	1,87E-01	5,087082652	6,39E+00	1,15E-02	1,03E-01	B3gnt7	1,14	No
ENSMUSG00000079451	-1,89E+00	-3,1076441	1,42E+00	2,33E-01	5,50E-01	Tmprss11g	0,27	No
ENSMUSG00000079455	-4,16E-01	0,30738232	2,58E+00	1,08E-01	4,03E-01	Gm16026	0,75	No
ENSMUSG00000079457	-4,76E-01	0,438729271	3,52E+00	6,05E-02	3,05E-01	Gm7609	0,72	No
ENSMUSG00000079462	-1,16E+00	-2,997970125	5,91E-01	4,42E-01	7,34E-01	Gm15737	0,45	No
ENSMUSG00000079465	-6,14E-01	-2,311153877	5,14E-01	4,73E-01	7,61E-01	Col4a3	0,65	No
ENSMUSG00000079467	3,73E-01	0,26151137	2,05E+00	1,52E-01	4,94E-01	Gm14966	1,30	No
ENSMUSG00000079469	4,69E-02	4,438732934	3,37E-01	5,62E-01	8,25E-01	Pigb	1,03	No
ENSMUSG00000079470	6,92E-01	4,152457655	6,40E+01	1,24E-15	2,04E-13	Utp14b	1,62	Yes
ENSMUSG00000079477	2,24E-02	8,899801425	1,55E-01	6,94E-01	9,02E-01	Rab7	1,02	No
ENSMUSG00000079478	1,07E-01	5,552311483	2,44E+00	1,18E-01	4,27E-01	Sssca1	1,08	No
ENSMUSG00000079484	-1,17E-01	0,577150126	2,32E-01	6,30E-01	8,62E-01	Phyhd1	0,92	No
ENSMUSG00000079491	-1,78E-01	3,073742242	2,71E+00	9,98E-02	3,92E-01	H2-T10	0,88	No
ENSMUSG00000079492	-2,80E-01	3,127751138	6,68E+00	9,75E-03	9,16E-02	Gm11127	0,82	No
ENSMUSG00000079494	-1,94E+00	-3,107619243	1,50E+00	2,21E-01	5,50E-01	Nat8f5	0,26	No
ENSMUSG00000079497	-9,83E-01	-2,311122782	1,17E+00	2,79E-01	5,76E-01	Gm13420	0,51	No
ENSMUSG00000079499	-1,36E-01	5,099046922	3,50E+00	6,14E-02	3,08E-01	6530402F18Rik	0,91	No
ENSMUSG00000079501	1,21E-01	6,218197607	3,54E+00	5,98E-02	3,03E-01	Gm5138	1,09	No
ENSMUSG00000079502	1,20E+00	-2,655165226	8,97E-01	3,43E-01	6,44E-01	Cfap77	2,30	No
ENSMUSG00000079505	-3,19E-01	3,798448089	1,21E+01	5,12E-04	9,35E-03	Gm11131	0,80	Yes
ENSMUSG00000079507	-1,59E-01	4,737041501	4,32E+00	3,78E-02	2,31E-01	H2-Q1	0,90	No
ENSMUSG00000079511	1,04E-01	3,689950917	1,20E+00	2,74E-01	5,70E-01	Gm42688	1,07	No
ENSMUSG00000079523	5,95E-02	6,958776191	9,54E-01	3,29E-01	6,29E-01	Tmsb10	1,04	No
ENSMUSG00000079546	-1,94E+00	-3,107619243	1,50E+00	2,21E-01	5,50E-01	Gm11146	0,26	No
ENSMUSG00000079547	1,38E-01	8,812432229	5,84E+00	1,57E-02	1,30E-01	H2-DMb1	1,10	No
ENSMUSG00000079550	2,48E-01	1,375712244	1,85E+00	1,74E-01	5,32E-01	Mpp4	1,19	No
ENSMUSG00000079553	2,62E-01	5,259028902	1,34E+01	2,49E-04	5,23E-03	Kifc1	1,20	Yes
ENSMUSG00000079554	8,85E-02	-1,165707153	3,29E-02	8,56E-01	9,93E-01	Aox2	1,06	No
ENSMUSG00000079555	-1,09E-02	4,328371577	1,75E-02	8,95E-01	1,00E+00	Haus3	0,99	No
ENSMUSG00000079557	-1,20E-02	6,176420445	3,49E-02	8,52E-01	9,90E-01	March2	0,99	No

ENSMUSG00000079560	-2,70E-02	-2,473432635	8,83E-04	9,76E-01	1,00E+00	Hoxa3	0,98	No
ENSMUSG00000079562	-9,22E-02	6,883595895	2,28E+00	1,31E-01	4,55E-01	Maea	0,94	No
ENSMUSG00000079563	1,38E-02	-0,52275864	1,46E-03	9,70E-01	1,00E+00	Pglyrp2	1,01	No
ENSMUSG00000079564	2,15E-01	-2,832010729	3,37E-02	8,54E-01	9,92E-01	Gm11149	1,16	No
ENSMUSG00000079575	-1,39E-01	7,556732446	5,52E+00	1,88E-02	1,45E-01	Rbpsuh-rs3	0,91	No
ENSMUSG00000079580	1,07E-01	-1,770635	2,50E-02	8,74E-01	1,00E+00	Tmem217	1,08	No
ENSMUSG00000079592	-5,28E-03	-3,045959076	5,58E-12	1,00E+00	1,00E+00	C1qtnf5	1,00	No
ENSMUSG00000079594	-5,10E-01	-2,800923688	1,31E-01	7,18E-01	9,14E-01	BC117090	0,70	No
ENSMUSG00000079597	-1,56E-01	2,646062851	1,57E+00	2,10E-01	5,50E-01	Gm5483	0,90	No
ENSMUSG00000079598	5,83E-01	3,248462059	2,90E+01	7,28E-08	4,18E-06	Clec2l	1,50	Yes
ENSMUSG00000079602	1,17E+00	-3,14612376	6,40E-01	4,24E-01	7,18E-01	9230102O04Rik	2,26	No
ENSMUSG00000079605	5,21E-02	2,726816086	1,91E-01	6,62E-01	8,83E-01	Zbtb9	1,04	No
ENSMUSG00000079608	-4,69E-01	0,006647279	2,48E+00	1,15E-01	4,20E-01	Stard6	0,72	No
ENSMUSG00000079610	-1,35E-02	2,887726584	1,37E-02	9,07E-01	1,00E+00	Ankrd39	0,99	No
ENSMUSG00000079614	1,57E-01	5,937013789	5,69E+00	1,71E-02	1,36E-01	Seh1l	1,11	No
ENSMUSG00000079620	-3,83E-02	-3,107651936	-7,91E-11	1,00E+00	1,00E+00	Muc4	0,97	No
ENSMUSG00000079625	-4,17E-01	2,656642903	1,15E+01	6,96E-04	1,20E-02	Tm4sf19	0,75	Yes
ENSMUSG00000079644	6,03E-01	-2,600741335	4,06E-01	5,24E-01	8,01E-01	Gm1110	1,52	No
ENSMUSG00000079645	-1,47E-01	-0,614241747	1,90E-01	6,63E-01	8,83E-01	Gm17193	0,90	No
ENSMUSG00000079652	-1,01E+00	-1,96317634	1,57E+00	2,10E-01	5,50E-01	Fam71f2	0,50	No
ENSMUSG00000079654	1,84E+00	-3,033632273	1,33E+00	2,49E-01	5,50E-01	Prrt4	3,58	No
ENSMUSG00000079657	-5,03E-01	0,019652956	3,17E+00	7,51E-02	3,49E-01	Rab26	0,71	No
ENSMUSG00000079658	-1,52E-01	5,382127241	4,70E+00	3,01E-02	1,99E-01	Eloc	0,90	No
ENSMUSG00000079659	7,84E-02	4,632868172	9,95E-01	3,19E-01	6,19E-01	Tmem243	1,06	No
ENSMUSG00000079671	6,07E-01	0,148520812	4,42E+00	3,55E-02	2,23E-01	2610203C22Rik	1,52	No
ENSMUSG00000079677	2,94E-02	3,028329101	6,88E-02	7,93E-01	9,56E-01	Fdx1l	1,02	No
ENSMUSG00000079681	2,05E-01	-1,831956519	8,51E-02	7,70E-01	9,42E-01	Zglp1	1,15	No
ENSMUSG00000079685	7,24E-02	7,484893949	1,49E+00	2,22E-01	5,50E-01	Ulbp1	1,05	No
ENSMUSG00000079700	2,13E+00	-2,770604555	2,47E+00	1,16E-01	4,21E-01	Fpr3	4,38	No
ENSMUSG00000079707	-3,13E-01	-1,44071963	3,60E-01	5,48E-01	8,15E-01	Tcte3	0,80	No
ENSMUSG00000079710	-3,05E-02	-0,468917142	7,57E-03	9,31E-01	1,00E+00	Gm3448	0,98	No
ENSMUSG00000079711	7,28E-01	-2,831991672	2,89E-01	5,91E-01	8,41E-01	Smok4a	1,66	No

ENSMUSG00000079722	8,37E-01	-2,268069782	8,42E-01	3,59E-01	6,60E-01	Ttll2	1,79	No
ENSMUSG00000079737	-4,43E-02	2,678703348	1,25E-01	7,24E-01	9,17E-01	3110001I22Rik	0,97	No
ENSMUSG00000079741	-1,94E+00	-3,107619243	1,50E+00	2,21E-01	5,50E-01	Nlrp4g	0,26	No
ENSMUSG00000079884	1,58E-02	7,092517549	6,83E-02	7,94E-01	9,56E-01	Gm10698	1,01	No
ENSMUSG00000079889	-1,20E-01	-0,079601181	1,35E-01	7,13E-01	9,11E-01	Gm11951	0,92	No
ENSMUSG00000079906	-1,15E-01	-0,088475151	1,36E-01	7,12E-01	9,11E-01	Gm15846	0,92	No
ENSMUSG00000079915	5,75E-01	-1,831975368	7,52E-01	3,86E-01	6,87E-01	Gm16073	1,49	No
ENSMUSG00000079941	-2,04E-02	6,738780571	1,10E-01	7,40E-01	9,27E-01	Gm11273	0,99	No
ENSMUSG00000079942	-2,09E-01	5,664797991	9,52E+00	2,03E-03	2,83E-02	Rpl28-ps3	0,87	Yes
ENSMUSG00000079962	-1,34E+00	-2,655110494	1,12E+00	2,89E-01	5,85E-01	Gm12643	0,40	No
ENSMUSG00000079988	4,42E-02	-1,600748872	6,15E-03	9,37E-01	1,00E+00	Gm11970	1,03	No
ENSMUSG00000079993	-3,83E-02	-3,107651936	-7,91E-11	1,00E+00	1,00E+00	Gm11799	0,97	No
ENSMUSG00000080002	1,69E-02	6,302891961	6,98E-02	7,92E-01	9,55E-01	Gm5566	1,01	No
ENSMUSG00000080006	-1,71E-01	1,306669622	8,66E-01	3,52E-01	6,53E-01	Rps19-ps7	0,89	No
ENSMUSG00000080021	-1,70E-01	4,957914981	5,20E+00	2,26E-02	1,64E-01	Gm5915	0,89	No
ENSMUSG00000080054	6,84E-01	-0,937911701	3,02E+00	8,21E-02	3,68E-01	Rpl12-ps1	1,61	No
ENSMUSG00000080058	2,23E-01	7,164249873	1,37E+01	2,16E-04	4,63E-03	Gm11175	1,17	Yes
ENSMUSG00000080059	-3,29E-01	2,917393937	8,32E+00	3,91E-03	4,66E-02	Rps19-ps3	0,80	Yes
ENSMUSG00000080076	-6,53E-02	-2,146110704	6,40E-03	9,36E-01	1,00E+00	Hist1h2aj	0,96	No
ENSMUSG00000080078	2,65E-01	3,213075506	6,53E+00	1,06E-02	9,75E-02	Gm15583	1,20	No
ENSMUSG00000080115	2,80E-01	-1,561146596	2,77E-01	5,99E-01	8,46E-01	Eef1akmt3	1,21	No
ENSMUSG00000080152	-8,54E-01	-2,831999582	4,00E-01	5,27E-01	8,03E-01	Gm12260	0,55	No
ENSMUSG00000080180	6,61E-01	-1,832100253	1,03E+00	3,10E-01	6,09E-01	Gm17235	1,58	No
ENSMUSG00000080198	2,20E-02	0,112167119	5,84E-03	9,39E-01	1,00E+00	Gm13573	1,02	No
ENSMUSG00000080205	-1,18E-01	2,822006388	9,95E-01	3,19E-01	6,19E-01	Gm15526	0,92	No
ENSMUSG00000080223	8,83E-02	1,628687638	2,62E-01	6,09E-01	8,51E-01	Gm13768	1,06	No
ENSMUSG00000080237	-3,84E-01	-0,946088666	8,40E-01	3,59E-01	6,61E-01	Gm14239	0,77	No
ENSMUSG00000080242	4,00E-02	8,925551366	4,91E-01	4,83E-01	7,71E-01	Gm15487	1,03	No
ENSMUSG00000080268	-4,40E-03	5,31909095	3,85E-03	9,51E-01	1,00E+00	Brms1	1,00	No
ENSMUSG00000080303	-2,55E-02	0,302627562	9,34E-03	9,23E-01	1,00E+00	Gm12419	0,98	No
ENSMUSG00000080316	-1,98E-01	1,304558418	1,19E+00	2,76E-01	5,73E-01	Spaca6	0,87	No
ENSMUSG00000080334	1,85E+00	-3,107657832	1,35E+00	2,46E-01	5,50E-01	Gm25110	3,61	No

ENSMUSG00000080342	-2,20E-01	-0,778162179	2,90E-01	5,90E-01	8,41E-01	Gm24589	0,86	No
ENSMUSG00000080350	1,24E+00	-2,770607092	9,48E-01	3,30E-01	6,31E-01	Gm26248	2,36	No
ENSMUSG00000080352	7,58E-02	2,331171211	3,03E-01	5,82E-01	8,38E-01	Gm26247	1,05	No
ENSMUSG00000080358	9,01E-03	-3,107646452	-5,16E-10	1,00E+00	1,00E+00	Gm26249	1,01	No
ENSMUSG00000080363	1,87E+00	-3,03364371	1,36E+00	2,43E-01	5,50E-01	Gm25778	3,66	No
ENSMUSG00000080364	-1,55E-01	-0,424868595	2,00E-01	6,54E-01	8,79E-01	Gm25777	0,90	No
ENSMUSG00000080365	-5,08E-01	-0,879762904	1,61E+00	2,05E-01	5,50E-01	Gm25776	0,70	No
ENSMUSG00000080366	-2,73E+00	-2,963241672	2,94E+00	8,62E-02	3,78E-01	Gm25775	0,15	No
ENSMUSG00000080370	7,63E-01	-2,289522746	1,04E+00	3,08E-01	6,06E-01	Gm22952	1,70	No
ENSMUSG00000080374	-4,30E-02	4,068839075	2,36E-01	6,27E-01	8,61E-01	Gm22953	0,97	No
ENSMUSG00000080384	-2,70E+00	-3,033614577	2,89E+00	8,93E-02	3,79E-01	Gm22105	0,15	No
ENSMUSG00000080396	-3,91E-01	-2,05171109	2,55E-01	6,13E-01	8,53E-01	Snord111	0,76	No
ENSMUSG00000080413	6,50E-01	-2,65511384	3,49E-01	5,54E-01	8,20E-01	Gm26473	1,57	No
ENSMUSG00000080440	1,07E+00	-2,355538449	1,47E+00	2,25E-01	5,50E-01	Gm25848	2,10	No
ENSMUSG00000080441	-1,88E-01	-2,126798423	5,12E-02	8,21E-01	9,72E-01	Mir1249	0,88	No
ENSMUSG00000080451	3,19E-01	-2,548267541	1,03E-01	7,48E-01	9,30E-01	Gm24193	1,25	No
ENSMUSG00000080452	2,50E-01	0,500078169	1,08E+00	2,99E-01	5,95E-01	Mir1306	1,19	No
ENSMUSG00000080454	-5,32E-01	-1,683101693	8,01E-01	3,71E-01	6,72E-01	Gm24195	0,69	No
ENSMUSG00000080455	-2,73E-02	-2,963256629	3,40E-04	9,85E-01	1,00E+00	Gm24194	0,98	No
ENSMUSG00000080461	-4,04E-01	-1,740753496	5,11E-01	4,75E-01	7,63E-01	Gm22488	0,76	No
ENSMUSG00000080463	-1,52E-01	-0,704653975	1,51E-01	6,97E-01	9,03E-01	Gm22489	0,90	No
ENSMUSG00000080465	2,59E-01	-1,946133753	1,47E-01	7,01E-01	9,05E-01	Gm22486	1,20	No
ENSMUSG00000080469	3,83E-01	-0,038524071	1,65E+00	1,99E-01	5,50E-01	Snord98	1,30	No
ENSMUSG00000080473	-7,15E-02	-2,401408398	7,07E-03	9,33E-01	1,00E+00	Gm25315	0,95	No
ENSMUSG00000080478	-4,16E-01	-2,146154324	2,89E-01	5,91E-01	8,41E-01	Snord23	0,75	No
ENSMUSG00000080485	1,33E-01	-2,146091021	3,08E-02	8,61E-01	9,96E-01	Gm23493	1,10	No
ENSMUSG00000080486	2,27E-01	0,443081022	8,16E-01	3,66E-01	6,67E-01	Snord100	1,17	No
ENSMUSG00000080506	-1,89E+00	-2,896152932	1,43E+00	2,31E-01	5,50E-01	Gm25042	0,27	No
ENSMUSG00000080516	-1,89E+00	-3,1076441	1,42E+00	2,33E-01	5,50E-01	Gm22197	0,27	No
ENSMUSG00000080517	1,81E+00	-3,10765737	1,28E+00	2,58E-01	5,50E-01	Gm23109	3,49	No
ENSMUSG00000080518	-1,17E+00	-2,963236728	5,92E-01	4,42E-01	7,34E-01	Gm22193	0,45	No
ENSMUSG00000080527	-1,24E-01	-0,19572978	1,50E-01	6,99E-01	9,04E-01	Gm23880	0,92	No

ENSMUSG00000080530	-2,51E-01	-1,246941928	2,89E-01	5,91E-01	8,41E-01	Gm25540	0,84	No
ENSMUSG00000080533	1,14E+00	-1,980544725	2,66E+00	1,03E-01	3,95E-01	Mir1191	2,21	No
ENSMUSG00000080538	-1,88E+00	-2,896153786	1,42E+00	2,33E-01	5,50E-01	Gm25541	0,27	No
ENSMUSG00000080540	1,22E+00	-3,146114919	6,90E-01	4,06E-01	7,02E-01	Gm22711	2,33	No
ENSMUSG00000080542	-1,37E+00	-2,83199071	1,18E+00	2,77E-01	5,74E-01	Gm22710	0,39	No
ENSMUSG00000080543	-3,88E-03	-3,03362684	4,02E-12	1,00E+00	1,00E+00	Gm22709	1,00	No
ENSMUSG00000080562	9,01E-03	-3,107646452	-5,16E-10	1,00E+00	1,00E+00	Gm26043	1,01	No
ENSMUSG00000080573	-1,90E+00	-2,963239763	1,44E+00	2,31E-01	5,50E-01	Mir467f	0,27	No
ENSMUSG00000080574	5,76E-01	-1,333386023	1,25E+00	2,64E-01	5,59E-01	Mir466i	1,49	No
ENSMUSG00000080594	-1,87E+00	-3,107637761	1,39E+00	2,38E-01	5,50E-01	Mir669h	0,27	No
ENSMUSG00000080597	-1,87E+00	-3,107637761	1,39E+00	2,38E-01	5,50E-01	Gm22033	0,27	No
ENSMUSG00000080609	-1,94E+00	-3,107619243	1,50E+00	2,21E-01	5,50E-01	Gm23844	0,26	No
ENSMUSG00000080610	9,54E-01	-2,051804567	1,74E+00	1,87E-01	5,50E-01	Snord110	1,94	No
ENSMUSG00000080613	1,02E+00	-1,963285657	2,04E+00	1,53E-01	4,96E-01	Gm22151	2,03	No
ENSMUSG00000080615	-1,19E-01	-1,322172196	5,55E-02	8,14E-01	9,68E-01	Snord99	0,92	No
ENSMUSG00000080626	1,03E+00	-1,963313661	1,71E+00	1,91E-01	5,50E-01	Mir1192	2,04	No
ENSMUSG00000080657	-1,87E+00	-3,107637761	1,39E+00	2,38E-01	5,50E-01	Mir669i	0,27	No
ENSMUSG00000080659	-5,39E-02	-2,832014942	1,32E-03	9,71E-01	1,00E+00	Gm24484	0,96	No
ENSMUSG00000080662	-9,32E-02	-2,600756511	5,67E-03	9,40E-01	1,00E+00	Mir1b	0,94	No
ENSMUSG00000080665	-5,87E-01	-2,655132158	2,70E-01	6,03E-01	8,48E-01	Mir1199	0,67	No
ENSMUSG00000080669	-2,49E-02	-3,146121042	-4,44E-11	1,00E+00	1,00E+00	Mir1224	0,98	No
ENSMUSG00000080678	1,87E+00	-3,107639691	1,38E+00	2,40E-01	5,50E-01	Gm23334	3,67	No
ENSMUSG00000080683	-4,89E-02	2,24236882	1,19E-01	7,31E-01	9,21E-01	Gm23991	0,97	No
ENSMUSG00000080700	-1,18E+00	-2,070127086	1,81E+00	1,79E-01	5,39E-01	Gm15981	0,44	No
ENSMUSG00000080701	-2,66E+00	-2,831960321	2,82E+00	9,31E-02	3,83E-01	Gm12340	0,16	No
ENSMUSG00000080703	-1,87E+00	-3,107637761	1,39E+00	2,38E-01	5,50E-01	Gm13183	0,27	No
ENSMUSG00000080708	1,01E-01	4,325676642	1,46E+00	2,27E-01	5,50E-01	Ccnd3-ps	1,07	No
ENSMUSG00000080709	1,87E+00	-3,107639691	1,38E+00	2,40E-01	5,50E-01	Gm14324	3,67	No
ENSMUSG00000080711	1,81E+00	-3,10765737	1,28E+00	2,58E-01	5,50E-01	Gm11686	3,49	No
ENSMUSG00000080712	6,04E-01	-2,133353639	8,40E-01	3,59E-01	6,61E-01	Hist3h2bb-ps	1,52	No
ENSMUSG00000080715	-3,14E-01	2,872949221	7,39E+00	6,57E-03	6,85E-02	Gm5406	0,80	No
ENSMUSG00000080716	6,13E-02	4,674718013	6,12E-01	4,34E-01	7,27E-01	Gm13503	1,04	No

ENSMUSG00000080717	-9,86E-03	1,025915267	2,39E-03	9,61E-01	1,00E+00	B230307C23Rik	0,99	No
ENSMUSG00000080719	1,63E+00	-2,770634521	1,83E+00	1,76E-01	5,36E-01	Gm15532	3,10	No
ENSMUSG00000080720	2,37E-01	-0,946050009	2,61E-01	6,10E-01	8,51E-01	Gm16077	1,18	No
ENSMUSG00000080723	-9,23E-02	-2,401414577	7,60E-03	9,31E-01	1,00E+00	Gm12564	0,94	No
ENSMUSG00000080726	-7,03E-02	-1,40135052	2,20E-02	8,82E-01	1,00E+00	Gm15366	0,95	No
ENSMUSG00000080727	4,39E-01	2,683541861	1,18E+01	5,79E-04	1,04E-02	C920021L13Rik	1,36	Yes
ENSMUSG00000080731	1,94E-01	-0,485253762	2,89E-01	5,91E-01	8,41E-01	Rps2-ps9	1,14	No
ENSMUSG00000080738	-5,14E-02	-2,770612208	2,33E-03	9,61E-01	1,00E+00	Mup-ps22	0,97	No
ENSMUSG00000080741	-5,94E-01	-1,770577837	1,01E+00	3,14E-01	6,14E-01	Gm11398	0,66	No
ENSMUSG00000080746	-2,42E-01	6,735910437	1,54E+01	8,88E-05	2,20E-03	Rpsa-ps12	0,85	Yes
ENSMUSG00000080747	1,24E-01	5,114335961	2,87E+00	9,03E-02	3,81E-01	Gm14016	1,09	No
ENSMUSG00000080753	-9,94E-01	-2,070175555	1,86E+00	1,73E-01	5,30E-01	Gm15613	0,50	No
ENSMUSG00000080759	1,25E-01	2,10597421	7,41E-01	3,89E-01	6,90E-01	Gm15573	1,09	No
ENSMUSG00000080763	3,77E-02	-1,098061634	6,90E-03	9,34E-01	1,00E+00	Gm12341	1,03	No
ENSMUSG00000080767	-3,83E-02	-3,107651936	-7,91E-11	1,00E+00	1,00E+00	Gm15595	0,97	No
ENSMUSG00000080768	1,49E-01	-0,548216627	1,63E-01	6,86E-01	8,97E-01	Gm12219	1,11	No
ENSMUSG00000080770	-1,89E+00	-3,1076441	1,42E+00	2,33E-01	5,50E-01	Gm11278	0,27	No
ENSMUSG00000080772	1,51E-01	5,658918044	4,93E+00	2,63E-02	1,82E-01	Gm12543	1,11	No
ENSMUSG00000080773	2,72E-01	-0,535359791	5,21E-01	4,70E-01	7,59E-01	Gm12955	1,21	No
ENSMUSG00000080776	-1,71E-01	8,437432951	8,88E+00	2,89E-03	3,72E-02	Gm12174	0,89	Yes
ENSMUSG00000080779	7,37E-01	-2,832043984	2,96E-01	5,87E-01	8,40E-01	Gm8731	1,67	No
ENSMUSG00000080782	-1,90E+00	-2,896159266	1,44E+00	2,30E-01	5,50E-01	Gm13495	0,27	No
ENSMUSG00000080783	-3,01E-01	1,8348139	3,78E+00	5,20E-02	2,78E-01	Gm8250	0,81	No
ENSMUSG00000080785	-8,07E-01	-2,831983513	3,58E-01	5,50E-01	8,17E-01	Gm11591	0,57	No
ENSMUSG00000080789	-1,87E+00	-3,107637761	1,39E+00	2,38E-01	5,50E-01	Gm14007	0,27	No
ENSMUSG00000080790	1,03E+00	-2,146108544	1,71E+00	1,92E-01	5,50E-01	Gm7638	2,04	No
ENSMUSG00000080791	-8,83E-02	2,426891137	4,41E-01	5,06E-01	7,91E-01	Akirin1-ps	0,94	No
ENSMUSG00000080793	-2,70E+00	-3,033614577	2,89E+00	8,93E-02	3,79E-01	Cbx3-ps3	0,15	No
ENSMUSG00000080797	1,21E+00	-0,641382841	9,47E+00	2,09E-03	2,90E-02	Gm15760	2,32	Yes
ENSMUSG00000080801	1,05E+00	-1,448754922	3,70E+00	5,44E-02	2,85E-01	Gm13310	2,07	No
ENSMUSG00000080806	-1,42E-01	-1,033698501	9,67E-02	7,56E-01	9,34E-01	Gm13005	0,91	No
ENSMUSG00000080808	-2,68E+00	-3,033645766	2,84E+00	9,17E-02	3,82E-01	Gm12608	0,16	No

ENSMUSG00000080810	-2,27E-01	-1,800990146	1,26E-01	7,23E-01	9,17E-01	Gm11737	0,85	No
ENSMUSG00000080814	1,65E+00	-2,770616865	1,87E+00	1,71E-01	5,27E-01	Gm12314	3,14	No
ENSMUSG00000080816	-5,29E-01	0,809809474	5,18E+00	2,28E-02	1,65E-01	Gm12447	0,69	No
ENSMUSG00000080823	-1,80E-02	-1,485288359	1,00E-03	9,75E-01	1,00E+00	Atp5l-ps2	0,99	No
ENSMUSG00000080824	-3,33E-02	2,455126869	6,28E-02	8,02E-01	9,61E-01	Gm9001	0,98	No
ENSMUSG00000080827	3,49E+00	-2,655168743	5,29E+00	2,15E-02	1,58E-01	Gm12086	11,23	No
ENSMUSG00000080831	-1,04E-01	-0,322090934	9,21E-02	7,62E-01	9,38E-01	Gm12529	0,93	No
ENSMUSG00000080832	-6,83E-02	6,595882495	1,20E+00	2,73E-01	5,69E-01	M6pr-ps	0,95	No
ENSMUSG00000080833	3,14E-02	-3,107617333	-7,69E-10	1,00E+00	1,00E+00	Gm11976	1,02	No
ENSMUSG00000080836	-1,54E-02	4,837005755	4,12E-02	8,39E-01	9,83E-01	Gm6444	0,99	No
ENSMUSG00000080839	-1,89E+00	-3,1076441	1,42E+00	2,33E-01	5,50E-01	Gm11625	0,27	No
ENSMUSG00000080840	-3,00E-01	0,779279475	1,78E+00	1,82E-01	5,45E-01	Gm11349	0,81	No
ENSMUSG00000080844	-2,85E-01	0,345096631	1,19E+00	2,76E-01	5,72E-01	Gm14107	0,82	No
ENSMUSG00000080846	-1,89E+00	-3,1076441	1,42E+00	2,33E-01	5,50E-01	Gm13379	0,27	No
ENSMUSG00000080848	-3,01E-01	7,1569591	2,50E+01	5,64E-07	2,66E-05	Gm9385	0,81	Yes
ENSMUSG00000080849	-5,30E-01	-2,185597691	4,51E-01	5,02E-01	7,87E-01	Gm15702	0,69	No
ENSMUSG00000080850	6,96E-01	-2,89613858	2,63E-01	6,08E-01	8,51E-01	Gm12439	1,62	No
ENSMUSG00000080851	8,29E-01	-2,497548213	5,99E-01	4,39E-01	7,32E-01	Gm12094	1,78	No
ENSMUSG00000080859	-2,32E-01	8,027219408	1,59E+01	6,74E-05	1,75E-03	Rpl10-ps1	0,85	Yes
ENSMUSG00000080866	-3,58E-01	-2,497568074	1,61E-01	6,88E-01	8,98E-01	Gm5687	0,78	No
ENSMUSG00000080868	-2,70E+00	-3,033614577	2,89E+00	8,93E-02	3,79E-01	Gm15745	0,15	No
ENSMUSG00000080869	2,64E-02	0,087033889	7,62E-03	9,30E-01	1,00E+00	Gm11856	1,02	No
ENSMUSG00000080870	-4,38E-01	-1,146123923	7,91E-01	3,74E-01	6,75E-01	Gm14111	0,74	No
ENSMUSG00000080873	-9,64E-02	1,493501498	2,92E-01	5,89E-01	8,41E-01	Rpl30-ps3	0,94	No
ENSMUSG00000080875	5,61E-02	5,57949577	6,63E-01	4,16E-01	7,11E-01	Gm7332	1,04	No
ENSMUSG00000080880	1,74E-01	-1,770609789	9,13E-02	7,63E-01	9,38E-01	Gm14278	1,13	No
ENSMUSG00000080885	-2,31E-01	7,654416527	1,54E+01	8,73E-05	2,17E-03	Rpl10-ps6	0,85	Yes
ENSMUSG00000080888	1,37E-01	-0,627576333	1,40E-01	7,08E-01	9,09E-01	Gm14387	1,10	No
ENSMUSG00000080892	-8,09E-02	1,006301136	1,60E-01	6,89E-01	8,99E-01	Gm9266	0,95	No
ENSMUSG00000080893	-1,18E-01	4,47344518	2,12E+00	1,46E-01	4,83E-01	Gm15920	0,92	No
ENSMUSG00000080902	2,06E-02	4,65294896	6,96E-02	7,92E-01	9,55E-01	Ywhaq-ps3	1,01	No
ENSMUSG00000080904	-2,15E-01	3,899816074	5,58E+00	1,81E-02	1,42E-01	Gm11966	0,86	No

ENSMUSG00000080906	-4,69E-02	-2,832012543	1,46E-03	9,70E-01	1,00E+00	Gm12095		0,97	No
ENSMUSG00000080911	-2,78E-01	1,180207515	1,88E+00	1,71E-01	5,27E-01	Gm11918		0,82	No
ENSMUSG00000080912	-1,89E+00	-3,1076441	1,42E+00	2,33E-01	5,50E-01	Gm12269		0,27	No
ENSMUSG00000080916	-1,26E+00	-2,655108316	1,53E+00	2,17E-01	5,50E-01	Gm12463		0,42	No
ENSMUSG00000080917	5,02E-03	4,113969741	3,36E-03	9,54E-01	1,00E+00	Gm15925		1,00	No
ENSMUSG00000080921	-1,70E-01	7,263545637	8,09E+00	4,45E-03	5,11E-02	Rpl38-ps2		0,89	No
ENSMUSG00000080928	-9,48E-02	1,100100467	2,19E-01	6,40E-01	8,70E-01	Hmgb1-ps6		0,94	No
ENSMUSG00000080929	2,98E-01	0,3588888	1,27E+00	2,60E-01	5,53E-01	Gm12891		1,23	No
ENSMUSG00000080932	-4,53E-01	1,077272279	5,03E+00	2,49E-02	1,74E-01	Gm10224		0,73	No
ENSMUSG00000080935	-1,19E-01	5,516695078	2,99E+00	8,40E-02	3,71E-01	Got2-ps1		0,92	No
ENSMUSG00000080936	7,20E-01	-2,963285673	2,81E-01	5,96E-01	8,44E-01	Olfr337-ps1		1,65	No
ENSMUSG00000080937	2,57E+00	-2,963293044	2,56E+00	1,10E-01	4,06E-01	Gm13510		5,93	No
ENSMUSG00000080946	-3,83E-02	-3,107651936	-7,91E-11	1,00E+00	1,00E+00	Gm12985		0,97	No
ENSMUSG00000080950	-7,16E-02	1,644668787	1,80E-01	6,72E-01	8,89E-01	Gm7278		0,95	No
ENSMUSG00000080951	3,44E-01	-1,070264375	5,06E-01	4,77E-01	7,65E-01	Gm12208		1,27	No
ENSMUSG00000080956	-7,72E-02	-2,311164352	6,80E-03	9,34E-01	1,00E+00	Gm16179		0,95	No
ENSMUSG00000080957	-1,46E+00	-2,107585051	3,82E+00	5,07E-02	2,73E-01	Gm15739		0,36	No
ENSMUSG00000080958	-5,12E-01	-1,997981944	4,21E-01	5,16E-01	7,97E-01	Gm11902		0,70	No
ENSMUSG00000080962	-1,38E+00	-2,896135155	1,20E+00	2,74E-01	5,71E-01	Gm11453		0,38	No
ENSMUSG00000080966	-5,94E-02	6,109465562	8,42E-01	3,59E-01	6,60E-01	Gm6263		0,96	No
ENSMUSG00000080969	-1,59E-01	1,986581581	1,14E+00	2,86E-01	5,82E-01	Gm12372		0,90	No
ENSMUSG00000080971	6,84E-01	-2,146160364	6,77E-01	4,11E-01	7,07E-01	Gm16128		1,61	No
ENSMUSG00000080972	-3,10E+00	-2,26796036	1,02E+01	1,44E-03	2,16E-02	Gm16061		0,12	Yes
ENSMUSG00000080977	-1,63E-01	2,874550286	1,92E+00	1,66E-01	5,19E-01	Gm13772		0,89	No
ENSMUSG00000080979	1,24E-01	2,875436528	1,11E+00	2,91E-01	5,87E-01	Gm13675		1,09	No
ENSMUSG00000080981	-3,05E-02	1,918485097	4,08E-02	8,40E-01	9,83E-01	Gm12161		0,98	No
ENSMUSG00000080982	-2,69E+00	-2,896097497	2,87E+00	9,05E-02	3,81E-01	Gm13549		0,16	No
ENSMUSG00000080989	-5,34E-02	4,747029948	4,81E-01	4,88E-01	7,75E-01	Gm14048		0,96	No
ENSMUSG00000080992	-3,27E-01	-2,088767788	2,29E-01	6,32E-01	8,64E-01	Psmb5-ps		0,80	No
ENSMUSG00000080993	-1,68E-01	-2,146117311	5,07E-02	8,22E-01	9,72E-01	Gm12903		0,89	No
ENSMUSG00000080994	7,33E-02	5,561422805	1,13E+00	2,88E-01	5,83E-01	Gm13464		1,05	No
ENSMUSG00000080999	-8,28E-02	-2,522726454	7,04E-03	9,33E-01	1,00E+00	Gm11336		0,94	No

ENSMUSG00000081000	3,14E-02	-3,107617333	-7,69E-10	1,00E+00	1,00E+00	Gm11200	1,02	No
ENSMUSG00000081002	-1,60E-01	2,522613212	1,56E+00	2,11E-01	5,50E-01	Gm14240	0,90	No
ENSMUSG00000081005	-1,15E+00	-1,895974225	2,60E+00	1,07E-01	4,00E-01	Gm13198	0,45	No
ENSMUSG00000081010	5,47E-02	3,687009671	3,29E-01	5,66E-01	8,28E-01	Gm13880	1,04	No
ENSMUSG00000081013	-1,03E+00	-1,378222344	3,07E+00	7,98E-02	3,61E-01	Gm14497	0,49	No
ENSMUSG00000081021	-1,52E-01	5,850358436	5,24E+00	2,21E-02	1,61E-01	Gm11964	0,90	No
ENSMUSG00000081023	-8,07E-01	-2,831972544	3,57E-01	5,50E-01	8,17E-01	Gm15766	0,57	No
ENSMUSG00000081025	-1,32E+00	-2,896135995	1,09E+00	2,97E-01	5,93E-01	Gm11943	0,40	No
ENSMUSG00000081026	1,85E+00	-2,963264462	1,34E+00	2,48E-01	5,50E-01	Gm11855	3,59	No
ENSMUSG00000081030	-8,31E-01	-2,770558106	3,79E-01	5,38E-01	8,09E-01	Gm12704	0,56	No
ENSMUSG00000081037	-1,44E+00	-1,378155708	7,64E+00	5,72E-03	6,18E-02	Gm13929	0,37	No
ENSMUSG00000081041	-2,53E-01	0,650158863	1,17E+00	2,80E-01	5,77E-01	Nlk-ps1	0,84	No
ENSMUSG00000081048	1,12E-01	-1,998031749	2,48E-02	8,75E-01	1,00E+00	Gm11760	1,08	No
ENSMUSG00000081051	-1,73E-01	8,912016879	9,26E+00	2,34E-03	3,18E-02	Gm15427	0,89	Yes
ENSMUSG00000081052	7,03E-02	3,478442584	4,89E-01	4,85E-01	7,72E-01	Gm6305	1,05	No
ENSMUSG00000081056	-7,08E-01	-2,03356355	8,52E-01	3,56E-01	6,57E-01	Gm13998	0,61	No
ENSMUSG00000081058	-1,02E-01	0,572117295	1,86E-01	6,66E-01	8,85E-01	Hist2h3c2	0,93	No
ENSMUSG00000081059	-1,80E-01	2,739725198	2,19E+00	1,39E-01	4,70E-01	Gm11945	0,88	No
ENSMUSG00000081067	3,17E-01	-2,49763878	1,02E-01	7,49E-01	9,31E-01	Gm12912	1,25	No
ENSMUSG00000081068	1,75E-01	3,047404422	2,47E+00	1,16E-01	4,21E-01	Gm12804	1,13	No
ENSMUSG00000081069	-6,80E-01	-1,863557687	1,05E+00	3,05E-01	6,01E-01	Gm13928	0,62	No
ENSMUSG00000081070	2,56E-02	4,72632967	1,10E-01	7,40E-01	9,26E-01	Gm13181	1,02	No
ENSMUSG00000081071	-8,37E-02	1,783734637	2,80E-01	5,97E-01	8,45E-01	Gm9836	0,94	No
ENSMUSG00000081076	-1,22E-01	4,667549182	2,47E+00	1,16E-01	4,21E-01	Rpsa-ps4	0,92	No
ENSMUSG00000081077	-5,89E-01	-2,600623495	2,72E-01	6,02E-01	8,48E-01	Gm13899	0,66	No
ENSMUSG00000081078	-7,81E-02	-1,146017805	2,71E-02	8,69E-01	1,00E+00	Gm12568	0,95	No
ENSMUSG00000081086	-5,90E-02	-2,146100716	5,64E-03	9,40E-01	1,00E+00	Gm9506	0,96	No
ENSMUSG00000081087	-2,06E-01	7,129432806	1,16E+01	6,49E-04	1,13E-02	Rps15a-ps7	0,87	Yes
ENSMUSG00000081093	1,71E-02	5,63775685	6,36E-02	8,01E-01	9,61E-01	Gm12565	1,01	No
ENSMUSG00000081094	-1,60E-01	9,13424246	7,94E+00	4,82E-03	5,45E-02	Rpl19-ps11	0,90	No
ENSMUSG00000081098	-2,51E+00	-2,600606073	3,83E+00	5,05E-02	2,72E-01	Gm12220	0,18	No
ENSMUSG00000081099	-1,89E+00	-3,1076441	1,42E+00	2,33E-01	5,50E-01	Gm5270	0,27	No

ENSMUSG00000081103	-3,93E-01	-1,355561643	5,20E-01	4,71E-01	7,59E-01	Rps12-ps26	0,76	No
ENSMUSG00000081104	6,93E-02	2,161248719	2,40E-01	6,24E-01	8,59E-01	Gm15989	1,05	No
ENSMUSG00000081110	1,82E+00	-2,832040072	1,32E+00	2,51E-01	5,50E-01	Gm12745	3,53	No
ENSMUSG00000081111	-2,41E-02	6,605151113	1,51E-01	6,98E-01	9,04E-01	Gm5913	0,98	No
ENSMUSG00000081113	-1,19E-01	5,50853686	2,95E+00	8,59E-02	3,77E-01	Gm7308	0,92	No
ENSMUSG00000081120	-3,83E-02	-3,107651936	-7,91E-11	1,00E+00	1,00E+00	Gm12458	0,97	No
ENSMUSG00000081121	-8,58E-02	5,980455371	1,71E+00	1,91E-01	5,50E-01	Gm12791	0,94	No
ENSMUSG00000081123	3,78E-01	-2,226338925	2,28E-01	6,33E-01	8,65E-01	Gm11469	1,30	No
ENSMUSG00000081126	7,56E-02	4,092027761	7,40E-01	3,90E-01	6,90E-01	Gm15784	1,05	No
ENSMUSG00000081127	-1,94E+00	-3,107619243	1,50E+00	2,21E-01	5,50E-01	Gm12677	0,26	No
ENSMUSG00000081128	-3,24E-02	3,620039347	1,13E-01	7,37E-01	9,25E-01	Gm13328	0,98	No
ENSMUSG00000081130	9,01E-03	-3,107646452	-5,16E-10	1,00E+00	1,00E+00	Gm8217	1,01	No
ENSMUSG00000081132	2,33E-01	-0,816417523	3,09E-01	5,78E-01	8,35E-01	Gm13744	1,18	No
ENSMUSG00000081136	-7,94E-01	-2,896102912	3,44E-01	5,57E-01	8,21E-01	Gm12990	0,58	No
ENSMUSG00000081138	-9,15E-02	3,889291263	1,02E+00	3,13E-01	6,13E-01	Gm12834	0,94	No
ENSMUSG00000081139	-8,37E-01	-2,401297198	1,02E+00	3,12E-01	6,12E-01	Gm13333	0,56	No
ENSMUSG00000081142	5,68E-01	-1,289571959	1,40E+00	2,38E-01	5,50E-01	Gm15497	1,48	No
ENSMUSG00000081143	-3,83E-02	-3,107651936	-7,91E-11	1,00E+00	1,00E+00	Gm15823	0,97	No
ENSMUSG00000081151	2,26E-02	2,702205863	3,40E-02	8,54E-01	9,92E-01	Gm11448	1,02	No
ENSMUSG00000081152	1,70E-01	3,287444214	2,67E+00	1,02E-01	3,94E-01	Gm12430	1,13	No
ENSMUSG00000081153	2,74E-02	-3,033621357	9,16E-12	1,00E+00	1,00E+00	Gm11401	1,02	No
ENSMUSG00000081156	-7,42E-02	-1,683112101	1,41E-02	9,05E-01	1,00E+00	Gm14425	0,95	No
ENSMUSG00000081157	2,24E-01	3,495780799	5,11E+00	2,38E-02	1,69E-01	Gm13552	1,17	No
ENSMUSG00000081161	-1,59E-02	-2,96323932	1,18E-04	9,91E-01	1,00E+00	Gm12783	0,99	No
ENSMUSG00000081165	-2,73E-01	-2,033624345	1,30E-01	7,19E-01	9,15E-01	Gm11783	0,83	No
ENSMUSG00000081169	-9,70E-01	-1,366768157	3,11E+00	7,80E-02	3,57E-01	Gm12551	0,51	No
ENSMUSG00000081170	2,03E-02	4,326854983	5,93E-02	8,08E-01	9,64E-01	Gm12922	1,01	No
ENSMUSG00000081176	3,80E-03	6,537453566	3,69E-03	9,52E-01	1,00E+00	H3f3a-ps2	1,00	No
ENSMUSG00000081179	1,02E-01	4,249875908	1,46E+00	2,27E-01	5,50E-01	Gm13136	1,07	No
ENSMUSG00000081185	-1,78E-01	2,238616053	1,59E+00	2,07E-01	5,50E-01	Gm4852	0,88	No
ENSMUSG00000081191	-3,83E-02	-3,107651936	-7,91E-11	1,00E+00	1,00E+00	Gm11271	0,97	No
ENSMUSG00000081201	-1,82E-01	4,061674986	4,27E+00	3,89E-02	2,34E-01	Smt3h2-ps4	0,88	No

ENSMUSG00000081206	-4,16E-01	-2,655082866	1,76E-01	6,75E-01	8,90E-01	Gm11935	0,75	No
ENSMUSG00000081208	5,78E-03	5,588442698	7,17E-03	9,33E-01	1,00E+00	Gm11400	1,00	No
ENSMUSG00000081210	-6,13E-01	0,471421352	6,17E+00	1,30E-02	1,13E-01	Gm12933	0,65	No
ENSMUSG00000081214	-1,73E-01	5,650610397	6,48E+00	1,09E-02	9,96E-02	Rpl35a-ps2	0,89	No
ENSMUSG00000081219	-1,42E-02	-0,66882976	1,20E-03	9,72E-01	1,00E+00	Bambi-ps1	0,99	No
ENSMUSG00000081223	4,86E-02	0,575185842	3,97E-02	8,42E-01	9,85E-01	Gm12247	1,03	No
ENSMUSG00000081226	2,38E-02	2,451638571	3,39E-02	8,54E-01	9,92E-01	Gm8802	1,02	No
ENSMUSG00000081227	-5,66E-02	4,067572602	4,14E-01	5,20E-01	7,99E-01	Gm13082	0,96	No
ENSMUSG00000081228	-1,36E-01	4,761177197	3,14E+00	7,63E-02	3,52E-01	Gm16089	0,91	No
ENSMUSG00000081236	-1,17E+00	-2,268045772	1,77E+00	1,83E-01	5,46E-01	Gm13574	0,44	No
ENSMUSG00000081237	9,01E-03	-3,107646452	-5,16E-10	1,00E+00	1,00E+00	Gm11385	1,01	No
ENSMUSG00000081238	-1,90E+00	-3,033647263	1,44E+00	2,30E-01	5,50E-01	Gm15836	0,27	No
ENSMUSG00000081239	-3,72E-01	4,939577618	2,48E+01	6,38E-07	2,96E-05	Gm11836	0,77	Yes
ENSMUSG00000081242	-3,32E-01	0,002055994	1,28E+00	2,59E-01	5,51E-01	Gm15719	0,79	No
ENSMUSG00000081244	2,34E-01	-2,205844357	9,76E-02	7,55E-01	9,34E-01	Gm13529	1,18	No
ENSMUSG00000081248	3,07E-01	0,019330989	1,02E+00	3,14E-01	6,14E-01	Gm13787	1,24	No
ENSMUSG00000081249	8,54E-02	-1,226397871	2,89E-02	8,65E-01	9,98E-01	Gm11517	1,06	No
ENSMUSG00000081251	4,05E-02	1,389267468	4,99E-02	8,23E-01	9,73E-01	Gm12164	1,03	No
ENSMUSG00000081252	-1,94E+00	-3,107619243	1,50E+00	2,21E-01	5,50E-01	Gm16032	0,26	No
ENSMUSG00000081254	-9,80E-02	3,696689469	1,05E+00	3,05E-01	6,02E-01	Gm12112	0,93	No
ENSMUSG00000081256	1,24E+00	-2,740822304	9,50E-01	3,30E-01	6,30E-01	Gm15791	2,36	No
ENSMUSG00000081257	1,64E-01	2,471521428	1,47E+00	2,26E-01	5,50E-01	Gm13607	1,12	No
ENSMUSG00000081258	-4,25E-02	3,233783823	1,59E-01	6,90E-01	8,99E-01	Gm14013	0,97	No
ENSMUSG00000081259	-3,44E-01	0,136442817	1,44E+00	2,30E-01	5,50E-01	Gm6893	0,79	No
ENSMUSG00000081261	-3,95E-02	-2,963274761	7,08E-04	9,79E-01	1,00E+00	Gm15370	0,97	No
ENSMUSG00000081262	-3,27E-02	1,630622608	3,79E-02	8,46E-01	9,87E-01	Gm12261	0,98	No
ENSMUSG00000081264	-3,88E-03	-3,03362684	4,02E-12	1,00E+00	1,00E+00	Gm14021	1,00	No
ENSMUSG00000081265	-1,94E+00	-3,107619243	1,50E+00	2,21E-01	5,50E-01	Gm11282	0,26	No
ENSMUSG00000081272	5,46E-02	8,678691227	9,10E-01	3,40E-01	6,41E-01	Gm13509	1,04	No
ENSMUSG00000081274	1,29E-01	-1,711788454	5,84E-02	8,09E-01	9,65E-01	Gm15727	1,09	No
ENSMUSG00000081277	-1,37E-02	4,854328599	3,25E-02	8,57E-01	9,94E-01	Gm15285	0,99	No
ENSMUSG00000081278	8,92E-02	2,425725584	4,35E-01	5,10E-01	7,93E-01	Gm13186	1,06	No

ENSMUSG00000081282	-1,99E+00	-2,655106177	2,99E+00	8,39E-02	3,71E-01	Gm13836	0,25	No
ENSMUSG00000081284	-7,26E-02	-2,770594557	2,22E-03	9,62E-01	1,00E+00	Gm13673	0,95	No
ENSMUSG00000081285	-6,56E-02	-2,896173716	2,94E-03	9,57E-01	1,00E+00	Gm18859	0,96	No
ENSMUSG00000081301	7,16E-01	-2,896139042	2,79E-01	5,97E-01	8,45E-01	Gm15951	1,64	No
ENSMUSG00000081302	-4,33E-02	4,258444753	2,65E-01	6,06E-01	8,50E-01	Gm12020	0,97	No
ENSMUSG00000081303	-5,79E-01	-1,711612007	1,12E+00	2,90E-01	5,86E-01	Gm16011	0,67	No
ENSMUSG00000081304	-1,94E+00	-3,107619243	1,50E+00	2,21E-01	5,50E-01	Gm11509	0,26	No
ENSMUSG00000081309	1,22E-01	3,63360975	1,62E+00	2,03E-01	5,50E-01	Gm12397	1,09	No
ENSMUSG00000081314	2,30E-02	1,066587083	1,34E-02	9,08E-01	1,00E+00	Gm14132	1,02	No
ENSMUSG00000081316	-6,56E-02	1,741923369	1,70E-01	6,80E-01	8,93E-01	Gm16205	0,96	No
ENSMUSG00000081317	1,57E+00	-2,448759673	2,70E+00	1,00E-01	3,92E-01	Gm12757	2,98	No
ENSMUSG00000081319	1,23E+00	-2,448710017	9,38E-01	3,33E-01	6,33E-01	Gm12936	2,35	No
ENSMUSG00000081321	1,34E+00	-2,49763913	1,83E+00	1,76E-01	5,35E-01	Gm15304	2,53	No
ENSMUSG00000081325	-1,37E-01	2,110125649	9,09E-01	3,40E-01	6,41E-01	Gm12599	0,91	No
ENSMUSG00000081329	1,11E+00	-1,574365018	3,58E+00	5,86E-02	2,99E-01	Gm12492	2,16	No
ENSMUSG00000081330	9,01E-03	-3,107646452	-5,16E-10	1,00E+00	1,00E+00	Gm13013	1,01	No
ENSMUSG00000081332	-4,47E-02	-2,770600874	9,54E-04	9,75E-01	1,00E+00	Gm12664	0,97	No
ENSMUSG00000081335	7,17E-01	-1,600812097	1,56E+00	2,12E-01	5,50E-01	Gm15786	1,64	No
ENSMUSG00000081339	3,95E-01	-1,627731966	4,67E-01	4,94E-01	7,80E-01	Gm16044	1,31	No
ENSMUSG00000081340	-1,20E+00	-2,185599013	1,87E+00	1,72E-01	5,28E-01	Gm12275	0,43	No
ENSMUSG00000081342	2,95E-01	1,526893763	2,78E+00	9,57E-02	3,86E-01	Gm11434	1,23	No
ENSMUSG00000081343	-2,46E-01	-2,107737186	1,19E-01	7,30E-01	9,21E-01	Gm16071	0,84	No
ENSMUSG00000081344	-1,37E-01	9,016910878	5,82E+00	1,58E-02	1,31E-01	Gm14303	0,91	No
ENSMUSG00000081352	2,81E-02	4,173917898	1,07E-01	7,44E-01	9,29E-01	Gm12389	1,02	No
ENSMUSG00000081353	-3,83E-02	-3,107651936	-7,91E-11	1,00E+00	1,00E+00	Gm13818	0,97	No
ENSMUSG00000081355	-8,39E-01	-2,863700663	3,86E-01	5,34E-01	8,07E-01	Gm15264	0,56	No
ENSMUSG00000081359	-2,12E-01	1,136775614	1,14E+00	2,85E-01	5,81E-01	Cox20-ps	0,86	No
ENSMUSG00000081369	6,76E-01	-0,641258691	3,14E+00	7,63E-02	3,52E-01	Gm11896	1,60	No
ENSMUSG00000081370	-4,60E-02	4,86526899	3,74E-01	5,41E-01	8,11E-01	Gm9105	0,97	No
ENSMUSG00000081372	-3,86E-01	-0,655037626	9,83E-01	3,21E-01	6,22E-01	Gm16171	0,77	No
ENSMUSG00000081376	1,25E+00	-2,770607553	9,70E-01	3,25E-01	6,25E-01	Gm11694	2,38	No
ENSMUSG00000081378	-1,29E-01	5,053129691	3,13E+00	7,71E-02	3,54E-01	Rps13-ps4	0,91	No

ENSMUSG00000081382	-1,88E-01	4,585003087	5,68E+00	1,71E-02	1,37E-01	Rpl18-ps1	0,88	No
ENSMUSG00000081389	-6,53E-02	-1,522563476	1,40E-02	9,06E-01	1,00E+00	Gm13368	0,96	No
ENSMUSG00000081394	-5,33E-02	-2,07015912	5,93E-03	9,39E-01	1,00E+00	Gm13215	0,96	No
ENSMUSG00000081400	-1,53E-01	7,949559192	6,94E+00	8,43E-03	8,24E-02	Gm13680	0,90	No
ENSMUSG00000081402	6,86E-02	3,901980629	5,76E-01	4,48E-01	7,38E-01	Gm15455	1,05	No
ENSMUSG00000081404	-5,61E-01	-0,205699395	2,84E+00	9,17E-02	3,82E-01	Gm15694	0,68	No
ENSMUSG00000081405	1,92E-01	2,931627176	2,84E+00	9,18E-02	3,82E-01	Gm13142	1,14	No
ENSMUSG00000081406	-1,23E-01	9,236723826	4,68E+00	3,06E-02	2,01E-01	Rps6-ps4	0,92	No
ENSMUSG00000081413	-2,49E-01	4,535390103	9,61E+00	1,93E-03	2,72E-02	Uqcrh-ps2	0,84	Yes
ENSMUSG00000081416	3,60E-01	-2,015772432	2,76E-01	6,00E-01	8,46E-01	Gm14383	1,28	No
ENSMUSG00000081429	-6,19E-02	-2,146135851	6,20E-03	9,37E-01	1,00E+00	Rps27-ps1	0,96	No
ENSMUSG00000081431	-8,75E-02	2,650331252	5,02E-01	4,78E-01	7,66E-01	Gm15483	0,94	No
ENSMUSG00000081433	-1,87E+00	-3,03364178	1,41E+00	2,36E-01	5,50E-01	Serpinb1-ps1	0,27	No
ENSMUSG00000081434	-9,78E-02	4,720857181	1,60E+00	2,05E-01	5,50E-01	Gm14165	0,93	No
ENSMUSG00000081436	8,32E-02	3,328176068	6,50E-01	4,20E-01	7,15E-01	Gm5912	1,06	No
ENSMUSG00000081437	3,14E-02	-3,107617333	-7,69E-10	1,00E+00	1,00E+00	Gm12402	1,02	No
ENSMUSG00000081440	4,24E-02	-3,033592245	-1,17E-09	1,00E+00	1,00E+00	Gm12464	1,03	No
ENSMUSG00000081441	4,36E-02	5,04407932	3,50E-01	5,54E-01	8,20E-01	Gm6161	1,03	No
ENSMUSG00000081445	-1,94E+00	-3,107619243	1,50E+00	2,21E-01	5,50E-01	Rpl23a-ps4	0,26	No
ENSMUSG00000081451	-6,76E-02	4,243279905	6,36E-01	4,25E-01	7,19E-01	Gm12734	0,95	No
ENSMUSG00000081453	-1,84E-01	6,643013487	8,86E+00	2,92E-03	3,76E-02	Gm6767	0,88	Yes
ENSMUSG00000081455	6,11E-02	3,399973476	3,54E-01	5,52E-01	8,18E-01	Hmgb1-ps3	1,04	No
ENSMUSG00000081457	-3,83E-02	-3,107651936	-7,91E-11	1,00E+00	1,00E+00	Gm13488	0,97	No
ENSMUSG00000081459	-4,70E-02	1,087923092	5,42E-02	8,16E-01	9,69E-01	Llph-ps1	0,97	No
ENSMUSG00000081460	2,51E-01	-1,510056414	2,44E-01	6,21E-01	8,57E-01	Gm13168	1,19	No
ENSMUSG00000081467	-7,82E-02	-2,268156672	5,47E-03	9,41E-01	1,00E+00	Gm13337	0,95	No
ENSMUSG00000081470	-2,82E-02	3,279483704	7,14E-02	7,89E-01	9,54E-01	Gm14131	0,98	No
ENSMUSG00000081476	3,63E-02	4,250742385	1,85E-01	6,67E-01	8,86E-01	Itpa-ps1	1,03	No
ENSMUSG00000081480	-8,76E-01	-2,070154848	1,38E+00	2,40E-01	5,50E-01	Gm13608	0,54	No
ENSMUSG00000081483	-2,02E-01	2,739937545	2,70E+00	1,00E-01	3,92E-01	Gm12445	0,87	No
ENSMUSG00000081485	-8,46E-03	6,429873147	1,80E-02	8,93E-01	1,00E+00	Gm12338	0,99	No
ENSMUSG00000081487	1,03E-01	0,32396207	1,63E-01	6,86E-01	8,97E-01	Gm13689	1,07	No

ENSMUSG00000081488	-1,67E+00	-2,107532392	4,25E+00	3,93E-02	2,35E-01	Gm15368		0,31	No
ENSMUSG00000081490	1,85E+00	-3,107657832	1,35E+00	2,46E-01	5,50E-01	Gm11830		3,61	No
ENSMUSG00000081491	-9,07E-01	-0,718897536	5,36E+00	2,07E-02	1,54E-01	Gm12778		0,53	No
ENSMUSG00000081492	-1,84E-01	0,644579392	6,14E-01	4,33E-01	7,27E-01	Gm9311		0,88	No
ENSMUSG00000081494	-2,29E-01	0,006500604	6,29E-01	4,28E-01	7,22E-01	Gm14130		0,85	No
ENSMUSG00000081496	-2,49E-02	-3,146121042	-4,44E-11	1,00E+00	1,00E+00	Gm13428		0,98	No
ENSMUSG00000081497	2,11E-01	-1,801095121	1,14E-01	7,36E-01	9,24E-01	Gm15560		1,16	No
ENSMUSG00000081499	-3,83E-02	-3,107651936	-7,91E-11	1,00E+00	1,00E+00	Gm12517		0,97	No
ENSMUSG00000081501	-1,12E+00	-2,929275006	5,52E-01	4,58E-01	7,47E-01	Gm13286		0,46	No
ENSMUSG00000081504	-1,86E+00	-3,033612668	1,39E+00	2,38E-01	5,50E-01	Gm12508		0,28	No
ENSMUSG00000081512	-6,18E-02	4,943938098	6,81E-01	4,09E-01	7,06E-01	Gm15821		0,96	No
ENSMUSG00000081515	-1,36E+00	-2,497587641	1,16E+00	2,82E-01	5,78E-01	Gm12311		0,39	No
ENSMUSG00000081516	-4,71E-01	0,814692094	4,51E+00	3,36E-02	2,16E-01	Gm12470		0,72	No
ENSMUSG00000081519	-1,99E-01	4,18494806	5,41E+00	2,01E-02	1,52E-01	Gm13331		0,87	No
ENSMUSG00000081520	-4,46E-01	0,921010286	4,78E+00	2,87E-02	1,93E-01	Gm16200		0,73	No
ENSMUSG00000081522	-6,07E-02	-2,77055561	2,50E-03	9,60E-01	1,00E+00	Gm13170		0,96	No
ENSMUSG00000081524	2,67E+00	-3,033636951	2,76E+00	9,68E-02	3,88E-01	Gm11658		6,36	No
ENSMUSG00000081528	-8,51E-02	-2,226248373	1,17E-02	9,14E-01	1,00E+00	Gm12924		0,94	No
ENSMUSG00000081534	1,22E-01	8,184007847	4,46E+00	3,47E-02	2,20E-01	Slc48a1		1,09	No
ENSMUSG00000081539	2,08E-01	-2,548213368	5,41E-02	8,16E-01	9,69E-01	Gm11990		1,16	No
ENSMUSG00000081540	-1,81E-01	0,906751211	7,14E-01	3,98E-01	6,98E-01	Gm12538		0,88	No
ENSMUSG00000081541	3,07E-01	-2,401439381	9,60E-02	7,57E-01	9,35E-01	Gm13940		1,24	No
ENSMUSG00000081544	-6,29E-02	2,916871698	2,93E-01	5,88E-01	8,40E-01	Gm13077		0,96	No
ENSMUSG00000081552	-3,89E-02	-2,831956393	7,45E-04	9,78E-01	1,00E+00	Gm12328		0,97	No
ENSMUSG00000081553	-2,23E-01	4,970045107	9,11E+00	2,54E-03	3,38E-02	Ccdc50-ps		0,86	Yes
ENSMUSG00000081557	-1,12E-01	1,095425416	3,30E-01	5,66E-01	8,28E-01	Gm5697		0,93	No
ENSMUSG00000081559	-1,35E-01	5,222447899	3,55E+00	5,97E-02	3,02E-01	Gm12411		0,91	No
ENSMUSG00000081562	3,46E-02	1,866414062	4,73E-02	8,28E-01	9,76E-01	Gm11575		1,02	No
ENSMUSG00000081564	3,99E+00	-2,711810741	7,71E+00	5,49E-03	5,99E-02	Gm13717		15,86	No
ENSMUSG00000081565	4,41E-01	-2,448678538	1,52E-01	6,97E-01	9,03E-01	Rps12-ps5		1,36	No
ENSMUSG00000081570	1,87E+00	-3,03364371	1,36E+00	2,43E-01	5,50E-01	Gm12192		3,66	No
ENSMUSG00000081572	-1,11E-01	-0,613980053	9,39E-02	7,59E-01	9,36E-01	Gm14138		0,93	No

ENSMUSG00000081574	-1,23E+00	-2,548150953	1,44E+00	2,29E-01	5,50E-01	Gm14429	0,43	No
ENSMUSG00000081575	-1,91E+00	-2,896158411	1,45E+00	2,28E-01	5,50E-01	Gm12090	0,27	No
ENSMUSG00000081578	1,58E-02	1,140693747	6,24E-03	9,37E-01	1,00E+00	Gm12611	1,01	No
ENSMUSG00000081580	1,81E+00	-3,10765737	1,28E+00	2,58E-01	5,50E-01	Gm14129	3,49	No
ENSMUSG00000081584	1,81E+00	-3,10765737	1,28E+00	2,58E-01	5,50E-01	Gm12716	3,49	No
ENSMUSG00000081585	-1,87E+00	-3,107637761	1,39E+00	2,38E-01	5,50E-01	Gm12926	0,27	No
ENSMUSG00000081593	-5,00E-02	1,532153523	8,42E-02	7,72E-01	9,43E-01	Gm11841	0,97	No
ENSMUSG00000081594	-2,65E+00	-2,963225595	2,80E+00	9,41E-02	3,84E-01	Gm15467	0,16	No
ENSMUSG00000081600	-1,01E-02	4,294093641	1,44E-02	9,04E-01	1,00E+00	Gm12286	0,99	No
ENSMUSG00000081602	-2,66E+00	-2,831967508	2,82E+00	9,32E-02	3,83E-01	Gm15369	0,16	No
ENSMUSG00000081604	-2,28E-01	7,354735748	1,46E+01	1,32E-04	3,06E-03	Gm11518	0,85	Yes
ENSMUSG00000081605	4,49E-01	-1,051873519	1,07E+00	3,02E-01	5,98E-01	Gm15953	1,37	No
ENSMUSG00000081608	6,19E-01	-2,226283105	4,28E-01	5,13E-01	7,95E-01	Gm16210	1,54	No
ENSMUSG00000081610	-8,03E-01	-0,929110328	3,66E+00	5,58E-02	2,90E-01	Gm13733	0,57	No
ENSMUSG00000081611	-7,52E-02	4,067850632	7,40E-01	3,90E-01	6,90E-01	Gm13651	0,95	No
ENSMUSG00000081613	-1,87E+00	-3,107637761	1,39E+00	2,38E-01	5,50E-01	Gm12109	0,27	No
ENSMUSG00000081619	-1,02E+00	-1,401052029	3,34E+00	6,78E-02	3,28E-01	Gm13351	0,49	No
ENSMUSG00000081627	-4,67E-02	-2,655093458	1,52E-03	9,69E-01	1,00E+00	Gm13158	0,97	No
ENSMUSG00000081629	-1,50E-01	4,179791003	3,08E+00	7,90E-02	3,60E-01	Eif3s6-ps1	0,90	No
ENSMUSG00000081631	-1,18E-01	2,376027648	7,79E-01	3,77E-01	6,78E-01	Gm12005	0,92	No
ENSMUSG00000081636	-1,90E+00	-3,033647263	1,44E+00	2,30E-01	5,50E-01	Gm15901	0,27	No
ENSMUSG00000081640	-1,73E-01	4,655974154	4,91E+00	2,67E-02	1,83E-01	Rplp0-ps1	0,89	No
ENSMUSG00000081642	-1,05E-01	0,769005597	2,23E-01	6,37E-01	8,68E-01	Gm13532	0,93	No
ENSMUSG00000081643	9,57E-02	6,961468479	2,47E+00	1,16E-01	4,22E-01	Gm11605	1,07	No
ENSMUSG00000081647	-7,45E-01	-2,448674062	6,21E-01	4,31E-01	7,24E-01	Gm13022	0,60	No
ENSMUSG00000081648	-4,61E-02	-2,497513346	1,51E-03	9,69E-01	1,00E+00	Gm13423	0,97	No
ENSMUSG00000081650	-8,98E-02	1,926424137	3,45E-01	5,57E-01	8,21E-01	Gm16181	0,94	No
ENSMUSG00000081651	5,49E-02	2,56597393	1,85E-01	6,67E-01	8,86E-01	Gm15530	1,04	No
ENSMUSG00000081656	-9,96E-02	3,590410525	1,03E+00	3,10E-01	6,09E-01	Gm11246	0,93	No
ENSMUSG00000081657	-3,16E+00	-2,832004344	4,21E+00	4,03E-02	2,38E-01	Gm15466	0,11	No
ENSMUSG00000081660	1,52E-01	3,793634007	2,63E+00	1,05E-01	3,97E-01	Gm11787	1,11	No
ENSMUSG00000081664	5,75E-02	1,771355505	1,27E-01	7,21E-01	9,16E-01	Gm15544	1,04	No

ENSMUSG00000081665	1,79E-01	6,162161644	7,67E+00	5,62E-03	6,09E-02	Gm15922	1,13	No
ENSMUSG00000081666	2,61E+00	-3,033672762	2,63E+00	1,05E-01	3,97E-01	Gm11677	6,10	No
ENSMUSG00000081668	-1,06E-01	-0,236798009	1,11E-01	7,39E-01	9,26E-01	Gm7869	0,93	No
ENSMUSG00000081669	-1,50E-01	4,432297494	3,32E+00	6,85E-02	3,29E-01	Npm3-ps1	0,90	No
ENSMUSG00000081670	1,89E-01	2,814890491	2,51E+00	1,13E-01	4,15E-01	Gm15697	1,14	No
ENSMUSG00000081671	4,24E-02	3,160642713	1,53E-01	6,96E-01	9,03E-01	Gm13167	1,03	No
ENSMUSG00000081672	-1,89E+00	-3,1076441	1,42E+00	2,33E-01	5,50E-01	Gm13000	0,27	No
ENSMUSG00000081675	-2,96E-01	0,616190099	1,50E+00	2,20E-01	5,50E-01	Gm12712	0,81	No
ENSMUSG00000081677	1,23E+00	-2,655089436	9,37E-01	3,33E-01	6,34E-01	Mdk-ps1	2,34	No
ENSMUSG00000081678	-3,83E-02	-3,107651936	-7,91E-11	1,00E+00	1,00E+00	Gm12959	0,97	No
ENSMUSG00000081679	-8,61E-03	-0,980549309	3,54E-04	9,85E-01	1,00E+00	Gm11874	0,99	No
ENSMUSG00000081683	7,56E-02	-1,740807706	1,26E-02	9,10E-01	1,00E+00	Fzd10	1,05	No
ENSMUSG00000081688	-2,71E+00	-3,033620915	2,92E+00	8,75E-02	3,78E-01	Gm12499	0,15	No
ENSMUSG00000081692	-1,89E+00	-2,896130167	1,43E+00	2,31E-01	5,50E-01	Gm15971	0,27	No
ENSMUSG00000081695	-3,31E-01	3,731152339	1,24E+01	4,33E-04	8,13E-03	Rpsa-ps5	0,80	Yes
ENSMUSG00000081700	-1,51E-01	5,329290786	4,57E+00	3,25E-02	2,10E-01	Atp5k-ps2	0,90	No
ENSMUSG00000081705	1,33E-01	2,21393462	8,31E-01	3,62E-01	6,63E-01	Gm12582	1,10	No
ENSMUSG00000081707	3,14E-02	-3,107617333	-7,69E-10	1,00E+00	1,00E+00	Gm11343	1,02	No
ENSMUSG00000081712	1,01E-01	3,708693722	1,11E+00	2,93E-01	5,88E-01	Gm12096	1,07	No
ENSMUSG00000081715	-4,12E-01	5,100404032	3,19E+01	1,66E-08	1,10E-06	Rps11-ps3	0,75	Yes
ENSMUSG00000081719	-1,87E+00	-3,107637761	1,39E+00	2,38E-01	5,50E-01	Gm13726	0,27	No
ENSMUSG00000081720	-4,87E-02	-2,711744789	1,62E-03	9,68E-01	1,00E+00	Gm12391	0,97	No
ENSMUSG00000081721	3,73E-01	1,535906002	4,33E+00	3,75E-02	2,30E-01	Gm13882	1,29	No
ENSMUSG00000081723	1,04E-01	5,57309836	2,31E+00	1,29E-01	4,50E-01	Gm15931	1,07	No
ENSMUSG00000081730	1,24E+00	-2,770618059	9,48E-01	3,30E-01	6,31E-01	Gm13785	2,36	No
ENSMUSG00000081731	7,57E-02	5,867552464	1,30E+00	2,54E-01	5,50E-01	Calr-ps	1,05	No
ENSMUSG00000081732	-2,62E-01	2,907826566	5,28E+00	2,16E-02	1,58E-01	Gm15495	0,83	No
ENSMUSG00000081733	1,22E+00	-2,711775297	9,22E-01	3,37E-01	6,38E-01	Gm12852	2,33	No
ENSMUSG00000081740	-2,27E-01	2,895161026	3,83E+00	5,02E-02	2,72E-01	Gm14279	0,85	No
ENSMUSG00000081744	4,89E-01	-2,448675987	3,24E-01	5,69E-01	8,30E-01	Gm5721	1,40	No
ENSMUSG00000081745	-2,47E-01	-1,448730725	2,42E-01	6,22E-01	8,58E-01	Gm15549	0,84	No
ENSMUSG00000081746	2,94E-01	-0,155939884	8,79E-01	3,48E-01	6,50E-01	Gm13925	1,23	No

ENSMUSG00000081747	1,15E+00	-2,655121981	1,26E+00	2,61E-01	5,55E-01	Rpl7a-ps9	2,22	No
ENSMUSG00000081749	-7,38E-02	-3,033659033	2,66E-03	9,59E-01	1,00E+00	Gm12241	0,95	No
ENSMUSG00000081751	-5,14E-01	-1,145970586	1,33E+00	2,49E-01	5,50E-01	Gm14251	0,70	No
ENSMUSG00000081756	-7,02E-01	-1,831839174	1,12E+00	2,89E-01	5,85E-01	Gm12291	0,61	No
ENSMUSG00000081757	1,64E+00	-2,832037269	1,85E+00	1,74E-01	5,31E-01	Gm13798	3,12	No
ENSMUSG00000081758	-3,94E-01	2,138887285	7,71E+00	5,48E-03	5,98E-02	Gm11460	0,76	No
ENSMUSG00000081766	3,14E-02	-3,107617333	-7,69E-10	1,00E+00	1,00E+00	2210409E12Rik	1,02	No
ENSMUSG00000081769	-5,51E-01	-0,711552147	2,16E+00	1,41E-01	4,75E-01	Gm12216	0,68	No
ENSMUSG00000081770	-1,87E+00	-3,107637761	1,39E+00	2,38E-01	5,50E-01	Rbpsuh-ps1	0,27	No
ENSMUSG00000081772	-1,09E-01	-0,321947642	1,03E-01	7,48E-01	9,30E-01	Gm13131	0,93	No
ENSMUSG00000081775	-7,25E-02	2,703924521	3,64E-01	5,46E-01	8,14E-01	Gm12420	0,95	No
ENSMUSG00000081777	9,01E-03	-3,107646452	-5,16E-10	1,00E+00	1,00E+00	Gm14159	1,01	No
ENSMUSG00000081783	-8,29E-01	-0,800782358	3,74E+00	5,32E-02	2,82E-01	Gm15599	0,56	No
ENSMUSG00000081787	-1,47E-02	5,326386762	4,35E-02	8,35E-01	9,80E-01	Gm13991	0,99	No
ENSMUSG00000081788	-1,87E-02	6,251195392	8,48E-02	7,71E-01	9,42E-01	Gm5898	0,99	No
ENSMUSG00000081789	5,12E-01	-1,401288547	9,60E-01	3,27E-01	6,28E-01	Gm11893	1,43	No
ENSMUSG00000081792	1,08E-01	6,246812299	2,84E+00	9,22E-02	3,82E-01	Anp32b-ps1	1,08	No
ENSMUSG00000081798	-1,13E+00	-2,801004119	7,50E-01	3,86E-01	6,87E-01	Gm8152	0,46	No
ENSMUSG00000081800	-3,16E-01	0,622273055	1,73E+00	1,88E-01	5,50E-01	Gm14200	0,80	No
ENSMUSG00000081801	4,93E-01	-2,355584019	3,30E-01	5,66E-01	8,28E-01	Dnmt3l-ps1	1,41	No
ENSMUSG00000081805	4,24E-03	2,260783285	8,81E-04	9,76E-01	1,00E+00	Gm14335	1,00	No
ENSMUSG00000081809	7,12E-01	-1,136244578	2,06E+00	1,51E-01	4,93E-01	Gm15539	1,64	No
ENSMUSG00000081810	-2,66E-01	5,018999555	1,30E+01	3,04E-04	6,12E-03	Gm11878	0,83	Yes
ENSMUSG00000081811	-2,04E+00	-2,711665771	3,16E+00	7,54E-02	3,49E-01	Gm12713	0,24	No
ENSMUSG00000081816	6,48E-02	2,068241988	1,99E-01	6,55E-01	8,79E-01	Gm15528	1,05	No
ENSMUSG00000081817	-5,08E-01	-0,641268071	2,02E+00	1,55E-01	4,99E-01	Gm13590	0,70	No
ENSMUSG00000081819	-4,72E-03	2,950628841	1,70E-03	9,67E-01	1,00E+00	Gm12722	1,00	No
ENSMUSG00000081824	-1,26E-01	4,925523923	2,80E+00	9,44E-02	3,85E-01	BC002163	0,92	No
ENSMUSG00000081829	6,84E-01	-1,54826742	1,61E+00	2,04E-01	5,50E-01	Gm15460	1,61	No
ENSMUSG00000081833	1,35E-01	3,282107135	1,65E+00	1,99E-01	5,50E-01	Gm13669	1,10	No
ENSMUSG00000081835	-1,13E-01	-2,963254935	5,86E-03	9,39E-01	1,00E+00	Gm11790	0,92	No
ENSMUSG00000081838	-6,43E-02	-2,548234065	4,73E-03	9,45E-01	1,00E+00	Gm13038	0,96	No

ENSMUSG00000081841	1,24E-01	2,621028853	9,92E-01	3,19E-01	6,20E-01	Gm13444	1,09	No
ENSMUSG00000081852	-2,88E-01	-2,311195575	1,44E-01	7,05E-01	9,07E-01	Gm12281	0,82	No
ENSMUSG00000081853	-3,42E-01	0,496342978	2,12E+00	1,45E-01	4,82E-01	Gm13735	0,79	No
ENSMUSG00000081854	1,84E+00	-2,896167046	1,33E+00	2,48E-01	5,50E-01	Gm11447	3,58	No
ENSMUSG00000081855	-2,51E-01	6,652774513	1,65E+01	4,99E-05	1,36E-03	Rpl17-ps5	0,84	Yes
ENSMUSG00000081857	-4,07E-02	0,74003525	3,24E-02	8,57E-01	9,94E-01	Gm13624	0,97	No
ENSMUSG00000081860	-9,57E-01	-2,548252635	8,02E-01	3,70E-01	6,72E-01	Gm11533	0,52	No
ENSMUSG00000081861	-8,19E-01	-2,401428704	9,78E-01	3,23E-01	6,23E-01	Gm14441	0,57	No
ENSMUSG00000081865	-2,46E-01	3,014292994	4,77E+00	2,89E-02	1,93E-01	Gm15484	0,84	No
ENSMUSG00000081866	1,87E+00	-3,03364371	1,36E+00	2,43E-01	5,50E-01	Gm12401	3,66	No
ENSMUSG00000081868	-1,64E-01	0,962049489	6,31E-01	4,27E-01	7,21E-01	Gm12039	0,89	No
ENSMUSG00000081871	-8,87E-02	3,966446488	9,65E-01	3,26E-01	6,27E-01	Gm11488	0,94	No
ENSMUSG00000081872	-4,45E-02	-2,655096141	1,78E-03	9,66E-01	1,00E+00	Gm15572	0,97	No
ENSMUSG00000081873	-2,71E+00	-2,963235336	2,92E+00	8,75E-02	3,78E-01	Gm15197	0,15	No
ENSMUSG00000081883	3,30E-01	-2,655148664	1,11E-01	7,39E-01	9,26E-01	Gm15574	1,26	No
ENSMUSG00000081884	9,21E-02	2,744973733	5,65E-01	4,52E-01	7,42E-01	Gm11821	1,07	No
ENSMUSG00000081885	1,23E-01	3,480167009	1,51E+00	2,19E-01	5,50E-01	Gm13231	1,09	No
ENSMUSG00000081887	1,33E-01	4,162969105	2,36E+00	1,24E-01	4,41E-01	Gm12097	1,10	No
ENSMUSG00000081888	7,64E-02	4,680626604	9,58E-01	3,28E-01	6,29E-01	Spcs2-ps	1,05	No
ENSMUSG00000081893	1,81E+00	-3,10765737	1,28E+00	2,58E-01	5,50E-01	Gm13273	3,49	No
ENSMUSG00000081894	-3,24E+00	-2,896104886	4,43E+00	3,53E-02	2,22E-01	Gm14212	0,11	No
ENSMUSG00000081895	-2,00E-01	7,491339979	1,14E+01	7,36E-04	1,26E-02	Rpl17-ps10	0,87	Yes
ENSMUSG00000081905	1,81E+00	-3,10765737	1,28E+00	2,58E-01	5,50E-01	Gm11370	3,49	No
ENSMUSG00000081906	5,90E-01	-0,529341969	3,03E+00	8,18E-02	3,67E-01	Rpl9-ps1	1,51	No
ENSMUSG00000081907	-1,53E-01	0,934377622	5,57E-01	4,56E-01	7,45E-01	Gm13731	0,90	No
ENSMUSG00000081911	1,29E-01	2,883243716	1,19E+00	2,75E-01	5,71E-01	Mrps36-ps2	1,09	No
ENSMUSG00000081920	1,09E-01	4,470425615	1,78E+00	1,83E-01	5,45E-01	Gm15359	1,08	No
ENSMUSG00000081921	-5,71E-01	-0,006699306	3,82E+00	5,06E-02	2,73E-01	Gm12858	0,67	No
ENSMUSG00000081924	1,21E-02	-3,033650469	7,34E-11	1,00E+00	1,00E+00	Gm11191	1,01	No
ENSMUSG00000081926	-2,66E-01	3,399266543	6,92E+00	8,53E-03	8,31E-02	Gm15536	0,83	No
ENSMUSG00000081929	-1,94E-01	5,243135794	7,41E+00	6,50E-03	6,80E-02	Rps11-ps2	0,87	No
ENSMUSG00000081932	-5,94E-01	-2,401365899	4,80E-01	4,88E-01	7,75E-01	Gm12331	0,66	No

ENSMUSG00000081944	-1,83E-01	0,655597711	5,84E-01	4,45E-01	7,36E-01	Gm12276	0,88	No
ENSMUSG00000081949	-2,36E+00	-2,997958949	2,18E+00	1,40E-01	4,72E-01	Gm14392	0,19	No
ENSMUSG00000081950	-1,89E+00	-3,1076441	1,42E+00	2,33E-01	5,50E-01	Gm11600	0,27	No
ENSMUSG00000081951	4,45E-01	-2,448660537	1,55E-01	6,94E-01	9,02E-01	Gm15352	1,36	No
ENSMUSG00000081952	1,93E-01	2,171584797	1,79E+00	1,80E-01	5,42E-01	Gm12178	1,14	No
ENSMUSG00000081953	1,39E-02	6,067174727	4,54E-02	8,31E-01	9,78E-01	Gm12435	1,01	No
ENSMUSG00000081954	-1,92E+00	-3,0336536	1,46E+00	2,27E-01	5,50E-01	Gm13626	0,26	No
ENSMUSG00000081957	2,79E-01	1,44148554	2,53E+00	1,11E-01	4,10E-01	Ak3l2-ps	1,21	No
ENSMUSG00000081958	-1,52E-02	-2,831963104	1,73E-04	9,90E-01	1,00E+00	Gm11594	0,99	No
ENSMUSG00000081965	1,44E-01	-1,26807879	6,72E-02	7,95E-01	9,57E-01	Gm11620	1,10	No
ENSMUSG00000081967	6,45E-02	4,04648858	5,36E-01	4,64E-01	7,53E-01	Gm14017	1,05	No
ENSMUSG00000081968	3,21E-01	-2,548244327	1,05E-01	7,46E-01	9,29E-01	Rpl23a-ps2	1,25	No
ENSMUSG00000081971	3,14E-02	-3,107617333	-7,69E-10	1,00E+00	1,00E+00	Gm14115	1,02	No
ENSMUSG00000081974	-9,55E-02	3,183829596	7,95E-01	3,73E-01	6,74E-01	Gm11960	0,94	No
ENSMUSG00000081975	-3,37E-01	5,070129347	2,10E+01	4,58E-06	1,67E-04	Gm12482	0,79	Yes
ENSMUSG00000081984	-3,51E-01	-0,510074433	1,01E+00	3,15E-01	6,16E-01	Dnajb3	0,78	No
ENSMUSG00000081985	1,02E-01	2,161910408	4,85E-01	4,86E-01	7,73E-01	Gng2-ps1	1,07	No
ENSMUSG00000081988	-1,94E+00	-3,107619243	1,50E+00	2,21E-01	5,50E-01	Gm15746	0,26	No
ENSMUSG00000081991	1,85E+00	-3,107657832	1,35E+00	2,46E-01	5,50E-01	Gm13859	3,61	No
ENSMUSG00000081992	-6,45E-02	6,31531522	1,03E+00	3,11E-01	6,11E-01	Gm13408	0,96	No
ENSMUSG00000081998	-3,17E-01	1,396517304	3,31E+00	6,89E-02	3,31E-01	Gm13245	0,80	No
ENSMUSG00000081999	6,80E-02	7,40453745	1,31E+00	2,53E-01	5,50E-01	Gm13461	1,05	No
ENSMUSG00000082001	-3,76E-01	-1,641338191	4,18E-01	5,18E-01	7,98E-01	Gm15607	0,77	No
ENSMUSG00000082006	7,13E-01	-2,831983747	2,77E-01	5,99E-01	8,46E-01	Gm13649	1,64	No
ENSMUSG00000082008	-1,98E-02	-3,033655952	2,83E-11	1,00E+00	1,00E+00	Gm12390	0,99	No
ENSMUSG00000082012	1,87E+00	-3,107639691	1,38E+00	2,40E-01	5,50E-01	Gm5257	3,67	No
ENSMUSG00000082013	-8,89E-03	0,010795695	9,44E-04	9,75E-01	1,00E+00	Gm13216	0,99	No
ENSMUSG00000082016	2,27E-01	8,091181656	1,52E+01	9,81E-05	2,39E-03	Pgam1-ps2	1,17	Yes
ENSMUSG00000082023	-1,92E+00	-3,0336536	1,46E+00	2,27E-01	5,50E-01	Gm14407	0,26	No
ENSMUSG00000082024	7,31E-01	-2,10771273	9,53E-01	3,29E-01	6,30E-01	Gm11794	1,66	No
ENSMUSG00000082028	2,45E-01	5,262070181	1,17E+01	6,21E-04	1,10E-02	Gm1826	1,19	Yes
ENSMUSG00000082029	-3,08E-02	6,611180043	2,45E-01	6,20E-01	8,57E-01	H3f3c	0,98	No

ENSMUSG00000082032	1,84E+00	-3,033649193	1,33E+00	2,49E-01	5,50E-01	Gm12271	3,58	No
ENSMUSG00000082036	-5,33E-01	-2,8320035	2,20E-01	6,39E-01	8,69E-01	Gm13724	0,69	No
ENSMUSG00000082040	8,69E-01	-2,401362084	9,09E-01	3,40E-01	6,41E-01	Gm12046	1,83	No
ENSMUSG00000082043	6,55E-02	1,539307926	1,45E-01	7,03E-01	9,06E-01	Gm12848	1,05	No
ENSMUSG00000082044	-1,81E-01	5,248542438	6,39E+00	1,15E-02	1,03E-01	Snrpert	0,88	No
ENSMUSG00000082045	-1,01E-01	3,472963241	1,00E+00	3,16E-01	6,17E-01	Gm11585	0,93	No
ENSMUSG00000082047	-1,89E+00	-3,1076441	1,42E+00	2,33E-01	5,50E-01	Gm12948	0,27	No
ENSMUSG00000082049	2,61E+00	-3,033672762	2,63E+00	1,05E-01	3,97E-01	Gm12105	6,10	No
ENSMUSG00000082051	-5,32E-02	2,790989779	1,97E-01	6,57E-01	8,80E-01	Gm16072	0,96	No
ENSMUSG00000082052	-7,13E-01	-1,332995133	1,92E+00	1,66E-01	5,18E-01	Ywhaq-ps2	0,61	No
ENSMUSG00000082057	2,01E-02	-1,522813944	1,45E-03	9,70E-01	1,00E+00	Gm15789	1,01	No
ENSMUSG00000082059	1,87E+00	-3,107639691	1,38E+00	2,40E-01	5,50E-01	Gm15183	3,67	No
ENSMUSG00000082062	9,96E-02	10,0556669	3,13E+00	7,68E-02	3,53E-01	Ftl2-ps	1,07	No
ENSMUSG00000082063	-1,87E+00	-3,107637761	1,39E+00	2,38E-01	5,50E-01	Gm12993	0,27	No
ENSMUSG00000082064	-2,25E-01	5,245787333	9,97E+00	1,59E-03	2,34E-02	Rpl5-ps2	0,86	Yes
ENSMUSG00000082066	1,44E-01	4,509727391	3,19E+00	7,39E-02	3,45E-01	Gm13904	1,10	No
ENSMUSG00000082072	-1,47E-01	-0,389904618	1,91E-01	6,62E-01	8,83E-01	Gm15785	0,90	No
ENSMUSG00000082079	-8,77E-01	-2,770573317	4,21E-01	5,17E-01	7,97E-01	Dnmt3c	0,54	No
ENSMUSG00000082080	7,98E-01	-0,548429501	4,96E+00	2,60E-02	1,80E-01	Gm9246	1,74	No
ENSMUSG00000082082	-9,99E-01	-2,601235609	9,98E-01	3,18E-01	6,19E-01	Gm13230	0,50	No
ENSMUSG00000082087	9,59E-02	4,595142107	1,45E+00	2,28E-01	5,50E-01	Gm12138	1,07	No
ENSMUSG00000082088	-8,07E-02	4,681327766	1,08E+00	2,99E-01	5,95E-01	Gm15753	0,95	No
ENSMUSG00000082093	1,25E+00	-1,896214138	3,28E+00	7,00E-02	3,34E-01	Gm15928	2,38	No
ENSMUSG00000082095	3,48E-02	2,555775957	7,59E-02	7,83E-01	9,50E-01	Gm11991	1,02	No
ENSMUSG00000082096	-1,73E-01	-0,668912138	2,16E-01	6,42E-01	8,71E-01	Gm13505	0,89	No
ENSMUSG00000082097	1,87E+00	-3,107639691	1,38E+00	2,40E-01	5,50E-01	Gm14039	3,67	No
ENSMUSG00000082099	-3,91E-01	4,114287683	1,97E+01	9,20E-06	3,12E-04	Gm12013	0,76	Yes
ENSMUSG00000082100	-2,18E-02	3,869541734	5,75E-02	8,10E-01	9,66E-01	Glns-ps1	0,99	No
ENSMUSG00000082101	-2,31E+00	-1,963176574	7,20E+00	7,27E-03	7,38E-02	Slfn14	0,20	No
ENSMUSG00000082103	-4,37E-01	-2,497554436	1,95E-01	6,59E-01	8,81E-01	Gm15320	0,74	No
ENSMUSG00000082107	7,46E-02	2,64839398	3,51E-01	5,53E-01	8,19E-01	Gm16216	1,05	No
ENSMUSG00000082108	4,09E-01	-2,77063723	1,29E-01	7,19E-01	9,15E-01	Gm13777	1,33	No

ENSMUSG00000082110	-4,23E-01	0,330924894	2,72E+00	9,91E-02	3,92E-01	Gm11196	0,75	No
ENSMUSG00000082111	4,11E-01	-1,600645529	5,05E-01	4,77E-01	7,65E-01	Gm12479	1,33	No
ENSMUSG00000082113	-1,76E+00	-2,600615253	1,55E+00	2,13E-01	5,50E-01	Gm13915	0,29	No
ENSMUSG00000082116	-5,61E-01	-2,268093892	4,66E-01	4,95E-01	7,80E-01	Gm12188	0,68	No
ENSMUSG00000082118	-3,83E-02	-3,107651936	-7,91E-11	1,00E+00	1,00E+00	Gm13862	0,97	No
ENSMUSG00000082120	-1,09E-01	4,224405633	1,69E+00	1,94E-01	5,50E-01	Gm15720	0,93	No
ENSMUSG00000082122	-4,26E-02	0,191245485	2,52E-02	8,74E-01	1,00E+00	Gm11809	0,97	No
ENSMUSG00000082123	-8,70E-01	-2,896167824	4,16E-01	5,19E-01	7,98E-01	Gm16149	0,55	No
ENSMUSG00000082127	-2,00E-01	2,456639406	2,30E+00	1,30E-01	4,52E-01	Gm13577	0,87	No
ENSMUSG00000082128	-1,79E-01	4,280096901	4,65E+00	3,10E-02	2,03E-01	Gm11824	0,88	No
ENSMUSG00000082130	7,44E-01	-2,963286135	3,02E-01	5,83E-01	8,38E-01	Gm15799	1,67	No
ENSMUSG00000082140	4,25E-01	-2,832034501	1,40E-01	7,08E-01	9,09E-01	Gm13094	1,34	No
ENSMUSG00000082141	8,20E-02	0,916023761	1,52E-01	6,97E-01	9,03E-01	Gm11212	1,06	No
ENSMUSG00000082144	4,33E-01	-2,401393067	1,47E-01	7,02E-01	9,05E-01	Gm12788	1,35	No
ENSMUSG00000082145	2,28E-02	3,767095434	6,04E-02	8,06E-01	9,63E-01	Gm12312	1,02	No
ENSMUSG00000082148	7,13E-01	-2,60070017	2,78E-01	5,98E-01	8,45E-01	Gm12266	1,64	No
ENSMUSG00000082151	-1,59E-02	-2,96323932	1,18E-04	9,91E-01	1,00E+00	Gm11810	0,99	No
ENSMUSG00000082154	1,41E-01	3,120022496	1,63E+00	2,02E-01	5,50E-01	Gm16464	1,10	No
ENSMUSG00000082160	-3,83E-02	-3,107651936	-7,91E-11	1,00E+00	1,00E+00	Gm11578	0,97	No
ENSMUSG00000082163	6,95E-02	1,464248599	1,51E-01	6,98E-01	9,04E-01	Gm14276	1,05	No
ENSMUSG00000082164	1,62E-01	0,425529087	4,14E-01	5,20E-01	7,99E-01	Gm13360	1,12	No
ENSMUSG00000082171	-8,51E-01	-1,378269629	2,86E+00	9,06E-02	3,82E-01	Rpl27-ps2	0,55	No
ENSMUSG00000082176	1,81E-01	-0,824274002	1,99E-01	6,55E-01	8,79E-01	Gm13163	1,13	No
ENSMUSG00000082179	-2,47E-01	-1,401275143	2,42E-01	6,23E-01	8,58E-01	Gm11407	0,84	No
ENSMUSG00000082180	-2,42E-01	-1,831863817	1,16E-01	7,34E-01	9,23E-01	Gm12230	0,85	No
ENSMUSG00000082183	1,45E-01	-1,600782379	6,88E-02	7,93E-01	9,56E-01	Gm15994	1,11	No
ENSMUSG00000082185	6,19E-02	5,010720835	7,03E-01	4,02E-01	7,00E-01	Gm12428	1,04	No
ENSMUSG00000082186	3,29E-01	-1,627785329	2,87E-01	5,92E-01	8,42E-01	Gm12403	1,26	No
ENSMUSG00000082187	-8,70E-01	-2,963240767	4,18E-01	5,18E-01	7,98E-01	Gm13670	0,55	No
ENSMUSG00000082190	2,64E+00	-2,71168463	2,69E+00	1,01E-01	3,92E-01	Gm13641	6,25	No
ENSMUSG00000082193	-1,75E-01	6,850540749	8,21E+00	4,17E-03	4,88E-02	Rpl5-ps1	0,89	Yes
ENSMUSG00000082194	1,71E-02	3,814022078	3,39E-02	8,54E-01	9,92E-01	Gm12444	1,01	No

ENSMUSG00000082195	1,41E-01	4,319692763	2,89E+00	8,92E-02	3,79E-01	Gm13034	1,10	No
ENSMUSG00000082196	-2,63E-01	-2,107687626	1,37E-01	7,12E-01	9,11E-01	Gm14231	0,83	No
ENSMUSG00000082200	-1,08E-01	-2,896148415	8,40E-03	9,27E-01	1,00E+00	Gm12871	0,93	No
ENSMUSG00000082203	2,32E-01	0,470902957	8,14E-01	3,67E-01	6,68E-01	Gm15636	1,17	No
ENSMUSG00000082210	1,87E+00	-3,107639691	1,38E+00	2,40E-01	5,50E-01	Gm14382	3,67	No
ENSMUSG00000082213	7,53E-01	-2,770600824	3,08E-01	5,79E-01	8,36E-01	Gm12102	1,69	No
ENSMUSG00000082217	4,04E-01	-0,165850969	1,64E+00	2,00E-01	5,50E-01	Gm1401	1,32	No
ENSMUSG00000082218	-1,86E-01	-0,006957765	3,94E-01	5,30E-01	8,05E-01	Gm13602	0,88	No
ENSMUSG00000082220	2,57E+00	-3,0336723	2,56E+00	1,10E-01	4,06E-01	Gm12943	5,96	No
ENSMUSG00000082233	-3,88E-03	-3,03362684	4,02E-12	1,00E+00	1,00E+00	Gm6506	1,00	No
ENSMUSG00000082234	1,81E+00	-3,10765737	1,28E+00	2,58E-01	5,50E-01	Gm16221	3,49	No
ENSMUSG00000082241	-3,70E-02	5,15455007	2,62E-01	6,09E-01	8,51E-01	Gng5-ps	0,97	No
ENSMUSG00000082243	6,55E-01	0,295258398	6,02E+00	1,41E-02	1,21E-01	Gm3810	1,57	No
ENSMUSG00000082247	-1,84E-01	1,66404958	1,22E+00	2,70E-01	5,66E-01	Gm16074	0,88	No
ENSMUSG00000082248	-2,73E-01	-0,024296331	8,43E-01	3,59E-01	6,60E-01	Gm13161	0,83	No
ENSMUSG00000082250	3,05E-01	-0,880104182	5,67E-01	4,51E-01	7,41E-01	Gm12181	1,24	No
ENSMUSG00000082252	7,52E-02	-0,015729429	6,45E-02	7,99E-01	9,60E-01	Gm11204	1,05	No
ENSMUSG00000082258	-1,04E-02	3,708341732	1,20E-02	9,13E-01	1,00E+00	Gm15289	0,99	No
ENSMUSG00000082264	1,84E-01	4,258951907	4,77E+00	2,89E-02	1,93E-01	Gm12799	1,14	No
ENSMUSG00000082265	-1,32E-01	-0,401293899	1,16E-01	7,33E-01	9,23E-01	Gm15547	0,91	No
ENSMUSG00000082266	-1,98E-02	-3,033655952	2,83E-11	1,00E+00	1,00E+00	Gm12303	0,99	No
ENSMUSG00000082272	-3,49E-02	4,703711961	2,01E-01	6,54E-01	8,78E-01	Gm11675	0,98	No
ENSMUSG00000082274	8,34E-02	5,575857493	1,48E+00	2,24E-01	5,50E-01	Gm14026	1,06	No
ENSMUSG00000082279	1,40E-01	3,812494142	2,24E+00	1,34E-01	4,61E-01	Gm12870	1,10	No
ENSMUSG00000082280	-8,23E-01	-2,6550782	3,73E-01	5,41E-01	8,11E-01	Gm5847	0,57	No
ENSMUSG00000082283	1,04E+00	-2,448755946	1,39E+00	2,39E-01	5,50E-01	Gm14472	2,06	No
ENSMUSG00000082286	-1,08E-01	4,630028035	1,91E+00	1,67E-01	5,19E-01	Pisd-ps1	0,93	No
ENSMUSG00000082287	-3,85E+00	-2,655111218	7,09E+00	7,75E-03	7,71E-02	Gm15333	0,07	No
ENSMUSG00000082289	-2,14E-01	1,916408111	1,98E+00	1,60E-01	5,07E-01	Gm15596	0,86	No
ENSMUSG00000082292	-8,33E-02	2,607198844	4,65E-01	4,95E-01	7,81E-01	Gm12250	0,94	No
ENSMUSG00000082296	-2,71E+00	-3,033620915	2,92E+00	8,75E-02	3,78E-01	Gm11450	0,15	No
ENSMUSG00000082304	-1,92E+00	-2,963214917	1,46E+00	2,27E-01	5,50E-01	Gm11198	0,26	No

ENSMUSG00000082305	7,74E-01	-0,832177706	3,88E+00	4,89E-02	2,66E-01	Gm2420	1,71	No
ENSMUSG00000082307	2,74E-02	-3,033621357	9,16E-12	1,00E+00	1,00E+00	Gm13418	1,02	No
ENSMUSG00000082308	8,70E-02	3,991839294	9,50E-01	3,30E-01	6,30E-01	Gm15770	1,06	No
ENSMUSG00000082309	-1,89E+00	-3,1076441	1,42E+00	2,33E-01	5,50E-01	Gm12123	0,27	No
ENSMUSG00000082310	-3,42E-02	1,804502826	4,51E-02	8,32E-01	9,78E-01	Gm12904	0,98	No
ENSMUSG00000082313	-1,89E+00	-3,033648118	1,42E+00	2,33E-01	5,50E-01	Gm11701	0,27	No
ENSMUSG00000082315	-1,88E+00	-2,448711663	4,15E+00	4,16E-02	2,42E-01	Gm16523	0,27	No
ENSMUSG00000082318	-9,13E-01	-2,655126213	7,28E-01	3,93E-01	6,94E-01	Gm14162	0,53	No
ENSMUSG00000082320	1,81E+00	-3,10765737	1,28E+00	2,58E-01	5,50E-01	Gm11877	3,49	No
ENSMUSG00000082321	-8,75E-02	5,433068033	1,56E+00	2,11E-01	5,50E-01	Gm14253	0,94	No
ENSMUSG00000082323	2,21E+00	-2,311212661	6,50E+00	1,08E-02	9,87E-02	Gm15314	4,63	No
ENSMUSG00000082328	7,19E-02	-1,088826312	2,54E-02	8,73E-01	1,00E+00	Gm13921	1,05	No
ENSMUSG00000082329	1,23E-01	3,905942064	1,82E+00	1,77E-01	5,37E-01	Gm14287	1,09	No
ENSMUSG00000082330	-5,88E-02	-3,033616507	1,62E-03	9,68E-01	1,00E+00	Ldha-ps	0,96	No
ENSMUSG00000082332	7,65E-02	0,977708415	1,22E-01	7,27E-01	9,19E-01	Gm13384	1,05	No
ENSMUSG00000082334	-2,37E-01	2,187271564	2,68E+00	1,02E-01	3,94E-01	Gm13244	0,85	No
ENSMUSG00000082343	-1,89E+00	-3,1076441	1,42E+00	2,33E-01	5,50E-01	Gm14404	0,27	No
ENSMUSG00000082345	3,66E-01	0,095791135	1,36E+00	2,43E-01	5,50E-01	Gm13622	1,29	No
ENSMUSG00000082348	-1,96E-01	2,302592623	2,06E+00	1,51E-01	4,93E-01	Gm12807	0,87	No
ENSMUSG00000082357	7,09E-01	-1,896168182	1,36E+00	2,44E-01	5,50E-01	Gm13542	1,63	No
ENSMUSG00000082361	-5,21E-01	-2,146091118	4,35E-01	5,10E-01	7,93E-01	Btc	0,70	No
ENSMUSG00000082364	-9,00E-01	-1,355553706	3,01E+00	8,27E-02	3,70E-01	Gm11955	0,54	No
ENSMUSG00000082365	8,90E-02	-1,311160678	3,52E-02	8,51E-01	9,90E-01	Gm15754	1,06	No
ENSMUSG00000082367	-2,69E+00	-2,831970912	2,88E+00	8,99E-02	3,80E-01	Gm12363	0,15	No
ENSMUSG00000082368	2,65E-01	1,334128733	2,06E+00	1,51E-01	4,93E-01	Gm11225	1,20	No
ENSMUSG00000082369	-3,20E+00	-2,963227503	4,31E+00	3,79E-02	2,31E-01	Gm14208	0,11	No
ENSMUSG00000082373	-6,43E-02	-2,83202899	2,81E-03	9,58E-01	1,00E+00	Gm13754	0,96	No
ENSMUSG00000082374	3,14E-02	-3,107617333	-7,69E-10	1,00E+00	1,00E+00	Gm12741	1,02	No
ENSMUSG00000082377	1,87E+00	-3,033632735	1,36E+00	2,43E-01	5,50E-01	Gm14415	3,66	No
ENSMUSG00000082379	-7,44E-02	-1,424848714	1,94E-02	8,89E-01	1,00E+00	Gm13884	0,95	No
ENSMUSG00000082380	1,56E-01	-0,683037006	1,54E-01	6,95E-01	9,02E-01	Gm12218	1,11	No
ENSMUSG00000082381	1,12E-01	2,754853064	8,79E-01	3,48E-01	6,50E-01	Gm12928	1,08	No

ENSMUSG00000082389	9,40E-02	3,891976358	1,06E+00	3,02E-01	5,99E-01	Mkrn1-ps1	1,07	No
ENSMUSG00000082390	1,87E+00	-3,107639691	1,38E+00	2,40E-01	5,50E-01	Gm12136	3,67	No
ENSMUSG00000082394	-6,53E-02	5,050270634	7,89E-01	3,74E-01	6,75E-01	Gm4596	0,96	No
ENSMUSG00000082395	3,78E-01	-1,355658437	5,79E-01	4,47E-01	7,37E-01	Gm15480	1,30	No
ENSMUSG00000082396	-1,01E+00	-2,268017351	1,56E+00	2,11E-01	5,50E-01	Zfp389	0,50	No
ENSMUSG00000082399	-1,68E-01	4,554799145	4,45E+00	3,50E-02	2,21E-01	Gm14036	0,89	No
ENSMUSG00000082402	-7,63E-01	-1,9631302	1,19E+00	2,76E-01	5,73E-01	Gm15621	0,59	No
ENSMUSG00000082405	2,61E+00	-3,033672762	2,63E+00	1,05E-01	3,97E-01	Gm16021	6,10	No
ENSMUSG00000082408	-1,72E+00	-2,832001194	2,06E+00	1,51E-01	4,92E-01	Gm13545	0,30	No
ENSMUSG00000082413	-2,71E+00	-2,896132076	2,92E+00	8,76E-02	3,78E-01	Gm13401	0,15	No
ENSMUSG00000082415	3,10E-01	0,111952151	1,25E+00	2,63E-01	5,57E-01	Gm12486	1,24	No
ENSMUSG00000082417	-1,99E-01	-0,093219528	4,41E-01	5,07E-01	7,91E-01	Gm13740	0,87	No
ENSMUSG00000082418	-8,21E-01	-2,448681178	9,83E-01	3,21E-01	6,22E-01	Gm13126	0,57	No
ENSMUSG00000082419	-2,98E-02	4,267757926	1,26E-01	7,23E-01	9,17E-01	Gm11425	0,98	No
ENSMUSG00000082420	-1,67E-02	5,817375748	6,22E-02	8,03E-01	9,62E-01	Gm6517	0,99	No
ENSMUSG00000082424	1,43E-01	6,171641182	4,94E+00	2,62E-02	1,81E-01	Gm13292	1,10	No
ENSMUSG00000082429	-7,50E-02	-2,548225897	5,11E-03	9,43E-01	1,00E+00	Gm13171	0,95	No
ENSMUSG00000082431	-1,85E-02	6,239114379	8,35E-02	7,73E-01	9,44E-01	Tdpx-ps1	0,99	No
ENSMUSG00000082433	1,27E-01	7,347949205	4,51E+00	3,37E-02	2,16E-01	Gm9025	1,09	No
ENSMUSG00000082436	1,75E-01	0,316816588	4,18E-01	5,18E-01	7,98E-01	Gm11688	1,13	No
ENSMUSG00000082453	2,61E-01	-0,136113092	6,90E-01	4,06E-01	7,03E-01	Gm11502	1,20	No
ENSMUSG00000082454	-1,28E-01	9,281650536	5,12E+00	2,36E-02	1,69E-01	Gm12183	0,91	No
ENSMUSG00000082455	1,79E+00	-2,832057733	1,29E+00	2,55E-01	5,50E-01	Gm13665	3,47	No
ENSMUSG00000082456	-1,45E-01	3,121053831	1,74E+00	1,87E-01	5,50E-01	Gm11598	0,90	No
ENSMUSG00000082457	-5,16E-01	-1,497540796	8,62E-01	3,53E-01	6,54E-01	Gm13776	0,70	No
ENSMUSG00000082460	-1,94E+00	-3,107619243	1,50E+00	2,21E-01	5,50E-01	Gm13685	0,26	No
ENSMUSG00000082463	-7,80E-02	-2,14607765	1,13E-02	9,15E-01	1,00E+00	Gm15765	0,95	No
ENSMUSG00000082465	-1,33E-01	5,076237882	3,29E+00	6,97E-02	3,33E-01	Rps6-ps3	0,91	No
ENSMUSG00000082473	2,56E-02	-1,497580721	2,37E-03	9,61E-01	1,00E+00	Kif22-ps	1,02	No
ENSMUSG00000082474	1,66E-01	3,064823002	2,30E+00	1,29E-01	4,51E-01	Gm8213	1,12	No
ENSMUSG00000082475	-8,61E-02	5,70984155	1,63E+00	2,02E-01	5,50E-01	Gm7206	0,94	No
ENSMUSG00000082476	5,31E-01	-1,655130363	1,00E+00	3,17E-01	6,18E-01	Gm12242	1,44	No

ENSMUSG00000082478	1,81E+00	-3,10765737	1,28E+00	2,58E-01	5,50E-01	Gm11619	3,49	No
ENSMUSG00000082480	4,25E-02	3,681847152	1,96E-01	6,58E-01	8,81E-01	Gm11687	1,03	No
ENSMUSG00000082481	-8,85E-01	-1,18552592	3,36E+00	6,67E-02	3,25E-01	Rps12-ps19	0,54	No
ENSMUSG00000082483	-2,71E+00	-3,033620915	2,92E+00	8,75E-02	3,78E-01	Gm13486	0,15	No
ENSMUSG00000082484	-7,86E-01	-1,165392401	2,39E+00	1,22E-01	4,36E-01	Gm16177	0,58	No
ENSMUSG00000082487	-1,28E-01	1,419540164	4,80E-01	4,88E-01	7,75E-01	Gm11628	0,92	No
ENSMUSG00000082491	-9,53E-02	4,932931415	1,64E+00	2,01E-01	5,50E-01	Gm5909	0,94	No
ENSMUSG00000082498	-4,35E-01	-2,655141366	1,94E-01	6,60E-01	8,82E-01	Gm12937	0,74	No
ENSMUSG00000082503	-4,00E-01	-2,185632134	3,33E-01	5,64E-01	8,26E-01	Gm12229	0,76	No
ENSMUSG00000082507	7,35E-01	-1,655154947	1,64E+00	2,01E-01	5,50E-01	Gm9378	1,66	No
ENSMUSG00000082510	-3,88E-02	1,621650643	5,24E-02	8,19E-01	9,71E-01	Gm11793	0,97	No
ENSMUSG00000082512	-2,43E-01	-0,600694665	4,54E-01	5,01E-01	7,86E-01	Gm13937	0,84	No
ENSMUSG00000082513	-3,86E+00	-2,401360343	7,13E+00	7,60E-03	7,62E-02	Gm15374	0,07	No
ENSMUSG00000082514	1,16E-01	-0,998107965	7,42E-02	7,85E-01	9,52E-01	Gm11452	1,08	No
ENSMUSG00000082515	5,86E-02	0,533139636	5,94E-02	8,07E-01	9,64E-01	Gm12902	1,04	No
ENSMUSG00000082516	-2,17E-02	3,963472118	5,88E-02	8,08E-01	9,65E-01	Gm13342	0,99	No
ENSMUSG00000082519	-1,44E-01	1,814778873	8,17E-01	3,66E-01	6,67E-01	Vamp7-ps	0,90	No
ENSMUSG00000082520	3,25E-01	-2,548215269	1,08E-01	7,43E-01	9,28E-01	Gm13349	1,25	No
ENSMUSG00000082529	9,01E-03	-3,107646452	-5,16E-10	1,00E+00	1,00E+00	Gm11199	1,01	No
ENSMUSG00000082530	-1,22E-02	3,230471019	1,31E-02	9,09E-01	1,00E+00	Gm12168	0,99	No
ENSMUSG00000082532	-9,74E-01	-2,448704144	1,15E+00	2,84E-01	5,81E-01	Rpl31-ps6	0,51	No
ENSMUSG00000082534	8,56E-02	3,266387619	6,52E-01	4,19E-01	7,15E-01	Gm13712	1,06	No
ENSMUSG00000082535	2,74E-02	0,508571554	1,27E-02	9,10E-01	1,00E+00	Gm7860	1,02	No
ENSMUSG00000082536	-2,02E-01	10,90880622	1,31E+01	2,95E-04	5,98E-03	Gm13456	0,87	Yes
ENSMUSG00000082543	-7,65E-02	-1,246983359	2,33E-02	8,79E-01	1,00E+00	Gm14336	0,95	No
ENSMUSG00000082548	9,01E-03	-3,107646452	-5,16E-10	1,00E+00	1,00E+00	Gm12377	1,01	No
ENSMUSG00000082550	1,00E+00	-0,726314483	5,34E+00	2,09E-02	1,55E-01	Gm12344	2,00	No
ENSMUSG00000082554	2,05E-01	0,966549629	8,99E-01	3,43E-01	6,43E-01	Gm15806	1,15	No
ENSMUSG00000082561	-4,20E-01	-2,929320178	1,09E-01	7,41E-01	9,27E-01	Gm15580	0,75	No
ENSMUSG00000082566	1,47E-01	-1,070181601	1,04E-01	7,47E-01	9,30E-01	Gm11485	1,11	No
ENSMUSG00000082570	4,74E-01	-2,600680117	1,75E-01	6,76E-01	8,91E-01	Gm15711	1,39	No
ENSMUSG00000082575	-1,51E-01	8,690485185	7,00E+00	8,16E-03	8,02E-02	Gm13050	0,90	No

ENSMUSG00000082576	3,46E-01	-1,448754033	4,15E-01	5,19E-01	7,98E-01	Gm12058	1,27	No
ENSMUSG00000082579	-1,69E+00	-2,655102197	1,97E+00	1,60E-01	5,08E-01	Gm11979	0,31	No
ENSMUSG00000082580	-3,73E-01	-0,448901827	1,29E+00	2,57E-01	5,50E-01	Gm13182	0,77	No
ENSMUSG00000082585	-4,10E-02	5,598761557	3,59E-01	5,49E-01	8,16E-01	Gm15387	0,97	No
ENSMUSG00000082587	1,86E+00	-3,033661846	1,35E+00	2,46E-01	5,50E-01	Gm12129	3,62	No
ENSMUSG00000082588	-8,00E-02	1,432104632	2,05E-01	6,51E-01	8,76E-01	Gm15443	0,95	No
ENSMUSG00000082592	-1,87E+00	-3,107637761	1,39E+00	2,38E-01	5,50E-01	Gm12950	0,27	No
ENSMUSG00000082593	-2,68E+00	-2,896144247	2,85E+00	9,15E-02	3,82E-01	Gm11331	0,16	No
ENSMUSG00000082596	-3,00E-01	0,066628925	9,57E-01	3,28E-01	6,29E-01	Gm14227	0,81	No
ENSMUSG00000082599	-3,58E+00	-2,770569053	5,77E+00	1,63E-02	1,33E-01	Gm13141	0,08	No
ENSMUSG00000082601	-6,42E-02	-1,548019534	1,45E-02	9,04E-01	1,00E+00	Gm12700	0,96	No
ENSMUSG00000082604	-1,89E-01	-0,56103219	2,52E-01	6,16E-01	8,54E-01	Gm8482	0,88	No
ENSMUSG00000082605	-5,19E-01	-0,71885227	1,75E+00	1,85E-01	5,49E-01	Gm16148	0,70	No
ENSMUSG00000082607	2,46E-01	0,49010036	1,01E+00	3,16E-01	6,16E-01	Gm12045	1,19	No
ENSMUSG00000082609	-5,81E-01	2,323734568	1,86E+01	1,59E-05	5,05E-04	Gm15464	0,67	Yes
ENSMUSG00000082610	4,59E-01	-2,711755758	1,64E-01	6,85E-01	8,97E-01	Gm13829	1,37	No
ENSMUSG00000082615	-3,83E-02	-3,107651936	-7,91E-11	1,00E+00	1,00E+00	Gm13239	0,97	No
ENSMUSG00000082623	-1,59E-02	-0,09818775	2,75E-03	9,58E-01	1,00E+00	Gm15493	0,99	No
ENSMUSG00000082626	-4,38E-02	3,71264003	2,17E-01	6,42E-01	8,71E-01	Rps13-ps5	0,97	No
ENSMUSG00000082632	-8,77E-01	-0,755640585	4,25E+00	3,92E-02	2,35E-01	Gm13365	0,54	No
ENSMUSG00000082633	2,62E+00	-3,033654625	2,66E+00	1,03E-01	3,95E-01	Gm15779	6,16	No
ENSMUSG00000082641	-7,83E-02	-2,832047505	4,20E-03	9,48E-01	1,00E+00	Rpl30-ps5	0,95	No
ENSMUSG00000082645	5,79E-02	-2,289547055	5,24E-03	9,42E-01	1,00E+00	Gm15481	1,04	No
ENSMUSG00000082646	2,65E+00	-3,033655087	2,73E+00	9,86E-02	3,91E-01	Gm12732	6,30	No
ENSMUSG00000082647	-6,19E-01	-2,226239746	7,44E-01	3,88E-01	6,89E-01	Gm14056	0,65	No
ENSMUSG00000082650	1,85E-01	-0,497728773	2,78E-01	5,98E-01	8,45E-01	Gm15358	1,14	No
ENSMUSG00000082651	-1,36E+00	-2,83195595	1,17E+00	2,80E-01	5,77E-01	Gm14001	0,39	No
ENSMUSG00000082653	-2,88E-02	-2,963268425	3,79E-04	9,84E-01	1,00E+00	Gm15897	0,98	No
ENSMUSG00000082658	2,61E+00	-3,033672762	2,63E+00	1,05E-01	3,97E-01	Fau-ps2	6,10	No
ENSMUSG00000082662	-3,57E-01	4,181764398	1,77E+01	2,62E-05	7,81E-04	Gm15516	0,78	Yes
ENSMUSG00000082663	7,43E-01	-2,963261206	3,01E-01	5,83E-01	8,38E-01	Trav13-3	1,67	No
ENSMUSG00000082665	1,19E-01	2,72303677	9,54E-01	3,29E-01	6,29E-01	Gm11470	1,09	No

ENSMUSG00000082674	1,15E-01	2,151925228	6,22E-01	4,30E-01	7,24E-01	Gm11914	1,08	No
ENSMUSG00000082676	4,24E-02	-3,033592245	-1,17E-09	1,00E+00	1,00E+00	Gm11843	1,03	No
ENSMUSG00000082677	-8,11E-01	-2,600594638	3,64E-01	5,46E-01	8,14E-01	Gm15371	0,57	No
ENSMUSG00000082678	-9,80E-02	2,585780179	5,95E-01	4,40E-01	7,32E-01	Gm12818	0,93	No
ENSMUSG00000082682	8,40E-01	-2,711767974	6,14E-01	4,33E-01	7,27E-01	Gm15349	1,79	No
ENSMUSG00000082684	4,26E-01	-2,65517552	1,42E-01	7,06E-01	9,08E-01	Gm12774	1,34	No
ENSMUSG00000082686	-3,82E-01	-0,321975403	9,50E-01	3,30E-01	6,30E-01	Gm12961	0,77	No
ENSMUSG00000082687	2,65E+00	-3,033655087	2,73E+00	9,86E-02	3,91E-01	Gm11474	6,30	No
ENSMUSG00000082688	-1,67E-02	-2,963250293	1,29E-04	9,91E-01	1,00E+00	Gm12124	0,99	No
ENSMUSG00000082691	1,07E-01	5,922394392	2,59E+00	1,08E-01	4,02E-01	Dynlt1-ps1	1,08	No
ENSMUSG00000082692	1,81E+00	-3,10765737	1,28E+00	2,58E-01	5,50E-01	H2af-ps	3,49	No
ENSMUSG00000082704	-1,72E+00	-2,770555969	2,06E+00	1,51E-01	4,93E-01	Gm12515	0,30	No
ENSMUSG00000082705	-1,22E-01	2,584385735	9,51E-01	3,29E-01	6,30E-01	Gm15616	0,92	No
ENSMUSG00000082706	3,54E-01	0,470986197	1,92E+00	1,65E-01	5,18E-01	Gm11663	1,28	No
ENSMUSG00000082709	2,46E-01	0,581126247	1,02E+00	3,13E-01	6,12E-01	Gm12362	1,19	No
ENSMUSG00000082711	-5,58E-01	-2,448601375	2,45E-01	6,21E-01	8,57E-01	Gm12987	0,68	No
ENSMUSG00000082715	-1,07E-01	5,496159444	2,40E+00	1,22E-01	4,35E-01	Gm11633	0,93	No
ENSMUSG00000082716	7,24E-01	-2,896104002	2,86E-01	5,93E-01	8,42E-01	Rpl31-ps5	1,65	No
ENSMUSG00000082721	-4,08E-02	-2,963255775	7,59E-04	9,78E-01	1,00E+00	Gm12087	0,97	No
ENSMUSG00000082724	1,09E-01	-0,567624986	8,93E-02	7,65E-01	9,40E-01	Gm14416	1,08	No
ENSMUSG00000082726	-7,38E-02	-3,033634644	2,66E-03	9,59E-01	1,00E+00	Gm11424	0,95	No
ENSMUSG00000082727	4,93E-01	-1,289545274	8,92E-01	3,45E-01	6,46E-01	Gm13260	1,41	No
ENSMUSG00000082737	4,92E-01	-1,711620155	6,09E-01	4,35E-01	7,28E-01	Gm12026	1,41	No
ENSMUSG00000082738	4,94E-02	0,451776738	3,94E-02	8,43E-01	9,85E-01	Gm15422	1,03	No
ENSMUSG00000082740	-3,89E-01	2,167989774	7,72E+00	5,47E-03	5,97E-02	Gm14127	0,76	No
ENSMUSG00000082741	-3,76E-01	1,619071494	5,10E+00	2,40E-02	1,70E-01	Gm9703	0,77	No
ENSMUSG00000082743	-1,52E-01	3,295881905	2,11E+00	1,46E-01	4,85E-01	Gm12988	0,90	No
ENSMUSG00000082744	2,55E+00	-2,378334268	5,87E+00	1,54E-02	1,28E-01	Gm15625	5,87	No
ENSMUSG00000082760	1,18E-01	0,948254824	3,06E-01	5,80E-01	8,37E-01	Gm15546	1,09	No
ENSMUSG00000082762	-3,00E-02	6,355797916	2,22E-01	6,38E-01	8,68E-01	Gm12366	0,98	No
ENSMUSG00000082765	-1,22E+00	-2,998024514	7,09E-01	4,00E-01	6,99E-01	Gm14411	0,43	No
ENSMUSG00000082766	7,36E-01	-2,89615794	2,95E-01	5,87E-01	8,40E-01	1700064H15Rik	1,67	No

ENSMUSG00000082768	-1,02E-01	-0,060905855	1,06E-01	7,44E-01	9,29E-01	Gm12727	0,93	No
ENSMUSG00000082769	5,42E-03	3,651140693	3,18E-03	9,55E-01	1,00E+00	Gm15793	1,00	No
ENSMUSG00000082774	-7,30E-01	-2,448635376	5,97E-01	4,40E-01	7,32E-01	Gm8805	0,60	No
ENSMUSG00000082779	-1,32E-01	0,244677883	2,32E-01	6,30E-01	8,63E-01	Gm13162	0,91	No
ENSMUSG00000082782	-1,17E-01	2,530525444	8,46E-01	3,58E-01	6,59E-01	Gm5319	0,92	No
ENSMUSG00000082789	1,36E-01	-2,185681434	3,22E-02	8,58E-01	9,94E-01	Gm13284	1,10	No
ENSMUSG00000082791	-4,13E-02	5,703826988	3,74E-01	5,41E-01	8,11E-01	Gm4875	0,97	No
ENSMUSG00000082792	5,78E-01	-2,03355424	7,60E-01	3,83E-01	6,85E-01	Gm12176	1,49	No
ENSMUSG00000082796	8,57E-01	-2,401419972	8,83E-01	3,47E-01	6,48E-01	Gm13385	1,81	No
ENSMUSG00000082804	-1,94E+00	-3,107619243	1,50E+00	2,21E-01	5,50E-01	Rpl17-ps1	0,26	No
ENSMUSG00000082806	1,13E+00	-1,600746999	2,58E+00	1,08E-01	4,03E-01	Rpl13-ps1	2,18	No
ENSMUSG00000082809	1,06E-01	7,968489048	3,33E+00	6,82E-02	3,29E-01	Gm14150	1,08	No
ENSMUSG00000082816	-1,88E-02	-0,998039547	1,94E-03	9,65E-01	1,00E+00	Gm11953	0,99	No
ENSMUSG00000082820	-6,18E-02	2,577683496	2,43E-01	6,22E-01	8,58E-01	Gm13803	0,96	No
ENSMUSG00000082826	3,65E-01	1,73158112	4,65E+00	3,11E-02	2,03E-01	Mif-ps3	1,29	No
ENSMUSG00000082828	6,36E-02	-0,995133339	2,29E-02	8,80E-01	1,00E+00	Gm16106	1,05	No
ENSMUSG00000082836	5,36E-02	2,740046248	1,94E-01	6,60E-01	8,82E-01	Gm13612	1,04	No
ENSMUSG00000082837	-3,57E-01	-2,401428949	1,60E-01	6,89E-01	8,99E-01	Gm15373	0,78	No
ENSMUSG00000082844	-4,27E-01	-1,574018299	5,29E-01	4,67E-01	7,55E-01	Gm13537	0,74	No
ENSMUSG00000082848	-4,17E-02	0,3333094	2,52E-02	8,74E-01	1,00E+00	Gm16066	0,97	No
ENSMUSG00000082852	-9,20E-01	-2,770574044	7,38E-01	3,90E-01	6,91E-01	Gm12021	0,53	No
ENSMUSG00000082860	1,23E-01	0,641352698	2,89E-01	5,91E-01	8,41E-01	Gm13204	1,09	No
ENSMUSG00000082861	-1,63E-01	-0,58730202	2,16E-01	6,42E-01	8,71E-01	Gm11362	0,89	No
ENSMUSG00000082864	-3,23E+00	-2,832003132	4,38E+00	3,64E-02	2,27E-01	Gm13358	0,11	No
ENSMUSG00000082867	-1,32E+00	-2,831981163	1,08E+00	2,98E-01	5,94E-01	Gm15730	0,40	No
ENSMUSG00000082870	1,87E+00	-3,107639691	1,38E+00	2,40E-01	5,50E-01	Gm12165	3,67	No
ENSMUSG00000082872	1,09E-01	3,326833302	1,08E+00	2,98E-01	5,94E-01	Gm15773	1,08	No
ENSMUSG00000082875	-6,93E-01	-0,831978725	3,18E+00	7,44E-02	3,47E-01	Gm12598	0,62	No
ENSMUSG00000082876	-2,14E-02	5,380964522	9,25E-02	7,61E-01	9,37E-01	Gm11889	0,99	No
ENSMUSG00000082878	-2,27E-02	2,670810003	3,43E-02	8,53E-01	9,91E-01	Gm12583	0,98	No
ENSMUSG00000082884	2,05E-01	3,038241508	3,45E+00	6,34E-02	3,14E-01	Gm13339	1,15	No
ENSMUSG00000082888	-5,89E-02	-2,497589368	3,97E-03	9,50E-01	1,00E+00	Gm12934	0,96	No

ENSMUSG00000082895	-3,94E-01	6,329420887	3,86E+01	5,21E-10	4,44E-08	Rpsa-ps9	0,76	Yes
ENSMUSG00000082896	2,08E-02	5,659314075	9,42E-02	7,59E-01	9,36E-01	Gm5844	1,01	No
ENSMUSG00000082897	3,14E-02	-3,107617333	-7,69E-10	1,00E+00	1,00E+00	Gm11982	1,02	No
ENSMUSG00000082901	-1,33E-01	3,970648381	2,25E+00	1,33E-01	4,60E-01	Gm14323	0,91	No
ENSMUSG00000082908	-5,03E-02	4,858312064	4,38E-01	5,08E-01	7,92E-01	Gm13736	0,97	No
ENSMUSG00000082911	1,59E+00	-2,770633681	1,73E+00	1,89E-01	5,50E-01	Gm14119	3,01	No
ENSMUSG00000082917	-1,93E+00	-2,963274349	1,47E+00	2,25E-01	5,50E-01	Gm11440	0,26	No
ENSMUSG00000082920	-2,26E-01	3,181782225	4,29E+00	3,83E-02	2,32E-01	Gm13864	0,86	No
ENSMUSG00000082922	-2,15E-01	0,191276524	5,95E-01	4,40E-01	7,32E-01	Gm6939	0,86	No
ENSMUSG00000082925	8,14E-02	4,492335056	1,02E+00	3,13E-01	6,13E-01	Gm13135	1,06	No
ENSMUSG00000082928	-5,60E-01	-1,627546018	9,41E-01	3,32E-01	6,32E-01	Gm12785	0,68	No
ENSMUSG00000082930	3,14E-02	-3,107617333	-7,69E-10	1,00E+00	1,00E+00	Gm13462	1,02	No
ENSMUSG00000082931	-1,34E-01	1,676466062	6,11E-01	4,35E-01	7,27E-01	Gm13586	0,91	No
ENSMUSG00000082932	9,01E-03	-3,107646452	-5,16E-10	1,00E+00	1,00E+00	Cyp2j8	1,01	No
ENSMUSG00000082935	-1,14E-01	6,475606248	3,33E+00	6,82E-02	3,29E-01	Gm7658	0,92	No
ENSMUSG00000082936	3,14E-01	-1,21616445	4,72E-01	4,92E-01	7,78E-01	Gm16166	1,24	No
ENSMUSG00000082938	4,58E-02	2,796651362	1,49E-01	6,99E-01	9,04E-01	Gm2810	1,03	No
ENSMUSG00000082941	-7,16E-02	-2,831969669	3,50E-03	9,53E-01	1,00E+00	Gm12850	0,95	No
ENSMUSG00000082945	-3,88E+00	-2,600684939	7,19E+00	7,31E-03	7,41E-02	Gm11540	0,07	No
ENSMUSG00000082951	-3,95E-01	0,897089114	3,25E+00	7,14E-02	3,38E-01	Gm13359	0,76	No
ENSMUSG00000082953	2,85E-02	3,547620033	8,25E-02	7,74E-01	9,44E-01	Gm13217	1,02	No
ENSMUSG00000082954	-1,94E+00	-3,107619243	1,50E+00	2,21E-01	5,50E-01	Gm15504	0,26	No
ENSMUSG00000082956	-4,31E-02	1,708771826	7,21E-02	7,88E-01	9,53E-01	Naip3	0,97	No
ENSMUSG00000082957	8,40E-02	0,187525239	9,05E-02	7,64E-01	9,39E-01	Gm15303	1,06	No
ENSMUSG00000082962	-3,23E+00	-2,770552905	4,38E+00	3,64E-02	2,27E-01	Gm10601	0,11	No
ENSMUSG00000082969	1,87E-01	-1,574260912	9,74E-02	7,55E-01	9,34E-01	Gm12630	1,14	No
ENSMUSG00000082971	3,51E-01	2,181234723	5,73E+00	1,67E-02	1,35E-01	Gm13517	1,28	No
ENSMUSG00000082978	-3,91E-01	3,654577359	1,68E+01	4,26E-05	1,19E-03	Rpsa-ps11	0,76	Yes
ENSMUSG00000082980	-3,83E-02	-3,107651936	-7,91E-11	1,00E+00	1,00E+00	Olfr1274-ps	0,97	No
ENSMUSG00000082981	-5,93E-01	-1,497719381	1,18E+00	2,78E-01	5,75E-01	Polr2k-ps	0,66	No
ENSMUSG00000082983	1,22E+00	-3,146114919	6,90E-01	4,06E-01	7,02E-01	Gm12318	2,33	No
ENSMUSG00000082985	4,28E-02	-1,574193792	5,78E-03	9,39E-01	1,00E+00	Gm14042	1,03	No

ENSMUSG00000082987	2,61E+00	-3,033672762	2,63E+00	1,05E-01	3,97E-01	Rps13-ps6	6,10	No
ENSMUSG00000082988	5,44E-02	5,202732521	5,80E-01	4,46E-01	7,37E-01	Gm5903	1,04	No
ENSMUSG00000082990	3,29E-01	0,12396688	1,26E+00	2,62E-01	5,57E-01	Gm14052	1,26	No
ENSMUSG00000082998	-1,00E-01	4,52274339	1,57E+00	2,11E-01	5,50E-01	Gm11221	0,93	No
ENSMUSG00000083004	-8,22E-01	-1,548185292	2,22E+00	1,36E-01	4,65E-01	Gm13413	0,57	No
ENSMUSG00000083006	7,41E-01	-2,37828866	6,37E-01	4,25E-01	7,19E-01	Gm15715	1,67	No
ENSMUSG00000083007	-3,30E-01	0,136438319	1,29E+00	2,56E-01	5,50E-01	Gm13623	0,80	No
ENSMUSG00000083008	1,59E+00	-2,770669833	1,73E+00	1,89E-01	5,50E-01	Gm12053	3,01	No
ENSMUSG00000083009	1,81E+00	-3,10765737	1,28E+00	2,58E-01	5,50E-01	Gm1335	3,49	No
ENSMUSG00000083011	-1,06E-01	4,350684385	1,64E+00	2,00E-01	5,50E-01	Gm12816	0,93	No
ENSMUSG00000083012	1,29E-01	4,14956651	2,20E+00	1,38E-01	4,68E-01	Fam220a	1,09	No
ENSMUSG00000083013	-2,39E-02	2,257249171	2,87E-02	8,65E-01	9,98E-01	Gm12978	0,98	No
ENSMUSG00000083014	1,36E+00	-2,311229105	1,88E+00	1,70E-01	5,26E-01	Gm11764	2,56	No
ENSMUSG00000083016	1,87E+00	-3,107639691	1,38E+00	2,40E-01	5,50E-01	Gm12618	3,67	No
ENSMUSG00000083017	-1,46E+00	-2,600669928	2,21E+00	1,37E-01	4,66E-01	Gm12163	0,36	No
ENSMUSG00000083020	-3,83E-02	-3,107651936	-7,91E-11	1,00E+00	1,00E+00	Gm15331	0,97	No
ENSMUSG00000083022	-2,72E-01	6,10413108	1,77E+01	2,60E-05	7,78E-04	Rps15a-ps6	0,83	Yes
ENSMUSG00000083027	2,81E-02	3,371197051	7,36E-02	7,86E-01	9,52E-01	Gm13140	1,02	No
ENSMUSG00000083034	-5,88E-02	-3,033616507	1,62E-03	9,68E-01	1,00E+00	Olfr334-ps1	0,96	No
ENSMUSG00000083036	-7,39E-02	-2,205804766	9,49E-03	9,22E-01	1,00E+00	Gm15959	0,95	No
ENSMUSG00000083038	-2,10E-01	3,057239901	3,66E+00	5,59E-02	2,90E-01	Gm13233	0,86	No
ENSMUSG00000083041	-7,83E-02	5,436685202	1,26E+00	2,62E-01	5,56E-01	Gmfg-ps	0,95	No
ENSMUSG00000083043	-2,36E-01	3,108863459	4,58E+00	3,24E-02	2,10E-01	Gm13433	0,85	No
ENSMUSG00000083044	6,16E-02	1,928231991	1,63E-01	6,86E-01	8,97E-01	Gm12416	1,04	No
ENSMUSG00000083045	-1,26E+00	-2,711724243	1,52E+00	2,17E-01	5,50E-01	Gm16100	0,42	No
ENSMUSG00000083054	1,17E+00	-3,14612376	6,40E-01	4,24E-01	7,18E-01	Gm12986	2,26	No
ENSMUSG00000083055	-7,40E-02	2,2488063	2,90E-01	5,90E-01	8,41E-01	Gm14140	0,95	No
ENSMUSG00000083061	-2,65E-01	7,830860517	2,06E+01	5,76E-06	2,05E-04	Gm12191	0,83	Yes
ENSMUSG00000083064	-2,74E-02	3,787106457	8,71E-02	7,68E-01	9,41E-01	Gm7785	0,98	No
ENSMUSG00000083070	-1,87E+00	-3,107637761	1,39E+00	2,38E-01	5,50E-01	Gm13350	0,27	No
ENSMUSG00000083072	-1,94E+00	-3,107619243	1,50E+00	2,21E-01	5,50E-01	Gm13668	0,26	No
ENSMUSG00000083075	-1,16E+00	-2,268116729	2,18E+00	1,40E-01	4,73E-01	Gm11980	0,45	No

ENSMUSG00000083076	-9,68E-01	-2,355526908	1,13E+00	2,87E-01	5,83E-01	Gm11408	0,51	No
ENSMUSG00000083077	-3,53E-01	-1,448722619	4,78E-01	4,89E-01	7,76E-01	Gm14126	0,78	No
ENSMUSG00000083081	-1,79E+00	-2,205807414	4,33E+00	3,75E-02	2,30E-01	Gm13223	0,29	No
ENSMUSG00000083083	4,55E-01	-1,683190474	5,72E-01	4,50E-01	7,40E-01	Gm15382	1,37	No
ENSMUSG00000083086	-2,70E+00	-3,033614577	2,89E+00	8,93E-02	3,79E-01	Gm15531	0,15	No
ENSMUSG00000083087	-1,18E-01	4,867789372	2,41E+00	1,21E-01	4,33E-01	Gm11249	0,92	No
ENSMUSG00000083097	-4,87E-02	6,153110765	5,66E-01	4,52E-01	7,42E-01	Gm14494	0,97	No
ENSMUSG00000083100	-1,01E+00	-1,65495478	2,60E+00	1,07E-01	4,01E-01	Gm6044	0,50	No
ENSMUSG00000083101	-3,16E-01	0,581530187	1,72E+00	1,89E-01	5,50E-01	Gm13430	0,80	No
ENSMUSG00000083104	-4,87E-01	-1,355529749	8,26E-01	3,64E-01	6,65E-01	Gm12917	0,71	No
ENSMUSG00000083107	2,21E-01	3,040412602	3,83E+00	5,02E-02	2,72E-01	Gm5303	1,17	No
ENSMUSG00000083111	1,70E-01	-0,48540864	2,60E-01	6,10E-01	8,51E-01	Gm14421	1,13	No
ENSMUSG00000083115	-5,41E-01	-2,311076229	4,69E-01	4,94E-01	7,79E-01	Gm13386	0,69	No
ENSMUSG00000083116	-2,72E-02	5,588108401	1,57E-01	6,92E-01	9,00E-01	Gm13410	0,98	No
ENSMUSG00000083118	-1,94E+00	-3,107619243	1,50E+00	2,21E-01	5,50E-01	Gm11609	0,26	No
ENSMUSG00000083121	-1,09E-01	1,552618129	3,95E-01	5,30E-01	8,05E-01	Gm6341	0,93	No
ENSMUSG00000083126	-3,53E-01	-0,424655	9,24E-01	3,36E-01	6,37E-01	Gm14109	0,78	No
ENSMUSG00000083139	-2,91E-01	2,987366328	6,64E+00	1,00E-02	9,33E-02	Gm12418	0,82	No
ENSMUSG00000083142	-1,92E+00	-3,033623267	1,46E+00	2,27E-01	5,50E-01	Gm12288	0,26	No
ENSMUSG00000083149	-6,88E-02	-2,896163841	2,08E-03	9,64E-01	1,00E+00	Gm11380	0,95	No
ENSMUSG00000083150	4,79E-01	-2,770633054	1,80E-01	6,71E-01	8,89E-01	Gm11852	1,39	No
ENSMUSG00000083152	-4,20E-01	0,099766739	2,11E+00	1,46E-01	4,85E-01	Apc-ps1	0,75	No
ENSMUSG00000083156	-1,20E-01	-0,257796756	1,42E-01	7,06E-01	9,08E-01	Gm12201	0,92	No
ENSMUSG00000083157	5,95E-01	-2,401385804	3,94E-01	5,30E-01	8,05E-01	Gm13511	1,51	No
ENSMUSG00000083159	3,00E-01	1,099954859	2,30E+00	1,30E-01	4,52E-01	Gm12180	1,23	No
ENSMUSG00000083161	5,23E-02	5,237615121	5,34E-01	4,65E-01	7,54E-01	Gm11427	1,04	No
ENSMUSG00000083163	2,65E+00	-3,033655087	2,73E+00	9,86E-02	3,91E-01	Gm11429	6,30	No
ENSMUSG00000083168	1,85E+00	-3,107657832	1,35E+00	2,46E-01	5,50E-01	Gm14201	3,61	No
ENSMUSG00000083169	4,88E-04	4,760128196	4,06E-05	9,95E-01	1,00E+00	Gm6580	1,00	No
ENSMUSG00000083170	-2,91E-01	-1,980485445	2,12E-01	6,45E-01	8,73E-01	Gm14405	0,82	No
ENSMUSG00000083171	1,95E-01	2,80334531	2,67E+00	1,03E-01	3,95E-01	Gm12321	1,14	No
ENSMUSG00000083176	-4,79E-01	-2,997999812	1,14E-01	7,35E-01	9,24E-01	D11Bhm181e	0,72	No

ENSMUSG00000083178	-1,46E-01	-2,088727692	4,34E-02	8,35E-01	9,80E-01	Gm12187	0,90	No
ENSMUSG00000083179	-7,34E-02	5,150814285	1,03E+00	3,10E-01	6,09E-01	Gm12693	0,95	No
ENSMUSG00000083183	1,77E-02	3,081385184	2,51E-02	8,74E-01	1,00E+00	Gm15575	1,01	No
ENSMUSG00000083186	1,20E-01	-0,25754883	1,52E-01	6,97E-01	9,03E-01	Gm11805	1,09	No
ENSMUSG00000083191	-2,71E+00	-3,033620915	2,92E+00	8,75E-02	3,78E-01	Gm15571	0,15	No
ENSMUSG00000083192	-1,70E-01	5,358064391	5,87E+00	1,54E-02	1,28E-01	Rpl31-ps12	0,89	No
ENSMUSG00000083193	-4,27E-01	-1,831996068	3,80E-01	5,38E-01	8,09E-01	4930595D18Rik	0,74	No
ENSMUSG00000083206	7,54E-01	-2,896122507	3,10E-01	5,78E-01	8,35E-01	Gm11338	1,69	No
ENSMUSG00000083208	-8,18E-02	-2,548202221	6,06E-03	9,38E-01	1,00E+00	Gm11522	0,94	No
ENSMUSG00000083209	4,09E-01	-2,770625443	1,29E-01	7,19E-01	9,15E-01	Gm11969	1,33	No
ENSMUSG00000083212	-1,87E+00	-3,107637761	1,39E+00	2,38E-01	5,50E-01	Gm12274	0,27	No
ENSMUSG00000083215	-9,52E-02	-0,175608294	8,90E-02	7,65E-01	9,40E-01	Rbmx2-ps	0,94	No
ENSMUSG00000083218	8,81E-02	4,591416476	1,22E+00	2,69E-01	5,65E-01	Gm16425	1,06	No
ENSMUSG00000083219	1,81E+00	-3,10765737	1,28E+00	2,58E-01	5,50E-01	Gm11410	3,49	No
ENSMUSG00000083220	-3,04E-01	0,281260851	1,22E+00	2,70E-01	5,67E-01	Gm12919	0,81	No
ENSMUSG00000083233	-2,70E+00	-3,033614577	2,89E+00	8,93E-02	3,79E-01	Gm13406	0,15	No
ENSMUSG00000083234	1,87E+00	-3,107639691	1,38E+00	2,40E-01	5,50E-01	Gm12262	3,67	No
ENSMUSG00000083235	-2,59E-01	-0,675975868	5,18E-01	4,72E-01	7,60E-01	Gm13246	0,84	No
ENSMUSG00000083237	2,10E-02	-1,378223426	1,59E-03	9,68E-01	1,00E+00	Gm11698	1,01	No
ENSMUSG00000083240	6,71E-02	6,416572856	1,12E+00	2,89E-01	5,85E-01	Gm13453	1,05	No
ENSMUSG00000083241	7,31E-01	-2,963243158	2,91E-01	5,90E-01	8,41E-01	Gm13259	1,66	No
ENSMUSG00000083242	9,76E-02	-0,185632248	9,57E-02	7,57E-01	9,35E-01	Gm15659	1,07	No
ENSMUSG00000083244	1,85E+00	-3,107657832	1,35E+00	2,46E-01	5,50E-01	Gm15180	3,61	No
ENSMUSG00000083245	-1,73E-02	-0,509892014	2,12E-03	9,63E-01	1,00E+00	Gm11185	0,99	No
ENSMUSG00000083246	-8,75E-02	1,179708506	2,00E-01	6,55E-01	8,79E-01	Gm11839	0,94	No
ENSMUSG00000083252	1,87E+00	-3,107639691	1,38E+00	2,40E-01	5,50E-01	Gm13210	3,67	No
ENSMUSG00000083257	6,17E-01	-2,401367972	4,24E-01	5,15E-01	7,96E-01	Gm15568	1,53	No
ENSMUSG00000083261	-8,26E-02	5,620386613	1,47E+00	2,25E-01	5,50E-01	Gm7816	0,94	No
ENSMUSG00000083264	-7,88E-02	-2,89616692	4,36E-03	9,47E-01	1,00E+00	Gm11461	0,95	No
ENSMUSG00000083268	-1,87E+00	-3,107637761	1,39E+00	2,38E-01	5,50E-01	Gm11511	0,27	No
ENSMUSG00000083270	-3,12E-02	4,331943881	1,41E-01	7,07E-01	9,08E-01	Gm13498	0,98	No
ENSMUSG00000083274	-2,68E+00	-3,033645766	2,84E+00	9,17E-02	3,82E-01	Zcchc9-ps	0,16	No

ENSMUSG00000083276	3,14E-02	-3,107617333	-7,69E-10	1,00E+00	1,00E+00	Gm13551	1,02	No
ENSMUSG00000083277	6,37E-01	-2,44867617	4,52E-01	5,01E-01	7,86E-01	Gm15376	1,55	No
ENSMUSG00000083279	-1,32E+00	-2,832010255	1,09E+00	2,96E-01	5,92E-01	Pex13-ps	0,40	No
ENSMUSG00000083280	9,62E-02	4,191368024	1,25E+00	2,64E-01	5,59E-01	Gm11838	1,07	No
ENSMUSG00000083281	1,65E-01	0,715701114	5,34E-01	4,65E-01	7,54E-01	Gm13332	1,12	No
ENSMUSG00000083282	7,06E-02	2,848057633	3,47E-01	5,56E-01	8,21E-01	Ctsf	1,05	No
ENSMUSG00000083287	-8,27E-02	5,25128693	1,37E+00	2,42E-01	5,50E-01	Gm13502	0,94	No
ENSMUSG00000083289	3,58E-01	-0,333372344	1,20E+00	2,73E-01	5,69E-01	Gm8812	1,28	No
ENSMUSG00000083298	8,93E-01	-1,863786166	2,03E+00	1,55E-01	4,98E-01	Gm12085	1,86	No
ENSMUSG00000083305	9,55E-02	0,233196545	1,23E-01	7,25E-01	9,18E-01	Gm13315	1,07	No
ENSMUSG00000083306	-2,67E+00	-2,448593492	6,57E+00	1,04E-02	9,60E-02	Gm13868	0,16	No
ENSMUSG00000083319	1,27E+00	-2,401430521	2,19E+00	1,39E-01	4,71E-01	Gm11284	2,41	No
ENSMUSG00000083320	4,39E-01	-1,205601484	7,97E-01	3,72E-01	6,73E-01	Gm13935	1,36	No
ENSMUSG00000083325	1,56E-02	5,717387006	5,38E-02	8,17E-01	9,69E-01	Gm14121	1,01	No
ENSMUSG00000083326	-2,48E-01	1,277360762	1,73E+00	1,89E-01	5,50E-01	Rpl38-ps1	0,84	No
ENSMUSG00000083328	-2,94E-01	4,646553083	1,40E+01	1,87E-04	4,11E-03	Gm11826	0,82	Yes
ENSMUSG00000083332	-2,15E-01	-1,683140051	1,24E-01	7,24E-01	9,18E-01	Gm7599	0,86	No
ENSMUSG00000083335	-1,87E+00	-3,107637761	1,39E+00	2,38E-01	5,50E-01	Gm13397	0,27	No
ENSMUSG00000083337	-3,50E-01	5,873565832	2,78E+01	1,34E-07	7,22E-06	Gm11539	0,78	Yes
ENSMUSG00000083338	-1,42E-01	1,7709682	8,20E-01	3,65E-01	6,66E-01	Gm12475	0,91	No
ENSMUSG00000083339	-1,06E+00	-1,146013957	5,12E+00	2,37E-02	1,69E-01	Gm11693	0,48	No
ENSMUSG00000083340	-1,36E+00	-2,832009022	1,17E+00	2,80E-01	5,77E-01	Gm13156	0,39	No
ENSMUSG00000083342	-7,36E-02	-2,44864231	6,20E-03	9,37E-01	1,00E+00	Gm14237	0,95	No
ENSMUSG00000083344	-1,05E+00	0,452162439	1,69E+01	4,04E-05	1,13E-03	Gm7363	0,48	Yes
ENSMUSG00000083353	-6,39E-02	-2,963280242	1,86E-03	9,66E-01	1,00E+00	Gm12540	0,96	No
ENSMUSG00000083356	-2,48E-01	0,266937334	8,25E-01	3,64E-01	6,65E-01	Gm11708	0,84	No
ENSMUSG00000083359	-3,83E-02	-3,107651936	-7,91E-11	1,00E+00	1,00E+00	Gm12656	0,97	No
ENSMUSG00000083368	1,87E+00	-3,107639691	1,38E+00	2,40E-01	5,50E-01	Gm13214	3,67	No
ENSMUSG00000083371	2,74E-02	-3,033621357	9,16E-12	1,00E+00	1,00E+00	Gm11422	1,02	No
ENSMUSG00000083372	3,36E-01	0,598591889	1,94E+00	1,64E-01	5,15E-01	Gm11235	1,26	No
ENSMUSG00000083373	-1,24E-01	2,815676865	1,08E+00	3,00E-01	5,96E-01	Gm13392	0,92	No
ENSMUSG00000083374	-1,53E-02	0,92747179	5,28E-03	9,42E-01	1,00E+00	Gm11449	0,99	No

ENSMUSG00000083377	-8,23E-02	-1,800976502	1,58E-02	9,00E-01	1,00E+00	Gm12653	0,94	No
ENSMUSG00000083378	-1,50E-01	4,758778384	3,79E+00	5,16E-02	2,77E-01	Gm13196	0,90	No
ENSMUSG00000083380	-4,33E-02	4,061047263	2,44E-01	6,21E-01	8,57E-01	Gm3244	0,97	No
ENSMUSG00000083382	4,29E-02	3,623267354	1,95E-01	6,59E-01	8,81E-01	Gm6433	1,03	No
ENSMUSG00000083390	1,85E+00	-3,107657832	1,35E+00	2,46E-01	5,50E-01	Gm13121	3,61	No
ENSMUSG00000083391	4,55E-01	-2,497578088	1,62E-01	6,88E-01	8,98E-01	Gm14148	1,37	No
ENSMUSG00000083394	-3,14E-01	4,237489334	1,37E+01	2,17E-04	4,64E-03	Gm11703	0,80	Yes
ENSMUSG00000083396	-1,57E-01	6,188145298	5,99E+00	1,44E-02	1,22E-01	Gm15542	0,90	No
ENSMUSG00000083397	-6,28E-01	-1,185381965	1,45E+00	2,28E-01	5,50E-01	Gm5466	0,65	No
ENSMUSG00000083404	-6,48E-02	-2,963249916	1,90E-03	9,65E-01	1,00E+00	Gm13019	0,96	No
ENSMUSG00000083405	-1,64E-01	7,906712078	7,89E+00	4,98E-03	5,58E-02	Gm15725	0,89	No
ENSMUSG00000083407	2,06E-01	1,295100813	1,23E+00	2,67E-01	5,63E-01	Gm13176	1,15	No
ENSMUSG00000083410	-1,98E-02	-3,033655952	2,83E-11	1,00E+00	1,00E+00	Gm14300	0,99	No
ENSMUSG00000083412	3,14E-02	-3,107617333	-7,69E-10	1,00E+00	1,00E+00	Gm12268	1,02	No
ENSMUSG00000083424	-2,33E-01	5,477943591	1,12E+01	8,22E-04	1,38E-02	Rpl35a-ps4	0,85	Yes
ENSMUSG00000083425	-1,87E+00	-3,107637761	1,39E+00	2,38E-01	5,50E-01	Gm13992	0,27	No
ENSMUSG00000083429	-1,73E+00	-2,655059731	2,08E+00	1,49E-01	4,90E-01	Gm15198	0,30	No
ENSMUSG00000083430	6,87E-01	-2,832001409	2,58E-01	6,12E-01	8,52E-01	Gm13006	1,61	No
ENSMUSG00000083431	-1,94E+00	-3,107619243	1,50E+00	2,21E-01	5,50E-01	Gm13437	0,26	No
ENSMUSG00000083435	-1,44E-01	-0,257493725	1,82E-01	6,70E-01	8,88E-01	Gm15824	0,91	No
ENSMUSG00000083441	-1,91E+00	-2,963238542	1,45E+00	2,29E-01	5,50E-01	Gm15329	0,27	No
ENSMUSG00000083443	1,64E-02	5,842925101	6,14E-02	8,04E-01	9,63E-01	Gm15519	1,01	No
ENSMUSG00000083449	-3,06E-01	2,993141864	7,32E+00	6,84E-03	7,05E-02	Gm13487	0,81	No
ENSMUSG00000083454	1,43E-02	2,641104655	1,34E-02	9,08E-01	1,00E+00	Gm13226	1,01	No
ENSMUSG00000083458	1,33E-02	5,787703654	3,96E-02	8,42E-01	9,85E-01	Gm5510	1,01	No
ENSMUSG00000083460	4,94E-01	-1,614179019	6,85E-01	4,08E-01	7,05E-01	Gm12182	1,41	No
ENSMUSG00000083461	6,30E-01	-2,44867571	4,42E-01	5,06E-01	7,91E-01	Gm12935	1,55	No
ENSMUSG00000083462	-2,21E-02	-0,668989512	3,37E-03	9,54E-01	1,00E+00	Gm12501	0,98	No
ENSMUSG00000083465	-2,93E-02	6,436357722	2,16E-01	6,42E-01	8,71E-01	Gm11652	0,98	No
ENSMUSG00000083471	1,87E+00	-3,107639691	1,38E+00	2,40E-01	5,50E-01	Gm12118	3,67	No
ENSMUSG00000083473	2,15E+00	-2,497667953	3,70E+00	5,43E-02	2,85E-01	Gm13207	4,44	No
ENSMUSG00000083477	-1,66E-01	5,504697749	5,85E+00	1,56E-02	1,29E-01	Gm5555	0,89	No

ENSMUSG00000083478	3,14E-02	-3,107617333	-7,69E-10	1,00E+00	1,00E+00	Gm15375	1,02	No
ENSMUSG00000083481	-7,93E-02	5,844361704	1,42E+00	2,34E-01	5,50E-01	Rps8-ps2	0,95	No
ENSMUSG00000083483	-1,46E-01	3,06271934	1,71E+00	1,91E-01	5,50E-01	Gm14044	0,90	No
ENSMUSG00000083495	3,14E-02	-3,107617333	-7,69E-10	1,00E+00	1,00E+00	Gm13877	1,02	No
ENSMUSG00000083496	-2,68E-01	5,358912	1,46E+01	1,30E-04	3,03E-03	Gm11263	0,83	Yes
ENSMUSG00000083500	-1,97E-01	1,342652854	1,10E+00	2,95E-01	5,90E-01	Gm15470	0,87	No
ENSMUSG00000083505	-7,18E-01	-2,600667221	5,76E-01	4,48E-01	7,38E-01	Gm7541	0,61	No
ENSMUSG00000083512	-2,19E-01	4,151899009	6,44E+00	1,12E-02	1,01E-01	Gm12749	0,86	No
ENSMUSG00000083522	-1,94E+00	-3,107619243	1,50E+00	2,21E-01	5,50E-01	Gm15321	0,26	No
ENSMUSG00000083528	1,87E+00	-3,107639691	1,38E+00	2,40E-01	5,50E-01	Gm13432	3,67	No
ENSMUSG00000083533	4,96E-02	2,204514362	1,26E-01	7,23E-01	9,17E-01	Gm13450	1,04	No
ENSMUSG00000083534	1,23E+00	-2,832030918	9,42E-01	3,32E-01	6,32E-01	H2-M6-ps	2,35	No
ENSMUSG00000083536	3,55E+00	-2,71167829	5,48E+00	1,93E-02	1,48E-01	Gm15808	11,72	No
ENSMUSG00000083538	5,34E-01	-1,770635851	8,13E-01	3,67E-01	6,68E-01	Gm16016	1,45	No
ENSMUSG00000083539	-1,94E+00	-3,107619243	1,50E+00	2,21E-01	5,50E-01	Gm12466	0,26	No
ENSMUSG00000083545	-6,47E-01	0,55176431	6,72E+00	9,52E-03	8,99E-02	Gm13320	0,64	No
ENSMUSG00000083546	3,14E-02	-3,107617333	-7,69E-10	1,00E+00	1,00E+00	Tpt1-ps1	1,02	No
ENSMUSG00000083547	-2,74E+00	-3,033596063	2,99E+00	8,38E-02	3,71E-01	Gm13321	0,15	No
ENSMUSG00000083549	-6,60E-02	-1,311231198	1,94E-02	8,89E-01	1,00E+00	Gm11227	0,96	No
ENSMUSG00000083553	-3,15E-01	-0,485199998	8,48E-01	3,57E-01	6,58E-01	Gm11989	0,80	No
ENSMUSG00000083555	1,88E+00	-2,896120282	1,36E+00	2,43E-01	5,50E-01	Gm12035	3,67	No
ENSMUSG00000083557	-2,90E-02	2,291967136	4,46E-02	8,33E-01	9,78E-01	Gm6877	0,98	No
ENSMUSG00000083562	-1,49E-01	-0,366907942	2,00E-01	6,55E-01	8,79E-01	Gm13234	0,90	No
ENSMUSG00000083563	-1,63E-01	6,907939806	7,12E+00	7,64E-03	7,65E-02	Gm13340	0,89	No
ENSMUSG00000083567	2,15E-01	1,313199825	1,30E+00	2,55E-01	5,50E-01	Gm11451	1,16	No
ENSMUSG00000083569	-5,70E-01	-2,497563617	2,55E-01	6,14E-01	8,53E-01	Gm13666	0,67	No
ENSMUSG00000083579	8,55E-01	-2,268067974	8,80E-01	3,48E-01	6,49E-01	Gm15538	1,81	No
ENSMUSG00000083580	-1,94E+00	-3,107619243	1,50E+00	2,21E-01	5,50E-01	Rpsa-ps3	0,26	No
ENSMUSG00000083582	-1,87E+00	-3,03364178	1,41E+00	2,36E-01	5,50E-01	Gm15393	0,27	No
ENSMUSG00000083594	1,87E+00	-3,107639691	1,38E+00	2,40E-01	5,50E-01	Gm13722	3,67	No
ENSMUSG00000083599	-1,36E-01	2,704494881	1,23E+00	2,66E-01	5,63E-01	Gm12821	0,91	No
ENSMUSG00000083606	-1,89E+00	-3,1076441	1,42E+00	2,33E-01	5,50E-01	Gm15916	0,27	No

ENSMUSG00000083609	-2,72E+00	-2,963211345	2,94E+00	8,63E-02	3,78E-01	Gm16007	0,15	No
ENSMUSG00000083610	-2,36E-02	2,786695898	3,95E-02	8,43E-01	9,85E-01	Oaz2-ps	0,98	No
ENSMUSG00000083614	-8,03E-01	-2,896132011	3,52E-01	5,53E-01	8,19E-01	Gm12795	0,57	No
ENSMUSG00000083618	-6,69E-02	-2,770615001	3,03E-03	9,56E-01	1,00E+00	Gm11771	0,95	No
ENSMUSG00000083619	-5,83E-02	5,710702484	7,50E-01	3,86E-01	6,87E-01	Gm14414	0,96	No
ENSMUSG00000083620	2,62E+00	-2,963293967	2,65E+00	1,04E-01	3,95E-01	Gm15529	6,13	No
ENSMUSG00000083622	-1,95E+00	-3,033628749	1,50E+00	2,21E-01	5,50E-01	Gm15570	0,26	No
ENSMUSG00000083623	1,05E-01	-0,268271746	1,00E-01	7,52E-01	9,32E-01	Gm7224	1,08	No
ENSMUSG00000083624	-1,94E+00	-3,107619243	1,50E+00	2,21E-01	5,50E-01	Gm12469	0,26	No
ENSMUSG00000083626	-2,23E-02	4,545918203	7,84E-02	7,79E-01	9,48E-01	Gm4459	0,98	No
ENSMUSG00000083627	-2,79E-01	0,090305452	9,22E-01	3,37E-01	6,38E-01	Gm12502	0,82	No
ENSMUSG00000083630	1,01E+00	-1,929354628	2,01E+00	1,56E-01	5,01E-01	Gm15602	2,02	No
ENSMUSG00000083642	2,57E-02	-1,355539046	2,40E-03	9,61E-01	1,00E+00	Gm13148	1,02	No
ENSMUSG00000083645	-1,14E-02	-3,033635021	1,50E-05	9,97E-01	1,00E+00	Gm14086	0,99	No
ENSMUSG00000083649	2,97E-01	-1,401419586	2,56E-01	6,13E-01	8,53E-01	Rasl2-9	1,23	No
ENSMUSG00000083650	8,41E-01	-1,268258252	2,67E+00	1,02E-01	3,94E-01	Gm13357	1,79	No
ENSMUSG00000083655	-1,35E-01	-0,175495765	1,88E-01	6,64E-01	8,84E-01	Gm12099	0,91	No
ENSMUSG00000083658	-8,06E-01	-1,80085729	1,55E+00	2,14E-01	5,50E-01	Gm15798	0,57	No
ENSMUSG00000083661	1,61E+00	-1,832024679	6,20E+00	1,28E-02	1,12E-01	Gm11473	3,06	No
ENSMUSG00000083664	-1,89E+00	-3,1076441	1,42E+00	2,33E-01	5,50E-01	Gm11433	0,27	No
ENSMUSG00000083669	2,91E-01	5,137651214	1,59E+01	6,77E-05	1,75E-03	Gm10169	1,22	Yes
ENSMUSG00000083671	7,31E-01	-2,963267542	2,91E-01	5,90E-01	8,41E-01	Gm14304	1,66	No
ENSMUSG00000083672	1,16E-01	0,028123767	1,59E-01	6,90E-01	8,99E-01	Kpna2-ps	1,08	No
ENSMUSG00000083674	2,72E+00	-2,107718311	6,99E+00	8,21E-03	8,06E-02	Zfp133-ps	6,57	No
ENSMUSG00000083677	-4,20E-01	0,337651586	2,40E+00	1,21E-01	4,34E-01	Gm11699	0,75	No
ENSMUSG00000083678	5,88E-03	4,961395527	6,19E-03	9,37E-01	1,00E+00	Gm12989	1,00	No
ENSMUSG00000083679	9,08E-02	5,758075434	1,83E+00	1,77E-01	5,36E-01	Gm12892	1,06	No
ENSMUSG00000083681	1,65E-01	3,761870385	3,03E+00	8,15E-02	3,66E-01	Gm6640	1,12	No
ENSMUSG00000083688	-2,28E-01	3,265315172	4,61E+00	3,17E-02	2,06E-01	Gm13743	0,85	No
ENSMUSG00000083692	-5,80E-02	5,241568547	6,53E-01	4,19E-01	7,14E-01	Gm9575	0,96	No
ENSMUSG00000083693	6,93E-02	-1,770581457	1,40E-02	9,06E-01	1,00E+00	Gm11971	1,05	No
ENSMUSG00000083700	-3,16E-01	3,025591586	7,63E+00	5,75E-03	6,20E-02	Gm2986	0,80	No

ENSMUSG00000083704	-6,48E-02	-2,963249916	1,90E-03	9,65E-01	1,00E+00	Gm13153	0,96	No
ENSMUSG00000083705	-6,95E-01	-0,257583363	4,98E+00	2,56E-02	1,78E-01	Gm8624	0,62	No
ENSMUSG00000083708	6,35E-02	2,754784304	2,93E-01	5,88E-01	8,40E-01	Gm13123	1,05	No
ENSMUSG00000083713	5,67E-01	-1,770634733	7,31E-01	3,93E-01	6,93E-01	Gm5883	1,48	No
ENSMUSG00000083716	-1,26E-01	7,890620539	4,62E+00	3,16E-02	2,06E-01	Gm13436	0,92	No
ENSMUSG00000083717	1,87E+00	-3,107639691	1,38E+00	2,40E-01	5,50E-01	Gm13865	3,67	No
ENSMUSG00000083718	-5,87E-01	-0,648002664	2,39E+00	1,22E-01	4,36E-01	Ccnb2-ps	0,67	No
ENSMUSG00000083723	-1,34E+00	-2,896111155	1,13E+00	2,89E-01	5,84E-01	Rpl23a-ps14	0,39	No
ENSMUSG00000083725	-2,10E-01	1,104260657	1,08E+00	2,99E-01	5,95E-01	Gm11865	0,86	No
ENSMUSG00000083728	-9,36E-01	-2,655064798	7,68E-01	3,81E-01	6,82E-01	Gm13854	0,52	No
ENSMUSG00000083731	-3,90E-02	-2,655097813	1,36E-03	9,71E-01	1,00E+00	Gm16202	0,97	No
ENSMUSG00000083732	3,14E+00	-2,89616069	4,02E+00	4,51E-02	2,53E-01	Gm14197	8,80	No
ENSMUSG00000083733	-1,87E+00	-3,107637761	1,39E+00	2,38E-01	5,50E-01	Gm13035	0,27	No
ENSMUSG00000083734	1,30E-02	1,389617016	4,87E-03	9,44E-01	1,00E+00	Gm16209	1,01	No
ENSMUSG00000083737	1,30E-01	0,638807893	2,94E-01	5,88E-01	8,40E-01	Prdx6-ps2	1,09	No
ENSMUSG00000083745	-1,33E+00	-2,770602484	1,11E+00	2,92E-01	5,87E-01	Gm13543	0,40	No
ENSMUSG00000083748	-2,63E-02	-3,033653157	2,33E-04	9,88E-01	1,00E+00	Gm11662	0,98	No
ENSMUSG00000083750	-1,94E+00	-3,107619243	1,50E+00	2,21E-01	5,50E-01	Gm14108	0,26	No
ENSMUSG00000083757	-3,22E-01	5,81596205	2,34E+01	1,32E-06	5,69E-05	Rps15a-ps4	0,80	Yes
ENSMUSG00000083761	4,52E-02	1,661363317	7,09E-02	7,90E-01	9,54E-01	Pgam1-ps1	1,03	No
ENSMUSG00000083764	-5,98E-02	3,834616979	4,19E-01	5,17E-01	7,98E-01	Gm11516	0,96	No
ENSMUSG00000083767	-7,31E-02	4,477102719	8,18E-01	3,66E-01	6,67E-01	Gm11405	0,95	No
ENSMUSG00000083771	-1,94E+00	-3,107619243	1,50E+00	2,21E-01	5,50E-01	Gm15988	0,26	No
ENSMUSG00000083773	8,36E-02	7,932143613	2,05E+00	1,52E-01	4,94E-01	Gm13394	1,06	No
ENSMUSG00000083774	-9,97E-02	4,13159775	1,31E+00	2,52E-01	5,50E-01	Gm7180	0,93	No
ENSMUSG00000083777	2,75E-01	0,412288815	1,09E+00	2,96E-01	5,92E-01	Gm13361	1,21	No
ENSMUSG00000083780	4,59E-01	-2,355538726	2,85E-01	5,93E-01	8,42E-01	Gm12728	1,37	No
ENSMUSG00000083783	-1,89E+00	-3,1076441	1,42E+00	2,33E-01	5,50E-01	Gm12739	0,27	No
ENSMUSG00000083791	-5,88E-02	-3,033652696	1,62E-03	9,68E-01	1,00E+00	Gm12721	0,96	No
ENSMUSG00000083793	-2,69E-01	2,381510991	3,89E+00	4,85E-02	2,65E-01	Gm14274	0,83	No
ENSMUSG00000083796	-1,86E-01	-0,107764525	3,17E-01	5,74E-01	8,33E-01	Gm13369	0,88	No
ENSMUSG00000083803	1,83E+00	-2,963282593	1,33E+00	2,50E-01	5,50E-01	Gm12455	3,56	No

ENSMUSG00000083804	9,34E-03	1,66959463	3,20E-03	9,55E-01	1,00E+00	Gm13676	1,01	No
ENSMUSG00000083808	1,82E+00	-3,033667329	1,31E+00	2,52E-01	5,50E-01	Gm3828	3,54	No
ENSMUSG00000083811	5,61E-02	-0,997984498	1,77E-02	8,94E-01	1,00E+00	Gm13071	1,04	No
ENSMUSG00000083812	1,81E+00	-3,10765737	1,28E+00	2,58E-01	5,50E-01	Gm5054	3,49	No
ENSMUSG00000083813	2,96E-02	3,348437674	8,12E-02	7,76E-01	9,45E-01	Gm15502	1,02	No
ENSMUSG00000083815	8,41E-01	-0,333269527	4,93E+00	2,64E-02	1,82E-01	Gm13435	1,79	No
ENSMUSG00000083816	1,60E-01	1,001929518	6,04E-01	4,37E-01	7,30E-01	Gm13033	1,12	No
ENSMUSG00000083817	1,07E+00	-2,497667382	1,46E+00	2,27E-01	5,50E-01	Gm14400	2,09	No
ENSMUSG00000083819	4,31E-02	2,962077829	1,50E-01	6,98E-01	9,04E-01	Gm11977	1,03	No
ENSMUSG00000083820	3,57E-02	3,659978603	1,38E-01	7,11E-01	9,10E-01	Gm6415	1,03	No
ENSMUSG00000083822	-9,68E-02	1,749171924	3,66E-01	5,45E-01	8,13E-01	Hmgb1-ps5	0,94	No
ENSMUSG00000083826	-1,37E-01	-1,051773313	1,04E-01	7,48E-01	9,30E-01	Gm13039	0,91	No
ENSMUSG00000083829	1,13E-02	5,800510173	2,87E-02	8,65E-01	9,98E-01	Gm2199	1,01	No
ENSMUSG00000083832	-1,13E-01	4,131357841	1,68E+00	1,95E-01	5,50E-01	Gm13771	0,92	No
ENSMUSG00000083833	-1,40E-01	4,854839257	3,41E+00	6,48E-02	3,19E-01	Gm13841	0,91	No
ENSMUSG00000083834	-2,93E-01	1,306664717	2,60E+00	1,07E-01	4,00E-01	Gm12577	0,82	No
ENSMUSG00000083836	2,04E-01	6,733627354	1,09E+01	9,64E-04	1,57E-02	Ldha-ps2	1,15	Yes
ENSMUSG00000083837	-1,89E+00	-3,1076441	1,42E+00	2,33E-01	5,50E-01	Gm14427	0,27	No
ENSMUSG00000083838	-3,84E-01	-1,146137983	6,87E-01	4,07E-01	7,04E-01	Gm11957	0,77	No
ENSMUSG00000083842	-2,73E-02	-2,963256629	3,40E-04	9,85E-01	1,00E+00	Gm12485	0,98	No
ENSMUSG00000083844	8,83E-03	3,727744884	8,79E-03	9,25E-01	1,00E+00	Ube2d-ps	1,01	No
ENSMUSG00000083846	7,43E-01	-2,963261206	3,01E-01	5,83E-01	8,38E-01	Gm13797	1,67	No
ENSMUSG00000083849	-3,74E-02	1,894460778	5,98E-02	8,07E-01	9,64E-01	Gm13477	0,97	No
ENSMUSG00000083852	-9,42E-02	-0,205576986	9,51E-02	7,58E-01	9,35E-01	Gm13007	0,94	No
ENSMUSG00000083853	9,99E-01	-0,535482685	5,99E+00	1,44E-02	1,22E-01	Gm15696	2,00	No
ENSMUSG00000083854	1,20E-01	4,078060524	1,89E+00	1,69E-01	5,23E-01	Dnajc19-ps	1,09	No
ENSMUSG00000083856	3,13E-02	3,57261134	1,03E-01	7,48E-01	9,30E-01	Gm13886	1,02	No
ENSMUSG00000083859	-9,06E-01	-2,088751681	1,75E+00	1,86E-01	5,50E-01	Gm12003	0,53	No
ENSMUSG00000083860	-4,30E-01	0,045261428	2,08E+00	1,49E-01	4,90E-01	Gm13313	0,74	No
ENSMUSG00000083863	3,23E-02	6,240918287	2,54E-01	6,15E-01	8,53E-01	Gm13341	1,02	No
ENSMUSG00000083864	-1,76E-01	2,476008095	1,86E+00	1,72E-01	5,29E-01	Gm13719	0,89	No
ENSMUSG00000083870	1,81E+00	-3,10765737	1,28E+00	2,58E-01	5,50E-01	Gm11761	3,49	No

ENSMUSG00000083873	1,81E+00	-3,10765737	1,28E+00	2,58E-01	5,50E-01	Vmn1r-ps92	3,49	No
ENSMUSG00000083874	-3,83E-02	-3,107651936	-7,91E-11	1,00E+00	1,00E+00	Gm7494	0,97	No
ENSMUSG00000083875	7,22E-01	-2,711718047	2,84E-01	5,94E-01	8,43E-01	Gm13831	1,65	No
ENSMUSG00000083876	-8,91E-01	-2,896142986	4,37E-01	5,09E-01	7,92E-01	Gm11439	0,54	No
ENSMUSG00000083878	-2,62E-01	0,112246236	8,28E-01	3,63E-01	6,64E-01	Gm12034	0,83	No
ENSMUSG00000083879	-9,04E-02	2,815627751	5,91E-01	4,42E-01	7,34E-01	Gm14038	0,94	No
ENSMUSG00000083885	2,87E-01	-2,711757995	8,33E-02	7,73E-01	9,44E-01	Gm13213	1,22	No
ENSMUSG00000083888	-6,96E-01	-0,587164195	3,21E+00	7,33E-02	3,44E-01	Gm12441	0,62	No
ENSMUSG00000083890	-2,69E-02	4,151095652	9,79E-02	7,54E-01	9,34E-01	Gm15703	0,98	No
ENSMUSG00000083899	-8,13E-03	7,177781774	1,83E-02	8,92E-01	1,00E+00	Gm12346	0,99	No
ENSMUSG00000083900	-7,58E-01	0,049732045	6,38E+00	1,15E-02	1,03E-01	Gm13294	0,59	No
ENSMUSG00000083902	-5,58E-02	6,458503258	7,85E-01	3,76E-01	6,76E-01	Gm15975	0,96	No
ENSMUSG00000083903	-2,66E-01	-1,146059985	2,64E-01	6,07E-01	8,50E-01	Gm5405	0,83	No
ENSMUSG00000083907	1,05E-01	3,293026656	9,96E-01	3,18E-01	6,19E-01	Plk-ps1	1,08	No
ENSMUSG00000083909	-1,77E+00	-1,627481468	7,54E+00	6,05E-03	6,46E-02	Gm15842	0,29	No
ENSMUSG00000083910	-1,87E+00	-3,107637761	1,39E+00	2,38E-01	5,50E-01	Gm13604	0,27	No
ENSMUSG00000083911	-4,54E-01	2,058853587	9,59E+00	1,95E-03	2,75E-02	Gm4342	0,73	Yes
ENSMUSG00000083914	-2,40E-01	2,770115352	4,13E+00	4,21E-02	2,44E-01	Rps18-ps1	0,85	No
ENSMUSG00000083915	-1,87E+00	-3,107637761	1,39E+00	2,38E-01	5,50E-01	Gm13932	0,27	No
ENSMUSG00000083921	1,89E+00	-2,96320077	1,38E+00	2,40E-01	5,50E-01	Gm15750	3,71	No
ENSMUSG00000083922	-1,91E+00	-2,963238542	1,45E+00	2,29E-01	5,50E-01	Gm3835	0,27	No
ENSMUSG00000083929	-1,14E-02	-3,033635021	1,50E-05	9,97E-01	1,00E+00	Gm10600	0,99	No
ENSMUSG00000083930	-5,41E-02	2,84658814	2,13E-01	6,45E-01	8,73E-01	Gm13042	0,96	No
ENSMUSG00000083937	-3,08E-02	4,499091896	1,47E-01	7,01E-01	9,05E-01	Cct3-ps1	0,98	No
ENSMUSG00000083943	2,78E-01	-1,14604531	3,85E-01	5,35E-01	8,07E-01	Gm11483	1,21	No
ENSMUSG00000083951	-8,48E-01	-2,832023561	3,94E-01	5,30E-01	8,05E-01	Gm13786	0,56	No
ENSMUSG00000083953	-1,83E-01	0,15990649	4,68E-01	4,94E-01	7,79E-01	Gm14049	0,88	No
ENSMUSG00000083956	-1,87E+00	-3,107637761	1,39E+00	2,38E-01	5,50E-01	Gm12361	0,27	No
ENSMUSG00000083961	1,19E+00	-3,070179505	6,63E-01	4,15E-01	7,11E-01	Gm13725	2,28	No
ENSMUSG00000083962	1,18E-01	-0,015438767	1,33E-01	7,15E-01	9,12E-01	Gm15707	1,09	No
ENSMUSG00000083964	-1,42E-01	-0,175594986	2,06E-01	6,50E-01	8,76E-01	Gm14122	0,91	No
ENSMUSG00000083974	1,90E+00	-2,10768527	4,40E+00	3,60E-02	2,25E-01	Gm16053	3,74	No

ENSMUSG00000083976	1,13E+00	-2,49756505	1,22E+00	2,69E-01	5,66E-01	Gm13664	2,19	No
ENSMUSG00000083979	-5,93E-02	-2,627659749	4,00E-03	9,50E-01	1,00E+00	Gm12527	0,96	No
ENSMUSG00000083988	9,78E-01	-2,627679712	8,72E-01	3,50E-01	6,52E-01	Rps10-ps4	1,97	No
ENSMUSG00000083989	2,61E+00	-3,033672762	2,63E+00	1,05E-01	3,97E-01	Gm12607	6,10	No
ENSMUSG00000083990	2,08E-01	-0,793508532	2,62E-01	6,09E-01	8,51E-01	Gm12101	1,16	No
ENSMUSG00000083992	-1,68E-01	9,214894371	8,81E+00	2,99E-03	3,82E-02	Gm11478	0,89	Yes
ENSMUSG00000083993	3,08E-01	1,385466581	2,75E+00	9,74E-02	3,90E-01	Gm14398	1,24	No
ENSMUSG00000083997	3,14E-02	-3,107617333	-7,69E-10	1,00E+00	1,00E+00	Gm12920	1,02	No
ENSMUSG00000083998	5,05E-01	-1,574111224	6,41E-01	4,23E-01	7,18E-01	Prdx2-rs3	1,42	No
ENSMUSG00000084004	2,91E-03	-1,051725935	4,04E-05	9,95E-01	1,00E+00	Gm12047	1,00	No
ENSMUSG00000084006	8,43E-02	2,304431911	3,70E-01	5,43E-01	8,12E-01	Gm14245	1,06	No
ENSMUSG00000084010	-1,87E+00	-3,107637761	1,39E+00	2,38E-01	5,50E-01	Gm13302	0,27	No
ENSMUSG00000084011	-1,06E-01	-3,033634182	5,86E-03	9,39E-01	1,00E+00	Gm12969	0,93	No
ENSMUSG00000084012	-3,21E+00	-2,929285214	4,33E+00	3,73E-02	2,30E-01	Gm11956	0,11	No
ENSMUSG00000084013	-1,39E-02	5,036239133	3,56E-02	8,50E-01	9,90E-01	Gm14270	0,99	No
ENSMUSG00000084017	-8,14E-02	-2,355575467	7,55E-03	9,31E-01	1,00E+00	Rpl21-ps9	0,95	No
ENSMUSG00000084018	1,09E-02	-1,268103785	4,88E-04	9,82E-01	1,00E+00	Gm11895	1,01	No
ENSMUSG00000084020	1,08E-01	4,081987968	1,50E+00	2,20E-01	5,50E-01	Wsb2-ps	1,08	No
ENSMUSG00000084021	-1,94E+00	-3,107619243	1,50E+00	2,21E-01	5,50E-01	Gm5832	0,26	No
ENSMUSG00000084024	-3,14E-01	-2,268037501	1,47E-01	7,01E-01	9,05E-01	Gm15937	0,80	No
ENSMUSG00000084028	-4,98E-02	-2,401357275	3,41E-03	9,53E-01	1,00E+00	Gm13983	0,97	No
ENSMUSG00000084032	1,82E+00	-3,033661385	1,31E+00	2,52E-01	5,50E-01	Gm12991	3,54	No
ENSMUSG00000084033	-2,20E-01	-0,36674307	4,16E-01	5,19E-01	7,98E-01	Gm7645	0,86	No
ENSMUSG00000084038	6,24E-01	0,483345	6,05E+00	1,39E-02	1,19E-01	Gm11295	1,54	No
ENSMUSG00000084048	9,01E-03	-3,107646452	-5,16E-10	1,00E+00	1,00E+00	Gm12042	1,01	No
ENSMUSG00000084050	-5,07E-02	-2,655102469	2,32E-03	9,62E-01	1,00E+00	Gm14439	0,97	No
ENSMUSG00000084055	2,74E-02	0,229541946	1,00E-02	9,20E-01	1,00E+00	Gm12004	1,02	No
ENSMUSG00000084064	-1,48E-01	1,627342877	7,78E-01	3,78E-01	6,79E-01	Gm12079	0,90	No
ENSMUSG00000084067	-1,32E-01	4,788213194	2,98E+00	8,45E-02	3,73E-01	Gm14269	0,91	No
ENSMUSG00000084071	-3,68E-02	1,323943446	3,91E-02	8,43E-01	9,85E-01	Gm13378	0,97	No
ENSMUSG00000084072	-6,61E-01	-3,070161976	1,72E-01	6,78E-01	8,92E-01	Gm11285	0,63	No
ENSMUSG00000084077	-5,10E-01	-1,051767494	1,42E+00	2,34E-01	5,50E-01	Gm11952	0,70	No

ENSMUSG00000084081	-2,00E-01	0,990949832	9,23E-01	3,37E-01	6,37E-01	Gm12057	0,87	No
ENSMUSG00000084083	-1,34E-01	1,633518761	6,32E-01	4,27E-01	7,21E-01	Gm15782	0,91	No
ENSMUSG00000084085	-1,22E+00	-3,146113954	6,96E-01	4,04E-01	7,01E-01	Gm16140	0,43	No
ENSMUSG00000084087	1,34E-01	-2,146155983	3,11E-02	8,60E-01	9,95E-01	Gm13650	1,10	No
ENSMUSG00000084088	1,25E+00	-2,311241476	2,14E+00	1,43E-01	4,79E-01	Gm12941	2,39	No
ENSMUSG00000084093	3,77E-02	4,801568501	2,42E-01	6,23E-01	8,58E-01	Gm16418	1,03	No
ENSMUSG00000084094	-1,73E-01	3,352040262	2,94E+00	8,66E-02	3,78E-01	Gm13961	0,89	No
ENSMUSG00000084096	1,87E+00	-3,107639691	1,38E+00	2,40E-01	5,50E-01	Vmn1r-ps135	3,67	No
ENSMUSG00000084098	5,32E-02	5,902640224	6,43E-01	4,23E-01	7,18E-01	Gm13422	1,04	No
ENSMUSG00000084099	-2,83E-03	3,873257948	9,47E-04	9,75E-01	1,00E+00	Gm12929	1,00	No
ENSMUSG00000084104	7,07E-02	5,24602703	9,87E-01	3,21E-01	6,21E-01	Gm13578	1,05	No
ENSMUSG00000084105	-1,50E+00	-2,497536401	2,34E+00	1,26E-01	4,45E-01	Gm13252	0,35	No
ENSMUSG00000084106	-1,63E-01	8,49719109	8,09E+00	4,45E-03	5,11E-02	Gm6136	0,89	No
ENSMUSG00000084107	-1,14E-02	-3,033635021	1,50E-05	9,97E-01	1,00E+00	Gm13700	0,99	No
ENSMUSG00000084108	9,01E-03	-3,107646452	-5,16E-10	1,00E+00	1,00E+00	Gm15794	1,01	No
ENSMUSG00000084111	-1,10E-01	8,019168419	3,59E+00	5,81E-02	2,97E-01	Gm15710	0,93	No
ENSMUSG00000084113	9,85E-02	4,128189509	1,30E+00	2,55E-01	5,50E-01	Gm14277	1,07	No
ENSMUSG00000084115	-1,19E-01	0,358647293	2,17E-01	6,41E-01	8,71E-01	Gm13566	0,92	No
ENSMUSG00000084116	-1,89E+00	-3,033648118	1,42E+00	2,33E-01	5,50E-01	Gm12460	0,27	No
ENSMUSG00000084120	-7,52E-01	-2,497604706	6,34E-01	4,26E-01	7,20E-01	Gm11624	0,59	No
ENSMUSG00000084124	-1,23E+00	-2,401383818	1,46E+00	2,27E-01	5,50E-01	Gm13588	0,43	No
ENSMUSG00000084127	2,85E-02	3,401675957	7,49E-02	7,84E-01	9,51E-01	Gm13169	1,02	No
ENSMUSG00000084128	1,73E-01	1,21028736	8,01E-01	3,71E-01	6,72E-01	Esrp2	1,13	No
ENSMUSG00000084129	-1,84E-02	1,896103478	1,37E-02	9,07E-01	1,00E+00	Hmgb1-ps1	0,99	No
ENSMUSG00000084132	7,39E-01	-2,832032194	2,97E-01	5,86E-01	8,39E-01	Gm15982	1,67	No
ENSMUSG00000084133	1,66E-01	3,864064044	3,26E+00	7,09E-02	3,37E-01	Gm12856	1,12	No
ENSMUSG00000084139	-4,61E-01	-1,497521388	5,66E-01	4,52E-01	7,42E-01	Gm12322	0,73	No
ENSMUSG00000084140	1,11E+00	-2,448699565	1,16E+00	2,81E-01	5,77E-01	Gm14000	2,15	No
ENSMUSG00000084143	-2,88E-02	-2,963268425	3,79E-04	9,84E-01	1,00E+00	Gm13400	0,98	No
ENSMUSG00000084144	3,14E-02	-3,107617333	-7,69E-10	1,00E+00	1,00E+00	Gm12516	1,02	No
ENSMUSG00000084145	-1,49E-01	4,691732664	3,53E+00	6,02E-02	3,04E-01	Gm12263	0,90	No
ENSMUSG00000084146	8,02E-01	-2,711742231	5,59E-01	4,55E-01	7,44E-01	Gm12104	1,74	No

ENSMUSG00000084148	-1,91E+00	-1,18541345	1,24E+01	4,23E-04	7,98E-03	Gm12380	0,27	Yes
ENSMUSG00000084149	-5,85E-02	3,163210269	2,96E-01	5,86E-01	8,39E-01	Gm12944	0,96	No
ENSMUSG00000084153	3,47E-02	5,057898848	2,25E-01	6,35E-01	8,67E-01	Gm12186	1,02	No
ENSMUSG00000084154	1,24E+00	-2,655167738	9,53E-01	3,29E-01	6,30E-01	Gm15644	2,36	No
ENSMUSG00000084159	-1,12E-01	6,207647562	3,04E+00	8,13E-02	3,66E-01	Gm12696	0,93	No
ENSMUSG00000084164	-8,10E-01	-2,963247942	3,56E-01	5,51E-01	8,17E-01	Gm13902	0,57	No
ENSMUSG00000084168	6,94E-03	3,115551948	4,04E-03	9,49E-01	1,00E+00	Atp5l2-ps	1,00	No
ENSMUSG00000084169	-7,49E-02	-0,963363233	2,63E-02	8,71E-01	1,00E+00	Gm12240	0,95	No
ENSMUSG00000084170	-1,12E+00	-1,226261516	4,96E+00	2,60E-02	1,80E-01	Gm11954	0,46	No
ENSMUSG00000084175	-1,89E+00	-3,033648118	1,42E+00	2,33E-01	5,50E-01	Gm11372	0,27	No
ENSMUSG00000084178	6,10E-01	-2,600723232	4,15E-01	5,20E-01	7,99E-01	Gm11759	1,53	No
ENSMUSG00000084183	-1,41E-01	2,954498898	1,55E+00	2,14E-01	5,50E-01	Gm12009	0,91	No
ENSMUSG00000084190	1,63E+00	-2,600723278	1,82E+00	1,77E-01	5,36E-01	Gm11869	3,10	No
ENSMUSG00000084192	-7,88E-02	-2,89616692	4,36E-03	9,47E-01	1,00E+00	Gm13282	0,95	No
ENSMUSG00000084195	-1,36E+00	-2,355547885	1,16E+00	2,82E-01	5,78E-01	Gm15372	0,39	No
ENSMUSG00000084198	2,67E+00	-3,033636951	2,76E+00	9,68E-02	3,88E-01	Gm13711	6,36	No
ENSMUSG00000084199	-2,72E+00	-2,711756211	2,92E+00	8,75E-02	3,78E-01	Gm12422	0,15	No
ENSMUSG00000084201	3,22E-01	-1,424975684	3,61E-01	5,48E-01	8,15E-01	Gm11926	1,25	No
ENSMUSG00000084203	-2,23E-01	-2,070191662	1,10E-01	7,41E-01	9,27E-01	Gm15593	0,86	No
ENSMUSG00000084207	-5,23E-02	-2,963267963	1,24E-03	9,72E-01	1,00E+00	Gm14070	0,96	No
ENSMUSG00000084208	1,53E-01	2,175754774	1,13E+00	2,88E-01	5,84E-01	Gm12792	1,11	No
ENSMUSG00000084213	-9,52E-01	-2,548223578	7,94E-01	3,73E-01	6,74E-01	Hspe1-ps5	0,52	No
ENSMUSG00000084215	1,94E-01	4,070628025	4,93E+00	2,65E-02	1,82E-01	Gm11814	1,14	No
ENSMUSG00000084217	-7,38E-02	-3,033634644	2,66E-03	9,59E-01	1,00E+00	Gm15488	0,95	No
ENSMUSG00000084218	9,01E-03	-3,107646452	-5,16E-10	1,00E+00	1,00E+00	Gm8145	1,01	No
ENSMUSG00000084220	2,70E-01	1,512962338	2,44E+00	1,18E-01	4,27E-01	Gm12387	1,21	No
ENSMUSG00000084221	6,07E-02	1,162003475	1,00E-01	7,52E-01	9,32E-01	Hmgb1-ps4	1,04	No
ENSMUSG00000084223	-9,27E-02	1,350373559	2,47E-01	6,19E-01	8,56E-01	Gm12244	0,94	No
ENSMUSG00000084224	-2,56E-01	-1,051732014	3,43E-01	5,58E-01	8,22E-01	Gm12337	0,84	No
ENSMUSG00000084230	4,14E-02	3,154507375	1,48E-01	7,00E-01	9,05E-01	Gm14388	1,03	No
ENSMUSG00000084231	-1,75E-01	-1,655191672	9,60E-02	7,57E-01	9,35E-01	Gm11975	0,89	No
ENSMUSG00000084235	-2,78E-01	5,999167685	1,80E+01	2,21E-05	6,80E-04	Gm15421	0,82	Yes

ENSMUSG00000084241	-2,62E-02	3,187419795	5,85E-02	8,09E-01	9,65E-01	Gm13416	0,98	No
ENSMUSG00000084243	7,44E-01	-2,896157547	3,02E-01	5,83E-01	8,38E-01	Gm13784	1,68	No
ENSMUSG00000084244	1,82E+00	-3,033667329	1,31E+00	2,52E-01	5,50E-01	Gm14020	3,54	No
ENSMUSG00000084249	1,22E+00	-2,896168849	9,19E-01	3,38E-01	6,38E-01	Gm12267	2,33	No
ENSMUSG00000084252	-6,33E-01	-1,587440054	1,13E+00	2,89E-01	5,84E-01	Gm12036	0,64	No
ENSMUSG00000084259	-2,04E-02	-0,574138816	2,97E-03	9,57E-01	1,00E+00	Gm12040	0,99	No
ENSMUSG00000084260	-1,54E-02	6,800026868	6,27E-02	8,02E-01	9,62E-01	Gm12844	0,99	No
ENSMUSG00000084264	1,12E+00	-2,655146038	1,19E+00	2,76E-01	5,73E-01	Gm14142	2,17	No
ENSMUSG00000084274	-3,19E-01	-0,145980179	9,75E-01	3,24E-01	6,24E-01	Gm12504	0,80	No
ENSMUSG00000084276	-1,86E+00	-2,963227946	1,40E+00	2,36E-01	5,50E-01	Gm8317	0,28	No
ENSMUSG00000084277	7,94E-02	0,324041876	8,24E-02	7,74E-01	9,45E-01	Gm11702	1,06	No
ENSMUSG00000084278	1,81E+00	-3,10765737	1,28E+00	2,58E-01	5,50E-01	Gm12189	3,49	No
ENSMUSG00000084279	8,02E-02	3,188770122	5,51E-01	4,58E-01	7,47E-01	Gm13232	1,06	No
ENSMUSG00000084288	9,01E-03	-3,107646452	-5,16E-10	1,00E+00	1,00E+00	Gm5228	1,01	No
ENSMUSG00000084291	-2,48E-01	3,42469786	6,04E+00	1,40E-02	1,20E-01	Gm6654	0,84	No
ENSMUSG00000084292	-1,61E-02	-2,448591388	3,00E-04	9,86E-01	1,00E+00	Gm13535	0,99	No
ENSMUSG00000084296	-1,35E+00	-2,89615366	1,14E+00	2,85E-01	5,82E-01	Scgb2b25-ps	0,39	No
ENSMUSG00000084301	1,66E-01	3,256545644	2,50E+00	1,14E-01	4,16E-01	Gm7856	1,12	No
ENSMUSG00000084302	-4,17E-02	-2,963202678	7,92E-04	9,78E-01	1,00E+00	Gm12194	0,97	No
ENSMUSG00000084304	-1,51E-01	5,351773994	4,63E+00	3,15E-02	2,06E-01	Gm6142	0,90	No
ENSMUSG00000084306	-1,37E+00	-2,711706704	1,17E+00	2,79E-01	5,75E-01	Gm11743	0,39	No
ENSMUSG00000084307	5,42E-02	0,010503821	3,11E-02	8,60E-01	9,95E-01	Gm12151	1,04	No
ENSMUSG00000084313	1,17E+00	-3,14612376	6,40E-01	4,24E-01	7,18E-01	Gm13809	2,26	No
ENSMUSG00000084314	-2,95E-01	4,121344811	1,16E+01	6,43E-04	1,13E-02	Rps15a-ps3	0,82	Yes
ENSMUSG00000084315	2,65E+00	-3,033655087	2,73E+00	9,86E-02	3,91E-01	Vmn1r-ps128	6,30	No
ENSMUSG00000084319	-1,08E-01	10,66224616	3,75E+00	5,28E-02	2,80E-01	Tpt1-ps3	0,93	No
ENSMUSG00000084320	-3,23E+00	-2,963208994	4,41E+00	3,57E-02	2,24E-01	Gm14125	0,11	No
ENSMUSG00000084323	-9,87E-02	5,75368405	2,16E+00	1,42E-01	4,76E-01	Gm14438	0,93	No
ENSMUSG00000084325	-6,69E-02	-0,389614955	3,95E-02	8,43E-01	9,85E-01	Gm15550	0,95	No
ENSMUSG00000084328	-2,36E-01	0,882843029	1,30E+00	2,54E-01	5,50E-01	Gm14046	0,85	No
ENSMUSG00000084329	-7,25E-03	5,651473213	1,14E-02	9,15E-01	1,00E+00	Gm6733	0,99	No
ENSMUSG00000084333	-1,25E-01	1,819556452	6,29E-01	4,28E-01	7,22E-01	Gm11599	0,92	No

ENSMUSG00000084334	2,01E-01	-0,175648945	4,00E-01	5,27E-01	8,04E-01	Gm13494	1,15	No
ENSMUSG00000084338	2,24E-01	1,326776769	1,36E+00	2,43E-01	5,50E-01	Gm13531	1,17	No
ENSMUSG00000084339	-8,62E-02	1,550329007	2,45E-01	6,21E-01	8,57E-01	Gm6141	0,94	No
ENSMUSG00000084342	-3,15E-02	4,943000469	1,78E-01	6,73E-01	8,90E-01	Gm11224	0,98	No
ENSMUSG00000084345	6,55E-02	2,448069681	2,64E-01	6,07E-01	8,50E-01	Rpl17-ps4	1,05	No
ENSMUSG00000084347	-6,14E-02	6,116208982	8,98E-01	3,43E-01	6,44E-01	Akt2-ps	0,96	No
ENSMUSG00000084350	1,24E-01	5,538698505	3,24E+00	7,18E-02	3,40E-01	Znf41-ps	1,09	No
ENSMUSG00000084353	7,53E-02	3,80064036	6,52E-01	4,20E-01	7,15E-01	Gm15452	1,05	No
ENSMUSG00000084362	-9,98E-01	-2,226202514	1,54E+00	2,15E-01	5,50E-01	2610024D14Rik	0,50	No
ENSMUSG00000084368	-3,83E-02	-3,107651936	-7,91E-11	1,00E+00	1,00E+00	Gm11572	0,97	No
ENSMUSG00000084370	4,53E-01	-0,535278652	1,48E+00	2,24E-01	5,50E-01	Gm15442	1,37	No
ENSMUSG00000084372	-3,83E-02	-3,107651936	-7,91E-11	1,00E+00	1,00E+00	Gm13988	0,97	No
ENSMUSG00000084373	-1,69E+00	-1,472923008	7,86E+00	5,05E-03	5,63E-02	Rps2-ps4	0,31	No
ENSMUSG00000084377	2,61E-01	-1,655141319	2,14E-01	6,44E-01	8,72E-01	Gm15302	1,20	No
ENSMUSG00000084379	1,87E+00	-3,107639691	1,38E+00	2,40E-01	5,50E-01	Gm12957	3,67	No
ENSMUSG00000084380	-1,51E+00	-2,497595721	2,35E+00	1,25E-01	4,43E-01	Tmc4-ps	0,35	No
ENSMUSG00000084381	-7,03E-01	-0,896072811	2,87E+00	9,02E-02	3,81E-01	AA413626	0,61	No
ENSMUSG00000084383	-2,20E-01	6,136283869	1,16E+01	6,52E-04	1,14E-02	Gm13370	0,86	Yes
ENSMUSG00000084384	5,60E-02	5,53251743	6,61E-01	4,16E-01	7,11E-01	Gm12251	1,04	No
ENSMUSG00000084390	-3,64E-01	3,47401304	1,36E+01	2,27E-04	4,83E-03	Gm15425	0,78	Yes
ENSMUSG00000084391	1,85E+00	-3,107657832	1,35E+00	2,46E-01	5,50E-01	Gm13967	3,61	No
ENSMUSG00000084399	-2,94E-01	-2,401397399	1,29E-01	7,20E-01	9,15E-01	Gm15666	0,82	No
ENSMUSG00000084400	7,35E-01	-2,896146969	2,94E-01	5,87E-01	8,40E-01	Gm11576	1,66	No
ENSMUSG00000084403	-8,07E-01	-2,831983513	3,58E-01	5,50E-01	8,17E-01	Rps15a-ps8	0,57	No
ENSMUSG00000084404	-5,88E-02	-3,033616507	1,62E-03	9,68E-01	1,00E+00	Gm12513	0,96	No
ENSMUSG00000084407	2,67E-01	0,875762279	1,50E+00	2,20E-01	5,50E-01	Gm14018	1,20	No
ENSMUSG00000084408	-3,81E-01	2,257099188	7,50E+00	6,18E-03	6,56E-02	Gm11870	0,77	No
ENSMUSG00000084411	-3,83E-02	-3,107651936	-7,91E-11	1,00E+00	1,00E+00	Gm11510	0,97	No
ENSMUSG00000084412	-3,83E-02	-3,107651936	-7,91E-11	1,00E+00	1,00E+00	Gm12044	0,97	No
ENSMUSG00000084413	9,13E-02	-0,165843292	8,57E-02	7,70E-01	9,42E-01	Gm12017	1,07	No
ENSMUSG00000084415	1,26E+00	-2,770618981	9,92E-01	3,19E-01	6,20E-01	Gm12837	2,40	No
ENSMUSG00000084416	-1,18E-01	8,792236537	4,28E+00	3,85E-02	2,33E-01	Rpl10a-ps1	0,92	No

ENSMUSG00000084419	-8,07E-01	-2,832006272	3,57E-01	5,50E-01	8,17E-01	Gm22275	0,57	No
ENSMUSG00000084422	2,15E-01	-1,740855822	1,18E-01	7,31E-01	9,22E-01	Gm25108	1,16	No
ENSMUSG00000084423	-3,88E-03	-3,03362684	4,02E-12	1,00E+00	1,00E+00	Gm25109	1,00	No
ENSMUSG00000084425	-6,30E-02	-2,497654528	4,55E-03	9,46E-01	1,00E+00	Gm25104	0,96	No
ENSMUSG00000084433	-2,69E-01	-0,146057796	7,51E-01	3,86E-01	6,87E-01	Gm25945	0,83	No
ENSMUSG00000084437	-1,87E+00	-3,03364178	1,41E+00	2,36E-01	5,50E-01	Gm23428	0,27	No
ENSMUSG00000084440	-1,61E-02	-0,641273909	1,71E-03	9,67E-01	1,00E+00	Gm26254	0,99	No
ENSMUSG00000084446	1,92E+00	-2,268115271	4,48E+00	3,43E-02	2,18E-01	n-R5s207	3,78	No
ENSMUSG00000084447	1,87E+00	-3,107639691	1,38E+00	2,40E-01	5,50E-01	Gm26251	3,67	No
ENSMUSG00000084453	2,90E-02	-3,070144931	3,20E-11	1,00E+00	1,00E+00	Gm24596	1,02	No
ENSMUSG00000084454	2,61E+00	-3,033672762	2,63E+00	1,05E-01	3,97E-01	Gm24592	6,10	No
ENSMUSG00000084457	-4,07E-01	-2,711739256	1,68E-01	6,82E-01	8,94E-01	n-R5s73	0,75	No
ENSMUSG00000084458	-3,74E-01	-1,460691674	4,15E-01	5,20E-01	7,99E-01	Mir1907	0,77	No
ENSMUSG00000084462	2,63E+00	-2,998018459	2,68E+00	1,02E-01	3,94E-01	n-R5s154	6,20	No
ENSMUSG00000084466	-5,47E-03	-2,963221188	1,50E-05	9,97E-01	1,00E+00	Gm22960	1,00	No
ENSMUSG00000084469	-1,87E+00	-3,033619006	1,41E+00	2,36E-01	5,50E-01	Gm22956	0,27	No
ENSMUSG00000084472	-5,14E-02	-2,770612208	2,33E-03	9,61E-01	1,00E+00	n-R5s23	0,97	No
ENSMUSG00000084477	3,05E-01	-1,997916957	9,51E-02	7,58E-01	9,35E-01	Gm25780	1,24	No
ENSMUSG00000084493	-5,88E-02	-2,896098572	1,55E-03	9,69E-01	1,00E+00	Gm22106	0,96	No
ENSMUSG00000084497	5,48E-02	-0,946046949	1,68E-02	8,97E-01	1,00E+00	Gm22107	1,04	No
ENSMUSG00000084504	-3,97E-01	-2,896156072	9,70E-02	7,56E-01	9,34E-01	Gm25323	0,76	No
ENSMUSG00000084505	-7,18E-01	-3,070161557	2,09E-01	6,48E-01	8,75E-01	Gm25322	0,61	No
ENSMUSG00000084507	3,61E-01	-0,300475981	1,22E+00	2,69E-01	5,65E-01	Gm25321	1,28	No
ENSMUSG00000084513	-1,89E+00	-3,1076441	1,42E+00	2,33E-01	5,50E-01	n-R5s179	0,27	No
ENSMUSG00000084514	1,14E-01	0,042370529	1,57E-01	6,92E-01	9,00E-01	Mir1893	1,08	No
ENSMUSG00000084516	1,80E-01	1,46108056	1,03E+00	3,11E-01	6,11E-01	Mir1902	1,13	No
ENSMUSG00000084519	9,89E-02	0,045332351	1,10E-01	7,40E-01	9,26E-01	Gm22478	1,07	No
ENSMUSG00000084522	-4,01E-02	-2,711715319	1,12E-03	9,73E-01	1,00E+00	Gm25819	0,97	No
ENSMUSG00000084523	-1,66E-01	1,112271773	6,96E-01	4,04E-01	7,01E-01	Mir1903	0,89	No
ENSMUSG00000084526	1,81E+00	-2,896148908	1,31E+00	2,53E-01	5,50E-01	n-R5s192	3,51	No
ENSMUSG00000084539	-1,89E+00	-3,1076441	1,42E+00	2,33E-01	5,50E-01	Gm25842	0,27	No
ENSMUSG00000084543	4,69E-01	-2,600674666	1,71E-01	6,79E-01	8,93E-01	Gm23184	1,38	No

ENSMUSG00000084544	-4,73E-01	-2,146067077	4,13E-01	5,21E-01	7,99E-01	Gm23183	0,72	No
ENSMUSG00000084547	-3,83E-02	-3,107651936	-7,91E-11	1,00E+00	1,00E+00	Gm24936	0,97	No
ENSMUSG00000084552	-3,21E+00	-2,831944582	4,33E+00	3,73E-02	2,30E-01	Gm24840	0,11	No
ENSMUSG00000084557	2,59E+00	-2,963287563	2,60E+00	1,07E-01	3,99E-01	Gm25279	6,03	No
ENSMUSG00000084558	1,87E+00	-3,107639691	1,38E+00	2,40E-01	5,50E-01	Gm24836	3,67	No
ENSMUSG00000084562	2,15E-01	-2,268109486	5,79E-02	8,10E-01	9,66E-01	n-R5s39	1,16	No
ENSMUSG00000084565	8,80E-01	-1,416902036	2,63E+00	1,05E-01	3,97E-01	Mir1901	1,84	No
ENSMUSG00000084566	-1,92E+00	-2,896164745	1,46E+00	2,27E-01	5,50E-01	Gm26477	0,26	No
ENSMUSG00000084571	-3,00E-02	-2,832010318	4,82E-04	9,82E-01	1,00E+00	n-R5s201	0,98	No
ENSMUSG00000084572	-1,89E+00	-3,033648118	1,42E+00	2,33E-01	5,50E-01	Gm23657	0,27	No
ENSMUSG00000084582	-7,38E-02	-3,033634644	2,66E-03	9,59E-01	1,00E+00	Gm25178	0,95	No
ENSMUSG00000084583	1,21E+00	-3,033635275	6,74E-01	4,12E-01	7,07E-01	Gm25177	2,31	No
ENSMUSG00000084587	4,27E+00	-2,655159919	9,37E+00	2,20E-03	3,02E-02	n-R5s174	19,25	Yes
ENSMUSG00000084590	2,09E-01	0,372249908	7,17E-01	3,97E-01	6,97E-01	Mir1896	1,16	No
ENSMUSG00000084592	4,50E-02	-0,226311852	2,10E-02	8,85E-01	1,00E+00	Mir1898	1,03	No
ENSMUSG00000084593	-5,47E-03	-2,963221188	1,50E-05	9,97E-01	1,00E+00	Gm22345	1,00	No
ENSMUSG00000084597	-3,83E-02	-3,107651936	-7,91E-11	1,00E+00	1,00E+00	Gm22343	0,97	No
ENSMUSG00000084600	-3,18E-01	-2,205813091	1,51E-01	6,97E-01	9,03E-01	Gm22201	0,80	No
ENSMUSG00000084601	-3,15E-01	-2,268104699	1,49E-01	7,00E-01	9,04E-01	Gm23111	0,80	No
ENSMUSG00000084603	-5,15E-01	0,458547874	3,88E+00	4,87E-02	2,66E-01	Gm22204	0,70	No
ENSMUSG00000084607	-9,25E-01	-2,548214707	7,49E-01	3,87E-01	6,87E-01	Gm22208	0,53	No
ENSMUSG00000084613	2,06E-02	-3,146103739	-9,26E-10	1,00E+00	1,00E+00	n-R5s164	1,01	No
ENSMUSG00000084624	-6,14E-01	-2,37831051	5,13E-01	4,74E-01	7,62E-01	n-R5s189	0,65	No
ENSMUSG00000084625	7,32E-01	-2,600653462	2,92E-01	5,89E-01	8,41E-01	Gm25544	1,66	No
ENSMUSG00000084628	3,16E+00	-2,896172123	4,07E+00	4,38E-02	2,49E-01	Gm22613	8,92	No
ENSMUSG00000084632	1,42E-01	-1,574170988	6,56E-02	7,98E-01	9,59E-01	Gm23887	1,10	No
ENSMUSG00000084641	-1,87E+00	-3,033619006	1,41E+00	2,36E-01	5,50E-01	n-R5s21	0,27	No
ENSMUSG00000084642	-2,65E+00	-3,033633091	2,78E+00	9,55E-02	3,86E-01	Gm24384	0,16	No
ENSMUSG00000084644	2,53E-01	-1,522685968	2,16E-01	6,42E-01	8,71E-01	Gm24379	1,19	No
ENSMUSG00000084648	-1,05E-01	-2,070212857	1,50E-02	9,03E-01	1,00E+00	Gm24378	0,93	No
ENSMUSG00000084649	-1,02E-01	-2,770578514	7,05E-03	9,33E-01	1,00E+00	n-R5s76	0,93	No
ENSMUSG00000084651	-2,36E+00	-2,998012052	2,18E+00	1,40E-01	4,72E-01	Mir1904	0,19	No

ENSMUSG00000084658	-4,95E-01	-0,372408294	1,98E+00	1,59E-01	5,07E-01	Gm22721	0,71	No
ENSMUSG00000084659	-1,13E-01	-2,963254935	5,86E-03	9,39E-01	1,00E+00	Gm22720	0,92	No
ENSMUSG00000084667	-2,20E-03	0,744200891	9,18E-05	9,92E-01	1,00E+00	Gm23212	1,00	No
ENSMUSG00000084669	1,87E+00	-3,107639691	1,38E+00	2,40E-01	5,50E-01	Gm23206	3,67	No
ENSMUSG00000084674	3,13E+00	-2,896142102	3,99E+00	4,58E-02	2,55E-01	Gm26027	8,74	No
ENSMUSG00000084686	-1,46E+00	-1,641229974	5,85E+00	1,55E-02	1,29E-01	Gm22027	0,36	No
ENSMUSG00000084693	-1,94E+00	-3,107619243	1,50E+00	2,21E-01	5,50E-01	Gm24894	0,26	No
ENSMUSG00000084694	1,84E+00	-3,033649193	1,33E+00	2,49E-01	5,50E-01	Gm24893	3,58	No
ENSMUSG00000084701	7,28E-01	-2,83202549	2,89E-01	5,91E-01	8,41E-01	n-R5s87	1,66	No
ENSMUSG00000084708	6,26E-03	1,828215324	1,54E-03	9,69E-01	1,00E+00	Gm22988	1,00	No
ENSMUSG00000084711	-7,74E-01	-1,711688877	1,77E+00	1,84E-01	5,47E-01	Gm24629	0,58	No
ENSMUSG00000084717	1,85E+00	-3,107657832	1,35E+00	2,46E-01	5,50E-01	Gm24630	3,61	No
ENSMUSG00000084728	-1,35E+00	-2,896126553	1,15E+00	2,84E-01	5,80E-01	Mir1905	0,39	No
ENSMUSG00000084729	1,50E-01	-2,226342502	3,92E-02	8,43E-01	9,85E-01	Gm24139	1,11	No
ENSMUSG00000084734	-1,87E+00	-3,107637761	1,39E+00	2,38E-01	5,50E-01	Gm25804	0,27	No
ENSMUSG00000084738	2,61E+00	-2,770638576	2,64E+00	1,04E-01	3,97E-01	Gm25809	6,10	No
ENSMUSG00000084739	-1,94E+00	-3,107619243	1,50E+00	2,21E-01	5,50E-01	Gm22067	0,26	No
ENSMUSG00000084744	-6,88E-02	-2,107590905	8,78E-03	9,25E-01	1,00E+00	Gm25291	0,95	No
ENSMUSG00000084745	-8,34E-01	-2,963223097	3,81E-01	5,37E-01	8,09E-01	Gm25290	0,56	No
ENSMUSG00000084749	-1,72E+00	-2,60063458	2,06E+00	1,51E-01	4,92E-01	Gm25288	0,30	No
ENSMUSG00000084751	8,29E-02	-1,929253989	1,52E-02	9,02E-01	1,00E+00	Mir1894	1,06	No
ENSMUSG00000084755	-1,54E-01	2,052900424	1,00E+00	3,16E-01	6,17E-01	Gm16276	0,90	No
ENSMUSG00000084756	1,82E+00	-2,963253027	1,31E+00	2,52E-01	5,50E-01	Gm13881	3,53	No
ENSMUSG00000084758	2,65E+00	-2,896119006	2,71E+00	9,98E-02	3,92E-01	Gm12798	6,28	No
ENSMUSG00000084760	1,11E-02	-1,289434094	5,07E-04	9,82E-01	1,00E+00	Gm15472	1,01	No
ENSMUSG00000084766	-2,75E+00	-2,963216826	2,99E+00	8,38E-02	3,71E-01	Gm12714	0,15	No
ENSMUSG00000084769	-3,64E-01	0,103895898	1,84E+00	1,75E-01	5,33E-01	Gm12061	0,78	No
ENSMUSG00000084772	-2,49E-02	0,32598857	9,43E-03	9,23E-01	1,00E+00	Gm15473	0,98	No
ENSMUSG00000084773	1,58E-01	-1,683034409	7,59E-02	7,83E-01	9,50E-01	Gm12159	1,12	No
ENSMUSG00000084775	-1,87E+00	-3,107637761	1,39E+00	2,38E-01	5,50E-01	Gm16741	0,27	No
ENSMUSG00000084780	-3,03E-01	2,839648367	6,64E+00	9,98E-03	9,32E-02	Gm15350	0,81	No
ENSMUSG00000084781	-1,15E-01	-0,2367756	1,13E-01	7,36E-01	9,25E-01	D930015M05Rik	0,92	No

ENSMUSG00000084782	-2,45E-01	-1,963129994	1,04E-01	7,47E-01	9,30E-01	Gm11213	0,84	No
ENSMUSG00000084783	1,85E+00	-3,107657832	1,35E+00	2,46E-01	5,50E-01	Gm15419	3,61	No
ENSMUSG00000084786	-2,27E-01	4,95378773	9,27E+00	2,33E-03	3,16E-02	Ubl5	0,85	Yes
ENSMUSG00000084790	4,07E-02	-0,548236419	1,30E-02	9,09E-01	1,00E+00	Gm15879	1,03	No
ENSMUSG00000084792	-7,72E-01	0,199431559	7,31E+00	6,85E-03	7,06E-02	1700056N10Rik	0,59	No
ENSMUSG00000084796	-7,37E-01	3,814113288	6,37E+01	1,46E-15	2,36E-13	Mir142hg	0,60	Yes
ENSMUSG00000084797	-2,64E-01	-0,205677776	6,15E-01	4,33E-01	7,26E-01	Gm14321	0,83	No
ENSMUSG00000084798	-3,85E-01	-1,800932041	3,44E-01	5,57E-01	8,21E-01	4930533B01Rik	0,77	No
ENSMUSG00000084799	-2,57E-02	2,633018544	4,22E-02	8,37E-01	9,81E-01	Ino80dos	0,98	No
ENSMUSG00000084800	-2,05E+00	-2,548244799	3,19E+00	7,40E-02	3,46E-01	Gm12205	0,24	No
ENSMUSG00000084803	2,05E-01	0,0451775	5,11E-01	4,75E-01	7,63E-01	5830444B04Rik	1,15	No
ENSMUSG00000084804	-3,93E-02	-2,896146176	7,35E-04	9,78E-01	1,00E+00	1700095J12Rik	0,97	No
ENSMUSG00000084805	1,87E+00	-3,107639691	1,38E+00	2,40E-01	5,50E-01	Gm12273	3,67	No
ENSMUSG00000084807	-5,88E-02	-3,033652696	1,62E-03	9,68E-01	1,00E+00	Gm13073	0,96	No
ENSMUSG00000084808	2,52E-01	2,350054445	3,55E+00	5,94E-02	3,02E-01	9430091E24Rik	1,19	No
ENSMUSG00000084809	-1,35E+00	-2,89615366	1,14E+00	2,85E-01	5,82E-01	Gm12631	0,39	No
ENSMUSG00000084816	3,14E+00	-2,832051852	4,02E+00	4,49E-02	2,53E-01	Platr29	8,81	No
ENSMUSG00000084817	-1,31E-01	8,232770188	5,13E+00	2,35E-02	1,68E-01	Gm5526	0,91	No
ENSMUSG00000084821	-2,03E-01	1,438580247	1,22E+00	2,70E-01	5,66E-01	Gm15880	0,87	No
ENSMUSG00000084823	-8,17E-01	-2,627653655	6,67E-01	4,14E-01	7,10E-01	Gm17216	0,57	No
ENSMUSG00000084824	-1,22E+00	-2,655080939	1,42E+00	2,33E-01	5,50E-01	Gm16344	0,43	No
ENSMUSG00000084825	2,62E+00	-3,033654625	2,66E+00	1,03E-01	3,95E-01	2410152P15Rik	6,16	No
ENSMUSG00000084826	1,43E-01	-1,378385269	7,16E-02	7,89E-01	9,54E-01	AI847159	1,10	No
ENSMUSG00000084828	-3,41E-01	-2,863722815	8,54E-02	7,70E-01	9,42E-01	Gm12367	0,79	No
ENSMUSG00000084833	1,87E+00	-3,107639691	1,38E+00	2,40E-01	5,50E-01	Gm12649	3,67	No
ENSMUSG00000084834	-2,49E-01	2,53400609	3,69E+00	5,48E-02	2,87E-01	4930565N06Rik	0,84	No
ENSMUSG00000084835	8,78E-02	3,199586111	6,57E-01	4,18E-01	7,13E-01	Gm12352	1,06	No
ENSMUSG00000084837	-1,87E+00	-3,070153968	1,40E+00	2,36E-01	5,50E-01	1700108N11Rik	0,27	No
ENSMUSG00000084838	-2,27E-01	5,411261744	1,06E+01	1,16E-03	1,82E-02	Gm10241	0,85	Yes
ENSMUSG00000084839	-3,78E-02	-2,832003524	7,08E-04	9,79E-01	1,00E+00	Gm14097	0,97	No
ENSMUSG00000084840	1,78E+00	-1,711856358	7,93E+00	4,86E-03	5,48E-02	Gm11468	3,43	No
ENSMUSG00000084843	-1,86E+00	-3,033612668	1,39E+00	2,38E-01	5,50E-01	B230312C02Rik	0,28	No

ENSMUSG00000084844	-6,75E-02	-1,042611404	2,39E-02	8,77E-01	1,00E+00	Hoxb3os	0,95	No
ENSMUSG00000084845	-9,24E-01	0,237283838	1,16E+01	6,75E-04	1,17E-02	Tmem240	0,53	Yes
ENSMUSG00000084846	9,70E-02	2,205741743	4,95E-01	4,82E-01	7,69E-01	A730011C13Rik	1,07	No
ENSMUSG00000084847	9,01E-03	-3,107646452	-5,16E-10	1,00E+00	1,00E+00	Gm15507	1,01	No
ENSMUSG00000084848	-2,24E-02	-0,289516463	4,47E-03	9,47E-01	1,00E+00	4933408N05Rik	0,98	No
ENSMUSG00000084849	-4,05E-01	-2,070177345	3,41E-01	5,60E-01	8,22E-01	Gm16105	0,76	No
ENSMUSG00000084850	1,87E+00	-3,03364371	1,36E+00	2,43E-01	5,50E-01	Gm12092	3,66	No
ENSMUSG00000084855	-1,87E+00	-3,107637761	1,39E+00	2,38E-01	5,50E-01	Gm9850	0,27	No
ENSMUSG00000084858	2,34E-01	-1,89610775	1,27E-01	7,22E-01	9,16E-01	Gm1980	1,18	No
ENSMUSG00000084860	-8,15E-01	-2,832001636	3,65E-01	5,46E-01	8,14E-01	2610204G07Rik	0,57	No
ENSMUSG00000084862	2,19E-02	2,356520456	2,60E-02	8,72E-01	1,00E+00	Gm16278	1,02	No
ENSMUSG00000084863	2,03E-01	1,002031079	9,74E-01	3,24E-01	6,24E-01	Gm12523	1,15	No
ENSMUSG00000084864	-2,38E-01	1,613229461	2,02E+00	1,56E-01	5,00E-01	1700027A07Rik	0,85	No
ENSMUSG00000084866	-2,80E-01	-1,070162702	3,93E-01	5,31E-01	8,05E-01	A930006K02Rik	0,82	No
ENSMUSG00000084869	5,41E-02	-2,247048167	4,21E-03	9,48E-01	1,00E+00	Gm13063	1,04	No
ENSMUSG00000084870	6,95E-01	-2,146130028	7,01E-01	4,03E-01	7,01E-01	Gm13954	1,62	No
ENSMUSG00000084874	-7,80E-02	4,603075487	9,77E-01	3,23E-01	6,23E-01	Gm12906	0,95	No
ENSMUSG00000084876	-1,12E+00	-2,711746525	9,36E-01	3,33E-01	6,34E-01	Gm14965	0,46	No
ENSMUSG00000084878	-5,97E-02	2,148163375	1,66E-01	6,83E-01	8,95E-01	Lrrc8dos	0,96	No
ENSMUSG00000084880	8,62E-02	3,321205609	6,72E-01	4,12E-01	7,08E-01	Tomm6os	1,06	No
ENSMUSG00000084881	1,54E-01	0,442070935	3,85E-01	5,35E-01	8,07E-01	Gm15585	1,11	No
ENSMUSG00000084883	3,12E-01	0,344654195	1,34E+00	2,46E-01	5,50E-01	Ccdc85c	1,24	No
ENSMUSG00000084884	4,24E-02	-3,033592245	-1,17E-09	1,00E+00	1,00E+00	Gm12289	1,03	No
ENSMUSG00000084891	2,59E+00	-2,963293505	2,61E+00	1,06E-01	3,99E-01	Gm12958	6,03	No
ENSMUSG00000084892	4,12E-01	1,50992982	5,09E+00	2,40E-02	1,70E-01	Gm14471	1,33	No
ENSMUSG00000084893	-6,15E-02	-1,929267121	5,26E-03	9,42E-01	1,00E+00	Hba-ps4	0,96	No
ENSMUSG00000084894	-9,50E-01	-2,401294538	7,90E-01	3,74E-01	6,75E-01	Gm13834	0,52	No
ENSMUSG00000084896	-7,80E-02	2,701046424	4,23E-01	5,15E-01	7,97E-01	Gm11632	0,95	No
ENSMUSG00000084897	1,79E+00	-3,033666867	1,28E+00	2,58E-01	5,50E-01	Gm14226	3,46	No
ENSMUSG00000084899	8,25E-02	4,555514964	1,06E+00	3,03E-01	6,00E-01	Gm15344	1,06	No
ENSMUSG00000084901	3,14E-02	-3,107617333	-7,69E-10	1,00E+00	1,00E+00	Gm13266	1,02	No
ENSMUSG00000084908	-1,33E+00	-2,448649313	1,12E+00	2,91E-01	5,86E-01	C79798	0,40	No

ENSMUSG00000084910	9,03E-01	-1,185695872	3,42E+00	6,44E-02	3,18E-01	C630043F03Rik	1,87	No
ENSMUSG00000084911	1,56E-01	2,871077859	1,80E+00	1,80E-01	5,41E-01	Gm16185	1,11	No
ENSMUSG00000084915	-1,18E-01	0,60452519	2,53E-01	6,15E-01	8,54E-01	C230037L18Rik	0,92	No
ENSMUSG00000084917	-1,08E-01	0,848915704	2,44E-01	6,22E-01	8,57E-01	Gm17477	0,93	No
ENSMUSG00000084918	3,62E-01	1,2400284	3,67E+00	5,55E-02	2,89E-01	Gm12708	1,28	No
ENSMUSG00000084925	-2,05E-01	0,705473502	7,82E-01	3,77E-01	6,78E-01	1810062O18Rik	0,87	No
ENSMUSG00000084930	9,52E-01	-1,278753826	3,54E+00	5,98E-02	3,03E-01	Gm11706	1,94	No
ENSMUSG00000084936	9,39E-02	3,121673267	7,54E-01	3,85E-01	6,86E-01	Gm11583	1,07	No
ENSMUSG00000084939	-1,33E+00	-2,831956329	1,11E+00	2,92E-01	5,87E-01	Gm830	0,40	No
ENSMUSG00000084940	-2,01E-01	-0,834717491	2,65E-01	6,07E-01	8,50E-01	Gm15935	0,87	No
ENSMUSG00000084941	5,13E-01	-0,871712108	1,76E+00	1,85E-01	5,49E-01	Gm11944	1,43	No
ENSMUSG00000084946	4,80E-01	-0,221263791	2,17E+00	1,41E-01	4,74E-01	Dlx1as	1,39	No
ENSMUSG00000084947	-3,23E+00	-2,963208994	4,41E+00	3,57E-02	2,24E-01	Gm15594	0,11	No
ENSMUSG00000084950	-1,62E-01	4,477444146	4,01E+00	4,52E-02	2,54E-01	Gm5577	0,89	No
ENSMUSG00000084951	-5,50E-01	-2,655103745	2,37E-01	6,26E-01	8,60E-01	Mipepos	0,68	No
ENSMUSG00000084956	-5,80E-01	2,326109476	1,85E+01	1,73E-05	5,44E-04	Gm16194	0,67	Yes
ENSMUSG00000084957	-6,07E-03	4,597432191	5,90E-03	9,39E-01	1,00E+00	Bbip1	1,00	No
ENSMUSG00000084960	1,03E-02	-1,007131561	5,83E-04	9,81E-01	1,00E+00	B430010I23Rik	1,01	No
ENSMUSG00000084964	-2,19E-01	0,175910741	5,76E-01	4,48E-01	7,38E-01	Gm15503	0,86	No
ENSMUSG00000084965	-1,88E+00	-3,10764093	1,41E+00	2,35E-01	5,50E-01	Gm13857	0,27	No
ENSMUSG00000084968	1,46E-02	0,906749392	4,75E-03	9,45E-01	1,00E+00	Gm12743	1,01	No
ENSMUSG00000084969	-3,25E-01	-2,497539106	1,32E-01	7,16E-01	9,13E-01	Gm11205	0,80	No
ENSMUSG00000084970	-2,80E-01	0,344926634	1,09E+00	2,96E-01	5,92E-01	1700060J05Rik	0,82	No
ENSMUSG00000084974	-5,68E-02	1,006558117	7,29E-02	7,87E-01	9,53E-01	Gm15567	0,96	No
ENSMUSG00000084978	4,23E-01	-2,71169387	1,39E-01	7,09E-01	9,09E-01	Gm11655	1,34	No
ENSMUSG00000084979	-9,66E-01	1,374475331	2,91E+01	6,99E-08	4,03E-06	Gm16267	0,51	Yes
ENSMUSG00000084980	-1,73E+00	-2,770598936	2,09E+00	1,48E-01	4,88E-01	Slc36a1os	0,30	No
ENSMUSG00000084984	-5,76E-02	3,225372688	2,92E-01	5,89E-01	8,41E-01	Far1os	0,96	No
ENSMUSG00000084985	-2,84E-01	-1,770570369	2,63E-01	6,08E-01	8,51E-01	Gm16135	0,82	No
ENSMUSG00000084986	1,35E-01	0,877969546	3,93E-01	5,31E-01	8,05E-01	Gm16224	1,10	No
ENSMUSG00000084989	1,08E+00	-2,18563178	1,48E+00	2,23E-01	5,50E-01	Crocc2	2,11	No
ENSMUSG00000084992	9,01E-03	-3,107646452	-5,16E-10	1,00E+00	1,00E+00	Gm13842	1,01	No

ENSMUSG00000084995	-6,14E-02	-2,497490511	2,54E-03	9,60E-01	1,00E+00	Lyzl4os	0,96	No
ENSMUSG00000084996	3,61E-01	-2,863726131	6,59E-02	7,97E-01	9,58E-01	Gm11419	1,28	No
ENSMUSG00000084997	-5,03E-03	1,95687627	1,12E-03	9,73E-01	1,00E+00	Gm15471	1,00	No
ENSMUSG00000084998	-4,80E-02	2,755532835	1,59E-01	6,90E-01	8,99E-01	Gm16279	0,97	No
ENSMUSG00000085002	-1,86E+00	-3,033612668	1,39E+00	2,38E-01	5,50E-01	Gm12984	0,28	No
ENSMUSG00000085003	2,59E+00	-2,963293505	2,61E+00	1,06E-01	3,99E-01	Pip5k1bos	6,03	No
ENSMUSG00000085004	-3,17E+00	-2,896113652	4,22E+00	4,00E-02	2,37E-01	5430427M07Rik	0,11	No
ENSMUSG00000085006	-8,75E-01	-2,226217086	1,37E+00	2,41E-01	5,50E-01	BC021767	0,55	No
ENSMUSG00000085009	1,79E-02	1,416008943	1,03E-02	9,19E-01	1,00E+00	Gm12977	1,01	No
ENSMUSG00000085010	-2,56E-01	-2,741415054	5,78E-02	8,10E-01	9,66E-01	Gssos1	0,84	No
ENSMUSG00000085012	6,89E-02	1,136451706	1,12E-01	7,38E-01	9,26E-01	Gm15492	1,05	No
ENSMUSG00000085016	-3,00E-01	-2,146137694	1,56E-01	6,93E-01	9,01E-01	Gm11335	0,81	No
ENSMUSG00000085017	-3,83E-02	-3,107651936	-7,91E-11	1,00E+00	1,00E+00	Gm13412	0,97	No
ENSMUSG00000085023	2,24E-01	4,39153739	7,41E+00	6,49E-03	6,79E-02	Gm12744	1,17	No
ENSMUSG00000085024	-1,36E-01	-1,015592063	8,49E-02	7,71E-01	9,42E-01	C230035I16Rik	0,91	No
ENSMUSG00000085026	-3,83E-02	-3,107651936	-7,91E-11	1,00E+00	1,00E+00	Srrm3os	0,97	No
ENSMUSG00000085027	1,79E+00	-3,033666867	1,28E+00	2,58E-01	5,50E-01	Gm11840	3,46	No
ENSMUSG00000085028	2,74E-01	1,685798044	2,93E+00	8,70E-02	3,78E-01	Slc2a4rg-ps	1,21	No
ENSMUSG00000085030	-2,77E-01	-0,64126299	5,56E-01	4,56E-01	7,45E-01	2810455O05Rik	0,83	No
ENSMUSG00000085033	9,43E-02	1,795173262	3,56E-01	5,51E-01	8,17E-01	Gm11646	1,07	No
ENSMUSG00000085035	-3,67E-01	-1,711549415	3,13E-01	5,76E-01	8,34E-01	Gm12031	0,78	No
ENSMUSG00000085036	2,05E-01	-2,35552165	6,31E-02	8,02E-01	9,61E-01	Gm14011	1,15	No
ENSMUSG00000085037	-3,70E-01	1,110117335	3,54E+00	6,00E-02	3,03E-01	4933421O10Rik	0,77	No
ENSMUSG00000085039	-1,27E-01	2,535022705	9,83E-01	3,21E-01	6,22E-01	Gm15927	0,92	No
ENSMUSG00000085042	2,26E-02	-0,289377042	4,83E-03	9,45E-01	1,00E+00	Abhd11os	1,02	No
ENSMUSG00000085043	9,51E-02	-1,424872956	3,91E-02	8,43E-01	9,85E-01	Gm13184	1,07	No
ENSMUSG00000085048	-1,58E-02	-0,305737042	2,33E-03	9,61E-01	1,00E+00	Gm15537	0,99	No
ENSMUSG00000085051	-8,79E-01	-2,770567379	4,22E-01	5,16E-01	7,97E-01	Gm11542	0,54	No
ENSMUSG00000085054	-1,32E-01	-0,200713984	1,70E-01	6,81E-01	8,93E-01	Gm15834	0,91	No
ENSMUSG00000085055	-1,23E+00	-3,146117124	7,12E-01	3,99E-01	6,98E-01	Gm15958	0,42	No
ENSMUSG00000085057	6,87E-01	1,208314383	1,26E+01	3,83E-04	7,38E-03	Gm13415	1,61	Yes
ENSMUSG00000085058	2,81E-01	0,595810982	1,29E+00	2,55E-01	5,50E-01	8030453O22Rik	1,22	No

ENSMUSG00000085063	-1,22E+00	-2,107572972	2,47E+00	1,16E-01	4,21E-01	Gm16024	0,43	No
ENSMUSG00000085070	1,85E+00	-3,107657832	1,35E+00	2,46E-01	5,50E-01	Gm13211	3,61	No
ENSMUSG00000085072	-8,25E-02	-1,216112333	2,70E-02	8,69E-01	1,00E+00	Ict1os	0,94	No
ENSMUSG00000085078	9,01E-03	-3,107646452	-5,16E-10	1,00E+00	1,00E+00	C030013C21Rik	1,01	No
ENSMUSG00000085080	6,39E-01	-2,548224649	3,38E-01	5,61E-01	8,24E-01	2310044K18Rik	1,56	No
ENSMUSG00000085081	-1,25E-01	-0,662141547	1,09E-01	7,41E-01	9,27E-01	Rptoros	0,92	No
ENSMUSG00000085083	5,97E-03	-3,1461183	-3,40E-10	1,00E+00	1,00E+00	Gm11615	1,00	No
ENSMUSG00000085084	1,26E+00	-2,107739119	3,33E+00	6,79E-02	3,28E-01	4930570G19Rik	2,39	No
ENSMUSG00000085085	-3,39E-01	-1,333214974	4,41E-01	5,07E-01	7,91E-01	1700086P04Rik	0,79	No
ENSMUSG00000085088	-5,47E-01	2,326590249	1,37E+01	2,15E-04	4,61E-03	4931413K12Rik	0,68	Yes
ENSMUSG00000085089	5,42E-01	-1,311162453	1,09E+00	2,96E-01	5,92E-01	Arhgap15os	1,46	No
ENSMUSG00000085091	4,65E-01	-2,711731709	1,69E-01	6,81E-01	8,94E-01	Egfros	1,38	No
ENSMUSG00000085093	7,04E-01	-2,070125298	7,19E-01	3,97E-01	6,97E-01	Gm12737	1,63	No
ENSMUSG00000085095	4,17E-01	-1,424816428	6,20E-01	4,31E-01	7,24E-01	Gm15635	1,33	No
ENSMUSG00000085097	1,85E+00	-3,107657832	1,35E+00	2,46E-01	5,50E-01	Gm13049	3,61	No
ENSMUSG00000085099	-2,31E-01	-0,141127648	5,28E-01	4,67E-01	7,56E-01	Gm16288	0,85	No
ENSMUSG00000085101	-5,10E-01	-2,74145986	2,24E-01	6,36E-01	8,67E-01	Platr16	0,70	No
ENSMUSG00000085102	6,54E-01	-2,77064121	3,54E-01	5,52E-01	8,18E-01	1700010K24Rik	1,57	No
ENSMUSG00000085105	4,30E-02	3,207634389	1,64E-01	6,85E-01	8,97E-01	Gm12758	1,03	No
ENSMUSG00000085106	2,03E-01	-0,946233857	2,29E-01	6,33E-01	8,65E-01	Gm16000	1,15	No
ENSMUSG00000085109	4,06E-01	1,556961355	5,10E+00	2,39E-02	1,70E-01	Gm15356	1,33	No
ENSMUSG00000085110	1,84E+00	-2,832047238	1,33E+00	2,48E-01	5,50E-01	Gm13920	3,59	No
ENSMUSG00000085111	-7,13E-02	1,711935341	1,93E-01	6,60E-01	8,82E-01	Ascl4	0,95	No
ENSMUSG00000085112	4,46E-01	-0,61427876	1,32E+00	2,50E-01	5,50E-01	A530072M11Rik	1,36	No
ENSMUSG00000085116	-9,40E-01	-2,6550963	7,74E-01	3,79E-01	6,80E-01	Gm2735	0,52	No
ENSMUSG00000085118	4,66E-01	-1,136492609	1,06E+00	3,03E-01	6,00E-01	Gm15774	1,38	No
ENSMUSG00000085124	3,14E-02	-3,107617333	-7,69E-10	1,00E+00	1,00E+00	Gm12766	1,02	No
ENSMUSG00000085126	-6,28E-01	0,112167016	4,63E+00	3,14E-02	2,05E-01	Gm12589	0,65	No
ENSMUSG00000085127	7,61E-01	-2,832015354	3,15E-01	5,75E-01	8,34E-01	4930444E06Rik	1,69	No
ENSMUSG00000085129	1,49E-01	0,694122718	4,29E-01	5,12E-01	7,95E-01	5031425F14Rik	1,11	No
ENSMUSG00000085132	-2,13E-02	-0,516301749	3,18E-03	9,55E-01	1,00E+00	Gm12265	0,99	No
ENSMUSG00000085133	-6,97E-01	-1,711574327	1,39E+00	2,39E-01	5,50E-01	B930095G15Rik	0,62	No

ENSMUSG00000085143	-3,18E-01	-1,896134472	2,82E-01	5,96E-01	8,44E-01	Gm11520	0,80	No
ENSMUSG00000085145	1,10E+00	-1,770658365	2,88E+00	8,94E-02	3,79E-01	Gm11614	2,15	No
ENSMUSG00000085147	7,31E-01	-2,963243158	2,91E-01	5,90E-01	8,41E-01	Gm12609	1,66	No
ENSMUSG00000085148	-2,09E-01	5,078535165	7,87E+00	5,03E-03	5,62E-02	Mir22hg	0,87	No
ENSMUSG00000085151	1,20E+00	-3,146123991	6,74E-01	4,12E-01	7,07E-01	1110018N20Rik	2,30	No
ENSMUSG00000085152	1,54E-01	-0,929379887	1,14E-01	7,36E-01	9,24E-01	Gm11496	1,11	No
ENSMUSG00000085154	3,18E-01	-1,548183726	3,64E-01	5,46E-01	8,14E-01	C130046K22Rik	1,25	No
ENSMUSG00000085156	-4,49E-01	4,246985165	2,90E+01	7,15E-08	4,11E-06	Snhg15	0,73	Yes
ENSMUSG00000085161	2,62E+00	-2,655141459	2,66E+00	1,03E-01	3,95E-01	Gm7580	6,16	No
ENSMUSG00000085165	-4,18E-02	0,880666773	3,84E-02	8,45E-01	9,86E-01	Gm12089	0,97	No
ENSMUSG00000085166	-1,30E-01	0,607196882	3,18E-01	5,73E-01	8,33E-01	Gm15522	0,91	No
ENSMUSG00000085167	-1,14E-02	-3,033635021	1,50E-05	9,97E-01	1,00E+00	Gm12063	0,99	No
ENSMUSG00000085168	-1,77E+00	-2,655068999	2,18E+00	1,40E-01	4,73E-01	Gm16252	0,29	No
ENSMUSG00000085169	-2,62E-01	-0,344224369	6,06E-01	4,36E-01	7,29E-01	Gm10785	0,83	No
ENSMUSG00000085170	1,82E+00	-3,033667329	1,31E+00	2,52E-01	5,50E-01	Lrrc75aos1	3,54	No
ENSMUSG00000085171	2,65E+00	-2,800942325	2,71E+00	1,00E-01	3,92E-01	D830026I12Rik	6,27	No
ENSMUSG00000085172	2,38E-04	6,331646887	1,41E-05	9,97E-01	1,00E+00	Gm6542	1,00	No
ENSMUSG00000085173	-1,42E-01	0,292216303	2,76E-01	5,99E-01	8,46E-01	Gm2869	0,91	No
ENSMUSG00000085174	-2,39E-02	2,081363956	2,60E-02	8,72E-01	1,00E+00	Gm16206	0,98	No
ENSMUSG00000085175	-3,99E-01	0,099842635	1,97E+00	1,61E-01	5,09E-01	Gm11423	0,76	No
ENSMUSG00000085177	1,81E+00	-3,10765737	1,28E+00	2,58E-01	5,50E-01	Gm11209	3,49	No
ENSMUSG00000085178	-3,31E-01	1,559203157	3,49E+00	6,16E-02	3,09E-01	Kdm6bos	0,80	No
ENSMUSG00000085179	1,95E-01	-1,929283365	7,69E-02	7,82E-01	9,49E-01	Gm13645	1,14	No
ENSMUSG00000085180	1,87E+00	-3,107639691	1,38E+00	2,40E-01	5,50E-01	AI838599	3,67	No
ENSMUSG00000085181	1,41E+00	-2,800970424	9,15E-01	3,39E-01	6,39E-01	Gm12709	2,66	No
ENSMUSG00000085184	7,32E-04	0,530184194	9,36E-06	9,98E-01	1,00E+00	4933439K11Rik	1,00	No
ENSMUSG00000085185	-3,33E-01	-0,389650365	9,29E-01	3,35E-01	6,36E-01	BC028777	0,79	No
ENSMUSG00000085189	-4,91E-01	-0,117001838	2,51E+00	1,13E-01	4,14E-01	Gm11963	0,71	No
ENSMUSG00000085192	-1,20E-01	-0,896010602	7,60E-02	7,83E-01	9,50E-01	Gm12195	0,92	No
ENSMUSG00000085196	-4,10E+00	-2,548157029	8,51E+00	3,53E-03	4,32E-02	Gm14963	0,06	Yes
ENSMUSG00000085197	7,78E-01	-2,83196814	3,28E-01	5,67E-01	8,29E-01	1700125H03Rik	1,71	No
ENSMUSG00000085199	5,16E-02	-1,669034096	7,76E-03	9,30E-01	1,00E+00	Gm15346	1,04	No

ENSMUSG00000085201	-2,69E+00	-2,896156915	2,88E+00	8,97E-02	3,80E-01	Nr6a1os	0,15	No
ENSMUSG00000085204	1,42E-01	-1,770650337	6,12E-02	8,05E-01	9,63E-01	Gm15327	1,10	No
ENSMUSG00000085205	-1,48E+00	-2,146018053	2,28E+00	1,31E-01	4,55E-01	Gm13185	0,36	No
ENSMUSG00000085206	-1,89E+00	-2,96318033	1,43E+00	2,33E-01	5,50E-01	Gm13380	0,27	No
ENSMUSG00000085207	1,09E+00	-2,378367795	1,13E+00	2,89E-01	5,84E-01	Gm11767	2,13	No
ENSMUSG00000085208	1,61E-01	6,780691817	6,86E+00	8,84E-03	8,53E-02	Brip1os	1,12	No
ENSMUSG00000085211	-1,83E-01	0,502608557	5,41E-01	4,62E-01	7,51E-01	B430219N15Rik	0,88	No
ENSMUSG00000085213	7,55E-01	-1,740933675	1,21E+00	2,72E-01	5,68E-01	Gm13091	1,69	No
ENSMUSG00000085223	-4,78E-01	-1,912592054	5,53E-01	4,57E-01	7,46E-01	Gm11210	0,72	No
ENSMUSG00000085227	4,12E-01	0,256564755	2,48E+00	1,15E-01	4,19E-01	6330418K02Rik	1,33	No
ENSMUSG00000085228	-3,53E+00	-2,896127731	5,59E+00	1,81E-02	1,41E-01	Gm14376	0,09	No
ENSMUSG00000085229	6,85E-01	-2,355611	6,81E-01	4,09E-01	7,06E-01	Gm11672	1,61	No
ENSMUSG00000085232	7,58E-01	-1,497594886	1,94E+00	1,64E-01	5,15E-01	Gm11962	1,69	No
ENSMUSG00000085234	-2,66E+00	-2,355499612	6,48E+00	1,09E-02	9,96E-02	Gm15614	0,16	No
ENSMUSG00000085236	-6,92E-01	1,854697876	2,02E+01	7,00E-06	2,43E-04	2610206C17Rik	0,62	Yes
ENSMUSG00000085237	-6,91E-01	-2,963254291	1,92E-01	6,61E-01	8,82E-01	Gm15406	0,62	No
ENSMUSG00000085238	3,54E+00	-2,832021497	5,46E+00	1,94E-02	1,48E-01	4930479D17Rik	11,66	No
ENSMUSG00000085241	-2,00E-01	1,95982744	1,71E+00	1,91E-01	5,50E-01	Snhg3	0,87	No
ENSMUSG00000085242	1,88E-01	2,542450181	2,21E+00	1,37E-01	4,67E-01	Gm11537	1,14	No
ENSMUSG00000085246	-1,21E+00	-2,185661237	1,90E+00	1,68E-01	5,22E-01	Gm15893	0,43	No
ENSMUSG00000085247	1,79E+00	-3,033666867	1,28E+00	2,58E-01	5,50E-01	4930545L23Rik	3,46	No
ENSMUSG00000085249	-2,71E-01	-1,401359957	2,57E-01	6,12E-01	8,52E-01	Gm12847	0,83	No
ENSMUSG00000085250	3,10E+00	-2,896159767	3,91E+00	4,80E-02	2,63E-01	Gm15234	8,56	No
ENSMUSG00000085255	3,04E-01	0,47739464	1,46E+00	2,27E-01	5,50E-01	Taco1os	1,23	No
ENSMUSG00000085256	-4,76E-01	-0,289483668	2,23E+00	1,35E-01	4,63E-01	Dmrta2os	0,72	No
ENSMUSG00000085260	-8,76E-02	-2,355602093	6,84E-03	9,34E-01	1,00E+00	Med9os	0,94	No
ENSMUSG00000085261	1,63E+00	-2,740874095	1,82E+00	1,77E-01	5,36E-01	Gm13814	3,09	No
ENSMUSG00000085262	6,24E-02	2,693744761	2,58E-01	6,12E-01	8,52E-01	Gm11574	1,04	No
ENSMUSG00000085264	1,96E-01	-2,333223661	5,77E-02	8,10E-01	9,66E-01	Gm15581	1,15	No
ENSMUSG00000085267	-1,90E+00	-3,033594154	1,44E+00	2,30E-01	5,50E-01	Gm14235	0,27	No
ENSMUSG00000085269	4,39E-01	0,206306153	2,88E+00	8,98E-02	3,80E-01	Gm15777	1,36	No
ENSMUSG00000085272	-7,25E-01	-2,548217321	5,88E-01	4,43E-01	7,35E-01	Sbk3	0,61	No

ENSMUSG00000085274	-3,57E-02	0,032481495	1,46E-02	9,04E-01	1,00E+00	BC046401	0,98	No
ENSMUSG00000085275	-2,67E+00	-2,770577046	2,85E+00	9,16E-02	3,82E-01	Gm14487	0,16	No
ENSMUSG00000085278	-1,19E-01	1,921972626	5,72E-01	4,49E-01	7,39E-01	Gm12841	0,92	No
ENSMUSG00000085279	-2,48E+00	-2,247100751	5,32E+00	2,11E-02	1,57E-01	Gm15965	0,18	No
ENSMUSG00000085281	-4,67E-02	1,594387946	7,12E-02	7,90E-01	9,54E-01	Gm13316	0,97	No
ENSMUSG00000085282	-5,76E-01	1,085577958	9,02E+00	2,66E-03	3,49E-02	Gm15663	0,67	Yes
ENSMUSG00000085286	4,58E-01	1,472188332	6,59E+00	1,03E-02	9,53E-02	Ube4bos3	1,37	No
ENSMUSG00000085287	-1,56E-02	1,203065804	6,49E-03	9,36E-01	1,00E+00	4833418N02Rik	0,99	No
ENSMUSG00000085289	2,55E-01	3,340436388	5,97E+00	1,46E-02	1,23E-01	Gm15337	1,19	No
ENSMUSG00000085293	1,80E+00	-3,070187705	1,28E+00	2,58E-01	5,50E-01	Gm13817	3,47	No
ENSMUSG00000085295	-8,21E-03	4,562112531	1,02E-02	9,20E-01	1,00E+00	4930430E12Rik	0,99	No
ENSMUSG00000085297	5,14E-01	-0,529154467	1,84E+00	1,75E-01	5,33E-01	Gm11651	1,43	No
ENSMUSG00000085298	-1,87E+00	-2,896094734	1,42E+00	2,34E-01	5,50E-01	F730035M05Rik	0,27	No
ENSMUSG00000085300	-8,15E-01	-2,770569811	3,66E-01	5,45E-01	8,13E-01	Gm16345	0,57	No
ENSMUSG00000085303	1,75E+00	-2,74091889	2,19E+00	1,39E-01	4,70E-01	Gm16751	3,36	No
ENSMUSG00000085304	1,13E-01	-0,305779615	1,27E-01	7,22E-01	9,16E-01	Gm12802	1,08	No
ENSMUSG00000085307	-6,40E-02	-2,600660034	3,73E-03	9,51E-01	1,00E+00	Gm11525	0,96	No
ENSMUSG00000085310	4,19E-01	1,055446385	4,52E+00	3,35E-02	2,15E-01	Gm11491	1,34	No
ENSMUSG00000085311	-2,33E-01	0,530403885	8,20E-01	3,65E-01	6,66E-01	Gm15445	0,85	No
ENSMUSG00000085315	-4,41E-01	-0,172131477	2,12E+00	1,46E-01	4,83E-01	A430018G15Rik	0,74	No
ENSMUSG00000085316	-7,22E-01	-0,278862283	5,12E+00	2,37E-02	1,69E-01	D330050G23Rik	0,61	No
ENSMUSG00000085317	-1,26E+00	-2,333569415	1,90E+00	1,68E-01	5,22E-01	Gssos2	0,42	No
ENSMUSG00000085319	-2,68E+00	-3,033645766	2,84E+00	9,17E-02	3,82E-01	Gm13330	0,16	No
ENSMUSG00000085321	1,87E+00	-3,107639691	1,38E+00	2,40E-01	5,50E-01	4930412F09Rik	3,67	No
ENSMUSG00000085323	-1,89E+00	-3,1076441	1,42E+00	2,33E-01	5,50E-01	9130410C08Rik	0,27	No
ENSMUSG00000085326	-3,06E-01	-0,704553897	6,75E-01	4,11E-01	7,07E-01	Gm15642	0,81	No
ENSMUSG00000085327	3,76E-01	-1,800977303	3,42E-01	5,59E-01	8,22E-01	Gm16104	1,30	No
ENSMUSG00000085328	1,05E+00	-2,14612663	1,79E+00	1,80E-01	5,42E-01	Gm17131	2,08	No
ENSMUSG00000085330	1,85E+00	-3,107657832	1,35E+00	2,46E-01	5,50E-01	Gm13417	3,61	No
ENSMUSG00000085331	1,65E-01	0,752912897	6,10E-01	4,35E-01	7,28E-01	Gm11274	1,12	No
ENSMUSG00000085333	-2,32E+00	-3,07016418	2,10E+00	1,47E-01	4,86E-01	1700030A11Rik	0,20	No
ENSMUSG00000085334	5,01E-02	4,299723022	3,64E-01	5,46E-01	8,14E-01	Gm12940	1,04	No

ENSMUSG00000085335	2,39E-01	-0,372729947	4,86E-01	4,86E-01	7,73E-01	Gm13684	1,18	No
ENSMUSG00000085337	8,84E-02	3,231888313	6,97E-01	4,04E-01	7,01E-01	Gm15964	1,06	No
ENSMUSG00000085340	-2,25E-01	3,074440677	4,28E+00	3,86E-02	2,33E-01	Gm16335	0,86	No
ENSMUSG00000085342	-2,13E-01	6,656357923	1,18E+01	5,90E-04	1,05E-02	Gm12254	0,86	Yes
ENSMUSG00000085347	-8,20E-01	-1,215892523	2,63E+00	1,05E-01	3,97E-01	Hk1os	0,57	No
ENSMUSG00000085351	-8,00E-01	-2,268080566	9,31E-01	3,35E-01	6,35E-01	Gm12472	0,57	No
ENSMUSG00000085353	9,01E-03	-3,107646452	-5,16E-10	1,00E+00	1,00E+00	1700092C17Rik	1,01	No
ENSMUSG00000085354	-6,23E-01	-1,477110723	1,42E+00	2,33E-01	5,50E-01	Gm2044	0,65	No
ENSMUSG00000085355	-3,20E-01	0,593001604	1,79E+00	1,80E-01	5,42E-01	3010003L21Rik	0,80	No
ENSMUSG00000085360	1,29E-01	1,709244492	6,17E-01	4,32E-01	7,26E-01	Arhgap27os2	1,09	No
ENSMUSG00000085361	1,10E+00	-2,311155155	1,14E+00	2,85E-01	5,81E-01	Gm16305	2,14	No
ENSMUSG00000085363	4,88E-02	0,036653927	2,63E-02	8,71E-01	1,00E+00	Gm15478	1,03	No
ENSMUSG00000085368	9,01E-03	-3,107646452	-5,16E-10	1,00E+00	1,00E+00	Gm16188	1,01	No
ENSMUSG00000085371	-1,69E+00	-2,770605625	1,99E+00	1,59E-01	5,06E-01	Gm3160	0,31	No
ENSMUSG00000085375	2,06E-01	-2,497538968	5,33E-02	8,17E-01	9,70E-01	Gm12506	1,15	No
ENSMUSG00000085376	-3,04E-01	-0,205876489	9,16E-01	3,39E-01	6,39E-01	Gm14508	0,81	No
ENSMUSG00000085377	3,50E+00	-2,832062758	5,31E+00	2,12E-02	1,57E-01	4930551L18Rik	11,29	No
ENSMUSG00000085378	9,46E-01	-1,089051142	4,41E+00	3,57E-02	2,24E-01	Gm11415	1,93	No
ENSMUSG00000085379	8,02E-02	0,344803159	9,74E-02	7,55E-01	9,34E-01	2310058D17Rik	1,06	No
ENSMUSG00000085385	4,05E-02	4,728200607	2,70E-01	6,03E-01	8,48E-01	Snhg17	1,03	No
ENSMUSG00000085388	1,23E+00	-2,57421552	9,42E-01	3,32E-01	6,32E-01	Ptf1aos	2,35	No
ENSMUSG00000085389	-1,50E+00	-2,548183068	2,33E+00	1,27E-01	4,46E-01	1700003M07Rik	0,35	No
ENSMUSG00000085391	1,62E+00	-2,600606177	1,80E+00	1,80E-01	5,41E-01	Gm16150	3,07	No
ENSMUSG00000085394	-2,71E+00	-3,033620915	2,92E+00	8,75E-02	3,78E-01	2210414B05Rik	0,15	No
ENSMUSG00000085397	-5,20E-02	-2,497530991	3,09E-03	9,56E-01	1,00E+00	Gm13524	0,96	No
ENSMUSG00000085398	-5,01E-01	-1,485198164	1,02E+00	3,13E-01	6,12E-01	4931406G06Rik	0,71	No
ENSMUSG00000085399	2,91E-01	1,225680799	2,23E+00	1,36E-01	4,64E-01	Foxd2os	1,22	No
ENSMUSG00000085401	1,31E-01	4,44955581	2,61E+00	1,06E-01	3,99E-01	Slc39a1-ps	1,09	No
ENSMUSG00000085403	1,42E-01	0,23712742	2,40E-01	6,24E-01	8,59E-01	Gm13068	1,10	No
ENSMUSG00000085408	-1,09E-01	1,985884228	5,45E-01	4,60E-01	7,49E-01	C530005A16Rik	0,93	No
ENSMUSG00000085409	4,84E-01	-2,497587191	3,18E-01	5,73E-01	8,33E-01	Gm16155	1,40	No
[truncated: 966,284 more chars]
