# Supplementary material for: Sialic acid blockade in dendritic cells enhances CD8+ T cell responses by facilitating high-avidity interactions
Source: Cell Mol Life Sci. 2022 Jan 28;79(2):98. doi: 10.1007/s00018-021-04027-x (PMC8799591; doi:10.1007/s00018-021-04027-x)
Supplement: Supplementary file 6 — Supplementary file6 (PDF 77 KB) [file 18_2021_4027_MOESM6_ESM.pdf]

Molecular Functions GO Analysis	
**ID**	**Description**
GO:0003779	actin binding
GO:0030246	carbohydrate binding
GO:0061134	peptidase regulator activity
GO:0050839	cell adhesion molecule binding
GO:0051015	actin filament binding
GO:0005201	extracellular matrix structural constituent
GO:0016810	hydrolase activity, acting on carbon-nitrogen (but not peptide) bonds
GO:0005178	integrin binding
GO:0048029	monosaccharide binding
GO:0031418	L-ascorbic acid binding

Biological Processes Go Analysis (simplified)	
**ID**	**Description**
GO:0031589	cell-substrate adhesion
GO:0006874	cellular calcium ion homeostasis
GO:0198738	cell-cell signaling by wnt
GO:0150063	visual system development
GO:0097746	regulation of blood vessel diameter
GO:0120162	positive regulation of cold-induced thermogenesis
GO:1905523	positive regulation of macrophage migration
GO:0098883	synapse pruning

GeneRatio	BgRatio	p.adjust	qvalue
19/382	427/20775	3,41E-02	2,92E-02
19/382	269/20775	4,59E-04	3,93E-04
13/382	239/20775	3,62E-02	3,11E-02
13/382	239/20775	3,62E-02	3,11E-02
12/382	198/20775	3,41E-02	2,92E-02
12/382	141/20775	4,22E-03	3,62E-03
10/382	133/20775	2,53E-02	2,17E-02
10/382	128/20775	2,53E-02	2,17E-02
8/382	85/20775	2,53E-02	2,17E-02
4/382	19/20775	3,41E-02	2,92E-02

GeneRatio	BgRatio	p.adjust	qvalue
24/387	340/21077	9,56E-05	7,83E-05
21/387	477/21077	1,55E-02	1,27E-02
18/387	432/21077	3,12E-02	2,56E-02
17/387	392/21077	2,99E-02	2,45E-02
10/387	160/21077	2,75E-02	2,25E-02
7/387	97/21077	4,23E-02	3,47E-02
4/387	27/21077	3,58E-02	2,93E-02
3/387	7/21077	1,47E-02	1,20E-02
